# Clonal Replacement of Epidemic Methicillin-Resistant *Staphylococcus aureus* Strains in a German University Hospital over a Period of Eleven Years

**DOI:** 10.1371/journal.pone.0028189

**Published:** 2011-11-30

**Authors:** Nicole Albrecht, Lutz Jatzwauk, Peter Slickers, Ralf Ehricht, Stefan Monecke

**Affiliations:** 1 Institute for Medical Microbiology and Hygiene, Technical University of Dresden, Dresden, Germany; 2 Hochschule Lausitz (FH) University of Applied Sciences, Senftenberg, Germany; 3 Department of Hospital Infection Control, Dresden University Hospital, Dresden, Germany; 4 Alere Technologies GmbH, Jena, Germany; Columbia University, United States of America

## Abstract

Worldwide, methicillin-resistant *Staphylococcus aureus* (MRSA) pose an increased risk for healthcare- and community-associated infections. Since the first report of MRSA in England in 1961, several distinct clones or strains have emerged. Changes within the MRSA population of whole countries, small regions or of single hospitals have been observed with some clones replacing others. In this study, the clonal replacement of MRSA isolates in a South-eastern German tertiary care hospital between 2000 and 2010 is described based on microarray analyses of 778 isolates and at least 50 MRSA per year. Within these eleven years, four common epidemic strains, CC22-MRSA-IV, CC45-MRSA-IV, CC5/ST228-MRSA-I (including a variant with a truncated SCC*mec* element) and CC5-MRSA-II were identified. The PVL-negative CC22-MRSA-IV strain (Barnim Epidemic Strain, UK-EMRSA-15) was detected for the first time in 2001 and its abundance increased since then to 58.6% in 2010. CC5-MRSA-II increased from 2% (2000) to about 30% (2003), and since then it fluctuates between 23 and 37% of isolates. CC5/ST228-MRSA-I decreased from about the half of tested isolates (2000) to 2.3% (2010). A similar trend was observed for CC45-MRSA-IV, which decreased drastically down to 3.4% in 2010 after reaching a maximum of 62.0% in 2002. Seventeen other PVL-negative MRSA strains were identified sporadically with no significant trend being observed. Seven PVL-positive MRSA strains were found, but they remained rare during the study period accounting together for 2.7% of isolates.

## Introduction

Shortly after the introduction of methicillin, first resistant isolates were observed in England [1]. Within the last fifty years, methicillin-resistant *Staphylococcus aureus* (MRSA) spread over the whole world. Resistance to methicillin is caused by an alternative penicillin-binding protein (PBP2a, [2]). It is encoded by *mecA*, which is located within mobile genetic elements known as “staphylococcal cassette chromosomes” *mec* (SCC*mec*, [3]–[7]). These are genomic islands of variable size (21–67 kb) always integrating at the same site on the *S. aureus* genome. Since these elements are mobile, unrelated strains might harbour the same SCC*mec* elements and otherwise related strains might differ in carriage of different SCC*mec* elements. With an increasing number of co-circulating MRSA strains, it can be expected that strains are in competition to each other. Strains which are less fit or less adapted to their environment thus should be replaced and a Darwinian evolution should take place within the global MRSA population. Although factors determining failure or success of individual clones have not yet been fully understood, it has been noted that MRSA epidemiology and population structure profoundly changed during the last two decades. Originally, MRSA was restricted to hospitals where few, multiresistant strains dominated (hospital-acquired or -associated MRSA, haMRSA). The abundance of these strains is directly influenced by hospital infection control practices, staff/patient ratio [8] and antibiotic usage as well as by patient risk factors. Since the mid-1990s, so-called community-associated MRSA (caMRSA) strains emerged spreading among healthy individuals outside of hospitals. Some of these strains [9] carry Panton-Valentine leukocidin (PVL), a long known [10], [11] virulence factor which is associated with chronic/recurrent skin and soft tissue infections. Another recent development is the spread of MRSA among livestock and people with animal contact (livestock-associated MRSA, laMRSA; [12]–[14]).

As a result of these developments, changes within the MRSA population structure of hospitals, regions and whole countries were observed. A well documented example is Ireland. From 1971 to 2002, CC8/ST250-MRSA-I got replaced by ST239-MRSA-III which in turn was replaced by CC5-MRSA-II, ST8-MRSA-IIA/B/C/D/E, ST8-MRSA-(IV+*ccrA/B*-4) [15] and by CC22-MRSA-IV, which is now the predominating strain [16], [17]. Portuguese studies show a replacement of ST239-MRSA-III by CC8/ST247-MRSA-I which later was replaced by another variant of ST239-MRSA-III. The latter one was later, after 2002, marginalised by CC22-MRSA-IV while recently CC5-MRSA-II appears to emerge [18]–[20]. In Japan, PVL-positive CC30-MRSA-IV got replaced by CC5-MRSA-II in the early 1990s [21]. Similar observations have been made in Mexico [22] and in Hungary, where CC5/ST228-MRSA-I and CC5-MRSA-II superseded CC8/ST239-MRSA-III [23].

The aim of the present study was to monitor changes of clonal affiliations of MRSA in a German university hospital over a period of eleven years. For that purpose, a large number of isolates (30–50% of all isolates of a given year) were characterised and assigned to epidemic strains.

## Results

From 2000 to 2010, 778 isolates were genotyped, *i.e.*, isolates of one-third to one-half of all MRSA patients identified during each year of the study period (Table 1). An overview on detected strains by year is given in Table 2. Full hybridisation profiles are provided as online file S1.

**Table 1:** Isolate numbers per year and patient demographics.

Year	Isolates tested	Total number of patients with MRSA in that year	Coverage in %	Originated from OPD/ER	Originated from standard wards	Originated from ICU	Gender ratio, male∶female	Age > = 15	Age 16–30	Age 31–45	Age 46–60	Age >61
**2000**	**51**	112	45.5%	**11** (21.6%)	**19** (37.3%)	**21** (41.2%)	**31∶20** (60.8∶39.2%)	**0** (0.0%)	**2** (3.9%)	**3** (5.9%)	**16** (31.4%)	**30** (58.8%)
**2001**	**50**	155	32.3%	**14** (28.0%)	**20** (40.0%)	**16** (32.0%)	**36∶14** (72.0∶28.0%)	**3** (6.0%)	**5** (10.0%)	**6** (12.0%)	**10** (20.0%)	**26** (52.0%)
**2002**	**54**	149	36.2%	**11** (20.4%)	**27** (50.0%)	**15** (27.8%)	**38∶16** (70.4∶29.6%)	**2** (3.7%)	**4** (7.4%)	**8** (14.8%)	**9** (16.7%)	**30** (55.6%)
**2003**	**58**	170	34.1%	**17** (29.3%)	**30** (51.7%)	**10** (17.2%)	**39∶19** (67.2∶32.8%)	**2** (3.4%)	**5** (8.6%)	**4** (6.9%)	**15** (25.9%)	**31** (53.4%)
**2004**	**59**	150	39.3%	**17** (28.8%)	**33** (55.9%)	**9** (15.3%)	**43∶16** (72.9∶27.1%)	**4** (6.8%)	**7** (11.9%)	**3** (5.1%)	**13** (22.0%)	**32** (54.2%)
**2005**	**59**	136	43.4%	**14** (23.7%)	**37** (62.7%)	**8** (13.6%)	**43∶16** (72.9∶27.1%)	**5** (8.5%)	**7** (11.9%)	**5** (8.5%)	**11** (18.6%)	**32** (54.2%)
**2006**	**60**	114	52.6%	**15** (25.0%)	**31** (51.7%)	**14** (23.3%)	**36∶24** (60.0∶40.0%)	**3** (5.0%)	**3** (5.0%)	**5** (8.3%)	**12** (20.0%)	**37** (61.7%)
**2007**	**109**	203	53.7%	**31** (28.4%)	**53** (48.6%)	**25** (22.9%)	**73∶36** (67.0∶33.0%)	**5** (4.6%)	**6** (5.5%)	**8** (7.3%)	**16** (14.7%)	**74** (67.9%)
**2008**	**106**	201	52.7%	**9** (8.5%)	**50** (47.2%)	**47** (44.3%)	**74∶32** (69.8∶30.2%)	**10** (9.4%)	**0** (0.0%)	**10** (9.4%)	**13** (12.3%)	**73** (68.9%)
**2009**	**85**	197	43.1%	**21** (24.7%)	**45** (52.9%)	**19** (22.4%)	**59∶26** (69.4∶30.6%)	**1** (1.2%)	**3** (3.5%)	**4** (4.7%)	**16** (18.8%)	**60** (70.6%)
**2010**	**87**	205	42.4%	**14** (16.1%)	**52** (59.8%)	**21** (24.1%)	**63∶22** (72.4∶25.3%)	**1** (1.1%)	**5** (5.7%)	**3** (3.4%)	**27** (31.0%)	**50** (57.5%)

If numbers for age/gender/ward affiliation do not add up to 100%, data were not available (anonymised samples from infection control unit; one case in 2002 and 2003, two cases in 2010). OPD/ER, outpatient departments and emergency rooms; ICU, intensive care units.

**Table 2:** Strain affiliations of characterised isolates by year.

Strain	Total	2000	2001	2002	2003	2004	2005	2006	2007	2008	2009	2010
**CC22-MRSA-IV**	**270** (34.7%)	**0** (0%)	**3** (6%)	**8** (14.8%)	**10** (17.2%)	**20** (33.9%)	**16** (27.1%)	**24** (40%)	**52** (47.7%)	**44** (41.5%)	**42** (49.4%)	**51** (58.6%)
**CC5-MRSA-II**	**202** (26%)	**1** (2%)	**5** (10%)	**5** (9.3%)	**17** (29.3%)	**20** (33.9%)	**21** (35.6%)	**17** (28.3%)	**25** (22.9%)	**39** (36.8%)	**29** (34.1%)	**23** (26.4%)
**CC45-MRSA-IV**	**174** (22.4%)	**19** (37.3%)	**31** (62%)	**34** (63%)	**24** (41.4%)	**13** (22%)	**12** (20.3%)	**12** (20%)	**10** (9.2%)	**13** (12.3%)	**3** (3.5%)	**3** (3.4%)
**CC5/ST228-MRSA-I**	**32** (4.1%)	**1** (2%)	**1** (2%)	**3** (5.6%)	**3** (5.2%)	**0** (0%)	**4** (6.8%)	**2** (3.3%)	**12** (11%)	**3** (2.8%)	**1** (1.2%)	**2** (2.3%)
**CC5/ST228-MRSA-trunc.SCC** ***mec***	**25** (3.2%)	**24** (47.1%)	**0** (0%)	**0** (0%)	**1** (1.7%)	**0** (0%)	**0** (0%)	**0** (0%)	**0** (0%)	**0** (0%)	**0** (0%)	**0** (0%)
**CC1-MRSA-IV**	**6** (0.8%)	**0** (0%)	**1** (2%)	**0** (0%)	**0** (0%)	**0** (0%)	**0** (0%)	**1** (1.7%)	**1** (0.9%)	**0** (0%)	**1** (1.2%)	**2** (2.3%)
**CC5-MRSA-IV**	**6** (0.8%)	**0** (0%)	**0** (0%)	**0** (0%)	**1** (1.7%)	**1** (1.7%)	**2** (3.4%)	**0** (0%)	**1** (0.9%)	**0** (0%)	**1** (1.2%)	**0** (0%)
**CC5-MRSA-IV/VI**	**1** (0.1%)	**0** (0%)	**0** (0%)	**0** (0%)	**0** (0%)	**1** (1.7%)	**0** (0%)	**0** (0%)	**0** (0%)	**0** (0%)	**0** (0%)	**0** (0%)
**CC7-MRSA-IV**	**1** (0.1%)	**0** (0%)	**0** (0%)	**0** (0%)	**0** (0%)	**0** (0%)	**0** (0%)	**0** (0%)	**0** (0%)	**1** (0.9%)	**0** (0%)	**0** (0%)
**CC8/ST247-MRSA-I**	**1** (0.1%)	**0** (0%)	**0** (0%)	**0** (0%)	**1** (1.7%)	**0** (0%)	**0** (0%)	**0** (0%)	**0** (0%)	**0** (0%)	**0** (0%)	**0** (0%)
**CC8/ST239-MRSA-III**	**6** (0.8%)	**0** (0%)	**5** (10%)	**0** (0%)	**0** (0%)	**0** (0%)	**0** (0%)	**0** (0%)	**0** (0%)	**1** (0.9%)	**0** (0%)	**0** (0%)
**CC8-MRSA-IV (Lyon clone)**	**5** (0.6%)	**0** (0%)	**0** (0%)	**0** (0%)	**0** (0%)	**0** (0%)	**0** (0%)	**1** (1.7%)	**4** (3.7%)	**0** (0%)	**0** (0%)	**0** (0%)
**CC8-MRSA-IV (UK-EMRSA-14)**	**1** (0.1%)	**0** (0%)	**0** (0%)	**0** (0%)	**0** (0%)	**0** (0%)	**0** (0%)	**0** (0%)	**0** (0%)	**1** (0.9%)	**0** (0%)	**0** (0%)
**CC8-MRSA-IV (USA500)**	**3** (0.4%)	**0** (0%)	**0** (0%)	**1** (1.9%)	**0** (0%)	**0** (0%)	**2** (3.4%)	**0** (0%)	**0** (0%)	**0** (0%)	**0** (0%)	**0** (0%)
**CC8/ST72-MRSA-IV**	**1** (0.1%)	**0** (0%)	**0** (0%)	**0** (0%)	**0** (0%)	**0** (0%)	**0** (0%)	**0** (0%)	**0** (0%)	**0** (0%)	**1** (1.2%)	**0** (0%)
**CC8-MRSA-V**	**1** (0.1%)	**0** (0%)	**0** (0%)	**0** (0%)	**0** (0%)	**0** (0%)	**0** (0%)	**1** (1.7%)	**0** (0%)	**0** (0%)	**0** (0%)	**0** (0%)
**CC8/ST254-MRSA-IV/V**	**3** (0.4%)	**1** (2%)	**2** (4%)	**0** (0%)	**0** (0%)	**0** (0%)	**0** (0%)	**0** (0%)	**0** (0%)	**0** (0%)	**0** (0%)	**0** (0%)
**CC8/ST254-MRSA-atyp. SCC** ***mec***	**8** (1%)	**4** (7.8%)	**1** (2%)	**1** (1.9%)	**1** (1.7%)	**0** (0%)	**0** (0%)	**0** (0%)	**0** (0%)	**1** (0.9%)	**0** (0%)	**0** (0%)
**CC22-MRSA-V**	**2** (0.3%)	**0** (0%)	**0** (0%)	**0** (0%)	**0** (0%)	**0** (0%)	**0** (0%)	**0** (0%)	**0** (0%)	**0** (0%)	**1** (1.2%)	**1** (1.1%)
**CC30/ST36-MRSA-II**	**1** (0.1%)	**0** (0%)	**0** (0%)	**1** (1.9%)	**0** (0%)	**0** (0%)	**0** (0%)	**0** (0%)	**0** (0%)	**0** (0%)	**0** (0%)	**0** (0%)
**CC88-MRSA-IV**	**1** (0.1%)	**0** (0%)	**0** (0%)	**0** (0%)	**0** (0%)	**0** (0%)	**0** (0%)	**0** (0%)	**0** (0%)	**1** (0.9%)	**0** (0%)	**0** (0%)
**CC97-MRSA-IV**	**1** (0.1%)	**0** (0%)	**0** (0%)	**0** (0%)	**0** (0%)	**0** (0%)	**0** (0%)	**0** (0%)	**1** (0.9%)	**0** (0%)	**0** (0%)	**0** (0%)
**CC398-MRSA-V**	**6** (0.8%)	**0** (0%)	**0** (0%)	**0** (0%)	**0** (0%)	**0** (0%)	**1** (1.7%)	**0** (0%)	**0** (0%)	**1** (0.9%)	**3** (3.5%)	**1** (1.1%)
**PVL-pos. CC1/ST772-MRSA-V**	**2** (0.3%)	**0** (0%)	**0** (0%)	**0** (0%)	**0** (0%)	**0** (0%)	**0** (0%)	**0** (0%)	**0** (0%)	**0** (0%)	**1** (1.2%)	**1** (1.1%)
**PVL-pos. CC8-MRSA-IV (USA300)**	**4** (0.5%)	**0** (0%)	**0** (0%)	**0** (0%)	**0** (0%)	**0** (0%)	**1** (1.7%)	**0** (0%)	**0** (0%)	**1** (0.9%)	**2** (2.4%)	**0** (0%)
**PVL-pos. CC22-MRSA-IV**	**3** (0.4%)	**1** (2%)	**1** (2%)	**1** (1.9%)	**0** (0%)	**0** (0%)	**0** (0%)	**0** (0%)	**0** (0%)	**0** (0%)	**0** (0%)	**0** (0%)
**PVL-pos. CC30-MRSA-IV**	**2** (0.3%)	**0** (0%)	**0** (0%)	**0** (0%)	**0** (0%)	**0** (0%)	**0** (0%)	**1** (1.7%)	**1** (0.9%)	**0** (0%)	**0** (0%)	**0** (0%)
**PVL-pos. CC59-MRSA-V_T_**	**1** (0.1%)	**0** (0%)	**0** (0%)	**0** (0%)	**0** (0%)	**0** (0%)	**0** (0%)	**0** (0%)	**1** (0.9%)	**0** (0%)	**0** (0%)	**0** (0%)
**PVL-pos. CC80-MRSA-IV**	**8** (1%)	**0** (0%)	**0** (0%)	**0** (0%)	**0** (0%)	**3** (5.1%)	**0** (0%)	**1** (1.7%)	**1** (0.9%)	**0** (0%)	**0** (0%)	**3** (3.4%)
**PVL-pos. CC152-MRSA-V**	**1** (0.1%)	**0** (0%)	**0** (0%)	**0** (0%)	**0** (0%)	**1** (1.7%)	**0** (0%)	**0** (0%)	**0** (0%)	**0** (0%)	**0** (0%)	**0** (0%)

### CC22-MRSA-IV (Barnim Epidemic MRSA)

The most common strain, to which 270 isolates (34.7%) belonged, was a PVL-negative CC22-MRSA-IV, known as Barnim EMRSA or UK-EMRSA-15. 193 isolates originated from male patients (72.0%, two anonymous cases excluded). The mean age of patients was 65 years and their median age was 69 years. 23.4% of isolates came from intensive care units (ICUs), 51.3% from wards and 11.9% from outpatient departments or emergency rooms (OPD/ER).

Beside SCC*mec* IV (2B), CC22-MRSA-IV nearly always carried *blaZ* (beta-lactamase). The macrolide/clindamycin resistance gene *erm*(C) was found in about half of isolates (51.5%). Other resistance genes were rare, being detectable in less than 2% of isolates. This included *tet*(K) and *tet*(M) (tetracycline resistance), *aadD* (tobramycin), *aacA-aphD* (gentamicin) as well as *qacC* (quaternary ammonium compounds). The gene *mupA*, encoding high-level mupirocin resistance, was not found.

CC22-MRSA-IV belonged to *agr* group I and capsule type 5. Isolates always harboured the enterotoxin gene cluster *egc* comprising genes *seg, sei, selm, seln, selo* and *selu/sely*. Enterotoxin genes C (*sec*) and L (*sel*) were found together in 14.1% of isolates.

### CC5-MRSA-II (Rhine-Hesse EMRSA)

Approximately one out of four tested isolates (202 isolates, 26.0%) belonged to CC5-MRSA-II, also known as Rhine-Hesse EMRSA, New York-Japan Clone or UK-EMRSA-3. 134 isolates, *i.e.*, 66.3% were obtained from male patients. The mean age of patients was 59 years, the median age 65 years. 34.3% of isolates originated from ICUs, while 54.2% came from wards and 11.4% from OPD/ER.

The macrolide/clindamycin resistance gene *erm*(A) was present in all isolates and 80.2% carried *aadD*. The beta-lactamase gene *blaZ* was common (81.2%). In contrast, *aacA-aphD*, *tet*(M) and *tet*(K) as well as a gene for resistance to quaternary ammonium compounds, *qacA*, were very rare being detectable in less than one percent of isolates. The *mupA* gene was not found.

Isolates of this strain belonged to *agr* group II and capsule type 5. All isolates carried the *egc* cluster. Furthermore, enterotoxin genes D (*sed*), J (*sej*) and R (*ser*) were commonly detected (85.1%) as well as a distinct variant of the enterotoxin A gene (*sea-N315*, 92.1%).

### CC45-MRSA-IV (Berlin EMRSA)

Approximately one fourth (174 isolates or 22.4%) of the isolates were identified as CC45-MRSA-IV, or Berlin EMRSA. Two-thirds of these isolates (n = 116) came from male patients. The mean age of patients was 61 years, and their median age was 63 years. 17.2% of isolates originated from ICUs, while 53.4% came from wards and 29.3% from OPD/ER.

The carriage of resistance genes in this strain was rather diverse. Neomycin/kanamycin and streptothricin resistance genes *aphA3* and *sat* were usually present (92.5%). *Erm*(C) was found in 24.7% of the tested isolates, while *erm*(A) was rarely detected (1.7%). Other resistance markers included *aadD* (5.7%), *aacA-aphD* (13.2%), *qacA* (8.6%), *qacC* (10.6%), *mupA* (2.9%) as well as, in less than 2% of isolates, *cat* (encoding resistance against chloramphenicol), *far1* (fusidic acid resistance), *tet*(M) and *tet*(K).

Berlin EMRSA isolates belonged to *agr* type I and capsule type 8. All isolates harboured the enterotoxin gene cluster *egc* although the enterotoxin gene *seg* was deleted from 52.3% of isolates. Enterotoxin genes *sec* and *sel* were occasionally found (2.9%). A single isolate was found to be positive for the ACME locus (encoding an arginine metabolic pathway which might play a role in survival on intact skin [24]).

### CC5/ST228-MRSA-I (South German EMRSA)

The fourth frequent strain was CC5/ST228-MRSA-I or South German EMRSA to which 57 isolates (7.3%) were assigned. Forty-six isolates, or 80.7%, originated from male patients. The mean age of patients was 58 and their median age 60 years. 50.9% of isolates came from ICUs, 35.1% from wards and 14.0% from OPD/ER.

Thirty-two isolates harboured a normal SCC*mec* I element while the others lacked cassette chromosome recombinase A and B genes (*ccrA/B*-1). With regard to antibiotic resistance markers, virtually all isolates tested carried *blaZ* (93.0%), *aphA3*+*sat* (98.2%) as well as *erm*(A) (94.7%) and *aacA-aphD* (94.7%). Neither *mupA* nor other resistance genes were found.

CC5/ST228-MRSA-I isolates belonged to *agr* group II and capsule type 5. The *egc*-cluster was usually present. However, in 24% of isolates of that strain, a large part of the *egc*-cluster (*seg, sei, selm, seln* and *selu/sely*) was deleted together with the neighbouring leukocidin genes *lukD/E*. The enterotoxin A gene, *sea*, was detected in a majority of isolates of the variant with a normal SCC*mec* I element (30 out of 32). It was always absent in isolates with a truncated SCC*mec* element.

### Sporadic PVL-negative strains

Beside the four common strains, another seventeen rare PVL-negative strains were identified.

A PVL-negative CC1-MRSA-IV strain was sporadically identified which was identical to West Australian (WA) MRSA-1/57 [25].

Sporadic CC5 strains included CC5-MRSA-IV (Paediatric clone). One isolate of a CC5/ST73-MRSA, associated with a journey to the Canary Islands, Spain, apparently had a composite SCC*mec* element yielding hybridisation signals for *mecA, delta mecR, ugpQ, Q9XB68-dcs, ccrA-2, ccrB-2, ccrA-4* and *ccrB-4* (referred to as CC5/ST73-MRSA-IV/VI).

Several strains belonged to CC8. The North German/Iberian clone CC8/ST247-MRSA-I was identified in a single case of a neurosurgical patient. A pandemic strain known as CC8/ST239-MRSA-III or Vienna/Hungarian/Brazilian clone was found infrequently, being involved in one outbreak and one isolated case (see below). Several PVL-negative CC8-MRSA-IV strains were discerned based on enterotoxin gene carriage. This included UK-EMRSA-14/WA-MRSA-5 (without enterotoxin genes), Lyon Clone/UK-EMRSA-2 (*sea* and/or *sed+sej+ser*) and USA500 (*seb+sek+seq*). The most common of the PVL-negative sporadic strains was CC8/ST254-MRSA, colloquially known as Hannover EMRSA or UK-EMRSA-10. There were two variants of this strain, which differed in SCC*mec* markers. One variant (referred to as CC8/ST254-MRSA-IV/V) reacted with probes for *mecA, delta mecR, ugpQ, Q9XB68-dcs, ccrA-2, ccrB-2, “ccrAA”* (a recombinase analogue, [25]), *ccrC* and, rarely, for the mercury resistance operon. The other variant yielded signals with probes for *mecA, delta mecR, ugpQ, Q9XB68-dcs* and the mercury resistance operon. One isolate of a CC8-MRSA-V was identified. An atypical CC8 strain, ST72-MRSA-IV, differed from other CC8 strains in the presence of *egc* and of CC5-like alleles of several genes encoding adhesion factors (*bbp, sdrC, sdrD*). It is known to occur in the USA and Portugal (USA700; [26], [27]). The travel history of this patient was not available, but an English name suggested foreign background.

Two isolates of a PVL-negative CC22-MRSA-V strain were found. These carried the *egc*-cluster along with *sec* and *sel*. With regard to resistance genes, *tet*(K) was present in both cases as well as Q6GD50, a putative fusidic acid resistance gene.

The recently emerging CC398-MRSA-V was found in six patients. This strain is mainly known from animals, especially from pigs [12]–[14] although transmissions to humans occasionally were observed. Indeed, one of the patients from this study (in 2010) was a retired butcher.

Very rare strains included CC7-MRSA-IV, CC88-MRSA-IV and CC30/ST36-MRSA-II which were found once each; CC97-MRSA-IV was detected in two cases.

### PVL-positive strains

Seven different PVL-positive strains were identified from a total of 21 patients (2.7%). Exactly two-thirds were male. Patients were clearly younger than other MRSA patients (mean, 32 years; median, 28 years). Eighteen cases were skin and soft tissue infections, *i.e.*, abscesses, furuncles *etc*. One isolate was recovered from a screening culture of a nasal swab. A high percentage of isolates came from OPD/ER (42.9%) while 47.6% originated from wards. Two isolates were recovered from bronchial secretions of intensive care patients (9.5%) who have been admitted for other reasons. Cases of necrotising pneumonia were not observed.

Two isolates of the PVL-positive strain CC1/ST772-MRSA-V (Bengal Bay Clone [28], [29]) were found. One of them was associated to a journey to India while for the other case no travel history was recorded. This strain belongs according to the MLST database to CC1. However, it differs from other CC1 strains in absence of the enterotoxin H gene *seh*, in the presence of *egc* and in *agr* and capsule types [25]. Isolates carried *blaZ, msr*(A), *mph*(C), *aacA-aphD, aphA3, sat*, and in one isolate, *erm*(C). Beside PVL genes, isolates harboured *sea, sec* and *sel*.

The PVL-positive CC8-MRSA-IV strain USA300, was identified four times only, and no evidence for a spread or expansion of that clone was noted. Isolates carried the ACME locus and, in 75.0%, *sek* and *seq*. PVL-positive CC22-MRSA-IV were found only three times, the last isolate in 2003. Two isolates of PVL-positive CC30-MRSA-IV (Southwest Pacific or West Samoan Phage Pattern Clone) were identified. Both cases presented with furuncles or “infected insect bites”. One isolate was gentamicin-resistant, carrying *aacA-aphD*. A PVL-positive CC59-MRSA-V_T_ (or 5C2&5), also known as Taiwan Clone, was found once. This strain harbours, beside PVL, enterotoxin genes *seb, sek* and *seq* as well as *erm*(B), which is an otherwise rare macrolide/clindamycin resistance determinant.

The most common PVL-positive strain, the CC80-MRSA-IV (European caMRSA Clone) was found in eight cases. It is characterised by carriage of virulence-associated genes *edinB* and *etD*. Isolates of this strain carried usually plasmid-borne [30] beta-lactamase, *tet*(K) and a fusidic acid resistance gene (*far1*).

CC152-MRSA-V was identified once. The isolate lacked enterotoxin genes and it was positive for *aacA-aphD* (gentamicin resistance gene).

### Temporal changes in the MRSA population

**Figure 1:**
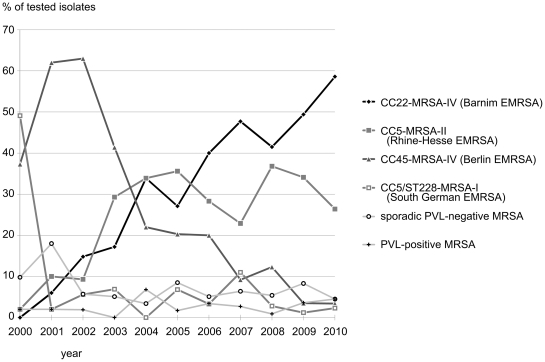
Temporal changes in the MRSA population.

Temporal changes in the MRSA population are presented in Figure 1.

In 2000, about half of the tested isolates were assigned to CC5/ST228-MRSA-I. The vast majority belonged to the variant with a truncated SCC*mec* element. Approximately one third of MRSA isolates were identified as CC45-MRSA-IV. The third common strain was CC8/ST254-MRSA to which, both variants combined, about 10.0% of isolates belonged. CC5-MRSA-II was rare (one isolate, 2.0%). The Barnim EMRSA, *i.e.*, PVL-negative CC22-MRSA-IV was not found. However, one isolate proved to be a PVL-positive CC22-MRSA-IV. Its isolation represents the earliest detection of PVL-MRSA in the Dresden region, approximately three years prior to the earliest yet described case [30].

In 2001, the most abundant strain was CC45-MRSA-IV, to which nearly two-thirds of typed isolates belonged. CC5-MRSA-II became more common (10.0%). The number of cases of CC5/ST228-MRSA-I significantly decreased to just one isolate and the variant with a truncated SCC*mec* element was not detected. Several isolates of CC8/ST239-MRSA-III were found to be identical to each other, and indeed they were epidemiologically linked. The index patient of this outbreak was repatriated after an accident and emergency admission in Greece. The prevalence of CC8/ST254-MRSA remained nearly stable and the PVL-negative CC22-MRSA-IV strain was noted for the first time (6.0%, or three isolates each). The PVL-positive CC22-MRSA-IV strain was found again in a single patient.

In 2002, the majority of isolates belonged to CC45-MRSA-IV. The Barnim EMRSA became more common, reaching about 15%. Three isolates of CC5/ST228-MRSA-I where found which carried un-truncated SCC*mec* I elements. Furthermore, miscellaneous PVL-negative strains were identified including CC5-MRSA-II, a CC8-MRSA-IV strain known as USA500, which was isolated from a trauma patient repatriated from Zimbabwe, and the only CC30/ST36-MRSA-II isolate identified during this study. The PVL-positive CC22-MRSA-IV strain was isolated once.

In 2003, less than half (41.4%) of MRSA isolates belonged to CC45-MRSA-IV. Most of the remaining isolates were assigned to CC5-MRSA-II (29.3%) and CC22-MRSA-IV (17.2%). CC5/ST228-MRSA-I was found only four times, including a last detection of the variant with the truncated SCC*mec* element. Further sporadic strains included CC8/ST247-MRSA-I, CC8/ST254-MRSA and CC5-MRSA-IV. PVL-positive strains were not identified.

In 2004, the majority of isolates were assigned to CC5-MRSA-II and CC22-MRSA-IV (33.9% each). In this year, the latter strain began to occur in wards and outpatient departments caring for patients with diabetic foot ulcers, where it was to become abundant in the following years. Further 22.0% of isolates belonged to CC45-MRSA-IV, including one ACME-positive isolate. Another isolate was identified as PVL-negative CC5/ST73-MRSA-IV/VI. This was acquired during a visit to the Canary Islands, Spain. Four isolates (6.8%) were identified as PVL-positive, three belonging to CC80-MRSA-IV and one to CC152-MRSA-V. Two isolates of the former strain where epidemiologically linked to each other, and an association to journeys either to Italy or to Kosovo was assumed [30].

CC5-MRSA-II dominated in 2005 (33.9%). Other common strains were CC45-MRSA-IV (20.3%), PVL-negative CC22-MRSA-IV (27.1%) and CC5/ST228-MRSA-I (6.8%). Sporadic strains included CC5-MRSA-IV as well as CC8-MRSA-IV (USA500), the later probably being imported from Ethiopia. The livestock-associated CC398-MRSA-V and the PVL-positive CC8-MRSA-IV (USA300) were both identified for the first time with one isolate each.

In 2006, PVL-negative CC22-MRSA-IV was the most common strain (40.0%) followed by CC5-MRSA-II (28.3%) and CC45-MRSA-IV (20.0%). CC5/ST228-MRSA-I has almost disappeared, being found only twice. One of these isolates was traced back to an outbreak in another hospital nearby. Two PVL-positive MRSA strains were identified as CC80-MRSA-IV and ST30-MRSA-IV, respectively.

In 2007, approximately half of isolates (47.7%) belonged to the PVL-negative CC22-MRSA-IV. A number of these isolates originated from wards and outpatient departments specialised in care of diabetic foot ulcers, where a chronic outbreak situation emerged lasting until 2010. Nearly one quarter (22.9%) of isolates belonged to CC5-MRSA-II. CC5/ST228-MRSA-I and CC45-MRSA-IV were less common strains (11.0% and, respectively, 9.2%). CC8-MRSA-IV (Lyon clone) was found in four patients (3.7%), one of whom already carried that strain in 2006. Three PVL-positive isolates were assigned to CC80-MRSA-IV, CC30-MRSA-IV and CC59-MRSA-V_T_.

PVL-negative CC22-MRSA-IV (41.5%) and CC5-MRSA-II (36.8%) were almost equally abundant strains in 2008. CC45-MRSA-IV accounted for 12.3% of the tested isolates. A decrease of CC5/ST228-MRSA-I to three isolates was noted. Single isolates of six further PVL-negative strains were identified including CC8/ST239-MRSA-III (associated with a hospitalisation during travel to Turkey) and CC398-MRSA-V. One isolate of PVL-positive CC8-MRSA-IV (USA300) was found.

In 2009, Barnim EMRSA accounted for nearly the half of MRSA cases (49.4%) showing predominance in medical/diabetological wards. Another 34.1% of isolates belonged to CC5-MRSA-II. CC45-MRSA-II nearly disappeared (three isolates, 3.5%). Another three isolates belonged to CC398-MRSA-V. Three PVL-positive isolates were found (3.5%). One belonged to CC1/ST772-MRSA-V and two to CC8-MRSA-IV (USA300).

In 2010, the PVL-negative CC22-MRSA-IV was again the most prevalent strain to which more than the half (58.6%) of all isolates belonged. The prevalence of CC5-MRSA-II decreased to 26.4%. As few as 3.4% of the isolates were identified as CC45-MRSA-IV, while 2.3% belonged to CC5/ST228-MRSA-I. One case of an infection with the livestock-associated CC398-MRSA-V was diagnosed. PVL-positive strains included CC1/ST772-MRSA-V, from a patient who travelled to India in 2009. The PVL-positive CC80-MRSA-IV clone was identified three times.

## Discussion

Four major epidemic strains show clear and distinct trends over the study time (Table 2 and Figure 1). These strains are the PVL-negative CC22-MRSA-IV (Barnim EMRSA) and CC5-MRSA-II (Rhine-Hesse EMRSA), CC5/ST228-MRSA-I (South German EMRSA, including a variant with a truncated SCC*mec* element) and CC45-MRSA-IV (Berlin EMRSA). From 2000 to 2010, an almost complete displacement of the latter two strains by the former two was observed.

PVL-negative CC22-MRSA-IV (Barnim EMRSA) predominated in recent years. This is a successful, pandemic strain which is common to abundant in many European countries [16], [17], [20], [31]–[33]. In Germany, this strain was first detected in 1996 [34] and its prevalence increased ever since [35]. This development is mirrored by our data according to which that strain became increasingly common during the last decade.

CC5-MRSA-II was very rare in the beginning of the study period and showed a marked increase. In very recent years it seems to be decreasing. It is especially common in intensive care and surgical units. Thus it might be that CC5-MRSA-II locally displaced CC5/ST228-MRSA-I which formerly was common in this type of wards. Future studies might show whether this strain is in competition with Barnim EMRSA or if they might co-exist in slightly different ecological niches.

CC5/ST228-MRSA-I decreased from approximately 50% to a much lower rate fluctuating between 4% and 11% (2007). The majority of the earlier isolates belonged to a distinct variant with a truncated SCC*mec* element not yet described from other regions [25]. This, as well as the high degree of homogeneity among these isolates, suggests an outbreak situation involving several surgical and medical departments prior to the start of the study period. This variant disappeared after 2000/2001 and was found only once ever since. More recent isolates of CC5/ST228-MRSA-I might be associated to transfers from patients from a long-term care facility nearby, in which this strain still regularly was found (author's unpublished observations and [36]).

CC45-MRSA-IV was found in Saxony as early as 1997–1999 [37], [38]. It showed an initial increase accounting for as much as about 60% of isolates in 2001/2002. Then, it decreased drastically and it was only found sporadically in recent years. A possible explanation on a local level is the move of the surgical wards and ICUs into a new building in the end of 2002. However, as this strain also used to be common in other units, this cannot be the only reason for its demise. Besides, this trend was also noted in nationwide surveys [35]. In other geographical regions, such as Belgium, CC45-MRSA-IV was more recently still the predominant MRSA strain [39].

The remaining PVL-negative MRSA strains occurred only sporadically and were so rare that no clear trends could be observed. For some of these strains, trends have been noted in other studies and it could be assumed that our observations mirror these developments although numbers of isolates were small. Examples are CC8/ST247-MRSA-I and CC8/ST254-MRSA. The former strain was detected only once within this study. It has been observed in the region prior to the start of the study period, from 1995–1997 [38], [40], probably being replaced by other strains in the years before 2000, as indicated by nationwide data [35], too. Observations from other countries [15], [20], [41], [42] also show that this strain is currently vanishing. CC8/ST254-MRSA was common until the 1990s in Germany and it was detected in Dresden in 1997, 1998 and 1999 [37], [38]). While it largely disappeared from human patients [35], this strain can still frequently detected among horses [43], [44]. Some globally important strains were conspicuously rare. Infections with these strains might be travel associated, as it was noted for CC8-MRSA-IV (USA500) which originated from patients with travel histories to Ethiopia, Mozambique and Zimbabwe. CC8/ST239-MRSA-III, which can be found essentially everywhere in the world [25], [45], was detected on two occasions including one outbreak with an index patient who acquired it in Greece, and one isolated case associated with medical care in Turkey. Interestingly, this strain is common in the geographically close Czech Republic [46], [47]. Since many years of intense cross-border traffic did not result in a significant spread into Saxony, community-borne transmission of that strain could be regarded as virtually non-existent. Contrarily, transmissions even to distant countries by medical evacuations have been observed. Examples include the 2001 outbreak described above as well as, at a much larger scale, a decade-long outbreak in Ireland following repatriation from Iraq of a single patient [48].

PVL-positive MRSA shows strong fluctuations which, because of the small absolute number of cases, can be considered as random. PVL-MRSA in the Dresden region did not show the massive increase during the study period which was observed, *e.g.*, in the USA or Australia during the same period. The least rare PVL-positive MRSA was CC80-MRSA-IV. This strain occurs across the Middle East and the Maghreb (North African) countries [49]–[54] as well as Europe where it appears to be generally rare, except in Greece [55]. The PVL-positive CC8-MRSA-IV (USA300) is epidemic in the USA and became there the predominant MRSA strain [56], [57]. In contrast, we found this strain in isolated cases only. Other European studies [58] did also not observe a major increase. This difference might lead to the hypothesis that the ecological niche of USA300 is already occupied by another successful strain such as CC22-MRSA-IV, despite it is PVL-negative. The earliest PVL-MRSA strain noted in the study period was PVL-positive CC22-MRSA-IV, isolated as early as 2000. A large scale nosocomial outbreak of PVL-positive CC22-MRSA-IV has been described shortly after in Bavaria, Germany [59]. This strain has in our study not been identified anymore after 2003. This might co-incite with the rise of PVL-negative CC22-MRSA-IV and thus it could be speculated whether some cross-immunity between strains existed on a population level. Other PVL-MRSA strains are epidemic in restricted geographic regions and cases observed in Europe are usually travel-associated [25], [60], [61]. In our study, detection of PVL-positive CC30-MRSA-IV, CC59-MRSA-V_T_ or CC1/ST772-MRSA-V was often connected to previous journeys. Apparently, no sustained subsequent spread in Saxony took place following such importations. However, the prevalence of PVL-MRSA in the population might be somewhat higher than expected from the data of this study, as patients with superficial skin and soft tissue infections, supposed insect or spider bites *etc.* might present rather to primary care facilities than to a tertiary care hospital.

The general reasons, why MRSA strains vanish or prevail are largely unknown. One factor could be the impact of SCC*mec* elements on the replication of *S. aureus* cells. SCC*mec* I elements are known to be associated with higher glucose consumption and lower growth rate compared to strains carrying SCC*mec* IV elements [62], [63]. This could contribute to the widely noted demise of SCC*mec* I strains [15], [20], [41], [42], [64] in a situation of competition to other strains. However, no comparable data are available for other SCC*mec* elements, and a displacement of one SCC*mec* IV strain by another (such as of Berlin EMRSA by Barnim EMRSA) cannot be explained by such a SCC*mec*-associated disadvantage.

Possible other reasons for a replacement of MRSA strains by others might theoretically include specific host preferences, differences in the resistance to antibiotics, in infectivity as well as the ability to cope with environmental stress, the potential for spread, or in virulence.

Gender preference, or a lack of it, is not likely to play a role in determining the “success” of an epidemic strain. It has previously been noted that a majority of MRSA patients was male [65]–[67]. In this study, two-thirds of patients were male, which was also the case in three of the four common strains as well as in PVL-positive strains. In one common strain (CC5/ST228-MRSA-I), the proportion of male patients was even higher. While a strain theoretically could become especially common when being able to colonise females to a comparable extend as males, none of the “successful” strains showed this behaviour. Another host factor could be the average age of the host population. Assuming a low grade of transmission, MRSA strains would gradually pass away together with the generation of hosts they actually reside in. However, this is unlikely as the currently most successful strain in our study (Barnim EMRSA) had the oldest host population.

Antibiotic resistance might be a factor in haMRSA, but many of the recently emerging caMRSA are less resistant. It will be interesting to watch in the future whether the exceptionally multi-resistant caMRSA strain CC1/ST772-MRSA-V might have an advantage compared to less resistant caMRSA strains. Changes in treatment as well as in hygiene regimes or implementation of a “search and destroy” policy should affect mainly hospital-acquired strains without having much impact on community- and livestock-associated strains.

With regard to transmissibility/infectivity, it was suggested that the presence of ACME contributed to the success of the USA300 strain [24], [68]. However, ACME is abundant in coagulase-negative staphylococci [69], [70], but very rare in any *S. aureus* other than USA300 [25]. Therefore, it still needs to be proven whether it indeed confers a selective advantage to *S. aureus*. Resistance to environmental stress, such as UV light, exsiccation or to changing temperatures might also contribute to the transmissibility and thus, to the success of a strain. Experimental data are still needed to verify this.

The presence of virulence factors (PVL) might have played a role in the spread of the USA300 strain. Contrarily, two other successful strains, CC22-MRSA-IV and CC398-MRSA-V, appear not to be particularly virulent, being negative for PVL, exfoliative toxin genes, *tst1* and, in case of CC398-MRSA-V, even for enterotoxin genes.

It can be assumed that the abundance of MRSA is also influenced by factors such as the presence of a susceptible host population and the absence of specific bacteriophages. A newly emerging strain might thus propagate unchecked until some kind of herd immunity evolved or until specific bacteriophages spread. Then it could be assumed that the rise and fall of MRSA strains just mirrored temporal changes in the whole *S. aureus* population, of which MRSA is just a fraction. In order to get a more complete picture of the population dynamics it would be necessary to type large collections of carriage isolates, including methicillin-susceptible *S. aureus*, and monitor possible changes in their clonal composition over extended periods of time. A better understanding of *S. aureus* population dynamics might contribute to the development of effective approaches to control and contain this important pathogen.

## Materials and Methods

### Bacterial isolates

The study was performed at a tertiary care hospital in Dresden, Saxony, *i.e.*, in the South-Eastern part of Germany. The hospital has approximately 1,250 beds and treats 53,000 in-patients per year (http://www.uniklinikum-dresden.de/das-klinikum/jahresbericht/ukd_jahresbericht_2009.pdf). The total incidence density was 0.74 MRSA cases per 1,000 patient-days (2010) which means it was below national average (1.14 MRSA cases per 1,000 patient-days, http://www.nrz-hygiene.de/en/surveillance/hospital-infection-surveillance-system/mrsa-kiss/421d00b8/627/720/).

MRSA isolates passed through standard clinical routine diagnostics. Specimens included screening samples and diagnostic samples from various infections. After primary culture and subculturing of single colonies, screening for clumping factor utilising the Pastorex StaphPlus kit was performed. Susceptibility tests were performed by a standard agar dilution procedure or by VITEK 1 or VITEK 2 systems (BioMerieux, Nuertingen, Germany). Methicillin resistance was confirmed by detection of PBP2a using the Innogenetics MRSA-screen agglutination assay (Innogenetics, Ghent, Belgium). Isolates were stored frozen using cryobank tubes (Microbank, Pro-Lab Diagnostics, Richmond Hill, Canada) at −80°C. For this study, one-third of the preserved isolates per year were chosen randomly and genotyped retrospectively. In addition to that, isolates were included which have been, largely prospectively, genotyped due to clinical or infection control relevance. This included all MRSA isolates from ICUs and diabetology wards, from suspected outbreak situations and from clinical conditions suggesting involvement of PVL. Only one isolate per patient and year was considered. Thus, one-third to one-half of all MRSA isolates of a given year were genotyped. Isolate numbers per year as well as patient demographics are shown in Table 1.

### DNA microarray-based typing

Most isolates were characterised using the Alere StaphyType DNA microarray which covers 334 target sequences (approximately 170 distinct genes and their allelic variants) including species markers, SCC*mec*, capsule and *agr* group typing markers, resistance genes, exotoxin, *set/ssl* and MSCRAMM genes. Primer/probe sequences have been provided previously [25]. Some of the prospectively tested isolates, mainly of the years 2007–08, have been tested using an older version of the array which covered less target genes (see file S1). Protocols have been previously described in detail [25], [36], [71] and are also provided by the kit manufacturer.

MRSA were grown on Columbia blood agar, harvested and enzymatically lysed. DNA was prepared using commercially available systems (spin columns or EZ1, Qiagen, Hilden, Germany). DNA samples were used as templates in a linear primer elongation with one primer per target. During amplification, biotin-16-dUTP was incorporated into the amplicons. These were hybridised to the microarray followed by the addition of horseradish-peroxidase-streptavidin. Finally, hybridisations were visualised by dye precipitation. An image of the microarray was taken and analysed using a designated reader and software (Alere Technologies GmbH, Jena, Germany) using a algorithm as described previously [25]. This included the assignment of isolates to clonal complexes (CC) or sequence types (ST), as defined by MLST [72], and to epidemic strains by comparison of hybridisation profiles to a reference database. Epidemic MRSA strains (EMRSA) were defined and named as in a previously published study [19].

### Sequence-based typing (MLST and *spa*)

Multilocus sequence typing (MLST) and *spa* typing procedures were performed on selected isolates for confirmatory reasons according to previously published protocols [72]. The sequences were analysed using the MLST website (http://saureus.mlst.net/) or, respectively, the Ridom nomenclature (http://spa.ridom.de/) and the SPATYPEMAPPER software (freeware, download at http://www.clondiag.com/fileadmin/Media/Downloads/SPATypeMapper_0_6.zip).

## Supporting Information

File S1 **Patient data and full hybridisation profiles.**
(PDF)
