# Supplementary material for: Clonal Replacement of Epidemic Methicillin-Resistant Staphylococcus aureus Strains in a German University Hospital over a Period of Eleven Years
Source: PLoS One. 2011 Nov 30;6(11):e28189. doi: 10.1371/journal.pone.0028189 (PMC3227659; doi:10.1371/journal.pone.0028189)
Supplement: File S1 — Patient data and full hybridisation profiles. (PDF) [file pone.0028189.s001.pdf]

ISOLATE:	STRAIN	WARD	AGE	GENDER	rrnD1 (S. aureus)	gapA	katA	coA	nuc1	spa	sbi	sarA	saeS	vraS	agrI	agrII	agrIII	agrIV	hld	mecA	delta_mecR	ugpQ	ccrA-1	ccrB-1	plsSCC (COL)	Q9XB68-dcs	ccrA-2	ccrB-2	kdpA-SCC	kdpB-SCC	kdpC-SCC	kdpD-SCC	kdpE-SCC	mecI	mecR	xylR	ccrA-3	ccrB-3	merA	merB	ccrAA (MRSAZH47) _probe 1	ccrAA (MRSAZH47) _probe 2	ccrC (85-2082)	ccrA-4	ccrB-4
					SPECIES MARKER							REGULATORY GENES								METHICILLIN RESISTANCE AND SCCmec TYPING																									
					Ribosomal sequence from S. aureus	glyceraldehyde 3-phosphate dehydro-genase, locus 1	katalase A	coagulase	thermo-stable extracellular nuclease	Protein A	IgG-binding protein	staphylo-coccal accessory regulator A	histidine protein kinase, sae locus	sensor protein	accessory gene regulator allele I	accessory gene regulator allele II	accessory gene regulator allele III	accessory gene regulator allele IV	haemolysin delta	alternate penicillin binding protein 2, defining MRSA	truncated signal transducer protein MecR1	glycerophosphoryl diester phosphodi-esterase, associated with mecA	cassette chromosome recombinase genes A/B-1		plasmin-sensitive surface protein	hypothetical protein from SCCmec elements	cassette chromosome recombinase genes A/B-2		potassium-trans-locating ATPase A, chain 2	potassium-transporting ATPase B, chain 1	potassium-trans-locating ATPase C, chain 2	sensor kinase protein	KDP operon transcrip-tional regulatory protein	methicillin-resistance regulatory protein	signal transducer protein MecR1	homolog of xylose repressor, associated with SCCmec-elements	cassette chromosome recombinase genes A/B-3		mercury resistance operon		cassette chromosome recombinase genes "ccrAA" (hypothetical) and ccrC			cassette chromosome recombinase genes A/B-4	
**2000**																																													
00V07201	CC5/ST228-MRSA, South German EMRSA (truncated SCCmec element)	SUR-ICU	53	f	POS	POS	POS	POS	POS	POS	POS	POS	POS	POS	NEG	POS	NEG	NEG	POS	POS	POS	POS	NEG	NEG	POS	POS	NEG	NEG	NEG	NEG	NEG	NEG	NEG	NEG	NEG	NEG	NEG	NEG	POS	POS	NEG	NEG	NEG	NEG	NEG
00V07634	CC5/ST228-MRSA, South German EMRSA (truncated SCCmec element)	SUR-ICU	46	m	POS	POS	POS	POS	POS	POS	POS	POS	POS	POS	NEG	POS	NEG	NEG	POS	POS	POS	POS	NEG	NEG	POS	POS	NEG	NEG	NEG	NEG	NEG	NEG	NEG	NEG	NEG	NEG	NEG	NEG	POS	POS	NEG	NEG	NEG	NEG	NEG
00V08701	CC5/ST228-MRSA, South German EMRSA (truncated SCCmec element)	MED-OPD	49	f	POS	POS	POS	POS	POS	POS	POS	POS	POS	POS	NEG	POS	NEG	NEG	POS	POS	POS	POS	NEG	NEG	POS	POS	NEG	NEG	NEG	NEG	NEG	NEG	NEG	NEG	NEG	NEG	NEG	NEG	POS	POS	NEG	NEG	NEG	NEG	NEG
00V09056	CC5/ST228-MRSA, South German EMRSA (truncated SCCmec element)	SUR-WARD	65	m	POS	POS	POS	POS	POS	POS	POS	POS	POS	POS	NEG	POS	NEG	NEG	POS	POS	POS	POS	NEG	NEG	POS	POS	NEG	NEG	NEG	NEG	NEG	NEG	NEG	NEG	NEG	NEG	NEG	NEG	POS	POS	NEG	NEG	NEG	NEG	NEG
00V10190	CC5/ST228-MRSA, South German EMRSA (truncated SCCmec element)	MED-OPD	46	f	POS	POS	POS	POS	POS	POS	POS	POS	POS	POS	NEG	POS	NEG	NEG	POS	POS	POS	POS	NEG	NEG	POS	POS	NEG	NEG	NEG	NEG	NEG	NEG	NEG	NEG	AMB	NEG	NEG	NEG	NEG	NEG	NEG	NEG	NEG	NEG	NEG
00V10643	CC5/ST228-MRSA, South German EMRSA (truncated SCCmec element)	SUR-ICU	35	m	POS	POS	POS	POS	POS	POS	POS	POS	POS	POS	NEG	POS	NEG	NEG	POS	POS	POS	POS	NEG	NEG	POS	POS	NEG	NEG	NEG	NEG	NEG	NEG	NEG	NEG	NEG	NEG	NEG	NEG	POS	POS	NEG	NEG	NEG	NEG	NEG
00V11732	CC5/ST228-MRSA, South German EMRSA (truncated SCCmec element)	SUR-ICU	19	m	POS	POS	POS	POS	POS	POS	POS	POS	POS	POS	NEG	POS	NEG	NEG	POS	POS	POS	POS	NEG	NEG	POS	POS	NEG	NEG	NEG	NEG	NEG	NEG	NEG	NEG	NEG	NEG	NEG	NEG	POS	POS	NEG	NEG	NEG	NEG	NEG
00V12300	CC5/ST228-MRSA, South German EMRSA (truncated SCCmec element)	SUR-WARD	49	m	POS	POS	POS	POS	POS	POS	POS	POS	POS	POS	NEG	POS	NEG	NEG	POS	POS	POS	POS	NEG	NEG	POS	POS	NEG	NEG	NEG	NEG	NEG	NEG	NEG	NEG	NEG	NEG	NEG	NEG	POS	POS	NEG	NEG	NEG	NEG	NEG
00V14652	CC5/ST228-MRSA, South German EMRSA (truncated SCCmec element)	SUR-ICU	48	m	POS	POS	POS	POS	POS	POS	POS	POS	POS	POS	NEG	POS	NEG	NEG	POS	POS	POS	POS	NEG	NEG	POS	POS	NEG	NEG	NEG	NEG	NEG	NEG	NEG	NEG	NEG	NEG	NEG	NEG	POS	POS	NEG	NEG	NEG	NEG	NEG
00V24941	CC5/ST228-MRSA, South German EMRSA (truncated SCCmec element)	MED-ICU	58	f	POS	POS	POS	POS	POS	POS	POS	POS	POS	POS	NEG	POS	NEG	NEG	POS	POS	POS	POS	NEG	NEG	POS	POS	NEG	NEG	NEG	NEG	NEG	NEG	NEG	NEG	NEG	NEG	NEG	NEG	POS	POS	NEG	NEG	NEG	NEG	NEG
00V29834	CC5/ST228-MRSA, South German EMRSA (truncated SCCmec element)	SUR-ICU	59	m	POS	POS	POS	POS	POS	POS	POS	POS	POS	POS	NEG	POS	NEG	NEG	POS	POS	POS	POS	NEG	NEG	POS	POS	NEG	NEG	NEG	NEG	NEG	NEG	NEG	NEG	NEG	NEG	NEG	NEG	POS	POS	NEG	NEG	NEG	NEG	NEG
00V29909	CC5/ST228-MRSA, South German EMRSA (truncated SCCmec element)	MED-WARD	75	m	POS	POS	POS	POS	POS	POS	POS	POS	POS	POS	NEG	POS	NEG	NEG	POS	POS	POS	POS	NEG	NEG	POS	POS	NEG	NEG	NEG	NEG	NEG	NEG	NEG	NEG	NEG	NEG	NEG	NEG	POS	POS	NEG	NEG	NEG	NEG	NEG
00V30456	CC5/ST228-MRSA, South German EMRSA (truncated SCCmec element)	MED-OPD	83	m	POS	POS	POS	POS	POS	POS	POS	POS	POS	POS	NEG	POS	NEG	NEG	POS	POS	POS	POS	NEG	NEG	POS	POS	NEG	NEG	NEG	NEG	NEG	NEG	NEG	NEG	NEG	NEG	NEG	NEG	POS	POS	NEG	NEG	NEG	NEG	NEG
00V30827	CC5/ST228-MRSA, South German EMRSA (truncated SCCmec element)	MED-WARD	62	m	POS	POS	POS	POS	POS	POS	POS	POS	POS	POS	NEG	POS	NEG	NEG	POS	POS	POS	POS	NEG	NEG	POS	POS	NEG	NEG	NEG	NEG	NEG	NEG	NEG	NEG	NEG	NEG	NEG	NEG	POS	POS	NEG	NEG	NEG	NEG	NEG
00V33204	CC5/ST228-MRSA, South German EMRSA (truncated SCCmec element)	SUR-ICU	66	f	POS	POS	POS	POS	POS	POS	POS	POS	POS	POS	NEG	POS	NEG	NEG	POS	POS	POS	POS	NEG	NEG	POS	POS	NEG	NEG	NEG	NEG	NEG	NEG	NEG	NEG	NEG	NEG	NEG	NEG	POS	POS	NEG	NEG	NEG	NEG	NEG
00V36490	CC5/ST228-MRSA, South German EMRSA (truncated SCCmec element)	SUR-ICU	74	f	POS	POS	POS	POS	POS	POS	POS	POS	POS	POS	NEG	POS	NEG	NEG	POS	POS	POS	POS	NEG	NEG	POS	POS	NEG	NEG	NEG	NEG	NEG	NEG	NEG	NEG	NEG	NEG	NEG	NEG	POS	POS	NEG	NEG	NEG	NEG	NEG
00V37079	CC5/ST228-MRSA, South German EMRSA (truncated SCCmec element)	SUR-ICU	61	m	POS	POS	POS	POS	POS	POS	POS	POS	POS	POS	NEG	POS	NEG	NEG	POS	POS	POS	POS	NEG	NEG	POS	POS	NEG	NEG	NEG	NEG	NEG	NEG	NEG	NEG	NEG	NEG	NEG	NEG	POS	POS	NEG	NEG	NEG	NEG	NEG
00V40116	CC5/ST228-MRSA, South German EMRSA (truncated SCCmec element)	MED-OPD	63	m	POS	POS	POS	POS	POS	POS	POS	POS	POS	POS	NEG	POS	NEG	NEG	POS	POS	POS	POS	NEG	NEG	POS	POS	NEG	NEG	NEG	NEG	NEG	NEG	NEG	NEG	NEG	NEG	NEG	NEG	POS	POS	NEG	NEG	NEG	NEG	NEG
00V41429	CC5/ST228-MRSA, South German EMRSA (truncated SCCmec element)	SUR-ICU	75	f	POS	POS	POS	POS	POS	POS	POS	POS	POS	POS	NEG	POS	NEG	NEG	POS	POS	POS	POS	NEG	NEG	POS	POS	NEG	NEG	NEG	NEG	NEG	NEG	NEG	NEG	NEG	NEG	NEG	NEG	POS	POS	NEG	NEG	NEG	NEG	NEG
00V42118	CC5/ST228-MRSA, South German EMRSA (truncated SCCmec element)	MED-ICU	63	m	POS		POS	POS	POS	POS	POS	POS	POS	POS	NEG	POS	NEG	NEG	POS	POS	POS	POS	NEG	NEG	POS	POS	NEG	NEG	NEG	NEG	NEG	NEG	NEG	NEG	NEG	NEG	NEG	NEG	POS	POS	NEG	NEG	NEG	NEG	NEG
00V44135	CC5/ST228-MRSA, South German EMRSA (truncated SCCmec element)	MED-ICU	44	m	POS	POS	POS	POS	POS	POS	POS	POS	POS	POS	NEG	POS	NEG	NEG	POS	POS	POS	POS	NEG	NEG	POS	POS	NEG	NEG	NEG	NEG	NEG	NEG	NEG	NEG	NEG	NEG	NEG	NEG	POS	POS	NEG	NEG	NEG	NEG	NEG
00V44538	CC5/ST228-MRSA, South German EMRSA (truncated SCCmec element)	MED-OPD	28	m	POS	POS	POS	POS	POS	POS	POS	POS	POS	POS	NEG	POS	NEG	NEG	POS	POS	POS	POS	NEG	NEG	POS	POS	NEG	NEG	NEG	NEG	NEG	NEG	NEG	NEG	NEG	NEG	NEG	NEG	POS	POS	NEG	NEG	NEG	NEG	NEG
00V44990	CC5/ST228-MRSA, South German EMRSA (truncated SCCmec element)	SUR-ICU	72	m	POS	POS	POS	POS	POS	POS	POS	POS	POS	POS	NEG	POS	NEG	NEG	POS	POS	POS	POS	NEG	NEG	POS	POS	NEG	NEG	NEG	NEG	NEG	NEG	NEG	NEG	NEG	NEG	NEG	NEG	POS	POS	NEG	NEG	NEG	NEG	NEG
00V46910	CC5/ST228-MRSA, South German EMRSA (truncated SCCmec element)	SUR-ICU	52	m	POS	POS	POS	POS	POS	POS	POS	POS	POS	POS	NEG	POS	NEG	NEG	POS	POS	POS	POS	NEG	NEG	POS	POS	NEG	NEG	NEG	NEG	NEG	NEG	NEG	NEG	NEG	NEG	NEG	NEG	POS	POS	NEG	NEG	NEG	NEG	NEG
00V07063	CC5/ST228-MRSA-I, South German EMRSA	MED-WARD	56	m	POS	POS	POS	POS	POS	POS	POS	POS	POS	POS	NEG	POS	NEG	NEG	POS	POS	POS	POS	POS	POS	POS	POS	NEG	NEG	NEG	NEG	NEG	NEG	NEG	NEG	POS	AMB	NEG	NEG	POS	POS	NEG	NEG	NEG	NEG	NEG
00V46921	CC5-MRSA-II, Rhine-Hesse EMRSA/UK-EMRSA-3	MED-WARD	73	f	POS	POS	POS	POS	POS	POS	POS	POS	POS	POS	NEG	POS	NEG	NEG	POS	POS	POS	POS	NEG	NEG	NEG	POS	POS	POS	POS	POS	POS	POS	POS	POS	POS	POS	NEG	NEG	NEG	NEG	NEG	NEG	NEG	NEG	NEG
00V08510	CC8/ST254-MRSA-(atypical SCCmec), Hannover EMRSA	MED-OPD	67	m	POS	POS	POS	POS	POS	POS	POS	POS	POS	POS	POS	NEG	NEG	NEG	POS	POS	POS	POS	NEG	NEG	NEG	POS	NEG	NEG	NEG	NEG	NEG	NEG	NEG	NEG	NEG	NEG	NEG	NEG	NEG	NEG	NEG	NEG	NEG	NEG	NEG
00V25538	CC8/ST254-MRSA-(atypical SCCmec), Hannover EMRSA	MED-OPD	65	m	POS	POS	POS	POS	POS	POS	POS	POS	POS	POS	POS	NEG	NEG	NEG	POS	POS	POS	POS	NEG	NEG	NEG	POS	NEG	NEG	NEG	NEG	NEG	NEG	NEG	NEG	NEG	NEG	NEG	NEG	NEG	NEG	NEG	NEG	NEG	NEG	NEG
00V38664	CC8/ST254-MRSA-(atypical SCCmec), Hannover EMRSA	MED-WARD	67	f	POS	POS	POS	POS	POS	POS	POS	POS	POS	POS	POS	NEG	NEG	NEG	POS	POS	POS	POS	NEG	NEG	NEG	POS	NEG	NEG	NEG	NEG	NEG	NEG	NEG	NEG	NEG	NEG	NEG	AMB	NEG	NEG	NEG	NEG	NEG	NEG	NEG
00V46488	CC8/ST254-MRSA-(atypical SCCmec), Hannover EMRSA	MED-WARD	69	m	POS	POS	POS	POS	POS	POS	POS	POS	POS	POS	POS	NEG	NEG	NEG	POS	POS	POS	POS	NEG	NEG	NEG	POS	NEG	NEG	NEG	NEG	NEG	NEG	NEG	NEG	NEG	NEG	NEG	NEG	NEG	NEG	NEG	NEG	NEG	NEG	NEG
00V44035	CC8/ST254-MRSA-IV/V, UK-EMRSA-10/Hannover EMRSA	MED-ICU	68	f	POS	POS	POS	POS	POS	POS	POS	POS	POS	POS	POS	NEG	NEG	NEG	POS	POS	POS	POS	NEG	NEG	NEG	AMB	NEG	NEG	NEG	NEG	NEG	NEG	NEG	NEG	NEG	NEG	NEG	NEG	NEG	NEG	NEG	NEG	POS	NEG	NEG
00V13324	CC22-MRSA-IV [PVL+]	SUR-WARD	55	f	POS	POS	POS	POS	POS	POS	POS	POS	POS	POS	POS	NEG	NEG	NEG	POS	POS	POS	POS	NEG	NEG	NEG	POS	POS	POS	NEG	NEG	NEG	NEG	NEG	NEG	NEG	NEG	NEG	NEG	NEG	NEG	NEG	NEG	NEG	NEG	NEG
00B02046	CC45-MRSA-IV, Berlin EMRSA	MED-ICU	68	m	POS	POS	POS	POS	POS	POS	POS	POS	POS	POS	POS	NEG	NEG	NEG	POS	POS	POS	POS	NEG	NEG	NEG	POS	POS	POS	NEG	NEG	NEG	NEG	NEG	NEG	NEG	NEG	NEG	NEG	NEG	NEG	NEG	NEG	NEG	NEG	NEG
00V11299	CC45-MRSA-IV, Berlin EMRSA	SUR-ICU	54	m	POS	POS	POS	POS	POS	POS	POS	POS	POS	POS	POS	NEG	NEG	NEG	POS	POS	POS	POS	NEG	NEG	NEG	POS	POS	POS	NEG	NEG	NEG	NEG	NEG	NEG	NEG	NEG	NEG	NEG	NEG	NEG	NEG	NEG	NEG	NEG	NEG
00V14125	CC45-MRSA-IV, Berlin EMRSA	SUR-WARD	76	f	POS	POS	POS	POS	POS	POS	POS	POS	POS	POS	POS	NEG	NEG	NEG	POS	POS	POS	POS	NEG	NEG	NEG	POS	POS	POS	NEG	NEG	NEG	NEG	NEG	NEG	NEG	NEG	NEG	NEG	NEG	NEG	NEG	NEG	NEG	NEG	NEG
00V16040	CC45-MRSA-IV, Berlin EMRSA	MED-WARD	72	f	POS	POS	POS	POS	POS	POS	POS	POS	POS	POS	POS	NEG	NEG	NEG	POS	POS	POS	POS	NEG	NEG	NEG	POS	POS	POS	NEG	NEG	NEG	NEG	NEG	NEG	NEG	NEG	NEG	NEG	NEG	NEG	NEG	NEG	NEG	NEG	NEG
00V25025	CC45-MRSA-IV, Berlin EMRSA	MED-WARD	67	f	POS	POS	POS	POS	POS	AMB	POS	POS	POS	POS	POS	NEG	NEG	NEG	POS	POS	POS	POS	NEG	NEG	NEG	POS	POS	POS	NEG	NEG	NEG	NEG	NEG	NEG	NEG	NEG	NEG	NEG	NEG	NEG	NEG	NEG	NEG	NEG	NEG
00V29618	CC45-MRSA-IV, Berlin EMRSA	MED-OPD	83	f	POS	POS	POS	POS	POS	AMB	POS	POS	POS	POS	POS	NEG	NEG	NEG	POS	POS	POS	POS	NEG	NEG	NEG	POS	POS	POS	NEG	NEG	NEG	NEG	NEG	NEG	NEG	NEG	NEG	NEG	NEG	NEG	NEG	NEG	NEG	NEG	NEG
00V30989	CC45-MRSA-IV, Berlin EMRSA	MED-WARD	82	m	POS	POS	POS	POS	POS	POS	POS	POS	POS	POS	POS	NEG	NEG	NEG	POS	POS	POS	POS	NEG	NEG	NEG	POS	POS	POS	NEG	NEG	NEG	NEG	NEG	NEG	NEG	NEG	NEG	NEG	NEG	NEG	NEG	NEG	NEG	NEG	NEG
00V31007	CC45-MRSA-IV, Berlin EMRSA	MED-WARD	61	m	POS	POS	POS	POS	POS	POS	POS	POS	POS	POS	POS	NEG	NEG	NEG	POS	POS	POS	POS	NEG	NEG	NEG	POS	POS	POS	NEG	NEG	NEG	NEG	NEG	NEG	NEG	NEG	NEG	NEG	NEG	NEG	NEG	NEG	NEG	NEG	NEG
00V31700	CC45-MRSA-IV, Berlin EMRSA	MED-WARD	33	f	POS	POS	POS	POS	POS	POS	POS	POS	POS	POS	POS	NEG	NEG	NEG	POS	POS	POS	POS	NEG	NEG	NEG	POS	POS	POS	NEG	NEG	NEG	NEG	NEG	NEG	NEG	NEG	NEG	NEG	NEG	NEG	NEG	NEG	NEG	NEG	NEG
00V32193	CC45-MRSA-IV, Berlin EMRSA	MED-WARD	69	f	POS	POS	POS	POS	POS	POS	POS	POS	POS	POS	POS	NEG	NEG	NEG	POS	POS	POS	POS	NEG	NEG	NEG	POS	POS	POS	NEG	NEG	NEG	NEG	NEG	NEG	NEG	NEG	NEG	NEG	NEG	NEG	NEG	NEG	NEG	NEG	NEG
00V43085	CC45-MRSA-IV, Berlin EMRSA	MED-OPD	59	f	POS	POS	POS	POS	POS	POS	POS	POS	POS	POS	POS	NEG	NEG	NEG	POS	POS	POS	POS	NEG	NEG	NEG	POS	POS	POS	NEG	NEG	NEG	NEG	NEG	NEG	NEG	NEG	NEG	NEG	NEG	NEG	NEG	NEG	NEG	NEG	NEG
00V44382	CC45-MRSA-IV, Berlin EMRSA	MED-ICU	63	m	POS	POS	POS	POS	POS	NEG	POS	POS	POS	POS	NEG	NEG	NEG	NEG	POS	POS	POS	POS	NEG	NEG	NEG	POS	POS	POS	NEG	NEG	NEG	NEG	NEG	NEG	NEG	NEG	NEG	NEG	NEG	NEG	NEG	NEG	NEG	NEG	NEG
00V46648	CC45-MRSA-IV, Berlin EMRSA	SUR-WARD	60	f	POS	POS	POS	POS	POS	POS	POS	POS	POS	POS	POS	NEG	NEG	NEG	POS	POS	POS	POS	NEG	NEG	NEG	POS	POS	POS	NEG	NEG	NEG	NEG	NEG	NEG	NEG	NEG	NEG	NEG	NEG	NEG	NEG	NEG	NEG	NEG	NEG
00V46843	CC45-MRSA-IV, Berlin EMRSA	SUR-WARD	54	m	POS	POS	POS	POS	POS	POS	POS	POS	POS	POS	POS	NEG	NEG	NEG	POS	POS	POS	POS	NEG	NEG	NEG	POS	POS	POS	NEG	NEG	NEG	NEG	NEG	NEG	NEG	NEG	NEG	NEG	NEG	NEG	NEG	NEG	NEG	NEG	NEG
00V47014	CC45-MRSA-IV, Berlin EMRSA	MED-ICU	64	m	POS	POS	POS	POS	POS	POS	POS	POS	POS	POS	POS	NEG	NEG	NEG	POS	POS	POS	POS	NEG	NEG	NEG	POS	POS	POS	NEG	NEG	NEG	NEG	NEG	NEG	NEG	NEG	NEG	NEG	NEG	NEG	NEG	NEG	NEG	NEG	NEG
00V47053	CC45-MRSA-IV, Berlin EMRSA	MED-OPD	78	f	POS	POS	POS	POS	POS	NEG	POS	POS	POS	POS	AMB	NEG	NEG	NEG	POS	POS	POS	POS	NEG	NEG	NEG	NEG	POS	POS	NEG	NEG	NEG	NEG	NEG	NEG	NEG	NEG	NEG	NEG	NEG	NEG	NEG	NEG	NEG	NEG	NEG
00V47227	CC45-MRSA-IV, Berlin EMRSA	MED-ICU	50	m	POS	POS	POS	POS	POS	POS	POS	POS	POS	POS	POS	NEG	NEG	NEG	POS	POS	POS	POS	NEG	NEG	NEG	POS	POS	POS	NEG	NEG	NEG	NEG	NEG	NEG	NEG	NEG	NEG	NEG	NEG	NEG	NEG	NEG	NEG	NEG	NEG
00V47429	CC45-MRSA-IV, Berlin EMRSA	SUR-WARD	83	m	POS	POS	POS	POS	POS	POS	POS	POS	POS	POS	POS	NEG	NEG	NEG	POS	POS	POS	POS	NEG	NEG	NEG	POS	POS	POS	NEG	NEG	NEG	NEG	NEG	NEG	NEG	NEG	NEG	NEG	NEG	NEG	NEG	NEG	NEG	NEG	NEG
00V47531	CC45-MRSA-IV, Berlin EMRSA	MED-OPD	73	m	POS	POS	POS	POS	POS	POS	POS	POS	POS	POS	POS	NEG	NEG	NEG	POS	POS	POS	POS	NEG	NEG	NEG	POS	POS	POS	NEG	NEG	NEG	NEG	NEG	NEG	NEG	NEG	NEG	NEG	NEG	NEG	NEG	NEG	NEG	NEG	NEG
**2001**																																													
01V27106	CC1-MRSA-IV, WA MRSA-1/57	SUR-WARD	10	m	POS	POS	POS	POS	POS	POS	POS	POS	POS	POS	NEG	NEG	POS	NEG	POS	POS	POS	POS	NEG	NEG	NEG	NEG	POS	POS	NEG	NEG	NEG	NEG	NEG	NEG	NEG	NEG	NEG	NEG	NEG	NEG	NEG	NEG	NEG	NEG	NEG
01V41025	CC5/ST228-MRSA-I, South German EMRSA	SUR-ICU	79	m	POS	POS	POS	POS	POS	POS	POS	POS	POS	POS	NEG	POS	NEG	NEG	POS	POS	POS	POS	POS	POS	POS	POS	NEG	NEG	NEG	NEG	NEG	NEG	NEG	NEG	NEG	AMB	NEG	NEG	NEG	NEG	NEG	NEG	NEG	NEG	NEG
01V12125	CC5-MRSA-II, Rhine-Hesse EMRSA/UK-EMRSA-3	MED-ICU	59	m	POS	POS	POS	POS	POS	POS	POS	POS	POS	POS	NEG	POS	NEG	NEG	POS	POS	POS	POS	NEG	NEG	NEG	POS	POS	POS	POS	POS	POS	POS	POS	POS	POS	POS	NEG	NEG	NEG	NEG	NEG	NEG	NEG	NEG	NEG
01V29747	CC5-MRSA-II, Rhine-Hesse EMRSA/UK-EMRSA-3	MED-ICU	73	f	POS	POS	POS	POS	POS	POS	POS	POS	POS	POS	NEG	POS	NEG	NEG	POS	POS	POS	POS	NEG	NEG	NEG	POS	POS	POS	POS	POS	POS	POS	POS	POS	POS	POS	NEG	NEG	NEG	NEG	NEG	NEG	NEG	NEG	NEG
01V32464	CC5-MRSA-II, Rhine-Hesse EMRSA/UK-EMRSA-3	MED-ICU	16	m	POS	POS	POS	POS	POS	POS	POS	POS	POS	POS	NEG	POS	NEG	NEG	POS	POS	POS	POS	NEG	NEG	NEG	POS	POS	POS	POS	POS	POS	POS	POS	POS	POS	POS	NEG	NEG	NEG	NEG	NEG	NEG	NEG	NEG	NEG
01V39703	CC5-MRSA-II, Rhine-Hesse EMRSA/UK-EMRSA-3	SUR-ICU	58	m	POS	POS	POS	POS	POS	POS	POS	POS	POS	POS	NEG	POS	NEG	NEG	POS	POS	POS	POS	NEG	NEG	NEG	POS	POS	POS	POS	POS	POS	POS	POS	POS	POS	POS	NEG	NEG	NEG	NEG	NEG	NEG	NEG	NEG	NEG
01V40228	CC5-MRSA-II, Rhine-Hesse EMRSA/UK-EMRSA-3	MED-OPD	61	m	POS	POS	POS	POS		POS	POS	POS	POS	POS	NEG	POS	NEG	NEG	POS	POS	POS	POS	NEG	NEG	NEG	POS	POS	POS	POS	POS	POS	POS	POS	POS	POS	POS	NEG	NEG	NEG	NEG	NEG	NEG	NEG	NEG	NEG
01B05861	CC8/ST239-MRSA-III, Vienna/Hungarian/Brazilian Clone	MED-ICU	49	m	POS	POS	POS	POS	POS	POS	POS	POS	POS	POS	POS	NEG	NEG	NEG	POS	POS	POS	POS	NEG	AMB	NEG	NEG	NEG	NEG	NEG	NEG	NEG	NEG	NEG	POS	POS	POS	POS	POS	POS	POS	NEG	NEG	POS	NEG	NEG
01V34572	CC8/ST239-MRSA-III, Vienna/Hungarian/Brazilian Clone	SUR-ICU	28	m	POS	POS	POS	POS	POS	POS	POS	POS	POS	POS	POS	NEG	NEG	NEG	POS	POS	POS	POS	NEG	NEG	NEG	NEG	NEG	NEG	NEG	NEG	NEG	NEG	NEG	POS	POS	POS	POS	POS	POS	POS	NEG	NEG	POS	NEG	NEG
01V36469	CC8/ST239-MRSA-III, Vienna/Hungarian/Brazilian Clone	SUR-ICU	38	m	POS	POS	POS	AMB	POS	POS	POS	POS	POS		POS	NEG	NEG	NEG	POS	POS	POS	POS	NEG	NEG	NEG	NEG	NEG	NEG	NEG	NEG	NEG	NEG	NEG	POS	POS	POS	POS	POS	POS	POS	NEG	NEG	POS	NEG	NEG
01V39123	CC8/ST239-MRSA-III, Vienna/Hungarian/Brazilian Clone	SUR-WARD	29	m	POS	POS	POS	POS	POS	POS	POS	POS	POS	POS	POS	NEG	NEG	NEG	POS	POS	POS	POS	NEG	NEG	NEG	NEG	NEG	NEG	NEG	NEG	NEG	NEG	NEG	POS	POS	POS	POS	POS	POS	POS	NEG	NEG	POS	NEG	NEG
01V39143	CC8/ST239-MRSA-III, Vienna/Hungarian/Brazilian Clone	MED-ICU	70	m	POS	POS	POS	POS	POS	POS	POS	POS	POS	POS	POS	NEG	NEG	NEG	POS	POS	POS	POS	NEG	NEG	NEG	NEG	NEG	NEG	NEG	NEG	NEG	NEG	NEG	POS	POS	POS	POS	POS	POS	POS	NEG	NEG	POS	NEG	NEG
01V10962	CC8/ST254-MRSA-(atypical SCCmec), Hannover EMRSA	MED-OPD	61	m	POS	POS	POS	POS	POS	POS	POS	POS	POS	POS	POS	NEG	NEG	NEG	POS	POS	POS	POS	NEG	NEG	NEG	POS	NEG	NEG	NEG	NEG	NEG	NEG	NEG	NEG	NEG	NEG	NEG	NEG	NEG	NEG	NEG	NEG	NEG	NEG	NEG
01V10963	CC8/ST254-MRSA-IV/V, UK-EMRSA-10/Hannover EMRSA	MED-OPD	57	m	POS	POS	POS	POS	POS	POS	POS	POS	POS	POS	POS	NEG	NEG	NEG	POS	POS	POS	POS	NEG	NEG	NEG	POS	NEG	NEG	NEG	NEG	NEG	NEG	NEG	NEG	NEG	NEG	NEG	AMB	NEG	NEG	NEG	NEG	POS	NEG	NEG
01V36287	CC8/ST254-MRSA-IV/V, UK-EMRSA-10/Hannover EMRSA	MED-OPD	69	m	POS	POS	POS	POS	POS	POS	POS	POS	POS	POS	POS	NEG	AMB	NEG	POS	POS	POS	POS	NEG	NEG	NEG	POS	NEG	NEG	NEG	NEG	NEG	NEG	NEG	NEG	NEG	NEG	NEG	NEG	NEG	NEG	NEG	NEG	POS	NEG	NEG
01V25329	CC22-MRSA-IV [PVL+]	MED-ICU	33	m	POS	POS	POS	POS	POS	POS	POS	POS	POS	POS	POS	NEG	NEG	NEG	POS	POS	POS	POS	NEG	NEG	NEG	POS	POS	POS	NEG	NEG	NEG	NEG	NEG	NEG	NEG	NEG	NEG	NEG	NEG	NEG	NEG	NEG	NEG	NEG	NEG
01V07770	CC22-MRSA-IV, Barnim/UK-EMRSA-15	MED-OPD	2	f	POS	POS	POS	POS		POS	POS	POS	POS	POS	POS	NEG	NEG	NEG	POS	POS			NEG	NEG	NEG	POS	POS	POS	NEG	NEG	NEG	NEG	NEG	NEG	NEG	NEG	NEG	NEG	NEG	NEG	NEG	NEG	NEG	NEG	NEG
01V20122	CC22-MRSA-IV, Barnim/UK-EMRSA-15	SUR-WARD	78	m	POS	POS	POS	POS	POS	POS	POS	POS	POS	POS	POS	NEG	NEG	NEG	POS	POS	POS	POS	NEG	NEG	NEG	POS	POS	POS	NEG	NEG	NEG	NEG	NEG	NEG	NEG	NEG	NEG	NEG	NEG	NEG	NEG	NEG	NEG	NEG	NEG
01V43277	CC22-MRSA-IV, Barnim/UK-EMRSA-15	MED-ICU	61	m	POS	POS	POS	POS	POS	POS	POS	POS	POS	POS	POS	NEG	NEG	NEG	POS	POS	POS	POS	NEG	NEG	NEG	POS	POS	POS	NEG	NEG	NEG	NEG	NEG	NEG	NEG	NEG	NEG	NEG	NEG	NEG	NEG	NEG	NEG	NEG	NEG
01F02744	CC45-MRSA-IV, Berlin EMRSA	MED-WARD	63	m	POS	POS	POS	POS	POS	POS	POS	POS	POS	POS	POS	NEG	NEG	NEG	POS	POS	POS	POS	NEG	NEG	NEG	POS	POS	POS	NEG	NEG	NEG	NEG	NEG	NEG	NEG	NEG	NEG	NEG	NEG	NEG	NEG	NEG	NEG	NEG	NEG
01V01127	CC45-MRSA-IV, Berlin EMRSA	SUR-ICU	68	f	POS	POS	POS	POS	POS	POS	POS	POS	POS	POS	POS	NEG	NEG	NEG	POS	POS	POS	POS	NEG	NEG	NEG	POS	POS	POS	NEG	NEG	NEG	NEG	NEG	NEG	NEG	NEG	NEG	NEG	NEG	NEG	NEG	NEG	NEG	NEG	NEG
01V01445	CC45-MRSA-IV, Berlin EMRSA	MED-WARD	61	m	POS	POS	POS	POS	POS	POS	POS	POS	POS	POS	POS	NEG	NEG	NEG	POS	POS	POS	POS	NEG	NEG	NEG	POS	POS	POS	NEG	NEG	NEG	NEG	NEG	NEG	NEG	NEG	NEG	NEG	NEG	NEG	NEG	NEG	NEG	NEG	NEG
01V02575	CC45-MRSA-IV, Berlin EMRSA	MED-OPD	44	m	POS	POS	POS	POS	POS	AMB	POS	POS	POS	POS	POS	NEG	NEG	NEG	POS	POS	POS	POS	NEG	NEG	NEG	POS	POS	POS	NEG	NEG	NEG	NEG	NEG	NEG	NEG	NEG	NEG	NEG	NEG	NEG	NEG	NEG	NEG	NEG	NEG
01V02636	CC45-MRSA-IV, Berlin EMRSA	MED-OPD	2	f	POS	POS	POS	POS	POS	POS	POS	POS	POS	POS	POS	NEG	NEG	NEG	POS	POS	POS	POS	NEG	NEG	NEG	POS	POS	POS	NEG	NEG	NEG	NEG	NEG	NEG	NEG	NEG	NEG	NEG	NEG	NEG	NEG	NEG	NEG	NEG	NEG
01V03483	CC45-MRSA-IV, Berlin EMRSA	MED-WARD	60	f	POS	POS	POS	POS	POS	POS	POS	POS	POS	POS	NEG	NEG	NEG	NEG	POS	POS	POS	POS	NEG	NEG	NEG	POS	POS	POS	NEG	NEG	NEG	NEG	NEG	NEG	NEG	NEG	NEG	NEG	NEG	NEG	NEG	NEG	NEG	NEG	NEG
01V04728	CC45-MRSA-IV, Berlin EMRSA	SUR-ICU	28	m	POS	POS	POS	POS	POS	POS	POS	POS	POS	POS	POS	NEG	NEG	NEG	POS	POS	POS	POS	NEG	NEG	NEG	POS	POS	POS	NEG	NEG	NEG	NEG	NEG	NEG	NEG	NEG	NEG	NEG	NEG	NEG	NEG	NEG	NEG	NEG	NEG
01V05972	CC45-MRSA-IV, Berlin EMRSA	SUR-WARD	32	m	POS	POS	POS	POS	POS	POS	POS	POS	POS	POS	POS	NEG	NEG	NEG	POS	POS	POS	POS	NEG	NEG	NEG	POS	POS	POS	NEG	NEG	NEG	NEG	NEG	NEG	NEG	NEG	NEG	NEG	NEG	NEG	NEG	NEG	NEG	NEG	NEG
01V07139	CC45-MRSA-IV, Berlin EMRSA	SUR-ICU	77	f	POS	POS	POS	POS	POS	POS	POS	POS	POS	POS	POS	NEG	NEG	NEG	POS	POS	POS	POS	NEG	NEG	NEG	POS	POS	POS	NEG	NEG	NEG	NEG	NEG	NEG	NEG	NEG	NEG	NEG	NEG	NEG	NEG	NEG	NEG	NEG	NEG
01V08133	CC45-MRSA-IV, Berlin EMRSA	MED-OPD	71	m	POS	POS	POS	POS	POS	POS	POS	POS	POS	POS	POS	NEG	NEG	NEG	POS	POS	POS	POS	NEG	NEG	NEG	POS	POS	POS	NEG	NEG	NEG	NEG	NEG	NEG	NEG	NEG	NEG	NEG	NEG	NEG	NEG	NEG	NEG	NEG	NEG
01V09872	CC45-MRSA-IV, Berlin EMRSA	MED-OPD	77	f	POS	POS	POS	POS	POS	POS	POS	POS	POS	POS	POS	NEG	NEG	NEG	POS	POS	POS	POS	NEG	NEG	NEG	POS	POS	POS	NEG	NEG	NEG	NEG	NEG	NEG	NEG	NEG	NEG	NEG	NEG	NEG	NEG	NEG	NEG	NEG	NEG
01V12465	CC45-MRSA-IV, Berlin EMRSA	MED-OPD	60	m	POS	POS	POS	POS	POS	AMB	POS	POS	POS	POS	POS	NEG	NEG	NEG	POS	POS	POS	POS	NEG	NEG	NEG	POS	POS	POS	NEG	NEG	NEG	NEG	NEG	NEG	NEG	NEG	NEG	NEG	NEG	NEG	NEG	NEG	NEG	NEG	NEG
01V16151	CC45-MRSA-IV, Berlin EMRSA	MED-OPD	72	m	POS	POS	POS	POS	POS	AMB	POS	POS	POS	POS	POS	NEG	NEG	NEG	POS	POS	POS	POS	NEG	NEG	NEG	POS	POS	POS	NEG	NEG	NEG	NEG	NEG	NEG	NEG	NEG	NEG	NEG	NEG	NEG	NEG	NEG	NEG	NEG	NEG
01V16873	CC45-MRSA-IV, Berlin EMRSA	MED-WARD	63	m	POS	POS	POS	POS	POS	POS	POS	POS	POS	POS	POS	NEG	NEG	NEG	POS	POS	POS	POS	NEG	NEG	NEG	POS	POS	POS	NEG	NEG	NEG	NEG	NEG	NEG	NEG	NEG	NEG	NEG	NEG	NEG	NEG	NEG	NEG	NEG	NEG
01V17796	CC45-MRSA-IV, Berlin EMRSA	SUR-WARD	26	m	POS	POS	POS	POS	POS	POS	POS	POS	POS	POS	POS	NEG	NEG	NEG	POS	POS	POS	POS	NEG	NEG	NEG	POS	POS	POS	NEG	NEG	NEG	NEG	NEG	NEG	NEG	NEG	NEG	NEG	NEG	NEG	NEG	NEG	NEG	NEG	NEG
01V18681	CC45-MRSA-IV, Berlin EMRSA	MED-WARD	74	f	POS	POS	POS	POS	POS	POS	POS	POS	POS	POS	POS	NEG	NEG	NEG	POS	POS	POS	POS	NEG	NEG	NEG	POS	POS	POS	NEG	NEG	NEG	NEG	NEG	NEG	NEG	NEG	NEG	NEG	NEG	NEG	NEG	NEG	NEG	NEG	NEG
01V20066	CC45-MRSA-IV, Berlin EMRSA	MED-WARD	78	f	POS	POS	POS	POS	POS	POS	POS	POS	POS	POS	POS	NEG	NEG	NEG	POS	POS	POS	POS	NEG	NEG	NEG	POS	POS	POS	NEG	NEG	NEG	NEG	NEG	NEG	NEG	NEG	NEG	NEG	NEG	NEG	NEG	NEG	NEG	NEG	NEG
01V23050	CC45-MRSA-IV, Berlin EMRSA	SUR-WARD	65	m	POS	POS	POS	POS	POS	POS	POS	POS	POS	POS	POS	NEG	NEG	NEG	POS	POS	POS	POS	NEG	NEG	NEG	POS	POS	POS	NEG	NEG	NEG	NEG	NEG	NEG	NEG	NEG	NEG	NEG	NEG	NEG	NEG	NEG	NEG	NEG	NEG
01V25584	CC45-MRSA-IV, Berlin EMRSA	SUR-WARD	61	m	POS	AMB	POS	POS	POS	NEG	AMB	POS	POS	POS	NEG	NEG	NEG	NEG	POS	POS	POS	POS	NEG	NEG	NEG	AMB	POS	POS	NEG	NEG	NEG	NEG	NEG	NEG	NEG	NEG	NEG	NEG	NEG	NEG	NEG	NEG	NEG	NEG	NEG
01V31705	CC45-MRSA-IV, Berlin EMRSA	MED-WARD	62	m	POS	POS	POS	POS	POS	POS	POS	POS	POS	POS	POS	NEG	NEG	NEG	POS	POS	POS	POS	NEG	NEG	NEG	POS	POS	POS	NEG	NEG	NEG	NEG	NEG	NEG	NEG	NEG	NEG	NEG	NEG	NEG	NEG	NEG	NEG	NEG	NEG
01V34968	CC45-MRSA-IV, Berlin EMRSA	SUR-WARD	82	f	POS	POS	POS	POS	POS	POS	POS	POS	POS	POS	POS	NEG	NEG	NEG	POS	POS	POS	POS	NEG	NEG	NEG	POS	POS	POS	NEG	NEG	NEG	NEG	NEG	NEG	NEG	NEG	NEG	NEG	NEG	NEG	NEG	NEG	NEG	NEG	NEG
01V36186	CC45-MRSA-IV, Berlin EMRSA	MED-ICU	61	m	POS	POS	POS	POS	POS	POS	POS	POS	POS	POS	AMB	NEG	NEG	NEG	POS	POS	POS	POS	NEG	NEG	NEG	AMB	POS	POS	NEG	NEG	NEG	NEG	NEG	NEG	NEG	NEG	NEG	NEG	NEG	NEG	NEG	NEG	NEG	NEG	NEG
01V36534	CC45-MRSA-IV, Berlin EMRSA	SUR-ICU	42	f	POS	POS	POS	POS	POS	POS	POS	POS	POS	POS	POS	NEG	NEG	NEG	POS	POS	POS	POS	NEG	NEG	NEG	POS	POS	POS	NEG	NEG	NEG	NEG	NEG	NEG	NEG	NEG	NEG	NEG	NEG	NEG	NEG	NEG	NEG	NEG	NEG
01V36588	CC45-MRSA-IV, Berlin EMRSA	MED-OPD	47	f	POS	POS	POS	POS	POS	POS	POS	POS	POS	POS	POS	NEG	NEG	NEG	POS	POS	POS	POS	NEG	NEG	NEG	POS	POS	POS	NEG	NEG	NEG	NEG	NEG	NEG	NEG	NEG	NEG	NEG	NEG	NEG	NEG	NEG	NEG	NEG	NEG
01V37990	CC45-MRSA-IV, Berlin EMRSA	MED-WARD	55	m	POS	POS	POS	POS	POS	POS	POS	POS	POS	POS	POS	NEG	NEG	NEG	POS	POS	POS	POS	NEG	NEG	NEG	POS	POS	POS	NEG	NEG	NEG	NEG	NEG	NEG	NEG	NEG	NEG	NEG	NEG	NEG	NEG	NEG	NEG	NEG	NEG
01V39610	CC45-MRSA-IV, Berlin EMRSA	MED-WARD	61	f	POS	POS	POS	POS	POS	POS	POS	POS	POS	POS	POS	NEG	NEG	NEG	POS	POS	POS	POS	NEG	AMB	NEG	POS	POS	POS	NEG	NEG	NEG	NEG	NEG	NEG	NEG	NEG	NEG	NEG	NEG	NEG	NEG	NEG	NEG	NEG	NEG
01V40229	CC45-MRSA-IV, Berlin EMRSA	SUR-WARD	65	m	POS	POS	POS	POS	POS	POS	POS	POS	POS	POS	POS	NEG	NEG	NEG	POS	POS	POS	POS	NEG	NEG	NEG	POS	POS	POS	NEG	NEG	NEG	NEG	NEG	NEG	NEG	NEG	NEG	NEG	NEG	NEG	NEG	NEG	NEG	NEG	NEG
01V40374	CC45-MRSA-IV, Berlin EMRSA	MED-OPD	60	m	POS	POS	POS	POS		POS	POS	POS			POS	NEG	NEG	NEG	POS	POS	POS	POS	NEG	NEG	NEG	POS	POS	POS	NEG	NEG	NEG	NEG	NEG	NEG	NEG	NEG	NEG	NEG	NEG	NEG	NEG	NEG	NEG	NEG	NEG
01V41056	CC45-MRSA-IV, Berlin EMRSA	MED-OPD	37	m	POS	POS	POS	POS	POS	POS	POS	POS	POS	POS	AMB	NEG	NEG	NEG	POS	POS	POS	POS	NEG	NEG	NEG	AMB	POS	POS	NEG	NEG	NEG	NEG	NEG	NEG	NEG	NEG	NEG	NEG	NEG	NEG	NEG	NEG	NEG	NEG	NEG
01V42009	CC45-MRSA-IV, Berlin EMRSA	MED-WARD	80	f	POS	POS	POS	POS	POS	POS	POS	POS	POS	POS	POS	NEG	NEG	NEG	POS	POS	POS	POS	NEG	NEG	NEG	POS	POS	POS	NEG	NEG	NEG	NEG	NEG	NEG	NEG	NEG	NEG	NEG	NEG	NEG	NEG	NEG	NEG	NEG	NEG
01V43511	CC45-MRSA-IV, Berlin EMRSA	SUR-WARD	60	m	POS	POS	POS	POS	POS	POS	POS	POS	POS	POS	AMB	NEG	NEG	NEG	POS	POS	POS	POS	NEG	NEG	NEG	AMB	POS	POS	NEG	NEG	NEG	NEG	NEG	NEG	NEG	NEG	NEG	NEG	NEG	NEG	NEG	NEG	NEG	NEG	NEG
**2002**																																													
02V10698	CC5/ST228-MRSA-I, South German EMRSA	SUR-WARD	30	m	POS	POS	POS	POS	POS	POS	POS	POS	POS	POS	NEG	POS	NEG	NEG	POS	POS	POS	POS	POS	POS	POS	AMB	NEG	NEG	NEG	NEG	NEG	NEG	NEG	NEG	NEG	NEG	NEG	NEG	NEG	NEG	NEG	NEG	NEG	NEG	NEG
02V13140	CC5/ST228-MRSA-I, South German EMRSA	MED-ICU	80	m	POS	POS	POS	POS	POS	POS	POS	POS	POS	POS	NEG	POS	NEG	NEG	POS	POS	POS	POS	POS	POS	POS	POS	NEG	NEG	NEG	NEG	NEG	NEG	NEG	NEG	NEG	NEG	NEG	NEG	POS	POS	NEG	NEG	NEG	NEG	NEG
02V20759	CC5/ST228-MRSA-I, South German EMRSA	SUR-WARD	14	m	POS	POS	POS	POS	POS	POS	POS	POS	POS	POS	NEG	POS	NEG	NEG	POS	POS	POS	POS	POS	POS	POS	POS	NEG	NEG	NEG	NEG	NEG	NEG	NEG	NEG	NEG	NEG	NEG	NEG	POS	POS	NEG	NEG	NEG	NEG	NEG
02B06271	CC5-MRSA-II, Rhine-Hesse EMRSA/UK-EMRSA-3	MED-WARD	67	m	POS	POS	POS	POS	POS	POS	POS	POS	POS	POS	NEG	POS	NEG	NEG	POS	POS	POS	POS	NEG	NEG	NEG	POS	POS	POS	POS	POS	POS	POS	POS	POS	POS	POS	NEG	NEG	NEG	NEG	NEG	NEG	NEG	NEG	NEG
02V04116	CC5-MRSA-II, Rhine-Hesse EMRSA/UK-EMRSA-3	MED-ICU	58	m	POS	POS	POS	POS		POS	POS	POS	POS	POS	NEG	POS	NEG	NEG	POS	POS	POS	POS	NEG	NEG	NEG	POS	POS	POS	POS	POS	POS	POS	POS	POS	POS	POS	NEG	NEG	NEG	NEG	NEG	NEG	NEG	NEG	NEG
02V29922	CC5-MRSA-II, Rhine-Hesse EMRSA/UK-EMRSA-3	MED-ICU	56	m	POS	POS	POS	POS		POS	POS	POS	POS	POS	NEG	POS	NEG	NEG	POS	POS	POS	POS	NEG	NEG	NEG	POS	POS	POS	POS	POS	POS	POS	POS	POS	POS	POS	NEG	NEG	NEG	NEG	NEG	NEG	NEG	NEG	NEG
02V33634	CC5-MRSA-II, Rhine-Hesse EMRSA/UK-EMRSA-3	MED-WARD	70	m	POS	POS	POS	POS	POS	POS	POS	POS	POS	POS	NEG	POS	NEG	NEG	POS	POS	POS	POS	NEG	NEG	NEG	POS	POS	POS	POS	POS	POS	POS	POS	POS	POS	POS	NEG	NEG	NEG	NEG	NEG	NEG	NEG	NEG	NEG
02V34748	CC5-MRSA-II, Rhine-Hesse EMRSA/UK-EMRSA-3	MED-WARD	62	m	POS	POS	POS	POS	POS	POS	POS	POS	POS	POS	NEG	POS	NEG	NEG	POS	POS	POS	POS	NEG	NEG	NEG	POS	POS	POS	POS	POS	POS	POS	POS	POS	POS	POS	NEG	NEG	NEG	NEG	NEG	NEG	NEG	NEG	NEG
02V17002	CC8/ST254-MRSA-(atypical SCCmec), Hannover EMRSA	MED-WARD	25	f	POS	POS	POS	POS	POS	POS	POS	POS	POS	POS	POS	NEG	NEG	NEG	POS	POS	POS	POS	NEG	NEG	NEG	POS	NEG	NEG	NEG	NEG	NEG	NEG	NEG	NEG	NEG	NEG	NEG	NEG	NEG	NEG	NEG	NEG	NEG	NEG	NEG
02V33579	CC8-MRSA-IV, USA500	SUR-ICU	42	m	POS	POS	POS	POS	POS	POS	POS	POS	POS	POS	POS	NEG	NEG	NEG	POS	POS	POS	POS	NEG	NEG	NEG	POS	POS	POS	NEG	NEG	NEG	NEG	NEG	NEG	NEG	NEG	NEG	NEG	POS	POS	NEG	NEG	NEG	NEG	NEG
02V15498	CC22-MRSA-IV [PVL+]	MED-WARD	62	m	POS	POS	POS	POS	POS	POS	POS	POS	POS	POS	POS	NEG	NEG	NEG	POS	POS	POS	POS	NEG	NEG	AMB	POS	POS	POS	NEG	NEG	NEG	NEG	NEG	NEG	NEG	NEG	NEG	NEG	NEG	NEG	NEG	NEG	NEG	NEG	NEG
02C05319	CC22-MRSA-IV, Barnim/UK-EMRSA-15	MED-ICU	73	m	POS	POS	POS	POS	POS	POS	POS	POS	POS	POS	POS	NEG	NEG	NEG	POS	POS	POS	POS	NEG	NEG	NEG	POS	POS	POS	NEG	NEG	NEG	NEG	NEG	NEG	NEG	NEG	NEG	NEG	NEG	NEG	NEG	NEG	NEG	NEG	NEG
02U10255	CC22-MRSA-IV, Barnim/UK-EMRSA-15	MED-WARD	0	m	POS	POS	POS	POS	POS	POS	POS	POS	POS	POS	POS	NEG	NEG	NEG	POS	POS	POS	POS	NEG	NEG	NEG	POS	POS	POS	NEG	NEG	NEG	NEG	NEG	NEG	NEG	NEG	NEG	NEG	NEG	NEG	NEG	NEG	NEG	NEG	NEG
02V12175	CC22-MRSA-IV, Barnim/UK-EMRSA-15	SUR-WARD	82	f	POS	POS	POS	POS	POS	POS	POS	POS	POS	POS	POS	NEG	NEG	NEG	POS	POS	POS	POS	NEG	NEG	NEG	AMB	POS	POS	NEG	NEG	NEG	NEG	NEG	NEG	NEG	NEG	NEG	NEG	NEG	NEG	NEG	NEG	NEG	NEG	NEG
02V13054	CC22-MRSA-IV, Barnim/UK-EMRSA-15		#WERT!	m	POS	POS	POS	POS	POS	POS	POS	POS	POS	POS	POS	NEG	NEG	NEG	POS	POS	POS	POS	NEG	NEG	NEG	POS	POS	POS	NEG	NEG	NEG	NEG	NEG	NEG	NEG	NEG	NEG	NEG	NEG	NEG	NEG	NEG	NEG	NEG	NEG
02V13950	CC22-MRSA-IV, Barnim/UK-EMRSA-15	MED-WARD	73	m	POS	POS	POS	POS	POS	POS	POS	POS	POS	POS	POS	NEG	NEG	NEG	POS	POS	POS	POS	NEG	NEG	NEG	AMB	POS	POS	NEG	NEG	NEG	NEG	NEG	NEG	NEG	NEG	NEG	NEG	NEG	NEG	NEG	NEG	NEG	NEG	NEG
02V21259	CC22-MRSA-IV, Barnim/UK-EMRSA-15	MED-WARD	70	m	POS	POS	POS	POS	POS	POS	POS	POS	POS	POS	POS	NEG	NEG	NEG	POS	POS	POS	POS	NEG	NEG	NEG	AMB	POS	POS	NEG	NEG	NEG	NEG	NEG	NEG	NEG	NEG	NEG	NEG	NEG	NEG	NEG	NEG	NEG	NEG	NEG
02V22657	CC22-MRSA-IV, Barnim/UK-EMRSA-15	SUR-WARD	81	m	POS	POS	POS	AMB	POS	POS	POS	POS	POS	POS	POS	NEG	NEG	NEG	POS	POS	POS	POS	NEG	NEG	NEG	POS	POS	POS	NEG	NEG	NEG	NEG	NEG	NEG	NEG	NEG	NEG	NEG	NEG	NEG	NEG	NEG	NEG	NEG	NEG
02V29051	CC22-MRSA-IV, Barnim/UK-EMRSA-15	MED-ICU	65	m	POS	POS	POS	POS	POS	POS	POS	POS	POS	POS	POS	NEG	NEG	NEG	POS	POS	POS	POS	NEG	NEG	NEG	POS	POS	POS	NEG	NEG	NEG	NEG	NEG	NEG	NEG	NEG	NEG	NEG	NEG	NEG	NEG	NEG	NEG	NEG	NEG
02V30792	CC30/ST36-MRSA-II, UK-EMRSA-16	MED-WARD	39	f	POS	POS	POS	POS	POS	POS	POS	POS	POS	POS	NEG	NEG	POS	NEG	POS	POS	POS	POS	NEG	NEG	NEG	POS	POS	POS	POS	POS	POS	POS	POS	POS	POS	POS	NEG	NEG	NEG	NEG	NEG	NEG	NEG	NEG	NEG
02U07096	CC45-MRSA-IV, Berlin EMRSA	MED-WARD	37	f	POS	POS	POS	POS	POS	POS	POS	POS	POS	POS	POS	NEG	NEG	NEG	POS	POS	POS	POS	NEG	NEG	NEG	POS	POS	POS	NEG	NEG	NEG	NEG	NEG	NEG	NEG	NEG	NEG	NEG	NEG	NEG	NEG	NEG	NEG	NEG	NEG
02V00570	CC45-MRSA-IV, Berlin EMRSA	MED-WARD	98	m	POS	POS	POS	POS	POS	POS	POS	POS	POS	POS	POS	NEG	NEG	NEG	POS	POS	POS	POS	NEG	NEG	NEG	AMB	POS	POS	NEG	NEG	NEG	NEG	NEG	NEG	NEG	NEG	NEG	NEG	NEG	NEG	NEG	NEG	NEG	NEG	NEG
02V01730	CC45-MRSA-IV, Berlin EMRSA	MED-OPD	84	m	POS	POS	POS	POS	POS	POS	POS	POS	POS	POS	AMB	NEG	NEG	NEG	POS	POS	POS	POS	NEG	NEG	NEG	AMB	POS	POS	NEG	NEG	NEG	NEG	NEG	NEG	NEG	NEG	NEG	NEG	NEG	NEG	NEG	NEG	NEG	NEG	NEG
02V01870	CC45-MRSA-IV, Berlin EMRSA	SUR-ICU	62	m	POS	POS	POS	POS	POS	AMB	POS	POS	POS	POS	POS	NEG	NEG	NEG	POS	POS	POS	POS	NEG	NEG	NEG	POS	POS	POS	NEG	NEG	NEG	NEG	NEG	NEG	NEG	NEG	NEG	NEG	NEG	NEG	NEG	NEG	NEG	NEG	NEG
02V04750	CC45-MRSA-IV, Berlin EMRSA	SUR-ICU	25	m	POS	POS	POS	POS	POS	POS	POS	POS	POS	POS	POS	NEG	NEG	NEG	POS	POS	POS	POS	NEG	NEG	NEG	POS	POS	POS	NEG	NEG	NEG	NEG	NEG	NEG	NEG	NEG	NEG	NEG	NEG	NEG	NEG	NEG	NEG	NEG	NEG
02V06592	CC45-MRSA-IV, Berlin EMRSA	SUR-ICU	53	m	POS	POS	POS	POS	POS	POS	POS	POS	POS	POS	POS	NEG	NEG	NEG	POS	POS	POS	POS	NEG	NEG	NEG	AMB	POS	POS	NEG	NEG	NEG	NEG	NEG	NEG	NEG	NEG	NEG	NEG	NEG	NEG	NEG	NEG	NEG	NEG	NEG
02V10868	CC45-MRSA-IV, Berlin EMRSA	SUR-ICU	41	m	POS	POS	POS	POS	POS	POS	POS	POS	POS	POS	POS	NEG	NEG	NEG	POS	POS	POS	POS	NEG	NEG	NEG	AMB	POS	POS	NEG	NEG	NEG	NEG	NEG	NEG	NEG	NEG	NEG	NEG	NEG	NEG	NEG	NEG	NEG	NEG	NEG
02V12394	CC45-MRSA-IV, Berlin EMRSA	MED-OPD	51	m	POS	POS	POS	POS	POS	POS	POS	POS	POS	POS	POS	NEG	NEG	NEG	POS	POS	POS	POS	NEG	NEG	NEG	POS	POS	POS	NEG	NEG	NEG	NEG	NEG	NEG	NEG	NEG	NEG	NEG	NEG	NEG	NEG	NEG	NEG	NEG	NEG
02V12942	CC45-MRSA-IV, Berlin EMRSA	SUR-ICU	66	f	POS	POS	POS	POS	POS	POS	POS	POS	POS	POS	POS	NEG	NEG	NEG	POS	POS	POS	POS	NEG	NEG	NEG	POS	POS	POS	NEG	NEG	NEG	NEG	NEG	NEG	NEG	NEG	NEG	NEG	NEG	NEG	NEG	NEG	NEG	NEG	NEG
02V13026	CC45-MRSA-IV, Berlin EMRSA	SUR-ICU	38	m	POS	POS	POS	POS	POS	POS	POS	POS	POS	POS	POS	NEG	NEG	NEG	POS	POS	POS	POS	NEG	NEG	NEG	POS	POS	POS	NEG	NEG	NEG	NEG	NEG	NEG	NEG	NEG	NEG	NEG	NEG	NEG	NEG	NEG	NEG	NEG	NEG
02V14700	CC45-MRSA-IV, Berlin EMRSA	MED-WARD	73	m	POS	POS	POS	POS	POS	POS	POS	POS	POS	POS	POS	NEG	NEG	NEG	POS	POS			NEG	NEG	NEG	POS	POS	POS	NEG	NEG	NEG	NEG	NEG	NEG	NEG	NEG	NEG	NEG	NEG	NEG	NEG	NEG	NEG	NEG	NEG
02V15358	CC45-MRSA-IV, Berlin EMRSA	MED-OPD	68	f	POS	POS	POS	POS	POS	POS	POS	POS	POS	POS	POS	NEG	NEG	NEG	POS	POS	POS	POS	NEG	NEG	NEG	AMB	POS	POS	NEG	NEG	NEG	NEG	NEG	NEG	NEG	NEG	NEG	NEG	NEG	NEG	NEG	NEG	NEG	NEG	NEG
02V16654	CC45-MRSA-IV, Berlin EMRSA	MED-OPD	78	f	POS	POS	POS	POS	POS	POS	POS	POS	POS	POS	POS	NEG	NEG	NEG	POS	POS	POS	POS	NEG	NEG	NEG	AMB	POS	POS	NEG	NEG	NEG	NEG	NEG	NEG	NEG	NEG	NEG	NEG	NEG	NEG	NEG	NEG	NEG	NEG	NEG
02V17120	CC45-MRSA-IV, Berlin EMRSA	SUR-WARD	41	m	POS	POS	POS	POS	POS	POS	POS	POS	POS	POS	POS	NEG	NEG	NEG	POS	POS	POS	POS	NEG	AMB	NEG	AMB	POS	POS	NEG	NEG	NEG	NEG	NEG	NEG	NEG	NEG	NEG	NEG	NEG	NEG	NEG	NEG	NEG	NEG	NEG
02V17136	CC45-MRSA-IV, Berlin EMRSA	MED-OPD	77	f	POS	POS	POS	POS	POS	POS	POS	POS	POS	POS	POS	NEG	NEG	NEG	POS	POS	POS	POS	NEG	NEG	NEG	POS	POS	POS	NEG	NEG	NEG	NEG	NEG	NEG	NEG	NEG	NEG	NEG	NEG	NEG	NEG	NEG	NEG	NEG	NEG
02V17558	CC45-MRSA-IV, Berlin EMRSA	MED-OPD	42	f	POS	POS	POS	POS	POS	POS	POS	POS	POS	POS	POS	NEG	NEG	NEG	POS	POS	POS	POS	NEG	NEG	NEG	AMB	POS	POS	NEG	NEG	NEG	NEG	NEG	NEG	NEG	NEG	NEG	NEG	NEG	NEG	NEG	NEG	NEG	NEG	NEG
02V17665	CC45-MRSA-IV, Berlin EMRSA	MED-WARD	58	f	POS	POS	POS	POS	POS	POS	POS	POS	POS	POS	POS	NEG	NEG	NEG	POS	POS	POS	POS	NEG	NEG	NEG	POS	POS	POS	NEG	NEG	NEG	NEG	NEG	NEG	NEG	NEG	NEG	NEG	NEG	NEG	NEG	NEG	NEG	NEG	NEG
02V20390	CC45-MRSA-IV, Berlin EMRSA	MED-OPD	41	m	POS	POS	POS	POS	POS	AMB	POS	POS	POS	POS	AMB	NEG	NEG	NEG	POS	POS	POS	POS	NEG	NEG	NEG	AMB	POS	POS	NEG	NEG	NEG	NEG	NEG	NEG	NEG	NEG	NEG	NEG	NEG	NEG	NEG	NEG	NEG	NEG	NEG
02V20626	CC45-MRSA-IV, Berlin EMRSA	SUR-WARD	77	f	POS	POS	POS	POS	POS	POS	POS	POS	POS	POS	POS	NEG	NEG	NEG	POS	POS	POS	POS	NEG	NEG	NEG	POS	POS	POS	NEG	NEG	NEG	NEG	NEG	NEG	NEG	NEG	NEG	NEG	NEG	NEG	NEG	NEG	NEG	NEG	NEG
02V20680	CC45-MRSA-IV, Berlin EMRSA	SUR-ICU	71	f	POS	POS	POS	POS	POS	POS	POS	POS	POS	POS	POS	NEG	NEG	NEG	POS	POS	POS	POS	NEG	NEG	NEG	POS	POS	POS	NEG	NEG	NEG	NEG	NEG	NEG	NEG	NEG	NEG	NEG	NEG	NEG	NEG	NEG	NEG	NEG	NEG
02V21980	CC45-MRSA-IV, Berlin EMRSA	MED-WARD	81	f	POS	POS	POS	POS	POS	AMB	POS	POS	POS	POS	POS	NEG	NEG	NEG	POS	POS	POS	POS	NEG	NEG	NEG	POS	POS	POS	NEG	NEG	NEG	NEG	NEG	NEG	NEG	NEG	NEG	NEG	NEG	NEG	NEG	NEG	NEG	NEG	NEG
02V22570	CC45-MRSA-IV, Berlin EMRSA	MED-WARD	71	f	POS	POS	POS	POS	POS	POS	POS	POS	POS	POS	POS	NEG	NEG	NEG	POS	POS	POS	POS	NEG	NEG	NEG	POS	POS	POS	NEG	NEG	NEG	NEG	NEG	NEG	NEG	NEG	NEG	NEG	NEG	NEG	NEG	NEG	NEG	NEG	NEG
02V22982	CC45-MRSA-IV, Berlin EMRSA	SUR-WARD	25	f	POS	POS	POS	POS	POS	POS	POS	POS	POS	POS	POS	NEG	NEG	NEG	POS	POS	POS	POS	NEG	NEG	NEG	POS	POS	POS	NEG	NEG	NEG	NEG	NEG	NEG	NEG	NEG	NEG	NEG	NEG	NEG	NEG	NEG	NEG	NEG	NEG
02V25503	CC45-MRSA-IV, Berlin EMRSA	MED-WARD	58	m	POS	POS	POS	POS	POS	POS	POS	POS	POS	POS	POS	NEG	NEG	NEG	POS	POS	POS	POS	NEG	NEG	NEG	POS	POS	POS	NEG	NEG	NEG	NEG	NEG	NEG	NEG	NEG	NEG	NEG	NEG	NEG	NEG	NEG	NEG	NEG	NEG
02V28454	CC45-MRSA-IV, Berlin EMRSA	SUR-ICU	75	m	POS	POS	POS	POS	POS	POS	POS	POS	POS	POS	POS	NEG	NEG	NEG	POS	POS	POS	POS	NEG	NEG	NEG	POS	POS	POS	NEG	NEG	NEG	NEG	NEG	NEG	NEG	NEG	NEG	NEG	NEG	NEG	NEG	NEG	NEG	NEG	NEG
02V29373	CC45-MRSA-IV, Berlin EMRSA	MED-WARD	78	m	POS	POS	POS	POS	POS	POS	POS	POS	POS	POS	POS	NEG	NEG	NEG	POS	POS	POS	POS	NEG	NEG	NEG	POS	POS	POS	NEG	NEG	NEG	NEG	NEG	NEG	NEG	NEG	NEG	NEG	NEG	NEG	NEG	NEG	NEG	NEG	NEG
02V30321	CC45-MRSA-IV, Berlin EMRSA	MED-OPD	70	m	POS	POS	POS	POS	POS	POS	POS	POS	POS	POS	POS	NEG	NEG	NEG	POS	POS	POS	POS	NEG	NEG	NEG	POS	POS	POS	NEG	NEG	NEG	NEG	NEG	NEG	NEG	NEG	NEG	NEG	NEG	NEG	NEG	NEG	NEG	NEG	NEG
02V30341	CC45-MRSA-IV, Berlin EMRSA	MED-WARD	78	m	POS	POS	POS	POS	POS	POS	POS	POS	POS	POS	POS	NEG	NEG	NEG	POS	POS	POS	POS	NEG	NEG	NEG	POS	POS	POS	NEG	NEG	NEG	NEG	NEG	NEG	NEG	NEG	NEG	NEG	NEG	NEG	NEG	NEG	NEG	NEG	NEG
02V31377	CC45-MRSA-IV, Berlin EMRSA	MED-OPD	53	m	POS	POS	POS	POS	POS	POS	POS	POS	POS	POS	POS	NEG	NEG	NEG	POS	POS	POS	POS	NEG	NEG	NEG	POS	POS	POS	NEG	NEG	NEG	NEG	NEG	NEG	NEG	NEG	NEG	NEG	NEG	NEG	NEG	NEG	NEG	NEG	NEG
02V31941	CC45-MRSA-IV, Berlin EMRSA	MED-WARD	62	m	POS	POS	POS	POS	POS	POS	POS	POS	POS	POS	POS	NEG	NEG	NEG	POS	POS	POS	POS	NEG	NEG	NEG	POS	POS	POS	NEG	NEG	NEG	NEG	NEG	NEG	NEG	NEG	NEG	NEG	NEG	NEG	NEG	NEG	NEG	NEG	NEG
02V34679	CC45-MRSA-IV, Berlin EMRSA	MED-OPD	57	m	POS	POS	POS	POS	POS	AMB	POS	POS	POS	POS	AMB	NEG	NEG	NEG	POS	POS	POS	POS	NEG	NEG	NEG	POS	POS	POS	NEG	NEG	NEG	NEG	NEG	NEG	NEG	NEG	NEG	NEG	NEG	NEG	NEG	NEG	NEG	NEG	NEG
02V35779	CC45-MRSA-IV, Berlin EMRSA	MED-OPD	47	f	POS	POS	POS	POS	POS	POS	POS	POS	POS	POS	POS	NEG	NEG	NEG	POS	POS	POS	POS	NEG	NEG	NEG	POS	POS	POS	NEG	NEG	NEG	NEG	NEG	NEG	NEG	NEG	NEG	NEG	NEG	NEG	NEG	NEG	NEG	NEG	NEG
02V36419	CC45-MRSA-IV, Berlin EMRSA	MED-WARD	63	m	POS	POS	POS	POS	POS	POS	POS	POS			POS	NEG	NEG	NEG	POS	POS	POS	POS	NEG	NEG	NEG	POS	POS	POS	NEG	NEG	NEG	NEG	NEG	NEG	NEG	NEG	NEG	NEG	NEG	NEG	NEG	NEG	NEG	NEG	NEG
02V36469	CC45-MRSA-IV, Berlin EMRSA	MED-ICU	62	m	POS	POS	POS	POS	POS	POS	POS	POS	POS	POS	POS	NEG	NEG	NEG	POS	POS	POS	POS	NEG	NEG	NEG	POS	POS	POS	NEG	NEG	NEG	NEG	NEG	NEG	NEG	NEG	NEG	NEG	NEG	NEG	NEG	NEG	NEG	NEG	NEG

ISOLATE:	STRAIN	RESISTANCE : PENICILLINASE			RESISTANCE : MLS-ANTIBIOTICS												RESISTANCE : AMINOGLYSOSIDES			RESISTANCE : MISCELLANEOUS GENES																RESISTANCE : EFFLUX SYSTEMS								RESISTANCE : GLYCOPEPTIDES		
		beta-lactamase	beta lactamase repressor (inhibitor)	beta-lactamase regulatory protein	rRNA adenine N-6-methyl-transferase, erythromycin/clindamycin resistance	erythro-mycin/clindamycin resistance	erythro-mycin/clindamycin resistance	Linco-samid-Nucleo-tidyltrans-ferase	energy-dependent efflux of erythro-mycin	macrolide efflux protein A	probable lysylphos-phatidyl-glycerol synthetase	virginia-mycin A acetyltransferase	acetyl-transferase inactivating streptogramin A	ATP binding protein, streptogramin-A-resistance		virginia-mycin B hydrolase	bifunctional enzyme Aac/Aph, gentamicin resistance	amino-glycoside adenyl-transferase,tobramycin resistance	3'5'-aminog-lycoside phospho-transferase, neo-/ kanamycin resistance	strepto-thricine-acetyl-transferase	dihydro-folate reductase type 1	fusidic acid resistence	hypothetical protein associated with fusidic acid resistance	mupirocin resistence protein	tetrazyklin-resistance	tetracycline-resistance	chloramphenicol acetyltransferase					23S rRNA methyltransferase	chloramphenicol /florfenicol exporter	metallothiol transferase		quaternary ammonium compound resistance protein A	quaternary ammonium compound resistance protein C						Transport/ Efflux protein	vancomycin resistance gene	vancomycin resistance gene from enterococci and Clostridium	teicoplanin resistance gene from enterococci
		blaZ	blaI	blaR	erm(A)	erm(B)	erm(C)	lnu(A)	msr(A)	mef(A)	mph(C)	vat(A)	vat(B)	vga(A)	vga(A) (BM 3327)	vgB(A)	aacA-aphD	aadD	aphA3	sat	dfrA	far1	Q6GD50	mupR	tet(K)	tet(M)	cat (total)	cat (pC221)	cat (pc223)	cat (pMC524)	cat (pSBK203R)	cfr	fexA	fosB	fosB (plasmid)	qacA	qacC (total)	qacC (consensus)	qacC (equine)	qacC (SA5)	qacC (Ssap)	qacC (ST94)	tetEfflux	vanA	vanB	vanZ
**2000**																																														
00V07201	CC5/ST228-MRSA, South German EMRSA (truncated SCCmec element)	POS	POS	POS	POS	NEG	NEG	NEG	NEG	NEG	NEG	NEG	NEG	NEG	NEG	NEG	POS	NEG	POS	POS	NEG	NEG	NEG	NEG	NEG	NEG	NEG	NEG	NEG	NEG	NEG	NEG	NEG	POS	NEG	POS	NEG	NEG	NEG	NEG	NEG	NEG	POS	NEG	NEG	NEG
00V07634	CC5/ST228-MRSA, South German EMRSA (truncated SCCmec element)	POS	POS	POS	POS	NEG	NEG	NEG	NEG	NEG	NEG	NEG	NEG	NEG	NEG	NEG	POS	NEG	POS	POS	NEG	NEG	NEG	NEG	NEG	NEG	NEG	NEG	NEG	NEG	NEG	NEG	NEG	POS	NEG	POS	NEG	NEG	NEG	NEG	NEG	NEG	POS	NEG	NEG	NEG
00V08701	CC5/ST228-MRSA, South German EMRSA (truncated SCCmec element)	POS	POS	POS	POS	NEG	NEG	NEG	NEG	NEG	NEG	NEG	NEG	NEG	NEG	NEG	POS	NEG	POS	POS	NEG	NEG	NEG	NEG	NEG	NEG	NEG	NEG	NEG	NEG	NEG	NEG	NEG	POS	NEG	POS	NEG	NEG	NEG	NEG	NEG	NEG	POS	NEG	NEG	NEG
00V09056	CC5/ST228-MRSA, South German EMRSA (truncated SCCmec element)	POS	POS	POS	POS	NEG	NEG	NEG	NEG	NEG	NEG	NEG	NEG	NEG	NEG	NEG	POS	NEG	POS	POS	NEG	NEG	NEG	NEG	NEG	NEG	NEG	NEG	NEG	NEG	NEG	NEG	NEG	POS	NEG	POS	NEG	NEG	NEG	NEG	NEG	NEG	POS	NEG	NEG	NEG
00V10190	CC5/ST228-MRSA, South German EMRSA (truncated SCCmec element)	NEG	NEG	NEG	POS	NEG	NEG	NEG	NEG	NEG	NEG	NEG	NEG	NEG	NEG	NEG	POS	NEG	POS	POS	NEG	NEG	NEG	NEG	NEG	NEG	NEG	NEG	NEG	NEG	NEG	NEG	NEG	POS	AMB	NEG	NEG	NEG	NEG	NEG	NEG	NEG	POS	NEG	NEG	NEG
00V10643	CC5/ST228-MRSA, South German EMRSA (truncated SCCmec element)	POS	POS	POS	POS	NEG	NEG	NEG	NEG	NEG	NEG	NEG	NEG	NEG	NEG	NEG	POS	NEG	POS	POS	NEG	NEG	NEG	NEG	NEG	NEG	NEG	NEG	NEG	NEG	NEG	NEG	NEG	POS	NEG	POS	NEG	NEG	NEG	NEG	NEG	NEG	POS	NEG	NEG	NEG
00V11732	CC5/ST228-MRSA, South German EMRSA (truncated SCCmec element)	POS	POS	POS	POS	NEG	NEG	NEG	NEG	NEG	NEG	NEG	NEG	NEG	NEG	NEG	POS	NEG	POS	POS	NEG	NEG	NEG	NEG	NEG	NEG	NEG	NEG	NEG	NEG	NEG	NEG	NEG	POS	NEG	POS	NEG	NEG	NEG	NEG	NEG	NEG	POS	NEG	NEG	NEG
00V12300	CC5/ST228-MRSA, South German EMRSA (truncated SCCmec element)	POS	POS	POS	POS	NEG	NEG	NEG	NEG	NEG	NEG	NEG	NEG	NEG	NEG	NEG	POS	NEG	POS	POS	NEG	NEG	NEG	NEG	NEG	NEG	NEG	NEG	NEG	NEG	NEG	NEG	NEG	POS	NEG	POS	NEG	NEG	NEG	NEG	NEG	NEG	POS	NEG	NEG	NEG
00V14652	CC5/ST228-MRSA, South German EMRSA (truncated SCCmec element)	POS	POS	POS	POS	NEG	NEG	NEG	NEG	NEG	NEG	NEG	NEG	NEG	NEG	NEG	POS	NEG	POS	POS	NEG	NEG	NEG	NEG	NEG	NEG	NEG	NEG	NEG	NEG	NEG	NEG	NEG	POS	NEG	POS	NEG	NEG	NEG	NEG	NEG	NEG	POS	NEG	NEG	NEG
00V24941	CC5/ST228-MRSA, South German EMRSA (truncated SCCmec element)	POS	POS	POS	POS	NEG	NEG	NEG	NEG	NEG	NEG	NEG	NEG	NEG	NEG	NEG	POS	NEG	POS	POS	NEG	NEG	NEG	NEG	NEG	NEG	NEG	NEG	NEG	NEG	NEG	NEG	NEG	POS	NEG	POS	NEG	NEG	NEG	NEG	NEG	NEG	POS	NEG	NEG	NEG
00V29834	CC5/ST228-MRSA, South German EMRSA (truncated SCCmec element)	POS	POS	POS	POS	NEG	NEG	NEG	NEG	NEG	NEG	NEG	NEG	NEG	NEG	NEG	POS	NEG	POS	POS	NEG	NEG	NEG	NEG	NEG	NEG	NEG	NEG	NEG	NEG	NEG	NEG	NEG	POS	NEG	POS	NEG	NEG	NEG	NEG	NEG	NEG	POS	NEG	NEG	NEG
00V29909	CC5/ST228-MRSA, South German EMRSA (truncated SCCmec element)	POS	POS	POS	POS	NEG	NEG	NEG	NEG	NEG	NEG	NEG	NEG	NEG	NEG	NEG	AMB	NEG	POS	POS	NEG	NEG	NEG	NEG	NEG	NEG	NEG	NEG	NEG	NEG	NEG	NEG	NEG	POS	NEG	POS	NEG	NEG	NEG	NEG	NEG	NEG	POS	NEG	NEG	NEG
00V30456	CC5/ST228-MRSA, South German EMRSA (truncated SCCmec element)	POS	POS	POS	POS	NEG	NEG	NEG	NEG	NEG	NEG	NEG	NEG	NEG	NEG	NEG	AMB	NEG	POS	POS	NEG	NEG	NEG	NEG	NEG	NEG	NEG	NEG	NEG	NEG	NEG	NEG	NEG	POS	NEG	POS	NEG	NEG	NEG	NEG	NEG	NEG	POS	NEG	NEG	NEG
00V30827	CC5/ST228-MRSA, South German EMRSA (truncated SCCmec element)	POS	POS	POS	POS	NEG	NEG	NEG	NEG	NEG	NEG	NEG	NEG	NEG	NEG	NEG	POS	NEG	POS	POS	NEG	NEG	NEG	NEG	NEG	NEG	NEG	NEG	NEG	NEG	NEG	NEG	NEG	POS	NEG	POS	NEG	NEG	NEG	NEG	NEG	NEG	POS	NEG	NEG	NEG
00V33204	CC5/ST228-MRSA, South German EMRSA (truncated SCCmec element)	POS	POS	POS	POS	NEG	NEG	NEG	NEG	NEG	NEG	NEG	NEG	NEG	NEG	NEG	POS	NEG	POS	POS	NEG	NEG	NEG	NEG	NEG	NEG	NEG	NEG	NEG	NEG	NEG	NEG	NEG	POS	NEG	POS	NEG	NEG	NEG	NEG	NEG	NEG	POS	NEG	NEG	NEG
00V36490	CC5/ST228-MRSA, South German EMRSA (truncated SCCmec element)	POS	POS	POS	POS	NEG	NEG	NEG	NEG	NEG	NEG	NEG	NEG	NEG	NEG	NEG	POS	NEG	POS	POS	NEG	NEG	NEG	NEG	NEG	NEG	NEG	NEG	NEG	NEG	NEG	NEG	NEG	POS	NEG	POS	NEG	NEG	NEG	NEG	NEG	NEG	POS	NEG	NEG	NEG
00V37079	CC5/ST228-MRSA, South German EMRSA (truncated SCCmec element)	POS	POS	POS	POS	NEG	NEG	NEG	NEG	NEG	NEG	NEG	NEG	NEG	NEG	NEG	POS	NEG	POS	POS	NEG	NEG	NEG	NEG	NEG	NEG	NEG	NEG	NEG	NEG	NEG	NEG	NEG	POS	NEG	POS	NEG	NEG	NEG	NEG	NEG	NEG	POS	NEG	NEG	NEG
00V40116	CC5/ST228-MRSA, South German EMRSA (truncated SCCmec element)	POS	POS	POS	POS	NEG	NEG	NEG	NEG	NEG	NEG	NEG	NEG	NEG	NEG	NEG	POS	NEG	POS	POS	NEG	NEG	NEG	NEG	NEG	NEG	NEG	NEG	NEG	NEG	NEG	NEG	NEG	POS	NEG	NEG	NEG	NEG	NEG	NEG	NEG	NEG	POS	NEG	NEG	NEG
00V41429	CC5/ST228-MRSA, South German EMRSA (truncated SCCmec element)	POS	POS	POS	POS	NEG	NEG	NEG	NEG	NEG	NEG	NEG	NEG	NEG	NEG	NEG	POS	NEG	POS	POS	NEG	NEG	NEG	NEG	NEG	NEG	NEG	NEG	NEG	NEG	NEG	NEG	NEG	POS	NEG	POS	NEG	NEG	NEG	NEG	NEG	NEG	POS	NEG	NEG	NEG
00V42118	CC5/ST228-MRSA, South German EMRSA (truncated SCCmec element)	POS	POS	POS	POS	NEG	NEG	NEG	NEG	NEG	NEG	NEG	NEG	NEG	NEG	NEG	POS	NEG	POS	POS	NEG	NEG	NEG	NEG	NEG	NEG	NEG	NEG	NEG	NEG	NEG	NEG	NEG	POS	NEG	POS	NEG	NEG	NEG	NEG	NEG	NEG	POS	NEG	NEG	NEG
00V44135	CC5/ST228-MRSA, South German EMRSA (truncated SCCmec element)	POS	POS	POS	POS	NEG	NEG	NEG	NEG	NEG	NEG	NEG	NEG	NEG	NEG	NEG	POS	NEG	POS	POS	NEG	NEG	NEG	NEG	NEG	NEG	NEG	NEG	NEG	NEG	NEG	NEG	NEG	POS	NEG	POS	NEG	NEG	NEG	NEG	NEG	NEG	POS	NEG	NEG	NEG
00V44538	CC5/ST228-MRSA, South German EMRSA (truncated SCCmec element)	POS	POS	POS	POS	NEG	NEG	NEG	NEG	NEG	NEG	NEG	NEG	NEG	NEG	NEG	POS	NEG	POS	POS	NEG	NEG	NEG	NEG	NEG	NEG	NEG	NEG	NEG	NEG	NEG	NEG	NEG	POS	NEG	NEG	NEG	NEG	NEG	NEG	NEG	NEG	POS	NEG	NEG	NEG
00V44990	CC5/ST228-MRSA, South German EMRSA (truncated SCCmec element)	POS	POS	POS	POS	NEG	NEG	NEG	NEG	NEG	NEG	NEG	NEG	NEG	NEG	NEG	POS	NEG	POS	POS	NEG	NEG	NEG	NEG	NEG	NEG	NEG	NEG	NEG	NEG	NEG	NEG	NEG	POS	NEG	POS	NEG	NEG	NEG	NEG	NEG	NEG	POS	NEG	NEG	NEG
00V46910	CC5/ST228-MRSA, South German EMRSA (truncated SCCmec element)	POS	POS	POS	POS	NEG	NEG	NEG	NEG	NEG	NEG	NEG	NEG	NEG	NEG	NEG	POS	NEG	POS	POS	NEG	NEG	NEG	NEG	NEG	NEG	NEG	NEG	NEG	NEG	NEG	NEG	NEG	POS	NEG	POS	NEG	NEG	NEG	NEG	NEG	NEG	POS	NEG	NEG	NEG
00V07063	CC5/ST228-MRSA-I, South German EMRSA	POS	POS	POS	POS	NEG	NEG	NEG	NEG	NEG	NEG	NEG	NEG	AMB	NEG	NEG	POS	NEG	POS	POS	NEG	NEG	NEG	NEG	NEG	NEG	NEG	NEG	NEG	NEG	NEG	NEG	NEG	POS	AMB	POS	NEG	NEG	NEG	NEG	NEG	NEG	POS	NEG	NEG	NEG
00V46921	CC5-MRSA-II, Rhine-Hesse EMRSA/UK-EMRSA-3	POS	POS	POS	POS	NEG	NEG	NEG	NEG	NEG	NEG	NEG	NEG	NEG	NEG	NEG	NEG	POS	NEG	NEG	NEG	NEG	NEG	NEG	NEG	NEG	NEG	NEG	NEG	NEG	NEG	NEG	NEG	POS	NEG	NEG	NEG	NEG	NEG	NEG	NEG	NEG	POS	NEG	NEG	NEG
00V08510	CC8/ST254-MRSA-(atypical SCCmec), Hannover EMRSA	POS	POS	POS	POS		NEG	NEG	NEG	NEG	NEG	NEG	NEG	NEG	NEG	NEG	POS	NEG	POS	POS	NEG	NEG	NEG	NEG	NEG	POS	POS	NEG	NEG	POS	NEG	NEG	NEG	POS	NEG	NEG	NEG	NEG	NEG	NEG	NEG	NEG	POS	NEG	NEG	NEG
00V25538	CC8/ST254-MRSA-(atypical SCCmec), Hannover EMRSA	NEG	NEG	NEG	POS	NEG	NEG	NEG	NEG	NEG	NEG	NEG	NEG	NEG	NEG	NEG	POS	NEG	POS	POS	NEG	NEG	NEG	NEG	NEG	POS	POS	NEG	NEG	POS	NEG	NEG	NEG	POS	NEG	NEG	NEG	NEG	NEG	NEG	NEG	NEG	POS	NEG	NEG	NEG
00V38664	CC8/ST254-MRSA-(atypical SCCmec), Hannover EMRSA	POS	POS	POS	POS		NEG	NEG	NEG	NEG	NEG	NEG	NEG	NEG	NEG	NEG	POS	NEG	POS	POS	NEG	NEG	NEG	NEG	NEG	POS	POS	POS	NEG	POS	NEG	NEG	NEG	POS	AMB	NEG	NEG	NEG	NEG	NEG	NEG	NEG	POS	NEG	NEG	NEG
00V46488	CC8/ST254-MRSA-(atypical SCCmec), Hannover EMRSA	NEG	NEG	NEG	POS	NEG	NEG	NEG	NEG	NEG	NEG	NEG	NEG	NEG	NEG	NEG	POS	NEG	POS	POS	NEG	NEG	NEG	NEG	NEG	POS	POS	NEG	NEG	POS	NEG	NEG	NEG	POS	NEG	NEG	NEG	NEG	NEG	NEG	NEG	NEG	POS	NEG	NEG	NEG
00V44035	CC8/ST254-MRSA-IV/V, UK-EMRSA-10/Hannover EMRSA	POS	POS	POS	POS		NEG	NEG	NEG	NEG	NEG	NEG	NEG	NEG	NEG	NEG	POS	NEG	POS	POS	NEG	NEG	NEG	NEG	NEG	POS	NEG	NEG	NEG	NEG	NEG	NEG	NEG	POS	NEG	NEG	NEG	NEG	NEG	NEG	NEG	NEG	POS	NEG	NEG	NEG
00V13324	CC22-MRSA-IV [PVL+]	POS	POS	AMB	NEG	NEG	NEG	NEG	NEG	NEG	NEG	NEG	NEG	NEG	NEG	NEG	NEG	NEG	NEG	NEG	NEG	NEG	NEG	NEG	NEG	NEG	NEG	NEG	NEG	NEG	NEG	NEG	NEG	NEG	NEG	NEG	NEG	NEG	NEG	NEG	NEG	NEG	NEG	NEG	NEG	NEG
00B02046	CC45-MRSA-IV, Berlin EMRSA	POS	POS	POS	NEG	NEG	NEG	NEG	NEG	NEG	NEG	NEG	NEG	NEG	NEG	NEG	NEG	NEG	POS	POS	NEG	NEG	NEG	NEG	NEG	NEG	NEG	NEG	NEG	NEG	NEG	NEG	NEG	NEG	NEG	NEG	NEG	NEG	NEG	NEG	NEG	NEG	POS	NEG	NEG	NEG
00V11299	CC45-MRSA-IV, Berlin EMRSA	POS	POS	POS	NEG	NEG	NEG	NEG	NEG	NEG	NEG	NEG	NEG	NEG	NEG	NEG	NEG	NEG	POS	POS	NEG	NEG	NEG	NEG	NEG	NEG	NEG	NEG	NEG	NEG	NEG	NEG	NEG	NEG	NEG	NEG	NEG	NEG	NEG	NEG	NEG	NEG	POS	NEG	NEG	NEG
00V14125	CC45-MRSA-IV, Berlin EMRSA	POS	POS	POS	NEG	NEG	NEG	NEG	NEG	NEG	NEG	NEG	NEG	NEG	NEG	NEG	NEG	NEG	POS	POS	NEG	NEG	NEG	NEG	NEG	NEG	NEG	NEG	NEG	NEG	NEG	NEG	NEG	NEG	NEG	NEG	NEG	NEG	NEG	NEG	NEG	NEG	POS	NEG	NEG	NEG
00V16040	CC45-MRSA-IV, Berlin EMRSA	POS	POS	POS	NEG	NEG	POS	NEG	NEG	NEG	NEG	NEG	NEG	NEG	NEG	NEG	POS	POS	POS	POS	POS	NEG	NEG	NEG	NEG	NEG	NEG	NEG	NEG	NEG	NEG	NEG	NEG	NEG	NEG	NEG	POS	POS	NEG	NEG	NEG	NEG	POS	NEG	NEG	NEG
00V25025	CC45-MRSA-IV, Berlin EMRSA	POS	POS	POS	NEG	NEG	NEG	NEG	NEG	NEG	NEG	NEG	NEG	NEG	NEG	NEG	NEG	NEG	POS	POS	NEG	NEG	NEG	NEG	NEG	NEG	NEG	NEG	NEG	NEG	NEG	NEG	NEG	NEG	NEG	NEG	NEG	NEG	NEG	NEG	NEG	NEG	POS	NEG	NEG	NEG
00V29618	CC45-MRSA-IV, Berlin EMRSA	POS	POS	POS	NEG	NEG	NEG	NEG	NEG	NEG	NEG	NEG	NEG	NEG	NEG	NEG	NEG	NEG	POS	POS	NEG	NEG	NEG	NEG	NEG	NEG	NEG	NEG	NEG	NEG	NEG	NEG	NEG	NEG	NEG	NEG	NEG	NEG	NEG	NEG	NEG	NEG	POS	NEG	NEG	NEG
00V30989	CC45-MRSA-IV, Berlin EMRSA	POS	POS	POS	NEG	NEG	POS	NEG	NEG	NEG	NEG	NEG	NEG	NEG	NEG	NEG	POS	NEG	POS	POS	NEG	NEG	NEG	NEG	NEG	NEG	POS	POS	NEG	NEG	NEG	NEG	NEG	NEG	NEG	NEG	NEG	NEG	NEG	NEG	NEG	NEG	POS	NEG	NEG	NEG
00V31007	CC45-MRSA-IV, Berlin EMRSA	POS	POS	POS	NEG	NEG	NEG	NEG	NEG	NEG	NEG	NEG	NEG	NEG	NEG	NEG	NEG	NEG	POS	POS	POS	NEG	NEG	NEG	NEG	NEG	NEG	NEG	NEG	NEG	NEG	NEG	NEG	NEG	NEG	POS	NEG	NEG	NEG	NEG	NEG	NEG	POS	NEG	NEG	NEG
00V31700	CC45-MRSA-IV, Berlin EMRSA	POS	POS	POS	NEG	NEG	NEG	NEG	NEG	NEG	NEG	NEG	NEG	NEG	NEG	NEG	NEG	NEG	POS	POS	NEG	NEG	NEG	NEG	NEG	NEG	NEG	NEG	NEG	NEG	NEG	NEG	NEG	NEG	NEG	NEG	NEG	NEG	NEG	NEG	NEG	NEG	POS	NEG	NEG	NEG
00V32193	CC45-MRSA-IV, Berlin EMRSA	POS	POS	POS	NEG	NEG	NEG	NEG	NEG	NEG	NEG	NEG	NEG	NEG	NEG	NEG	NEG	NEG	POS	POS	NEG	NEG	NEG	NEG	NEG	NEG	NEG	NEG	NEG	NEG	NEG	NEG	NEG	NEG	NEG	NEG	NEG	NEG	NEG	NEG	NEG	NEG	POS	NEG	NEG	NEG
00V43085	CC45-MRSA-IV, Berlin EMRSA	POS	POS	POS	NEG	NEG	POS	NEG	NEG	NEG	NEG	NEG	NEG	NEG	NEG	NEG	NEG	NEG	POS	POS	NEG	NEG	NEG	NEG	NEG	NEG	NEG	NEG	NEG	NEG	NEG	NEG	NEG	NEG	NEG	NEG	NEG	NEG	NEG	NEG	NEG	NEG	POS	NEG	NEG	NEG
00V44382	CC45-MRSA-IV, Berlin EMRSA	POS	POS	POS	NEG	NEG	NEG	NEG	NEG	NEG	NEG	NEG	NEG	NEG	NEG	NEG	NEG	NEG	POS	POS	NEG	NEG	NEG	NEG	NEG	NEG	NEG	NEG	NEG	NEG	NEG	NEG	NEG	NEG	NEG	POS	NEG	NEG	NEG	NEG	NEG	NEG	NEG	NEG	NEG	NEG
00V46648	CC45-MRSA-IV, Berlin EMRSA	POS	POS	POS	NEG	NEG	NEG	NEG	NEG	NEG	NEG	NEG	NEG	NEG	NEG	NEG	NEG	NEG	POS	POS	NEG	NEG	NEG	NEG	NEG	NEG	NEG	NEG	NEG	NEG	NEG	NEG	NEG	NEG	NEG	POS	NEG	NEG	NEG	NEG	NEG	NEG	POS	NEG	NEG	NEG
00V46843	CC45-MRSA-IV, Berlin EMRSA	POS	POS	POS	NEG	NEG	NEG	NEG	NEG	NEG	NEG	NEG	NEG	NEG	NEG	NEG	NEG	NEG	POS	POS	NEG	NEG	NEG	NEG	NEG	NEG	NEG	NEG	NEG	NEG	NEG	NEG	NEG	NEG	NEG	NEG	NEG	NEG	NEG	NEG	NEG	NEG	POS	NEG	NEG	NEG
00V47014	CC45-MRSA-IV, Berlin EMRSA	POS	POS	POS	NEG	NEG	NEG	NEG	NEG	NEG	NEG	NEG	NEG	NEG	NEG	NEG	NEG	NEG	POS	POS	NEG	NEG	NEG	NEG	NEG	NEG	NEG	NEG	NEG	NEG	NEG	NEG	NEG	NEG	NEG	NEG	NEG	NEG	NEG	NEG	NEG	NEG	POS	NEG	NEG	NEG
00V47053	CC45-MRSA-IV, Berlin EMRSA	POS	POS	POS	NEG		NEG	NEG	NEG	NEG	NEG	NEG	NEG	NEG	NEG	NEG	NEG	NEG	POS	POS	NEG	NEG	NEG	NEG	NEG	NEG	NEG	NEG	NEG	NEG	NEG	NEG	NEG	NEG	NEG	NEG	NEG	NEG	NEG	NEG	NEG	NEG	NEG	NEG	NEG	NEG
00V47227	CC45-MRSA-IV, Berlin EMRSA	POS	POS	POS	NEG	NEG	NEG	NEG	NEG	NEG	NEG	NEG	NEG	NEG	NEG	NEG	NEG	NEG	POS	POS	NEG	NEG	NEG	NEG	NEG	NEG	NEG	NEG	NEG	NEG	NEG	NEG	NEG	NEG	NEG	NEG	NEG	NEG	NEG	NEG	NEG	NEG	POS	NEG	NEG	NEG
00V47429	CC45-MRSA-IV, Berlin EMRSA	POS	POS	POS	NEG	NEG	POS	NEG	NEG	NEG	NEG	NEG	NEG	NEG	NEG	NEG	POS	NEG	POS	POS	POS	NEG	NEG	NEG	NEG	NEG	NEG	NEG	NEG	NEG	NEG	NEG	NEG	NEG	NEG	NEG	POS	POS	NEG	NEG	NEG	NEG	POS	NEG	NEG	NEG
00V47531	CC45-MRSA-IV, Berlin EMRSA	POS	POS	POS	NEG	NEG	NEG	NEG	NEG	NEG	NEG	NEG	NEG	NEG	NEG	NEG	NEG	NEG	POS	POS	NEG	NEG	NEG	NEG	NEG	NEG	NEG	NEG	NEG	NEG	NEG	NEG	NEG	NEG	NEG	NEG	NEG	NEG	NEG	NEG	NEG	NEG	POS	NEG	NEG	NEG
**2001**																																														
01V27106	CC1-MRSA-IV, WA MRSA-1/57	POS	POS	POS	NEG		NEG	NEG	NEG	NEG	NEG	NEG	NEG	NEG	NEG	NEG	NEG	NEG	NEG	NEG	NEG	NEG	NEG	NEG	NEG	NEG	NEG	NEG	NEG	NEG	NEG	NEG	NEG	NEG	NEG	NEG	NEG	NEG	NEG	NEG	NEG	NEG	POS	NEG	NEG	NEG
01V41025	CC5/ST228-MRSA-I, South German EMRSA	POS	POS	POS	POS	NEG	NEG	NEG	NEG	NEG	NEG	NEG	NEG	AMB	NEG	NEG	POS	NEG	POS	POS	NEG	NEG	NEG	NEG	NEG	NEG	NEG	NEG	NEG	NEG	NEG	NEG	NEG	POS	AMB	POS	NEG	NEG	NEG	NEG	NEG	NEG	POS	NEG	NEG	NEG
01V12125	CC5-MRSA-II, Rhine-Hesse EMRSA/UK-EMRSA-3	POS	POS	POS	POS	NEG	NEG	NEG	NEG	NEG	NEG	NEG	NEG	NEG	NEG	NEG	NEG	POS	NEG	NEG	NEG	NEG	NEG	NEG	NEG	NEG	NEG	NEG	NEG	NEG	NEG	NEG	NEG	POS	NEG	NEG	NEG	NEG	NEG	NEG	NEG	NEG	POS	NEG	NEG	NEG
01V29747	CC5-MRSA-II, Rhine-Hesse EMRSA/UK-EMRSA-3	POS	POS	POS	POS	NEG	NEG	NEG	NEG	NEG	NEG	NEG	NEG	NEG	NEG	NEG	NEG	POS	NEG	NEG	NEG	NEG	NEG	NEG	NEG	NEG	NEG	NEG	NEG	NEG	NEG	NEG	NEG	POS	NEG	NEG	NEG	NEG	NEG	NEG	NEG	NEG	POS	NEG	NEG	NEG
01V32464	CC5-MRSA-II, Rhine-Hesse EMRSA/UK-EMRSA-3	POS	POS	POS	POS		NEG	NEG	NEG	NEG	NEG	NEG	NEG	NEG	NEG	NEG	NEG	POS	NEG	NEG	NEG	NEG	NEG	NEG	NEG	NEG	NEG	NEG	NEG	NEG	NEG	NEG	NEG	POS	NEG	NEG	NEG	NEG	NEG	NEG	NEG	NEG	POS	NEG	NEG	NEG
01V39703	CC5-MRSA-II, Rhine-Hesse EMRSA/UK-EMRSA-3	POS	POS	POS	POS	NEG	NEG	NEG	NEG	NEG	NEG	NEG	NEG	NEG	NEG	NEG	NEG	POS	NEG	NEG	NEG	NEG	NEG	NEG	NEG	NEG	NEG	NEG	NEG	NEG	NEG	NEG	NEG	POS	NEG	NEG	NEG	NEG	NEG	NEG	NEG	NEG	POS	NEG	NEG	NEG
01V40228	CC5-MRSA-II, Rhine-Hesse EMRSA/UK-EMRSA-3	POS			POS		NEG	NEG	NEG			NEG	NEG	NEG	NEG	NEG	NEG	POS	NEG	NEG	NEG	NEG		NEG	NEG	NEG																		NEG	NEG	NEG
01B05861	CC8/ST239-MRSA-III, Vienna/Hungarian/Brazilian Clone	POS	POS	POS	NEG	NEG	POS	NEG	NEG	NEG	NEG	NEG	NEG	NEG	NEG	NEG	AMB	NEG	POS	POS	NEG	NEG	NEG	NEG	NEG	POS	NEG	NEG	NEG	NEG	NEG	NEG	NEG	POS	NEG	NEG	NEG	NEG	NEG	NEG	NEG	NEG	POS	NEG	NEG	NEG
01V34572	CC8/ST239-MRSA-III, Vienna/Hungarian/Brazilian Clone	POS	POS	POS	NEG		POS	NEG	NEG	NEG	NEG	NEG	NEG	NEG	NEG	NEG	POS	NEG	POS	POS	NEG	NEG	NEG	NEG	NEG	POS	NEG	NEG	NEG	NEG	NEG	NEG	NEG	POS	NEG	NEG	NEG	NEG	NEG	NEG	NEG	NEG	POS	NEG	NEG	NEG
01V36469	CC8/ST239-MRSA-III, Vienna/Hungarian/Brazilian Clone	POS	POS	POS	NEG	NEG	POS	NEG	NEG	NEG	NEG	NEG	NEG	NEG	NEG	NEG	AMB	NEG	POS	POS	NEG	NEG	NEG	NEG	NEG	POS	NEG	NEG	NEG	NEG	NEG	NEG	NEG	POS	NEG	NEG	NEG	NEG	NEG	NEG	NEG	NEG	POS	NEG	NEG	NEG
01V39123	CC8/ST239-MRSA-III, Vienna/Hungarian/Brazilian Clone	POS	POS	POS	NEG	NEG	POS	NEG	NEG	NEG	NEG	NEG	NEG	NEG	NEG	NEG	POS	NEG	POS	POS	NEG	NEG	NEG	NEG	NEG	POS	NEG	NEG	NEG	NEG	NEG	NEG	NEG	POS	AMB	NEG	NEG	NEG	NEG	NEG	NEG	NEG	POS	NEG	NEG	NEG
01V39143	CC8/ST239-MRSA-III, Vienna/Hungarian/Brazilian Clone	POS	POS	POS	NEG	NEG	POS	NEG	NEG	NEG	NEG	NEG	NEG	NEG	NEG	NEG	NEG	NEG	POS	POS	NEG	NEG	NEG	NEG	NEG	POS	NEG	NEG	NEG	NEG	NEG	NEG	NEG	POS	NEG	NEG	NEG	NEG	NEG	NEG	NEG	NEG	POS	NEG	NEG	NEG
01V10962	CC8/ST254-MRSA-(atypical SCCmec), Hannover EMRSA	POS	POS	POS	POS		NEG	NEG	NEG	NEG	NEG	NEG	NEG	NEG	NEG	NEG	POS	NEG	POS	POS	NEG	NEG	NEG	NEG	NEG	POS	POS	POS	NEG	POS	NEG	NEG	NEG	POS	AMB	NEG	NEG	NEG	NEG	NEG	NEG	NEG	POS	NEG	NEG	NEG
01V10963	CC8/ST254-MRSA-IV/V, UK-EMRSA-10/Hannover EMRSA	POS	POS	POS	POS		NEG	NEG	NEG	NEG	NEG	NEG	NEG	NEG	NEG	NEG	POS	NEG	POS	POS	NEG	NEG	NEG	NEG	NEG	POS	NEG	NEG	NEG	NEG	NEG	NEG	NEG	POS	AMB	NEG	NEG	NEG	NEG	NEG	NEG	NEG	POS	NEG	NEG	NEG
01V36287	CC8/ST254-MRSA-IV/V, UK-EMRSA-10/Hannover EMRSA	POS	POS	POS	POS	NEG	NEG	NEG	NEG	NEG	NEG	NEG	NEG	NEG	NEG	NEG	POS	NEG	POS	POS	NEG	NEG	NEG	NEG	NEG	POS	NEG	NEG	NEG	NEG	NEG	NEG	NEG	POS	NEG	NEG	NEG	NEG	NEG	NEG	NEG	NEG	POS	NEG	NEG	NEG
01V25329	CC22-MRSA-IV [PVL+]	POS	POS	AMB	NEG	NEG	NEG	NEG	NEG	NEG	NEG	NEG	NEG	NEG	NEG	NEG	NEG	NEG	NEG	NEG	NEG	NEG	NEG	NEG	NEG	NEG	NEG	NEG	NEG	NEG	NEG	NEG	NEG	NEG	NEG	NEG	NEG	NEG	NEG	NEG	NEG	NEG	NEG	NEG	NEG	NEG
01V07770	CC22-MRSA-IV, Barnim/UK-EMRSA-15	POS			NEG		POS	NEG	NEG			NEG	NEG	NEG	NEG	NEG	NEG	NEG	NEG	NEG	NEG	NEG		NEG	NEG	NEG																		NEG	NEG	NEG
01V20122	CC22-MRSA-IV, Barnim/UK-EMRSA-15	POS	POS	POS	NEG	NEG	NEG	NEG	NEG	NEG	NEG	NEG	NEG	NEG	NEG	NEG	NEG	NEG	NEG	NEG	NEG	NEG	NEG	NEG	NEG	NEG	NEG	NEG	NEG	NEG	NEG	NEG	NEG	NEG	NEG	NEG	NEG	NEG	NEG	NEG	NEG	NEG	NEG	NEG	NEG	NEG
01V43277	CC22-MRSA-IV, Barnim/UK-EMRSA-15	POS	POS	POS	NEG	NEG	NEG	NEG	NEG	NEG	NEG	NEG	NEG	NEG	NEG	NEG	NEG	NEG	NEG	NEG	NEG	NEG	NEG	NEG	NEG	NEG	NEG	NEG	NEG	NEG	NEG	NEG	NEG	NEG	NEG	NEG	NEG	NEG	NEG	NEG	NEG	NEG	NEG	NEG	NEG	NEG
01F02744	CC45-MRSA-IV, Berlin EMRSA	POS	POS	POS	NEG	NEG	NEG	NEG	NEG	NEG	NEG	NEG	NEG	NEG	NEG	NEG	NEG	NEG	POS	POS	NEG	NEG	NEG	NEG	NEG	NEG	NEG	NEG	NEG	NEG	NEG	NEG	NEG	NEG	NEG	NEG	NEG	NEG	NEG	NEG	NEG	NEG	POS	NEG	NEG	NEG
01V01127	CC45-MRSA-IV, Berlin EMRSA	POS	POS	POS	NEG	NEG	POS	NEG	NEG	NEG	NEG	NEG	NEG	NEG	NEG	NEG	POS	NEG	POS	POS	POS	NEG	NEG	NEG	NEG	NEG	NEG	NEG	NEG	NEG	NEG	NEG	NEG	NEG	NEG	NEG	POS	POS	NEG	NEG	NEG	NEG	POS	NEG	NEG	NEG
01V01445	CC45-MRSA-IV, Berlin EMRSA	POS	POS	POS	NEG	NEG	NEG	NEG	NEG	NEG	NEG	NEG	NEG	NEG	NEG	NEG	NEG	NEG	POS	POS	NEG	NEG	NEG	NEG	NEG	NEG	NEG	NEG	NEG	NEG	NEG	NEG	NEG	NEG	NEG	NEG	NEG	NEG	NEG	NEG	NEG	NEG	POS	NEG	NEG	NEG
01V02575	CC45-MRSA-IV, Berlin EMRSA	POS	POS	POS	NEG	NEG	NEG	NEG	NEG	NEG	NEG	NEG	NEG	NEG	NEG	NEG	NEG	NEG	POS	POS	NEG	NEG	NEG	NEG	NEG	NEG	NEG	NEG	NEG	NEG	NEG	NEG	NEG	NEG	NEG	NEG	NEG	NEG	NEG	NEG	NEG	NEG	NEG	NEG	NEG	NEG
01V02636	CC45-MRSA-IV, Berlin EMRSA	POS	POS	POS	NEG	NEG	NEG	NEG	NEG	NEG	NEG	NEG	NEG	NEG	NEG	NEG	NEG	NEG	POS	POS	NEG	NEG	NEG	NEG	NEG	NEG	NEG	NEG	NEG	NEG	NEG	NEG	NEG	NEG	NEG	NEG	NEG	NEG	NEG	NEG	NEG	NEG	POS	NEG	NEG	NEG
01V03483	CC45-MRSA-IV, Berlin EMRSA	POS	POS	POS	NEG	NEG	POS	NEG	NEG	NEG	NEG	NEG	NEG	NEG	NEG	NEG	NEG	NEG	POS	POS	NEG	NEG	NEG	NEG	NEG	NEG	NEG	NEG	NEG	NEG	NEG	NEG	AMB	NEG	NEG	NEG	NEG	NEG	NEG	NEG	NEG	NEG	NEG	NEG	NEG	NEG
01V04728	CC45-MRSA-IV, Berlin EMRSA	POS	POS	POS	NEG	NEG	NEG	NEG	NEG	NEG	NEG	NEG	NEG	NEG	NEG	NEG	NEG	NEG	POS	POS	NEG	NEG	NEG	NEG	NEG	NEG	NEG	NEG	NEG	NEG	NEG	NEG	NEG	NEG	NEG	NEG	NEG	NEG	NEG	NEG	NEG	NEG	POS	NEG	NEG	NEG
01V05972	CC45-MRSA-IV, Berlin EMRSA	POS	POS	POS	NEG	NEG	NEG	NEG	NEG	NEG	NEG	NEG	NEG	NEG	NEG	NEG	NEG	NEG	AMB	POS	NEG	NEG	NEG	NEG	NEG	NEG	NEG	NEG	NEG	NEG	NEG	NEG	NEG	NEG	NEG	NEG	NEG	NEG	NEG	NEG	NEG	NEG	AMB	NEG	NEG	NEG
01V07139	CC45-MRSA-IV, Berlin EMRSA	POS	POS	POS	NEG	NEG	NEG	NEG	NEG	NEG	NEG	NEG	NEG	NEG	NEG	NEG	NEG	NEG	POS	POS	NEG	NEG	NEG	NEG	NEG	NEG	NEG	NEG	NEG	NEG	NEG	NEG	NEG	NEG	NEG	NEG	NEG	NEG	NEG	NEG	NEG	NEG	POS	NEG	NEG	NEG
01V08133	CC45-MRSA-IV, Berlin EMRSA	POS	POS	POS	NEG	NEG	NEG	NEG	NEG	NEG	NEG	NEG	NEG	NEG	NEG	NEG	NEG	NEG	POS	POS	NEG	NEG	NEG	NEG	NEG	NEG	NEG	NEG	NEG	NEG	NEG	NEG	NEG	NEG	NEG	NEG	NEG	NEG	NEG	NEG	NEG	NEG	POS	NEG	NEG	NEG
01V09872	CC45-MRSA-IV, Berlin EMRSA	POS	POS	POS	NEG	NEG	NEG	NEG	NEG	NEG	NEG	NEG	NEG	NEG	NEG	NEG	NEG	NEG	POS	POS	NEG	NEG	NEG	NEG	NEG	NEG	NEG	NEG	NEG	NEG	NEG	NEG	NEG	NEG	NEG	NEG	NEG	NEG	NEG	NEG	NEG	NEG	POS	NEG	NEG	NEG
01V12465	CC45-MRSA-IV, Berlin EMRSA	POS	POS	POS	NEG	NEG	NEG	NEG	NEG	NEG	NEG	NEG	NEG	NEG	NEG	NEG	NEG	NEG	POS	POS	NEG	NEG	NEG	NEG	NEG	NEG	NEG	NEG	NEG	NEG	NEG	NEG	NEG	NEG	NEG	NEG	NEG	NEG	NEG	NEG	NEG	NEG	POS	NEG	NEG	NEG
01V16151	CC45-MRSA-IV, Berlin EMRSA	POS	POS	POS	NEG	NEG	POS	NEG	NEG	NEG	NEG	NEG	NEG	NEG	NEG	NEG	NEG	NEG	POS	POS	NEG	NEG	NEG	NEG	NEG	NEG	NEG	NEG	NEG	NEG	NEG	NEG	NEG	NEG	NEG	NEG	NEG	NEG	NEG	NEG	NEG	NEG	AMB	NEG	NEG	NEG
01V16873	CC45-MRSA-IV, Berlin EMRSA	POS	POS	POS	NEG	NEG	NEG	NEG	NEG	NEG	NEG	NEG	NEG	NEG	NEG	NEG	NEG	NEG	POS	POS	NEG	NEG	NEG	NEG	NEG	NEG	NEG	NEG	NEG	NEG	NEG	NEG	NEG	NEG	NEG	NEG	NEG	NEG	NEG	NEG	NEG	NEG	POS	NEG	NEG	NEG
01V17796	CC45-MRSA-IV, Berlin EMRSA	POS	POS	POS	NEG	NEG	NEG	NEG	NEG	NEG	NEG	NEG	NEG	NEG	NEG	NEG	NEG	NEG	POS	POS	NEG	NEG	NEG	NEG	NEG	NEG	NEG	NEG	NEG	NEG	NEG	NEG	NEG	NEG	NEG	NEG	NEG	NEG	NEG	NEG	NEG	NEG	POS	NEG	NEG	NEG
01V18681	CC45-MRSA-IV, Berlin EMRSA	POS	POS	POS	NEG	NEG	NEG	NEG	NEG	NEG	NEG	NEG	NEG	NEG	NEG	NEG	NEG	NEG	POS	POS	NEG	NEG	NEG	NEG	NEG	NEG	NEG	NEG	NEG	NEG	NEG	NEG	NEG	NEG	NEG	NEG	NEG	NEG	NEG	NEG	NEG	NEG	POS	NEG	NEG	NEG
01V20066	CC45-MRSA-IV, Berlin EMRSA	POS	POS	POS	NEG	NEG	NEG	NEG	NEG	NEG	NEG	NEG	NEG	NEG	NEG	NEG	NEG	NEG	POS	POS	NEG	NEG	NEG	NEG	NEG	NEG	NEG	NEG	NEG	NEG	NEG	NEG	NEG	NEG	NEG	NEG	NEG	NEG	NEG	NEG	NEG	NEG	POS	NEG	NEG	NEG
01V23050	CC45-MRSA-IV, Berlin EMRSA	POS	POS	POS	NEG	NEG	NEG	NEG	NEG	NEG	NEG	NEG	NEG	NEG	NEG	NEG	NEG	NEG	POS	POS	NEG	NEG	NEG	NEG	NEG	NEG	NEG	NEG	NEG	NEG	NEG	NEG	NEG	NEG	NEG	NEG	NEG	NEG	NEG	NEG	NEG	NEG	POS	NEG	NEG	NEG
01V25584	CC45-MRSA-IV, Berlin EMRSA	POS	POS	POS	NEG	NEG	NEG	NEG	NEG	NEG	NEG	NEG	NEG	NEG	NEG	NEG	NEG	NEG	NEG	POS	NEG	NEG	NEG	NEG	NEG	NEG	NEG	NEG	NEG	NEG	NEG	NEG	NEG	NEG	NEG	NEG	NEG	NEG	NEG	NEG	NEG	NEG	AMB	NEG	NEG	NEG
01V31705	CC45-MRSA-IV, Berlin EMRSA	POS	POS	POS	NEG	NEG	NEG	NEG	NEG	NEG	NEG	NEG	NEG	NEG	NEG	NEG	NEG	NEG	POS	POS	NEG	NEG	NEG	NEG	NEG	NEG	NEG	NEG	NEG	NEG	NEG	NEG	NEG	NEG	NEG	NEG	NEG	NEG	NEG	NEG	NEG	NEG	POS	NEG	NEG	NEG
01V34968	CC45-MRSA-IV, Berlin EMRSA	POS	POS	POS	NEG	NEG	NEG	NEG	NEG	NEG	NEG	NEG	NEG	NEG	NEG	NEG	NEG	NEG	POS	POS	NEG	NEG	NEG	NEG	NEG	NEG	NEG	NEG	NEG	NEG	NEG	NEG	NEG	NEG	NEG	NEG	NEG	NEG	NEG	NEG	NEG	NEG	POS	NEG	NEG	NEG
01V36186	CC45-MRSA-IV, Berlin EMRSA	POS	POS	POS	NEG	NEG	NEG	NEG	NEG	NEG	NEG	NEG	NEG	NEG	NEG	NEG	NEG	NEG	POS	POS	NEG	NEG	NEG	NEG	NEG	NEG	NEG	NEG	NEG	NEG	NEG	NEG	NEG	NEG	NEG	NEG	NEG	NEG	NEG	NEG	NEG	NEG	POS	NEG	NEG	NEG
01V36534	CC45-MRSA-IV, Berlin EMRSA	POS	POS	POS	NEG	NEG	NEG	AMB	NEG	NEG	NEG	NEG	NEG	NEG	NEG	NEG	POS	POS	POS	POS	NEG	NEG	NEG	POS	NEG	NEG	NEG	NEG	NEG	NEG	NEG	NEG	NEG	NEG	NEG	NEG	NEG	NEG	NEG	NEG	NEG	NEG	POS	NEG	NEG	NEG
01V36588	CC45-MRSA-IV, Berlin EMRSA	POS	POS	POS	NEG	NEG	NEG	NEG	NEG	NEG	NEG	NEG	NEG	NEG	NEG	NEG	NEG	NEG	POS	POS	NEG	NEG	NEG	NEG	NEG	NEG	NEG	NEG	NEG	NEG	NEG	NEG	NEG	NEG	NEG	NEG	NEG	NEG	NEG	NEG	NEG	NEG	POS	NEG	NEG	NEG
01V37990	CC45-MRSA-IV, Berlin EMRSA	POS	POS	POS	NEG	NEG	NEG	NEG	NEG	NEG	NEG	NEG	NEG	NEG	NEG	NEG	NEG	NEG	POS	POS	NEG	NEG	NEG	NEG	NEG	NEG	NEG	NEG	NEG	NEG	NEG	NEG	NEG	NEG	NEG	NEG	NEG	NEG	NEG	NEG	NEG	NEG	POS	NEG	NEG	NEG
01V39610	CC45-MRSA-IV, Berlin EMRSA	POS	POS	POS	NEG	NEG	NEG	NEG	NEG	NEG	NEG	NEG	NEG	POS	NEG	NEG	NEG	NEG	POS	POS	NEG	NEG	NEG	NEG	NEG	NEG	NEG	NEG	NEG	NEG	NEG	NEG	NEG	AMB	NEG	NEG	NEG	NEG	NEG	NEG	NEG	NEG	POS	NEG	NEG	NEG
01V40229	CC45-MRSA-IV, Berlin EMRSA	POS	POS	POS	NEG	NEG	NEG	NEG	NEG	NEG	NEG	NEG	NEG	NEG	NEG	NEG	NEG	NEG	POS	POS	NEG	NEG	NEG	NEG	NEG	NEG	NEG	NEG	NEG	NEG	NEG	NEG	NEG	NEG	NEG	NEG	NEG	NEG	NEG	NEG	NEG	NEG	POS	NEG	NEG	NEG
01V40374	CC45-MRSA-IV, Berlin EMRSA	POS			NEG		POS	NEG	NEG			NEG	NEG	NEG	NEG	NEG	NEG	NEG	POS	POS	NEG	NEG		NEG	NEG	NEG																		NEG	NEG	NEG
01V41056	CC45-MRSA-IV, Berlin EMRSA	POS	POS	POS	NEG	NEG	NEG	NEG	NEG	NEG	NEG	NEG	NEG	NEG	NEG	NEG	NEG	NEG	AMB	POS	NEG	NEG	NEG	NEG	NEG	NEG	NEG	NEG	NEG	NEG	NEG	NEG	NEG	NEG	NEG	NEG	NEG	NEG	NEG	NEG	NEG	NEG	AMB	NEG	NEG	NEG
01V42009	CC45-MRSA-IV, Berlin EMRSA	POS	POS	POS	NEG	NEG	NEG	NEG	NEG	NEG	NEG	NEG	NEG	NEG	NEG	NEG	NEG	NEG	POS	POS	NEG	NEG	NEG	NEG	NEG	NEG	NEG	NEG	NEG	NEG	NEG	NEG	NEG	NEG	NEG	NEG	NEG	NEG	NEG	NEG	NEG	NEG	POS	NEG	NEG	NEG
01V43511	CC45-MRSA-IV, Berlin EMRSA	POS	POS	POS	NEG	NEG	NEG	NEG	NEG	NEG	NEG	NEG	NEG	NEG	NEG	NEG	NEG	NEG	POS	POS	NEG	NEG	NEG	NEG	NEG	NEG	NEG	NEG	NEG	NEG	NEG	NEG	NEG	NEG	NEG	NEG	NEG	NEG	NEG	NEG	NEG	NEG	POS	NEG	NEG	NEG
**2002**																																														
02V10698	CC5/ST228-MRSA-I, South German EMRSA	POS	POS	POS	POS	NEG	NEG	NEG	NEG	NEG	NEG	NEG	NEG	NEG	NEG	NEG	POS	NEG	POS	POS	NEG	NEG	NEG	NEG	NEG	NEG	NEG	NEG	NEG	NEG	NEG	NEG	NEG	POS	NEG	POS	NEG	NEG	NEG	NEG	NEG	NEG	POS	NEG	NEG	NEG
02V13140	CC5/ST228-MRSA-I, South German EMRSA	POS	POS	POS	POS	NEG	NEG	NEG	NEG	NEG	NEG	NEG	NEG	NEG	NEG	NEG	POS	NEG	NEG	NEG	NEG	NEG	NEG	NEG	NEG	NEG	NEG	NEG	NEG	NEG	NEG	NEG	NEG	POS	NEG	POS	NEG	NEG	NEG	NEG	NEG	NEG	POS	NEG	NEG	NEG
02V20759	CC5/ST228-MRSA-I, South German EMRSA	POS	POS	POS	POS	NEG	NEG	NEG	NEG	NEG	NEG	NEG	NEG	NEG	NEG	NEG	POS	NEG	POS	POS	NEG	NEG	NEG	NEG	NEG	NEG	NEG	NEG	NEG	NEG	NEG	NEG	NEG	POS	NEG	POS	NEG	NEG	NEG	NEG	NEG	NEG	POS	NEG	NEG	NEG
02B06271	CC5-MRSA-II, Rhine-Hesse EMRSA/UK-EMRSA-3	POS	POS	POS	POS	NEG	NEG	NEG	NEG	NEG	NEG	NEG	NEG	NEG	NEG	NEG	NEG	POS	NEG	NEG	NEG	NEG	NEG	NEG	NEG	NEG	NEG	NEG	NEG	NEG	NEG	NEG	NEG	POS	NEG	NEG	NEG	NEG	NEG	NEG	NEG	NEG	POS	NEG	NEG	NEG
02V04116	CC5-MRSA-II, Rhine-Hesse EMRSA/UK-EMRSA-3	POS	POS	POS	POS	NEG	NEG	NEG	NEG	NEG	NEG	NEG	NEG	NEG	NEG	NEG	NEG	POS	NEG	NEG	NEG	NEG	NEG	NEG	NEG	NEG	NEG	NEG	NEG	NEG	NEG	NEG	NEG	POS	NEG	NEG	NEG	NEG	NEG	NEG	NEG	NEG	POS	NEG	NEG	NEG
02V29922	CC5-MRSA-II, Rhine-Hesse EMRSA/UK-EMRSA-3	NEG			POS		NEG	NEG	NEG			NEG	NEG	NEG	NEG	NEG	NEG	POS	NEG	NEG	NEG	NEG		NEG	NEG	NEG																		NEG	NEG	NEG
02V33634	CC5-MRSA-II, Rhine-Hesse EMRSA/UK-EMRSA-3	POS	POS	POS	POS	NEG	NEG	NEG	NEG	NEG	NEG	NEG	NEG	NEG	NEG	NEG	NEG	POS	NEG	NEG	NEG	NEG	NEG	NEG	NEG	NEG	NEG	NEG	NEG	NEG	NEG	NEG	NEG	POS	NEG	NEG	NEG	NEG	NEG	NEG	NEG	NEG	POS	NEG	NEG	NEG
02V34748	CC5-MRSA-II, Rhine-Hesse EMRSA/UK-EMRSA-3	POS	POS	POS	POS	NEG	NEG	NEG	NEG	NEG	NEG	NEG	NEG	NEG	NEG	NEG	NEG	POS	NEG	NEG	NEG	NEG	NEG	NEG	NEG	NEG	NEG	NEG	NEG	NEG	NEG	NEG	NEG	POS	NEG	NEG	NEG	NEG	NEG	NEG	NEG	NEG	POS	NEG	NEG	NEG
02V17002	CC8/ST254-MRSA-(atypical SCCmec), Hannover EMRSA	POS	POS	POS	NEG	NEG	NEG	NEG	NEG	NEG	NEG	NEG	NEG	NEG	NEG	NEG	POS	NEG	POS	POS	NEG	NEG	NEG	NEG	NEG	[illegible]	[illegible]	[illegible]	[illegible]	[illegible]	[illegible]	[illegible]	[illegible]	[illegible]	[illegible]	[illegible]	[illegible]	[illegible]	[illegible]	[illegible]	[illegible]	[illegible]	[illegible]	[illegible]	[illegible]	[illegible]
02V33579	CC8-MRSA-IV, USA500	POS	POS	POS	NEG		POS	NEG	NEG	NEG	NEG	NEG	NEG	NEG	NEG	NEG	POS	NEG	NEG	NEG	POS	NEG	NEG	NEG	NEG	[illegible]	[illegible]	[illegible]	[illegible]	[illegible]	[illegible]	[illegible]	[illegible]	[illegible]	[illegible]	[illegible]	[illegible]	[illegible]	[illegible]	[illegible]	[illegible]	[illegible]	[illegible]	[illegible]	[illegible]	[illegible]
02V15498	CC22-MRSA-IV [PVL+]	POS	POS	POS	NEG	NEG	NEG	NEG	NEG	NEG	NEG	NEG	NEG	NEG	NEG	NEG	NEG	NEG	NEG	NEG	NEG	NEG	NEG	NEG	NEG	[illegible]	[illegible]	[illegible]	[illegible]	[illegible]	[illegible]	[illegible]	[illegible]	[illegible]	[illegible]	[illegible]	[illegible]	[illegible]	[illegible]	[illegible]	[illegible]	[illegible]	[illegible]	[illegible]	[illegible]	[illegible]
02C05319	CC22-MRSA-IV, Barnim/UK-EMRSA-15	POS	POS	POS	NEG	NEG	POS	NEG	NEG	NEG	NEG	NEG	NEG	NEG	NEG	NEG	NEG	NEG	NEG	NEG	NEG	NEG	NEG	NEG	NEG	[illegible]	[illegible]	[illegible]	[illegible]	[illegible]	[illegible]	[illegible]	[illegible]	[illegible]	[illegible]	[illegible]	[illegible]	[illegible]	[illegible]	[illegible]	[illegible]	[illegible]	[illegible]	[illegible]	[illegible]	[illegible]
02U10255	CC22-MRSA-IV, Barnim/UK-EMRSA-15	POS	POS	POS	NEG	NEG	POS	NEG	NEG	NEG	NEG	NEG	NEG	NEG	NEG	NEG	NEG	NEG	NEG	NEG	NEG	NEG	NEG	NEG	NEG	[illegible]	[illegible]	[illegible]	[illegible]	[illegible]	[illegible]	[illegible]	[illegible]	[illegible]	[illegible]	[illegible]	[illegible]	[illegible]	[illegible]	[illegible]	[illegible]	[illegible]	[illegible]	[illegible]	[illegible]	[illegible]
02V12175	CC22-MRSA-IV, Barnim/UK-EMRSA-15	POS	POS	POS	NEG	NEG	POS	NEG	NEG	NEG	NEG	NEG	NEG	NEG	NEG	NEG	NEG	NEG	NEG	NEG	NEG	NEG	NEG	NEG	NEG	[illegible]	[illegible]	[illegible]	[illegible]	[illegible]	[illegible]	[illegible]	[illegible]	[illegible]	[illegible]	[illegible]	[illegible]	[illegible]	[illegible]	[illegible]	[illegible]	[illegible]	[illegible]	[illegible]	[illegible]	[illegible]
02V13054	CC22-MRSA-IV, Barnim/UK-EMRSA-15	POS	POS	POS	NEG	NEG	POS	NEG	NEG	NEG	NEG	NEG	NEG	NEG	NEG	NEG	NEG	NEG	NEG	NEG	NEG	NEG	NEG	NEG	NEG	[illegible]	[illegible]	[illegible]	[illegible]	[illegible]	[illegible]	[illegible]	[illegible]	[illegible]	[illegible]	[illegible]	[illegible]	[illegible]	[illegible]	[illegible]	[illegible]	[illegible]	[illegible]	[illegible]	[illegible]	[illegible]
02V13950	CC22-MRSA-IV, Barnim/UK-EMRSA-15	POS	POS	POS	NEG	NEG	POS	NEG	NEG	NEG	NEG	NEG	NEG	NEG	NEG	NEG	NEG	NEG	NEG	NEG	NEG	NEG	NEG	NEG	NEG	[illegible]	[illegible]	[illegible]	[illegible]	[illegible]	[illegible]	[illegible]	[illegible]	[illegible]	[illegible]	[illegible]	[illegible]	[illegible]	[illegible]	[illegible]	[illegible]	[illegible]	[illegible]	[illegible]	[illegible]	[illegible]
02V21259	CC22-MRSA-IV, Barnim/UK-EMRSA-15	POS	POS	POS	NEG	NEG	POS	NEG	NEG	NEG	NEG	NEG	NEG	NEG	NEG	NEG	NEG	NEG	NEG	NEG	NEG	NEG	NEG	NEG	NEG	[illegible]	[illegible]	[illegible]	[illegible]	[illegible]	[illegible]	[illegible]	[illegible]	[illegible]	[illegible]	[illegible]	[illegible]	[illegible]	[illegible]	[illegible]	[illegible]	[illegible]	[illegible]	[illegible]	[illegible]	[illegible]
02V22657	CC22-MRSA-IV, Barnim/UK-EMRSA-15	POS	POS	POS	NEG	NEG	POS	NEG	NEG	NEG	NEG	NEG	NEG	NEG	NEG	NEG	NEG	NEG	NEG	NEG	NEG	NEG	NEG	NEG	NEG	[illegible]	[illegible]	[illegible]	[illegible]	[illegible]	[illegible]	[illegible]	[illegible]	[illegible]	[illegible]	[illegible]	[illegible]	[illegible]	[illegible]	[illegible]	[illegible]	[illegible]	[illegible]	[illegible]	[illegible]	[illegible]
02V29051	CC22-MRSA-IV, Barnim/UK-EMRSA-15	POS	POS	POS	NEG	NEG	POS	NEG	NEG	NEG	NEG	NEG	NEG	NEG	NEG	NEG	NEG	NEG	NEG	NEG	NEG	NEG	NEG	NEG	NEG	[illegible]	[illegible]	[illegible]	[illegible]	[illegible]	[illegible]	[illegible]	[illegible]	[illegible]	[illegible]	[illegible]	[illegible]	[illegible]	[illegible]	[illegible]	[illegible]	[illegible]	[illegible]	[illegible]	[illegible]	[illegible]
02V30792	CC30/ST36-MRSA-II, UK-EMRSA-16	POS	POS	POS	POS	NEG	NEG	NEG	NEG	NEG	NEG	NEG	NEG	NEG	NEG	NEG	POS	POS	NEG	NEG	POS	NEG	NEG	NEG	NEG	[illegible]	[illegible]	[illegible]	[illegible]	[illegible]	[illegible]	[illegible]	[illegible]	[illegible]	[illegible]	[illegible]	[illegible]	[illegible]	[illegible]	[illegible]	[illegible]	[illegible]	[illegible]	[illegible]	[illegible]	[illegible]
02U07096	CC45-MRSA-IV, Berlin EMRSA	POS	POS	POS	NEG	NEG	NEG	NEG	NEG	NEG	NEG	NEG	NEG	NEG	NEG	NEG	NEG	NEG	POS	POS	NEG	NEG	NEG	NEG	NEG	[illegible]	[illegible]	[illegible]	[illegible]	[illegible]	[illegible]	[illegible]	[illegible]	[illegible]	[illegible]	[illegible]	[illegible]	[illegible]	[illegible]	[illegible]	[illegible]	[illegible]	[illegible]	[illegible]	[illegible]	[illegible]
02V00570	CC45-MRSA-IV, Berlin EMRSA	POS	POS	POS	NEG	NEG	POS	NEG	NEG	NEG	NEG	NEG	NEG	NEG	NEG	NEG	NEG	NEG	POS	POS	NEG	NEG	NEG	NEG	NEG	[illegible]	[illegible]	[illegible]	[illegible]	[illegible]	[illegible]	[illegible]	[illegible]	[illegible]	[illegible]	[illegible]	[illegible]	[illegible]	[illegible]	[illegible]	[illegible]	[illegible]	[illegible]	[illegible]	[illegible]	[illegible]
02V01730	CC45-MRSA-IV, Berlin EMRSA	POS	POS	POS	NEG	NEG	NEG	NEG	NEG	NEG	NEG	NEG	NEG	NEG	NEG	NEG	NEG	NEG	POS	POS	NEG	NEG	NEG	NEG	NEG	[illegible]	[illegible]	[illegible]	[illegible]	[illegible]	[illegible]	[illegible]	[illegible]	[illegible]	[illegible]	[illegible]	[illegible]	[illegible]	[illegible]	[illegible]	[illegible]	[illegible]	[illegible]	[illegible]	[illegible]	[illegible]
02V01870	CC45-MRSA-IV, Berlin EMRSA	POS	POS	POS	NEG	NEG	NEG	NEG	NEG	NEG	NEG	NEG	NEG	NEG	NEG	NEG	NEG	NEG	POS	POS	NEG	NEG	NEG	NEG	NEG	[illegible]	[illegible]	[illegible]	[illegible]	[illegible]	[illegible]	[illegible]	[illegible]	[illegible]	[illegible]	[illegible]	[illegible]	[illegible]	[illegible]	[illegible]	[illegible]	[illegible]	[illegible]	[illegible]	[illegible]	[illegible]
02V04750	CC45-MRSA-IV, Berlin EMRSA	POS	POS	POS	NEG	NEG	NEG	NEG	NEG	NEG	NEG	NEG	NEG	NEG	NEG	NEG	NEG	NEG	POS	POS	NEG	NEG	NEG	NEG	NEG	[illegible]	[illegible]	[illegible]	[illegible]	[illegible]	[illegible]	[illegible]	[illegible]	[illegible]	[illegible]	[illegible]	[illegible]	[illegible]	[illegible]	[illegible]	[illegible]	[illegible]	[illegible]	[illegible]	[illegible]	[illegible]
02V06592	CC45-MRSA-IV, Berlin EMRSA	POS	POS	POS	NEG	NEG	NEG	NEG	NEG	NEG	NEG	NEG	NEG	NEG	NEG	NEG	NEG	NEG	POS	POS	NEG	NEG	NEG	NEG	NEG	[illegible]	[illegible]	[illegible]	[illegible]	[illegible]	[illegible]	[illegible]	[illegible]	[illegible]	[illegible]	[illegible]	[illegible]	[illegible]	[illegible]	[illegible]	[illegible]	[illegible]	[illegible]	[illegible]	[illegible]	[illegible]
02V10868	CC45-MRSA-IV, Berlin EMRSA	POS	POS	POS	NEG	NEG	NEG	NEG	NEG	NEG	NEG	NEG	NEG	NEG	NEG	NEG	NEG	NEG	POS	POS	NEG	NEG	NEG	NEG	NEG	[illegible]	[illegible]	[illegible]	[illegible]	[illegible]	[illegible]	[illegible]	[illegible]	[illegible]	[illegible]	[illegible]	[illegible]	[illegible]	[illegible]	[illegible]	[illegible]	[illegible]	[illegible]	[illegible]	[illegible]	[illegible]
02V12394	CC45-MRSA-IV, Berlin EMRSA	POS	POS	POS	NEG	NEG	POS	NEG	NEG	NEG	NEG	NEG	NEG	NEG	NEG	NEG	POS	NEG	POS	POS	NEG	NEG	NEG	NEG	NEG	[illegible]	[illegible]	[illegible]	[illegible]	[illegible]	[illegible]	[illegible]	[illegible]	[illegible]	[illegible]	[illegible]	[illegible]	[illegible]	[illegible]	[illegible]	[illegible]	[illegible]	[illegible]	[illegible]	[illegible]	[illegible]
02V12942	CC45-MRSA-IV, Berlin EMRSA	POS	POS	POS	NEG	NEG	NEG	NEG	NEG	NEG	NEG	NEG	NEG	NEG	NEG	NEG	NEG	NEG	POS	POS	NEG	NEG	NEG	NEG	NEG	[illegible]	[illegible]	[illegible]	[illegible]	[illegible]	[illegible]	[illegible]	[illegible]	[illegible]	[illegible]	[illegible]	[illegible]	[illegible]	[illegible]	[illegible]	[illegible]	[illegible]	[illegible]	[illegible]	[illegible]	[illegible]
02V13026	CC45-MRSA-IV, Berlin EMRSA	POS	POS	POS	NEG	NEG	POS	NEG	NEG	NEG	NEG	NEG	NEG	NEG	NEG	NEG	NEG	NEG	POS	POS	NEG	NEG	NEG	NEG	NEG	[illegible]	[illegible]	[illegible]	[illegible]	[illegible]	[illegible]	[illegible]	[illegible]	[illegible]	[illegible]	[illegible]	[illegible]	[illegible]	[illegible]	[illegible]	[illegible]	[illegible]	[illegible]	[illegible]	[illegible]	[illegible]
02V14700	CC45-MRSA-IV, Berlin EMRSA	POS			NEG		POS	NEG	NEG			NEG	NEG	NEG	NEG	NEG	POS	NEG	POS	POS	POS	NEG		NEG	NEG	[illegible]	[illegible]	[illegible]	[illegible]	[illegible]	[illegible]	[illegible]	[illegible]	[illegible]	[illegible]	[illegible]	[illegible]	[illegible]	[illegible]	[illegible]	[illegible]	[illegible]	[illegible]	[illegible]	[illegible]	[illegible]
02V15358	CC45-MRSA-IV, Berlin EMRSA	POS	POS	POS	NEG	NEG	POS	NEG	NEG	NEG	NEG	NEG	NEG	NEG	NEG	NEG	NEG	NEG	POS	POS	NEG	NEG	NEG	NEG	NEG	[illegible]	[illegible]	[illegible]	[illegible]	[illegible]	[illegible]	[illegible]	[illegible]	[illegible]	[illegible]	[illegible]	[illegible]	[illegible]	[illegible]	[illegible]	[illegible]	[illegible]	[illegible]	[illegible]	[illegible]	[illegible]
02V16654	CC45-MRSA-IV, Berlin EMRSA	POS	POS	POS	NEG	NEG	NEG	NEG	NEG	NEG	NEG	NEG	NEG	NEG	NEG	NEG	NEG	NEG	POS	POS	NEG	NEG	NEG	NEG	NEG	[illegible]	[illegible]	[illegible]	[illegible]	[illegible]	[illegible]	[illegible]	[illegible]	[illegible]	[illegible]	[illegible]	[illegible]	[illegible]	[illegible]	[illegible]	[illegible]	[illegible]	[illegible]	[illegible]	[illegible]	[illegible]
02V17120	CC45-MRSA-IV, Berlin EMRSA	POS	POS	POS	NEG	NEG	NEG	NEG	NEG	NEG	NEG	NEG	NEG	AMB	NEG	NEG	NEG	NEG	POS	POS	NEG	NEG	NEG	NEG	NEG	[illegible]	[illegible]	[illegible]	[illegible]	[illegible]	[illegible]	[illegible]	[illegible]	[illegible]	[illegible]	[illegible]	[illegible]	[illegible]	[illegible]	[illegible]	[illegible]	[illegible]	[illegible]	[illegible]	[illegible]	[illegible]
02V17136	CC45-MRSA-IV, Berlin EMRSA	POS	POS	POS	NEG	NEG	NEG	NEG	NEG	NEG	NEG	NEG	NEG	NEG	NEG	NEG	NEG	NEG	POS	POS	NEG	NEG	NEG	NEG	NEG	[illegible]	[illegible]	[illegible]	[illegible]	[illegible]	[illegible]	[illegible]	[illegible]	[illegible]	[illegible]	[illegible]	[illegible]	[illegible]	[illegible]	[illegible]	[illegible]	[illegible]	[illegible]	[illegible]	[illegible]	[illegible]
02V17558	CC45-MRSA-IV, Berlin EMRSA	POS	POS	POS	NEG	NEG	NEG	NEG	NEG	NEG	NEG	NEG	NEG	NEG	NEG	NEG	POS	POS	POS	POS	NEG	NEG	NEG	NEG	NEG	[illegible]	[illegible]	[illegible]	[illegible]	[illegible]	[illegible]	[illegible]	[illegible]	[illegible]	[illegible]	[illegible]	[illegible]	[illegible]	[illegible]	[illegible]	[illegible]	[illegible]	[illegible]	[illegible]	[illegible]	[illegible]
02V17665	CC45-MRSA-IV, Berlin EMRSA	POS	POS	POS	NEG	NEG	POS	NEG	NEG	NEG	NEG	NEG	NEG	NEG	NEG	NEG	NEG	NEG	POS	POS	NEG	NEG	NEG	NEG	NEG	[illegible]	[illegible]	[illegible]	[illegible]	[illegible]	[illegible]	[illegible]	[illegible]	[illegible]	[illegible]	[illegible]	[illegible]	[illegible]	[illegible]	[illegible]	[illegible]	[illegible]	[illegible]	[illegible]	[illegible]	[illegible]
02V20390	CC45-MRSA-IV, Berlin EMRSA	POS	POS	POS	NEG	NEG	NEG	NEG	NEG	NEG	NEG	NEG	NEG	NEG	NEG	NEG	NEG	NEG	NEG	NEG	NEG	NEG	NEG	NEG	AMB	[illegible]	[illegible]	[illegible]	[illegible]	[illegible]	[illegible]	[illegible]	[illegible]	[illegible]	[illegible]	[illegible]	[illegible]	[illegible]	[illegible]	[illegible]	[illegible]	[illegible]	[illegible]	[illegible]	[illegible]	[illegible]
02V20626	CC45-MRSA-IV, Berlin EMRSA	POS	POS	POS	NEG	NEG	POS	NEG	NEG	NEG	NEG	NEG	NEG	NEG	NEG	NEG	POS	NEG	POS	POS	POS	NEG	NEG	NEG	NEG	[illegible]	[illegible]	[illegible]	[illegible]	[illegible]	[illegible]	[illegible]	[illegible]	[illegible]	[illegible]	[illegible]	[illegible]	[illegible]	[illegible]	[illegible]	[illegible]	[illegible]	[illegible]	[illegible]	[illegible]	[illegible]
02V20680	CC45-MRSA-IV, Berlin EMRSA	POS	POS	POS	NEG	NEG	NEG	NEG	NEG	NEG	NEG	NEG	NEG	NEG	NEG	NEG	NEG	NEG	POS	POS	NEG	NEG	NEG	NEG	NEG	[illegible]	[illegible]	[illegible]	[illegible]	[illegible]	[illegible]	[illegible]	[illegible]	[illegible]	[illegible]	[illegible]	[illegible]	[illegible]	[illegible]	[illegible]	[illegible]	[illegible]	[illegible]	[illegible]	[illegible]	[illegible]
02V21980	CC45-MRSA-IV, Berlin EMRSA	POS	POS	POS	NEG	NEG	POS	NEG	NEG	NEG	NEG	NEG	NEG	NEG	NEG	NEG	POS	POS	POS	POS	POS	POS	NEG	NEG	NEG	[illegible]	[illegible]	[illegible]	[illegible]	[illegible]	[illegible]	[illegible]	[illegible]	[illegible]	[illegible]	[illegible]	[illegible]	[illegible]	[illegible]	[illegible]	[illegible]	[illegible]	[illegible]	[illegible]	[illegible]	[illegible]
02V22570	CC45-MRSA-IV, Berlin EMRSA	POS	POS	POS	NEG	NEG	POS	NEG	NEG	NEG	NEG	NEG	NEG	NEG	NEG	NEG	POS	NEG	POS	POS	POS	NEG	NEG	NEG	NEG	[illegible]	[illegible]	[illegible]	[illegible]	[illegible]	[illegible]	[illegible]	[illegible]	[illegible]	[illegible]	[illegible]	[illegible]	[illegible]	[illegible]	[illegible]	[illegible]	[illegible]	[illegible]	[illegible]	[illegible]	[illegible]
02V22982	CC45-MRSA-IV, Berlin EMRSA	POS	POS	POS	NEG	NEG	NEG	NEG	NEG	NEG	NEG	NEG	NEG	NEG	NEG	NEG	NEG	NEG	POS	POS	NEG	NEG	NEG	NEG	NEG	[illegible]	[illegible]	[illegible]	[illegible]	[illegible]	[illegible]	[illegible]	[illegible]	[illegible]	[illegible]	[illegible]	[illegible]	[illegible]	[illegible]	[illegible]	[illegible]	[illegible]	[illegible]	[illegible]	[illegible]	[illegible]
02V25503	CC45-MRSA-IV, Berlin EMRSA	POS	POS	POS	NEG	NEG	POS	NEG	NEG	NEG	NEG	NEG	NEG	NEG	NEG	NEG	POS	NEG	POS	POS	POS	NEG	NEG	NEG	NEG	[illegible]	[illegible]	[illegible]	[illegible]	[illegible]	[illegible]	[illegible]	[illegible]	[illegible]	[illegible]	[illegible]	[illegible]	[illegible]	[illegible]	[illegible]	[illegible]	[illegible]	[illegible]	[illegible]	[illegible]	[illegible]
02V28454	CC45-MRSA-IV, Berlin EMRSA	POS	POS	POS	NEG	NEG	POS	NEG	NEG	NEG	NEG	NEG	NEG	NEG	NEG	NEG	NEG	NEG	POS	POS	NEG	NEG	NEG	NEG	NEG	[illegible]	[illegible]	[illegible]	[illegible]	[illegible]	[illegible]	[illegible]	[illegible]	[illegible]	[illegible]	[illegible]	[illegible]	[illegible]	[illegible]	[illegible]	[illegible]	[illegible]	[illegible]	[illegible]	[illegible]	[illegible]
02V29373	CC45-MRSA-IV, Berlin EMRSA	POS	POS	POS	NEG	NEG	NEG	NEG	NEG	NEG	NEG	NEG	NEG	NEG	NEG	NEG	NEG	NEG	POS	POS	NEG	NEG	NEG	NEG	NEG	[illegible]	[illegible]	[illegible]	[illegible]	[illegible]	[illegible]	[illegible]	[illegible]	[illegible]	[illegible]	[illegible]	[illegible]	[illegible]	[illegible]	[illegible]	[illegible]	[illegible]	[illegible]	[illegible]	[illegible]	[illegible]
02V30321	CC45-MRSA-IV, Berlin EMRSA	POS	POS	POS	NEG	NEG	POS	NEG	NEG	NEG	NEG	NEG	NEG	NEG	NEG	NEG	NEG	NEG	POS	POS	NEG	NEG	NEG	NEG	NEG	[illegible]	[illegible]	[illegible]	[illegible]	[illegible]	[illegible]	[illegible]	[illegible]	[illegible]	[illegible]	[illegible]	[illegible]	[illegible]	[illegible]	[illegible]	[illegible]	[illegible]	[illegible]	[illegible]	[illegible]	[illegible]
02V30341	CC45-MRSA-IV, Berlin EMRSA	POS	POS	POS	NEG	NEG	NEG	NEG	NEG	NEG	NEG	NEG	NEG	NEG	NEG	NEG	NEG	NEG	POS	POS	NEG	NEG	NEG	NEG	NEG	[illegible]	[illegible]	[illegible]	[illegible]	[illegible]	[illegible]	[illegible]	[illegible]	[illegible]	[illegible]	[illegible]	[illegible]	[illegible]	[illegible]	[illegible]	[illegible]	[illegible]	[illegible]	[illegible]	[illegible]	[illegible]
02V31377	CC45-MRSA-IV, Berlin EMRSA	POS	POS	POS	NEG	NEG	NEG	NEG	NEG	NEG	NEG	NEG	NEG	NEG	NEG	NEG	POS	NEG	POS	POS	POS	NEG	NEG	NEG	NEG	[illegible]	[illegible]	[illegible]	[illegible]	[illegible]	[illegible]	[illegible]	[illegible]	[illegible]	[illegible]	[illegible]	[illegible]	[illegible]	[illegible]	[illegible]	[illegible]	[illegible]	[illegible]	[illegible]	[illegible]	[illegible]
02V31941	CC45-MRSA-IV, Berlin EMRSA	POS	POS	POS	NEG	NEG	NEG	NEG	NEG	NEG	NEG	NEG	NEG	NEG	NEG	NEG	NEG	NEG	POS	POS	NEG	NEG	NEG	NEG	NEG	[illegible]	[illegible]	[illegible]	[illegible]	[illegible]	[illegible]	[illegible]	[illegible]	[illegible]	[illegible]	[illegible]	[illegible]	[illegible]	[illegible]	[illegible]	[illegible]	[illegible]	[illegible]	[illegible]	[illegible]	[illegible]
02V34679	CC45-MRSA-IV, Berlin EMRSA	POS	POS	POS	NEG	NEG	POS	NEG	NEG	NEG	NEG	NEG	NEG	NEG	NEG	NEG	POS	NEG	POS	POS	POS	NEG	NEG	NEG	NEG	[illegible]	[illegible]	[illegible]	[illegible]	[illegible]	[illegible]	[illegible]	[illegible]	[illegible]	[illegible]	[illegible]	[illegible]	[illegible]	[illegible]	[illegible]	[illegible]	[illegible]	[illegible]	[illegible]	[illegible]	[illegible]
02V35779	CC45-MRSA-IV, Berlin EMRSA	POS	POS	POS	NEG		POS	NEG	NEG	NEG	NEG	NEG	NEG	NEG	NEG	NEG	POS	NEG	POS	POS	POS	NEG	NEG	NEG	NEG	[illegible]	[illegible]	[illegible]	[illegible]	[illegible]	[illegible]	[illegible]	[illegible]	[illegible]	[illegible]	[illegible]	[illegible]	[illegible]	[illegible]	[illegible]	[illegible]	[illegible]	[illegible]	[illegible]	[illegible]	[illegible]
02V36419	CC45-MRSA-IV, Berlin EMRSA	POS			NEG		POS	NEG	NEG			NEG	NEG	NEG	NEG	NEG	POS	NEG	POS	POS	POS	NEG		NEG	NEG	[illegible]	[illegible]	[illegible]	[illegible]	[illegible]	[illegible]	[illegible]	[illegible]	[illegible]	[illegible]	[illegible]	[illegible]	[illegible]	[illegible]	[illegible]	[illegible]	[illegible]	[illegible]	[illegible]	[illegible]	[illegible]
02V36469	CC45-MRSA-IV, Berlin EMRSA	POS	POS	POS	NEG	AMB	POS	NEG	NEG	NEG	NEG	NEG	NEG	NEG	NEG	NEG	POS	NEG	POS	POS	NEG	NEG	NEG	NEG	NEG	[illegible]	[illegible]	[illegible]	[illegible]	[illegible]	[illegible]	[illegible]	[illegible]	[illegible]	[illegible]	[illegible]	[illegible]	[illegible]	[illegible]	[illegible]	[illegible]	[illegible]	[illegible]	[illegible]	[illegible]	[illegible]

ISOLATE:	STRAIN	VIRULENCE : TOX.SCHOCK.TOXIN			VIRULENCE : ENTEROTOXINS																						VIRULENCE : HLG AND LEUKOCIDINS												
		toxic shock syndrome toxin 1			enterotoxin A	enterotoxin A, allele from strain 320E	enterotoxin A, allele from strain N315 = entero-toxin P	enterotoxin B	enterotoxin C	enterotoxin D	enterotoxin E	enterotoxin G	enterotoxin H	enterotoxin I	enterotoxin J	enterotoxin K	enterotoxin L	enterotoxin M	enterotoxin N		enterotoxin O	enterotoxin Q	enterotoxin R	enterotoxin U	enterotoxin-like protein ORF CM14	haemolysin gamma / leukocidin, component B	haemolysin gamma / leukocidin, component C		haemolysin gamma, component A	Panton Valentine leukocidin F component	Panton Valentine leukocidin S component	F component from hypothetical leukocidin from ruminants	S component from hypothetical leukocidin from ruminants	leukocidin D component	leukocidin E component	leukocidin/ haemolysin toxin family protein	leukocidin/haemolysin toxin family protein	
		tst1 (consensus)	tst1 ("human" allele)	tst1 ("bovine" allele)	sea	sea (320E)	sea (N315) / sep	seb	sec	sed	sef	seg	seh	sei	sej	sek	sel	selm	seln (consensus)	seln (other than RF122)	selo	seq	ser	selu	ORF CM14	lukF	lukS	lukS (ST22+ST45)	hlgA	lukF-PV	lukS-PV	lukF-PV (P83)	lukM	lukD	lukE	lukX	lukY	lukY (ST30+ST45)
**2000**																																						
00V07201	CC5/ST228-MRSA, South German EMRSA (truncated SCCmec element)	NEG	NEG	NEG	NEG	NEG	NEG	NEG	NEG	NEG	NEG	POS	NEG	POS	NEG	NEG	NEG	POS	POS	POS	POS	NEG	NEG	POS	NEG	POS	POS	AMB	POS	NEG	NEG	NEG	NEG	POS	POS	POS	POS	NEG
00V07634	CC5/ST228-MRSA, South German EMRSA (truncated SCCmec element)	NEG	NEG	NEG	NEG	NEG	NEG	NEG	NEG	NEG	NEG	POS	NEG	POS	NEG	NEG	NEG	POS	POS	POS	POS	NEG	NEG	POS	NEG	POS	POS	POS	POS	NEG	NEG	NEG	NEG	POS	POS	POS	POS	NEG
00V08701	CC5/ST228-MRSA, South German EMRSA (truncated SCCmec element)	NEG	NEG	NEG	NEG	NEG	NEG	NEG	NEG	NEG	NEG	POS	NEG	POS	NEG	NEG	NEG	POS	POS	POS	POS	NEG	NEG	POS	NEG	POS	POS	NEG	POS	NEG	NEG	NEG	NEG	POS	POS	POS	POS	NEG
00V09056	CC5/ST228-MRSA, South German EMRSA (truncated SCCmec element)	NEG	NEG	NEG	NEG	NEG	NEG	NEG	NEG	NEG	NEG	POS	NEG	POS	NEG	NEG	NEG	POS	POS	POS	POS	NEG	NEG	POS	NEG	POS	POS	AMB	POS	NEG	NEG	NEG	NEG	POS	POS	POS	POS	NEG
00V10190	CC5/ST228-MRSA, South German EMRSA (truncated SCCmec element)	NEG	NEG	NEG	NEG	NEG	NEG	NEG	NEG	NEG	NEG	POS	NEG	POS	NEG	NEG	NEG	POS	POS	POS	POS	NEG	NEG	POS	NEG	POS	POS	AMB	POS	NEG	NEG	NEG	NEG	POS	POS	POS	POS	NEG
00V10643	CC5/ST228-MRSA, South German EMRSA (truncated SCCmec element)	NEG	NEG	NEG	NEG	NEG	NEG	NEG	NEG	NEG	NEG	POS	NEG	POS	NEG	NEG	NEG	POS	POS	POS	POS	NEG	NEG	POS	NEG	POS	POS	AMB	POS	NEG	NEG	NEG	NEG	POS	POS	POS	POS	NEG
00V11732	CC5/ST228-MRSA, South German EMRSA (truncated SCCmec element)	NEG	NEG	NEG	NEG	NEG	NEG	NEG	NEG	NEG	NEG	POS	NEG	POS	NEG	NEG	NEG	POS	POS	POS	POS	NEG	NEG	POS	NEG	POS	POS	AMB	POS	NEG	NEG	NEG	NEG	POS	POS	POS	POS	NEG
00V12300	CC5/ST228-MRSA, South German EMRSA (truncated SCCmec element)	NEG	NEG	NEG	NEG	NEG	NEG	NEG	NEG	NEG	NEG	POS	NEG	POS	NEG	NEG	NEG	POS	POS	POS	POS	NEG	NEG	POS	NEG	POS	POS	AMB	POS	NEG	NEG	NEG	NEG	POS	POS	POS	POS	NEG
00V14652	CC5/ST228-MRSA, South German EMRSA (truncated SCCmec element)	NEG	NEG	NEG	NEG	NEG	NEG	NEG	NEG	NEG	NEG	POS	NEG	POS	NEG	NEG	NEG	POS	POS	POS	POS	NEG	NEG	POS	NEG	POS	POS	AMB	POS	NEG	NEG	NEG	NEG	POS	POS	POS	POS	NEG
00V24941	CC5/ST228-MRSA, South German EMRSA (truncated SCCmec element)	NEG	NEG	NEG	NEG	NEG	NEG	NEG	NEG	NEG	NEG	POS	NEG	POS	NEG	NEG	NEG	POS	POS	POS	POS	NEG	NEG	POS	NEG	POS	POS	AMB	POS	NEG	NEG	NEG	NEG	POS	POS	POS	POS	NEG
00V29834	CC5/ST228-MRSA, South German EMRSA (truncated SCCmec element)	NEG	NEG	NEG	NEG	NEG	NEG	NEG	NEG	NEG	NEG	POS	NEG	POS	NEG	NEG	NEG	POS	POS	POS	POS	NEG	NEG	POS	NEG	POS	POS	POS	POS	NEG	NEG	NEG	NEG	POS	POS	POS	POS	NEG
00V29909	CC5/ST228-MRSA, South German EMRSA (truncated SCCmec element)	NEG	NEG	NEG	NEG	NEG	NEG	NEG	NEG	NEG	NEG	POS	NEG	POS	NEG	NEG	NEG	POS	POS	POS	POS	NEG	NEG	POS	NEG	POS	POS	NEG	POS	NEG	NEG	NEG	NEG	POS	POS	POS	POS	NEG
00V30456	CC5/ST228-MRSA, South German EMRSA (truncated SCCmec element)	NEG	NEG	NEG	NEG	NEG	NEG	NEG	NEG	NEG	NEG	POS	NEG	POS	NEG	NEG	NEG	POS	POS	POS	POS	NEG	NEG	POS	NEG	POS	POS	NEG	POS	NEG	NEG	NEG	NEG	POS	POS	POS	POS	NEG
00V30827	CC5/ST228-MRSA, South German EMRSA (truncated SCCmec element)	NEG	NEG	NEG	NEG	NEG	NEG	NEG	NEG	NEG	NEG	POS	NEG	POS	NEG	NEG	NEG	POS	POS	POS	POS	NEG	NEG	POS	NEG	POS	POS	POS	POS	NEG	NEG	NEG	NEG	POS	POS	POS	POS	NEG
00V33204	CC5/ST228-MRSA, South German EMRSA (truncated SCCmec element)	NEG	NEG	NEG	NEG	NEG	NEG	NEG	NEG	NEG	NEG	POS	NEG	POS	NEG	NEG	NEG	POS	POS	POS	POS	NEG	NEG	POS	NEG	POS	POS	AMB	POS	NEG	NEG	NEG	NEG	POS	POS	POS	POS	NEG
00V36490	CC5/ST228-MRSA, South German EMRSA (truncated SCCmec element)	NEG	NEG	NEG	NEG	NEG	NEG	NEG	NEG	NEG	NEG	POS	NEG	POS	NEG	NEG	NEG	POS	POS	POS	POS	NEG	NEG	POS	NEG	POS	POS	POS	POS	NEG	NEG	NEG	NEG	POS	POS	POS	POS	NEG
00V37079	CC5/ST228-MRSA, South German EMRSA (truncated SCCmec element)	NEG	NEG	NEG	NEG	NEG	NEG	NEG	NEG	NEG	NEG	POS	NEG	POS	NEG	NEG	NEG	POS	POS	POS	POS	NEG	NEG	POS	NEG	POS	POS	POS	POS	NEG	NEG	NEG	NEG	POS	POS	POS	POS	NEG
00V40116	CC5/ST228-MRSA, South German EMRSA (truncated SCCmec element)	NEG	NEG	NEG	NEG	NEG	NEG	NEG	NEG	NEG	NEG	POS	NEG	POS	NEG	NEG	NEG	POS	POS	POS	POS	NEG	NEG	POS	NEG	POS	POS	POS	POS	NEG	NEG	NEG	NEG	POS	POS	POS	POS	NEG
00V41429	CC5/ST228-MRSA, South German EMRSA (truncated SCCmec element)	NEG	NEG	NEG	NEG	NEG	NEG	NEG	NEG	NEG	NEG	POS	NEG	POS	NEG	NEG	NEG	POS	POS	POS	POS	NEG	NEG	POS	NEG	POS	POS	POS	POS	NEG	NEG	NEG	NEG	POS	POS	POS	POS	NEG
00V42118	CC5/ST228-MRSA, South German EMRSA (truncated SCCmec element)	NEG	NEG	NEG	NEG	NEG	NEG	NEG	NEG	NEG	NEG	POS	NEG	POS	NEG	NEG	NEG	POS	POS	POS	POS	NEG	NEG	POS	NEG	POS	POS	POS	POS	NEG	NEG	NEG	NEG	POS	POS	POS	POS	NEG
00V44135	CC5/ST228-MRSA, South German EMRSA (truncated SCCmec element)	NEG	NEG	NEG	NEG	NEG	NEG	NEG	NEG	NEG	NEG	POS	NEG	POS	NEG	NEG	NEG	POS	POS	POS	POS	NEG	NEG	POS	NEG	POS	POS	POS	POS	NEG	NEG	NEG	NEG	POS	POS	POS	POS	NEG
00V44538	CC5/ST228-MRSA, South German EMRSA (truncated SCCmec element)	NEG	NEG	NEG	NEG	NEG	NEG	NEG	NEG	NEG	NEG	POS	NEG	POS	NEG	NEG	NEG	POS	POS	POS	POS	NEG	NEG	POS	NEG	POS	POS	AMB	POS	NEG	NEG	NEG	NEG	POS	POS	POS	POS	NEG
00V44990	CC5/ST228-MRSA, South German EMRSA (truncated SCCmec element)	NEG	NEG	NEG	NEG	NEG	NEG	NEG	NEG	NEG	NEG	POS	NEG	POS	NEG	NEG	NEG	POS	POS	POS	POS	NEG	NEG	POS	NEG	POS	POS	AMB	POS	NEG	NEG	NEG	NEG	POS	POS	POS	POS	NEG
00V46910	CC5/ST228-MRSA, South German EMRSA (truncated SCCmec element)	NEG	NEG	NEG	NEG	NEG	NEG	NEG	NEG	NEG	NEG	POS	NEG	POS	NEG	NEG	NEG	POS	POS	POS	POS	NEG	NEG	POS	NEG	POS	POS	AMB	POS	NEG	NEG	NEG	NEG	POS	POS	POS	POS	NEG
00V07063	CC5/ST228-MRSA-I, South German EMRSA	NEG	NEG	NEG	NEG	NEG	NEG	NEG	NEG	NEG	NEG	POS	NEG	POS	NEG	NEG	NEG	POS	POS	POS	POS	NEG	NEG	POS	NEG	POS	POS	POS	POS	NEG	NEG	AMB	NEG	POS	POS	POS	POS	NEG
00V46921	CC5-MRSA-II, Rhine-Hesse EMRSA/UK-EMRSA-3	NEG	NEG	NEG	NEG	NEG	POS	NEG	NEG	POS	NEG	POS	NEG	POS	POS	NEG	NEG	POS	POS	POS	POS	NEG	POS	POS	NEG	POS	POS	POS	POS	NEG	NEG	NEG	NEG	POS	POS	POS	POS	NEG
00V08510	CC8/ST254-MRSA-(atypical SCCmec), Hannover EMRSA	NEG	NEG	NEG	POS	NEG	NEG	POS	NEG	NEG	NEG	NEG	NEG	NEG	NEG	POS	NEG	NEG	NEG	NEG	NEG	POS	NEG	NEG	NEG	POS	POS	NEG	POS	NEG	NEG	NEG	NEG	POS	POS	POS	POS	NEG
00V25538	CC8/ST254-MRSA-(atypical SCCmec), Hannover EMRSA	NEG	NEG	NEG	POS	NEG	NEG	POS	NEG	NEG	NEG	NEG	NEG	NEG	NEG	POS	NEG	NEG	NEG	NEG	NEG	POS	NEG	NEG	NEG	POS	POS	NEG	POS	NEG	NEG	NEG	NEG	POS	POS	POS	POS	NEG
00V38664	CC8/ST254-MRSA-(atypical SCCmec), Hannover EMRSA	NEG	NEG	NEG	POS	NEG	NEG	POS	NEG	NEG	NEG	NEG	NEG	NEG	NEG	POS	NEG	NEG	NEG	NEG	NEG	POS	NEG	NEG	NEG	POS	POS	POS	POS	NEG	NEG	AMB	NEG	POS	POS	POS	POS	NEG
00V46488	CC8/ST254-MRSA-(atypical SCCmec), Hannover EMRSA	NEG	NEG	NEG	POS	NEG	NEG	POS	NEG	NEG	NEG	NEG	NEG	NEG	NEG	POS	NEG	NEG	NEG	NEG	NEG	POS	NEG	NEG	NEG	POS	POS	AMB	POS	NEG	NEG	NEG	NEG	POS	POS	POS	POS	NEG
00V44035	CC8/ST254-MRSA-IV/V, UK-EMRSA-10/Hannover EMRSA	NEG	NEG	NEG	POS	NEG	NEG	POS	NEG	NEG	NEG	NEG	NEG	NEG	NEG	POS	NEG	NEG	NEG	NEG	NEG	POS	NEG	NEG	NEG	POS	POS	NEG	POS	NEG	NEG	NEG	NEG	POS	POS	NEG	POS	NEG
00V13324	CC22-MRSA-IV [PVL+]	NEG	NEG	NEG	NEG	NEG	NEG	NEG	NEG	NEG	NEG	POS	NEG	POS	NEG	NEG	NEG	POS	POS	POS	POS	NEG	NEG	POS	NEG	NEG	NEG	POS	AMB	AMB	POS	NEG	NEG	NEG	NEG	AMB	POS	NEG
00B02046	CC45-MRSA-IV, Berlin EMRSA	NEG	NEG	NEG	NEG	NEG	NEG	NEG	NEG	NEG	NEG	NEG	NEG	POS	NEG	NEG	NEG	POS	POS	POS	POS	NEG	NEG	POS	NEG	POS	AMB	POS	POS	NEG	NEG	NEG	NEG	NEG	NEG	POS	NEG	POS
00V11299	CC45-MRSA-IV, Berlin EMRSA	NEG	NEG	NEG	NEG	NEG	NEG	NEG	NEG	NEG	NEG	NEG	NEG	POS	NEG	NEG	NEG	POS	POS	POS	POS	NEG	NEG	POS	NEG	POS	AMB	POS	POS	NEG	NEG	NEG	NEG	NEG	NEG	POS	NEG	POS
00V14125	CC45-MRSA-IV, Berlin EMRSA	NEG	NEG	NEG	NEG	NEG	NEG	NEG	NEG	NEG	NEG	NEG	NEG	POS	NEG	NEG	NEG	POS	POS	POS	POS	NEG	NEG	POS	NEG	POS	AMB	POS	POS	NEG	NEG	NEG	NEG	NEG	NEG	POS	NEG	POS
00V16040	CC45-MRSA-IV, Berlin EMRSA	NEG	NEG	NEG	NEG	NEG	NEG	NEG	NEG	NEG	NEG	POS	NEG	POS	NEG	NEG	NEG	POS	POS	POS	POS	NEG	NEG	POS	NEG	POS	AMB	POS	POS	NEG	NEG	NEG	NEG	NEG	NEG	POS	NEG	POS
00V25025	CC45-MRSA-IV, Berlin EMRSA	NEG	NEG	NEG	NEG	NEG	NEG	NEG	NEG	NEG	NEG	NEG	NEG	POS	NEG	NEG	NEG	POS	POS	POS	POS	NEG	NEG	POS	NEG	POS	AMB	POS	POS	NEG	NEG	NEG	NEG	NEG	NEG	POS	NEG	POS
00V29618	CC45-MRSA-IV, Berlin EMRSA	NEG	NEG	NEG	NEG	NEG	NEG	NEG	NEG	NEG	NEG	POS	NEG	POS	NEG	NEG	NEG	POS	POS	POS	POS	NEG	NEG	POS	NEG	POS	AMB	POS	POS	NEG	NEG	NEG	NEG	NEG	NEG	POS	NEG	POS
00V30989	CC45-MRSA-IV, Berlin EMRSA	NEG	NEG	NEG	NEG	NEG	NEG	NEG	NEG	NEG	NEG	POS	NEG	POS	NEG	NEG	NEG	POS	POS	POS	POS	NEG	NEG	POS	NEG	POS	AMB	POS	POS	NEG	NEG	NEG	NEG	NEG	NEG	POS	NEG	POS
00V31007	CC45-MRSA-IV, Berlin EMRSA	NEG	NEG	NEG	NEG	NEG	NEG	NEG	NEG	NEG	NEG	NEG	NEG	POS	NEG	NEG	NEG	POS	POS	POS	POS	NEG	NEG	POS	NEG	POS	AMB	POS	POS	NEG	NEG	NEG	NEG	NEG	NEG	POS	NEG	POS
00V31700	CC45-MRSA-IV, Berlin EMRSA	NEG	NEG	NEG	NEG	NEG	NEG	NEG	NEG	NEG	NEG	NEG	NEG	POS	NEG	NEG	NEG	POS	POS	POS	POS	NEG	NEG	POS	NEG	POS	AMB	POS	POS	NEG	NEG	NEG	NEG	NEG	NEG	POS	NEG	POS
00V32193	CC45-MRSA-IV, Berlin EMRSA	NEG	NEG	NEG	NEG	NEG	NEG	NEG	NEG	NEG	NEG	NEG	NEG	POS	NEG	NEG	NEG	POS	POS	POS	POS	NEG	NEG	POS	NEG	POS	POS	POS	POS	NEG	NEG	NEG	NEG	NEG	NEG	POS	NEG	POS
00V43085	CC45-MRSA-IV, Berlin EMRSA	NEG	NEG	NEG	NEG	NEG	NEG	NEG	NEG	NEG	NEG	POS	NEG	POS	NEG	NEG	NEG	POS	POS	POS	POS	NEG	NEG	POS	NEG	POS	POS	POS	POS	NEG	NEG	NEG	NEG	NEG	NEG	POS	NEG	POS
00V44382	CC45-MRSA-IV, Berlin EMRSA	NEG	NEG	NEG	NEG	NEG	NEG	NEG	NEG	NEG	NEG	NEG	NEG	POS	NEG	NEG	NEG	POS	POS	POS	POS	NEG	NEG	POS	NEG	POS	NEG	POS	POS	NEG	NEG	NEG	NEG	NEG	NEG	POS	NEG	POS
00V46648	CC45-MRSA-IV, Berlin EMRSA	NEG	NEG	NEG	NEG	NEG	NEG	NEG	NEG	NEG	NEG	NEG	NEG	POS	NEG	NEG	NEG	POS	POS	POS	POS	NEG	NEG	POS	NEG	POS	POS	POS	POS	NEG	NEG	NEG	NEG	NEG	NEG	POS	NEG	POS
00V46843	CC45-MRSA-IV, Berlin EMRSA	NEG	NEG	NEG	NEG	NEG	NEG	NEG	NEG	NEG	NEG	POS	NEG	POS	NEG	NEG	NEG	POS	POS	POS	POS	NEG	NEG	POS	NEG	POS	POS	POS	POS	NEG	NEG	NEG	NEG	NEG	NEG	POS	NEG	POS
00V47014	CC45-MRSA-IV, Berlin EMRSA	NEG	NEG	NEG	NEG	NEG	NEG	NEG	NEG	NEG	NEG	POS	NEG	POS	NEG	NEG	NEG	POS	POS	POS	POS	NEG	NEG	POS	NEG	POS	POS	POS	POS	NEG	NEG	NEG	NEG	NEG	NEG	POS	NEG	POS
00V47053	CC45-MRSA-IV, Berlin EMRSA	NEG	NEG	NEG	NEG	NEG	NEG	NEG	NEG	NEG	NEG	NEG	NEG	POS	NEG	NEG	NEG	POS	POS	POS	POS	NEG	NEG	POS	NEG	POS	NEG	POS	POS	NEG	NEG	NEG	NEG	NEG	NEG	AMB	NEG	POS
00V47227	CC45-MRSA-IV, Berlin EMRSA	NEG	NEG	NEG	NEG	NEG	NEG	NEG	NEG	NEG	NEG	NEG	NEG	POS	NEG	NEG	NEG	POS	POS	POS	POS	NEG	NEG	POS	NEG	POS	POS	POS	POS	NEG	NEG	NEG	NEG	NEG	NEG	POS	NEG	POS
00V47429	CC45-MRSA-IV, Berlin EMRSA	NEG	NEG	NEG	NEG	NEG	NEG	NEG	NEG	NEG	NEG	NEG	NEG	POS	NEG	NEG	NEG	POS	POS	POS	POS	NEG	NEG	POS	NEG	POS	AMB	POS	POS	NEG	NEG	NEG	NEG	NEG	NEG	POS	NEG	POS
00V47531	CC45-MRSA-IV, Berlin EMRSA	NEG	NEG	NEG	NEG	NEG	NEG	NEG	NEG	NEG	NEG	POS	NEG	POS	NEG	NEG	NEG	POS	POS	POS	POS	NEG	NEG	POS	NEG	POS	POS	POS	POS	NEG	NEG	NEG	NEG	NEG	NEG	POS	NEG	POS
**2001**																																						
01V27106	CC1-MRSA-IV, WA MRSA-1/57	NEG	NEG	NEG	POS	NEG	NEG	NEG	NEG	NEG	NEG	NEG	POS	NEG	NEG	POS	NEG	NEG	NEG	NEG	NEG	POS	NEG	NEG	NEG	POS	POS	AMB	POS	NEG	NEG	NEG	NEG	POS	POS	POS	POS	NEG
01V41025	CC5/ST228-MRSA-I, South German EMRSA	NEG	NEG	NEG	POS	AMB	NEG	NEG	NEG	NEG	NEG	NEG	NEG	NEG	NEG	NEG	NEG	NEG	NEG	NEG	NEG	NEG	NEG	NEG	NEG	POS	POS	POS	POS	NEG	NEG	NEG	NEG	POS	NEG	POS	POS	NEG
01V12125	CC5-MRSA-II, Rhine-Hesse EMRSA/UK-EMRSA-3	NEG	NEG	NEG	NEG	NEG	POS	NEG	NEG	POS	NEG	POS	NEG	POS	POS	NEG	NEG	POS	POS	POS	POS	NEG	POS	POS	NEG	POS	POS	POS	POS	NEG	NEG	NEG	NEG	POS	POS	POS	POS	NEG
01V29747	CC5-MRSA-II, Rhine-Hesse EMRSA/UK-EMRSA-3	NEG	NEG	NEG	NEG	NEG	POS	NEG	NEG	POS	NEG	POS	NEG	POS	POS	NEG	NEG	POS	POS	POS	POS	NEG	POS	POS	NEG	POS	POS	POS	POS	NEG	NEG	NEG	NEG	POS	POS	POS	POS	NEG
01V32464	CC5-MRSA-II, Rhine-Hesse EMRSA/UK-EMRSA-3	NEG	NEG	NEG	NEG	NEG	POS	NEG	NEG	POS	NEG	POS	NEG	POS	POS	NEG	NEG	POS	POS	POS	POS	NEG	POS	POS	NEG	POS	POS	NEG	POS	NEG	NEG	NEG	NEG	POS	POS	AMB	POS	NEG
01V39703	CC5-MRSA-II, Rhine-Hesse EMRSA/UK-EMRSA-3	NEG	NEG	NEG	NEG	NEG	POS	NEG	NEG	POS	NEG	POS	NEG	POS	POS	NEG	NEG	POS	POS	POS	POS	NEG	POS	POS	NEG	POS	POS	AMB	POS	NEG	NEG	NEG	NEG	POS	POS	POS	POS	NEG
01V40228	CC5-MRSA-II, Rhine-Hesse EMRSA/UK-EMRSA-3		NEG	NEG	NEG	NEG	POS	NEG	NEG	POS	NEG	POS	NEG	POS	POS		NEG	POS		POS	POS		POS			POS	POS		POS	NEG	NEG	NEG	NEG	POS	POS	RANDOM	POS	NEG
01B05861	CC8/ST239-MRSA-III, Vienna/Hungarian/Brazilian Clone	NEG	NEG	NEG	POS	NEG	NEG	NEG	NEG	NEG	NEG	NEG	NEG	NEG	NEG	NEG	NEG	NEG	NEG	NEG	NEG	NEG	NEG	NEG	NEG	POS	POS	NEG	POS	NEG	NEG	NEG	NEG	POS	POS	POS	POS	NEG
01V34572	CC8/ST239-MRSA-III, Vienna/Hungarian/Brazilian Clone	NEG	NEG	NEG	POS	NEG	NEG	NEG	NEG	NEG	NEG	NEG	NEG	NEG	NEG	NEG	NEG	NEG	NEG	NEG	NEG	NEG	NEG	NEG	NEG	POS	POS	NEG	POS	NEG	NEG	NEG	NEG	POS	POS	POS	POS	NEG
01V36469	CC8/ST239-MRSA-III, Vienna/Hungarian/Brazilian Clone	NEG	NEG	NEG	POS	NEG	NEG	NEG	NEG	NEG	NEG	NEG	NEG	NEG	NEG	NEG	NEG	NEG	NEG	NEG	NEG	NEG	NEG	NEG	NEG	POS	POS	NEG	POS	NEG	NEG	NEG	NEG	POS	POS	POS	POS	NEG
01V39123	CC8/ST239-MRSA-III, Vienna/Hungarian/Brazilian Clone	NEG	AMB	NEG	POS	NEG	NEG	NEG	NEG	NEG	NEG	NEG	NEG	NEG	NEG	NEG	NEG	NEG	NEG	NEG	NEG	NEG	NEG	NEG	NEG	POS	POS	POS	POS	NEG	NEG	NEG	NEG	POS	POS	POS	POS	NEG
01V39143	CC8/ST239-MRSA-III, Vienna/Hungarian/Brazilian Clone	NEG	NEG	NEG	POS	NEG	NEG	NEG	NEG	NEG	NEG	NEG	NEG	NEG	NEG	NEG	NEG	NEG	NEG	NEG	NEG	NEG	NEG	NEG	NEG	POS	POS	NEG	POS	NEG	NEG	NEG	NEG	POS	POS	AMB	POS	NEG
01V10962	CC8/ST254-MRSA-(atypical SCCmec), Hannover EMRSA	NEG	NEG	NEG	POS	NEG	NEG	POS	NEG	NEG	NEG	NEG	NEG	NEG	NEG	POS	NEG	NEG	NEG	NEG	NEG	POS	NEG	NEG	NEG	POS	POS	POS	POS	NEG	NEG	NEG	NEG	POS	POS	POS	POS	NEG
01V10963	CC8/ST254-MRSA-IV/V, UK-EMRSA-10/Hannover EMRSA	NEG	NEG	NEG	POS	AMB	NEG	POS	NEG	NEG	NEG	NEG	NEG	NEG	NEG	POS	NEG	NEG	NEG	NEG	NEG	POS	NEG	NEG	NEG	POS	POS	POS	POS	NEG	NEG	NEG	NEG	POS	POS	POS	POS	NEG
01V36287	CC8/ST254-MRSA-IV/V, UK-EMRSA-10/Hannover EMRSA	NEG	NEG	NEG	POS	NEG	NEG	POS	NEG	NEG	NEG	NEG	NEG	NEG	NEG	POS	NEG	NEG	NEG	NEG	NEG	POS	NEG	NEG	NEG	POS	POS	AMB	POS	NEG	NEG	NEG	NEG	POS	POS	POS	POS	NEG
01V25329	CC22-MRSA-IV [PVL+]		NEG	NEG	NEG	NEG	NEG	NEG	NEG	NEG	NEG	POS	NEG	POS	NEG	NEG	NEG	POS	POS	POS	POS	NEG	NEG	POS	NEG	POS	AMB	POS	POS	POS	POS	NEG	NEG	NEG	NEG	NEG	POS	NEG
01V07770	CC22-MRSA-IV, Barnim/UK-EMRSA-15		NEG	NEG	NEG	NEG	NEG	NEG	POS	NEG	NEG	POS	NEG	POS	NEG		POS	POS		POS	POS		NEG			POS	NEG		NEG	NEG	NEG	NEG	NEG	NEG	NEG	NEG	POS	NEG
01V20122	CC22-MRSA-IV, Barnim/UK-EMRSA-15	NEG	NEG	NEG	NEG	NEG	NEG	NEG	POS	NEG	NEG	AMB	NEG	POS	NEG	NEG	POS	POS	POS	POS	POS	NEG	NEG	POS	NEG	NEG	NEG	POS	AMB	NEG	NEG	NEG	NEG	NEG	NEG	POS	POS	NEG
01V43277	CC22-MRSA-IV, Barnim/UK-EMRSA-15	NEG	NEG	NEG	NEG	NEG	NEG	NEG	POS	NEG	NEG	POS	NEG	POS	NEG	NEG	POS	POS	POS	POS	POS	NEG	NEG	POS	NEG	POS	POS	POS	POS	NEG	NEG	NEG	NEG	NEG	NEG	POS	POS	NEG
01F02744	CC45-MRSA-IV, Berlin EMRSA	NEG	NEG	NEG	NEG	NEG	NEG	NEG	NEG	NEG	NEG	POS	NEG	POS	NEG	NEG	NEG	POS	POS	POS	POS	NEG	NEG	POS	NEG	POS	NEG	POS	POS	NEG	NEG	NEG	NEG	NEG	NEG	POS	NEG	POS
01V01127	CC45-MRSA-IV, Berlin EMRSA	NEG	NEG	NEG	NEG	NEG	NEG	NEG	NEG	NEG	NEG	POS	NEG	POS	NEG	NEG	NEG	POS	POS	POS	POS	NEG	NEG	POS	NEG	POS	NEG	POS	POS	NEG	NEG	NEG	NEG	NEG	NEG	POS	NEG	POS
01V01445	CC45-MRSA-IV, Berlin EMRSA	NEG	NEG	NEG	NEG	NEG	NEG	NEG	NEG	NEG	NEG	NEG	NEG	POS	NEG	NEG	NEG	POS	POS	POS	POS	NEG	NEG	POS	NEG	POS	AMB	POS	POS	NEG	NEG	NEG	NEG	NEG	NEG	POS	NEG	POS
01V02575	CC45-MRSA-IV, Berlin EMRSA	NEG	NEG	NEG	NEG	NEG	NEG	NEG	NEG	NEG	NEG	NEG	NEG	POS	NEG	NEG	NEG	POS	POS	POS	POS	NEG	NEG	POS	NEG	POS	NEG	POS	POS	NEG	NEG	NEG	NEG	NEG	NEG	POS	NEG	POS
01V02636	CC45-MRSA-IV, Berlin EMRSA	NEG	NEG	NEG	NEG	NEG	NEG	NEG	NEG	NEG	NEG	NEG	NEG	POS	NEG	NEG	NEG	POS	POS	POS	POS	NEG	NEG	POS	NEG	POS	AMB	POS	POS	NEG	NEG	NEG	NEG	NEG	NEG	POS	NEG	POS
01V03483	CC45-MRSA-IV, Berlin EMRSA	NEG	NEG	NEG	NEG	NEG	NEG	NEG	NEG	NEG	NEG	NEG	NEG	POS	NEG	NEG	NEG	POS	POS	POS	POS	NEG	NEG	POS	NEG	POS	NEG	POS	POS	NEG	NEG	NEG	NEG	NEG	NEG	POS	NEG	POS
01V04728	CC45-MRSA-IV, Berlin EMRSA	NEG	NEG	NEG	NEG	NEG	NEG	NEG	NEG	NEG	NEG	POS	NEG	POS	NEG	NEG	NEG	POS	POS	POS	POS	NEG	NEG	POS	NEG	POS	AMB	POS	POS	NEG	NEG	NEG	NEG	NEG	NEG	AMB	NEG	POS
01V05972	CC45-MRSA-IV, Berlin EMRSA	NEG	NEG	NEG	NEG	NEG	NEG	NEG	NEG	NEG	NEG	NEG	NEG	POS	NEG	NEG	NEG	POS	POS	POS	POS	NEG	NEG	POS	NEG	POS	NEG	POS	POS	NEG	NEG	NEG	NEG	NEG	NEG	POS	NEG	POS
01V07139	CC45-MRSA-IV, Berlin EMRSA	NEG	NEG	NEG	NEG	NEG	NEG	NEG	NEG	NEG	NEG	NEG	NEG	POS	NEG	NEG	NEG	POS	POS	POS	POS	NEG	NEG	POS	NEG	POS	POS	POS	POS	NEG	NEG	NEG	NEG	NEG	NEG	POS	NEG	POS
01V08133	CC45-MRSA-IV, Berlin EMRSA	NEG	NEG	NEG	NEG	NEG	NEG	NEG	NEG	NEG	NEG	NEG	NEG	POS	NEG	NEG	NEG	POS	POS	POS	POS	NEG	NEG	POS	NEG	POS	AMB	POS	POS	NEG	NEG	NEG	NEG	NEG	NEG	POS	NEG	POS
01V09872	CC45-MRSA-IV, Berlin EMRSA	NEG	NEG	NEG	NEG	NEG	NEG	NEG	NEG	NEG	NEG	NEG	NEG	POS	NEG	NEG	NEG	POS	POS	POS	POS	NEG	NEG	POS	NEG	POS	AMB	POS	POS	NEG	NEG	NEG	NEG	NEG	NEG	POS	NEG	POS
01V12465	CC45-MRSA-IV, Berlin EMRSA	NEG	NEG	NEG	NEG	NEG	NEG	NEG	NEG	NEG	NEG	NEG	NEG	POS	NEG	NEG	NEG	POS	POS	POS	POS	NEG	NEG	POS	NEG	POS	NEG	POS	POS	NEG	NEG	NEG	NEG	NEG	NEG	POS	NEG	POS
01V16151	CC45-MRSA-IV, Berlin EMRSA	NEG	NEG	NEG	NEG	NEG	NEG	NEG	NEG	NEG	NEG	POS	NEG	POS	NEG	NEG	NEG	POS	POS	POS	POS	NEG	NEG	POS	NEG	POS	NEG	POS	POS	NEG	NEG	NEG	NEG	NEG	NEG	POS	NEG	POS
01V16873	CC45-MRSA-IV, Berlin EMRSA	NEG	NEG	NEG	NEG	NEG	NEG	NEG	NEG	NEG	NEG	NEG	NEG	POS	NEG	NEG	NEG	POS	POS	POS	POS	NEG	NEG	POS	NEG	POS	AMB	POS	POS	NEG	NEG	NEG	NEG	NEG	NEG	POS	NEG	POS
01V17796	CC45-MRSA-IV, Berlin EMRSA	NEG	NEG	NEG	NEG	NEG	NEG	NEG	NEG	NEG	NEG	NEG	NEG	POS	NEG	NEG	NEG	POS	POS	POS	POS	NEG	NEG	POS	NEG	POS	AMB	POS	POS	NEG	NEG	NEG	NEG	NEG	NEG	POS	NEG	POS
01V18681	CC45-MRSA-IV, Berlin EMRSA	NEG	NEG	NEG	NEG	NEG	NEG	NEG	NEG	NEG	NEG	NEG	NEG	POS	NEG	NEG	NEG	POS	POS	POS	POS	NEG	NEG	POS	NEG	POS	AMB	POS	POS	NEG	NEG	NEG	NEG	NEG	NEG	POS	NEG	POS
01V20066	CC45-MRSA-IV, Berlin EMRSA	NEG	NEG	NEG	NEG	NEG	NEG	NEG	NEG	NEG	NEG	NEG	NEG	POS	NEG	NEG	NEG	POS	POS	POS	POS	NEG	NEG	POS	NEG	POS	AMB	POS	POS	NEG	NEG	NEG	NEG	NEG	NEG	POS	NEG	POS
01V23050	CC45-MRSA-IV, Berlin EMRSA	NEG	NEG	NEG	NEG	NEG	NEG	NEG	NEG	NEG	NEG	POS	NEG	POS	NEG	NEG	NEG	POS	POS	POS	POS	NEG	NEG	POS	NEG	POS	AMB	POS	POS	NEG	NEG	NEG	NEG	NEG	NEG	POS	NEG	POS
01V25584	CC45-MRSA-IV, Berlin EMRSA	NEG	NEG	NEG	NEG	NEG	NEG	NEG	NEG	NEG	NEG	NEG	NEG	POS	NEG	NEG	NEG	POS	POS	AMB	AMB	NEG	NEG	POS	NEG	POS	NEG	POS	NEG	NEG	NEG	NEG	NEG	NEG	NEG	AMB	NEG	AMB
01V31705	CC45-MRSA-IV, Berlin EMRSA	NEG	NEG	NEG	NEG	NEG	NEG	NEG	NEG	NEG	NEG	POS	NEG	POS	NEG	NEG	NEG	POS	POS	POS	POS	NEG	NEG	POS	NEG	POS	POS	POS	POS	NEG	NEG	NEG	NEG	NEG	NEG	POS	NEG	POS
01V34968	CC45-MRSA-IV, Berlin EMRSA	NEG	NEG	NEG	NEG	NEG	NEG	NEG	NEG	NEG	NEG	NEG	NEG	POS	NEG	NEG	NEG	POS	POS	POS	POS	NEG	NEG	POS	NEG	POS	POS	POS	POS	NEG	NEG	NEG	NEG	NEG	NEG	POS	NEG	POS
01V36186	CC45-MRSA-IV, Berlin EMRSA	NEG	NEG	NEG	NEG	NEG	NEG	NEG	NEG	NEG	NEG	POS	NEG	POS	NEG	NEG	NEG	POS	POS	POS	POS	NEG	NEG	POS	NEG	POS	POS	POS	POS	NEG	NEG	NEG	NEG	NEG	NEG	POS	NEG	POS
01V36534	CC45-MRSA-IV, Berlin EMRSA	NEG	NEG	NEG	NEG	NEG	NEG	NEG	NEG	NEG	NEG	NEG	NEG	POS	NEG	NEG	NEG	POS	POS	POS	POS	NEG	NEG	POS	NEG	POS	NEG	POS	POS	NEG	NEG	NEG	NEG	NEG	NEG	POS	NEG	POS
01V36588	CC45-MRSA-IV, Berlin EMRSA	NEG	NEG	NEG	NEG	NEG	NEG	NEG	NEG	NEG	NEG	POS	NEG	POS	NEG	NEG	NEG	POS	POS	POS	POS	NEG	NEG	POS	NEG	POS	AMB	POS	POS	NEG	NEG	NEG	NEG	NEG	NEG	POS	NEG	POS
01V37990	CC45-MRSA-IV, Berlin EMRSA	NEG	NEG	NEG	NEG	NEG	NEG	NEG	NEG	NEG	NEG	NEG	NEG	POS	NEG	NEG	NEG	POS	POS	POS	POS	NEG	NEG	POS	NEG	POS	POS	POS	POS	NEG	NEG	NEG	NEG	NEG	NEG	POS	NEG	POS
01V39610	CC45-MRSA-IV, Berlin EMRSA	NEG	NEG	NEG	NEG	NEG	NEG	NEG	NEG	NEG	NEG	POS	NEG	POS	NEG	NEG	NEG	POS	POS	POS	POS	NEG	NEG	POS	NEG	POS	POS	POS	POS	NEG	NEG	NEG	NEG	NEG	NEG	POS	NEG	POS
01V40229	CC45-MRSA-IV, Berlin EMRSA	NEG	NEG	NEG	NEG	NEG	NEG	NEG	NEG	NEG	NEG	NEG	NEG	POS	NEG	NEG	NEG	POS	POS	POS	POS	NEG	NEG	POS	NEG	POS	POS	POS	POS	NEG	NEG	NEG	NEG	NEG	NEG	POS	NEG	POS
01V40374	CC45-MRSA-IV, Berlin EMRSA		NEG	NEG	NEG	NEG	NEG	NEG	NEG	NEG	NEG	POS	NEG	POS	NEG		NEG	POS		POS	POS		NEG			POS	NEG		POS	NEG	NEG	NEG	NEG	NEG	NEG	POS	NEG	POS
01V41056	CC45-MRSA-IV, Berlin EMRSA	NEG	NEG	NEG	NEG	NEG	NEG	NEG	NEG	NEG	NEG	POS	NEG	POS	NEG	NEG	NEG	POS	POS	POS	POS	NEG	NEG	POS	NEG	POS	NEG	POS	POS	NEG	NEG	NEG	NEG	NEG	NEG	POS	NEG	POS
01V42009	CC45-MRSA-IV, Berlin EMRSA	NEG	NEG	NEG	NEG	NEG	NEG	NEG	NEG	NEG	NEG	POS	NEG	POS	NEG	NEG	NEG	POS	POS	POS	POS	NEG	NEG	POS	NEG	POS	POS	POS	POS	NEG	NEG	NEG	NEG	NEG	NEG	POS	NEG	POS
01V43511	CC45-MRSA-IV, Berlin EMRSA	NEG	NEG	NEG	NEG	NEG	NEG	NEG	NEG	NEG	NEG	NEG	NEG	POS	NEG	NEG	NEG	POS	POS	POS	POS	NEG	NEG	POS	NEG	POS	NEG	POS	AMB	NEG	NEG	NEG	NEG	NEG	NEG	POS	NEG	POS
**2002**																																						
02V10698	CC5/ST228-MRSA-I, South German EMRSA	NEG	NEG	NEG	POS	NEG	NEG	NEG	NEG	NEG	NEG	POS	NEG	POS	NEG	NEG	NEG	POS	POS	POS	POS	NEG	NEG	POS	NEG	POS	POS	POS	POS	NEG	NEG	NEG	NEG	POS	POS	POS	POS	NEG
02V13140	CC5/ST228-MRSA-I, South German EMRSA	NEG	NEG	NEG	NEG	NEG	NEG	NEG	NEG	NEG	NEG	POS	NEG	POS	NEG	NEG	NEG	POS	POS	POS	POS	NEG	NEG	POS	NEG	POS	POS	AMB	POS	NEG	NEG	NEG	NEG	POS	POS	POS	POS	NEG
02V20759	CC5/ST228-MRSA-I, South German EMRSA	NEG	NEG	NEG	POS	NEG	NEG	NEG	NEG	NEG	NEG	POS	NEG	POS	NEG	NEG	NEG	POS	POS	POS	POS	NEG	NEG	POS	NEG	POS	POS	NEG	POS	NEG	NEG	NEG	NEG	POS	AMB	AMB	POS	NEG
02B06271	CC5-MRSA-II, Rhine-Hesse EMRSA/UK-EMRSA-3	NEG	NEG	NEG	NEG	NEG	POS	NEG	NEG	POS	NEG	POS	NEG	POS	POS	NEG	NEG	POS	POS	POS	POS	NEG	POS	POS	NEG	POS	POS	AMB	POS	NEG	NEG	NEG	NEG	POS	POS	POS	POS	NEG
02V04116	CC5-MRSA-II, Rhine-Hesse EMRSA/UK-EMRSA-3	NEG	NEG	NEG	NEG	NEG	POS	NEG	NEG	POS	NEG	POS	NEG	POS	POS	NEG	NEG	POS	POS	POS	POS	NEG	POS	POS	NEG	POS	POS	POS	POS	NEG	NEG	NEG	NEG	POS	POS	POS	POS	NEG
02V29922	CC5-MRSA-II, Rhine-Hesse EMRSA/UK-EMRSA-3		NEG	NEG	NEG	NEG	POS	NEG	NEG	POS	NEG	POS	NEG	POS	POS		NEG	POS		POS	POS		POS			POS	POS		POS	NEG	NEG	NEG	NEG	POS	POS	POS	POS	NEG
02V33634	CC5-MRSA-II, Rhine-Hesse EMRSA/UK-EMRSA-3	NEG	NEG	NEG	NEG	NEG	POS	NEG	NEG	POS	NEG	POS	NEG	POS	POS	NEG	NEG	POS	POS	POS	POS	NEG	POS	POS	NEG	POS	POS	AMB	POS	NEG	NEG	NEG	NEG	POS	POS	POS	POS	NEG
02V34748	CC5-MRSA-II, Rhine-Hesse EMRSA/UK-EMRSA-3	NEG	NEG	NEG	NEG	NEG	POS	NEG	NEG	POS	NEG	POS	NEG	POS	POS	NEG	NEG	POS	POS	POS	POS	NEG	POS	POS	NEG	POS	POS	POS	POS	NEG	NEG	NEG	NEG	POS	POS	POS	POS	NEG
02V17002	CC8/ST254-MRSA-(atypical SCCmec), Hannover EMRSA	NEG	NEG	NEG	NEG	NEG	NEG	POS	NEG	NEG	NEG	NEG	NEG	NEG	NEG	POS	NEG	NEG	NEG	NEG	NEG	POS	NEG	NEG	NEG	POS	POS	NEG	POS	NEG	NEG	NEG	NEG	POS	POS	POS	POS	NEG
02V33579	CC8-MRSA-IV, USA500	AMB	NEG	NEG	POS	NEG	NEG	POS	NEG	NEG	NEG	NEG	NEG	NEG	NEG	NEG	NEG	NEG	NEG	NEG	NEG	NEG	NEG	NEG	NEG	POS	POS	POS	POS	NEG	NEG	NEG	NEG	POS	POS	AMB	POS	NEG
02V15498	CC22-MRSA-IV [PVL+]	NEG	NEG	NEG	NEG	NEG	NEG	NEG	NEG	NEG	NEG	POS	NEG	POS	NEG	NEG	NEG	POS	POS	POS	POS	NEG	NEG	POS	NEG	AMB	NEG	POS	AMB	POS	POS	NEG	NEG	NEG	NEG	POS	POS	NEG
02C05319	CC22-MRSA-IV, Barnim/UK-EMRSA-15	NEG	NEG	NEG	NEG	NEG	NEG	NEG	NEG	NEG	NEG	POS	NEG	POS	NEG	NEG	NEG	POS	POS	POS	POS	NEG	NEG	POS	NEG	POS	AMB	POS	POS	NEG	NEG	NEG	NEG	NEG	NEG	POS	POS	NEG
02U10255	CC22-MRSA-IV, Barnim/UK-EMRSA-15	NEG	NEG	NEG	NEG	NEG	NEG	NEG	NEG	NEG	NEG	POS	NEG	POS	NEG	NEG	NEG	POS	POS	POS	POS	NEG	NEG	POS	NEG	AMB	AMB	POS	POS	NEG	NEG	NEG	NEG	NEG	NEG	POS	POS	NEG
02V12175	CC22-MRSA-IV, Barnim/UK-EMRSA-15	NEG	NEG	NEG	NEG	NEG	NEG	NEG	NEG	NEG	NEG	POS	NEG	POS	NEG	NEG	NEG	POS	POS	POS	POS	NEG	NEG	POS	NEG	POS	AMB	POS	POS	NEG	NEG	NEG	NEG	NEG	NEG	POS	POS	NEG
02V13054	CC22-MRSA-IV, Barnim/UK-EMRSA-15	NEG	NEG	NEG	NEG	NEG	NEG	NEG	NEG	NEG	NEG	POS	NEG	POS	NEG	NEG	NEG	POS	POS	POS	POS	NEG	NEG	POS	NEG	POS	AMB	POS	POS	NEG	NEG	NEG	NEG	NEG	NEG	POS	POS	NEG
02V13950	CC22-MRSA-IV, Barnim/UK-EMRSA-15	NEG	NEG	NEG	NEG	NEG	NEG	NEG	NEG	NEG	NEG	POS	NEG	POS	NEG	NEG	NEG	POS	POS	POS	POS	NEG	NEG	POS	NEG	POS	AMB	POS	POS	NEG	NEG	NEG	NEG	NEG	NEG	POS	POS	NEG
02V21259	CC22-MRSA-IV, Barnim/UK-EMRSA-15	NEG	NEG	NEG	NEG	NEG	NEG	NEG	POS	NEG	NEG	POS	NEG	POS	NEG	NEG	POS	POS	POS	POS	POS	NEG	NEG	POS	NEG	POS	AMB	POS	POS	NEG	NEG	NEG	NEG	NEG	NEG	POS	POS	NEG
02V22657	CC22-MRSA-IV, Barnim/UK-EMRSA-15	NEG	NEG	NEG	NEG	NEG	NEG	NEG	NEG	NEG	NEG	POS	NEG	POS	NEG	NEG	NEG	POS	POS	POS	POS	NEG	NEG	POS	NEG	NEG	NEG	POS	POS	NEG	NEG	NEG	NEG	NEG	NEG	POS	POS	NEG
02V29051	CC22-MRSA-IV, Barnim/UK-EMRSA-15	NEG	NEG	NEG	NEG	NEG	NEG	NEG	NEG	NEG	NEG	POS	NEG	POS	NEG	NEG	NEG	POS	POS	POS	POS	NEG	NEG	POS	NEG	POS	AMB	POS	POS	NEG	NEG	NEG	NEG	NEG	NEG	POS	POS	NEG
02V30792	CC30/ST36-MRSA-II, UK-EMRSA-16	POS	POS	NEG	POS	NEG	NEG	NEG	NEG	NEG	NEG	POS	NEG	POS	NEG	NEG	NEG	POS	POS	POS	POS	NEG	NEG	POS	NEG	POS	POS	AMB	POS	NEG	NEG	NEG	NEG	NEG	NEG	POS	POS	NEG
02U07096	CC45-MRSA-IV, Berlin EMRSA	NEG	NEG	NEG	NEG	NEG	NEG	NEG	NEG	NEG	NEG	NEG	NEG	POS	NEG	NEG	NEG	POS	POS	POS	POS	NEG	NEG	POS	NEG	POS	AMB	POS	POS	NEG	NEG	NEG	NEG	NEG	NEG	POS	NEG	POS
02V00570	CC45-MRSA-IV, Berlin EMRSA	NEG	NEG	NEG	NEG	NEG	NEG	NEG	NEG	NEG	NEG	POS	NEG	POS	NEG	NEG	NEG	POS	POS	POS	POS	NEG	NEG	POS	NEG	POS	NEG	POS	POS	NEG	NEG	NEG	NEG	NEG	NEG	POS	NEG	POS
02V01730	CC45-MRSA-IV, Berlin EMRSA	NEG	NEG	NEG	NEG	NEG	NEG	NEG	NEG	NEG	NEG	POS	NEG	POS	NEG	NEG	NEG	POS	POS	POS	POS	NEG	NEG	POS	NEG	POS	NEG	POS	POS	NEG	NEG	NEG	NEG	NEG	NEG	POS	NEG	POS
02V01870	CC45-MRSA-IV, Berlin EMRSA	NEG	NEG	NEG	NEG	NEG	NEG	NEG	NEG	NEG	NEG	NEG	NEG	POS	NEG	NEG	NEG	POS	POS	POS	POS	NEG	NEG	POS	NEG	POS	AMB	POS	POS	NEG	NEG	NEG	NEG	NEG	NEG	AMB	NEG	POS
02V04750	CC45-MRSA-IV, Berlin EMRSA	NEG	NEG	NEG	NEG	NEG	NEG	NEG	NEG	NEG	NEG	NEG	NEG	POS	NEG	NEG	NEG	POS	POS	POS	POS	NEG	NEG	POS	NEG	POS	POS	POS	POS	NEG	NEG	NEG	NEG	NEG	NEG	POS	NEG	POS
02V06592	CC45-MRSA-IV, Berlin EMRSA	NEG	NEG	NEG	NEG	NEG	NEG	NEG	NEG	NEG	NEG	NEG	NEG	POS	NEG	NEG	NEG	POS	POS	POS	POS	NEG	NEG	POS	NEG	POS	POS	POS	POS	NEG	NEG	NEG	NEG	NEG	NEG	POS	NEG	POS
02V10868	CC45-MRSA-IV, Berlin EMRSA	NEG	NEG	NEG	NEG	NEG	NEG	NEG	NEG	NEG	NEG	NEG	NEG	POS	NEG	NEG	NEG	POS	POS	POS	POS	NEG	NEG	POS	NEG	POS	AMB	POS	POS	NEG	NEG	NEG	NEG	NEG	NEG	POS	NEG	POS
02V12394	CC45-MRSA-IV, Berlin EMRSA	NEG	NEG	NEG	NEG	NEG	NEG	NEG	NEG	NEG	NEG	NEG	NEG	POS	NEG	NEG	NEG	POS	POS	POS	POS	NEG	NEG	POS	NEG	POS	AMB	POS	POS	NEG	NEG	NEG	NEG	NEG	NEG	POS	NEG	POS
02V12942	CC45-MRSA-IV, Berlin EMRSA	NEG	NEG	NEG	NEG	NEG	NEG	NEG	NEG	NEG	NEG	POS	NEG	POS	NEG	NEG	NEG	POS	POS	POS	POS	NEG	NEG	POS	NEG	POS	AMB	POS	POS	NEG	NEG	NEG	NEG	NEG	NEG	POS	NEG	POS
02V13026	CC45-MRSA-IV, Berlin EMRSA	NEG	NEG	NEG	NEG	NEG	NEG	NEG	NEG	NEG	NEG	NEG	NEG	POS	NEG	NEG	NEG	POS	POS	POS	POS	NEG	NEG	POS	NEG	POS	AMB	POS	POS	NEG	NEG	NEG	NEG	NEG	NEG	POS	NEG	POS
02V14700	CC45-MRSA-IV, Berlin EMRSA		NEG	NEG	NEG	NEG	NEG	NEG	NEG	NEG	NEG	POS	NEG	POS	NEG		NEG	POS		POS	POS		NEG			POS	NEG		POS	NEG	NEG	NEG	NEG	NEG	NEG	POS	NEG	POS
02V15358	CC45-MRSA-IV, Berlin EMRSA	NEG	NEG	NEG	NEG	NEG	NEG	NEG	NEG	NEG	NEG	POS	NEG	POS	NEG	NEG	NEG	POS	POS	POS	POS	NEG	NEG	POS	NEG	POS	AMB	POS	POS	NEG	NEG	NEG	NEG	NEG	NEG	POS	NEG	POS
02V16654	CC45-MRSA-IV, Berlin EMRSA	NEG	NEG	NEG	NEG	NEG	NEG	NEG	NEG	NEG	NEG	NEG	NEG	POS	NEG	NEG	NEG	POS	POS	POS	POS	NEG	NEG	POS	NEG	POS	AMB	POS	POS	NEG	NEG	NEG	NEG	NEG	NEG	POS	NEG	POS
02V17120	CC45-MRSA-IV, Berlin EMRSA	NEG	NEG	NEG	NEG	NEG	NEG	NEG	NEG	NEG	NEG	NEG	NEG	POS	NEG	NEG	NEG	POS	POS	POS	POS	NEG	NEG	POS	NEG	POS	POS	POS	POS	NEG	NEG	NEG	NEG	NEG	NEG	POS	NEG	POS
02V17136	CC45-MRSA-IV, Berlin EMRSA	NEG	NEG	NEG	NEG	NEG	NEG	NEG	NEG	NEG	NEG	NEG	NEG	POS	NEG	NEG	NEG	POS	POS	POS	POS	NEG	NEG	POS	NEG	POS	AMB	POS	POS	NEG	NEG	NEG	NEG	NEG	NEG	POS	NEG	POS
02V17558	CC45-MRSA-IV, Berlin EMRSA	NEG	NEG	NEG	NEG	NEG	NEG	NEG	NEG	NEG	NEG	NEG	NEG	POS	NEG	NEG	NEG	POS	POS	POS	POS	NEG	NEG	POS	NEG	POS	AMB	POS	POS	NEG	NEG	NEG	NEG	NEG	NEG	POS	NEG	POS
02V17665	CC45-MRSA-IV, Berlin EMRSA	NEG	NEG	NEG	NEG	NEG	NEG	NEG	NEG	NEG	NEG	NEG	NEG	POS	NEG	NEG	NEG	POS	POS	POS	POS	NEG	NEG	POS	NEG	POS	NEG	POS	POS	NEG	NEG	NEG	NEG	NEG	NEG	POS	NEG	POS
02V20390	CC45-MRSA-IV, Berlin EMRSA	NEG	NEG	NEG	NEG	NEG	NEG	NEG	POS	NEG	NEG	POS	NEG	POS	NEG	NEG	POS	POS	POS	POS	AMB	NEG	NEG	POS	NEG	POS	NEG	POS	POS	NEG	NEG	NEG	NEG	NEG	NEG	POS	NEG	POS
02V20626	CC45-MRSA-IV, Berlin EMRSA	NEG	NEG	NEG	NEG	NEG	NEG	NEG	NEG	NEG	NEG	POS	NEG	POS	NEG	NEG	NEG	POS	POS	POS	POS	NEG	NEG	POS	NEG	POS	AMB	POS	POS	NEG	NEG	NEG	NEG	NEG	NEG	POS	NEG	POS
02V20680	CC45-MRSA-IV, Berlin EMRSA	NEG	NEG	NEG	NEG	NEG	NEG	NEG	NEG	NEG	NEG	POS	NEG	POS	NEG	NEG	NEG	POS	POS	POS	POS	NEG	NEG	POS	NEG	POS	POS	POS	POS	NEG	NEG	NEG	NEG	NEG	NEG	AMB	NEG	POS
02V21980	CC45-MRSA-IV, Berlin EMRSA	NEG	NEG	NEG	NEG	NEG	NEG	NEG	NEG	NEG	NEG	NEG	NEG	POS	NEG	NEG	NEG	POS	POS	POS	POS	NEG	NEG	POS	NEG	POS	NEG	POS	POS	NEG	NEG	NEG	NEG	NEG	NEG	AMB	NEG	POS
02V22570	CC45-MRSA-IV, Berlin EMRSA	NEG	NEG	NEG	NEG	NEG	NEG	NEG	NEG	NEG	NEG	POS	NEG	POS	NEG	NEG	NEG	POS	POS	POS	POS	NEG	NEG	POS	NEG	POS	AMB	POS	POS	NEG	NEG	NEG	NEG	NEG	NEG	POS	NEG	POS
02V22982	CC45-MRSA-IV, Berlin EMRSA	NEG	NEG	NEG	NEG	NEG	NEG	NEG	NEG	NEG	NEG	POS	NEG	POS	NEG	NEG	NEG	POS	POS	POS	POS	NEG	NEG	POS	NEG	POS	POS	POS	POS	NEG	NEG	NEG	NEG	NEG	NEG	POS	NEG	POS
02V25503	CC45-MRSA-IV, Berlin EMRSA	NEG	NEG	NEG	NEG	NEG	NEG	NEG	NEG	NEG	NEG	POS	NEG	POS	NEG	NEG	NEG	POS	POS	POS	POS	NEG	NEG	POS	NEG	POS	AMB	POS	POS	NEG	NEG	NEG	NEG	NEG	NEG	POS	NEG	POS
02V28454	CC45-MRSA-IV, Berlin EMRSA	NEG	NEG	NEG	NEG	NEG	NEG	NEG	NEG	NEG	NEG	NEG	NEG	POS	NEG	NEG	NEG	POS	POS	POS	POS	NEG	NEG	POS	NEG	POS	AMB	POS	POS	NEG	NEG	NEG	NEG	NEG	NEG	POS	NEG	POS
02V29373	CC45-MRSA-IV, Berlin EMRSA	NEG	NEG	NEG	NEG	NEG	NEG	NEG	NEG	NEG	NEG	NEG	NEG	POS	NEG	NEG	NEG	POS	POS	POS	POS	NEG	NEG	POS	NEG	POS	AMB	POS	POS	NEG	NEG	NEG	NEG	NEG	NEG	POS	NEG	POS
02V30321	CC45-MRSA-IV, Berlin EMRSA	NEG	NEG	NEG	NEG	NEG	NEG	NEG	NEG	NEG	NEG	POS	NEG	POS	NEG	NEG	NEG	POS	POS	POS	POS	NEG	NEG	POS	NEG	POS	AMB	POS	POS	NEG	NEG	NEG	NEG	NEG	NEG	POS	NEG	POS
02V30341	CC45-MRSA-IV, Berlin EMRSA	NEG	NEG	NEG	NEG	NEG	NEG	NEG	NEG	NEG	NEG	POS	NEG	POS	NEG	NEG	NEG	POS	POS	POS	POS	NEG	NEG	POS	NEG	POS	AMB	POS	POS	NEG	NEG	NEG	NEG	NEG	NEG	POS	NEG	POS
02V31377	CC45-MRSA-IV, Berlin EMRSA	NEG	NEG	NEG	NEG	NEG	NEG	NEG	NEG	NEG	NEG	POS	NEG	POS	NEG	NEG	NEG	POS	POS	POS	POS	NEG	NEG	POS	NEG	POS	AMB	POS	POS	NEG	NEG	NEG	NEG	NEG	NEG	POS	NEG	POS
02V31941	CC45-MRSA-IV, Berlin EMRSA	NEG	NEG	NEG	NEG	NEG	NEG	NEG	NEG	NEG	NEG	NEG	NEG	POS	NEG	NEG	NEG	POS	POS	POS	POS	NEG	NEG	POS	NEG	POS	AMB	POS	POS	NEG	NEG	NEG	NEG	NEG	NEG	POS	NEG	POS
02V34679	CC45-MRSA-IV, Berlin EMRSA	NEG	NEG	NEG	NEG	NEG	NEG	NEG	NEG	NEG	NEG	POS	NEG	POS	NEG	NEG	NEG	POS	POS	POS	POS	NEG	NEG	POS	NEG	POS	NEG	POS	POS	NEG	NEG	NEG	NEG	NEG	NEG	AMB	NEG	POS
02V35779	CC45-MRSA-IV, Berlin EMRSA	NEG	NEG	NEG	NEG	NEG	NEG	NEG	NEG	NEG	NEG	POS	NEG	POS	NEG	NEG	NEG	POS	POS	POS	POS	NEG	NEG	POS	NEG	POS	POS	POS	POS	NEG	NEG	NEG	NEG	NEG	NEG	POS	NEG	POS
02V36419	CC45-MRSA-IV, Berlin EMRSA		NEG	NEG	NEG	NEG	NEG	NEG	NEG	NEG	NEG	POS	NEG	POS	NEG		NEG	POS		POS	POS		NEG			POS	NEG		POS	NEG	NEG	NEG	NEG	NEG	NEG	POS	NEG	POS
02V36469	CC45-MRSA-IV, Berlin EMRSA	NEG	AMB	NEG	NEG	NEG	NEG	NEG	NEG	NEG	NEG	NEG	NEG	POS	NEG	NEG	NEG	POS	POS	POS	POS	NEG	NEG	POS	NEG	POS	POS	POS	POS	NEG	NEG	NEG	NEG	NEG	NEG	POS	NEG	POS

ISOLATE:	STRAIN	VIRULENCE : HAEMOLYSINS								VIRULENCE : HLB-CONV PHAGES			VIRULENCE : EXFOL.TOXINS			VIRULENCE : EPITHEL. DIFF. INHIB			VIRULENCE : ACME LOCUS
		putative membrane protein	haemolysin alpha	putative membrane protein		haemolysin beta				staphylo-kinase	chemotaxis-inhibiting protein (CHIPS)	Staphyl. Comple-ment inhibitor	exfoliative toxin serotype A	exfoliative toxin serotype B	exfoliative toxin D	epidermal cell differen-tiation inhibitor	epidermal cell differen-tiation inhibitor B	epidermal cell differen-tiation inhibitor C	ACME-locus
		hl	hla	hlIII (consensus)	hlIII (other than RF122)	hlb_probe 1	hlb_probe 2	hlb_probe 3	un-disrupted hlb	sak	chp	scn	etA	etB	etD	edinA	edinB	edinC	arcA-SCC
**2000**																			
00V07201	CC5/ST228-MRSA, South German EMRSA (truncated SCCmec element)	POS	POS	POS	POS	POS	POS	POS	NEG	POS	POS	POS	NEG	NEG	NEG	NEG	NEG	NEG	NEG
00V07634	CC5/ST228-MRSA, South German EMRSA (truncated SCCmec element)	POS	POS	POS	POS	POS	POS	POS	NEG	POS	POS	POS	NEG	NEG	NEG	NEG	NEG	NEG	NEG
00V08701	CC5/ST228-MRSA, South German EMRSA (truncated SCCmec element)	POS	POS	POS	POS	NEG	POS	NEG	POS	NEG	NEG	NEG	NEG	NEG	NEG	NEG	NEG	NEG	NEG
00V09056	CC5/ST228-MRSA, South German EMRSA (truncated SCCmec element)	POS	POS	POS	POS	POS	POS	POS	NEG	POS	POS	POS	NEG	NEG	NEG	NEG	NEG	NEG	NEG
00V10190	CC5/ST228-MRSA, South German EMRSA (truncated SCCmec element)	POS	POS	POS	POS	POS	POS	POS	AMB	POS	POS	POS	NEG	NEG	NEG	NEG	NEG	NEG	NEG
00V10643	CC5/ST228-MRSA, South German EMRSA (truncated SCCmec element)	POS	POS	POS	POS	POS	POS	POS	NEG	POS	POS	POS	NEG	NEG	NEG	NEG	NEG	NEG	NEG
00V11732	CC5/ST228-MRSA, South German EMRSA (truncated SCCmec element)	POS	POS	POS	POS	POS	POS	AMB	NEG	POS	POS	POS	NEG	NEG	NEG	NEG	NEG	NEG	NEG
00V12300	CC5/ST228-MRSA, South German EMRSA (truncated SCCmec element)	POS	POS	POS	POS	AMB	POS	AMB	NEG	POS	POS	POS	NEG	NEG	NEG	NEG	NEG	NEG	NEG
00V14652	CC5/ST228-MRSA, South German EMRSA (truncated SCCmec element)	POS	POS	POS	POS	POS	POS	AMB	NEG	POS	POS	POS	NEG	NEG	NEG	NEG	NEG	NEG	NEG
00V24941	CC5/ST228-MRSA, South German EMRSA (truncated SCCmec element)	POS	POS	POS	POS	POS	POS	AMB	NEG	POS	POS	POS	NEG	NEG	NEG	NEG	NEG	NEG	NEG
00V29834	CC5/ST228-MRSA, South German EMRSA (truncated SCCmec element)	POS	POS	POS	POS	AMB	POS	AMB	NEG	POS	POS	POS	NEG	NEG	NEG	NEG	NEG	NEG	NEG
00V29909	CC5/ST228-MRSA, South German EMRSA (truncated SCCmec element)	POS	POS	POS	POS	AMB	POS	AMB	NEG	POS	POS	POS	NEG	NEG	NEG	NEG	NEG	NEG	NEG
00V30456	CC5/ST228-MRSA, South German EMRSA (truncated SCCmec element)	POS	POS	POS	POS	AMB	POS	NEG	NEG	POS	POS	POS	NEG	NEG	NEG	NEG	NEG	NEG	NEG
00V30827	CC5/ST228-MRSA, South German EMRSA (truncated SCCmec element)	POS	POS	POS	POS	POS	POS	POS	NEG	POS	POS	POS	NEG	NEG	NEG	NEG	NEG	NEG	NEG
00V33204	CC5/ST228-MRSA, South German EMRSA (truncated SCCmec element)	POS	POS	POS	POS	POS	POS	AMB	NEG	POS	POS	POS	NEG	NEG	NEG	NEG	NEG	NEG	NEG
00V36490	CC5/ST228-MRSA, South German EMRSA (truncated SCCmec element)	POS	POS	POS	POS	POS	POS	POS	AMB	POS	POS	POS	NEG	NEG	NEG	NEG	NEG	NEG	NEG
00V37079	CC5/ST228-MRSA, South German EMRSA (truncated SCCmec element)	POS	POS	POS	POS	AMB	POS	AMB	POS	NEG	NEG	NEG	NEG	NEG	NEG	NEG	NEG	NEG	NEG
00V40116	CC5/ST228-MRSA, South German EMRSA (truncated SCCmec element)	POS	POS	POS	POS	POS	POS	POS	AMB	POS	POS	POS	NEG	NEG	NEG	NEG	NEG	NEG	NEG
00V41429	CC5/ST228-MRSA, South German EMRSA (truncated SCCmec element)	POS	POS	POS	POS	POS	POS	POS	POS	POS	POS	POS	NEG	NEG	NEG	NEG	NEG	NEG	NEG
00V42118	CC5/ST228-MRSA, South German EMRSA (truncated SCCmec element)	POS	POS	POS	POS	POS	POS	POS	NEG	POS	POS	POS	NEG	NEG	NEG	NEG	NEG	NEG	NEG
00V44135	CC5/ST228-MRSA, South German EMRSA (truncated SCCmec element)	POS	POS	POS	POS	POS	POS	POS	AMB	POS	POS	POS	NEG	NEG	NEG	NEG	NEG	NEG	NEG
00V44538	CC5/ST228-MRSA, South German EMRSA (truncated SCCmec element)	POS	POS	POS	POS	POS	POS	AMB	NEG	POS	POS	POS	NEG	NEG	NEG	NEG	NEG	NEG	NEG
00V44990	CC5/ST228-MRSA, South German EMRSA (truncated SCCmec element)	POS	POS	POS	POS	POS	POS	AMB	NEG	POS	POS	POS	NEG	NEG	NEG	NEG	NEG	NEG	NEG
00V46910	CC5/ST228-MRSA, South German EMRSA (truncated SCCmec element)	POS	POS	POS	POS	POS	POS	POS	NEG	POS	POS	POS	NEG	NEG	NEG	NEG	NEG	NEG	NEG
00V07063	CC5/ST228-MRSA-I, South German EMRSA	POS	POS	POS	POS	POS	POS	POS	AMB	POS	POS	POS	NEG	NEG	NEG	NEG	NEG	NEG	NEG
00V46921	CC5-MRSA-II, Rhine-Hesse EMRSA/UK-EMRSA-3	POS	POS	POS	POS	POS	POS	POS	NEG	POS	POS	POS	NEG	NEG	NEG	NEG	NEG	NEG	NEG
00V08510	CC8/ST254-MRSA-(atypical SCCmec), Hannover EMRSA	POS	POS	POS	POS	POS	POS	POS	NEG	POS	NEG	POS	NEG	NEG	NEG	NEG	NEG	NEG	NEG
00V25538	CC8/ST254-MRSA-(atypical SCCmec), Hannover EMRSA	POS	POS	POS	POS	POS	POS	POS	NEG	POS	NEG	POS	NEG	NEG	NEG	NEG	NEG	NEG	NEG
00V38664	CC8/ST254-MRSA-(atypical SCCmec), Hannover EMRSA	POS	POS	POS	POS	POS	POS	POS	NEG	POS	NEG	POS	NEG	NEG	NEG	NEG	NEG	NEG	NEG
00V46488	CC8/ST254-MRSA-(atypical SCCmec), Hannover EMRSA	POS	POS	POS	POS	POS	POS	POS	NEG	POS	NEG	POS	NEG	NEG	NEG	NEG	NEG	NEG	NEG
00V44035	CC8/ST254-MRSA-IV/V, UK-EMRSA-10/Hannover EMRSA	POS	POS	POS	POS	POS	POS	POS	NEG	POS	NEG	POS	NEG	NEG	NEG	NEG	NEG	NEG	NEG
00V13324	CC22-MRSA-IV [PVL+]	POS	NEG	NEG	NEG	POS	POS	AMB	NEG	POS	POS	POS	NEG	NEG	NEG	NEG	NEG	NEG	NEG
00B02046	CC45-MRSA-IV, Berlin EMRSA	POS	POS	POS	NEG	POS	POS	NEG	NEG	POS	POS	POS	NEG	NEG	NEG	NEG	NEG	NEG	NEG
00V11299	CC45-MRSA-IV, Berlin EMRSA	POS	POS	POS	NEG	POS	POS	NEG	NEG	POS	POS	POS	NEG	NEG	NEG	NEG	NEG	NEG	NEG
00V14125	CC45-MRSA-IV, Berlin EMRSA	POS	POS	POS	NEG	AMB	AMB	NEG	NEG	POS	POS	POS	NEG	NEG	NEG	NEG	NEG	NEG	NEG
00V16040	CC45-MRSA-IV, Berlin EMRSA	POS	POS	POS	NEG	NEG	AMB	NEG	NEG	POS	POS	POS	NEG	NEG	NEG	NEG	NEG	NEG	NEG
00V25025	CC45-MRSA-IV, Berlin EMRSA	POS	POS	POS	NEG	AMB	AMB	NEG	NEG	POS	POS	POS	NEG	NEG	NEG	NEG	NEG	NEG	NEG
00V29618	CC45-MRSA-IV, Berlin EMRSA	POS	POS	POS	NEG	AMB	AMB	NEG	NEG	POS	POS	POS	NEG	NEG	NEG	NEG	NEG	NEG	NEG
00V30989	CC45-MRSA-IV, Berlin EMRSA	POS	POS	POS	NEG	POS	POS	NEG	NEG	POS	POS	POS	NEG	NEG	NEG	NEG	NEG	NEG	NEG
00V31007	CC45-MRSA-IV, Berlin EMRSA	POS	POS	POS	NEG	AMB	AMB	NEG	NEG	POS	POS	POS	NEG	NEG	NEG	NEG	NEG	NEG	NEG
00V31700	CC45-MRSA-IV, Berlin EMRSA	POS	POS	POS	NEG	AMB	POS	NEG	NEG	POS	POS	POS	NEG	NEG	NEG	NEG	NEG	NEG	NEG
00V32193	CC45-MRSA-IV, Berlin EMRSA	POS	POS	POS	NEG	POS	POS	NEG	NEG	POS	POS	POS	NEG	NEG	NEG	NEG	NEG	NEG	NEG
00V43085	CC45-MRSA-IV, Berlin EMRSA	POS	POS	POS	NEG	POS	POS	NEG	NEG	POS	POS	POS	NEG	NEG	NEG	NEG	NEG	NEG	NEG
00V44382	CC45-MRSA-IV, Berlin EMRSA	AMB	NEG	POS	NEG	NEG	NEG	NEG	NEG	POS	POS	POS	NEG	NEG	NEG	NEG	NEG	NEG	NEG
00V46648	CC45-MRSA-IV, Berlin EMRSA	POS	POS	POS	NEG	POS	POS	NEG	NEG	POS	POS	POS	NEG	NEG	NEG	NEG	NEG	NEG	NEG
00V46843	CC45-MRSA-IV, Berlin EMRSA	POS	POS	POS	NEG	POS	POS	NEG	NEG	POS	POS	POS	NEG	NEG	NEG	NEG	NEG	NEG	NEG
00V47014	CC45-MRSA-IV, Berlin EMRSA	POS	POS	POS	NEG	POS	POS	NEG	NEG	POS	POS	POS	NEG	NEG	NEG	NEG	NEG	NEG	NEG
00V47053	CC45-MRSA-IV, Berlin EMRSA	POS	POS	POS	NEG	NEG	NEG	NEG	NEG	POS	POS	POS	NEG	NEG	NEG	NEG	NEG	NEG	NEG
00V47227	CC45-MRSA-IV, Berlin EMRSA	POS	POS	POS	NEG	POS	POS	NEG	NEG	POS	POS	POS	NEG	NEG	NEG	NEG	NEG	NEG	NEG
00V47429	CC45-MRSA-IV, Berlin EMRSA	POS	POS	POS	NEG	AMB	POS	NEG	NEG	POS	POS	POS	NEG	NEG	NEG	NEG	NEG	NEG	NEG
00V47531	CC45-MRSA-IV, Berlin EMRSA	POS	POS	POS	NEG	POS	POS	NEG	NEG	POS	POS	POS	NEG	NEG	NEG	NEG	NEG	NEG	NEG
**2001**																			
01V27106	CC1-MRSA-IV, WA MRSA-1/57	POS	POS	POS	POS	POS	POS	POS	NEG	POS	NEG	POS	NEG	NEG	NEG	NEG	NEG	NEG	NEG
01V41025	CC5/ST228-MRSA-I, South German EMRSA	POS	POS	POS	POS	POS	POS	POS	AMB	POS	AMB	POS	NEG	NEG	NEG	NEG	NEG	NEG	NEG
01V12125	CC5-MRSA-II, Rhine-Hesse EMRSA/UK-EMRSA-3	POS	POS	POS	POS	POS	POS	AMB	NEG	POS	POS	POS	NEG	NEG	NEG	NEG	NEG	NEG	NEG
01V29747	CC5-MRSA-II, Rhine-Hesse EMRSA/UK-EMRSA-3	POS	POS	POS	POS	POS	POS	POS	NEG	POS	POS	POS	NEG	NEG	NEG	NEG	NEG	NEG	NEG
01V32464	CC5-MRSA-II, Rhine-Hesse EMRSA/UK-EMRSA-3	POS	POS	POS	POS	AMB	POS	POS	NEG	POS	POS	POS	NEG	NEG	NEG	NEG	NEG	NEG	NEG
01V39703	CC5-MRSA-II, Rhine-Hesse EMRSA/UK-EMRSA-3	POS	POS	POS	POS	POS	POS	AMB	NEG	POS	POS	POS	NEG	NEG	NEG	NEG	NEG	NEG	NEG
01V40228	CC5-MRSA-II, Rhine-Hesse EMRSA/UK-EMRSA-3	POS	POS		POS				NEG	POS			NEG	NEG	NEG	NEG	NEG	NEG	
01B05861	CC8/ST239-MRSA-III, Vienna/Hungarian/Brazilian Clone	POS	POS	POS	POS	POS	POS	POS	NEG	POS	NEG	POS	NEG	NEG	NEG	NEG	NEG	NEG	NEG
01V34572	CC8/ST239-MRSA-III, Vienna/Hungarian/Brazilian Clone	POS	POS	POS	POS	POS	POS	POS	NEG	POS	NEG	POS	NEG	NEG	NEG	NEG	NEG	NEG	NEG
01V36469	CC8/ST239-MRSA-III, Vienna/Hungarian/Brazilian Clone	POS	AMB	POS	POS	AMB	POS	NEG	NEG	POS	NEG	POS	NEG	NEG	NEG	NEG	NEG	NEG	NEG
01V39123	CC8/ST239-MRSA-III, Vienna/Hungarian/Brazilian Clone	POS	POS	POS	POS	POS	POS	POS	POS	POS	NEG	POS	NEG	NEG	NEG	NEG	NEG	NEG	NEG
01V39143	CC8/ST239-MRSA-III, Vienna/Hungarian/Brazilian Clone	POS	POS	POS	POS	POS	POS	POS	NEG	POS	NEG	POS	NEG	NEG	NEG	NEG	NEG	NEG	NEG
01V10962	CC8/ST254-MRSA-(atypical SCCmec), Hannover EMRSA	POS	POS	POS	POS	POS	POS	POS	NEG	POS	NEG	POS	NEG	NEG	NEG	NEG	NEG	NEG	NEG
01V10963	CC8/ST254-MRSA-IV/V, UK-EMRSA-10/Hannover EMRSA	POS	POS	POS	POS	POS	POS	POS	NEG	POS	NEG	POS	NEG	NEG	NEG	NEG	NEG	NEG	NEG
01V36287	CC8/ST254-MRSA-IV/V, UK-EMRSA-10/Hannover EMRSA	POS	POS	POS	POS	POS	POS	POS	NEG	POS	NEG	POS	NEG	NEG	NEG	NEG	NEG	NEG	NEG
01V25329	CC22-MRSA-IV [PVL+]	POS	POS	NEG	NEG	POS	POS	POS	NEG	POS	POS	POS	NEG	NEG	NEG	NEG	NEG	NEG	NEG
01V07770	CC22-MRSA-IV, Barnim/UK-EMRSA-15	POS	POS		NEG				NEG	POS			NEG	NEG	NEG	NEG	NEG	NEG	
01V20122	CC22-MRSA-IV, Barnim/UK-EMRSA-15	POS	NEG	NEG	NEG	POS	POS	NEG	NEG	POS	POS	POS	NEG	NEG	NEG	NEG	NEG	NEG	NEG
01V43277	CC22-MRSA-IV, Barnim/UK-EMRSA-15	POS	POS	NEG	NEG	POS	POS	POS	NEG	POS	POS	POS	NEG	NEG	NEG	NEG	NEG	NEG	NEG
01F02744	CC45-MRSA-IV, Berlin EMRSA	POS	POS	POS	NEG	AMB	AMB	NEG	NEG	POS	POS	POS	NEG	NEG	NEG	NEG	NEG	NEG	NEG
01V01127	CC45-MRSA-IV, Berlin EMRSA	POS	POS	POS	NEG	AMB	POS	NEG	NEG	POS	POS	POS	NEG	NEG	NEG	NEG	NEG	NEG	NEG
01V01445	CC45-MRSA-IV, Berlin EMRSA	POS	POS	POS	NEG	POS	POS	NEG	NEG	POS	POS	POS	NEG	NEG	NEG	NEG	NEG	NEG	NEG
01V02575	CC45-MRSA-IV, Berlin EMRSA	POS	AMB	POS	NEG	NEG	NEG	NEG	NEG	POS	POS	POS	NEG	NEG	NEG	NEG	NEG	NEG	NEG
01V02636	CC45-MRSA-IV, Berlin EMRSA	POS	POS	POS	NEG	AMB	POS	NEG	NEG	POS	POS	POS	NEG	NEG	NEG	NEG	NEG	NEG	NEG
01V03483	CC45-MRSA-IV, Berlin EMRSA	AMB	NEG	POS	NEG	NEG	NEG	NEG	NEG	POS	POS	POS	NEG	NEG	NEG	NEG	NEG	NEG	NEG
01V04728	CC45-MRSA-IV, Berlin EMRSA	POS	POS	POS	NEG	AMB	POS	NEG	NEG	POS	POS	POS	NEG	NEG	NEG	NEG	NEG	NEG	NEG
01V05972	CC45-MRSA-IV, Berlin EMRSA	POS	AMB	POS	NEG	NEG	NEG	NEG	NEG	POS	POS	POS	NEG	NEG	NEG	NEG	NEG	NEG	NEG
01V07139	CC45-MRSA-IV, Berlin EMRSA	POS	POS	POS	NEG	POS	POS	NEG	NEG	POS	POS	POS	NEG	NEG	NEG	NEG	NEG	NEG	NEG
01V08133	CC45-MRSA-IV, Berlin EMRSA	POS	POS	POS	NEG	AMB	POS	NEG	NEG	POS	POS	POS	NEG	NEG	NEG	NEG	NEG	NEG	NEG
01V09872	CC45-MRSA-IV, Berlin EMRSA	POS	POS	POS	NEG	AMB	POS	NEG	NEG	POS	POS	POS	NEG	NEG	NEG	NEG	NEG	NEG	NEG
01V12465	CC45-MRSA-IV, Berlin EMRSA	POS	POS	POS	NEG	NEG	AMB	NEG	NEG	POS	POS	POS	NEG	NEG	NEG	NEG	NEG	NEG	NEG
01V16151	CC45-MRSA-IV, Berlin EMRSA	POS	POS	POS	NEG	NEG	AMB	NEG	NEG	POS	POS	POS	NEG	NEG	NEG	NEG	NEG	NEG	NEG
01V16873	CC45-MRSA-IV, Berlin EMRSA	POS	POS	POS	NEG	POS	POS	NEG	NEG	POS	POS	POS	NEG	NEG	NEG	NEG	NEG	NEG	NEG
01V17796	CC45-MRSA-IV, Berlin EMRSA	POS	POS	POS	NEG	AMB	POS	NEG	NEG	POS	POS	POS	NEG	NEG	NEG	NEG	NEG	NEG	NEG
01V18681	CC45-MRSA-IV, Berlin EMRSA	POS	POS	POS	NEG	AMB	POS	NEG	NEG	POS	POS	POS	NEG	NEG	NEG	NEG	NEG	NEG	NEG
01V20066	CC45-MRSA-IV, Berlin EMRSA	POS	POS	POS	NEG	AMB	POS	NEG	NEG	POS	POS	POS	NEG	NEG	NEG	NEG	NEG	NEG	NEG
01V23050	CC45-MRSA-IV, Berlin EMRSA	POS	POS	POS	NEG	NEG	NEG	NEG	POS	POS	NEG	NEG	NEG	NEG	NEG	NEG	NEG	NEG	NEG
01V25584	CC45-MRSA-IV, Berlin EMRSA	NEG	NEG	POS	NEG	NEG	NEG	NEG	NEG	POS	POS	POS	NEG	NEG	NEG	NEG	NEG	NEG	NEG
01V31705	CC45-MRSA-IV, Berlin EMRSA	POS	POS	POS	NEG	POS	POS	NEG	NEG	POS	POS	POS	NEG	NEG	NEG	NEG	NEG	NEG	NEG
01V34968	CC45-MRSA-IV, Berlin EMRSA	POS	POS	POS	NEG	POS	POS	NEG	NEG	POS	POS	POS	NEG	NEG	NEG	NEG	NEG	NEG	NEG
01V36186	CC45-MRSA-IV, Berlin EMRSA	POS	POS	POS	NEG	NEG	NEG	NEG	NEG	POS	POS	POS	NEG	NEG	NEG	NEG	NEG	NEG	NEG
01V36534	CC45-MRSA-IV, Berlin EMRSA	POS	POS	POS	NEG	AMB	POS	NEG	NEG	POS	POS	POS	NEG	NEG	NEG	NEG	NEG	NEG	NEG
01V36588	CC45-MRSA-IV, Berlin EMRSA	POS	POS	POS	NEG	POS	POS	NEG	NEG	POS	POS	POS	NEG	NEG	NEG	NEG	NEG	NEG	NEG
01V37990	CC45-MRSA-IV, Berlin EMRSA	POS	POS	POS	NEG	POS	POS	NEG	NEG	POS	POS	POS	NEG	NEG	NEG	NEG	NEG	NEG	NEG
01V39610	CC45-MRSA-IV, Berlin EMRSA	POS	POS	POS	NEG	POS	POS	NEG	POS	POS	POS	POS	NEG	NEG	NEG	NEG	NEG	NEG	NEG
01V40229	CC45-MRSA-IV, Berlin EMRSA	POS	POS	POS	NEG	POS	POS	NEG	NEG	POS	POS	POS	NEG	NEG	NEG	NEG	NEG	NEG	NEG
01V40374	CC45-MRSA-IV, Berlin EMRSA	POS	POS		NEG				NEG	POS			NEG	NEG	NEG	NEG	NEG	NEG	
01V41056	CC45-MRSA-IV, Berlin EMRSA	POS	POS	POS	NEG	NEG	AMB	NEG	NEG	POS	POS	POS	NEG	NEG	NEG	NEG	NEG	NEG	NEG
01V42009	CC45-MRSA-IV, Berlin EMRSA	POS	POS	POS	NEG	AMB	POS	NEG	NEG	POS	POS	POS	NEG	NEG	NEG	NEG	NEG	NEG	NEG
01V43511	CC45-MRSA-IV, Berlin EMRSA	AMB	POS	POS	NEG	AMB	POS	NEG	NEG	POS	POS	POS	NEG	NEG	NEG	NEG	NEG	NEG	NEG
**2002**																			
02V10698	CC5/ST228-MRSA-I, South German EMRSA	POS	POS	POS	POS	POS	POS	AMB	NEG	POS	NEG	POS	NEG	NEG	NEG	NEG	NEG	NEG	NEG
02V13140	CC5/ST228-MRSA-I, South German EMRSA	POS	POS	POS	POS	POS	POS	POS	AMB	POS	POS	POS	NEG	NEG	NEG	NEG	NEG	NEG	NEG
02V20759	CC5/ST228-MRSA-I, South German EMRSA	POS	POS	POS	POS	AMB	POS	AMB	NEG	POS	NEG	POS	NEG	NEG	NEG	NEG	NEG	NEG	NEG
02B06271	CC5-MRSA-II, Rhine-Hesse EMRSA/UK-EMRSA-3	POS	POS	POS	POS	POS	POS	AMB	NEG	POS	POS	POS	NEG	NEG	NEG	NEG	NEG	NEG	NEG
02V04116	CC5-MRSA-II, Rhine-Hesse EMRSA/UK-EMRSA-3	POS	POS	POS	POS	POS	POS	AMB	NEG	POS	POS	POS	NEG	NEG	NEG	NEG	NEG	NEG	NEG
02V29922	CC5-MRSA-II, Rhine-Hesse EMRSA/UK-EMRSA-3	POS	NEG		POS				NEG	POS			NEG	NEG	NEG	NEG	NEG	NEG	
02V33634	CC5-MRSA-II, Rhine-Hesse EMRSA/UK-EMRSA-3	POS	POS	POS	POS	POS	POS	AMB	NEG	POS	POS	POS	NEG	NEG	NEG	NEG	NEG	NEG	NEG
02V34748	CC5-MRSA-II, Rhine-Hesse EMRSA/UK-EMRSA-3	POS	POS	POS	POS	POS	POS	POS	NEG	POS	POS	POS	NEG	NEG	NEG	NEG	NEG	NEG	NEG
02V17002	CC8/ST254-MRSA-(atypical SCCmec), Hannover EMRSA	POS	POS	POS	POS	POS	POS	AMB	POS	NEG	NEG	NEG	NEG	NEG	NEG	NEG	NEG	NEG	NEG
02V33579	CC8-MRSA-IV, USA500	POS	POS	POS	POS	POS	POS	POS	NEG	POS	NEG	POS	NEG	NEG	NEG	NEG	NEG	NEG	NEG
02V15498	CC22-MRSA-IV [PVL+]	POS	POS	NEG	NEG	POS	POS	POS	NEG	POS	POS	POS	NEG	NEG	NEG	NEG	NEG	NEG	NEG
02C05319	CC22-MRSA-IV, Barnim/UK-EMRSA-15	POS	POS	NEG	NEG	POS	POS	POS	NEG	POS	POS	POS	NEG	NEG	NEG	NEG	NEG	NEG	NEG
02U10255	CC22-MRSA-IV, Barnim/UK-EMRSA-15	POS	POS	NEG	NEG	POS	POS	POS	NEG	POS	POS	POS	NEG	NEG	NEG	NEG	NEG	NEG	NEG
02V12175	CC22-MRSA-IV, Barnim/UK-EMRSA-15	POS	POS	NEG	NEG	POS	POS	POS	NEG	POS	POS	POS	NEG	NEG	NEG	NEG	NEG	NEG	NEG
02V13054	CC22-MRSA-IV, Barnim/UK-EMRSA-15	POS	POS	NEG	NEG	POS	POS	POS	NEG	POS	POS	POS	NEG	NEG	NEG	NEG	NEG	NEG	NEG
02V13950	CC22-MRSA-IV, Barnim/UK-EMRSA-15	POS	POS	NEG	NEG	POS	POS	POS	NEG	POS	POS	POS	NEG	NEG	NEG	NEG	NEG	NEG	NEG
02V21259	CC22-MRSA-IV, Barnim/UK-EMRSA-15	POS	POS	NEG	NEG	POS	POS	POS	NEG	POS	POS	POS	NEG	NEG	NEG	NEG	NEG	NEG	NEG
02V22657	CC22-MRSA-IV, Barnim/UK-EMRSA-15	POS	NEG	NEG	NEG	POS	POS	AMB	NEG	POS	POS	POS	NEG	NEG	NEG	NEG	NEG	NEG	NEG
02V29051	CC22-MRSA-IV, Barnim/UK-EMRSA-15	POS	POS	NEG	NEG	POS	POS	POS	NEG	POS	POS	POS	NEG	NEG	NEG	NEG	NEG	NEG	NEG
02V30792	CC30/ST36-MRSA-II, UK-EMRSA-16	POS	POS	POS	POS	POS	POS	AMB	NEG	POS	POS	POS	NEG	NEG	NEG	NEG	NEG	NEG	NEG
02U07096	CC45-MRSA-IV, Berlin EMRSA	POS	POS	POS	NEG	AMB	POS	NEG	NEG	POS	POS	POS	NEG	NEG	NEG	NEG	NEG	NEG	NEG
02V00570	CC45-MRSA-IV, Berlin EMRSA	POS	POS	POS	NEG	NEG	POS	NEG	NEG	POS	POS	POS	NEG	NEG	NEG	NEG	NEG	NEG	NEG
02V01730	CC45-MRSA-IV, Berlin EMRSA	POS	POS	POS	NEG	NEG	NEG	NEG	NEG	POS	POS	POS	NEG	NEG	NEG	NEG	NEG	NEG	NEG
02V01870	CC45-MRSA-IV, Berlin EMRSA	POS	POS	POS	NEG	AMB	AMB	NEG	NEG	POS	POS	POS	NEG	NEG	NEG	NEG	NEG	NEG	NEG
02V04750	CC45-MRSA-IV, Berlin EMRSA	POS	POS	POS	NEG	AMB	POS	NEG	NEG	POS	POS	POS	NEG	NEG	NEG	NEG	NEG	NEG	NEG
02V06592	CC45-MRSA-IV, Berlin EMRSA	POS	POS	POS	NEG	AMB	POS	NEG	NEG	POS	POS	POS	NEG	NEG	NEG	NEG	NEG	NEG	NEG
02V10868	CC45-MRSA-IV, Berlin EMRSA	POS	POS	POS	NEG	AMB	POS	NEG	NEG	POS	POS	POS	NEG	NEG	NEG	NEG	NEG	NEG	NEG
02V12394	CC45-MRSA-IV, Berlin EMRSA	POS	POS	POS	NEG	AMB	POS	NEG	NEG	POS	POS	POS	NEG	NEG	NEG	NEG	NEG	NEG	NEG
02V12942	CC45-MRSA-IV, Berlin EMRSA	POS	POS	POS	NEG	AMB	POS	NEG	NEG	POS	POS	POS	NEG	NEG	NEG	NEG	NEG	NEG	NEG
02V13026	CC45-MRSA-IV, Berlin EMRSA	POS	POS	POS	NEG	AMB	POS	NEG	NEG	POS	POS	POS	NEG	NEG	NEG	NEG	NEG	NEG	NEG
02V14700	CC45-MRSA-IV, Berlin EMRSA	POS	POS		NEG				NEG	POS			NEG	NEG	NEG	NEG	NEG	NEG	
02V15358	CC45-MRSA-IV, Berlin EMRSA	POS	POS	POS	NEG	AMB	POS	NEG	NEG	POS	POS	POS	NEG	NEG	NEG	NEG	NEG	NEG	NEG
02V16654	CC45-MRSA-IV, Berlin EMRSA	POS	POS	POS	NEG	AMB	AMB	NEG	NEG	POS	POS	POS	NEG	NEG	NEG	NEG	NEG	NEG	NEG
02V17120	CC45-MRSA-IV, Berlin EMRSA	POS	POS	POS	NEG	POS	POS	NEG	NEG	POS	POS	POS	NEG	NEG	NEG	NEG	NEG	NEG	NEG
02V17136	CC45-MRSA-IV, Berlin EMRSA	POS	POS	POS	NEG	AMB	AMB	NEG	NEG	POS	POS	POS	NEG	NEG	NEG	NEG	NEG	NEG	NEG
02V17558	CC45-MRSA-IV, Berlin EMRSA	POS	POS	POS	NEG	AMB	AMB	NEG	NEG	POS	POS	POS	NEG	NEG	NEG	NEG	NEG	NEG	NEG
02V17665	CC45-MRSA-IV, Berlin EMRSA	POS	POS	POS	NEG	NEG	AMB	NEG	NEG	POS	POS	POS	NEG	NEG	NEG	NEG	NEG	NEG	NEG
02V20390	CC45-MRSA-IV, Berlin EMRSA	AMB	AMB	POS	NEG	NEG	NEG	NEG	NEG	POS	POS	POS	NEG	NEG	NEG	NEG	NEG	NEG	NEG
02V20626	CC45-MRSA-IV, Berlin EMRSA	POS	POS	POS	NEG	AMB	POS	NEG	NEG	POS	POS	POS	NEG	NEG	NEG	NEG	NEG	NEG	NEG
02V20680	CC45-MRSA-IV, Berlin EMRSA	POS	POS	POS	NEG	NEG	NEG	NEG	NEG	POS	POS	POS	NEG	NEG	NEG	NEG	NEG	NEG	NEG
02V21980	CC45-MRSA-IV, Berlin EMRSA	POS	POS	POS	NEG	NEG	AMB	NEG	NEG	POS	POS	POS	NEG	NEG	NEG	NEG	NEG	NEG	NEG
02V22570	CC45-MRSA-IV, Berlin EMRSA	POS	POS	POS	NEG	POS	POS	NEG	NEG	POS	POS	POS	NEG	NEG	NEG	NEG	NEG	NEG	NEG
02V22982	CC45-MRSA-IV, Berlin EMRSA	POS	POS	POS	NEG	POS	POS	NEG	NEG	POS	POS	POS	NEG	NEG	NEG	NEG	NEG	NEG	NEG
02V25503	CC45-MRSA-IV, Berlin EMRSA	POS	POS	POS	NEG	POS	POS	NEG	NEG	POS	POS	POS	NEG	NEG	NEG	NEG	NEG	NEG	NEG
02V28454	CC45-MRSA-IV, Berlin EMRSA	POS	POS	POS	NEG	POS	POS	NEG	NEG	POS	POS	POS	NEG	NEG	NEG	NEG	NEG	NEG	NEG
02V29373	CC45-MRSA-IV, Berlin EMRSA	POS	POS	POS	NEG	AMB	POS	NEG	NEG	POS	POS	POS	NEG	NEG	NEG	NEG	NEG	NEG	NEG
02V30321	CC45-MRSA-IV, Berlin EMRSA	POS	POS	POS	NEG	POS	POS	NEG	NEG	POS	POS	POS	NEG	NEG	NEG	NEG	NEG	NEG	NEG
02V30341	CC45-MRSA-IV, Berlin EMRSA	POS	POS	POS	NEG	NEG	AMB	NEG	NEG	POS	POS	POS	NEG	NEG	NEG	NEG	NEG	NEG	NEG
02V31377	CC45-MRSA-IV, Berlin EMRSA	POS	POS	POS	NEG	AMB	POS	NEG	NEG	POS	POS	POS	NEG	NEG	NEG	NEG	NEG	NEG	NEG
02V31941	CC45-MRSA-IV, Berlin EMRSA	POS	POS	POS	NEG	POS	POS	NEG	NEG	POS	POS	POS	NEG	NEG	NEG	NEG	NEG	NEG	NEG
02V34679	CC45-MRSA-IV, Berlin EMRSA	POS	POS	POS	NEG	NEG	NEG	NEG	NEG	POS	POS	POS	NEG	NEG	NEG	NEG	NEG	NEG	NEG
02V35779	CC45-MRSA-IV, Berlin EMRSA	POS	AMB	POS	NEG	NEG	NEG	NEG	NEG	POS	POS	POS	NEG	NEG	NEG	NEG	NEG	NEG	NEG
02V36419	CC45-MRSA-IV, Berlin EMRSA	POS	AMB		NEG				NEG	POS			NEG	NEG	NEG	NEG	NEG	NEG	
02V36469	CC45-MRSA-IV, Berlin EMRSA	POS	POS	POS	NEG	POS	POS	NEG	NEG	POS	POS	POS	NEG	NEG	NEG	NEG	NEG	NEG	NEG

ISOLATE:	VIRULENCE : ACME LOCUS			VIRULENCE : PROTEASES										VIRULENCE : STAPHYLOCOCCAL SUPERANTIGEN/ENTEROTOXIN-LIKE GENES (SET/SSL)						
	ACME-locus: ornithincarb-amoyltrans-ferase	ACME-locus: carbamat-kinase	ACME-locus: arginine/orni-thine-antiporter	aureolysin			serin- protease A	serin- protease B	serin- protease E	glutamylendopeptidase	staphopain B, protease	staphopain A (staphylopain A), protease		staphyl. exotoxin-like protein	staphylococcal superantigen-like protein 1					
	arcB-SCC	arcC-SCC	arcD-SCC	aur (consensus)	aur (other than MRSA252)	aur (MRSA252)	splA	splB	splE	sspA	sspB	sspP (consensus)	sspP (other than ST93)	setC	ssl01/set6_probe1_11	ssl01/set6_probe2_11	ssl01/set6_probe1_12	ssl01/set6_probe2_12	ssl01/set6_probe4_11	ssl01/set6_probeRF122
**2000**																				
00V07201	NEG	NEG	NEG	POS	POS	NEG	POS	POS	NEG	POS	POS	POS	POS	POS	POS	NEG	POS	NEG	POS	NEG
00V07634	NEG	NEG	NEG	POS	POS	NEG	POS	POS	NEG	POS	POS	POS	POS	POS	POS	NEG	POS	NEG	POS	NEG
00V08701	NEG	NEG	NEG	POS	POS	NEG	POS	POS	NEG	POS	POS	POS	POS	POS	POS	NEG	POS	NEG	POS	NEG
00V09056	NEG	NEG	NEG	POS	POS	NEG	POS	POS	NEG	POS	POS	POS	POS	POS	POS	NEG	POS	NEG	POS	NEG
00V10190	NEG	NEG	NEG	POS	POS	NEG	POS	POS	NEG	POS	POS	POS	POS	POS	POS	POS	POS	AMB	POS	NEG
00V10643	NEG	NEG	NEG	POS	POS	NEG	POS	POS	NEG	POS	POS	POS	POS	POS	POS	POS	POS	NEG	POS	NEG
00V11732	NEG	NEG	NEG	POS	POS	NEG	POS	POS	NEG	POS	POS	POS	POS	POS	POS	NEG	POS	NEG	POS	NEG
00V12300	NEG	NEG	NEG	POS	POS	NEG	POS	POS	NEG	POS	POS	POS	POS	POS	POS	NEG	POS	NEG	POS	NEG
00V14652	NEG	NEG	NEG	POS	POS	NEG	POS	POS	NEG	POS	POS	POS	POS	POS	POS	NEG	POS	NEG	POS	NEG
00V24941	NEG	NEG	NEG	POS	POS	NEG	POS	POS	NEG	POS	POS	POS	POS	POS	POS	NEG	POS	NEG	POS	NEG
00V29834	NEG	NEG	NEG	POS	POS	NEG	POS	POS	NEG	POS	POS	POS	POS	POS	POS	NEG	POS	NEG	POS	NEG
00V29909	NEG	NEG	NEG	POS	POS	NEG	POS	POS	NEG	POS	POS	POS	POS	POS	POS	NEG	POS	NEG	AMB	NEG
00V30456	NEG	NEG	NEG	POS	POS	NEG	POS	POS	NEG	POS	POS	POS	POS	POS	POS	NEG	POS	NEG	POS	NEG
00V30827	NEG	NEG	NEG	POS	POS	NEG	POS	POS	NEG	POS	POS	POS	POS	POS	POS	NEG	POS	NEG	POS	NEG
00V33204	NEG	NEG	NEG	POS	POS	NEG	POS	POS	NEG	POS	POS	POS	POS	POS	POS	NEG	POS	NEG	POS	NEG
00V36490	NEG	NEG	NEG	POS	POS	NEG	POS	POS	NEG	POS	POS	POS	POS	POS	POS	NEG	POS	NEG	POS	NEG
00V37079	NEG	NEG	NEG	POS	POS	NEG	POS	POS	NEG	POS	POS	POS	POS	POS	POS	NEG	POS	NEG	POS	NEG
00V40116	NEG	NEG	NEG	POS	POS	NEG	POS	POS	NEG	POS	POS	POS	POS	POS	POS	NEG	POS	NEG	POS	NEG
00V41429	NEG	NEG	NEG	POS	POS	NEG	POS	POS	NEG	POS	POS	POS	POS	POS	POS	NEG	POS	NEG	POS	NEG
00V42118	NEG	NEG	NEG	POS	POS	NEG	POS	POS	NEG	POS	POS	POS	POS	POS	POS	NEG	POS	NEG	POS	NEG
00V44135	NEG	NEG	NEG	POS	POS	NEG	POS	POS	NEG	POS	POS	POS	POS	POS	POS	NEG	POS	NEG	POS	NEG
00V44538	NEG	NEG	NEG	POS	POS	NEG	POS	POS	NEG	POS	POS	POS	POS	POS	POS	NEG	POS	NEG	POS	NEG
00V44990	NEG	NEG	NEG	POS	POS	NEG	POS	POS	NEG	POS	POS	POS	POS	POS	POS	NEG	POS	NEG	POS	NEG
00V46910	NEG	NEG	NEG	POS	POS	NEG	POS	POS	NEG	POS	POS	POS	POS	POS	POS	NEG	POS	NEG	POS	NEG
00V07063	NEG	NEG	NEG	POS	POS	NEG	POS	POS	NEG	POS	POS	POS	POS	POS	POS	POS	POS	AMB	POS	NEG
00V46921	NEG	NEG	NEG	POS	POS	NEG	POS	POS	NEG	POS	POS	POS	POS	POS	POS	NEG	POS	NEG	POS	NEG
00V08510	NEG	NEG	NEG	POS	POS	NEG	POS	POS	POS	POS	POS	POS	POS	POS						NEG
00V25538	NEG	NEG	NEG	POS	POS	NEG	POS	POS	POS	POS	POS	POS	POS	POS	POS	NEG	NEG	NEG	POS	NEG
00V38664	NEG	NEG	NEG	POS	POS	NEG	POS	POS	POS	POS	POS	POS	POS	POS						NEG
00V46488	NEG	NEG	NEG	POS	POS	NEG	POS	POS	POS	POS	POS	POS	POS	POS	POS	NEG	AMB	NEG	POS	NEG
00V44035	NEG	NEG	NEG	POS	POS	NEG	POS	POS	POS	POS	POS	POS	POS	POS						NEG
00V13324	NEG	NEG	NEG	POS	NEG	POS	NEG	NEG	NEG	POS	POS	POS	POS	POS	NEG	POS	POS	NEG	NEG	NEG
00B02046	NEG	NEG	NEG	POS	NEG	POS	NEG	NEG	NEG	POS	POS	POS	POS	POS	NEG	POS	POS	NEG	POS	NEG
00V11299	NEG	NEG	NEG	POS	NEG	POS	NEG	NEG	NEG	POS	POS	POS	POS	POS	NEG	POS	POS	NEG	POS	NEG
00V14125	NEG	NEG	NEG	POS	NEG	POS	NEG	NEG	NEG	POS	POS	POS	POS	AMB	NEG	POS	AMB	NEG	POS	NEG
00V16040	NEG	NEG	NEG	AMB	NEG	POS	NEG	NEG	NEG	POS	POS	POS	POS	NEG	NEG	POS	NEG	NEG	AMB	NEG
00V25025	NEG	NEG	NEG	AMB	NEG	POS	NEG	NEG	NEG	POS	POS	POS	POS	AMB	NEG	POS	AMB	NEG	AMB	NEG
00V29618	NEG	NEG	NEG	AMB	NEG	POS	NEG	NEG	NEG	POS	POS	POS	POS	AMB	NEG	POS	AMB	NEG	AMB	NEG
00V30989	NEG	NEG	NEG	AMB	NEG	POS	NEG	NEG	NEG	POS	POS	POS	POS	AMB	NEG	POS	NEG	NEG	AMB	NEG
00V31007	NEG	NEG	NEG	AMB	NEG	POS	NEG	NEG	NEG	POS	POS	POS	POS	AMB	NEG	POS	AMB	NEG	AMB	NEG
00V31700	NEG	NEG	NEG	AMB	NEG	POS	NEG	NEG	NEG	POS	POS	POS	POS	AMB	NEG	POS	AMB	NEG	POS	NEG
00V32193	NEG	NEG	NEG	AMB	NEG	POS	NEG	NEG	NEG	POS	POS	POS	POS	POS	NEG	POS	AMB	NEG	POS	NEG
00V43085	NEG	NEG	NEG	AMB	NEG	POS	NEG	NEG	NEG	POS	POS	POS	POS	AMB	NEG	POS	AMB	NEG	POS	NEG
00V44382	NEG	NEG	NEG	NEG	NEG	POS	NEG	NEG	NEG	POS	POS	POS	POS	NEG	NEG	AMB	NEG	NEG	NEG	NEG
00V46648	NEG	NEG	NEG	POS	NEG	POS	NEG	NEG	NEG	POS	POS	POS	POS	POS	NEG	POS	AMB	NEG	POS	NEG
00V46843	NEG	NEG	NEG	POS	NEG	POS	NEG	NEG	NEG	POS	POS	POS	POS	POS	NEG	POS	POS	NEG	POS	NEG
00V47014	NEG	NEG	NEG	POS	NEG	POS	NEG	NEG	NEG	POS	POS	POS	POS	POS	NEG	POS	POS	NEG	POS	NEG
00V47053	NEG	NEG	NEG	NEG	NEG	POS	NEG	NEG	NEG	POS	POS	POS	POS	POS						NEG
00V47227	NEG	NEG	NEG	POS	NEG	POS	NEG	NEG	NEG	POS	POS	POS	POS	POS	NEG	POS	POS	NEG	POS	NEG
00V47429	NEG	NEG	NEG	AMB	NEG	POS	NEG	NEG	NEG	POS	POS	POS	POS	AMB	NEG	POS	NEG	NEG	AMB	NEG
00V47531	NEG	NEG	NEG	POS	NEG	POS	NEG	NEG	NEG	POS	POS	POS	POS	POS	NEG	POS	POS	NEG	POS	NEG
**2001**																				
01V27106	NEG	NEG	NEG	POS	POS	NEG	POS	POS	POS	POS	POS	POS	POS	POS	NEG	POS	NEG	POS	NEG	NEG
01V41025	NEG	NEG	NEG	POS	POS	NEG	POS	POS	NEG	POS	POS	POS	POS	POS	POS	POS	POS	AMB	POS	NEG
01V12125	NEG	NEG	NEG	POS	POS	NEG	POS	POS	NEG	POS	POS	POS	POS	POS	POS	NEG	POS	NEG	POS	NEG
01V29747	NEG	NEG	NEG	POS	POS	NEG	POS	POS	NEG	POS	POS	POS	POS	POS	POS	NEG	POS	NEG	POS	NEG
01V32464	NEG	NEG	NEG	POS	POS	NEG	POS	POS	NEG	POS	POS	POS	POS	POS	POS	NEG	POS	NEG	POS	NEG
01V39703	NEG	NEG	NEG	POS	POS	NEG	POS	POS	NEG	POS	POS	POS	POS	POS	POS	NEG	POS	NEG	POS	NEG
01V40228							POS	POS						POS	POS	NEG	POS	NEG	POS	
01B05861	NEG	NEG	NEG	POS	NEG	POS	POS	POS	POS	POS	POS	POS	POS	POS	POS	NEG	NEG	NEG	POS	NEG
01V34572	NEG	NEG	NEG	NEG	NEG	POS	POS	POS	POS	POS	POS	POS	POS	POS						NEG
01V36469	NEG	NEG	NEG	POS	NEG	POS	POS	POS	POS	POS	POS	POS	POS	POS						NEG
01V39123	NEG	NEG	NEG	POS	NEG	POS	POS	POS	POS	POS	POS	POS	POS	POS	POS	NEG	POS	NEG	POS	NEG
01V39143	NEG	NEG	NEG	POS	NEG	POS	POS	POS	POS	POS	POS	POS	POS	POS	POS	NEG	POS	NEG	POS	NEG
01V10962	NEG	NEG	NEG	POS	POS	NEG	POS	POS	POS	POS	POS	POS	POS	POS						NEG
01V10963	NEG	NEG	NEG	POS	POS	NEG	POS	POS	POS	POS	POS	POS	POS	POS						NEG
01V36287	NEG	NEG	NEG	POS	POS	NEG	POS	POS	POS	POS	POS	POS	POS	POS	POS	NEG	AMB	NEG	POS	NEG
01V25329	NEG	NEG	NEG	POS	NEG	POS	NEG	NEG	NEG	POS	POS	POS	POS	POS	NEG	POS	AMB	NEG	NEG	NEG
01V07770							NEG	NEG						POS						
01V20122	NEG	NEG	NEG	POS	NEG	POS	NEG	NEG	NEG	POS	POS	POS	POS	POS	NEG	POS	POS	NEG	NEG	NEG
01V43277	NEG	NEG	NEG	POS	NEG	POS	NEG	NEG	NEG	POS	POS	POS	POS	POS	NEG	POS	POS	NEG	NEG	NEG
01F02744	NEG	NEG	NEG	AMB	NEG	POS	NEG	NEG	NEG	POS	POS	POS	POS	NEG	NEG	POS	NEG	NEG	AMB	NEG
01V01127	NEG	NEG	NEG	AMB	NEG	POS	NEG	NEG	NEG	POS	POS	POS	POS	AMB	NEG	POS	NEG	NEG	AMB	NEG
01V01445	NEG	NEG	NEG	POS	NEG	POS	NEG	NEG	NEG	POS	POS	POS	POS	POS	NEG	POS	POS	NEG	POS	NEG
01V02575	NEG	NEG	NEG	NEG	NEG	POS	NEG	NEG	NEG	POS	POS	POS	POS	NEG	NEG	AMB	NEG	NEG	NEG	NEG
01V02636	NEG	NEG	NEG	POS	NEG	POS	NEG	NEG	NEG	POS	POS	POS	POS	POS	NEG	POS	AMB	NEG	POS	NEG
01V03483	NEG	NEG	NEG	NEG	NEG	POS	NEG	NEG	NEG	POS	POS	POS	POS	NEG	NEG	AMB	NEG	NEG	NEG	NEG
01V04728	NEG	NEG	NEG	AMB	NEG	POS	NEG	NEG	NEG	POS	POS	POS	POS	AMB	NEG	POS	AMB	NEG	POS	NEG
01V05972	NEG	NEG	NEG	AMB	NEG	POS	NEG	NEG	NEG	POS	POS	POS	POS	NEG	NEG	AMB	NEG	NEG	NEG	NEG
01V07139	NEG	NEG	NEG	POS	NEG	POS	NEG	NEG	NEG	POS	POS	POS	POS	POS	NEG	POS	AMB	NEG	POS	NEG
01V08133	NEG	NEG	NEG	AMB	NEG	POS	NEG	NEG	NEG	POS	POS	POS	POS	AMB	NEG	POS	NEG	NEG	POS	NEG
01V09872	NEG	NEG	NEG	AMB	NEG	POS	NEG	NEG	NEG	POS	POS	POS	POS	AMB	NEG	POS	AMB	NEG	POS	NEG
01V12465	NEG	NEG	NEG	AMB	NEG	POS	NEG	NEG	NEG	POS	POS	POS	POS	NEG	NEG	POS	NEG	NEG	AMB	NEG
01V16151	NEG	NEG	NEG	AMB	NEG	POS	NEG	NEG	NEG	POS	POS	POS	POS	NEG	NEG	POS	NEG	NEG	AMB	NEG
01V16873	NEG	NEG	NEG	AMB	NEG	POS	NEG	NEG	NEG	POS	POS	POS	POS	AMB	NEG	POS	AMB	NEG	POS	NEG
01V17796	NEG	NEG	NEG	POS	NEG	POS	NEG	NEG	NEG	POS	POS	POS	POS	AMB	NEG	POS	AMB	NEG	POS	NEG
01V18681	NEG	NEG	NEG	AMB	NEG	POS	NEG	NEG	NEG	POS	POS	POS	POS	AMB	NEG	POS	AMB	NEG	POS	NEG
01V20066	NEG	NEG	NEG	AMB	NEG	POS	NEG	NEG	NEG	POS	POS	POS	POS	AMB	NEG	POS	AMB	NEG	POS	NEG
01V23050	NEG	NEG	NEG	AMB	NEG	POS	NEG	NEG	NEG	POS	POS	POS	POS	POS	NEG	POS	AMB	NEG	POS	NEG
01V25584	NEG	NEG	NEG	NEG	NEG	POS	NEG	NEG	NEG	POS	POS	POS	POS	NEG	NEG	NEG	NEG	NEG	NEG	NEG
01V31705	NEG	NEG	NEG	POS	NEG	POS	NEG	NEG	NEG	POS	POS	POS	POS	POS	NEG	POS	POS	NEG	POS	NEG
01V34968	NEG	NEG	NEG	POS	NEG	POS	NEG	NEG	NEG	POS	POS	POS	POS	POS	NEG	POS	POS	NEG	POS	NEG
01V36186	NEG	NEG	NEG	NEG	NEG	POS	NEG	NEG	NEG	POS	POS	POS	POS	NEG	NEG	AMB	NEG	NEG	NEG	NEG
01V36534	NEG	NEG	NEG	AMB	NEG	POS	NEG	NEG	NEG	POS	POS	POS	POS	AMB	NEG	POS	NEG	NEG	AMB	NEG
01V36588	NEG	NEG	NEG	POS	NEG	POS	NEG	NEG	NEG	POS	POS	POS	POS	POS	NEG	POS	POS	NEG	POS	NEG
01V37990	NEG	NEG	NEG	POS	NEG	POS	NEG	NEG	NEG	POS	POS	POS	POS	POS	NEG	POS	POS	NEG	POS	NEG
01V39610	NEG	NEG	NEG	POS	NEG	POS	NEG	NEG	NEG	POS	POS	POS	POS	POS	NEG	POS	POS	NEG	POS	NEG
01V40229	NEG	NEG	NEG	AMB	NEG	POS	NEG	NEG	NEG	POS	POS	POS	POS	POS	NEG	POS	AMB	NEG	POS	NEG
01V40374							NEG	NEG						POS						
01V41056	NEG	NEG	NEG	NEG	NEG	POS	NEG	NEG	NEG	POS	POS	POS	POS	NEG	NEG	POS	NEG	NEG	NEG	AMB
01V42009	NEG	NEG	NEG	AMB	NEG	POS	NEG	NEG	NEG	POS	POS	POS	POS	AMB	NEG	POS	AMB	NEG	POS	NEG
01V43511	NEG	NEG	NEG	NEG	NEG	POS	NEG	NEG	NEG	POS	POS	POS	POS	NEG	NEG	POS	NEG	NEG	NEG	NEG
**2002**																				
02V10698	NEG	NEG	NEG	POS	POS	NEG	POS	POS	NEG	POS	POS	POS	POS	POS	POS	NEG	POS	NEG	POS	NEG
02V13140	NEG	NEG	NEG	POS	POS	NEG	POS	POS	NEG	POS	POS	POS	POS	POS	POS	NEG	POS	NEG	POS	NEG
02V20759	NEG	NEG	NEG	AMB	POS	NEG	POS	POS	NEG	POS	POS	POS	POS	POS	POS	NEG	POS	NEG	POS	NEG
02B06271	NEG	NEG	NEG	POS	POS	NEG	POS	POS	NEG	POS	POS	POS	POS	POS	POS	NEG	POS	NEG	POS	NEG
02V04116	NEG	NEG	NEG	POS	POS	NEG	POS	POS	NEG	POS	POS	POS	POS	POS	POS	NEG	POS	NEG	POS	NEG
02V29922							POS	POS						POS	POS	NEG	POS	NEG	POS	
02V33634	NEG	NEG	NEG	POS	POS	NEG	POS	POS	NEG	POS	POS	POS	POS	POS	POS	NEG	POS	NEG	POS	NEG
02V34748	NEG	NEG	NEG	POS	POS	NEG	POS	POS	NEG	POS	POS	POS	POS	POS	POS	NEG	POS	NEG	POS	NEG
02V17002	NEG	NEG	NEG	POS	POS	NEG	POS	POS	POS	POS	POS	POS	POS	POS	POS	NEG	NEG	NEG	POS	NEG
02V33579	AMB	NEG	NEG	POS	POS	NEG	POS	POS	POS	POS	POS	POS	POS	POS						NEG
02V15498	NEG	NEG	NEG	POS	NEG	POS	NEG	NEG	NEG	POS	POS	POS	POS	POS	NEG	POS	POS	NEG	NEG	NEG
02C05319	NEG	NEG	NEG	POS	NEG	POS	NEG	NEG	NEG	POS	POS	POS	POS	POS	NEG	POS	POS	NEG	NEG	NEG
02U10255	NEG	NEG	NEG	POS	NEG	POS	NEG	NEG	NEG	POS	POS	POS	POS	POS	NEG	POS	AMB	NEG	NEG	NEG
02V12175	NEG	NEG	NEG	POS	NEG	POS	NEG	NEG	NEG	POS	POS	POS	POS	POS	NEG	POS	POS	NEG	NEG	NEG
02V13054	NEG	NEG	NEG	POS	NEG	POS	NEG	NEG	NEG	POS	POS	POS	POS	POS	NEG	POS	POS	NEG	NEG	NEG
02V13950	NEG	NEG	NEG	POS	NEG	POS	NEG	NEG	NEG	POS	POS	POS	POS	POS	NEG	POS	POS	NEG	NEG	NEG
02V21259	NEG	NEG	NEG	AMB	NEG	POS	NEG	NEG	NEG	POS	POS	POS	POS	POS	NEG	POS	POS	NEG	NEG	NEG
02V22657	NEG	NEG	NEG	POS	NEG	POS	NEG	NEG	NEG	POS	POS	POS	POS	POS	NEG	POS	POS	NEG	NEG	NEG
02V29051	NEG	NEG	NEG	POS	NEG	POS	NEG	NEG	NEG	POS	POS	POS	POS	POS	NEG	POS	POS	NEG	NEG	NEG
02V30792	NEG	NEG	NEG	POS	NEG	POS	NEG	NEG	POS	POS	POS	POS	POS	NEG	NEG	POS	POS	NEG	POS	NEG
02U07096	NEG	NEG	NEG	AMB	NEG	POS	NEG	NEG	NEG	POS	POS	POS	POS	POS	NEG	POS	AMB	NEG	POS	NEG
02V00570	NEG	NEG	NEG	NEG	NEG	POS	NEG	NEG	NEG	POS	POS	POS	POS	AMB	NEG	POS	NEG	NEG	POS	NEG
02V01730	NEG	NEG	NEG	NEG	NEG	POS	NEG	NEG	NEG	POS	POS	POS	POS	NEG	NEG	AMB	NEG	NEG	NEG	NEG
02V01870	NEG	NEG	NEG	NEG	NEG	POS	NEG	NEG	NEG	POS	POS	POS	POS	AMB	NEG	POS	AMB	NEG	POS	NEG
02V04750	NEG	NEG	NEG	POS	NEG	POS	NEG	NEG	NEG	POS	POS	POS	POS	POS	NEG	POS	POS	NEG	POS	NEG
02V06592	NEG	NEG	NEG	AMB	NEG	POS	NEG	NEG	NEG	POS	POS	POS	POS	POS	NEG	POS	AMB	NEG	POS	NEG
02V10868	NEG	NEG	NEG	AMB	NEG	POS	NEG	NEG	NEG	POS	POS	POS	POS	AMB	NEG	POS	AMB	NEG	POS	NEG
02V12394	NEG	NEG	NEG	AMB	NEG	POS	NEG	NEG	NEG	POS	POS	POS	POS	AMB	NEG	POS	AMB	NEG	POS	NEG
02V12942	NEG	NEG	NEG	AMB	NEG	POS	NEG	NEG	NEG	POS	POS	POS	POS	POS	NEG	POS	AMB	NEG	POS	NEG
02V13026	NEG	NEG	NEG	AMB	NEG	POS	NEG	NEG	NEG	POS	POS	POS	POS	POS	NEG	POS	POS	NEG	POS	NEG
02V14700							NEG	NEG						NEG						
02V15358	NEG	NEG	NEG	AMB	NEG	POS	NEG	NEG	NEG	POS	POS	POS	POS	AMB	NEG	POS	AMB	NEG	POS	NEG
02V16654	NEG	NEG	NEG	NEG	NEG	POS	NEG	NEG	NEG	POS	POS	POS	POS	AMB	NEG	POS	AMB	NEG	AMB	NEG
02V17120	NEG	NEG	NEG	POS	NEG	POS	NEG	NEG	NEG	POS	POS	POS	POS	POS	NEG	POS	POS	NEG	POS	NEG
02V17136	NEG	NEG	NEG	NEG	NEG	POS	NEG	NEG	NEG	POS	POS	POS	POS	AMB	NEG	POS	AMB	NEG	POS	NEG
02V17558	NEG	NEG	NEG	NEG	NEG	POS	NEG	NEG	NEG	POS	POS	POS	POS	AMB	NEG	POS	AMB	NEG	POS	NEG
02V17665	NEG	NEG	NEG	NEG	NEG	POS	NEG	NEG	NEG	POS	POS	POS	POS	AMB	NEG	POS	NEG	NEG	AMB	NEG
02V20390	NEG	NEG	NEG	NEG	NEG	POS	NEG	NEG	NEG	POS	POS	POS	POS	NEG	NEG	POS	NEG	NEG	NEG	NEG
02V20626	NEG	NEG	NEG	NEG	NEG	POS	NEG	NEG	NEG	POS	POS	POS	POS	AMB	NEG	POS	AMB	NEG	POS	NEG
02V20680	NEG	NEG	NEG	NEG	NEG	POS	NEG	NEG	NEG	POS	POS	POS	POS	NEG	NEG	POS	NEG	NEG	AMB	NEG
02V21980	NEG	NEG	NEG	NEG	NEG	POS	NEG	NEG	NEG	POS	POS	POS	POS	NEG	NEG	POS	NEG	NEG	AMB	NEG
02V22570	NEG	NEG	NEG	AMB	NEG	POS	NEG	NEG	NEG	POS	POS	POS	POS	POS	NEG	POS	AMB	NEG	POS	NEG
02V22982	NEG	NEG	NEG	POS	NEG	POS	NEG	NEG	NEG	POS	POS	POS	POS	POS	NEG	POS	AMB	NEG	POS	NEG
02V25503	NEG	NEG	NEG	AMB	NEG	POS	NEG	NEG	NEG	POS	POS	POS	POS	POS	NEG	POS	AMB	NEG	POS	NEG
02V28454	NEG	NEG	NEG	AMB	NEG	POS	NEG	NEG	NEG	POS	POS	POS	POS	POS	NEG	POS	AMB	NEG	POS	NEG
02V29373	NEG	NEG	NEG	AMB	NEG	POS	NEG	NEG	NEG	POS	POS	POS	POS	AMB	NEG	POS	AMB	NEG	POS	NEG
02V30321	NEG	NEG	NEG	POS	NEG	POS	NEG	NEG	NEG	POS	POS	POS	POS	POS	NEG	POS	POS	NEG	POS	NEG
02V30341	NEG	NEG	NEG	NEG	NEG	POS	NEG	NEG	NEG	POS	POS	POS	POS	AMB	NEG	POS	NEG	NEG	AMB	NEG
02V31377	NEG	NEG	NEG	AMB	NEG	POS	NEG	NEG	NEG	POS	POS	POS	POS	AMB	NEG	POS	AMB	NEG	AMB	NEG
02V31941	NEG	NEG	NEG	POS	NEG	POS	NEG	NEG	NEG	POS	POS	POS	POS	POS	NEG	POS	AMB	NEG	POS	NEG
02V34679	NEG	NEG	NEG	NEG	NEG	POS	NEG	NEG	NEG	POS	POS	POS	POS	NEG	NEG	POS	NEG	NEG	NEG	NEG
02V35779	NEG	NEG	NEG	NEG	NEG	POS	NEG	NEG	NEG	POS	POS	POS	POS	AMB						NEG
02V36419							NEG	NEG						AMB						
02V36469	NEG	NEG	NEG	POS	NEG	POS	NEG	NEG	NEG	POS	POS	POS	POS	POS	NEG	POS	POS	NEG	POS	NEG

ISOLATE:	STRAIN	VIRULENCE : STAPHYLOCOCCAL SUPERANTIGEN/ENTEROTOXIN-LIKE GENES (SET/SSL)																																	CAPSULE- AND BIOFILM-ASSOCIATED GENES																
		staphylococcal superantigen-like protein 2		staphylococcal superantigen-like protein 3			staphylococcal superantigen-like protein 4		staphylococcal superantigen-like protein 5				staphylococcal superantigen-like protein 6		staphylococcal superantigen-like protein 7			staphylococcal superantigen-like protein 8		staphylococcal superantigen-like protein 9			staphylococcal superantigen-like protein 10			staphylococcal superantigene-like protein 11				staphylococcal exotoxin-like protein, second locus					Capsule type 1	capsular poly-saccharide synthesis enzyme	O-antigen poly-merase	capsular poly-saccharide biosyn- thesis protein	Capsule type 5	capsular poly-saccharide synthesis enzyme	O-antigen poly-merase	capsular poly-saccharide biosyn- thesis protein	Capsule type 8	capsular poly-saccharide synthesis enzyme	capsular poly-saccharide biosyn- thesis protein	O-antigen poly-merase	capsular poly-saccharide biosyn- thesis protein	intercellular adhesion protein A	intercellular adhesion protein C	biofilm PIA synthesis protein D	surface protein involved in biofilm formation
		ssl02/set7	ssl02/set7 (MRSA252)	ssl03/set8_probe 1	ssl03/set8_probe 2	ssl03/set8 (MRSA252, SAR0424)	ssl04/set9	ssl04/set9 (MRSA252, SAR0425)	ssl05/set3_probe 1	ssl05/set3 (RF122, probe-611)	ssl05/set3_probe 2 (612)	ssl05/set3 (MRSA252)	ssl06/set21	ssl06 (NCTC8325+MW2)	ssl07/set1	ssl07/set1 (MRSA252)	ssl07/set1 (AF188836)	ssl08/set12_probe 1	ssl08/set12_probe 2	ssl09/set5_probe 1	ssl09/set5_probe 2	ssl09/set5 (MRSA252)	ssl10/set4	ssl10 (RF122)	ssl10/set4 (MRSA252)	ssl11/set2 (COL)	ssl11+set2(Mu50+N315)	ssl11+set2(MW2+MSSA 476)	ssl11/set2 (MRSA252)	setB3	setB3 (MRSA252)	setB2	setB2 (MRSA252)	setB1	cap 1 (total)	capH1	capJ1	capK1	cap 5 (total)	capH5	capJ5	capK5	cap 8 (total)	capH8	capI8	capJ8	capK8	icaA	icaC	icaD	bap
**2000**																																																			
00V07201	CC5/ST228-MRSA, South German EMRSA (truncated SCCmec element)	POS	AMB	POS	POS	NEG	POS	AMB	POS	AMB	POS	NEG	NEG	NEG	POS	AMB	AMB	POS	POS	POS	POS	NEG	POS	AMB	AMB	NEG	POS	NEG	NEG	POS	NEG	POS	NEG	POS	NEG	NEG	NEG	NEG	POS	POS	POS	POS	NEG	NEG	NEG	NEG	NEG	POS	POS	POS	NEG
00V07634	CC5/ST228-MRSA, South German EMRSA (truncated SCCmec element)	POS	AMB	POS	POS	NEG	POS	NEG	POS	AMB	POS	NEG	NEG	NEG	POS	AMB	AMB	POS	POS	POS	POS	NEG	POS	AMB	AMB	NEG	POS	NEG	NEG	POS	NEG	POS	NEG	POS	NEG	NEG	NEG	NEG	POS	POS	POS	POS	NEG	NEG	NEG	NEG	NEG	POS	POS	POS	NEG
00V08701	CC5/ST228-MRSA, South German EMRSA (truncated SCCmec element)	POS	NEG	POS	POS	NEG	POS	NEG	POS	POS	NEG	NEG	NEG	NEG	POS	AMB	NEG	POS	POS	POS	POS	NEG	POS	NEG	NEG	NEG	POS	NEG	NEG	POS	NEG	POS	NEG	POS	NEG	NEG	NEG	NEG	POS	POS	POS	POS	NEG	NEG	NEG	NEG	NEG	POS	POS	POS	NEG
00V09056	CC5/ST228-MRSA, South German EMRSA (truncated SCCmec element)	POS	AMB	POS	POS	NEG	POS	NEG	POS	AMB	POS	NEG	NEG	NEG	POS	AMB	AMB	POS	POS	POS	POS	NEG	POS	NEG	NEG	NEG	POS	NEG	NEG	POS	NEG	POS	NEG	POS	NEG	NEG	NEG	NEG	POS	POS	POS	POS	NEG	NEG	NEG	NEG	NEG	POS	POS	POS	NEG
00V10190	CC5/ST228-MRSA, South German EMRSA (truncated SCCmec element)	POS	AMB	POS	POS	NEG	POS	AMB	POS	POS	AMB	NEG	NEG	NEG	AMB	POS	AMB	POS	POS	POS	POS	NEG	POS	AMB	AMB	NEG	POS	NEG	NEG	POS	NEG	POS	NEG	POS	NEG	NEG	NEG	NEG	POS	POS	POS	POS	NEG	NEG	NEG	NEG	NEG	POS	POS	POS	NEG
00V10643	CC5/ST228-MRSA, South German EMRSA (truncated SCCmec element)	POS	AMB	POS	POS	NEG	POS	AMB	POS	AMB	POS	NEG	NEG	NEG	AMB	POS	AMB	POS	POS	POS	POS	NEG	POS	AMB	AMB	NEG	POS	NEG	NEG	POS	NEG	POS	NEG	POS	NEG	NEG	NEG	NEG	POS	POS	POS	POS	NEG	NEG	NEG	NEG	NEG	POS	POS	POS	NEG
00V11732	CC5/ST228-MRSA, South German EMRSA (truncated SCCmec element)	POS	AMB	POS	POS	NEG	POS	NEG	POS	POS	NEG	NEG	NEG	NEG	POS	AMB	AMB	POS	POS	POS	POS	NEG	POS	NEG	NEG	NEG	POS	NEG	NEG	POS	NEG	POS	NEG	POS	NEG	NEG	NEG	NEG	POS	POS	POS	POS	NEG	NEG	NEG	NEG	NEG	POS	POS	POS	NEG
00V12300	CC5/ST228-MRSA, South German EMRSA (truncated SCCmec element)	POS	AMB	POS	POS	NEG	POS	NEG	POS	POS	NEG	NEG	NEG	NEG	POS	AMB	NEG	POS	POS	POS	POS	NEG	POS	NEG	NEG	NEG	POS	NEG	NEG	POS	NEG	POS	NEG	POS	NEG	NEG	NEG	NEG	POS	POS	POS	POS	NEG	NEG	NEG	NEG	NEG	POS	POS	POS	NEG
00V14652	CC5/ST228-MRSA, South German EMRSA (truncated SCCmec element)	POS	AMB	POS	POS	NEG	POS	NEG	POS	POS	NEG	NEG	NEG	NEG	POS	AMB	NEG	POS	POS	POS	POS	NEG	POS	NEG	NEG	NEG	POS	NEG	NEG	POS	NEG	POS	NEG	POS	NEG	NEG	NEG	NEG	POS	POS	POS	POS	NEG	NEG	NEG	NEG	NEG	POS	POS	POS	NEG
00V24941	CC5/ST228-MRSA, South German EMRSA (truncated SCCmec element)	POS	AMB	POS	POS	NEG	POS	NEG	POS	POS	NEG	NEG	NEG	NEG	POS	AMB	AMB	POS	POS	POS	POS	NEG	POS	NEG	AMB	NEG	POS	NEG	NEG	POS	NEG	POS	NEG	POS	NEG	NEG	NEG	NEG	POS	POS	POS	POS	NEG	NEG	NEG	NEG	NEG	POS	POS	POS	NEG
00V29834	CC5/ST228-MRSA, South German EMRSA (truncated SCCmec element)	POS	AMB	POS	POS	NEG	POS	NEG	POS	POS	NEG	NEG	NEG	NEG	POS	AMB	NEG	POS	POS	POS	POS	NEG	POS	NEG	NEG	NEG	POS	NEG	NEG	POS	NEG	POS	NEG	POS	NEG	NEG	NEG	NEG	POS	POS	POS	POS	NEG	NEG	NEG	NEG	NEG	POS	POS	POS	NEG
00V29909	CC5/ST228-MRSA, South German EMRSA (truncated SCCmec element)	POS	AMB	POS	POS	NEG	POS	NEG	POS	POS	NEG	NEG	NEG	NEG	POS	AMB	NEG	POS	POS	POS	POS	NEG	POS	NEG	NEG	NEG	POS	NEG	NEG	POS	NEG	POS	NEG	POS	NEG	NEG	NEG	NEG	POS	POS	POS	POS	NEG	NEG	NEG	NEG	NEG	POS	POS	POS	NEG
00V30456	CC5/ST228-MRSA, South German EMRSA (truncated SCCmec element)	POS	NEG	POS	POS	NEG	POS	NEG	POS	POS	NEG	NEG	NEG	NEG	POS	AMB	NEG	POS	POS	POS	POS	NEG	POS	NEG	NEG	NEG	POS	NEG	NEG	POS	NEG	POS	NEG	POS	NEG	NEG	NEG	NEG	POS	POS	POS	POS	NEG	NEG	NEG	NEG	NEG	POS	POS	POS	NEG
00V30827	CC5/ST228-MRSA, South German EMRSA (truncated SCCmec element)	POS	AMB	POS	POS	NEG	POS	NEG	POS	AMB	POS	NEG	NEG	NEG	POS	AMB	AMB	POS	POS	POS	POS	NEG	POS	AMB	AMB	NEG	POS	NEG	NEG	POS	NEG	POS	NEG	POS	NEG	NEG	NEG	NEG	POS	POS	POS	POS	NEG	NEG	NEG	NEG	NEG	POS	POS	POS	NEG
00V33204	CC5/ST228-MRSA, South German EMRSA (truncated SCCmec element)	POS	AMB	POS	POS	NEG	POS	NEG	POS	AMB	POS	NEG	NEG	NEG	POS	AMB	AMB	POS	POS	POS	POS	NEG	POS	NEG	AMB	NEG	POS	NEG	NEG	POS	NEG	POS	NEG	POS	NEG	NEG	NEG	NEG	POS	POS	POS	POS	NEG	NEG	NEG	NEG	NEG	POS	POS	POS	NEG
00V36490	CC5/ST228-MRSA, South German EMRSA (truncated SCCmec element)	POS	AMB	POS	POS	NEG	POS	NEG	POS	AMB	POS	NEG	NEG	NEG	POS	AMB	AMB	POS	POS	POS	POS	NEG	POS	AMB	AMB	NEG	POS	NEG	NEG	POS	NEG	POS	NEG	POS	NEG	NEG	NEG	NEG	POS	POS	POS	POS	NEG	NEG	NEG	NEG	NEG	POS	POS	POS	NEG
00V37079	CC5/ST228-MRSA, South German EMRSA (truncated SCCmec element)	POS	AMB	POS	POS	NEG	POS	NEG	POS	AMB	POS	NEG	NEG	NEG	POS	AMB	AMB	POS	POS	POS	POS	NEG	POS	AMB	AMB	NEG	POS	NEG	NEG	POS	NEG	POS	NEG	POS	NEG	NEG	NEG	NEG	POS	POS	POS	POS	NEG	NEG	NEG	NEG	NEG	POS	POS	POS	NEG
00V40116	CC5/ST228-MRSA, South German EMRSA (truncated SCCmec element)	POS	AMB	POS	POS	NEG	POS	AMB	POS	AMB	POS	NEG	NEG	NEG	POS	AMB	AMB	POS	POS	POS	POS	NEG	POS	AMB	AMB	NEG	POS	NEG	NEG	POS	NEG	POS	NEG	POS	NEG	NEG	NEG	NEG	POS	POS	POS	POS	NEG	NEG	NEG	NEG	NEG	POS	POS	POS	NEG
00V41429	CC5/ST228-MRSA, South German EMRSA (truncated SCCmec element)	POS	AMB	POS	POS	NEG	POS	NEG	POS	POS	NEG	NEG	NEG	NEG	POS	AMB	AMB	POS	POS	POS	POS	NEG	POS	NEG	AMB	NEG	POS	NEG	NEG	POS	NEG	POS	NEG	POS	NEG	NEG	NEG	NEG	POS	POS	POS	POS	NEG	NEG	NEG	NEG	NEG	POS	POS	POS	NEG
00V42118	CC5/ST228-MRSA, South German EMRSA (truncated SCCmec element)	POS	AMB	POS	POS	NEG	POS	NEG	POS	AMB	POS	NEG	NEG	NEG	POS	AMB	AMB	POS	POS	POS	POS	NEG	POS	AMB	AMB	NEG	POS	NEG	NEG	POS	NEG	POS	NEG	POS	NEG	NEG	NEG	NEG	POS	POS	POS	POS	NEG	NEG	NEG	NEG	NEG	POS	POS	POS	NEG
00V44135	CC5/ST228-MRSA, South German EMRSA (truncated SCCmec element)	POS	AMB	POS	POS	NEG	POS	NEG	POS	POS	NEG	NEG	NEG	NEG	POS	AMB	AMB	POS	POS	POS	POS	NEG	POS	AMB	AMB	NEG	POS	NEG	NEG	POS	NEG	POS	NEG	POS	NEG	NEG	NEG	NEG	POS	POS	POS	POS	NEG	NEG	NEG	NEG	NEG	POS	POS	POS	NEG
00V44538	CC5/ST228-MRSA, South German EMRSA (truncated SCCmec element)	POS	AMB	POS	POS	NEG	POS	NEG	POS	POS	NEG	NEG	NEG	NEG	POS	AMB	NEG	POS	POS	POS	POS	NEG	POS	NEG	AMB	NEG	POS	NEG	NEG	POS	NEG	POS	NEG	POS	NEG	NEG	NEG	NEG	POS	POS	POS	POS	NEG	NEG	NEG	NEG	NEG	POS	POS	POS	NEG
00V44990	CC5/ST228-MRSA, South German EMRSA (truncated SCCmec element)	POS	AMB	POS	POS	NEG	POS	NEG	POS	POS	NEG	NEG	NEG	NEG	POS	AMB	NEG	POS	POS	POS	POS	NEG	POS	NEG	AMB	NEG	POS	NEG	NEG	POS	NEG	POS	NEG	POS	NEG	NEG	NEG	NEG	POS	POS	POS	POS	NEG	NEG	NEG	NEG	NEG	POS	POS	POS	NEG
00V46910	CC5/ST228-MRSA, South German EMRSA (truncated SCCmec element)	POS	AMB	POS	POS	NEG	POS	NEG	POS	POS	NEG	NEG	NEG	NEG	POS	AMB	NEG	POS	POS	POS	POS	NEG	POS	NEG	AMB	NEG	POS	NEG	NEG	POS	NEG	POS	NEG	POS	NEG	NEG	NEG	NEG	POS	POS	POS	POS	NEG	NEG	NEG	NEG	NEG	POS	POS	POS	NEG
00V07063	CC5/ST228-MRSA-I, South German EMRSA	POS	AMB	POS	POS	NEG	POS	AMB	POS	AMB	POS	NEG	NEG	AMB	POS	AMB	AMB	POS	POS	POS	POS	NEG	POS	AMB	AMB	NEG	POS	NEG	NEG	POS	NEG	POS	NEG	POS	AMB	AMB	POS	NEG	POS	POS	POS	POS	NEG	NEG	NEG	NEG	NEG	POS	POS	POS	NEG
00V46921	CC5-MRSA-II, Rhine-Hesse EMRSA/UK-EMRSA-3	POS	AMB	POS	POS	NEG	POS	NEG	POS	AMB	POS	NEG	NEG	NEG	POS	AMB	AMB	POS	POS	POS	POS	NEG	POS	AMB	AMB	NEG	POS	NEG	NEG	POS	NEG	POS	NEG	POS	NEG	NEG	NEG	NEG	POS	POS	POS	POS	NEG	NEG	NEG	NEG	NEG	POS	POS	POS	NEG
00V08510	CC8/ST254-MRSA-(atypical SCCmec), Hannover EMRSA	POS	NEG	POS	POS	NEG	POS	NEG	POS	NEG	POS	NEG	NEG	POS	POS	NEG	NEG	POS	POS	POS	POS	NEG	POS	NEG	NEG	POS	NEG	NEG	NEG	POS	NEG	POS	NEG	POS	NEG	NEG	NEG	NEG	POS	POS	POS	POS	NEG	NEG	NEG	NEG	NEG	POS	POS	POS	NEG
00V25538	CC8/ST254-MRSA-(atypical SCCmec), Hannover EMRSA	POS	NEG	POS	POS	NEG	POS	NEG	POS	POS	NEG	NEG	POS	POS	POS	AMB	NEG	POS	POS	POS	POS	NEG	POS	NEG	NEG	POS	NEG	NEG	NEG	POS	NEG	POS	NEG	POS	NEG	NEG	NEG	NEG	POS	POS	POS	POS	NEG	NEG	NEG	NEG	NEG	POS	POS	POS	NEG
00V38664	CC8/ST254-MRSA-(atypical SCCmec), Hannover EMRSA	POS	POS	POS	POS	NEG	POS	NEG	POS	AMB	POS	NEG	NEG	POS	POS	AMB	AMB	POS	POS	POS	POS	NEG	NEG	AMB	NEG	POS	NEG	NEG	NEG	POS	NEG	POS	NEG	POS	NEG	NEG	NEG	NEG	POS	POS	POS	POS	NEG	NEG	NEG	NEG	NEG	POS	POS	POS	NEG
00V46488	CC8/ST254-MRSA-(atypical SCCmec), Hannover EMRSA	POS	AMB	POS	POS	NEG	POS	NEG	POS	POS	NEG	NEG	POS	POS	POS	AMB	NEG	POS	POS	POS	POS	NEG	POS	NEG	AMB	POS	NEG	NEG	NEG	POS	NEG	POS	NEG	POS	NEG	NEG	NEG	NEG	POS	POS	POS	POS	NEG	NEG	NEG	NEG	NEG	POS	POS	POS	NEG
00V44035	CC8/ST254-MRSA-IV/V, UK-EMRSA-10/Hannover EMRSA	POS	NEG	POS	POS	NEG	POS	NEG	POS	NEG	POS	NEG	NEG	POS	POS	AMB	NEG	POS	POS	POS	NEG	NEG	POS	NEG	NEG	POS	NEG	NEG	NEG	POS	NEG	POS	NEG	POS	NEG	NEG	NEG	NEG	POS	POS	POS	POS	NEG	NEG	NEG	NEG	NEG	POS	POS	POS	NEG
00V13324	CC22-MRSA-IV [PVL+]	AMB	POS	NEG	NEG	AMB	NEG	POS	POS	NEG	NEG	POS	NEG	NEG	NEG	AMB	POS	NEG	NEG	POS	POS	NEG	NEG	NEG	NEG	NEG	NEG	NEG	NEG	NEG	NEG	NEG	NEG	NEG	NEG	NEG	NEG	NEG	POS	POS	POS	POS	NEG	NEG	NEG	NEG	NEG	POS	POS	POS	NEG
00B02046	CC45-MRSA-IV, Berlin EMRSA	AMB	POS	NEG	NEG	NEG	NEG	POS	NEG	NEG	NEG	POS	NEG	AMB	NEG	NEG	NEG	NEG	NEG	NEG	NEG	POS	POS	NEG	AMB	NEG	NEG	NEG	NEG	NEG	POS	NEG	POS	NEG	NEG	NEG	NEG	NEG	NEG	NEG	NEG	NEG	POS	POS	POS	POS	POS	POS	POS	POS	NEG
00V11299	CC45-MRSA-IV, Berlin EMRSA	AMB	POS	NEG	NEG	NEG	NEG	POS	NEG	NEG	NEG	POS	NEG	AMB	NEG	NEG	NEG	NEG	NEG	NEG	NEG	POS	AMB	NEG	POS	NEG	NEG	NEG	NEG	NEG	POS	NEG	POS	AMB	NEG	NEG	NEG	NEG	NEG	NEG	NEG	NEG	POS	POS	POS	POS	POS	POS	POS	POS	NEG
00V14125	CC45-MRSA-IV, Berlin EMRSA	AMB	POS	NEG	NEG	NEG	NEG	POS	NEG	NEG	NEG	POS	NEG	NEG	NEG	NEG	NEG	NEG	NEG	NEG	NEG	POS	AMB	NEG	POS	NEG	NEG	NEG	NEG	NEG	POS	NEG	NEG	NEG	NEG	NEG	NEG	NEG	NEG	NEG	NEG	NEG	POS	POS	POS	POS	POS	POS	POS	POS	NEG
00V16040	CC45-MRSA-IV, Berlin EMRSA	NEG	POS	NEG	NEG	NEG	NEG	POS	NEG	NEG	NEG	POS	NEG	NEG	NEG	NEG	NEG	NEG	NEG	NEG	NEG	POS	AMB	NEG	POS	NEG	NEG	NEG	NEG	NEG	POS	NEG	NEG	NEG	NEG	NEG	NEG	NEG	NEG	NEG	NEG	NEG	POS	POS	POS	POS	POS	POS	POS	POS	NEG
00V25025	CC45-MRSA-IV, Berlin EMRSA	AMB	POS	NEG	NEG	NEG	NEG	AMB	NEG	NEG	NEG	POS	NEG	NEG	NEG	NEG	NEG	NEG	NEG	NEG	NEG	POS	AMB	NEG	POS	NEG	NEG	NEG	NEG	NEG	POS	NEG	NEG	NEG	NEG	NEG	NEG	NEG	NEG	NEG	NEG	NEG	POS	POS	POS	POS	POS	POS	POS	POS	NEG
00V29618	CC45-MRSA-IV, Berlin EMRSA	AMB	POS	NEG	NEG	NEG	NEG	AMB	NEG	NEG	NEG	POS	NEG	NEG	NEG	NEG	NEG	NEG	NEG	NEG	NEG	POS	AMB	NEG	POS	NEG	NEG	NEG	NEG	NEG	POS	NEG	NEG	NEG	NEG	NEG	NEG	NEG	NEG	NEG	NEG	NEG	POS	POS	POS	POS	POS	POS	POS	POS	NEG
00V30989	CC45-MRSA-IV, Berlin EMRSA	NEG	POS	NEG	NEG	NEG	NEG	POS	NEG	NEG	NEG	POS	NEG	NEG	NEG	NEG	NEG	NEG	NEG	NEG	NEG	POS	AMB	NEG	POS	NEG	NEG	NEG	NEG	NEG	POS	NEG	NEG	NEG	NEG	NEG	NEG	NEG	NEG	NEG	NEG	NEG	POS	POS	POS	POS	POS	POS	POS	POS	NEG
00V31007	CC45-MRSA-IV, Berlin EMRSA	NEG	POS	NEG	NEG	NEG	NEG	AMB	NEG	NEG	NEG	POS	NEG	NEG	NEG	NEG	NEG	NEG	NEG	NEG	NEG	POS	AMB	NEG	POS	NEG	NEG	NEG	NEG	NEG	POS	NEG	NEG	NEG	NEG	NEG	NEG	NEG	NEG	NEG	NEG	NEG	POS	POS	POS	POS	POS	POS	POS	POS	NEG
00V31700	CC45-MRSA-IV, Berlin EMRSA	AMB	POS	NEG	NEG	NEG	NEG	AMB	NEG	NEG	NEG	POS	NEG	NEG	NEG	NEG	NEG	NEG	NEG	NEG	NEG	POS	AMB	NEG	POS	NEG	NEG	NEG	NEG	NEG	POS	NEG	NEG	NEG	NEG	NEG	NEG	NEG	NEG	NEG	NEG	NEG	POS	POS	POS	POS	POS	POS	POS	POS	NEG
00V32193	CC45-MRSA-IV, Berlin EMRSA	AMB	POS	NEG	NEG	NEG	NEG	AMB	NEG	NEG	NEG	POS	NEG	NEG	NEG	NEG	NEG	NEG	NEG	NEG	NEG	POS	AMB	NEG	POS	NEG	NEG	NEG	NEG	NEG	POS	NEG	NEG	NEG	NEG	NEG	NEG	NEG	NEG	NEG	NEG	NEG	POS	POS	POS	POS	POS	POS	POS	POS	NEG
00V43085	CC45-MRSA-IV, Berlin EMRSA	AMB	POS	NEG	NEG	NEG	NEG	AMB	NEG	NEG	NEG	POS	NEG	NEG	NEG	NEG	NEG	NEG	NEG	NEG	NEG	POS	AMB	NEG	POS	NEG	NEG	NEG	NEG	NEG	POS	NEG	NEG	NEG	NEG	NEG	NEG	NEG	NEG	NEG	NEG	NEG	POS	POS	POS	POS	POS	POS	POS	POS	NEG
00V44382	CC45-MRSA-IV, Berlin EMRSA	NEG	POS	NEG	NEG	NEG	NEG	AMB	NEG	NEG	NEG	POS	NEG	NEG	NEG	NEG	NEG	NEG	NEG	NEG	NEG	POS	AMB	NEG	POS	NEG	NEG	NEG	NEG	NEG	POS	NEG	NEG	NEG	NEG	NEG	NEG	NEG	NEG	NEG	NEG	NEG	POS	POS	POS	POS	POS	POS	POS	POS	NEG
00V46648	CC45-MRSA-IV, Berlin EMRSA	AMB	POS	NEG	NEG	NEG	NEG	POS	NEG	NEG	NEG	POS	NEG	NEG	NEG	NEG	NEG	NEG	NEG	NEG	NEG	POS	AMB	NEG	POS	NEG	NEG	NEG	NEG	NEG	POS	NEG	AMB	NEG	NEG	NEG	NEG	NEG	NEG	NEG	NEG	NEG	POS	POS	POS	POS	POS	POS	POS	POS	NEG
00V46843	CC45-MRSA-IV, Berlin EMRSA	AMB	POS	NEG	NEG	NEG	NEG	POS	NEG	NEG	NEG	POS	NEG	AMB	NEG	NEG	NEG	NEG	NEG	NEG	NEG	POS	AMB	NEG	POS	NEG	NEG	NEG	NEG	NEG	POS	NEG	AMB	NEG	NEG	NEG	NEG	NEG	NEG	NEG	NEG	NEG	POS	POS	POS	POS	POS	POS	POS	POS	NEG
00V47014	CC45-MRSA-IV, Berlin EMRSA	AMB	POS	NEG	NEG	NEG	NEG	POS	NEG	NEG	NEG	POS	NEG	NEG	NEG	NEG	NEG	NEG	NEG	NEG	NEG	POS	POS	NEG	AMB	NEG	NEG	NEG	NEG	NEG	POS	NEG	NEG	NEG	NEG	NEG	NEG	NEG	NEG	NEG	NEG	NEG	POS	POS	POS	POS	POS	POS	POS	POS	NEG
00V47053	CC45-MRSA-IV, Berlin EMRSA	NEG	POS	NEG	NEG	NEG	NEG	POS	NEG	NEG	NEG	POS	NEG	NEG	NEG	NEG	NEG	NEG	NEG	NEG	NEG	POS	NEG	NEG	POS	NEG	NEG	NEG	NEG	NEG	POS	NEG	NEG	POS	NEG	NEG	NEG	NEG	NEG	NEG	NEG	NEG	POS	POS	POS	POS	POS	POS	POS	POS	NEG
00V47227	CC45-MRSA-IV, Berlin EMRSA	AMB	POS	NEG	AMB	NEG	NEG	POS	NEG	POS	NEG	POS	NEG	POS	NEG	NEG	NEG	NEG	NEG	NEG	NEG	POS	AMB	AMB	POS	NEG	NEG	NEG	NEG	NEG	POS	NEG	AMB	AMB	NEG	NEG	NEG	NEG	NEG	NEG	NEG	NEG	POS	POS	POS	POS	POS	POS	POS	POS	NEG
00V47429	CC45-MRSA-IV, Berlin EMRSA	NEG	POS	NEG	NEG	NEG	NEG	AMB	NEG	NEG	NEG	POS	NEG	NEG	NEG	NEG	NEG	NEG	NEG	NEG	NEG	POS	AMB	NEG	POS	NEG	NEG	NEG	NEG	NEG	POS	NEG	NEG	NEG	NEG	NEG	NEG	NEG	NEG	NEG	NEG	NEG	POS	POS	POS	POS	POS	POS	POS	POS	NEG
00V47531	CC45-MRSA-IV, Berlin EMRSA	AMB	POS	NEG	NEG	NEG	NEG	POS	NEG	NEG	NEG	POS	NEG	AMB	NEG	NEG	NEG	NEG	NEG	NEG	NEG	POS	AMB	NEG	POS	NEG	NEG	NEG	NEG	NEG	POS	NEG	AMB	NEG	NEG	NEG	NEG	NEG	NEG	NEG	NEG	NEG	POS	POS	POS	POS	POS	POS	POS	POS	NEG
**2001**																																																			
01V27106	CC1-MRSA-IV, WA MRSA-1/57	POS	NEG	POS	POS	NEG	POS	NEG	POS	AMB	POS	NEG	POS	POS	POS	AMB	AMB	POS	POS	POS	AMB	NEG	POS	NEG	NEG	NEG	NEG	POS	NEG	POS	NEG	POS	NEG	POS	NEG	NEG	NEG	NEG	NEG	NEG	NEG	NEG	POS	POS	POS	POS	POS	POS	POS	POS	NEG
01V41025	CC5/ST228-MRSA-I, South German EMRSA	POS	AMB	POS	POS	NEG	POS	AMB	POS	AMB	POS	NEG	NEG	AMB	AMB	POS	AMB	POS	POS	POS	POS	NEG	POS	AMB	AMB	NEG	POS	NEG	NEG	POS	NEG	POS	NEG	POS	AMB	AMB	POS	NEG	POS	POS	POS	POS	NEG	NEG	NEG	NEG	NEG	POS	POS	POS	NEG
01V12125	CC5-MRSA-II, Rhine-Hesse EMRSA/UK-EMRSA-3	POS	AMB	POS	POS	NEG	POS	NEG	POS	AMB	POS	NEG	NEG	NEG	POS	AMB	NEG	POS	POS	POS	POS	NEG	POS	NEG	AMB	NEG	POS	NEG	NEG	POS	NEG	POS	NEG	POS	NEG	NEG	NEG	NEG	POS	POS	POS	POS	NEG	NEG	NEG	NEG	NEG	POS	POS	POS	NEG
01V29747	CC5-MRSA-II, Rhine-Hesse EMRSA/UK-EMRSA-3	POS	AMB	POS	POS	NEG	POS	NEG	POS	POS	NEG	NEG	NEG	NEG	POS	AMB	AMB	POS	POS	POS	POS	NEG	POS	NEG	AMB	NEG	POS	NEG	NEG	POS	NEG	POS	NEG	POS	NEG	NEG	NEG	NEG	POS	POS	POS	POS	NEG	NEG	NEG	NEG	NEG	POS	POS	POS	NEG
01V32464	CC5-MRSA-II, Rhine-Hesse EMRSA/UK-EMRSA-3	POS	NEG	POS	POS	NEG	POS	NEG	POS	NEG	POS	NEG	NEG	NEG	POS	AMB	AMB	POS	POS	POS	NEG	NEG	POS	NEG	NEG	NEG	POS	NEG	NEG	POS	NEG	POS	NEG	POS	NEG	NEG	NEG	NEG	POS	POS	POS	POS	NEG	NEG	NEG	NEG	NEG	POS	POS	POS	NEG
01V39703	CC5-MRSA-II, Rhine-Hesse EMRSA/UK-EMRSA-3	POS	NEG	POS	POS	NEG	POS	NEG	POS	AMB	POS	NEG	NEG	NEG	POS	AMB	NEG	POS	POS	POS	POS	NEG	POS	NEG	NEG	NEG	POS	NEG	NEG	POS	NEG	POS	NEG	POS	NEG	NEG	NEG	NEG	POS	POS	POS	POS	NEG	NEG	NEG	NEG	NEG	POS	POS	POS	NEG
01V40228	CC5-MRSA-II, Rhine-Hesse EMRSA/UK-EMRSA-3	POS	POS	POS		NEG	POS	NEG	POS			NEG	NEG		POS	AMB	AMB	POS		POS		NEG	POS		NEG	NEG	POS	NEG	NEG	POS	NEG	POS	NEG	POS																	
01B05861	CC8/ST239-MRSA-III, Vienna/Hungarian/Brazilian Clone	POS	NEG	POS	POS	NEG	POS	NEG	POS	POS	NEG	NEG	POS	POS	POS	AMB	NEG	POS	POS	POS	POS	NEG	POS	NEG	NEG	POS	NEG	NEG	NEG	POS	NEG	POS	NEG	POS	NEG	NEG	NEG	NEG	NEG	NEG	NEG	NEG	POS	POS	POS	POS	POS	POS	POS	POS	NEG
01V34572	CC8/ST239-MRSA-III, Vienna/Hungarian/Brazilian Clone	POS	NEG	POS	POS	NEG	POS	NEG	POS	NEG	POS	NEG	NEG	POS	POS	AMB	NEG	POS	POS	POS	NEG	NEG	POS	NEG	NEG	POS	NEG	NEG	NEG	POS	NEG	POS	NEG	POS	NEG	NEG	NEG	NEG	NEG	NEG	NEG	NEG	POS	POS	POS	POS	POS	POS	POS	POS	NEG
01V36469	CC8/ST239-MRSA-III, Vienna/Hungarian/Brazilian Clone	POS	NEG	POS	POS	NEG	POS	NEG	POS	NEG	POS	NEG	POS	POS	POS	NEG	NEG	POS	POS	AMB	POS	NEG	POS	NEG	NEG	POS	NEG	NEG	NEG	POS	NEG	POS	NEG	POS	NEG	NEG	NEG	NEG	NEG	NEG	NEG	NEG	POS	POS	POS	POS	POS	POS	POS	POS	NEG
01V39123	CC8/ST239-MRSA-III, Vienna/Hungarian/Brazilian Clone	POS	AMB	POS	POS	NEG	POS	NEG	POS	POS	AMB	NEG	POS	POS	AMB	POS	AMB	POS	POS	POS	POS	NEG	AMB	AMB	POS	POS	NEG	NEG	NEG	POS	NEG	POS	NEG	POS	NEG	NEG	NEG	NEG	NEG	NEG	NEG	NEG	POS	POS	POS	POS	POS	POS	POS	POS	NEG
01V39143	CC8/ST239-MRSA-III, Vienna/Hungarian/Brazilian Clone	POS	NEG	POS	POS	NEG	POS	NEG	POS	NEG	POS	NEG	NEG	NEG	POS	AMB	NEG	POS	POS	POS	POS	NEG	POS	NEG	NEG	POS	NEG	NEG	NEG	POS	NEG	POS	NEG	POS	NEG	NEG	NEG	NEG	NEG	NEG	NEG	NEG	POS	AMB	POS	POS	POS	POS	POS	POS	NEG
01V10962	CC8/ST254-MRSA-(atypical SCCmec), Hannover EMRSA	POS	POS	POS	POS	NEG	POS	NEG	POS	AMB	POS	NEG	NEG	POS	POS	AMB	AMB	POS	POS	POS	POS	NEG	NEG	AMB	NEG	POS	NEG	NEG	NEG	POS	NEG	POS	NEG	POS	NEG	NEG	NEG	NEG	POS	POS	POS	POS	NEG	NEG	NEG	NEG	NEG	POS	POS	POS	NEG
01V10963	CC8/ST254-MRSA-IV/V, UK-EMRSA-10/Hannover EMRSA	POS	POS	POS	POS	NEG	POS	NEG	POS	AMB	POS	NEG	NEG	POS	POS	AMB	AMB	POS	POS	POS	POS	NEG	NEG	POS	NEG	POS	NEG	NEG	NEG	POS	NEG	POS	AMB	POS	NEG	NEG	NEG	NEG	POS	POS	POS	POS	NEG	NEG	NEG	NEG	NEG	POS	POS	POS	NEG
01V36287	CC8/ST254-MRSA-IV/V, UK-EMRSA-10/Hannover EMRSA	POS	AMB	POS	POS	NEG	POS	NEG	POS	POS	NEG	NEG	POS	POS	POS	AMB	NEG	POS	POS	POS	POS	NEG	POS	NEG	AMB	POS	NEG	NEG	NEG	POS	NEG	POS	NEG	POS	NEG	NEG	NEG	NEG	POS	POS	POS	POS	NEG	NEG	NEG	NEG	NEG	POS	POS	POS	NEG
01V25329	CC22-MRSA-IV [PVL+]	AMB	POS	NEG	NEG	NEG	NEG	AMB	POS	NEG	NEG	POS	NEG	NEG	AMB	AMB	POS	NEG	NEG	POS	POS	NEG	NEG	NEG	NEG	NEG	NEG	NEG	NEG	NEG	NEG	NEG	NEG	NEG	NEG	NEG	NEG	NEG	POS	POS	POS	POS	NEG	NEG	NEG	NEG	NEG	POS	POS	POS	NEG
01V07770	CC22-MRSA-IV, Barnim/UK-EMRSA-15	POS	POS	NEG		POS	NEG	POS	POS			POS	NEG		AMB	AMB	POS	NEG		NEG		NEG	NEG		NEG	NEG	NEG	NEG	NEG	NEG	NEG	NEG	NEG	NEG																	
01V20122	CC22-MRSA-IV, Barnim/UK-EMRSA-15	AMB	POS	NEG	NEG	NEG	NEG	POS	POS	NEG	NEG	POS	NEG	NEG	NEG	NEG	POS	NEG	NEG	POS	POS	NEG	NEG	NEG	NEG	NEG	NEG	NEG	NEG	NEG	NEG	NEG	NEG	NEG	NEG	NEG	NEG	NEG	POS	POS	POS	POS	NEG	NEG	NEG	NEG	NEG	POS	POS	POS	NEG
01V43277	CC22-MRSA-IV, Barnim/UK-EMRSA-15	AMB	POS	NEG	NEG	NEG	NEG	POS	POS	NEG	NEG	POS	NEG	NEG	AMB	AMB	POS	NEG	NEG	POS	POS	NEG	NEG	NEG	NEG	NEG	NEG	NEG	NEG	NEG	NEG	NEG	NEG	NEG	NEG	NEG	NEG	NEG	POS	POS	POS	POS	NEG	NEG	NEG	NEG	NEG	POS	POS	POS	NEG
01F02744	CC45-MRSA-IV, Berlin EMRSA	NEG	POS	NEG	NEG	NEG	NEG	AMB	NEG	NEG	NEG	POS	NEG	NEG	NEG	NEG	NEG	NEG	NEG	NEG	NEG	POS	AMB	NEG	POS	NEG	NEG	NEG	NEG	NEG	POS	NEG	NEG	NEG	NEG	NEG	NEG	NEG	NEG	NEG	NEG	NEG	POS	POS	POS	POS	POS	POS	POS	POS	NEG
01V01127	CC45-MRSA-IV, Berlin EMRSA	NEG	POS	NEG	NEG	NEG	NEG	AMB	NEG	NEG	NEG	POS	NEG	NEG	NEG	NEG	NEG	NEG	NEG	NEG	NEG	POS	AMB	NEG	POS	NEG	NEG	NEG	NEG	NEG	POS	NEG	NEG	NEG	NEG	NEG	NEG	NEG	NEG	NEG	NEG	NEG	POS	POS	POS	POS	POS	POS	POS	POS	NEG
01V01445	CC45-MRSA-IV, Berlin EMRSA	AMB	POS	NEG	NEG	NEG	NEG	AMB	NEG	NEG	NEG	POS	NEG	POS	NEG	NEG	NEG	NEG	NEG	NEG	NEG	POS	AMB	NEG	POS	NEG	NEG	NEG	NEG	NEG	POS	NEG	POS	AMB	NEG	NEG	NEG	NEG	NEG	NEG	NEG	NEG	POS	POS	POS	POS	POS	POS	POS	POS	NEG
01V02575	CC45-MRSA-IV, Berlin EMRSA	NEG	POS	NEG	NEG	NEG	NEG	POS	NEG	NEG	NEG	POS	NEG	NEG	NEG	NEG	NEG	NEG	NEG	NEG	NEG	POS	AMB	NEG	POS	NEG	NEG	NEG	NEG	NEG	POS	NEG	NEG	NEG	NEG	NEG	NEG	NEG	NEG	NEG	NEG	NEG	POS	POS	POS	POS	POS	POS	POS	POS	NEG
01V02636	CC45-MRSA-IV, Berlin EMRSA	AMB	POS	NEG	NEG	NEG	NEG	AMB	NEG	NEG	NEG	POS	NEG	NEG	NEG	NEG	NEG	NEG	NEG	NEG	NEG	POS	AMB	NEG	POS	NEG	NEG	NEG	NEG	NEG	POS	NEG	NEG	NEG	NEG	NEG	NEG	NEG	NEG	NEG	NEG	NEG	POS	POS	POS	POS	POS	POS	POS	POS	NEG
01V03483	CC45-MRSA-IV, Berlin EMRSA	NEG	POS	NEG	NEG	NEG	NEG	POS	NEG	NEG	NEG	POS	NEG	NEG	NEG	NEG	NEG	NEG	NEG	NEG	NEG	POS	AMB	NEG	POS	NEG	NEG	NEG	NEG	NEG	POS	NEG	NEG	NEG	NEG	NEG	NEG	NEG	NEG	NEG	NEG	NEG	POS	POS	POS	POS	POS	POS	POS	POS	NEG
01V04728	CC45-MRSA-IV, Berlin EMRSA	AMB	POS	NEG	NEG	NEG	NEG	AMB	NEG	NEG	NEG	POS	NEG	AMB	NEG	NEG	NEG	NEG	NEG	NEG	NEG	POS	AMB	NEG	POS	NEG	NEG	NEG	NEG	NEG	POS	NEG	POS	AMB	NEG	NEG	NEG	NEG	NEG	NEG	NEG	NEG	POS	POS	POS	POS	POS	POS	POS	POS	NEG
01V05972	CC45-MRSA-IV, Berlin EMRSA	NEG	POS	NEG	NEG	NEG	NEG	POS	NEG	NEG	NEG	NEG	NEG	NEG	NEG	NEG	NEG	NEG	NEG	NEG	NEG	AMB	AMB	NEG	POS	NEG	NEG	NEG	NEG	NEG	POS	NEG	POS	NEG	NEG	NEG	NEG	NEG	NEG	NEG	NEG	NEG	POS	AMB	POS	POS	POS	POS	POS	POS	NEG
01V07139	CC45-MRSA-IV, Berlin EMRSA	AMB	POS	NEG	NEG	NEG	NEG	AMB	NEG	NEG	NEG	POS	NEG	NEG	NEG	NEG	NEG	NEG	NEG	NEG	NEG	POS	AMB	NEG	POS	NEG	NEG	NEG	NEG	NEG	POS	NEG	NEG	NEG	NEG	NEG	NEG	NEG	NEG	NEG	NEG	NEG	POS	POS	POS	POS	POS	POS	POS	POS	NEG
01V08133	CC45-MRSA-IV, Berlin EMRSA	NEG	POS	NEG	NEG	NEG	NEG	AMB	NEG	NEG	NEG	POS	NEG	NEG	NEG	NEG	NEG	NEG	NEG	NEG	NEG	POS	AMB	NEG	POS	NEG	NEG	NEG	NEG	NEG	POS	NEG	NEG	NEG	NEG	NEG	NEG	NEG	NEG	NEG	NEG	NEG	POS	POS	POS	POS	POS	POS	POS	POS	NEG
01V09872	CC45-MRSA-IV, Berlin EMRSA	NEG	POS	NEG	NEG	NEG	NEG	AMB	NEG	NEG	NEG	POS	NEG	NEG	NEG	NEG	NEG	NEG	NEG	NEG	NEG	POS	AMB	NEG	POS	NEG	NEG	NEG	NEG	NEG	POS	NEG	NEG	NEG	NEG	NEG	NEG	NEG	NEG	NEG	NEG	NEG	POS	POS	POS	POS	POS	POS	POS	POS	NEG
01V12465	CC45-MRSA-IV, Berlin EMRSA	NEG	POS	NEG	NEG	NEG	NEG	AMB	NEG	NEG	NEG	POS	NEG	NEG	NEG	NEG	NEG	NEG	NEG	NEG	NEG	POS	AMB	NEG	POS	NEG	NEG	NEG	NEG	NEG	POS	NEG	NEG	NEG	NEG	NEG	NEG	NEG	NEG	NEG	NEG	NEG	POS	POS	POS	POS	POS	POS	POS	POS	NEG
01V16151	CC45-MRSA-IV, Berlin EMRSA	NEG	POS	NEG	NEG	NEG	NEG	AMB	NEG	NEG	NEG	POS	NEG	NEG	NEG	NEG	NEG	NEG	NEG	NEG	NEG	POS	AMB	NEG	POS	NEG	NEG	NEG	NEG	NEG	POS	NEG	NEG	NEG	NEG	NEG	NEG	NEG	NEG	NEG	NEG	NEG	POS	POS	POS	POS	POS	POS	POS	POS	NEG
01V16873	CC45-MRSA-IV, Berlin EMRSA	AMB	POS	NEG	NEG	NEG	NEG	AMB	NEG	NEG	NEG	POS	NEG	AMB	NEG	NEG	NEG	NEG	NEG	NEG	NEG	POS	AMB	NEG	POS	NEG	NEG	NEG	NEG	NEG	POS	NEG	AMB	NEG	NEG	NEG	NEG	NEG	NEG	NEG	NEG	NEG	POS	POS	POS	POS	POS	POS	POS	POS	NEG
01V17796	CC45-MRSA-IV, Berlin EMRSA	AMB	POS	NEG	NEG	NEG	NEG	AMB	NEG	NEG	NEG	POS	NEG	NEG	NEG	NEG	NEG	NEG	NEG	NEG	NEG	POS	AMB	NEG	POS	NEG	NEG	NEG	NEG	NEG	POS	NEG	NEG	NEG	NEG	NEG	NEG	NEG	NEG	NEG	NEG	NEG	POS	POS	POS	POS	POS	POS	POS	POS	NEG
01V18681	CC45-MRSA-IV, Berlin EMRSA	AMB	POS	NEG	NEG	NEG	NEG	POS	NEG	NEG	NEG	POS	NEG	AMB	NEG	NEG	NEG	NEG	NEG	NEG	NEG	POS	AMB	NEG	POS	NEG	NEG	NEG	NEG	NEG	POS	NEG	NEG	NEG	NEG	NEG	NEG	NEG	NEG	NEG	NEG	NEG	POS	POS	POS	POS	POS	POS	POS	POS	NEG
01V20066	CC45-MRSA-IV, Berlin EMRSA	AMB	POS	NEG	NEG	NEG	NEG	AMB	NEG	NEG	NEG	POS	NEG	NEG	NEG	NEG	NEG	NEG	NEG	NEG	NEG	POS	AMB	NEG	POS	NEG	NEG	NEG	NEG	NEG	POS	NEG	NEG	NEG	NEG	NEG	NEG	NEG	NEG	NEG	NEG	NEG	POS	POS	POS	POS	POS	POS	POS	POS	NEG
01V23050	CC45-MRSA-IV, Berlin EMRSA	AMB	POS	NEG	NEG	NEG	NEG	AMB	NEG	NEG	NEG	POS	NEG	NEG	NEG	NEG	NEG	NEG	NEG	NEG	NEG	POS	AMB	NEG	POS	NEG	NEG	NEG	NEG	NEG	POS	NEG	NEG	NEG	NEG	NEG	NEG	NEG	NEG	NEG	NEG	NEG	POS	POS	POS	POS	POS	POS	POS	POS	NEG
01V25584	CC45-MRSA-IV, Berlin EMRSA	NEG	POS	NEG	NEG	NEG	NEG	AMB	NEG	NEG	NEG	NEG	NEG	NEG	NEG	NEG	NEG	NEG	NEG	NEG	NEG	AMB	AMB	NEG	POS	NEG	NEG	NEG	NEG	NEG	POS	NEG	NEG	NEG	NEG	NEG	NEG	NEG	NEG	NEG	NEG	NEG	POS	NEG	POS	POS	POS	POS	POS	AMB	NEG
01V31705	CC45-MRSA-IV, Berlin EMRSA	AMB	POS	NEG	NEG	NEG	NEG	POS	NEG	NEG	NEG	POS	NEG	NEG	NEG	NEG	NEG	NEG	NEG	NEG	NEG	POS	POS	NEG	AMB	NEG	NEG	NEG	NEG	NEG	POS	NEG	NEG	NEG	NEG	NEG	NEG	NEG	NEG	NEG	NEG	NEG	POS	POS	POS	POS	POS	POS	POS	POS	NEG
01V34968	CC45-MRSA-IV, Berlin EMRSA	AMB	POS	NEG	NEG	NEG	NEG	POS	NEG	NEG	NEG	POS	NEG	NEG	NEG	NEG	NEG	NEG	NEG	NEG	NEG	POS	POS	NEG	AMB	NEG	NEG	NEG	NEG	NEG	POS	NEG	NEG	NEG	NEG	NEG	NEG	NEG	NEG	NEG	NEG	NEG	POS	POS	POS	POS	POS	POS	POS	POS	NEG
01V36186	CC45-MRSA-IV, Berlin EMRSA	NEG	POS	NEG	NEG	NEG	NEG	POS	NEG	NEG	NEG	POS	NEG	NEG	NEG	NEG	NEG	NEG	NEG	NEG	NEG	POS	AMB	NEG	POS	NEG	NEG	NEG	NEG	NEG	POS	NEG	NEG	NEG	NEG	NEG	NEG	NEG	NEG	NEG	NEG	NEG	POS	AMB	POS	POS	POS	POS	POS	POS	NEG
01V36534	CC45-MRSA-IV, Berlin EMRSA	NEG	POS	NEG	NEG	NEG	NEG	AMB	NEG	NEG	NEG	POS	NEG	NEG	NEG	NEG	NEG	NEG	NEG	NEG	NEG	POS	AMB	NEG	POS	NEG	NEG	NEG	NEG	NEG	POS	NEG	NEG	NEG	NEG	NEG	NEG	NEG	NEG	NEG	NEG	NEG	POS	POS	POS	POS	POS	POS	POS	POS	NEG
01V36588	CC45-MRSA-IV, Berlin EMRSA	AMB	POS	NEG	NEG	NEG	NEG	POS	NEG	NEG	NEG	POS	NEG	NEG	NEG	NEG	NEG	NEG	NEG	NEG	NEG	POS	AMB	NEG	POS	NEG	NEG	NEG	NEG	NEG	POS	NEG	NEG	NEG	NEG	NEG	NEG	NEG	NEG	NEG	NEG	NEG	POS	POS	POS	POS	POS	POS	POS	POS	NEG
01V37990	CC45-MRSA-IV, Berlin EMRSA	AMB	POS	NEG	NEG	NEG	NEG	AMB	NEG	NEG	NEG	POS	NEG	NEG	NEG	NEG	NEG	NEG	NEG	NEG	NEG	POS	POS	NEG	AMB	NEG	NEG	NEG	NEG	NEG	POS	NEG	NEG	NEG	NEG	NEG	NEG	NEG	NEG	NEG	NEG	NEG	POS	POS	POS	POS	POS	POS	POS	POS	NEG
01V39610	CC45-MRSA-IV, Berlin EMRSA	AMB	POS	AMB	POS	NEG	NEG	POS	NEG	POS	NEG	POS	NEG	POS	NEG	NEG	NEG	NEG	NEG	NEG	NEG	POS	POS	NEG	AMB	NEG	NEG	NEG	NEG	NEG	POS	NEG	POS	POS	NEG	NEG	NEG	AMB	NEG	NEG	NEG	NEG	POS	POS	POS	POS	POS	POS	POS	POS	NEG
01V40229	CC45-MRSA-IV, Berlin EMRSA	AMB	POS	NEG	NEG	NEG	NEG	POS	NEG	NEG	NEG	POS	NEG	NEG	NEG	NEG	NEG	NEG	NEG	NEG	NEG	POS	AMB	NEG	POS	NEG	NEG	NEG	NEG	NEG	POS	NEG	NEG	NEG	NEG	NEG	NEG	NEG	NEG	NEG	NEG	NEG	POS	POS	POS	POS	POS	POS	POS	POS	NEG
01V40374	CC45-MRSA-IV, Berlin EMRSA	AMB	POS	NEG		NEG	NEG	POS	NEG			POS	NEG		NEG	NEG	NEG	NEG		NEG		POS	NEG		POS	NEG	NEG	NEG	NEG	NEG	POS	NEG	AMB	NEG																	
01V41056	CC45-MRSA-IV, Berlin EMRSA	NEG	POS	NEG	NEG	NEG	NEG	POS	NEG	NEG	NEG	POS	NEG	NEG	NEG	NEG	NEG	NEG	NEG	NEG	NEG	POS	AMB	NEG	POS	NEG	NEG	NEG	NEG	NEG	POS	NEG	NEG	NEG	NEG	NEG	NEG	NEG	NEG	NEG	NEG	NEG	POS	NEG	POS	POS	POS	POS	POS	POS	NEG
01V42009	CC45-MRSA-IV, Berlin EMRSA	AMB	POS	NEG	NEG	NEG	NEG	POS	NEG	NEG	NEG	POS	NEG	NEG	NEG	NEG	NEG	NEG	NEG	NEG	NEG	POS	AMB	NEG	POS	NEG	NEG	NEG	NEG	NEG	POS	NEG	NEG	NEG	NEG	NEG	NEG	NEG	NEG	NEG	NEG	NEG	POS	POS	POS	POS	POS	POS	POS	POS	NEG
01V43511	CC45-MRSA-IV, Berlin EMRSA	NEG	POS	NEG	NEG	NEG	NEG	POS	NEG	NEG	NEG	AMB	NEG	NEG	NEG	NEG	NEG	NEG	NEG	NEG	NEG	POS	AMB	NEG	POS	NEG	NEG	NEG	NEG	NEG	POS	NEG	NEG	NEG	NEG	NEG	NEG	NEG	NEG	NEG	NEG	NEG	POS	AMB	POS	POS	POS	POS	POS	POS	NEG
**2002**																																																			
02V10698	CC5/ST228-MRSA-I, South German EMRSA	POS	AMB	POS	POS	NEG	POS	NEG	POS	AMB	POS	NEG	NEG	NEG	POS	AMB	AMB	POS	POS	POS	POS	NEG	POS	AMB	AMB	NEG	POS	NEG	NEG	POS	NEG	POS	NEG	POS	NEG	NEG	NEG	NEG	POS	POS	POS	POS	NEG	NEG	NEG	NEG	NEG	POS	POS	POS	NEG
02V13140	CC5/ST228-MRSA-I, South German EMRSA	POS	AMB	POS	POS	NEG	POS	NEG	POS	POS	NEG	NEG	NEG	NEG	POS	AMB	AMB	POS	POS	POS	POS	NEG	POS	AMB	AMB	NEG	POS	NEG	NEG	POS	NEG	POS	NEG	POS	NEG	NEG	NEG	NEG	POS	POS	POS	POS	NEG	NEG	NEG	NEG	NEG	POS	POS	POS	NEG
02V20759	CC5/ST228-MRSA-I, South German EMRSA	POS	NEG	POS	POS	NEG	POS	NEG	POS	POS	NEG	NEG	NEG	NEG	POS	AMB	NEG	POS	POS	POS	POS	NEG	POS	NEG	NEG	NEG	POS	NEG	NEG	POS	NEG	POS	NEG	POS	NEG	NEG	NEG	NEG	POS	POS	POS	POS	NEG	NEG	NEG	NEG	NEG	POS	POS	POS	NEG
02B06271	CC5-MRSA-II, Rhine-Hesse EMRSA/UK-EMRSA-3	POS	AMB	POS	POS	NEG	POS	NEG	POS	AMB	POS	NEG	NEG	NEG	POS	AMB	AMB	POS	POS	POS	POS	NEG	POS	NEG	AMB	NEG	POS	NEG	NEG	POS	NEG	POS	NEG	POS	NEG	NEG	NEG	NEG	POS	POS	POS	POS	NEG	NEG	NEG	NEG	NEG	POS	POS	POS	NEG
02V04116	CC5-MRSA-II, Rhine-Hesse EMRSA/UK-EMRSA-3	POS	AMB	POS	POS	NEG	POS	NEG	POS	AMB	POS	NEG	NEG	NEG	POS	AMB	AMB	POS	POS	POS	POS	NEG	POS	NEG	AMB	NEG	POS	NEG	NEG	POS	NEG	POS	NEG	POS	NEG	NEG	NEG	NEG	POS	POS	POS	POS	NEG	NEG	NEG	NEG	NEG	POS	POS	POS	NEG
02V29922	CC5-MRSA-II, Rhine-Hesse EMRSA/UK-EMRSA-3	POS	AMB	POS		NEG	POS	NEG	POS			NEG	NEG		POS	AMB	AMB	POS		POS		NEG	POS		NEG	NEG	POS	NEG	NEG	POS	NEG	POS	NEG	POS																	
02V33634	CC5-MRSA-II, Rhine-Hesse EMRSA/UK-EMRSA-3	POS	AMB	POS	POS	NEG	POS	NEG	POS	AMB	POS	NEG	NEG	NEG	POS	AMB	AMB	POS	POS	POS	POS	NEG	POS	NEG	AMB	NEG	POS	NEG	NEG	POS	NEG	POS	NEG	POS	NEG	NEG	NEG	NEG	POS	POS	POS	POS	NEG	NEG	NEG	NEG	NEG	POS	POS	POS	NEG
02V34748	CC5-MRSA-II, Rhine-Hesse EMRSA/UK-EMRSA-3	POS	AMB	POS	POS	NEG	POS	NEG	POS	AMB	POS	NEG	NEG	NEG	POS	AMB	AMB	POS	POS	POS	POS	NEG	POS	AMB	AMB	NEG	POS	NEG	NEG	POS	NEG	POS	NEG	POS	NEG	NEG	NEG	NEG	POS	POS	POS	POS	NEG	NEG	NEG	NEG	NEG	POS	POS	POS	NEG
02V17002	CC8/ST254-MRSA-(atypical SCCmec), Hannover EMRSA	POS	NEG	POS	POS	NEG	POS	NEG	POS	AMB	POS	NEG	POS	POS	POS	AMB	NEG	POS	POS	POS	POS	NEG	POS	NEG	NEG	POS	NEG	NEG	NEG	POS	NEG	POS	NEG	POS	NEG	NEG	NEG	NEG	POS	POS	POS	POS	NEG	NEG	NEG	NEG	NEG	POS	POS	POS	NEG
02V33579	CC8-MRSA-IV, USA500	POS	NEG	POS	POS	NEG	POS	NEG	POS	AMB	POS	NEG	NEG	POS	POS	AMB	NEG	POS	POS	POS	POS	NEG	POS	AMB	NEG	POS	NEG	NEG	NEG	POS	NEG	POS	NEG	POS	NEG	NEG	NEG	NEG	POS	POS	POS	POS	NEG	NEG	NEG	NEG	NEG	POS	POS	POS	NEG
02V15498	CC22-MRSA-IV [PVL+]	AMB	POS	NEG	NEG	NEG	NEG	POS	POS	NEG	NEG	POS	NEG	NEG	AMB	AMB	POS	NEG	NEG	POS	POS	NEG	NEG	NEG	NEG	NEG	NEG	NEG	NEG	NEG	NEG	NEG	NEG	NEG	NEG	NEG	NEG	NEG	POS	POS	POS	POS	NEG	NEG	NEG	NEG	NEG	POS	POS	POS	NEG
02C05319	CC22-MRSA-IV, Barnim/UK-EMRSA-15	AMB	POS	NEG	NEG	AMB	NEG	AMB	POS	NEG	NEG	POS	NEG	NEG	AMB	AMB	POS	NEG	NEG	POS	POS	NEG	POS	NEG	NEG	NEG	NEG	NEG	NEG	NEG	NEG	NEG	NEG	NEG	NEG	NEG	NEG	NEG	POS	POS	POS	POS	NEG	NEG	NEG	NEG	NEG	POS	POS	POS	NEG
02U10255	CC22-MRSA-IV, Barnim/UK-EMRSA-15	AMB	POS	NEG	NEG	NEG	NEG	AMB	POS	NEG	NEG	POS	NEG	NEG	AMB	AMB	POS	NEG	NEG	POS	POS	NEG	POS	NEG	NEG	NEG	NEG	NEG	NEG	NEG	NEG	NEG	NEG	NEG	NEG	NEG	NEG	NEG	POS	POS	POS	POS	NEG	NEG	NEG	NEG	NEG	POS	POS	POS	NEG
02V12175	CC22-MRSA-IV, Barnim/UK-EMRSA-15	AMB	POS	NEG	NEG	NEG	NEG	AMB	POS	NEG	NEG	POS	NEG	NEG	AMB	AMB	POS	NEG	NEG	POS	POS	NEG	POS	NEG	AMB	NEG	NEG	NEG	NEG	NEG	NEG	NEG	NEG	NEG	NEG	NEG	NEG	NEG	POS	POS	POS	POS	NEG	NEG	NEG	NEG	NEG	POS	POS	POS	NEG
02V13054	CC22-MRSA-IV, Barnim/UK-EMRSA-15	POS	AMB	NEG	NEG	AMB	NEG	AMB	POS	NEG	NEG	POS	NEG	NEG	AMB	AMB	POS	NEG	NEG	POS	POS	NEG	POS	NEG	NEG	NEG	NEG	NEG	NEG	NEG	NEG	NEG	NEG	NEG	NEG	NEG	NEG	NEG	POS	POS	POS	POS	NEG	NEG	NEG	NEG	NEG	POS	POS	POS	NEG
02V13950	CC22-MRSA-IV, Barnim/UK-EMRSA-15	AMB	POS	NEG	NEG	NEG	NEG	AMB	POS	NEG	NEG	POS	NEG	AMB	AMB	AMB	POS	NEG	NEG	POS	POS	NEG	POS	NEG	NEG	NEG	NEG	NEG	NEG	NEG	NEG	NEG	NEG	NEG	NEG	NEG	NEG	NEG	POS	POS	POS	POS	NEG	NEG	NEG	NEG	NEG	POS	POS	POS	NEG
02V21259	CC22-MRSA-IV, Barnim/UK-EMRSA-15	AMB	POS	NEG	NEG	NEG	NEG	POS	POS	NEG	NEG	POS	NEG	NEG	AMB	AMB	POS	NEG	NEG	POS	POS	NEG	NEG	NEG	NEG	NEG	NEG	NEG	NEG	NEG	NEG	NEG	NEG	NEG	NEG	NEG	NEG	NEG	POS	POS	POS	POS	NEG	NEG	NEG	NEG	NEG	POS	POS	POS	NEG
02V22657	CC22-MRSA-IV, Barnim/UK-EMRSA-15	AMB	POS	NEG	NEG	NEG	NEG	POS	POS	NEG	NEG	POS	NEG	NEG	NEG	AMB	POS	NEG	NEG	POS	POS	NEG	NEG	NEG	NEG	NEG	NEG	NEG	NEG	NEG	NEG	NEG	NEG	NEG	NEG	NEG	NEG	NEG	POS	POS	POS	POS	NEG	NEG	NEG	NEG	NEG	POS	POS	POS	NEG
02V29051	CC22-MRSA-IV, Barnim/UK-EMRSA-15	AMB	POS	NEG	NEG	AMB	NEG	AMB	POS	NEG	NEG	POS	NEG	NEG	AMB	AMB	POS	NEG	NEG	POS	POS	NEG	POS	NEG	NEG	NEG	NEG	NEG	NEG	NEG	NEG	NEG	NEG	NEG	NEG	NEG	NEG	NEG	POS	POS	POS	POS	NEG	NEG	NEG	NEG	NEG	POS	POS	POS	NEG
02V30792	CC30/ST36-MRSA-II, UK-EMRSA-16	AMB	POS	NEG	NEG	POS	NEG	POS	NEG	NEG	NEG	POS	NEG	AMB	NEG	POS	AMB	NEG	NEG	NEG	NEG	POS	AMB	NEG	POS	NEG	NEG	NEG	POS	NEG	POS	NEG	POS	POS	NEG	NEG	NEG	NEG	NEG	NEG	NEG	NEG	POS	POS	POS	POS	POS	POS	POS	POS	NEG
02U07096	CC45-MRSA-IV, Berlin EMRSA	AMB	POS	NEG	NEG	NEG	NEG	AMB	NEG	NEG	NEG	POS	NEG	NEG	NEG	NEG	NEG	NEG	NEG	NEG	NEG	POS	AMB	NEG	POS	NEG	NEG	NEG	NEG	NEG	POS	NEG	NEG	NEG	NEG	NEG	NEG	NEG	NEG	NEG	NEG	NEG	POS	POS	POS	POS	POS	POS	POS	POS	NEG
02V00570	CC45-MRSA-IV, Berlin EMRSA	AMB	POS	NEG	NEG	NEG	NEG	POS	NEG	NEG	NEG	POS	NEG	NEG	NEG	NEG	NEG	NEG	NEG	NEG	NEG	POS	AMB	NEG	POS	NEG	NEG	NEG	NEG	NEG	POS	NEG	NEG	NEG	NEG	NEG	NEG	NEG	NEG	NEG	NEG	NEG	POS	POS	POS	POS	POS	POS	POS	POS	NEG
02V01730	CC45-MRSA-IV, Berlin EMRSA	NEG	POS	NEG	NEG	NEG	NEG	POS	NEG	NEG	NEG	POS	NEG	NEG	NEG	NEG	NEG	NEG	NEG	NEG	NEG	POS	AMB	NEG	POS	NEG	NEG	NEG	NEG	NEG	POS	NEG	NEG	NEG	NEG	NEG	NEG	NEG	NEG	NEG	NEG	NEG	POS	POS	POS	POS	POS	POS	POS	POS	NEG
02V01870	CC45-MRSA-IV, Berlin EMRSA	AMB	POS	NEG	NEG	NEG	NEG	AMB	NEG	NEG	NEG	POS	NEG	NEG	NEG	NEG	NEG	NEG	NEG	NEG	NEG	POS	AMB	NEG	POS	NEG	NEG	NEG	NEG	NEG	POS	NEG	NEG	NEG	NEG	NEG	NEG	NEG	NEG	NEG	NEG	NEG	POS	POS	POS	POS	POS	POS	POS	POS	NEG
02V04750	CC45-MRSA-IV, Berlin EMRSA	AMB	POS	NEG	NEG	NEG	NEG	AMB	NEG	NEG	NEG	POS	NEG	AMB	NEG	NEG	NEG	NEG	NEG	NEG	NEG	POS	AMB	NEG	POS	NEG	NEG	NEG	NEG	NEG	POS	NEG	AMB	NEG	NEG	NEG	NEG	NEG	NEG	NEG	NEG	NEG	POS	POS	POS	POS	POS	POS	POS	POS	NEG
02V06592	CC45-MRSA-IV, Berlin EMRSA	AMB	POS	NEG	NEG	NEG	NEG	AMB	NEG	NEG	NEG	POS	NEG	AMB	NEG	NEG	NEG	NEG	NEG	NEG	NEG	POS	AMB	NEG	POS	NEG	NEG	NEG	NEG	NEG	POS	NEG	NEG	NEG	NEG	NEG	NEG	NEG	NEG	NEG	NEG	NEG	POS	POS	POS	POS	POS	POS	POS	POS	NEG
02V10868	CC45-MRSA-IV, Berlin EMRSA	AMB	POS	NEG	NEG	NEG	NEG	AMB	NEG	NEG	NEG	POS	NEG	NEG	NEG	NEG	NEG	NEG	NEG	NEG	NEG	POS	AMB	NEG	POS	NEG	NEG	NEG	NEG	NEG	POS	NEG	AMB	NEG	NEG	NEG	NEG	NEG	NEG	NEG	NEG	NEG	POS	POS	POS	POS	POS	POS	POS	POS	NEG
02V12394	CC45-MRSA-IV, Berlin EMRSA	AMB	POS	NEG	NEG	NEG	NEG	AMB	NEG	NEG	NEG	POS	NEG	NEG	NEG	NEG	NEG	NEG	NEG	NEG	NEG	POS	AMB	NEG	POS	NEG	NEG	NEG	NEG	NEG	POS	NEG	AMB	NEG	NEG	NEG	NEG	NEG	NEG	NEG	NEG	NEG	POS	POS	POS	POS	POS	POS	POS	POS	NEG
02V12942	CC45-MRSA-IV, Berlin EMRSA	AMB	POS	NEG	NEG	NEG	NEG	AMB	NEG	NEG	NEG	POS	NEG	NEG	NEG	NEG	NEG	NEG	NEG	NEG	NEG	POS	AMB	NEG	POS	NEG	NEG	NEG	NEG	NEG	POS	NEG	AMB	NEG	NEG	NEG	NEG	NEG	NEG	NEG	NEG	NEG	POS	POS	POS	POS	POS	POS	POS	POS	NEG
02V13026	CC45-MRSA-IV, Berlin EMRSA	AMB	POS	NEG	NEG	NEG	NEG	AMB	NEG	NEG	NEG	AMB	NEG	AMB	NEG	NEG	NEG	NEG	NEG	NEG	NEG	POS	AMB	NEG	POS	NEG	NEG	NEG	NEG	NEG	POS	NEG	AMB	NEG	NEG	NEG	NEG	NEG	NEG	NEG	NEG	NEG	POS	POS	POS	POS	POS	POS	POS	POS	NEG
02V14700	CC45-MRSA-IV, Berlin EMRSA	NEG	POS	NEG		NEG	NEG	NEG	NEG			POS	NEG		NEG	NEG	NEG	NEG		NEG		POS	NEG		POS	NEG	NEG	NEG	NEG	NEG	POS	NEG	NEG	NEG																	
02V15358	CC45-MRSA-IV, Berlin EMRSA	AMB	POS	NEG	NEG	NEG	NEG	AMB	NEG	NEG	NEG	POS	NEG	NEG	NEG	NEG	NEG	NEG	NEG	NEG	NEG	POS	AMB	NEG	POS	NEG	NEG	NEG	NEG	NEG	POS	NEG	AMB	NEG	NEG	NEG	NEG	NEG	NEG	NEG	NEG	NEG	POS	POS	POS	POS	POS	POS	POS	POS	NEG
02V16654	CC45-MRSA-IV, Berlin EMRSA	AMB	POS	NEG	NEG	NEG	NEG	AMB	NEG	NEG	NEG	POS	NEG	NEG	NEG	NEG	NEG	NEG	NEG	NEG	NEG	POS	AMB	NEG	POS	NEG	NEG	NEG	NEG	NEG	POS	NEG	NEG	NEG	NEG	NEG	NEG	NEG	NEG	NEG	NEG	NEG	POS	POS	POS	POS	POS	POS	POS	POS	NEG
02V17120	CC45-MRSA-IV, Berlin EMRSA	AMB	POS	NEG	NEG	NEG	NEG	POS	NEG	NEG	NEG	POS	NEG	POS	NEG	NEG	NEG	NEG	NEG	NEG	NEG	POS	AMB	NEG	POS	NEG	NEG	NEG	NEG	NEG	POS	NEG	POS	AMB	NEG	NEG	NEG	NEG	NEG	NEG	NEG	NEG	POS	POS	POS	POS	POS	POS	POS	POS	NEG
02V17136	CC45-MRSA-IV, Berlin EMRSA	AMB	POS	NEG	NEG	NEG	NEG	AMB	NEG	NEG	NEG	POS	NEG	NEG	NEG	NEG	NEG	NEG	NEG	NEG	NEG	POS	AMB	NEG	POS	NEG	NEG	NEG	NEG	NEG	POS	NEG	AMB	NEG	NEG	NEG	NEG	NEG	NEG	NEG	NEG	NEG	POS	POS	POS	POS	POS	POS	POS	POS	NEG
02V17558	CC45-MRSA-IV, Berlin EMRSA	AMB	POS	NEG	NEG	NEG	NEG	AMB	NEG	NEG	NEG	POS	NEG	NEG	NEG	NEG	NEG	NEG	NEG	NEG	NEG	POS	AMB	NEG	POS	NEG	NEG	NEG	NEG	NEG	POS	NEG	NEG	NEG	NEG	NEG	NEG	NEG	NEG	NEG	NEG	NEG	POS	POS	POS	POS	POS	POS	POS	POS	NEG
02V17665	CC45-MRSA-IV, Berlin EMRSA	NEG	POS	NEG	NEG	NEG	NEG	POS	NEG	NEG	NEG	POS	NEG	NEG	NEG	NEG	NEG	NEG	NEG	NEG	NEG	POS	AMB	NEG	POS	NEG	NEG	NEG	NEG	NEG	POS	NEG	NEG	NEG	NEG	NEG	NEG	NEG	NEG	NEG	NEG	NEG	POS	AMB	POS	POS	POS	POS	POS	POS	NEG
02V20390	CC45-MRSA-IV, Berlin EMRSA	NEG	POS	NEG	NEG	NEG	NEG	POS	NEG	NEG	NEG	POS	NEG	NEG	NEG	NEG	NEG	NEG	NEG	NEG	NEG	POS	AMB	NEG	POS	NEG	NEG	NEG	NEG	NEG	POS	NEG	NEG	NEG	NEG	NEG	NEG	NEG	NEG	NEG	NEG	NEG	POS	POS	POS	POS	POS	POS	POS	POS	NEG
02V20626	CC45-MRSA-IV, Berlin EMRSA	AMB	POS	NEG	NEG	NEG	NEG	POS	NEG	NEG	NEG	POS	NEG	NEG	NEG	NEG	NEG	NEG	NEG	NEG	NEG	POS	AMB	NEG	POS	NEG	NEG	NEG	NEG	NEG	POS	NEG	NEG	NEG	NEG	NEG	NEG	NEG	NEG	NEG	NEG	NEG	POS	POS	POS	POS	POS	POS	POS	POS	NEG
02V20680	CC45-MRSA-IV, Berlin EMRSA	NEG	POS	NEG	NEG	NEG	NEG	POS	NEG	NEG	NEG	POS	NEG	NEG	NEG	NEG	NEG	NEG	NEG	NEG	NEG	POS	AMB	NEG	POS	NEG	NEG	NEG	NEG	NEG	POS	NEG	NEG	NEG	NEG	NEG	NEG	NEG	NEG	NEG	NEG	NEG	POS	AMB	POS	POS	POS	POS	POS	POS	NEG
02V21980	CC45-MRSA-IV, Berlin EMRSA	NEG	POS	NEG	NEG	NEG	NEG	POS	NEG	NEG	NEG	POS	NEG	NEG	NEG	NEG	NEG	NEG	NEG	NEG	NEG	POS	AMB	NEG	POS	NEG	NEG	NEG	NEG	NEG	POS	NEG	NEG	NEG	NEG	NEG	NEG	NEG	NEG	NEG	NEG	NEG	POS	AMB	POS	POS	POS	POS	POS	POS	NEG
02V22570	CC45-MRSA-IV, Berlin EMRSA	AMB	POS	NEG	NEG	NEG	NEG	AMB	NEG	NEG	NEG	POS	NEG	AMB	NEG	NEG	NEG	NEG	NEG	NEG	NEG	POS	AMB	NEG	POS	NEG	NEG	NEG	NEG	NEG	POS	NEG	AMB	NEG	NEG	NEG	NEG	NEG	NEG	NEG	NEG	NEG	POS	POS	POS	POS	POS	POS	POS	POS	NEG
02V22982	CC45-MRSA-IV, Berlin EMRSA	AMB	POS	NEG	NEG	NEG	NEG	POS	NEG	NEG	NEG	POS	NEG	AMB	NEG	NEG	NEG	NEG	NEG	NEG	NEG	POS	AMB	NEG	POS	NEG	NEG	NEG	NEG	NEG	POS	NEG	AMB	NEG	NEG	NEG	NEG	NEG	NEG	NEG	NEG	NEG	POS	POS	POS	POS	POS	POS	POS	POS	NEG
02V25503	CC45-MRSA-IV, Berlin EMRSA	AMB	POS	NEG	NEG	NEG	NEG	AMB	NEG	NEG	NEG	POS	NEG	AMB	NEG	NEG	NEG	NEG	NEG	NEG	NEG	POS	AMB	NEG	POS	NEG	NEG	NEG	NEG	NEG	POS	NEG	NEG	NEG	NEG	NEG	NEG	NEG	NEG	NEG	NEG	NEG	POS	POS	POS	POS	POS	POS	POS	POS	NEG
02V28454	CC45-MRSA-IV, Berlin EMRSA	AMB	POS	NEG	NEG	NEG	NEG	AMB	NEG	NEG	NEG	POS	NEG	AMB	NEG	NEG	NEG	NEG	NEG	NEG	NEG	POS	AMB	NEG	POS	NEG	NEG	NEG	NEG	NEG	POS	NEG	AMB	NEG	NEG	NEG	NEG	NEG	NEG	NEG	NEG	NEG	POS	POS	POS	POS	POS	POS	POS	POS	NEG
02V29373	CC45-MRSA-IV, Berlin EMRSA	AMB	POS	NEG	NEG	NEG	NEG	AMB	NEG	NEG	NEG	POS	NEG	AMB	NEG	NEG	NEG	NEG	NEG	NEG	NEG	POS	AMB	NEG	POS	NEG	NEG	NEG	NEG	NEG	POS	NEG	AMB	NEG	NEG	NEG	NEG	NEG	NEG	NEG	NEG	NEG	POS	POS	POS	POS	POS	POS	POS	POS	NEG
02V30321	CC45-MRSA-IV, Berlin EMRSA	AMB	POS	NEG	NEG	NEG	NEG	AMB	NEG	NEG	NEG	POS	NEG	POS	NEG	NEG	NEG	NEG	NEG	NEG	NEG	POS	AMB	NEG	POS	NEG	NEG	NEG	NEG	NEG	POS	NEG	AMB	NEG	NEG	NEG	NEG	NEG	NEG	NEG	NEG	NEG	POS	POS	POS	POS	POS	POS	POS	POS	NEG
02V30341	CC45-MRSA-IV, Berlin EMRSA	NEG	POS	NEG	NEG	NEG	NEG	AMB	NEG	NEG	NEG	POS	NEG	NEG	NEG	NEG	NEG	NEG	NEG	NEG	NEG	POS	AMB	NEG	POS	NEG	NEG	NEG	NEG	NEG	POS	NEG	NEG	NEG	NEG	NEG	NEG	NEG	NEG	NEG	NEG	NEG	POS	POS	POS	POS	POS	POS	POS	POS	NEG
02V31377	CC45-MRSA-IV, Berlin EMRSA	AMB	POS	NEG	NEG	NEG	NEG	AMB	NEG	NEG	NEG	POS	NEG	NEG	NEG	NEG	NEG	NEG	NEG	NEG	NEG	POS	AMB	NEG	POS	NEG	NEG	NEG	NEG	NEG	POS	NEG	NEG	NEG	NEG	NEG	NEG	NEG	NEG	NEG	NEG	NEG	POS	POS	POS	POS	POS	POS	POS	POS	NEG
02V31941	CC45-MRSA-IV, Berlin EMRSA	NEG	POS	NEG	NEG	NEG	NEG	AMB	NEG	NEG	NEG	POS	NEG	AMB	NEG	NEG	NEG	NEG	NEG	NEG	NEG	POS	NEG	NEG	NEG	NEG	NEG	NEG	NEG	NEG	POS	NEG	AMB	NEG	NEG	NEG	NEG	NEG	NEG	NEG	NEG	NEG	POS	POS	POS	POS	POS	POS	POS	POS	NEG
02V34679	CC45-MRSA-IV, Berlin EMRSA	NEG	POS	NEG	NEG	NEG	NEG	AMB	NEG	NEG	NEG	POS	NEG	NEG	NEG	NEG	NEG	NEG	NEG	NEG	NEG	POS	AMB	NEG	POS	NEG	NEG	NEG	NEG	NEG	POS	NEG	NEG	NEG	NEG	NEG	NEG	NEG	NEG	NEG	NEG	NEG	POS	AMB	POS	POS	POS	POS	POS	POS	NEG
02V35779	CC45-MRSA-IV, Berlin EMRSA	NEG	POS	NEG	NEG	NEG	NEG	NEG	NEG	NEG	NEG	AMB	NEG	NEG	NEG	NEG	NEG	NEG	NEG	NEG	NEG	POS	NEG	NEG	POS	NEG	NEG	NEG	NEG	NEG	POS	NEG	NEG	NEG	NEG	NEG	NEG	NEG	NEG	NEG	NEG	NEG	POS	POS	POS	POS	POS	POS	POS	POS	NEG
02V36419	CC45-MRSA-IV, Berlin EMRSA	NEG	POS	NEG		NEG	NEG	NEG	NEG			AMB	NEG		NEG	NEG	NEG	NEG		NEG		POS	NEG		POS	NEG	NEG	NEG	NEG	NEG	POS	NEG	NEG	NEG																	
02V36469	CC45-MRSA-IV, Berlin EMRSA	AMB	POS	NEG	NEG	NEG	NEG	POS	NEG	NEG	NEG	POS	NEG	POS	NEG	NEG	NEG	NEG	NEG	NEG	NEG	POS	AMB	NEG	POS	NEG	NEG	NEG	NEG	NEG	POS	NEG	POS	AMB	NEG	NEG	NEG	NEG	NEG	NEG	NEG	NEG	POS	POS	POS	POS	POS	POS	POS	POS	NEG

ISOLATE:	STRAIN	ADHAESION FACTORS / GENES ENCODING MICROBIAL SURFACE COMPONENTS RECOGNIZING ADHESIVE MATRIX MOLECULES (MSCRAMM GENES)																																															
		bone sialoprotein-binding protein							clumping factor A					clumping factor B					collagen-binding adhesin	cell wall associated fibronectin-binding protein	cell surface elastin binding protein					enolase	fibrinogen binding protein (19 kDa)		fibronectin-binding protein A						fibronectin-binding protein B							Major histocompatibility complex class II analog protein (=Extracellular adherence protein, eap)				Staphylococcus aureus surface protein G			
		bbp (total)	bbp (consensus)	bbp (COL+MW2)	bbp (MRSA252)	bbp (Mu50)	bbp (RF122)	bbp (ST45)	clfA (total)	clfA (consensus)	clfA (COL+RF122)	clfA (MRSA252)	clfA (Mu50+MW2)	clfB (total)	clfB (consensus)	clfB (COL+Mu50)	clfB (MW2)	clfB (RF122)	cna	ebh (consensus)	ebpS (total)	ebpS_probe 612	ebpS_probe 614	ebpS (01-1111)	ebpS (COL)	eno	fib	fib (MRSA252)	fnbA (total)	fnbA (consensus)	fnbA (COL)	fnbA (MRSA252)	fnbA (Mu50+MW2)	fnbA (RF122)	fnbB (total)	fnbB (COL)	fnbB (COL+Mu50+MW2)	fnbB (Mu50)	fnbB (MW2)	fnbB (ST15)	fnbB (ST45-2)	map (total)	map (COL)	map (MRSA252)	map (Mu50+MW2)	sasG (total)	sasG (COL+Mu50)	sasG (MW2)	sasG (other than MRSA252+RF122)
**2000**																																																	
00V07201	CC5/ST228-MRSA, South German EMRSA (truncated SCCmec element)	POS	POS	NEG	NEG	POS	NEG	NEG	POS	POS	NEG	NEG	POS	POS	POS	POS	NEG	NEG	NEG	POS	POS	POS	POS	NEG	NEG	POS	POS	NEG	POS	POS	NEG	NEG	POS	NEG	NEG	NEG	NEG	NEG	NEG	NEG	NEG	POS	POS	NEG	POS	POS	POS	NEG	POS
00V07634	CC5/ST228-MRSA, South German EMRSA (truncated SCCmec element)	POS	POS	AMB	NEG	POS	NEG	NEG	POS	POS	NEG	NEG	POS	POS	POS	POS	NEG	NEG	NEG	POS	POS	POS	POS	NEG	NEG	POS	POS	NEG	POS	POS	NEG	NEG	POS	NEG	NEG	NEG	NEG	NEG	NEG	NEG	NEG	POS	POS	NEG	POS	POS	POS	NEG	POS
00V08701	CC5/ST228-MRSA, South German EMRSA (truncated SCCmec element)	POS	POS	NEG	NEG	POS	NEG	NEG	POS	POS	NEG	NEG	POS	POS	POS	POS	NEG	NEG	NEG	POS	POS	POS	POS	NEG	NEG	POS	POS	NEG	POS	POS	NEG	NEG	POS	NEG	NEG	NEG	NEG	NEG	NEG	NEG	NEG	POS	AMB	NEG	POS	POS	POS	NEG	POS
00V09056	CC5/ST228-MRSA, South German EMRSA (truncated SCCmec element)	POS	POS	NEG	NEG	POS	NEG	NEG	POS	POS	NEG	NEG	POS	POS	POS	POS	NEG	NEG	NEG	POS	POS	POS	POS	NEG	NEG	POS	POS	NEG	POS	POS	NEG	NEG	POS	NEG	NEG	NEG	NEG	NEG	NEG	NEG	NEG	POS	POS	NEG	POS	POS	POS	NEG	POS
00V10190	CC5/ST228-MRSA, South German EMRSA (truncated SCCmec element)	POS	POS	AMB	NEG	POS	NEG	AMB	POS	POS	NEG	NEG	POS	POS	POS	POS	NEG	NEG	NEG	POS	POS	POS	POS	NEG	AMB	POS	POS	NEG	POS	POS	NEG	NEG	POS	NEG	NEG	NEG	NEG	NEG	NEG	NEG	NEG	POS	POS	AMB	POS	POS	POS	NEG	POS
00V10643	CC5/ST228-MRSA, South German EMRSA (truncated SCCmec element)	POS	POS	NEG	NEG	POS	NEG	NEG	POS	POS	NEG	NEG	POS	POS	POS	POS	NEG	NEG	NEG	POS	POS	POS	POS	NEG	NEG	POS	POS	NEG	POS	POS	NEG	NEG	POS	NEG	NEG	NEG	NEG	NEG	NEG	NEG	NEG	POS	POS	NEG	POS	POS	POS	NEG	POS
00V11732	CC5/ST228-MRSA, South German EMRSA (truncated SCCmec element)	POS	POS	NEG	NEG	POS	NEG	NEG	POS	POS	NEG	NEG	POS	POS	POS	POS	NEG	NEG	NEG	POS	POS	POS	POS	NEG	NEG	POS	POS	NEG	POS	POS	NEG	NEG	POS	NEG	NEG	NEG	NEG	NEG	NEG	NEG	NEG	POS	AMB	NEG	POS	POS	POS	NEG	POS
00V12300	CC5/ST228-MRSA, South German EMRSA (truncated SCCmec element)	POS	POS	NEG	NEG	POS	NEG	NEG	POS	POS	NEG	NEG	POS	POS	POS	POS	NEG	NEG	NEG	POS	POS	POS	POS	NEG	NEG	POS	POS	NEG	POS	POS	NEG	NEG	POS	NEG	NEG	NEG	NEG	NEG	NEG	NEG	NEG	POS	AMB	NEG	POS	POS	POS	NEG	POS
00V14652	CC5/ST228-MRSA, South German EMRSA (truncated SCCmec element)	POS	POS	NEG	NEG	POS	NEG	NEG	POS	POS	NEG	NEG	POS	POS	POS	POS	NEG	NEG	NEG	POS	POS	POS	POS	NEG	NEG	POS	POS	NEG	POS	POS	NEG	NEG	POS	NEG	NEG	NEG	NEG	NEG	NEG	NEG	NEG	POS	POS	NEG	POS	POS	POS	NEG	POS
00V24941	CC5/ST228-MRSA, South German EMRSA (truncated SCCmec element)	POS	POS	NEG	NEG	POS	NEG	NEG	POS	POS	NEG	NEG	POS	POS	POS	POS	NEG	NEG	NEG	POS	POS	POS	POS	NEG	NEG	POS	POS	NEG	POS	POS	NEG	NEG	POS	NEG	NEG	NEG	NEG	NEG	NEG	NEG	NEG	POS	POS	NEG	POS	POS	POS	NEG	POS
00V29834	CC5/ST228-MRSA, South German EMRSA (truncated SCCmec element)	POS	POS	NEG	NEG	POS	NEG	NEG	POS	POS	NEG	NEG	POS	POS	POS	POS	NEG	NEG	NEG	POS	POS	POS	POS	NEG	NEG	POS	POS	NEG	POS	POS	NEG	NEG	POS	NEG	NEG	NEG	NEG	NEG	NEG	NEG	NEG	POS	NEG	NEG	POS	POS	POS	NEG	POS
00V29909	CC5/ST228-MRSA, South German EMRSA (truncated SCCmec element)	POS	POS	NEG	NEG	POS	NEG	NEG	POS	POS	NEG	NEG	POS	POS	POS	POS	NEG	NEG	NEG	POS	POS	POS	POS	NEG	NEG	POS	POS	NEG	POS	POS	NEG	NEG	POS	NEG	NEG	NEG	NEG	NEG	NEG	NEG	NEG	POS	NEG	NEG	POS	POS	POS	NEG	POS
00V30456	CC5/ST228-MRSA, South German EMRSA (truncated SCCmec element)	POS	POS	NEG	NEG	POS	NEG	NEG	POS	POS	NEG	NEG	POS	POS	POS	POS	NEG	NEG	NEG	POS	POS	POS	POS	NEG	NEG	POS	POS	NEG	POS	POS	NEG	NEG	POS	NEG	NEG	NEG	NEG	NEG	NEG	NEG	NEG	POS	NEG	NEG	POS	POS	POS	NEG	POS
00V30827	CC5/ST228-MRSA, South German EMRSA (truncated SCCmec element)	POS	POS	NEG	NEG	POS	NEG	NEG	POS	POS	NEG	NEG	POS	POS	POS	POS	NEG	NEG	NEG	POS	POS	POS	POS	NEG	NEG	POS	POS	NEG	POS	POS	NEG	NEG	POS	NEG	NEG	NEG	NEG	NEG	NEG	NEG	NEG	POS	POS	NEG	POS	POS	POS	NEG	POS
00V33204	CC5/ST228-MRSA, South German EMRSA (truncated SCCmec element)	POS	POS	NEG	NEG	POS	NEG	NEG	POS	POS	NEG	NEG	POS	POS	POS	POS	NEG	NEG	NEG	POS	POS	POS	POS	NEG	NEG	POS	POS	NEG	POS	POS	NEG	NEG	POS	NEG	NEG	NEG	NEG	NEG	NEG	NEG	NEG	POS	AMB	NEG	POS	POS	POS	NEG	POS
00V36490	CC5/ST228-MRSA, South German EMRSA (truncated SCCmec element)	POS	POS	NEG	NEG	POS	NEG	NEG	POS	POS	NEG	NEG	POS	POS	POS	POS	NEG	NEG	NEG	POS	POS	POS	POS	NEG	NEG	POS	POS	NEG	POS	POS	NEG	NEG	POS	NEG	NEG	NEG	NEG	NEG	NEG	NEG	NEG	POS	AMB	NEG	POS	POS	POS	NEG	POS
00V37079	CC5/ST228-MRSA, South German EMRSA (truncated SCCmec element)	POS	POS	NEG	NEG	POS	NEG	NEG	POS	POS	NEG	NEG	POS	POS	POS	POS	NEG	NEG	NEG	POS	POS	POS	POS	NEG	NEG	POS	POS	NEG	POS	POS	NEG	NEG	POS	NEG	NEG	NEG	NEG	NEG	NEG	NEG	NEG	POS	POS	NEG	POS	POS	POS	NEG	POS
00V40116	CC5/ST228-MRSA, South German EMRSA (truncated SCCmec element)	POS	POS	AMB	NEG	POS	NEG	NEG	POS	POS	NEG	NEG	POS	POS	POS	POS	NEG	NEG	NEG	POS	POS	POS	POS	NEG	NEG	POS	POS	NEG	POS	POS	NEG	NEG	POS	NEG	NEG	NEG	NEG	NEG	NEG	NEG	NEG	POS	POS	NEG	POS	POS	POS	NEG	POS
00V41429	CC5/ST228-MRSA, South German EMRSA (truncated SCCmec element)	POS	POS	NEG	NEG	POS	NEG	NEG	POS	POS	NEG	NEG	POS	POS	POS	POS	NEG	NEG	NEG	POS	POS	POS	POS	NEG	NEG	POS	POS	NEG	POS	POS	NEG	NEG	POS	NEG	NEG	NEG	NEG	NEG	NEG	NEG	NEG	POS	POS	NEG	POS	POS	POS	NEG	POS
00V42118	CC5/ST228-MRSA, South German EMRSA (truncated SCCmec element)	POS	POS	AMB	NEG	POS	NEG	NEG	POS	POS	NEG	NEG	POS	POS	POS	POS	NEG	NEG	NEG	POS	POS	POS	POS	NEG	NEG	POS	POS	NEG	POS	POS	NEG	NEG	POS	NEG	NEG	NEG	NEG	NEG	NEG	NEG	NEG	POS	POS	NEG	POS	POS	POS	NEG	POS
00V44135	CC5/ST228-MRSA, South German EMRSA (truncated SCCmec element)	POS	POS	NEG	NEG	POS	NEG	NEG	POS	POS	NEG	NEG	POS	POS	POS	POS	NEG	NEG	NEG	POS	POS	POS	POS	NEG	NEG	POS	POS	NEG	POS	POS	NEG	NEG	POS	NEG	NEG	NEG	NEG	NEG	NEG	NEG	NEG	POS	POS	NEG	POS	POS	POS	NEG	POS
00V44538	CC5/ST228-MRSA, South German EMRSA (truncated SCCmec element)	POS	POS	NEG	NEG	POS	NEG	NEG	POS	POS	NEG	NEG	POS	POS	POS	POS	NEG	NEG	NEG	POS	POS	POS	POS	NEG	NEG	POS	POS	NEG	POS	POS	NEG	NEG	POS	NEG	NEG	NEG	NEG	NEG	NEG	NEG	NEG	POS	POS	NEG	POS	POS	POS	NEG	POS
00V44990	CC5/ST228-MRSA, South German EMRSA (truncated SCCmec element)	POS	POS	NEG	NEG	POS	NEG	NEG	POS	POS	NEG	NEG	POS	POS	POS	POS	NEG	NEG	NEG	POS	POS	POS	POS	NEG	NEG	POS	POS	NEG	POS	POS	NEG	NEG	POS	NEG	NEG	NEG	NEG	NEG	NEG	NEG	NEG	POS	AMB	NEG	POS	POS	POS	NEG	POS
00V46910	CC5/ST228-MRSA, South German EMRSA (truncated SCCmec element)	POS	POS	NEG	NEG	POS	NEG	NEG	POS	POS	NEG	NEG	POS	POS	POS	POS	NEG	NEG	NEG	POS	POS	POS	POS	NEG	NEG	POS	POS	NEG	POS	POS	NEG	NEG	POS	NEG	NEG	NEG	NEG	NEG	NEG	NEG	NEG	POS	POS	NEG	POS	POS	POS	NEG	POS
00V07063	CC5/ST228-MRSA-I, South German EMRSA	POS	POS	AMB	NEG	POS	NEG	AMB	POS	POS	NEG	NEG	POS	POS	POS	POS	NEG	NEG	NEG	POS	POS	POS	POS	NEG	NEG	POS	POS	NEG	POS	POS	NEG	NEG	POS	NEG	NEG	NEG	NEG	NEG	NEG	NEG	NEG	POS	POS	AMB	POS	POS	POS	AMB	POS
00V46921	CC5-MRSA-II, Rhine-Hesse EMRSA/UK-EMRSA-3	POS	POS	NEG	NEG	POS	NEG	NEG	POS	POS	NEG	NEG	POS	POS	POS	POS	NEG	NEG	NEG	POS	POS	POS	POS	NEG	NEG	POS	POS	NEG	POS	POS	NEG	NEG	POS	NEG	POS	NEG	AMB	POS	NEG	NEG	NEG	POS	POS	NEG	POS	POS	POS	NEG	POS
00V08510	CC8/ST254-MRSA-(atypical SCCmec), Hannover EMRSA	NEG	NEG	NEG	NEG	NEG	NEG	NEG	POS	POS	POS	NEG	POS	POS	POS	POS	NEG	NEG	NEG	POS	POS	POS	POS	NEG	POS	POS	POS	NEG	POS	POS	POS	NEG	NEG	NEG	POS	POS	POS	NEG	NEG	NEG	NEG	POS	POS	NEG	NEG	POS	POS	NEG	POS
00V25538	CC8/ST254-MRSA-(atypical SCCmec), Hannover EMRSA	NEG	NEG	NEG	NEG	NEG	NEG	NEG	POS	POS	POS	AMB	AMB	POS	POS	POS	NEG	NEG	NEG	POS	POS	POS	POS	NEG	POS	POS	POS	NEG	POS	POS	POS	NEG	NEG	NEG	POS	POS	AMB	NEG	NEG	NEG	NEG	POS	POS	NEG	NEG	POS	POS	NEG	POS
00V38664	CC8/ST254-MRSA-(atypical SCCmec), Hannover EMRSA	NEG	NEG	NEG	NEG	NEG	NEG	NEG	POS	POS	POS	POS	POS	POS	POS	POS	NEG	NEG	NEG	POS	POS	POS	POS	NEG	POS	POS	POS	NEG	POS	POS	POS	NEG	NEG	NEG	POS	POS	POS	POS	NEG	NEG	NEG	POS	POS	NEG	POS	POS	POS	NEG	POS
00V46488	CC8/ST254-MRSA-(atypical SCCmec), Hannover EMRSA	NEG	NEG	NEG	NEG	NEG	NEG	NEG	POS	POS	POS	AMB	AMB	POS	POS	POS	NEG	NEG	NEG	POS	POS	POS	POS	NEG	POS	POS	POS	NEG	POS	POS	POS	NEG	NEG	NEG	POS	POS	AMB	AMB	NEG	NEG	NEG	POS	POS	NEG	NEG	POS	POS	NEG	POS
00V44035	CC8/ST254-MRSA-IV/V, UK-EMRSA-10/Hannover EMRSA	NEG	NEG	NEG	NEG	NEG	NEG	NEG	POS	POS	POS	AMB	POS	POS	POS	POS	NEG	NEG	NEG	POS	POS	POS	POS	NEG	POS	POS	POS	NEG	POS	POS	POS	NEG	NEG	NEG	POS	POS	POS	NEG	NEG	NEG	NEG	POS	POS	NEG	NEG	POS	POS	NEG	POS
00V13324	CC22-MRSA-IV [PVL+]	POS	POS	NEG	NEG	NEG	NEG	NEG	POS	POS	NEG	NEG	POS	POS	POS	NEG	NEG	NEG	POS	NEG	POS	AMB	POS	NEG	POS	POS	NEG	POS	POS	POS	NEG	NEG	POS	NEG	POS	NEG	AMB	NEG	POS	NEG	NEG	POS	NEG	NEG	POS	POS	NEG	POS	NEG
00B02046	CC45-MRSA-IV, Berlin EMRSA	POS	POS	NEG	NEG	NEG	NEG	POS	POS	POS	AMB	NEG	POS	POS	POS	NEG	AMB	POS	POS	POS	POS	NEG	POS	POS	NEG	POS	NEG	POS	POS	POS	NEG	NEG	POS	NEG	POS	NEG	AMB	POS	NEG	NEG	NEG	POS	NEG	POS	NEG	NEG	NEG	NEG	NEG
00V11299	CC45-MRSA-IV, Berlin EMRSA	POS	POS	NEG	NEG	AMB	NEG	POS	POS	POS	AMB	NEG	POS	POS	POS	NEG	AMB	POS	POS	POS	POS	NEG	POS	POS	NEG	POS	NEG	POS	POS	POS	NEG	NEG	POS	NEG	POS	NEG	AMB	POS	NEG	NEG	NEG	POS	NEG	POS	NEG	NEG	NEG	NEG	NEG
00V14125	CC45-MRSA-IV, Berlin EMRSA	POS	POS	NEG	NEG	NEG	NEG	POS	POS	POS	AMB	NEG	POS	POS	POS	NEG	POS	AMB	POS	POS	POS	NEG	POS	POS	NEG	POS	NEG	POS	POS	POS	NEG	NEG	POS	NEG	POS	NEG	POS	AMB	NEG	NEG	NEG	POS	NEG	POS	NEG	NEG	NEG	NEG	NEG
00V16040	CC45-MRSA-IV, Berlin EMRSA	POS	POS	NEG	NEG	NEG	NEG	POS	POS	POS	NEG	NEG	POS	POS	POS	NEG	POS	AMB	POS	POS	POS	NEG	POS	POS	NEG	POS	NEG	POS	POS	POS	NEG	NEG	POS	NEG	POS	NEG	POS	AMB	NEG	NEG	NEG	POS	NEG	POS	NEG	NEG	NEG	NEG	NEG
00V25025	CC45-MRSA-IV, Berlin EMRSA	POS	POS	NEG	NEG	NEG	NEG	POS	POS	POS	AMB	NEG	POS	POS	POS	NEG	POS	AMB	POS	POS	POS	NEG	POS	POS	NEG	POS	NEG	POS	POS	POS	NEG	NEG	POS	NEG	POS	NEG	AMB	POS	NEG	AMB	NEG	POS	NEG	POS	NEG	NEG	NEG	NEG	NEG
00V29618	CC45-MRSA-IV, Berlin EMRSA	POS	POS	NEG	NEG	NEG	NEG	POS	POS	POS	AMB	NEG	POS	POS	POS	NEG	POS	AMB	POS	POS	POS	NEG	POS	POS	NEG	POS	NEG	POS	POS	POS	NEG	NEG	POS	NEG	POS	NEG	POS	AMB	NEG	NEG	NEG	POS	NEG	POS	NEG	NEG	NEG	NEG	NEG
00V30989	CC45-MRSA-IV, Berlin EMRSA	POS	POS	NEG	NEG	NEG	NEG	POS	POS	POS	AMB	NEG	POS	POS	POS	NEG	POS	AMB	POS	POS	POS	NEG	POS	POS	NEG	POS	NEG	POS	POS	POS	NEG	NEG	POS	NEG	POS	NEG	POS	AMB	NEG	AMB	NEG	POS	NEG	POS	NEG	NEG	NEG	NEG	NEG
00V31007	CC45-MRSA-IV, Berlin EMRSA	POS	POS	NEG	NEG	NEG	NEG	POS	POS	POS	AMB	NEG	POS	POS	POS	NEG	POS	AMB	POS	POS	POS	NEG	POS	POS	NEG	POS	NEG	POS	POS	POS	NEG	NEG	POS	NEG	POS	NEG	POS	AMB	NEG	AMB	NEG	POS	NEG	POS	NEG	NEG	NEG	NEG	NEG
00V31700	CC45-MRSA-IV, Berlin EMRSA	POS	POS	NEG	NEG	NEG	NEG	POS	POS	POS	AMB	NEG	POS	POS	POS	NEG	POS	AMB	POS	POS	POS	NEG	POS	POS	NEG	POS	NEG	POS	POS	POS	NEG	NEG	POS	NEG	POS	NEG	POS	AMB	NEG	AMB	NEG	POS	NEG	POS	NEG	NEG	NEG	NEG	NEG
00V32193	CC45-MRSA-IV, Berlin EMRSA	POS	POS	NEG	NEG	NEG	NEG	POS	POS	POS	AMB	NEG	POS	POS	POS	NEG	POS	AMB	POS	POS	POS	NEG	POS	POS	NEG	POS	NEG	POS	POS	POS	NEG	NEG	POS	NEG	POS	NEG	POS	AMB	NEG	AMB	NEG	POS	NEG	POS	NEG	NEG	NEG	NEG	NEG
00V43085	CC45-MRSA-IV, Berlin EMRSA	POS	POS	NEG	NEG	NEG	NEG	POS	POS	POS	AMB	NEG	POS	POS	POS	NEG	POS	AMB	POS	POS	POS	NEG	POS	POS	NEG	POS	NEG	POS	POS	POS	NEG	NEG	POS	NEG	POS	NEG	POS	AMB	NEG	NEG	NEG	POS	NEG	POS	NEG	NEG	NEG	NEG	NEG
00V44382	CC45-MRSA-IV, Berlin EMRSA	POS	POS	NEG	NEG	NEG	NEG	POS	POS	POS	NEG	NEG	POS	POS	POS	NEG	POS	AMB	POS	POS	POS	NEG	POS	POS	NEG	POS	NEG	AMB	POS	POS	NEG	NEG	POS	NEG	POS	NEG	POS	AMB	NEG	NEG	NEG	POS	NEG	POS	NEG	NEG	NEG	NEG	NEG
00V46648	CC45-MRSA-IV, Berlin EMRSA	POS	POS	NEG	NEG	NEG	NEG	POS	POS	POS	AMB	NEG	POS	POS	POS	NEG	POS	AMB	POS	POS	POS	NEG	POS	POS	NEG	POS	NEG	POS	POS	POS	NEG	NEG	POS	NEG	POS	NEG	POS	AMB	NEG	AMB	NEG	POS	NEG	POS	NEG	NEG	NEG	NEG	NEG
00V46843	CC45-MRSA-IV, Berlin EMRSA	POS	POS	NEG	NEG	NEG	NEG	POS	POS	POS	AMB	NEG	POS	POS	POS	NEG	POS	AMB	POS	POS	POS	NEG	POS	POS	NEG	POS	NEG	POS	POS	POS	NEG	NEG	POS	NEG	POS	NEG	POS	AMB	NEG	AMB	NEG	POS	NEG	POS	NEG	NEG	NEG	NEG	NEG
00V47014	CC45-MRSA-IV, Berlin EMRSA	POS	POS	NEG	NEG	NEG	NEG	POS	POS	POS	AMB	NEG	POS	POS	POS	NEG	POS	AMB	POS	POS	POS	NEG	POS	POS	NEG	POS	NEG	POS	POS	POS	NEG	NEG	POS	NEG	POS	NEG	POS	AMB	NEG	NEG	NEG	POS	NEG	POS	NEG	NEG	NEG	NEG	NEG
00V47053	CC45-MRSA-IV, Berlin EMRSA	POS	POS	NEG	NEG	NEG	NEG	POS	POS	POS	NEG	NEG	POS	POS	POS	NEG	POS	POS	POS	POS	POS	NEG	POS	POS	NEG	POS	NEG	POS	POS	POS	NEG	NEG	POS	NEG	POS	NEG	POS	POS	NEG	NEG	NEG	POS	NEG	POS	NEG	NEG	NEG	NEG	NEG
00V47227	CC45-MRSA-IV, Berlin EMRSA	POS	POS	AMB	NEG	NEG	NEG	POS	POS	POS	POS	NEG	AMB	POS	POS	NEG	AMB	POS	POS	POS	POS	NEG	POS	POS	NEG	POS	NEG	POS	POS	POS	NEG	NEG	POS	NEG	POS	NEG	POS	AMB	NEG	AMB	NEG	POS	NEG	POS	NEG	NEG	NEG	NEG	NEG
00V47429	CC45-MRSA-IV, Berlin EMRSA	POS	POS	NEG	NEG	NEG	NEG	POS	POS	POS	AMB	NEG	POS	POS	POS	NEG	POS	AMB	POS	POS	POS	NEG	POS	POS	NEG	POS	NEG	POS	POS	POS	NEG	NEG	POS	NEG	POS	NEG	POS	AMB	NEG	AMB	NEG	POS	NEG	POS	NEG	NEG	NEG	NEG	NEG
00V47531	CC45-MRSA-IV, Berlin EMRSA	POS	POS	AMB	NEG	NEG	NEG	POS	POS	POS	AMB	NEG	POS	POS	POS	NEG	POS	AMB	POS	POS	POS	NEG	POS	POS	NEG	POS	NEG	POS	POS	POS	NEG	NEG	POS	NEG	POS	NEG	POS	AMB	NEG	AMB	NEG	POS	NEG	POS	NEG	NEG	NEG	NEG	NEG
**2001**																																																	
01V27106	CC1-MRSA-IV, WA MRSA-1/57	POS	POS	POS	NEG	NEG	NEG	NEG	POS	POS	NEG	NEG	POS	POS	POS	NEG	POS	POS	POS	POS	POS	POS	POS	NEG	NEG	POS	POS	NEG	POS	POS	NEG	NEG	POS	NEG	POS	NEG	POS	NEG	POS	NEG	NEG	POS	NEG	NEG	POS	POS	NEG	POS	POS
01V41025	CC5/ST228-MRSA-I, South German EMRSA	POS	POS	AMB	NEG	POS	NEG	AMB	POS	POS	NEG	NEG	POS	POS	POS	POS	NEG	NEG	NEG	POS	POS	POS	POS	NEG	NEG	POS	POS	NEG	POS	POS	NEG	NEG	POS	NEG	NEG	NEG	NEG	NEG	NEG	NEG	NEG	POS	POS	AMB	POS	POS	POS	AMB	POS
01V12125	CC5-MRSA-II, Rhine-Hesse EMRSA/UK-EMRSA-3	POS	POS	NEG	NEG	POS	NEG	NEG	POS	POS	NEG	NEG	POS	POS	POS	POS	NEG	NEG	NEG	POS	POS	POS	POS	NEG	NEG	POS	POS	NEG	POS	POS	NEG	NEG	POS	NEG	POS	NEG	POS	AMB	NEG	NEG	NEG	POS	POS	NEG	POS	POS	POS	NEG	POS
01V29747	CC5-MRSA-II, Rhine-Hesse EMRSA/UK-EMRSA-3	POS	POS	NEG	NEG	POS	NEG	NEG	POS	POS	NEG	NEG	POS	POS	POS	POS	NEG	NEG	NEG	POS	POS	POS	POS	NEG	NEG	POS	POS	NEG	POS	POS	NEG	NEG	POS	NEG	POS	NEG	AMB	POS	NEG	NEG	NEG	POS	POS	NEG	POS	POS	POS	NEG	POS
01V32464	CC5-MRSA-II, Rhine-Hesse EMRSA/UK-EMRSA-3	POS	POS	NEG	NEG	POS	NEG	NEG	POS	POS	NEG	NEG	POS	POS	POS	POS	NEG	NEG	NEG	POS	POS	POS	POS	NEG	NEG	POS	POS	NEG	POS	POS	NEG	NEG	POS	NEG	POS	NEG	POS	POS	NEG	NEG	NEG	POS	NEG	NEG	POS	POS	POS	NEG	POS
01V39703	CC5-MRSA-II, Rhine-Hesse EMRSA/UK-EMRSA-3	POS	POS	NEG	NEG	POS	NEG	NEG	POS	POS	NEG	NEG	POS	POS	POS	POS	NEG	NEG	NEG	POS	POS	POS	POS	NEG	NEG	POS	POS	NEG	POS	POS	NEG	NEG	POS	NEG	POS	NEG	POS	AMB	NEG	NEG	NEG	POS	AMB	NEG	POS	POS	POS	NEG	POS
01V40228	CC5-MRSA-II, Rhine-Hesse EMRSA/UK-EMRSA-3																																																
01B05861	CC8/ST239-MRSA-III, Vienna/Hungarian/Brazilian Clone	POS	POS	POS	NEG	NEG	NEG	NEG	POS	POS	POS	AMB	AMB	POS	POS	NEG	NEG	NEG	POS	POS	POS	POS	POS	NEG	POS	POS	POS	NEG	NEG	NEG	NEG	NEG	NEG	NEG	POS	POS	AMB	NEG	NEG	NEG	NEG	POS	POS	NEG	NEG	POS	POS	NEG	POS
01V34572	CC8/ST239-MRSA-III, Vienna/Hungarian/Brazilian Clone	POS	POS	POS	NEG	NEG	NEG	NEG	POS	POS	POS	AMB	POS	POS	POS	NEG	NEG	NEG	POS	POS	POS	AMB	POS	NEG	POS	POS	POS	NEG	NEG	NEG	NEG	NEG	NEG	NEG	POS	POS	POS	NEG	NEG	NEG	NEG	POS	POS	NEG	NEG	POS	POS	NEG	POS
01V36469	CC8/ST239-MRSA-III, Vienna/Hungarian/Brazilian Clone	POS	POS	POS	NEG	NEG	NEG	NEG	POS	POS	POS	NEG	AMB	POS	POS	NEG	NEG	NEG	POS	POS	POS	POS	POS	NEG	AMB	POS	POS	NEG	NEG	NEG	NEG	NEG	NEG	NEG	POS	AMB	POS	NEG	NEG	NEG	NEG	POS	POS	NEG	NEG	POS	POS	NEG	POS
01V39123	CC8/ST239-MRSA-III, Vienna/Hungarian/Brazilian Clone	POS	POS	POS	NEG	AMB	NEG	AMB	POS	POS	POS	AMB	AMB	POS	POS	NEG	NEG	POS	POS	POS	POS	POS	POS	NEG	POS	POS	POS	NEG	NEG	NEG	NEG	NEG	NEG	NEG	POS	POS	AMB	AMB	NEG	NEG	NEG	POS	POS	NEG	AMB	POS	POS	POS	POS
01V39143	CC8/ST239-MRSA-III, Vienna/Hungarian/Brazilian Clone	POS	POS	POS	NEG	NEG	NEG	NEG	POS	POS	POS	AMB	AMB	POS	POS	NEG	NEG	NEG	POS	POS	POS	POS	POS	NEG	POS	POS	POS	NEG	NEG	NEG	NEG	NEG	NEG	NEG	POS	POS	AMB	NEG	NEG	NEG	NEG	POS	POS	NEG	NEG	POS	POS	NEG	POS
01V10962	CC8/ST254-MRSA-(atypical SCCmec), Hannover EMRSA	NEG	NEG	NEG	NEG	NEG	NEG	NEG	POS	POS	POS	NEG	POS	POS	POS	POS	NEG	NEG	NEG	POS	POS	POS	POS	NEG	POS	POS	POS	NEG	POS	POS	POS	NEG	NEG	NEG	POS	POS	POS	POS	NEG	NEG	NEG	POS	POS	NEG	AMB	POS	POS	NEG	POS
01V10963	CC8/ST254-MRSA-IV/V, UK-EMRSA-10/Hannover EMRSA	NEG	NEG	NEG	NEG	NEG	NEG	NEG	POS	POS	POS	NEG	POS	POS	POS	POS	NEG	NEG	NEG	POS	POS	POS	POS	NEG	POS	POS	POS	NEG	POS	POS	POS	NEG	NEG	NEG	POS	POS	POS	POS	NEG	NEG	NEG	POS	POS	NEG	POS	POS	POS	NEG	POS
01V36287	CC8/ST254-MRSA-IV/V, UK-EMRSA-10/Hannover EMRSA	NEG	NEG	NEG	NEG	NEG	NEG	NEG	POS	POS	POS	AMB	AMB	POS	POS	POS	NEG	NEG	NEG	POS	POS	POS	POS	NEG	POS	POS	POS	NEG	POS	POS	POS	NEG	NEG	NEG	POS	POS	AMB	AMB	NEG	NEG	NEG	POS	POS	NEG	NEG	POS	POS	NEG	POS
01V25329	CC22-MRSA-IV [PVL+]	POS	POS	NEG	NEG	NEG	NEG	NEG	POS	POS	NEG	NEG	POS	POS	POS	NEG	NEG	POS	POS	NEG	POS	POS	POS	NEG	POS	POS	NEG	POS	POS	POS	NEG	NEG	POS	NEG	POS	NEG	AMB	AMB	POS	NEG	NEG	POS	NEG	NEG	POS	POS	NEG	POS	NEG
01V07770	CC22-MRSA-IV, Barnim/UK-EMRSA-15																																																
01V20122	CC22-MRSA-IV, Barnim/UK-EMRSA-15	POS	POS	NEG	NEG	NEG	NEG	NEG	POS	POS	NEG	NEG	POS	POS	POS	NEG	NEG	NEG	POS	NEG	POS	NEG	POS	NEG	POS	POS	NEG	AMB	POS	POS	NEG	NEG	POS	NEG	NEG	NEG	NEG	NEG	NEG	NEG	NEG	POS	NEG	NEG	POS	POS	NEG	POS	POS
01V43277	CC22-MRSA-IV, Barnim/UK-EMRSA-15	POS	POS	NEG	NEG	NEG	NEG	NEG	POS	POS	NEG	NEG	POS	POS	POS	NEG	NEG	NEG	POS	NEG	POS	POS	POS	NEG	POS	POS	NEG	POS	POS	POS	NEG	NEG	POS	NEG	NEG	NEG	NEG	NEG	NEG	NEG	NEG	POS	NEG	NEG	POS	POS	NEG	POS	POS
01F02744	CC45-MRSA-IV, Berlin EMRSA	POS	POS	NEG	NEG	NEG	NEG	POS	POS	POS	AMB	NEG	POS	POS	POS	NEG	POS	AMB	POS	POS	POS	NEG	POS	POS	NEG	POS	NEG	POS	POS	POS	NEG	NEG	POS	NEG	POS	NEG	POS	AMB	NEG	AMB	NEG	POS	NEG	POS	NEG	NEG	NEG	NEG	NEG
01V01127	CC45-MRSA-IV, Berlin EMRSA	POS	POS	NEG	NEG	NEG	NEG	POS	POS	POS	AMB	NEG	POS	POS	POS	NEG	POS	AMB	POS	POS	POS	NEG	POS	POS	NEG	POS	NEG	POS	POS	POS	NEG	NEG	POS	NEG	POS	NEG	POS	AMB	NEG	AMB	NEG	POS	NEG	POS	NEG	NEG	NEG	NEG	NEG
01V01445	CC45-MRSA-IV, Berlin EMRSA	POS	POS	AMB	NEG	AMB	NEG	POS	POS	POS	AMB	NEG	POS	POS	POS	NEG	POS	AMB	POS	POS	POS	NEG	POS	POS	NEG	POS	NEG	POS	POS	POS	NEG	NEG	POS	NEG	POS	NEG	POS	AMB	NEG	NEG	NEG	POS	NEG	POS	NEG	NEG	NEG	NEG	NEG
01V02575	CC45-MRSA-IV, Berlin EMRSA	POS	POS	NEG	NEG	NEG	NEG	POS	POS	POS	NEG	NEG	POS	POS	POS	NEG	POS	AMB	POS	POS	POS	NEG	POS	POS	NEG	POS	NEG	POS	POS	POS	NEG	NEG	POS	NEG	POS	NEG	POS	AMB	NEG	NEG	NEG	POS	NEG	POS	NEG	NEG	NEG	NEG	NEG
01V02636	CC45-MRSA-IV, Berlin EMRSA	POS	POS	NEG	NEG	NEG	NEG	POS	POS	POS	AMB	NEG	POS	POS	POS	NEG	POS	AMB	POS	POS	POS	NEG	POS	POS	NEG	POS	NEG	POS	POS	POS	NEG	NEG	POS	NEG	POS	NEG	POS	AMB	NEG	NEG	NEG	POS	NEG	POS	NEG	NEG	NEG	NEG	NEG
01V03483	CC45-MRSA-IV, Berlin EMRSA	POS	POS	NEG	NEG	NEG	NEG	POS	POS	POS	NEG	NEG	POS	POS	POS	NEG	POS	AMB	POS	POS	POS	NEG	POS	POS	NEG	POS	NEG	AMB	POS	POS	NEG	NEG	POS	NEG	POS	NEG	AMB	POS	NEG	NEG	NEG	POS	NEG	POS	NEG	NEG	NEG	NEG	NEG
01V04728	CC45-MRSA-IV, Berlin EMRSA	POS	POS	NEG	NEG	NEG	NEG	POS	POS	POS	AMB	NEG	POS	POS	POS	NEG	POS	AMB	POS	POS	POS	NEG	POS	POS	NEG	POS	NEG	POS	POS	POS	NEG	NEG	POS	NEG	POS	NEG	POS	AMB	NEG	NEG	NEG	POS	NEG	POS	NEG	NEG	NEG	NEG	NEG
01V05972	CC45-MRSA-IV, Berlin EMRSA	POS	POS	NEG	NEG	NEG	NEG	POS	POS	POS	NEG	NEG	POS	POS	POS	NEG	POS	AMB	POS	POS	POS	NEG	POS	AMB	NEG	POS	NEG	NEG	POS	POS	NEG	NEG	POS	NEG	POS	NEG	POS	AMB	NEG	NEG	NEG	POS	NEG	POS	NEG	NEG	NEG	NEG	NEG
01V07139	CC45-MRSA-IV, Berlin EMRSA	POS	POS	NEG	NEG	NEG	NEG	POS	POS	POS	AMB	NEG	POS	POS	POS	NEG	POS	AMB	POS	POS	POS	NEG	POS	POS	NEG	POS	NEG	POS	POS	POS	NEG	NEG	POS	NEG	POS	NEG	POS	AMB	NEG	NEG	NEG	POS	NEG	POS	NEG	NEG	NEG	NEG	NEG
01V08133	CC45-MRSA-IV, Berlin EMRSA	POS	POS	NEG	NEG	NEG	NEG	POS	POS	POS	AMB	NEG	POS	POS	POS	NEG	POS	AMB	POS	POS	POS	NEG	POS	POS	NEG	POS	NEG	POS	POS	POS	NEG	NEG	POS	NEG	POS	NEG	POS	AMB	NEG	NEG	NEG	POS	NEG	POS	NEG	NEG	NEG	NEG	NEG
01V09872	CC45-MRSA-IV, Berlin EMRSA	POS	POS	NEG	NEG	NEG	NEG	POS	POS	POS	AMB	NEG	POS	POS	POS	NEG	POS	AMB	POS	POS	POS	NEG	POS	POS	NEG	POS	NEG	POS	POS	POS	NEG	NEG	POS	NEG	POS	NEG	POS	AMB	NEG	NEG	NEG	POS	NEG	POS	NEG	NEG	NEG	NEG	NEG
01V12465	CC45-MRSA-IV, Berlin EMRSA	POS	POS	NEG	NEG	NEG	NEG	POS	POS	POS	AMB	NEG	POS	POS	POS	NEG	POS	AMB	POS	POS	POS	NEG	POS	POS	NEG	POS	NEG	POS	POS	POS	NEG	NEG	POS	NEG	POS	NEG	POS	AMB	NEG	NEG	NEG	POS	NEG	POS	NEG	NEG	NEG	NEG	NEG
01V16151	CC45-MRSA-IV, Berlin EMRSA	POS	POS	NEG	NEG	NEG	NEG	POS	POS	POS	AMB	NEG	POS	POS	POS	NEG	POS	AMB	POS	POS	POS	NEG	POS	POS	NEG	POS	NEG	POS	POS	POS	NEG	NEG	POS	NEG	POS	NEG	POS	AMB	NEG	NEG	NEG	POS	NEG	POS	NEG	NEG	NEG	NEG	NEG
01V16873	CC45-MRSA-IV, Berlin EMRSA	POS	POS	NEG	NEG	NEG	NEG	POS	POS	POS	AMB	NEG	POS	POS	POS	NEG	POS	AMB	POS	POS	POS	NEG	POS	POS	NEG	POS	NEG	POS	POS	POS	NEG	NEG	POS	NEG	POS	NEG	POS	AMB	NEG	AMB	NEG	POS	NEG	POS	NEG	NEG	NEG	NEG	NEG
01V17796	CC45-MRSA-IV, Berlin EMRSA	POS	POS	NEG	NEG	NEG	NEG	POS	POS	POS	AMB	NEG	POS	POS	POS	NEG	POS	AMB	POS	POS	POS	NEG	POS	POS	NEG	POS	NEG	POS	POS	POS	NEG	NEG	POS	NEG	POS	NEG	POS	AMB	NEG	AMB	NEG	POS	NEG	POS	NEG	NEG	NEG	NEG	NEG
01V18681	CC45-MRSA-IV, Berlin EMRSA	POS	POS	NEG	NEG	NEG	NEG	POS	POS	POS	AMB	NEG	POS	POS	POS	NEG	POS	AMB	POS	POS	POS	NEG	POS	POS	NEG	POS	NEG	POS	POS	POS	NEG	NEG	POS	NEG	POS	NEG	POS	AMB	NEG	NEG	NEG	POS	NEG	POS	NEG	NEG	NEG	NEG	NEG
01V20066	CC45-MRSA-IV, Berlin EMRSA	POS	POS	NEG	NEG	NEG	NEG	POS	POS	POS	AMB	NEG	POS	POS	POS	NEG	POS	AMB	POS	POS	POS	NEG	POS	POS	NEG	POS	NEG	POS	POS	POS	NEG	NEG	POS	NEG	POS	NEG	POS	AMB	NEG	NEG	NEG	POS	NEG	POS	NEG	NEG	NEG	NEG	NEG
01V23050	CC45-MRSA-IV, Berlin EMRSA	POS	POS	NEG	NEG	NEG	NEG	POS	POS	POS	AMB	NEG	POS	POS	POS	NEG	POS	AMB	POS	POS	POS	NEG	POS	POS	NEG	POS	NEG	POS	POS	POS	NEG	NEG	POS	NEG	POS	NEG	POS	AMB	NEG	NEG	NEG	POS	NEG	POS	NEG	NEG	NEG	NEG	NEG
01V25584	CC45-MRSA-IV, Berlin EMRSA	POS	POS	NEG	NEG	NEG	NEG	POS	POS	POS	NEG	NEG	POS	POS	POS	NEG	POS	AMB	AMB	POS	POS	NEG	POS	NEG	NEG	POS	NEG	NEG	POS	POS	NEG	NEG	POS	NEG	POS	NEG	POS	AMB	NEG	NEG	NEG	POS	NEG	POS	NEG	NEG	NEG	NEG	NEG
01V31705	CC45-MRSA-IV, Berlin EMRSA	POS	POS	NEG	NEG	NEG	NEG	POS	POS	POS	AMB	NEG	POS	POS	POS	NEG	POS	AMB	POS	POS	POS	NEG	POS	POS	NEG	POS	NEG	POS	POS	POS	NEG	NEG	POS	NEG	POS	NEG	POS	AMB	NEG	AMB	NEG	POS	NEG	POS	NEG	NEG	NEG	NEG	NEG
01V34968	CC45-MRSA-IV, Berlin EMRSA	POS	POS	NEG	NEG	NEG	NEG	POS	POS	POS	AMB	NEG	POS	POS	POS	NEG	POS	AMB	POS	POS	POS	NEG	POS	POS	NEG	POS	NEG	POS	POS	POS	NEG	NEG	POS	NEG	POS	NEG	POS	AMB	NEG	AMB	NEG	POS	NEG	POS	NEG	NEG	NEG	NEG	NEG
01V36186	CC45-MRSA-IV, Berlin EMRSA	POS	POS	NEG	NEG	NEG	NEG	POS	POS	POS	NEG	NEG	POS	POS	POS	NEG	POS	AMB	POS	POS	POS	NEG	POS	AMB	NEG	POS	NEG	AMB	POS	POS	NEG	NEG	POS	NEG	POS	NEG	POS	AMB	NEG	NEG	NEG	POS	NEG	POS	NEG	NEG	NEG	NEG	NEG
01V36534	CC45-MRSA-IV, Berlin EMRSA	POS	POS	NEG	NEG	NEG	NEG	POS	POS	POS	AMB	NEG	POS	POS	POS	NEG	POS	AMB	POS	POS	POS	NEG	POS	POS	NEG	POS	NEG	POS	POS	POS	NEG	NEG	POS	NEG	POS	NEG	POS	AMB	NEG	NEG	NEG	POS	NEG	POS	NEG	NEG	NEG	NEG	NEG
01V36588	CC45-MRSA-IV, Berlin EMRSA	POS	POS	NEG	NEG	NEG	NEG	POS	POS	POS	AMB	NEG	POS	POS	POS	NEG	POS	AMB	POS	POS	POS	NEG	POS	POS	NEG	POS	NEG	POS	POS	POS	NEG	NEG	POS	NEG	POS	NEG	POS	AMB	NEG	AMB	NEG	POS	NEG	POS	NEG	NEG	NEG	NEG	NEG
01V37990	CC45-MRSA-IV, Berlin EMRSA	POS	POS	NEG	NEG	NEG	NEG	POS	POS	POS	AMB	NEG	POS	POS	POS	NEG	AMB	POS	POS	POS	POS	NEG	POS	POS	NEG	POS	NEG	POS	POS	POS	NEG	NEG	POS	NEG	POS	NEG	POS	AMB	NEG	NEG	NEG	POS	NEG	POS	NEG	NEG	NEG	NEG	NEG
01V39610	CC45-MRSA-IV, Berlin EMRSA	POS	POS	AMB	NEG	AMB	NEG	POS	POS	POS	POS	NEG	AMB	POS	POS	AMB	POS	AMB	POS	POS	POS	NEG	POS	POS	AMB	POS	NEG	POS	POS	POS	NEG	NEG	POS	NEG	POS	NEG	POS	AMB	NEG	AMB	NEG	POS	POS	POS	NEG	NEG	NEG	AMB	NEG
01V40229	CC45-MRSA-IV, Berlin EMRSA	POS	POS	NEG	NEG	NEG	NEG	POS	POS	POS	AMB	NEG	POS	POS	POS	NEG	POS	AMB	POS	POS	POS	NEG	POS	POS	NEG	POS	NEG	POS	POS	POS	NEG	NEG	POS	NEG	POS	NEG	POS	AMB	NEG	NEG	NEG	POS	NEG	POS	NEG	NEG	NEG	NEG	NEG
01V40374	CC45-MRSA-IV, Berlin EMRSA																																																
01V41056	CC45-MRSA-IV, Berlin EMRSA	POS	POS	NEG	NEG	NEG	NEG	POS	POS	POS	NEG	NEG	POS	POS	POS	NEG	POS	AMB	POS	POS	POS	NEG	POS	NEG	NEG	POS	NEG	POS	POS	POS	NEG	NEG	POS	NEG	POS	NEG	POS	AMB	NEG	NEG	NEG	POS	NEG	POS	NEG	NEG	NEG	NEG	NEG
01V42009	CC45-MRSA-IV, Berlin EMRSA	POS	POS	NEG	NEG	NEG	NEG	POS	POS	POS	AMB	NEG	POS	POS	POS	NEG	POS	AMB	POS	POS	POS	NEG	POS	POS	NEG	POS	NEG	POS	POS	POS	NEG	NEG	POS	NEG	POS	NEG	POS	AMB	NEG	NEG	NEG	POS	NEG	POS	NEG	NEG	NEG	NEG	NEG
01V43511	CC45-MRSA-IV, Berlin EMRSA	POS	POS	NEG	NEG	NEG	NEG	POS	POS	POS	NEG	NEG	POS	POS	POS	NEG	POS	AMB	POS	POS	POS	NEG	POS	AMB	NEG	POS	NEG	POS	POS	POS	NEG	NEG	POS	NEG	POS	NEG	POS	AMB	NEG	NEG	NEG	POS	NEG	POS	NEG	NEG	NEG	NEG	NEG
**2002**																																																	
02V10698	CC5/ST228-MRSA-I, South German EMRSA	POS	POS	NEG	NEG	POS	NEG	NEG	NEG	NEG	NEG	NEG	NEG	POS	POS	POS	NEG	NEG	NEG	POS	POS	POS	POS	NEG	NEG	POS	POS	NEG	POS	POS	NEG	NEG	POS	NEG	NEG	NEG	NEG	NEG	NEG	NEG	NEG	POS	POS	NEG	POS	POS	POS	NEG	POS
02V13140	CC5/ST228-MRSA-I, South German EMRSA	POS	POS	NEG	NEG	POS	NEG	NEG	POS	POS	NEG	NEG	POS	POS	POS	POS	NEG	NEG	NEG	POS	POS	POS	POS	NEG	NEG	POS	POS	NEG	POS	POS	NEG	NEG	POS	NEG	NEG	NEG	NEG	NEG	NEG	NEG	NEG	POS	POS	NEG	POS	POS	POS	NEG	POS
02V20759	CC5/ST228-MRSA-I, South German EMRSA	POS	POS	NEG	NEG	POS	NEG	NEG	POS	POS	NEG	NEG	POS	POS	POS	POS	NEG	NEG	NEG	POS	POS	AMB	POS	NEG	NEG	POS	POS	NEG	POS	POS	NEG	NEG	POS	NEG	NEG	NEG	NEG	NEG	NEG	NEG	NEG	POS	NEG	NEG	POS	POS	POS	NEG	POS
02B06271	CC5-MRSA-II, Rhine-Hesse EMRSA/UK-EMRSA-3	POS	POS	NEG	NEG	POS	NEG	NEG	POS	POS	NEG	NEG	POS	POS	POS	POS	NEG	NEG	NEG	POS	POS	POS	POS	NEG	NEG	POS	POS	NEG	POS	POS	NEG	NEG	POS	NEG	POS	NEG	AMB	POS	NEG	NEG	NEG	POS	POS	NEG	POS	POS	POS	NEG	POS
02V04116	CC5-MRSA-II, Rhine-Hesse EMRSA/UK-EMRSA-3	POS	POS	NEG	NEG	POS	NEG	NEG	POS	POS	NEG	NEG	POS	POS	POS	POS	NEG	NEG	NEG	POS	POS	POS	POS	NEG	NEG	POS	POS	NEG	POS	POS	NEG	NEG	POS	NEG	POS	NEG	POS	AMB	NEG	NEG	NEG	POS	AMB	NEG	POS	POS	POS	NEG	POS
02V29922	CC5-MRSA-II, Rhine-Hesse EMRSA/UK-EMRSA-3																																																
02V33634	CC5-MRSA-II, Rhine-Hesse EMRSA/UK-EMRSA-3	POS	POS	NEG	NEG	POS	NEG	NEG	POS	POS	NEG	NEG	POS	POS	POS	POS	NEG	NEG	NEG	POS	POS	POS	POS	NEG	NEG	POS	POS	NEG	POS	POS	NEG	NEG	POS	NEG	POS	NEG	AMB	POS	NEG	NEG	NEG	POS	AMB	NEG	POS	POS	POS	NEG	POS
02V34748	CC5-MRSA-II, Rhine-Hesse EMRSA/UK-EMRSA-3	POS	POS	NEG	NEG	POS	NEG	NEG	POS	POS	NEG	NEG	POS	POS	POS	POS	NEG	NEG	NEG	POS	POS	POS	POS	NEG	NEG	POS	POS	NEG	POS	POS	NEG	NEG	POS	NEG	POS	NEG	AMB	POS	NEG	AMB	NEG	POS	POS	NEG	POS	POS	POS	NEG	POS
02V17002	CC8/ST254-MRSA-(atypical SCCmec), Hannover EMRSA	NEG	NEG	NEG	NEG	NEG	NEG	NEG	POS	POS	POS	AMB	AMB	POS	POS	POS	NEG	NEG	NEG	POS	POS	POS	POS	NEG	POS	POS	POS	NEG	POS	POS	POS	NEG	NEG	NEG	POS	AMB	POS	NEG	NEG	NEG	NEG	POS	POS	NEG	NEG	POS	POS	NEG	POS
02V33579	CC8-MRSA-IV, USA500	POS	POS	POS	NEG	POS	POS	AMB	POS	POS	POS	POS	POS	POS	POS	POS	NEG	NEG	NEG	POS	POS	POS	POS	NEG	POS	POS	POS	AMB	POS	POS	POS	NEG	NEG	NEG	POS	POS	POS	POS	NEG	NEG	NEG	POS	POS	NEG	POS	POS	POS	AMB	POS
02V15498	CC22-MRSA-IV [PVL+]	POS	POS	NEG	NEG	NEG	NEG	NEG	POS	POS	NEG	NEG	POS	POS	POS	NEG	NEG	NEG	POS	NEG	POS	POS	POS	NEG	POS	POS	NEG	POS	POS	POS	NEG	NEG	POS	NEG	POS	NEG	AMB	NEG	POS	NEG	NEG	NEG	NEG	NEG	NEG	POS	NEG	AMB	POS
02C05319	CC22-MRSA-IV, Barnim/UK-EMRSA-15	POS	POS	NEG	NEG	NEG	NEG	NEG	POS	POS	NEG	NEG	POS	POS	POS	NEG	NEG	POS	POS	NEG	POS	POS	POS	NEG	POS	POS	NEG	POS	POS	POS	NEG	NEG	POS	NEG	NEG	NEG	NEG	NEG	NEG	NEG	NEG	POS	NEG	NEG	POS	POS	NEG	POS	POS
02U10255	CC22-MRSA-IV, Barnim/UK-EMRSA-15	POS	POS	NEG	NEG	NEG	NEG	NEG	POS	POS	NEG	NEG	POS	POS	POS	NEG	NEG	POS	POS	NEG	POS	POS	POS	NEG	POS	POS	NEG	POS	POS	POS	NEG	NEG	POS	NEG	NEG	NEG	NEG	NEG	NEG	NEG	NEG	POS	NEG	NEG	POS	POS	NEG	POS	POS
02V12175	CC22-MRSA-IV, Barnim/UK-EMRSA-15	POS	POS	NEG	NEG	NEG	NEG	NEG	POS	POS	NEG	NEG	POS	POS	POS	NEG	NEG	POS	POS	NEG	POS	POS	POS	NEG	POS	POS	NEG	POS	POS	POS	NEG	NEG	POS	NEG	NEG	NEG	NEG	NEG	NEG	NEG	NEG	POS	NEG	NEG	POS	POS	NEG	POS	POS
02V13054	CC22-MRSA-IV, Barnim/UK-EMRSA-15	POS	POS	NEG	NEG	NEG	NEG	NEG	POS	POS	NEG	NEG	POS	POS	POS	NEG	NEG	POS	POS	NEG	POS	POS	POS	NEG	POS	POS	NEG	POS	POS	POS	NEG	NEG	POS	NEG	NEG	NEG	NEG	NEG	NEG	NEG	NEG	POS	NEG	NEG	POS	POS	NEG	POS	POS
02V13950	CC22-MRSA-IV, Barnim/UK-EMRSA-15	POS	POS	NEG	NEG	NEG	NEG	NEG	POS	POS	NEG	NEG	POS	POS	POS	NEG	NEG	POS	POS	NEG	POS	POS	POS	NEG	POS	POS	NEG	POS	POS	POS	NEG	NEG	POS	NEG	NEG	NEG	NEG	NEG	NEG	NEG	NEG	POS	NEG	NEG	POS	POS	NEG	POS	POS
02V21259	CC22-MRSA-IV, Barnim/UK-EMRSA-15	POS	POS	NEG	NEG	NEG	NEG	NEG	POS	POS	NEG	NEG	POS	POS	POS	NEG	NEG	POS	POS	NEG	POS	POS	POS	NEG	POS	POS	NEG	POS	POS	POS	NEG	NEG	POS	NEG	NEG	NEG	NEG	NEG	NEG	NEG	NEG	NEG	NEG	NEG	NEG	POS	NEG	POS	POS
02V22657	CC22-MRSA-IV, Barnim/UK-EMRSA-15	POS	POS	NEG	NEG	NEG	NEG	NEG	POS	POS	NEG	NEG	POS	POS	POS	NEG	NEG	NEG	POS	NEG	POS	NEG	POS	NEG	POS	POS	NEG	AMB	POS	POS	NEG	NEG	POS	NEG	NEG	NEG	NEG	NEG	NEG	NEG	NEG	POS	NEG	NEG	POS	POS	NEG	POS	POS
02V29051	CC22-MRSA-IV, Barnim/UK-EMRSA-15	POS	POS	NEG	NEG	NEG	NEG	NEG	POS	POS	NEG	NEG	POS	POS	POS	NEG	NEG	POS	POS	NEG	POS	POS	POS	NEG	POS	POS	NEG	POS	POS	POS	NEG	NEG	POS	NEG	NEG	NEG	NEG	NEG	NEG	NEG	NEG	POS	NEG	NEG	POS	POS	NEG	POS	POS
02V30792	CC30/ST36-MRSA-II, UK-EMRSA-16	POS	POS	NEG	POS	NEG	NEG	NEG	POS	POS	AMB	POS	NEG	POS	POS	POS	NEG	NEG	POS	POS	POS	POS	POS	NEG	NEG	POS	NEG	POS	POS	POS	NEG	POS	NEG	NEG	NEG	NEG	NEG	NEG	NEG	NEG	NEG	POS	NEG	POS	NEG	NEG	NEG	NEG	NEG
02U07096	CC45-MRSA-IV, Berlin EMRSA	POS	POS	NEG	NEG	NEG	NEG	POS	POS	POS	AMB	NEG	POS	POS	POS	NEG	POS	AMB	POS	POS	POS	NEG	POS	POS	NEG	POS	NEG	POS	POS	POS	NEG	NEG	POS	NEG	POS	NEG	POS	AMB	NEG	AMB	NEG	POS	NEG	POS	NEG	NEG	NEG	NEG	NEG
02V00570	CC45-MRSA-IV, Berlin EMRSA	POS	POS	NEG	NEG	NEG	NEG	POS	POS	POS	NEG	NEG	POS	POS	POS	NEG	POS	AMB	POS	POS	POS	NEG	POS	POS	NEG	POS	NEG	POS	POS	POS	NEG	NEG	POS	NEG	POS	NEG	POS	AMB	NEG	NEG	NEG	POS	NEG	POS	NEG	NEG	NEG	NEG	NEG
02V01730	CC45-MRSA-IV, Berlin EMRSA	POS	POS	NEG	NEG	NEG	NEG	POS	POS	POS	NEG	NEG	POS	POS	POS	NEG	POS	AMB	POS	POS	POS	NEG	POS	AMB	NEG	POS	NEG	POS	POS	POS	NEG	NEG	POS	NEG	POS	NEG	POS	AMB	NEG	NEG	NEG	POS	NEG	POS	NEG	NEG	NEG	NEG	NEG
02V01870	CC45-MRSA-IV, Berlin EMRSA	POS	POS	NEG	NEG	NEG	NEG	POS	POS	POS	AMB	NEG	POS	POS	POS	NEG	POS	AMB	POS	POS	POS	NEG	POS	POS	NEG	POS	NEG	POS	POS	POS	NEG	NEG	POS	NEG	POS	NEG	POS	AMB	NEG	NEG	NEG	POS	NEG	POS	NEG	NEG	NEG	NEG	NEG
02V04750	CC45-MRSA-IV, Berlin EMRSA	POS	POS	NEG	NEG	NEG	NEG	POS	POS	POS	AMB	NEG	POS	POS	POS	NEG	POS	AMB	POS	POS	POS	NEG	POS	POS	NEG	POS	NEG	POS	POS	POS	NEG	NEG	POS	NEG	POS	NEG	POS	AMB	NEG	AMB	NEG	POS	NEG	POS	NEG	NEG	NEG	NEG	NEG
02V06592	CC45-MRSA-IV, Berlin EMRSA	POS	POS	NEG	NEG	NEG	NEG	POS	POS	POS	AMB	NEG	POS	POS	POS	NEG	POS	AMB	POS	POS	POS	NEG	POS	POS	NEG	POS	NEG	POS	POS	POS	NEG	NEG	POS	NEG	POS	NEG	POS	AMB	NEG	NEG	NEG	POS	NEG	POS	NEG	NEG	NEG	NEG	NEG
02V10868	CC45-MRSA-IV, Berlin EMRSA	POS	POS	NEG	NEG	NEG	NEG	POS	POS	POS	AMB	NEG	POS	POS	POS	NEG	POS	AMB	POS	POS	POS	NEG	POS	POS	NEG	POS	NEG	POS	POS	POS	NEG	NEG	POS	NEG	POS	NEG	POS	AMB	NEG	NEG	NEG	POS	NEG	POS	NEG	NEG	NEG	NEG	NEG
02V12394	CC45-MRSA-IV, Berlin EMRSA	POS	POS	NEG	NEG	NEG	NEG	POS	POS	POS	AMB	NEG	POS	POS	POS	NEG	POS	AMB	POS	POS	POS	NEG	POS	POS	NEG	POS	NEG	POS	POS	POS	NEG	NEG	POS	NEG	POS	NEG	POS	AMB	NEG	NEG	NEG	POS	NEG	POS	NEG	NEG	NEG	NEG	NEG
02V12942	CC45-MRSA-IV, Berlin EMRSA	POS	POS	NEG	NEG	NEG	NEG	POS	POS	POS	AMB	NEG	POS	POS	POS	NEG	POS	AMB	POS	POS	POS	NEG	POS	POS	NEG	POS	NEG	POS	POS	POS	NEG	NEG	POS	NEG	POS	NEG	POS	AMB	NEG	NEG	NEG	POS	NEG	POS	NEG	NEG	NEG	NEG	NEG
02V13026	CC45-MRSA-IV, Berlin EMRSA	POS	POS	NEG	NEG	NEG	NEG	POS	POS	POS	AMB	NEG	POS	POS	POS	NEG	POS	AMB	POS	POS	POS	NEG	POS	POS	NEG	POS	NEG	POS	POS	POS	NEG	NEG	POS	NEG	POS	NEG	POS	AMB	NEG	AMB	NEG	POS	NEG	POS	NEG	NEG	NEG	NEG	NEG
02V14700	CC45-MRSA-IV, Berlin EMRSA																																																
02V15358	CC45-MRSA-IV, Berlin EMRSA	POS	POS	NEG	NEG	NEG	NEG	POS	POS	POS	AMB	NEG	POS	POS	POS	NEG	POS	AMB	POS	POS	POS	NEG	POS	POS	NEG	POS	NEG	POS	POS	POS	NEG	NEG	POS	NEG	POS	NEG	POS	AMB	NEG	NEG	NEG	POS	NEG	POS	NEG	NEG	NEG	NEG	NEG
02V16654	CC45-MRSA-IV, Berlin EMRSA	POS	POS	NEG	NEG	NEG	NEG	POS	POS	POS	AMB	NEG	POS	POS	POS	NEG	POS	AMB	POS	POS	POS	NEG	POS	POS	NEG	POS	NEG	POS	POS	POS	NEG	NEG	POS	NEG	POS	NEG	POS	AMB	NEG	NEG	NEG	POS	NEG	POS	NEG	NEG	NEG	NEG	NEG
02V17120	CC45-MRSA-IV, Berlin EMRSA	POS	POS	AMB	NEG	NEG	NEG	POS	POS	POS	POS	NEG	AMB	POS	POS	NEG	POS	AMB	POS	POS	POS	NEG	POS	POS	NEG	POS	NEG	POS	POS	POS	NEG	NEG	POS	NEG	POS	NEG	AMB	POS	NEG	AMB	NEG	POS	NEG	POS	NEG	NEG	NEG	NEG	NEG
02V17136	CC45-MRSA-IV, Berlin EMRSA	POS	POS	NEG	NEG	NEG	NEG	POS	POS	POS	AMB	NEG	POS	POS	POS	NEG	POS	AMB	POS	POS	POS	NEG	POS	POS	NEG	POS	NEG	POS	POS	POS	NEG	NEG	POS	NEG	POS	NEG	POS	AMB	NEG	NEG	NEG	POS	NEG	POS	NEG	NEG	NEG	NEG	NEG
02V17558	CC45-MRSA-IV, Berlin EMRSA	POS	POS	NEG	NEG	NEG	NEG	POS	POS	POS	AMB	NEG	POS	POS	POS	NEG	POS	AMB	POS	POS	POS	NEG	POS	POS	NEG	POS	NEG	POS	POS	POS	NEG	NEG	POS	NEG	POS	NEG	POS	AMB	NEG	NEG	NEG	POS	NEG	POS	NEG	NEG	NEG	NEG	NEG
02V17665	CC45-MRSA-IV, Berlin EMRSA	POS	POS	NEG	NEG	NEG	NEG	POS	POS	POS	NEG	NEG	POS	POS	POS	NEG	POS	AMB	POS	POS	POS	NEG	POS	AMB	NEG	POS	NEG	POS	POS	POS	NEG	NEG	POS	NEG	POS	NEG	POS	AMB	NEG	NEG	NEG	POS	NEG	POS	NEG	NEG	NEG	NEG	NEG
02V20390	CC45-MRSA-IV, Berlin EMRSA	POS	POS	NEG	NEG	NEG	NEG	POS	POS	POS	NEG	NEG	POS	POS	POS	NEG	POS	AMB	POS	POS	POS	NEG	POS	POS	NEG	POS	NEG	AMB	POS	POS	NEG	NEG	POS	NEG	POS	NEG	POS	AMB	NEG	NEG	NEG	POS	NEG	POS	NEG	NEG	NEG	NEG	NEG
02V20626	CC45-MRSA-IV, Berlin EMRSA	POS	POS	NEG	NEG	NEG	NEG	POS	POS	POS	AMB	NEG	POS	POS	POS	NEG	POS	AMB	POS	POS	POS	NEG	POS	AMB	NEG	POS	NEG	POS	POS	POS	NEG	NEG	POS	NEG	POS	NEG	POS	AMB	NEG	NEG	NEG	POS	NEG	POS	NEG	NEG	NEG	NEG	NEG
02V20680	CC45-MRSA-IV, Berlin EMRSA	POS	POS	NEG	NEG	NEG	NEG	POS	POS	POS	NEG	NEG	POS	POS	POS	NEG	POS	AMB	POS	POS	POS	NEG	POS	AMB	NEG	POS	NEG	AMB	POS	POS	NEG	NEG	AMB	NEG	POS	NEG	POS	AMB	NEG	NEG	NEG	POS	NEG	POS	NEG	NEG	NEG	NEG	NEG
02V21980	CC45-MRSA-IV, Berlin EMRSA	POS	POS	NEG	NEG	NEG	NEG	POS	POS	POS	NEG	NEG	POS	POS	POS	NEG	POS	AMB	POS	POS	POS	NEG	POS	AMB	NEG	POS	NEG	POS	POS	POS	NEG	NEG	POS	NEG	POS	NEG	POS	AMB	NEG	NEG	NEG	POS	NEG	POS	NEG	NEG	NEG	NEG	NEG
02V22570	CC45-MRSA-IV, Berlin EMRSA	POS	POS	NEG	NEG	NEG	NEG	POS	POS	POS	AMB	NEG	POS	POS	POS	NEG	POS	AMB	POS	POS	POS	NEG	POS	POS	NEG	POS	NEG	POS	POS	POS	NEG	NEG	POS	NEG	POS	NEG	POS	AMB	NEG	AMB	NEG	POS	NEG	POS	NEG	NEG	NEG	NEG	NEG
02V22982	CC45-MRSA-IV, Berlin EMRSA	POS	POS	AMB	NEG	NEG	NEG	POS	POS	POS	AMB	AMB	POS	POS	POS	NEG	POS	AMB	POS	POS	POS	NEG	POS	POS	NEG	POS	NEG	POS	POS	POS	NEG	NEG	POS	NEG	POS	NEG	POS	AMB	NEG	AMB	NEG	POS	NEG	POS	NEG	NEG	NEG	NEG	NEG
02V25503	CC45-MRSA-IV, Berlin EMRSA	POS	POS	NEG	NEG	NEG	NEG	POS	POS	POS	AMB	NEG	POS	POS	POS	NEG	POS	AMB	POS	POS	POS	NEG	POS	POS	NEG	POS	NEG	POS	POS	POS	NEG	NEG	POS	NEG	POS	NEG	POS	AMB	NEG	AMB	NEG	POS	NEG	POS	NEG	NEG	NEG	NEG	NEG
02V28454	CC45-MRSA-IV, Berlin EMRSA	POS	POS	AMB	NEG	NEG	NEG	POS	POS	POS	AMB	NEG	POS	POS	POS	NEG	POS	AMB	POS	POS	POS	NEG	POS	POS	NEG	POS	NEG	POS	POS	POS	NEG	NEG	POS	NEG	POS	NEG	POS	AMB	NEG	AMB	NEG	POS	NEG	POS	NEG	NEG	NEG	NEG	NEG
02V29373	CC45-MRSA-IV, Berlin EMRSA	POS	POS	NEG	NEG	NEG	NEG	POS	POS	POS	AMB	NEG	POS	POS	POS	NEG	POS	AMB	POS	POS	POS	NEG	POS	POS	NEG	POS	NEG	POS	POS	POS	NEG	NEG	POS	NEG	POS	NEG	POS	AMB	NEG	AMB	NEG	POS	NEG	POS	NEG	NEG	NEG	NEG	NEG
02V30321	CC45-MRSA-IV, Berlin EMRSA	POS	POS	AMB	NEG	NEG	NEG	POS	POS	POS	AMB	NEG	POS	POS	POS	NEG	POS	AMB	POS	POS	POS	NEG	POS	POS	NEG	POS	NEG	POS	POS	POS	NEG	NEG	POS	NEG	POS	NEG	POS	AMB	NEG	AMB	NEG	POS	NEG	POS	NEG	NEG	NEG	NEG	NEG
02V30341	CC45-MRSA-IV, Berlin EMRSA	POS	POS	NEG	NEG	NEG	NEG	POS	POS	POS	AMB	NEG	POS	POS	POS	NEG	POS	AMB	POS	POS	POS	NEG	POS	POS	NEG	POS	NEG	POS	POS	POS	NEG	NEG	POS	NEG	POS	NEG	POS	AMB	NEG	NEG	NEG	POS	NEG	POS	NEG	NEG	NEG	NEG	NEG
02V31377	CC45-MRSA-IV, Berlin EMRSA	POS	POS	NEG	NEG	NEG	NEG	POS	POS	POS	AMB	NEG	POS	POS	POS	NEG	POS	AMB	POS	POS	POS	NEG	POS	POS	NEG	POS	NEG	POS	POS	POS	NEG	NEG	POS	NEG	POS	NEG	AMB	POS	NEG	AMB	NEG	POS	NEG	POS	NEG	NEG	NEG	NEG	NEG
02V31941	CC45-MRSA-IV, Berlin EMRSA	POS	POS	AMB	NEG	NEG	NEG	POS	POS	POS	AMB	NEG	POS	POS	POS	NEG	POS	AMB	POS	POS	POS	NEG	POS	POS	NEG	POS	NEG	NEG	POS	POS	NEG	NEG	POS	NEG	POS	NEG	POS	AMB	NEG	AMB	NEG	POS	NEG	POS	NEG	NEG	NEG	NEG	NEG
02V34679	CC45-MRSA-IV, Berlin EMRSA	POS	POS	NEG	NEG	NEG	NEG	POS	POS	POS	NEG	NEG	POS	POS	POS	NEG	POS	AMB	POS	POS	POS	NEG	POS	AMB	NEG	POS	NEG	AMB	POS	POS	NEG	NEG	POS	NEG	POS	NEG	POS	AMB	NEG	NEG	NEG	POS	NEG	POS	NEG	NEG	NEG	NEG	NEG
02V35779	CC45-MRSA-IV, Berlin EMRSA	POS	POS	NEG	NEG	NEG	NEG	POS	POS	POS	NEG	NEG	POS	POS	POS	NEG	POS	POS	POS	POS	POS	NEG	POS	AMB	NEG	POS	NEG	POS	POS	NEG	NEG	NEG	POS	NEG	POS	NEG	POS	POS	NEG	NEG	NEG	POS	NEG	POS	NEG	NEG	NEG	NEG	NEG
02V36419	CC45-MRSA-IV, Berlin EMRSA																																																
02V36469	CC45-MRSA-IV, Berlin EMRSA	POS	POS	AMB	NEG	AMB	NEG	POS	POS	POS	POS	NEG	AMB	POS	POS	NEG	POS	AMB	POS	POS	POS	NEG	POS	POS	NEG	POS	NEG	POS	POS	POS	NEG	NEG	POS	NEG	POS	NEG	AMB	POS	NEG	AMB	NEG	POS	NEG	POS	NEG	NEG	NEG	NEG	NEG

ISOLATE:	STRAIN	ADHAESION FACTORS / GENES ENCODING MICROBIAL SURFACE COMPONENTS RECOGNIZING ADHESIVE MATRIX MOLECULES (MSCRAMM GENES)																		IMMUNOD.ANTIGEN B		DEFENSIN RESIST.		TRANSFERRIN BINDING PROT			PUTATIVE TRANSPORTER		TYPE I RESTRICTION-MODIFICATION SYSTEM, SINGLE SEQUENCE SPECIFICITY PROTEIN												MISCELLANEOUS GENES			HYALURONATE LYASE							
		Ser-Asp rich fibrinogen-/bone sialoprotein-binding protein C							Ser-Asp rich fibrinogen-/bone sialoprotein-binding protein D					van Willebrand factor binding protein						immunodominant antigen B		defensin resistance protein		transferrin-binding protein			hypothetical protein, similar to integral membrane protein LmrP		type I site-specific deoxyribo-nuclease subunit, 1st locus	type I site-specific deoxyribonuclease subunit, 2nd locus			type I site-specific deoxyribonuclease subunit, 3rd locus					type I site-specific deoxyribonuclease subunit, unknown locus			hypothetical protein, located next to serine protease operon	Unspecific efflux/trans-porter	hypothetical protein	hyaluronate lyase, first / second locus			hyaluronate lyase, second locus				
		sdrC (total)	sdrC (consensus)	sdrC (B1)	sdrC (COL)	sdrC (Mu50)	sdrC (MW2+MRSA252+RF122)	sdrC (other than MRSA252+RF122)	sdrD (total)	sdrD (consensus)	sdrD (COL+MW2)	sdrD (Mu50)	sdrD (other)	vwb (total)	vwb (consensus)	vwb (COL+MW2)	vwb (MRSA252)	vwb (Mu50)	vwb (RF122)	isaB	isaB (MRSA252)	mprF (COL+MW2)	mprF (Mu50+MRSA252)	isdA (consensus)	isdA (MRSA252)	isdA (other than MRSA252)	lmrP (other than RF122)	lmrP (RF122)	hsdS1 (RF122)	hsdS2 (Mu50+N315+COL+USA300+NCTC8325)	hsdS2 (MW2+MSSA476)	hsdS2 (MRSA252)	hsdS3 (all other than RF122+MRSA252)	hsdS3 (COL+USA300+NCTC8325+MW2+MSSA476+RF122)	hsdS3 (Mu50+N315)	hsdS3 (CCS1+MRSA252)	hsdS3 (MRSA252)	hsdSx (CC25)	hsdSx (CC15)	hsdSx (etd)	Q2FXC0	Q2YUB3	Q7A4X2	hysA1 (MRSA252)	hysA1 (MRSA252+RF122) and/or hysA2 (consensus)	hysA1 (MRSA252+RF122) and/or hysA2 (COL+USA300)	hysA2 (all other than MRSA252)	hysA2 (COL+USA300+NCTC8325)	hysA2 (all other than COL+USA300+NCTC8325)	hysA2 (all other than COL+USA300+NCTC8325)	hysA2 (MRSA252)
**2000**																																																			
00V07201	CC5/ST228-MRSA, South German EMRSA (truncated SCCmec element)	POS	POS	NEG	NEG	POS	NEG	POS	POS	POS	NEG	POS	NEG	POS	POS	NEG	NEG	POS	NEG	POS	AMB	AMB	POS	POS	NEG	POS	POS	NEG	NEG	NEG	NEG	NEG	POS	NEG	POS	NEG	NEG	POS	NEG	NEG	NEG	NEG	POS	NEG	POS	NEG	POS	NEG	POS	POS	NEG
00V07634	CC5/ST228-MRSA, South German EMRSA (truncated SCCmec element)	POS	POS	NEG	NEG	POS	NEG	POS	POS	POS	NEG	POS	NEG	POS	POS	NEG	NEG	POS	NEG	POS	AMB	AMB	POS	POS	NEG	POS	POS	NEG	NEG	NEG	AMB	NEG	POS	NEG	POS	NEG	NEG	POS	NEG	NEG	NEG	NEG	POS	NEG	POS	NEG	POS	NEG	POS	POS	AMB
00V08701	CC5/ST228-MRSA, South German EMRSA (truncated SCCmec element)	POS	POS	NEG	NEG	POS	NEG	POS	POS	POS	NEG	POS	NEG	POS	POS	NEG	NEG	POS	NEG	POS	AMB	AMB	POS	POS	NEG	POS	POS	NEG	NEG	NEG	NEG	NEG	POS	NEG	POS	NEG	NEG	POS	NEG	NEG	NEG	NEG	POS	NEG	POS	NEG	POS	NEG	POS	POS	NEG
00V09056	CC5/ST228-MRSA, South German EMRSA (truncated SCCmec element)	POS	POS	NEG	NEG	POS	NEG	POS	POS	POS	NEG	POS	NEG	POS	POS	NEG	NEG	POS	NEG	POS	AMB	AMB	POS	POS	NEG	POS	POS	NEG	NEG	NEG	NEG	NEG	POS	NEG	POS	NEG	NEG	POS	NEG	NEG	NEG	NEG	POS	NEG	POS	NEG	POS	NEG	POS	POS	NEG
00V10190	CC5/ST228-MRSA, South German EMRSA (truncated SCCmec element)	POS	POS	NEG	NEG	POS	AMB	POS	POS	POS	NEG	POS	NEG	POS	POS	NEG	NEG	POS	NEG	POS	AMB	POS	AMB	POS	AMB	POS	POS	NEG	NEG	NEG	NEG	NEG	POS	NEG	POS	AMB	NEG	POS	AMB	NEG	NEG	POS	POS	NEG	POS	NEG	POS	NEG	POS	POS	AMB
00V10643	CC5/ST228-MRSA, South German EMRSA (truncated SCCmec element)	POS	POS	NEG	NEG	POS	NEG	POS	POS	POS	NEG	POS	NEG	POS	POS	NEG	NEG	POS	NEG	POS	AMB	AMB	POS	POS	NEG	POS	POS	NEG	NEG	NEG	NEG	NEG	POS	NEG	POS	NEG	NEG	POS	NEG	NEG	NEG	AMB	POS	NEG	POS	NEG	POS	NEG	POS	POS	NEG
00V11732	CC5/ST228-MRSA, South German EMRSA (truncated SCCmec element)	POS	POS	NEG	NEG	POS	NEG	POS	POS	POS	NEG	POS	NEG	POS	POS	NEG	NEG	POS	NEG	POS	AMB	AMB	POS	POS	NEG	POS	POS	NEG	NEG	NEG	NEG	NEG	POS	NEG	POS	NEG	NEG	POS	NEG	NEG	NEG	NEG	POS	NEG	POS	NEG	POS	NEG	POS	POS	NEG
00V12300	CC5/ST228-MRSA, South German EMRSA (truncated SCCmec element)	POS	POS	NEG	NEG	POS	NEG	POS	POS	POS	NEG	POS	NEG	POS	POS	NEG	NEG	POS	NEG	POS	AMB	AMB	POS	POS	NEG	POS	POS	NEG	NEG	NEG	NEG	NEG	POS	NEG	POS	NEG	NEG	POS	NEG	NEG	NEG	NEG	POS	NEG	POS	NEG	POS	NEG	POS	POS	NEG
00V14652	CC5/ST228-MRSA, South German EMRSA (truncated SCCmec element)	POS	[illegible]	[illegible]	[illegible]	[illegible]	[illegible]	[illegible]	[illegible]	[illegible]	[illegible]	[illegible]	[illegible]	[illegible]	[illegible]	[illegible]	[illegible]	[illegible]	[illegible]	[illegible]	[illegible]	[illegible]	[illegible]	[illegible]	[illegible]	[illegible]	[illegible]	[illegible]	[illegible]	[illegible]	[illegible]	[illegible]	[illegible]	[illegible]	[illegible]	[illegible]	[illegible]	[illegible]	[illegible]	[illegible]	[illegible]	[illegible]	[illegible]	[illegible]	[illegible]	[illegible]	[illegible]	[illegible]	[illegible]	[illegible]	[illegible]
00V24941	CC5/ST228-MRSA, South German EMRSA (truncated SCCmec element)	POS	[illegible]	[illegible]	[illegible]	[illegible]	[illegible]	[illegible]	[illegible]	[illegible]	[illegible]	[illegible]	[illegible]	[illegible]	[illegible]	[illegible]	[illegible]	[illegible]	[illegible]	[illegible]	[illegible]	[illegible]	[illegible]	[illegible]	[illegible]	[illegible]	[illegible]	[illegible]	[illegible]	[illegible]	[illegible]	[illegible]	[illegible]	[illegible]	[illegible]	[illegible]	[illegible]	[illegible]	[illegible]	[illegible]	[illegible]	[illegible]	[illegible]	[illegible]	[illegible]	[illegible]	[illegible]	[illegible]	[illegible]	[illegible]	[illegible]
00V29834	CC5/ST228-MRSA, South German EMRSA (truncated SCCmec element)	POS	[illegible]	[illegible]	[illegible]	[illegible]	[illegible]	[illegible]	[illegible]	[illegible]	[illegible]	[illegible]	[illegible]	[illegible]	[illegible]	[illegible]	[illegible]	[illegible]	[illegible]	[illegible]	[illegible]	[illegible]	[illegible]	[illegible]	[illegible]	[illegible]	[illegible]	[illegible]	[illegible]	[illegible]	[illegible]	[illegible]	[illegible]	[illegible]	[illegible]	[illegible]	[illegible]	[illegible]	[illegible]	[illegible]	[illegible]	[illegible]	[illegible]	[illegible]	[illegible]	[illegible]	[illegible]	[illegible]	[illegible]	[illegible]	[illegible]
00V29909	CC5/ST228-MRSA, South German EMRSA (truncated SCCmec element)	POS	[illegible]	[illegible]	[illegible]	[illegible]	[illegible]	[illegible]	[illegible]	[illegible]	[illegible]	[illegible]	[illegible]	[illegible]	[illegible]	[illegible]	[illegible]	[illegible]	[illegible]	[illegible]	[illegible]	[illegible]	[illegible]	[illegible]	[illegible]	[illegible]	[illegible]	[illegible]	[illegible]	[illegible]	[illegible]	[illegible]	[illegible]	[illegible]	[illegible]	[illegible]	[illegible]	[illegible]	[illegible]	[illegible]	[illegible]	[illegible]	[illegible]	[illegible]	[illegible]	[illegible]	[illegible]	[illegible]	[illegible]	[illegible]	[illegible]
00V30456	CC5/ST228-MRSA, South German EMRSA (truncated SCCmec element)	POS	[illegible]	[illegible]	[illegible]	[illegible]	[illegible]	[illegible]	[illegible]	[illegible]	[illegible]	[illegible]	[illegible]	[illegible]	[illegible]	[illegible]	[illegible]	[illegible]	[illegible]	[illegible]	[illegible]	[illegible]	[illegible]	[illegible]	[illegible]	[illegible]	[illegible]	[illegible]	[illegible]	[illegible]	[illegible]	[illegible]	[illegible]	[illegible]	[illegible]	[illegible]	[illegible]	[illegible]	[illegible]	[illegible]	[illegible]	[illegible]	[illegible]	[illegible]	[illegible]	[illegible]	[illegible]	[illegible]	[illegible]	[illegible]	[illegible]
00V30827	CC5/ST228-MRSA, South German EMRSA (truncated SCCmec element)	POS	[illegible]	[illegible]	[illegible]	[illegible]	[illegible]	[illegible]	[illegible]	[illegible]	[illegible]	[illegible]	[illegible]	[illegible]	[illegible]	[illegible]	[illegible]	[illegible]	[illegible]	[illegible]	[illegible]	[illegible]	[illegible]	[illegible]	[illegible]	[illegible]	[illegible]	[illegible]	[illegible]	[illegible]	[illegible]	[illegible]	[illegible]	[illegible]	[illegible]	[illegible]	[illegible]	[illegible]	[illegible]	[illegible]	[illegible]	[illegible]	[illegible]	[illegible]	[illegible]	[illegible]	[illegible]	[illegible]	[illegible]	[illegible]	[illegible]
00V33204	CC5/ST228-MRSA, South German EMRSA (truncated SCCmec element)	POS	[illegible]	[illegible]	[illegible]	[illegible]	[illegible]	[illegible]	[illegible]	[illegible]	[illegible]	[illegible]	[illegible]	[illegible]	[illegible]	[illegible]	[illegible]	[illegible]	[illegible]	[illegible]	[illegible]	[illegible]	[illegible]	[illegible]	[illegible]	[illegible]	[illegible]	[illegible]	[illegible]	[illegible]	[illegible]	[illegible]	[illegible]	[illegible]	[illegible]	[illegible]	[illegible]	[illegible]	[illegible]	[illegible]	[illegible]	[illegible]	[illegible]	[illegible]	[illegible]	[illegible]	[illegible]	[illegible]	[illegible]	[illegible]	[illegible]
00V36490	CC5/ST228-MRSA, South German EMRSA (truncated SCCmec element)	POS	[illegible]	[illegible]	[illegible]	[illegible]	[illegible]	[illegible]	[illegible]	[illegible]	[illegible]	[illegible]	[illegible]	[illegible]	[illegible]	[illegible]	[illegible]	[illegible]	[illegible]	[illegible]	[illegible]	[illegible]	[illegible]	[illegible]	[illegible]	[illegible]	[illegible]	[illegible]	[illegible]	[illegible]	[illegible]	[illegible]	[illegible]	[illegible]	[illegible]	[illegible]	[illegible]	[illegible]	[illegible]	[illegible]	[illegible]	[illegible]	[illegible]	[illegible]	[illegible]	[illegible]	[illegible]	[illegible]	[illegible]	[illegible]	[illegible]
00V37079	CC5/ST228-MRSA, South German EMRSA (truncated SCCmec element)	POS	[illegible]	[illegible]	[illegible]	[illegible]	[illegible]	[illegible]	[illegible]	[illegible]	[illegible]	[illegible]	[illegible]	[illegible]	[illegible]	[illegible]	[illegible]	[illegible]	[illegible]	[illegible]	[illegible]	[illegible]	[illegible]	[illegible]	[illegible]	[illegible]	[illegible]	[illegible]	[illegible]	[illegible]	[illegible]	[illegible]	[illegible]	[illegible]	[illegible]	[illegible]	[illegible]	[illegible]	[illegible]	[illegible]	[illegible]	[illegible]	[illegible]	[illegible]	[illegible]	[illegible]	[illegible]	[illegible]	[illegible]	[illegible]	[illegible]
00V40116	CC5/ST228-MRSA, South German EMRSA (truncated SCCmec element)	POS	[illegible]	[illegible]	[illegible]	[illegible]	[illegible]	[illegible]	[illegible]	[illegible]	[illegible]	[illegible]	[illegible]	[illegible]	[illegible]	[illegible]	[illegible]	[illegible]	[illegible]	[illegible]	[illegible]	[illegible]	[illegible]	[illegible]	[illegible]	[illegible]	[illegible]	[illegible]	[illegible]	[illegible]	[illegible]	[illegible]	[illegible]	[illegible]	[illegible]	[illegible]	[illegible]	[illegible]	[illegible]	[illegible]	[illegible]	[illegible]	[illegible]	[illegible]	[illegible]	[illegible]	[illegible]	[illegible]	[illegible]	[illegible]	[illegible]
00V41429	CC5/ST228-MRSA, South German EMRSA (truncated SCCmec element)	POS	[illegible]	[illegible]	[illegible]	[illegible]	[illegible]	[illegible]	[illegible]	[illegible]	[illegible]	[illegible]	[illegible]	[illegible]	[illegible]	[illegible]	[illegible]	[illegible]	[illegible]	[illegible]	[illegible]	[illegible]	[illegible]	[illegible]	[illegible]	[illegible]	[illegible]	[illegible]	[illegible]	[illegible]	[illegible]	[illegible]	[illegible]	[illegible]	[illegible]	[illegible]	[illegible]	[illegible]	[illegible]	[illegible]	[illegible]	[illegible]	[illegible]	[illegible]	[illegible]	[illegible]	[illegible]	[illegible]	[illegible]	[illegible]	[illegible]
00V42118	CC5/ST228-MRSA, South German EMRSA (truncated SCCmec element)	POS	[illegible]	[illegible]	[illegible]	[illegible]	[illegible]	[illegible]	[illegible]	[illegible]	[illegible]	[illegible]	[illegible]	[illegible]	[illegible]	[illegible]	[illegible]	[illegible]	[illegible]	[illegible]	[illegible]	[illegible]	[illegible]	[illegible]	[illegible]	[illegible]	[illegible]	[illegible]	[illegible]	[illegible]	[illegible]	[illegible]	[illegible]	[illegible]	[illegible]	[illegible]	[illegible]	[illegible]	[illegible]	[illegible]	[illegible]	[illegible]	[illegible]	[illegible]	[illegible]	[illegible]	[illegible]	[illegible]	[illegible]	[illegible]	[illegible]
00V44135	CC5/ST228-MRSA, South German EMRSA (truncated SCCmec element)	POS	[illegible]	[illegible]	[illegible]	[illegible]	[illegible]	[illegible]	[illegible]	[illegible]	[illegible]	[illegible]	[illegible]	[illegible]	[illegible]	[illegible]	[illegible]	[illegible]	[illegible]	[illegible]	[illegible]	[illegible]	[illegible]	[illegible]	[illegible]	[illegible]	[illegible]	[illegible]	[illegible]	[illegible]	[illegible]	[illegible]	[illegible]	[illegible]	[illegible]	[illegible]	[illegible]	[illegible]	[illegible]	[illegible]	[illegible]	[illegible]	[illegible]	[illegible]	[illegible]	[illegible]	[illegible]	[illegible]	[illegible]	[illegible]	[illegible]
00V44538	CC5/ST228-MRSA, South German EMRSA (truncated SCCmec element)	POS	[illegible]	[illegible]	[illegible]	[illegible]	[illegible]	[illegible]	[illegible]	[illegible]	[illegible]	[illegible]	[illegible]	[illegible]	[illegible]	[illegible]	[illegible]	[illegible]	[illegible]	[illegible]	[illegible]	[illegible]	[illegible]	[illegible]	[illegible]	[illegible]	[illegible]	[illegible]	[illegible]	[illegible]	[illegible]	[illegible]	[illegible]	[illegible]	[illegible]	[illegible]	[illegible]	[illegible]	[illegible]	[illegible]	[illegible]	[illegible]	[illegible]	[illegible]	[illegible]	[illegible]	[illegible]	[illegible]	[illegible]	[illegible]	[illegible]
00V44990	CC5/ST228-MRSA, South German EMRSA (truncated SCCmec element)	POS	[illegible]	[illegible]	[illegible]	[illegible]	[illegible]	[illegible]	[illegible]	[illegible]	[illegible]	[illegible]	[illegible]	[illegible]	[illegible]	[illegible]	[illegible]	[illegible]	[illegible]	[illegible]	[illegible]	[illegible]	[illegible]	[illegible]	[illegible]	[illegible]	[illegible]	[illegible]	[illegible]	[illegible]	[illegible]	[illegible]	[illegible]	[illegible]	[illegible]	[illegible]	[illegible]	[illegible]	[illegible]	[illegible]	[illegible]	[illegible]	[illegible]	[illegible]	[illegible]	[illegible]	[illegible]	[illegible]	[illegible]	[illegible]	[illegible]
00V46910	CC5/ST228-MRSA, South German EMRSA (truncated SCCmec element)	POS	[illegible]	[illegible]	[illegible]	[illegible]	[illegible]	[illegible]	[illegible]	[illegible]	[illegible]	[illegible]	[illegible]	[illegible]	[illegible]	[illegible]	[illegible]	[illegible]	[illegible]	[illegible]	[illegible]	[illegible]	[illegible]	[illegible]	[illegible]	[illegible]	[illegible]	[illegible]	[illegible]	[illegible]	[illegible]	[illegible]	[illegible]	[illegible]	[illegible]	[illegible]	[illegible]	[illegible]	[illegible]	[illegible]	[illegible]	[illegible]	[illegible]	[illegible]	[illegible]	[illegible]	[illegible]	[illegible]	[illegible]	[illegible]	[illegible]
00V07063	CC5/ST228-MRSA-I, South German EMRSA	POS	[illegible]	[illegible]	[illegible]	[illegible]	[illegible]	[illegible]	[illegible]	[illegible]	[illegible]	[illegible]	[illegible]	[illegible]	[illegible]	[illegible]	[illegible]	[illegible]	[illegible]	[illegible]	[illegible]	[illegible]	[illegible]	[illegible]	[illegible]	[illegible]	[illegible]	[illegible]	[illegible]	[illegible]	[illegible]	[illegible]	[illegible]	[illegible]	[illegible]	[illegible]	[illegible]	[illegible]	[illegible]	[illegible]	[illegible]	[illegible]	[illegible]	[illegible]	[illegible]	[illegible]	[illegible]	[illegible]	[illegible]	[illegible]	[illegible]
00V46921	CC5-MRSA-II, Rhine-Hesse EMRSA/UK-EMRSA-3	POS	[illegible]	[illegible]	[illegible]	[illegible]	[illegible]	[illegible]	[illegible]	[illegible]	[illegible]	[illegible]	[illegible]	[illegible]	[illegible]	[illegible]	[illegible]	[illegible]	[illegible]	[illegible]	[illegible]	[illegible]	[illegible]	[illegible]	[illegible]	[illegible]	[illegible]	[illegible]	[illegible]	[illegible]	[illegible]	[illegible]	[illegible]	[illegible]	[illegible]	[illegible]	[illegible]	[illegible]	[illegible]	[illegible]	[illegible]	[illegible]	[illegible]	[illegible]	[illegible]	[illegible]	[illegible]	[illegible]	[illegible]	[illegible]	[illegible]
00V08510	CC8/ST254-MRSA-(atypical SCCmec), Hannover EMRSA	POS	[illegible]	[illegible]	[illegible]	[illegible]	[illegible]	[illegible]	[illegible]	[illegible]	[illegible]	[illegible]	[illegible]	[illegible]	[illegible]	[illegible]	[illegible]	[illegible]	[illegible]	[illegible]	[illegible]	[illegible]	[illegible]	[illegible]	[illegible]	[illegible]	[illegible]	[illegible]	[illegible]	[illegible]	[illegible]	[illegible]	[illegible]	[illegible]	[illegible]	[illegible]	[illegible]	[illegible]	[illegible]	[illegible]	[illegible]	[illegible]	[illegible]	[illegible]	[illegible]	[illegible]	[illegible]	[illegible]	[illegible]	[illegible]	[illegible]
00V25538	CC8/ST254-MRSA-(atypical SCCmec), Hannover EMRSA	POS	[illegible]	[illegible]	[illegible]	[illegible]	[illegible]	[illegible]	[illegible]	[illegible]	[illegible]	[illegible]	[illegible]	[illegible]	[illegible]	[illegible]	[illegible]	[illegible]	[illegible]	[illegible]	[illegible]	[illegible]	[illegible]	[illegible]	[illegible]	[illegible]	[illegible]	[illegible]	[illegible]	[illegible]	[illegible]	[illegible]	[illegible]	[illegible]	[illegible]	[illegible]	[illegible]	[illegible]	[illegible]	[illegible]	[illegible]	[illegible]	[illegible]	[illegible]	[illegible]	[illegible]	[illegible]	[illegible]	[illegible]	[illegible]	[illegible]
00V38664	CC8/ST254-MRSA-(atypical SCCmec), Hannover EMRSA	POS	[illegible]	[illegible]	[illegible]	[illegible]	[illegible]	[illegible]	[illegible]	[illegible]	[illegible]	[illegible]	[illegible]	[illegible]	[illegible]	[illegible]	[illegible]	[illegible]	[illegible]	[illegible]	[illegible]	[illegible]	[illegible]	[illegible]	[illegible]	[illegible]	[illegible]	[illegible]	[illegible]	[illegible]	[illegible]	[illegible]	[illegible]	[illegible]	[illegible]	[illegible]	[illegible]	[illegible]	[illegible]	[illegible]	[illegible]	[illegible]	[illegible]	[illegible]	[illegible]	[illegible]	[illegible]	[illegible]	[illegible]	[illegible]	[illegible]
00V46488	CC8/ST254-MRSA-(atypical SCCmec), Hannover EMRSA	POS	[illegible]	[illegible]	[illegible]	[illegible]	[illegible]	[illegible]	[illegible]	[illegible]	[illegible]	[illegible]	[illegible]	[illegible]	[illegible]	[illegible]	[illegible]	[illegible]	[illegible]	[illegible]	[illegible]	[illegible]	[illegible]	[illegible]	[illegible]	[illegible]	[illegible]	[illegible]	[illegible]	[illegible]	[illegible]	[illegible]	[illegible]	[illegible]	[illegible]	[illegible]	[illegible]	[illegible]	[illegible]	[illegible]	[illegible]	[illegible]	[illegible]	[illegible]	[illegible]	[illegible]	[illegible]	[illegible]	[illegible]	[illegible]	[illegible]
00V44035	CC8/ST254-MRSA-IV/V, UK-EMRSA-10/Hannover EMRSA	POS	[illegible]	[illegible]	[illegible]	[illegible]	[illegible]	[illegible]	[illegible]	[illegible]	[illegible]	[illegible]	[illegible]	[illegible]	[illegible]	[illegible]	[illegible]	[illegible]	[illegible]	[illegible]	[illegible]	[illegible]	[illegible]	[illegible]	[illegible]	[illegible]	[illegible]	[illegible]	[illegible]	[illegible]	[illegible]	[illegible]	[illegible]	[illegible]	[illegible]	[illegible]	[illegible]	[illegible]	[illegible]	[illegible]	[illegible]	[illegible]	[illegible]	[illegible]	[illegible]	[illegible]	[illegible]	[illegible]	[illegible]	[illegible]	[illegible]
00V13324	CC22-MRSA-IV [PVL+]	POS	[illegible]	[illegible]	[illegible]	[illegible]	[illegible]	[illegible]	[illegible]	[illegible]	[illegible]	[illegible]	[illegible]	[illegible]	[illegible]	[illegible]	[illegible]	[illegible]	[illegible]	[illegible]	[illegible]	[illegible]	[illegible]	[illegible]	[illegible]	[illegible]	[illegible]	[illegible]	[illegible]	[illegible]	[illegible]	[illegible]	[illegible]	[illegible]	[illegible]	[illegible]	[illegible]	[illegible]	[illegible]	[illegible]	[illegible]	[illegible]	[illegible]	[illegible]	[illegible]	[illegible]	[illegible]	[illegible]	[illegible]	[illegible]	[illegible]
00B02046	CC45-MRSA-IV, Berlin EMRSA	POS	[illegible]	[illegible]	[illegible]	[illegible]	[illegible]	[illegible]	[illegible]	[illegible]	[illegible]	[illegible]	[illegible]	[illegible]	[illegible]	[illegible]	[illegible]	[illegible]	[illegible]	[illegible]	[illegible]	[illegible]	[illegible]	[illegible]	[illegible]	[illegible]	[illegible]	[illegible]	[illegible]	[illegible]	[illegible]	[illegible]	[illegible]	[illegible]	[illegible]	[illegible]	[illegible]	[illegible]	[illegible]	[illegible]	[illegible]	[illegible]	[illegible]	[illegible]	[illegible]	[illegible]	[illegible]	[illegible]	[illegible]	[illegible]	[illegible]
00V11299	CC45-MRSA-IV, Berlin EMRSA	POS	[illegible]	[illegible]	[illegible]	[illegible]	[illegible]	[illegible]	[illegible]	[illegible]	[illegible]	[illegible]	[illegible]	[illegible]	[illegible]	[illegible]	[illegible]	[illegible]	[illegible]	[illegible]	[illegible]	[illegible]	[illegible]	[illegible]	[illegible]	[illegible]	[illegible]	[illegible]	[illegible]	[illegible]	[illegible]	[illegible]	[illegible]	[illegible]	[illegible]	[illegible]	[illegible]	[illegible]	[illegible]	[illegible]	[illegible]	[illegible]	[illegible]	[illegible]	[illegible]	[illegible]	[illegible]	[illegible]	[illegible]	[illegible]	[illegible]
00V14125	CC45-MRSA-IV, Berlin EMRSA	POS	[illegible]	[illegible]	[illegible]	[illegible]	[illegible]	[illegible]	[illegible]	[illegible]	[illegible]	[illegible]	[illegible]	[illegible]	[illegible]	[illegible]	[illegible]	[illegible]	[illegible]	[illegible]	[illegible]	[illegible]	[illegible]	[illegible]	[illegible]	[illegible]	[illegible]	[illegible]	[illegible]	[illegible]	[illegible]	[illegible]	[illegible]	[illegible]	[illegible]	[illegible]	[illegible]	[illegible]	[illegible]	[illegible]	[illegible]	[illegible]	[illegible]	[illegible]	[illegible]	[illegible]	[illegible]	[illegible]	[illegible]	[illegible]	[illegible]
00V16040	CC45-MRSA-IV, Berlin EMRSA	POS	[illegible]	[illegible]	[illegible]	[illegible]	[illegible]	[illegible]	[illegible]	[illegible]	[illegible]	[illegible]	[illegible]	[illegible]	[illegible]	[illegible]	[illegible]	[illegible]	[illegible]	[illegible]	[illegible]	[illegible]	[illegible]	[illegible]	[illegible]	[illegible]	[illegible]	[illegible]	[illegible]	[illegible]	[illegible]	[illegible]	[illegible]	[illegible]	[illegible]	[illegible]	[illegible]	[illegible]	[illegible]	[illegible]	[illegible]	[illegible]	[illegible]	[illegible]	[illegible]	[illegible]	[illegible]	[illegible]	[illegible]	[illegible]	[illegible]
00V25025	CC45-MRSA-IV, Berlin EMRSA	POS	[illegible]	[illegible]	[illegible]	[illegible]	[illegible]	[illegible]	[illegible]	[illegible]	[illegible]	[illegible]	[illegible]	[illegible]	[illegible]	[illegible]	[illegible]	[illegible]	[illegible]	[illegible]	[illegible]	[illegible]	[illegible]	[illegible]	[illegible]	[illegible]	[illegible]	[illegible]	[illegible]	[illegible]	[illegible]	[illegible]	[illegible]	[illegible]	[illegible]	[illegible]	[illegible]	[illegible]	[illegible]	[illegible]	[illegible]	[illegible]	[illegible]	[illegible]	[illegible]	[illegible]	[illegible]	[illegible]	[illegible]	[illegible]	[illegible]
00V29618	CC45-MRSA-IV, Berlin EMRSA	POS	[illegible]	[illegible]	[illegible]	[illegible]	[illegible]	[illegible]	[illegible]	[illegible]	[illegible]	[illegible]	[illegible]	[illegible]	[illegible]	[illegible]	[illegible]	[illegible]	[illegible]	[illegible]	[illegible]	[illegible]	[illegible]	[illegible]	[illegible]	[illegible]	[illegible]	[illegible]	[illegible]	[illegible]	[illegible]	[illegible]	[illegible]	[illegible]	[illegible]	[illegible]	[illegible]	[illegible]	[illegible]	[illegible]	[illegible]	[illegible]	[illegible]	[illegible]	[illegible]	[illegible]	[illegible]	[illegible]	[illegible]	[illegible]	[illegible]
00V30989	CC45-MRSA-IV, Berlin EMRSA	POS	[illegible]	[illegible]	[illegible]	[illegible]	[illegible]	[illegible]	[illegible]	[illegible]	[illegible]	[illegible]	[illegible]	[illegible]	[illegible]	[illegible]	[illegible]	[illegible]	[illegible]	[illegible]	[illegible]	[illegible]	[illegible]	[illegible]	[illegible]	[illegible]	[illegible]	[illegible]	[illegible]	[illegible]	[illegible]	[illegible]	[illegible]	[illegible]	[illegible]	[illegible]	[illegible]	[illegible]	[illegible]	[illegible]	[illegible]	[illegible]	[illegible]	[illegible]	[illegible]	[illegible]	[illegible]	[illegible]	[illegible]	[illegible]	[illegible]
00V31007	CC45-MRSA-IV, Berlin EMRSA	POS	[illegible]	[illegible]	[illegible]	[illegible]	[illegible]	[illegible]	[illegible]	[illegible]	[illegible]	[illegible]	[illegible]	[illegible]	[illegible]	[illegible]	[illegible]	[illegible]	[illegible]	[illegible]	[illegible]	[illegible]	[illegible]	[illegible]	[illegible]	[illegible]	[illegible]	[illegible]	[illegible]	[illegible]	[illegible]	[illegible]	[illegible]	[illegible]	[illegible]	[illegible]	[illegible]	[illegible]	[illegible]	[illegible]	[illegible]	[illegible]	[illegible]	[illegible]	[illegible]	[illegible]	[illegible]	[illegible]	[illegible]	[illegible]	[illegible]
00V31700	CC45-MRSA-IV, Berlin EMRSA	POS	[illegible]	[illegible]	[illegible]	[illegible]	[illegible]	[illegible]	[illegible]	[illegible]	[illegible]	[illegible]	[illegible]	[illegible]	[illegible]	[illegible]	[illegible]	[illegible]	[illegible]	[illegible]	[illegible]	[illegible]	[illegible]	[illegible]	[illegible]	[illegible]	[illegible]	[illegible]	[illegible]	[illegible]	[illegible]	[illegible]	[illegible]	[illegible]	[illegible]	[illegible]	[illegible]	[illegible]	[illegible]	[illegible]	[illegible]	[illegible]	[illegible]	[illegible]	[illegible]	[illegible]	[illegible]	[illegible]	[illegible]	[illegible]	[illegible]
00V32193	CC45-MRSA-IV, Berlin EMRSA	POS	[illegible]	[illegible]	[illegible]	[illegible]	[illegible]	[illegible]	[illegible]	[illegible]	[illegible]	[illegible]	[illegible]	[illegible]	[illegible]	[illegible]	[illegible]	[illegible]	[illegible]	[illegible]	[illegible]	[illegible]	[illegible]	[illegible]	[illegible]	[illegible]	[illegible]	[illegible]	[illegible]	[illegible]	[illegible]	[illegible]	[illegible]	[illegible]	[illegible]	[illegible]	[illegible]	[illegible]	[illegible]	[illegible]	[illegible]	[illegible]	[illegible]	[illegible]	[illegible]	[illegible]	[illegible]	[illegible]	[illegible]	[illegible]	[illegible]
00V43085	CC45-MRSA-IV, Berlin EMRSA	POS	[illegible]	[illegible]	[illegible]	[illegible]	[illegible]	[illegible]	[illegible]	[illegible]	[illegible]	[illegible]	[illegible]	[illegible]	[illegible]	[illegible]	[illegible]	[illegible]	[illegible]	[illegible]	[illegible]	[illegible]	[illegible]	[illegible]	[illegible]	[illegible]	[illegible]	[illegible]	[illegible]	[illegible]	[illegible]	[illegible]	[illegible]	[illegible]	[illegible]	[illegible]	[illegible]	[illegible]	[illegible]	[illegible]	[illegible]	[illegible]	[illegible]	[illegible]	[illegible]	[illegible]	[illegible]	[illegible]	[illegible]	[illegible]	[illegible]
00V44382	CC45-MRSA-IV, Berlin EMRSA	POS	[illegible]	[illegible]	[illegible]	[illegible]	[illegible]	[illegible]	[illegible]	[illegible]	[illegible]	[illegible]	[illegible]	[illegible]	[illegible]	[illegible]	[illegible]	[illegible]	[illegible]	[illegible]	[illegible]	[illegible]	[illegible]	[illegible]	[illegible]	[illegible]	[illegible]	[illegible]	[illegible]	[illegible]	[illegible]	[illegible]	[illegible]	[illegible]	[illegible]	[illegible]	[illegible]	[illegible]	[illegible]	[illegible]	[illegible]	[illegible]	[illegible]	[illegible]	[illegible]	[illegible]	[illegible]	[illegible]	[illegible]	[illegible]	[illegible]
00V46648	CC45-MRSA-IV, Berlin EMRSA	POS	[illegible]	[illegible]	[illegible]	[illegible]	[illegible]	[illegible]	[illegible]	[illegible]	[illegible]	[illegible]	[illegible]	[illegible]	[illegible]	[illegible]	[illegible]	[illegible]	[illegible]	[illegible]	[illegible]	[illegible]	[illegible]	[illegible]	[illegible]	[illegible]	[illegible]	[illegible]	[illegible]	[illegible]	[illegible]	[illegible]	[illegible]	[illegible]	[illegible]	[illegible]	[illegible]	[illegible]	[illegible]	[illegible]	[illegible]	[illegible]	[illegible]	[illegible]	[illegible]	[illegible]	[illegible]	[illegible]	[illegible]	[illegible]	[illegible]
00V46843	CC45-MRSA-IV, Berlin EMRSA	POS	[illegible]	[illegible]	[illegible]	[illegible]	[illegible]	[illegible]	[illegible]	[illegible]	[illegible]	[illegible]	[illegible]	[illegible]	[illegible]	[illegible]	[illegible]	[illegible]	[illegible]	[illegible]	[illegible]	[illegible]	[illegible]	[illegible]	[illegible]	[illegible]	[illegible]	[illegible]	[illegible]	[illegible]	[illegible]	[illegible]	[illegible]	[illegible]	[illegible]	[illegible]	[illegible]	[illegible]	[illegible]	[illegible]	[illegible]	[illegible]	[illegible]	[illegible]	[illegible]	[illegible]	[illegible]	[illegible]	[illegible]	[illegible]	[illegible]
00V47014	CC45-MRSA-IV, Berlin EMRSA	POS	[illegible]	[illegible]	[illegible]	[illegible]	[illegible]	[illegible]	[illegible]	[illegible]	[illegible]	[illegible]	[illegible]	[illegible]	[illegible]	[illegible]	[illegible]	[illegible]	[illegible]	[illegible]	[illegible]	[illegible]	[illegible]	[illegible]	[illegible]	[illegible]	[illegible]	[illegible]	[illegible]	[illegible]	[illegible]	[illegible]	[illegible]	[illegible]	[illegible]	[illegible]	[illegible]	[illegible]	[illegible]	[illegible]	[illegible]	[illegible]	[illegible]	[illegible]	[illegible]	[illegible]	[illegible]	[illegible]	[illegible]	[illegible]	[illegible]
00V47053	CC45-MRSA-IV, Berlin EMRSA	POS	[illegible]	[illegible]	[illegible]	[illegible]	[illegible]	[illegible]	[illegible]	[illegible]	[illegible]	[illegible]	[illegible]	[illegible]	[illegible]	[illegible]	[illegible]	[illegible]	[illegible]	[illegible]	[illegible]	[illegible]	[illegible]	[illegible]	[illegible]	[illegible]	[illegible]	[illegible]	[illegible]	[illegible]	[illegible]	[illegible]	[illegible]	[illegible]	[illegible]	[illegible]	[illegible]	[illegible]	[illegible]	[illegible]	[illegible]	[illegible]	[illegible]	[illegible]	[illegible]	[illegible]	[illegible]	[illegible]	[illegible]	[illegible]	[illegible]
00V47227	CC45-MRSA-IV, Berlin EMRSA	POS	[illegible]	[illegible]	[illegible]	[illegible]	[illegible]	[illegible]	[illegible]	[illegible]	[illegible]	[illegible]	[illegible]	[illegible]	[illegible]	[illegible]	[illegible]	[illegible]	[illegible]	[illegible]	[illegible]	[illegible]	[illegible]	[illegible]	[illegible]	[illegible]	[illegible]	[illegible]	[illegible]	[illegible]	[illegible]	[illegible]	[illegible]	[illegible]	[illegible]	[illegible]	[illegible]	[illegible]	[illegible]	[illegible]	[illegible]	[illegible]	[illegible]	[illegible]	[illegible]	[illegible]	[illegible]	[illegible]	[illegible]	[illegible]	[illegible]
00V47429	CC45-MRSA-IV, Berlin EMRSA	POS	[illegible]	[illegible]	[illegible]	[illegible]	[illegible]	[illegible]	[illegible]	[illegible]	[illegible]	[illegible]	[illegible]	[illegible]	[illegible]	[illegible]	[illegible]	[illegible]	[illegible]	[illegible]	[illegible]	[illegible]	[illegible]	[illegible]	[illegible]	[illegible]	[illegible]	[illegible]	[illegible]	[illegible]	[illegible]	[illegible]	[illegible]	[illegible]	[illegible]	[illegible]	[illegible]	[illegible]	[illegible]	[illegible]	[illegible]	[illegible]	[illegible]	[illegible]	[illegible]	[illegible]	[illegible]	[illegible]	[illegible]	[illegible]	[illegible]
00V47531	CC45-MRSA-IV, Berlin EMRSA	POS	[illegible]	[illegible]	[illegible]	[illegible]	[illegible]	[illegible]	[illegible]	[illegible]	[illegible]	[illegible]	[illegible]	[illegible]	[illegible]	[illegible]	[illegible]	[illegible]	[illegible]	[illegible]	[illegible]	[illegible]	[illegible]	[illegible]	[illegible]	[illegible]	[illegible]	[illegible]	[illegible]	[illegible]	[illegible]	[illegible]	[illegible]	[illegible]	[illegible]	[illegible]	[illegible]	[illegible]	[illegible]	[illegible]	[illegible]	[illegible]	[illegible]	[illegible]	[illegible]	[illegible]	[illegible]	[illegible]	[illegible]	[illegible]	[illegible]
**2001**																																																			
01V27106	CC1-MRSA-IV, WA MRSA-1/57	POS	[illegible]	[illegible]	[illegible]	[illegible]	[illegible]	[illegible]	[illegible]	[illegible]	[illegible]	[illegible]	[illegible]	[illegible]	[illegible]	[illegible]	[illegible]	[illegible]	[illegible]	[illegible]	[illegible]	[illegible]	[illegible]	[illegible]	[illegible]	[illegible]	[illegible]	[illegible]	[illegible]	[illegible]	[illegible]	[illegible]	[illegible]	[illegible]	[illegible]	[illegible]	[illegible]	[illegible]	[illegible]	[illegible]	[illegible]	[illegible]	[illegible]	[illegible]	[illegible]	[illegible]	[illegible]	[illegible]	[illegible]	[illegible]	[illegible]
01V41025	CC5/ST228-MRSA-I, South German EMRSA	POS	[illegible]	[illegible]	[illegible]	[illegible]	[illegible]	[illegible]	[illegible]	[illegible]	[illegible]	[illegible]	[illegible]	[illegible]	[illegible]	[illegible]	[illegible]	[illegible]	[illegible]	[illegible]	[illegible]	[illegible]	[illegible]	[illegible]	[illegible]	[illegible]	[illegible]	[illegible]	[illegible]	[illegible]	[illegible]	[illegible]	[illegible]	[illegible]	[illegible]	[illegible]	[illegible]	[illegible]	[illegible]	[illegible]	[illegible]	[illegible]	[illegible]	[illegible]	[illegible]	[illegible]	[illegible]	[illegible]	[illegible]	[illegible]	[illegible]
01V12125	CC5-MRSA-II, Rhine-Hesse EMRSA/UK-EMRSA-3	POS	[illegible]	[illegible]	[illegible]	[illegible]	[illegible]	[illegible]	[illegible]	[illegible]	[illegible]	[illegible]	[illegible]	[illegible]	[illegible]	[illegible]	[illegible]	[illegible]	[illegible]	[illegible]	[illegible]	[illegible]	[illegible]	[illegible]	[illegible]	[illegible]	[illegible]	[illegible]	[illegible]	[illegible]	[illegible]	[illegible]	[illegible]	[illegible]	[illegible]	[illegible]	[illegible]	[illegible]	[illegible]	[illegible]	[illegible]	[illegible]	[illegible]	[illegible]	[illegible]	[illegible]	[illegible]	[illegible]	[illegible]	[illegible]	[illegible]
01V29747	CC5-MRSA-II, Rhine-Hesse EMRSA/UK-EMRSA-3	POS	[illegible]	[illegible]	[illegible]	[illegible]	[illegible]	[illegible]	[illegible]	[illegible]	[illegible]	[illegible]	[illegible]	[illegible]	[illegible]	[illegible]	[illegible]	[illegible]	[illegible]	[illegible]	[illegible]	[illegible]	[illegible]	[illegible]	[illegible]	[illegible]	[illegible]	[illegible]	[illegible]	[illegible]	[illegible]	[illegible]	[illegible]	[illegible]	[illegible]	[illegible]	[illegible]	[illegible]	[illegible]	[illegible]	[illegible]	[illegible]	[illegible]	[illegible]	[illegible]	[illegible]	[illegible]	[illegible]	[illegible]	[illegible]	[illegible]
01V32464	CC5-MRSA-II, Rhine-Hesse EMRSA/UK-EMRSA-3	POS	[illegible]	[illegible]	[illegible]	[illegible]	[illegible]	[illegible]	[illegible]	[illegible]	[illegible]	[illegible]	[illegible]	[illegible]	[illegible]	[illegible]	[illegible]	[illegible]	[illegible]	[illegible]	[illegible]	[illegible]	[illegible]	[illegible]	[illegible]	[illegible]	[illegible]	[illegible]	[illegible]	[illegible]	[illegible]	[illegible]	[illegible]	[illegible]	[illegible]	[illegible]	[illegible]	[illegible]	[illegible]	[illegible]	[illegible]	[illegible]	[illegible]	[illegible]	[illegible]	[illegible]	[illegible]	[illegible]	[illegible]	[illegible]	[illegible]
01V39703	CC5-MRSA-II, Rhine-Hesse EMRSA/UK-EMRSA-3	POS	[illegible]	[illegible]	[illegible]	[illegible]	[illegible]	[illegible]	[illegible]	[illegible]	[illegible]	[illegible]	[illegible]	[illegible]	[illegible]	[illegible]	[illegible]	[illegible]	[illegible]	[illegible]	[illegible]	[illegible]	[illegible]	[illegible]	[illegible]	[illegible]	[illegible]	[illegible]	[illegible]	[illegible]	[illegible]	[illegible]	[illegible]	[illegible]	[illegible]	[illegible]	[illegible]	[illegible]	[illegible]	[illegible]	[illegible]	[illegible]	[illegible]	[illegible]	[illegible]	[illegible]	[illegible]	[illegible]	[illegible]	[illegible]	[illegible]
01V40228	CC5-MRSA-II, Rhine-Hesse EMRSA/UK-EMRSA-3																																																		
01B05861	CC8/ST239-MRSA-III, Vienna/Hungarian/Brazilian Clone	POS	[illegible]	[illegible]	[illegible]	[illegible]	[illegible]	[illegible]	[illegible]	[illegible]	[illegible]	[illegible]	[illegible]	[illegible]	[illegible]	[illegible]	[illegible]	[illegible]	[illegible]	[illegible]	[illegible]	[illegible]	[illegible]	[illegible]	[illegible]	[illegible]	[illegible]	[illegible]	[illegible]	[illegible]	[illegible]	[illegible]	[illegible]	[illegible]	[illegible]	[illegible]	[illegible]	[illegible]	[illegible]	[illegible]	[illegible]	[illegible]	[illegible]	[illegible]	[illegible]	[illegible]	[illegible]	[illegible]	[illegible]	[illegible]	[illegible]
01V34572	CC8/ST239-MRSA-III, Vienna/Hungarian/Brazilian Clone	POS	[illegible]	[illegible]	[illegible]	[illegible]	[illegible]	[illegible]	[illegible]	[illegible]	[illegible]	[illegible]	[illegible]	[illegible]	[illegible]	[illegible]	[illegible]	[illegible]	[illegible]	[illegible]	[illegible]	[illegible]	[illegible]	[illegible]	[illegible]	[illegible]	[illegible]	[illegible]	[illegible]	[illegible]	[illegible]	[illegible]	[illegible]	[illegible]	[illegible]	[illegible]	[illegible]	[illegible]	[illegible]	[illegible]	[illegible]	[illegible]	[illegible]	[illegible]	[illegible]	[illegible]	[illegible]	[illegible]	[illegible]	[illegible]	[illegible]
01V36469	CC8/ST239-MRSA-III, Vienna/Hungarian/Brazilian Clone	POS	[illegible]	[illegible]	[illegible]	[illegible]	[illegible]	[illegible]	[illegible]	[illegible]	[illegible]	[illegible]	[illegible]	[illegible]	[illegible]	[illegible]	[illegible]	[illegible]	[illegible]	[illegible]	[illegible]	[illegible]	[illegible]	[illegible]	[illegible]	[illegible]	[illegible]	[illegible]	[illegible]	[illegible]	[illegible]	[illegible]	[illegible]	[illegible]	[illegible]	[illegible]	[illegible]	[illegible]	[illegible]	[illegible]	[illegible]	[illegible]	[illegible]	[illegible]	[illegible]	[illegible]	[illegible]	[illegible]	[illegible]	[illegible]	[illegible]
01V39123	CC8/ST239-MRSA-III, Vienna/Hungarian/Brazilian Clone	POS	[illegible]	[illegible]	[illegible]	[illegible]	[illegible]	[illegible]	[illegible]	[illegible]	[illegible]	[illegible]	[illegible]	[illegible]	[illegible]	[illegible]	[illegible]	[illegible]	[illegible]	[illegible]	[illegible]	[illegible]	[illegible]	[illegible]	[illegible]	[illegible]	[illegible]	[illegible]	[illegible]	[illegible]	[illegible]	[illegible]	[illegible]	[illegible]	[illegible]	[illegible]	[illegible]	[illegible]	[illegible]	[illegible]	[illegible]	[illegible]	[illegible]	[illegible]	[illegible]	[illegible]	[illegible]	[illegible]	[illegible]	[illegible]	[illegible]
01V39143	CC8/ST239-MRSA-III, Vienna/Hungarian/Brazilian Clone	POS	[illegible]	[illegible]	[illegible]	[illegible]	[illegible]	[illegible]	[illegible]	[illegible]	[illegible]	[illegible]	[illegible]	[illegible]	[illegible]	[illegible]	[illegible]	[illegible]	[illegible]	[illegible]	[illegible]	[illegible]	[illegible]	[illegible]	[illegible]	[illegible]	[illegible]	[illegible]	[illegible]	[illegible]	[illegible]	[illegible]	[illegible]	[illegible]	[illegible]	[illegible]	[illegible]	[illegible]	[illegible]	[illegible]	[illegible]	[illegible]	[illegible]	[illegible]	[illegible]	[illegible]	[illegible]	[illegible]	[illegible]	[illegible]	[illegible]
01V10962	CC8/ST254-MRSA-(atypical SCCmec), Hannover EMRSA	POS	[illegible]	[illegible]	[illegible]	[illegible]	[illegible]	[illegible]	[illegible]	[illegible]	[illegible]	[illegible]	[illegible]	[illegible]	[illegible]	[illegible]	[illegible]	[illegible]	[illegible]	[illegible]	[illegible]	[illegible]	[illegible]	[illegible]	[illegible]	[illegible]	[illegible]	[illegible]	[illegible]	[illegible]	[illegible]	[illegible]	[illegible]	[illegible]	[illegible]	[illegible]	[illegible]	[illegible]	[illegible]	[illegible]	[illegible]	[illegible]	[illegible]	[illegible]	[illegible]	[illegible]	[illegible]	[illegible]	[illegible]	[illegible]	[illegible]
01V10963	CC8/ST254-MRSA-IV/V, UK-EMRSA-10/Hannover EMRSA	POS	[illegible]	[illegible]	[illegible]	[illegible]	[illegible]	[illegible]	[illegible]	[illegible]	[illegible]	[illegible]	[illegible]	[illegible]	[illegible]	[illegible]	[illegible]	[illegible]	[illegible]	[illegible]	[illegible]	[illegible]	[illegible]	[illegible]	[illegible]	[illegible]	[illegible]	[illegible]	[illegible]	[illegible]	[illegible]	[illegible]	[illegible]	[illegible]	[illegible]	[illegible]	[illegible]	[illegible]	[illegible]	[illegible]	[illegible]	[illegible]	[illegible]	[illegible]	[illegible]	[illegible]	[illegible]	[illegible]	[illegible]	[illegible]	[illegible]
01V36287	CC8/ST254-MRSA-IV/V, UK-EMRSA-10/Hannover EMRSA	POS	[illegible]	[illegible]	[illegible]	[illegible]	[illegible]	[illegible]	[illegible]	[illegible]	[illegible]	[illegible]	[illegible]	[illegible]	[illegible]	[illegible]	[illegible]	[illegible]	[illegible]	[illegible]	[illegible]	[illegible]	[illegible]	[illegible]	[illegible]	[illegible]	[illegible]	[illegible]	[illegible]	[illegible]	[illegible]	[illegible]	[illegible]	[illegible]	[illegible]	[illegible]	[illegible]	[illegible]	[illegible]	[illegible]	[illegible]	[illegible]	[illegible]	[illegible]	[illegible]	[illegible]	[illegible]	[illegible]	[illegible]	[illegible]	[illegible]
01V25329	CC22-MRSA-IV [PVL+]	POS	[illegible]	[illegible]	[illegible]	[illegible]	[illegible]	[illegible]	[illegible]	[illegible]	[illegible]	[illegible]	[illegible]	[illegible]	[illegible]	[illegible]	[illegible]	[illegible]	[illegible]	[illegible]	[illegible]	[illegible]	[illegible]	[illegible]	[illegible]	[illegible]	[illegible]	[illegible]	[illegible]	[illegible]	[illegible]	[illegible]	[illegible]	[illegible]	[illegible]	[illegible]	[illegible]	[illegible]	[illegible]	[illegible]	[illegible]	[illegible]	[illegible]	[illegible]	[illegible]	[illegible]	[illegible]	[illegible]	[illegible]	[illegible]	[illegible]
01V07770	CC22-MRSA-IV, Barnim/UK-EMRSA-15																																																		
01V20122	CC22-MRSA-IV, Barnim/UK-EMRSA-15	POS	[illegible]	[illegible]	[illegible]	[illegible]	[illegible]	[illegible]	[illegible]	[illegible]	[illegible]	[illegible]	[illegible]	[illegible]	[illegible]	[illegible]	[illegible]	[illegible]	[illegible]	[illegible]	[illegible]	[illegible]	[illegible]	[illegible]	[illegible]	[illegible]	[illegible]	[illegible]	[illegible]	[illegible]	[illegible]	[illegible]	[illegible]	[illegible]	[illegible]	[illegible]	[illegible]	[illegible]	[illegible]	[illegible]	[illegible]	[illegible]	[illegible]	[illegible]	[illegible]	[illegible]	[illegible]	[illegible]	[illegible]	[illegible]	[illegible]
01V43277	CC22-MRSA-IV, Barnim/UK-EMRSA-15	POS	[illegible]	[illegible]	[illegible]	[illegible]	[illegible]	[illegible]	[illegible]	[illegible]	[illegible]	[illegible]	[illegible]	[illegible]	[illegible]	[illegible]	[illegible]	[illegible]	[illegible]	[illegible]	[illegible]	[illegible]	[illegible]	[illegible]	[illegible]	[illegible]	[illegible]	[illegible]	[illegible]	[illegible]	[illegible]	[illegible]	[illegible]	[illegible]	[illegible]	[illegible]	[illegible]	[illegible]	[illegible]	[illegible]	[illegible]	[illegible]	[illegible]	[illegible]	[illegible]	[illegible]	[illegible]	[illegible]	[illegible]	[illegible]	[illegible]
01F02744	CC45-MRSA-IV, Berlin EMRSA	POS	[illegible]	[illegible]	[illegible]	[illegible]	[illegible]	[illegible]	[illegible]	[illegible]	[illegible]	[illegible]	[illegible]	[illegible]	[illegible]	[illegible]	[illegible]	[illegible]	[illegible]	[illegible]	[illegible]	[illegible]	[illegible]	[illegible]	[illegible]	[illegible]	[illegible]	[illegible]	[illegible]	[illegible]	[illegible]	[illegible]	[illegible]	[illegible]	[illegible]	[illegible]	[illegible]	[illegible]	[illegible]	[illegible]	[illegible]	[illegible]	[illegible]	[illegible]	[illegible]	[illegible]	[illegible]	[illegible]	[illegible]	[illegible]	[illegible]
01V01127	CC45-MRSA-IV, Berlin EMRSA	POS	[illegible]	[illegible]	[illegible]	[illegible]	[illegible]	[illegible]	[illegible]	[illegible]	[illegible]	[illegible]	[illegible]	[illegible]	[illegible]	[illegible]	[illegible]	[illegible]	[illegible]	[illegible]	[illegible]	[illegible]	[illegible]	[illegible]	[illegible]	[illegible]	[illegible]	[illegible]	[illegible]	[illegible]	[illegible]	[illegible]	[illegible]	[illegible]	[illegible]	[illegible]	[illegible]	[illegible]	[illegible]	[illegible]	[illegible]	[illegible]	[illegible]	[illegible]	[illegible]	[illegible]	[illegible]	[illegible]	[illegible]	[illegible]	[illegible]
01V01445	CC45-MRSA-IV, Berlin EMRSA	POS	[illegible]	[illegible]	[illegible]	[illegible]	[illegible]	[illegible]	[illegible]	[illegible]	[illegible]	[illegible]	[illegible]	[illegible]	[illegible]	[illegible]	[illegible]	[illegible]	[illegible]	[illegible]	[illegible]	[illegible]	[illegible]	[illegible]	[illegible]	[illegible]	[illegible]	[illegible]	[illegible]	[illegible]	[illegible]	[illegible]	[illegible]	[illegible]	[illegible]	[illegible]	[illegible]	[illegible]	[illegible]	[illegible]	[illegible]	[illegible]	[illegible]	[illegible]	[illegible]	[illegible]	[illegible]	[illegible]	[illegible]	[illegible]	[illegible]
01V02575	CC45-MRSA-IV, Berlin EMRSA	POS	[illegible]	[illegible]	[illegible]	[illegible]	[illegible]	[illegible]	[illegible]	[illegible]	[illegible]	[illegible]	[illegible]	[illegible]	[illegible]	[illegible]	[illegible]	[illegible]	[illegible]	[illegible]	[illegible]	[illegible]	[illegible]	[illegible]	[illegible]	[illegible]	[illegible]	[illegible]	[illegible]	[illegible]	[illegible]	[illegible]	[illegible]	[illegible]	[illegible]	[illegible]	[illegible]	[illegible]	[illegible]	[illegible]	[illegible]	[illegible]	[illegible]	[illegible]	[illegible]	[illegible]	[illegible]	[illegible]	[illegible]	[illegible]	[illegible]
01V02636	CC45-MRSA-IV, Berlin EMRSA	POS	[illegible]	[illegible]	[illegible]	[illegible]	[illegible]	[illegible]	[illegible]	[illegible]	[illegible]	[illegible]	[illegible]	[illegible]	[illegible]	[illegible]	[illegible]	[illegible]	[illegible]	[illegible]	[illegible]	[illegible]	[illegible]	[illegible]	[illegible]	[illegible]	[illegible]	[illegible]	[illegible]	[illegible]	[illegible]	[illegible]	[illegible]	[illegible]	[illegible]	[illegible]	[illegible]	[illegible]	[illegible]	[illegible]	[illegible]	[illegible]	[illegible]	[illegible]	[illegible]	[illegible]	[illegible]	[illegible]	[illegible]	[illegible]	[illegible]
01V03483	CC45-MRSA-IV, Berlin EMRSA	POS	[illegible]	[illegible]	[illegible]	[illegible]	[illegible]	[illegible]	[illegible]	[illegible]	[illegible]	[illegible]	[illegible]	[illegible]	[illegible]	[illegible]	[illegible]	[illegible]	[illegible]	[illegible]	[illegible]	[illegible]	[illegible]	[illegible]	[illegible]	[illegible]	[illegible]	[illegible]	[illegible]	[illegible]	[illegible]	[illegible]	[illegible]	[illegible]	[illegible]	[illegible]	[illegible]	[illegible]	[illegible]	[illegible]	[illegible]	[illegible]	[illegible]	[illegible]	[illegible]	[illegible]	[illegible]	[illegible]	[illegible]	[illegible]	[illegible]
01V04728	CC45-MRSA-IV, Berlin EMRSA	POS	[illegible]	[illegible]	[illegible]	[illegible]	[illegible]	[illegible]	[illegible]	[illegible]	[illegible]	[illegible]	[illegible]	[illegible]	[illegible]	[illegible]	[illegible]	[illegible]	[illegible]	[illegible]	[illegible]	[illegible]	[illegible]	[illegible]	[illegible]	[illegible]	[illegible]	[illegible]	[illegible]	[illegible]	[illegible]	[illegible]	[illegible]	[illegible]	[illegible]	[illegible]	[illegible]	[illegible]	[illegible]	[illegible]	[illegible]	[illegible]	[illegible]	[illegible]	[illegible]	[illegible]	[illegible]	[illegible]	[illegible]	[illegible]	[illegible]
01V05972	CC45-MRSA-IV, Berlin EMRSA	POS	[illegible]	[illegible]	[illegible]	[illegible]	[illegible]	[illegible]	[illegible]	[illegible]	[illegible]	[illegible]	[illegible]	[illegible]	[illegible]	[illegible]	[illegible]	[illegible]	[illegible]	[illegible]	[illegible]	[illegible]	[illegible]	[illegible]	[illegible]	[illegible]	[illegible]	[illegible]	[illegible]	[illegible]	[illegible]	[illegible]	[illegible]	[illegible]	[illegible]	[illegible]	[illegible]	[illegible]	[illegible]	[illegible]	[illegible]	[illegible]	[illegible]	[illegible]	[illegible]	[illegible]	[illegible]	[illegible]	[illegible]	[illegible]	[illegible]
01V07139	CC45-MRSA-IV, Berlin EMRSA	POS	[illegible]	[illegible]	[illegible]	[illegible]	[illegible]	[illegible]	[illegible]	[illegible]	[illegible]	[illegible]	[illegible]	[illegible]	[illegible]	[illegible]	[illegible]	[illegible]	[illegible]	[illegible]	[illegible]	[illegible]	[illegible]	[illegible]	[illegible]	[illegible]	[illegible]	[illegible]	[illegible]	[illegible]	[illegible]	[illegible]	[illegible]	[illegible]	[illegible]	[illegible]	[illegible]	[illegible]	[illegible]	[illegible]	[illegible]	[illegible]	[illegible]	[illegible]	[illegible]	[illegible]	[illegible]	[illegible]	[illegible]	[illegible]	[illegible]
01V08133	CC45-MRSA-IV, Berlin EMRSA	POS	[illegible]	[illegible]	[illegible]	[illegible]	[illegible]	[illegible]	[illegible]	[illegible]	[illegible]	[illegible]	[illegible]	[illegible]	[illegible]	[illegible]	[illegible]	[illegible]	[illegible]	[illegible]	[illegible]	[illegible]	[illegible]	[illegible]	[illegible]	[illegible]	[illegible]	[illegible]	[illegible]	[illegible]	[illegible]	[illegible]	[illegible]	[illegible]	[illegible]	[illegible]	[illegible]	[illegible]	[illegible]	[illegible]	[illegible]	[illegible]	[illegible]	[illegible]	[illegible]	[illegible]	[illegible]	[illegible]	[illegible]	[illegible]	[illegible]
01V09872	CC45-MRSA-IV, Berlin EMRSA	POS	[illegible]	[illegible]	[illegible]	[illegible]	[illegible]	[illegible]	[illegible]	[illegible]	[illegible]	[illegible]	[illegible]	[illegible]	[illegible]	[illegible]	[illegible]	[illegible]	[illegible]	[illegible]	[illegible]	[illegible]	[illegible]	[illegible]	[illegible]	[illegible]	[illegible]	[illegible]	[illegible]	[illegible]	[illegible]	[illegible]	[illegible]	[illegible]	[illegible]	[illegible]	[illegible]	[illegible]	[illegible]	[illegible]	[illegible]	[illegible]	[illegible]	[illegible]	[illegible]	[illegible]	[illegible]	[illegible]	[illegible]	[illegible]	[illegible]
01V12465	CC45-MRSA-IV, Berlin EMRSA	POS	[illegible]	[illegible]	[illegible]	[illegible]	[illegible]	[illegible]	[illegible]	[illegible]	[illegible]	[illegible]	[illegible]	[illegible]	[illegible]	[illegible]	[illegible]	[illegible]	[illegible]	[illegible]	[illegible]	[illegible]	[illegible]	[illegible]	[illegible]	[illegible]	[illegible]	[illegible]	[illegible]	[illegible]	[illegible]	[illegible]	[illegible]	[illegible]	[illegible]	[illegible]	[illegible]	[illegible]	[illegible]	[illegible]	[illegible]	[illegible]	[illegible]	[illegible]	[illegible]	[illegible]	[illegible]	[illegible]	[illegible]	[illegible]	[illegible]
01V16151	CC45-MRSA-IV, Berlin EMRSA	POS	[illegible]	[illegible]	[illegible]	[illegible]	[illegible]	[illegible]	[illegible]	[illegible]	[illegible]	[illegible]	[illegible]	[illegible]	[illegible]	[illegible]	[illegible]	[illegible]	[illegible]	[illegible]	[illegible]	[illegible]	[illegible]	[illegible]	[illegible]	[illegible]	[illegible]	[illegible]	[illegible]	[illegible]	[illegible]	[illegible]	[illegible]	[illegible]	[illegible]	[illegible]	[illegible]	[illegible]	[illegible]	[illegible]	[illegible]	[illegible]	[illegible]	[illegible]	[illegible]	[illegible]	[illegible]	[illegible]	[illegible]	[illegible]	[illegible]
01V16873	CC45-MRSA-IV, Berlin EMRSA	POS	[illegible]	[illegible]	[illegible]	[illegible]	[illegible]	[illegible]	[illegible]	[illegible]	[illegible]	[illegible]	[illegible]	[illegible]	[illegible]	[illegible]	[illegible]	[illegible]	[illegible]	[illegible]	[illegible]	[illegible]	[illegible]	[illegible]	[illegible]	[illegible]	[illegible]	[illegible]	[illegible]	[illegible]	[illegible]	[illegible]	[illegible]	[illegible]	[illegible]	[illegible]	[illegible]	[illegible]	[illegible]	[illegible]	[illegible]	[illegible]	[illegible]	[illegible]	[illegible]	[illegible]	[illegible]	[illegible]	[illegible]	[illegible]	[illegible]
01V17796	CC45-MRSA-IV, Berlin EMRSA	POS	[illegible]	[illegible]	[illegible]	[illegible]	[illegible]	[illegible]	[illegible]	[illegible]	[illegible]	[illegible]	[illegible]	[illegible]	[illegible]	[illegible]	[illegible]	[illegible]	[illegible]	[illegible]	[illegible]	[illegible]	[illegible]	[illegible]	[illegible]	[illegible]	[illegible]	[illegible]	[illegible]	[illegible]	[illegible]	[illegible]	[illegible]	[illegible]	[illegible]	[illegible]	[illegible]	[illegible]	[illegible]	[illegible]	[illegible]	[illegible]	[illegible]	[illegible]	[illegible]	[illegible]	[illegible]	[illegible]	[illegible]	[illegible]	[illegible]
01V18681	CC45-MRSA-IV, Berlin EMRSA	POS	[illegible]	[illegible]	[illegible]	[illegible]	[illegible]	[illegible]	[illegible]	[illegible]	[illegible]	[illegible]	[illegible]	[illegible]	[illegible]	[illegible]	[illegible]	[illegible]	[illegible]	[illegible]	[illegible]	[illegible]	[illegible]	[illegible]	[illegible]	[illegible]	[illegible]	[illegible]	[illegible]	[illegible]	[illegible]	[illegible]	[illegible]	[illegible]	[illegible]	[illegible]	[illegible]	[illegible]	[illegible]	[illegible]	[illegible]	[illegible]	[illegible]	[illegible]	[illegible]	[illegible]	[illegible]	[illegible]	[illegible]	[illegible]	[illegible]
01V20066	CC45-MRSA-IV, Berlin EMRSA	POS	[illegible]	[illegible]	[illegible]	[illegible]	[illegible]	[illegible]	[illegible]	[illegible]	[illegible]	[illegible]	[illegible]	[illegible]	[illegible]	[illegible]	[illegible]	[illegible]	[illegible]	[illegible]	[illegible]	[illegible]	[illegible]	[illegible]	[illegible]	[illegible]	[illegible]	[illegible]	[illegible]	[illegible]	[illegible]	[illegible]	[illegible]	[illegible]	[illegible]	[illegible]	[illegible]	[illegible]	[illegible]	[illegible]	[illegible]	[illegible]	[illegible]	[illegible]	[illegible]	[illegible]	[illegible]	[illegible]	[illegible]	[illegible]	[illegible]
01V23050	CC45-MRSA-IV, Berlin EMRSA	POS	[illegible]	[illegible]	[illegible]	[illegible]	[illegible]	[illegible]	[illegible]	[illegible]	[illegible]	[illegible]	[illegible]	[illegible]	[illegible]	[illegible]	[illegible]	[illegible]	[illegible]	[illegible]	[illegible]	[illegible]	[illegible]	[illegible]	[illegible]	[illegible]	[illegible]	[illegible]	[illegible]	[illegible]	[illegible]	[illegible]	[illegible]	[illegible]	[illegible]	[illegible]	[illegible]	[illegible]	[illegible]	[illegible]	[illegible]	[illegible]	[illegible]	[illegible]	[illegible]	[illegible]	[illegible]	[illegible]	[illegible]	[illegible]	[illegible]
01V25584	CC45-MRSA-IV, Berlin EMRSA	POS	[illegible]	[illegible]	[illegible]	[illegible]	[illegible]	[illegible]	[illegible]	[illegible]	[illegible]	[illegible]	[illegible]	[illegible]	[illegible]	[illegible]	[illegible]	[illegible]	[illegible]	[illegible]	[illegible]	[illegible]	[illegible]	[illegible]	[illegible]	[illegible]	[illegible]	[illegible]	[illegible]	[illegible]	[illegible]	[illegible]	[illegible]	[illegible]	[illegible]	[illegible]	[illegible]	[illegible]	[illegible]	[illegible]	[illegible]	[illegible]	[illegible]	[illegible]	[illegible]	[illegible]	[illegible]	[illegible]	[illegible]	[illegible]	[illegible]
01V31705	CC45-MRSA-IV, Berlin EMRSA	POS	[illegible]	[illegible]	[illegible]	[illegible]	[illegible]	[illegible]	[illegible]	[illegible]	[illegible]	[illegible]	[illegible]	[illegible]	[illegible]	[illegible]	[illegible]	[illegible]	[illegible]	[illegible]	[illegible]	[illegible]	[illegible]	[illegible]	[illegible]	[illegible]	[illegible]	[illegible]	[illegible]	[illegible]	[illegible]	[illegible]	[illegible]	[illegible]	[illegible]	[illegible]	[illegible]	[illegible]	[illegible]	[illegible]	[illegible]	[illegible]	[illegible]	[illegible]	[illegible]	[illegible]	[illegible]	[illegible]	[illegible]	[illegible]	[illegible]
01V34968	CC45-MRSA-IV, Berlin EMRSA	POS	[illegible]	[illegible]	[illegible]	[illegible]	[illegible]	[illegible]	[illegible]	[illegible]	[illegible]	[illegible]	[illegible]	[illegible]	[illegible]	[illegible]	[illegible]	[illegible]	[illegible]	[illegible]	[illegible]	[illegible]	[illegible]	[illegible]	[illegible]	[illegible]	[illegible]	[illegible]	[illegible]	[illegible]	[illegible]	[illegible]	[illegible]	[illegible]	[illegible]	[illegible]	[illegible]	[illegible]	[illegible]	[illegible]	[illegible]	[illegible]	[illegible]	[illegible]	[illegible]	[illegible]	[illegible]	[illegible]	[illegible]	[illegible]	[illegible]
01V36186	CC45-MRSA-IV, Berlin EMRSA	POS	[illegible]	[illegible]	[illegible]	[illegible]	[illegible]	[illegible]	[illegible]	[illegible]	[illegible]	[illegible]	[illegible]	[illegible]	[illegible]	[illegible]	[illegible]	[illegible]	[illegible]	[illegible]	[illegible]	[illegible]	[illegible]	[illegible]	[illegible]	[illegible]	[illegible]	[illegible]	[illegible]	[illegible]	[illegible]	[illegible]	[illegible]	[illegible]	[illegible]	[illegible]	[illegible]	[illegible]	[illegible]	[illegible]	[illegible]	[illegible]	[illegible]	[illegible]	[illegible]	[illegible]	[illegible]	[illegible]	[illegible]	[illegible]	[illegible]
01V36534	CC45-MRSA-IV, Berlin EMRSA	POS	[illegible]	[illegible]	[illegible]	[illegible]	[illegible]	[illegible]	[illegible]	[illegible]	[illegible]	[illegible]	[illegible]	[illegible]	[illegible]	[illegible]	[illegible]	[illegible]	[illegible]	[illegible]	[illegible]	[illegible]	[illegible]	[illegible]	[illegible]	[illegible]	[illegible]	[illegible]	[illegible]	[illegible]	[illegible]	[illegible]	[illegible]	[illegible]	[illegible]	[illegible]	[illegible]	[illegible]	[illegible]	[illegible]	[illegible]	[illegible]	[illegible]	[illegible]	[illegible]	[illegible]	[illegible]	[illegible]	[illegible]	[illegible]	[illegible]
01V36588	CC45-MRSA-IV, Berlin EMRSA	POS	[illegible]	[illegible]	[illegible]	[illegible]	[illegible]	[illegible]	[illegible]	[illegible]	[illegible]	[illegible]	[illegible]	[illegible]	[illegible]	[illegible]	[illegible]	[illegible]	[illegible]	[illegible]	[illegible]	[illegible]	[illegible]	[illegible]	[illegible]	[illegible]	[illegible]	[illegible]	[illegible]	[illegible]	[illegible]	[illegible]	[illegible]	[illegible]	[illegible]	[illegible]	[illegible]	[illegible]	[illegible]	[illegible]	[illegible]	[illegible]	[illegible]	[illegible]	[illegible]	[illegible]	[illegible]	[illegible]	[illegible]	[illegible]	[illegible]
01V37990	CC45-MRSA-IV, Berlin EMRSA	POS	[illegible]	[illegible]	[illegible]	[illegible]	[illegible]	[illegible]	[illegible]	[illegible]	[illegible]	[illegible]	[illegible]	[illegible]	[illegible]	[illegible]	[illegible]	[illegible]	[illegible]	[illegible]	[illegible]	[illegible]	[illegible]	[illegible]	[illegible]	[illegible]	[illegible]	[illegible]	[illegible]	[illegible]	[illegible]	[illegible]	[illegible]	[illegible]	[illegible]	[illegible]	[illegible]	[illegible]	[illegible]	[illegible]	[illegible]	[illegible]	[illegible]	[illegible]	[illegible]	[illegible]	[illegible]	[illegible]	[illegible]	[illegible]	[illegible]
01V39610	CC45-MRSA-IV, Berlin EMRSA	POS	[illegible]	[illegible]	[illegible]	[illegible]	[illegible]	[illegible]	[illegible]	[illegible]	[illegible]	[illegible]	[illegible]	[illegible]	[illegible]	[illegible]	[illegible]	[illegible]	[illegible]	[illegible]	[illegible]	[illegible]	[illegible]	[illegible]	[illegible]	[illegible]	[illegible]	[illegible]	[illegible]	[illegible]	[illegible]	[illegible]	[illegible]	[illegible]	[illegible]	[illegible]	[illegible]	[illegible]	[illegible]	[illegible]	[illegible]	[illegible]	[illegible]	[illegible]	[illegible]	[illegible]	[illegible]	[illegible]	[illegible]	[illegible]	[illegible]
01V40229	CC45-MRSA-IV, Berlin EMRSA	POS	[illegible]	[illegible]	[illegible]	[illegible]	[illegible]	[illegible]	[illegible]	[illegible]	[illegible]	[illegible]	[illegible]	[illegible]	[illegible]	[illegible]	[illegible]	[illegible]	[illegible]	[illegible]	[illegible]	[illegible]	[illegible]	[illegible]	[illegible]	[illegible]	[illegible]	[illegible]	[illegible]	[illegible]	[illegible]	[illegible]	[illegible]	[illegible]	[illegible]	[illegible]	[illegible]	[illegible]	[illegible]	[illegible]	[illegible]	[illegible]	[illegible]	[illegible]	[illegible]	[illegible]	[illegible]	[illegible]	[illegible]	[illegible]	[illegible]
01V40374	CC45-MRSA-IV, Berlin EMRSA																																																		
01V41056	CC45-MRSA-IV, Berlin EMRSA	POS	[illegible]	[illegible]	[illegible]	[illegible]	[illegible]	[illegible]	[illegible]	[illegible]	[illegible]	[illegible]	[illegible]	[illegible]	[illegible]	[illegible]	[illegible]	[illegible]	[illegible]	[illegible]	[illegible]	[illegible]	[illegible]	[illegible]	[illegible]	[illegible]	[illegible]	[illegible]	[illegible]	[illegible]	[illegible]	[illegible]	[illegible]	[illegible]	[illegible]	[illegible]	[illegible]	[illegible]	[illegible]	[illegible]	[illegible]	[illegible]	[illegible]	[illegible]	[illegible]	[illegible]	[illegible]	[illegible]	[illegible]	[illegible]	[illegible]
01V42009	CC45-MRSA-IV, Berlin EMRSA	POS	[illegible]	[illegible]	[illegible]	[illegible]	[illegible]	[illegible]	[illegible]	[illegible]	[illegible]	[illegible]	[illegible]	[illegible]	[illegible]	[illegible]	[illegible]	[illegible]	[illegible]	[illegible]	[illegible]	[illegible]	[illegible]	[illegible]	[illegible]	[illegible]	[illegible]	[illegible]	[illegible]	[illegible]	[illegible]	[illegible]	[illegible]	[illegible]	[illegible]	[illegible]	[illegible]	[illegible]	[illegible]	[illegible]	[illegible]	[illegible]	[illegible]	[illegible]	[illegible]	[illegible]	[illegible]	[illegible]	[illegible]	[illegible]	[illegible]
01V43511	CC45-MRSA-IV, Berlin EMRSA	POS	[illegible]	[illegible]	[illegible]	[illegible]	[illegible]	[illegible]	[illegible]	[illegible]	[illegible]	[illegible]	[illegible]	[illegible]	[illegible]	[illegible]	[illegible]	[illegible]	[illegible]	[illegible]	[illegible]	[illegible]	[illegible]	[illegible]	[illegible]	[illegible]	[illegible]	[illegible]	[illegible]	[illegible]	[illegible]	[illegible]	[illegible]	[illegible]	[illegible]	[illegible]	[illegible]	[illegible]	[illegible]	[illegible]	[illegible]	[illegible]	[illegible]	[illegible]	[illegible]	[illegible]	[illegible]	[illegible]	[illegible]	[illegible]	[illegible]
**2002**																																																			
02V10698	CC5/ST228-MRSA-I, South German EMRSA	POS	[illegible]	[illegible]	[illegible]	[illegible]	[illegible]	[illegible]	[illegible]	[illegible]	[illegible]	[illegible]	[illegible]	[illegible]	[illegible]	[illegible]	[illegible]	[illegible]	[illegible]	[illegible]	[illegible]	[illegible]	[illegible]	[illegible]	[illegible]	[illegible]	[illegible]	[illegible]	[illegible]	[illegible]	[illegible]	[illegible]	[illegible]	[illegible]	[illegible]	[illegible]	[illegible]	[illegible]	[illegible]	[illegible]	[illegible]	[illegible]	[illegible]	[illegible]	[illegible]	[illegible]	[illegible]	[illegible]	[illegible]	[illegible]	[illegible]
02V13140	CC5/ST228-MRSA-I, South German EMRSA	POS	[illegible]	[illegible]	[illegible]	[illegible]	[illegible]	[illegible]	[illegible]	[illegible]	[illegible]	[illegible]	[illegible]	[illegible]	[illegible]	[illegible]	[illegible]	[illegible]	[illegible]	[illegible]	[illegible]	[illegible]	[illegible]	[illegible]	[illegible]	[illegible]	[illegible]	[illegible]	[illegible]	[illegible]	[illegible]	[illegible]	[illegible]	[illegible]	[illegible]	[illegible]	[illegible]	[illegible]	[illegible]	[illegible]	[illegible]	[illegible]	[illegible]	[illegible]	[illegible]	[illegible]	[illegible]	[illegible]	[illegible]	[illegible]	[illegible]
02V20759	CC5/ST228-MRSA-I, South German EMRSA	POS	[illegible]	[illegible]	[illegible]	[illegible]	[illegible]	[illegible]	[illegible]	[illegible]	[illegible]	[illegible]	[illegible]	[illegible]	[illegible]	[illegible]	[illegible]	[illegible]	[illegible]	[illegible]	[illegible]	[illegible]	[illegible]	[illegible]	[illegible]	[illegible]	[illegible]	[illegible]	[illegible]	[illegible]	[illegible]	[illegible]	[illegible]	[illegible]	[illegible]	[illegible]	[illegible]	[illegible]	[illegible]	[illegible]	[illegible]	[illegible]	[illegible]	[illegible]	[illegible]	[illegible]	[illegible]	[illegible]	[illegible]	[illegible]	[illegible]
02B06271	CC5-MRSA-II, Rhine-Hesse EMRSA/UK-EMRSA-3	POS	[illegible]	[illegible]	[illegible]	[illegible]	[illegible]	[illegible]	[illegible]	[illegible]	[illegible]	[illegible]	[illegible]	[illegible]	[illegible]	[illegible]	[illegible]	[illegible]	[illegible]	[illegible]	[illegible]	[illegible]	[illegible]	[illegible]	[illegible]	[illegible]	[illegible]	[illegible]	[illegible]	[illegible]	[illegible]	[illegible]	[illegible]	[illegible]	[illegible]	[illegible]	[illegible]	[illegible]	[illegible]	[illegible]	[illegible]	[illegible]	[illegible]	[illegible]	[illegible]	[illegible]	[illegible]	[illegible]	[illegible]	[illegible]	[illegible]
02V04116	CC5-MRSA-II, Rhine-Hesse EMRSA/UK-EMRSA-3	POS	[illegible]	[illegible]	[illegible]	[illegible]	[illegible]	[illegible]	[illegible]	[illegible]	[illegible]	[illegible]	[illegible]	[illegible]	[illegible]	[illegible]	[illegible]	[illegible]	[illegible]	[illegible]	[illegible]	[illegible]	[illegible]	[illegible]	[illegible]	[illegible]	[illegible]	[illegible]	[illegible]	[illegible]	[illegible]	[illegible]	[illegible]	[illegible]	[illegible]	[illegible]	[illegible]	[illegible]	[illegible]	[illegible]	[illegible]	[illegible]	[illegible]	[illegible]	[illegible]	[illegible]	[illegible]	[illegible]	[illegible]	[illegible]	[illegible]
02V29922	CC5-MRSA-II, Rhine-Hesse EMRSA/UK-EMRSA-3																																																		
02V33634	CC5-MRSA-II, Rhine-Hesse EMRSA/UK-EMRSA-3	POS	[illegible]	[illegible]	[illegible]	[illegible]	[illegible]	[illegible]	[illegible]	[illegible]	[illegible]	[illegible]	[illegible]	[illegible]	[illegible]	[illegible]	[illegible]	[illegible]	[illegible]	[illegible]	[illegible]	[illegible]	[illegible]	[illegible]	[illegible]	[illegible]	[illegible]	[illegible]	[illegible]	[illegible]	[illegible]	[illegible]	[illegible]	[illegible]	[illegible]	[illegible]	[illegible]	[illegible]	[illegible]	[illegible]	[illegible]	[illegible]	[illegible]	[illegible]	[illegible]	[illegible]	[illegible]	[illegible]	[illegible]	[illegible]	[illegible]
02V34748	CC5-MRSA-II, Rhine-Hesse EMRSA/UK-EMRSA-3	POS	[illegible]	[illegible]	[illegible]	[illegible]	[illegible]	[illegible]	[illegible]	[illegible]	[illegible]	[illegible]	[illegible]	[illegible]	[illegible]	[illegible]	[illegible]	[illegible]	[illegible]	[illegible]	[illegible]	[illegible]	[illegible]	[illegible]	[illegible]	[illegible]	[illegible]	[illegible]	[illegible]	[illegible]	[illegible]	[illegible]	[illegible]	[illegible]	[illegible]	[illegible]	[illegible]	[illegible]	[illegible]	[illegible]	[illegible]	[illegible]	[illegible]	[illegible]	[illegible]	[illegible]	[illegible]	[illegible]	[illegible]	[illegible]	[illegible]
02V17002	CC8/ST254-MRSA-(atypical SCCmec), Hannover EMRSA	POS	[illegible]	[illegible]	[illegible]	[illegible]	[illegible]	[illegible]	[illegible]	[illegible]	[illegible]	[illegible]	[illegible]	[illegible]	[illegible]	[illegible]	[illegible]	[illegible]	[illegible]	[illegible]	[illegible]	[illegible]	[illegible]	[illegible]	[illegible]	[illegible]	[illegible]	[illegible]	[illegible]	[illegible]	[illegible]	[illegible]	[illegible]	[illegible]	[illegible]	[illegible]	[illegible]	[illegible]	[illegible]	[illegible]	[illegible]	[illegible]	[illegible]	[illegible]	[illegible]	[illegible]	[illegible]	[illegible]	[illegible]	[illegible]	[illegible]
02V33579	CC8-MRSA-IV, USA500	POS	[illegible]	[illegible]	[illegible]	[illegible]	[illegible]	[illegible]	[illegible]	[illegible]	[illegible]	[illegible]	[illegible]	[illegible]	[illegible]	[illegible]	[illegible]	[illegible]	[illegible]	[illegible]	[illegible]	[illegible]	[illegible]	[illegible]	[illegible]	[illegible]	[illegible]	[illegible]	[illegible]	[illegible]	[illegible]	[illegible]	[illegible]	[illegible]	[illegible]	[illegible]	[illegible]	[illegible]	[illegible]	[illegible]	[illegible]	[illegible]	[illegible]	[illegible]	[illegible]	[illegible]	[illegible]	[illegible]	[illegible]	[illegible]	[illegible]
02V15498	CC22-MRSA-IV [PVL+]	POS	[illegible]	[illegible]	[illegible]	[illegible]	[illegible]	[illegible]	[illegible]	[illegible]	[illegible]	[illegible]	[illegible]	[illegible]	[illegible]	[illegible]	[illegible]	[illegible]	[illegible]	[illegible]	[illegible]	[illegible]	[illegible]	[illegible]	[illegible]	[illegible]	[illegible]	[illegible]	[illegible]	[illegible]	[illegible]	[illegible]	[illegible]	[illegible]	[illegible]	[illegible]	[illegible]	[illegible]	[illegible]	[illegible]	[illegible]	[illegible]	[illegible]	[illegible]	[illegible]	[illegible]	[illegible]	[illegible]	[illegible]	[illegible]	[illegible]
02C05319	CC22-MRSA-IV, Barnim/UK-EMRSA-15	POS	[illegible]	[illegible]	[illegible]	[illegible]	[illegible]	[illegible]	[illegible]	[illegible]	[illegible]	[illegible]	[illegible]	[illegible]	[illegible]	[illegible]	[illegible]	[illegible]	[illegible]	[illegible]	[illegible]	[illegible]	[illegible]	[illegible]	[illegible]	[illegible]	[illegible]	[illegible]	[illegible]	[illegible]	[illegible]	[illegible]	[illegible]	[illegible]	[illegible]	[illegible]	[illegible]	[illegible]	[illegible]	[illegible]	[illegible]	[illegible]	[illegible]	[illegible]	[illegible]	[illegible]	[illegible]	[illegible]	[illegible]	[illegible]	[illegible]
02U10255	CC22-MRSA-IV, Barnim/UK-EMRSA-15	POS	[illegible]	[illegible]	[illegible]	[illegible]	[illegible]	[illegible]	[illegible]	[illegible]	[illegible]	[illegible]	[illegible]	[illegible]	[illegible]	[illegible]	[illegible]	[illegible]	[illegible]	[illegible]	[illegible]	[illegible]	[illegible]	[illegible]	[illegible]	[illegible]	[illegible]	[illegible]	[illegible]	[illegible]	[illegible]	[illegible]	[illegible]	[illegible]	[illegible]	[illegible]	[illegible]	[illegible]	[illegible]	[illegible]	[illegible]	[illegible]	[illegible]	[illegible]	[illegible]	[illegible]	[illegible]	[illegible]	[illegible]	[illegible]	[illegible]
02V12175	CC22-MRSA-IV, Barnim/UK-EMRSA-15	POS	[illegible]	[illegible]	[illegible]	[illegible]	[illegible]	[illegible]	[illegible]	[illegible]	[illegible]	[illegible]	[illegible]	[illegible]	[illegible]	[illegible]	[illegible]	[illegible]	[illegible]	[illegible]	[illegible]	[illegible]	[illegible]	[illegible]	[illegible]	[illegible]	[illegible]	[illegible]	[illegible]	[illegible]	[illegible]	[illegible]	[illegible]	[illegible]	[illegible]	[illegible]	[illegible]	[illegible]	[illegible]	[illegible]	[illegible]	[illegible]	[illegible]	[illegible]	[illegible]	[illegible]	[illegible]	[illegible]	[illegible]	[illegible]	[illegible]
02V13054	CC22-MRSA-IV, Barnim/UK-EMRSA-15	POS	[illegible]	[illegible]	[illegible]	[illegible]	[illegible]	[illegible]	[illegible]	[illegible]	[illegible]	[illegible]	[illegible]	[illegible]	[illegible]	[illegible]	[illegible]	[illegible]	[illegible]	[illegible]	[illegible]	[illegible]	[illegible]	[illegible]	[illegible]	[illegible]	[illegible]	[illegible]	[illegible]	[illegible]	[illegible]	[illegible]	[illegible]	[illegible]	[illegible]	[illegible]	[illegible]	[illegible]	[illegible]	[illegible]	[illegible]	[illegible]	[illegible]	[illegible]	[illegible]	[illegible]	[illegible]	[illegible]	[illegible]	[illegible]	[illegible]
02V13950	CC22-MRSA-IV, Barnim/UK-EMRSA-15	POS	[illegible]	[illegible]	[illegible]	[illegible]	[illegible]	[illegible]	[illegible]	[illegible]	[illegible]	[illegible]	[illegible]	[illegible]	[illegible]	[illegible]	[illegible]	[illegible]	[illegible]	[illegible]	[illegible]	[illegible]	[illegible]	[illegible]	[illegible]	[illegible]	[illegible]	[illegible]	[illegible]	[illegible]	[illegible]	[illegible]	[illegible]	[illegible]	[illegible]	[illegible]	[illegible]	[illegible]	[illegible]	[illegible]	[illegible]	[illegible]	[illegible]	[illegible]	[illegible]	[illegible]	[illegible]	[illegible]	[illegible]	[illegible]	[illegible]
02V21259	CC22-MRSA-IV, Barnim/UK-EMRSA-15	POS	[illegible]	[illegible]	[illegible]	[illegible]	[illegible]	[illegible]	[illegible]	[illegible]	[illegible]	[illegible]	[illegible]	[illegible]	[illegible]	[illegible]	[illegible]	[illegible]	[illegible]	[illegible]	[illegible]	[illegible]	[illegible]	[illegible]	[illegible]	[illegible]	[illegible]	[illegible]	[illegible]	[illegible]	[illegible]	[illegible]	[illegible]	[illegible]	[illegible]	[illegible]	[illegible]	[illegible]	[illegible]	[illegible]	[illegible]	[illegible]	[illegible]	[illegible]	[illegible]	[illegible]	[illegible]	[illegible]	[illegible]	[illegible]	[illegible]
02V22657	CC22-MRSA-IV, Barnim/UK-EMRSA-15	POS	[illegible]	[illegible]	[illegible]	[illegible]	[illegible]	[illegible]	[illegible]	[illegible]	[illegible]	[illegible]	[illegible]	[illegible]	[illegible]	[illegible]	[illegible]	[illegible]	[illegible]	[illegible]	[illegible]	[illegible]	[illegible]	[illegible]	[illegible]	[illegible]	[illegible]	[illegible]	[illegible]	[illegible]	[illegible]	[illegible]	[illegible]	[illegible]	[illegible]	[illegible]	[illegible]	[illegible]	[illegible]	[illegible]	[illegible]	[illegible]	[illegible]	[illegible]	[illegible]	[illegible]	[illegible]	[illegible]	[illegible]	[illegible]	[illegible]
02V29051	CC22-MRSA-IV, Barnim/UK-EMRSA-15	POS	[illegible]	[illegible]	[illegible]	[illegible]	[illegible]	[illegible]	[illegible]	[illegible]	[illegible]	[illegible]	[illegible]	[illegible]	[illegible]	[illegible]	[illegible]	[illegible]	[illegible]	[illegible]	[illegible]	[illegible]	[illegible]	[illegible]	[illegible]	[illegible]	[illegible]	[illegible]	[illegible]	[illegible]	[illegible]	[illegible]	[illegible]	[illegible]	[illegible]	[illegible]	[illegible]	[illegible]	[illegible]	[illegible]	[illegible]	[illegible]	[illegible]	[illegible]	[illegible]	[illegible]	[illegible]	[illegible]	[illegible]	[illegible]	[illegible]
02V30792	CC30/ST36-MRSA-II, UK-EMRSA-16	POS	[illegible]	[illegible]	[illegible]	[illegible]	[illegible]	[illegible]	[illegible]	[illegible]	[illegible]	[illegible]	[illegible]	[illegible]	[illegible]	[illegible]	[illegible]	[illegible]	[illegible]	[illegible]	[illegible]	[illegible]	[illegible]	[illegible]	[illegible]	[illegible]	[illegible]	[illegible]	[illegible]	[illegible]	[illegible]	[illegible]	[illegible]	[illegible]	[illegible]	[illegible]	[illegible]	[illegible]	[illegible]	[illegible]	[illegible]	[illegible]	[illegible]	[illegible]	[illegible]	[illegible]	[illegible]	[illegible]	[illegible]	[illegible]	[illegible]
02U07096	CC45-MRSA-IV, Berlin EMRSA	POS	[illegible]	[illegible]	[illegible]	[illegible]	[illegible]	[illegible]	[illegible]	[illegible]	[illegible]	[illegible]	[illegible]	[illegible]	[illegible]	[illegible]	[illegible]	[illegible]	[illegible]	[illegible]	[illegible]	[illegible]	[illegible]	[illegible]	[illegible]	[illegible]	[illegible]	[illegible]	[illegible]	[illegible]	[illegible]	[illegible]	[illegible]	[illegible]	[illegible]	[illegible]	[illegible]	[illegible]	[illegible]	[illegible]	[illegible]	[illegible]	[illegible]	[illegible]	[illegible]	[illegible]	[illegible]	[illegible]	[illegible]	[illegible]	[illegible]
02V00570	CC45-MRSA-IV, Berlin EMRSA	POS	[illegible]	[illegible]	[illegible]	[illegible]	[illegible]	[illegible]	[illegible]	[illegible]	[illegible]	[illegible]	[illegible]	[illegible]	[illegible]	[illegible]	[illegible]	[illegible]	[illegible]	[illegible]	[illegible]	[illegible]	[illegible]	[illegible]	[illegible]	[illegible]	[illegible]	[illegible]	[illegible]	[illegible]	[illegible]	[illegible]	[illegible]	[illegible]	[illegible]	[illegible]	[illegible]	[illegible]	[illegible]	[illegible]	[illegible]	[illegible]	[illegible]	[illegible]	[illegible]	[illegible]	[illegible]	[illegible]	[illegible]	[illegible]	[illegible]
02V01730	CC45-MRSA-IV, Berlin EMRSA	POS	[illegible]	[illegible]	[illegible]	[illegible]	[illegible]	[illegible]	[illegible]	[illegible]	[illegible]	[illegible]	[illegible]	[illegible]	[illegible]	[illegible]	[illegible]	[illegible]	[illegible]	[illegible]	[illegible]	[illegible]	[illegible]	[illegible]	[illegible]	[illegible]	[illegible]	[illegible]	[illegible]	[illegible]	[illegible]	[illegible]	[illegible]	[illegible]	[illegible]	[illegible]	[illegible]	[illegible]	[illegible]	[illegible]	[illegible]	[illegible]	[illegible]	[illegible]	[illegible]	[illegible]	[illegible]	[illegible]	[illegible]	[illegible]	[illegible]
02V01870	CC45-MRSA-IV, Berlin EMRSA	POS	[illegible]	[illegible]	[illegible]	[illegible]	[illegible]	[illegible]	[illegible]	[illegible]	[illegible]	[illegible]	[illegible]	[illegible]	[illegible]	[illegible]	[illegible]	[illegible]	[illegible]	[illegible]	[illegible]	[illegible]	[illegible]	[illegible]	[illegible]	[illegible]	[illegible]	[illegible]	[illegible]	[illegible]	[illegible]	[illegible]	[illegible]	[illegible]	[illegible]	[illegible]	[illegible]	[illegible]	[illegible]	[illegible]	[illegible]	[illegible]	[illegible]	[illegible]	[illegible]	[illegible]	[illegible]	[illegible]	[illegible]	[illegible]	[illegible]
02V04750	CC45-MRSA-IV, Berlin EMRSA	POS	[illegible]	[illegible]	[illegible]	[illegible]	[illegible]	[illegible]	[illegible]	[illegible]	[illegible]	[illegible]	[illegible]	[illegible]	[illegible]	[illegible]	[illegible]	[illegible]	[illegible]	[illegible]	[illegible]	[illegible]	[illegible]	[illegible]	[illegible]	[illegible]	[illegible]	[illegible]	[illegible]	[illegible]	[illegible]	[illegible]	[illegible]	[illegible]	[illegible]	[illegible]	[illegible]	[illegible]	[illegible]	[illegible]	[illegible]	[illegible]	[illegible]	[illegible]	[illegible]	[illegible]	[illegible]	[illegible]	[illegible]	[illegible]	[illegible]
02V06592	CC45-MRSA-IV, Berlin EMRSA	POS	[illegible]	[illegible]	[illegible]	[illegible]	[illegible]	[illegible]	[illegible]	[illegible]	[illegible]	[illegible]	[illegible]	[illegible]	[illegible]	[illegible]	[illegible]	[illegible]	[illegible]	[illegible]	[illegible]	[illegible]	[illegible]	[illegible]	[illegible]	[illegible]	[illegible]	[illegible]	[illegible]	[illegible]	[illegible]	[illegible]	[illegible]	[illegible]	[illegible]	[illegible]	[illegible]	[illegible]	[illegible]	[illegible]	[illegible]	[illegible]	[illegible]	[illegible]	[illegible]	[illegible]	[illegible]	[illegible]	[illegible]	[illegible]	[illegible]
02V10868	CC45-MRSA-IV, Berlin EMRSA	POS	[illegible]	[illegible]	[illegible]	[illegible]	[illegible]	[illegible]	[illegible]	[illegible]	[illegible]	[illegible]	[illegible]	[illegible]	[illegible]	[illegible]	[illegible]	[illegible]	[illegible]	[illegible]	[illegible]	[illegible]	[illegible]	[illegible]	[illegible]	[illegible]	[illegible]	[illegible]	[illegible]	[illegible]	[illegible]	[illegible]	[illegible]	[illegible]	[illegible]	[illegible]	[illegible]	[illegible]	[illegible]	[illegible]	[illegible]	[illegible]	[illegible]	[illegible]	[illegible]	[illegible]	[illegible]	[illegible]	[illegible]	[illegible]	[illegible]
02V12394	CC45-MRSA-IV, Berlin EMRSA	POS	[illegible]	[illegible]	[illegible]	[illegible]	[illegible]	[illegible]	[illegible]	[illegible]	[illegible]	[illegible]	[illegible]	[illegible]	[illegible]	[illegible]	[illegible]	[illegible]	[illegible]	[illegible]	[illegible]	[illegible]	[illegible]	[illegible]	[illegible]	[illegible]	[illegible]	[illegible]	[illegible]	[illegible]	[illegible]	[illegible]	[illegible]	[illegible]	[illegible]	[illegible]	[illegible]	[illegible]	[illegible]	[illegible]	[illegible]	[illegible]	[illegible]	[illegible]	[illegible]	[illegible]	[illegible]	[illegible]	[illegible]	[illegible]	[illegible]
02V12942	CC45-MRSA-IV, Berlin EMRSA	POS	[illegible]	[illegible]	[illegible]	[illegible]	[illegible]	[illegible]	[illegible]	[illegible]	[illegible]	[illegible]	[illegible]	[illegible]	[illegible]	[illegible]	[illegible]	[illegible]	[illegible]	[illegible]	[illegible]	[illegible]	[illegible]	[illegible]	[illegible]	[illegible]	[illegible]	[illegible]	[illegible]	[illegible]	[illegible]	[illegible]	[illegible]	[illegible]	[illegible]	[illegible]	[illegible]	[illegible]	[illegible]	[illegible]	[illegible]	[illegible]	[illegible]	[illegible]	[illegible]	[illegible]	[illegible]	[illegible]	[illegible]	[illegible]	[illegible]
02V13026	CC45-MRSA-IV, Berlin EMRSA	POS	[illegible]	[illegible]	[illegible]	[illegible]	[illegible]	[illegible]	[illegible]	[illegible]	[illegible]	[illegible]	[illegible]	[illegible]	[illegible]	[illegible]	[illegible]	[illegible]	[illegible]	[illegible]	[illegible]	[illegible]	[illegible]	[illegible]	[illegible]	[illegible]	[illegible]	[illegible]	[illegible]	[illegible]	[illegible]	[illegible]	[illegible]	[illegible]	[illegible]	[illegible]	[illegible]	[illegible]	[illegible]	[illegible]	[illegible]	[illegible]	[illegible]	[illegible]	[illegible]	[illegible]	[illegible]	[illegible]	[illegible]	[illegible]	[illegible]
02V14700	CC45-MRSA-IV, Berlin EMRSA																																																		
02V15358	CC45-MRSA-IV, Berlin EMRSA	POS	[illegible]	[illegible]	[illegible]	[illegible]	[illegible]	[illegible]	[illegible]	[illegible]	[illegible]	[illegible]	[illegible]	[illegible]	[illegible]	[illegible]	[illegible]	[illegible]	[illegible]	[illegible]	[illegible]	[illegible]	[illegible]	[illegible]	[illegible]	[illegible]	[illegible]	[illegible]	[illegible]	[illegible]	[illegible]	[illegible]	[illegible]	[illegible]	[illegible]	[illegible]	[illegible]	[illegible]	[illegible]	[illegible]	[illegible]	[illegible]	[illegible]	[illegible]	[illegible]	[illegible]	[illegible]	[illegible]	[illegible]	[illegible]	[illegible]
02V16654	CC45-MRSA-IV, Berlin EMRSA	POS	[illegible]	[illegible]	[illegible]	[illegible]	[illegible]	[illegible]	[illegible]	[illegible]	[illegible]	[illegible]	[illegible]	[illegible]	[illegible]	[illegible]	[illegible]	[illegible]	[illegible]	[illegible]	[illegible]	[illegible]	[illegible]	[illegible]	[illegible]	[illegible]	[illegible]	[illegible]	[illegible]	[illegible]	[illegible]	[illegible]	[illegible]	[illegible]	[illegible]	[illegible]	[illegible]	[illegible]	[illegible]	[illegible]	[illegible]	[illegible]	[illegible]	[illegible]	[illegible]	[illegible]	[illegible]	[illegible]	[illegible]	[illegible]	[illegible]
02V17120	CC45-MRSA-IV, Berlin EMRSA	POS	[illegible]	[illegible]	[illegible]	[illegible]	[illegible]	[illegible]	[illegible]	[illegible]	[illegible]	[illegible]	[illegible]	[illegible]	[illegible]	[illegible]	[illegible]	[illegible]	[illegible]	[illegible]	[illegible]	[illegible]	[illegible]	[illegible]	[illegible]	[illegible]	[illegible]	[illegible]	[illegible]	[illegible]	[illegible]	[illegible]	[illegible]	[illegible]	[illegible]	[illegible]	[illegible]	[illegible]	[illegible]	[illegible]	[illegible]	[illegible]	[illegible]	[illegible]	[illegible]	[illegible]	[illegible]	[illegible]	[illegible]	[illegible]	[illegible]
02V17136	CC45-MRSA-IV, Berlin EMRSA	POS	[illegible]	[illegible]	[illegible]	[illegible]	[illegible]	[illegible]	[illegible]	[illegible]	[illegible]	[illegible]	[illegible]	[illegible]	[illegible]	[illegible]	[illegible]	[illegible]	[illegible]	[illegible]	[illegible]	[illegible]	[illegible]	[illegible]	[illegible]	[illegible]	[illegible]	[illegible]	[illegible]	[illegible]	[illegible]	[illegible]	[illegible]	[illegible]	[illegible]	[illegible]	[illegible]	[illegible]	[illegible]	[illegible]	[illegible]	[illegible]	[illegible]	[illegible]	[illegible]	[illegible]	[illegible]	[illegible]	[illegible]	[illegible]	[illegible]
02V17558	CC45-MRSA-IV, Berlin EMRSA	POS	[illegible]	[illegible]	[illegible]	[illegible]	[illegible]	[illegible]	[illegible]	[illegible]	[illegible]	[illegible]	[illegible]	[illegible]	[illegible]	[illegible]	[illegible]	[illegible]	[illegible]	[illegible]	[illegible]	[illegible]	[illegible]	[illegible]	[illegible]	[illegible]	[illegible]	[illegible]	[illegible]	[illegible]	[illegible]	[illegible]	[illegible]	[illegible]	[illegible]	[illegible]	[illegible]	[illegible]	[illegible]	[illegible]	[illegible]	[illegible]	[illegible]	[illegible]	[illegible]	[illegible]	[illegible]	[illegible]	[illegible]	[illegible]	[illegible]
02V17665	CC45-MRSA-IV, Berlin EMRSA	POS	[illegible]	[illegible]	[illegible]	[illegible]	[illegible]	[illegible]	[illegible]	[illegible]	[illegible]	[illegible]	[illegible]	[illegible]	[illegible]	[illegible]	[illegible]	[illegible]	[illegible]	[illegible]	[illegible]	[illegible]	[illegible]	[illegible]	[illegible]	[illegible]	[illegible]	[illegible]	[illegible]	[illegible]	[illegible]	[illegible]	[illegible]	[illegible]	[illegible]	[illegible]	[illegible]	[illegible]	[illegible]	[illegible]	[illegible]	[illegible]	[illegible]	[illegible]	[illegible]	[illegible]	[illegible]	[illegible]	[illegible]	[illegible]	[illegible]
02V20390	CC45-MRSA-IV, Berlin EMRSA	POS	[illegible]	[illegible]	[illegible]	[illegible]	[illegible]	[illegible]	[illegible]	[illegible]	[illegible]	[illegible]	[illegible]	[illegible]	[illegible]	[illegible]	[illegible]	[illegible]	[illegible]	[illegible]	[illegible]	[illegible]	[illegible]	[illegible]	[illegible]	[illegible]	[illegible]	[illegible]	[illegible]	[illegible]	[illegible]	[illegible]	[illegible]	[illegible]	[illegible]	[illegible]	[illegible]	[illegible]	[illegible]	[illegible]	[illegible]	[illegible]	[illegible]	[illegible]	[illegible]	[illegible]	[illegible]	[illegible]	[illegible]	[illegible]	[illegible]
02V20626	CC45-MRSA-IV, Berlin EMRSA	POS	[illegible]	[illegible]	[illegible]	[illegible]	[illegible]	[illegible]	[illegible]	[illegible]	[illegible]	[illegible]	[illegible]	[illegible]	[illegible]	[illegible]	[illegible]	[illegible]	[illegible]	[illegible]	[illegible]	[illegible]	[illegible]	[illegible]	[illegible]	[illegible]	[illegible]	[illegible]	[illegible]	[illegible]	[illegible]	[illegible]	[illegible]	[illegible]	[illegible]	[illegible]	[illegible]	[illegible]	[illegible]	[illegible]	[illegible]	[illegible]	[illegible]	[illegible]	[illegible]	[illegible]	[illegible]	[illegible]	[illegible]	[illegible]	[illegible]
02V20680	CC45-MRSA-IV, Berlin EMRSA	POS	[illegible]	[illegible]	[illegible]	[illegible]	[illegible]	[illegible]	[illegible]	[illegible]	[illegible]	[illegible]	[illegible]	[illegible]	[illegible]	[illegible]	[illegible]	[illegible]	[illegible]	[illegible]	[illegible]	[illegible]	[illegible]	[illegible]	[illegible]	[illegible]	[illegible]	[illegible]	[illegible]	[illegible]	[illegible]	[illegible]	[illegible]	[illegible]	[illegible]	[illegible]	[illegible]	[illegible]	[illegible]	[illegible]	[illegible]	[illegible]	[illegible]	[illegible]	[illegible]	[illegible]	[illegible]	[illegible]	[illegible]	[illegible]	[illegible]
02V21980	CC45-MRSA-IV, Berlin EMRSA	POS	[illegible]	[illegible]	[illegible]	[illegible]	[illegible]	[illegible]	[illegible]	[illegible]	[illegible]	[illegible]	[illegible]	[illegible]	[illegible]	[illegible]	[illegible]	[illegible]	[illegible]	[illegible]	[illegible]	[illegible]	[illegible]	[illegible]	[illegible]	[illegible]	[illegible]	[illegible]	[illegible]	[illegible]	[illegible]	[illegible]	[illegible]	[illegible]	[illegible]	[illegible]	[illegible]	[illegible]	[illegible]	[illegible]	[illegible]	[illegible]	[illegible]	[illegible]	[illegible]	[illegible]	[illegible]	[illegible]	[illegible]	[illegible]	[illegible]
02V22570	CC45-MRSA-IV, Berlin EMRSA	POS	[illegible]	[illegible]	[illegible]	[illegible]	[illegible]	[illegible]	[illegible]	[illegible]	[illegible]	[illegible]	[illegible]	[illegible]	[illegible]	[illegible]	[illegible]	[illegible]	[illegible]	[illegible]	[illegible]	[illegible]	[illegible]	[illegible]	[illegible]	[illegible]	[illegible]	[illegible]	[illegible]	[illegible]	[illegible]	[illegible]	[illegible]	[illegible]	[illegible]	[illegible]	[illegible]	[illegible]	[illegible]	[illegible]	[illegible]	[illegible]	[illegible]	[illegible]	[illegible]	[illegible]	[illegible]	[illegible]	[illegible]	[illegible]	[illegible]
02V22982	CC45-MRSA-IV, Berlin EMRSA	POS	[illegible]	[illegible]	[illegible]	[illegible]	[illegible]	[illegible]	[illegible]	[illegible]	[illegible]	[illegible]	[illegible]	[illegible]	[illegible]	[illegible]	[illegible]	[illegible]	[illegible]	[illegible]	[illegible]	[illegible]	[illegible]	[illegible]	[illegible]	[illegible]	[illegible]	[illegible]	[illegible]	[illegible]	[illegible]	[illegible]	[illegible]	[illegible]	[illegible]	[illegible]	[illegible]	[illegible]	[illegible]	[illegible]	[illegible]	[illegible]	[illegible]	[illegible]	[illegible]	[illegible]	[illegible]	[illegible]	[illegible]	[illegible]	[illegible]
02V25503	CC45-MRSA-IV, Berlin EMRSA	POS	[illegible]	[illegible]	[illegible]	[illegible]	[illegible]	[illegible]	[illegible]	[illegible]	[illegible]	[illegible]	[illegible]	[illegible]	[illegible]	[illegible]	[illegible]	[illegible]	[illegible]	[illegible]	[illegible]	[illegible]	[illegible]	[illegible]	[illegible]	[illegible]	[illegible]	[illegible]	[illegible]	[illegible]	[illegible]	[illegible]	[illegible]	[illegible]	[illegible]	[illegible]	[illegible]	[illegible]	[illegible]	[illegible]	[illegible]	[illegible]	[illegible]	[illegible]	[illegible]	[illegible]	[illegible]	[illegible]	[illegible]	[illegible]	[illegible]
02V28454	CC45-MRSA-IV, Berlin EMRSA	POS	[illegible]	[illegible]	[illegible]	[illegible]	[illegible]	[illegible]	[illegible]	[illegible]	[illegible]	[illegible]	[illegible]	[illegible]	[illegible]	[illegible]	[illegible]	[illegible]	[illegible]	[illegible]	[illegible]	[illegible]	[illegible]	[illegible]	[illegible]	[illegible]	[illegible]	[illegible]	[illegible]	[illegible]	[illegible]	[illegible]	[illegible]	[illegible]	[illegible]	[illegible]	[illegible]	[illegible]	[illegible]	[illegible]	[illegible]	[illegible]	[illegible]	[illegible]	[illegible]	[illegible]	[illegible]	[illegible]	[illegible]	[illegible]	[illegible]
02V29373	CC45-MRSA-IV, Berlin EMRSA	POS	[illegible]	[illegible]	[illegible]	[illegible]	[illegible]	[illegible]	[illegible]	[illegible]	[illegible]	[illegible]	[illegible]	[illegible]	[illegible]	[illegible]	[illegible]	[illegible]	[illegible]	[illegible]	[illegible]	[illegible]	[illegible]	[illegible]	[illegible]	[illegible]	[illegible]	[illegible]	[illegible]	[illegible]	[illegible]	[illegible]	[illegible]	[illegible]	[illegible]	[illegible]	[illegible]	[illegible]	[illegible]	[illegible]	[illegible]	[illegible]	[illegible]	[illegible]	[illegible]	[illegible]	[illegible]	[illegible]	[illegible]	[illegible]	[illegible]
02V30321	CC45-MRSA-IV, Berlin EMRSA	POS	[illegible]	[illegible]	[illegible]	[illegible]	[illegible]	[illegible]	[illegible]	[illegible]	[illegible]	[illegible]	[illegible]	[illegible]	[illegible]	[illegible]	[illegible]	[illegible]	[illegible]	[illegible]	[illegible]	[illegible]	[illegible]	[illegible]	[illegible]	[illegible]	[illegible]	[illegible]	[illegible]	[illegible]	[illegible]	[illegible]	[illegible]	[illegible]	[illegible]	[illegible]	[illegible]	[illegible]	[illegible]	[illegible]	[illegible]	[illegible]	[illegible]	[illegible]	[illegible]	[illegible]	[illegible]	[illegible]	[illegible]	[illegible]	[illegible]
02V30341	CC45-MRSA-IV, Berlin EMRSA	POS	[illegible]	[illegible]	[illegible]	[illegible]	[illegible]	[illegible]	[illegible]	[illegible]	[illegible]	[illegible]	[illegible]	[illegible]	[illegible]	[illegible]	[illegible]	[illegible]	[illegible]	[illegible]	[illegible]	[illegible]	[illegible]	[illegible]	[illegible]	[illegible]	[illegible]	[illegible]	[illegible]	[illegible]	[illegible]	[illegible]	[illegible]	[illegible]	[illegible]	[illegible]	[illegible]	[illegible]	[illegible]	[illegible]	[illegible]	[illegible]	[illegible]	[illegible]	[illegible]	[illegible]	[illegible]	[illegible]	[illegible]	[illegible]	[illegible]
02V31377	CC45-MRSA-IV, Berlin EMRSA	POS	[illegible]	[illegible]	[illegible]	[illegible]	[illegible]	[illegible]	[illegible]	[illegible]	[illegible]	[illegible]	[illegible]	[illegible]	[illegible]	[illegible]	[illegible]	[illegible]	[illegible]	[illegible]	[illegible]	[illegible]	[illegible]	[illegible]	[illegible]	[illegible]	[illegible]	[illegible]	[illegible]	[illegible]	[illegible]	[illegible]	[illegible]	[illegible]	[illegible]	[illegible]	[illegible]	[illegible]	[illegible]	[illegible]	[illegible]	[illegible]	[illegible]	[illegible]	[illegible]	[illegible]	[illegible]	[illegible]	[illegible]	[illegible]	[illegible]
02V31941	CC45-MRSA-IV, Berlin EMRSA	POS	[illegible]	[illegible]	[illegible]	[illegible]	[illegible]	[illegible]	[illegible]	[illegible]	[illegible]	[illegible]	[illegible]	[illegible]	[illegible]	[illegible]	[illegible]	[illegible]	[illegible]	[illegible]	[illegible]	[illegible]	[illegible]	[illegible]	[illegible]	[illegible]	[illegible]	[illegible]	[illegible]	[illegible]	[illegible]	[illegible]	[illegible]	[illegible]	[illegible]	[illegible]	[illegible]	[illegible]	[illegible]	[illegible]	[illegible]	[illegible]	[illegible]	[illegible]	[illegible]	[illegible]	[illegible]	[illegible]	[illegible]	[illegible]	[illegible]
02V34679	CC45-MRSA-IV, Berlin EMRSA	POS	[illegible]	[illegible]	[illegible]	[illegible]	[illegible]	[illegible]	[illegible]	[illegible]	[illegible]	[illegible]	[illegible]	[illegible]	[illegible]	[illegible]	[illegible]	[illegible]	[illegible]	[illegible]	[illegible]	[illegible]	[illegible]	[illegible]	[illegible]	[illegible]	[illegible]	[illegible]	[illegible]	[illegible]	[illegible]	[illegible]	[illegible]	[illegible]	[illegible]	[illegible]	[illegible]	[illegible]	[illegible]	[illegible]	[illegible]	[illegible]	[illegible]	[illegible]	[illegible]	[illegible]	[illegible]	[illegible]	[illegible]	[illegible]	[illegible]
02V35779	CC45-MRSA-IV, Berlin EMRSA	POS	[illegible]	[illegible]	[illegible]	[illegible]	[illegible]	[illegible]	[illegible]	[illegible]	[illegible]	[illegible]	[illegible]	[illegible]	[illegible]	[illegible]	[illegible]	[illegible]	[illegible]	[illegible]	[illegible]	[illegible]	[illegible]	[illegible]	[illegible]	[illegible]	[illegible]	[illegible]	[illegible]	[illegible]	[illegible]	[illegible]	[illegible]	[illegible]	[illegible]	[illegible]	[illegible]	[illegible]	[illegible]	[illegible]	[illegible]	[illegible]	[illegible]	[illegible]	[illegible]	[illegible]	[illegible]	[illegible]	[illegible]	[illegible]	[illegible]
02V36419	CC45-MRSA-IV, Berlin EMRSA																																																		
02V36469	CC45-MRSA-IV, Berlin EMRSA	POS	[illegible]	[illegible]	[illegible]	[illegible]	[illegible]	[illegible]	[illegible]	[illegible]	[illegible]	[illegible]	[illegible]	[illegible]	[illegible]	[illegible]	[illegible]	[illegible]	[illegible]	[illegible]	[illegible]	[illegible]	[illegible]	[illegible]	[illegible]	[illegible]	[illegible]	[illegible]	[illegible]	[illegible]	[illegible]	[illegible]	[illegible]	[illegible]	[illegible]	[illegible]	[illegible]	[illegible]	[illegible]	[illegible]	[illegible]	[illegible]	[illegible]	[illegible]	[illegible]	[illegible]	[illegible]	[illegible]	[illegible]	[illegible]	[illegible]

ISOLATE:	STRAIN	WARD	AGE	GENDER	SPECIES MARKER							REGULATORY GENES								METHICILLIN RESISTANCE AND SCCmec TYPING																									
					Ribosomal sequence from S. aureus	glyceraldehyde 3-phosphate dehydro-genase, locus 1	katalase A	coagulase	thermo-stable extracellular nuclease	Protein A	IgG-binding protein	staphylo-coccal accessory regulator A	histidine protein kinase, sae locus	sensor protein	accessory gene regulator allele I	accessory gene regulator allele II	accessory gene regulator allele III	accessory gene regulator allele IV	haemolysin delta	alternate penicillin binding protein 2, defining MRSA	truncated signal transducer protein MecR1	glycerophosphoryl diester phosphodi-esterase, associated with mecA	cassette chromosome recombinase genes A/B-1		plasmin-sensitive surface protein	hypothetical protein from SCCmec elements	cassette chromosome recombinase genes A/B-2		potassium-trans-locating ATPase A, chain 2	potassium-transporting ATPase B, chain 1	potassium-trans-locating ATPase C, chain 2	sensor kinase protein	KDP operon transcrip-tional regulatory protein	methicillin-resistance regulatory protein	signal transducer protein MecR1	homolog of xylose repressor, associated with SCCmec-elements	cassette chromosome recombinase genes A/B-3		mercury resistance operon		cassette chromosome recombinase genes "ccrAA" (hypothetical) and ccrC			cassette chromosome recombinase genes A/B-4	
					rrnD1 (S. aureus)	gapA	katA	coA	nuc1	spa	sbi	sarA	saeS	vraS	agrI	agrII	agrIII	agrIV	hld	mecA	delta_mecR	ugpQ	ccrA-1	ccrB-1	plsSCC (COL)	Q9XB68-dcs	ccrA-2	ccrB-2	kdpA-SCC	kdpB-SCC	kdpC-SCC	kdpD-SCC	kdpE-SCC	mecI	mecR	xylR	ccrA-3	ccrB-3	merA	merB	ccrAA (MRSAZH47) _probe 1	ccrAA (MRSAZH47) _probe 2	ccrC (85-2082)	ccrA-4	ccrB-4
**2003**																																													
03F01030	CC5/ST228-MRSA, South German EMRSA (truncated SCCmec element)	MED-WARD	50	m	POS	POS	POS	POS	POS	POS	POS	POS	POS	POS	NEG	POS	NEG	NEG	POS	POS	POS	POS	NEG	NEG	POS	POS	NEG	NEG	NEG	NEG	NEG	NEG	NEG	NEG	NEG	NEG	NEG	NEG	POS	POS	NEG	NEG	NEG	NEG	NEG
03V05419	CC5/ST228-MRSA-I, South German EMRSA	SUR-ICU	77	m	POS	POS	POS	POS	POS	POS	POS	POS	POS	POS	NEG	POS	NEG	NEG	POS	POS	POS	POS	POS	POS	POS	POS	NEG	NEG	NEG	NEG	NEG	NEG	NEG	NEG	NEG	NEG	NEG	NEG	POS	POS	NEG	NEG	NEG	NEG	NEG
03V08986	CC5/ST228-MRSA-I, South German EMRSA	MED-OPD	62	m	POS	POS	POS	POS	POS	POS	POS	POS	POS	POS	NEG	POS	NEG	NEG	POS	POS	POS	POS	POS	POS	POS	POS	NEG	NEG	NEG	NEG	NEG	NEG	NEG	NEG	NEG	AMB	NEG	NEG	NEG	NEG	NEG	NEG	NEG	NEG	NEG
03V39123	CC5/ST228-MRSA-I, South German EMRSA	SUR-WARD	36	m	POS	POS	POS	POS		POS	POS	POS			NEG	POS	NEG	NEG	POS	POS																									
03B05186	CC5-MRSA-II, Rhine-Hesse EMRSA/UK-EMRSA-3	MED-ICU	82	m	POS	POS	POS	POS	POS	POS	POS	POS	POS	POS	NEG	POS	NEG	NEG	POS	POS	POS	POS	NEG	NEG	NEG	AMB	POS	POS	POS	POS	POS	POS	POS	POS	POS	POS	NEG	NEG	NEG	NEG	NEG	NEG	NEG	NEG	NEG
03U00003	CC5-MRSA-II, Rhine-Hesse EMRSA/UK-EMRSA-3	SUR-ICU	67	m	POS	POS	POS	POS	POS	POS	POS	POS	POS	POS	NEG	POS	NEG	NEG	POS	POS	POS	POS	NEG	NEG	NEG	POS	POS	POS	POS	POS	POS	POS	POS	POS	POS	POS	NEG	NEG	NEG	NEG	NEG	NEG	NEG	NEG	NEG
03V01438	CC5-MRSA-II, Rhine-Hesse EMRSA/UK-EMRSA-3	MED-WARD	75	f	POS	POS	POS	POS	POS	POS	POS	POS	POS	POS	NEG	POS	NEG	NEG	POS	POS	POS	POS	NEG	NEG	NEG	POS	POS	POS	POS	POS	POS	POS	POS	POS	POS	POS	NEG	NEG	NEG	NEG	NEG	NEG	NEG	NEG	NEG
03V05075	CC5-MRSA-II, Rhine-Hesse EMRSA/UK-EMRSA-3			f	POS	POS	POS	POS	POS	POS	POS	POS	POS	POS	NEG	POS	NEG	NEG	POS	POS	POS	POS	NEG	NEG	NEG	POS	POS	POS	POS	POS	POS	POS	POS	POS	POS	POS	NEG	NEG	NEG	NEG	NEG	NEG	NEG	NEG	NEG
03V11014	CC5-MRSA-II, Rhine-Hesse EMRSA/UK-EMRSA-3	MED-ICU	67	m	POS	POS	POS	POS	POS	POS	POS	POS	POS	POS	NEG	POS	NEG	NEG	POS	POS	POS	POS	NEG	NEG	NEG	POS	POS	POS	POS	POS	POS	POS	POS	POS	POS	POS	NEG	NEG	NEG	NEG	NEG	NEG	NEG	NEG	NEG
03V12917	CC5-MRSA-II, Rhine-Hesse EMRSA/UK-EMRSA-3	MED-ICU	52	m	POS	POS	POS	POS	POS	POS	POS	POS	POS	POS	NEG	POS	NEG	NEG	POS	POS	POS	POS	NEG	NEG	NEG	POS	POS	POS	POS	POS	POS	POS	POS	POS	POS	POS	NEG	NEG	NEG	NEG	NEG	NEG	NEG	NEG	NEG
03V14467	CC5-MRSA-II, Rhine-Hesse EMRSA/UK-EMRSA-3	MED-OPD	54	f	POS	POS	POS	POS	POS	POS	POS	POS	POS	POS	NEG	POS	NEG	NEG	POS	POS	POS	POS	NEG	NEG	NEG	POS	POS	POS	POS	POS	POS	POS	POS	POS	POS	POS	NEG	NEG	NEG	NEG	NEG	NEG	NEG	NEG	NEG
03V19357	CC5-MRSA-II, Rhine-Hesse EMRSA/UK-EMRSA-3	MED-ICU	71	m	POS	POS	POS	POS	POS	POS	POS	POS	POS	POS	NEG	POS	NEG	NEG	POS	POS	POS	POS	NEG	NEG	NEG	POS	POS	POS	POS	POS	POS	POS	POS	POS	POS	POS	NEG	NEG	NEG	NEG	NEG	NEG	NEG	NEG	NEG
03V20186	CC5-MRSA-II, Rhine-Hesse EMRSA/UK-EMRSA-3	SUR-ICU	50	m	POS	POS	POS	POS	POS	POS	POS	POS	POS	POS	NEG	POS	NEG	NEG	POS	POS	POS	POS	NEG	NEG	NEG	POS	POS	POS	POS	POS	POS	POS	POS	POS	POS	POS	NEG	NEG	NEG	NEG	NEG	NEG	NEG	NEG	NEG
03V25349	CC5-MRSA-II, Rhine-Hesse EMRSA/UK-EMRSA-3	MED-WARD	52	m	POS	POS	POS	POS	POS	POS	POS	POS	POS	POS	NEG	POS	NEG	NEG	POS	POS	POS	POS	NEG	NEG	NEG	POS	POS	POS	POS	POS	POS	POS	POS	POS	POS	POS	NEG	NEG	NEG	NEG	NEG	NEG	NEG	NEG	NEG
03V28948	CC5-MRSA-II, Rhine-Hesse EMRSA/UK-EMRSA-3	MED-WARD	60	f	POS	POS	POS	POS	POS	POS	POS	POS	POS	POS	NEG	POS	NEG	NEG	POS	POS	POS	POS	NEG	NEG	NEG	AMB	POS	POS	POS	AMB	POS	POS	POS	POS	POS	POS	NEG	NEG	NEG	NEG	NEG	NEG	NEG	NEG	NEG
03V30841	CC5-MRSA-II, Rhine-Hesse EMRSA/UK-EMRSA-3	MED-WARD	28	f	POS	POS	POS	POS		POS	POS	POS			NEG	POS	NEG	NEG	POS	POS																									
03V32896	CC5-MRSA-II, Rhine-Hesse EMRSA/UK-EMRSA-3	MED-OPD	59	m	POS	POS	POS	POS	POS	POS	POS	POS	POS	POS	NEG	POS	NEG	NEG	POS	POS	POS	POS	NEG	NEG	NEG	POS	POS	POS	POS	POS	POS	POS	POS	POS	POS	POS	NEG	NEG	NEG	NEG	NEG	NEG	NEG	NEG	NEG
03V33415	CC5-MRSA-II, Rhine-Hesse EMRSA/UK-EMRSA-3	MED-WARD	59	m	POS	POS	POS	POS	POS	POS	POS	POS	POS	POS	NEG	POS	NEG	NEG	POS	POS	POS	POS	NEG	NEG	NEG	POS	POS	POS	POS	POS	POS	POS	POS	POS	POS	POS	NEG	NEG	NEG	NEG	NEG	NEG	NEG	NEG	NEG
03V34628	CC5-MRSA-II, Rhine-Hesse EMRSA/UK-EMRSA-3	SUR-WARD	17	m	POS	POS	POS	POS	POS	POS	POS	POS	POS	POS	NEG	POS	NEG	NEG	POS	POS	POS	POS	NEG	NEG	NEG	POS	POS	POS	POS	POS	POS	POS	POS	POS	POS	POS	NEG	NEG	NEG	NEG	NEG	NEG	NEG	NEG	NEG
03V37078	CC5-MRSA-II, Rhine-Hesse EMRSA/UK-EMRSA-3	MED-ICU	41	m	POS	POS	POS	POS	POS	POS	POS	POS	POS	POS	NEG	POS	NEG	NEG	POS	POS	POS	POS	NEG	NEG	NEG	AMB	POS	POS	POS	POS	POS	POS	POS	POS	POS	POS	NEG	NEG	NEG	NEG	NEG	NEG	NEG	NEG	NEG
03V41645	CC5-MRSA-II, Rhine-Hesse EMRSA/UK-EMRSA-3	MED-WARD	48	m	POS	POS	POS	POS	POS	POS	POS	POS	POS	POS	NEG	POS	NEG	NEG	POS	POS	POS	POS	NEG	NEG	NEG	POS	POS	POS	POS	POS	POS	POS	POS	POS	POS	POS	NEG	NEG	NEG	NEG	NEG	NEG	NEG	NEG	NEG
03V03012	CC5-MRSA-IV, Paediatric clone	MED-WARD	42	f	POS	POS	POS	POS	POS	POS	POS	POS	POS	POS	NEG	POS	NEG	NEG	POS	POS	POS	POS	NEG	NEG	NEG	POS	POS	POS	NEG	NEG	NEG	NEG	NEG	NEG	NEG	POS	NEG	NEG	NEG	NEG	NEG	NEG	NEG	NEG	NEG
03V02863	CC8/ST247-MRSA-I, North German/Iberian EMRSA	SUR-WARD	78	f	POS	POS	POS	POS	POS	POS	POS	POS	POS	POS	POS	NEG	NEG	NEG	POS	POS	POS	POS	POS	POS	POS	POS	NEG	NEG	NEG	NEG	NEG	NEG	NEG	NEG	NEG	NEG	NEG	NEG	NEG	NEG	NEG	NEG	NEG	NEG	NEG
03V14882	CC8/ST254-MRSA-(atypical SCCmec), Hannover EMRSA	MED-WARD	26	f	POS	POS	POS	POS	POS	POS	POS	POS	POS	POS	POS	NEG	NEG	NEG	POS	POS	POS	POS	NEG	NEG	NEG	POS	NEG	NEG	NEG	NEG	NEG	NEG	NEG	NEG	NEG	NEG	NEG	NEG	POS	POS	NEG	NEG	NEG	NEG	NEG
03B00404	CC22-MRSA-IV, Barnim/UK-EMRSA-15	MED-OPD	99	f	POS	POS	POS	POS	POS	POS	POS	POS	POS	POS	POS	NEG	NEG	NEG	POS	POS	POS	POS	NEG	NEG	NEG	POS	POS	POS	NEG	NEG	NEG	NEG	NEG	NEG	NEG	NEG	NEG	NEG	NEG	NEG	NEG	NEG	NEG	NEG	NEG
03U00892	CC22-MRSA-IV, Barnim/UK-EMRSA-15	MED-WARD	1	m	POS	POS	POS	POS		POS	POS	POS			POS	NEG	NEG	NEG	POS	POS																									
03V01422	CC22-MRSA-IV, Barnim/UK-EMRSA-15	MED-OPD	71	f	POS	POS	POS	POS	POS	POS	POS	POS	POS	POS	POS	NEG	NEG	NEG	POS	POS	POS	POS	NEG	NEG	NEG	POS	POS	POS	NEG	NEG	NEG	NEG	NEG	NEG	NEG	NEG	NEG	NEG	NEG	NEG	NEG	NEG	NEG	NEG	NEG
03V04424	CC22-MRSA-IV, Barnim/UK-EMRSA-15	MED-OPD	74	m	POS	POS	POS	POS	POS	POS	POS	POS	POS	POS	POS	NEG	NEG	AMB	POS	POS	POS	POS	NEG	NEG	NEG	POS	POS	POS	NEG	NEG	NEG	NEG	NEG	NEG	NEG	NEG	NEG	NEG	NEG	NEG	NEG	NEG	NEG	NEG	NEG
03V05690	CC22-MRSA-IV, Barnim/UK-EMRSA-15	MED-WARD	77	m	POS	POS	POS	POS	POS	POS	POS	POS	POS	POS	POS	NEG	NEG	NEG	POS	POS	POS	POS	NEG	NEG	NEG	POS	POS	POS	NEG	NEG	NEG	NEG	NEG	NEG	NEG	NEG	NEG	NEG	NEG	NEG	NEG	NEG	NEG	NEG	NEG
03V07912	CC22-MRSA-IV, Barnim/UK-EMRSA-15	MED-OPD	12	m	POS	POS	POS	POS	POS	POS	POS	POS	POS	POS	POS	NEG	NEG	NEG	POS	POS	POS	POS	NEG	AMB	NEG	POS	POS	POS	NEG	NEG	NEG	NEG	NEG	NEG	AMB	AMB	NEG	NEG	NEG	NEG	NEG	NEG	NEG	NEG	NEG
03V23798	CC22-MRSA-IV, Barnim/UK-EMRSA-15	SUR-WARD	82	f	POS	POS	POS	POS	POS	POS	POS	POS	POS	POS	POS	NEG	NEG	NEG	POS	POS	POS	POS	NEG	NEG	NEG	POS	POS	POS	NEG	NEG	NEG	NEG	NEG	NEG	NEG	NEG	NEG	NEG	NEG	NEG	NEG	NEG	NEG	NEG	NEG
03V25914	CC22-MRSA-IV, Barnim/UK-EMRSA-15	MED-WARD	75	m	POS	POS	POS	POS	POS	POS	POS	POS	POS	POS	POS	NEG	NEG	NEG	POS	POS	POS	POS	NEG	NEG	NEG	POS	POS	POS	NEG	NEG	NEG	NEG	NEG	NEG	NEG	NEG	NEG	NEG	NEG	NEG	NEG	NEG	NEG	NEG	NEG
03V29751	CC22-MRSA-IV, Barnim/UK-EMRSA-15	MED-WARD	60	m	POS	POS	POS	POS	POS	POS	POS	POS	POS	POS	POS	NEG	NEG	NEG	POS	POS	POS	POS	NEG	NEG	NEG	AMB	POS	POS	NEG	NEG	NEG	NEG	NEG	NEG	NEG	NEG	NEG	NEG	NEG	NEG	NEG	NEG	NEG	NEG	NEG
03V30201	CC22-MRSA-IV, Barnim/UK-EMRSA-15	MED-WARD	68	f	POS	POS	POS	POS	POS	POS	POS	POS	POS	POS	POS	NEG	NEG	AMB	POS	POS	POS	POS	NEG	NEG	NEG	POS	POS	POS	NEG	NEG	NEG	NEG	NEG	NEG	NEG	NEG	NEG	NEG	NEG	NEG	NEG	NEG	NEG	NEG	NEG
03B01781	CC45-MRSA-IV, Berlin EMRSA	MED-OPD	37	m	POS	POS	POS	POS	POS	POS	POS	POS	POS	POS	POS	NEG	NEG	NEG	POS	POS	POS	POS	NEG	NEG	NEG	POS	POS	POS	NEG	NEG	NEG	NEG	NEG	NEG	NEG	NEG	NEG	NEG	NEG	NEG	NEG	NEG	NEG	NEG	NEG
03U05014	CC45-MRSA-IV, Berlin EMRSA	MED-WARD	88	m	POS	POS	POS	POS	POS	POS	POS	POS	POS	POS	POS	NEG	NEG	NEG	POS	POS	POS	POS	NEG	NEG	NEG	POS	POS	POS	NEG	NEG	NEG	NEG	NEG	NEG	NEG	NEG	NEG	NEG	NEG	NEG	NEG	NEG	NEG	NEG	NEG
03U11106	CC45-MRSA-IV, Berlin EMRSA	MED-WARD	70	f	POS	POS	POS	POS	POS	POS	POS	POS	POS	POS	POS	NEG	NEG	NEG	POS	POS	POS	POS	NEG	NEG	NEG	POS	POS	POS	NEG	NEG	NEG	NEG	NEG	NEG	NEG	NEG	NEG	NEG	NEG	NEG	NEG	NEG	NEG	NEG	NEG
03V00742	CC45-MRSA-IV, Berlin EMRSA	MED-OPD	54	f	POS	POS	POS	POS	POS	POS	POS	POS	POS	POS	POS	NEG	NEG	NEG	POS	POS	POS	POS	NEG	NEG	NEG	POS	POS	POS	NEG	NEG	NEG	NEG	NEG	NEG	NEG	NEG	NEG	NEG	NEG	NEG	NEG	NEG	NEG	NEG	NEG
03V01902	CC45-MRSA-IV, Berlin EMRSA	MED-OPD	83	m	POS	POS	POS	POS	POS	POS	POS	POS	POS	POS	POS	NEG	NEG	NEG	POS	POS	POS	POS	NEG	NEG	NEG	POS	POS	POS	NEG	NEG	NEG	NEG	NEG	NEG	NEG	NEG	NEG	NEG	NEG	NEG	NEG	NEG	NEG	NEG	NEG
03V02256	CC45-MRSA-IV, Berlin EMRSA	MED-OPD	53	m	POS	POS	POS	POS	POS	POS	POS	POS	POS	POS	POS	NEG	NEG	NEG	POS	POS	POS	POS	NEG	NEG	NEG	POS	POS	POS	NEG	NEG	NEG	NEG	NEG	NEG	NEG	NEG	NEG	NEG	NEG	NEG	NEG	NEG	NEG	NEG	NEG
03V02262	CC45-MRSA-IV, Berlin EMRSA	MED-OPD	78	f	POS	POS	POS	POS	POS	POS	POS	POS	POS	POS	POS	NEG	NEG	NEG	POS	POS	POS	POS	NEG	NEG	NEG	POS	POS	POS	NEG	NEG	NEG	NEG	NEG	NEG	NEG	NEG	NEG	NEG	NEG	NEG	NEG	NEG	NEG	NEG	NEG
03V04701	CC45-MRSA-IV, Berlin EMRSA	SUR-WARD	25	f	POS	POS	POS	POS	POS	AMB	POS	POS	POS	POS	AMB	NEG	NEG	NEG	POS	POS	POS	POS	NEG	NEG	NEG	POS	POS	POS	NEG	NEG	NEG	NEG	NEG	NEG	NEG	NEG	NEG	NEG	NEG	NEG	NEG	NEG	NEG	NEG	NEG
03V05042	CC45-MRSA-IV, Berlin EMRSA	MED-WARD	69	m	POS	POS	POS	POS	POS	POS	POS	POS	POS	POS	POS	NEG	NEG	NEG	POS	POS	POS	POS	NEG	NEG	NEG	POS	POS	POS	NEG	NEG	NEG	NEG	NEG	NEG	NEG	NEG	NEG	NEG	NEG	NEG	NEG	NEG	NEG	NEG	NEG
03V05377	CC45-MRSA-IV, Berlin EMRSA	MED-OPD	66	m	POS	POS	POS	POS	POS	POS	POS	POS	POS	POS	POS	NEG	NEG	NEG	POS	POS	POS	POS	NEG	NEG	NEG	POS	POS	POS	NEG	NEG	NEG	NEG	NEG	NEG	NEG	NEG	NEG	NEG	NEG	NEG	NEG	NEG	NEG	NEG	NEG
03V05507	CC45-MRSA-IV, Berlin EMRSA	MED-WARD	80	m	POS	POS	POS	POS	POS	POS	POS	POS	POS	POS	AMB	NEG	NEG	NEG	POS	POS	POS	POS	NEG	NEG	NEG	POS	POS	POS	NEG	NEG	NEG	NEG	NEG	NEG	NEG	NEG	NEG	NEG	NEG	NEG	NEG	NEG	NEG	NEG	NEG
03V08214	CC45-MRSA-IV, Berlin EMRSA	MED-ICU	23	m	POS	POS	POS	POS	POS	POS	POS	POS	POS	POS	POS	NEG	NEG	NEG	POS	POS	POS	POS	NEG	NEG	NEG	POS	POS	POS	NEG	NEG	NEG	NEG	NEG	NEG	NEG	NEG	NEG	NEG	NEG	NEG	NEG	NEG	NEG	NEG	NEG
03V08525	CC45-MRSA-IV, Berlin EMRSA	MED-WARD	63	m	POS	POS	POS	POS	POS	AMB	POS	POS	POS	POS	POS	NEG	NEG	NEG	POS	POS	POS	POS	NEG	NEG	NEG	POS	POS	POS	NEG	NEG	NEG	NEG	NEG	NEG	NEG	NEG	NEG	NEG	NEG	NEG	NEG	NEG	NEG	NEG	NEG
03V11910	CC45-MRSA-IV, Berlin EMRSA	MED-WARD	51	f	POS	POS	POS	POS	POS	NEG	POS	POS	POS	POS	AMB	NEG	NEG	NEG	POS	POS	POS	POS	NEG	NEG	NEG	AMB	POS	POS	NEG	NEG	NEG	NEG	NEG	NEG	NEG	NEG	NEG	NEG	NEG	NEG	NEG	NEG	NEG	NEG	NEG
03V14481	CC45-MRSA-IV, Berlin EMRSA	SUR-WARD	56	m	POS	POS	POS	POS	POS	POS	POS	POS	POS	POS	POS	NEG	NEG	NEG	POS	POS	POS	POS	NEG	NEG	NEG	POS	POS	POS	NEG	NEG	NEG	NEG	NEG	NEG	NEG	NEG	NEG	NEG	NEG	NEG	NEG	NEG	NEG	NEG	NEG
03V15082	CC45-MRSA-IV, Berlin EMRSA	SUR-ICU	75	m	POS	POS	POS	POS	POS	POS	POS	POS	POS	POS	POS	NEG	NEG	NEG	POS	POS	POS	POS	NEG	NEG	NEG	POS	POS	POS	NEG	NEG	NEG	NEG	NEG	NEG	NEG	NEG	NEG	NEG	NEG	NEG	NEG	NEG	NEG	NEG	NEG
03V17508	CC45-MRSA-IV, Berlin EMRSA	SUR-WARD	87	f	POS	POS	POS	POS	POS	AMB	POS	POS	POS	POS	AMB	NEG	NEG	NEG	POS	POS	POS	POS	NEG	NEG	NEG	POS	POS	POS	NEG	NEG	NEG	NEG	NEG	NEG	NEG	NEG	NEG	NEG	NEG	NEG	NEG	NEG	NEG	NEG	NEG
03V20150	CC45-MRSA-IV, Berlin EMRSA	SUR-WARD	84	m	POS	POS	POS	POS	POS	NEG	POS	POS	POS	POS	AMB	NEG	NEG	NEG	POS	POS	POS	POS	NEG	NEG	NEG	POS	POS	POS	NEG	NEG	NEG	NEG	NEG	NEG	NEG	NEG	NEG	NEG	NEG	NEG	NEG	NEG	NEG	NEG	NEG
03V22437	CC45-MRSA-IV, Berlin EMRSA	SUR-WARD	49	m	POS	POS	POS	POS	POS	NEG	POS	POS	POS	POS	AMB	NEG	NEG	NEG	POS	POS	POS	POS	NEG	NEG	NEG	POS	POS	POS	NEG	NEG	NEG	NEG	NEG	NEG	NEG	NEG	NEG	NEG	NEG	NEG	NEG	NEG	NEG	NEG	NEG
03V24471	CC45-MRSA-IV, Berlin EMRSA	SUR-WARD	76	m	POS	POS	POS	POS	POS	POS	POS	POS	POS	POS	POS	NEG	NEG	NEG	POS	POS	POS	POS	NEG	NEG	NEG	POS	POS	POS	NEG	NEG	NEG	NEG	NEG	NEG	NEG	NEG	NEG	NEG	NEG	NEG	NEG	NEG	NEG	NEG	NEG
03V28070	CC45-MRSA-IV, Berlin EMRSA	MED-OPD	57	m	POS	POS	POS	POS	POS	NEG	AMB	POS	POS	POS	AMB	NEG	NEG	NEG	POS	POS	POS	POS	NEG	NEG	NEG	AMB	POS	POS	NEG	NEG	NEG	NEG	NEG	NEG	NEG	NEG	NEG	NEG	NEG	NEG	NEG	NEG	NEG	NEG	NEG
03V28448	CC45-MRSA-IV, Berlin EMRSA	MED-OPD	63	f	POS	POS	POS	POS	POS	POS	POS	POS	POS	POS	POS	NEG	NEG	NEG	POS	POS	POS	POS	NEG	NEG	NEG	POS	POS	POS	NEG	NEG	NEG	NEG	NEG	NEG	NEG	NEG	NEG	NEG	NEG	NEG	NEG	NEG	NEG	NEG	NEG
03V34589	CC45-MRSA-IV, Berlin EMRSA	MED-OPD	65	m	POS	POS	POS	POS	POS	POS	POS	POS	POS	POS	POS	NEG	NEG	NEG	POS	POS	POS	POS	NEG	NEG	NEG	POS	POS	POS	NEG	NEG	NEG	NEG	NEG	NEG	NEG	NEG	NEG	NEG	NEG	NEG	NEG	NEG	NEG	NEG	NEG
03V39774	CC45-MRSA-IV, Berlin EMRSA	MED-OPD	56	m	POS	POS	POS	POS	POS	POS	POS	POS	POS	POS	POS	NEG	NEG	NEG	POS	POS	POS	POS	NEG	NEG	NEG	POS	POS	POS	NEG	NEG	NEG	NEG	NEG	NEG	NEG	NEG	NEG	NEG	NEG	NEG	NEG	NEG	NEG	NEG	NEG
**2004**																																													
04B05423	CC5-MRSA-II, Rhine-Hesse EMRSA/UK-EMRSA-3	SUR-ICU	73	m	POS	POS	POS	POS	POS	POS	POS	POS	POS	POS	NEG	POS	NEG	NEG	POS	POS	POS	POS	NEG	NEG	NEG	POS	POS	POS	POS	POS	POS	POS	POS	POS	POS	POS	NEG	NEG	NEG	NEG	NEG	NEG	NEG	NEG	NEG
04U02171	CC5-MRSA-II, Rhine-Hesse EMRSA/UK-EMRSA-3	MED-OPD	60	f	POS	POS	POS	POS	POS	POS	POS	POS	POS	POS	NEG	POS	NEG	NEG	POS	POS	POS	POS	NEG	NEG	NEG	POS	POS	POS	POS	POS	POS	POS	POS	POS	POS	POS	NEG	NEG	NEG	NEG	NEG	NEG	NEG	NEG	NEG
04U03682	CC5-MRSA-II, Rhine-Hesse EMRSA/UK-EMRSA-3	MED-WARD	19	m	POS	POS	POS	POS	POS	POS	POS	POS	POS	POS	NEG	POS	NEG	NEG	POS	POS	POS	POS	NEG	NEG	NEG	AMB	POS	POS	POS	AMB	POS	POS	POS	POS	POS	POS	NEG	NEG	NEG	NEG	NEG	NEG	NEG	NEG	NEG
04U06618	CC5-MRSA-II, Rhine-Hesse EMRSA/UK-EMRSA-3	MED-WARD	49	f	POS	POS	POS	POS	POS	POS	POS	POS	POS	POS	NEG	POS	NEG	NEG	POS	POS	POS	POS	NEG	NEG	NEG	POS	POS	POS	POS	POS	POS	POS	POS	POS	POS	POS	NEG	NEG	NEG	NEG	NEG	NEG	NEG	NEG	NEG
04U12578	CC5-MRSA-II, Rhine-Hesse EMRSA/UK-EMRSA-3	MED-WARD	82	m	POS	POS	POS	POS	POS	POS	POS	POS	POS	POS	NEG	POS	NEG	NEG	POS	POS	POS	POS	NEG	NEG	NEG	POS	POS	POS	POS	POS	POS	POS	POS	POS	POS	POS	NEG	NEG	NEG	NEG	NEG	NEG	NEG	NEG	NEG
04V02465	CC5-MRSA-II, Rhine-Hesse EMRSA/UK-EMRSA-3	MED-WARD	51	m	POS	POS	POS	POS	POS	POS	POS	POS	POS	POS	NEG	POS	NEG	NEG	POS	POS	POS	POS	NEG	NEG	NEG	POS	POS	POS	POS	POS	POS	POS	POS	POS	POS	POS	NEG	NEG	NEG	NEG	NEG	NEG	NEG	NEG	NEG
04V03642	CC5-MRSA-II, Rhine-Hesse EMRSA/UK-EMRSA-3	SUR-WARD	53	m	POS	POS	POS	POS	POS	POS	POS	POS	POS	POS	NEG	POS	NEG	NEG	POS	POS	POS	POS	NEG	NEG	NEG	AMB	POS	POS	POS	AMB	POS	POS	POS	POS	POS	POS	NEG	NEG	NEG	NEG	NEG	NEG	NEG	NEG	NEG
04V07657	CC5-MRSA-II, Rhine-Hesse EMRSA/UK-EMRSA-3	MED-WARD	0	m	POS	POS	POS	POS	POS	POS	POS	POS	POS	POS	NEG	POS	NEG	NEG	POS	POS	POS	POS	NEG	NEG	NEG	AMB	POS	POS	POS	AMB	POS	POS	POS	POS	POS	POS	NEG	NEG	NEG	NEG	NEG	NEG	NEG	NEG	NEG
04V12223	CC5-MRSA-II, Rhine-Hesse EMRSA/UK-EMRSA-3	SUR-WARD	68	m	POS	POS	POS	POS	POS	POS	POS	POS	POS	POS	NEG	POS	NEG	NEG	POS	POS	POS	POS	NEG	NEG	NEG	AMB	POS	POS	POS	AMB	POS	POS	POS	POS	POS	POS	NEG	NEG	NEG	NEG	NEG	NEG	NEG	NEG	NEG
04V12529	CC5-MRSA-II, Rhine-Hesse EMRSA/UK-EMRSA-3	MED-WARD	80	f	POS	POS	POS	POS	POS	POS	POS	POS	POS	POS	NEG	POS	NEG	NEG	POS	POS	POS	POS	NEG	NEG	NEG	POS	POS	POS	POS	POS	POS	POS	POS	POS	POS	POS	NEG	NEG	NEG	NEG	NEG	NEG	NEG	NEG	NEG
04V13633	CC5-MRSA-II, Rhine-Hesse EMRSA/UK-EMRSA-3	MED-WARD	22	f	POS	POS	POS	POS	POS	POS	POS	POS	POS	POS	NEG	POS	NEG	NEG	POS	POS	POS	POS	NEG	NEG	NEG	POS	POS	POS	POS	POS	POS	POS	POS	POS	POS	POS	NEG	NEG	NEG	NEG	NEG	NEG	NEG	NEG	NEG
04V16499	CC5-MRSA-II, Rhine-Hesse EMRSA/UK-EMRSA-3	MED-OPD	77	f	POS	POS	POS	POS	POS	POS	POS	POS	POS	POS	NEG	POS	NEG	NEG	POS	POS	POS	POS	NEG	NEG	NEG	POS	POS	POS	POS	POS	POS	POS	POS	POS	POS	POS	NEG	NEG	NEG	NEG	NEG	NEG	NEG	NEG	NEG
04V17015	CC5-MRSA-II, Rhine-Hesse EMRSA/UK-EMRSA-3	MED-ICU	65	m	POS	POS	POS	POS	POS	POS	POS	POS	POS	POS	NEG	POS	NEG	NEG	POS	POS	POS	POS	NEG	NEG	NEG	POS	POS	POS	POS	POS	POS	POS	POS	POS	POS	POS	NEG	NEG	NEG	NEG	NEG	NEG	NEG	NEG	NEG
04V20684	CC5-MRSA-II, Rhine-Hesse EMRSA/UK-EMRSA-3	SUR-WARD	62	m	POS	POS	POS	POS		POS	POS	POS			NEG	POS	NEG	NEG	POS	POS																									
04V24615	CC5-MRSA-II, Rhine-Hesse EMRSA/UK-EMRSA-3	SUR-WARD	77	m	POS	POS	POS	POS	POS	POS	POS	POS	POS	POS	NEG	POS	NEG	NEG	POS	POS	POS	POS	NEG	NEG	NEG	POS	POS	POS	POS	POS	POS	POS	POS	POS	POS	POS	NEG	NEG	NEG	NEG	NEG	NEG	NEG	NEG	NEG
04V24901	CC5-MRSA-II, Rhine-Hesse EMRSA/UK-EMRSA-3	SUR-WARD	84	m	POS	POS	POS	POS	POS	POS	POS	POS	POS	POS	NEG	POS	NEG	NEG	POS	POS	POS	POS	NEG	NEG	NEG	POS	POS	POS	POS	POS	POS	POS	POS	POS	POS	POS	NEG	NEG	NEG	NEG	NEG	NEG	NEG	NEG	NEG
04V25081	CC5-MRSA-II, Rhine-Hesse EMRSA/UK-EMRSA-3	MED-WARD	74	m	POS	POS	POS	POS	POS	POS	POS	POS	POS	POS	NEG	POS	NEG	NEG	POS	POS	POS	POS	NEG	NEG	NEG	POS	POS	POS	POS	POS	POS	POS	POS	POS	POS	POS	NEG	NEG	NEG	NEG	NEG	NEG	NEG	NEG	NEG
04V27293	CC5-MRSA-II, Rhine-Hesse EMRSA/UK-EMRSA-3	SUR-WARD	58	m	POS	POS	POS	POS	POS	POS	POS	POS	POS	POS	NEG	POS	NEG	NEG	POS	POS	POS	POS	NEG	NEG	NEG	POS	POS	POS	POS	POS	POS	POS	POS	POS	POS	POS	NEG	NEG	NEG	NEG	NEG	NEG	NEG	NEG	NEG
04V27311	CC5-MRSA-II, Rhine-Hesse EMRSA/UK-EMRSA-3	MED-OPD	64	m	POS	POS	POS	POS	POS	POS	POS	POS	POS	POS	NEG	POS	NEG	NEG	POS	POS	POS	POS	NEG	NEG	NEG	POS	POS	POS	POS	AMB	POS	POS	POS	POS	POS	POS	NEG	NEG	NEG	NEG	NEG	NEG	NEG	NEG	NEG
04V33459	CC5-MRSA-II, Rhine-Hesse EMRSA/UK-EMRSA-3	MED-OPD	64	m	POS	POS	POS	POS	POS	POS	POS	POS	POS	POS	NEG	POS	NEG	NEG	POS	POS	POS	POS	NEG	NEG	NEG	POS	POS	POS	POS	POS	POS	POS	POS	POS	POS	POS	NEG	NEG	NEG	NEG	NEG	NEG	NEG	NEG	NEG
04V39909	CC5-MRSA-IV&VI	MED-ICU	92	m	POS	POS	POS	POS	POS	POS	POS	POS	POS	POS	NEG	POS	NEG	NEG	POS	POS	POS	POS	NEG	NEG	NEG	AMB	POS	POS	NEG	NEG	NEG	NEG	NEG	NEG	NEG	NEG	NEG		NEG	NEG	NEG	NEG	NEG	AMB	POS
04U02421	CC5-MRSA-IV, Paediatric clone	SUR-ICU	2	m	POS	POS	POS	POS	POS	POS	POS	POS	POS	POS	NEG	POS	NEG	NEG	POS	POS	POS	POS	NEG	NEG	NEG	POS	POS	POS	NEG	NEG	NEG	NEG	NEG	NEG	NEG	NEG	NEG	NEG	POS	POS	NEG	NEG	NEG	NEG	NEG
04V09807	CC80-MRSA-IV [PVL+], European caMRSA Clone	SUR-WARD	28	f	POS	POS	POS	POS		POS	POS	POS			NEG	NEG	POS	NEG	POS	POS																									
04V16073	CC80-MRSA-IV [PVL+], European caMRSA Clone	SUR-OPD	34	m	POS	POS	POS	POS	POS	POS	POS	POS	POS	POS	NEG	NEG	POS	NEG	POS	POS	POS	POS	NEG	NEG	NEG	POS	POS	POS	NEG	NEG	NEG	NEG	NEG	NEG	NEG	NEG	NEG	NEG	NEG	NEG	NEG	NEG	NEG	NEG	NEG
04V23548	CC80-MRSA-IV [PVL+], European caMRSA Clone	MED-ICU	54	m	POS	POS	POS	POS		POS	POS	POS			NEG	NEG	POS	NEG	POS	POS																									
04V03289	CC22-MRSA-IV, Barnim/UK-EMRSA-15	SUR-ICU	60	m	POS	POS	POS	POS		POS	POS				POS	NEG	NEG	NEG	POS	POS																									
04V03847	CC22-MRSA-IV, Barnim/UK-EMRSA-15	MED-icu	0	m	POS	POS	POS	POS		POS	POS				POS	NEG	NEG	NEG	POS	POS																									
04V04595	CC22-MRSA-IV, Barnim/UK-EMRSA-15	MED-OPD	70	m	POS	POS	POS	POS	POS	POS	POS	POS	POS	POS	POS	NEG	NEG	AMB	POS	POS	POS	POS	NEG	NEG	NEG	POS	POS	POS	NEG	NEG	NEG	NEG	NEG	NEG	NEG	NEG	NEG	NEG	NEG	NEG	NEG	NEG	NEG	NEG	NEG
04V05029	CC22-MRSA-IV, Barnim/UK-EMRSA-15	MED-OPD	36	m	POS	POS	POS	AMB	POS	POS	POS	POS	POS	POS	POS	NEG	NEG	NEG	POS	POS	POS	POS	NEG	NEG	NEG	NEG	POS	POS	NEG	NEG	NEG	NEG	NEG	NEG	NEG	NEG	NEG	NEG	NEG	NEG	NEG	NEG	NEG	NEG	NEG
04V07875	CC22-MRSA-IV, Barnim/UK-EMRSA-15	MED-WARD	77	f	POS	POS	AMB	NEG	AMB	AMB	AMB	POS	AMB	POS	NEG	NEG	NEG	NEG	AMB	POS	POS	POS	NEG	NEG	NEG	NEG	POS	POS	NEG	NEG	NEG	NEG	NEG	NEG	NEG	NEG	NEG	NEG	NEG	NEG	NEG	NEG	NEG	NEG	NEG
04V14909	CC22-MRSA-IV, Barnim/UK-EMRSA-15	MED-WARD	27	f	POS	POS	POS	POS	POS	POS	POS	POS	POS	POS	POS	NEG	NEG	NEG	POS	POS	POS	POS	NEG	NEG	NEG	POS	POS	POS	NEG	NEG	NEG	NEG	NEG	NEG	NEG	NEG	NEG	NEG	NEG	NEG	NEG	NEG	NEG	NEG	NEG
04V18654	CC22-MRSA-IV, Barnim/UK-EMRSA-15	MED-OPD	75	m	POS	POS	POS	POS	POS	POS	POS	POS	POS	POS	POS	NEG	NEG	NEG	POS	POS	POS	POS	NEG	NEG	NEG	POS	POS	POS	NEG	NEG	NEG	NEG	NEG	NEG	NEG	NEG	NEG	NEG	NEG	NEG	NEG	NEG	NEG	NEG	NEG
04V18815	CC22-MRSA-IV, Barnim/UK-EMRSA-15	SUR-WARD	75	m	POS	POS	POS	POS	POS	POS	POS	POS	POS	POS	POS	NEG	NEG	AMB	POS	POS	POS	POS	NEG	NEG	NEG	POS	POS	POS	NEG	NEG	NEG	NEG	NEG	NEG	NEG	NEG	NEG	NEG	NEG	NEG	NEG	NEG	NEG	NEG	NEG
04V29721	CC22-MRSA-IV, Barnim/UK-EMRSA-15	MED-WARD	73	m	POS	POS	POS	POS	POS	POS	POS	POS	POS	POS	POS	NEG	NEG	NEG	POS	POS	POS	POS	NEG	NEG	NEG	AMB	POS	POS	NEG	NEG	NEG	NEG	NEG	NEG	NEG	NEG	NEG	NEG	NEG	NEG	NEG	NEG	NEG	NEG	NEG
04V32759	CC22-MRSA-IV, Barnim/UK-EMRSA-15	MED-OPD	65	m	POS	POS	POS	POS	POS	POS	POS	POS	POS	POS	POS	NEG	NEG	NEG	POS	POS	POS	POS	NEG	NEG	NEG	POS	POS	POS	NEG	NEG	NEG	NEG	NEG	NEG	NEG	NEG	NEG	NEG	NEG	NEG	NEG	NEG	NEG	NEG	NEG
04V32919	CC22-MRSA-IV, Barnim/UK-EMRSA-15	MED-WARD	67	m	POS	POS	POS	POS	POS	POS	POS	POS	POS	POS	POS	NEG	NEG	POS	POS	POS	POS	POS	NEG	NEG	NEG	POS	POS	POS	NEG	NEG	NEG	NEG	NEG	NEG	NEG	NEG	NEG	NEG	NEG	NEG	NEG	NEG	NEG	NEG	NEG
04V33780	CC22-MRSA-IV, Barnim/UK-EMRSA-15	MED-OPD	52	m	POS	POS	POS	POS	POS	POS	POS	POS	POS	POS	POS	NEG	NEG	NEG	POS	POS	POS	POS	NEG	NEG	NEG	POS	POS	POS	NEG	NEG	NEG	NEG	NEG	NEG	NEG	NEG	NEG	NEG	NEG	NEG	NEG	NEG	NEG	NEG	NEG
04V35396	CC22-MRSA-IV, Barnim/UK-EMRSA-15	MED-WARD	30	f	POS	POS	POS	POS	POS	POS	POS	POS	POS	POS	POS	NEG	NEG	AMB	POS	POS	POS	POS	NEG	NEG	NEG	POS	POS	POS	NEG	NEG	NEG	NEG	NEG	NEG	NEG	NEG	NEG	NEG	NEG	NEG	NEG	NEG	NEG	NEG	NEG
04V37107	CC22-MRSA-IV, Barnim/UK-EMRSA-15	MED-OPD	63	f	POS	POS	POS	POS	POS	POS	POS	POS	POS	POS	POS	NEG	NEG	NEG	POS	POS	POS	POS	NEG	NEG	NEG	POS	POS	POS	NEG	NEG	NEG	NEG	NEG	NEG	NEG	NEG	NEG	NEG	NEG	NEG	NEG	NEG	NEG	NEG	NEG
04V37518	CC22-MRSA-IV, Barnim/UK-EMRSA-15	MED-OPD	55	m	POS	POS	POS	POS	POS	POS	POS	POS	POS	POS	POS	NEG	NEG	NEG	POS	POS	POS	POS	NEG	NEG	NEG	POS	POS	POS	NEG	NEG	NEG	NEG	NEG	NEG	NEG	NEG	NEG	NEG	NEG	NEG	NEG	NEG	NEG	NEG	NEG
04V38199	CC22-MRSA-IV, Barnim/UK-EMRSA-15	MED-WARD	28	m	POS	POS	POS	POS	POS	POS	POS	POS	POS	POS	POS	NEG	NEG	NEG	POS	POS	POS	POS	NEG	NEG	NEG	NEG	POS	POS	NEG	NEG	NEG	NEG	NEG	NEG	NEG	NEG	NEG	NEG	NEG	NEG	NEG	NEG	NEG	NEG	NEG
04V38358	CC22-MRSA-IV, Barnim/UK-EMRSA-15	MED-OPD	63	m	POS	POS	POS	POS	POS	POS	POS	POS	POS	POS	POS	NEG	NEG	NEG	POS	POS	POS	POS	NEG	NEG	NEG	AMB	POS	POS	NEG	NEG	NEG	NEG	NEG	NEG	NEG	NEG	NEG	NEG	NEG	NEG	NEG	NEG	NEG	NEG	NEG
04V38902	CC22-MRSA-IV, Barnim/UK-EMRSA-15	MED-WARD	63	m	POS	POS	POS	POS	POS	POS	POS	POS	POS	POS	POS	NEG	NEG	NEG	POS	POS	POS	POS	NEG	NEG	NEG	AMB	POS	POS	NEG	NEG	NEG	NEG	NEG	NEG	NEG	NEG	NEG	NEG	NEG	NEG	NEG	NEG	NEG	NEG	NEG
04V39203	CC22-MRSA-IV, Barnim/UK-EMRSA-15	MED-OPD	60	m	POS	POS	POS	POS	POS	POS	POS	POS	POS	POS	POS	NEG	NEG	AMB	POS	POS	POS	POS	NEG	NEG	NEG	POS	POS	POS	NEG	NEG	NEG	NEG	NEG	NEG	NEG	NEG	NEG	NEG	NEG	NEG	NEG	NEG	NEG	NEG	NEG
04V40423	CC22-MRSA-IV, Barnim/UK-EMRSA-15	MED-ICU	65	f	POS	POS	POS	POS	POS	POS	POS	POS	POS	POS	POS	NEG	NEG	NEG	POS	POS	POS	POS	NEG	NEG	NEG	AMB	POS	POS	NEG	NEG	NEG	NEG	NEG	NEG	NEG	NEG	NEG	NEG	NEG	NEG	NEG	NEG	NEG	NEG	NEG
04V08036	CC45-MRSA-IV [ACME+]	SUR-WARD	62	m	POS	POS	POS	POS	POS	POS	POS	POS	POS	POS	POS	NEG	NEG	NEG	POS	POS	POS	POS	NEG	NEG	NEG	POS	POS	POS	NEG	NEG	NEG	NEG	NEG	NEG	NEG	NEG	NEG	NEG	NEG	NEG	NEG	NEG	NEG	NEG	NEG
04U01119	CC45-MRSA-IV, Berlin EMRSA	MED-WARD	27	m	POS	POS	POS	POS	POS	POS	POS	POS	POS	POS	POS	NEG	NEG	NEG	POS	POS	POS	POS	NEG	NEG	NEG	POS	POS	POS	NEG	NEG	NEG	NEG	NEG	NEG	NEG	NEG	NEG	NEG	NEG	NEG	NEG	NEG	NEG	NEG	NEG
04V01185	CC45-MRSA-IV, Berlin EMRSA	MED-WARD	40	m	POS	POS	POS	POS	POS	POS	POS	POS	POS	POS	POS	NEG	NEG	NEG	POS	POS	POS	POS	NEG	NEG	NEG	AMB	POS	POS	NEG	NEG	NEG	NEG	NEG	NEG	NEG	NEG	NEG	NEG	NEG	NEG	NEG	NEG	NEG	NEG	NEG
04V06276	CC45-MRSA-IV, Berlin EMRSA	MED-WARD	50	f	POS	POS	POS	POS	POS	POS	POS	POS	POS	POS	POS	NEG	NEG	NEG	POS	POS	POS	POS	NEG	NEG	NEG	POS	POS	POS	NEG	NEG	NEG	NEG	NEG	NEG	NEG	NEG	NEG	NEG	NEG	NEG	NEG	NEG	NEG	NEG	NEG
04V06677	CC45-MRSA-IV, Berlin EMRSA	SUR-WARD	68	f	POS	POS	POS	POS	POS	NEG	POS	POS	POS	POS	AMB	NEG	NEG	NEG	POS	POS	POS	POS	NEG	NEG	NEG	POS	POS	POS	NEG	NEG	NEG	NEG	NEG	NEG	NEG	NEG	NEG	NEG	NEG	NEG	NEG	NEG	NEG	NEG	NEG
04V06688	CC45-MRSA-IV, Berlin EMRSA	MED-WARD	81	m	POS	POS	POS	POS	POS	POS	POS	POS	POS	POS	POS	NEG	NEG	NEG	POS	POS	POS	POS	NEG	NEG	NEG	POS	POS	POS	NEG	NEG	NEG	NEG	NEG	NEG	NEG	NEG	NEG	NEG	NEG	NEG	NEG	NEG	NEG	NEG	NEG
04V14835	CC45-MRSA-IV, Berlin EMRSA	MED-WARD	55	m	POS	POS	POS	POS	POS	POS	POS	POS	POS	POS	POS	NEG	NEG	NEG	POS	POS	POS	POS	NEG	NEG	NEG	POS	POS	POS	NEG	NEG	NEG	NEG	NEG	NEG	NEG	NEG	NEG	NEG	NEG	NEG	NEG	NEG	NEG	NEG	NEG
04V22079	CC45-MRSA-IV, Berlin EMRSA	MED-WARD	50	f	POS	POS	POS	POS	POS	POS	POS	POS	POS	POS	POS	NEG	NEG	NEG	POS	POS	POS	POS	NEG	NEG	NEG	POS	POS	POS	NEG	NEG	NEG	NEG	NEG	NEG	NEG	NEG	NEG	NEG	NEG	NEG	NEG	NEG	NEG	NEG	NEG
04V29100	CC45-MRSA-IV, Berlin EMRSA	MED-WARD	7	f	POS	POS	POS	POS		POS	POS	POS			POS	NEG	NEG	NEG	POS	POS																									
04V31370	CC45-MRSA-IV, Berlin EMRSA	MED-ICU	80	m	POS	POS	POS	POS	POS	POS	POS	POS	POS	POS	POS	NEG	NEG	NEG	POS	POS	POS	POS	NEG	NEG	NEG	POS	POS	POS	NEG	NEG	NEG	NEG	NEG	NEG	NEG	NEG	NEG	NEG	NEG	NEG	NEG	NEG	NEG	NEG	NEG
04V38442	CC45-MRSA-IV, Berlin EMRSA	MED-WARD	80	f	POS	POS	POS	POS	POS	POS	POS	POS	POS	POS	POS	NEG	NEG	NEG	POS	POS	POS	POS	NEG	NEG	NEG	POS	POS	POS	NEG	NEG	NEG	NEG	NEG	NEG	NEG	NEG	NEG	NEG	NEG	NEG	NEG	NEG	NEG	NEG	NEG
04V39012	CC45-MRSA-IV, Berlin EMRSA	MED-OPD	60	m	POS	POS	POS	POS	POS	POS	POS	POS	POS	POS	POS	NEG	NEG	NEG	POS	POS	POS	POS	NEG	NEG	NEG	POS	POS	POS	NEG	NEG	NEG	NEG	NEG	NEG	NEG	NEG	NEG	NEG	NEG	NEG	NEG	NEG	NEG	NEG	NEG
04V39577	CC45-MRSA-IV, Berlin EMRSA	MED-OPD	54	m	POS	POS	POS	POS	POS	POS	POS	POS	POS	POS	POS	NEG	NEG	NEG	POS	POS	POS	POS	NEG	NEG	NEG	POS	POS	POS	NEG	NEG	NEG	NEG	NEG	NEG	NEG	NEG	NEG	NEG	NEG	NEG	NEG	NEG	NEG	NEG	NEG
04V22872	CC152-MRSA-V [PVL+]	MED-OPD	54	m	POS	POS	POS	POS	POS	POS	POS	POS	POS	POS	POS	NEG	NEG	POS	POS	POS	NEG	POS	NEG	NEG	NEG	NEG	NEG	NEG	NEG	NEG	NEG	NEG	NEG	NEG	NEG	NEG	NEG	NEG	NEG	NEG	POS	POS	POS	NEG	NEG

ISOLATE:	STRAIN	RESISTANCE : PENICILLINASE			RESISTANCE : MLS-ANTIBIOTICS												RESISTANCE : AMINOGLYSOSIDES			RESISTANCE : MISCELLANEOUS GENES																RESISTANCE : EFFLUX SYSTEMS								RESISTANCE : GLYCOPEPTIDES		
		beta-lactamase	beta lactamase repressor (inhibitor)	beta-lactamase regulatory protein	rRNA adenine N-6-methyl-transferase, erythromycin/clindamycin resistance	erythro-mycin/clindamycin resistance	erythro-mycin/clindamycin resistance	Linco-samid-Nucleo-tidyltrans-ferase	energy-dependent efflux of erythro-mycin	macrolide efflux protein A	probable lysylphos-phatidyl-glycerol synthetase	virginia-mycin A acetyltransferase	acetyl-transferase inactivating streptogramin A	ATP binding protein, streptogramin-A-resistance		virginia-mycin B hydrolase	bifunctional enzyme Aac/Aph, gentamicin resistance	amino-glycoside adenyl-transferase,tobramycin resistance	3'5'-aminoglycoside phospho-transferase, neo-/ kanamycin resistance	strepto-thricine-acetyl-transferase	dihydro-folate reductase type 1	fusidic acid resistence	hypothetical protein associated with fusidic acid resistance	mupirocin resistence protein	tetrazyklin-resistance	tetracycline-resistance	chloramphenicol acetyltransferase					23S rRNA methyltransfera se	chloramphenicol /florfenicol exporter	metallothiol transferase		quaternary ammonium compound resistance protein A	quaternary ammonium compound resistance protein C						Transport/ Efflux protein	vancomycin resistance gene	vancomycin resistance gene from enterococci and Clostridium	teicoplanin resistance gene from enterococci
		blaZ	blaI	blaR	erm(A)	erm(B)	erm(C)	lnu(A)	msr(A)	mef(A)	mph(C)	vat(A)	vat(B)	vga(A)	vga(A) (BM 3327)	vgB(A)	aacA-aphD	aadD	aphA3	sat	dfrA	far1	Q6GD50	mupR	tet(K)	tet(M)	cat (total)	cat (pC221)	cat (pc223)	cat (pMC524)	cat (pSBK203R)	cfr	fexA	fosB	fosB (plasmid)	qacA	qacC (total)	qacC (consensus)	qacC (equine)	qacC (SA5)	qacC (Ssap)	qacC (ST94)	tetEfflux	vanA	vanB	vanZ
**2003**																																														
03F01030	CC5/ST228-MRSA, South German EMRSA (truncated SCCmec element)	POS	POS	POS	NEG	NEG	NEG	NEG	NEG	NEG	NEG	NEG	NEG	NEG	NEG	NEG	POS	NEG	POS	POS	NEG	NEG	NEG	NEG	NEG	NEG	NEG	NEG	NEG	NEG	NEG	NEG	NEG	POS	NEG	POS	NEG	NEG	NEG	NEG	NEG	NEG	POS	NEG	NEG	NEG
03V05419	CC5/ST228-MRSA-I, South German EMRSA	POS	POS	POS	POS	NEG	NEG	NEG	NEG	NEG	NEG	NEG	NEG	NEG	NEG	NEG	POS	NEG	POS	POS	NEG	NEG	NEG	NEG	NEG	NEG	NEG	NEG	NEG	NEG	NEG	NEG	NEG	POS	NEG	POS	NEG	NEG	NEG	NEG	NEG	NEG	POS	NEG	NEG	NEG
03V08986	CC5/ST228-MRSA-I, South German EMRSA	NEG	NEG	NEG	POS	NEG	NEG	NEG	NEG	NEG	NEG	NEG	NEG	NEG	NEG	NEG	POS	NEG	POS	POS	NEG	NEG	NEG	NEG	NEG	NEG	NEG	NEG	NEG	NEG	NEG	NEG	NEG	POS	NEG	NEG	NEG	NEG	NEG	NEG	NEG	NEG	POS	NEG	NEG	NEG
03V39123	CC5/ST228-MRSA-I, South German EMRSA	POS			POS		NEG	NEG	NEG			NEG	NEG	NEG	NEG	NEG	POS	NEG	POS	POS	NEG	NEG		NEG	NEG	NEG																		NEG	NEG	NEG
03B05186	CC5-MRSA-II, Rhine-Hesse EMRSA/UK-EMRSA-3	POS	POS	POS	POS	NEG	NEG	NEG	NEG	NEG	NEG	NEG	NEG	NEG	NEG	NEG	NEG	POS	NEG	NEG	NEG	NEG	NEG	NEG	NEG	NEG	NEG	NEG	NEG	NEG	NEG	NEG	NEG	POS	NEG	NEG	NEG	NEG	NEG	NEG	NEG	NEG	POS	NEG	NEG	NEG
03U00003	CC5-MRSA-II, Rhine-Hesse EMRSA/UK-EMRSA-3	POS	POS	POS	POS	NEG	NEG	NEG	NEG	NEG	NEG	NEG	NEG	NEG	NEG	NEG	NEG	POS	NEG	NEG	NEG	NEG	NEG	NEG	NEG	NEG	NEG	NEG	NEG	NEG	NEG	NEG	NEG	POS	NEG	NEG	NEG	NEG	NEG	NEG	NEG	NEG	POS	NEG	NEG	NEG
03V01438	CC5-MRSA-II, Rhine-Hesse EMRSA/UK-EMRSA-3	POS	POS	POS	POS	NEG	NEG	NEG	NEG	NEG	NEG	NEG	NEG	NEG	NEG	NEG	NEG	POS	NEG	NEG	NEG	NEG	NEG	NEG	NEG	NEG	NEG	NEG	NEG	NEG	NEG	NEG	NEG	POS	NEG	NEG	NEG	NEG	NEG	NEG	NEG	NEG	POS	NEG	NEG	NEG
03V05075	CC5-MRSA-II, Rhine-Hesse EMRSA/UK-EMRSA-3	POS	POS	POS	POS	NEG	NEG	NEG	NEG	NEG	NEG	NEG	NEG	NEG	NEG	NEG	NEG	POS	NEG	NEG	NEG	NEG	NEG	NEG	NEG	NEG	NEG	NEG	NEG	NEG	NEG	NEG	NEG	POS	NEG	NEG	NEG	NEG	NEG	NEG	NEG	NEG	POS	NEG	NEG	NEG
03V11014	CC5-MRSA-II, Rhine-Hesse EMRSA/UK-EMRSA-3	POS	POS	POS	POS	NEG	NEG	NEG	NEG	NEG	NEG	NEG	NEG	NEG	NEG	NEG	NEG	POS	NEG	NEG	NEG	NEG	NEG	NEG	NEG	NEG	NEG	NEG	NEG	NEG	NEG	NEG	NEG	POS	NEG	NEG	NEG	NEG	NEG	NEG	NEG	NEG	POS	NEG	NEG	NEG
03V12917	CC5-MRSA-II, Rhine-Hesse EMRSA/UK-EMRSA-3	POS	POS	POS	POS	NEG	NEG	NEG	NEG	NEG	NEG	NEG	NEG	NEG	NEG	NEG	NEG	POS	NEG	NEG	NEG	NEG	NEG	NEG	NEG	NEG	NEG	NEG	NEG	NEG	NEG	NEG	NEG	POS	NEG	NEG	NEG	NEG	NEG	NEG	NEG	NEG	POS	NEG	NEG	NEG
03V14467	CC5-MRSA-II, Rhine-Hesse EMRSA/UK-EMRSA-3	POS	POS	POS	POS	NEG	NEG	NEG	NEG	NEG	NEG	NEG	NEG	NEG	NEG	NEG	NEG	POS	NEG	NEG	NEG	NEG	NEG	NEG	NEG	NEG	NEG	NEG	NEG	NEG	NEG	NEG	NEG	POS	NEG	NEG	NEG	NEG	NEG	NEG	NEG	NEG	POS	NEG	NEG	NEG
03V19357	CC5-MRSA-II, Rhine-Hesse EMRSA/UK-EMRSA-3	POS	POS	POS	POS	NEG	NEG	NEG	NEG	NEG	NEG	NEG	NEG	NEG	NEG	NEG	NEG	AMB	NEG	NEG	NEG	NEG	NEG	NEG	NEG	NEG	NEG	NEG	NEG	NEG	NEG	NEG	NEG	POS	NEG	NEG	NEG	NEG	NEG	NEG	NEG	NEG	POS	NEG	NEG	NEG
03V20186	CC5-MRSA-II, Rhine-Hesse EMRSA/UK-EMRSA-3	POS	POS	POS	POS	NEG	NEG	NEG	NEG	NEG	NEG	NEG	NEG	NEG	NEG	NEG	NEG	POS	NEG	NEG	NEG	NEG	NEG	NEG	NEG	NEG	NEG	NEG	NEG	NEG	NEG	NEG	NEG	POS	NEG	NEG	NEG	NEG	NEG	NEG	NEG	NEG	POS	NEG	NEG	NEG
03V25349	CC5-MRSA-II, Rhine-Hesse EMRSA/UK-EMRSA-3	POS	POS	POS	POS	NEG	NEG	NEG	NEG	NEG	NEG	NEG	NEG	NEG	NEG	NEG	NEG	POS	NEG	NEG	NEG	NEG	NEG	NEG	NEG	NEG	NEG	NEG	NEG	NEG	NEG	NEG	NEG	POS	NEG	NEG	NEG	NEG	NEG	NEG	NEG	NEG	POS	NEG	NEG	NEG
03V28948	CC5-MRSA-II, Rhine-Hesse EMRSA/UK-EMRSA-3	POS	POS	POS	POS	NEG	NEG	NEG	NEG	NEG	NEG	NEG	NEG	NEG	NEG	NEG	NEG	AMB	NEG	NEG	NEG	NEG	NEG	NEG	NEG	NEG	NEG	NEG	NEG	NEG	NEG	NEG	NEG	POS	NEG	NEG	NEG	NEG	NEG	NEG	NEG	NEG	POS	NEG	NEG	NEG
03V30841	CC5-MRSA-II, Rhine-Hesse EMRSA/UK-EMRSA-3	POS			POS		NEG	NEG	NEG			NEG	NEG	NEG	NEG	NEG	NEG	POS	NEG	NEG	NEG	NEG		NEG	NEG	NEG																		NEG	NEG	NEG
03V32896	CC5-MRSA-II, Rhine-Hesse EMRSA/UK-EMRSA-3	POS	POS	POS	POS	NEG	NEG	NEG	NEG	NEG	NEG	NEG	NEG	NEG	NEG	NEG	NEG	POS	NEG	NEG	NEG	NEG	NEG	NEG	NEG	NEG	NEG	NEG	NEG	NEG	NEG	NEG	NEG	POS	NEG	NEG	NEG	NEG	NEG	NEG	NEG	NEG	POS	NEG	NEG	NEG
03V33415	CC5-MRSA-II, Rhine-Hesse EMRSA/UK-EMRSA-3	NEG	NEG	NEG	POS	NEG	NEG	NEG	NEG	NEG	NEG	NEG	NEG	NEG	NEG	NEG	NEG	POS	NEG	NEG	NEG	NEG	NEG	NEG	NEG	NEG	NEG	NEG	NEG	NEG	NEG	NEG	NEG	POS	NEG	NEG	NEG	NEG	NEG	NEG	NEG	NEG	POS	NEG	NEG	NEG
03V34628	CC5-MRSA-II, Rhine-Hesse EMRSA/UK-EMRSA-3	POS	POS	POS	POS	NEG	NEG	NEG	NEG	NEG	NEG	NEG	NEG	AMB	NEG	NEG	NEG	POS	NEG	NEG	NEG	NEG	NEG	NEG	NEG	NEG	NEG	NEG	NEG	NEG	NEG	NEG	NEG	POS	AMB	NEG	NEG	NEG	NEG	NEG	NEG	NEG	POS	NEG	NEG	NEG
03V37078	CC5-MRSA-II, Rhine-Hesse EMRSA/UK-EMRSA-3	NEG	NEG	NEG	POS	NEG	NEG	NEG	NEG	NEG	NEG	NEG	NEG	NEG	NEG	NEG	NEG	POS	NEG	NEG	NEG	NEG	NEG	NEG	NEG	NEG	NEG	NEG	NEG	NEG	NEG	NEG	NEG	POS	NEG	NEG	NEG	NEG	NEG	NEG	NEG	NEG	POS	NEG	NEG	NEG
03V41645	CC5-MRSA-II, Rhine-Hesse EMRSA/UK-EMRSA-3	POS	POS	POS	POS	NEG	NEG	NEG	NEG	NEG	NEG	NEG	NEG	NEG	NEG	NEG	NEG	POS	NEG	NEG	NEG	NEG	NEG	NEG	NEG	NEG	NEG	NEG	NEG	NEG	NEG	NEG	NEG	POS	NEG	NEG	NEG	NEG	NEG	NEG	NEG	NEG	POS	NEG	NEG	NEG
03V03012	CC5-MRSA-IV, Paediatric clone	POS	POS	POS	NEG		NEG	NEG	NEG	NEG	NEG	NEG	NEG	NEG	NEG	NEG	NEG	NEG	NEG	NEG	NEG	NEG	NEG	NEG	NEG	NEG	NEG	NEG	NEG	NEG	NEG	NEG	NEG	POS	NEG	NEG	NEG	NEG	NEG	NEG	NEG	NEG	POS	NEG	NEG	NEG
03V02863	CC8/ST247-MRSA-I, North German/Iberian EMRSA	NEG	NEG	NEG	POS	NEG	NEG	NEG	NEG	NEG	NEG	NEG	NEG	NEG	NEG	NEG	POS	POS	NEG	NEG	NEG	NEG	NEG	NEG	NEG	POS	NEG	NEG	NEG	NEG	NEG	NEG	NEG	POS	NEG	NEG	NEG	NEG	NEG	NEG	NEG	NEG	POS	NEG	NEG	NEG
03V14882	CC8/ST254-MRSA-(atypical SCCmec), Hannover EMRSA	POS	POS	POS	NEG	NEG	NEG	NEG	NEG	NEG	NEG	NEG	NEG	NEG	NEG	NEG	POS	NEG	POS	POS	NEG	NEG	NEG	NEG	NEG	POS	NEG	NEG	NEG	NEG	NEG	NEG	NEG	POS	NEG	NEG	NEG	NEG	NEG	NEG	NEG	NEG	POS	NEG	NEG	NEG
03B00404	CC22-MRSA-IV, Barnim/UK-EMRSA-15	POS	POS	POS	NEG	NEG	POS	NEG	NEG	NEG	NEG	NEG	NEG	NEG	NEG	NEG	NEG	NEG	NEG	NEG	NEG	NEG	NEG	NEG	NEG	NEG	NEG	NEG	NEG	NEG	NEG	NEG	NEG	NEG	NEG	NEG	NEG	NEG	NEG	NEG	NEG	NEG	NEG	NEG	NEG	NEG
03U00892	CC22-MRSA-IV, Barnim/UK-EMRSA-15	POS			NEG		POS	NEG	NEG			NEG	NEG	NEG	NEG	NEG	NEG	NEG	NEG	NEG	NEG	NEG		NEG	NEG	NEG																		NEG	NEG	NEG
03V01422	CC22-MRSA-IV, Barnim/UK-EMRSA-15	POS	POS	POS	NEG	NEG	POS	NEG	NEG	NEG	NEG	NEG	NEG	NEG	NEG	NEG	NEG	NEG	NEG	NEG	NEG	NEG	NEG	NEG	NEG	NEG	NEG	NEG	NEG	NEG	NEG	NEG	NEG	NEG	NEG	NEG	NEG	NEG	NEG	NEG	NEG	NEG	NEG	NEG	NEG	NEG
03V04424	CC22-MRSA-IV, Barnim/UK-EMRSA-15	POS	POS	POS	NEG	NEG	POS	NEG	NEG	NEG	NEG	NEG	NEG	NEG	NEG	NEG	NEG	NEG	NEG	NEG	NEG	NEG	NEG	NEG	NEG	NEG	NEG	NEG	NEG	NEG	NEG	NEG	NEG	NEG	NEG	NEG	NEG	NEG	NEG	NEG	NEG	NEG	NEG	NEG	NEG	NEG
03V05690	CC22-MRSA-IV, Barnim/UK-EMRSA-15	POS	POS	POS	NEG	NEG	NEG	NEG	NEG	NEG	NEG	NEG	NEG	NEG	NEG	NEG	NEG	POS	NEG	NEG	NEG	NEG	NEG	NEG	NEG	NEG	NEG	NEG	NEG	NEG	NEG	NEG	NEG	NEG	NEG	NEG	NEG	NEG	NEG	NEG	NEG	NEG	NEG	NEG	NEG	NEG
03V07912	CC22-MRSA-IV, Barnim/UK-EMRSA-15	POS	POS	POS	NEG		POS	NEG	NEG	NEG	NEG	NEG	NEG	NEG	NEG	NEG	NEG	NEG	NEG	NEG	NEG	NEG	NEG	NEG	NEG	NEG	NEG	NEG	NEG	NEG	NEG	NEG	NEG	NEG	NEG	NEG	NEG	NEG	NEG	NEG	NEG	NEG	NEG	NEG	NEG	NEG
03V23798	CC22-MRSA-IV, Barnim/UK-EMRSA-15	POS	POS	POS	NEG	NEG	POS	NEG	NEG	NEG	NEG	NEG	NEG	NEG	NEG	NEG	NEG	NEG	NEG	NEG	NEG	NEG	NEG	NEG	NEG	NEG	NEG	NEG	NEG	NEG	NEG	NEG	NEG	NEG	NEG	NEG	NEG	NEG	NEG	NEG	NEG	NEG	NEG	NEG	NEG	NEG
03V25914	CC22-MRSA-IV, Barnim/UK-EMRSA-15	POS	POS	POS	NEG	NEG	POS	NEG	NEG	NEG	NEG	NEG	NEG	NEG	NEG	NEG	NEG	NEG	NEG	NEG	NEG	NEG	NEG	NEG	NEG	NEG	NEG	NEG	NEG	NEG	NEG	NEG	NEG	NEG	NEG	NEG	NEG	NEG	NEG	NEG	NEG	NEG	NEG	NEG	NEG	NEG
03V29751	CC22-MRSA-IV, Barnim/UK-EMRSA-15	POS	POS	POS	NEG	NEG	NEG	NEG	NEG	NEG	NEG	NEG	NEG	NEG	NEG	NEG	NEG	NEG	NEG	NEG	NEG	NEG	NEG	NEG	NEG	NEG	NEG	NEG	NEG	NEG	NEG	NEG	NEG	NEG	NEG	NEG	NEG	NEG	NEG	NEG	NEG	NEG	NEG	NEG	NEG	NEG
03V30201	CC22-MRSA-IV, Barnim/UK-EMRSA-15	POS	POS	POS	NEG	NEG	POS	NEG	NEG	NEG	NEG	NEG	NEG	NEG	NEG	NEG	NEG	NEG	NEG	NEG	NEG	NEG	NEG	NEG	NEG	NEG	NEG	NEG	NEG	NEG	NEG	NEG	NEG	NEG	NEG	NEG	NEG	NEG	NEG	NEG	NEG	NEG	NEG	NEG	NEG	NEG
03B01781	CC45-MRSA-IV, Berlin EMRSA	POS	POS	POS	NEG	NEG	POS	NEG	NEG	NEG	NEG	NEG	NEG	NEG	NEG	NEG	NEG	NEG	POS	POS	NEG	NEG	NEG	NEG	NEG	NEG	NEG	NEG	NEG	NEG	NEG	NEG	NEG	NEG	NEG	NEG	NEG	NEG	NEG	NEG	NEG	NEG	POS	NEG	NEG	NEG
03U05014	CC45-MRSA-IV, Berlin EMRSA	POS	POS	POS	NEG	NEG	NEG	NEG	NEG	NEG	NEG	NEG	NEG	NEG	NEG	NEG	NEG	NEG	POS	POS	NEG	NEG	NEG	NEG	NEG	NEG	NEG	NEG	NEG	NEG	NEG	NEG	NEG	NEG	NEG	NEG	NEG	NEG	NEG	NEG	NEG	NEG	POS	NEG	NEG	NEG
03U11106	CC45-MRSA-IV, Berlin EMRSA	POS	POS	POS	NEG	NEG	NEG	NEG	NEG	NEG	NEG	NEG	NEG	NEG	NEG	NEG	NEG	NEG	NEG	NEG	NEG	NEG	NEG	NEG	NEG	NEG	NEG	NEG	NEG	NEG	NEG	NEG	NEG	NEG	NEG	NEG	NEG	NEG	NEG	NEG	NEG	NEG	POS	NEG	NEG	NEG
03V00742	CC45-MRSA-IV, Berlin EMRSA	POS	POS	POS	NEG	NEG	NEG	NEG	NEG	NEG	NEG	NEG	NEG	NEG	NEG	NEG	NEG	NEG	POS	POS	NEG	NEG	NEG	NEG	NEG	NEG	NEG	NEG	NEG	NEG	NEG	NEG	NEG	NEG	NEG	NEG	NEG	NEG	NEG	NEG	NEG	NEG	POS	NEG	NEG	NEG
03V01902	CC45-MRSA-IV, Berlin EMRSA	POS	POS	POS	NEG	NEG	POS	NEG	NEG	NEG	NEG	NEG	NEG	NEG	NEG	NEG	NEG	NEG	POS	POS	NEG	NEG	NEG	NEG	NEG	NEG	NEG	NEG	NEG	NEG	NEG	NEG	NEG	NEG	NEG	NEG	NEG	NEG	NEG	NEG	NEG	NEG	POS	NEG	NEG	NEG
03V02256	CC45-MRSA-IV, Berlin EMRSA	POS	POS	POS	NEG	NEG	POS	NEG	NEG	NEG	NEG	NEG	NEG	NEG	NEG	NEG	NEG	NEG	POS	POS	NEG	NEG	NEG	NEG	POS	NEG	NEG	NEG	NEG	NEG	NEG	NEG	NEG	NEG	NEG	NEG	NEG	NEG	NEG	NEG	NEG	NEG	POS	NEG	NEG	NEG
03V02262	CC45-MRSA-IV, Berlin EMRSA	POS	POS	POS	NEG	NEG	NEG	NEG	NEG	NEG	NEG	NEG	NEG	NEG	NEG	NEG	NEG	NEG	POS	POS	NEG	NEG	NEG	NEG	NEG	NEG	NEG	NEG	NEG	NEG	NEG	NEG	NEG	NEG	NEG	NEG	NEG	NEG	NEG	NEG	NEG	NEG	POS	NEG	NEG	NEG
03V04701	CC45-MRSA-IV, Berlin EMRSA	POS	POS	POS	NEG	NEG	NEG	NEG	NEG	NEG	NEG	NEG	NEG	NEG	NEG	NEG	NEG	NEG	POS	POS	NEG	NEG	NEG	NEG	NEG	NEG	NEG	NEG	NEG	NEG	NEG	NEG	NEG	NEG	NEG	NEG	NEG	NEG	NEG	NEG	NEG	NEG	AMB	NEG	NEG	NEG
03V05042	CC45-MRSA-IV, Berlin EMRSA	POS	POS	POS	NEG	NEG	NEG	NEG	NEG	NEG	NEG	NEG	NEG	NEG	NEG	NEG	NEG	NEG	POS	POS	POS	NEG	NEG	NEG	NEG	NEG	NEG	NEG	NEG	NEG	NEG	NEG	NEG	NEG	NEG	NEG	POS	POS	NEG	NEG	NEG	NEG	POS	NEG	NEG	NEG
03V05377	CC45-MRSA-IV, Berlin EMRSA	POS	POS	POS	NEG	NEG	NEG	NEG	NEG	NEG	NEG	NEG	NEG	NEG	NEG	NEG	POS	NEG	POS	POS	POS	NEG	NEG	NEG	NEG	NEG	NEG	NEG	NEG	NEG	NEG	NEG	NEG	NEG	NEG	NEG	POS	POS	NEG	NEG	NEG	NEG	POS	NEG	NEG	NEG
03V05507	CC45-MRSA-IV, Berlin EMRSA	POS	POS	POS	NEG	NEG	NEG	NEG	NEG	NEG	NEG	NEG	NEG	NEG	NEG	NEG	NEG	NEG	POS	POS	NEG	NEG	NEG	NEG	NEG	NEG	NEG	NEG	NEG	NEG	NEG	NEG	NEG	NEG	NEG	NEG	NEG	NEG	NEG	NEG	NEG	NEG	POS	NEG	NEG	NEG
03V08214	CC45-MRSA-IV, Berlin EMRSA	POS	POS	POS	NEG	NEG	POS	NEG	NEG	NEG	NEG	NEG	NEG	NEG	NEG	NEG	POS	POS	POS	POS	POS	POS	NEG	NEG	NEG	NEG	NEG	NEG	NEG	NEG	NEG	NEG	NEG	NEG	NEG	NEG	POS	POS	NEG	NEG	NEG	NEG	POS	NEG	NEG	NEG
03V08525	CC45-MRSA-IV, Berlin EMRSA	POS	POS	POS	NEG	NEG	POS	NEG	NEG	NEG	NEG	NEG	NEG	NEG	NEG	NEG	NEG	NEG	POS	POS	NEG	NEG	NEG	NEG	NEG	NEG	NEG	NEG	NEG	NEG	NEG	NEG	NEG	NEG	NEG	NEG	NEG	NEG	NEG	NEG	NEG	NEG	AMB	NEG	NEG	NEG
03V11910	CC45-MRSA-IV, Berlin EMRSA	POS	POS	POS	NEG	NEG	NEG	NEG	NEG	NEG	NEG	NEG	NEG	NEG	NEG	NEG	NEG	NEG	POS	POS	NEG	NEG	NEG	NEG	NEG	NEG	NEG	NEG	NEG	NEG	NEG	NEG	NEG	NEG	NEG	NEG	NEG	NEG	NEG	NEG	NEG	NEG	NEG	NEG	NEG	NEG
03V14481	CC45-MRSA-IV, Berlin EMRSA	POS	POS	POS	NEG	NEG	POS	NEG	NEG	NEG	NEG	NEG	NEG	NEG	NEG	NEG	NEG	NEG	POS	POS	NEG	NEG	NEG	NEG	NEG	NEG	NEG	NEG	NEG	NEG	NEG	NEG	NEG	NEG	NEG	NEG	NEG	NEG	NEG	NEG	NEG	NEG	POS	NEG	NEG	NEG
03V15082	CC45-MRSA-IV, Berlin EMRSA	POS	POS	POS	NEG	NEG	NEG	NEG	NEG	NEG	NEG	NEG	NEG	NEG	NEG	NEG	NEG	NEG	POS	POS	NEG	NEG	NEG	NEG	NEG	NEG	NEG	NEG	NEG	NEG	NEG	NEG	NEG	NEG	NEG	NEG	NEG	NEG	NEG	NEG	NEG	NEG	POS	NEG	NEG	NEG
03V17508	CC45-MRSA-IV, Berlin EMRSA	POS	POS	POS	NEG	NEG	POS	NEG	NEG	NEG	NEG	NEG	NEG	NEG	NEG	NEG	NEG	NEG	POS	POS	NEG	NEG	NEG	NEG	NEG	NEG	NEG	NEG	NEG	NEG	NEG	NEG	NEG	NEG	NEG	NEG	NEG	NEG	NEG	NEG	NEG	NEG	AMB	NEG	NEG	NEG
03V20150	CC45-MRSA-IV, Berlin EMRSA	POS	POS	POS	NEG	NEG	POS	NEG	NEG	NEG	NEG	NEG	NEG	NEG	NEG	NEG	NEG	NEG	POS	POS	NEG	NEG	NEG	NEG	NEG	NEG	NEG	NEG	NEG	NEG	NEG	NEG	NEG	NEG	NEG	POS	NEG	NEG	NEG	NEG	NEG	NEG	NEG	NEG	NEG	NEG
03V22437	CC45-MRSA-IV, Berlin EMRSA	POS	POS	POS	NEG	NEG	NEG	NEG	NEG	NEG	NEG	NEG	NEG	NEG	NEG	NEG	NEG	NEG	POS	POS	NEG	NEG	NEG	NEG	NEG	NEG	NEG	NEG	NEG	NEG	NEG	NEG	NEG	NEG	NEG	NEG	NEG	NEG	NEG	NEG	NEG	NEG	AMB	NEG	NEG	NEG
03V24471	CC45-MRSA-IV, Berlin EMRSA	POS	POS	POS	NEG	NEG	NEG	NEG	NEG	NEG	NEG	NEG	NEG	NEG	NEG	NEG	NEG	NEG	POS	POS	NEG	NEG	NEG	NEG	NEG	NEG	NEG	NEG	NEG	NEG	NEG	NEG	NEG	NEG	NEG	NEG	NEG	NEG	NEG	NEG	NEG	NEG	POS	NEG	NEG	NEG
03V28070	CC45-MRSA-IV, Berlin EMRSA	POS	POS	POS	NEG	AMB	NEG	NEG	NEG	NEG	NEG	NEG	NEG	NEG	NEG	NEG	NEG	NEG	AMB	POS	NEG	NEG	NEG	NEG	NEG	NEG	NEG	NEG	NEG	NEG	NEG	NEG	NEG	NEG	NEG	NEG	NEG	NEG	NEG	NEG	NEG	NEG	NEG	NEG	NEG	NEG
03V28448	CC45-MRSA-IV, Berlin EMRSA	POS	POS	POS	NEG	NEG	NEG	NEG	NEG	NEG	NEG	NEG	NEG	NEG	NEG	NEG	NEG	NEG	POS	POS	NEG	NEG	NEG	NEG	NEG	NEG	NEG	NEG	NEG	NEG	NEG	NEG	NEG	NEG	NEG	NEG	NEG	NEG	NEG	NEG	NEG	NEG	POS	NEG	NEG	NEG
03V34589	CC45-MRSA-IV, Berlin EMRSA	POS	POS	POS	NEG	NEG	AMB	NEG	NEG	NEG	NEG	NEG	NEG	NEG	NEG	NEG	NEG	NEG	POS	POS	NEG	NEG	NEG	NEG	POS	NEG	NEG	NEG	NEG	NEG	NEG	NEG	NEG	NEG	NEG	POS	NEG	NEG	NEG	NEG	NEG	NEG	POS	NEG	NEG	NEG
03V39774	CC45-MRSA-IV, Berlin EMRSA	POS	POS	POS	NEG	NEG	NEG	NEG	NEG	NEG	NEG	NEG	NEG	NEG	NEG	NEG	NEG	NEG	POS	POS	NEG	NEG	NEG	NEG	NEG	NEG	NEG	NEG	NEG	NEG	NEG	NEG	NEG	NEG	NEG	NEG	POS	POS	NEG	NEG	NEG	NEG	POS	NEG	NEG	NEG
**2004**																																														
04B05423	CC5-MRSA-II, Rhine-Hesse EMRSA/UK-EMRSA-3	NEG	NEG	NEG	POS	NEG	NEG	NEG	NEG	NEG	NEG	NEG	NEG	NEG	NEG	NEG	NEG	POS	NEG	NEG	NEG	NEG	NEG	NEG	NEG	NEG	NEG	NEG	NEG	NEG	NEG	NEG	NEG	POS	NEG	NEG	NEG	NEG	NEG	NEG	NEG	NEG	POS	NEG	NEG	NEG
04U02171	CC5-MRSA-II, Rhine-Hesse EMRSA/UK-EMRSA-3	POS	POS	POS	POS	NEG	NEG	NEG	NEG	NEG	NEG	NEG	NEG	NEG	NEG	NEG	NEG	POS	NEG	NEG	NEG	NEG	NEG	NEG	NEG	NEG	NEG	NEG	NEG	NEG	NEG	NEG	NEG	POS	NEG	NEG	NEG	NEG	NEG	NEG	NEG	NEG	POS	NEG	NEG	NEG
04U03682	CC5-MRSA-II, Rhine-Hesse EMRSA/UK-EMRSA-3	POS	POS	POS	POS	NEG	NEG	NEG	NEG	NEG	NEG	NEG	NEG	NEG	NEG	NEG	NEG	POS	NEG	NEG	NEG	NEG	NEG	NEG	NEG	NEG	NEG	NEG	NEG	NEG	NEG	NEG	NEG	POS	NEG	NEG	NEG	NEG	NEG	NEG	NEG	NEG	POS	NEG	NEG	NEG
04U06618	CC5-MRSA-II, Rhine-Hesse EMRSA/UK-EMRSA-3	POS	POS	POS	POS	NEG	NEG	NEG	NEG	NEG	NEG	NEG	NEG	NEG	NEG	NEG	NEG	NEG	NEG	NEG	NEG	NEG	NEG	NEG	NEG	NEG	NEG	NEG	NEG	NEG	NEG	NEG	NEG	POS	NEG	NEG	NEG	NEG	NEG	NEG	NEG	NEG	POS	NEG	NEG	NEG
04U12578	CC5-MRSA-II, Rhine-Hesse EMRSA/UK-EMRSA-3	POS	POS	POS	POS	NEG	NEG	NEG	NEG	NEG	NEG	NEG	NEG	NEG	NEG	NEG	NEG	NEG	NEG	NEG	NEG	NEG	NEG	NEG	NEG	NEG	NEG	NEG	NEG	NEG	NEG	NEG	NEG	POS	NEG	NEG	NEG	NEG	NEG	NEG	NEG	NEG	POS	NEG	NEG	NEG
04V02465	CC5-MRSA-II, Rhine-Hesse EMRSA/UK-EMRSA-3	POS	POS	POS	POS	NEG	NEG	NEG	NEG	NEG	NEG	NEG	NEG	NEG	NEG	NEG	NEG	POS	NEG	NEG	NEG	NEG	NEG	NEG	NEG	NEG	NEG	NEG	NEG	NEG	NEG	NEG	NEG	POS	NEG	NEG	NEG	NEG	NEG	NEG	NEG	NEG	POS	NEG	NEG	NEG
04V03642	CC5-MRSA-II, Rhine-Hesse EMRSA/UK-EMRSA-3	NEG	NEG	NEG	POS	NEG	NEG	NEG	NEG	NEG	NEG	NEG	NEG	NEG	NEG	NEG	NEG	AMB	NEG	NEG	NEG	NEG	NEG	NEG	NEG	NEG	NEG	NEG	NEG	NEG	NEG	NEG	NEG	POS	NEG	NEG	NEG	NEG	NEG	NEG	NEG	NEG	POS	NEG	NEG	NEG
04V07657	CC5-MRSA-II, Rhine-Hesse EMRSA/UK-EMRSA-3	POS	POS	POS	POS	NEG	NEG	NEG	NEG	NEG	NEG	NEG	NEG	NEG	NEG	NEG	NEG	POS	NEG	NEG	NEG	NEG	NEG	NEG	NEG	NEG	NEG	NEG	NEG	NEG	NEG	NEG	NEG	POS	NEG	NEG	NEG	NEG	NEG	NEG	NEG	NEG	POS	NEG	NEG	NEG
04V12223	CC5-MRSA-II, Rhine-Hesse EMRSA/UK-EMRSA-3	POS	POS	POS	POS	NEG	NEG	NEG	NEG	NEG	NEG	NEG	NEG	NEG	NEG	NEG	NEG	AMB	NEG	NEG	NEG	NEG	NEG	NEG	NEG	NEG	NEG	NEG	NEG	NEG	NEG	NEG	NEG	POS	NEG	NEG	NEG	NEG	NEG	NEG	NEG	NEG	POS	NEG	NEG	NEG
04V12529	CC5-MRSA-II, Rhine-Hesse EMRSA/UK-EMRSA-3	POS	POS	POS	POS	NEG	NEG	NEG	NEG	NEG	NEG	NEG	NEG	NEG	NEG	NEG	NEG	POS	NEG	NEG	NEG	NEG	NEG	NEG	NEG	NEG	NEG	NEG	NEG	NEG	NEG	NEG	NEG	POS	NEG	NEG	NEG	NEG	NEG	NEG	NEG	NEG	POS	NEG	NEG	NEG
04V13633	CC5-MRSA-II, Rhine-Hesse EMRSA/UK-EMRSA-3	POS	POS	POS	POS	NEG	NEG	NEG	NEG	NEG	NEG	NEG	NEG	NEG	NEG	NEG	NEG	AMB	NEG	NEG	NEG	NEG	NEG	NEG	NEG	NEG	NEG	NEG	NEG	NEG	NEG	NEG	NEG	POS	NEG	NEG	NEG	NEG	NEG	NEG	NEG	NEG	POS	NEG	NEG	NEG
04V16499	CC5-MRSA-II, Rhine-Hesse EMRSA/UK-EMRSA-3	POS	POS	POS	POS	NEG	NEG	NEG	NEG	NEG	NEG	NEG	NEG	NEG	NEG	NEG	NEG	POS	NEG	NEG	NEG	NEG	NEG	NEG	NEG	NEG	NEG	NEG	NEG	NEG	NEG	NEG	NEG	POS	NEG	NEG	NEG	NEG	NEG	NEG	NEG	NEG	POS	NEG	NEG	NEG
04V17015	CC5-MRSA-II, Rhine-Hesse EMRSA/UK-EMRSA-3	POS	POS	POS	POS	NEG	NEG	NEG	NEG	NEG	NEG	NEG	NEG	NEG	NEG	NEG	NEG	POS	NEG	NEG	NEG	NEG	NEG	NEG	NEG	NEG	NEG	NEG	NEG	NEG	NEG	NEG	NEG	POS	NEG	NEG	NEG	NEG	NEG	NEG	NEG	NEG	POS	NEG	NEG	NEG
04V20684	CC5-MRSA-II, Rhine-Hesse EMRSA/UK-EMRSA-3	NEG			POS		NEG	NEG	NEG			NEG	NEG	NEG	NEG	NEG	NEG	POS	NEG	NEG	NEG	NEG		NEG	NEG	NEG																		NEG	NEG	NEG
04V24615	CC5-MRSA-II, Rhine-Hesse EMRSA/UK-EMRSA-3	NEG	NEG	NEG	POS	NEG	NEG	NEG	NEG	NEG	NEG	NEG	NEG	NEG	NEG	NEG	NEG	POS	NEG	NEG	NEG	NEG	NEG	NEG	NEG	NEG	NEG	NEG	NEG	NEG	NEG	NEG	NEG	POS	NEG	NEG	NEG	NEG	NEG	NEG	NEG	NEG	POS	NEG	NEG	NEG
04V24901	CC5-MRSA-II, Rhine-Hesse EMRSA/UK-EMRSA-3	NEG	NEG	NEG	POS	NEG	NEG	NEG	NEG	NEG	NEG	NEG	NEG	NEG	NEG	NEG	NEG	POS	NEG	NEG	NEG	NEG	NEG	NEG	NEG	NEG	NEG	NEG	NEG	NEG	NEG	NEG	NEG	POS	NEG	NEG	NEG	NEG	NEG	NEG	NEG	NEG	POS	NEG	NEG	NEG
04V25081	CC5-MRSA-II, Rhine-Hesse EMRSA/UK-EMRSA-3	POS	POS	POS	POS	NEG	NEG	NEG	NEG	NEG	NEG	NEG	NEG	NEG	NEG	NEG	NEG	POS	NEG	NEG	NEG	NEG	NEG	NEG	NEG	NEG	NEG	NEG	NEG	NEG	NEG	NEG	NEG	POS	NEG	NEG	NEG	NEG	NEG	NEG	NEG	NEG	POS	NEG	NEG	NEG
04V27293	CC5-MRSA-II, Rhine-Hesse EMRSA/UK-EMRSA-3	NEG	NEG	NEG	POS	NEG	NEG	NEG	NEG	NEG	NEG	NEG	NEG	NEG	NEG	NEG	NEG	POS	NEG	NEG	NEG	NEG	NEG	NEG	NEG	NEG	NEG	NEG	NEG	NEG	NEG	NEG	NEG	POS	NEG	NEG	NEG	NEG	NEG	NEG	NEG	NEG	POS	NEG	NEG	NEG
04V27311	CC5-MRSA-II, Rhine-Hesse EMRSA/UK-EMRSA-3	NEG	NEG	NEG	POS	NEG	NEG	NEG	NEG	NEG	NEG	NEG	NEG	NEG	NEG	NEG	NEG	AMB	NEG	NEG	NEG	NEG	NEG	NEG	NEG	NEG	NEG	NEG	NEG	NEG	NEG	NEG	NEG	POS	NEG	NEG	NEG	NEG	NEG	NEG	NEG	NEG	POS	NEG	NEG	NEG
04V33459	CC5-MRSA-II, Rhine-Hesse EMRSA/UK-EMRSA-3	POS	POS	POS	POS	NEG	NEG	NEG	NEG	NEG	NEG	NEG	NEG	NEG	NEG	NEG	NEG	POS	NEG	NEG	NEG	NEG	NEG	NEG	NEG	NEG	NEG	NEG	NEG	NEG	NEG	NEG	NEG	POS	NEG	NEG	NEG	NEG	NEG	NEG	NEG	NEG	POS	NEG	NEG	NEG
04V39909	CC5-MRSA-IV&VI	POS	POS	POS	NEG	NEG	POS	NEG	POS	NEG	POS	NEG	NEG	NEG	NEG	NEG	NEG	POS	POS	POS	NEG	NEG	NEG	NEG	NEG	NEG	NEG	NEG	NEG	NEG	NEG	NEG	NEG	POS	NEG	NEG	NEG	NEG	NEG	NEG	NEG	NEG	POS	NEG	NEG	NEG
04U02421	CC5-MRSA-IV, Paediatric clone	POS	POS	POS	NEG		NEG	NEG	NEG	NEG	NEG	NEG	NEG	NEG	NEG	NEG	NEG	POS	NEG	NEG	NEG	NEG	NEG	NEG	NEG	NEG	NEG	NEG	NEG	NEG	NEG	NEG	NEG	POS	NEG	NEG	NEG	NEG	NEG	NEG	NEG	NEG	POS	NEG	NEG	NEG
04V09807	CC80-MRSA-IV [PVL+], European caMRSA Clone	POS			NEG		NEG	NEG	NEG			NEG	NEG	NEG	NEG	NEG	NEG	NEG	POS	POS	NEG	POS		NEG	POS	NEG																		NEG	NEG	NEG
04V16073	CC80-MRSA-IV [PVL+], European caMRSA Clone	POS	POS	POS	NEG	NEG	NEG	NEG	NEG	NEG	NEG	NEG	NEG	NEG	NEG	NEG	NEG	NEG	POS	POS	NEG	POS	NEG	NEG	POS	NEG	NEG	NEG	NEG	NEG	NEG	NEG	NEG	NEG	NEG	NEG	NEG	NEG	NEG	NEG	NEG	NEG	POS	NEG	NEG	NEG
04V23548	CC80-MRSA-IV [PVL+], European caMRSA Clone	POS			NEG		NEG	NEG	NEG			NEG	NEG	NEG	NEG	NEG	NEG	NEG	POS	POS	NEG	POS		NEG	POS	NEG																		NEG	NEG	NEG
04V03289	CC22-MRSA-IV, Barnim/UK-EMRSA-15	POS			NEG		POS	NEG	NEG			NEG	NEG	NEG	NEG	NEG	NEG	NEG	NEG	NEG	NEG	NEG		NEG	NEG	NEG																		NEG	NEG	NEG
04V03847	CC22-MRSA-IV, Barnim/UK-EMRSA-15	POS			NEG		POS	NEG	NEG			NEG	NEG	NEG	NEG	NEG	NEG	NEG	NEG	NEG	NEG	NEG		NEG	NEG	NEG																		NEG	NEG	NEG
04V04595	CC22-MRSA-IV, Barnim/UK-EMRSA-15	POS	POS	POS	NEG	NEG	NEG	NEG	NEG	NEG	NEG	NEG	NEG	NEG	NEG	NEG	NEG	NEG	NEG	NEG	NEG	NEG	NEG	NEG	NEG	NEG	NEG	NEG	NEG	NEG	NEG	NEG	NEG	NEG	NEG	POS	NEG	NEG	NEG	NEG	NEG	NEG	NEG	NEG	NEG	NEG
04V05029	CC22-MRSA-IV, Barnim/UK-EMRSA-15	POS	POS	NEG	NEG	NEG	POS	NEG	NEG	NEG	NEG	NEG	NEG	NEG	NEG	NEG	NEG	NEG	NEG	NEG	NEG	NEG	NEG	NEG	NEG	NEG	NEG	NEG	NEG	NEG	NEG	NEG	NEG	NEG	NEG	NEG	NEG	NEG	NEG	NEG	NEG	NEG	NEG	NEG	NEG	NEG
04V07875	CC22-MRSA-IV, Barnim/UK-EMRSA-15	POS	POS	NEG	NEG	NEG	POS	NEG	NEG	NEG	NEG	NEG	NEG	NEG	NEG	NEG	NEG	NEG	NEG	NEG	NEG	NEG	NEG	NEG	NEG	NEG	NEG	NEG	NEG	NEG	NEG	NEG	NEG	NEG	NEG	NEG	NEG	NEG	NEG	NEG	NEG	NEG	NEG	NEG	NEG	NEG
04V14909	CC22-MRSA-IV, Barnim/UK-EMRSA-15	POS	POS	AMB	NEG	NEG	POS	NEG	NEG	NEG	NEG	NEG	NEG	NEG	NEG	NEG	NEG	NEG	NEG	NEG	NEG	NEG	NEG	NEG	NEG	NEG	NEG	NEG	NEG	NEG	NEG	NEG	NEG	NEG	NEG	NEG	NEG	NEG	NEG	NEG	NEG	NEG	NEG	NEG	NEG	NEG
04V18654	CC22-MRSA-IV, Barnim/UK-EMRSA-15	POS	POS	POS	NEG	NEG	NEG	NEG	NEG	NEG	NEG	NEG	NEG	NEG	NEG	NEG	NEG	NEG	NEG	NEG	NEG	NEG	NEG	NEG	NEG	NEG	NEG	NEG	NEG	NEG	NEG	NEG	NEG	NEG	NEG	POS	NEG	NEG	NEG	NEG	NEG	NEG	NEG	NEG	NEG	NEG
04V18815	CC22-MRSA-IV, Barnim/UK-EMRSA-15	POS	POS	POS	NEG	NEG	POS	NEG	NEG	NEG	NEG	NEG	NEG	NEG	NEG	NEG	NEG	NEG	NEG	AMB	NEG	NEG	NEG	NEG	NEG	NEG	NEG	NEG	NEG	NEG	NEG	NEG	NEG	NEG	NEG	NEG	NEG	NEG	NEG	NEG	NEG	NEG	NEG	NEG	NEG	NEG
04V29721	CC22-MRSA-IV, Barnim/UK-EMRSA-15	POS	POS	NEG	NEG	NEG	AMB	NEG	NEG	NEG	NEG	NEG	NEG	NEG	NEG	NEG	NEG	NEG	NEG	NEG	NEG	NEG	NEG	NEG	NEG	NEG	NEG	NEG	NEG	NEG	NEG	NEG	NEG	NEG	NEG	NEG	NEG	NEG	NEG	NEG	NEG	NEG	NEG	NEG	NEG	NEG
04V32759	CC22-MRSA-IV, Barnim/UK-EMRSA-15	POS	POS	POS	NEG	NEG	NEG	NEG	NEG	NEG	NEG	NEG	NEG	NEG	NEG	NEG	NEG	NEG	NEG	NEG	NEG	NEG	NEG	NEG	NEG	NEG	NEG	NEG	NEG	NEG	NEG	NEG	NEG	NEG	NEG	NEG	NEG	NEG	NEG	NEG	NEG	NEG	NEG	NEG	NEG	NEG
04V32919	CC22-MRSA-IV, Barnim/UK-EMRSA-15	POS	POS	POS	NEG	NEG	NEG	NEG	NEG	NEG	NEG	NEG	NEG	AMB	NEG	NEG	NEG	NEG	NEG	NEG	AMB	NEG	NEG	NEG	NEG	NEG	NEG	NEG	NEG	NEG	NEG	NEG	NEG	NEG	NEG	NEG	NEG	NEG	NEG	NEG	NEG	NEG	AMB	NEG	NEG	NEG
04V33780	CC22-MRSA-IV, Barnim/UK-EMRSA-15	POS	POS	POS	NEG	NEG	POS	NEG	NEG	NEG	NEG	NEG	NEG	NEG	NEG	NEG	NEG	NEG	NEG	NEG	NEG	NEG	NEG	NEG	NEG	NEG	NEG	NEG	NEG	NEG	NEG	NEG	NEG	NEG	NEG	NEG	NEG	NEG	NEG	NEG	NEG	NEG	NEG	NEG	NEG	NEG
04V35396	CC22-MRSA-IV, Barnim/UK-EMRSA-15	POS	POS	POS	NEG	NEG	NEG	NEG	NEG	NEG	NEG	NEG	NEG	NEG	NEG	NEG	NEG	NEG	NEG	NEG	NEG	NEG	NEG	NEG	NEG	NEG	NEG	NEG	NEG	NEG	NEG	NEG	NEG	NEG	NEG	NEG	NEG	NEG	NEG	NEG	NEG	NEG	NEG	NEG	NEG	NEG
04V37107	CC22-MRSA-IV, Barnim/UK-EMRSA-15	POS	POS	POS	NEG	NEG	NEG	NEG	NEG	NEG	NEG	NEG	NEG	NEG	NEG	NEG	NEG	NEG	NEG	NEG	NEG	NEG	NEG	NEG	NEG	NEG	NEG	NEG	NEG	NEG	NEG	NEG	NEG	NEG	NEG	NEG	NEG	NEG	NEG	NEG	NEG	NEG	NEG	NEG	NEG	NEG
04V37518	CC22-MRSA-IV, Barnim/UK-EMRSA-15	POS	POS	AMB	NEG	NEG	NEG	NEG	NEG	NEG	NEG	NEG	NEG	NEG	NEG	NEG	NEG	NEG	NEG	NEG	NEG	NEG	NEG	NEG	NEG	NEG	NEG	NEG	NEG	NEG	NEG	NEG	NEG	NEG	NEG	NEG	NEG	NEG	NEG	NEG	NEG	NEG	NEG	NEG	NEG	NEG
04V38199	CC22-MRSA-IV, Barnim/UK-EMRSA-15	NEG	POS	POS	NEG		POS	NEG	NEG	NEG	NEG	NEG	NEG	NEG	NEG	NEG	NEG	NEG	NEG	NEG	NEG	NEG	NEG	NEG	NEG	NEG	NEG	NEG	NEG	NEG	NEG	NEG	NEG	NEG	NEG	NEG	NEG	NEG	NEG	NEG	NEG	NEG	NEG	NEG	NEG	NEG
04V38358	CC22-MRSA-IV, Barnim/UK-EMRSA-15	POS	POS	AMB	NEG	NEG	NEG	NEG	NEG	NEG	NEG	NEG	NEG	NEG	NEG	NEG	NEG	NEG	NEG	NEG	NEG	NEG	NEG	NEG	NEG	NEG	NEG	NEG	NEG	NEG	NEG	NEG	NEG	NEG	NEG	NEG	NEG	NEG	NEG	NEG	NEG	NEG	NEG	NEG	NEG	NEG
04V38902	CC22-MRSA-IV, Barnim/UK-EMRSA-15	POS	POS	AMB	NEG	NEG	POS	NEG	NEG	NEG	NEG	NEG	NEG	NEG	NEG	NEG	NEG	NEG	NEG	NEG	NEG	NEG	NEG	NEG	NEG	NEG	NEG	NEG	NEG	NEG	NEG	NEG	NEG	NEG	NEG	NEG	NEG	NEG	NEG	NEG	NEG	NEG	NEG	NEG	NEG	NEG
04V39203	CC22-MRSA-IV, Barnim/UK-EMRSA-15	POS	POS	POS	NEG	NEG	POS	NEG	NEG	NEG	NEG	NEG	NEG	NEG	NEG	NEG	NEG	NEG	NEG	NEG	NEG	NEG	NEG	NEG	POS	NEG	NEG	NEG	NEG	NEG	NEG	NEG	NEG	NEG	NEG	NEG	NEG	NEG	NEG	NEG	NEG	NEG	NEG	NEG	NEG	NEG
04V40423	CC22-MRSA-IV, Barnim/UK-EMRSA-15	POS	POS	AMB	NEG	NEG	AMB	NEG	NEG	NEG	NEG	NEG	NEG	NEG	NEG	NEG	NEG	NEG	NEG	NEG	NEG	NEG	NEG	NEG	NEG	NEG	NEG	NEG	NEG	NEG	NEG	NEG	NEG	NEG	NEG	NEG	NEG	NEG	NEG	NEG	NEG	NEG	NEG	NEG	NEG	NEG
04V08036	CC45-MRSA-IV [ACME+]	POS	POS	POS	NEG	NEG	NEG	NEG	NEG	NEG	NEG	NEG	NEG	AMB	NEG	NEG	NEG	NEG	POS	POS	NEG	NEG	NEG	NEG	NEG	NEG	NEG	NEG	NEG	NEG	NEG	NEG	NEG	NEG	NEG	NEG	NEG	NEG	NEG	NEG	NEG	NEG	POS	NEG	NEG	NEG
04U01119	CC45-MRSA-IV, Berlin EMRSA	POS	POS	POS	NEG	NEG	NEG	NEG	NEG	NEG	NEG	NEG	NEG	NEG	NEG	NEG	NEG	NEG	NEG	NEG	NEG	NEG	NEG	NEG	NEG	NEG	NEG	NEG	NEG	NEG	NEG	NEG	NEG	NEG	NEG	NEG	NEG	NEG	NEG	NEG	NEG	NEG	POS	NEG	NEG	NEG
04V01185	CC45-MRSA-IV, Berlin EMRSA	POS	POS	POS	NEG	NEG	NEG	NEG	NEG	NEG	NEG	NEG	NEG	NEG	NEG	NEG	NEG	NEG	POS	POS	NEG	NEG	NEG	NEG	NEG	NEG	NEG	NEG	NEG	NEG	NEG	NEG	NEG	NEG	NEG	NEG	NEG	NEG	NEG	NEG	NEG	NEG	POS	NEG	NEG	NEG
04V06276	CC45-MRSA-IV, Berlin EMRSA	POS	POS	POS	NEG	NEG	NEG	NEG	NEG	NEG	NEG	NEG	NEG	NEG	NEG	NEG	NEG	NEG	POS	POS	NEG	NEG	NEG	NEG	NEG	NEG	NEG	NEG	NEG	NEG	NEG	NEG	NEG	NEG	NEG	NEG	NEG	NEG	NEG	NEG	NEG	NEG	POS	NEG	NEG	NEG
04V06677	CC45-MRSA-IV, Berlin EMRSA	POS	POS	POS	NEG	NEG	POS	NEG	NEG	NEG	NEG	NEG	NEG	NEG	NEG	NEG	NEG	NEG	POS	POS	NEG	NEG	NEG	NEG	NEG	NEG	NEG	NEG	NEG	NEG	NEG	NEG	NEG	NEG	NEG	NEG	NEG	NEG	NEG	NEG	NEG	NEG	NEG	NEG	NEG	NEG
04V06688	CC45-MRSA-IV, Berlin EMRSA	POS	POS	POS	NEG	NEG	NEG	NEG	NEG	NEG	NEG	NEG	NEG	NEG	NEG	NEG	NEG	NEG	POS	POS	NEG	NEG	NEG	NEG	NEG	NEG	NEG	NEG	NEG	NEG	NEG	NEG	NEG	NEG	NEG	NEG	NEG	NEG	NEG	NEG	NEG	NEG	POS	NEG	NEG	NEG
04V14835	CC45-MRSA-IV, Berlin EMRSA	POS	POS	POS	NEG	NEG	NEG	NEG	NEG	NEG	NEG	NEG	NEG	NEG	NEG	NEG	NEG	NEG	POS	POS	NEG	NEG	NEG	NEG	NEG	NEG	NEG	NEG	NEG	NEG	NEG	NEG	NEG	NEG	NEG	POS	NEG	NEG	NEG	NEG	NEG	NEG	POS	NEG	NEG	NEG
04V22079	CC45-MRSA-IV, Berlin EMRSA	POS	POS	POS	NEG	NEG	POS	NEG	NEG	NEG	NEG	NEG	NEG	NEG	NEG	NEG	NEG	NEG	NEG	NEG	NEG	NEG	NEG	NEG	NEG	NEG	NEG	NEG	NEG	NEG	NEG	NEG	NEG	NEG	NEG	NEG	NEG	NEG	NEG	NEG	NEG	NEG	POS	NEG	NEG	NEG
04V29100	CC45-MRSA-IV, Berlin EMRSA	POS			NEG		NEG	NEG	NEG			NEG	NEG	NEG	NEG	NEG	NEG	NEG	POS	POS	NEG	NEG		NEG	NEG	NEG																		NEG	NEG	NEG
04V31370	CC45-MRSA-IV, Berlin EMRSA	POS	POS	POS	NEG	NEG	NEG	NEG	NEG	NEG	NEG	NEG	NEG	NEG	NEG	NEG	NEG	NEG	POS	POS	NEG	NEG	NEG	NEG	NEG	NEG	NEG	NEG	NEG	NEG	NEG	NEG	NEG	NEG	NEG	NEG	NEG	NEG	NEG	NEG	NEG	NEG	POS	NEG	NEG	NEG
04V38442	CC45-MRSA-IV, Berlin EMRSA	POS	POS	POS	NEG	NEG	NEG	NEG	NEG	NEG	NEG	NEG	NEG	NEG	NEG	NEG	NEG	NEG	POS	POS	NEG	NEG	NEG	NEG	NEG	NEG	NEG	NEG	NEG	NEG	NEG	NEG	NEG	NEG	NEG	NEG	NEG	NEG	NEG	NEG	NEG	NEG	POS	NEG	NEG	NEG
04V39012	CC45-MRSA-IV, Berlin EMRSA	POS	POS	POS	NEG	NEG	NEG	NEG	NEG	NEG	NEG	NEG	NEG	NEG	NEG	NEG	NEG	NEG	POS	POS	NEG	NEG	NEG	NEG	NEG	NEG	NEG	NEG	NEG	NEG	NEG	NEG	NEG	NEG	NEG	NEG	NEG	NEG	NEG	NEG	NEG	NEG	POS	NEG	NEG	NEG
04V39577	CC45-MRSA-IV, Berlin EMRSA	POS	POS	POS	NEG	NEG	POS	NEG	NEG	NEG	NEG	NEG	NEG	NEG	NEG	NEG	POS	POS	POS	POS	POS	NEG	NEG	NEG	NEG	NEG	NEG	NEG	NEG	NEG	NEG	NEG	NEG	NEG	NEG	NEG	NEG	NEG	NEG	NEG	NEG	NEG	POS	NEG	NEG	NEG
04V22872	CC152-MRSA-V [PVL+]	POS	POS	POS	NEG	NEG	NEG	NEG	NEG	NEG	NEG	NEG	NEG	AMB	NEG	NEG	POS	NEG	NEG	NEG	NEG	NEG	NEG	NEG	NEG	NEG	NEG	NEG	NEG	NEG	NEG	NEG	NEG	NEG	NEG	NEG	NEG	NEG	NEG	NEG	NEG	NEG	POS	NEG	NEG	NEG

ISOLATE:	STRAIN	VIRULENCE : TOX.SCHOCK.TOXIN			VIRULENCE : ENTEROTOXINS					
		toxic shock syndrome toxin 1			enterotoxin A	enterotoxin A, allele from strain 320E	enterotoxin A, allele from strain N315 = entero-toxin P	enterotoxin B	enterotoxin C	enterotoxin D
		tst1 (consensus)	tst1 ("human" allele)	tst1 ("bovine" allele)	sea	sea (320E)	sea (N315) / sep	seb	sec	sed
**2003**										
03F01030	CC5/ST228-MRSA, South German EMRSA (truncated SCCmec element)	NEG	NEG	NEG	NEG	NEG	NEG	NEG	NEG	NEG
03V05419	CC5/ST228-MRSA-I, South German EMRSA	NEG	NEG	NEG	POS	NEG	NEG	NEG	NEG	NEG
03V08986	CC5/ST228-MRSA-I, South German EMRSA	NEG	NEG	NEG	POS	NEG	NEG	NEG	NEG	NEG
03V39123	CC5/ST228-MRSA-I, South German EMRSA		NEG	NEG	POS	NEG	NEG	NEG	NEG	NEG
03B05186	CC5-MRSA-II, Rhine-Hesse EMRSA/UK-EMRSA-3	NEG	NEG	NEG	NEG	NEG	POS	NEG	NEG	POS
03U00003	CC5-MRSA-II, Rhine-Hesse EMRSA/UK-EMRSA-3	NEG	NEG	NEG	NEG	NEG	POS	NEG	NEG	POS
03V01438	CC5-MRSA-II, Rhine-Hesse EMRSA/UK-EMRSA-3	NEG	NEG	NEG	NEG	NEG	POS	NEG	NEG	POS
03V05075	CC5-MRSA-II, Rhine-Hesse EMRSA/UK-EMRSA-3	NEG	NEG	NEG	NEG	NEG	POS	NEG	NEG	POS
03V11014	CC5-MRSA-II, Rhine-Hesse EMRSA/UK-EMRSA-3	NEG	NEG	NEG	NEG	NEG	POS	NEG	NEG	POS
03V12917	CC5-MRSA-II, Rhine-Hesse EMRSA/UK-EMRSA-3	NEG	NEG	NEG	NEG	NEG	POS	NEG	NEG	POS
03V14467	CC5-MRSA-II, Rhine-Hesse EMRSA/UK-EMRSA-3	NEG	NEG	NEG	NEG	NEG	POS	NEG	NEG	POS
03V19357	CC5-MRSA-II, Rhine-Hesse EMRSA/UK-EMRSA-3	NEG	NEG	NEG	NEG	NEG	POS	NEG	NEG	POS
03V20186	CC5-MRSA-II, Rhine-Hesse EMRSA/UK-EMRSA-3	NEG	NEG	NEG	NEG	NEG	POS	NEG	NEG	POS
03V25349	CC5-MRSA-II, Rhine-Hesse EMRSA/UK-EMRSA-3	NEG	NEG	NEG	NEG	NEG	POS	NEG	NEG	POS
03V28948	CC5-MRSA-II, Rhine-Hesse EMRSA/UK-EMRSA-3	NEG	NEG	NEG	NEG	NEG	POS	NEG	NEG	POS
03V30841	CC5-MRSA-II, Rhine-Hesse EMRSA/UK-EMRSA-3		NEG	NEG	NEG	NEG	POS	NEG	NEG	POS
03V32896	CC5-MRSA-II, Rhine-Hesse EMRSA/UK-EMRSA-3	NEG	NEG	NEG	NEG	NEG	POS	NEG	NEG	POS
03V33415	CC5-MRSA-II, Rhine-Hesse EMRSA/UK-EMRSA-3	NEG	NEG	NEG	NEG	NEG	POS	NEG	NEG	NEG
03V34628	CC5-MRSA-II, Rhine-Hesse EMRSA/UK-EMRSA-3	NEG	NEG	NEG	NEG	NEG	POS	NEG	NEG	POS
03V37078	CC5-MRSA-II, Rhine-Hesse EMRSA/UK-EMRSA-3	NEG	NEG	NEG	NEG	NEG	POS	NEG	NEG	NEG
03V41645	CC5-MRSA-II, Rhine-Hesse EMRSA/UK-EMRSA-3	NEG	NEG	NEG	NEG	NEG	POS	NEG	NEG	POS
03V03012	CC5-MRSA-IV, Paediatric clone	NEG	NEG	NEG	NEG	NEG	NEG	NEG	NEG	NEG
03V02863	CC8/ST247-MRSA-I, North German/Iberian EMRSA	NEG	NEG	NEG	POS	NEG	NEG	NEG	NEG	NEG
03V14882	CC8/ST254-MRSA-(atypical SCCmec), Hannover EMRSA	NEG	NEG	NEG	NEG	NEG	NEG	POS	NEG	NEG
03B00404	CC22-MRSA-IV, Barnim/UK-EMRSA-15	NEG	NEG	NEG	NEG	NEG	NEG	NEG	NEG	NEG
03U00892	CC22-MRSA-IV, Barnim/UK-EMRSA-15		NEG	NEG	NEG	NEG	NEG	NEG	NEG	NEG
03V01422	CC22-MRSA-IV, Barnim/UK-EMRSA-15	NEG	NEG	NEG	NEG	NEG	NEG	NEG	POS	NEG
03V04424	CC22-MRSA-IV, Barnim/UK-EMRSA-15	NEG	NEG	NEG	NEG	NEG	NEG	NEG	NEG	NEG
03V05690	CC22-MRSA-IV, Barnim/UK-EMRSA-15	NEG	NEG	NEG	NEG	NEG	NEG	NEG	NEG	NEG
03V07912	CC22-MRSA-IV, Barnim/UK-EMRSA-15	NEG	NEG	NEG	NEG	NEG	NEG	NEG	NEG	NEG
03V23798	CC22-MRSA-IV, Barnim/UK-EMRSA-15	NEG	NEG	NEG	NEG	NEG	NEG	NEG	NEG	NEG
03V25914	CC22-MRSA-IV, Barnim/UK-EMRSA-15	NEG	NEG	NEG	NEG	NEG	NEG	NEG	NEG	NEG
03V29751	CC22-MRSA-IV, Barnim/UK-EMRSA-15	NEG	NEG	NEG	NEG	NEG	NEG	NEG	NEG	NEG
03V30201	CC22-MRSA-IV, Barnim/UK-EMRSA-15	NEG	NEG	NEG	NEG	NEG	NEG	NEG	POS	NEG
03B01781	CC45-MRSA-IV, Berlin EMRSA	NEG	NEG	NEG	NEG	NEG	NEG	NEG	NEG	NEG
03U05014	CC45-MRSA-IV, Berlin EMRSA	NEG	NEG	NEG	NEG	NEG	NEG	NEG	NEG	NEG
03U11106	CC45-MRSA-IV, Berlin EMRSA	NEG	NEG	NEG	NEG	NEG	NEG	NEG	POS	NEG
03V00742	CC45-MRSA-IV, Berlin EMRSA	NEG	NEG	NEG	NEG	NEG	NEG	NEG	NEG	NEG
03V01902	CC45-MRSA-IV, Berlin EMRSA	NEG	NEG	NEG	NEG	NEG	NEG	NEG	NEG	NEG
03V02256	CC45-MRSA-IV, Berlin EMRSA	NEG	NEG	NEG	NEG	NEG	NEG	NEG	NEG	NEG
03V02262	CC45-MRSA-IV, Berlin EMRSA	NEG	NEG	NEG	NEG	NEG	NEG	NEG	NEG	NEG
03V04701	CC45-MRSA-IV, Berlin EMRSA	NEG	NEG	NEG	NEG	NEG	NEG	NEG	NEG	NEG
03V05042	CC45-MRSA-IV, Berlin EMRSA	NEG	NEG	NEG	NEG	NEG	NEG	NEG	NEG	NEG
03V05377	CC45-MRSA-IV, Berlin EMRSA	NEG	NEG	NEG	NEG	NEG	NEG	NEG	NEG	NEG
03V05507	CC45-MRSA-IV, Berlin EMRSA	NEG	NEG	NEG	NEG	NEG	NEG	NEG	NEG	NEG
03V08214	CC45-MRSA-IV, Berlin EMRSA	NEG	NEG	NEG	NEG	NEG	NEG	NEG	NEG	NEG
03V08525	CC45-MRSA-IV, Berlin EMRSA	NEG	NEG	NEG	NEG	NEG	NEG	NEG	NEG	NEG
03V11910	CC45-MRSA-IV, Berlin EMRSA	NEG	NEG	NEG	NEG	NEG	NEG	NEG	NEG	NEG
03V14481	CC45-MRSA-IV, Berlin EMRSA	NEG	NEG	NEG	NEG	NEG	NEG	NEG	NEG	NEG
03V15082	CC45-MRSA-IV, Berlin EMRSA	NEG	NEG	NEG	NEG	NEG	NEG	NEG	NEG	NEG
03V17508	CC45-MRSA-IV, Berlin EMRSA	NEG	NEG	NEG	NEG	NEG	NEG	NEG	NEG	NEG
03V20150	CC45-MRSA-IV, Berlin EMRSA	NEG	NEG	NEG	NEG	NEG	NEG	NEG	NEG	NEG
03V22437	CC45-MRSA-IV, Berlin EMRSA	NEG	NEG	NEG	NEG	NEG	NEG	NEG	NEG	NEG
03V24471	CC45-MRSA-IV, Berlin EMRSA	NEG	NEG	NEG	NEG	NEG	NEG	NEG	NEG	NEG
03V28070	CC45-MRSA-IV, Berlin EMRSA	NEG	NEG	NEG	NEG	NEG	NEG	NEG	NEG	NEG
03V28448	CC45-MRSA-IV, Berlin EMRSA	NEG	NEG	NEG	NEG	NEG	NEG	NEG	NEG	NEG
03V34589	CC45-MRSA-IV, Berlin EMRSA	NEG	NEG	NEG	NEG	NEG	NEG	NEG	NEG	NEG
03V39774	CC45-MRSA-IV, Berlin EMRSA	NEG	NEG	NEG	NEG	NEG	NEG	NEG	NEG	NEG
**2004**										
04B05423	CC5-MRSA-II, Rhine-Hesse EMRSA/UK-EMRSA-3	NEG	NEG	NEG	NEG	NEG	POS	NEG	NEG	NEG
04U02171	CC5-MRSA-II, Rhine-Hesse EMRSA/UK-EMRSA-3	NEG	NEG	NEG	NEG	NEG	POS	NEG	NEG	POS
04U03682	CC5-MRSA-II, Rhine-Hesse EMRSA/UK-EMRSA-3	NEG	NEG	NEG	NEG	NEG	NEG	NEG	NEG	POS
04U06618	CC5-MRSA-II, Rhine-Hesse EMRSA/UK-EMRSA-3	NEG	NEG	NEG	NEG	NEG	POS	NEG	NEG	POS
04U12578	CC5-MRSA-II, Rhine-Hesse EMRSA/UK-EMRSA-3	NEG	NEG	NEG	NEG	NEG	POS	NEG	NEG	POS
04V02465	CC5-MRSA-II, Rhine-Hesse EMRSA/UK-EMRSA-3	NEG	NEG	NEG	NEG	NEG	POS	NEG	NEG	POS
04V03642	CC5-MRSA-II, Rhine-Hesse EMRSA/UK-EMRSA-3	NEG	NEG	NEG	NEG	NEG	POS	NEG	NEG	POS
04V07657	CC5-MRSA-II, Rhine-Hesse EMRSA/UK-EMRSA-3	NEG	NEG	NEG	NEG	NEG	POS	NEG	NEG	POS
04V12223	CC5-MRSA-II, Rhine-Hesse EMRSA/UK-EMRSA-3	NEG	NEG	NEG	NEG	NEG	POS	NEG	NEG	POS
04V12529	CC5-MRSA-II, Rhine-Hesse EMRSA/UK-EMRSA-3	NEG	NEG	NEG	NEG	NEG	POS	NEG	NEG	POS
04V13633	CC5-MRSA-II, Rhine-Hesse EMRSA/UK-EMRSA-3	NEG	NEG	NEG	NEG	NEG	POS	NEG	NEG	POS
04V16499	CC5-MRSA-II, Rhine-Hesse EMRSA/UK-EMRSA-3	NEG	NEG	NEG	NEG	NEG	POS	NEG	NEG	POS
04V17015	CC5-MRSA-II, Rhine-Hesse EMRSA/UK-EMRSA-3	NEG	NEG	NEG	NEG	NEG	POS	NEG	NEG	POS
04V20684	CC5-MRSA-II, Rhine-Hesse EMRSA/UK-EMRSA-3		NEG	NEG	NEG	NEG	POS	NEG	NEG	POS
04V24615	CC5-MRSA-II, Rhine-Hesse EMRSA/UK-EMRSA-3	NEG	NEG	NEG	NEG	NEG	POS	NEG	NEG	POS
04V24901	CC5-MRSA-II, Rhine-Hesse EMRSA/UK-EMRSA-3	NEG	NEG	NEG	NEG	NEG	POS	NEG	NEG	NEG
04V25081	CC5-MRSA-II, Rhine-Hesse EMRSA/UK-EMRSA-3	NEG	NEG	NEG	NEG	NEG	POS	NEG	NEG	POS
04V27293	CC5-MRSA-II, Rhine-Hesse EMRSA/UK-EMRSA-3	NEG	NEG	NEG	NEG	NEG	POS	NEG	NEG	POS
04V27311	CC5-MRSA-II, Rhine-Hesse EMRSA/UK-EMRSA-3	NEG	NEG	NEG	NEG	NEG	POS	NEG	NEG	POS
04V33459	CC5-MRSA-II, Rhine-Hesse EMRSA/UK-EMRSA-3	NEG	NEG	NEG	NEG	NEG	POS	NEG	NEG	POS
04V39909	CC5-MRSA-IV&VI	NEG	NEG	NEG	NEG	NEG	NEG	NEG	NEG	NEG
04U02421	CC5-MRSA-IV, Paediatric clone	NEG	NEG	NEG	NEG	NEG	POS	POS	NEG	POS
04V09807	CC80-MRSA-IV [PVL+], European caMRSA Clone		NEG	NEG	NEG	NEG	NEG	NEG	NEG	NEG
04V16073	CC80-MRSA-IV [PVL+], European caMRSA Clone	NEG	NEG	NEG	NEG	NEG	NEG	NEG	NEG	NEG
04V23548	CC80-MRSA-IV [PVL+], European caMRSA Clone		NEG	NEG	NEG	NEG	NEG	NEG	NEG	NEG
04V03289	CC22-MRSA-IV, Barnim/UK-EMRSA-15		NEG	NEG	NEG	NEG	NEG	NEG	NEG	NEG
04V03847	CC22-MRSA-IV, Barnim/UK-EMRSA-15		NEG	NEG	NEG	NEG	NEG	NEG	NEG	NEG
04V04595	CC22-MRSA-IV, Barnim/UK-EMRSA-15	NEG	NEG	NEG	NEG	NEG	NEG	NEG	NEG	NEG
04V05029	CC22-MRSA-IV, Barnim/UK-EMRSA-15	NEG	NEG	NEG	NEG	NEG	NEG	NEG	AMB	NEG
04V07875	CC22-MRSA-IV, Barnim/UK-EMRSA-15	NEG	NEG	NEG	NEG	NEG	NEG	NEG	NEG	NEG
04V14909	CC22-MRSA-IV, Barnim/UK-EMRSA-15	NEG	NEG	NEG	NEG	NEG	NEG	NEG	NEG	NEG
04V18654	CC22-MRSA-IV, Barnim/UK-EMRSA-15	NEG	NEG	NEG	NEG	NEG	NEG	NEG	NEG	NEG
04V18815	CC22-MRSA-IV, Barnim/UK-EMRSA-15	NEG	AMB	NEG	NEG	NEG	NEG	NEG	NEG	NEG
04V29721	CC22-MRSA-IV, Barnim/UK-EMRSA-15	NEG	NEG	NEG	NEG	NEG	NEG	NEG	NEG	NEG
04V32759	CC22-MRSA-IV, Barnim/UK-EMRSA-15	NEG	NEG	NEG	NEG	NEG	NEG	NEG	NEG	NEG
04V32919	CC22-MRSA-IV, Barnim/UK-EMRSA-15	NEG	NEG	NEG	NEG	NEG	NEG	AMB	POS	NEG
04V33780	CC22-MRSA-IV, Barnim/UK-EMRSA-15	NEG	NEG	NEG	NEG	NEG	NEG	NEG	NEG	NEG
04V35396	CC22-MRSA-IV, Barnim/UK-EMRSA-15	NEG	NEG	NEG	NEG	NEG	NEG	AMB	POS	NEG
04V37107	CC22-MRSA-IV, Barnim/UK-EMRSA-15	NEG	NEG	NEG	NEG	NEG	NEG	NEG	NEG	NEG
04V37518	CC22-MRSA-IV, Barnim/UK-EMRSA-15	NEG	NEG	NEG	NEG	NEG	NEG	NEG	NEG	NEG
04V38199	CC22-MRSA-IV, Barnim/UK-EMRSA-15	NEG	NEG	NEG	NEG	NEG	NEG	NEG	POS	NEG
04V38358	CC22-MRSA-IV, Barnim/UK-EMRSA-15	NEG	NEG	NEG	NEG	NEG	NEG	NEG	NEG	NEG
04V38902	CC22-MRSA-IV, Barnim/UK-EMRSA-15	NEG	NEG	NEG	NEG	NEG	NEG	NEG	NEG	NEG
04V39203	CC22-MRSA-IV, Barnim/UK-EMRSA-15	NEG	NEG	NEG	NEG	NEG	NEG	NEG	NEG	NEG
04V40423	CC22-MRSA-IV, Barnim/UK-EMRSA-15	NEG	NEG	NEG	NEG	NEG	NEG	NEG	NEG	NEG
04V08036	CC45-MRSA-IV [ACME+]	NEG	NEG	NEG	NEG	NEG	NEG	NEG	NEG	NEG
04U01119	CC45-MRSA-IV, Berlin EMRSA	NEG	NEG	NEG	NEG	NEG	NEG	NEG	NEG	NEG
04V01185	CC45-MRSA-IV, Berlin EMRSA	NEG	NEG	NEG	NEG	NEG	NEG	NEG	NEG	NEG
04V06276	CC45-MRSA-IV, Berlin EMRSA	NEG	NEG	NEG	NEG	NEG	NEG	NEG	NEG	NEG
04V06677	CC45-MRSA-IV, Berlin EMRSA	NEG	NEG	NEG	NEG	NEG	NEG	NEG	NEG	NEG
04V06688	CC45-MRSA-IV, Berlin EMRSA	NEG	NEG	NEG	NEG	NEG	NEG	NEG	NEG	NEG
04V14835	CC45-MRSA-IV, Berlin EMRSA	NEG	NEG	NEG	NEG	NEG	NEG	NEG	NEG	NEG
04V22079	CC45-MRSA-IV, Berlin EMRSA	NEG	NEG	NEG	NEG	NEG	NEG	NEG	POS	NEG
04V29100	CC45-MRSA-IV, Berlin EMRSA		NEG	NEG	NEG	NEG	NEG	NEG	NEG	NEG
04V31370	CC45-MRSA-IV, Berlin EMRSA	NEG	NEG	NEG	NEG	NEG	NEG	NEG	NEG	NEG
04V38442	CC45-MRSA-IV, Berlin EMRSA	NEG	NEG	NEG	NEG	NEG	NEG	NEG	NEG	NEG
04V39012	CC45-MRSA-IV, Berlin EMRSA	NEG	NEG	NEG	NEG	NEG	NEG	NEG	NEG	NEG
04V39577	CC45-MRSA-IV, Berlin EMRSA	NEG	NEG	NEG	NEG	NEG	NEG	NEG	NEG	NEG
04V22872	CC152-MRSA-V [PVL+]	NEG	NEG	NEG	NEG	NEG	NEG	NEG	NEG	NEG

ISOLATE:	VIRULENCE : ENTEROTOXINS														
	enterotoxin E	enterotoxin G	enterotoxin H	enterotoxin I	enterotoxin J	enterotoxin K	enterotoxin L	enterotoxin M	enterotoxin N		enterotoxin O	enterotoxin Q	enterotoxin R	enterotoxin U	enterotoxin-like protein ORF CM14
	sef	seg	seh	sei	sej	sek	sel	selm	seln (consensus)	seln (other than RF122)	selo	seq	ser	selu	ORF CM14
**2003**															
03F01030	NEG	POS	NEG	POS	NEG	NEG	NEG	POS	POS	POS	POS	NEG	NEG	POS	NEG
03V05419	NEG	NEG	NEG	NEG	NEG	NEG	NEG	NEG	NEG	NEG	POS	NEG	NEG	NEG	NEG
03V08986	NEG	POS	NEG	POS	NEG	NEG	NEG	POS	POS	POS	POS	NEG	NEG	POS	NEG
03V39123	NEG	POS	NEG	POS	NEG		NEG	POS		POS	POS		NEG		
03B05186	NEG	POS	NEG	POS	POS	NEG	NEG	POS	POS	POS	POS	NEG	POS	POS	NEG
03U00003	NEG	POS	NEG	POS	POS	NEG	NEG	POS	POS	POS	POS	NEG	POS	POS	NEG
03V01438	NEG	POS	NEG	POS	POS	NEG	NEG	POS	POS	POS	POS	NEG	POS	POS	NEG
03V05075	NEG	POS	NEG	POS	POS	NEG	NEG	POS	POS	POS	POS	NEG	POS	POS	NEG
03V11014	NEG	POS	NEG	POS	POS	NEG	NEG	POS	POS	POS	POS	NEG	POS	POS	NEG
03V12917	NEG	POS	NEG	POS	POS	NEG	NEG	POS	POS	POS	POS	NEG	POS	POS	NEG
03V14467	NEG	POS	NEG	POS	POS	NEG	NEG	POS	POS	POS	POS	NEG	POS	POS	NEG
03V19357	NEG	POS	NEG	POS	POS	NEG	NEG	POS	POS	POS	POS	NEG	POS	POS	NEG
03V20186	NEG	POS	NEG	POS	POS	NEG	NEG	POS	POS	POS	POS	NEG	POS	POS	NEG
03V25349	NEG	POS	NEG	POS	POS	NEG	NEG	POS	POS	POS	POS	NEG	POS	POS	NEG
03V28948	NEG	POS	NEG	POS	POS	NEG	NEG	POS	POS	POS	POS	NEG	POS	POS	NEG
03V30841	NEG	POS	NEG	POS	POS		NEG	POS		POS	POS		POS		
03V32896	NEG	POS	NEG	POS	POS	NEG	NEG	POS	POS	POS	POS	NEG	NEG	POS	NEG
03V33415	NEG	POS	NEG	POS	NEG	NEG	NEG	POS	POS	POS	POS	NEG	NEG	POS	NEG
03V34628	NEG	POS	NEG	POS	POS	NEG	NEG	POS	POS	POS	POS	NEG	POS	POS	NEG
03V37078	NEG	AMB	NEG	POS	NEG	NEG	NEG	POS	POS	POS	POS	NEG	NEG	POS	NEG
03V41645	NEG	POS	NEG	POS	POS	NEG	NEG	POS	POS	POS	POS	NEG	POS	POS	NEG
03V03012	NEG	POS	NEG	POS	NEG	NEG	NEG	POS	POS	POS	POS	NEG	NEG	POS	NEG
03V02863	NEG	NEG	NEG	NEG	NEG	NEG	NEG	NEG	NEG	NEG	NEG	NEG	NEG	NEG	NEG
03V14882	NEG	NEG	NEG	NEG	NEG	POS	NEG	NEG	NEG	NEG	NEG	POS	NEG	NEG	NEG
03B00404	NEG	POS	NEG	POS	NEG	NEG	NEG	POS	POS	POS	POS	NEG	NEG	POS	NEG
03U00892	NEG	POS	NEG	POS	NEG		NEG	POS		POS	POS		NEG		
03V01422	NEG	POS	NEG	POS	NEG	NEG	POS	POS	POS	POS	POS	NEG	NEG	POS	NEG
03V04424	NEG	POS	NEG	POS	NEG	NEG	NEG	POS	POS	POS	POS	NEG	NEG	POS	NEG
03V05690	NEG	POS	NEG	POS	NEG	NEG	NEG	POS	POS	POS	POS	NEG	NEG	POS	NEG
03V07912	NEG	POS	NEG	POS	NEG	NEG	NEG	POS	POS	POS	POS	NEG	NEG	POS	NEG
03V23798	NEG	POS	NEG	POS	NEG	NEG	NEG	POS	POS	POS	POS	NEG	NEG	POS	NEG
03V25914	NEG	POS	NEG	POS	NEG	NEG	NEG	POS	POS	POS	POS	NEG	NEG	POS	NEG
03V29751	NEG	POS	NEG	POS	NEG	NEG	NEG	POS	POS	POS	POS	NEG	NEG	POS	NEG
03V30201	NEG	POS	NEG	POS	NEG	NEG	POS	POS	POS	POS	POS	NEG	NEG	POS	NEG
03B01781	NEG	NEG	NEG	POS	NEG	NEG	NEG	POS	POS	POS	POS	NEG	NEG	POS	NEG
03U05014	NEG	POS	NEG	POS	NEG	NEG	NEG	POS	POS	POS	POS	NEG	NEG	POS	NEG
03U11106	NEG	POS	NEG	POS	NEG	NEG	POS	POS	POS	POS	POS	NEG	NEG	POS	NEG
03V00742	NEG	NEG	NEG	POS	NEG	NEG	NEG	POS	POS	POS	POS	NEG	NEG	POS	NEG
03V01902	NEG	POS	NEG	POS	NEG	NEG	NEG	POS	POS	POS	POS	NEG	NEG	POS	NEG
03V02256	NEG	POS	NEG	POS	NEG	NEG	NEG	POS	POS	POS	POS	NEG	NEG	POS	NEG
03V02262	NEG	NEG	NEG	POS	NEG	NEG	NEG	POS	POS	POS	POS	NEG	NEG	POS	NEG
03V04701	NEG	POS	NEG	POS	NEG	NEG	NEG	POS	POS	POS	POS	NEG	NEG	POS	NEG
03V05042	NEG	NEG	NEG	POS	NEG	NEG	NEG	POS	POS	POS	POS	NEG	NEG	POS	NEG
03V05377	NEG	POS	NEG	POS	NEG	NEG	NEG	POS	POS	POS	POS	NEG	NEG	POS	NEG
03V05507	NEG	NEG	NEG	POS	NEG	NEG	NEG	POS	POS	POS	POS	NEG	NEG	POS	NEG
03V08214	NEG	POS	NEG	POS	NEG	NEG	NEG	POS	POS	POS	POS	NEG	NEG	POS	NEG
03V08525	NEG	NEG	NEG	POS	NEG	NEG	NEG	POS	POS	POS	POS	NEG	NEG	POS	NEG
03V11910	NEG	NEG	NEG	POS	NEG	NEG	NEG	POS	POS	POS	POS	NEG	NEG	POS	NEG
03V14481	NEG	POS	NEG	POS	NEG	NEG	NEG	POS	POS	POS	POS	NEG	NEG	POS	NEG
03V15082	NEG	NEG	NEG	POS	NEG	NEG	NEG	POS	POS	POS	POS	NEG	NEG	POS	NEG
03V17508	NEG	NEG	NEG	POS	NEG	NEG	NEG	POS	POS	POS	POS	NEG	NEG	POS	NEG
03V20150	NEG	NEG	NEG	POS	NEG	NEG	NEG	POS	POS	POS	POS	NEG	NEG	POS	NEG
03V22437	NEG	POS	NEG	POS	NEG	NEG	NEG	POS	POS	POS	POS	NEG	NEG	POS	NEG
03V24471	NEG	POS	NEG	POS	NEG	NEG	NEG	POS	POS	POS	POS	NEG	NEG	POS	NEG
03V28070	NEG	POS	NEG	POS	NEG	NEG	NEG	POS	POS	POS	AMB	NEG	NEG	POS	NEG
03V28448	NEG	POS	NEG	POS	NEG	NEG	NEG	POS	POS	POS	POS	NEG	NEG	POS	NEG
03V34589	NEG	POS	NEG	POS	NEG	NEG	NEG	POS	POS	POS	POS	NEG	NEG	POS	NEG
03V39774	NEG	NEG	NEG	POS	NEG	NEG	NEG	POS	POS	POS	POS	NEG	NEG	POS	NEG
**2004**															
04B05423	NEG	POS	NEG	POS	NEG	NEG	NEG	POS	POS	POS	POS	NEG	NEG	POS	NEG
04U02171	NEG	POS	NEG	POS	POS	NEG	NEG	POS	POS	POS	POS	NEG	POS	POS	NEG
04U03682	NEG	POS	NEG	POS	POS	NEG	NEG	POS	POS	POS	POS	NEG	POS	POS	NEG
04U06618	NEG	POS	NEG	POS	POS	NEG	NEG	POS	POS	POS	POS	NEG	POS	POS	NEG
04U12578	NEG	POS	NEG	POS	POS	NEG	NEG	POS	POS	POS	POS	NEG	POS	POS	NEG
04V02465	NEG	POS	NEG	POS	POS	NEG	NEG	POS	POS	POS	POS	NEG	POS	POS	NEG
04V03642	NEG	POS	NEG	POS	POS	NEG	NEG	POS	POS	POS	POS	NEG	POS	POS	NEG
04V07657	NEG	POS	NEG	POS	POS	NEG	NEG	POS	POS	POS	POS	NEG	POS	POS	NEG
04V12223	NEG	POS	NEG	POS	POS	NEG	NEG	POS	POS	POS	POS	NEG	POS	POS	NEG
04V12529	NEG	POS	NEG	POS	POS	NEG	NEG	POS	POS	POS	POS	NEG	POS	POS	NEG
04V13633	NEG	POS	NEG	POS	POS	NEG	NEG	POS	POS	POS	POS	NEG	POS	POS	NEG
04V16499	NEG	POS	NEG	POS	POS	NEG	NEG	POS	POS	POS	POS	NEG	POS	POS	NEG
04V17015	NEG	POS	NEG	POS	POS	NEG	NEG	POS	POS	POS	POS	NEG	POS	POS	NEG
04V20684	NEG	POS	NEG	POS	POS		NEG	POS		POS	POS		POS		
04V24615	NEG	POS	NEG	POS	POS	NEG	NEG	POS	POS	POS	POS	NEG	POS	POS	NEG
04V24901	NEG	POS	NEG	POS	NEG	NEG	NEG	POS	POS	POS	POS	NEG	NEG	POS	NEG
04V25081	NEG	POS	NEG	POS	POS	NEG	NEG	POS	POS	POS	POS	NEG	POS	POS	NEG
04V27293	NEG	POS	NEG	POS	POS	NEG	NEG	POS	POS	POS	POS	NEG	POS	POS	NEG
04V27311	NEG	POS	NEG	POS	POS	NEG	NEG	POS	POS	POS	AMB	NEG	POS	POS	NEG
04V33459	NEG	POS	NEG	POS	POS	NEG	NEG	POS	POS	POS	POS	NEG	POS	POS	NEG
04V39909	NEG	POS	NEG	POS	NEG	NEG	NEG	POS	POS	POS	POS	NEG	NEG	POS	NEG
04U02421	NEG	POS	NEG	POS	POS	NEG	NEG	POS	NEG	POS	POS	NEG	POS	POS	NEG
04V09807	NEG	NEG	NEG	NEG	NEG		NEG	NEG		NEG	NEG		NEG		
04V16073	NEG	NEG	NEG	NEG	NEG	NEG	NEG	NEG	NEG	NEG	NEG	NEG	NEG	NEG	NEG
04V23548	NEG	NEG	NEG	NEG	NEG		NEG	NEG		NEG	NEG		NEG		
04V03289	NEG	POS	NEG	POS	NEG		NEG	POS		POS	POS		NEG		
04V03847	NEG	POS	NEG	POS	NEG		NEG	POS		POS	POS		NEG		
04V04595	NEG	POS	NEG	POS	NEG	NEG	NEG	POS	POS	POS	POS	NEG	NEG	POS	NEG
04V05029	NEG	POS	NEG	POS	NEG	NEG	AMB	POS	POS	POS	AMB	NEG	NEG	POS	NEG
04V07875	NEG	POS	NEG	AMB	NEG	NEG	NEG	POS	POS	AMB	NEG	NEG	NEG	POS	NEG
04V14909	NEG	POS	NEG	POS	NEG	NEG	NEG	POS	POS	POS	POS	NEG	NEG	POS	NEG
04V18654	NEG	POS	NEG	POS	NEG	NEG	NEG	POS	POS	POS	POS	NEG	NEG	POS	NEG
04V18815	NEG	POS	NEG	POS	NEG	NEG	NEG	POS	POS	POS	POS	NEG	NEG	POS	NEG
04V29721	NEG	POS	NEG	POS	NEG	NEG	NEG	POS	POS	AMB	AMB	NEG	NEG	POS	NEG
04V32759	NEG	POS	NEG	POS	NEG	NEG	NEG	POS	POS	POS	POS	NEG	NEG	POS	NEG
04V32919	NEG	POS	NEG	POS	NEG	NEG	POS	POS	POS	POS	POS	NEG	NEG	POS	NEG
04V33780	NEG	POS	NEG	POS	NEG	NEG	NEG	POS	POS	POS	POS	NEG	NEG	POS	NEG
04V35396	NEG	POS	NEG	POS	NEG	NEG	POS	POS	POS	POS	POS	NEG	NEG	POS	NEG
04V37107	NEG	POS	NEG	POS	NEG	NEG	NEG	POS	POS	POS	POS	NEG	NEG	POS	NEG
04V37518	NEG	POS	NEG	POS	NEG	NEG	NEG	POS	POS	POS	POS	NEG	NEG	POS	NEG
04V38199	NEG	POS	NEG	POS	NEG	NEG	POS	POS	AMB	POS	NEG	NEG	NEG	POS	NEG
04V38358	NEG	POS	NEG	POS	NEG	NEG	NEG	POS	POS	AMB	AMB	NEG	NEG	POS	NEG
04V38902	NEG	POS	NEG	POS	NEG	NEG	NEG	POS	POS	NEG	AMB	NEG	NEG	POS	NEG
04V39203	NEG	POS	NEG	POS	NEG	NEG	NEG	POS	POS	POS	POS	NEG	NEG	POS	NEG
04V40423	NEG	POS	NEG	POS	NEG	NEG	NEG	POS	POS	AMB	NEG	NEG	NEG	POS	NEG
04V08036	NEG	NEG	NEG	POS	NEG	NEG	NEG	POS	POS	POS	POS	NEG	NEG	POS	NEG
04U01119	NEG	NEG	NEG	POS	NEG	NEG	NEG	POS	POS	POS	POS	NEG	NEG	POS	NEG
04V01185	NEG	POS	NEG	POS	NEG	NEG	NEG	POS	POS	POS	POS	NEG	NEG	POS	NEG
04V06276	NEG	POS	NEG	POS	NEG	NEG	NEG	POS	POS	POS	POS	NEG	NEG	POS	NEG
04V06677	NEG	POS	NEG	POS	NEG	NEG	NEG	POS	POS	POS	POS	NEG	NEG	POS	NEG
04V06688	NEG	POS	NEG	POS	NEG	NEG	NEG	POS	POS	POS	POS	NEG	NEG	POS	NEG
04V14835	NEG	POS	NEG	POS	NEG	NEG	NEG	POS	POS	POS	POS	NEG	NEG	POS	NEG
04V22079	NEG	POS	NEG	POS	NEG	NEG	POS	POS	POS	POS	POS	NEG	NEG	POS	NEG
04V29100	NEG	NEG	NEG	POS	NEG		NEG	POS		POS	POS		NEG		
04V31370	NEG	POS	NEG	POS	NEG	NEG	NEG	POS	POS	POS	POS	NEG	NEG	POS	NEG
04V38442	NEG	POS	NEG	POS	NEG	NEG	NEG	POS	POS	POS	POS	NEG	NEG	POS	NEG
04V39012	NEG	POS	NEG	POS	NEG	NEG	NEG	POS	POS	POS	POS	NEG	NEG	POS	NEG
04V39577	NEG	AMB	NEG	POS	NEG	NEG	NEG	POS	POS	POS	POS	NEG	NEG	POS	NEG
04V22872	NEG	NEG	NEG	NEG	NEG	NEG	NEG	NEG	NEG	NEG	NEG	NEG	NEG	NEG	NEG

ISOLATE:	VIRULENCE : HLG AND LEUKOCIDINS												
	haemolysin gamma / leukocidin, component B	haemolysin gamma / leukocidin, component C		haemolysin gamma, component A	Panton Valentine leukocidin F component	Panton Valentine leukocidin S component	F component from hypothetical leukocidin from ruminants	S component from hypothetical leukocidin from ruminants	leukocidin D component	leukocidin E component	leukocidin/ haemolysin toxin family protein	leukocidin/haemolysin toxin family protein	
	lukF	lukS	lukS (ST22+ST45)	hlgA	lukF-PV	lukS-PV	lukF-PV (P83)	lukM	lukD	lukE	lukX	lukY	lukY (ST30+ST45)
**2003**													
03F01030	POS	POS	POS	POS	NEG	NEG	NEG	NEG	POS	POS	POS	POS	NEG
03V05419	AMB	POS	NEG	POS	NEG	NEG	NEG	NEG	NEG	NEG	POS	POS	NEG
03V08986	POS	POS	POS	POS	NEG	NEG	NEG	NEG	POS	POS	POS	POS	NEG
03V39123	POS	POS		POS	NEG	NEG	NEG	NEG	POS	POS	POS	POS	NEG
03B05186	POS	POS	NEG	POS	NEG	NEG	NEG	NEG	POS	POS	AMB	POS	NEG
03U00003	POS	POS	AMB	POS	NEG	NEG	NEG	NEG	POS	POS	POS	POS	NEG
03V01438	POS	POS	NEG	POS	NEG	NEG	NEG	NEG	POS	POS	POS	POS	NEG
03V05075	POS	POS	NEG	POS	NEG	NEG	NEG	NEG	POS	POS	POS	POS	NEG
03V11014	POS	POS	POS	POS	NEG	NEG	NEG	NEG	POS	POS	POS	POS	NEG
03V12917	POS	POS	POS	POS	NEG	NEG	NEG	NEG	POS	POS	POS	POS	NEG
03V14467	POS	POS	POS	POS	NEG	NEG	NEG	NEG	POS	POS	POS	POS	NEG
03V19357	POS	POS	NEG	POS	NEG	NEG	NEG	NEG	POS	POS	POS	POS	NEG
03V20186	POS	POS	POS	POS	NEG	NEG	NEG	NEG	POS	POS	POS	POS	NEG
03V25349	POS	POS	POS	POS	NEG	NEG	NEG	NEG	POS	POS	POS	POS	NEG
03V28948	POS	POS	NEG	POS	NEG	NEG	NEG	NEG	POS	POS	POS	POS	NEG
03V30841	POS	POS		POS	NEG	NEG	NEG	NEG	POS	POS	NEG	POS	NEG
03V32896	POS	POS	NEG	POS	NEG	NEG	NEG	NEG	POS	POS	POS	POS	NEG
03V33415	POS	POS	AMB	POS	NEG	NEG	NEG	NEG	POS	POS	POS	POS	NEG
03V34628	POS	POS	POS	POS	NEG	NEG	AMB	NEG	POS	POS	POS	POS	NEG
03V37078	POS	POS	NEG	POS	NEG	NEG	NEG	NEG	POS	AMB	AMB	POS	NEG
03V41645	POS	POS	POS	POS	NEG	NEG	NEG	NEG	POS	POS	POS	POS	NEG
03V03012	POS	POS	AMB	POS	NEG	NEG	NEG	NEG	POS	POS	NEG	POS	NEG
03V02863	POS	POS	AMB	POS	NEG	NEG	NEG	NEG	POS	POS	POS	POS	NEG
03V14882	POS	POS	AMB	POS	NEG	NEG	NEG	NEG	POS	POS	POS	POS	NEG
03B00404	POS	AMB	POS	AMB	NEG	NEG	NEG	NEG	NEG	NEG	POS	POS	NEG
03U00892	NEG	NEG		NEG	NEG	NEG	NEG	NEG	NEG	NEG	POS	POS	NEG
03V01422	AMB	NEG	POS	POS	NEG	NEG	NEG	NEG	NEG	NEG	POS	POS	NEG
03V04424	POS	POS	POS	POS	NEG	NEG	NEG	NEG	NEG	NEG	POS	POS	NEG
03V05690	NEG	NEG	POS	NEG	NEG	NEG	NEG	NEG	NEG	NEG	POS	POS	NEG
03V07912	NEG	NEG	POS	RANDOM	NEG	NEG	NEG	NEG	RANDOM	NEG	POS	POS	NEG
03V23798	NEG	NEG	POS	NEG	NEG	NEG	NEG	NEG	NEG	NEG	POS	POS	NEG
03V25914	NEG	NEG	POS	NEG	NEG	NEG	NEG	NEG	NEG	NEG	POS	POS	NEG
03V29751	AMB	NEG	POS	AMB	NEG	NEG	NEG	NEG	NEG	NEG	AMB	POS	NEG
03V30201	POS	AMB	POS	POS	NEG	NEG	NEG	NEG	NEG	NEG	POS	POS	NEG
03B01781	POS	AMB	POS	POS	NEG	NEG	NEG	NEG	NEG	NEG	POS	NEG	POS
03U05014	POS	POS	POS	POS	NEG	NEG	NEG	NEG	NEG	NEG	POS	NEG	POS
03U11106	POS	POS	POS	POS	NEG	NEG	NEG	NEG	NEG	NEG	POS	NEG	POS
03V00742	POS	NEG	POS	POS	NEG	NEG	NEG	NEG	NEG	NEG	POS	NEG	POS
03V01902	POS	AMB	POS	POS	NEG	NEG	NEG	NEG	NEG	NEG	POS	NEG	POS
03V02256	POS	AMB	POS	POS	NEG	NEG	NEG	NEG	NEG	NEG	POS	NEG	POS
03V02262	POS	AMB	POS	POS	NEG	NEG	NEG	NEG	NEG	NEG	POS	NEG	POS
03V04701	POS	NEG	POS	POS	NEG	NEG	NEG	NEG	NEG	NEG	POS	NEG	POS
03V05042	POS	POS	POS	POS	NEG	NEG	NEG	NEG	NEG	NEG	POS	NEG	POS
03V05377	POS	AMB	POS	POS	NEG	NEG	NEG	NEG	NEG	NEG	POS	NEG	POS
03V05507	POS	NEG	POS	POS	NEG	NEG	NEG	NEG	NEG	NEG	POS	NEG	POS
03V08214	POS	AMB	POS	POS	NEG	NEG	NEG	NEG	NEG	NEG	POS	NEG	POS
03V08525	POS	AMB	POS	POS	NEG	NEG	NEG	NEG	NEG	NEG	POS	NEG	POS
03V11910	POS	NEG	POS	AMB	NEG	NEG	NEG	NEG	NEG	NEG	POS	NEG	POS
03V14481	POS	AMB	POS	POS	NEG	NEG	NEG	NEG	NEG	NEG	POS	NEG	POS
03V15082	POS	POS	POS	POS	NEG	NEG	NEG	NEG	NEG	NEG	POS	NEG	POS
03V17508	POS	NEG	POS	POS	NEG	NEG	NEG	NEG	NEG	NEG	POS	NEG	POS
03V20150	POS	NEG	POS	POS	NEG	NEG	NEG	NEG	NEG	NEG	POS	NEG	POS
03V22437	POS	NEG	POS	AMB	NEG	NEG	NEG	NEG	NEG	NEG	POS	NEG	POS
03V24471	POS	AMB	POS	POS	NEG	NEG	NEG	NEG	NEG	NEG	POS	NEG	POS
03V28070	POS	NEG	POS	AMB	NEG	NEG	NEG	NEG	NEG	NEG	POS	NEG	AMB
03V28448	POS	AMB	POS	POS	NEG	NEG	NEG	NEG	NEG	NEG	POS	NEG	POS
03V34589	POS	AMB	POS	POS	NEG	NEG	NEG	NEG	NEG	NEG	POS	NEG	POS
03V39774	POS	AMB	POS	POS	NEG	NEG	NEG	NEG	NEG	NEG	POS	NEG	POS
**2004**													
04B05423	POS	POS	NEG	POS	NEG	NEG	NEG	NEG	POS	POS	POS	POS	NEG
04U02171	POS	POS	POS	POS	NEG	NEG	NEG	NEG	POS	POS	POS	POS	NEG
04U03682	POS	POS	NEG	POS	NEG	NEG	NEG	NEG	POS	POS	POS	AMB	NEG
04U06618	POS	POS	POS	POS	NEG	NEG	NEG	NEG	POS	POS	POS	POS	NEG
04U12578	POS	POS	NEG	POS	NEG	NEG	NEG	NEG	POS	POS	POS	POS	NEG
04V02465	POS	POS	NEG	POS	NEG	NEG	NEG	NEG	POS	POS	POS	POS	NEG
04V03642	POS	POS	NEG	POS	NEG	NEG	NEG	NEG	POS	POS	POS	POS	NEG
04V07657	POS	POS	NEG	POS	NEG	NEG	NEG	NEG	POS	POS	POS	POS	NEG
04V12223	POS	POS	NEG	POS	NEG	NEG	NEG	NEG	POS	POS	POS	POS	NEG
04V12529	POS	POS	NEG	POS	NEG	NEG	NEG	NEG	POS	POS	POS	POS	NEG
04V13633	POS	POS	NEG	POS	NEG	NEG	NEG	NEG	POS	POS	POS	POS	NEG
04V16499	POS	POS	POS	POS	NEG	NEG	NEG	NEG	POS	POS	POS	POS	NEG
04V17015	POS	POS	POS	POS	NEG	NEG	NEG	NEG	POS	POS	POS	POS	NEG
04V20684	POS	POS		POS	NEG	NEG	NEG	NEG	POS	POS	POS	POS	NEG
04V24615	POS	POS	NEG	POS	NEG	NEG	NEG	NEG	POS	POS	POS	POS	NEG
04V24901	POS	POS	NEG	POS	NEG	NEG	NEG	NEG	POS	POS	POS	POS	NEG
04V25081	POS	POS	NEG	POS	NEG	NEG	NEG	NEG	POS	POS	POS	POS	NEG
04V27293	POS	POS	AMB	POS	NEG	NEG	NEG	NEG	POS	POS	POS	POS	NEG
04V27311	POS	POS	NEG	POS	NEG	NEG	NEG	NEG	POS	POS	POS	POS	NEG
04V33459	POS	POS	POS	POS	NEG	NEG	NEG	NEG	POS	POS	POS	POS	NEG
04V39909	POS	POS	AMB	POS	NEG	NEG	NEG	NEG	POS	POS	POS	POS	NEG
04U02421	POS	POS	AMB	POS	NEG	NEG	NEG	NEG	POS	POS	NEG	POS	NEG
04V09807	POS	POS		POS	POS	POS	NEG	NEG	POS	POS	POS	POS	NEG
04V16073	POS	POS	NEG	POS	POS	POS	NEG	NEG	POS	POS	POS	POS	NEG
04V23548	POS	POS		POS	POS	POS	NEG	NEG	POS	POS	POS	POS	NEG
04V03289	POS	NEG		POS	NEG	NEG	NEG	NEG	NEG	NEG	POS	NEG	NEG
04V03847	POS	NEG		POS	NEG	NEG	NEG	NEG	NEG	NEG	NEG	POS	NEG
04V04595	POS	POS	POS	POS	NEG	NEG	NEG	NEG	NEG	NEG	POS	POS	NEG
04V05029	NEG	NEG	POS	NEG	NEG	NEG	NEG	NEG	NEG	NEG	AMB	AMB	NEG
04V07875	NEG	NEG	POS	NEG	NEG	NEG	NEG	NEG	NEG	NEG	AMB	NEG	NEG
04V14909	NEG	NEG	POS	NEG	NEG	NEG	NEG	NEG	NEG	NEG	POS	POS	NEG
04V18654	AMB	AMB	POS	POS	NEG	NEG	NEG	NEG	NEG	NEG	POS	POS	NEG
04V18815	POS	POS	POS	POS	NEG	NEG	NEG	NEG	NEG	NEG	POS	POS	NEG
04V29721	NEG	NEG	POS	NEG	NEG	NEG	NEG	NEG	NEG	NEG	POS	POS	NEG
04V32759	NEG	NEG	POS	NEG	NEG	NEG	NEG	NEG	NEG	NEG	POS	POS	NEG
04V32919	POS	POS	POS	POS	NEG	NEG	NEG	NEG	NEG	NEG	POS	POS	NEG
04V33780	NEG	NEG	POS	NEG	NEG	NEG	NEG	NEG	NEG	NEG	POS	POS	NEG
04V35396	POS	POS	POS	POS	NEG	NEG	NEG	NEG	NEG	NEG	POS	POS	NEG
04V37107	AMB	AMB	POS	POS	NEG	NEG	NEG	NEG	NEG	NEG	POS	POS	NEG
04V37518	NEG	NEG	POS	NEG	NEG	NEG	NEG	NEG	NEG	NEG	POS	POS	NEG
04V38199	NEG	NEG	POS	NEG	NEG	NEG	NEG	NEG	NEG	NEG	NEG	POS	NEG
04V38358	NEG	NEG	POS	NEG	NEG	NEG	NEG	NEG	NEG	NEG	POS	POS	NEG
04V38902	NEG	NEG	POS	NEG	NEG	NEG	NEG	NEG	NEG	NEG	POS	POS	NEG
04V39203	POS	AMB	POS	POS	NEG	NEG	NEG	NEG	NEG	NEG	POS	POS	NEG
04V40423	NEG	NEG	POS	NEG	NEG	NEG	NEG	NEG	NEG	NEG	POS	POS	NEG
04V08036	POS	POS	POS	POS	NEG	NEG	NEG	NEG	NEG	NEG	POS	NEG	POS
04U01119	POS	NEG	POS	POS	NEG	NEG	NEG	NEG	NEG	NEG	POS	NEG	POS
04V01185	POS	NEG	POS	POS	NEG	NEG	NEG	NEG	NEG	NEG	POS	NEG	POS
04V06276	POS	NEG	POS	POS	NEG	NEG	NEG	NEG	NEG	NEG	POS	NEG	POS
04V06677	POS	NEG	POS	POS	NEG	NEG	NEG	NEG	NEG	NEG	POS	NEG	POS
04V06688	POS	AMB	POS	POS	NEG	NEG	NEG	NEG	NEG	NEG	POS	NEG	POS
04V14835	POS	POS	POS	POS	NEG	NEG	NEG	NEG	NEG	NEG	POS	NEG	POS
04V22079	POS	NEG	POS	POS	NEG	NEG	NEG	NEG	NEG	NEG	POS	NEG	POS
04V29100	POS	NEG		POS	NEG	NEG	NEG	NEG	NEG	NEG	POS	NEG	POS
04V31370	POS	NEG	POS	POS	NEG	NEG	NEG	NEG	NEG	NEG	AMB	NEG	POS
04V38442	POS	POS	POS	POS	NEG	NEG	NEG	NEG	NEG	NEG	POS	NEG	POS
04V39012	POS	POS	POS	POS	NEG	NEG	NEG	NEG	NEG	NEG	POS	NEG	POS
04V39577	POS	POS	POS	POS	NEG	NEG	NEG	NEG	NEG	NEG	POS	NEG	POS
04V22872	POS	NEG	NEG	POS	POS	POS	AMB	NEG	POS	NEG	AMB	POS	NEG

ISOLATE:	STRAIN	VIRULENCE : HAEMOLYSINS								VIRULENCE : HLB-CONV PHAGES			VIRULENCE : EXFOL.TOXINS			VIRULENCE : EPITHEL. DIFF. INHIB			VIRULENCE : ACME LOCUS				VIRULENCE : PROTEASES										VIRULENCE : STAPHYLOCOCCAL SUPERANTIGEN/ENTEROTOXIN-LIKE GENES (SET/SSL)						
		putative membrane protein	haemolysin alpha	putative membrane protein		haemolysin beta				staphylo-kinase	chemotaxis-inhibiting protein (CHIPS)	Staphyl. Comple-ment inhibitor	exfoliative toxin serotype A	exfoliative toxin serotype B	exfoliative toxin D	epidermal cell differen-tiation inhibitor	epidermal cell differen-tiation inhibitor B	epidermal cell differen-tiation inhibitor C	ACME-locus	ACME-locus: ornithincarb-amoyltrans-ferase	ACME-locus: carbamat-kinase	ACME-locus: arginine/orni-thine-antiporter	aureolysin			serin- protease A	serin- protease B	serin- protease E	glutamylendope ptidase	staphopain B, protease	staphopain A (staphylopain A), protease		staphyl. exotoxin-like protein	staphylococcal superantigen-like protein 1					
		hl	hla	hlIII (consensus)	hlIII (other than RF122)	hlb_probe 1	hlb_probe 2	hlb_probe 3	un-disrupted hlb	sak	chp	scn	etA	etB	etD	edinA	edinB	edinC	arcA-SCC	arcB-SCC	arcC-SCC	arcD-SCC	aur (consensus)	aur (other than MRSA252)	aur (MRSA252)	splA	splB	splE	sspA	sspB	sspP (consensus)	sspP (other than ST93)	setC	ssl01/set6_probe1_11	ssl01/set6_probe2_11	ssl01/set6_probe1_12	ssl01/set6_probe2_12	ssl01/set6_probe4_11	ssl01/set6_probeRF122
**2003**																																							
03F01030	CC5/ST228-MRSA, South German EMRSA (truncated SCCmec element)	POS	POS	POS	POS	POS	POS	POS	NEG	POS	POS	POS	NEG	NEG	NEG	NEG	NEG	NEG	NEG	NEG	NEG	NEG	POS	POS	NEG	POS	POS	NEG	POS	POS	POS	POS	POS	POS	NEG	POS	NEG	POS	NEG
03V05419	CC5/ST228-MRSA-I, South German EMRSA	POS	POS	POS	POS	AMB	POS	NEG	NEG	POS	AMB	POS	NEG	NEG	NEG	NEG	NEG	NEG	NEG	NEG	NEG	NEG	POS	POS	NEG	POS	POS	NEG	POS	POS	POS	POS	POS	POS	NEG	POS	NEG	POS	NEG
03V08986	CC5/ST228-MRSA-I, South German EMRSA	POS	POS	POS	POS	POS	POS	AMB	NEG	POS	AMB	POS	NEG	NEG	NEG	NEG	NEG	NEG	NEG	NEG	NEG	NEG	POS	POS	NEG	POS	POS	NEG	POS	POS	POS	POS	POS	POS	NEG	POS	NEG	POS	NEG
03V39123	CC5/ST228-MRSA-I, South German EMRSA	POS	POS		POS				AMB	POS			NEG	NEG	NEG	NEG	NEG	NEG								POS	POS						POS	POS	NEG	POS	NEG	POS	
03B05186	CC5-MRSA-II, Rhine-Hesse EMRSA/UK-EMRSA-3	POS	POS	POS	POS	AMB	POS	NEG	NEG	POS	POS	POS	NEG	NEG	NEG	NEG	NEG	NEG	NEG	NEG	NEG	NEG	POS	POS	NEG	POS	POS	NEG	POS	POS	POS	POS	POS	POS	NEG	POS	NEG	AMB	NEG
03U00003	CC5-MRSA-II, Rhine-Hesse EMRSA/UK-EMRSA-3	POS	POS	POS	POS	POS	POS	AMB	NEG	POS	POS	POS	NEG	NEG	NEG	NEG	NEG	NEG	NEG	NEG	NEG	NEG	POS	POS	NEG	POS	POS	NEG	POS	POS	POS	POS	POS	POS	NEG	POS	NEG	POS	NEG
03V01438	CC5-MRSA-II, Rhine-Hesse EMRSA/UK-EMRSA-3	POS	POS	POS	POS	AMB	POS	AMB	NEG	POS	POS	POS	NEG	NEG	NEG	NEG	NEG	NEG	NEG	NEG	NEG	NEG	POS	POS	NEG	POS	POS	NEG	POS	POS	POS	POS	POS	POS	NEG	POS	NEG	POS	NEG
03V05075	CC5-MRSA-II, Rhine-Hesse EMRSA/UK-EMRSA-3	POS	POS	POS	POS	AMB	POS	AMB	NEG	POS	POS	POS	NEG	NEG	NEG	NEG	NEG	NEG	NEG	NEG	NEG	NEG	POS	POS	NEG	POS	POS	NEG	POS	POS	POS	POS	POS	POS	NEG	POS	NEG	POS	NEG
03V11014	CC5-MRSA-II, Rhine-Hesse EMRSA/UK-EMRSA-3	POS	POS	POS	POS	POS	POS	POS	NEG	POS	POS	POS	NEG	NEG	NEG	NEG	NEG	NEG	NEG	NEG	NEG	NEG	POS	POS	NEG	POS	POS	NEG	POS	POS	POS	POS	POS	POS	NEG	POS	NEG	POS	NEG
03V12917	CC5-MRSA-II, Rhine-Hesse EMRSA/UK-EMRSA-3	POS	POS	POS	POS	POS	POS	POS	NEG	POS	POS	POS	NEG	NEG	NEG	NEG	NEG	NEG	NEG	NEG	NEG	NEG	POS	POS	NEG	POS	POS	NEG	POS	POS	POS	POS	POS	POS	NEG	POS	NEG	POS	NEG
03V14467	CC5-MRSA-II, Rhine-Hesse EMRSA/UK-EMRSA-3	POS	POS	POS	POS	POS	POS	POS	AMB	POS	POS	POS	NEG	NEG	NEG	NEG	NEG	NEG	NEG	NEG	NEG	NEG	POS	POS	NEG	POS	POS	NEG	POS	POS	POS	POS	POS	POS	AMB	POS	NEG	POS	NEG
03V19357	CC5-MRSA-II, Rhine-Hesse EMRSA/UK-EMRSA-3	POS	POS	POS	POS	AMB	POS	NEG	NEG	POS	POS	POS	NEG	NEG	NEG	NEG	NEG	NEG	NEG	NEG	NEG	NEG	POS	POS	NEG	POS	POS	NEG	POS	POS	POS	POS	POS	POS	NEG	POS	NEG	AMB	NEG
03V20186	CC5-MRSA-II, Rhine-Hesse EMRSA/UK-EMRSA-3	POS	POS	POS	POS	POS	POS	POS	NEG	POS	POS	POS	NEG	NEG	NEG	NEG	NEG	NEG	NEG	NEG	NEG	NEG	POS	POS	NEG	POS	POS	NEG	POS	POS	POS	POS	POS	POS	NEG	POS	NEG	POS	NEG
03V25349	CC5-MRSA-II, Rhine-Hesse EMRSA/UK-EMRSA-3	POS	POS	POS	POS	POS	POS	POS	NEG	POS	POS	POS	NEG	NEG	NEG	NEG	NEG	NEG	NEG	NEG	NEG	NEG	POS	POS	NEG	POS	POS	NEG	POS	POS	POS	POS	POS	POS	AMB	POS	AMB	POS	NEG
03V28948	CC5-MRSA-II, Rhine-Hesse EMRSA/UK-EMRSA-3	POS	POS	POS	POS	NEG	POS	NEG	NEG	POS	POS	POS	NEG	NEG	NEG	NEG	NEG	NEG	NEG	NEG	NEG	NEG	POS	POS	NEG	POS	POS	NEG	POS	POS	POS	POS	POS	POS	NEG	POS	NEG	AMB	NEG
03V30841	CC5-MRSA-II, Rhine-Hesse EMRSA/UK-EMRSA-3	POS	POS		POS				NEG	POS			NEG	NEG	NEG	NEG	NEG	NEG								POS	POS						POS	POS	NEG	POS	NEG	POS	
03V32896	CC5-MRSA-II, Rhine-Hesse EMRSA/UK-EMRSA-3	POS	POS	POS	POS	AMB	POS	NEG	NEG	POS	POS	POS	NEG	NEG	NEG	NEG	NEG	NEG	NEG	NEG	NEG	NEG	POS	POS	NEG	POS	POS	NEG	POS	POS	POS	POS	POS	POS	NEG	POS	NEG	POS	NEG
03V33415	CC5-MRSA-II, Rhine-Hesse EMRSA/UK-EMRSA-3	POS	POS	POS	POS	POS	POS	AMB	NEG	POS	POS	POS	NEG	NEG	NEG	NEG	NEG	NEG	NEG	NEG	NEG	NEG	POS	POS	NEG	POS	POS	NEG	POS	POS	POS	POS	POS	POS	NEG	POS	NEG	POS	NEG
03V34628	CC5-MRSA-II, Rhine-Hesse EMRSA/UK-EMRSA-3	POS	POS	POS	POS	POS	POS	POS	AMB	POS	POS	POS	NEG	NEG	NEG	NEG	NEG	NEG	NEG	NEG	NEG	NEG	POS	POS	NEG	POS	POS	NEG	POS	POS	POS	POS	POS	POS	AMB	POS	AMB	POS	NEG
03V37078	CC5-MRSA-II, Rhine-Hesse EMRSA/UK-EMRSA-3	POS	POS	POS	POS	AMB	POS	AMB	NEG	POS	POS	POS	NEG	NEG	NEG	NEG	NEG	NEG	NEG	NEG	NEG	NEG	AMB	POS	NEG	POS	POS	NEG	POS	POS	POS	POS	POS	POS	NEG	POS	NEG	AMB	NEG
03V41645	CC5-MRSA-II, Rhine-Hesse EMRSA/UK-EMRSA-3	POS	POS	POS	POS	POS	POS	POS	NEG	POS	POS	POS	NEG	NEG	NEG	NEG	NEG	NEG	NEG	NEG	NEG	NEG	POS	POS	NEG	POS	POS	NEG	POS	POS	POS	POS	POS	POS	NEG	POS	NEG	POS	NEG
03V03012	CC5-MRSA-IV, Paediatric clone	POS	POS	POS	POS	POS	POS	POS	NEG	POS	POS	POS	NEG	NEG	NEG	NEG	NEG	NEG	NEG	NEG	NEG	NEG	POS	POS	NEG	POS	POS	NEG	POS	POS	POS	POS	POS						NEG
03V02863	CC8/ST247-MRSA-I, North German/Iberian EMRSA	POS	POS	POS	POS	POS	POS	POS	NEG	POS	POS	POS	NEG	NEG	NEG	NEG	NEG	NEG	NEG	NEG	NEG	NEG	POS	POS	NEG	POS	POS	POS	POS	POS	POS	POS	POS	POS	NEG	NEG	NEG	POS	NEG
03V14882	CC8/ST254-MRSA-(atypical SCCmec), Hannover EMRSA	POS	POS	POS	POS	POS	POS	POS	POS	NEG	NEG	NEG	NEG	NEG	NEG	NEG	NEG	NEG	NEG	NEG	NEG	NEG	POS	POS	NEG	POS	POS	POS	POS	POS	POS	POS	POS	POS	NEG	AMB	NEG	POS	NEG
03B00404	CC22-MRSA-IV, Barnim/UK-EMRSA-15	POS	POS	NEG	NEG	POS	POS	POS	NEG	POS	POS	POS	NEG	NEG	NEG	NEG	NEG	NEG	NEG	NEG	NEG	NEG	POS	NEG	POS	NEG	NEG	NEG	POS	POS	POS	POS	POS	NEG	POS	NEG	NEG	NEG	NEG
03U00892	CC22-MRSA-IV, Barnim/UK-EMRSA-15	POS	POS		NEG				NEG	POS			NEG	NEG	NEG	NEG	NEG	NEG								NEG	NEG						POS						
03V01422	CC22-MRSA-IV, Barnim/UK-EMRSA-15	POS	POS	NEG	NEG	POS	POS	POS	POS	NEG	NEG	NEG	NEG	NEG	NEG	NEG	NEG	NEG	NEG	NEG	NEG	NEG	POS	NEG	POS	NEG	NEG	NEG	POS	POS	POS	POS	POS	NEG	POS	AMB	NEG	NEG	NEG
03V04424	CC22-MRSA-IV, Barnim/UK-EMRSA-15	POS	POS	NEG	NEG	POS	POS	POS	POS	NEG	NEG	NEG	NEG	NEG	NEG	NEG	NEG	NEG	NEG	NEG	NEG	NEG	POS	NEG	POS	NEG	NEG	NEG	POS	POS	POS	POS	POS	NEG	POS	POS	NEG	NEG	NEG
03V05690	CC22-MRSA-IV, Barnim/UK-EMRSA-15	POS	POS	NEG	NEG	POS	POS	POS	NEG	POS	POS	POS	NEG	NEG	NEG	NEG	NEG	NEG	NEG	NEG	NEG	NEG	POS	NEG	POS	NEG	NEG	NEG	POS	POS	POS	POS	POS	NEG	POS	NEG	NEG	NEG	NEG
03V07912	CC22-MRSA-IV, Barnim/UK-EMRSA-15	POS	POS	NEG	NEG	POS	POS	POS	NEG	POS	POS	POS	NEG	NEG	NEG	NEG	NEG	NEG	NEG	NEG	NEG	NEG	POS	NEG	POS	NEG	NEG	NEG	POS	POS	POS	POS	POS						NEG
03V23798	CC22-MRSA-IV, Barnim/UK-EMRSA-15	POS	AMB	NEG	NEG	POS	POS	POS	NEG	POS	POS	POS	NEG	NEG	NEG	NEG	NEG	NEG	NEG	NEG	NEG	NEG	POS	NEG	POS	NEG	NEG	NEG	POS	POS	POS	POS	POS	NEG	POS	NEG	NEG	NEG	NEG
03V25914	CC22-MRSA-IV, Barnim/UK-EMRSA-15	POS	POS	NEG	NEG	POS	POS	POS	NEG	POS	POS	POS	NEG	NEG	NEG	NEG	NEG	NEG	NEG	NEG	NEG	NEG	POS	NEG	POS	NEG	NEG	NEG	POS	POS	POS	POS	POS	NEG	POS	NEG	NEG	NEG	NEG
03V29751	CC22-MRSA-IV, Barnim/UK-EMRSA-15	POS	AMB	NEG	NEG	POS	POS	AMB	NEG	POS	POS	POS	NEG	NEG	NEG	NEG	NEG	NEG	NEG	NEG	NEG	NEG	POS	NEG	POS	NEG	NEG	NEG	POS	POS	POS	POS	POS	NEG	POS	AMB	NEG	NEG	NEG
03V30201	CC22-MRSA-IV, Barnim/UK-EMRSA-15	POS	POS	NEG	NEG	POS	POS	POS	NEG	POS	POS	POS	NEG	NEG	NEG	NEG	NEG	NEG	NEG	NEG	NEG	NEG	POS	NEG	POS	NEG	NEG	NEG	POS	POS	POS	POS	POS	NEG	POS	POS	NEG	NEG	NEG
03B01781	CC45-MRSA-IV, Berlin EMRSA	POS	POS	POS	NEG	AMB	POS	NEG	NEG	POS	POS	POS	NEG	NEG	NEG	NEG	NEG	NEG	NEG	NEG	NEG	NEG	AMB	NEG	POS	NEG	NEG	NEG	POS	POS	POS	POS	AMB	NEG	POS	AMB	NEG	POS	NEG
03U05014	CC45-MRSA-IV, Berlin EMRSA	POS	POS	POS	NEG	POS	POS	NEG	NEG	POS	POS	POS	NEG	NEG	NEG	NEG	NEG	NEG	NEG	NEG	NEG	NEG	POS	NEG	POS	NEG	NEG	NEG	POS	POS	POS	POS	POS	NEG	POS	AMB	NEG	POS	NEG
03U11106	CC45-MRSA-IV, Berlin EMRSA	POS	POS	POS	NEG	NEG	NEG	NEG	NEG	POS	POS	POS	NEG	NEG	NEG	NEG	NEG	NEG	NEG	NEG	NEG	NEG	POS	NEG	POS	NEG	NEG	NEG	POS	POS	POS	POS	POS	NEG	POS	POS	NEG	POS	NEG
03V00742	CC45-MRSA-IV, Berlin EMRSA	POS	POS	POS	NEG	AMB	AMB	NEG	NEG	POS	POS	POS	NEG	NEG	NEG	NEG	NEG	NEG	NEG	NEG	NEG	NEG	AMB	NEG	POS	NEG	NEG	NEG	POS	POS	POS	POS	NEG	NEG	POS	NEG	NEG	AMB	NEG
03V01902	CC45-MRSA-IV, Berlin EMRSA	POS	POS	POS	NEG	AMB	POS	NEG	NEG	POS	POS	POS	NEG	NEG	NEG	NEG	NEG	NEG	NEG	NEG	NEG	NEG	AMB	NEG	POS	NEG	NEG	NEG	POS	POS	POS	POS	AMB	NEG	POS	AMB	NEG	POS	NEG
03V02256	CC45-MRSA-IV, Berlin EMRSA	POS	POS	POS	NEG	POS	POS	NEG	NEG	POS	POS	POS	NEG	NEG	NEG	NEG	NEG	NEG	NEG	NEG	NEG	NEG	POS	NEG	POS	NEG	NEG	NEG	POS	POS	POS	POS	AMB	NEG	POS	AMB	NEG	POS	NEG
03V02262	CC45-MRSA-IV, Berlin EMRSA	POS	POS	POS	NEG	NEG	NEG	NEG	POS	NEG	NEG	NEG	NEG	NEG	NEG	NEG	NEG	NEG	NEG	NEG	NEG	NEG	AMB	NEG	POS	NEG	NEG	NEG	POS	POS	POS	POS	AMB	NEG	POS	NEG	NEG	AMB	NEG
03V04701	CC45-MRSA-IV, Berlin EMRSA	POS	POS	POS	NEG	NEG	AMB	NEG	NEG	POS	POS	POS	NEG	NEG	NEG	NEG	NEG	NEG	NEG	NEG	NEG	NEG	AMB	NEG	POS	NEG	NEG	NEG	POS	POS	POS	POS	NEG	NEG	POS	NEG	NEG	AMB	NEG
03V05042	CC45-MRSA-IV, Berlin EMRSA	POS	POS	POS	NEG	POS	POS	NEG	NEG	POS	POS	POS	NEG	NEG	NEG	NEG	NEG	NEG	NEG	NEG	NEG	NEG	POS	NEG	POS	NEG	NEG	NEG	POS	POS	POS	POS	POS	NEG	POS	AMB	NEG	POS	NEG
03V05377	CC45-MRSA-IV, Berlin EMRSA	POS	POS	POS	NEG	AMB	POS	NEG	NEG	POS	POS	POS	NEG	NEG	NEG	NEG	NEG	NEG	NEG	NEG	NEG	NEG	POS	NEG	POS	NEG	NEG	NEG	POS	POS	POS	POS	AMB	NEG	POS	AMB	NEG	AMB	NEG
03V05507	CC45-MRSA-IV, Berlin EMRSA	POS	POS	POS	NEG	NEG	AMB	NEG	NEG	POS	POS	POS	NEG	NEG	NEG	NEG	NEG	NEG	NEG	NEG	NEG	NEG	AMB	NEG	POS	NEG	NEG	NEG	POS	POS	POS	POS	NEG	NEG	POS	NEG	NEG	AMB	NEG
03V08214	CC45-MRSA-IV, Berlin EMRSA	POS	POS	POS	NEG	POS	POS	NEG	NEG	POS	POS	POS	NEG	NEG	NEG	NEG	NEG	NEG	NEG	NEG	NEG	NEG	POS	NEG	POS	NEG	NEG	NEG	POS	POS	POS	POS	POS	NEG	POS	POS	NEG	POS	NEG
03V08525	CC45-MRSA-IV, Berlin EMRSA	POS	POS	POS	NEG	AMB	AMB	NEG	NEG	POS	POS	POS	NEG	NEG	NEG	NEG	NEG	NEG	NEG	NEG	NEG	NEG	AMB	NEG	POS	NEG	NEG	NEG	POS	POS	POS	POS	AMB	NEG	POS	NEG	NEG	AMB	NEG
03V11910	CC45-MRSA-IV, Berlin EMRSA	AMB	AMB	POS	NEG	NEG	NEG	NEG	NEG	POS	POS	POS	NEG	NEG	NEG	NEG	NEG	NEG	NEG	NEG	NEG	NEG	NEG	NEG	POS	NEG	NEG	NEG	POS	POS	POS	POS	NEG	NEG	POS	NEG	NEG	NEG	NEG
03V14481	CC45-MRSA-IV, Berlin EMRSA	POS	POS	POS	NEG	AMB	AMB	NEG	NEG	POS	POS	POS	NEG	NEG	NEG	NEG	NEG	NEG	NEG	NEG	NEG	NEG	AMB	NEG	POS	NEG	NEG	NEG	POS	POS	POS	POS	AMB	NEG	POS	NEG	NEG	AMB	NEG
03V15082	CC45-MRSA-IV, Berlin EMRSA	POS	POS	POS	NEG	POS	POS	NEG	NEG	POS	POS	POS	NEG	NEG	NEG	NEG	NEG	NEG	NEG	NEG	NEG	NEG	POS	NEG	POS	NEG	NEG	NEG	POS	POS	POS	POS	POS	NEG	POS	POS	NEG	POS	NEG
03V17508	CC45-MRSA-IV, Berlin EMRSA	POS	AMB	POS	NEG	NEG	NEG	NEG	NEG	POS	POS	POS	NEG	NEG	NEG	NEG	NEG	NEG	NEG	NEG	NEG	NEG	NEG	NEG	POS	NEG	NEG	NEG	POS	POS	POS	POS	NEG	NEG	POS	NEG	NEG	NEG	NEG
03V20150	CC45-MRSA-IV, Berlin EMRSA	AMB	AMB	POS	NEG	NEG	NEG	NEG	NEG	POS	POS	POS	NEG	NEG	NEG	NEG	NEG	NEG	NEG	NEG	NEG	NEG	NEG	NEG	POS	NEG	NEG	NEG	POS	POS	POS	POS	NEG	NEG	POS	NEG	NEG	NEG	NEG
03V22437	CC45-MRSA-IV, Berlin EMRSA	AMB	AMB	POS	NEG	NEG	NEG	NEG	NEG	POS	POS	POS	NEG	NEG	NEG	NEG	NEG	NEG	NEG	NEG	NEG	NEG	NEG	NEG	POS	NEG	NEG	NEG	POS	POS	POS	POS	NEG	NEG	POS	NEG	NEG	NEG	NEG
03V24471	CC45-MRSA-IV, Berlin EMRSA	POS	POS	POS	NEG	AMB	POS	NEG	NEG	POS	POS	POS	NEG	NEG	NEG	NEG	NEG	NEG	NEG	NEG	NEG	NEG	POS	NEG	POS	NEG	NEG	NEG	POS	POS	POS	POS	AMB	NEG	POS	AMB	NEG	POS	NEG
03V28070	CC45-MRSA-IV, Berlin EMRSA	NEG	NEG	POS	NEG	NEG	NEG	NEG	NEG	POS	POS	POS	NEG	NEG	NEG	NEG	NEG	NEG	NEG	NEG	NEG	NEG	NEG	NEG	POS	NEG	NEG	NEG	POS	POS	POS	POS	NEG	NEG	POS	NEG	NEG	NEG	NEG
03V28448	CC45-MRSA-IV, Berlin EMRSA	POS	POS	POS	NEG	AMB	AMB	NEG	NEG	POS	POS	POS	NEG	NEG	NEG	NEG	NEG	NEG	NEG	NEG	NEG	NEG	AMB	NEG	POS	NEG	NEG	NEG	POS	POS	POS	POS	AMB	NEG	POS	AMB	NEG	AMB	NEG
03V34589	CC45-MRSA-IV, Berlin EMRSA	POS	POS	POS	NEG	AMB	POS	NEG	NEG	POS	POS	POS	NEG	NEG	NEG	NEG	NEG	NEG	NEG	NEG	NEG	NEG	AMB	NEG	POS	NEG	NEG	NEG	POS	POS	POS	POS	AMB	NEG	POS	AMB	NEG	POS	NEG
03V39774	CC45-MRSA-IV, Berlin EMRSA	POS	POS	POS	NEG	AMB	POS	NEG	NEG	POS	POS	POS	NEG	NEG	NEG	NEG	NEG	NEG	NEG	NEG	NEG	NEG	POS	NEG	POS	NEG	NEG	NEG	POS	POS	POS	POS	AMB	NEG	POS	NEG	NEG	AMB	NEG
**2004**																																							
04B05423	CC5-MRSA-II, Rhine-Hesse EMRSA/UK-EMRSA-3	POS	POS	POS	POS	AMB	POS	NEG	NEG	POS	POS	POS	NEG	NEG	NEG	NEG	NEG	NEG	NEG	NEG	NEG	NEG	POS	POS	NEG	POS	POS	NEG	POS	POS	POS	POS	POS	POS	NEG	POS	NEG	AMB	NEG
04U02171	CC5-MRSA-II, Rhine-Hesse EMRSA/UK-EMRSA-3	POS	POS	POS	POS	POS	POS	POS	NEG	POS	POS	POS	NEG	NEG	NEG	NEG	NEG	NEG	NEG	NEG	NEG	NEG	POS	POS	NEG	POS	POS	NEG	POS	POS	POS	POS	POS	POS	AMB	POS	NEG	POS	NEG
04U03682	CC5-MRSA-II, Rhine-Hesse EMRSA/UK-EMRSA-3	POS	POS	POS	POS	NEG	POS	NEG	NEG	POS	POS	POS	NEG	NEG	NEG	NEG	NEG	NEG	NEG	NEG	NEG	NEG	POS	POS	NEG	POS	POS	NEG	POS	POS	POS	POS	POS	POS	NEG	POS	NEG	AMB	NEG
04U06618	CC5-MRSA-II, Rhine-Hesse EMRSA/UK-EMRSA-3	POS	POS	POS	POS	POS	POS	POS	NEG	POS	POS	POS	NEG	NEG	NEG	NEG	NEG	NEG	NEG	NEG	NEG	NEG	POS	POS	NEG	POS	POS	NEG	POS	POS	POS	POS	POS	POS	AMB	POS	NEG	POS	NEG
04U12578	CC5-MRSA-II, Rhine-Hesse EMRSA/UK-EMRSA-3	POS	POS	POS	POS	AMB	POS	NEG	NEG	POS	POS	POS	NEG	NEG	NEG	NEG	NEG	NEG	NEG	NEG	NEG	NEG	POS	POS	NEG	POS	POS	NEG	POS	POS	POS	POS	POS	POS	NEG	POS	NEG	AMB	NEG
04V02465	CC5-MRSA-II, Rhine-Hesse EMRSA/UK-EMRSA-3	POS	POS	POS	POS	AMB	POS	AMB	NEG	POS	POS	POS	NEG	NEG	NEG	NEG	NEG	NEG	NEG	NEG	NEG	NEG	POS	POS	NEG	POS	POS	NEG	POS	POS	POS	POS	POS	POS	NEG	POS	NEG	POS	NEG
04V03642	CC5-MRSA-II, Rhine-Hesse EMRSA/UK-EMRSA-3	POS	POS	POS	POS	NEG	POS	NEG	NEG	POS	POS	POS	NEG	NEG	NEG	NEG	NEG	NEG	NEG	NEG	NEG	NEG	POS	POS	NEG	POS	POS	NEG	POS	POS	POS	POS	POS	POS	NEG	POS	NEG	AMB	NEG
04V07657	CC5-MRSA-II, Rhine-Hesse EMRSA/UK-EMRSA-3	POS	POS	POS	POS	NEG	POS	NEG	NEG	POS	POS	POS	NEG	NEG	NEG	NEG	NEG	NEG	NEG	NEG	NEG	NEG	POS	POS	NEG	POS	POS	NEG	POS	POS	POS	POS	POS	POS	NEG	POS	NEG	AMB	NEG
04V12223	CC5-MRSA-II, Rhine-Hesse EMRSA/UK-EMRSA-3	POS	POS	POS	POS	NEG	POS	NEG	NEG	POS	POS	POS	NEG	NEG	NEG	NEG	NEG	NEG	NEG	NEG	NEG	NEG	POS	POS	NEG	POS	POS	NEG	POS	POS	POS	POS	POS	POS	NEG	POS	NEG	AMB	NEG
04V12529	CC5-MRSA-II, Rhine-Hesse EMRSA/UK-EMRSA-3	POS	POS	POS	POS	NEG	POS	NEG	NEG	POS	POS	POS	NEG	NEG	NEG	NEG	NEG	NEG	NEG	NEG	NEG	NEG	POS	POS	NEG	POS	POS	NEG	POS	POS	POS	POS	POS	POS	NEG	POS	NEG	AMB	NEG
04V13633	CC5-MRSA-II, Rhine-Hesse EMRSA/UK-EMRSA-3	POS	POS	POS	POS	AMB	POS	NEG	NEG	POS	POS	POS	NEG	NEG	NEG	NEG	NEG	NEG	NEG	NEG	NEG	NEG	POS	POS	NEG	POS	POS	NEG	POS	POS	POS	POS	POS	POS	NEG	POS	NEG	AMB	NEG
04V16499	CC5-MRSA-II, Rhine-Hesse EMRSA/UK-EMRSA-3	POS	POS	POS	POS	POS	POS	POS	NEG	POS	POS	POS	NEG	NEG	NEG	NEG	NEG	NEG	NEG	NEG	NEG	NEG	POS	POS	NEG	POS	POS	NEG	POS	POS	POS	POS	POS	POS	AMB	POS	NEG	POS	NEG
04V17015	CC5-MRSA-II, Rhine-Hesse EMRSA/UK-EMRSA-3	POS	POS	POS	POS	POS	POS	POS	NEG	POS	POS	POS	NEG	NEG	NEG	NEG	NEG	NEG	NEG	NEG	NEG	NEG	POS	POS	NEG	POS	POS	NEG	POS	POS	POS	POS	POS	POS	AMB	POS	NEG	POS	NEG
04V20684	CC5-MRSA-II, Rhine-Hesse EMRSA/UK-EMRSA-3	POS	POS		POS				NEG	POS			NEG	NEG	NEG	NEG	NEG	NEG								POS	POS						POS	POS	NEG	POS	NEG	POS	
04V24615	CC5-MRSA-II, Rhine-Hesse EMRSA/UK-EMRSA-3	POS	POS	POS	POS	AMB	POS	NEG	NEG	POS	POS	POS	NEG	NEG	NEG	NEG	NEG	NEG	NEG	NEG	NEG	NEG	POS	POS	NEG	POS	POS	NEG	POS	POS	POS	POS	POS	POS	NEG	POS	NEG	POS	NEG
04V24901	CC5-MRSA-II, Rhine-Hesse EMRSA/UK-EMRSA-3	POS	POS	POS	POS	POS	POS	AMB	NEG	POS	POS	POS	NEG	NEG	NEG	NEG	NEG	NEG	NEG	NEG	NEG	NEG	POS	POS	NEG	POS	POS	NEG	POS	POS	POS	POS	POS	POS	NEG	POS	NEG	POS	NEG
04V25081	CC5-MRSA-II, Rhine-Hesse EMRSA/UK-EMRSA-3	POS	POS	POS	POS	POS	POS	AMB	NEG	POS	POS	POS	NEG	NEG	NEG	NEG	NEG	NEG	NEG	NEG	NEG	NEG	POS	POS	NEG	POS	POS	NEG	POS	POS	POS	POS	POS	POS	NEG	POS	NEG	POS	NEG
04V27293	CC5-MRSA-II, Rhine-Hesse EMRSA/UK-EMRSA-3	POS	POS	POS	POS	POS	POS	POS	NEG	POS	POS	POS	NEG	NEG	NEG	NEG	NEG	NEG	NEG	NEG	NEG	NEG	POS	POS	NEG	POS	POS	NEG	POS	POS	POS	POS	POS	POS	NEG	POS	NEG	POS	NEG
04V27311	CC5-MRSA-II, Rhine-Hesse EMRSA/UK-EMRSA-3	POS	POS	POS	POS	NEG	POS	NEG	NEG	POS	POS	POS	NEG	NEG	NEG	NEG	NEG	NEG	NEG	NEG	NEG	NEG	POS	POS	NEG	POS	POS	NEG	POS	POS	POS	POS	POS	POS	NEG	POS	NEG	NEG	NEG
04V33459	CC5-MRSA-II, Rhine-Hesse EMRSA/UK-EMRSA-3	POS	POS	POS	POS	POS	POS	POS	NEG	POS	POS	POS	NEG	NEG	NEG	NEG	NEG	NEG	NEG	NEG	NEG	NEG	POS	POS	NEG	POS	POS	NEG	POS	POS	POS	POS	POS	POS	NEG	POS	NEG	POS	NEG
04V39909	CC5-MRSA-IV&VI	POS	POS	POS	POS	POS	POS	AMB	AMB	POS	NEG	POS	NEG	NEG	NEG	NEG	NEG	NEG	NEG	NEG	NEG	NEG	POS	POS	NEG	POS	POS	NEG	POS	POS	POS	POS	POS						NEG
04U02421	CC5-MRSA-IV, Paediatric clone	POS	POS	POS	POS	POS	POS	POS	NEG	POS	POS	POS	NEG	NEG	NEG	NEG	NEG	NEG	NEG	NEG	NEG	NEG	POS	POS	NEG	POS	POS	NEG	POS	POS	POS	POS	POS						NEG
04V09807	CC80-MRSA-IV [PVL+], European caMRSA Clone	POS	POS		POS				NEG	POS			NEG	NEG	POS	NEG	POS	NEG								POS	POS						POS						
04V16073	CC80-MRSA-IV [PVL+], European caMRSA Clone	POS	POS	POS	POS	AMB	POS	NEG	NEG	POS	NEG	POS	NEG	NEG	POS	NEG	POS	NEG	NEG	NEG	NEG	NEG	POS	POS	NEG	POS	POS	NEG	POS	POS	POS	POS	POS						POS
04V23548	CC80-MRSA-IV [PVL+], European caMRSA Clone	POS	POS		POS				NEG	POS			NEG	NEG	POS	NEG	POS	NEG								POS	POS						POS						
04V03289	CC22-MRSA-IV, Barnim/UK-EMRSA-15	POS	POS		NEG				POS	POS			NEG	NEG	NEG	NEG	NEG	NEG								NEG	NEG						POS						
04V03847	CC22-MRSA-IV, Barnim/UK-EMRSA-15	POS	POS		NEG				POS	POS			NEG	NEG	NEG	NEG	NEG	NEG								NEG	NEG						POS						
04V04595	CC22-MRSA-IV, Barnim/UK-EMRSA-15	POS	POS	NEG	NEG	POS	POS	POS	NEG	POS	POS	POS	NEG	NEG	NEG	NEG	NEG	NEG	NEG	NEG	NEG	NEG	POS	NEG	POS	NEG	NEG	NEG	POS	POS	POS	POS	POS	NEG	POS	POS	NEG	NEG	NEG
04V05029	CC22-MRSA-IV, Barnim/UK-EMRSA-15	POS	NEG	NEG	NEG	AMB	POS	NEG	NEG	POS	POS	AMB	NEG	NEG	NEG	NEG	NEG	NEG	NEG	NEG	NEG	NEG	POS	NEG	POS	NEG	NEG	NEG	POS	POS	POS	POS	POS	NEG	POS	NEG	NEG	NEG	NEG
04V07875	CC22-MRSA-IV, Barnim/UK-EMRSA-15	POS	NEG	NEG	NEG	NEG	POS	NEG	NEG	AMB	POS	AMB	NEG	NEG	NEG	NEG	NEG	NEG	NEG	NEG	NEG	NEG	POS	NEG	POS	NEG	NEG	NEG	POS	AMB	AMB	POS	POS	NEG	AMB	NEG	NEG	NEG	NEG
04V14909	CC22-MRSA-IV, Barnim/UK-EMRSA-15	POS	NEG	NEG	NEG	POS	POS	AMB	NEG	POS	POS	POS	NEG	NEG	NEG	NEG	NEG	NEG	NEG	NEG	NEG	NEG	POS	NEG	POS	NEG	NEG	NEG	POS	POS	POS	POS	POS	NEG	POS	NEG	NEG	NEG	NEG
04V18654	CC22-MRSA-IV, Barnim/UK-EMRSA-15	POS	POS	NEG	NEG	POS	POS	POS	NEG	POS	POS	POS	NEG	NEG	NEG	NEG	NEG	NEG	NEG	NEG	NEG	NEG	POS	NEG	POS	NEG	NEG	NEG	POS	POS	POS	POS	POS	NEG	POS	NEG	NEG	NEG	NEG
04V18815	CC22-MRSA-IV, Barnim/UK-EMRSA-15	POS	POS	NEG	NEG	POS	POS	POS	NEG	POS	POS	POS	NEG	NEG	NEG	NEG	NEG	NEG	NEG	NEG	NEG	NEG	POS	NEG	POS	NEG	NEG	NEG	POS	POS	POS	POS	POS	NEG	POS	POS	NEG	NEG	NEG
04V29721	CC22-MRSA-IV, Barnim/UK-EMRSA-15	POS	NEG	NEG	NEG	POS	POS	NEG	NEG	POS	POS	POS	NEG	NEG	NEG	NEG	NEG	NEG	NEG	NEG	NEG	NEG	POS	NEG	POS	NEG	NEG	NEG	POS	POS	POS	POS	POS	NEG	POS	NEG	NEG	NEG	NEG
04V32759	CC22-MRSA-IV, Barnim/UK-EMRSA-15	POS	AMB	NEG	NEG	POS	POS	POS	NEG	POS	POS	POS	NEG	NEG	NEG	NEG	NEG	NEG	NEG	NEG	NEG	NEG	POS	NEG	POS	NEG	NEG	NEG	POS	POS	POS	POS	POS	NEG	POS	NEG	NEG	NEG	NEG
04V32919	CC22-MRSA-IV, Barnim/UK-EMRSA-15	POS	POS	POS	NEG	POS	POS	POS	POS	POS	POS	POS	NEG	NEG	NEG	NEG	NEG	NEG	NEG	NEG	NEG	NEG	POS	NEG	POS	NEG	NEG	NEG	POS	POS	POS	POS	POS	NEG	POS	POS	NEG	NEG	NEG
04V33780	CC22-MRSA-IV, Barnim/UK-EMRSA-15	POS	AMB	NEG	NEG	POS	POS	POS	NEG	POS	POS	POS	NEG	NEG	NEG	NEG	NEG	NEG	NEG	NEG	NEG	NEG	POS	NEG	POS	NEG	NEG	NEG	POS	POS	POS	POS	POS	NEG	POS	AMB	NEG	NEG	NEG
04V35396	CC22-MRSA-IV, Barnim/UK-EMRSA-15	POS	POS	NEG	NEG	POS	POS	POS	NEG	POS	POS	POS	NEG	NEG	NEG	NEG	NEG	NEG	NEG	NEG	NEG	NEG	POS	NEG	POS	NEG	NEG	NEG	POS	POS	POS	POS	POS	NEG	POS	POS	NEG	NEG	NEG
04V37107	CC22-MRSA-IV, Barnim/UK-EMRSA-15	POS	POS	NEG	NEG	POS	POS	POS	NEG	POS	POS	POS	NEG	NEG	NEG	NEG	NEG	NEG	NEG	NEG	NEG	NEG	POS	NEG	POS	NEG	NEG	NEG	POS	POS	POS	POS	POS	NEG	POS	POS	NEG	NEG	NEG
04V37518	CC22-MRSA-IV, Barnim/UK-EMRSA-15	POS	NEG	NEG	NEG	POS	POS	AMB	NEG	POS	POS	POS	NEG	NEG	NEG	NEG	NEG	NEG	NEG	NEG	NEG	NEG	POS	NEG	POS	NEG	NEG	NEG	POS	POS	POS	POS	POS	NEG	POS	NEG	NEG	NEG	NEG
04V38199	CC22-MRSA-IV, Barnim/UK-EMRSA-15	POS	POS	NEG	NEG	POS	POS	POS	NEG	POS	POS	POS	NEG	NEG	NEG	NEG	NEG	NEG	NEG	NEG	NEG	NEG	POS	NEG	POS	NEG	NEG	NEG	POS	POS	POS	POS	POS						NEG
04V38358	CC22-MRSA-IV, Barnim/UK-EMRSA-15	POS	AMB	NEG	NEG	POS	POS	AMB	NEG	POS	POS	POS	NEG	NEG	NEG	NEG	NEG	NEG	NEG	NEG	NEG	NEG	POS	NEG	POS	NEG	NEG	NEG	POS	POS	POS	POS	POS	NEG	POS	NEG	NEG	NEG	NEG
04V38902	CC22-MRSA-IV, Barnim/UK-EMRSA-15	POS	NEG	NEG	NEG	POS	POS	AMB	NEG	POS	POS	POS	NEG	NEG	NEG	NEG	NEG	NEG	NEG	NEG	NEG	NEG	POS	NEG	POS	NEG	NEG	NEG	POS	POS	POS	POS	POS	NEG	POS	NEG	NEG	NEG	NEG
04V39203	CC22-MRSA-IV, Barnim/UK-EMRSA-15	POS	POS	NEG	NEG	POS	POS	POS	NEG	POS	POS	POS	NEG	NEG	NEG	NEG	NEG	NEG	NEG	NEG	NEG	NEG	POS	NEG	POS	NEG	NEG	NEG	POS	POS	POS	POS	POS	NEG	POS	POS	NEG	NEG	NEG
04V40423	CC22-MRSA-IV, Barnim/UK-EMRSA-15	POS	AMB	NEG	NEG	POS	POS	AMB	NEG	POS	POS	POS	NEG	NEG	NEG	NEG	NEG	NEG	NEG	NEG	NEG	NEG	POS	NEG	POS	NEG	NEG	NEG	POS	POS	POS	POS	POS	NEG	POS	NEG	NEG	NEG	NEG
04V08036	CC45-MRSA-IV [ACME+]	POS	POS	POS	NEG	POS	POS	NEG	NEG	POS	POS	POS	NEG	NEG	NEG	NEG	NEG	NEG	POS	POS	POS	POS	POS	NEG	POS	NEG	NEG	NEG	POS	POS	POS	POS	POS	NEG	POS	POS	NEG	POS	NEG
04U01119	CC45-MRSA-IV, Berlin EMRSA	POS	POS	POS	NEG	AMB	AMB	NEG	NEG	POS	POS	POS	NEG	NEG	NEG	NEG	NEG	NEG	NEG	NEG	NEG	NEG	AMB	NEG	POS	NEG	NEG	NEG	POS	POS	POS	POS	NEG	NEG	POS	NEG	NEG	AMB	NEG
04V01185	CC45-MRSA-IV, Berlin EMRSA	POS	POS	POS	NEG	AMB	AMB	NEG	NEG	POS	POS	POS	NEG	NEG	NEG	NEG	NEG	NEG	NEG	NEG	NEG	NEG	AMB	NEG	POS	NEG	NEG	NEG	POS	POS	POS	POS	NEG	NEG	POS	NEG	NEG	POS	NEG
04V06276	CC45-MRSA-IV, Berlin EMRSA	POS	POS	POS	NEG	AMB	AMB	NEG	NEG	POS	POS	POS	NEG	NEG	NEG	NEG	NEG	NEG	NEG	NEG	NEG	NEG	AMB	NEG	POS	NEG	NEG	NEG	POS	POS	POS	POS	NEG	NEG	POS	NEG	NEG	AMB	NEG
04V06677	CC45-MRSA-IV, Berlin EMRSA	POS	NEG	POS	NEG	NEG	NEG	NEG	NEG	POS	POS	POS	NEG	NEG	NEG	NEG	NEG	NEG	NEG	NEG	NEG	NEG	NEG	NEG	POS	NEG	NEG	NEG	POS	POS	POS	POS	NEG	NEG	POS	NEG	NEG	NEG	NEG
04V06688	CC45-MRSA-IV, Berlin EMRSA	POS	POS	POS	NEG	AMB	POS	NEG	NEG	POS	POS	POS	NEG	NEG	NEG	NEG	NEG	NEG	NEG	NEG	NEG	NEG	POS	NEG	POS	NEG	NEG	NEG	POS	POS	POS	POS	POS	NEG	POS	AMB	NEG	POS	NEG
04V14835	CC45-MRSA-IV, Berlin EMRSA	POS	POS	POS	NEG	POS	POS	NEG	NEG	POS	POS	POS	NEG	NEG	NEG	NEG	NEG	NEG	NEG	NEG	NEG	NEG	POS	NEG	POS	NEG	NEG	NEG	POS	POS	POS	POS	POS	NEG	POS	POS	NEG	POS	NEG
04V22079	CC45-MRSA-IV, Berlin EMRSA	POS	POS	POS	NEG	AMB	POS	NEG	NEG	POS	POS	POS	NEG	NEG	NEG	NEG	NEG	NEG	NEG	NEG	NEG	NEG	NEG	NEG	POS	NEG	NEG	NEG	POS	POS	POS	POS	AMB	NEG	POS	NEG	NEG	AMB	NEG
04V29100	CC45-MRSA-IV, Berlin EMRSA	POS	POS		NEG				NEG	POS			NEG	NEG	NEG	NEG	NEG	NEG								NEG	NEG						NEG						
04V31370	CC45-MRSA-IV, Berlin EMRSA	POS	POS	POS	NEG	NEG	NEG	NEG	NEG	POS	POS	POS	NEG	NEG	NEG	NEG	NEG	NEG	NEG	NEG	NEG	NEG	AMB	NEG	POS	NEG	NEG	NEG	POS	POS	POS	POS	NEG	NEG	POS	NEG	NEG	NEG	NEG
04V38442	CC45-MRSA-IV, Berlin EMRSA	POS	POS	POS	NEG	POS	POS	NEG	AMB	POS	POS	POS	NEG	NEG	NEG	NEG	NEG	NEG	NEG	NEG	NEG	NEG	POS	NEG	POS	NEG	NEG	NEG	POS	POS	POS	POS	POS	NEG	POS	POS	NEG	POS	NEG
04V39012	CC45-MRSA-IV, Berlin EMRSA	POS	POS	POS	NEG	POS	POS	NEG	NEG	POS	POS	POS	NEG	NEG	NEG	NEG	NEG	NEG	NEG	NEG	NEG	NEG	POS	NEG	POS	NEG	NEG	NEG	POS	POS	POS	POS	POS	NEG	POS	POS	NEG	POS	NEG
04V39577	CC45-MRSA-IV, Berlin EMRSA	POS	POS	POS	NEG	POS	POS	NEG	NEG	POS	POS	POS	NEG	NEG	NEG	NEG	NEG	NEG	NEG	NEG	NEG	NEG	POS	NEG	POS	NEG	NEG	NEG	POS	POS	POS	POS	POS	NEG	POS	AMB	NEG	POS	NEG
04V22872	CC152-MRSA-V [PVL+]	NEG	POS	POS	NEG	AMB	AMB	POS	POS	POS	NEG	POS	NEG	NEG	NEG	NEG	POS	NEG	NEG	NEG	NEG	NEG	POS	POS	NEG	NEG	NEG	NEG	POS	POS	POS	POS	NEG	NEG	NEG	NEG	POS	NEG	POS

VIRULENCE : STAPHYLOCOCCAL SUPERANTIGEN/ENTEROTOXIN-LIKE GENES (SET/SSL)

ISOLATE:	STRAIN	staphylococcal superantigen-like protein 2		staphylococcal superantigen-like protein 3			staphylococcal superantigen-like protein 4		staphylococcal superantigen-like protein 5				staphylococcal superantigen-like protein 6		staphylococcal superantigen-like protein 7		
		ssl02/set7	ssl02/set7 (MRSA252)	ssl03/set8_probe 1	ssl03/set8_probe 2	ssl03/set8 (MRSA252, SAR0424)	ssl04/set9	ssl04/set9 (MRSA252, SAR0425)	ssl05/set3_probe 1	ssl05/set3 (RF122, probe-611)	ssl05/set3_probe 2 (612)	ssl05/set3 (MRSA252)	ssl06/set21	ssl06 (NCTC8325+MW2)	ssl07/set1	ssl07/set1 (MRSA252)	ssl07/set1 (AF188836)
**2003**																	
03F01030	CC5/ST228-MRSA, South German EMRSA (truncated SCCmec element)	POS	AMB	POS	POS	NEG	POS	NEG	POS	AMB	POS	NEG	NEG	NEG	POS	AMB	AMB
03V05419	CC5/ST228-MRSA-I, South German EMRSA	POS	AMB	POS	POS	NEG	POS	NEG	POS	NEG	POS	NEG	NEG	NEG	POS	AMB	NEG
03V08986	CC5/ST228-MRSA-I, South German EMRSA	POS	AMB	POS	POS	NEG	POS	AMB	POS	AMB	POS	NEG	NEG	NEG	POS	AMB	AMB
03V39123	CC5/ST228-MRSA-I, South German EMRSA	POS	AMB	POS		NEG	POS	NEG	POS			NEG	NEG		POS	AMB	AMB
03B05186	CC5-MRSA-II, Rhine-Hesse EMRSA/UK-EMRSA-3	POS	NEG	POS	POS	NEG	POS	NEG	POS	NEG	POS	NEG	NEG	NEG	POS	AMB	NEG
03U00003	CC5-MRSA-II, Rhine-Hesse EMRSA/UK-EMRSA-3	POS	AMB	POS	POS	NEG	POS	NEG	POS	AMB	POS	NEG	NEG	NEG	POS	AMB	AMB
03V01438	CC5-MRSA-II, Rhine-Hesse EMRSA/UK-EMRSA-3	POS	AMB	POS	POS	NEG	POS	NEG	POS	AMB	POS	NEG	NEG	NEG	POS	AMB	NEG
03V05075	CC5-MRSA-II, Rhine-Hesse EMRSA/UK-EMRSA-3	POS	NEG	POS	POS	NEG	POS	NEG	POS	NEG	POS	NEG	NEG	NEG	POS	AMB	NEG
03V11014	CC5-MRSA-II, Rhine-Hesse EMRSA/UK-EMRSA-3	POS	AMB	POS	POS	NEG	POS	NEG	POS	AMB	POS	NEG	NEG	NEG	POS	AMB	AMB
03V12917	CC5-MRSA-II, Rhine-Hesse EMRSA/UK-EMRSA-3	POS	AMB	POS	POS	NEG	POS	NEG	POS	AMB	POS	NEG	NEG	NEG	POS	AMB	AMB
03V14467	CC5-MRSA-II, Rhine-Hesse EMRSA/UK-EMRSA-3	POS	AMB	POS	POS	NEG	POS	AMB	POS	AMB	POS	NEG	NEG	AMB	POS	AMB	AMB
03V19357	CC5-MRSA-II, Rhine-Hesse EMRSA/UK-EMRSA-3	POS	NEG	POS	POS	NEG	POS	NEG	POS	NEG	POS	NEG	NEG	NEG	POS	AMB	NEG
03V20186	CC5-MRSA-II, Rhine-Hesse EMRSA/UK-EMRSA-3	POS	AMB	POS	POS	NEG	POS	NEG	POS	AMB	POS	NEG	NEG	NEG	POS	AMB	NEG
03V25349	CC5-MRSA-II, Rhine-Hesse EMRSA/UK-EMRSA-3	POS	AMB	POS	POS	NEG	POS	AMB	POS	AMB	POS	NEG	NEG	NEG	POS	AMB	AMB
03V28948	CC5-MRSA-II, Rhine-Hesse EMRSA/UK-EMRSA-3	POS	NEG	POS	POS	NEG	AMB	NEG	POS	NEG	POS	NEG	NEG	NEG	POS	AMB	NEG
03V30841	CC5-MRSA-II, Rhine-Hesse EMRSA/UK-EMRSA-3	POS	NEG	POS		NEG	POS	NEG	POS			NEG	NEG		POS	AMB	NEG
03V32896	CC5-MRSA-II, Rhine-Hesse EMRSA/UK-EMRSA-3	POS	NEG	POS	POS	NEG	POS	NEG	POS	NEG	POS	NEG	NEG	NEG	POS	AMB	NEG
03V33415	CC5-MRSA-II, Rhine-Hesse EMRSA/UK-EMRSA-3	POS	AMB	POS	POS	NEG	POS	NEG	POS	AMB	POS	NEG	NEG	NEG	POS	AMB	NEG
03V34628	CC5-MRSA-II, Rhine-Hesse EMRSA/UK-EMRSA-3	POS	AMB	POS	POS	NEG	POS	AMB	POS	AMB	POS	NEG	NEG	AMB	POS	AMB	AMB
03V37078	CC5-MRSA-II, Rhine-Hesse EMRSA/UK-EMRSA-3	POS	NEG	POS	POS	NEG	POS	NEG	POS	NEG	POS	NEG	NEG	NEG	POS	AMB	NEG
03V41645	CC5-MRSA-II, Rhine-Hesse EMRSA/UK-EMRSA-3	POS	AMB	POS	POS	NEG	POS	NEG	POS	AMB	POS	NEG	NEG	NEG	POS	AMB	NEG
03V03012	CC5-MRSA-IV, Paediatric clone	POS	NEG	POS	POS	NEG	POS	NEG	POS	POS	POS	NEG	NEG	NEG	POS	AMB	NEG
03V02863	CC8/ST247-MRSA-I, North German/Iberian EMRSA	POS	NEG	POS	POS	NEG	POS	NEG	POS	AMB	POS	NEG	POS	POS	POS	AMB	NEG
03V14882	CC8/ST254-MRSA-(atypical SCCmec), Hannover EMRSA	POS	NEG	POS	POS	NEG	POS	NEG	POS	AMB	POS	NEG	POS	POS	POS	AMB	NEG
03B00404	CC22-MRSA-IV, Barnim/UK-EMRSA-15	POS	AMB	NEG	NEG	NEG	NEG	POS	POS	NEG	NEG	POS	NEG	NEG	AMB	AMB	POS
03U00892	CC22-MRSA-IV, Barnim/UK-EMRSA-15	POS	POS	NEG		POS	NEG	POS	POS			POS	NEG		AMB	AMB	POS
03V01422	CC22-MRSA-IV, Barnim/UK-EMRSA-15	AMB	POS	NEG	NEG	NEG	NEG	POS	POS	NEG	NEG	POS	NEG	NEG	AMB	AMB	POS
03V04424	CC22-MRSA-IV, Barnim/UK-EMRSA-15	AMB	POS	NEG	NEG	NEG	NEG	POS	POS	NEG	NEG	POS	NEG	NEG	AMB	AMB	POS
03V05690	CC22-MRSA-IV, Barnim/UK-EMRSA-15	POS	AMB	NEG	NEG	NEG	NEG	POS	POS	NEG	NEG	POS	NEG	NEG	NEG	AMB	POS
03V07912	CC22-MRSA-IV, Barnim/UK-EMRSA-15	POS	POS	NEG	NEG	POS	NEG	POS	POS	AMB	NEG	POS	NEG	NEG	AMB	AMB	POS
03V23798	CC22-MRSA-IV, Barnim/UK-EMRSA-15	POS	AMB	NEG	NEG	NEG	NEG	POS	POS	NEG	NEG	POS	NEG	NEG	NEG	AMB	POS
03V25914	CC22-MRSA-IV, Barnim/UK-EMRSA-15	POS	AMB	NEG	NEG	NEG	NEG	POS	POS	NEG	NEG	POS	NEG	NEG	AMB	AMB	POS
03V29751	CC22-MRSA-IV, Barnim/UK-EMRSA-15	AMB	POS	NEG	NEG	NEG	NEG	AMB	POS	NEG	NEG	POS	NEG	NEG	AMB	AMB	POS
03V30201	CC22-MRSA-IV, Barnim/UK-EMRSA-15	AMB	POS	NEG	NEG	NEG	NEG	POS	POS	NEG	NEG	POS	NEG	NEG	AMB	AMB	POS
03B01781	CC45-MRSA-IV, Berlin EMRSA	AMB	POS	NEG	NEG	NEG	NEG	AMB	NEG	NEG	NEG	POS	NEG	AMB	NEG	NEG	NEG
03U05014	CC45-MRSA-IV, Berlin EMRSA	AMB	POS	NEG	NEG	NEG	NEG	POS	NEG	NEG	NEG	POS	NEG	AMB	NEG	NEG	NEG
03U11106	CC45-MRSA-IV, Berlin EMRSA	AMB	POS	NEG	NEG	NEG	NEG	POS	NEG	NEG	NEG	POS	NEG	POS	NEG	AMB	POS
03V00742	CC45-MRSA-IV, Berlin EMRSA	NEG	POS	NEG	NEG	NEG	NEG	AMB	NEG	NEG	NEG	POS	NEG	NEG	NEG	NEG	NEG
03V01902	CC45-MRSA-IV, Berlin EMRSA	AMB	POS	NEG	NEG	NEG	NEG	POS	NEG	NEG	NEG	POS	NEG	AMB	NEG	NEG	NEG
03V02256	CC45-MRSA-IV, Berlin EMRSA	NEG	POS	NEG	NEG	NEG	NEG	POS	NEG	NEG	NEG	POS	NEG	AMB	NEG	NEG	NEG
03V02262	CC45-MRSA-IV, Berlin EMRSA	NEG	POS	NEG	NEG	NEG	NEG	POS	NEG	NEG	NEG	POS	NEG	NEG	NEG	NEG	NEG
03V04701	CC45-MRSA-IV, Berlin EMRSA	NEG	POS	NEG	NEG	NEG	NEG	AMB	NEG	NEG	NEG	POS	NEG	NEG	NEG	NEG	NEG
03V05042	CC45-MRSA-IV, Berlin EMRSA	AMB	POS	NEG	NEG	NEG	NEG	POS	NEG	NEG	NEG	POS	NEG	AMB	NEG	NEG	NEG
03V05377	CC45-MRSA-IV, Berlin EMRSA	NEG	POS	NEG	NEG	NEG	NEG	AMB	NEG	NEG	NEG	POS	NEG	NEG	NEG	NEG	NEG
03V05507	CC45-MRSA-IV, Berlin EMRSA	NEG	POS	NEG	NEG	NEG	NEG	AMB	NEG	NEG	NEG	POS	NEG	NEG	NEG	NEG	NEG
03V08214	CC45-MRSA-IV, Berlin EMRSA	AMB	POS	NEG	NEG	NEG	NEG	POS	NEG	NEG	NEG	POS	NEG	AMB	NEG	NEG	NEG
03V08525	CC45-MRSA-IV, Berlin EMRSA	AMB	POS	NEG	NEG	NEG	NEG	AMB	NEG	NEG	NEG	POS	NEG	NEG	NEG	NEG	NEG
03V11910	CC45-MRSA-IV, Berlin EMRSA	NEG	POS	NEG	NEG	NEG	NEG	AMB	NEG	NEG	NEG	AMB	NEG	NEG	NEG	NEG	NEG
03V14481	CC45-MRSA-IV, Berlin EMRSA	NEG	POS	NEG	NEG	NEG	NEG	AMB	NEG	NEG	NEG	POS	NEG	NEG	NEG	NEG	NEG
03V15082	CC45-MRSA-IV, Berlin EMRSA	AMB	POS	NEG	NEG	NEG	NEG	POS	NEG	POS	NEG	POS	NEG	POS	NEG	NEG	NEG
03V17508	CC45-MRSA-IV, Berlin EMRSA	NEG	POS	NEG	NEG	NEG	NEG	AMB	NEG	NEG	NEG	POS	NEG	NEG	NEG	NEG	NEG
03V20150	CC45-MRSA-IV, Berlin EMRSA	NEG	POS	NEG	NEG	NEG	NEG	AMB	NEG	NEG	NEG	POS	NEG	NEG	NEG	NEG	NEG
03V22437	CC45-MRSA-IV, Berlin EMRSA	NEG	POS	NEG	NEG	NEG	NEG	NEG	NEG	NEG	NEG	AMB	NEG	NEG	NEG	NEG	NEG
03V24471	CC45-MRSA-IV, Berlin EMRSA	NEG	POS	NEG	NEG	NEG	NEG	POS	NEG	NEG	NEG	POS	NEG	NEG	NEG	NEG	NEG
03V28070	CC45-MRSA-IV, Berlin EMRSA	NEG	POS	NEG	NEG	NEG	NEG	AMB	NEG	NEG	NEG	AMB	NEG	NEG	NEG	NEG	NEG
03V28448	CC45-MRSA-IV, Berlin EMRSA	AMB	POS	NEG	NEG	NEG	NEG	AMB	NEG	NEG	NEG	POS	NEG	NEG	NEG	NEG	NEG
03V34589	CC45-MRSA-IV, Berlin EMRSA	AMB	POS	NEG	NEG	NEG	NEG	AMB	NEG	NEG	NEG	POS	NEG	NEG	NEG	NEG	NEG
03V39774	CC45-MRSA-IV, Berlin EMRSA	NEG	POS	NEG	NEG	NEG	NEG	POS	NEG	NEG	NEG	POS	NEG	NEG	NEG	NEG	NEG
**2004**																	
04B05423	CC5-MRSA-II, Rhine-Hesse EMRSA/UK-EMRSA-3	POS	NEG	POS	POS	NEG	POS	NEG	POS	NEG	POS	NEG	NEG	NEG	POS	NEG	NEG
04U02171	CC5-MRSA-II, Rhine-Hesse EMRSA/UK-EMRSA-3	POS	AMB	POS	POS	NEG	POS	NEG	POS	AMB	POS	NEG	NEG	NEG	POS	AMB	AMB
04U03682	CC5-MRSA-II, Rhine-Hesse EMRSA/UK-EMRSA-3	POS	NEG	POS	POS	NEG	POS	NEG	POS	NEG	POS	NEG	NEG	NEG	POS	NEG	NEG
04U06618	CC5-MRSA-II, Rhine-Hesse EMRSA/UK-EMRSA-3	POS	AMB	POS	POS	NEG	POS	NEG	POS	AMB	POS	NEG	NEG	NEG	POS	AMB	AMB
04U12578	CC5-MRSA-II, Rhine-Hesse EMRSA/UK-EMRSA-3	POS	NEG	POS	POS	NEG	POS	NEG	POS	NEG	POS	NEG	NEG	NEG	POS	AMB	NEG
04V02465	CC5-MRSA-II, Rhine-Hesse EMRSA/UK-EMRSA-3	POS	NEG	POS	POS	NEG	POS	NEG	POS	NEG	POS	NEG	NEG	NEG	POS	AMB	NEG
04V03642	CC5-MRSA-II, Rhine-Hesse EMRSA/UK-EMRSA-3	POS	NEG	POS	POS	NEG	POS	NEG	POS	NEG	POS	NEG	NEG	NEG	POS	NEG	NEG
04V07657	CC5-MRSA-II, Rhine-Hesse EMRSA/UK-EMRSA-3	POS	NEG	POS	POS	NEG	POS	NEG	POS	NEG	POS	NEG	NEG	NEG	POS	NEG	NEG
04V12223	CC5-MRSA-II, Rhine-Hesse EMRSA/UK-EMRSA-3	POS	NEG	POS	POS	NEG	POS	NEG	POS	NEG	POS	NEG	NEG	NEG	POS	NEG	NEG
04V12529	CC5-MRSA-II, Rhine-Hesse EMRSA/UK-EMRSA-3	POS	NEG	POS	POS	NEG	POS	NEG	POS	NEG	POS	NEG	NEG	NEG	POS	NEG	NEG
04V13633	CC5-MRSA-II, Rhine-Hesse EMRSA/UK-EMRSA-3	POS	NEG	POS	POS	NEG	POS	NEG	POS	NEG	POS	NEG	NEG	NEG	POS	NEG	NEG
04V16499	CC5-MRSA-II, Rhine-Hesse EMRSA/UK-EMRSA-3	POS	AMB	POS	POS	NEG	POS	NEG	POS	AMB	POS	NEG	NEG	NEG	POS	AMB	AMB
04V17015	CC5-MRSA-II, Rhine-Hesse EMRSA/UK-EMRSA-3	POS	AMB	POS	POS	NEG	POS	NEG	POS	AMB	POS	NEG	NEG	NEG	POS	AMB	AMB
04V20684	CC5-MRSA-II, Rhine-Hesse EMRSA/UK-EMRSA-3	POS	POS	POS		NEG	POS	NEG	POS			NEG	NEG		AMB	POS	AMB
04V24615	CC5-MRSA-II, Rhine-Hesse EMRSA/UK-EMRSA-3	POS	NEG	POS	POS	NEG	POS	NEG	POS	NEG	POS	NEG	NEG	NEG	POS	NEG	NEG
04V24901	CC5-MRSA-II, Rhine-Hesse EMRSA/UK-EMRSA-3	POS	NEG	POS	POS	NEG	POS	NEG	POS	NEG	POS	NEG	NEG	NEG	POS	AMB	NEG
04V25081	CC5-MRSA-II, Rhine-Hesse EMRSA/UK-EMRSA-3	POS	NEG	POS	POS	NEG	POS	NEG	POS	NEG	POS	NEG	NEG	NEG	POS	AMB	NEG
04V27293	CC5-MRSA-II, Rhine-Hesse EMRSA/UK-EMRSA-3	POS	AMB	POS	POS	NEG	POS	NEG	POS	AMB	POS	NEG	NEG	NEG	POS	AMB	AMB
04V27311	CC5-MRSA-II, Rhine-Hesse EMRSA/UK-EMRSA-3	POS	NEG	POS	POS	NEG	POS	NEG	POS	NEG	POS	NEG	NEG	NEG	POS	NEG	NEG
04V33459	CC5-MRSA-II, Rhine-Hesse EMRSA/UK-EMRSA-3	POS	AMB	POS	POS	NEG	POS	NEG	POS	AMB	POS	NEG	NEG	NEG	POS	AMB	AMB
04V39909	CC5-MRSA-IV&VI	POS	NEG	POS	POS	NEG	POS	NEG	POS	NEG	POS	NEG	NEG	NEG	POS	AMB	AMB
04U02421	CC5-MRSA-IV, Paediatric clone	POS	NEG	POS	POS	NEG	POS	NEG	POS	POS	POS	NEG	NEG	NEG	POS	AMB	NEG
04V09807	CC80-MRSA-IV [PVL+], European caMRSA Clone	POS	NEG	POS		NEG	POS	NEG	POS			NEG	NEG		POS	NEG	NEG
04V16073	CC80-MRSA-IV [PVL+], European caMRSA Clone	POS	NEG	POS	POS	NEG	POS	NEG	POS	POS	POS	NEG	NEG	NEG	POS	NEG	NEG
04V23548	CC80-MRSA-IV [PVL+], European caMRSA Clone	POS	NEG	POS		NEG	POS	NEG	POS			NEG	NEG		POS	NEG	NEG
04V03289	CC22-MRSA-IV, Barnim/UK-EMRSA-15	POS	POS	NEG		POS	NEG	POS	POS			POS	NEG		NEG	AMB	POS
04V03847	CC22-MRSA-IV, Barnim/UK-EMRSA-15	POS	POS	NEG		NEG	NEG	POS	POS			POS	NEG		NEG	AMB	POS
04V04595	CC22-MRSA-IV, Barnim/UK-EMRSA-15	AMB	POS	NEG	NEG	NEG	NEG	AMB	POS	POS	NEG	POS	NEG	AMB	AMB	AMB	POS
04V05029	CC22-MRSA-IV, Barnim/UK-EMRSA-15	AMB	POS	NEG	NEG	NEG	NEG	AMB	AMB	NEG	NEG	POS	NEG	NEG	NEG	AMB	POS
04V07875	CC22-MRSA-IV, Barnim/UK-EMRSA-15	NEG	POS	NEG	NEG	NEG	NEG	NEG	NEG	NEG	NEG	POS	NEG	NEG	NEG	NEG	POS
04V14909	CC22-MRSA-IV, Barnim/UK-EMRSA-15	POS	AMB	NEG	NEG	NEG	NEG	POS	POS	NEG	NEG	POS	NEG	NEG	NEG	AMB	POS
04V18654	CC22-MRSA-IV, Barnim/UK-EMRSA-15	AMB	POS	NEG	NEG	NEG	NEG	POS	POS	NEG	NEG	POS	NEG	NEG	AMB	AMB	POS
04V18815	CC22-MRSA-IV, Barnim/UK-EMRSA-15	AMB	POS	AMB	NEG	AMB	NEG	POS	POS	POS	NEG	POS	NEG	POS	AMB	POS	AMB
04V29721	CC22-MRSA-IV, Barnim/UK-EMRSA-15	NEG	POS	NEG	NEG	NEG	NEG	POS	AMB	NEG	NEG	POS	NEG	NEG	NEG	NEG	POS
04V32759	CC22-MRSA-IV, Barnim/UK-EMRSA-15	AMB	POS	NEG	NEG	NEG	NEG	POS	POS	NEG	NEG	POS	NEG	NEG	NEG	AMB	POS
04V32919	CC22-MRSA-IV, Barnim/UK-EMRSA-15	AMB	POS	AMB	POS	POS	NEG	POS	AMB	POS	NEG	POS	NEG	POS	AMB	POS	AMB
04V33780	CC22-MRSA-IV, Barnim/UK-EMRSA-15	AMB	POS	NEG	NEG	AMB	NEG	POS	POS	NEG	NEG	POS	NEG	NEG	NEG	AMB	POS
04V35396	CC22-MRSA-IV, Barnim/UK-EMRSA-15	AMB	POS	AMB	NEG	AMB	NEG	POS	POS	POS	NEG	POS	NEG	POS	AMB	POS	AMB
04V37107	CC22-MRSA-IV, Barnim/UK-EMRSA-15	AMB	POS	NEG	NEG	NEG	NEG	POS	POS	NEG	NEG	POS	NEG	NEG	AMB	AMB	POS
04V37518	CC22-MRSA-IV, Barnim/UK-EMRSA-15	POS	AMB	NEG	NEG	NEG	NEG	POS	POS	NEG	NEG	POS	NEG	NEG	NEG	AMB	POS
04V38199	CC22-MRSA-IV, Barnim/UK-EMRSA-15	NEG	NEG	NEG	NEG	NEG	NEG	NEG	POS	NEG	NEG	NEG	NEG	NEG	AMB	AMB	POS
04V38358	CC22-MRSA-IV, Barnim/UK-EMRSA-15	AMB	POS	NEG	NEG	NEG	NEG	POS	POS	NEG	NEG	POS	NEG	NEG	NEG	AMB	POS
04V38902	CC22-MRSA-IV, Barnim/UK-EMRSA-15	AMB	POS	NEG	NEG	NEG	NEG	POS	AMB	NEG	NEG	POS	NEG	NEG	NEG	AMB	POS
04V39203	CC22-MRSA-IV, Barnim/UK-EMRSA-15	AMB	POS	NEG	NEG	AMB	NEG	AMB	POS	POS	NEG	POS	NEG	AMB	AMB	AMB	POS
04V40423	CC22-MRSA-IV, Barnim/UK-EMRSA-15	POS	AMB	NEG	NEG	NEG	NEG	POS	POS	NEG	NEG	POS	NEG	NEG	NEG	AMB	POS
04V08036	CC45-MRSA-IV [ACME+]	AMB	POS	NEG	NEG	NEG	NEG	POS	NEG	NEG	NEG	POS	NEG	POS	NEG	NEG	NEG
04U01119	CC45-MRSA-IV, Berlin EMRSA	NEG	POS	NEG	NEG	NEG	NEG	AMB	NEG	NEG	NEG	POS	NEG	NEG	NEG	NEG	NEG
04V01185	CC45-MRSA-IV, Berlin EMRSA	NEG	POS	NEG	NEG	NEG	NEG	AMB	NEG	NEG	NEG	POS	NEG	NEG	NEG	NEG	NEG
04V06276	CC45-MRSA-IV, Berlin EMRSA	NEG	POS	NEG	NEG	NEG	NEG	POS	NEG	NEG	NEG	POS	NEG	NEG	NEG	NEG	NEG
04V06677	CC45-MRSA-IV, Berlin EMRSA	NEG	POS	NEG	NEG	NEG	NEG	AMB	NEG	NEG	NEG	POS	NEG	NEG	NEG	NEG	NEG
04V06688	CC45-MRSA-IV, Berlin EMRSA	AMB	POS	NEG	NEG	NEG	NEG	POS	NEG	NEG	NEG	POS	NEG	NEG	NEG	NEG	NEG
04V14835	CC45-MRSA-IV, Berlin EMRSA	AMB	POS	NEG	NEG	NEG	NEG	POS	NEG	NEG	NEG	POS	NEG	POS	NEG	NEG	NEG
04V22079	CC45-MRSA-IV, Berlin EMRSA	NEG	POS	NEG	NEG	NEG	NEG	POS	NEG	NEG	NEG	POS	NEG	NEG	NEG	NEG	NEG
04V29100	CC45-MRSA-IV, Berlin EMRSA	NEG	POS	NEG		NEG	NEG	NEG	NEG			NEG	NEG		NEG	NEG	NEG
04V31370	CC45-MRSA-IV, Berlin EMRSA	NEG	POS	NEG	NEG	NEG	NEG	POS	NEG	NEG	NEG	POS	NEG	NEG	NEG	NEG	NEG
04V38442	CC45-MRSA-IV, Berlin EMRSA	AMB	POS	NEG	NEG	NEG	NEG	POS	NEG	NEG	NEG	POS	NEG	POS	NEG	NEG	NEG
04V39012	CC45-MRSA-IV, Berlin EMRSA	AMB	POS	NEG	NEG	NEG	NEG	POS	NEG	NEG	NEG	POS	NEG	POS	NEG	NEG	NEG
04V39577	CC45-MRSA-IV, Berlin EMRSA	AMB	POS	NEG	NEG	NEG	NEG	POS	NEG	NEG	NEG	POS	NEG	POS	NEG	NEG	POS
04V22872	CC152-MRSA-V [PVL+]	AMB	POS	NEG	NEG	NEG	NEG	NEG	NEG	NEG	NEG	NEG	NEG	NEG	NEG	NEG	NEG

ISOLATE:	staphylococcal superantigen-like protein 8		staphylococcal superantigen-like protein 9			staphylococcal superantigen-like protein 10			staphylococcal superantigene-like protein 11				staphylococcal exotoxin-like protein, second locus				
	ssl08/set12_probe 1	ssl08/set12_probe 2	ssl09/set5_probe 1	ssl09/set5_probe 2	ssl09/set5 (MRSA252)	ssl10/set4	ssl10 (RF122)	ssl10/set4 (MRSA252)	ssl11/set2 (COL)	ssl11+set2(Mu50+N315)	ssl11+set2(MW2+MSSA 476)	ssl11/set2 (MRSA252)	setB3	setB3 (MRSA252)	setB2	setB2 (MRSA252)	setB1
**2003**																	
03F01030	POS	POS	POS	POS	NEG	POS	NEG	AMB	NEG	POS	NEG	NEG	POS	NEG	POS	NEG	POS
03V05419	POS	POS	POS	POS	NEG	POS	NEG	NEG	NEG	POS	NEG	NEG	POS	NEG	POS	NEG	POS
03V08986	POS	POS	POS	POS	NEG	POS	NEG	AMB	NEG	POS	NEG	NEG	POS	NEG	POS	NEG	POS
03V39123	POS		POS		NEG	POS		NEG	NEG	POS	NEG	NEG	POS	NEG	POS	NEG	POS
03B05186	POS	POS	POS	POS	NEG	POS	NEG	NEG	NEG	POS	NEG	NEG	POS	NEG	POS	NEG	POS
03U00003	POS	POS	POS	POS	NEG	POS	NEG	AMB	NEG	POS	NEG	NEG	POS	NEG	POS	NEG	POS
03V01438	POS	POS	POS	POS	NEG	POS	NEG	NEG	NEG	POS	NEG	NEG	POS	NEG	POS	NEG	POS
03V05075	POS	POS	POS	POS	NEG	POS	NEG	NEG	NEG	POS	NEG	NEG	POS	NEG	POS	NEG	POS
03V11014	POS	POS	POS	POS	NEG	POS	NEG	AMB	NEG	POS	NEG	NEG	POS	NEG	POS	NEG	POS
03V12917	POS	POS	POS	POS	NEG	POS	AMB	AMB	NEG	POS	NEG	NEG	POS	NEG	POS	NEG	POS
03V14467	POS	POS	POS	POS	NEG	POS	AMB	AMB	NEG	POS	NEG	NEG	POS	NEG	POS	NEG	POS
03V19357	POS	POS	POS	POS	NEG	POS	NEG	NEG	NEG	POS	NEG	NEG	POS	NEG	POS	NEG	POS
03V20186	POS	POS	POS	POS	NEG	POS	NEG	AMB	NEG	POS	NEG	NEG	POS	NEG	POS	NEG	POS
03V25349	POS	POS	POS	POS	NEG	POS	AMB	AMB	NEG	POS	NEG	NEG	POS	NEG	POS	NEG	POS
03V28948	POS	POS	POS	POS	NEG	POS	NEG	NEG	NEG	POS	NEG	NEG	POS	NEG	POS	NEG	POS
03V30841	POS		POS		NEG	POS		NEG	NEG	POS	NEG	NEG	POS	NEG	POS	NEG	POS
03V32896	POS	POS	POS	POS	NEG	POS	NEG	NEG	NEG	POS	NEG	NEG	POS	NEG	POS	NEG	POS
03V33415	POS	POS	POS	POS	NEG	POS	NEG	AMB	NEG	POS	NEG	NEG	POS	NEG	POS	NEG	POS
03V34628	POS	POS	POS	POS	NEG	POS	AMB	AMB	NEG	POS	NEG	NEG	POS	NEG	POS	NEG	POS
03V37078	POS	POS	POS	POS	NEG	POS	NEG	NEG	NEG	POS	NEG	NEG	POS	NEG	POS	NEG	POS
03V41645	POS	POS	POS	POS	NEG	POS	NEG	AMB	NEG	POS	NEG	NEG	POS	NEG	POS	NEG	POS
03V03012	POS	POS	POS	POS	NEG	POS	NEG	NEG	POS	POS	NEG	NEG	POS	NEG	POS	NEG	POS
03V02863	POS	POS	POS	POS	NEG	POS	NEG	NEG	POS	NEG	NEG	NEG	POS	NEG	POS	NEG	POS
03V14882	POS	POS	POS	POS	NEG	POS	NEG	AMB	POS	NEG	NEG	NEG	POS	NEG	POS	NEG	POS
03B00404	NEG	NEG	POS	POS	NEG	NEG	NEG	NEG	NEG	NEG	NEG	NEG	NEG	NEG	NEG	NEG	NEG
03U00892	NEG		NEG		NEG	NEG		NEG	NEG	NEG	NEG	NEG	NEG	NEG	NEG	NEG	NEG
03V01422	NEG	NEG	POS	POS	NEG	POS	NEG	NEG	NEG	NEG	NEG	NEG	NEG	NEG	NEG	NEG	NEG
03V04424	NEG	NEG	POS	POS	NEG	POS	NEG	NEG	NEG	NEG	NEG	NEG	NEG	NEG	NEG	NEG	NEG
03V05690	NEG	NEG	AMB	POS	NEG	NEG	NEG	NEG	NEG	NEG	NEG	NEG	NEG	NEG	NEG	NEG	NEG
03V07912	NEG	NEG	RANDOM	POS	NEG	NEG	NEG	RANDOM	NEG	NEG	NEG	NEG	NEG	NEG	RANDOM	NEG	RANDOM
03V23798	NEG	NEG	AMB	POS	NEG	NEG	NEG	NEG	NEG	NEG	NEG	NEG	NEG	NEG	NEG	NEG	NEG
03V25914	NEG	NEG	AMB	POS	NEG	NEG	NEG	NEG	NEG	NEG	NEG	NEG	NEG	NEG	NEG	NEG	NEG
03V29751	NEG	NEG	POS	POS	NEG	NEG	NEG	NEG	NEG	NEG	NEG	NEG	NEG	NEG	NEG	NEG	NEG
03V30201	NEG	NEG	POS	POS	NEG	POS	NEG	NEG	NEG	NEG	NEG	NEG	NEG	NEG	NEG	NEG	NEG
03B01781	NEG	NEG	NEG	NEG	POS	AMB	NEG	POS	NEG	NEG	NEG	NEG	NEG	POS	NEG	AMB	NEG
03U05014	NEG	NEG	NEG	NEG	POS	AMB	NEG	POS	NEG	NEG	NEG	NEG	NEG	POS	NEG	NEG	NEG
03U11106	NEG	NEG	NEG	NEG	POS	AMB	AMB	POS	NEG	NEG	NEG	NEG	NEG	POS	NEG	AMB	NEG
03V00742	NEG	NEG	NEG	NEG	POS	AMB	NEG	POS	NEG	NEG	NEG	NEG	NEG	POS	NEG	NEG	NEG
03V01902	NEG	NEG	NEG	NEG	POS	AMB	NEG	POS	NEG	NEG	NEG	NEG	NEG	POS	NEG	AMB	NEG
03V02256	NEG	NEG	NEG	NEG	POS	AMB	NEG	POS	NEG	NEG	NEG	NEG	NEG	POS	NEG	AMB	NEG
03V02262	NEG	NEG	NEG	NEG	POS	AMB	NEG	POS	NEG	NEG	NEG	NEG	NEG	POS	NEG	NEG	NEG
03V04701	NEG	NEG	NEG	NEG	POS	AMB	NEG	POS	NEG	NEG	NEG	NEG	NEG	POS	NEG	NEG	NEG
03V05042	NEG	NEG	NEG	NEG	POS	AMB	NEG	POS	NEG	NEG	NEG	NEG	NEG	POS	NEG	POS	NEG
03V05377	NEG	NEG	NEG	NEG	POS	AMB	NEG	POS	NEG	NEG	NEG	NEG	NEG	POS	NEG	AMB	AMB
03V05507	NEG	NEG	NEG	NEG	POS	AMB	NEG	POS	NEG	NEG	NEG	NEG	NEG	POS	NEG	NEG	NEG
03V08214	NEG	NEG	NEG	NEG	POS	AMB	NEG	POS	NEG	NEG	NEG	NEG	NEG	POS	NEG	AMB	NEG
03V08525	NEG	NEG	NEG	NEG	POS	AMB	NEG	POS	NEG	NEG	NEG	NEG	NEG	POS	NEG	NEG	NEG
03V11910	NEG	NEG	NEG	NEG	POS	AMB	NEG	POS	NEG	NEG	NEG	NEG	NEG	POS	NEG	NEG	NEG
03V14481	NEG	NEG	NEG	NEG	POS	AMB	NEG	POS	NEG	NEG	NEG	NEG	NEG	POS	NEG	NEG	NEG
03V15082	NEG	NEG	NEG	NEG	POS	AMB	NEG	POS	NEG	NEG	NEG	NEG	NEG	POS	NEG	POS	AMB
03V17508	NEG	NEG	NEG	NEG	POS	AMB	NEG	POS	NEG	NEG	NEG	NEG	NEG	POS	NEG	NEG	NEG
03V20150	NEG	NEG	NEG	NEG	POS	AMB	NEG	POS	NEG	NEG	NEG	NEG	NEG	POS	NEG	NEG	NEG
03V22437	NEG	NEG	NEG	NEG	POS	AMB	NEG	POS	NEG	NEG	NEG	NEG	NEG	POS	NEG	NEG	NEG
03V24471	NEG	NEG	NEG	NEG	POS	AMB	NEG	POS	NEG	NEG	NEG	NEG	NEG	POS	NEG	AMB	NEG
03V28070	NEG	NEG	NEG	NEG	POS	AMB	NEG	POS	NEG	NEG	NEG	NEG	NEG	POS	NEG	NEG	NEG
03V28448	NEG	NEG	NEG	NEG	POS	AMB	NEG	POS	NEG	NEG	NEG	NEG	NEG	POS	NEG	NEG	NEG
03V34589	NEG	NEG	NEG	NEG	POS	AMB	NEG	POS	NEG	NEG	NEG	NEG	NEG	POS	NEG	NEG	NEG
03V39774	NEG	NEG	NEG	NEG	POS	AMB	NEG	POS	NEG	NEG	NEG	NEG	NEG	POS	NEG	NEG	NEG
**2004**																	
04B05423	POS	POS	POS	POS	NEG	POS	NEG	NEG	NEG	POS	NEG	NEG	POS	NEG	POS	NEG	POS
04U02171	POS	POS	POS	POS	NEG	POS	AMB	AMB	NEG	POS	NEG	NEG	POS	NEG	POS	NEG	POS
04U03682	POS	POS	POS	POS	NEG	POS	NEG	NEG	NEG	POS	NEG	NEG	POS	NEG	POS	NEG	POS
04U06618	POS	POS	POS	POS	NEG	POS	NEG	AMB	NEG	POS	NEG	NEG	POS	NEG	POS	NEG	POS
04U12578	POS	POS	POS	POS	NEG	POS	NEG	NEG	NEG	POS	NEG	NEG	POS	NEG	POS	NEG	POS
04V02465	POS	POS	POS	POS	NEG	POS	NEG	NEG	NEG	POS	NEG	NEG	POS	NEG	POS	NEG	POS
04V03642	POS	POS	POS	POS	NEG	POS	NEG	NEG	NEG	POS	NEG	NEG	POS	NEG	POS	NEG	POS
04V07657	POS	POS	POS	POS	NEG	POS	NEG	NEG	NEG	POS	NEG	NEG	POS	NEG	POS	NEG	POS
04V12223	POS	POS	POS	POS	NEG	POS	NEG	NEG	NEG	POS	NEG	NEG	POS	NEG	POS	NEG	POS
04V12529	POS	POS	POS	POS	NEG	POS	NEG	NEG	NEG	POS	NEG	NEG	POS	NEG	POS	NEG	POS
04V13633	POS	POS	POS	POS	NEG	POS	NEG	NEG	NEG	POS	NEG	NEG	POS	NEG	POS	NEG	POS
04V16499	POS	POS	POS	POS	NEG	POS	AMB	AMB	NEG	POS	NEG	NEG	POS	NEG	POS	NEG	POS
04V17015	POS	POS	POS	POS	NEG	POS	AMB	AMB	NEG	POS	NEG	NEG	POS	NEG	POS	NEG	POS
04V20684	POS		POS		NEG	POS		NEG	NEG	POS	NEG	NEG	POS	NEG	POS	NEG	POS
04V24615	POS	POS	POS	POS	NEG	POS	NEG	NEG	NEG	POS	NEG	NEG	POS	NEG	POS	NEG	POS
04V24901	POS	POS	POS	POS	NEG	POS	NEG	NEG	NEG	POS	NEG	NEG	POS	NEG	POS	NEG	POS
04V25081	POS	POS	POS	POS	NEG	POS	NEG	NEG	NEG	POS	NEG	NEG	POS	NEG	POS	NEG	POS
04V27293	POS	POS	POS	POS	NEG	POS	NEG	NEG	NEG	POS	NEG	NEG	POS	NEG	POS	NEG	POS
04V27311	POS	POS	POS	POS	NEG	POS	NEG	NEG	NEG	POS	NEG	NEG	POS	NEG	POS	NEG	POS
04V33459	POS	POS	POS	POS	NEG	POS	AMB	AMB	NEG	POS	NEG	NEG	POS	NEG	POS	NEG	POS
04V39909	POS	POS	POS	POS	NEG	POS	NEG	NEG	NEG	POS	NEG	NEG	POS	NEG	POS	NEG	POS
04U02421	POS	POS	POS	POS	NEG	POS	NEG	NEG	NEG	POS	NEG	NEG	POS	NEG	POS	NEG	POS
04V09807	POS		POS		NEG	POS		NEG	NEG	NEG	NEG	NEG	POS	NEG	POS	NEG	POS
04V16073	POS	POS	POS	POS	NEG	POS	NEG	NEG	NEG	NEG	NEG	NEG	POS	NEG	POS	NEG	POS
04V23548	POS		POS		NEG	POS		NEG	NEG	NEG	NEG	NEG	POS	NEG	POS	NEG	POS
04V03289	NEG		POS		NEG	NEG		POS	NEG	NEG	NEG	NEG	NEG	NEG	AMB	NEG	POS
04V03847	NEG		NEG		NEG	NEG		NEG	NEG	NEG	NEG	NEG	NEG	NEG	NEG	NEG	NEG
04V04595	NEG	NEG	POS	POS	NEG	POS	NEG	NEG	NEG	NEG	NEG	NEG	NEG	NEG	NEG	NEG	NEG
04V05029	NEG	NEG	NEG	NEG	NEG	NEG	NEG	NEG	NEG	NEG	NEG	NEG	NEG	NEG	NEG	NEG	NEG
04V07875	NEG	NEG	NEG	NEG	NEG	NEG	NEG	NEG	NEG	NEG	NEG	NEG	NEG	NEG	NEG	NEG	NEG
04V14909	NEG	NEG	AMB	AMB	NEG	NEG	NEG	NEG	NEG	NEG	NEG	NEG	NEG	NEG	NEG	NEG	NEG
04V18654	NEG	NEG	POS	POS	NEG	NEG	NEG	NEG	NEG	NEG	NEG	NEG	NEG	NEG	NEG	NEG	NEG
04V18815	NEG	NEG	POS	POS	NEG	POS	NEG	AMB	NEG	NEG	NEG	NEG	NEG	NEG	NEG	NEG	NEG
04V29721	NEG	NEG	NEG	NEG	NEG	NEG	NEG	NEG	NEG	NEG	NEG	NEG	NEG	NEG	NEG	NEG	NEG
04V32759	NEG	NEG	AMB	POS	NEG	NEG	NEG	NEG	NEG	NEG	NEG	NEG	NEG	NEG	NEG	NEG	NEG
04V32919	NEG	NEG	POS	POS	NEG	POS	AMB	AMB	NEG	NEG	AMB	NEG	NEG	NEG	AMB	NEG	POS
04V33780	NEG	NEG	POS	POS	NEG	AMB	NEG	NEG	NEG	NEG	NEG	NEG	NEG	NEG	NEG	NEG	NEG
04V35396	NEG	NEG	POS	POS	NEG	POS	NEG	NEG	NEG	NEG	NEG	NEG	NEG	NEG	NEG	NEG	NEG
04V37107	NEG	NEG	POS	POS	NEG	POS	NEG	NEG	NEG	NEG	NEG	NEG	NEG	NEG	NEG	NEG	NEG
04V37518	NEG	NEG	NEG	AMB	NEG	NEG	NEG	NEG	NEG	NEG	NEG	NEG	NEG	NEG	NEG	NEG	NEG
04V38199	NEG	NEG	NEG	NEG	NEG	NEG	NEG	NEG	NEG	NEG	NEG	NEG	NEG	NEG	NEG	NEG	NEG
04V38358	NEG	NEG	NEG	NEG	NEG	NEG	NEG	NEG	NEG	NEG	NEG	NEG	NEG	NEG	NEG	NEG	NEG
04V38902	NEG	NEG	NEG	NEG	NEG	NEG	NEG	NEG	NEG	NEG	NEG	NEG	NEG	NEG	NEG	NEG	NEG
04V39203	NEG	NEG	POS	POS	NEG	POS	NEG	NEG	NEG	NEG	NEG	NEG	NEG	NEG	NEG	NEG	NEG
04V40423	NEG	NEG	NEG	NEG	NEG	NEG	NEG	NEG	NEG	NEG	NEG	NEG	NEG	NEG	NEG	NEG	NEG
04V08036	NEG	NEG	NEG	NEG	POS	AMB	AMB	POS	NEG	NEG	NEG	NEG	NEG	POS	NEG	AMB	AMB
04U01119	NEG	NEG	NEG	NEG	POS	POS	NEG	AMB	NEG	NEG	NEG	NEG	NEG	POS	NEG	NEG	NEG
04V01185	NEG	NEG	NEG	NEG	POS	AMB	NEG	POS	NEG	NEG	NEG	NEG	NEG	POS	NEG	NEG	NEG
04V06276	NEG	NEG	NEG	NEG	POS	AMB	NEG	POS	NEG	NEG	NEG	NEG	NEG	POS	NEG	NEG	NEG
04V06677	NEG	NEG	NEG	NEG	POS	AMB	NEG	POS	NEG	NEG	NEG	NEG	NEG	POS	NEG	NEG	NEG
04V06688	NEG	NEG	NEG	NEG	POS	AMB	NEG	POS	NEG	NEG	NEG	NEG	NEG	POS	NEG	NEG	NEG
04V14835	NEG	NEG	NEG	NEG	POS	AMB	NEG	POS	NEG	NEG	NEG	NEG	NEG	POS	NEG	AMB	NEG
04V22079	NEG	NEG	NEG	NEG	POS	AMB	NEG	POS	NEG	NEG	NEG	NEG	NEG	POS	NEG	NEG	NEG
04V29100	NEG		NEG		POS	NEG		POS	NEG	NEG	NEG	NEG	NEG	POS	NEG	NEG	NEG
04V31370	NEG	NEG	NEG	NEG	POS	AMB	NEG	POS	NEG	NEG	NEG	NEG	NEG	POS	NEG	POS	AMB
04V38442	NEG	NEG	NEG	NEG	POS	AMB	NEG	POS	NEG	NEG	NEG	NEG	NEG	POS	NEG	AMB	NEG
04V39012	NEG	NEG	NEG	NEG	POS	AMB	NEG	POS	NEG	NEG	NEG	NEG	NEG	POS	NEG	AMB	NEG
04V39577	NEG	NEG	NEG	NEG	POS	AMB	NEG	POS	NEG	NEG	NEG	NEG	NEG	POS	NEG	NEG	NEG
04V22872	NEG	NEG	NEG	NEG	NEG	POS	NEG	AMB	NEG	NEG	NEG	NEG	POS	NEG	POS	NEG	POS

CAPSULE- AND BIOFILM-ASSOCIATED GENES

ISOLATE:	Capsule type 1	capsular poly-saccharide synthesis enzyme	O-antigen poly-merase	capsular poly-saccharide biosyn- thesis protein	Capsule type 5	capsular poly-saccharide synthesis enzyme	O-antigen poly-merase	capsular poly-saccharide biosyn- thesis protein	Capsule type 8	capsular poly-saccharide synthesis enzyme	capsular poly-saccharide biosyn- thesis protein	O-antigen poly-merase	capsular poly-saccharide biosyn- thesis protein	intercellular adhesion protein A	intercellular adhesion protein C	biofilm PIA synthesis protein D	surface protein involved in biofilm formation
	cap 1 (total)	capH1	capJ1	capK1	cap 5 (total)	capH5	capJ5	capK5	cap 8 (total)	capH8	capI8	capJ8	capK8	icaA	icaC	icaD	bap
**2003**																	
03F01030	NEG	NEG	NEG	NEG	POS	POS	POS	POS	NEG	NEG	NEG	NEG	NEG	POS	POS	POS	NEG
03V05419	NEG	NEG	NEG	NEG	POS	POS	POS	POS	NEG	NEG	NEG	NEG	NEG	POS	POS	POS	NEG
03V08986	NEG	NEG	NEG	NEG	POS	POS	POS	POS	NEG	NEG	NEG	NEG	NEG	POS	POS	POS	NEG
03V39123																	
03B05186	NEG	NEG	NEG	NEG	POS	POS	POS	POS	NEG	NEG	NEG	NEG	NEG	POS	POS	POS	NEG
03U00003	NEG	NEG	NEG	NEG	POS	POS	POS	POS	NEG	NEG	NEG	NEG	NEG	POS	POS	POS	NEG
03V01438	NEG	NEG	NEG	NEG	POS	POS	POS	POS	NEG	NEG	NEG	NEG	NEG	POS	POS	POS	NEG
03V05075	NEG	NEG	NEG	NEG	POS	POS	POS	POS	NEG	NEG	NEG	NEG	NEG	POS	POS	POS	NEG
03V11014	NEG	NEG	NEG	NEG	POS	POS	POS	POS	NEG	NEG	NEG	NEG	NEG	POS	POS	POS	NEG
03V12917	NEG	NEG	NEG	NEG	POS	POS	POS	POS	NEG	NEG	NEG	NEG	NEG	POS	POS	POS	NEG
03V14467	NEG	NEG	AMB	NEG	POS	POS	POS	POS	NEG	NEG	NEG	NEG	NEG	POS	POS	POS	NEG
03V19357	NEG	NEG	NEG	NEG	POS	POS	POS	POS	NEG	NEG	NEG	NEG	NEG	POS	POS	POS	NEG
03V20186	NEG	NEG	NEG	NEG	POS	POS	POS	POS	NEG	NEG	NEG	NEG	NEG	POS	POS	POS	NEG
03V25349	NEG	NEG	AMB	NEG	POS	POS	POS	POS	NEG	NEG	NEG	NEG	NEG	POS	POS	POS	NEG
03V28948	NEG	NEG	NEG	NEG	POS	POS	POS	POS	NEG	NEG	NEG	NEG	NEG	POS	POS	POS	NEG
03V30841																	
03V32896	NEG	NEG	NEG	NEG	POS	POS	POS	POS	NEG	NEG	NEG	NEG	NEG	POS	POS	POS	NEG
03V33415	NEG	NEG	NEG	NEG	POS	POS	POS	POS	NEG	NEG	NEG	NEG	NEG	POS	POS	POS	NEG
03V34628	NEG	NEG	POS	NEG	POS	POS	POS	POS	NEG	NEG	NEG	NEG	NEG	POS	POS	POS	NEG
03V37078	NEG	NEG	NEG	NEG	POS	POS	POS	POS	NEG	NEG	NEG	NEG	NEG	POS	POS	POS	NEG
03V41645	NEG	NEG	NEG	NEG	POS	POS	POS	POS	NEG	NEG	NEG	NEG	NEG	POS	POS	POS	NEG
03V03012	NEG	NEG	NEG	NEG	POS	POS	POS	POS	NEG	NEG	NEG	NEG	NEG	POS	POS	POS	NEG
03V02863	NEG	NEG	NEG	NEG	POS	POS	POS	POS	NEG	NEG	NEG	NEG	NEG	POS	POS	POS	NEG
03V14882	NEG	NEG	NEG	NEG	POS	POS	POS	POS	NEG	NEG	NEG	NEG	NEG	POS	POS	POS	NEG
03B00404	NEG	NEG	NEG	NEG	POS	POS	POS	POS	NEG	NEG	NEG	NEG	NEG	POS	POS	POS	NEG
03U00892																	
03V01422	NEG	NEG	NEG	NEG	POS	POS	POS	POS	NEG	NEG	NEG	NEG	NEG	POS	POS	POS	NEG
03V04424	NEG	NEG	NEG	NEG	POS	POS	POS	POS	NEG	NEG	NEG	NEG	NEG	POS	POS	POS	NEG
03V05690	NEG	NEG	NEG	NEG	POS	POS	POS	POS	NEG	NEG	NEG	NEG	NEG	POS	POS	POS	NEG
03V07912	NEG	NEG	NEG	NEG	POS	POS	POS	POS	NEG	NEG	NEG	NEG	NEG	POS	POS	POS	NEG
03V23798	NEG	NEG	NEG	NEG	POS	POS	POS	POS	NEG	NEG	NEG	NEG	NEG	POS	POS	POS	NEG
03V25914	NEG	NEG	NEG	NEG	POS	POS	POS	POS	NEG	NEG	NEG	NEG	NEG	POS	POS	POS	NEG
03V29751	NEG	NEG	NEG	NEG	POS	POS	POS	POS	NEG	NEG	NEG	NEG	NEG	POS	POS	POS	NEG
03V30201	NEG	NEG	NEG	NEG	POS	POS	POS	POS	NEG	NEG	NEG	NEG	NEG	POS	POS	POS	NEG
03B01781	NEG	NEG	NEG	NEG	NEG	NEG	NEG	NEG	POS	POS	POS	POS	POS	POS	POS	POS	NEG
03U05014	NEG	NEG	NEG	NEG	NEG	NEG	NEG	NEG	POS	POS	POS	POS	POS	POS	POS	POS	NEG
03U11106	NEG	NEG	NEG	NEG	NEG	NEG	NEG	NEG	POS	POS	POS	POS	POS	POS	POS	POS	NEG
03V00742	NEG	NEG	NEG	NEG	NEG	NEG	NEG	NEG	POS	POS	POS	POS	POS	POS	POS	POS	NEG
03V01902	NEG	NEG	NEG	NEG	NEG	NEG	NEG	NEG	POS	POS	POS	POS	POS	POS	POS	POS	NEG
03V02256	NEG	NEG	NEG	NEG	NEG	NEG	NEG	NEG	POS	POS	POS	POS	POS	POS	POS	POS	NEG
03V02262	NEG	NEG	NEG	NEG	NEG	NEG	NEG	NEG	POS	POS	POS	POS	POS	POS	POS	POS	NEG
03V04701	NEG	NEG	NEG	NEG	NEG	NEG	NEG	NEG	POS	POS	POS	POS	POS	POS	POS	POS	NEG
03V05042	NEG	NEG	NEG	NEG	NEG	NEG	NEG	NEG	POS	POS	POS	POS	POS	POS	POS	POS	NEG
03V05377	NEG	NEG	NEG	NEG	NEG	NEG	NEG	NEG	POS	POS	POS	POS	POS	POS	POS	POS	NEG
03V05507	NEG	NEG	NEG	NEG	NEG	NEG	NEG	NEG	POS	POS	POS	POS	POS	POS	POS	POS	NEG
03V08214	NEG	NEG	NEG	NEG	NEG	NEG	NEG	NEG	POS	POS	POS	POS	POS	POS	POS	POS	NEG
03V08525	NEG	NEG	NEG	NEG	NEG	NEG	NEG	NEG	POS	POS	POS	POS	POS	POS	POS	POS	NEG
03V11910	NEG	NEG	NEG	NEG	NEG	NEG	NEG	NEG	POS	POS	POS	POS	POS	POS	POS	POS	NEG
03V14481	NEG	NEG	NEG	NEG	NEG	NEG	NEG	NEG	POS	POS	POS	POS	POS	POS	POS	POS	NEG
03V15082	NEG	NEG	NEG	NEG	NEG	NEG	NEG	NEG	POS	POS	POS	POS	POS	POS	POS	POS	NEG
03V17508	NEG	NEG	NEG	NEG	NEG	NEG	NEG	NEG	POS	POS	POS	POS	POS	POS	POS	POS	NEG
03V20150	NEG	NEG	NEG	NEG	NEG	NEG	NEG	NEG	POS	POS	POS	POS	POS	POS	POS	POS	NEG
03V22437	NEG	NEG	NEG	NEG	NEG	NEG	NEG	NEG	POS	POS	POS	POS	POS	POS	POS	POS	NEG
03V24471	NEG	NEG	NEG	NEG	NEG	NEG	NEG	NEG	POS	POS	POS	POS	POS	POS	POS	POS	NEG
03V28070	NEG	NEG	NEG	NEG	NEG	NEG	NEG	NEG	POS	AMB	POS	POS	POS	POS	POS	POS	NEG
03V28448	NEG	NEG	NEG	NEG	NEG	NEG	NEG	NEG	POS	POS	POS	POS	POS	POS	POS	POS	NEG
03V34589	NEG	NEG	NEG	NEG	NEG	NEG	NEG	NEG	POS	POS	POS	POS	POS	POS	POS	POS	NEG
03V39774	NEG	NEG	NEG	NEG	NEG	NEG	NEG	NEG	POS	POS	POS	POS	POS	POS	POS	POS	NEG
**2004**																	
04B05423	NEG	NEG	NEG	NEG	POS	POS	POS	POS	NEG	NEG	NEG	NEG	NEG	POS	POS	POS	NEG
04U02171	NEG	NEG	AMB	NEG	POS	POS	POS	POS	NEG	NEG	NEG	NEG	NEG	POS	POS	POS	NEG
04U03682	NEG	NEG	NEG	NEG	POS	POS	POS	POS	NEG	NEG	NEG	NEG	NEG	POS	POS	POS	NEG
04U06618	NEG	NEG	AMB	NEG	POS	POS	POS	POS	NEG	NEG	NEG	NEG	NEG	POS	POS	POS	NEG
04U12578	NEG	NEG	NEG	NEG	POS	POS	POS	POS	NEG	NEG	NEG	NEG	NEG	POS	POS	POS	NEG
04V02465	NEG	NEG	NEG	NEG	POS	POS	POS	POS	NEG	NEG	NEG	NEG	NEG	POS	POS	POS	NEG
04V03642	NEG	NEG	NEG	NEG	POS	POS	POS	POS	NEG	NEG	NEG	NEG	NEG	POS	POS	POS	NEG
04V07657	NEG	NEG	NEG	NEG	POS	POS	POS	POS	NEG	NEG	NEG	NEG	NEG	POS	POS	POS	NEG
04V12223	NEG	NEG	NEG	NEG	POS	POS	POS	POS	NEG	NEG	NEG	NEG	NEG	POS	POS	POS	NEG
04V12529	NEG	NEG	NEG	NEG	POS	POS	POS	POS	NEG	NEG	NEG	NEG	NEG	POS	POS	POS	NEG
04V13633	NEG	NEG	NEG	NEG	POS	POS	POS	POS	NEG	NEG	NEG	NEG	NEG	POS	POS	POS	NEG
04V16499	NEG	NEG	AMB	NEG	POS	POS	POS	POS	NEG	NEG	NEG	NEG	NEG	POS	POS	POS	NEG
04V17015	NEG	NEG	AMB	NEG	POS	POS	POS	POS	NEG	NEG	NEG	NEG	NEG	POS	POS	POS	NEG
04V20684																	
04V24615	NEG	NEG	NEG	NEG	POS	POS	POS	POS	NEG	NEG	NEG	NEG	NEG	POS	POS	POS	NEG
04V24901	NEG	NEG	NEG	NEG	POS	POS	POS	POS	NEG	NEG	NEG	NEG	NEG	POS	POS	POS	NEG
04V25081	NEG	NEG	NEG	NEG	POS	POS	POS	POS	NEG	NEG	NEG	NEG	NEG	POS	POS	POS	NEG
04V27293	NEG	NEG	NEG	NEG	POS	POS	POS	POS	NEG	NEG	NEG	NEG	NEG	POS	POS	POS	NEG
04V27311	NEG	NEG	NEG	NEG	POS	POS	POS	POS	NEG	NEG	NEG	NEG	NEG	POS	POS	POS	NEG
04V33459	NEG	NEG	AMB	NEG	POS	POS	POS	POS	NEG	NEG	NEG	NEG	NEG	POS	POS	POS	NEG
04V39909	NEG	NEG	NEG	NEG	POS	POS	POS	POS	NEG	NEG	NEG	NEG	NEG	POS	POS	POS	NEG
04U02421	NEG	NEG	NEG	NEG	POS	POS	POS	POS	NEG	NEG	NEG	NEG	NEG	POS	POS	POS	NEG
04V09807																	
04V16073	NEG	NEG	NEG	NEG	NEG	NEG	NEG	NEG	POS	POS	POS	POS	POS	POS	POS	POS	NEG
04V23548																	
04V03289																	
04V03847																	
04V04595	NEG	NEG	NEG	NEG	POS	POS	POS	POS	NEG	NEG	NEG	NEG	NEG	POS	POS	POS	NEG
04V05029	NEG	NEG	NEG	NEG	POS	POS	POS	POS	NEG	NEG	NEG	NEG	NEG	POS	NEG	POS	NEG
04V07875	NEG	NEG	NEG	NEG	POS	POS	AMB	POS	NEG	NEG	NEG	NEG	NEG	AMB	NEG	POS	NEG
04V14909	NEG	NEG	NEG	NEG	POS	POS	POS	POS	NEG	NEG	NEG	NEG	NEG	POS	AMB	POS	NEG
04V18654	NEG	NEG	NEG	NEG	POS	POS	POS	POS	NEG	NEG	NEG	NEG	NEG	POS	POS	POS	NEG
04V18815	NEG	NEG	AMB	NEG	POS	POS	POS	POS	NEG	NEG	NEG	NEG	NEG	POS	POS	POS	NEG
04V29721	NEG	NEG	NEG	NEG	POS	POS	POS	POS	NEG	NEG	NEG	NEG	NEG	POS	AMB	POS	NEG
04V32759	NEG	NEG	NEG	NEG	POS	POS	POS	POS	NEG	NEG	NEG	NEG	NEG	POS	POS	POS	NEG
04V32919	NEG	NEG	POS	NEG	POS	POS	POS	POS	NEG	NEG	NEG	NEG	NEG	POS	POS	POS	NEG
04V33780	NEG	NEG	NEG	NEG	POS	POS	POS	POS	NEG	NEG	NEG	NEG	NEG	POS	POS	POS	NEG
04V35396	NEG	NEG	AMB	NEG	POS	POS	POS	POS	NEG	NEG	NEG	NEG	NEG	POS	POS	POS	NEG
04V37107	NEG	NEG	NEG	NEG	POS	POS	POS	POS	NEG	NEG	NEG	NEG	NEG	POS	POS	POS	NEG
04V37518	NEG	NEG	NEG	NEG	POS	POS	POS	POS	NEG	NEG	NEG	NEG	NEG	POS	AMB	POS	NEG
04V38199	NEG	NEG	NEG	NEG	POS	POS	POS	POS	NEG	NEG	NEG	NEG	NEG	POS	AMB	POS	NEG
04V38358	NEG	NEG	NEG	NEG	POS	POS	POS	POS	NEG	NEG	NEG	NEG	NEG	POS	AMB	POS	NEG
04V38902	NEG	NEG	NEG	NEG	POS	POS	POS	POS	NEG	NEG	NEG	NEG	NEG	POS	NEG	POS	NEG
04V39203	NEG	NEG	AMB	NEG	POS	POS	POS	POS	NEG	NEG	NEG	NEG	NEG	POS	POS	POS	NEG
04V40423	NEG	NEG	NEG	NEG	POS	POS	POS	POS	NEG	NEG	NEG	NEG	NEG	POS	AMB	POS	NEG
04V08036	NEG	NEG	NEG	NEG	NEG	NEG	NEG	NEG	POS	POS	POS	POS	POS	POS	POS	POS	NEG
04U01119	NEG	NEG	NEG	NEG	NEG	NEG	NEG	NEG	POS	POS	POS	POS	POS	POS	POS	POS	NEG
04V01185	NEG	NEG	NEG	NEG	NEG	NEG	NEG	NEG	POS	POS	POS	POS	POS	POS	POS	POS	NEG
04V06276	NEG	NEG	NEG	NEG	NEG	NEG	NEG	NEG	POS	POS	POS	POS	POS	POS	POS	POS	NEG
04V06677	NEG	NEG	NEG	NEG	NEG	NEG	NEG	NEG	POS	POS	POS	POS	POS	POS	POS	POS	NEG
04V06688	NEG	NEG	NEG	NEG	NEG	NEG	NEG	NEG	POS	POS	POS	POS	POS	POS	POS	POS	NEG
04V14835	NEG	NEG	NEG	NEG	NEG	NEG	NEG	NEG	POS	POS	POS	POS	POS	POS	POS	POS	NEG
04V22079	NEG	NEG	NEG	NEG	NEG	NEG	NEG	NEG	POS	POS	POS	POS	POS	POS	POS	POS	NEG
04V29100																	
04V31370	NEG	NEG	NEG	NEG	NEG	NEG	NEG	NEG	POS	POS	POS	POS	POS	POS	POS	POS	NEG
04V38442	NEG	NEG	NEG	NEG	NEG	NEG	NEG	NEG	POS	POS	POS	POS	POS	POS	POS	POS	NEG
04V39012	NEG	NEG	NEG	NEG	NEG	NEG	NEG	NEG	POS	POS	POS	POS	POS	POS	POS	POS	NEG
04V39577	NEG	NEG	NEG	NEG	AMB	AMB	AMB	NEG	POS	POS	POS	POS	POS	POS	POS	POS	NEG
04V22872	NEG	AMB	NEG	NEG	POS	POS	POS	POS	NEG	NEG	NEG	NEG	NEG	POS	NEG	POS	NEG

ADHAESION FACTORS / GENES ENCODING MICROBIAL SURFACE COMPONENTS RECOGNIZING ADHESIVE MATRIX MOLECULES (MSCRAMM GENES)

ISOLATE:	STRAIN	bone sialoprotein-binding protein							clumping factor A					clumping factor B					collagen-binding adhesin	cell wall associated fibronectin-binding protein
		bbp (total)	bbp (consensus)	bbp (COL+MW2)	bbp (MRSA252)	bbp (Mu50)	bbp (RF122)	bbp (ST45)	clfA (total)	clfA (consensus)	clfA (COL+RF122)	clfA (MRSA252)	clfA (Mu50+MW2)	clfB (total)	clfB (consensus)	clfB (COL+Mu50)	clfB (MW2)	clfB (RF122)	cna	ebh (consensus)
**2003**																				
03F01030	CC5/ST228-MRSA, South German EMRSA (truncated SCCmec element)	POS	POS	AMB	NEG	POS	NEG	NEG	POS	POS	NEG	NEG	POS	POS	POS	POS	NEG	NEG	NEG	POS
03V05419	CC5/ST228-MRSA-I, South German EMRSA	POS	POS	NEG	NEG	POS	NEG	NEG	POS	POS	NEG	NEG	POS	POS	POS	POS	NEG	NEG	NEG	POS
03V08986	CC5/ST228-MRSA-I, South German EMRSA	POS	POS	AMB	NEG	POS	NEG	AMB	POS	POS	NEG	NEG	POS	POS	POS	POS	NEG	NEG	NEG	POS
03V39123	CC5/ST228-MRSA-I, South German EMRSA																			
03B05186	CC5-MRSA-II, Rhine-Hesse EMRSA/UK-EMRSA-3	POS	POS	NEG	NEG	POS	NEG	NEG	POS	POS	NEG	NEG	POS	POS	POS	POS	NEG	NEG	NEG	POS
03U00003	CC5-MRSA-II, Rhine-Hesse EMRSA/UK-EMRSA-3	POS	POS	NEG	NEG	POS	NEG	NEG	POS	POS	NEG	NEG	POS	POS	POS	POS	NEG	NEG	NEG	POS
03V01438	CC5-MRSA-II, Rhine-Hesse EMRSA/UK-EMRSA-3	POS	POS	NEG	NEG	POS	NEG	NEG	POS	POS	NEG	NEG	POS	POS	POS	POS	NEG	NEG	NEG	POS
03V05075	CC5-MRSA-II, Rhine-Hesse EMRSA/UK-EMRSA-3	POS	POS	NEG	NEG	POS	NEG	NEG	POS	POS	NEG	NEG	POS	POS	POS	POS	NEG	NEG	NEG	POS
03V11014	CC5-MRSA-II, Rhine-Hesse EMRSA/UK-EMRSA-3	POS	POS	NEG	NEG	POS	NEG	NEG	POS	POS	NEG	NEG	POS	POS	POS	POS	NEG	NEG	NEG	POS
03V12917	CC5-MRSA-II, Rhine-Hesse EMRSA/UK-EMRSA-3	POS	POS	NEG	NEG	POS	NEG	NEG	POS	POS	NEG	NEG	POS	POS	POS	POS	NEG	NEG	NEG	POS
03V14467	CC5-MRSA-II, Rhine-Hesse EMRSA/UK-EMRSA-3	POS	POS	AMB	NEG	POS	NEG	NEG	POS	POS	NEG	NEG	POS	POS	POS	POS	NEG	NEG	NEG	POS
03V19357	CC5-MRSA-II, Rhine-Hesse EMRSA/UK-EMRSA-3	POS	POS	NEG	NEG	POS	NEG	NEG	POS	POS	NEG	NEG	POS	POS	POS	POS	NEG	NEG	NEG	POS
03V20186	CC5-MRSA-II, Rhine-Hesse EMRSA/UK-EMRSA-3	POS	POS	NEG	NEG	POS	NEG	NEG	POS	POS	NEG	NEG	POS	POS	POS	POS	NEG	NEG	NEG	POS
03V25349	CC5-MRSA-II, Rhine-Hesse EMRSA/UK-EMRSA-3	POS	POS	AMB	NEG	POS	NEG	AMB	POS	POS	NEG	NEG	POS	POS	POS	POS	NEG	NEG	NEG	POS
03V28948	CC5-MRSA-II, Rhine-Hesse EMRSA/UK-EMRSA-3	POS	POS	NEG	NEG	POS	NEG	NEG	POS	POS	NEG	NEG	POS	POS	POS	POS	NEG	NEG	NEG	POS
03V30841	CC5-MRSA-II, Rhine-Hesse EMRSA/UK-EMRSA-3																			
03V32896	CC5-MRSA-II, Rhine-Hesse EMRSA/UK-EMRSA-3	POS	POS	NEG	NEG	POS	NEG	NEG	POS	POS	NEG	NEG	POS	POS	POS	POS	NEG	NEG	NEG	POS
03V33415	CC5-MRSA-II, Rhine-Hesse EMRSA/UK-EMRSA-3	POS	POS	NEG	NEG	POS	NEG	NEG	POS	POS	NEG	NEG	POS	POS	POS	POS	NEG	NEG	NEG	POS
03V34628	CC5-MRSA-II, Rhine-Hesse EMRSA/UK-EMRSA-3	POS	POS	AMB	NEG	POS	NEG	AMB	POS	POS	NEG	NEG	POS	POS	POS	POS	NEG	NEG	NEG	POS
03V37078	CC5-MRSA-II, Rhine-Hesse EMRSA/UK-EMRSA-3	POS	POS	NEG	NEG	POS	NEG	NEG	POS	POS	NEG	NEG	POS	POS	POS	POS	NEG	NEG	NEG	POS
03V41645	CC5-MRSA-II, Rhine-Hesse EMRSA/UK-EMRSA-3	POS	POS	NEG	NEG	POS	NEG	NEG	POS	POS	NEG	NEG	POS	POS	POS	POS	NEG	NEG	NEG	POS
03V03012	CC5-MRSA-IV, Paediatric clone	POS	POS	NEG	NEG	POS	NEG	NEG	POS	POS	NEG	NEG	POS	POS	POS	POS	NEG	NEG	NEG	POS
03V02863	CC8/ST247-MRSA-I, North German/Iberian EMRSA	NEG	NEG	NEG	NEG	NEG	NEG	NEG	POS	POS	POS	AMB	AMB	POS	POS	POS	NEG	NEG	NEG	POS
03V14882	CC8/ST254-MRSA-(atypical SCCmec), Hannover EMRSA	NEG	NEG	NEG	NEG	NEG	NEG	NEG	POS	POS	POS	AMB	AMB	POS	POS	POS	NEG	NEG	NEG	POS
03B00404	CC22-MRSA-IV, Barnim/UK-EMRSA-15	POS	POS	NEG	NEG	NEG	NEG	NEG	POS	POS	NEG	NEG	POS	POS	POS	NEG	NEG	POS	POS	NEG
03U00892	CC22-MRSA-IV, Barnim/UK-EMRSA-15																			
03V01422	CC22-MRSA-IV, Barnim/UK-EMRSA-15	POS	POS	NEG	NEG	NEG	NEG	NEG	POS	POS	NEG	NEG	POS	POS	POS	NEG	NEG	NEG	POS	NEG
03V04424	CC22-MRSA-IV, Barnim/UK-EMRSA-15	POS	POS	NEG	NEG	NEG	NEG	NEG	POS	POS	NEG	NEG	POS	POS	POS	NEG	NEG	POS	POS	NEG
03V05690	CC22-MRSA-IV, Barnim/UK-EMRSA-15	POS	POS	NEG	NEG	NEG	NEG	NEG	POS	POS	NEG	NEG	POS	POS	POS	NEG	NEG	NEG	POS	NEG
03V07912	CC22-MRSA-IV, Barnim/UK-EMRSA-15	POS	POS	NEG	NEG	NEG	NEG	NEG	POS	POS	NEG	NEG	POS	POS	POS	NEG	NEG	POS	POS	NEG
03V23798	CC22-MRSA-IV, Barnim/UK-EMRSA-15	POS	POS	NEG	NEG	NEG	NEG	NEG	POS	POS	NEG	NEG	POS	POS	POS	NEG	NEG	NEG	POS	NEG
03V25914	CC22-MRSA-IV, Barnim/UK-EMRSA-15	POS	POS	NEG	NEG	NEG	NEG	NEG	POS	POS	NEG	NEG	POS	POS	POS	NEG	NEG	NEG	POS	NEG
03V29751	CC22-MRSA-IV, Barnim/UK-EMRSA-15	POS	POS	NEG	NEG	NEG	NEG	NEG	POS	POS	NEG	NEG	POS	POS	POS	NEG	NEG	NEG	POS	NEG
03V30201	CC22-MRSA-IV, Barnim/UK-EMRSA-15	POS	POS	NEG	NEG	NEG	NEG	NEG	POS	POS	NEG	NEG	POS	POS	POS	NEG	NEG	POS	POS	NEG
03B01781	CC45-MRSA-IV, Berlin EMRSA	POS	POS	NEG	NEG	AMB	NEG	POS	POS	POS	AMB	NEG	POS	POS	POS	NEG	POS	AMB	POS	POS
03U05014	CC45-MRSA-IV, Berlin EMRSA	POS	POS	NEG	NEG	NEG	NEG	POS	POS	POS	AMB	NEG	POS	POS	POS	NEG	POS	AMB	POS	POS
03U11106	CC45-MRSA-IV, Berlin EMRSA	POS	NEG	NEG	NEG	NEG	NEG	POS	POS	POS	POS	NEG	AMB	POS	POS	NEG	POS	AMB	POS	POS
03V00742	CC45-MRSA-IV, Berlin EMRSA	POS	POS	NEG	NEG	NEG	NEG	POS	POS	POS	AMB	NEG	POS	POS	POS	NEG	POS	AMB	POS	POS
03V01902	CC45-MRSA-IV, Berlin EMRSA	POS	POS	NEG	NEG	NEG	NEG	POS	POS	POS	AMB	NEG	POS	POS	POS	NEG	POS	AMB	POS	POS
03V02256	CC45-MRSA-IV, Berlin EMRSA	POS	POS	NEG	NEG	NEG	NEG	POS	POS	POS	AMB	NEG	POS	POS	POS	NEG	POS	AMB	POS	POS
03V02262	CC45-MRSA-IV, Berlin EMRSA	POS	POS	NEG	NEG	NEG	NEG	POS	POS	POS	AMB	NEG	POS	POS	POS	NEG	POS	AMB	POS	POS
03V04701	CC45-MRSA-IV, Berlin EMRSA	POS	POS	NEG	NEG	NEG	NEG	POS	POS	POS	AMB	NEG	POS	POS	POS	NEG	POS	AMB	POS	POS
03V05042	CC45-MRSA-IV, Berlin EMRSA	POS	POS	AMB	NEG	AMB	NEG	POS	POS	POS	AMB	NEG	POS	POS	POS	NEG	POS	AMB	POS	POS
03V05377	CC45-MRSA-IV, Berlin EMRSA	POS	POS	NEG	NEG	NEG	NEG	POS	POS	POS	AMB	NEG	POS	POS	POS	NEG	POS	AMB	POS	POS
03V05507	CC45-MRSA-IV, Berlin EMRSA	POS	POS	NEG	NEG	NEG	NEG	POS	POS	POS	AMB	NEG	POS	POS	POS	NEG	POS	AMB	POS	POS
03V08214	CC45-MRSA-IV, Berlin EMRSA	POS	POS	AMB	NEG	NEG	NEG	POS	POS	POS	AMB	NEG	POS	POS	POS	NEG	POS	AMB	POS	POS
03V08525	CC45-MRSA-IV, Berlin EMRSA	POS	POS	NEG	NEG	NEG	NEG	POS	POS	POS	AMB	NEG	POS	POS	POS	NEG	POS	AMB	POS	POS
03V11910	CC45-MRSA-IV, Berlin EMRSA	POS	POS	NEG	NEG	NEG	NEG	POS	POS	POS	NEG	NEG	POS	POS	POS	NEG	POS	AMB	POS	POS
03V14481	CC45-MRSA-IV, Berlin EMRSA	POS	POS	NEG	NEG	NEG	NEG	POS	POS	POS	AMB	NEG	POS	POS	POS	NEG	POS	AMB	POS	POS
03V15082	CC45-MRSA-IV, Berlin EMRSA	POS	POS	AMB	NEG	NEG	NEG	POS	POS	POS	POS	AMB	AMB	POS	POS	NEG	POS	AMB	POS	POS
03V17508	CC45-MRSA-IV, Berlin EMRSA	POS	POS	NEG	NEG	NEG	NEG	POS	POS	POS	NEG	NEG	POS	POS	POS	NEG	POS	AMB	POS	POS
03V20150	CC45-MRSA-IV, Berlin EMRSA	POS	POS	NEG	NEG	NEG	NEG	POS	POS	POS	NEG	NEG	POS	POS	POS	NEG	POS	AMB	POS	POS
03V22437	CC45-MRSA-IV, Berlin EMRSA	POS	POS	NEG	NEG	NEG	NEG	POS	POS	POS	NEG	NEG	POS	POS	POS	NEG	POS	AMB	POS	POS
03V24471	CC45-MRSA-IV, Berlin EMRSA	POS	POS	NEG	NEG	NEG	NEG	POS	POS	POS	AMB	NEG	POS	POS	POS	NEG	POS	AMB	POS	POS
03V28070	CC45-MRSA-IV, Berlin EMRSA	NEG	NEG	NEG	NEG	NEG	NEG	NEG	POS	POS	NEG	NEG	POS	POS	POS	NEG	POS	AMB	POS	POS
03V28448	CC45-MRSA-IV, Berlin EMRSA	POS	POS	NEG	NEG	NEG	NEG	POS	POS	POS	AMB	NEG	POS	POS	POS	NEG	POS	AMB	POS	POS
03V34589	CC45-MRSA-IV, Berlin EMRSA	POS	POS	NEG	NEG	NEG	NEG	POS	POS	POS	AMB	NEG	POS	POS	POS	NEG	POS	AMB	POS	POS
03V39774	CC45-MRSA-IV, Berlin EMRSA	POS	POS	NEG	NEG	NEG	NEG	POS	POS	POS	AMB	NEG	POS	POS	POS	NEG	POS	AMB	POS	POS
**2004**																				
04B05423	CC5-MRSA-II, Rhine-Hesse EMRSA/UK-EMRSA-3	POS	POS	NEG	NEG	POS	NEG	NEG	POS	POS	NEG	NEG	POS	POS	POS	POS	NEG	NEG	NEG	POS
04U02171	CC5-MRSA-II, Rhine-Hesse EMRSA/UK-EMRSA-3	POS	POS	AMB	NEG	POS	NEG	AMB	POS	POS	NEG	NEG	POS	POS	POS	POS	NEG	NEG	NEG	POS
04U03682	CC5-MRSA-II, Rhine-Hesse EMRSA/UK-EMRSA-3	POS	POS	NEG	NEG	POS	NEG	NEG	POS	POS	NEG	NEG	POS	POS	POS	POS	NEG	NEG	NEG	POS
04U06618	CC5-MRSA-II, Rhine-Hesse EMRSA/UK-EMRSA-3	POS	POS	AMB	NEG	POS	NEG	NEG	POS	POS	NEG	NEG	POS	POS	POS	POS	NEG	NEG	NEG	POS
04U12578	CC5-MRSA-II, Rhine-Hesse EMRSA/UK-EMRSA-3	POS	POS	NEG	NEG	POS	NEG	NEG	POS	POS	NEG	NEG	POS	POS	POS	POS	NEG	NEG	NEG	POS
04V02465	CC5-MRSA-II, Rhine-Hesse EMRSA/UK-EMRSA-3	POS	POS	NEG	NEG	POS	NEG	NEG	POS	POS	NEG	NEG	POS	POS	POS	POS	NEG	NEG	NEG	POS
04V03642	CC5-MRSA-II, Rhine-Hesse EMRSA/UK-EMRSA-3	POS	POS	NEG	NEG	POS	NEG	NEG	POS	POS	NEG	NEG	POS	POS	POS	POS	NEG	NEG	NEG	POS
04V07657	CC5-MRSA-II, Rhine-Hesse EMRSA/UK-EMRSA-3	POS	POS	NEG	NEG	POS	NEG	NEG	POS	POS	NEG	NEG	POS	POS	POS	POS	NEG	NEG	NEG	POS
04V12223	CC5-MRSA-II, Rhine-Hesse EMRSA/UK-EMRSA-3	POS	POS	NEG	NEG	POS	NEG	NEG	POS	POS	NEG	NEG	POS	POS	POS	POS	NEG	NEG	NEG	POS
04V12529	CC5-MRSA-II, Rhine-Hesse EMRSA/UK-EMRSA-3	POS	POS	NEG	NEG	POS	NEG	NEG	POS	POS	NEG	NEG	POS	POS	POS	POS	NEG	NEG	NEG	POS
04V13633	CC5-MRSA-II, Rhine-Hesse EMRSA/UK-EMRSA-3	POS	POS	NEG	NEG	POS	NEG	NEG	POS	POS	NEG	NEG	POS	POS	POS	POS	NEG	NEG	NEG	POS
04V16499	CC5-MRSA-II, Rhine-Hesse EMRSA/UK-EMRSA-3	POS	POS	AMB	NEG	POS	NEG	AMB	POS	POS	NEG	NEG	POS	POS	POS	POS	NEG	NEG	NEG	POS
04V17015	CC5-MRSA-II, Rhine-Hesse EMRSA/UK-EMRSA-3	POS	POS	AMB	NEG	POS	NEG	NEG	POS	POS	NEG	NEG	POS	POS	POS	POS	NEG	NEG	NEG	POS
04V20684	CC5-MRSA-II, Rhine-Hesse EMRSA/UK-EMRSA-3																			
04V24615	CC5-MRSA-II, Rhine-Hesse EMRSA/UK-EMRSA-3	NEG	NEG	NEG	NEG	NEG	NEG	NEG	POS	POS	NEG	NEG	POS	POS	POS	POS	NEG	NEG	NEG	POS
04V24901	CC5-MRSA-II, Rhine-Hesse EMRSA/UK-EMRSA-3	POS	POS	NEG	NEG	POS	NEG	NEG	POS	POS	NEG	NEG	POS	POS	POS	POS	NEG	NEG	NEG	POS
04V25081	CC5-MRSA-II, Rhine-Hesse EMRSA/UK-EMRSA-3	POS	POS	NEG	NEG	POS	NEG	NEG	POS	POS	NEG	NEG	POS	POS	POS	POS	NEG	NEG	NEG	POS
04V27293	CC5-MRSA-II, Rhine-Hesse EMRSA/UK-EMRSA-3	POS	POS	NEG	NEG	POS	NEG	NEG	POS	POS	NEG	NEG	POS	POS	POS	POS	NEG	NEG	NEG	POS
04V27311	CC5-MRSA-II, Rhine-Hesse EMRSA/UK-EMRSA-3	POS	POS	NEG	NEG	POS	NEG	NEG	POS	POS	NEG	NEG	POS	POS	POS	POS	NEG	NEG	NEG	POS
04V33459	CC5-MRSA-II, Rhine-Hesse EMRSA/UK-EMRSA-3	POS	POS	AMB	NEG	POS	NEG	AMB	POS	POS	NEG	NEG	POS	POS	POS	POS	NEG	NEG	NEG	POS
04V39909	CC5-MRSA-IV&VI	NEG	NEG	NEG	NEG	NEG	NEG	NEG	POS	POS	NEG	NEG	POS	POS	POS	POS	NEG	NEG	NEG	POS
04U02421	CC5-MRSA-IV, Paediatric clone	POS	POS	NEG	NEG	POS	NEG	NEG	POS	POS	NEG	NEG	POS	POS	POS	POS	NEG	NEG	NEG	POS
04V09807	CC80-MRSA-IV [PVL+], European caMRSA Clone																			
04V16073	CC80-MRSA-IV [PVL+], European caMRSA Clone	POS	POS	NEG	NEG	POS	NEG	NEG	POS	POS	NEG	NEG	POS	POS	POS	POS	NEG	NEG	NEG	POS
04V23548	CC80-MRSA-IV [PVL+], European caMRSA Clone																			
04V03289	CC22-MRSA-IV, Barnim/UK-EMRSA-15																			
04V03847	CC22-MRSA-IV, Barnim/UK-EMRSA-15																			
04V04595	CC22-MRSA-IV, Barnim/UK-EMRSA-15	POS	POS	NEG	NEG	NEG	NEG	NEG	POS	POS	NEG	NEG	POS	POS	POS	NEG	NEG	POS	POS	NEG
04V05029	CC22-MRSA-IV, Barnim/UK-EMRSA-15	NEG	AMB	NEG	NEG	NEG	NEG	NEG	POS	POS	NEG	NEG	POS	POS	POS	NEG	NEG	NEG	POS	NEG
04V07875	CC22-MRSA-IV, Barnim/UK-EMRSA-15	NEG	AMB	NEG	NEG	NEG	NEG	NEG	POS	POS	NEG	NEG	POS	POS	POS	NEG	NEG	NEG	AMB	NEG
04V14909	CC22-MRSA-IV, Barnim/UK-EMRSA-15	POS	POS	NEG	NEG	NEG	NEG	NEG	POS	POS	NEG	NEG	POS	POS	POS	NEG	NEG	NEG	POS	NEG
04V18654	CC22-MRSA-IV, Barnim/UK-EMRSA-15	POS	POS	NEG	NEG	NEG	NEG	NEG	POS	POS	NEG	NEG	POS	POS	POS	NEG	NEG	POS	POS	NEG
04V18815	CC22-MRSA-IV, Barnim/UK-EMRSA-15	POS	POS	NEG	NEG	NEG	NEG	POS	POS	POS	NEG	NEG	POS	POS	POS	NEG	NEG	POS	POS	NEG
04V29721	CC22-MRSA-IV, Barnim/UK-EMRSA-15	POS	POS	NEG	NEG	NEG	NEG	NEG	POS	POS	NEG	NEG	POS	POS	POS	NEG	NEG	NEG	POS	NEG
04V32759	CC22-MRSA-IV, Barnim/UK-EMRSA-15	POS	POS	NEG	NEG	NEG	NEG	NEG	POS	POS	NEG	NEG	POS	POS	POS	NEG	NEG	NEG	POS	NEG
04V32919	CC22-MRSA-IV, Barnim/UK-EMRSA-15	POS	POS	NEG	NEG	POS	AMB	NEG	POS	POS	NEG	NEG	POS	POS	POS	NEG	NEG	POS	POS	NEG
04V33780	CC22-MRSA-IV, Barnim/UK-EMRSA-15	POS	POS	NEG	NEG	AMB	NEG	NEG	POS	POS	NEG	NEG	POS	POS	POS	NEG	NEG	AMB	POS	NEG
04V35396	CC22-MRSA-IV, Barnim/UK-EMRSA-15	POS	POS	NEG	NEG	NEG	NEG	POS	POS	POS	NEG	NEG	POS	POS	POS	NEG	NEG	POS	POS	NEG
04V37107	CC22-MRSA-IV, Barnim/UK-EMRSA-15	POS	POS	NEG	NEG	NEG	NEG	NEG	POS	POS	NEG	NEG	POS	POS	POS	NEG	NEG	POS	POS	NEG
04V37518	CC22-MRSA-IV, Barnim/UK-EMRSA-15	POS	POS	NEG	NEG	NEG	NEG	NEG	POS	POS	NEG	NEG	POS	POS	POS	NEG	NEG	NEG	POS	NEG
04V38199	CC22-MRSA-IV, Barnim/UK-EMRSA-15	POS	POS	NEG	NEG	NEG	NEG	NEG	POS	POS	NEG	NEG	POS	POS	POS	NEG	NEG	POS	POS	NEG
04V38358	CC22-MRSA-IV, Barnim/UK-EMRSA-15	POS	POS	NEG	NEG	NEG	NEG	NEG	POS	POS	NEG	NEG	POS	POS	POS	NEG	NEG	NEG	POS	NEG
04V38902	CC22-MRSA-IV, Barnim/UK-EMRSA-15	POS	POS	NEG	NEG	NEG	NEG	NEG	POS	POS	NEG	NEG	POS	POS	POS	NEG	NEG	NEG	POS	NEG
04V39203	CC22-MRSA-IV, Barnim/UK-EMRSA-15	POS	POS	NEG	NEG	NEG	NEG	NEG	POS	POS	NEG	NEG	POS	POS	POS	NEG	NEG	POS	POS	NEG
04V40423	CC22-MRSA-IV, Barnim/UK-EMRSA-15	POS	POS	NEG	NEG	NEG	NEG	NEG	POS	POS	NEG	NEG	POS	POS	POS	NEG	NEG	NEG	POS	NEG
04V08036	CC45-MRSA-IV [ACME+]	POS	POS	AMB	NEG	NEG	NEG	POS	POS	POS	POS	AMB	AMB	POS	POS	NEG	POS	AMB	POS	POS
04U01119	CC45-MRSA-IV, Berlin EMRSA	POS	POS	NEG	NEG	NEG	NEG	POS	POS	POS	AMB	NEG	POS	POS	POS	NEG	POS	AMB	POS	POS
04V01185	CC45-MRSA-IV, Berlin EMRSA	POS	POS	NEG	NEG	NEG	NEG	POS	POS	POS	NEG	NEG	POS	POS	POS	NEG	POS	AMB	POS	POS
04V06276	CC45-MRSA-IV, Berlin EMRSA	POS	POS	NEG	NEG	NEG	NEG	POS	POS	POS	AMB	NEG	POS	POS	POS	NEG	POS	AMB	POS	POS
04V06677	CC45-MRSA-IV, Berlin EMRSA	POS	POS	NEG	NEG	NEG	NEG	POS	POS	POS	NEG	NEG	POS	POS	POS	NEG	POS	AMB	POS	POS
04V06688	CC45-MRSA-IV, Berlin EMRSA	POS	POS	NEG	NEG	NEG	NEG	POS	POS	POS	AMB	NEG	POS	POS	POS	NEG	POS	AMB	POS	POS
04V14835	CC45-MRSA-IV, Berlin EMRSA	POS	POS	AMB	NEG	NEG	NEG	POS	POS	POS	POS	AMB	AMB	POS	POS	NEG	POS	AMB	POS	POS
04V22079	CC45-MRSA-IV, Berlin EMRSA	POS	POS	NEG	NEG	NEG	NEG	POS	POS	POS	AMB	NEG	POS	POS	POS	NEG	POS	AMB	POS	POS
04V29100	CC45-MRSA-IV, Berlin EMRSA																			
04V31370	CC45-MRSA-IV, Berlin EMRSA	POS	POS	NEG	NEG	NEG	NEG	POS	POS	POS	NEG	NEG	POS	POS	POS	NEG	POS	AMB	POS	POS
04V38442	CC45-MRSA-IV, Berlin EMRSA	POS	POS	AMB	NEG	NEG	NEG	POS	POS	POS	POS	NEG	AMB	POS	POS	NEG	POS	AMB	POS	POS
04V39012	CC45-MRSA-IV, Berlin EMRSA	POS	POS	AMB	NEG	NEG	NEG	POS	POS	POS	AMB	NEG	POS	POS	POS	NEG	POS	AMB	POS	POS
04V39577	CC45-MRSA-IV, Berlin EMRSA	POS	POS	AMB	NEG	NEG	NEG	POS	POS	POS	AMB	NEG	POS	POS	POS	NEG	POS	AMB	POS	POS
04V22872	CC152-MRSA-V [PVL+]	POS	POS	POS	NEG	NEG	NEG	AMB	POS	POS	POS	AMB	AMB	POS	POS	NEG	POS	AMB	POS	POS

ISOLATE:	cell surface elastin binding protein					enolase	fibrinogen binding protein (19 kDa)		fibronectin-binding protein A						fibronectin-binding protein B							Major histocompatibility complex class II analog protein (=Extracellular adherence protein, eap)				Staphylococcus aureus surface protein G			
	ebpS (total)	ebpS_probe 612	ebpS_probe 614	ebpS (01-1111)	ebpS (COL)	eno	fib	fib (MRSA252)	fnbA (total)	fnbA (consensus)	fnbA (COL)	fnbA (MRSA252)	fnbA (Mu50+MW2)	fnbA (RF122)	fnbB (total)	fnbB (COL)	fnbB (COL+Mu50+MW2)	fnbB (Mu50)	fnbB (MW2)	fnbB (ST15)	fnbB (ST45-2)	map (total)	map (COL)	map (MRSA252)	map (Mu50+MW2)	sasG (total)	sasG (COL+Mu50)	sasG (MW2)	sasG (other than MRSA252+RF122)
**2003**																													
03F01030	POS	POS	POS	NEG	NEG	POS	POS	NEG	POS	POS	NEG	NEG	POS	NEG	NEG	NEG	NEG	NEG	NEG	NEG	NEG	POS	POS	NEG	POS	POS	POS	NEG	POS
03V05419	POS	AMB	POS	NEG	NEG	POS	POS	NEG	POS	POS	NEG	NEG	POS	NEG	NEG	NEG	NEG	NEG	NEG	NEG	NEG	POS	POS	NEG	POS	POS	POS	NEG	POS
03V08986	POS	POS	POS	NEG	NEG	POS	POS	NEG	POS	POS	NEG	NEG	POS	NEG	NEG	NEG	NEG	NEG	NEG	NEG	NEG	POS	POS	NEG	POS	POS	POS	NEG	POS
03V39123																													
03B05186	POS	POS	POS	NEG	NEG	POS	POS	NEG	POS	POS	NEG	NEG	POS	NEG	POS	NEG	POS	AMB	NEG	NEG	NEG	POS	NEG	NEG	POS	POS	POS	NEG	POS
03U00003	POS	POS	POS	NEG	NEG	POS	POS	NEG	POS	POS	NEG	NEG	POS	NEG	POS	NEG	POS	AMB	NEG	NEG	NEG	POS	AMB	NEG	POS	POS	POS	NEG	POS
03V01438	POS	POS	POS	NEG	NEG	POS	POS	NEG	POS	POS	NEG	NEG	POS	NEG	POS	NEG	POS	AMB	NEG	NEG	NEG	POS	NEG	NEG	POS	POS	POS	NEG	POS
03V05075	POS	POS	POS	NEG	NEG	POS	POS	NEG	POS	POS	NEG	NEG	POS	NEG	POS	NEG	POS	AMB	NEG	NEG	NEG	POS	NEG	NEG	POS	POS	POS	NEG	POS
03V11014	POS	POS	POS	NEG	NEG	POS	POS	NEG	POS	POS	NEG	NEG	POS	NEG	POS	NEG	POS	AMB	NEG	AMB	NEG	POS	POS	NEG	POS	POS	POS	NEG	POS
03V12917	POS	POS	POS	NEG	NEG	POS	POS	NEG	POS	POS	NEG	NEG	POS	NEG	POS	NEG	AMB	POS	NEG	AMB	NEG	POS	POS	NEG	POS	POS	POS	NEG	POS
03V14467	POS	POS	POS	NEG	NEG	POS	POS	NEG	POS	POS	NEG	NEG	POS	NEG	POS	NEG	AMB	POS	NEG	AMB	NEG	POS	POS	AMB	POS	POS	POS	NEG	POS
03V19357	POS	POS	POS	NEG	NEG	POS	POS	NEG	POS	POS	NEG	NEG	POS	NEG	POS	NEG	POS	AMB	NEG	NEG	NEG	POS	NEG	NEG	POS	POS	POS	NEG	POS
03V20186	POS	POS	POS	NEG	NEG	POS	POS	NEG	POS	POS	NEG	NEG	POS	NEG	POS	NEG	POS	AMB	NEG	AMB	NEG	POS	POS	NEG	POS	POS	POS	NEG	POS
03V25349	POS	POS	POS	NEG	NEG	POS	POS	NEG	POS	POS	NEG	NEG	POS	NEG	POS	NEG	AMB	POS	NEG	AMB	NEG	POS	POS	NEG	POS	POS	POS	NEG	POS
03V28948	POS	POS	POS	NEG	NEG	POS	POS	NEG	POS	POS	NEG	NEG	POS	NEG	POS	NEG	POS	AMB	NEG	NEG	NEG	POS	NEG	NEG	POS	POS	POS	NEG	POS
03V30841																													
03V32896	POS	POS	POS	NEG	NEG	POS	POS	NEG	POS	POS	NEG	NEG	POS	NEG	POS	NEG	POS	AMB	NEG	NEG	NEG	POS	AMB	NEG	POS	POS	POS	NEG	POS
03V33415	POS	POS	POS	NEG	NEG	POS	POS	NEG	POS	POS	NEG	NEG	POS	NEG	POS	NEG	POS	AMB	NEG	AMB	NEG	POS	POS	NEG	POS	POS	POS	NEG	POS
03V34628	POS	POS	POS	NEG	AMB	POS	POS	NEG	POS	POS	NEG	NEG	POS	NEG	POS	NEG	AMB	POS	NEG	AMB	NEG	POS	POS	AMB	POS	POS	POS	NEG	POS
03V37078	POS	AMB	POS	NEG	NEG	POS	POS	NEG	POS	POS	NEG	NEG	POS	NEG	POS	NEG	AMB	POS	NEG	NEG	NEG	POS	AMB	NEG	POS	POS	POS	NEG	POS
03V41645	POS	POS	POS	NEG	NEG	POS	POS	NEG	POS	POS	NEG	NEG	POS	NEG	POS	NEG	POS	AMB	NEG	AMB	NEG	POS	POS	NEG	POS	POS	POS	NEG	POS
03V03012	POS	POS	POS	NEG	NEG	POS	POS	NEG	POS	POS	NEG	NEG	POS	NEG	POS	NEG	POS	POS	NEG	NEG	NEG	POS	NEG	NEG	POS	POS	POS	NEG	POS
03V02863	POS	POS	POS	NEG	POS	POS	POS	NEG	POS	POS	POS	NEG	NEG	NEG	POS	POS	AMB	NEG	NEG	NEG	NEG	POS	POS	NEG	NEG	POS	POS	NEG	POS
03V14882	POS	POS	POS	NEG	POS	POS	POS	NEG	POS	POS	POS	NEG	NEG	NEG	POS	POS	AMB	AMB	NEG	NEG	NEG	POS	POS	NEG	NEG	POS	POS	NEG	POS
03B00404	POS	POS	POS	NEG	POS	POS	NEG	POS	POS	POS	NEG	NEG	POS	NEG	NEG	NEG	NEG	NEG	NEG	NEG	NEG	POS	NEG	NEG	POS	POS	NEG	POS	POS
03U00892																													
03V01422	POS	POS	POS	NEG	POS	POS	NEG	POS	POS	POS	NEG	NEG	POS	NEG	NEG	NEG	NEG	NEG	NEG	NEG	NEG	POS	NEG	NEG	POS	POS	NEG	POS	POS
03V04424	POS	POS	POS	NEG	POS	POS	NEG	POS	POS	POS	NEG	NEG	POS	NEG	NEG	NEG	NEG	NEG	NEG	NEG	NEG	POS	NEG	NEG	POS	POS	NEG	POS	POS
03V05690	POS	POS	POS	NEG	POS	POS	NEG	POS	POS	POS	NEG	NEG	POS	NEG	POS	NEG	AMB	NEG	POS	NEG	NEG	POS	NEG	NEG	POS	POS	NEG	POS	POS
03V07912	POS	POS	POS	NEG	POS	POS	NEG	POS	POS	POS	NEG	NEG	POS	NEG	POS	NEG	POS	AMB	POS	NEG	NEG	POS	NEG	NEG	POS	POS	NEG	POS	POS
03V23798	POS	POS	POS	NEG	POS	POS	NEG	POS	POS	POS	NEG	NEG	POS	NEG	NEG	NEG	NEG	NEG	NEG	NEG	NEG	NEG	NEG	NEG	AMB	POS	NEG	POS	POS
03V25914	POS	POS	POS	NEG	POS	POS	NEG	POS	POS	POS	NEG	NEG	POS	NEG	NEG	NEG	NEG	NEG	NEG	NEG	NEG	NEG	NEG	NEG	AMB	POS	NEG	POS	POS
03V29751	POS	AMB	POS	NEG	POS	POS	NEG	POS	POS	POS	NEG	NEG	POS	NEG	NEG	NEG	NEG	NEG	NEG	NEG	NEG	NEG	NEG	NEG	AMB	POS	NEG	POS	POS
03V30201	POS	POS	POS	NEG	POS	POS	NEG	POS	POS	POS	NEG	NEG	POS	NEG	NEG	NEG	NEG	NEG	NEG	NEG	NEG	POS	NEG	NEG	POS	POS	NEG	POS	POS
03B01781	POS	NEG	POS	AMB	NEG	POS	NEG	AMB	POS	POS	NEG	NEG	POS	NEG	POS	NEG	POS	AMB	NEG	AMB	NEG	POS	NEG	POS	NEG	NEG	NEG	NEG	NEG
03U05014	POS	NEG	POS	POS	NEG	POS	NEG	POS	POS	POS	NEG	NEG	POS	NEG	POS	NEG	POS	AMB	NEG	AMB	NEG	POS	NEG	POS	NEG	NEG	NEG	NEG	NEG
03U11106	POS	NEG	POS	POS	NEG	POS	NEG	POS	POS	POS	NEG	NEG	POS	NEG	POS	NEG	AMB	POS	NEG	AMB	NEG	POS	NEG	POS	NEG	NEG	NEG	NEG	NEG
03V00742	POS	NEG	POS	POS	NEG	POS	NEG	POS	POS	POS	NEG	NEG	POS	NEG	POS	NEG	POS	AMB	NEG	AMB	NEG	POS	NEG	POS	NEG	NEG	NEG	NEG	NEG
03V01902	POS	NEG	POS	POS	NEG	POS	NEG	POS	POS	POS	NEG	NEG	POS	NEG	POS	NEG	POS	AMB	NEG	AMB	NEG	POS	NEG	POS	NEG	NEG	NEG	NEG	NEG
03V02256	POS	NEG	POS	POS	NEG	POS	NEG	POS	POS	POS	NEG	NEG	POS	NEG	POS	NEG	POS	AMB	NEG	AMB	NEG	POS	NEG	POS	NEG	NEG	NEG	NEG	NEG
03V02262	POS	NEG	POS	POS	NEG	POS	NEG	POS	POS	POS	NEG	NEG	POS	NEG	POS	NEG	POS	AMB	NEG	AMB	NEG	POS	NEG	POS	NEG	NEG	NEG	NEG	NEG
03V04701	POS	NEG	POS	POS	NEG	POS	NEG	POS	POS	POS	NEG	NEG	POS	NEG	POS	NEG	POS	AMB	NEG	NEG	NEG	POS	NEG	POS	NEG	NEG	NEG	NEG	NEG
03V05042	POS	NEG	POS	POS	NEG	POS	NEG	POS	POS	POS	NEG	NEG	POS	NEG	POS	NEG	POS	AMB	NEG	AMB	NEG	POS	NEG	POS	NEG	NEG	NEG	NEG	NEG
03V05377	POS	NEG	POS	POS	NEG	POS	NEG	POS	POS	POS	NEG	NEG	POS	NEG	POS	NEG	POS	AMB	NEG	AMB	NEG	POS	NEG	POS	NEG	NEG	NEG	NEG	NEG
03V05507	POS	NEG	POS	POS	NEG	POS	NEG	POS	POS	POS	NEG	NEG	POS	NEG	POS	NEG	POS	AMB	NEG	NEG	NEG	POS	NEG	POS	NEG	NEG	NEG	NEG	NEG
03V08214	POS	NEG	POS	POS	NEG	POS	NEG	AMB	POS	POS	NEG	NEG	POS	NEG	POS	NEG	POS	AMB	NEG	AMB	NEG	POS	NEG	POS	NEG	NEG	NEG	NEG	NEG
03V08525	POS	NEG	POS	POS	NEG	POS	NEG	POS	POS	POS	NEG	NEG	POS	NEG	POS	NEG	POS	AMB	NEG	NEG	NEG	POS	NEG	POS	NEG	NEG	NEG	NEG	NEG
03V11910	POS	NEG	POS	POS	NEG	POS	NEG	AMB	POS	POS	NEG	NEG	POS	NEG	POS	NEG	POS	AMB	NEG	NEG	NEG	POS	NEG	POS	NEG	NEG	NEG	NEG	NEG
03V14481	POS	NEG	POS	POS	NEG	POS	NEG	POS	POS	POS	NEG	NEG	POS	NEG	POS	NEG	POS	AMB	NEG	NEG	NEG	POS	NEG	POS	NEG	NEG	NEG	NEG	NEG
03V15082	POS	NEG	POS	POS	NEG	POS	NEG	POS	POS	POS	NEG	NEG	POS	NEG	POS	NEG	AMB	POS	NEG	AMB	NEG	POS	NEG	POS	NEG	NEG	NEG	NEG	NEG
03V17508	POS	NEG	POS	POS	NEG	POS	NEG	POS	POS	POS	NEG	NEG	POS	NEG	POS	NEG	POS	AMB	NEG	NEG	NEG	POS	NEG	POS	NEG	NEG	NEG	NEG	NEG
03V20150	POS	NEG	POS	POS	NEG	POS	NEG	POS	POS	POS	NEG	NEG	POS	NEG	POS	NEG	AMB	POS	NEG	NEG	NEG	POS	NEG	POS	NEG	NEG	NEG	NEG	NEG
03V22437	POS	NEG	POS	AMB	NEG	POS	NEG	AMB	POS	POS	NEG	NEG	POS	NEG	POS	NEG	POS	AMB	NEG	NEG	NEG	POS	NEG	POS	NEG	NEG	NEG	NEG	NEG
03V24471	POS	NEG	POS	POS	NEG	POS	NEG	POS	POS	POS	NEG	NEG	POS	NEG	POS	NEG	POS	AMB	NEG	NEG	NEG	POS	NEG	POS	NEG	NEG	NEG	NEG	NEG
03V28070	POS	NEG	POS	AMB	NEG	POS	NEG	AMB	POS	POS	NEG	NEG	POS	NEG	POS	NEG	AMB	POS	NEG	NEG	NEG	POS	NEG	POS	NEG	NEG	NEG	NEG	NEG
03V28448	POS	NEG	POS	POS	NEG	POS	NEG	POS	POS	POS	NEG	NEG	POS	NEG	POS	NEG	POS	AMB	NEG	AMB	NEG	POS	NEG	POS	NEG	NEG	NEG	NEG	NEG
03V34589	POS	NEG	POS	POS	NEG	POS	NEG	POS	POS	POS	NEG	NEG	POS	NEG	POS	NEG	POS	AMB	NEG	AMB	NEG	POS	NEG	POS	NEG	NEG	NEG	NEG	NEG
03V39774	POS	NEG	POS	POS	NEG	POS	NEG	POS	POS	POS	NEG	NEG	POS	NEG	NEG	NEG	NEG	NEG	NEG	NEG	NEG	POS	NEG	POS	NEG	NEG	NEG	NEG	NEG
**2004**																													
04B05423	POS	POS	POS	NEG	NEG	POS	POS	NEG	POS	POS	NEG	NEG	POS	NEG	POS	NEG	POS	AMB	NEG	NEG	NEG	POS	NEG	NEG	POS	POS	POS	NEG	POS
04U02171	POS	POS	POS	NEG	NEG	POS	POS	NEG	POS	POS	NEG	NEG	POS	NEG	POS	NEG	AMB	POS	NEG	AMB	NEG	POS	POS	NEG	POS	POS	POS	NEG	POS
04U03682	POS	POS	POS	NEG	NEG	POS	POS	NEG	POS	POS	NEG	NEG	POS	NEG	POS	NEG	POS	AMB	NEG	NEG	NEG	POS	NEG	NEG	POS	POS	POS	NEG	POS
04U06618	POS	POS	POS	NEG	NEG	POS	POS	NEG	POS	POS	NEG	NEG	POS	NEG	POS	NEG	AMB	POS	NEG	AMB	NEG	POS	POS	NEG	POS	POS	POS	NEG	POS
04U12578	POS	POS	POS	NEG	NEG	POS	POS	NEG	POS	POS	NEG	NEG	POS	NEG	POS	NEG	POS	AMB	NEG	NEG	NEG	POS	NEG	NEG	POS	POS	POS	NEG	POS
04V02465	POS	POS	POS	NEG	NEG	POS	POS	NEG	POS	POS	NEG	NEG	POS	NEG	POS	NEG	POS	AMB	NEG	NEG	NEG	POS	NEG	NEG	POS	POS	POS	NEG	POS
04V03642	POS	POS	POS	NEG	NEG	POS	POS	NEG	POS	POS	NEG	NEG	POS	NEG	POS	NEG	POS	AMB	NEG	NEG	NEG	POS	NEG	NEG	POS	POS	POS	NEG	POS
04V07657	POS	POS	POS	NEG	NEG	POS	POS	NEG	POS	POS	NEG	NEG	POS	NEG	POS	NEG	POS	AMB	NEG	NEG	NEG	POS	NEG	NEG	POS	POS	POS	NEG	POS
04V12223	POS	POS	POS	NEG	NEG	POS	POS	NEG	POS	POS	NEG	NEG	POS	NEG	POS	NEG	POS	AMB	NEG	NEG	NEG	POS	NEG	NEG	POS	POS	POS	NEG	POS
04V12529	POS	POS	POS	NEG	NEG	POS	POS	NEG	POS	POS	NEG	NEG	POS	NEG	POS	NEG	POS	AMB	NEG	NEG	NEG	POS	NEG	NEG	POS	POS	POS	NEG	POS
04V13633	POS	POS	POS	NEG	NEG	POS	POS	NEG	POS	POS	NEG	NEG	POS	NEG	POS	NEG	POS	AMB	NEG	NEG	NEG	POS	NEG	NEG	POS	POS	POS	NEG	POS
04V16499	POS	POS	POS	NEG	NEG	POS	POS	NEG	POS	POS	NEG	NEG	POS	NEG	POS	NEG	POS	AMB	NEG	AMB	NEG	POS	POS	NEG	POS	POS	POS	NEG	POS
04V17015	POS	POS	POS	NEG	NEG	POS	POS	NEG	POS	POS	NEG	NEG	POS	NEG	POS	NEG	AMB	POS	NEG	AMB	NEG	POS	POS	NEG	POS	POS	POS	NEG	POS
04V20684																													
04V24615	POS	POS	POS	NEG	NEG	POS	POS	NEG	POS	POS	NEG	NEG	POS	NEG	POS	NEG	POS	AMB	NEG	NEG	NEG	POS	NEG	NEG	POS	POS	POS	NEG	POS
04V24901	POS	POS	POS	NEG	NEG	POS	POS	NEG	POS	POS	NEG	NEG	POS	NEG	POS	NEG	AMB	POS	NEG	NEG	NEG	POS	POS	NEG	POS	POS	POS	NEG	POS
04V25081	POS	POS	POS	NEG	NEG	POS	POS	NEG	POS	POS	NEG	NEG	POS	NEG	POS	NEG	AMB	POS	NEG	NEG	NEG	POS	POS	NEG	POS	POS	POS	NEG	POS
04V27293	POS	POS	POS	NEG	NEG	POS	POS	NEG	POS	POS	NEG	NEG	POS	NEG	POS	NEG	AMB	POS	NEG	NEG	NEG	POS	POS	NEG	POS	POS	POS	NEG	POS
04V27311	POS	POS	POS	NEG	NEG	POS	POS	NEG	POS	POS	NEG	NEG	POS	NEG	POS	NEG	POS	AMB	NEG	NEG	NEG	POS	NEG	NEG	POS	POS	POS	NEG	POS
04V33459	POS	POS	POS	NEG	NEG	POS	POS	NEG	POS	POS	NEG	NEG	POS	NEG	POS	NEG	AMB	POS	NEG	AMB	NEG	POS	POS	NEG	POS	POS	POS	NEG	POS
04V39909	POS	POS	POS	NEG	NEG	POS	POS	NEG	POS	POS	NEG	NEG	POS	NEG	POS	NEG	POS	POS	NEG	NEG	NEG	POS	NEG	NEG	POS	POS	POS	NEG	POS
04U02421	POS	POS	POS	NEG	NEG	POS	POS	NEG	POS	POS	NEG	NEG	POS	NEG	POS	NEG	POS	POS	NEG	NEG	NEG	POS	NEG	NEG	POS	POS	POS	NEG	POS
04V09807																													
04V16073	POS	POS	POS	NEG	NEG	POS	POS	NEG	POS	POS	NEG	NEG	NEG	NEG	POS	NEG	POS	NEG	POS	NEG	NEG	POS	AMB	NEG	POS	POS	NEG	POS	POS
04V23548																													
04V03289																													
04V03847																													
04V04595	POS	POS	POS	NEG	POS	POS	NEG	POS	POS	POS	NEG	NEG	POS	NEG	NEG	NEG	NEG	NEG	NEG	NEG	NEG	POS	NEG	NEG	POS	POS	NEG	POS	POS
04V05029	POS	AMB	POS	NEG	AMB	POS	NEG	POS	POS	POS	NEG	NEG	POS	NEG	NEG	NEG	NEG	NEG	NEG	NEG	NEG	NEG	NEG	NEG	NEG	POS	NEG	POS	POS
04V07875	POS	NEG	POS	NEG	NEG	POS	NEG	POS	POS	POS	NEG	NEG	POS	NEG	NEG	NEG	NEG	NEG	NEG	NEG	NEG	NEG	NEG	NEG	NEG	POS	NEG	POS	POS
04V14909	POS	POS	POS	NEG	POS	POS	NEG	POS	POS	POS	NEG	NEG	POS	NEG	NEG	NEG	NEG	NEG	NEG	NEG	NEG	NEG	NEG	NEG	AMB	POS	NEG	POS	POS
04V18654	POS	POS	POS	NEG	POS	POS	NEG	POS	POS	POS	NEG	NEG	POS	NEG	NEG	NEG	NEG	NEG	NEG	NEG	NEG	POS	NEG	NEG	POS	POS	NEG	POS	POS
04V18815	POS	POS	POS	NEG	POS	POS	NEG	POS	POS	POS	NEG	NEG	POS	NEG	NEG	NEG	NEG	NEG	NEG	NEG	NEG	POS	NEG	NEG	POS	POS	NEG	POS	POS
04V29721	POS	POS	POS	NEG	AMB	POS	NEG	POS	POS	POS	NEG	NEG	POS	NEG	NEG	NEG	NEG	NEG	NEG	NEG	NEG	NEG	NEG	NEG	NEG	POS	NEG	POS	POS
04V32759	POS	POS	POS	NEG	POS	POS	NEG	POS	POS	POS	NEG	NEG	POS	NEG	NEG	NEG	NEG	NEG	NEG	NEG	NEG	POS	NEG	NEG	POS	POS	NEG	POS	POS
04V32919	POS	POS	POS	NEG	POS	POS	NEG	POS	POS	POS	NEG	NEG	POS	NEG	NEG	NEG	NEG	NEG	NEG	NEG	NEG	POS	AMB	NEG	POS	POS	NEG	POS	POS
04V33780	POS	POS	POS	NEG	POS	POS	NEG	POS	POS	POS	NEG	NEG	POS	NEG	NEG	NEG	NEG	NEG	NEG	NEG	NEG	POS	NEG	NEG	POS	POS	NEG	POS	POS
04V35396	POS	POS	POS	NEG	POS	POS	NEG	POS	POS	POS	NEG	NEG	POS	NEG	NEG	NEG	NEG	NEG	NEG	NEG	NEG	POS	NEG	NEG	POS	POS	NEG	POS	POS
04V37107	POS	POS	POS	NEG	POS	POS	NEG	POS	POS	POS	NEG	NEG	POS	NEG	NEG	NEG	NEG	NEG	NEG	NEG	NEG	POS	NEG	NEG	POS	POS	NEG	POS	POS
04V37518	POS	POS	POS	NEG	POS	POS	NEG	POS	POS	POS	NEG	NEG	POS	NEG	NEG	NEG	NEG	NEG	NEG	NEG	NEG	NEG	NEG	NEG	AMB	POS	NEG	POS	POS
04V38199	POS	POS	POS	NEG	POS	POS	NEG	POS	POS	POS	NEG	NEG	POS	NEG	NEG	NEG	NEG	NEG	NEG	NEG	NEG	NEG	NEG	NEG	NEG	POS	NEG	POS	POS
04V38358	POS	POS	POS	NEG	AMB	POS	NEG	POS	POS	POS	NEG	NEG	POS	NEG	NEG	NEG	NEG	NEG	NEG	NEG	NEG	NEG	NEG	NEG	NEG	POS	NEG	POS	POS
04V38902	POS	AMB	POS	NEG	NEG	POS	NEG	POS	POS	POS	NEG	NEG	POS	NEG	NEG	NEG	NEG	NEG	NEG	NEG	NEG	NEG	NEG	NEG	NEG	POS	NEG	POS	POS
04V39203	POS	POS	POS	NEG	POS	POS	NEG	POS	POS	POS	NEG	NEG	POS	NEG	NEG	NEG	NEG	NEG	NEG	NEG	NEG	POS	NEG	NEG	POS	POS	NEG	POS	POS
04V40423	POS	POS	POS	NEG	AMB	POS	NEG	POS	POS	POS	NEG	NEG	POS	NEG	NEG	NEG	NEG	NEG	NEG	NEG	NEG	NEG	NEG	NEG	NEG	POS	NEG	POS	POS
04V08036	POS	NEG	POS	POS	NEG	POS	NEG	POS	POS	POS	NEG	NEG	POS	NEG	POS	NEG	POS	AMB	NEG	AMB	NEG	POS	NEG	POS	NEG	NEG	NEG	NEG	NEG
04U01119	POS	NEG	POS	POS	NEG	POS	NEG	POS	POS	POS	NEG	NEG	POS	NEG	POS	NEG	POS	AMB	NEG	NEG	NEG	POS	NEG	POS	NEG	NEG	NEG	NEG	NEG
04V01185	POS	NEG	POS	POS	NEG	POS	NEG	POS	POS	POS	NEG	NEG	POS	NEG	POS	NEG	AMB	POS	NEG	NEG	NEG	POS	NEG	POS	NEG	NEG	NEG	NEG	NEG
04V06276	POS	NEG	POS	POS	NEG	POS	NEG	POS	POS	POS	NEG	NEG	POS	NEG	POS	NEG	POS	AMB	NEG	NEG	NEG	POS	NEG	POS	NEG	NEG	NEG	NEG	NEG
04V06677	POS	NEG	POS	POS	NEG	POS	NEG	POS	POS	POS	NEG	NEG	POS	NEG	POS	NEG	POS	AMB	NEG	NEG	NEG	POS	NEG	POS	NEG	NEG	NEG	NEG	NEG
04V06688	POS	NEG	POS	POS	NEG	POS	NEG	POS	POS	POS	NEG	NEG	POS	NEG	POS	NEG	POS	AMB	NEG	NEG	NEG	POS	NEG	POS	NEG	NEG	NEG	NEG	NEG
04V14835	POS	NEG	POS	POS	NEG	POS	NEG	POS	POS	POS	NEG	NEG	POS	NEG	POS	NEG	POS	AMB	NEG	AMB	NEG	POS	NEG	POS	NEG	NEG	NEG	NEG	NEG
04V22079	POS	NEG	POS	POS	NEG	POS	NEG	POS	POS	POS	NEG	NEG	POS	NEG	POS	NEG	POS	AMB	NEG	NEG	NEG	POS	NEG	POS	NEG	NEG	NEG	NEG	NEG
04V29100																													
04V31370	POS	NEG	POS	AMB	NEG	POS	NEG	NEG	POS	POS	NEG	NEG	POS	NEG	POS	NEG	POS	AMB	NEG	NEG	NEG	POS	NEG	POS	NEG	NEG	NEG	NEG	NEG
04V38442	POS	NEG	POS	POS	NEG	POS	NEG	POS	POS	POS	NEG	NEG	POS	NEG	POS	NEG	POS	AMB	NEG	AMB	NEG	POS	NEG	POS	NEG	NEG	NEG	NEG	NEG
04V39012	POS	NEG	POS	POS	NEG	POS	NEG	POS	POS	POS	NEG	NEG	POS	NEG	POS	NEG	POS	AMB	NEG	AMB	NEG	POS	NEG	POS	NEG	NEG	NEG	NEG	NEG
04V39577	POS	NEG	POS	POS	NEG	POS	NEG	POS	POS	POS	NEG	NEG	POS	NEG	POS	NEG	POS	AMB	NEG	AMB	NEG	POS	NEG	POS	NEG	NEG	NEG	AMB	NEG
04V22872	POS	POS	POS	NEG	NEG	POS	NEG	NEG	POS	POS	NEG	NEG	NEG	NEG	POS	NEG	AMB	POS	NEG	NEG	NEG	NEG	NEG	NEG	NEG	NEG	NEG	AMB	NEG

ISOLATE:	STRAIN	sdrC (total)	sdrC (consensus)	sdrC (B1)	sdrC (COL)	sdrC (Mu50)	sdrC (MW2+MRSA252+RF122)	sdrC (other than MRSA252+RF122)	sdrD (total)	sdrD (consensus)	sdrD (COL+MW2)	sdrD (Mu50)	sdrD (other)	vwb (total)	vwb (consensus)	vwb (COL+MW2)	vwb (MRSA252)	vwb (Mu50)	vwb (RF122)	isaB	isaB (MRSA252)	mprF (COL+MW2)	mprF (Mu50+MRSA252)	isdA (consensus)	isdA (MRSA252)	isdA (other than MRSA252)	lmrP (other than RF122)	lmrP (RF122)	hsdS1 (RF122)	hsdS2 (Mu50+N315+COL+USA300+NCTC8325)	hsdS2 (MW2+MSSA476)	hsdS2 (MRSA252)	hsdS3 (all other than RF122+MRSA252)	hsdS3 (COL+USA300+NCTC8325+MW2+MSSA476+RF122)	hsdS3 (Mu50+N315)	hsdS3 (CCS1+MRSA252)	hsdS3 (MRSA252)	hsdSx (CC25)	hsdSx (CC15)	hsdSx (etd)	Q2FXC0	Q2YUB3	Q7A4X2	hysA1 (MRSA252)	hysA1 (MRSA252+RF122) and/or hysA2 (consensus)	hysA1 (MRSA252+RF122) and/or hysA2 (COL+USA300)	hysA2 (all other than MRSA252)	hysA2 (COL+USA300+NCTC8325)	hysA2 (all other than COL+USA300+NCTC8325)	hysA2 (all other than COL+USA300+NCTC8325)	hysA2 (MRSA252)
		ADHAESION FACTORS / GENES ENCODING MICROBIAL SURFACE COMPONENTS RECOGNIZING ADHESIVE MATRIX MOLECULES (MSCRAMM GENES)																		IMMUNOD.ANTIGEN B		DEFENSIN RESIST.		TRANSFERRIN BINDING PROT			PUTATIVE TRANSPORTER		TYPE I RESTRICTION-MODIFICATION SYSTEM, SINGLE SEQUENCE SPECIFICITY PROTEIN												MISCELLANEOUS GENES			HYALURONATE LYASE							
		Ser-Asp rich fibrinogen-/bone sialoprotein-binding protein C							Ser-Asp rich fibrinogen-/bone sialoprotein-binding protein D					van Willebrand factor binding protein						immunodominant antigen B		defensin resistance protein		transferrin-binding protein			hypothetical protein, similar to integral membrane protein LmrP		type I site-specific deoxyribo-nuclease subunit, 1st locus	type I site-specific deoxyribonuclease subunit, 2nd locus			type I site-specific deoxyribonuclease subunit, 3rd locus					type I site-specific deoxyribonuclease subunit, unknown locus			hypothetical protein, located next to serine protease operon	Unspecific efflux/trans-porter	hypothetical protein	hyaluronate lyase, first / second locus			hyaluronate lyase, second locus				
**2003**																																																			
03F01030	CC5/ST228-MRSA, South German EMRSA (truncated SCCmec element)	POS	POS	NEG	NEG	POS	NEG	POS	POS	POS	NEG	POS	NEG	POS	POS	NEG	NEG	POS	NEG	POS	AMB	AMB	POS	POS	NEG	POS	POS	NEG	NEG	NEG	NEG	NEG	POS	NEG	POS	NEG	NEG	POS	NEG	NEG	NEG	NEG	POS	NEG	POS	NEG	POS	NEG	POS	POS	NEG
03V05419	CC5/ST228-MRSA-I, South German EMRSA	POS	POS	NEG	NEG	POS	NEG	POS	NEG	NEG	NEG	NEG	NEG	POS	POS	NEG	NEG	POS	NEG	POS	AMB	AMB	POS	POS	NEG	POS	POS	NEG	NEG	NEG	NEG	NEG	POS	NEG	POS	NEG	NEG	POS	NEG	NEG	NEG	NEG	NEG	NEG	POS	NEG	AMB	NEG	POS	POS	NEG
03V08986	CC5/ST228-MRSA-I, South German EMRSA	POS	POS	NEG	NEG	POS	NEG	POS	POS	POS	NEG	POS	NEG	POS	POS	NEG	NEG	POS	NEG	POS	AMB	AMB	POS	POS	AMB	POS	POS	NEG	NEG	NEG	NEG	NEG	POS	NEG	POS	NEG	NEG	POS	AMB	NEG	NEG	AMB	POS	NEG	POS	NEG	AMB	NEG	POS	POS	NEG
03V39123	CC5/ST228-MRSA-I, South German EMRSA																																																		
03B05186	CC5-MRSA-II, Rhine-Hesse EMRSA/UK-EMRSA-3	POS	POS	NEG	NEG	POS	NEG	POS	POS	POS	NEG	POS	NEG	POS	POS	NEG	NEG	POS	NEG	POS	AMB	AMB	POS	POS	NEG	POS	POS	NEG	NEG	POS	NEG	NEG	POS	NEG	POS	NEG	NEG	POS	NEG	NEG	NEG	NEG	POS	NEG	POS	NEG	POS	NEG	POS	POS	NEG
03U00003	CC5-MRSA-II, Rhine-Hesse EMRSA/UK-EMRSA-3	POS	POS	NEG	NEG	POS	NEG	POS	POS	POS	NEG	POS	NEG	POS	POS	NEG	NEG	POS	NEG	POS	AMB	AMB	POS	POS	NEG	POS	POS	NEG	NEG	POS	NEG	NEG	POS	NEG	POS	NEG	NEG	POS	NEG	NEG	NEG	NEG	POS	NEG	POS	NEG	POS	NEG	POS	POS	NEG
03V01438	CC5-MRSA-II, Rhine-Hesse EMRSA/UK-EMRSA-3	POS	POS	NEG	NEG	POS	NEG	POS	POS	POS	NEG	POS	NEG	POS	POS	NEG	NEG	POS	NEG	POS	AMB	AMB	POS	POS	NEG	POS	POS	NEG	NEG	POS	NEG	NEG	POS	NEG	POS	NEG	NEG	POS	NEG	NEG	NEG	NEG	POS	NEG	POS	NEG	POS	NEG	POS	POS	NEG
03V05075	CC5-MRSA-II, Rhine-Hesse EMRSA/UK-EMRSA-3	POS	POS	NEG	NEG	POS	NEG	POS	POS	POS	NEG	POS	NEG	POS	POS	NEG	NEG	POS	NEG	POS	AMB	AMB	POS	POS	NEG	POS	POS	NEG	NEG	POS	NEG	NEG	POS	NEG	POS	NEG	NEG	POS	NEG	NEG	NEG	NEG	POS	NEG	POS	NEG	POS	NEG	POS	POS	NEG
03V11014	CC5-MRSA-II, Rhine-Hesse EMRSA/UK-EMRSA-3	POS	POS	NEG	NEG	POS	NEG	POS	POS	POS	NEG	POS	NEG	POS	POS	NEG	NEG	POS	NEG	POS	AMB	AMB	POS	POS	NEG	POS	POS	NEG	NEG	POS	NEG	NEG	POS	NEG	POS	NEG	NEG	POS	NEG	NEG	NEG	NEG	POS	NEG	POS	NEG	POS	NEG	POS	POS	NEG
03V12917	CC5-MRSA-II, Rhine-Hesse EMRSA/UK-EMRSA-3	POS	POS	NEG	NEG	POS	NEG	POS	POS	POS	NEG	POS	NEG	POS	POS	NEG	NEG	POS	NEG	POS	AMB	AMB	POS	POS	NEG	POS	POS	NEG	NEG	POS	NEG	NEG	POS	NEG	POS	NEG	NEG	POS	NEG	NEG	NEG	AMB	POS	NEG	POS	NEG	POS	NEG	POS	POS	NEG
03V14467	CC5-MRSA-II, Rhine-Hesse EMRSA/UK-EMRSA-3	POS	POS	AMB	NEG	POS	NEG	POS	POS	POS	NEG	POS	NEG	POS	POS	NEG	NEG	POS	NEG	POS	AMB	AMB	POS	POS	NEG	POS	POS	NEG	NEG	POS	NEG	NEG	POS	NEG	POS	NEG	NEG	POS	NEG	NEG	NEG	NEG	POS	NEG	POS	NEG	POS	NEG	POS	POS	NEG
03V19357	CC5-MRSA-II, Rhine-Hesse EMRSA/UK-EMRSA-3	POS	POS	NEG	NEG	POS	NEG	POS	POS	POS	NEG	POS	NEG	POS	POS	NEG	NEG	POS	NEG	POS	AMB	AMB	POS	POS	NEG	POS	POS	NEG	NEG	POS	NEG	NEG	POS	NEG	POS	NEG	NEG	POS	NEG	NEG	NEG	NEG	POS	NEG	POS	NEG	POS	NEG	POS	POS	NEG
03V20186	CC5-MRSA-II, Rhine-Hesse EMRSA/UK-EMRSA-3	POS	POS	NEG	NEG	POS	NEG	POS	POS	POS	NEG	POS	NEG	POS	POS	NEG	NEG	POS	NEG	POS	AMB	AMB	POS	POS	NEG	POS	POS	NEG	NEG	POS	NEG	NEG	POS	NEG	POS	NEG	NEG	POS	NEG	NEG	NEG	NEG	POS	NEG	POS	NEG	POS	NEG	POS	POS	NEG
03V25349	CC5-MRSA-II, Rhine-Hesse EMRSA/UK-EMRSA-3	POS	POS	AMB	NEG	POS	NEG	POS	POS	POS	NEG	POS	NEG	POS	POS	NEG	NEG	POS	NEG	POS	AMB	POS	AMB	POS	AMB	POS	POS	NEG	NEG	POS	POS	NEG	POS	NEG	POS	NEG	NEG	POS	AMB	NEG	NEG	POS	POS	NEG	POS	NEG	POS	NEG	POS	POS	NEG
03V28948	CC5-MRSA-II, Rhine-Hesse EMRSA/UK-EMRSA-3	POS	POS	NEG	NEG	POS	NEG	POS	POS	POS	NEG	POS	NEG	POS	POS	NEG	NEG	POS	NEG	POS	AMB	AMB	POS	POS	NEG	POS	POS	NEG	NEG	POS	NEG	NEG	POS	NEG	POS	NEG	NEG	POS	NEG	NEG	NEG	NEG	POS	NEG	POS	NEG	POS	NEG	POS	POS	NEG
03V30841	CC5-MRSA-II, Rhine-Hesse EMRSA/UK-EMRSA-3																																																		
03V32896	CC5-MRSA-II, Rhine-Hesse EMRSA/UK-EMRSA-3	POS	POS	NEG	NEG	POS	NEG	POS	POS	POS	NEG	POS	NEG	POS	POS	NEG	NEG	POS	NEG	POS	AMB	AMB	POS	POS	NEG	POS	POS	NEG	NEG	POS	NEG	NEG	POS	NEG	POS	NEG	NEG	POS	NEG	NEG	NEG	NEG	POS	NEG	POS	NEG	NEG	NEG	POS	POS	NEG
03V33415	CC5-MRSA-II, Rhine-Hesse EMRSA/UK-EMRSA-3	POS	POS	NEG	NEG	POS	NEG	POS	POS	POS	NEG	POS	NEG	POS	POS	NEG	NEG	POS	NEG	POS	AMB	AMB	POS	POS	NEG	POS	POS	NEG	NEG	POS	NEG	NEG	POS	NEG	POS	NEG	NEG	POS	NEG	NEG	NEG	NEG	POS	NEG	POS	NEG	POS	NEG	POS	POS	NEG
03V34628	CC5-MRSA-II, Rhine-Hesse EMRSA/UK-EMRSA-3	POS	POS	AMB	NEG	POS	AMB	POS	POS	POS	NEG	POS	NEG	POS	POS	NEG	NEG	POS	NEG	POS	AMB	POS	AMB	POS	AMB	POS	POS	NEG	NEG	POS	AMB	NEG	POS	NEG	POS	NEG	NEG	POS	AMB	NEG	NEG	AMB	POS	NEG	POS	NEG	POS	NEG	POS	POS	NEG
03V37078	CC5-MRSA-II, Rhine-Hesse EMRSA/UK-EMRSA-3	POS	POS	NEG	NEG	POS	NEG	POS	POS	POS	NEG	POS	NEG	POS	POS	NEG	NEG	POS	NEG	POS	AMB	AMB	POS	POS	NEG	POS	POS	NEG	NEG	POS	NEG	NEG	POS	NEG	POS	NEG	NEG	POS	NEG	NEG	NEG	NEG	POS	NEG	POS	NEG	NEG	NEG	POS	POS	NEG
03V41645	CC5-MRSA-II, Rhine-Hesse EMRSA/UK-EMRSA-3	POS	POS	NEG	NEG	POS	NEG	POS	POS	POS	NEG	POS	NEG	POS	POS	NEG	NEG	POS	NEG	POS	AMB	AMB	POS	POS	NEG	POS	POS	NEG	NEG	POS	NEG	NEG	POS	NEG	POS	NEG	NEG	POS	NEG	NEG	NEG	NEG	POS	NEG	POS	NEG	POS	NEG	POS	POS	NEG
03V03012	CC5-MRSA-IV, Paediatric clone	POS	POS	NEG	NEG	POS	NEG	POS	POS	POS	NEG	POS	NEG	POS	POS	NEG	NEG	POS	NEG	POS	POS	POS	POS	POS	NEG	POS	POS	NEG	NEG	POS	NEG	NEG	POS	NEG	POS	NEG	NEG	POS	NEG	NEG	NEG	NEG	POS	NEG	POS	NEG	NEG	NEG	POS	POS	NEG
03V02863	CC8/ST247-MRSA-I, North German/Iberian EMRSA	POS	POS	NEG	POS	NEG	NEG	POS	POS	POS	POS	NEG	NEG	POS	POS	POS	NEG	NEG	NEG	POS	AMB	POS	AMB	POS	NEG	POS	POS	NEG	NEG	POS	NEG	NEG	POS	POS	NEG	NEG	NEG	POS	NEG	NEG	POS	NEG	NEG	NEG	POS	POS	POS	POS	NEG	NEG	NEG
03V14882	CC8/ST254-MRSA-(atypical SCCmec), Hannover EMRSA	POS	POS	NEG	POS	NEG	NEG	POS	POS	POS	POS	NEG	NEG	POS	POS	POS	NEG	NEG	NEG	AMB	POS	POS	AMB	POS	NEG	POS	POS	NEG	NEG	POS	NEG	NEG	POS	POS	NEG	NEG	NEG	POS	NEG	NEG	POS	NEG	NEG	NEG	POS	POS	POS	POS	NEG	NEG	NEG
03B00404	CC22-MRSA-IV, Barnim/UK-EMRSA-15	POS	POS	NEG	NEG	POS	NEG	POS	POS	POS	NEG	NEG	POS	POS	POS	NEG	NEG	NEG	POS	NEG	POS	AMB	POS	POS	NEG	POS	POS	NEG	NEG	POS	NEG	NEG	NEG	NEG	NEG	NEG	NEG	AMB	NEG	NEG	NEG	NEG	POS	NEG	POS	NEG	AMB	NEG	POS	POS	AMB
03U00892	CC22-MRSA-IV, Barnim/UK-EMRSA-15																																																		
03V01422	CC22-MRSA-IV, Barnim/UK-EMRSA-15	POS	POS	NEG	NEG	POS	NEG	POS	POS	POS	NEG	NEG	POS	POS	POS	NEG	NEG	NEG	POS	NEG	POS	AMB	POS	POS	NEG	POS	POS	NEG	NEG	POS	NEG	NEG	NEG	NEG	NEG	NEG	NEG	POS	NEG	NEG	NEG	NEG	POS	NEG	POS	NEG	NEG	NEG	POS	POS	NEG
03V04424	CC22-MRSA-IV, Barnim/UK-EMRSA-15	POS	POS	NEG	NEG	POS	NEG	POS	POS	POS	NEG	NEG	POS	POS	POS	NEG	NEG	NEG	POS	NEG	POS	AMB	POS	POS	NEG	POS	POS	NEG	NEG	POS	NEG	NEG	AMB	NEG	NEG	NEG	NEG	POS	NEG	NEG	NEG	NEG	POS	NEG	POS	NEG	NEG	NEG	POS	POS	AMB
03V05690	CC22-MRSA-IV, Barnim/UK-EMRSA-15	POS	POS	NEG	NEG	POS	NEG	POS	POS	POS	NEG	NEG	POS	POS	POS	NEG	NEG	NEG	POS	NEG	POS	AMB	POS	POS	NEG	POS	POS	NEG	NEG	POS	NEG	NEG	NEG	NEG	NEG	NEG	NEG	NEG	NEG	NEG	NEG	NEG	POS	NEG	POS	NEG	NEG	NEG	POS	POS	AMB
03V07912	CC22-MRSA-IV, Barnim/UK-EMRSA-15	POS	POS	NEG	NEG	POS	NEG	POS	POS	POS	NEG	NEG	POS	POS	POS	NEG	NEG	NEG	POS	NEG	POS	POS	POS	POS	POS	POS	POS	NEG	NEG	POS	NEG	NEG	POS	NEG	NEG	NEG	NEG	POS	NEG	NEG	NEG	NEG	POS	NEG	POS	NEG	NEG	NEG	POS	POS	NEG
03V23798	CC22-MRSA-IV, Barnim/UK-EMRSA-15	POS	POS	NEG	NEG	POS	NEG	POS	POS	POS	NEG	NEG	POS	POS	POS	NEG	NEG	NEG	POS	NEG	POS	AMB	POS	POS	NEG	POS	POS	NEG	NEG	POS	NEG	NEG	NEG	NEG	NEG	NEG	NEG	NEG	NEG	NEG	NEG	NEG	POS	NEG	POS	NEG	NEG	NEG	POS	POS	NEG
03V25914	CC22-MRSA-IV, Barnim/UK-EMRSA-15	POS	POS	NEG	NEG	POS	NEG	POS	POS	POS	NEG	NEG	POS	POS	POS	NEG	NEG	NEG	POS	NEG	POS	AMB	POS	POS	NEG	POS	POS	NEG	NEG	POS	NEG	NEG	NEG	NEG	NEG	NEG	NEG	NEG	NEG	NEG	NEG	NEG	POS	NEG	POS	NEG	NEG	NEG	POS	POS	AMB
03V29751	CC22-MRSA-IV, Barnim/UK-EMRSA-15	POS	POS	NEG	NEG	POS	NEG	POS	POS	POS	NEG	NEG	POS	POS	POS	NEG	NEG	NEG	POS	NEG	POS	AMB	POS	POS	NEG	POS	POS	NEG	NEG	POS	NEG	NEG	NEG	NEG	NEG	NEG	NEG	AMB	NEG	NEG	NEG	NEG	POS	NEG	POS	NEG	NEG	NEG	POS	POS	NEG
03V30201	CC22-MRSA-IV, Barnim/UK-EMRSA-15	POS	POS	NEG	NEG	POS	NEG	POS	POS	POS	NEG	NEG	POS	POS	POS	NEG	NEG	NEG	POS	NEG	POS	AMB	POS	POS	AMB	POS	POS	NEG	NEG	POS	NEG	NEG	AMB	NEG	NEG	NEG	NEG	POS	AMB	NEG	NEG	NEG	POS	NEG	POS	NEG	NEG	NEG	POS	POS	AMB
03B01781	CC45-MRSA-IV, Berlin EMRSA	POS	POS	POS	NEG	NEG	AMB	NEG	POS	POS	POS	NEG	NEG	POS	AMB	NEG	POS	NEG	NEG	NEG	POS	AMB	POS	POS	POS	AMB	POS	NEG	NEG	NEG	NEG	POS	NEG	NEG	NEG	NEG	NEG	AMB	NEG	NEG	NEG	NEG	NEG	NEG	POS	NEG	AMB	NEG	POS	NEG	NEG
03U05014	CC45-MRSA-IV, Berlin EMRSA	POS	POS	POS	NEG	NEG	NEG	NEG	POS	POS	POS	NEG	NEG	POS	NEG	NEG	POS	NEG	NEG	NEG	POS	AMB	POS	POS	POS	AMB	POS	NEG	NEG	NEG	NEG	POS	NEG	NEG	NEG	NEG	NEG	POS	NEG	NEG	NEG	NEG	POS	NEG	POS	NEG	AMB	NEG	POS	NEG	NEG
03U11106	CC45-MRSA-IV, Berlin EMRSA	POS	POS	POS	NEG	NEG	NEG	NEG	POS	POS	POS	NEG	NEG	POS	AMB	NEG	POS	NEG	NEG	NEG	POS	AMB	POS	POS	POS	AMB	POS	NEG	NEG	NEG	AMB	POS	NEG	NEG	NEG	NEG	NEG	POS	AMB	NEG	NEG	POS	POS	NEG	POS	NEG	POS	NEG	POS	NEG	NEG
03V00742	CC45-MRSA-IV, Berlin EMRSA	POS	POS	POS	NEG	NEG	NEG	NEG	POS	POS	POS	NEG	NEG	POS	NEG	NEG	POS	NEG	NEG	NEG	POS	NEG	POS	POS	POS	AMB	POS	NEG	NEG	NEG	NEG	POS	NEG	NEG	NEG	NEG	NEG	AMB	NEG	NEG	NEG	NEG	NEG	NEG	POS	NEG	AMB	NEG	POS	NEG	NEG
03V01902	CC45-MRSA-IV, Berlin EMRSA	POS	POS	POS	NEG	NEG	NEG	NEG	POS	POS	POS	NEG	NEG	POS	NEG	NEG	POS	NEG	NEG	NEG	POS	AMB	POS	POS	POS	AMB	POS	NEG	NEG	NEG	NEG	POS	NEG	NEG	NEG	NEG	NEG	POS	AMB	NEG	NEG	NEG	POS	NEG	POS	NEG	NEG	NEG	POS	NEG	NEG
03V02256	CC45-MRSA-IV, Berlin EMRSA	POS	POS	POS	NEG	NEG	NEG	NEG	POS	POS	POS	NEG	NEG	POS	NEG	NEG	POS	NEG	NEG	NEG	POS	AMB	POS	POS	POS	AMB	POS	NEG	NEG	NEG	NEG	POS	NEG	NEG	NEG	NEG	NEG	POS	AMB	NEG	NEG	NEG	POS	NEG	POS	NEG	AMB	NEG	POS	NEG	NEG
03V02262	CC45-MRSA-IV, Berlin EMRSA	POS	POS	POS	NEG	NEG	NEG	NEG	POS	POS	POS	NEG	NEG	POS	NEG	NEG	POS	NEG	NEG	NEG	POS	NEG	POS	POS	POS	AMB	POS	NEG	NEG	NEG	NEG	POS	NEG	NEG	NEG	NEG	NEG	AMB	NEG	NEG	NEG	NEG	NEG	NEG	POS	NEG	NEG	NEG	POS	NEG	NEG
03V04701	CC45-MRSA-IV, Berlin EMRSA	POS	POS	POS	NEG	NEG	NEG	NEG	POS	POS	POS	NEG	NEG	POS	NEG	NEG	POS	NEG	NEG	NEG	POS	NEG	NEG	POS	POS	NEG	POS	NEG	NEG	NEG	NEG	POS	NEG	NEG	NEG	NEG	NEG	AMB	NEG	NEG	NEG	NEG	POS	NEG	POS	NEG	NEG	NEG	AMB	NEG	NEG
03V05042	CC45-MRSA-IV, Berlin EMRSA	POS	POS	POS	NEG	NEG	NEG	NEG	POS	POS	POS	NEG	NEG	POS	NEG	NEG	POS	NEG	NEG	NEG	POS	AMB	POS	POS	POS	AMB	POS	NEG	NEG	NEG	AMB	POS	NEG	NEG	NEG	NEG	NEG	POS	AMB	NEG	NEG	NEG	NEG	NEG	POS	NEG	NEG	NEG	POS	NEG	NEG
03V05377	CC45-MRSA-IV, Berlin EMRSA	POS	POS	POS	NEG	NEG	NEG	NEG	POS	POS	POS	NEG	NEG	POS	AMB	NEG	POS	NEG	NEG	NEG	POS	AMB	POS	POS	POS	AMB	POS	NEG	NEG	NEG	AMB	POS	NEG	NEG	NEG	NEG	NEG	POS	NEG	NEG	NEG	NEG	POS	NEG	POS	NEG	POS	NEG	POS	NEG	NEG
03V05507	CC45-MRSA-IV, Berlin EMRSA	POS	POS	POS	NEG	NEG	NEG	NEG	POS	POS	POS	NEG	NEG	POS	NEG	NEG	POS	NEG	NEG	NEG	POS	NEG	POS	POS	POS	AMB	POS	NEG	NEG	NEG	NEG	POS	NEG	NEG	NEG	NEG	NEG	AMB	NEG	NEG	NEG	NEG	NEG	NEG	POS	NEG	AMB	NEG	AMB	NEG	NEG
03V08214	CC45-MRSA-IV, Berlin EMRSA	POS	POS	POS	NEG	NEG	NEG	NEG	POS	POS	POS	NEG	NEG	POS	NEG	NEG	POS	NEG	NEG	NEG	POS	AMB	POS	POS	POS	AMB	POS	NEG	NEG	NEG	NEG	POS	NEG	NEG	NEG	NEG	NEG	POS	AMB	NEG	NEG	NEG	POS	NEG	POS	NEG	NEG	NEG	POS	NEG	NEG
03V08525	CC45-MRSA-IV, Berlin EMRSA	POS	POS	POS	NEG	NEG	NEG	NEG	POS	POS	POS	NEG	NEG	POS	NEG	NEG	POS	NEG	NEG	NEG	POS	NEG	NEG	POS	POS	AMB	POS	NEG	NEG	NEG	NEG	POS	NEG	NEG	NEG	NEG	NEG	AMB	NEG	NEG	NEG	NEG	NEG	NEG	POS	NEG	NEG	NEG	AMB	NEG	NEG
03V11910	CC45-MRSA-IV, Berlin EMRSA	POS	POS	POS	NEG	NEG	NEG	NEG	POS	POS	POS	NEG	NEG	POS	NEG	NEG	POS	NEG	NEG	NEG	POS	NEG	NEG	POS	POS	NEG	POS	NEG	NEG	NEG	NEG	POS	NEG	NEG	NEG	NEG	NEG	NEG	NEG	NEG	NEG	NEG	NEG	NEG	AMB	NEG	NEG	NEG	NEG	NEG	NEG
03V14481	CC45-MRSA-IV, Berlin EMRSA	POS	POS	POS	NEG	NEG	NEG	NEG	POS	POS	POS	NEG	NEG	POS	NEG	NEG	POS	NEG	NEG	NEG	POS	NEG	NEG	POS	POS	AMB	POS	NEG	NEG	NEG	NEG	POS	NEG	NEG	NEG	NEG	NEG	AMB	NEG	NEG	NEG	NEG	POS	NEG	POS	NEG	NEG	NEG	POS	NEG	NEG
03V15082	CC45-MRSA-IV, Berlin EMRSA	POS	POS	POS	NEG	NEG	AMB	NEG	POS	POS	POS	NEG	NEG	POS	POS	NEG	POS	NEG	NEG	NEG	POS	AMB	POS	POS	POS	AMB	POS	NEG	NEG	NEG	POS	POS	NEG	NEG	NEG	NEG	NEG	POS	POS	NEG	NEG	AMB	NEG	NEG	POS	NEG	POS	NEG	POS	NEG	NEG
03V17508	CC45-MRSA-IV, Berlin EMRSA	POS	POS	POS	NEG	NEG	NEG	NEG	POS	POS	POS	NEG	NEG	POS	NEG	NEG	POS	NEG	NEG	NEG	POS	NEG	NEG	POS	POS	NEG	POS	NEG	NEG	NEG	NEG	POS	NEG	NEG	NEG	NEG	NEG	NEG	NEG	NEG	NEG	NEG	NEG	NEG	AMB	NEG	NEG	NEG	NEG	NEG	NEG
03V20150	CC45-MRSA-IV, Berlin EMRSA	POS	POS	POS	NEG	NEG	NEG	NEG	POS	POS	POS	NEG	NEG	POS	NEG	NEG	POS	NEG	NEG	NEG	POS	NEG	NEG	POS	POS	NEG	POS	NEG	NEG	NEG	NEG	POS	NEG	NEG	NEG	NEG	NEG	NEG	NEG	NEG	NEG	NEG	NEG	NEG	AMB	NEG	NEG	NEG	NEG	NEG	NEG
03V22437	CC45-MRSA-IV, Berlin EMRSA	POS	POS	POS	NEG	NEG	NEG	NEG	POS	POS	POS	NEG	NEG	POS	NEG	NEG	POS	NEG	NEG	NEG	POS	NEG	NEG	POS	POS	NEG	POS	NEG	NEG	NEG	NEG	POS	NEG	NEG	NEG	NEG	NEG	NEG	NEG	NEG	NEG	NEG	POS	NEG	AMB	NEG	NEG	NEG	NEG	NEG	NEG
03V24471	CC45-MRSA-IV, Berlin EMRSA	POS	POS	POS	NEG	NEG	NEG	NEG	POS	POS	POS	NEG	NEG	POS	NEG	NEG	POS	NEG	NEG	NEG	POS	NEG	POS	POS	POS	AMB	POS	NEG	NEG	NEG	NEG	POS	NEG	NEG	NEG	NEG	NEG	AMB	NEG	NEG	NEG	NEG	POS	NEG	POS	NEG	AMB	NEG	POS	NEG	NEG
03V28070	CC45-MRSA-IV, Berlin EMRSA	POS	POS	POS	NEG	NEG	NEG	NEG	NEG	NEG	NEG	NEG	NEG	POS	NEG	NEG	POS	NEG	NEG	NEG	POS	NEG	NEG	POS	POS	NEG	POS	NEG	NEG	NEG	NEG	POS	NEG	NEG	NEG	NEG	NEG	NEG	NEG	NEG	NEG	NEG	POS	NEG	NEG	NEG	NEG	NEG	NEG	NEG	NEG
03V28448	CC45-MRSA-IV, Berlin EMRSA	POS	POS	POS	NEG	NEG	NEG	NEG	POS	POS	POS	NEG	NEG	POS	NEG	NEG	POS	NEG	NEG	NEG	POS	NEG	POS	POS	POS	AMB	POS	NEG	NEG	NEG	NEG	POS	NEG	NEG	NEG	NEG	NEG	AMB	NEG	NEG	NEG	NEG	POS	NEG	POS	NEG	NEG	NEG	POS	NEG	NEG
03V34589	CC45-MRSA-IV, Berlin EMRSA	POS	POS	POS	NEG	NEG	NEG	NEG	POS	POS	POS	NEG	NEG	POS	NEG	NEG	POS	NEG	NEG	NEG	POS	AMB	POS	POS	POS	AMB	POS	NEG	NEG	NEG	NEG	POS	NEG	NEG	NEG	NEG	NEG	AMB	NEG	NEG	NEG	NEG	POS	NEG	POS	NEG	NEG	NEG	POS	NEG	NEG
03V39774	CC45-MRSA-IV, Berlin EMRSA	POS	POS	POS	NEG	NEG	NEG	NEG	POS	POS	POS	NEG	NEG	POS	NEG	NEG	POS	NEG	NEG	NEG	POS	NEG	NEG	POS	POS	AMB	POS	NEG	NEG	NEG	NEG	POS	NEG	NEG	NEG	NEG	NEG	AMB	NEG	NEG	NEG	NEG	NEG	NEG	POS	NEG	NEG	NEG	POS	NEG	NEG
**2004**																																																			
04B05423	CC5-MRSA-II, Rhine-Hesse EMRSA/UK-EMRSA-3	POS	POS	NEG	NEG	POS	NEG	POS	POS	POS	NEG	POS	NEG	POS	POS	NEG	NEG	POS	NEG	POS	AMB	NEG	POS	POS	NEG	POS	POS	NEG	NEG	POS	NEG	NEG	POS	NEG	POS	NEG	NEG	POS	NEG	NEG	NEG	NEG	POS	NEG	POS	NEG	POS	NEG	POS	POS	NEG
04U02171	CC5-MRSA-II, Rhine-Hesse EMRSA/UK-EMRSA-3	POS	POS	NEG	NEG	POS	NEG	POS	POS	POS	NEG	POS	NEG	POS	POS	NEG	NEG	POS	NEG	POS	AMB	AMB	POS	POS	AMB	POS	POS	NEG	NEG	POS	AMB	NEG	POS	NEG	POS	NEG	NEG	POS	AMB	NEG	NEG	AMB	POS	NEG	POS	NEG	POS	NEG	POS	POS	NEG
04U03682	CC5-MRSA-II, Rhine-Hesse EMRSA/UK-EMRSA-3	POS	POS	NEG	NEG	POS	NEG	POS	POS	POS	NEG	POS	NEG	POS	POS	NEG	NEG	POS	NEG	POS	AMB	NEG	POS	POS	NEG	POS	POS	NEG	NEG	POS	NEG	NEG	POS	NEG	POS	NEG	NEG	POS	NEG	NEG	NEG	NEG	POS	NEG	POS	NEG	POS	NEG	POS	POS	NEG
04U06618	CC5-MRSA-II, Rhine-Hesse EMRSA/UK-EMRSA-3	POS	POS	AMB	NEG	POS	NEG	POS	POS	POS	NEG	POS	NEG	POS	POS	NEG	NEG	POS	NEG	POS	AMB	POS	AMB	POS	AMB	POS	POS	NEG	NEG	POS	AMB	NEG	POS	NEG	POS	NEG	NEG	POS	AMB	NEG	NEG	AMB	POS	NEG	POS	NEG	POS	NEG	POS	POS	NEG
04U12578	CC5-MRSA-II, Rhine-Hesse EMRSA/UK-EMRSA-3	POS	POS	NEG	NEG	POS	NEG	POS	POS	POS	NEG	POS	NEG	POS	POS	NEG	NEG	POS	NEG	POS	AMB	AMB	POS	POS	NEG	POS	POS	NEG	NEG	POS	NEG	NEG	POS	NEG	POS	NEG	NEG	POS	NEG	NEG	NEG	NEG	POS	NEG	POS	NEG	POS	NEG	POS	POS	NEG
04V02465	CC5-MRSA-II, Rhine-Hesse EMRSA/UK-EMRSA-3	POS	POS	NEG	NEG	POS	NEG	POS	POS	POS	NEG	POS	NEG	POS	POS	NEG	NEG	POS	NEG	POS	AMB	AMB	POS	POS	NEG	POS	POS	NEG	NEG	POS	NEG	NEG	POS	NEG	POS	NEG	NEG	POS	NEG	NEG	NEG	NEG	POS	NEG	POS	NEG	POS	NEG	POS	POS	NEG
04V03642	CC5-MRSA-II, Rhine-Hesse EMRSA/UK-EMRSA-3	POS	POS	NEG	NEG	POS	NEG	POS	POS	POS	NEG	POS	NEG	POS	POS	NEG	NEG	POS	NEG	POS	AMB	NEG	POS	POS	NEG	POS	POS	NEG	NEG	POS	NEG	NEG	POS	NEG	POS	NEG	NEG	POS	NEG	NEG	NEG	NEG	POS	NEG	POS	NEG	POS	NEG	POS	POS	NEG
04V07657	CC5-MRSA-II, Rhine-Hesse EMRSA/UK-EMRSA-3	POS	POS	NEG	NEG	POS	NEG	POS	POS	POS	NEG	POS	NEG	POS	POS	NEG	NEG	POS	NEG	POS	AMB	NEG	POS	POS	NEG	POS	POS	NEG	NEG	POS	NEG	NEG	POS	NEG	POS	NEG	NEG	POS	NEG	NEG	NEG	NEG	POS	NEG	POS	NEG	POS	NEG	POS	POS	NEG
04V12223	CC5-MRSA-II, Rhine-Hesse EMRSA/UK-EMRSA-3	POS	POS	NEG	NEG	POS	NEG	POS	POS	POS	NEG	POS	NEG	POS	POS	NEG	NEG	POS	NEG	POS	AMB	NEG	POS	POS	NEG	POS	POS	NEG	NEG	POS	NEG	NEG	POS	NEG	POS	NEG	NEG	POS	NEG	NEG	NEG	NEG	POS	NEG	POS	NEG	POS	NEG	POS	POS	NEG
04V12529	CC5-MRSA-II, Rhine-Hesse EMRSA/UK-EMRSA-3	POS	POS	NEG	NEG	POS	NEG	POS	POS	POS	NEG	POS	NEG	POS	POS	NEG	NEG	POS	NEG	POS	AMB	NEG	POS	POS	NEG	POS	POS	NEG	NEG	POS	NEG	NEG	POS	NEG	POS	NEG	NEG	POS	NEG	NEG	NEG	NEG	POS	NEG	POS	NEG	POS	NEG	POS	POS	NEG
04V13633	CC5-MRSA-II, Rhine-Hesse EMRSA/UK-EMRSA-3	POS	POS	NEG	NEG	POS	NEG	POS	POS	POS	NEG	POS	NEG	POS	POS	NEG	NEG	POS	NEG	POS	AMB	NEG	POS	POS	NEG	POS	POS	NEG	NEG	POS	NEG	NEG	POS	NEG	POS	NEG	NEG	POS	NEG	NEG	NEG	NEG	POS	NEG	POS	NEG	POS	NEG	POS	POS	NEG
04V16499	CC5-MRSA-II, Rhine-Hesse EMRSA/UK-EMRSA-3	POS	POS	NEG	NEG	POS	NEG	POS	POS	POS	NEG	POS	NEG	POS	POS	NEG	NEG	POS	NEG	POS	AMB	AMB	POS	POS	AMB	POS	POS	NEG	NEG	POS	AMB	NEG	POS	NEG	POS	NEG	NEG	POS	AMB	NEG	NEG	NEG	POS	NEG	POS	NEG	POS	NEG	POS	POS	NEG
04V17015	CC5-MRSA-II, Rhine-Hesse EMRSA/UK-EMRSA-3	POS	POS	AMB	NEG	POS	NEG	POS	POS	POS	NEG	POS	NEG	POS	POS	NEG	NEG	POS	NEG	POS	AMB	POS	AMB	POS	AMB	POS	POS	NEG	NEG	POS	AMB	NEG	POS	NEG	POS	NEG	NEG	POS	NEG	NEG	NEG	AMB	POS	NEG	POS	NEG	POS	NEG	POS	POS	NEG
04V20684	CC5-MRSA-II, Rhine-Hesse EMRSA/UK-EMRSA-3																																																		
04V24615	CC5-MRSA-II, Rhine-Hesse EMRSA/UK-EMRSA-3	POS	POS	NEG	NEG	POS	NEG	POS	POS	POS	NEG	POS	NEG	POS	POS	NEG	NEG	POS	NEG	POS	AMB	AMB	POS	POS	NEG	POS	POS	NEG	NEG	POS	NEG	NEG	POS	NEG	POS	NEG	NEG	POS	NEG	NEG	NEG	NEG	POS	NEG	POS	NEG	POS	NEG	POS	POS	NEG
04V24901	CC5-MRSA-II, Rhine-Hesse EMRSA/UK-EMRSA-3	POS	POS	NEG	NEG	POS	NEG	POS	POS	POS	NEG	POS	NEG	POS	POS	NEG	NEG	POS	NEG	POS	AMB	AMB	POS	POS	NEG	POS	POS	NEG	NEG	POS	NEG	NEG	POS	NEG	POS	NEG	NEG	POS	NEG	NEG	NEG	NEG	POS	NEG	POS	NEG	POS	NEG	POS	POS	NEG
04V25081	CC5-MRSA-II, Rhine-Hesse EMRSA/UK-EMRSA-3	POS	POS	NEG	NEG	POS	NEG	POS	POS	POS	NEG	POS	NEG	POS	POS	NEG	NEG	POS	NEG	POS	AMB	AMB	POS	POS	NEG	POS	POS	NEG	NEG	POS	NEG	NEG	POS	NEG	POS	NEG	NEG	POS	NEG	NEG	NEG	NEG	POS	NEG	POS	NEG	POS	NEG	POS	POS	NEG
04V27293	CC5-MRSA-II, Rhine-Hesse EMRSA/UK-EMRSA-3	POS	POS	NEG	NEG	POS	NEG	POS	POS	POS	NEG	POS	NEG	POS	POS	NEG	NEG	POS	NEG	POS	AMB	AMB	POS	POS	NEG	POS	POS	NEG	NEG	POS	NEG	NEG	POS	NEG	POS	NEG	NEG	POS	NEG	NEG	NEG	NEG	POS	NEG	POS	NEG	POS	NEG	POS	POS	NEG
04V27311	CC5-MRSA-II, Rhine-Hesse EMRSA/UK-EMRSA-3	POS	POS	NEG	NEG	POS	NEG	POS	POS	POS	NEG	POS	NEG	POS	POS	NEG	NEG	POS	NEG	POS	AMB	AMB	POS	POS	NEG	POS	POS	NEG	NEG	POS	NEG	NEG	POS	NEG	POS	NEG	NEG	POS	NEG	NEG	NEG	NEG	POS	NEG	POS	NEG	POS	NEG	POS	POS	NEG
04V33459	CC5-MRSA-II, Rhine-Hesse EMRSA/UK-EMRSA-3	POS	POS	NEG	NEG	POS	NEG	POS	POS	POS	NEG	POS	NEG	POS	POS	NEG	NEG	POS	NEG	POS	AMB	AMB	POS	POS	NEG	POS	POS	NEG	NEG	POS	NEG	NEG	POS	NEG	POS	NEG	NEG	POS	AMB	NEG	NEG	NEG	POS	NEG	POS	NEG	POS	NEG	POS	POS	NEG
04V39909	CC5-MRSA-IV&VI	POS	POS	NEG	NEG	POS	NEG	POS	NEG	NEG	NEG	NEG	NEG	POS	POS	NEG	NEG	POS	NEG	POS	NEG	NEG	POS	POS	NEG	POS	POS	NEG	NEG	POS	AMB	NEG	POS	NEG	AMB	NEG	NEG	POS	NEG	NEG	NEG	NEG	POS	NEG	POS	NEG	POS	NEG	POS	POS	NEG
04U02421	CC5-MRSA-IV, Paediatric clone	POS	POS	NEG	NEG	POS	NEG	POS	POS	POS	NEG	POS	NEG	POS	POS	NEG	NEG	POS	NEG	POS	POS	POS	POS	POS	NEG	POS	POS	NEG	NEG	POS	NEG	NEG	POS	NEG	POS	NEG	NEG	POS	NEG	NEG	NEG	NEG	POS	NEG	POS	NEG	POS	NEG	POS	POS	NEG
04V09807	CC80-MRSA-IV [PVL+], European caMRSA Clone																																																		
04V16073	CC80-MRSA-IV [PVL+], European caMRSA Clone	POS	POS	NEG	POS	NEG	NEG	POS	POS	POS	NEG	NEG	POS	POS	POS	NEG	POS	NEG	NEG	POS	NEG	NEG	POS	POS	NEG	POS	POS	NEG	NEG	NEG	NEG	NEG	NEG	NEG	NEG	NEG	NEG	NEG	NEG	POS	NEG	NEG	NEG	NEG	POS	NEG	AMB	NEG	POS	POS	NEG
04V23548	CC80-MRSA-IV [PVL+], European caMRSA Clone																																																		
04V03289	CC22-MRSA-IV, Barnim/UK-EMRSA-15																																																		
04V03847	CC22-MRSA-IV, Barnim/UK-EMRSA-15																																																		
04V04595	CC22-MRSA-IV, Barnim/UK-EMRSA-15	POS	POS	NEG	NEG	POS	NEG	POS	POS	POS	NEG	NEG	POS	POS	POS	NEG	NEG	NEG	POS	NEG	POS	AMB	POS	POS	AMB	POS	POS	NEG	NEG	POS	NEG	NEG	AMB	NEG	NEG	NEG	NEG	POS	NEG	NEG	NEG	NEG	POS	NEG	POS	NEG	NEG	NEG	POS	POS	AMB
04V05029	CC22-MRSA-IV, Barnim/UK-EMRSA-15	POS	POS	NEG	NEG	POS	NEG	POS	NEG	AMB	NEG	NEG	AMB	POS	POS	NEG	NEG	NEG	POS	NEG	POS	NEG	POS	POS	NEG	POS	POS	NEG	NEG	POS	NEG	NEG	NEG	NEG	NEG	NEG	NEG	NEG	NEG	NEG	NEG	NEG	AMB	NEG	POS	NEG	NEG	NEG	POS	POS	NEG
04V07875	CC22-MRSA-IV, Barnim/UK-EMRSA-15	POS	POS	NEG	NEG	POS	NEG	POS	NEG	NEG	NEG	NEG	NEG	POS	POS	NEG	NEG	NEG	POS	NEG	POS	NEG	POS	POS	NEG	POS	POS	NEG	NEG	AMB	NEG	NEG	NEG	NEG	NEG	NEG	NEG	NEG	NEG	NEG	NEG	NEG	NEG	NEG	POS	NEG	NEG	NEG	POS	NEG	NEG
04V14909	CC22-MRSA-IV, Barnim/UK-EMRSA-15	POS	POS	NEG	NEG	POS	NEG	POS	POS	POS	NEG	NEG	POS	POS	POS	NEG	NEG	NEG	POS	NEG	POS	NEG	POS	POS	NEG	POS	POS	NEG	NEG	POS	NEG	NEG	NEG	NEG	NEG	NEG	NEG	NEG	NEG	NEG	NEG	NEG	POS	NEG	POS	NEG	NEG	NEG	POS	POS	NEG
04V18654	CC22-MRSA-IV, Barnim/UK-EMRSA-15	POS	POS	NEG	NEG	POS	NEG	POS	POS	POS	NEG	NEG	POS	POS	POS	NEG	NEG	NEG	POS	NEG	POS	AMB	POS	POS	NEG	POS	POS	NEG	NEG	POS	NEG	NEG	NEG	NEG	NEG	NEG	NEG	AMB	NEG	NEG	NEG	NEG	POS	NEG	POS	NEG	NEG	NEG	POS	POS	AMB
04V18815	CC22-MRSA-IV, Barnim/UK-EMRSA-15	POS	POS	NEG	NEG	POS	NEG	POS	POS	POS	NEG	NEG	POS	POS	POS	NEG	NEG	NEG	POS	NEG	POS	POS	AMB	POS	AMB	POS	POS	NEG	NEG	POS	AMB	NEG	AMB	NEG	NEG	NEG	NEG	POS	AMB	NEG	NEG	NEG	POS	NEG	POS	NEG	AMB	NEG	POS	POS	POS
04V29721	CC22-MRSA-IV, Barnim/UK-EMRSA-15	POS	POS	NEG	NEG	POS	NEG	POS	POS	POS	NEG	NEG	AMB	POS	POS	NEG	NEG	NEG	POS	NEG	POS	AMB	POS	POS	NEG	POS	POS	NEG	NEG	POS	NEG	NEG	NEG	NEG	NEG	NEG	NEG	NEG	NEG	NEG	NEG	NEG	POS	NEG	POS	NEG	NEG	NEG	POS	POS	NEG
04V32759	CC22-MRSA-IV, Barnim/UK-EMRSA-15	POS	POS	NEG	NEG	POS	NEG	POS	POS	POS	NEG	NEG	POS	POS	POS	NEG	NEG	NEG	POS	NEG	POS	AMB	POS	POS	NEG	POS	POS	NEG	NEG	POS	NEG	NEG	NEG	NEG	NEG	NEG	NEG	NEG	NEG	NEG	NEG	NEG	POS	NEG	POS	NEG	NEG	NEG	POS	POS	NEG
04V32919	CC22-MRSA-IV, Barnim/UK-EMRSA-15	POS	POS	AMB	NEG	POS	POS	POS	POS	POS	NEG	NEG	POS	POS	POS	NEG	NEG	NEG	POS	AMB	POS	POS	AMB	POS	AMB	POS	POS	NEG	NEG	POS	POS	NEG	POS	NEG	NEG	NEG	NEG	POS	NEG	NEG	NEG	POS	POS	NEG	POS	NEG	POS	NEG	POS	POS	POS
04V33780	CC22-MRSA-IV, Barnim/UK-EMRSA-15	POS	POS	NEG	NEG	POS	NEG	POS	POS	POS	NEG	NEG	POS	POS	POS	NEG	NEG	NEG	POS	NEG	POS	AMB	POS	POS	NEG	POS	POS	NEG	NEG	POS	NEG	NEG	NEG	NEG	NEG	NEG	NEG	AMB	NEG	NEG	NEG	NEG	POS	NEG	POS	NEG	NEG	NEG	POS	POS	NEG
04V35396	CC22-MRSA-IV, Barnim/UK-EMRSA-15	POS	POS	NEG	NEG	POS	NEG	POS	POS	POS	NEG	NEG	POS	POS	POS	NEG	NEG	NEG	POS	NEG	POS	POS	AMB	POS	AMB	POS	POS	NEG	NEG	POS	AMB	NEG	POS	NEG	NEG	NEG	NEG	POS	AMB	NEG	NEG	AMB	POS	NEG	POS	NEG	POS	NEG	POS	POS	POS
04V37107	CC22-MRSA-IV, Barnim/UK-EMRSA-15	POS	POS	NEG	NEG	POS	NEG	POS	POS	POS	NEG	NEG	POS	POS	POS	NEG	NEG	NEG	POS	NEG	POS	AMB	POS	POS	NEG	POS	POS	NEG	NEG	POS	NEG	NEG	NEG	NEG	NEG	NEG	NEG	POS	NEG	NEG	NEG	NEG	POS	NEG	POS	NEG	NEG	NEG	POS	POS	NEG
04V37518	CC22-MRSA-IV, Barnim/UK-EMRSA-15	POS	POS	NEG	NEG	POS	NEG	POS	POS	POS	NEG	NEG	POS	POS	POS	NEG	NEG	NEG	POS	NEG	POS	AMB	POS	POS	NEG	POS	POS	NEG	NEG	POS	NEG	NEG	NEG	NEG	NEG	NEG	NEG	NEG	NEG	NEG	NEG	NEG	POS	NEG	POS	NEG	NEG	NEG	POS	POS	NEG
04V38199	CC22-MRSA-IV, Barnim/UK-EMRSA-15	POS	POS	NEG	NEG	POS	NEG	POS	POS	POS	NEG	NEG	POS	POS	POS	NEG	NEG	NEG	POS	NEG	POS	AMB	POS	POS	NEG	POS	POS	NEG	NEG	POS	NEG	NEG	POS	NEG	NEG	NEG	NEG	POS	NEG	NEG	NEG	NEG	POS	NEG	POS	NEG	NEG	NEG	POS	POS	NEG
04V38358	CC22-MRSA-IV, Barnim/UK-EMRSA-15	POS	POS	NEG	NEG	POS	NEG	POS	POS	POS	NEG	NEG	AMB	POS	POS	NEG	NEG	NEG	POS	NEG	POS	NEG	POS	POS	NEG	POS	POS	NEG	NEG	POS	NEG	NEG	NEG	NEG	NEG	NEG	NEG	NEG	NEG	NEG	NEG	NEG	POS	NEG	POS	NEG	NEG	NEG	POS	POS	NEG
04V38902	CC22-MRSA-IV, Barnim/UK-EMRSA-15	POS	POS	NEG	NEG	POS	NEG	POS	POS	POS	NEG	NEG	NEG	POS	POS	NEG	NEG	NEG	POS	NEG	POS	NEG	POS	POS	NEG	POS	POS	NEG	NEG	POS	NEG	NEG	NEG	NEG	NEG	NEG	NEG	NEG	NEG	NEG	NEG	NEG	POS	NEG	POS	NEG	NEG	NEG	POS	POS	NEG
04V39203	CC22-MRSA-IV, Barnim/UK-EMRSA-15	POS	POS	NEG	NEG	POS	NEG	POS	POS	POS	NEG	NEG	POS	POS	POS	NEG	NEG	NEG	POS	NEG	POS	AMB	POS	POS	AMB	POS	POS	NEG	NEG	POS	NEG	NEG	AMB	NEG	NEG	NEG	NEG	POS	NEG	NEG	NEG	NEG	POS	NEG	POS	NEG	NEG	NEG	POS	POS	NEG
04V40423	CC22-MRSA-IV, Barnim/UK-EMRSA-15	POS	POS	NEG	NEG	POS	NEG	POS	POS	POS	NEG	NEG	AMB	POS	POS	NEG	NEG	NEG	POS	NEG	POS	NEG	POS	POS	NEG	POS	POS	NEG	NEG	POS	NEG	NEG	NEG	NEG	NEG	NEG	NEG	NEG	NEG	NEG	NEG	NEG	POS	NEG	POS	NEG	NEG	NEG	POS	POS	NEG
04V08036	CC45-MRSA-IV [ACME+]	POS	POS	POS	NEG	NEG	#ZAHL!	NEG	POS	POS	POS	NEG	NEG	POS	POS	NEG	POS	NEG	NEG	NEG	POS	AMB	POS	POS	POS	AMB	POS	NEG	NEG	NEG	NEG	POS	NEG	NEG	NEG	NEG	NEG	POS	AMB	NEG	NEG	NEG	NEG	NEG	POS	NEG	POS	NEG	POS	NEG	NEG
04U01119	CC45-MRSA-IV, Berlin EMRSA	POS	POS	POS	NEG	NEG	NEG	NEG	POS	POS	POS	NEG	NEG	POS	NEG	NEG	POS	NEG	NEG	NEG	POS	NEG	POS	POS	POS	AMB	POS	NEG	NEG	NEG	NEG	POS	NEG	NEG	NEG	NEG	NEG	AMB	NEG	NEG	NEG	NEG	NEG	NEG	POS	NEG	AMB	NEG	POS	NEG	NEG
04V01185	CC45-MRSA-IV, Berlin EMRSA	POS	POS	POS	NEG	NEG	NEG	NEG	POS	POS	POS	NEG	NEG	POS	NEG	NEG	POS	NEG	NEG	NEG	POS	NEG	POS	POS	POS	NEG	POS	NEG	NEG	NEG	NEG	POS	NEG	NEG	NEG	NEG	NEG	NEG	NEG	NEG	NEG	NEG	POS	NEG	POS	NEG	NEG	NEG	AMB	NEG	NEG
04V06276	CC45-MRSA-IV, Berlin EMRSA	POS	POS	POS	NEG	NEG	NEG	NEG	POS	POS	POS	NEG	NEG	POS	NEG	NEG	POS	NEG	NEG	NEG	POS	NEG	POS	POS	POS	AMB	POS	NEG	NEG	NEG	NEG	POS	NEG	NEG	NEG	NEG	NEG	AMB	NEG	NEG	NEG	NEG	POS	NEG	POS	NEG	NEG	NEG	POS	NEG	NEG
04V06677	CC45-MRSA-IV, Berlin EMRSA	POS	POS	POS	NEG	NEG	NEG	NEG	POS	POS	POS	NEG	NEG	POS	NEG	NEG	POS	NEG	NEG	NEG	POS	NEG	NEG	POS	POS	NEG	POS	NEG	NEG	NEG	NEG	POS	NEG	NEG	NEG	NEG	NEG	NEG	NEG	NEG	NEG	NEG	POS	NEG	AMB	NEG	NEG	NEG	NEG	NEG	NEG
04V06688	CC45-MRSA-IV, Berlin EMRSA	POS	POS	POS	NEG	NEG	NEG	NEG	POS	POS	POS	NEG	NEG	POS	NEG	NEG	POS	NEG	NEG	NEG	POS	NEG	POS	POS	POS	AMB	POS	NEG	NEG	NEG	NEG	POS	NEG	NEG	NEG	NEG	NEG	POS	NEG	NEG	NEG	NEG	POS	NEG	POS	NEG	AMB	NEG	POS	NEG	NEG
04V14835	CC45-MRSA-IV, Berlin EMRSA	POS	POS	POS	NEG	NEG	AMB	NEG	POS	POS	POS	NEG	NEG	POS	AMB	NEG	POS	NEG	NEG	NEG	POS	AMB	POS	POS	POS	AMB	POS	NEG	NEG	NEG	NEG	POS	NEG	NEG	NEG	NEG	NEG	POS	NEG	NEG	NEG	NEG	POS	NEG	POS	NEG	NEG	NEG	POS	NEG	NEG
04V22079	CC45-MRSA-IV, Berlin EMRSA	POS	POS	POS	NEG	NEG	NEG	NEG	POS	POS	POS	NEG	NEG	POS	NEG	NEG	POS	NEG	NEG	NEG	POS	NEG	NEG	POS	POS	AMB	POS	NEG	NEG	NEG	NEG	POS	NEG	NEG	NEG	NEG	NEG	AMB	NEG	NEG	NEG	NEG	POS	NEG	POS	NEG	NEG	NEG	POS	NEG	NEG
04V29100	CC45-MRSA-IV, Berlin EMRSA																																																		
04V31370	CC45-MRSA-IV, Berlin EMRSA	POS	POS	POS	NEG	NEG	AMB	NEG	POS	POS	POS	NEG	NEG	POS	NEG	NEG	POS	NEG	NEG	NEG	POS	NEG	POS	POS	POS	NEG	POS	NEG	NEG	NEG	AMB	POS	NEG	NEG	NEG	NEG	NEG	NEG	AMB	NEG	NEG	NEG	POS	NEG	POS	NEG	AMB	NEG	NEG	NEG	NEG
04V38442	CC45-MRSA-IV, Berlin EMRSA	POS	POS	POS	NEG	NEG	AMB	NEG	POS	POS	POS	NEG	NEG	POS	AMB	NEG	POS	NEG	NEG	NEG	POS	AMB	POS	POS	POS	AMB	POS	NEG	NEG	NEG	AMB	POS	NEG	NEG	NEG	NEG	NEG	POS	AMB	NEG	NEG	NEG	POS	NEG	POS	NEG	POS	NEG	POS	NEG	NEG
04V39012	CC45-MRSA-IV, Berlin EMRSA	POS	POS	POS	NEG	NEG	NEG	NEG	POS	POS	POS	NEG	NEG	POS	AMB	NEG	POS	NEG	NEG	NEG	POS	AMB	POS	POS	POS	AMB	POS	NEG	NEG	NEG	AMB	POS	NEG	NEG	NEG	NEG	NEG	POS	NEG	NEG	NEG	NEG	POS	NEG	POS	NEG	AMB	NEG	POS	NEG	NEG
04V39577	CC45-MRSA-IV, Berlin EMRSA	POS	POS	POS	NEG	POS	NEG	AMB	POS	POS	POS	NEG	NEG	POS	POS	NEG	POS	NEG	AMB	NEG	POS	AMB	POS	POS	POS	AMB	POS	NEG	NEG	AMB	AMB	POS	NEG	NEG	NEG	NEG	NEG	POS	AMB	NEG	NEG	NEG	NEG	NEG	POS	NEG	NEG	NEG	POS	NEG	NEG
04V22872	CC152-MRSA-V [PVL+]	NEG	NEG	NEG	NEG	NEG	NEG	NEG	POS	POS	POS	NEG	NEG	POS	POS	NEG	NEG	NEG	NEG	AMB	POS	POS	NEG	POS	NEG	NEG	NEG	POS	NEG	POS	AMB	NEG	POS	NEG	NEG	POS	POS	POS	AMB	POS	NEG	POS	NEG	NEG	POS	POS	NEG	POS	NEG	NEG	POS

ISOLATE:	STRAIN	WARD	AGE	GENDER	SPECIES MARKER							REGULATORY GENES								METHICILLIN RESISTANCE AND SCCmec TYPING
					Ribosomal sequence from S. aureus	glyceraldehyde 3-phosphate dehydro-genase, locus 1	katalase A	coagulase	thermo-stable extracellular nuclease	Protein A	IgG-binding protein	staphylo-coccal accessory regulator A	histidine protein kinase, sae locus	sensor protein	accessory gene regulator allele I	accessory gene regulator allele II	accessory gene regulator allele III	accessory gene regulator allele IV	haemolysin delta	alternate penicillin binding protein 2, defining MRSA
					rrnD1 (S. aureus)	gapA	katA	coA	nuc1	spa	sbi	sarA	saeS	vraS	agrI	agrII	agrIII	agrIV	hld	mecA
**2005**																				
05B01179	CC5/ST228-MRSA-I, South German EMRSA	MED-WARD	77	m	POS	POS	POS	AMB	POS	POS	POS	POS	POS	POS	NEG	POS	NEG	NEG	POS	POS
05B02687	CC5/ST228-MRSA-I, South German EMRSA	SUR-WARD	58	m	POS	POS	POS	POS	POS	POS	POS	POS	POS	POS	NEG	POS	NEG	NEG	POS	POS
05V08483	CC5/ST228-MRSA-I, South German EMRSA	SUR-OPD	26	m	POS	POS	POS	POS	POS	POS	POS	POS	POS	POS	NEG	POS	NEG	NEG	POS	POS
05V19001	CC5/ST228-MRSA-I, South German EMRSA	SUR-WARD	31	m	POS	POS	POS	POS	POS	POS	POS	POS	POS	POS	NEG	POS	NEG	NEG	POS	POS
05B04626	CC5-MRSA-II, Rhine-Hesse EMRSA/UK-EMRSA-3	SUR-ICU	54	m	POS	POS	POS	NEG	POS	POS	POS	POS	POS	POS	NEG	POS	NEG	NEG	AMB	POS
05B07903	CC5-MRSA-II, Rhine-Hesse EMRSA/UK-EMRSA-3	MED-icu	55	m	POS	POS	POS	POS	POS	POS	POS	POS	POS	POS	NEG	POS	NEG	NEG	POS	POS
05U00062	CC5-MRSA-II, Rhine-Hesse EMRSA/UK-EMRSA-3	MED-WARD	85	f	POS	POS	POS	POS	POS	POS	POS	POS	POS	POS	NEG	POS	NEG	NEG	POS	POS
05U03117	CC5-MRSA-II, Rhine-Hesse EMRSA/UK-EMRSA-3	MED-WARD	80	f	POS	POS	POS	POS	POS	POS	POS	POS	POS	POS	NEG	POS	NEG	NEG	POS	POS
05U10392	CC5-MRSA-II, Rhine-Hesse EMRSA/UK-EMRSA-3	MED-OPD	24	m	POS	POS	POS	POS	POS	POS	POS	POS	POS	POS	NEG	POS	NEG	NEG	POS	POS
05V03312	CC5-MRSA-II, Rhine-Hesse EMRSA/UK-EMRSA-3	MED-ICU	81	m	POS	POS	POS	POS	POS	POS	POS	POS	POS	POS	NEG	POS	NEG	NEG	POS	POS
05V08095	CC5-MRSA-II, Rhine-Hesse EMRSA/UK-EMRSA-3	MED-WARD	57	m	POS	POS	POS	POS	POS	POS	POS	POS	POS	POS	NEG	POS	NEG	NEG	POS	POS
05V09015	CC5-MRSA-II, Rhine-Hesse EMRSA/UK-EMRSA-3	MED-WARD	64	m	POS	POS	POS	POS	POS	POS	POS	POS	POS	POS	NEG	POS	NEG	NEG	POS	POS
05V09326	CC5-MRSA-II, Rhine-Hesse EMRSA/UK-EMRSA-3	MED-WARD	77	m	POS	POS	POS	POS	POS	POS	POS	POS	POS	POS	NEG	POS	NEG	NEG	POS	POS
05V10265	CC5-MRSA-II, Rhine-Hesse EMRSA/UK-EMRSA-3	MED-OPD	52	m	POS	POS	POS	POS	POS	POS	POS	POS	POS	POS	NEG	POS	NEG	NEG	POS	POS
05V13052	CC5-MRSA-II, Rhine-Hesse EMRSA/UK-EMRSA-3	SUR-OPD	61	m	POS	POS	POS	POS	POS	POS	POS	POS	POS	POS	NEG	POS	NEG	NEG	POS	POS
05V13470	CC5-MRSA-II, Rhine-Hesse EMRSA/UK-EMRSA-3	MED-WARD	46	f	POS	POS	POS	POS	POS	POS	POS	POS	POS	POS	NEG	POS	NEG	NEG	POS	POS
05V13891	CC5-MRSA-II, Rhine-Hesse EMRSA/UK-EMRSA-3	MED-WARD	40	m	POS	POS	POS	POS	POS	POS	POS	POS	POS	POS	NEG	POS	NEG	NEG	POS	POS
05V15616	CC5-MRSA-II, Rhine-Hesse EMRSA/UK-EMRSA-3	MED-WARD	15	f	POS	POS	POS	POS		POS	POS	POS			NEG	POS	NEG	NEG	POS	POS
05V16845	CC5-MRSA-II, Rhine-Hesse EMRSA/UK-EMRSA-3	MED-ICU	88	f	POS	POS	POS	POS	POS	POS	POS	POS	POS	POS	NEG	POS	NEG	NEG	POS	POS
05V17541	CC5-MRSA-II, Rhine-Hesse EMRSA/UK-EMRSA-3	SUR-WARD	52	m	POS	POS	POS	POS	POS	POS	POS	POS	POS	POS	NEG	POS	NEG	NEG	POS	POS
05V19105	CC5-MRSA-II, Rhine-Hesse EMRSA/UK-EMRSA-3	SUR-ICU	78	m	POS	POS	POS	POS		POS	POS	POS			NEG	POS	NEG	NEG	POS	POS
05V22148	CC5-MRSA-II, Rhine-Hesse EMRSA/UK-EMRSA-3	MED-OPD	77	m	POS	POS	POS	POS	POS	POS	POS	POS	POS	POS	NEG	POS	NEG	NEG	POS	POS
05V33740	CC5-MRSA-II, Rhine-Hesse EMRSA/UK-EMRSA-3	MED-icu	0	m	POS	POS	POS	POS	POS	POS	POS	POS	POS	POS	NEG	POS	NEG	NEG	POS	POS
05V38425	CC5-MRSA-II, Rhine-Hesse EMRSA/UK-EMRSA-3	MED-WARD	86	f	POS	POS	POS	POS	POS	POS	POS	POS	POS	POS	NEG	POS	NEG	NEG	POS	POS
05V39987	CC5-MRSA-II, Rhine-Hesse EMRSA/UK-EMRSA-3	SUR-WARD	27	m	POS	POS	POS	POS	POS	POS	POS	POS	POS	POS	NEG	POS	NEG	NEG	POS	POS
05F00089	CC5-MRSA-IV, Paediatric clone	MED-WARD	0	m	POS	POS	POS	POS	POS	POS	POS	POS	POS	POS	NEG	POS	NEG	NEG	POS	POS
05V01416	CC5-MRSA-IV, Paediatric clone	MED-WARD	29	f	POS	POS	AMB	AMB	POS	POS	AMB	POS	POS	POS	NEG	POS	NEG	NEG	POS	POS
05V16386	CC8-MRSA-IV, USA300	MED-WARD	29	m	POS	POS	POS	POS	POS	POS	POS	POS	POS	POS	POS	NEG	NEG	NEG	POS	POS
05V04920	CC8-MRSA-IV, USA500	SUR-WARD	43	m	POS	POS	POS	POS	POS	POS	POS	POS	POS	POS	POS	NEG	NEG	NEG	POS	POS
05V21159	CC8-MRSA-IV, USA500	SUR-Ward	2	f	POS	POS	POS	POS	POS	POS	POS	POS	POS	POS	POS	NEG	NEG	NEG	POS	POS
05B00867	CC22-MRSA-IV, Barnim/UK-EMRSA-15	MED-icu	70	f	POS	POS	POS	POS	POS	POS	POS	POS	POS	POS	POS	NEG	NEG	NEG	POS	POS
05V03174	CC22-MRSA-IV, Barnim/UK-EMRSA-15	MED-OPD	80	m	POS	POS	POS	POS	POS	POS	POS	POS	POS	POS	POS	NEG	NEG	AMB	POS	POS
05V07331	CC22-MRSA-IV, Barnim/UK-EMRSA-15	MED-OPD	61	m	POS	POS	POS	POS	POS	POS	POS	POS	POS	POS	POS	NEG	NEG	NEG	POS	POS
05V07371	CC22-MRSA-IV, Barnim/UK-EMRSA-15	MED-WARD	29	m	POS	POS	POS	POS	POS	POS	POS	POS	POS	POS	POS	NEG	NEG	NEG	POS	POS
05V08145	CC22-MRSA-IV, Barnim/UK-EMRSA-15	MED-OPD	61	m	POS	POS	POS	POS	POS	POS	POS	POS	POS	POS	POS	NEG	NEG	NEG	POS	POS
05V08595	CC22-MRSA-IV, Barnim/UK-EMRSA-15	MED-WARD	55	m	POS	POS	POS	POS	POS	POS	POS	POS	POS	POS	POS	NEG	NEG	NEG	POS	POS
05V12803	CC22-MRSA-IV, Barnim/UK-EMRSA-15	MED-OPD	71	m	POS	POS	POS	POS	POS	POS	POS	POS	POS	POS	POS	NEG	NEG	NEG	POS	POS
05V14596	CC22-MRSA-IV, Barnim/UK-EMRSA-15	SUR-WARD	84	f	POS	POS	POS	POS	POS	POS	POS	POS	POS	POS	POS	NEG	NEG	AMB	POS	POS
05V16691	CC22-MRSA-IV, Barnim/UK-EMRSA-15	MED-WARD	74	f	POS	POS	POS	POS		POS	POS	POS			POS	NEG	NEG	NEG	POS	POS
05V18550	CC22-MRSA-IV, Barnim/UK-EMRSA-15	MED-WARD	1	m	POS	POS	POS	POS	POS	POS	POS	POS	POS	POS	POS	NEG	NEG	AMB	POS	POS
05V19034	CC22-MRSA-IV, Barnim/UK-EMRSA-15	MED-WARD	69	m	POS	POS	POS	POS	POS	POS	POS	POS	POS	POS	POS	NEG	NEG	AMB	POS	POS
05V19333	CC22-MRSA-IV, Barnim/UK-EMRSA-15	MED-WARD	33	f	POS	POS	POS	POS		POS	POS	POS			POS	NEG	NEG	NEG	POS	POS
05V19701	CC22-MRSA-IV, Barnim/UK-EMRSA-15	MED-WARD	49	m	POS	POS	POS	POS	POS	POS	POS	POS	POS	POS	POS	NEG	NEG	NEG	POS	POS
05V20072	CC22-MRSA-IV, Barnim/UK-EMRSA-15	SUR-WARD	68	m	POS	POS	POS	POS	POS	POS	POS	POS	POS	POS	POS	NEG	NEG	NEG	POS	POS
05V24930	CC22-MRSA-IV, Barnim/UK-EMRSA-15	SUR-WARD	54	m	POS	POS	POS	POS	POS	POS	POS	POS	POS	POS	POS	NEG	NEG	AMB	POS	POS
05V31461	CC22-MRSA-IV, Barnim/UK-EMRSA-15	MED-WARD	71	m	POS	POS	POS	POS	POS	POS	POS	POS	POS	POS	POS	NEG	NEG	AMB	POS	POS
05B05671	CC45-MRSA-IV, Berlin EMRSA	SUR-WARD	64	f	POS	POS	POS	POS	POS	POS	POS	POS	POS	POS	POS	NEG	NEG	NEG	POS	POS
05V01452	CC45-MRSA-IV, Berlin EMRSA	SUR-OPD	77	m	POS	POS	POS	POS	POS	POS	POS	POS	POS	POS	POS	NEG	NEG	NEG	POS	POS
05V01617	CC45-MRSA-IV, Berlin EMRSA	MED-OPD	60	m	POS	POS	POS	POS	POS	POS	POS	POS	POS	POS	POS	NEG	NEG	NEG	POS	POS
05V16986	CC45-MRSA-IV, Berlin EMRSA	MED-WARD	71	f	POS	POS	POS	POS	POS	NEG	AMB	POS	POS	POS	NEG	NEG	NEG	NEG	POS	POS
05V18357	CC45-MRSA-IV, Berlin EMRSA	SUR-WARD	44	f	POS	POS	POS	POS	POS	POS	POS	POS	POS	POS	POS	NEG	NEG	NEG	POS	POS
05V23111	CC45-MRSA-IV, Berlin EMRSA	MED-OPD	56	m	POS	POS	POS	POS	POS	POS	POS	POS	POS	POS	POS	NEG	NEG	NEG	POS	POS
05V23414	CC45-MRSA-IV, Berlin EMRSA	MED-WARD	61	m	POS	POS	POS	POS		POS	POS	POS			POS	NEG	NEG	NEG	POS	POS
05V23858	CC45-MRSA-IV, Berlin EMRSA	MED-ICU	70	m	POS	POS	POS	POS	POS	POS	POS	POS	POS	POS	POS	NEG	NEG	NEG	POS	POS
05V26241	CC45-MRSA-IV, Berlin EMRSA	MED-WARD	71	m	POS	POS	POS	POS	POS	POS	POS	POS	POS	POS	POS	NEG	NEG	NEG	POS	POS
05V28399	CC45-MRSA-IV, Berlin EMRSA	SUR-OPD	82	m	POS	POS	POS	POS	POS	POS	POS	POS	POS	POS	POS	NEG	NEG	NEG	POS	POS
05V32647	CC45-MRSA-IV, Berlin EMRSA	MED-OPD	70	m	POS	POS	POS	POS		NEG	POS	POS			POS	NEG	NEG	NEG	POS	POS
05V37969	CC45-MRSA-IV, Berlin EMRSA	MED-WARD	63	f	POS	POS	POS	POS	POS	POS	POS	POS	POS	POS	POS	NEG	NEG	NEG	POS	POS
05U10018	CC398-MRSA-V	MED-WARD	0	m	POS	POS	POS	POS	POS	POS	POS	POS	POS	POS	POS	NEG	NEG	NEG	POS	POS
**2006**																				
06V36353	CC1-MRSA-IV, WA MRSA-1/57	MED-OPD	92	f	POS	POS	POS	POS	POS	POS	POS	POS	POS	POS	NEG	NEG	POS	NEG	POS	POS
06V40202	CC5/ST228-MRSA-I, South German EMRSA	SUR-ICU	67	f	POS	POS	POS	POS		POS	POS	POS			NEG	POS	NEG	NEG	POS	POS
06V41266	CC5/ST228-MRSA-I, South German EMRSA	SUR-ICU	59	f	POS	POS	POS	POS	POS	POS	POS	POS	POS	POS	NEG	POS	NEG	NEG	POS	POS
06B09097	CC5-MRSA-II, Rhine-Hesse EMRSA/UK-EMRSA-3	SUR-ICU	76	m	POS	POS	POS	POS		POS	POS				NEG	POS	NEG	NEG	POS	POS
06F00244	CC5-MRSA-II, Rhine-Hesse EMRSA/UK-EMRSA-3	MED-WARD	78	f	POS	POS	POS	POS	POS	POS	POS	POS	POS	POS	NEG	POS	NEG	NEG	POS	POS
06V03913	CC5-MRSA-II, Rhine-Hesse EMRSA/UK-EMRSA-3	SUR-WARD	19	m	POS	POS	POS	POS	POS	POS	POS	POS	POS	POS	NEG	POS	NEG	NEG	POS	POS
06V04337	CC5-MRSA-II, Rhine-Hesse EMRSA/UK-EMRSA-3	MED-WARD	50	f	POS	POS	POS	POS	POS	POS	POS	POS	POS	POS	NEG	POS	NEG	NEG	POS	POS
06V06785	CC5-MRSA-II, Rhine-Hesse EMRSA/UK-EMRSA-3	MED-WARD	4	f	POS	POS	POS	POS	POS	POS	POS	POS	POS	POS	NEG	POS	NEG	NEG	POS	POS
06V12258	CC5-MRSA-II, Rhine-Hesse EMRSA/UK-EMRSA-3	SUR-WARD	38	m	POS	POS	POS	POS	POS	POS	POS	POS	POS	POS	NEG	POS	NEG	NEG	POS	POS
06V20553	CC5-MRSA-II, Rhine-Hesse EMRSA/UK-EMRSA-3	MED-icu	0	m	POS	POS	POS	POS	POS	POS	POS	POS	POS	POS	NEG	POS	NEG	NEG	POS	POS
06V22564	CC5-MRSA-II, Rhine-Hesse EMRSA/UK-EMRSA-3	MED-WARD	22	f	POS	POS	POS	POS		POS	POS	POS			NEG	POS	NEG	NEG	POS	POS
06V23750	CC5-MRSA-II, Rhine-Hesse EMRSA/UK-EMRSA-3	MED-WARD	78	m	POS	POS	POS	POS	POS	POS	POS	POS	POS	POS	NEG	POS	NEG	NEG	POS	POS
06V27332	CC5-MRSA-II, Rhine-Hesse EMRSA/UK-EMRSA-3	MED-WARD	79	f	POS	POS	POS	POS	POS	POS	POS	POS	POS	POS	NEG	POS	NEG	NEG	POS	POS
06V29484	CC5-MRSA-II, Rhine-Hesse EMRSA/UK-EMRSA-3	SUR-ICU	72	m	POS	POS	POS	POS	POS	POS	POS	POS	POS	POS	NEG	POS	NEG	NEG	POS	POS
06V31278	CC5-MRSA-II, Rhine-Hesse EMRSA/UK-EMRSA-3	SUR-ICU	55	m	POS	POS	POS	POS		POS	POS	POS			NEG	POS	NEG	NEG	POS	POS
06V36043	CC5-MRSA-II, Rhine-Hesse EMRSA/UK-EMRSA-3	SUR-ICU	49	m	POS	POS	POS	POS	POS	POS	POS	POS	POS	POS	NEG	POS	NEG	NEG	POS	POS
06V36349	CC5-MRSA-II, Rhine-Hesse EMRSA/UK-EMRSA-3	MED-WARD	53	m	POS	POS	POS	POS	POS	POS	POS	POS	POS	POS	NEG	POS	NEG	NEG	POS	POS
06V39695	CC5-MRSA-II, Rhine-Hesse EMRSA/UK-EMRSA-3	MED-OPD	43	f	POS	POS	POS	POS	POS	POS	POS	POS	POS	POS	NEG	POS	NEG	NEG	POS	POS
06V40571	CC5-MRSA-II, Rhine-Hesse EMRSA/UK-EMRSA-3	SUR-OPD	35	m	POS	POS	POS	POS		POS	POS	POS			NEG	POS	NEG	NEG	POS	POS
06V42188	CC5-MRSA-II, Rhine-Hesse EMRSA/UK-EMRSA-3	MED-WARD	66	m	POS	POS	POS	POS	POS	POS	POS	POS	POS	POS	NEG	POS	NEG	NEG	POS	POS
06V42146	CC8-MRSA-IV, Lyon Clone/UK-EMRSA-2	MED-OPD	43	f	POS	POS	POS	POS		POS	POS	POS			POS	NEG	NEG	NEG	POS	POS
06V28560	CC8-MRSA-V	MED-WARD	54	m	POS	POS	POS	POS	POS	POS	POS	POS	POS	POS	POS	NEG	NEG	NEG	POS	POS
06F00837	CC22-MRSA-IV, Barnim/UK-EMRSA-15	MED-WARD	83	f	POS	POS	POS	POS	POS	POS	POS	POS	POS	POS	POS	NEG	NEG	NEG	POS	POS
06F01638	CC22-MRSA-IV, Barnim/UK-EMRSA-15	MED-WARD	73	f	POS	POS	POS	POS	POS	POS	POS	POS	POS	POS	POS	NEG	NEG	AMB	POS	POS
06F04643	CC22-MRSA-IV, Barnim/UK-EMRSA-15	MED-WARD	70	m	POS	POS	POS	POS		POS	POS				AMB	NEG	NEG	NEG	POS	POS
06F05580	CC22-MRSA-IV, Barnim/UK-EMRSA-15	MED-WARD	81	f	POS	POS	POS	POS	POS	POS	POS	POS	POS	POS	POS	NEG	NEG	NEG	POS	POS
06U05469	CC22-MRSA-IV, Barnim/UK-EMRSA-15	MED-WARD	83	f	POS	POS	POS	POS	POS	POS	POS	POS	POS	POS	POS	NEG	NEG	AMB	POS	POS
06U17314	CC22-MRSA-IV, Barnim/UK-EMRSA-15	MED-ICU	53	f	POS	POS	POS	POS	POS	POS	POS	POS	POS	POS	POS	NEG	NEG	NEG	POS	POS
06V08377	CC22-MRSA-IV, Barnim/UK-EMRSA-15	MED-OPD	62	m	POS	POS	POS	POS	POS	POS	POS	POS	POS	POS	POS	NEG	NEG	AMB	POS	POS
06V08474	CC22-MRSA-IV, Barnim/UK-EMRSA-15	SUR-ICU	70	m	POS	POS	POS	POS	POS	POS	POS	POS	POS	POS	POS	NEG	NEG	NEG	POS	POS
06V09374	CC22-MRSA-IV, Barnim/UK-EMRSA-15	SUR-WARD	71	f	POS	POS	POS	POS	POS	POS	POS	POS	POS	POS	POS	NEG	NEG	AMB	POS	POS
06V09605	CC22-MRSA-IV, Barnim/UK-EMRSA-15	SUR-WARD	61	f	POS	POS	POS	POS	POS	POS	POS	POS	POS	POS	POS	NEG	NEG	NEG	POS	POS
06V18687	CC22-MRSA-IV, Barnim/UK-EMRSA-15	SUR-WARD	87	f	POS	POS	POS	POS	POS	POS	POS	POS	POS	POS	POS	NEG	NEG	NEG	POS	POS
06V21697	CC22-MRSA-IV, Barnim/UK-EMRSA-15	MED-OPD	77	m	POS	POS	POS	POS	POS	POS	POS	POS	POS	POS	POS	NEG	NEG	AMB	POS	POS
06V23749	CC22-MRSA-IV, Barnim/UK-EMRSA-15	SUR-WARD	72	m	POS	POS	POS	POS	POS	POS	POS	POS	POS	POS	POS	NEG	NEG	NEG	POS	POS
06V29915	CC22-MRSA-IV, Barnim/UK-EMRSA-15	MED-WARD	56	m	POS	POS	POS	POS	POS	POS	POS	POS	POS	POS	POS	NEG	NEG	AMB	POS	POS
06V31778	CC22-MRSA-IV, Barnim/UK-EMRSA-15	MED-OPD	75	f	POS	POS	POS	POS	POS	POS	POS	POS	POS	POS	POS	NEG	NEG	AMB	POS	POS
06V39089	CC22-MRSA-IV, Barnim/UK-EMRSA-15	MED-WARD	78	m	POS	POS	POS	POS	POS	POS	POS	POS	POS	POS	POS	NEG	NEG	NEG	POS	POS
06V41512	CC22-MRSA-IV, Barnim/UK-EMRSA-15	SUR-WARD	93	f	POS	POS	POS	POS		POS	POS	POS			POS	NEG	NEG	NEG	POS	POS
06V42186	CC22-MRSA-IV, Barnim/UK-EMRSA-15	MED-OPD	85	m	POS	POS	POS	POS	POS	POS	POS	POS	POS	POS	POS	NEG	NEG	NEG	POS	POS
06V42236	CC22-MRSA-IV, Barnim/UK-EMRSA-15	MED-WARD	77	m	POS	POS	POS	POS	POS	POS	POS	POS	POS	POS	POS	NEG	NEG	NEG	POS	POS
06V43697	CC22-MRSA-IV, Barnim/UK-EMRSA-15	MED-OPD	62	m	POS	POS	POS	POS	POS	POS	POS	POS	POS	POS	POS	NEG	NEG	AMB	POS	POS
06V44153	CC22-MRSA-IV, Barnim/UK-EMRSA-15	MED-OPD	70	m	POS	POS	POS	POS	POS	POS	POS	POS	POS	POS	POS	NEG	NEG	NEG	POS	POS
06V45679	CC22-MRSA-IV, Barnim/UK-EMRSA-15	MED-OPD	47	m	POS	POS	POS	POS	POS	POS	POS	POS	POS	POS	POS	NEG	NEG	NEG	POS	POS
06V45831	CC22-MRSA-IV, Barnim/UK-EMRSA-15	SUR-WARD	73	f	POS	POS	POS	POS	POS	POS	POS	POS	POS	POS	POS	NEG	NEG	NEG	POS	POS
06V46314	CC22-MRSA-IV, Barnim/UK-EMRSA-15	SUR-ICU	50	f	POS	POS	POS	POS	POS	POS	POS	POS	POS	POS	POS	NEG	NEG	NEG	POS	POS
06V34729	CC30-MRSA-IV [PVL+], Southwest Pacific Clone	MED-OPD	28	f	POS	POS	POS	POS	POS	POS	POS	POS	POS	POS	NEG	NEG	POS	NEG	POS	POS
06B09900	CC45-MRSA-IV, Berlin EMRSA	MED-WARD	45	m	POS	POS	POS	POS	POS	POS	POS	POS	POS	POS	POS	NEG	NEG	NEG	POS	POS
06V00922	CC45-MRSA-IV, Berlin EMRSA	SUR-WARD	76	m	POS	POS	POS	POS	POS	POS	POS	POS	POS	POS	POS	NEG	NEG	NEG	POS	POS
06V09551	CC45-MRSA-IV, Berlin EMRSA	MED-WARD	45	m	POS	POS	POS	POS	POS	POS	POS	POS	POS	POS	POS	NEG	NEG	NEG	POS	POS
06V10719	CC45-MRSA-IV, Berlin EMRSA	MED-WARD	83	m	POS	POS	POS	POS	POS	POS	POS	POS	POS	POS	POS	NEG	NEG	NEG	POS	POS
06V11090	CC45-MRSA-IV, Berlin EMRSA	MED-WARD	57	m	POS	POS	POS	POS	POS	AMB	POS	POS	POS	POS	AMB	NEG	NEG	NEG	POS	POS
06V14383	CC45-MRSA-IV, Berlin EMRSA	SUR-ICU	80	m	POS	POS	POS	POS	POS	POS	POS	POS	POS	POS	AMB	NEG	NEG	NEG	POS	POS
06V21475	CC45-MRSA-IV, Berlin EMRSA	MED-ICU	73	m	POS	POS	POS	POS	POS	AMB	POS	POS	POS	POS	AMB	NEG	NEG	NEG	POS	POS
06V24231	CC45-MRSA-IV, Berlin EMRSA	MED-OPD	87	m	POS	POS	POS	POS	POS	AMB	POS	POS	POS	POS	POS	NEG	NEG	NEG	POS	POS
06V32992	CC45-MRSA-IV, Berlin EMRSA	MED-ICU	74	f	POS	POS	POS	POS	POS	AMB	POS	POS	POS	POS	AMB	NEG	NEG	NEG	POS	POS
06V38753	CC45-MRSA-IV, Berlin EMRSA	SUR-ICU	76	m	POS	POS	POS	POS	POS	POS	POS	POS	POS	POS	POS	NEG	NEG	NEG	POS	POS
06V39848	CC45-MRSA-IV, Berlin EMRSA	MED-OPD	64	m	POS	POS	POS	POS		AMB	POS	POS			POS	NEG	NEG	NEG	POS	POS
06V42259	CC45-MRSA-IV, Berlin EMRSA	MED-WARD	66	m	POS	POS	POS	POS	POS	POS	POS	POS	POS	POS	POS	NEG	NEG	NEG	POS	POS
06V33960	CC80-MRSA-IV [PVL+], European caMRSA Clone	MED-OPD	5	m	POS	POS	POS	POS		POS	POS	POS			NEG	NEG	POS	NEG	POS	POS

ISOLATE:	METHICILLIN RESISTANCE AND SCCmec TYPING																								
	truncated signal transducer protein MecR1	glycerophosphoryl diester phosphodi-esterase, associated with mecA	cassette chromosome recombinase genes A/B-1		plasmin-sensitive surface protein	hypothetical protein from SCCmec elements	cassette chromosome recombinase genes A/B-2		potassium-trans-locating ATPase A, chain 2	potassium-transporting ATPase B, chain 1	potassium-trans-locating ATPase C, chain 2	sensor kinase protein	KDP operon transcrip-tional regulatory protein	methicillin-resistance regulatory protein	signal transducer protein MecR1	homolog of xylose repressor, associated with SCCmec-elements	cassette chromosome recombinase genes A/B-3		mercury resistance operon		cassette chromosome recombinase genes "ccrAA" (hypothetical) and ccrC			cassette chromosome recombinase genes A/B-4	
	delta_mecR	ugpQ	ccrA-1	ccrB-1	plsSCC (COL)	Q9XB68-dcs	ccrA-2	ccrB-2	kdpA-SCC	kdpB-SCC	kdpC-SCC	kdpD-SCC	kdpE-SCC	mecI	mecR	xylR	ccrA-3	ccrB-3	merA	merB	ccrAA (MRSAZH47) _probe 1	ccrAA (MRSAZH47) _probe 2	ccrC (85-2082)	ccrA-4	ccrB-4
**2005**																									
05B01179	POS	POS	POS	POS	POS	NEG	NEG	NEG	NEG	NEG	NEG	NEG	NEG	NEG	NEG	NEG	NEG	NEG	POS	POS	NEG	NEG	NEG	NEG	NEG
05B02687	POS	POS	POS	POS	POS	NEG	NEG	NEG	NEG	NEG	NEG	NEG	NEG	NEG	NEG	NEG	NEG	NEG	POS	POS	NEG	NEG	NEG	NEG	NEG
05V08483	POS	POS	POS	POS	POS	NEG	NEG	NEG	NEG	NEG	NEG	NEG	NEG	NEG	NEG	NEG	NEG	NEG	POS	POS	NEG	NEG	NEG	NEG	NEG
05V19001	POS	POS	POS	POS	POS	NEG	NEG	NEG	NEG	NEG	NEG	NEG	NEG	NEG	NEG	NEG	NEG	NEG	NEG	NEG	NEG	NEG	NEG	NEG	NEG
05B04626	POS	POS	NEG	NEG	NEG	NEG	POS	POS	POS	POS	POS	POS	POS	POS	POS	POS	NEG	NEG	NEG	NEG	NEG	NEG	NEG	NEG	NEG
05B07903	POS	POS	NEG	NEG	NEG	POS	POS	POS	POS	POS	POS	POS	POS	POS	POS	POS	NEG	NEG	NEG	NEG	NEG	NEG	NEG	NEG	NEG
05U00062	POS	POS	NEG	NEG	NEG	POS	POS	POS	POS	POS	POS	POS	POS	POS	POS	POS	NEG	NEG	NEG	NEG	NEG	NEG	NEG	NEG	NEG
05U03117	POS	POS	NEG	NEG	NEG	AMB	POS	POS	POS	AMB	POS	POS	POS	POS	POS	POS	NEG	NEG	NEG	NEG	NEG	NEG	NEG	NEG	NEG
05U10392	POS	POS	NEG	NEG	NEG	AMB	POS	POS	POS	POS	POS	POS	POS	POS	POS	POS	NEG	NEG	NEG	NEG	NEG	NEG	NEG	NEG	NEG
05V03312	POS	POS	NEG	NEG	NEG	POS	POS	POS	POS	POS	POS	POS	POS	POS	POS	POS	NEG	NEG	NEG	NEG	NEG	NEG	NEG	NEG	NEG
05V08095	POS	POS	NEG	NEG	NEG	POS	POS	POS	POS	POS	POS	POS	POS	POS	POS	POS	NEG	NEG	NEG	NEG	NEG	NEG	NEG	NEG	NEG
05V09015	POS	POS	NEG	NEG	NEG	POS	POS	POS	POS	POS	POS	POS	POS	POS	POS	POS	NEG	NEG	NEG	NEG	NEG	NEG	NEG	NEG	NEG
05V09326	POS	POS	NEG	NEG	NEG	POS	POS	POS	POS	POS	POS	POS	POS	POS	POS	POS	NEG	NEG	NEG	NEG	NEG	NEG	NEG	NEG	NEG
05V10265	POS	POS	NEG	NEG	NEG	AMB	POS	POS	POS	POS	POS	POS	POS	POS	POS	POS	NEG	NEG	NEG	NEG	NEG	NEG	NEG	NEG	NEG
05V13052	POS	POS	NEG	NEG	NEG	POS	POS	POS	POS	POS	POS	POS	POS	POS	POS	POS	NEG	NEG	NEG	NEG	NEG	NEG	NEG	NEG	NEG
05V13470	POS	POS	NEG	NEG	NEG	POS	POS	POS	POS	POS	POS	POS	POS	POS	POS	POS	NEG	NEG	NEG	NEG	NEG	NEG	NEG	NEG	NEG
05V13891	POS	POS	NEG	NEG	NEG	#ZAHL!	POS	POS	POS	AMB	POS	POS	POS	POS	POS	POS	NEG	NEG	NEG	NEG	NEG	NEG	NEG	NEG	NEG
05V15616																									
05V16845	POS	POS	NEG	NEG	NEG	POS	POS	POS	POS	POS	POS	POS	POS	POS	POS	POS	NEG	NEG	NEG	NEG	NEG	NEG	NEG	NEG	NEG
05V17541	POS	POS	NEG	NEG	NEG	AMB	POS	POS	POS	AMB	POS	POS	POS	POS	POS	POS	NEG	NEG	NEG	NEG	NEG	NEG	NEG	NEG	NEG
05V19105																									
05V22148	POS	POS	NEG	NEG	NEG	POS	POS	POS	POS	POS	POS	POS	POS	POS	POS	POS	NEG	NEG	NEG	NEG	NEG	NEG	NEG	NEG	NEG
05V33740	POS	POS	NEG	NEG	NEG	NEG	POS	POS	POS	POS	POS	POS	POS	POS	POS	POS	NEG	NEG	NEG	NEG	NEG	NEG	NEG	NEG	NEG
05V38425	POS	POS	NEG	NEG	NEG	NEG	POS	POS	POS	POS	POS	POS	POS	POS	POS	POS	NEG	NEG	NEG	NEG	NEG	NEG	NEG	NEG	NEG
05V39987	POS	POS	NEG	NEG	NEG	POS	POS	POS	POS	POS	POS	POS	POS	POS	POS	POS	NEG	NEG	NEG	NEG	NEG	NEG	NEG	NEG	NEG
05F00089	POS	POS	NEG	NEG	NEG	NEG	POS	POS	NEG	NEG	NEG	NEG	NEG	NEG	NEG	NEG	NEG	NEG	NEG	NEG	NEG	NEG	NEG	NEG	NEG
05V01416	POS	POS	NEG	NEG	NEG	NEG	POS	POS	NEG	NEG	NEG	NEG	NEG	NEG	NEG	NEG	NEG	NEG	NEG	NEG	NEG	NEG	NEG	NEG	NEG
05V16386	POS	POS	NEG	NEG	NEG	NEG	POS	POS	NEG	NEG	NEG	NEG	NEG	NEG	NEG	NEG	NEG	NEG	NEG	NEG	NEG	NEG	NEG	NEG	NEG
05V04920	POS	POS	NEG	NEG	NEG	NEG	POS	POS	NEG	NEG	NEG	NEG	NEG	NEG	NEG	NEG	NEG	NEG	NEG	NEG	NEG	NEG	NEG	NEG	NEG
05V21159	POS	POS	NEG	NEG	NEG	NEG	POS	POS	NEG	NEG	NEG	NEG	NEG	NEG	NEG	NEG	NEG	NEG	NEG	NEG	NEG	NEG	NEG	NEG	NEG
05B00867	POS	POS	NEG	NEG	NEG	POS	POS	POS	NEG	NEG	NEG	NEG	NEG	NEG	NEG	NEG	NEG	AMB	NEG	NEG	NEG	NEG	NEG	NEG	NEG
05V03174	POS	POS	NEG	NEG	NEG	POS	POS	POS	NEG	NEG	NEG	NEG	NEG	NEG	NEG	NEG	NEG	NEG	NEG	NEG	NEG	NEG	NEG	NEG	NEG
05V07331	POS	POS	NEG	NEG	NEG	POS	POS	POS	NEG	NEG	NEG	NEG	NEG	NEG	NEG	NEG	NEG	NEG	NEG	NEG	NEG	NEG	NEG	NEG	NEG
05V07371	POS	POS	NEG	NEG	NEG	POS	POS	POS	NEG	NEG	NEG	NEG	NEG	NEG	NEG	NEG	NEG	NEG	NEG	NEG	NEG	NEG	NEG	NEG	NEG
05V08145	POS	POS	NEG	NEG	NEG	POS	POS	POS	NEG	NEG	NEG	NEG	NEG	NEG	NEG	NEG	NEG	NEG	NEG	NEG	NEG	NEG	NEG	NEG	NEG
05V08595	POS	POS	NEG	NEG	NEG	POS	POS	POS	NEG	NEG	NEG	NEG	NEG	NEG	NEG	NEG	NEG	NEG	NEG	NEG	NEG	NEG	NEG	NEG	NEG
05V12803	POS	POS	NEG	NEG	NEG	POS	POS	POS	NEG	NEG	NEG	NEG	NEG	NEG	NEG	NEG	NEG	NEG	NEG	NEG	NEG	NEG	NEG	NEG	NEG
05V14596	POS	POS	NEG	NEG	NEG	POS	POS	POS	NEG	NEG	NEG	NEG	NEG	NEG	NEG	NEG	NEG	NEG	NEG	NEG	NEG	NEG	NEG	NEG	NEG
05V16691																									
05V18550	POS	POS	NEG	NEG	NEG	POS	POS	POS	NEG	NEG	NEG	NEG	NEG	NEG	NEG	NEG	NEG	NEG	NEG	NEG	NEG	NEG	NEG	NEG	NEG
05V19034	POS	POS	NEG	NEG	NEG	POS	POS	POS	NEG	NEG	NEG	NEG	NEG	NEG	NEG	NEG	NEG	NEG	NEG	NEG	NEG	NEG	NEG	NEG	NEG
05V19333																									
05V19701	POS	POS	NEG	NEG	NEG	POS	POS	POS	NEG	NEG	NEG	NEG	NEG	NEG	NEG	NEG	NEG	NEG	NEG	NEG	NEG	NEG	NEG	NEG	NEG
05V20072	POS	POS	NEG	NEG	NEG	POS	POS	POS	NEG	NEG	NEG	NEG	NEG	NEG	NEG	NEG	NEG	NEG	NEG	NEG	NEG	NEG	NEG	NEG	NEG
05V24930	POS	POS	NEG	NEG	NEG	POS	POS	POS	NEG	NEG	NEG	NEG	NEG	NEG	NEG	NEG	NEG	NEG	NEG	NEG	NEG	NEG	NEG	NEG	NEG
05V31461	POS	POS	NEG	NEG	NEG	POS	POS	POS	NEG	NEG	NEG	NEG	NEG	NEG	NEG	NEG	NEG	NEG	NEG	NEG	NEG	NEG	NEG	NEG	NEG
05B05671	POS	POS	NEG	NEG	NEG	POS	POS	POS	NEG	NEG	NEG	NEG	NEG	NEG	NEG	NEG	NEG	NEG	NEG	NEG	NEG	NEG	NEG	NEG	NEG
05V01452	POS	POS	NEG	NEG	NEG	POS	POS	POS	NEG	NEG	NEG	NEG	NEG	NEG	NEG	NEG	NEG	NEG	NEG	NEG	AMB	NEG	NEG	NEG	NEG
05V01617	POS	POS	NEG	NEG	NEG	POS	POS	POS	NEG	NEG	NEG	NEG	NEG	NEG	NEG	NEG	NEG	NEG	NEG	NEG	NEG	NEG	NEG	NEG	NEG
05V16986	POS	POS	NEG	NEG	NEG	AMB	POS	POS	NEG	NEG	NEG	NEG	NEG	NEG	NEG	NEG	NEG	NEG	NEG	NEG	NEG	NEG	NEG	NEG	NEG
05V18357	POS	POS	NEG	NEG	NEG	NEG	POS	POS	NEG	NEG	NEG	NEG	NEG	NEG	NEG	NEG	NEG	NEG	NEG	NEG	NEG	NEG	NEG	NEG	NEG
05V23111	POS	POS	NEG	NEG	NEG	POS	POS	POS	NEG	NEG	NEG	NEG	NEG	NEG	NEG	NEG	NEG	NEG	NEG	NEG	NEG	NEG	NEG	NEG	NEG
05V23414																									
05V23858	POS	POS	NEG	NEG	NEG	POS	POS	POS	NEG	NEG	NEG	NEG	NEG	NEG	NEG	NEG	NEG	NEG	NEG	NEG	NEG	NEG	NEG	NEG	NEG
05V26241	POS	POS	NEG	NEG	NEG	POS	POS	POS	NEG	NEG	NEG	NEG	NEG	NEG	NEG	NEG	NEG	NEG	NEG	NEG	NEG	NEG	NEG	NEG	NEG
05V28399	POS	POS	NEG	NEG	NEG	POS	POS	POS	NEG	NEG	NEG	NEG	NEG	NEG	NEG	NEG	NEG	NEG	NEG	NEG	NEG	NEG	NEG	NEG	NEG
05V32647																									
05V37969	POS	POS	NEG	NEG	NEG	POS	POS	POS	NEG	NEG	NEG	NEG	NEG	NEG	NEG	NEG	NEG	NEG	NEG	NEG	NEG	NEG	NEG	NEG	NEG
05U10018	NEG	POS	NEG	NEG	NEG	NEG	NEG	NEG	NEG	NEG	NEG	NEG	NEG	NEG	NEG	NEG	NEG	NEG	NEG	NEG	POS	POS	POS	NEG	NEG
**2006**																									
06V36353	POS	POS	NEG	NEG	NEG	NEG	POS	POS	NEG	NEG	NEG	NEG	NEG	NEG	NEG	NEG	NEG	NEG	NEG	NEG	NEG	NEG	NEG	NEG	NEG
06V40202																									
06V41266	POS	POS	POS	POS	POS	POS	NEG	NEG	NEG	NEG	NEG	NEG	NEG	NEG	NEG	NEG	NEG	NEG	POS	POS	NEG	NEG	NEG	NEG	NEG
06B09097																									
06F00244	POS	POS	NEG	NEG	NEG	POS	POS	POS	POS	POS	POS	POS	POS	POS	POS	POS	NEG	NEG	NEG	NEG	NEG	NEG	NEG	NEG	NEG
06V03913	POS	POS	NEG	NEG	NEG	POS	POS	POS	POS	POS	POS	POS	POS	POS	POS	POS	NEG	NEG	NEG	NEG	NEG	NEG	NEG	NEG	NEG
06V04337	POS	POS	NEG	NEG	NEG	POS	POS	POS	POS	POS	POS	POS	POS	POS	POS	POS	NEG	NEG	NEG	NEG	NEG	NEG	NEG	NEG	NEG
06V06785	POS	POS	NEG	NEG	NEG	POS	POS	POS	POS	POS	POS	POS	POS	POS	POS	POS	NEG	NEG	NEG	NEG	NEG	NEG	NEG	NEG	NEG
06V12258	POS	POS	NEG	NEG	NEG	POS	POS	POS	POS	POS	POS	POS	POS	POS	POS	POS	NEG	NEG	NEG	NEG	NEG	NEG	NEG	NEG	NEG
06V20553	POS	POS	NEG	NEG	NEG	POS	POS	POS	POS	POS	POS	POS	POS	POS	POS	POS	NEG	NEG	NEG	NEG	NEG	NEG	NEG	NEG	NEG
06V22564																									
06V23750	POS	POS	NEG	NEG	NEG	POS	POS	POS	POS	POS	POS	POS	POS	POS	POS	POS	NEG	NEG	NEG	NEG	NEG	NEG	NEG	NEG	NEG
06V27332	POS	POS	NEG	NEG	NEG	POS	POS	POS	POS	POS	POS	POS	POS	POS	POS	POS	NEG	NEG	NEG	NEG	NEG	NEG	NEG	NEG	NEG
06V29484	POS	POS	NEG	NEG	NEG	POS	POS	POS	POS	POS	POS	POS	POS	POS	POS	POS	NEG	NEG	NEG	NEG	NEG	NEG	NEG	NEG	NEG
06V31278																									
06V36043	POS	POS	NEG	NEG	NEG	NEG	POS	POS	POS	POS	POS	POS	POS	POS	POS	POS	NEG	NEG	NEG	NEG	NEG	NEG	NEG	NEG	NEG
06V36349	POS	POS	NEG	NEG	NEG	POS	POS	POS	POS	POS	POS	POS	POS	POS	POS	POS	NEG	NEG	NEG	NEG	NEG	NEG	NEG	NEG	NEG
06V39695	POS	POS	NEG	NEG	NEG	AMB	POS	POS	POS	POS	POS	POS	POS	POS	POS	POS	NEG	NEG	NEG	NEG	NEG	NEG	NEG	NEG	NEG
06V40571																									
06V42188	POS	POS	NEG	NEG	NEG	POS	POS	POS	POS	POS	POS	POS	POS	POS	POS	POS	NEG	NEG	NEG	NEG	NEG	NEG	NEG	NEG	NEG
06V42146																									
06V28560	NEG	POS	NEG	NEG	NEG	NEG	NEG	NEG	NEG	NEG	NEG	NEG	NEG	NEG	NEG	NEG	NEG	NEG	NEG	NEG	POS	POS	POS	NEG	NEG
06F00837	POS	POS	NEG	NEG	NEG	POS	POS	POS	NEG	NEG	NEG	NEG	NEG	NEG	NEG	NEG	NEG	NEG	NEG	NEG	NEG	NEG	NEG	NEG	NEG
06F01638	POS	POS	NEG	NEG	NEG	POS	POS	POS	NEG	NEG	NEG	NEG	NEG	NEG	NEG	NEG	NEG	NEG	NEG	NEG	NEG	NEG	NEG	NEG	NEG
06F04643																									
06F05580	POS	POS	NEG	NEG	NEG	POS	POS	POS	NEG	NEG	NEG	NEG	NEG	NEG	NEG	NEG	NEG	NEG	NEG	NEG	NEG	NEG	NEG	NEG	NEG
06U05469	POS	POS	NEG	NEG	NEG	POS	POS	POS	NEG	NEG	NEG	NEG	NEG	NEG	NEG	NEG	NEG	NEG	NEG	NEG	NEG	NEG	NEG	NEG	NEG
06U17314	POS	POS	NEG	NEG	NEG	POS	POS	POS	NEG	NEG	NEG	NEG	NEG	NEG	NEG	NEG	NEG	NEG	NEG	NEG	NEG	NEG	NEG	NEG	NEG
06V08377	POS	POS	NEG	NEG	NEG	POS	POS	POS	NEG	NEG	NEG	NEG	NEG	NEG	NEG	NEG	NEG	NEG	NEG	NEG	NEG	NEG	NEG	NEG	NEG
06V08474	POS	POS	NEG	NEG	NEG	POS	POS	POS	NEG	NEG	NEG	NEG	NEG	NEG	NEG	NEG	NEG	NEG	NEG	NEG	NEG	NEG	NEG	NEG	NEG
06V09374	POS	POS	NEG	NEG	NEG	POS	POS	POS	NEG	NEG	NEG	NEG	NEG	NEG	NEG	NEG	NEG	NEG	NEG	NEG	NEG	NEG	NEG	NEG	NEG
06V09605	POS	POS	NEG	NEG	NEG	POS	POS	POS	NEG	NEG	NEG	NEG	NEG	NEG	NEG	NEG	NEG	NEG	NEG	NEG	NEG	NEG	NEG	NEG	NEG
06V18687	POS	POS	NEG	NEG	NEG	POS	POS	POS	NEG	NEG	NEG	NEG	NEG	NEG	NEG	NEG	NEG	NEG	NEG	NEG	NEG	NEG	NEG	NEG	NEG
06V21697	POS	POS	NEG	NEG	NEG	POS	POS	POS	NEG	NEG	NEG	NEG	NEG	NEG	NEG	NEG	NEG	NEG	NEG	NEG	NEG	NEG	NEG	NEG	NEG
06V23749	POS	POS	NEG	NEG	NEG	POS	POS	POS	NEG	NEG	NEG	NEG	NEG	NEG	NEG	NEG	NEG	NEG	NEG	NEG	NEG	NEG	NEG	NEG	NEG
06V29915	POS	POS	NEG	NEG	NEG	POS	POS	POS	NEG	NEG	NEG	NEG	NEG	NEG	NEG	NEG	NEG	NEG	NEG	NEG	NEG	NEG	NEG	NEG	NEG
06V31778	POS	POS	NEG	NEG	NEG	POS	POS	POS	NEG	NEG	NEG	AMB	NEG	NEG	NEG	AMB	NEG	NEG	NEG	NEG	NEG	NEG	NEG	NEG	NEG
06V39089	POS	POS	NEG	NEG	NEG	POS	POS	POS	NEG	NEG	NEG	NEG	NEG	NEG	NEG	NEG	NEG	NEG	NEG	NEG	NEG	NEG	NEG	NEG	NEG
06V41512																									
06V42186	POS	POS	NEG	NEG	NEG	POS	POS	POS	NEG	NEG	NEG	NEG	NEG	NEG	NEG	NEG	NEG	NEG	NEG	NEG	NEG	NEG	NEG	NEG	NEG
06V42236	POS	POS	NEG	NEG	NEG	POS	POS	POS	NEG	NEG	NEG	NEG	NEG	NEG	NEG	NEG	NEG	NEG	NEG	NEG	NEG	NEG	NEG	NEG	NEG
06V43697	POS	POS	NEG	NEG	NEG	POS	POS	POS	NEG	NEG	NEG	NEG	NEG	NEG	NEG	NEG	NEG	NEG	NEG	NEG	NEG	NEG	NEG	NEG	NEG
06V44153	POS	POS	NEG	NEG	NEG	POS	POS	#ZAHL!	NEG	NEG	NEG	NEG	NEG	NEG	NEG	NEG	NEG	NEG	NEG	NEG	NEG	NEG	NEG	NEG	NEG
06V45679	POS	POS	NEG	NEG	NEG	POS	POS	POS	NEG	NEG	NEG	NEG	NEG	NEG	NEG	NEG	NEG	NEG	NEG	NEG	NEG	NEG	NEG	NEG	NEG
06V45831	POS	POS	NEG	NEG	NEG	POS	POS	POS	NEG	NEG	NEG	NEG	NEG	NEG	NEG	NEG	NEG	NEG	NEG	NEG	NEG	NEG	NEG	NEG	NEG
06V46314	POS	POS	NEG	NEG	NEG	POS	POS	POS	NEG	NEG	NEG	NEG	NEG	NEG	NEG	NEG	NEG	NEG	NEG	NEG	NEG	NEG	NEG	NEG	NEG
06V34729	POS	POS	NEG	NEG	NEG	NEG	POS	POS	NEG	NEG	NEG	NEG	NEG	NEG	NEG	NEG	NEG	AMB	NEG	NEG	NEG	NEG	NEG	NEG	NEG
06B09900	POS	POS	NEG	NEG	NEG	POS	POS	POS	NEG	NEG	NEG	NEG	NEG	NEG	NEG	NEG	NEG	NEG	NEG	NEG	NEG	NEG	NEG	NEG	NEG
06V00922	POS	POS	NEG	NEG	NEG	POS	POS	POS	NEG	NEG	NEG	NEG	NEG	NEG	NEG	NEG	NEG	NEG	NEG	NEG	NEG	NEG	NEG	NEG	NEG
06V09551	POS	POS	NEG	NEG	NEG	POS	POS	POS	NEG	NEG	NEG	NEG	NEG	NEG	NEG	NEG	NEG	NEG	NEG	NEG	NEG	NEG	NEG	NEG	NEG
06V10719	POS	POS	NEG	NEG	NEG	POS	POS	POS	NEG	NEG	NEG	NEG	NEG	NEG	NEG	NEG	NEG	NEG	NEG	NEG	NEG	NEG	NEG	NEG	NEG
06V11090	POS	POS	NEG	NEG	NEG	POS	POS	POS	NEG	NEG	NEG	NEG	NEG	NEG	NEG	NEG	NEG	NEG	NEG	NEG	NEG	NEG	NEG	NEG	NEG
06V14383	POS	POS	NEG	NEG	NEG	POS	POS	POS	NEG	NEG	NEG	NEG	NEG	NEG	NEG	NEG	NEG	NEG	NEG	NEG	NEG	NEG	NEG	NEG	NEG
06V21475	POS	POS	NEG	NEG	NEG	POS	POS	POS	NEG	NEG	NEG	NEG	NEG	NEG	NEG	NEG	NEG	NEG	NEG	NEG	NEG	NEG	NEG	NEG	NEG
06V24231	POS	POS	NEG	NEG	NEG	POS	POS	POS	NEG	NEG	NEG	NEG	NEG	NEG	NEG	NEG	NEG	NEG	NEG	NEG	NEG	NEG	NEG	NEG	NEG
06V32992	POS	POS	NEG	NEG	NEG	POS	POS	POS	NEG	NEG	NEG	NEG	NEG	NEG	NEG	NEG	NEG	NEG	NEG	NEG	NEG	NEG	NEG	NEG	NEG
06V38753	POS	POS	NEG	NEG	NEG	POS	POS	POS	NEG	NEG	NEG	NEG	NEG	NEG	NEG	NEG	NEG	NEG	NEG	NEG	NEG	NEG	NEG	NEG	NEG
06V39848																									
06V42259	POS	POS	NEG	NEG	NEG	POS	POS	POS	NEG	NEG	NEG	NEG	NEG	NEG	NEG	NEG	NEG	NEG	NEG	NEG	NEG	NEG	NEG	NEG	NEG
06V33960																									

ISOLATE:	STRAIN	RESISTANCE : PENICILLINASE			RESISTANCE : MLS-ANTIBIOTICS												RESISTANCE : AMINOGLYSOSIDES			RESISTANCE : MISCELLANEOUS GENES																RESISTANCE : EFFLUX SYSTEMS								RESISTANCE : GLYCOPEPTIDES		
		beta-lactamase	beta lactamase repressor (inhibitor)	beta-lactamase regulatory protein	rRNA adenine N-6-methyl-transferase, erythromycin/clindamycin resistance	erythro-mycin/clindamycin resistance	erythro-mycin/clindamycin resistance	Linco-samid-Nucleo-tidyltrans-ferase	energy-dependent efflux of erythro-mycin	macrolide efflux protein A	probable lysylphos-phatidyl-glycerol synthetase	virginia-mycin A acetyltransferase	acetyl-transferase inactivating streptogramin A	ATP binding protein, streptogramin-A-resistance		virginia-mycin B hydrolase	bifunctional enzyme Aac/Aph, gentamicin resistance	amino-glycoside adenyl-transferase,tobramycin resistance	3'5'-aminog-lycoside phospho-transferase, neo- / kanamycin resistance	strepto-thricine-acetyl-transferase	dihydro-folate reductase type 1	fusidic acid resistence	hypothetical protein associated with fusidic acid resistance	mupirocin resistence protein	tetrazyklin-resistance	tetracycline-resistance	chloramphenicol acetyltransferase					23S rRNA methyltransferase	chloramphenicol /florfenicol exporter	metallothiol transferase		quaternary ammonium compound resistance protein A	quaternary ammonium compound resistance protein C						Transport/ Efflux protein	vancomycin resistance gene	vancomycin resistance gene from enterococci and Clostridium	teicoplanin resistance gene from enterococci
		blaZ	blaI	blaR	erm(A)	erm(B)	erm(C)	lnu(A)	msr(A)	mef(A)	mph(C)	vat(A)	vat(B)	vga(A)	vga(A) (BM 3327)	vgB(A)	aacA-aphD	aadD	aphA3	sat	dfrA	far1	Q6GD50	mupR	tet(K)	tet(M)	cat (total)	cat (pC221)	cat (pc223)	cat (pMC524)	cat (pSBK203R)	cfr	fexA	fosB	fosB (plasmid)	qacA	qacC (total)	qacC (consensus)	qacC (equine)	qacC (SA5)	qacC (Ssap)	qacC (ST94)	tetEfflux	vanA	vanB	vanZ
**2005**																																														
05B01179	CC5/ST228-MRSA-I, South German EMRSA	POS	POS	POS	POS		NEG	NEG	NEG	NEG	NEG	NEG	NEG	NEG	NEG	NEG	POS	NEG	POS	POS	NEG	NEG	NEG	NEG	NEG	NEG	NEG	NEG	NEG	NEG	NEG	NEG	NEG	POS	NEG	POS	NEG	NEG	NEG	NEG	NEG	NEG	POS	NEG	NEG	NEG
05B02687	CC5/ST228-MRSA-I, South German EMRSA	POS	POS	POS	POS		NEG	NEG	NEG	NEG	NEG	NEG	NEG	NEG	NEG	NEG	POS	NEG	POS	POS	NEG	NEG	NEG	NEG	NEG	NEG	NEG	NEG	NEG	NEG	NEG	NEG	NEG	POS	NEG	POS	NEG	NEG	NEG	NEG	NEG	NEG	POS	NEG	NEG	NEG
05V08483	CC5/ST228-MRSA-I, South German EMRSA	POS	POS	POS	POS		NEG	NEG	NEG	NEG	NEG	NEG	NEG	NEG	NEG	NEG	POS	NEG	POS	POS	NEG	NEG	NEG	NEG	NEG	NEG	NEG	NEG	NEG	NEG	NEG	NEG	NEG	POS	NEG	POS	NEG	NEG	NEG	NEG	NEG	NEG	POS	NEG	NEG	NEG
05V19001	CC5/ST228-MRSA-I, South German EMRSA	POS	POS	POS	POS		NEG	NEG	NEG	NEG	NEG	NEG	NEG	NEG	NEG	NEG	POS	NEG	POS	POS	NEG	NEG	NEG	NEG	NEG	NEG	NEG	NEG	NEG	NEG	NEG	NEG	NEG	POS	NEG	POS	NEG	NEG	NEG	NEG	NEG	NEG	POS	NEG	NEG	NEG
05B04626	CC5-MRSA-II, Rhine-Hesse EMRSA/UK-EMRSA-3	POS	POS	POS	POS		NEG	NEG	NEG	NEG	NEG	NEG	NEG	NEG	NEG	NEG	NEG	POS	NEG	NEG	NEG	NEG	NEG	NEG	NEG	NEG	NEG	NEG	NEG	NEG	NEG	NEG	NEG	POS	NEG	NEG	NEG	NEG	NEG	NEG	NEG	NEG	POS	NEG	NEG	NEG
05B07903	CC5-MRSA-II, Rhine-Hesse EMRSA/UK-EMRSA-3	NEG	NEG	NEG	POS		NEG	NEG	NEG	NEG	NEG	NEG	NEG	NEG	NEG	NEG	NEG	POS	NEG	NEG	NEG	NEG	NEG	NEG	NEG	NEG	NEG	NEG	NEG	NEG	NEG	NEG	NEG	POS	NEG	NEG	NEG	NEG	NEG	NEG	NEG	NEG	POS	NEG	NEG	NEG
05U00062	CC5-MRSA-II, Rhine-Hesse EMRSA/UK-EMRSA-3	POS	POS	POS	POS	NEG	NEG	NEG	NEG	NEG	NEG	NEG	NEG	NEG	NEG	NEG	NEG	POS	NEG	NEG	NEG	NEG	NEG	NEG	NEG	NEG	NEG	NEG	NEG	NEG	NEG	NEG	NEG	POS	NEG	NEG	NEG	NEG	NEG	NEG	NEG	NEG	POS	NEG	NEG	NEG
05U03117	CC5-MRSA-II, Rhine-Hesse EMRSA/UK-EMRSA-3	POS	POS	POS	POS	NEG	NEG	NEG	NEG	NEG	NEG	NEG	NEG	NEG	NEG	NEG	NEG	AMB	NEG	NEG	NEG	NEG	NEG	NEG	NEG	NEG	NEG	NEG	NEG	NEG	NEG	NEG	NEG	POS	NEG	NEG	NEG	NEG	NEG	NEG	NEG	NEG	POS	NEG	NEG	NEG
05U10392	CC5-MRSA-II, Rhine-Hesse EMRSA/UK-EMRSA-3	POS	POS	POS	POS	NEG	NEG	NEG	NEG	NEG	NEG	NEG	NEG	NEG	NEG	NEG	NEG	POS	NEG	NEG	NEG	NEG	NEG	NEG	NEG	NEG	NEG	NEG	NEG	NEG	NEG	NEG	NEG	POS	NEG	NEG	NEG	NEG	NEG	NEG	NEG	NEG	POS	NEG	NEG	NEG
05V03312	CC5-MRSA-II, Rhine-Hesse EMRSA/UK-EMRSA-3	POS	POS	POS	POS		NEG	NEG	NEG	NEG	NEG	NEG	NEG	NEG	NEG	NEG	NEG	POS	NEG	NEG	NEG	NEG	NEG	NEG	NEG	NEG	NEG	NEG	NEG	NEG	NEG	NEG	NEG	POS	NEG	NEG	NEG	NEG	NEG	NEG	NEG	NEG	POS	NEG	NEG	NEG
05V08095	CC5-MRSA-II, Rhine-Hesse EMRSA/UK-EMRSA-3	POS	POS	POS	POS	NEG	NEG	NEG	NEG	NEG	NEG	NEG	NEG	NEG	NEG	NEG	NEG	POS	NEG	NEG	NEG	NEG	NEG	NEG	NEG	NEG	NEG	NEG	NEG	NEG	NEG	NEG	NEG	POS	NEG	NEG	NEG	NEG	NEG	NEG	NEG	NEG	POS	NEG	NEG	NEG
05V09015	CC5-MRSA-II, Rhine-Hesse EMRSA/UK-EMRSA-3	POS	POS	POS	POS	NEG	NEG	NEG	NEG	NEG	NEG	NEG	NEG	NEG	NEG	NEG	NEG	POS	NEG	NEG	NEG	NEG	NEG	NEG	NEG	NEG	NEG	NEG	NEG	NEG	NEG	NEG	NEG	POS	NEG	NEG	NEG	NEG	NEG	NEG	NEG	NEG	POS	NEG	NEG	NEG
05V09326	CC5-MRSA-II, Rhine-Hesse EMRSA/UK-EMRSA-3	POS	POS	POS	POS	NEG	NEG	NEG	NEG	NEG	NEG	NEG	NEG	NEG	NEG	NEG	NEG	POS	NEG	NEG	NEG	NEG	NEG	NEG	NEG	NEG	NEG	NEG	NEG	NEG	NEG	NEG	NEG	POS	NEG	NEG	NEG	NEG	NEG	NEG	NEG	NEG	POS	NEG	NEG	NEG
05V10265	CC5-MRSA-II, Rhine-Hesse EMRSA/UK-EMRSA-3	NEG	NEG	NEG	POS		NEG	NEG	NEG	NEG	NEG	NEG	NEG	NEG	NEG	NEG	NEG	POS	NEG	NEG	NEG	NEG	NEG	NEG	NEG	NEG	NEG	NEG	NEG	NEG	NEG	NEG	NEG	POS	NEG	NEG	NEG	NEG	NEG	NEG	NEG	NEG	POS	NEG	NEG	NEG
05V13052	CC5-MRSA-II, Rhine-Hesse EMRSA/UK-EMRSA-3	POS	POS	POS	POS		NEG	NEG	NEG	NEG	NEG	NEG	NEG	NEG	NEG	NEG	NEG	POS	NEG	NEG	NEG	NEG	NEG	NEG	NEG	NEG	NEG	NEG	NEG	NEG	NEG	NEG	NEG	POS	NEG	NEG	NEG	NEG	NEG	NEG	NEG	NEG	POS	NEG	NEG	NEG
05V13470	CC5-MRSA-II, Rhine-Hesse EMRSA/UK-EMRSA-3	POS	POS	POS	POS	NEG	NEG	NEG	NEG	NEG	NEG	NEG	NEG	NEG	NEG	NEG	NEG	POS	NEG	NEG	NEG	NEG	NEG	NEG	NEG	NEG	NEG	NEG	NEG	NEG	NEG	NEG	NEG	POS	NEG	NEG	NEG	NEG	NEG	NEG	NEG	NEG	POS	NEG	NEG	NEG
05V13891	CC5-MRSA-II, Rhine-Hesse EMRSA/UK-EMRSA-3	NEG	NEG	NEG	POS	NEG	NEG	NEG	NEG	NEG	NEG	NEG	NEG	NEG	NEG	NEG	NEG	NEG	NEG	NEG	NEG	NEG	NEG	NEG	NEG	NEG	NEG	NEG	NEG	NEG	NEG	NEG	NEG	POS	NEG	NEG	NEG	NEG	NEG	NEG	NEG	NEG	POS	NEG	NEG	NEG
05V15616	CC5-MRSA-II, Rhine-Hesse EMRSA/UK-EMRSA-3	POS			POS		NEG	NEG	NEG			NEG	NEG	NEG	NEG	NEG	NEG	NEG	NEG	NEG	NEG	NEG		NEG	NEG	NEG																		NEG	NEG	NEG
05V16845	CC5-MRSA-II, Rhine-Hesse EMRSA/UK-EMRSA-3	POS	POS	POS	POS	NEG	NEG	NEG	NEG	NEG	NEG	NEG	NEG	NEG	NEG	NEG	NEG	POS	NEG	NEG	NEG	NEG	NEG	NEG	NEG	NEG	NEG	NEG	NEG	NEG	NEG	NEG	NEG	POS	NEG	NEG	NEG	NEG	NEG	NEG	NEG	NEG	POS	NEG	NEG	NEG
05V17541	CC5-MRSA-II, Rhine-Hesse EMRSA/UK-EMRSA-3	NEG	NEG	NEG	POS	NEG	NEG	NEG	NEG	NEG	NEG	NEG	NEG	NEG	NEG	NEG	NEG	NEG	NEG	NEG	NEG	NEG	NEG	NEG	NEG	NEG	NEG	NEG	NEG	NEG	NEG	NEG	NEG	POS	NEG	NEG	NEG	NEG	NEG	NEG	NEG	NEG	POS	NEG	NEG	NEG
05V19105	CC5-MRSA-II, Rhine-Hesse EMRSA/UK-EMRSA-3	POS			POS		NEG	NEG	NEG			NEG	NEG	NEG	NEG	NEG	NEG	POS	NEG	NEG	NEG	NEG		NEG	NEG	NEG																		NEG	NEG	NEG
05V22148	CC5-MRSA-II, Rhine-Hesse EMRSA/UK-EMRSA-3	POS	POS	POS	POS	NEG	NEG	NEG	NEG	NEG	NEG	NEG	NEG	NEG	NEG	NEG	NEG	POS	NEG	NEG	NEG	NEG	NEG	NEG	NEG	NEG	NEG	NEG	NEG	NEG	NEG	NEG	NEG	POS	NEG	NEG	NEG	NEG	NEG	NEG	NEG	NEG	POS	NEG	NEG	NEG
05V33740	CC5-MRSA-II, Rhine-Hesse EMRSA/UK-EMRSA-3	POS	POS	POS	POS	NEG	NEG	NEG	NEG	NEG	NEG	NEG	NEG	NEG	NEG	NEG	NEG	POS	NEG	NEG	NEG	NEG	NEG	NEG	NEG	NEG	NEG	NEG	NEG	NEG	NEG	NEG	NEG	POS	NEG	NEG	NEG	NEG	NEG	NEG	NEG	NEG	POS	NEG	NEG	NEG
05V38425	CC5-MRSA-II, Rhine-Hesse EMRSA/UK-EMRSA-3	POS	POS	POS	POS	NEG	NEG	NEG	NEG	NEG	NEG	NEG	NEG	NEG	NEG	NEG	NEG	POS	NEG	NEG	NEG	NEG	NEG	NEG	NEG	NEG	NEG	NEG	NEG	NEG	NEG	NEG	NEG	POS	NEG	NEG	NEG	NEG	NEG	NEG	NEG	NEG	POS	NEG	NEG	NEG
05V39987	CC5-MRSA-II, Rhine-Hesse EMRSA/UK-EMRSA-3	NEG	NEG	NEG	POS	NEG	NEG	NEG	NEG	NEG	NEG	NEG	NEG	NEG	NEG	NEG	NEG	POS	NEG	NEG	NEG	NEG	NEG	NEG	NEG	NEG	NEG	NEG	NEG	NEG	NEG	NEG	NEG	POS	NEG	NEG	NEG	NEG	NEG	NEG	NEG	NEG	POS	NEG	NEG	NEG
05F00089	CC5-MRSA-IV, Paediatric clone	POS	POS	POS	NEG		POS	NEG	NEG	NEG	NEG	NEG	NEG	NEG	NEG	NEG	NEG	POS	NEG	NEG	NEG	NEG	NEG	NEG	NEG	NEG	NEG	NEG	NEG	NEG	NEG	NEG	NEG	POS	NEG	NEG	NEG	NEG	NEG	NEG	NEG	NEG	POS	NEG	NEG	NEG
05V01416	CC5-MRSA-IV, Paediatric clone	POS	POS	POS	NEG	NEG	NEG	NEG	NEG	NEG	NEG	NEG	NEG	NEG	NEG	NEG	NEG	NEG	NEG	NEG	NEG	NEG	NEG	NEG	NEG	NEG	NEG	NEG	NEG	NEG	NEG	NEG	NEG	POS	NEG	NEG	NEG	NEG	NEG	NEG	NEG	NEG	AMB	NEG	NEG	NEG
05V16386	CC8-MRSA-IV, USA300	POS	POS	POS	NEG		NEG	NEG	POS	NEG	POS	NEG	NEG	NEG	NEG	NEG	NEG	NEG	POS	POS	NEG	NEG	NEG	NEG	NEG	NEG	NEG	NEG	NEG	NEG	NEG	NEG	NEG	POS	NEG	NEG	NEG	NEG	NEG	NEG	NEG	NEG	POS	NEG	NEG	NEG
05V04920	CC8-MRSA-IV, USA500	POS	POS	POS	NEG		POS	NEG	NEG	NEG	NEG	NEG	NEG	NEG	NEG	NEG	POS	NEG	NEG	NEG	NEG	NEG	NEG	NEG	NEG	POS	NEG	NEG	NEG	NEG	NEG	NEG	NEG	POS	NEG	POS	POS	NEG	POS	NEG	NEG	NEG	POS	NEG	NEG	NEG
05V21159	CC8-MRSA-IV, USA500	POS	POS	POS	NEG		POS	NEG	NEG	NEG	NEG	NEG	NEG	NEG	NEG	NEG	NEG	NEG	NEG	NEG	NEG	NEG	NEG	NEG	NEG	NEG	NEG	NEG	NEG	NEG	NEG	NEG	NEG	POS	NEG	NEG	NEG	NEG	NEG	NEG	NEG	NEG	POS	NEG	NEG	NEG
05B00867	CC22-MRSA-IV, Barnim/UK-EMRSA-15	POS	POS	POS	NEG		NEG	NEG	NEG	NEG	NEG	NEG	NEG	NEG	NEG	NEG	NEG	NEG	NEG	NEG	NEG	NEG	NEG	NEG	NEG	NEG	NEG	NEG	NEG	NEG	NEG	NEG	NEG	NEG	NEG	NEG	NEG	NEG	NEG	NEG	NEG	NEG	NEG	NEG	NEG	NEG
05V03174	CC22-MRSA-IV, Barnim/UK-EMRSA-15	POS	POS	POS	NEG	NEG	NEG	NEG	NEG	NEG	NEG	NEG	NEG	NEG	NEG	NEG	NEG	NEG	NEG	NEG	NEG	NEG	NEG	NEG	NEG	NEG	NEG	NEG	NEG	NEG	NEG	NEG	NEG	NEG	NEG	NEG	NEG	NEG	NEG	NEG	NEG	NEG	NEG	NEG	NEG	NEG
05V07331	CC22-MRSA-IV, Barnim/UK-EMRSA-15	POS	POS	POS	NEG	NEG	NEG	NEG	NEG	NEG	NEG	NEG	NEG	NEG	NEG	NEG	NEG	NEG	NEG	NEG	NEG	NEG	NEG	NEG	NEG	NEG	NEG	NEG	NEG	NEG	NEG	NEG	NEG	NEG	NEG	NEG	NEG	NEG	NEG	NEG	NEG	NEG	NEG	NEG	NEG	NEG
05V07371	CC22-MRSA-IV, Barnim/UK-EMRSA-15	POS	POS	POS	NEG	NEG	POS	NEG	NEG	NEG	NEG	NEG	NEG	NEG	NEG	NEG	NEG	NEG	NEG	NEG	NEG	NEG	NEG	NEG	NEG	NEG	NEG	NEG	NEG	NEG	NEG	NEG	NEG	NEG	NEG	NEG	NEG	NEG	NEG	NEG	NEG	NEG	NEG	NEG	NEG	NEG
05V08145	CC22-MRSA-IV, Barnim/UK-EMRSA-15	POS	POS	AMB	NEG	NEG	POS	NEG	NEG	NEG	NEG	NEG	NEG	NEG	NEG	NEG	NEG	NEG	NEG	NEG	NEG	NEG	NEG	NEG	AMB	NEG	NEG	NEG	NEG	NEG	NEG	NEG	NEG	NEG	NEG	NEG	NEG	NEG	NEG	NEG	NEG	NEG	NEG	NEG	NEG	NEG
05V08595	CC22-MRSA-IV, Barnim/UK-EMRSA-15	POS	POS	POS	NEG	NEG	NEG	NEG	NEG	NEG	NEG	NEG	NEG	NEG	NEG	NEG	NEG	NEG	NEG	NEG	NEG	NEG	NEG	NEG	NEG	NEG	NEG	NEG	NEG	NEG	NEG	NEG	NEG	NEG	NEG	NEG	NEG	NEG	NEG	NEG	NEG	NEG	NEG	NEG	NEG	NEG
05V12803	CC22-MRSA-IV, Barnim/UK-EMRSA-15	POS	POS	POS	NEG	NEG	NEG	NEG	NEG	NEG	NEG	NEG	NEG	NEG	NEG	NEG	NEG	NEG	NEG	NEG	NEG	NEG	NEG	NEG	NEG	NEG	NEG	NEG	NEG	NEG	NEG	NEG	NEG	NEG	NEG	POS	NEG	NEG	NEG	NEG	NEG	NEG	NEG	NEG	NEG	NEG
05V14596	CC22-MRSA-IV, Barnim/UK-EMRSA-15	POS	POS	POS	NEG		POS	NEG	NEG	NEG	NEG	NEG	NEG	NEG	NEG	NEG	NEG	NEG	NEG	NEG	NEG	NEG	NEG	NEG	NEG	NEG	NEG	NEG	NEG	NEG	NEG	NEG	NEG	NEG	NEG	NEG	NEG	NEG	NEG	NEG	NEG	NEG	NEG	NEG	NEG	NEG
05V16691	CC22-MRSA-IV, Barnim/UK-EMRSA-15	POS			NEG		NEG	NEG	NEG			NEG	NEG	NEG	NEG	NEG	NEG	NEG	NEG	NEG	NEG	NEG		NEG	NEG	NEG																		NEG	NEG	NEG
05V18550	CC22-MRSA-IV, Barnim/UK-EMRSA-15	POS	POS	POS	NEG	NEG	NEG	NEG	NEG	NEG	NEG	NEG	NEG	NEG	NEG	NEG	NEG	NEG	NEG	NEG	NEG	NEG	NEG	NEG	NEG	NEG	NEG	NEG	NEG	NEG	NEG	NEG	NEG	NEG	NEG	NEG	NEG	NEG	NEG	NEG	NEG	NEG	NEG	NEG	NEG	NEG
05V19034	CC22-MRSA-IV, Barnim/UK-EMRSA-15	POS	POS	POS	NEG	NEG	NEG	NEG	NEG	NEG	NEG	NEG	NEG	NEG	NEG	NEG	NEG	NEG	NEG	NEG	NEG	NEG	NEG	NEG	NEG	NEG	NEG	NEG	NEG	NEG	NEG	NEG	NEG	NEG	NEG	NEG	NEG	NEG	NEG	NEG	NEG	NEG	NEG	NEG	NEG	NEG
05V19333	CC22-MRSA-IV, Barnim/UK-EMRSA-15	POS			NEG		NEG	NEG	NEG			NEG	NEG	NEG	NEG	NEG	NEG	NEG	NEG	NEG	NEG	NEG		NEG	NEG	NEG																		NEG	NEG	NEG
05V19701	CC22-MRSA-IV, Barnim/UK-EMRSA-15	POS	POS	POS	NEG	NEG	NEG	NEG	NEG	NEG	NEG	NEG	NEG	NEG	NEG	NEG	NEG	NEG	NEG	NEG	NEG	NEG	NEG	NEG	NEG	NEG	NEG	NEG	NEG	NEG	NEG	NEG	NEG	NEG	NEG	NEG	NEG	NEG	NEG	NEG	NEG	NEG	NEG	NEG	NEG	NEG
05V20072	CC22-MRSA-IV, Barnim/UK-EMRSA-15	POS	POS	POS	NEG	NEG	NEG	NEG	NEG	NEG	NEG	NEG	NEG	NEG	NEG	NEG	NEG	NEG	NEG	NEG	NEG	NEG	NEG	NEG	NEG	NEG	NEG	NEG	NEG	NEG	NEG	NEG	NEG	NEG	NEG	NEG	NEG	NEG	NEG	NEG	NEG	NEG	NEG	NEG	NEG	NEG
05V24930	CC22-MRSA-IV, Barnim/UK-EMRSA-15	POS	POS	POS	NEG	NEG	POS	NEG	NEG	NEG	NEG	NEG	NEG	NEG	NEG	NEG	NEG	NEG	NEG	NEG	NEG	NEG	NEG	NEG	NEG	NEG	NEG	NEG	NEG	NEG	NEG	NEG	NEG	NEG	NEG	NEG	NEG	NEG	NEG	NEG	NEG	NEG	NEG	NEG	NEG	NEG
05V31461	CC22-MRSA-IV, Barnim/UK-EMRSA-15	POS	POS	POS	NEG	NEG	POS	NEG	NEG	NEG	NEG	NEG	NEG	NEG	NEG	NEG	NEG	NEG	NEG	NEG	NEG	NEG	NEG	NEG	NEG	NEG	NEG	NEG	NEG	NEG	NEG	NEG	NEG	NEG	NEG	NEG	NEG	NEG	NEG	NEG	NEG	NEG	NEG	NEG	NEG	NEG
05B05671	CC45-MRSA-IV, Berlin EMRSA	POS	POS	POS	NEG		NEG	NEG	NEG	NEG	NEG	NEG	NEG	NEG	NEG	NEG	NEG	NEG	POS	POS	NEG	NEG	NEG	NEG	NEG	NEG	NEG	NEG	NEG	NEG	NEG	NEG	NEG	NEG	NEG	NEG	NEG	NEG	NEG	NEG	NEG	NEG	POS	NEG	NEG	NEG
05V01452	CC45-MRSA-IV, Berlin EMRSA	POS	POS	POS	NEG		POS	NEG	NEG	NEG	NEG	NEG	NEG	NEG	NEG	NEG	NEG	NEG	POS	POS	NEG	NEG	NEG	NEG	NEG	NEG	NEG	NEG	NEG	NEG	NEG	NEG	NEG	NEG	NEG	NEG	NEG	NEG	NEG	NEG	NEG	NEG	POS	NEG	NEG	NEG
05V01617	CC45-MRSA-IV, Berlin EMRSA	POS	POS	POS	NEG	NEG	POS	NEG	NEG	NEG	NEG	NEG	NEG	NEG	NEG	NEG	NEG	NEG	POS	POS	NEG	NEG	NEG	NEG	NEG	NEG	NEG	NEG	NEG	NEG	NEG	NEG	NEG	NEG	NEG	NEG	NEG	NEG	NEG	NEG	NEG	NEG	POS	NEG	NEG	NEG
05V16986	CC45-MRSA-IV, Berlin EMRSA	POS	POS	POS	NEG	NEG	NEG	NEG	NEG	NEG	NEG	NEG	NEG	NEG	NEG	NEG	NEG	NEG	AMB	POS	NEG	NEG	NEG	NEG	NEG	NEG	NEG	NEG	NEG	NEG	NEG	NEG	NEG	NEG	NEG	NEG	NEG	NEG	NEG	NEG	NEG	NEG	AMB	NEG	NEG	NEG
05V18357	CC45-MRSA-IV, Berlin EMRSA	POS	POS	POS	NEG		NEG	NEG	NEG	NEG	NEG	NEG	NEG	NEG	NEG	NEG	NEG	NEG	POS	POS	NEG	NEG	NEG	NEG	NEG	NEG	NEG	NEG	NEG	NEG	NEG	NEG	NEG	NEG	NEG	NEG	NEG	NEG	NEG	NEG	NEG	NEG	NEG	NEG	NEG	NEG
05V23111	CC45-MRSA-IV, Berlin EMRSA	POS	POS	POS	NEG	NEG	NEG	NEG	NEG	NEG	NEG	NEG	NEG	NEG	NEG	NEG	NEG	NEG	POS	POS	NEG	NEG	NEG	NEG	NEG	NEG	NEG	NEG	NEG	NEG	NEG	NEG	NEG	NEG	NEG	NEG	NEG	NEG	NEG	NEG	NEG	NEG	POS	NEG	NEG	NEG
05V23414	CC45-MRSA-IV, Berlin EMRSA	POS			NEG		NEG	NEG	NEG			NEG	NEG	NEG	NEG	NEG	NEG	NEG	POS	POS	NEG	NEG		NEG	NEG	NEG																		NEG	NEG	NEG
05V23858	CC45-MRSA-IV, Berlin EMRSA	POS	POS	POS	NEG	NEG	NEG	NEG	NEG	NEG	NEG	NEG	NEG	NEG	NEG	NEG	NEG	NEG	POS	POS	NEG	NEG	NEG	NEG	NEG	NEG	NEG	NEG	NEG	NEG	NEG	NEG	NEG	NEG	NEG	NEG	NEG	NEG	NEG	NEG	NEG	NEG	POS	NEG	NEG	NEG
05V26241	CC45-MRSA-IV, Berlin EMRSA	POS	POS	POS	NEG	NEG	POS	NEG	NEG	NEG	NEG	NEG	NEG	NEG	NEG	NEG	NEG	POS	POS	POS	NEG	NEG	NEG	POS	NEG	NEG	NEG	NEG	NEG	NEG	NEG	NEG	NEG	NEG	NEG	NEG	POS	POS	NEG	NEG	NEG	NEG	POS	NEG	NEG	NEG
05V28399	CC45-MRSA-IV, Berlin EMRSA	POS	POS	POS	NEG	NEG	NEG	NEG	NEG	NEG	NEG	NEG	NEG	NEG	NEG	NEG	NEG	NEG	POS	POS	NEG	NEG	NEG	NEG	NEG	NEG	NEG	NEG	NEG	NEG	NEG	NEG	NEG	NEG	NEG	NEG	NEG	NEG	NEG	NEG	NEG	NEG	POS	NEG	NEG	NEG
05V32647	CC45-MRSA-IV, Berlin EMRSA	POS			NEG		NEG	NEG	NEG			NEG	NEG	NEG	NEG	NEG	NEG	NEG	POS	POS	NEG	NEG		NEG	NEG	NEG																		NEG	NEG	NEG
05V37969	CC45-MRSA-IV, Berlin EMRSA	POS	POS	POS	NEG		NEG	NEG	NEG	NEG	NEG	NEG	NEG	NEG	NEG	NEG	NEG	NEG	POS	POS	NEG	NEG	NEG	NEG	NEG	NEG	NEG	NEG	NEG	NEG	NEG	NEG	NEG	NEG	NEG	NEG	NEG	NEG	NEG	NEG	NEG	NEG	POS	NEG	NEG	NEG
05U10018	CC398-MRSA-V	POS	POS	POS	NEG		NEG	NEG	NEG	NEG	NEG	NEG	NEG	NEG	NEG	NEG	NEG	NEG	NEG	NEG	NEG	NEG	NEG	NEG	AMB	POS	NEG	NEG	NEG	NEG	NEG	NEG	NEG	NEG	NEG	NEG	NEG	NEG	NEG	NEG	NEG	NEG	POS	NEG	NEG	NEG
**2006**																																														
06V36353	CC1-MRSA-IV, WA MRSA-1/57	POS	POS	POS	NEG		NEG	NEG	NEG	NEG	NEG	NEG	NEG	NEG	NEG	NEG	NEG	NEG	NEG	NEG	NEG	NEG	NEG	NEG	NEG	NEG	NEG	NEG	NEG	NEG	NEG	NEG	NEG	NEG	NEG	NEG	NEG	NEG	NEG	NEG	NEG	NEG	POS	NEG	NEG	NEG
06V40202	CC5/ST228-MRSA-I, South German EMRSA	POS			POS		NEG	NEG	NEG			NEG	NEG	NEG	NEG	NEG	POS	NEG	POS	POS	NEG	NEG		NEG	NEG	NEG																		NEG	NEG	NEG
06V41266	CC5/ST228-MRSA-I, South German EMRSA	POS	POS	POS	POS	NEG	NEG	NEG	NEG	NEG	NEG	NEG	NEG	NEG	NEG	NEG	POS	NEG	POS	POS	NEG	NEG	NEG	NEG	NEG	NEG	NEG	NEG	NEG	NEG	NEG	NEG	NEG	POS	NEG	POS	NEG	NEG	NEG	NEG	NEG	NEG	POS	NEG	NEG	NEG
06B09097	CC5-MRSA-II, Rhine-Hesse EMRSA/UK-EMRSA-3	NEG			POS		NEG	NEG	NEG			NEG	NEG	NEG	NEG	NEG	NEG	POS	NEG	NEG	NEG	NEG		NEG	NEG	NEG																		NEG	NEG	NEG
06F00244	CC5-MRSA-II, Rhine-Hesse EMRSA/UK-EMRSA-3	POS	POS	POS	POS	NEG	NEG	NEG	NEG	NEG	NEG	NEG	NEG	NEG	NEG	NEG	NEG	POS	NEG	NEG	NEG	NEG	NEG	NEG	NEG	NEG	NEG	NEG	NEG	NEG	NEG	NEG	NEG	POS	NEG	NEG	NEG	NEG	NEG	NEG	NEG	NEG	POS	NEG	NEG	NEG
06V03913	CC5-MRSA-II, Rhine-Hesse EMRSA/UK-EMRSA-3	POS	POS	POS	POS	NEG	NEG	NEG	NEG	NEG	NEG	NEG	NEG	NEG	NEG	NEG	NEG	POS	NEG	NEG	NEG	NEG	NEG	NEG	NEG	NEG	NEG	NEG	NEG	NEG	NEG	NEG	NEG	POS	NEG	NEG	NEG	NEG	NEG	NEG	NEG	NEG	POS	NEG	NEG	NEG
06V04337	CC5-MRSA-II, Rhine-Hesse EMRSA/UK-EMRSA-3	POS	POS	POS	POS	NEG	NEG	NEG	NEG	NEG	NEG	NEG	NEG	NEG	NEG	NEG	NEG	POS	NEG	NEG	NEG	NEG	NEG	NEG	NEG	NEG	NEG	NEG	NEG	NEG	NEG	NEG	NEG	POS	NEG	NEG	NEG	NEG	NEG	NEG	NEG	NEG	POS	NEG	NEG	NEG
06V06785	CC5-MRSA-II, Rhine-Hesse EMRSA/UK-EMRSA-3	POS	POS	POS	POS	NEG	NEG	NEG	NEG	NEG	NEG	NEG	NEG	NEG	NEG	NEG	NEG	POS	NEG	NEG	NEG	NEG	NEG	NEG	NEG	NEG	NEG	NEG	NEG	NEG	NEG	NEG	NEG	POS	NEG	NEG	NEG	NEG	NEG	NEG	NEG	NEG	POS	NEG	NEG	NEG
06V12258	CC5-MRSA-II, Rhine-Hesse EMRSA/UK-EMRSA-3	POS	POS	POS	POS	NEG	NEG	NEG	NEG	NEG	NEG	NEG	NEG	NEG	NEG	NEG	NEG	POS	NEG	NEG	NEG	NEG	NEG	NEG	NEG	NEG	NEG	NEG	NEG	NEG	NEG	NEG	NEG	POS	NEG	NEG	NEG	NEG	NEG	NEG	NEG	NEG	POS	NEG	NEG	NEG
06V20553	CC5-MRSA-II, Rhine-Hesse EMRSA/UK-EMRSA-3	POS	POS	POS	POS	NEG	NEG	NEG	NEG	NEG	NEG	NEG	NEG	NEG	NEG	NEG	NEG	POS	NEG	NEG	NEG	NEG	NEG	NEG	NEG	NEG	NEG	NEG	NEG	NEG	NEG	NEG	NEG	POS	NEG	NEG	NEG	NEG	NEG	NEG	NEG	NEG	POS	NEG	NEG	NEG
06V22564	CC5-MRSA-II, Rhine-Hesse EMRSA/UK-EMRSA-3	POS			POS		NEG	NEG	NEG			NEG	NEG	NEG	NEG	NEG	NEG	POS	NEG	NEG	NEG	NEG		NEG	NEG	NEG																		NEG	NEG	NEG
06V23750	CC5-MRSA-II, Rhine-Hesse EMRSA/UK-EMRSA-3	POS	POS	POS	POS	NEG	NEG	NEG	NEG	NEG	NEG	NEG	NEG	NEG	NEG	NEG	NEG	POS	NEG	NEG	NEG	NEG	NEG	NEG	NEG	NEG	NEG	NEG	NEG	NEG	NEG	NEG	NEG	POS	NEG	NEG	NEG	NEG	NEG	NEG	NEG	NEG	POS	NEG	NEG	NEG
06V27332	CC5-MRSA-II, Rhine-Hesse EMRSA/UK-EMRSA-3	POS	POS	POS	POS	NEG	NEG	NEG	NEG	NEG	NEG	NEG	NEG	NEG	NEG	NEG	NEG	POS	NEG	NEG	NEG	NEG	NEG	NEG	NEG	NEG	NEG	NEG	NEG	NEG	NEG	NEG	NEG	POS	NEG	NEG	NEG	NEG	NEG	NEG	NEG	NEG	POS	NEG	NEG	NEG
06V29484	CC5-MRSA-II, Rhine-Hesse EMRSA/UK-EMRSA-3	POS	POS	POS	POS	NEG	NEG	NEG	NEG	NEG	NEG	NEG	NEG	NEG	NEG	NEG	NEG	POS	NEG	NEG	NEG	NEG	NEG	NEG	NEG	NEG	NEG	NEG	NEG	NEG	NEG	NEG	NEG	POS	NEG	NEG	NEG	NEG	NEG	NEG	NEG	NEG	POS	NEG	NEG	NEG
06V31278	CC5-MRSA-II, Rhine-Hesse EMRSA/UK-EMRSA-3	NEG			POS		NEG	NEG	NEG			NEG	NEG	NEG	NEG	NEG	NEG	POS	NEG	NEG	NEG	NEG		NEG	NEG	NEG																		NEG	NEG	NEG
06V36043	CC5-MRSA-II, Rhine-Hesse EMRSA/UK-EMRSA-3	POS	POS	POS	POS		NEG	NEG	NEG	NEG	NEG	NEG	NEG	NEG	NEG	NEG	POS	POS	POS	POS	NEG	NEG	NEG	NEG	NEG	POS	NEG	NEG	NEG	NEG	NEG	NEG	NEG	POS	NEG	NEG	NEG	NEG	NEG	NEG	NEG	NEG	POS	NEG	NEG	NEG
06V36349	CC5-MRSA-II, Rhine-Hesse EMRSA/UK-EMRSA-3	POS	POS	POS	POS	NEG	NEG	NEG	NEG	NEG	NEG	NEG	NEG	NEG	NEG	NEG	NEG	POS	NEG	NEG	NEG	NEG	NEG	NEG	NEG	NEG	NEG	NEG	NEG	NEG	NEG	NEG	NEG	POS	NEG	NEG	NEG	NEG	NEG	NEG	NEG	NEG	POS	NEG	NEG	NEG
06V39695	CC5-MRSA-II, Rhine-Hesse EMRSA/UK-EMRSA-3	POS	POS	POS	POS	NEG	NEG	NEG	NEG	NEG	NEG	NEG	NEG	NEG	NEG	NEG	NEG	NEG	NEG	NEG	NEG	NEG	NEG	NEG	NEG	NEG	NEG	NEG	NEG	NEG	NEG	NEG	NEG	POS	NEG	NEG	NEG	NEG	NEG	NEG	NEG	NEG	POS	NEG	NEG	NEG
06V40571	CC5-MRSA-II, Rhine-Hesse EMRSA/UK-EMRSA-3	POS			POS		NEG	NEG	NEG			NEG	NEG	NEG	NEG	NEG	NEG	POS	NEG	NEG	NEG	NEG		NEG	NEG	NEG																		NEG	NEG	NEG
06V42188	CC5-MRSA-II, Rhine-Hesse EMRSA/UK-EMRSA-3	NEG	NEG	NEG	POS	NEG	NEG	NEG	NEG	NEG	NEG	NEG	NEG	NEG	NEG	NEG	NEG	POS	NEG	NEG	NEG	NEG	NEG	NEG	NEG	NEG	NEG	NEG	NEG	NEG	NEG	NEG	NEG	POS	NEG	NEG	NEG	NEG	NEG	NEG	NEG	NEG	POS	NEG	NEG	NEG
06V42146	CC8-MRSA-IV, Lyon Clone/UK-EMRSA-2	POS			NEG		NEG	NEG	NEG			NEG	NEG	NEG	NEG	NEG	NEG	POS	NEG	NEG	NEG	NEG		NEG	NEG	NEG																		NEG	NEG	NEG
06V28560	CC8-MRSA-V	NEG	NEG	NEG	NEG	NEG	POS	NEG	NEG	NEG	NEG	NEG	NEG	NEG	NEG	NEG	NEG	NEG	NEG	NEG	NEG	NEG	NEG	NEG	POS	NEG	NEG	NEG	NEG	NEG	NEG	NEG	NEG	POS	NEG	NEG	NEG	NEG	NEG	NEG	NEG	NEG	POS	NEG	NEG	NEG
06F00837	CC22-MRSA-IV, Barnim/UK-EMRSA-15	POS	POS	POS	NEG	NEG	POS	NEG	NEG	NEG	NEG	NEG	NEG	NEG	NEG	NEG	NEG	NEG	NEG	NEG	NEG	NEG	NEG	NEG	NEG	NEG	NEG	NEG	NEG	NEG	NEG	NEG	NEG	NEG	NEG	NEG	NEG	NEG	NEG	NEG	NEG	NEG	NEG	NEG	NEG	NEG
06F01638	CC22-MRSA-IV, Barnim/UK-EMRSA-15	POS	POS	POS	NEG	NEG	POS	NEG	NEG	NEG	NEG	NEG	NEG	NEG	NEG	NEG	NEG	NEG	NEG	NEG	NEG	NEG	NEG	NEG	NEG	NEG	NEG	NEG	NEG	NEG	NEG	NEG	NEG	NEG	NEG	NEG	NEG	NEG	NEG	NEG	NEG	NEG	NEG	NEG	NEG	NEG
06F04643	CC22-MRSA-IV, Barnim/UK-EMRSA-15	AMB			NEG		NEG	NEG	NEG			NEG	NEG	NEG	NEG	NEG	NEG	NEG	NEG	NEG	NEG	NEG		NEG	NEG	NEG																		NEG	NEG	NEG
06F05580	CC22-MRSA-IV, Barnim/UK-EMRSA-15	POS	POS	POS	NEG	NEG	POS	NEG	NEG	NEG	NEG	NEG	NEG	NEG	NEG	NEG	NEG	NEG	NEG	NEG	NEG	NEG	NEG	NEG	NEG	NEG	NEG	NEG	NEG	NEG	NEG	NEG	NEG	NEG	NEG	NEG	NEG	NEG	NEG	NEG	NEG	NEG	NEG	NEG	NEG	NEG
06U05469	CC22-MRSA-IV, Barnim/UK-EMRSA-15	POS	POS	POS	NEG	NEG	POS	NEG	NEG	NEG	NEG	NEG	NEG	NEG	NEG	NEG	NEG	NEG	NEG	NEG	NEG	NEG	NEG	NEG	NEG	NEG	NEG	NEG	NEG	NEG	NEG	NEG	NEG	NEG	NEG	NEG	NEG	NEG	NEG	NEG	NEG	NEG	NEG	NEG	NEG	NEG
06U17314	CC22-MRSA-IV, Barnim/UK-EMRSA-15	POS	POS	POS	NEG	NEG	NEG	NEG	NEG	NEG	NEG	NEG	NEG	NEG	NEG	NEG	NEG	NEG	NEG	NEG	NEG	NEG	NEG	NEG	NEG	NEG	NEG	NEG	NEG	NEG	NEG	NEG	NEG	NEG	NEG	NEG	NEG	NEG	NEG	NEG	NEG	NEG	NEG	NEG	NEG	NEG
06V08377	CC22-MRSA-IV, Barnim/UK-EMRSA-15	POS	POS	POS	NEG	NEG	NEG	NEG	NEG	NEG	NEG	NEG	NEG	NEG	NEG	NEG	NEG	NEG	NEG	NEG	NEG	NEG	NEG	NEG	NEG	NEG	NEG	NEG	NEG	NEG	NEG	NEG	NEG	NEG	NEG	NEG	NEG	NEG	NEG	NEG	NEG	NEG	NEG	NEG	NEG	NEG
06V08474	CC22-MRSA-IV, Barnim/UK-EMRSA-15	POS	POS	POS	NEG	NEG	POS	NEG	NEG	NEG	NEG	NEG	NEG	NEG	NEG	NEG	NEG	NEG	NEG	NEG	NEG	NEG	NEG	NEG	NEG	NEG	NEG	NEG	NEG	NEG	NEG	NEG	NEG	NEG	NEG	NEG	NEG	NEG	NEG	NEG	NEG	NEG	NEG	NEG	NEG	NEG
06V09374	CC22-MRSA-IV, Barnim/UK-EMRSA-15	POS	POS	POS	NEG	NEG	POS	NEG	NEG	NEG	NEG	NEG	NEG	NEG	NEG	NEG	NEG	NEG	NEG	NEG	NEG	NEG	NEG	NEG	NEG	NEG	NEG	NEG	NEG	NEG	NEG	NEG	NEG	NEG	NEG	NEG	NEG	NEG	NEG	NEG	NEG	NEG	NEG	NEG	NEG	NEG
06V09605	CC22-MRSA-IV, Barnim/UK-EMRSA-15	POS	POS	POS	NEG	NEG	POS	NEG	NEG	NEG	NEG	NEG	NEG	NEG	NEG	NEG	NEG	NEG	NEG	NEG	NEG	NEG	NEG	NEG	NEG	NEG	NEG	NEG	NEG	NEG	NEG	NEG	NEG	NEG	NEG	NEG	NEG	NEG	NEG	NEG	NEG	NEG	NEG	NEG	NEG	NEG
06V18687	CC22-MRSA-IV, Barnim/UK-EMRSA-15	POS	POS	AMB	NEG	NEG	NEG	NEG	NEG	NEG	NEG	NEG	NEG	NEG	NEG	NEG	NEG	NEG	NEG	NEG	NEG	NEG	NEG	NEG	NEG	NEG	NEG	NEG	NEG	NEG	NEG	NEG	NEG	NEG	NEG	NEG	NEG	NEG	NEG	NEG	NEG	NEG	NEG	NEG	NEG	NEG
06V21697	CC22-MRSA-IV, Barnim/UK-EMRSA-15	POS	POS	POS	NEG	NEG	POS	NEG	NEG	NEG	NEG	NEG	NEG	NEG	NEG	NEG	NEG	NEG	NEG	AMB	NEG	NEG	NEG	NEG	NEG	NEG	NEG	NEG	NEG	NEG	NEG	NEG	NEG	NEG	NEG	NEG	NEG	NEG	NEG	NEG	NEG	NEG	NEG	NEG	NEG	NEG
06V23749	CC22-MRSA-IV, Barnim/UK-EMRSA-15	POS	POS	POS	NEG	NEG	POS	NEG	NEG	NEG	NEG	NEG	NEG	NEG	NEG	NEG	NEG	NEG	NEG	NEG	NEG	NEG	NEG	NEG	NEG	NEG	NEG	NEG	NEG	NEG	NEG	NEG	NEG	NEG	NEG	NEG	NEG	NEG	NEG	NEG	NEG	NEG	NEG	NEG	NEG	NEG
06V29915	CC22-MRSA-IV, Barnim/UK-EMRSA-15	POS	POS	POS	NEG	NEG	NEG	NEG	NEG	NEG	NEG	NEG	NEG	NEG	NEG	NEG	NEG	NEG	NEG	NEG	NEG	NEG	NEG	NEG	NEG	NEG	NEG	NEG	NEG	NEG	NEG	NEG	NEG	NEG	NEG	NEG	NEG	NEG	NEG	NEG	NEG	NEG	NEG	NEG	NEG	NEG
06V31778	CC22-MRSA-IV, Barnim/UK-EMRSA-15	POS	POS	POS	NEG	NEG	NEG	NEG	NEG	NEG	NEG	NEG	NEG	NEG	NEG	NEG	NEG	NEG	NEG	NEG	NEG	NEG	NEG	NEG	NEG	NEG	NEG	NEG	NEG	NEG	NEG	NEG	NEG	NEG	NEG	NEG	NEG	NEG	NEG	NEG	NEG	NEG	NEG	NEG	NEG	NEG
06V39089	CC22-MRSA-IV, Barnim/UK-EMRSA-15	POS	POS	POS	NEG	NEG	NEG	NEG	NEG	NEG	NEG	NEG	NEG	NEG	NEG	NEG	NEG	NEG	NEG	NEG	NEG	NEG	NEG	NEG	NEG	NEG	NEG	NEG	NEG	NEG	NEG	NEG	NEG	NEG	NEG	NEG	NEG	NEG	NEG	NEG	NEG	NEG	NEG	NEG	NEG	NEG
06V41512	CC22-MRSA-IV, Barnim/UK-EMRSA-15	POS			NEG		POS	NEG	NEG			NEG	NEG	NEG	NEG	NEG	NEG	NEG	NEG	NEG	NEG	NEG		NEG	NEG	NEG																		NEG	NEG	NEG
06V42186	CC22-MRSA-IV, Barnim/UK-EMRSA-15	POS	POS	POS	NEG	NEG	NEG	NEG	NEG	NEG	NEG	NEG	NEG	NEG	NEG	NEG	NEG	NEG	NEG	NEG	NEG	NEG	NEG	NEG	NEG	NEG	NEG	NEG	NEG	NEG	NEG	NEG	NEG	NEG	NEG	NEG	NEG	NEG	NEG	NEG	NEG	NEG	NEG	NEG	NEG	NEG
06V42236	CC22-MRSA-IV, Barnim/UK-EMRSA-15	POS	POS	POS	NEG	NEG	POS	NEG	NEG	NEG	NEG	NEG	NEG	NEG	NEG	NEG	NEG	NEG	NEG	NEG	NEG	NEG	NEG	NEG	NEG	NEG	NEG	NEG	NEG	NEG	NEG	NEG	NEG	NEG	NEG	NEG	NEG	NEG	NEG	NEG	NEG	NEG	NEG	NEG	NEG	NEG
06V43697	CC22-MRSA-IV, Barnim/UK-EMRSA-15	POS	POS	POS	NEG	NEG	POS	NEG	NEG	NEG	NEG	NEG	NEG	NEG	NEG	NEG	NEG	NEG	NEG	NEG	NEG	NEG	NEG	NEG	NEG	NEG	NEG	NEG	NEG	NEG	NEG	NEG	NEG	NEG	NEG	NEG	NEG	NEG	NEG	NEG	NEG	NEG	NEG	NEG	NEG	NEG
06V44153	CC22-MRSA-IV, Barnim/UK-EMRSA-15	POS	POS	POS	NEG	NEG	POS	NEG	NEG	NEG	NEG	NEG	NEG	NEG	NEG	NEG	NEG	NEG	NEG	NEG	NEG	NEG	NEG	NEG	NEG	NEG	NEG	NEG	NEG	NEG	NEG	NEG	NEG	NEG	NEG	NEG	NEG	NEG	NEG	NEG	NEG	NEG	NEG	NEG	NEG	NEG
06V45679	CC22-MRSA-IV, Barnim/UK-EMRSA-15	POS	POS	POS	NEG	NEG	POS	NEG	NEG	NEG	NEG	NEG	NEG	NEG	NEG	NEG	NEG	NEG	NEG	NEG	NEG	NEG	NEG	NEG	NEG	NEG	NEG	NEG	NEG	NEG	NEG	NEG	NEG	NEG	NEG	NEG	NEG	NEG	NEG	NEG	NEG	NEG	NEG	NEG	NEG	NEG
06V45831	CC22-MRSA-IV, Barnim/UK-EMRSA-15	POS	POS	POS	NEG	NEG	NEG	NEG	NEG	NEG	NEG	NEG	NEG	NEG	NEG	NEG	NEG	NEG	NEG	NEG	NEG	NEG	NEG	NEG	NEG	NEG	NEG	NEG	NEG	NEG	NEG	NEG	NEG	NEG	NEG	NEG	NEG	NEG	NEG	NEG	NEG	NEG	NEG	NEG	NEG	NEG
06V46314	CC22-MRSA-IV, Barnim/UK-EMRSA-15	POS	POS	POS	NEG	NEG	NEG	NEG	NEG	NEG	NEG	NEG	NEG	NEG	NEG	NEG	NEG	NEG	NEG	NEG	NEG	NEG	NEG	NEG	NEG	NEG	NEG	NEG	NEG	NEG	NEG	NEG	NEG	NEG	NEG	NEG	NEG	NEG	NEG	NEG	NEG	NEG	NEG	NEG	NEG	NEG
06V34729	CC30-MRSA-IV [PVL+], Southwest Pacific Clone	POS	POS	POS	NEG		NEG	NEG	NEG	NEG	NEG	NEG	NEG	NEG	NEG	NEG	NEG	NEG	NEG	NEG	NEG	NEG	NEG	NEG	NEG	NEG	NEG	NEG	NEG	NEG	NEG	NEG	NEG	POS	NEG	NEG	NEG	NEG	NEG	NEG	NEG	NEG	POS	NEG	NEG	NEG
06B09900	CC45-MRSA-IV, Berlin EMRSA	POS	POS	POS	NEG	NEG	NEG	NEG	NEG	NEG	NEG	NEG	NEG	NEG	NEG	NEG	NEG	NEG	POS	POS	NEG	NEG	NEG	NEG	NEG	NEG	NEG	NEG	NEG	NEG	NEG	NEG	NEG	NEG	NEG	NEG	NEG	NEG	NEG	NEG	NEG	NEG	POS	NEG	NEG	NEG
06V00922	CC45-MRSA-IV, Berlin EMRSA	POS	POS	POS	NEG	NEG	NEG	NEG	NEG	NEG	NEG	NEG	NEG	NEG	NEG	NEG	NEG	NEG	POS	POS	NEG	NEG	NEG	NEG	NEG	NEG	NEG	NEG	NEG	NEG	NEG	NEG	NEG	NEG	NEG	NEG	NEG	NEG	NEG	NEG	NEG	NEG	POS	NEG	NEG	NEG
06V09551	CC45-MRSA-IV, Berlin EMRSA	POS	POS	POS	NEG	NEG	NEG	NEG	NEG	NEG	NEG	NEG	NEG	NEG	NEG	NEG	NEG	NEG	NEG	NEG	NEG	NEG	NEG	NEG	NEG	NEG	NEG	NEG	NEG	NEG	NEG	NEG	NEG	NEG	NEG	NEG	NEG	NEG	NEG	NEG	NEG	NEG	POS	NEG	NEG	NEG
06V10719	CC45-MRSA-IV, Berlin EMRSA	POS	POS	POS	NEG	NEG	NEG	NEG	NEG	NEG	NEG	NEG	NEG	NEG	NEG	NEG	NEG	NEG	POS	POS	NEG	NEG	NEG	NEG	NEG	NEG	NEG	NEG	NEG	NEG	NEG	NEG	NEG	NEG	NEG	NEG	NEG	NEG	NEG	NEG	NEG	NEG	POS	NEG	NEG	NEG
06V11090	CC45-MRSA-IV, Berlin EMRSA	POS	POS	POS	NEG	NEG	NEG	NEG	NEG	NEG	NEG	NEG	NEG	NEG	NEG	NEG	NEG	NEG	POS	POS	NEG	NEG	NEG	NEG	NEG	NEG	NEG	NEG	NEG	NEG	NEG	NEG	NEG	NEG	NEG	NEG	NEG	NEG	NEG	NEG	NEG	NEG	AMB	NEG	NEG	NEG
06V14383	CC45-MRSA-IV, Berlin EMRSA	POS	POS	POS	NEG	NEG	NEG	NEG	NEG	NEG	NEG	NEG	NEG	NEG	NEG	NEG	NEG	NEG	POS	POS	NEG	NEG	NEG	NEG	NEG	NEG	NEG	NEG	NEG	NEG	NEG	NEG	NEG	NEG	NEG	NEG	NEG	NEG	NEG	NEG	NEG	NEG	POS	NEG	NEG	NEG
06V21475	CC45-MRSA-IV, Berlin EMRSA	POS	POS	POS	NEG	NEG	NEG	NEG	NEG	NEG	NEG	NEG	NEG	NEG	NEG	NEG	NEG	NEG	POS	POS	NEG	NEG	NEG	NEG	NEG	NEG	NEG	NEG	NEG	NEG	NEG	NEG	NEG	NEG	NEG	NEG	NEG	NEG	NEG	NEG	NEG	NEG	POS	NEG	NEG	NEG
06V24231	CC45-MRSA-IV, Berlin EMRSA	POS	POS	POS	NEG	NEG	NEG	NEG	NEG	NEG	NEG	NEG	NEG	NEG	NEG	NEG	NEG	NEG	POS	POS	NEG	NEG	NEG	NEG	NEG	NEG	NEG	NEG	NEG	NEG	NEG	NEG	NEG	NEG	NEG	NEG	NEG	NEG	NEG	NEG	NEG	NEG	POS	NEG	NEG	NEG
06V32992	CC45-MRSA-IV, Berlin EMRSA	POS	POS	POS	NEG	NEG	NEG	NEG	NEG	NEG	NEG	NEG	NEG	NEG	NEG	NEG	NEG	NEG	POS	POS	NEG	NEG	NEG	NEG	NEG	NEG	NEG	NEG	NEG	NEG	NEG	NEG	NEG	NEG	NEG	NEG	NEG	NEG	NEG	NEG	NEG	NEG	POS	NEG	NEG	NEG
06V38753	CC45-MRSA-IV, Berlin EMRSA	POS	POS	POS	NEG	NEG	NEG	NEG	NEG	NEG	NEG	NEG	NEG	NEG	NEG	NEG	NEG	NEG	POS	POS	NEG	NEG	NEG	NEG	NEG	NEG	NEG	NEG	NEG	NEG	NEG	NEG	NEG	NEG	NEG	NEG	NEG	NEG	NEG	NEG	NEG	NEG	POS	NEG	NEG	NEG
06V39848	CC45-MRSA-IV, Berlin EMRSA	POS			NEG		NEG	NEG	NEG			NEG	NEG	NEG	NEG	NEG	NEG	NEG	POS	POS	NEG	NEG		NEG	NEG	NEG																		NEG	NEG	NEG
06V42259	CC45-MRSA-IV, Berlin EMRSA	POS	POS	POS	NEG	NEG	NEG	NEG	NEG	NEG	NEG	NEG	NEG	NEG	NEG	NEG	NEG	NEG	POS	POS	NEG	NEG	NEG	NEG	NEG	NEG	NEG	NEG	NEG	NEG	NEG	NEG	NEG	NEG	NEG	NEG	NEG	NEG	NEG	NEG	NEG	NEG	POS	NEG	NEG	NEG
06V33960	CC80-MRSA-IV [PVL+], European caMRSA Clone	POS			NEG		NEG	NEG	NEG			NEG	NEG	NEG	NEG	NEG	NEG	NEG	POS	POS	NEG	POS		NEG	POS	NEG																		NEG	NEG	NEG

ISOLATE:	STRAIN	VIRULENCE : TOX.SCHOCK.TOXIN			VIRULENCE : ENTEROTOXINS																					VIRULENCE : HLG AND LEUKOCIDINS												
		toxic shock syndrome toxin 1			enterotoxin A	enterotoxin A, allele from strain 320E	enterotoxin A, allele from strain N315 = entero-toxin P	enterotoxin B	enterotoxin C	enterotoxin D	enterotoxin E	enterotoxin G	enterotoxin H	enterotoxin I	enterotoxin J	enterotoxin K	enterotoxin L	enterotoxin M	enterotoxin N		enterotoxin O	enterotoxin Q	enterotoxin R	enterotoxin U	enterotoxin-like protein ORF CM14	haemolysin gamma / leukocidin, component B	haemolysin gamma / leukocidin, component C		haemolysin gamma, component A	Panton Valentine leukocidin F component	Panton Valentine leukocidin S component	F component from hypothetical leukocidin from ruminants	S component from hypothetical leukocidin from ruminants	leukocidin D component	leukocidin E component	leukocidin/ haemolysin toxin family protein	leukocidin/haemolysin toxin family protein	
		tst1 (consensus)	tst1 ("human" allele)	tst1 ("bovine" allele)	sea	sea (320E)	sea (N315) / sep	seb	sec	sed	sef	seg	seh	sei	sej	sek	sel	selm	seln (consensus)	seln (other than RF122)	selo	seq	ser	selu	ORF CM14	lukF	lukS	lukS (ST22+ST45)	hlgA	lukF-PV	lukS-PV	lukF-PV (P83)	lukM	lukD	lukE	lukX	lukY	lukY (ST30+ST45)
**2005**																																						
05B01179	CC5/ST228-MRSA-I, South German EMRSA	NEG	NEG	NEG	POS	NEG	NEG	NEG	NEG	NEG	NEG	POS	NEG	POS	NEG	NEG	NEG	POS	AMB	POS	POS	NEG	NEG	POS	NEG	POS	POS	AMB	POS	NEG	NEG	NEG	NEG	POS	POS	NEG	POS	NEG
05B02687	CC5/ST228-MRSA-I, South German EMRSA	NEG	NEG	NEG	POS	NEG	NEG	NEG	NEG	NEG	NEG	POS	NEG	POS	NEG	NEG	NEG	POS	POS	POS	POS	NEG	NEG	POS	NEG	POS	POS	POS	POS	NEG	NEG	NEG	NEG	POS	POS	NEG	POS	NEG
05V08483	CC5/ST228-MRSA-I, South German EMRSA	NEG	NEG	NEG	POS	NEG	NEG	NEG	NEG	NEG	NEG	NEG	NEG	NEG	NEG	NEG	NEG	NEG	NEG	NEG	POS	NEG	NEG	NEG	NEG	POS	POS	NEG	POS	NEG	NEG	NEG	NEG	NEG	NEG	POS	POS	NEG
05V19001	CC5/ST228-MRSA-I, South German EMRSA	NEG	NEG	NEG	POS	AMB	NEG	NEG	NEG	NEG	NEG	NEG	NEG	NEG	NEG	NEG	NEG	NEG	NEG	NEG	POS	NEG	NEG	NEG	NEG	POS	POS	AMB	POS	NEG	NEG	NEG	NEG	NEG	NEG	POS	POS	NEG
05B04626	CC5-MRSA-II, Rhine-Hesse EMRSA/UK-EMRSA-3	NEG	NEG	NEG	NEG	NEG	POS	NEG	NEG	POS	NEG	AMB	NEG	POS	POS	NEG	NEG	POS	NEG	POS	AMB	NEG	AMB	POS	NEG	POS	AMB	NEG	POS	NEG	NEG	NEG	NEG	POS	POS	NEG	POS	NEG
05B07903	CC5-MRSA-II, Rhine-Hesse EMRSA/UK-EMRSA-3	NEG	NEG	NEG	NEG	NEG	POS	NEG	NEG	NEG	NEG	POS	NEG	POS	NEG	NEG	NEG	POS	POS	POS	POS	AMB	NEG	POS	NEG	POS	POS	NEG	POS	NEG	NEG	NEG	NEG	POS	POS	POS	POS	NEG
05U00062	CC5-MRSA-II, Rhine-Hesse EMRSA/UK-EMRSA-3	NEG	NEG	NEG	NEG	NEG	POS	NEG	NEG	POS	NEG	POS	NEG	POS	POS	NEG	NEG	POS	POS	POS	POS	NEG	POS	POS	NEG	POS	POS	NEG	POS	NEG	NEG	NEG	NEG	POS	POS	POS	POS	NEG
05U03117	CC5-MRSA-II, Rhine-Hesse EMRSA/UK-EMRSA-3	NEG	NEG	NEG	NEG	NEG	POS	NEG	NEG	POS	NEG	POS	NEG	POS	POS	NEG	NEG	POS	POS	POS	POS	NEG	POS	POS	NEG	POS	POS	NEG	POS	NEG	NEG	NEG	NEG	POS	POS	POS	AMB	NEG
05U10392	CC5-MRSA-II, Rhine-Hesse EMRSA/UK-EMRSA-3	NEG	NEG	NEG	NEG	NEG	NEG	NEG	NEG	POS	NEG	POS	NEG	POS	POS	NEG	NEG	POS	POS	POS	POS	NEG	AMB	POS	NEG	POS	POS	NEG	POS	NEG	NEG	NEG	NEG	POS	AMB	AMB	POS	NEG
05V03312	CC5-MRSA-II, Rhine-Hesse EMRSA/UK-EMRSA-3	NEG	NEG	NEG	NEG	NEG	POS	NEG	NEG	POS	NEG	POS	NEG	POS	POS	NEG	NEG	POS	POS	POS	POS	NEG	POS	POS	NEG	POS	POS	POS	POS	NEG	NEG	AMB	NEG	POS	POS	POS	POS	NEG
05V08095	CC5-MRSA-II, Rhine-Hesse EMRSA/UK-EMRSA-3	NEG	NEG	NEG	NEG	NEG	POS	NEG	NEG	POS	NEG	POS	NEG	POS	POS	NEG	NEG	POS	POS	POS	POS	NEG	POS	POS	NEG	POS	POS	AMB	POS	NEG	NEG	NEG	NEG	POS	POS	POS	POS	NEG
05V09015	CC5-MRSA-II, Rhine-Hesse EMRSA/UK-EMRSA-3	NEG	NEG	NEG	NEG	NEG	POS	NEG	NEG	POS	NEG	POS	NEG	POS	POS	NEG	NEG	POS	POS	POS	POS	NEG	POS	POS	NEG	POS	POS	AMB	POS	NEG	NEG	NEG	NEG	POS	POS	AMB	POS	NEG
05V09326	CC5-MRSA-II, Rhine-Hesse EMRSA/UK-EMRSA-3	NEG	NEG	NEG	NEG	NEG	POS	NEG	NEG	POS	NEG	POS	NEG	POS	POS	NEG	NEG	POS	POS	POS	POS	NEG	POS	POS	NEG	POS	POS	POS	POS	NEG	NEG	NEG	NEG	POS	POS	POS	POS	NEG
05V10265	CC5-MRSA-II, Rhine-Hesse EMRSA/UK-EMRSA-3	NEG	NEG	NEG	NEG	NEG	POS	NEG	NEG	NEG	NEG	POS	NEG	POS	NEG	NEG	NEG	POS	POS	POS	POS	NEG	NEG	POS	NEG	POS	POS	NEG	POS	NEG	NEG	NEG	NEG	POS	POS	POS	POS	NEG
05V13052	CC5-MRSA-II, Rhine-Hesse EMRSA/UK-EMRSA-3	NEG	NEG	NEG	NEG	AMB	POS	NEG	NEG	POS	NEG	POS	NEG	POS	POS	NEG	NEG	POS	POS	POS	POS	NEG	POS	POS	NEG	POS	POS	NEG	POS	NEG	NEG	AMB	NEG	POS	POS	POS	POS	NEG
05V13470	CC5-MRSA-II, Rhine-Hesse EMRSA/UK-EMRSA-3	NEG	NEG	NEG	NEG	NEG	POS	NEG	NEG	POS	NEG	POS	NEG	POS	POS	NEG	NEG	POS	POS	POS	POS	NEG	POS	POS	NEG	POS	POS	AMB	POS	NEG	NEG	NEG	NEG	POS	POS	POS	POS	NEG
05V13891	CC5-MRSA-II, Rhine-Hesse EMRSA/UK-EMRSA-3	NEG	NEG	NEG	NEG	NEG	POS	NEG	NEG	NEG	NEG	POS	NEG	POS	NEG	NEG	NEG	POS	POS	AMB	AMB	NEG	NEG	POS	NEG	POS	POS	NEG	AMB	NEG	NEG	NEG	NEG	POS	POS	POS	AMB	NEG
05V15616	CC5-MRSA-II, Rhine-Hesse EMRSA/UK-EMRSA-3		NEG	NEG	NEG	NEG	POS	NEG	NEG	POS	NEG	POS	NEG	POS	POS		NEG	POS		POS	POS		POS			POS	POS		POS	NEG	NEG	NEG	NEG	POS	POS	POS	POS	NEG
05V16845	CC5-MRSA-II, Rhine-Hesse EMRSA/UK-EMRSA-3	NEG	NEG	NEG	NEG	NEG	POS	NEG	NEG	POS	NEG	POS	NEG	POS	POS	NEG	NEG	POS	POS	POS	POS	NEG	POS	POS	NEG	POS	POS	AMB	POS	NEG	NEG	NEG	NEG	POS	POS	POS	POS	NEG
05V17541	CC5-MRSA-II, Rhine-Hesse EMRSA/UK-EMRSA-3	NEG	NEG	NEG	NEG	NEG	POS	NEG	NEG	NEG	NEG	POS	NEG	POS	NEG	NEG	NEG	POS	POS	AMB	AMB	NEG	NEG	POS	NEG	POS	POS	NEG	AMB	NEG	NEG	NEG	NEG	POS	POS	POS	POS	NEG
05V19105	CC5-MRSA-II, Rhine-Hesse EMRSA/UK-EMRSA-3		NEG	NEG	NEG	NEG	POS	NEG	NEG	POS	NEG	POS	NEG	POS	POS		NEG	POS		POS	POS		POS			POS	POS		POS	NEG	NEG	NEG	NEG	POS	POS	POS	POS	NEG
05V22148	CC5-MRSA-II, Rhine-Hesse EMRSA/UK-EMRSA-3	NEG	NEG	NEG	NEG	NEG	POS	NEG	NEG	POS	NEG	POS	NEG	POS	POS	NEG	NEG	POS	POS	POS	POS	NEG	POS	POS	NEG	POS	POS	AMB	POS	NEG	NEG	NEG	NEG	POS	POS	POS	POS	NEG
05V33740	CC5-MRSA-II, Rhine-Hesse EMRSA/UK-EMRSA-3	NEG	NEG	NEG	NEG	NEG	POS	NEG	NEG	POS	NEG	POS	NEG	POS	POS	NEG	NEG	POS	POS	POS	POS	NEG	NEG	POS	NEG	POS	POS	NEG	POS	NEG	NEG	NEG	NEG	POS	POS	AMB	POS	NEG
05V38425	CC5-MRSA-II, Rhine-Hesse EMRSA/UK-EMRSA-3	NEG	NEG	NEG	NEG	NEG	POS	NEG	NEG	POS	NEG	POS	NEG	POS	POS	NEG	NEG	POS	POS	POS	POS	NEG	AMB	POS	NEG	POS	POS	NEG	POS	NEG	NEG	NEG	NEG	POS	AMB	POS	POS	NEG
05V39987	CC5-MRSA-II, Rhine-Hesse EMRSA/UK-EMRSA-3	NEG	NEG	NEG	NEG	NEG	POS	NEG	NEG	NEG	NEG	POS	NEG	POS	NEG	NEG	NEG	POS	POS	POS	POS	NEG	NEG	POS	NEG	POS	POS	NEG	POS	NEG	NEG	NEG	NEG	POS	POS	POS	POS	NEG
05F00089	CC5-MRSA-IV, Paediatric clone	NEG	NEG	NEG	NEG	NEG	POS	NEG	NEG	POS	NEG	POS	NEG	POS	POS	NEG	NEG	POS	POS	POS	POS	NEG	POS	POS	NEG	POS	POS	NEG	POS	NEG	NEG	NEG	NEG	POS	POS	POS	POS	NEG
05V01416	CC5-MRSA-IV, Paediatric clone	NEG	NEG	NEG	NEG	NEG	POS	NEG	NEG	POS	NEG	POS	NEG	POS	POS	NEG	NEG	POS	POS	NEG	NEG	NEG	AMB	POS	NEG	POS	POS	NEG	NEG	NEG	NEG	NEG	NEG	POS	AMB	AMB	NEG	NEG
05V16386	CC8-MRSA-IV, USA300	NEG	NEG	NEG	NEG	NEG	NEG	NEG	NEG	NEG	NEG	NEG	NEG	NEG	NEG	POS	NEG	NEG	NEG	NEG	NEG	POS	NEG	NEG	NEG	POS	POS	NEG	POS	POS	POS	NEG	NEG	POS	POS	NEG	POS	NEG
05V04920	CC8-MRSA-IV, USA500	NEG	NEG	NEG	POS	NEG	NEG	POS	NEG	NEG	NEG	NEG	NEG	NEG	NEG	POS	NEG	NEG	NEG	NEG	NEG	POS	NEG	NEG	NEG	POS	POS	NEG	POS	NEG	NEG	NEG	NEG	POS	POS	POS	POS	NEG
05V21159	CC8-MRSA-IV, USA500	NEG	NEG	NEG	NEG	NEG	NEG	POS	NEG	NEG	NEG	NEG	NEG	NEG	NEG	POS	NEG	NEG	NEG	NEG	NEG	POS	NEG	NEG	NEG	POS	POS	NEG	POS	NEG	NEG	NEG	NEG	POS	POS	NEG	POS	NEG
05B00867	CC22-MRSA-IV, Barnim/UK-EMRSA-15	NEG	NEG	NEG	NEG	NEG	NEG	NEG	NEG	NEG	NEG	POS	NEG	POS	NEG	NEG	NEG	POS	AMB	POS	POS	NEG	NEG	POS	NEG	NEG	NEG	POS	NEG	NEG	NEG	NEG	NEG	NEG	NEG	AMB	POS	NEG
05V03174	CC22-MRSA-IV, Barnim/UK-EMRSA-15	NEG	NEG	NEG	NEG	NEG	NEG	NEG	NEG	NEG	NEG	POS	NEG	POS	NEG	NEG	NEG	POS	POS	POS	POS	NEG	NEG	POS	NEG	POS	POS	POS	POS	NEG	NEG	NEG	NEG	NEG	NEG	POS	POS	NEG
05V07331	CC22-MRSA-IV, Barnim/UK-EMRSA-15	NEG	NEG	NEG	NEG	NEG	NEG	NEG	NEG	NEG	NEG	POS	NEG	POS	NEG	NEG	NEG	POS	POS	POS	POS	NEG	NEG	POS	NEG	POS	AMB	POS	POS	NEG	NEG	NEG	NEG	NEG	NEG	POS	POS	NEG
05V07371	CC22-MRSA-IV, Barnim/UK-EMRSA-15	NEG	NEG	NEG	NEG	NEG	NEG	NEG	POS	NEG	NEG	POS	NEG	POS	NEG	NEG	POS	POS	POS	POS	POS	NEG	NEG	POS	NEG	AMB	AMB	POS	POS	NEG	NEG	NEG	NEG	NEG	NEG	POS	POS	NEG
05V08145	CC22-MRSA-IV, Barnim/UK-EMRSA-15	NEG	NEG	NEG	NEG	NEG	NEG	NEG	NEG	NEG	NEG	POS	NEG	POS	NEG	NEG	NEG	POS	POS	POS	POS	NEG	NEG	POS	NEG	NEG	NEG	POS	NEG	NEG	NEG	NEG	NEG	NEG	NEG	POS	POS	NEG
05V08595	CC22-MRSA-IV, Barnim/UK-EMRSA-15	NEG	NEG	NEG	NEG	NEG	NEG	NEG	NEG	NEG	NEG	POS	NEG	POS	NEG	NEG	NEG	POS	POS	POS	POS	NEG	NEG	POS	NEG	POS	AMB	POS	POS	NEG	NEG	NEG	NEG	NEG	NEG	POS	POS	NEG
05V12803	CC22-MRSA-IV, Barnim/UK-EMRSA-15	NEG	NEG	NEG	NEG	NEG	NEG	NEG	NEG	NEG	NEG	POS	NEG	POS	NEG	NEG	NEG	POS	POS	POS	POS	NEG	NEG	POS	NEG	POS	AMB	POS	POS	NEG	NEG	NEG	NEG	NEG	NEG	POS	POS	NEG
05V14596	CC22-MRSA-IV, Barnim/UK-EMRSA-15	NEG	NEG	NEG	NEG	NEG	NEG	NEG	NEG	NEG	NEG	POS	NEG	POS	NEG	NEG	NEG	POS	POS	POS	POS	NEG	NEG	POS	NEG	NEG	NEG	POS	NEG	NEG	NEG	NEG	NEG	NEG	NEG	POS	POS	NEG
05V16691	CC22-MRSA-IV, Barnim/UK-EMRSA-15		NEG	NEG	NEG	NEG	NEG	NEG	NEG	NEG	NEG	POS	NEG	POS	NEG		NEG	POS		POS	POS		NEG			POS	NEG		POS	NEG	NEG	NEG	NEG	NEG	NEG	POS	POS	NEG
05V18550	CC22-MRSA-IV, Barnim/UK-EMRSA-15	NEG	NEG	NEG	NEG	NEG	NEG	NEG	NEG	NEG	NEG	POS	NEG	POS	NEG	NEG	NEG	POS	POS	POS	POS	NEG	NEG	POS	NEG	POS	POS	POS	POS	NEG	NEG	NEG	NEG	NEG	NEG	POS	POS	NEG
05V19034	CC22-MRSA-IV, Barnim/UK-EMRSA-15	NEG	NEG	NEG	NEG	NEG	NEG	NEG	NEG	NEG	NEG	POS	NEG	POS	NEG	NEG	NEG	POS	POS	POS	POS	NEG	NEG	POS	NEG	POS	POS	POS	POS	NEG	NEG	NEG	NEG	NEG	NEG	POS	POS	NEG
05V19333	CC22-MRSA-IV, Barnim/UK-EMRSA-15		NEG	NEG	NEG	NEG	NEG	NEG	NEG	NEG	NEG	POS	NEG	POS	NEG		NEG	POS		POS	POS		NEG			NEG	NEG		NEG	NEG	NEG	NEG	NEG	NEG	NEG	NEG	POS	NEG
05V19701	CC22-MRSA-IV, Barnim/UK-EMRSA-15	NEG	NEG	NEG	NEG	NEG	NEG	NEG	NEG	NEG	NEG	POS	NEG	POS	NEG	NEG	NEG	POS	POS	POS	POS	NEG	NEG	POS	NEG	AMB	NEG	POS	NEG	NEG	NEG	NEG	NEG	NEG	NEG	POS	POS	NEG
05V20072	CC22-MRSA-IV, Barnim/UK-EMRSA-15	NEG	NEG	NEG	NEG	NEG	NEG	NEG	POS	NEG	NEG	POS	NEG	POS	NEG	NEG	POS	POS	POS	POS	POS	NEG	NEG	POS	NEG	AMB	AMB	POS	POS	NEG	NEG	NEG	NEG	NEG	NEG	POS	POS	NEG
05V24930	CC22-MRSA-IV, Barnim/UK-EMRSA-15	NEG	NEG	NEG	NEG	NEG	NEG	NEG	NEG	NEG	NEG	POS	NEG	POS	NEG	NEG	NEG	POS	POS	POS	POS	NEG	NEG	POS	NEG	POS	POS	POS	POS	NEG	NEG	NEG	NEG	NEG	NEG	POS	POS	NEG
05V31461	CC22-MRSA-IV, Barnim/UK-EMRSA-15	NEG	NEG	NEG	NEG	NEG	NEG	NEG	NEG	NEG	NEG	POS	NEG	POS	NEG	NEG	NEG	POS	POS	POS	POS	NEG	NEG	POS	NEG	POS	POS	POS	POS	NEG	NEG	NEG	NEG	NEG	NEG	POS	POS	NEG
05B05671	CC45-MRSA-IV, Berlin EMRSA	NEG	NEG	NEG	NEG	NEG	NEG	NEG	NEG	NEG	NEG	NEG	NEG	POS	NEG	NEG	NEG	POS	POS	POS	POS	NEG	NEG	POS	NEG	POS	AMB	POS	POS	NEG	NEG	NEG	NEG	NEG	NEG	POS	NEG	POS
05V01452	CC45-MRSA-IV, Berlin EMRSA	NEG	NEG	NEG	NEG	NEG	NEG	NEG	NEG	NEG	NEG	POS	NEG	POS	NEG	NEG	NEG	POS	POS	POS	POS	NEG	NEG	POS	NEG	POS	NEG	POS	POS	NEG	NEG	AMB	NEG	NEG	NEG	POS	NEG	POS
05V01617	CC45-MRSA-IV, Berlin EMRSA	NEG	AMB	NEG	NEG	NEG	NEG	NEG	NEG	NEG	NEG	POS	NEG	POS	NEG	NEG	NEG	POS	POS	POS	POS	NEG	NEG	POS	NEG	POS	POS	POS	POS	NEG	NEG	NEG	NEG	NEG	NEG	POS	NEG	POS
05V16986	CC45-MRSA-IV, Berlin EMRSA	NEG	NEG	NEG	NEG	NEG	NEG	NEG	NEG	NEG	NEG	POS	NEG	POS	NEG	NEG	NEG	POS	POS	AMB	AMB	NEG	NEG	POS	NEG	POS	NEG	POS	NEG	NEG	NEG	NEG	NEG	NEG	NEG	POS	NEG	POS
05V18357	CC45-MRSA-IV, Berlin EMRSA	NEG	NEG	NEG	NEG	NEG	NEG	NEG	NEG	NEG	NEG	NEG	NEG	POS	NEG	NEG	NEG	POS	POS	POS	POS	NEG	NEG	POS	NEG	POS	POS	POS	POS	NEG	NEG	NEG	NEG	NEG	NEG	POS	NEG	POS
05V23111	CC45-MRSA-IV, Berlin EMRSA	NEG	NEG	NEG	NEG	NEG	NEG	NEG	NEG	NEG	NEG	POS	NEG	POS	NEG	NEG	NEG	POS	POS	POS	POS	NEG	NEG	POS	NEG	POS	POS	POS	POS	NEG	NEG	NEG	NEG	NEG	NEG	POS	NEG	POS
05V23414	CC45-MRSA-IV, Berlin EMRSA		NEG	NEG	NEG	NEG	NEG	NEG	NEG	NEG	NEG	POS	NEG	POS	NEG		NEG	POS		POS	POS		NEG			POS	NEG		POS	NEG	NEG	NEG	NEG	NEG	NEG	POS	NEG	POS
05V23858	CC45-MRSA-IV, Berlin EMRSA	NEG	NEG	NEG	NEG	NEG	NEG	NEG	NEG	NEG	NEG	NEG	NEG	POS	NEG	NEG	NEG	POS	POS	POS	POS	NEG	NEG	POS	NEG	POS	NEG	POS	POS	NEG	NEG	NEG	NEG	NEG	NEG	POS	NEG	POS
05V26241	CC45-MRSA-IV, Berlin EMRSA	NEG	NEG	NEG	NEG	NEG	NEG	NEG	NEG	NEG	NEG	POS	NEG	POS	NEG	NEG	NEG	POS	POS	POS	POS	NEG	NEG	POS	NEG	POS	AMB	POS	POS	NEG	NEG	NEG	NEG	NEG	NEG	POS	NEG	POS
05V28399	CC45-MRSA-IV, Berlin EMRSA	NEG	NEG	NEG	NEG	NEG	NEG	NEG	NEG	NEG	NEG	POS	NEG	POS	NEG	NEG	NEG	POS	POS	POS	POS	NEG	NEG	POS	NEG	POS	POS	POS	POS	NEG	NEG	NEG	NEG	NEG	NEG	POS	NEG	POS
05V32647	CC45-MRSA-IV, Berlin EMRSA		NEG	NEG	NEG	NEG	NEG	NEG	NEG	NEG	NEG	POS	NEG	POS	NEG		NEG	POS		POS	NEG		NEG			POS	NEG		POS	NEG	NEG	NEG	NEG	NEG	NEG	NEG	NEG	POS
05V37969	CC45-MRSA-IV, Berlin EMRSA	NEG	NEG	NEG	NEG	NEG	NEG	NEG	NEG	NEG	NEG	NEG	NEG	POS	NEG	NEG	NEG	POS	POS	POS	POS	NEG	NEG	POS	NEG	POS	NEG	POS	POS	NEG	NEG	NEG	NEG	NEG	NEG	POS	NEG	POS
05U10018	CC398-MRSA-V	NEG	NEG	NEG	NEG	NEG	NEG	NEG	NEG	NEG	NEG	NEG	NEG	NEG	NEG	NEG	NEG	NEG	NEG	NEG	NEG	NEG	NEG	NEG	NEG	POS	POS	NEG	POS	NEG	NEG	NEG	NEG	NEG	NEG	NEG	POS	NEG
**2006**																																						
06V36353	CC1-MRSA-IV, WA MRSA-1/57	NEG	NEG	NEG	POS	NEG	NEG	NEG	NEG	NEG	NEG	NEG	POS	NEG	NEG	POS	NEG	NEG	NEG	NEG	NEG	POS	NEG	NEG	NEG	POS	POS	NEG	POS	NEG	NEG	NEG	NEG	POS	POS	NEG	POS	NEG
06V40202	CC5/ST228-MRSA-I, South German EMRSA		NEG	NEG	POS	NEG	NEG	NEG	NEG	NEG	NEG	POS	NEG	POS	NEG		NEG	POS		POS	POS		NEG			POS	POS		POS	NEG	NEG	NEG	NEG	POS	POS	POS	POS	NEG
06V41266	CC5/ST228-MRSA-I, South German EMRSA	NEG	NEG	NEG	POS	NEG	NEG	NEG	NEG	NEG	NEG	NEG	NEG	NEG	NEG	NEG	NEG	NEG	NEG	NEG	POS	NEG	NEG	NEG	NEG	POS	POS	NEG	POS	NEG	NEG	NEG	NEG	NEG	NEG	AMB	POS	NEG
06B09097	CC5-MRSA-II, Rhine-Hesse EMRSA/UK-EMRSA-3		NEG	NEG	NEG	NEG	POS	NEG	NEG	NEG	NEG	POS	NEG	POS	NEG		NEG	POS		POS	POS		NEG			POS	POS		POS	NEG	NEG	NEG	NEG	POS	POS	NEG	POS	NEG
06F00244	CC5-MRSA-II, Rhine-Hesse EMRSA/UK-EMRSA-3	NEG	NEG	NEG	NEG	NEG	POS	NEG	NEG	POS	NEG	POS	NEG	POS	POS	NEG	NEG	POS	POS	POS	POS	NEG	POS	POS	NEG	POS	POS	AMB	POS	NEG	NEG	NEG	NEG	POS	POS	POS	POS	NEG
06V03913	CC5-MRSA-II, Rhine-Hesse EMRSA/UK-EMRSA-3	NEG	NEG	NEG	NEG	NEG	POS	NEG	NEG	POS	NEG	POS	NEG	POS	POS	NEG	NEG	POS	POS	POS	POS	NEG	POS	POS	NEG	POS	POS	POS	POS	NEG	NEG	NEG	NEG	POS	POS	POS	POS	NEG
06V04337	CC5-MRSA-II, Rhine-Hesse EMRSA/UK-EMRSA-3	NEG	NEG	NEG	NEG	NEG	POS	NEG	NEG	POS	NEG	POS	NEG	POS	POS	NEG	NEG	POS	POS	POS	POS	NEG	POS	POS	NEG	POS	POS	POS	POS	NEG	NEG	NEG	NEG	POS	POS	POS	POS	NEG
06V06785	CC5-MRSA-II, Rhine-Hesse EMRSA/UK-EMRSA-3	NEG	NEG	NEG	NEG	NEG	POS	NEG	NEG	POS	NEG	POS	NEG	POS	POS	NEG	NEG	POS	POS	POS	POS	NEG	POS	POS	NEG	POS	POS	POS	POS	NEG	NEG	NEG	NEG	POS	POS	POS	POS	NEG
06V12258	CC5-MRSA-II, Rhine-Hesse EMRSA/UK-EMRSA-3	NEG	NEG	NEG	NEG	NEG	POS	NEG	NEG	POS	NEG	POS	NEG	POS	POS	NEG	NEG	POS	POS	POS	POS	NEG	POS	POS	NEG	POS	POS	POS	POS	NEG	NEG	NEG	NEG	POS	POS	POS	POS	NEG
06V20553	CC5-MRSA-II, Rhine-Hesse EMRSA/UK-EMRSA-3	NEG	NEG	NEG	NEG	NEG	POS	NEG	NEG	POS	NEG	POS	NEG	POS	POS	NEG	NEG	POS	POS	POS	POS	NEG	POS	POS	NEG	POS	POS	POS	POS	NEG	NEG	NEG	NEG	POS	POS	POS	POS	NEG
06V22564	CC5-MRSA-II, Rhine-Hesse EMRSA/UK-EMRSA-3		NEG	NEG	NEG	NEG	POS	NEG	NEG	POS	NEG	POS	NEG	POS	POS		NEG	POS		POS	POS		POS			POS	POS		POS	NEG	NEG	NEG	NEG	POS	POS	POS	POS	NEG
06V23750	CC5-MRSA-II, Rhine-Hesse EMRSA/UK-EMRSA-3	NEG	NEG	NEG	NEG	NEG	POS	NEG	NEG	POS	NEG	POS	NEG	POS	POS	NEG	NEG	POS	POS	POS	POS	NEG	POS	POS	NEG	POS	POS	POS	POS	NEG	NEG	NEG	NEG	POS	POS	POS	POS	NEG
06V27332	CC5-MRSA-II, Rhine-Hesse EMRSA/UK-EMRSA-3	NEG	NEG	NEG	NEG	NEG	POS	NEG	NEG	POS	NEG	POS	NEG	POS	POS	NEG	NEG	POS	POS	POS	POS	NEG	POS	POS	NEG	POS	POS	AMB	POS	NEG	NEG	NEG	NEG	POS	POS	POS	POS	NEG
06V29484	CC5-MRSA-II, Rhine-Hesse EMRSA/UK-EMRSA-3	NEG	NEG	NEG	NEG	NEG	POS	NEG	NEG	POS	NEG	POS	NEG	POS	POS	NEG	NEG	POS	POS	POS	POS	NEG	POS	POS	NEG	POS	POS	AMB	POS	NEG	NEG	NEG	NEG	POS	POS	AMB	POS	NEG
06V31278	CC5-MRSA-II, Rhine-Hesse EMRSA/UK-EMRSA-3		NEG	NEG	NEG	NEG	POS	NEG	NEG	NEG	NEG	POS	NEG	POS	NEG		NEG	POS		POS	POS		NEG			POS	POS		POS	NEG	NEG	NEG	NEG	POS	POS	POS	POS	NEG
06V36043	CC5-MRSA-II, Rhine-Hesse EMRSA/UK-EMRSA-3	NEG	NEG	NEG	NEG	NEG	POS	NEG	NEG	POS	NEG	POS	NEG	POS	POS	NEG	NEG	POS	POS	POS	POS	NEG	POS	POS	NEG	POS	POS	POS	POS	NEG	NEG	NEG	NEG	POS	POS	POS	POS	NEG
06V36349	CC5-MRSA-II, Rhine-Hesse EMRSA/UK-EMRSA-3	NEG	NEG	NEG	NEG	NEG	POS	NEG	NEG	POS	NEG	POS	NEG	POS	POS	NEG	NEG	POS	POS	POS	POS	NEG	POS	POS	NEG	POS	POS	POS	POS	NEG	NEG	NEG	NEG	POS	POS	POS	POS	NEG
06V39695	CC5-MRSA-II, Rhine-Hesse EMRSA/UK-EMRSA-3	NEG	NEG	NEG	NEG	NEG	POS	NEG	NEG	POS	NEG	POS	NEG	POS	POS	NEG	NEG	POS	POS	POS	POS	NEG	POS	POS	NEG	POS	POS	NEG	POS	NEG	NEG	NEG	NEG	POS	POS	POS	POS	NEG
06V40571	CC5-MRSA-II, Rhine-Hesse EMRSA/UK-EMRSA-3		NEG	NEG	NEG	NEG	POS	NEG	NEG	POS	NEG	POS	NEG	POS	POS		NEG	POS		AMB	POS		POS			POS	POS		POS	NEG	NEG	NEG	NEG	POS	POS	NEG	POS	NEG
06V42188	CC5-MRSA-II, Rhine-Hesse EMRSA/UK-EMRSA-3	NEG	NEG	NEG	NEG	NEG	POS	NEG	NEG	NEG	NEG	POS	NEG	POS	NEG	NEG	NEG	POS	POS	POS	POS	NEG	NEG	POS	NEG	POS	POS	POS	POS	NEG	NEG	NEG	NEG	POS	POS	POS	POS	NEG
06V42146	CC8-MRSA-IV, Lyon Clone/UK-EMRSA-2		NEG	NEG	POS	NEG	NEG	NEG	NEG	POS	NEG	NEG	NEG	NEG	POS		NEG	NEG		NEG	NEG		POS			POS	POS		POS	NEG	NEG	NEG	NEG	POS	POS	POS	POS	NEG
06V28560	CC8-MRSA-V	NEG	NEG	NEG	NEG	NEG	NEG	NEG	NEG	NEG	NEG	NEG	NEG	NEG	NEG	NEG	NEG	NEG	NEG	NEG	NEG	NEG	NEG	NEG	NEG	POS	POS	NEG	POS	NEG	NEG	NEG	NEG	POS	POS	POS	POS	NEG
06F00837	CC22-MRSA-IV, Barnim/UK-EMRSA-15	NEG	NEG	NEG	NEG	NEG	NEG	NEG	NEG	NEG	NEG	POS	NEG	POS	NEG	NEG	NEG	POS	POS	POS	POS	NEG	NEG	POS	NEG	POS	AMB	POS	POS	NEG	NEG	NEG	NEG	NEG	NEG	POS	POS	NEG
06F01638	CC22-MRSA-IV, Barnim/UK-EMRSA-15	NEG	NEG	NEG	NEG	NEG	NEG	NEG	NEG	NEG	NEG	POS	NEG	POS	NEG	NEG	NEG	POS	POS	POS	POS	NEG	NEG	POS	NEG	POS	POS	POS	POS	NEG	NEG	NEG	NEG	NEG	NEG	POS	POS	NEG
06F04643	CC22-MRSA-IV, Barnim/UK-EMRSA-15		NEG	NEG	NEG	NEG	NEG	NEG	NEG	NEG	NEG	POS	NEG	POS	NEG		NEG	POS		AMB	POS		NEG			NEG	NEG		NEG	NEG	NEG	NEG	NEG	NEG	NEG	NEG	POS	NEG
06F05580	CC22-MRSA-IV, Barnim/UK-EMRSA-15	NEG	NEG	NEG	NEG	NEG	NEG	NEG	NEG	NEG	NEG	POS	NEG	POS	NEG	NEG	NEG	POS	POS	POS	POS	NEG	NEG	POS	NEG	NEG	NEG	POS	NEG	NEG	NEG	NEG	NEG	NEG	NEG	POS	POS	NEG
06U05469	CC22-MRSA-IV, Barnim/UK-EMRSA-15	NEG	NEG	NEG	NEG	NEG	NEG	NEG	NEG	NEG	NEG	POS	NEG	POS	NEG	NEG	NEG	POS	POS	POS	POS	NEG	NEG	POS	NEG	POS	POS	POS	POS	NEG	NEG	NEG	NEG	NEG	NEG	POS	POS	NEG
06U17314	CC22-MRSA-IV, Barnim/UK-EMRSA-15	NEG	NEG	NEG	NEG	NEG	NEG	NEG	NEG	NEG	NEG	NEG	NEG	POS	NEG	NEG	NEG	POS	NEG	NEG	POS	NEG	NEG	POS	NEG	AMB	AMB	POS	POS	NEG	NEG	NEG	NEG	NEG	NEG	POS	POS	NEG
06V08377	CC22-MRSA-IV, Barnim/UK-EMRSA-15	NEG	NEG	NEG	NEG	NEG	NEG	NEG	NEG	NEG	NEG	POS	NEG	POS	NEG	NEG	NEG	POS	POS	POS	POS	NEG	NEG	POS	NEG	POS	POS	POS	POS	NEG	NEG	NEG	NEG	NEG	NEG	POS	POS	NEG
06V08474	CC22-MRSA-IV, Barnim/UK-EMRSA-15	NEG	NEG	NEG	NEG	NEG	NEG	NEG	NEG	NEG	NEG	POS	NEG	POS	NEG	NEG	NEG	POS	POS	POS	POS	NEG	NEG	POS	NEG	POS	AMB	POS	POS	NEG	NEG	NEG	NEG	NEG	NEG	POS	POS	NEG
06V09374	CC22-MRSA-IV, Barnim/UK-EMRSA-15	NEG	NEG	NEG	NEG	NEG	NEG	AMB	POS	NEG	NEG	POS	NEG	POS	NEG	NEG	POS	POS	POS	POS	POS	NEG	NEG	POS	NEG	POS	POS	POS	POS	NEG	NEG	NEG	NEG	NEG	NEG	POS	POS	NEG
06V09605	CC22-MRSA-IV, Barnim/UK-EMRSA-15	NEG	NEG	NEG	NEG	NEG	NEG	NEG	POS	NEG	NEG	POS	NEG	POS	NEG	NEG	POS	POS	POS	POS	POS	NEG	NEG	POS	NEG	AMB	AMB	POS	POS	NEG	NEG	NEG	NEG	NEG	NEG	POS	POS	NEG
06V18687	CC22-MRSA-IV, Barnim/UK-EMRSA-15	NEG	NEG	NEG	NEG	NEG	NEG	NEG	NEG	NEG	NEG	POS	NEG	POS	NEG	NEG	NEG	POS	POS	POS	POS	NEG	NEG	POS	NEG	NEG	NEG	POS	AMB	NEG	NEG	NEG	NEG	NEG	NEG	POS	POS	NEG
06V21697	CC22-MRSA-IV, Barnim/UK-EMRSA-15	NEG	NEG	NEG	NEG	NEG	NEG	NEG	NEG	NEG	NEG	POS	NEG	POS	NEG	NEG	NEG	POS	POS	POS	POS	NEG	NEG	POS	NEG	POS	POS	POS	POS	NEG	NEG	NEG	NEG	NEG	NEG	POS	POS	NEG
06V23749	CC22-MRSA-IV, Barnim/UK-EMRSA-15	NEG	NEG	NEG	NEG	NEG	NEG	NEG	NEG	NEG	NEG	POS	NEG	POS	NEG	NEG	NEG	POS	POS	POS	POS	NEG	NEG	POS	NEG	AMB	AMB	POS	POS	NEG	NEG	NEG	NEG	NEG	NEG	POS	POS	NEG
06V29915	CC22-MRSA-IV, Barnim/UK-EMRSA-15	NEG	NEG	NEG	NEG	NEG	NEG	NEG	NEG	NEG	NEG	POS	NEG	POS	NEG	NEG	NEG	POS	POS	POS	POS	NEG	NEG	POS	NEG	POS	POS	POS	POS	NEG	NEG	NEG	NEG	NEG	NEG	POS	POS	NEG
06V31778	CC22-MRSA-IV, Barnim/UK-EMRSA-15	NEG	NEG	NEG	NEG	NEG	NEG	NEG	NEG	POS	NEG	POS	NEG	POS	NEG	NEG	NEG	POS	POS	POS	POS	NEG	NEG	POS	NEG	POS	POS	POS	POS	NEG	NEG	NEG	NEG	NEG	NEG	POS	POS	NEG
06V39089	CC22-MRSA-IV, Barnim/UK-EMRSA-15	NEG	NEG	NEG	NEG	NEG	NEG	NEG	NEG	NEG	NEG	POS	NEG	POS	NEG	NEG	NEG	POS	POS	POS	POS	NEG	NEG	POS	NEG	POS	POS	POS	POS	NEG	NEG	NEG	NEG	NEG	NEG	POS	POS	NEG
06V41512	CC22-MRSA-IV, Barnim/UK-EMRSA-15		NEG	NEG	NEG	NEG	NEG	NEG	NEG	NEG	NEG	POS	NEG	POS	NEG		NEG	POS		POS	POS		NEG			AMB	NEG		AMB	NEG	NEG	NEG	NEG	NEG	NEG	POS	POS	NEG
06V42186	CC22-MRSA-IV, Barnim/UK-EMRSA-15	NEG	NEG	NEG	NEG	NEG	NEG	NEG	NEG	NEG	NEG	POS	NEG	POS	NEG	NEG	NEG	POS	POS	POS	POS	NEG	NEG	POS	NEG	AMB	NEG	POS	NEG	NEG	NEG	NEG	NEG	NEG	NEG	POS	POS	NEG
06V42236	CC22-MRSA-IV, Barnim/UK-EMRSA-15	NEG	NEG	NEG	NEG	NEG	NEG	NEG	POS	NEG	NEG	POS	NEG	POS	NEG	NEG	POS	POS	POS	POS	POS	NEG	NEG	POS	NEG	AMB	NEG	POS	POS	NEG	NEG	NEG	NEG	NEG	NEG	POS	POS	NEG
06V43697	CC22-MRSA-IV, Barnim/UK-EMRSA-15	NEG	NEG	NEG	NEG	NEG	NEG	NEG	NEG	NEG	NEG	POS	NEG	POS	NEG	NEG	NEG	POS	POS	POS	POS	NEG	NEG	POS	NEG	POS	POS	POS	POS	NEG	NEG	NEG	NEG	NEG	NEG	POS	POS	NEG
06V44153	CC22-MRSA-IV, Barnim/UK-EMRSA-15	NEG	NEG	NEG	NEG	NEG	NEG	NEG	NEG	NEG	NEG	POS	NEG	POS	NEG	NEG	NEG	POS	POS	POS	POS	NEG	NEG	POS	NEG	NEG	NEG	POS	AMB	NEG	NEG	NEG	NEG	NEG	NEG	AMB	POS	NEG
06V45679	CC22-MRSA-IV, Barnim/UK-EMRSA-15	NEG	NEG	NEG	NEG	NEG	NEG	NEG	POS	NEG	NEG	POS	NEG	POS	NEG	NEG	POS	POS	POS	POS	AMB	NEG	NEG	POS	NEG	NEG	NEG	POS	NEG	NEG	NEG	NEG	NEG	NEG	NEG	AMB	POS	NEG
06V45831	CC22-MRSA-IV, Barnim/UK-EMRSA-15	NEG	NEG	NEG	NEG	NEG	NEG	NEG	NEG	NEG	NEG	POS	NEG	POS	NEG	NEG	NEG	POS	POS	POS	POS	NEG	NEG	POS	NEG	NEG	NEG	POS	NEG	NEG	NEG	NEG	NEG	NEG	NEG	POS	POS	NEG
06V46314	CC22-MRSA-IV, Barnim/UK-EMRSA-15	NEG	NEG	NEG	NEG	NEG	NEG	NEG	NEG	NEG	NEG	NEG	NEG	POS	NEG	NEG	NEG	POS	NEG	NEG	POS	NEG	NEG	POS	NEG	AMB	AMB	POS	AMB	NEG	NEG	NEG	NEG	NEG	NEG	POS	POS	NEG
06V34729	CC30-MRSA-IV [PVL+], Southwest Pacific Clone	NEG	NEG	NEG	NEG	NEG	NEG	NEG	NEG	NEG	NEG	POS	NEG	POS	NEG	NEG	NEG	POS	POS	POS	POS	NEG	NEG	POS	NEG	POS	POS	POS	POS	POS	POS	NEG	NEG	NEG	NEG	POS	NEG	POS
06B09900	CC45-MRSA-IV, Berlin EMRSA	NEG	NEG	NEG	NEG	NEG	NEG	NEG	NEG	NEG	NEG	NEG	NEG	POS	NEG	NEG	NEG	POS	POS	POS	POS	NEG	NEG	POS	NEG	POS	POS	POS	POS	NEG	NEG	NEG	NEG	NEG	NEG	POS	NEG	POS
06V00922	CC45-MRSA-IV, Berlin EMRSA	NEG	NEG	NEG	NEG	NEG	NEG	NEG	NEG	NEG	NEG	NEG	NEG	POS	NEG	NEG	NEG	POS	POS	POS	POS	NEG	NEG	POS	NEG	POS	AMB	POS	POS	NEG	NEG	NEG	NEG	NEG	NEG	POS	NEG	POS
06V09551	CC45-MRSA-IV, Berlin EMRSA	NEG	NEG	NEG	NEG	NEG	NEG	NEG	POS	NEG	NEG	POS	NEG	POS	NEG	NEG	POS	POS	POS	POS	POS	NEG	NEG	POS	NEG	POS	POS	POS	POS	NEG	NEG	NEG	NEG	NEG	NEG	POS	NEG	POS
06V10719	CC45-MRSA-IV, Berlin EMRSA	NEG	NEG	NEG	NEG	NEG	NEG	NEG	NEG	NEG	NEG	POS	NEG	POS	NEG	NEG	NEG	POS	POS	POS	POS	NEG	NEG	POS	NEG	POS	AMB	POS	POS	NEG	NEG	NEG	NEG	NEG	NEG	POS	NEG	POS
06V11090	CC45-MRSA-IV, Berlin EMRSA	NEG	NEG	NEG	NEG	NEG	NEG	NEG	NEG	NEG	NEG	NEG	NEG	POS	NEG	NEG	NEG	POS	POS	POS	POS	NEG	NEG	POS	NEG	POS	NEG	POS	POS	NEG	NEG	NEG	NEG	NEG	NEG	AMB	NEG	POS
06V14383	CC45-MRSA-IV, Berlin EMRSA	NEG	NEG	NEG	NEG	NEG	NEG	NEG	NEG	NEG	NEG	NEG	NEG	POS	NEG	NEG	NEG	POS	POS	POS	POS	NEG	NEG	POS	NEG	POS	NEG	POS	POS	NEG	NEG	NEG	NEG	NEG	NEG	POS	NEG	POS
06V21475	CC45-MRSA-IV, Berlin EMRSA	NEG	NEG	NEG	NEG	NEG	NEG	NEG	NEG	NEG	NEG	NEG	NEG	POS	NEG	NEG	NEG	POS	POS	POS	POS	NEG	NEG	POS	NEG	POS	NEG	POS	POS	NEG	NEG	NEG	NEG	NEG	NEG	POS	NEG	POS
06V24231	CC45-MRSA-IV, Berlin EMRSA	NEG	NEG	NEG	NEG	NEG	NEG	NEG	NEG	NEG	NEG	NEG	NEG	POS	NEG	NEG	NEG	POS	POS	POS	POS	NEG	NEG	POS	NEG	POS	NEG	POS	POS	NEG	NEG	NEG	NEG	NEG	NEG	POS	NEG	POS
06V32992	CC45-MRSA-IV, Berlin EMRSA	NEG	NEG	NEG	NEG	NEG	NEG	NEG	NEG	NEG	NEG	NEG	NEG	POS	NEG	NEG	NEG	POS	POS	POS	POS	NEG	NEG	POS	NEG	POS	NEG	POS	POS	NEG	NEG	NEG	NEG	NEG	NEG	AMB	NEG	POS
06V38753	CC45-MRSA-IV, Berlin EMRSA	NEG	NEG	NEG	NEG	NEG	NEG	NEG	NEG	NEG	NEG	NEG	NEG	POS	NEG	NEG	NEG	POS	POS	POS	POS	NEG	NEG	POS	NEG	POS	NEG	POS	POS	NEG	NEG	NEG	NEG	NEG	NEG	POS	NEG	POS
06V39848	CC45-MRSA-IV, Berlin EMRSA		NEG	NEG	NEG	NEG	NEG	NEG	NEG	NEG	NEG	NEG	NEG	POS	NEG		NEG	POS		POS	POS		NEG			POS	NEG		POS	NEG	NEG	NEG	NEG	NEG	NEG	AMB	NEG	POS
06V42259	CC45-MRSA-IV, Berlin EMRSA	NEG	NEG	NEG	NEG	NEG	NEG	NEG	NEG	NEG	NEG	NEG	NEG	POS	NEG	NEG	NEG	POS	POS	POS	POS	NEG	NEG	POS	NEG	POS	POS	POS	POS	NEG	NEG	NEG	NEG	NEG	NEG	POS	NEG	POS
06V33960	CC80-MRSA-IV [PVL+], European caMRSA Clone		NEG	NEG	NEG	NEG	NEG	NEG	NEG	NEG	NEG	NEG	NEG	NEG	NEG		NEG	NEG		NEG	NEG		NEG			POS	POS		POS	POS	POS	amb	NEG	POS	POS	POS	POS	NEG

ISOLATE:	STRAIN	VIRULENCE : HAEMOLYSINS								VIRULENCE : HLB-CONV PHAGES			VIRULENCE : EXFOL.TOXINS			VIRULENCE : EPITHEL. DIFF. INHIB			VIRULENCE : ACME LOCUS				VIRULENCE : PROTEASES										VIRULENCE : STAPHYLOCOCCAL SUPERANTIGEN/ENTEROTOXIN-LIKE GENES (SET/SSL)						
		putative membrane protein	haemolysin alpha	putative membrane protein		haemolysin beta				staphylo-kinase	chemotaxis-inhibiting protein (CHIPS)	Staphyl. Comple-ment inhibitor	exfoliative toxin serotype A	exfoliative toxin serotype B	exfoliative toxin D	epidermal cell differen-tiation inhibitor	epidermal cell differen-tiation inhibitor B	epidermal cell differen-tiation inhibitor C	ACME-locus	ACME-locus: ornithincarb-amoyltrans-ferase	ACME-locus: carbamat-kinase	ACME-locus: arginine/omi-thine-antiporter	aureolysin			serin- protease A	serin- protease B	serin- protease E	glutamylendopeptidase	staphopain B, protease	staphopain A (staphylopain A), protease		staphyl. exotoxin-like protein	staphylococcal superantigen-like protein 1					
		hl	hla	hIIII (consensus)	hIIII (other than RF122)	hlb_probe 1	hlb_probe 2	hlb_probe 3	un-disrupted hlb	sak	chp	scn	etA	etB	etD	edinA	edinB	edinC	arcA-SCC	arcB-SCC	arcC-SCC	arcD-SCC	aur (consensus)	aur (other than MRSA252)	aur (MRSA252)	splA	splB	splE	sspA	sspB	sspP (consensus)	sspP (other than ST93)	setC	ssl01/set6_probe1_11	ssl01/set6_probe2_11	ssl01/set6_probe1_12	ssl01/set6_probe2_12	ssl01/set6_probe4_11	ssl01/set6_probeRF122
**2005**																																							
05B01179	CC5/ST228-MRSA-I, South German EMRSA	POS	POS	POS	POS	POS	POS	AMB	NEG	POS	NEG	POS	NEG	NEG	NEG	NEG	NEG	NEG	NEG	NEG	NEG	NEG	POS	POS	NEG	POS	POS	NEG	POS	POS	POS	POS	POS	POS	NEG	POS	NEG	POS	NEG
05B02687	CC5/ST228-MRSA-I, South German EMRSA	POS	POS	POS	POS	POS	POS	POS	NEG	POS	NEG	POS	NEG	NEG	NEG	NEG	NEG	NEG	NEG	NEG	NEG	NEG	POS	POS	NEG	POS	POS	NEG	POS	POS	POS	POS	POS	POS	NEG	POS	NEG	POS	NEG
05V08483	CC5/ST228-MRSA-I, South German EMRSA	POS	POS	POS	POS	POS	POS	POS	NEG	POS	NEG	POS	NEG	NEG	NEG	NEG	NEG	NEG	NEG	NEG	NEG	NEG	POS	POS	NEG	POS	POS	NEG	POS	POS	POS	POS	POS	POS	NEG	POS	NEG	POS	NEG
05V19001	CC5/ST228-MRSA-I, South German EMRSA	POS	POS	POS	POS	AMB	POS	AMB	NEG	POS	NEG	POS	NEG	NEG	NEG	NEG	NEG	NEG	NEG	NEG	NEG	NEG	AMB	POS	NEG	POS	POS	NEG	POS	POS	POS	POS	POS	POS	AMB	POS	NEG	POS	NEG
05B04626	CC5-MRSA-II, Rhine-Hesse EMRSA/UK-EMRSA-3	POS	POS	POS	AMB	NEG	POS	NEG	NEG	AMB	POS	POS	NEG	NEG	NEG	NEG	NEG	NEG	NEG	NEG	NEG	NEG	POS	POS	NEG	POS	POS	NEG	POS	POS	POS	POS	POS	POS	NEG	POS	NEG	POS	NEG
05B07903	CC5-MRSA-II, Rhine-Hesse EMRSA/UK-EMRSA-3	POS	POS	POS	POS	POS	POS	AMB	NEG	POS	POS	POS	NEG	NEG	NEG	NEG	NEG	NEG	NEG	NEG	NEG	NEG	POS	POS	NEG	POS	POS	NEG	POS	POS	POS	POS	POS	POS	NEG	POS	NEG	POS	NEG
05U00062	CC5-MRSA-II, Rhine-Hesse EMRSA/UK-EMRSA-3	POS	POS	POS	POS	AMB	POS	NEG	NEG	POS	POS	POS	NEG	NEG	NEG	NEG	NEG	NEG	NEG	NEG	NEG	NEG	POS	POS	NEG	POS	POS	NEG	POS	POS	POS	POS	POS	POS	NEG	POS	NEG	AMB	NEG
05U03117	CC5-MRSA-II, Rhine-Hesse EMRSA/UK-EMRSA-3	POS	POS	POS	POS	NEG	POS	NEG	NEG	POS	POS	POS	NEG	NEG	NEG	NEG	NEG	NEG	NEG	NEG	NEG	NEG	POS	POS	NEG	POS	POS	NEG	POS	POS	POS	POS	POS	POS	NEG	POS	NEG	NEG	NEG
05U10392	CC5-MRSA-II, Rhine-Hesse EMRSA/UK-EMRSA-3	POS	POS	POS	POS	AMB	POS	NEG	NEG	POS	POS	POS	NEG	NEG	NEG	NEG	NEG	NEG	NEG	NEG	NEG	NEG	POS	POS	NEG	POS	POS	NEG	POS	POS	POS	POS	POS	POS	NEG	POS	NEG	POS	NEG
05V03312	CC5-MRSA-II, Rhine-Hesse EMRSA/UK-EMRSA-3	POS	POS	POS	POS	POS	POS	POS	NEG	POS	POS	POS	NEG	NEG	NEG	NEG	NEG	NEG	NEG	NEG	NEG	NEG	POS	POS	NEG	POS	POS	NEG	POS	POS	POS	POS	POS	POS	NEG	POS	NEG	POS	NEG
05V08095	CC5-MRSA-II, Rhine-Hesse EMRSA/UK-EMRSA-3	POS	POS	POS	POS	POS	POS	POS	NEG	POS	POS	POS	NEG	NEG	NEG	NEG	NEG	NEG	NEG	NEG	NEG	NEG	POS	POS	NEG	POS	POS	NEG	POS	POS	POS	POS	POS	POS	NEG	POS	NEG	POS	NEG
05V09015	CC5-MRSA-II, Rhine-Hesse EMRSA/UK-EMRSA-3	POS	POS	POS	POS	AMB	POS	AMB	NEG	POS	POS	POS	NEG	NEG	NEG	NEG	NEG	NEG	NEG	NEG	NEG	NEG	POS	POS	NEG	POS	POS	NEG	POS	POS	POS	POS	POS	POS	NEG	POS	NEG	POS	NEG
05V09326	CC5-MRSA-II, Rhine-Hesse EMRSA/UK-EMRSA-3	POS	POS	POS	POS	POS	POS	POS	NEG	POS	POS	POS	NEG	NEG	NEG	NEG	NEG	NEG	NEG	NEG	NEG	NEG	POS	POS	NEG	POS	POS	NEG	POS	POS	POS	POS	POS	POS	NEG	POS	NEG	POS	NEG
05V10265	CC5-MRSA-II, Rhine-Hesse EMRSA/UK-EMRSA-3	POS	POS	POS	POS	AMB	POS	AMB	NEG	POS	POS	POS	NEG	NEG	NEG	NEG	NEG	NEG	NEG	NEG	NEG	NEG	POS	AMB	NEG	POS	POS	NEG	POS	POS	POS	POS	POS	POS	NEG	POS	NEG	POS	NEG
05V13052	CC5-MRSA-II, Rhine-Hesse EMRSA/UK-EMRSA-3	POS	POS	POS	POS	NEG	POS	NEG	NEG	POS	POS	POS	NEG	NEG	NEG	NEG	NEG	NEG	NEG	NEG	NEG	NEG	POS	POS	NEG	POS	POS	NEG	POS	POS	POS	POS	POS	POS	AMB	POS	NEG	POS	NEG
05V13470	CC5-MRSA-II, Rhine-Hesse EMRSA/UK-EMRSA-3	POS	POS	POS	POS	POS	POS	AMB	NEG	POS	POS	POS	NEG	NEG	NEG	NEG	NEG	NEG	NEG	NEG	NEG	NEG	POS	POS	NEG	POS	POS	NEG	POS	POS	POS	POS	POS	POS	NEG	POS	NEG	POS	NEG
05V13891	CC5-MRSA-II, Rhine-Hesse EMRSA/UK-EMRSA-3	POS	POS	POS	POS	NEG	POS	NEG	NEG	POS	POS	POS	NEG	NEG	NEG	NEG	NEG	NEG	NEG	NEG	NEG	NEG	POS	POS	NEG	POS	POS	NEG	POS	POS	POS	POS	POS	POS	NEG	POS	NEG	NEG	NEG
05V15616	CC5-MRSA-II, Rhine-Hesse EMRSA/UK-EMRSA-3	POS	POS		POS				NEG	POS			NEG	NEG	NEG	NEG	NEG	NEG								POS	POS						POS	POS	NEG	POS	NEG	POS	
05V16845	CC5-MRSA-II, Rhine-Hesse EMRSA/UK-EMRSA-3	POS	POS	POS	POS	POS	POS	POS	NEG	POS	POS	POS	NEG	NEG	NEG	NEG	NEG	NEG	NEG	NEG	NEG	NEG	POS	POS	NEG	POS	POS	NEG	POS	POS	POS	POS	POS	POS	NEG	POS	NEG	POS	NEG
05V17541	CC5-MRSA-II, Rhine-Hesse EMRSA/UK-EMRSA-3	POS	POS	POS	POS	NEG	POS	NEG	NEG	POS	POS	POS	NEG	NEG	NEG	NEG	NEG	NEG	NEG	NEG	NEG	NEG	POS	POS	NEG	POS	POS	NEG	POS	POS	POS	POS	POS	POS	NEG	POS	NEG	NEG	NEG
05V19105	CC5-MRSA-II, Rhine-Hesse EMRSA/UK-EMRSA-3	POS	POS		POS				NEG	POS			NEG	NEG	NEG	NEG	NEG	NEG								POS	POS						POS	POS	NEG	POS	NEG	POS	
05V22148	CC5-MRSA-II, Rhine-Hesse EMRSA/UK-EMRSA-3	POS	POS	POS	POS	POS	POS	POS	NEG	POS	POS	POS	NEG	NEG	NEG	NEG	NEG	NEG	NEG	NEG	NEG	NEG	POS	POS	NEG	POS	POS	NEG	POS	POS	POS	POS	POS	POS	NEG	POS	NEG	POS	NEG
05V33740	CC5-MRSA-II, Rhine-Hesse EMRSA/UK-EMRSA-3	POS	NEG	POS	POS	AMB	POS	NEG	NEG	POS	POS	POS	NEG	NEG	NEG	NEG	NEG	NEG	NEG	NEG	NEG	NEG	AMB	POS	NEG	POS	POS	NEG	POS	POS	POS	POS	POS	POS	NEG	POS	NEG	AMB	NEG
05V38425	CC5-MRSA-II, Rhine-Hesse EMRSA/UK-EMRSA-3	POS	NEG	POS	POS	AMB	POS	NEG	NEG	POS	POS	POS	NEG	NEG	NEG	NEG	NEG	NEG	NEG	NEG	NEG	NEG	POS	POS	NEG	POS	POS	NEG	POS	POS	POS	POS	POS	POS	NEG	POS	NEG	POS	NEG
05V39987	CC5-MRSA-II, Rhine-Hesse EMRSA/UK-EMRSA-3	POS	POS	POS	POS	AMB	POS	AMB	NEG	POS	POS	POS	NEG	NEG	NEG	NEG	NEG	NEG	NEG	NEG	NEG	NEG	POS	POS	NEG	POS	POS	NEG	POS	POS	POS	POS	POS	POS	NEG	POS	NEG	POS	NEG
05F00089	CC5-MRSA-IV, Paediatric clone	POS	POS	POS	POS	POS	POS	POS	NEG	POS	POS	POS	NEG	NEG	NEG	NEG	NEG	NEG	NEG	NEG	NEG	NEG	POS	POS	NEG	POS	POS	NEG	POS	POS	POS	POS	POS						NEG
05V01416	CC5-MRSA-IV, Paediatric clone	POS	AMB	POS	POS	NEG	POS	NEG	NEG	POS	POS	POS	NEG	NEG	NEG	NEG	NEG	NEG	NEG	NEG	NEG	NEG	POS	POS	NEG	POS	POS	NEG	POS	POS	POS	POS	POS	POS	NEG	POS	NEG	NEG	NEG
05V16386	CC8-MRSA-IV, USA300	POS	POS	POS	POS	POS	POS	POS	NEG	POS	POS	POS	NEG	NEG	NEG	NEG	NEG	NEG	POS	POS	POS	POS	AMB	AMB	NEG	POS	POS	POS	POS	POS	POS	POS	POS						AMB
05V04920	CC8-MRSA-IV, USA500	POS	POS	POS	POS	AMB	POS	AMB	POS	NEG	NEG	NEG	NEG	NEG	NEG	NEG	NEG	NEG	NEG	NEG	NEG	NEG	POS	POS	NEG	POS	POS	POS	POS	POS	POS	POS	POS						NEG
05V21159	CC8-MRSA-IV, USA500	POS	POS	POS	POS	POS	POS	POS	NEG	POS	NEG	POS	NEG	NEG	NEG	NEG	NEG	NEG	NEG	NEG	NEG	NEG	POS	POS	NEG	POS	POS	POS	POS	POS	POS	POS	POS						NEG
05B00867	CC22-MRSA-IV, Barnim/UK-EMRSA-15	POS	POS	NEG	NEG	POS	POS	POS	NEG	POS	POS	POS	NEG	NEG	NEG	NEG	NEG	NEG	NEG	NEG	NEG	NEG	POS	NEG	POS	NEG	NEG	NEG	POS	POS	POS	POS	POS						NEG
05V03174	CC22-MRSA-IV, Barnim/UK-EMRSA-15	POS	POS	NEG	NEG	POS	POS	POS	NEG	POS	POS	POS	NEG	NEG	NEG	NEG	NEG	NEG	NEG	NEG	NEG	NEG	POS	NEG	POS	NEG	NEG	NEG	POS	POS	POS	POS	POS	NEG	POS	POS	NEG	NEG	NEG
05V07331	CC22-MRSA-IV, Barnim/UK-EMRSA-15	POS	POS	NEG	NEG	POS	POS	POS	NEG	POS	POS	POS	NEG	NEG	NEG	NEG	NEG	NEG	NEG	NEG	NEG	NEG	POS	NEG	POS	NEG	NEG	NEG	POS	POS	POS	POS	POS	NEG	POS	POS	NEG	NEG	NEG
05V07371	CC22-MRSA-IV, Barnim/UK-EMRSA-15	POS	POS	NEG	NEG	POS	POS	AMB	POS	NEG	NEG	NEG	NEG	NEG	NEG	NEG	NEG	NEG	NEG	NEG	NEG	NEG	POS	NEG	POS	NEG	NEG	NEG	POS	POS	POS	POS	POS	NEG	POS	AMB	NEG	NEG	NEG
05V08145	CC22-MRSA-IV, Barnim/UK-EMRSA-15	POS	AMB	NEG	NEG	POS	POS	AMB	NEG	POS	POS	POS	NEG	NEG	NEG	NEG	NEG	NEG	NEG	NEG	NEG	NEG	POS	NEG	POS	NEG	NEG	NEG	POS	POS	POS	POS	POS	NEG	POS	NEG	NEG	NEG	NEG
05V08595	CC22-MRSA-IV, Barnim/UK-EMRSA-15	POS	POS	NEG	NEG	POS	POS	POS	NEG	POS	POS	POS	NEG	NEG	NEG	NEG	NEG	NEG	NEG	NEG	NEG	NEG	POS	NEG	POS	NEG	NEG	NEG	POS	POS	POS	POS	POS	NEG	POS	AMB	NEG	NEG	NEG
05V12803	CC22-MRSA-IV, Barnim/UK-EMRSA-15	POS	POS	NEG	NEG	POS	POS	POS	NEG	POS	POS	POS	NEG	NEG	NEG	NEG	NEG	NEG	NEG	NEG	NEG	NEG	POS	NEG	POS	NEG	NEG	NEG	POS	POS	POS	POS	POS	NEG	POS	AMB	NEG	NEG	NEG
05V14596	CC22-MRSA-IV, Barnim/UK-EMRSA-15	POS	POS	NEG	NEG	POS	POS	NEG	NEG	POS	POS	POS	NEG	NEG	NEG	NEG	NEG	NEG	NEG	NEG	NEG	NEG	POS	NEG	POS	NEG	NEG	NEG	POS	POS	POS	POS	POS						NEG
05V16691	CC22-MRSA-IV, Barnim/UK-EMRSA-15	POS	POS		NEG				NEG	POS			NEG	NEG	NEG	NEG	NEG	NEG								NEG	NEG						POS						
05V18550	CC22-MRSA-IV, Barnim/UK-EMRSA-15	POS	POS	NEG	NEG	POS	POS	POS	NEG	POS	POS	POS	NEG	NEG	NEG	NEG	NEG	NEG	NEG	NEG	NEG	NEG	POS	NEG	POS	NEG	NEG	NEG	POS	POS	POS	POS	POS	NEG	POS	POS	NEG	NEG	NEG
05V19034	CC22-MRSA-IV, Barnim/UK-EMRSA-15	POS	POS	NEG	NEG	POS	POS	POS	NEG	POS	POS	POS	NEG	NEG	NEG	NEG	NEG	NEG	NEG	NEG	NEG	NEG	POS	NEG	POS	NEG	NEG	NEG	POS	POS	POS	POS	POS	NEG	POS	POS	NEG	NEG	NEG
05V19333	CC22-MRSA-IV, Barnim/UK-EMRSA-15	POS	POS		NEG				NEG	POS			NEG	NEG	NEG	NEG	NEG	NEG								NEG	NEG						POS						
05V19701	CC22-MRSA-IV, Barnim/UK-EMRSA-15	POS	POS	NEG	NEG	POS	POS	POS	NEG	POS	POS	POS	NEG	NEG	NEG	NEG	NEG	NEG	NEG	NEG	NEG	NEG	POS	NEG	POS	NEG	NEG	NEG	POS	POS	POS	POS	POS	NEG	POS	NEG	NEG	NEG	NEG
05V20072	CC22-MRSA-IV, Barnim/UK-EMRSA-15	POS	POS	NEG	NEG	POS	POS	AMB	POS	NEG	NEG	NEG	NEG	NEG	NEG	NEG	NEG	NEG	NEG	NEG	NEG	NEG	POS	NEG	POS	NEG	NEG	NEG	POS	POS	POS	POS	POS	NEG	POS	AMB	NEG	NEG	NEG
05V24930	CC22-MRSA-IV, Barnim/UK-EMRSA-15	POS	POS	NEG	NEG	POS	POS	POS	NEG	POS	POS	POS	NEG	NEG	NEG	NEG	NEG	NEG	NEG	NEG	NEG	NEG	POS	NEG	POS	NEG	NEG	NEG	POS	POS	POS	POS	POS	NEG	POS	POS	NEG	NEG	NEG
05V31461	CC22-MRSA-IV, Barnim/UK-EMRSA-15	POS	POS	NEG	NEG	POS	POS	POS	POS	NEG	AMB	NEG	NEG	NEG	NEG	NEG	NEG	NEG	NEG	NEG	NEG	NEG	POS	NEG	POS	NEG	NEG	NEG	POS	POS	POS	POS	POS	NEG	POS	POS	NEG	NEG	NEG
05B05671	CC45-MRSA-IV, Berlin EMRSA	POS	POS	POS	NEG	NEG	AMB	NEG	NEG	POS	POS	POS	NEG	NEG	NEG	NEG	NEG	NEG	NEG	NEG	NEG	NEG	AMB	NEG	POS	NEG	NEG	NEG	POS	POS	POS	POS	POS						NEG
05V01452	CC45-MRSA-IV, Berlin EMRSA	POS	POS	POS	NEG	NEG	NEG	NEG	NEG	POS	POS	POS	NEG	NEG	NEG	NEG	NEG	NEG	NEG	NEG	NEG	NEG	AMB	NEG	POS	NEG	NEG	NEG	POS	POS	POS	POS	POS						NEG
05V01617	CC45-MRSA-IV, Berlin EMRSA	POS	POS	POS	NEG	POS	POS	NEG	NEG	POS	POS	POS	NEG	NEG	NEG	NEG	NEG	NEG	NEG	NEG	NEG	NEG	POS	NEG	POS	NEG	NEG	NEG	POS	POS	POS	POS	POS	NEG	POS	POS	NEG	POS	NEG
05V16986	CC45-MRSA-IV, Berlin EMRSA	NEG	AMB	POS	NEG	NEG	NEG	NEG	NEG	POS	POS	POS	NEG	NEG	NEG	NEG	NEG	NEG	NEG	NEG	NEG	NEG	NEG	NEG	POS	NEG	NEG	NEG	POS	POS	POS	POS	NEG	NEG	POS	NEG	NEG	NEG	NEG
05V18357	CC45-MRSA-IV, Berlin EMRSA	POS	POS	POS	NEG	NEG	NEG	NEG	NEG	POS	POS	POS	NEG	NEG	NEG	NEG	NEG	NEG	NEG	NEG	NEG	NEG	NEG	NEG	POS	NEG	NEG	NEG	POS	POS	POS	POS	POS						NEG
05V23111	CC45-MRSA-IV, Berlin EMRSA	POS	POS	POS	NEG	POS	POS	NEG	NEG	POS	POS	POS	NEG	NEG	NEG	NEG	NEG	NEG	NEG	NEG	NEG	NEG	POS	NEG	POS	NEG	NEG	NEG	POS	POS	POS	POS	POS	NEG	POS	POS	NEG	POS	NEG
05V23414	CC45-MRSA-IV, Berlin EMRSA	POS	POS		NEG				NEG	POS			NEG	NEG	NEG	NEG	NEG	NEG								NEG	NEG						NEG						
05V23858	CC45-MRSA-IV, Berlin EMRSA	POS	POS	POS	NEG	NEG	NEG	NEG	NEG	POS	POS	POS	NEG	NEG	NEG	NEG	NEG	NEG	NEG	NEG	NEG	NEG	AMB	NEG	POS	NEG	NEG	NEG	POS	POS	POS	POS	AMB	NEG	POS	NEG	NEG	AMB	NEG
05V26241	CC45-MRSA-IV, Berlin EMRSA	POS	POS	POS	NEG	POS	POS	NEG	NEG	POS	POS	POS	NEG	NEG	NEG	NEG	NEG	NEG	NEG	NEG	NEG	NEG	AMB	NEG	POS	NEG	NEG	NEG	POS	POS	POS	POS	AMB	NEG	POS	AMB	NEG	POS	NEG
05V28399	CC45-MRSA-IV, Berlin EMRSA	POS	POS	POS	NEG	POS	POS	NEG	NEG	POS	POS	POS	NEG	NEG	NEG	NEG	NEG	NEG	NEG	NEG	NEG	NEG	POS	NEG	POS	NEG	NEG	NEG	POS	POS	POS	POS	POS	NEG	POS	POS	NEG	POS	NEG
05V32647	CC45-MRSA-IV, Berlin EMRSA	POS	NEG		NEG				NEG	POS			NEG	NEG	NEG	NEG	NEG	NEG								NEG	NEG						NEG						
05V37969	CC45-MRSA-IV, Berlin EMRSA	POS	POS	POS	NEG	POS	POS	NEG	NEG	POS	POS	POS	NEG	NEG	NEG	NEG	NEG	NEG	NEG	NEG	NEG	NEG	POS	NEG	POS	NEG	NEG	NEG	POS	POS	POS	POS	AMB	NEG	POS	NEG	NEG	POS	NEG
05U10018	CC398-MRSA-V	POS	POS	POS	POS	NEG	NEG	NEG	NEG	NEG	NEG	NEG	NEG	NEG	NEG	NEG	NEG	NEG	NEG	NEG	NEG	NEG	NEG	NEG	POS	NEG	NEG	NEG	POS	POS	POS	POS	POS						NEG
**2006**																																							
06V36353	CC1-MRSA-IV, WA MRSA-1/57	POS	POS	POS	POS	NEG	POS	NEG	NEG	POS	NEG	POS	NEG	NEG	NEG	NEG	NEG	NEG	NEG	NEG	NEG	NEG	NEG	AMB	NEG	POS	POS	POS	POS	POS	POS	POS	POS	NEG	POS	NEG	POS	NEG	NEG
06V40202	CC5/ST228-MRSA-I, South German EMRSA	POS	POS		POS				NEG	POS			NEG	NEG	NEG	NEG	NEG	NEG								POS	POS						POS	POS	NEG	POS	NEG	POS	
06V41266	CC5/ST228-MRSA-I, South German EMRSA	POS	POS	POS	POS	AMB	POS	AMB	NEG	POS	NEG	POS	NEG	NEG	NEG	NEG	NEG	NEG	NEG	NEG	NEG	NEG	POS	POS	NEG	POS	POS	NEG	POS	POS	POS	POS	POS	POS	NEG	POS	NEG	POS	NEG
06B09097	CC5-MRSA-II, Rhine-Hesse EMRSA/UK-EMRSA-3	POS	POS		POS				NEG	POS			NEG	NEG	NEG	NEG	NEG	NEG								POS	POS						POS	POS	NEG	POS	NEG	POS	
06F00244	CC5-MRSA-II, Rhine-Hesse EMRSA/UK-EMRSA-3	POS	POS	POS	POS	POS	POS	AMB	NEG	POS	POS	POS	NEG	NEG	NEG	NEG	NEG	NEG	NEG	NEG	NEG	NEG	POS	POS	NEG	POS	POS	NEG	POS	POS	POS	POS	POS	POS	NEG	POS	NEG	POS	NEG
06V03913	CC5-MRSA-II, Rhine-Hesse EMRSA/UK-EMRSA-3	POS	POS	POS	POS	POS	POS	POS	NEG	POS	POS	POS	NEG	NEG	NEG	NEG	NEG	NEG	NEG	NEG	NEG	NEG	POS	POS	NEG	POS	POS	NEG	POS	POS	POS	POS	POS	POS	NEG	POS	NEG	POS	NEG
06V04337	CC5-MRSA-II, Rhine-Hesse EMRSA/UK-EMRSA-3	POS	POS	POS	POS	POS	POS	AMB	NEG	POS	POS	POS	NEG	NEG	NEG	NEG	NEG	NEG	NEG	NEG	NEG	NEG	POS	POS	NEG	POS	POS	NEG	POS	POS	POS	POS	POS	POS	NEG	POS	NEG	POS	NEG
06V06785	CC5-MRSA-II, Rhine-Hesse EMRSA/UK-EMRSA-3	POS	POS	POS	POS	POS	POS	POS	NEG	POS	POS	POS	NEG	NEG	NEG	NEG	NEG	NEG	NEG	NEG	NEG	NEG	POS	POS	NEG	POS	POS	NEG	POS	POS	POS	POS	POS	POS	NEG	POS	NEG	POS	NEG
06V12258	CC5-MRSA-II, Rhine-Hesse EMRSA/UK-EMRSA-3	POS	POS	POS	POS	POS	POS	POS	AMB	POS	POS	POS	NEG	NEG	NEG	NEG	NEG	NEG	NEG	NEG	NEG	NEG	POS	POS	NEG	POS	POS	NEG	POS	POS	POS	POS	POS	POS	NEG	POS	NEG	POS	NEG
06V20553	CC5-MRSA-II, Rhine-Hesse EMRSA/UK-EMRSA-3	POS	POS	POS	POS	POS	POS	POS	NEG	POS	POS	POS	NEG	NEG	NEG	NEG	NEG	NEG	NEG	NEG	NEG	NEG	POS	POS	NEG	POS	POS	NEG	POS	POS	POS	POS	POS	POS	NEG	POS	NEG	POS	NEG
06V22564	CC5-MRSA-II, Rhine-Hesse EMRSA/UK-EMRSA-3	POS	POS		POS				NEG	POS			NEG	NEG	NEG	NEG	NEG	NEG								POS	POS						POS	POS	NEG	POS	NEG	POS	
06V23750	CC5-MRSA-II, Rhine-Hesse EMRSA/UK-EMRSA-3	POS	POS	POS	POS	POS	POS	POS	NEG	POS	POS	POS	NEG	NEG	NEG	NEG	NEG	NEG	NEG	NEG	NEG	NEG	POS	POS	NEG	POS	POS	NEG	POS	POS	POS	POS	POS	POS	NEG	POS	NEG	POS	NEG
06V27332	CC5-MRSA-II, Rhine-Hesse EMRSA/UK-EMRSA-3	POS	POS	POS	POS	POS	POS	AMB	NEG	POS	POS	POS	NEG	NEG	NEG	NEG	NEG	NEG	NEG	NEG	NEG	NEG	POS	POS	NEG	POS	POS	NEG	POS	POS	POS	POS	POS	POS	NEG	POS	NEG	POS	NEG
06V29484	CC5-MRSA-II, Rhine-Hesse EMRSA/UK-EMRSA-3	POS	POS	POS	POS	AMB	POS	AMB	NEG	POS	POS	POS	NEG	NEG	NEG	NEG	NEG	NEG	NEG	NEG	NEG	NEG	POS	POS	NEG	POS	POS	NEG	POS	POS	POS	POS	POS	POS	NEG	POS	NEG	AMB	NEG
06V31278	CC5-MRSA-II, Rhine-Hesse EMRSA/UK-EMRSA-3	POS	POS		POS				NEG	POS			NEG	NEG	NEG	NEG	NEG	NEG								POS	POS						POS	POS	NEG	POS	NEG	POS	
06V36043	CC5-MRSA-II, Rhine-Hesse EMRSA/UK-EMRSA-3	POS	POS	POS	POS	POS	POS	AMB	NEG	POS	POS	POS	NEG	NEG	NEG	NEG	NEG	NEG	NEG	NEG	NEG	NEG	POS	POS	NEG	POS	POS	NEG	POS	POS	POS	POS	POS						NEG
06V36349	CC5-MRSA-II, Rhine-Hesse EMRSA/UK-EMRSA-3	POS	POS	POS	POS	POS	POS	POS	NEG	POS	POS	POS	NEG	NEG	NEG	NEG	NEG	NEG	NEG	NEG	NEG	NEG	POS	POS	NEG	POS	POS	NEG	POS	POS	POS	POS	POS	POS	NEG	POS	NEG	POS	NEG
06V39695	CC5-MRSA-II, Rhine-Hesse EMRSA/UK-EMRSA-3	POS	POS	POS	POS	AMB	POS	NEG	NEG	POS	POS	POS	NEG	NEG	NEG	NEG	NEG	NEG	NEG	NEG	NEG	NEG	POS	POS	NEG	POS	POS	NEG	POS	POS	POS	POS	POS	POS	NEG	POS	NEG	POS	NEG
06V40571	CC5-MRSA-II, Rhine-Hesse EMRSA/UK-EMRSA-3	POS	POS		POS				NEG	POS			NEG	NEG	NEG	NEG	NEG	NEG								POS	POS						POS	POS	NEG	POS	NEG	POS	
06V42188	CC5-MRSA-II, Rhine-Hesse EMRSA/UK-EMRSA-3	POS	POS	POS	POS	POS	POS	POS	NEG	POS	POS	POS	NEG	NEG	NEG	NEG	NEG	NEG	NEG	NEG	NEG	NEG	POS	POS	NEG	POS	POS	NEG	POS	POS	POS	POS	POS	POS	NEG	POS	NEG	POS	NEG
06V42146	CC8-MRSA-IV, Lyon Clone/UK-EMRSA-2	POS	POS		POS				NEG	POS			NEG	NEG	NEG	NEG	NEG	NEG								POS	POS						POS						
06V28560	CC8-MRSA-V	POS	POS	POS	POS	POS	POS	POS	NEG	POS	NEG	POS	NEG	NEG	NEG	NEG	NEG	NEG	NEG	NEG	NEG	NEG	POS	POS	NEG	POS	POS	POS	POS	POS	POS	POS	POS						NEG
06F00837	CC22-MRSA-IV, Barnim/UK-EMRSA-15	POS	POS	NEG	NEG	POS	POS	POS	NEG	POS	POS	POS	NEG	NEG	NEG	NEG	NEG	NEG	NEG	NEG	NEG	NEG	POS	NEG	POS	NEG	NEG	NEG	POS	POS	POS	POS	POS	NEG	POS	POS	NEG	NEG	NEG
06F01638	CC22-MRSA-IV, Barnim/UK-EMRSA-15	POS	POS	NEG	NEG	POS	POS	POS	NEG	POS	POS	POS	NEG	NEG	NEG	NEG	NEG	NEG	NEG	NEG	NEG	NEG	POS	NEG	POS	NEG	NEG	NEG	POS	POS	POS	POS	POS	NEG	POS	POS	NEG	NEG	NEG
06F04643	CC22-MRSA-IV, Barnim/UK-EMRSA-15	POS	AMB		NEG				NEG	POS			NEG	NEG	NEG	NEG	NEG	NEG								NEG	NEG						POS						
06F05580	CC22-MRSA-IV, Barnim/UK-EMRSA-15	POS	POS	NEG	NEG	POS	POS	POS	NEG	POS	POS	POS	NEG	NEG	NEG	NEG	NEG	NEG	NEG	NEG	NEG	NEG	POS	NEG	POS	NEG	NEG	NEG	POS	POS	POS	POS	POS	NEG	POS	AMB	NEG	NEG	NEG
06U05469	CC22-MRSA-IV, Barnim/UK-EMRSA-15	POS	POS	NEG	NEG	POS	POS	POS	NEG	POS	POS	POS	NEG	NEG	NEG	NEG	NEG	NEG	NEG	NEG	NEG	NEG	POS	NEG	POS	NEG	NEG	NEG	POS	POS	POS	POS	POS	NEG	POS	POS	NEG	NEG	NEG
06U17314	CC22-MRSA-IV, Barnim/UK-EMRSA-15	POS	POS	NEG	NEG	POS	POS	POS	NEG	POS	POS	POS	NEG	NEG	NEG	NEG	NEG	NEG	NEG	NEG	NEG	NEG	POS	NEG	POS	NEG	NEG	NEG	POS	POS	POS	POS	POS	NEG	POS	AMB	NEG	NEG	NEG
06V08377	CC22-MRSA-IV, Barnim/UK-EMRSA-15	POS	POS	NEG	NEG	POS	POS	POS	NEG	POS	POS	POS	NEG	NEG	NEG	NEG	NEG	NEG	NEG	NEG	NEG	NEG	POS	NEG	POS	NEG	NEG	NEG	POS	POS	POS	POS	POS	NEG	POS	POS	NEG	NEG	NEG
06V08474	CC22-MRSA-IV, Barnim/UK-EMRSA-15	POS	POS	NEG	NEG	POS	POS	POS	NEG	POS	POS	POS	NEG	NEG	NEG	NEG	NEG	NEG	NEG	NEG	NEG	NEG	POS	NEG	POS	NEG	NEG	NEG	POS	POS	POS	POS	POS	NEG	POS	POS	NEG	NEG	NEG
06V09374	CC22-MRSA-IV, Barnim/UK-EMRSA-15	POS	POS	NEG	NEG	POS	POS	POS	NEG	POS	POS	POS	NEG	NEG	NEG	NEG	NEG	NEG	NEG	NEG	NEG	NEG	POS	NEG	POS	NEG	NEG	NEG	POS	POS	POS	POS	POS	NEG	POS	POS	NEG	NEG	NEG
06V09605	CC22-MRSA-IV, Barnim/UK-EMRSA-15	POS	POS	NEG	NEG	POS	POS	POS	NEG	POS	POS	POS	NEG	NEG	NEG	NEG	NEG	NEG	NEG	NEG	NEG	NEG	POS	NEG	POS	NEG	NEG	NEG	POS	POS	POS	POS	POS	NEG	POS	POS	NEG	NEG	NEG
06V18687	CC22-MRSA-IV, Barnim/UK-EMRSA-15	POS	AMB	NEG	NEG	POS	POS	POS	NEG	POS	POS	POS	NEG	NEG	NEG	NEG	NEG	NEG	NEG	NEG	NEG	NEG	POS	NEG	POS	NEG	NEG	NEG	POS	POS	POS	POS	POS	NEG	POS	AMB	NEG	NEG	NEG
06V21697	CC22-MRSA-IV, Barnim/UK-EMRSA-15	POS	POS	NEG	NEG	POS	POS	POS	NEG	POS	POS	POS	NEG	NEG	NEG	NEG	NEG	NEG	NEG	NEG	NEG	NEG	POS	NEG	POS	NEG	NEG	NEG	POS	POS	POS	POS	POS	NEG	POS	POS	NEG	NEG	NEG
06V23749	CC22-MRSA-IV, Barnim/UK-EMRSA-15	POS	POS	NEG	NEG	POS	POS	POS	NEG	POS	POS	POS	NEG	NEG	NEG	NEG	NEG	NEG	NEG	NEG	NEG	NEG	POS	NEG	POS	NEG	NEG	NEG	POS	POS	POS	POS	POS	NEG	POS	POS	NEG	NEG	NEG
06V29915	CC22-MRSA-IV, Barnim/UK-EMRSA-15	POS	POS	NEG	NEG	POS	POS	POS	NEG	POS	POS	POS	NEG	NEG	NEG	NEG	NEG	NEG	NEG	NEG	NEG	NEG	POS	NEG	POS	NEG	NEG	NEG	POS	POS	POS	POS	POS	NEG	POS	POS	NEG	NEG	NEG
06V31778	CC22-MRSA-IV, Barnim/UK-EMRSA-15	POS	POS	NEG	NEG	POS	POS	POS	NEG	POS	POS	POS	NEG	NEG	NEG	NEG	NEG	NEG	NEG	NEG	NEG	NEG	POS	NEG	POS	NEG	NEG	NEG	POS	POS	POS	POS	POS	AMB	POS	POS	NEG	NEG	NEG
06V39089	CC22-MRSA-IV, Barnim/UK-EMRSA-15	POS	POS	NEG	NEG	POS	POS	POS	NEG	POS	POS	POS	NEG	NEG	NEG	NEG	NEG	NEG	NEG	NEG	NEG	NEG	POS	NEG	POS	NEG	NEG	NEG	POS	POS	POS	POS	POS	NEG	POS	POS	NEG	NEG	NEG
06V41512	CC22-MRSA-IV, Barnim/UK-EMRSA-15	POS	POS		NEG				NEG	POS			NEG	NEG	NEG	NEG	NEG	NEG								NEG	NEG						POS						
06V42186	CC22-MRSA-IV, Barnim/UK-EMRSA-15	POS	POS	NEG	NEG	POS	POS	POS	NEG	POS	POS	POS	NEG	NEG	NEG	NEG	NEG	NEG	NEG	NEG	NEG	NEG	POS	NEG	POS	NEG	NEG	NEG	POS	POS	POS	POS	POS	NEG	POS	AMB	NEG	NEG	NEG
06V42236	CC22-MRSA-IV, Barnim/UK-EMRSA-15	POS	POS	NEG	NEG	POS	POS	POS	NEG	POS	POS	POS	NEG	NEG	NEG	NEG	NEG	NEG	NEG	NEG	NEG	NEG	POS	NEG	POS	NEG	NEG	NEG	POS	POS	POS	POS	POS	NEG	POS	NEG	NEG	NEG	NEG
06V43697	CC22-MRSA-IV, Barnim/UK-EMRSA-15	POS	POS	NEG	NEG	POS	POS	POS	NEG	POS	POS	POS	NEG	NEG	NEG	NEG	NEG	NEG	NEG	NEG	NEG	NEG	POS	NEG	POS	NEG	NEG	NEG	POS	POS	POS	POS	POS	NEG	POS	POS	NEG	NEG	NEG
06V44153	CC22-MRSA-IV, Barnim/UK-EMRSA-15	POS	AMB	NEG	NEG	POS	POS	POS	NEG	POS	POS	POS	NEG	NEG	NEG	NEG	NEG	NEG	NEG	NEG	NEG	NEG	POS	NEG	POS	NEG	NEG	NEG	POS	POS	POS	POS	POS	NEG	POS	NEG	NEG	NEG	NEG
06V45679	CC22-MRSA-IV, Barnim/UK-EMRSA-15	POS	AMB	NEG	NEG	POS	POS	POS	NEG	POS	POS	POS	NEG	NEG	NEG	NEG	NEG	NEG	NEG	NEG	NEG	NEG	POS	NEG	POS	NEG	NEG	NEG	POS	POS	POS	POS	POS	NEG	POS	NEG	NEG	NEG	NEG
06V45831	CC22-MRSA-IV, Barnim/UK-EMRSA-15	POS	AMB	NEG	NEG	POS	POS	POS	NEG	POS	POS	POS	NEG	NEG	NEG	NEG	NEG	NEG	NEG	NEG	NEG	NEG	POS	NEG	POS	NEG	NEG	NEG	POS	POS	POS	POS	POS	NEG	POS	AMB	NEG	NEG	NEG
06V46314	CC22-MRSA-IV, Barnim/UK-EMRSA-15	POS	AMB	NEG	NEG	POS	POS	POS	NEG	POS	POS	POS	NEG	NEG	NEG	NEG	NEG	NEG	NEG	NEG	NEG	NEG	POS	NEG	POS	NEG	NEG	NEG	POS	POS	POS	POS	POS	NEG	POS	POS	NEG	NEG	NEG
06V34729	CC30-MRSA-IV [PVL+], Southwest Pacific Clone	POS	POS	POS	POS	POS	POS	POS	NEG	POS	POS	POS	NEG	NEG	NEG	NEG	NEG	NEG	NEG	NEG	NEG	NEG	POS	NEG	POS	NEG	NEG	POS	POS	POS	POS	POS	NEG						NEG
06B09900	CC45-MRSA-IV, Berlin EMRSA	POS	POS	POS	NEG	POS	POS	NEG	NEG	POS	POS	POS	NEG	NEG	NEG	NEG	NEG	NEG	NEG	NEG	NEG	NEG	POS	NEG	POS	NEG	NEG	NEG	POS	POS	POS	POS	POS	NEG	POS	AMB	NEG	POS	NEG
06V00922	CC45-MRSA-IV, Berlin EMRSA	POS	POS	POS	NEG	AMB	POS	NEG	NEG	POS	POS	POS	NEG	NEG	NEG	NEG	NEG	NEG	NEG	NEG	NEG	NEG	AMB	NEG	POS	NEG	NEG	NEG	POS	POS	POS	POS	AMB	NEG	POS	NEG	NEG	POS	NEG
06V09551	CC45-MRSA-IV, Berlin EMRSA	POS	POS	POS	NEG	POS	POS	NEG	NEG	POS	POS	POS	NEG	NEG	NEG	NEG	NEG	NEG	NEG	NEG	NEG	NEG	POS	NEG	POS	NEG	NEG	NEG	POS	POS	POS	POS	AMB	NEG	POS	AMB	NEG	POS	NEG
06V10719	CC45-MRSA-IV, Berlin EMRSA	POS	POS	POS	NEG	AMB	POS	NEG	NEG	POS	POS	POS	NEG	NEG	NEG	NEG	NEG	NEG	NEG	NEG	NEG	NEG	POS	NEG	POS	NEG	NEG	NEG	POS	POS	POS	POS	AMB	NEG	POS	NEG	NEG	POS	NEG
06V11090	CC45-MRSA-IV, Berlin EMRSA	AMB	AMB	POS	NEG	NEG	AMB	NEG	NEG	POS	POS	POS	NEG	NEG	NEG	NEG	NEG	NEG	NEG	NEG	NEG	NEG	NEG	NEG	POS	NEG	NEG	NEG	POS	POS	POS	POS	AMB	NEG	POS	NEG	NEG	NEG	NEG
06V14383	CC45-MRSA-IV, Berlin EMRSA	POS	POS	POS	NEG	NEG	AMB	NEG	NEG	POS	POS	POS	NEG	NEG	NEG	NEG	NEG	NEG	NEG	NEG	NEG	NEG	AMB	NEG	POS	NEG	NEG	NEG	POS	POS	POS	POS	NEG	NEG	POS	NEG	NEG	NEG	NEG
06V21475	CC45-MRSA-IV, Berlin EMRSA	POS	POS	POS	NEG	NEG	AMB	NEG	NEG	POS	POS	POS	NEG	NEG	NEG	NEG	NEG	NEG	NEG	NEG	NEG	NEG	NEG	NEG	POS	NEG	NEG	NEG	POS	POS	POS	POS	NEG	NEG	POS	NEG	NEG	NEG	NEG
06V24231	CC45-MRSA-IV, Berlin EMRSA	POS	AMB	POS	NEG	NEG	AMB	NEG	NEG	POS	POS	POS	NEG	NEG	NEG	NEG	NEG	NEG	NEG	NEG	NEG	NEG	AMB	NEG	POS	NEG	NEG	NEG	POS	POS	POS	POS	NEG	NEG	POS	NEG	NEG	NEG	NEG
06V32992	CC45-MRSA-IV, Berlin EMRSA	AMB	AMB	POS	NEG	NEG	AMB	NEG	NEG	POS	POS	POS	NEG	NEG	NEG	NEG	NEG	NEG	NEG	NEG	NEG	NEG	NEG	NEG	POS	NEG	NEG	NEG	POS	POS	POS	POS	NEG	NEG	POS	NEG	NEG	NEG	NEG
06V38753	CC45-MRSA-IV, Berlin EMRSA	POS	POS	POS	NEG	NEG	AMB	NEG	NEG	POS	POS	POS	NEG	NEG	NEG	NEG	NEG	NEG	NEG	NEG	NEG	NEG	NEG	NEG	POS	NEG	NEG	NEG	POS	POS	POS	POS	NEG	NEG	POS	NEG	NEG	NEG	NEG
06V39848	CC45-MRSA-IV, Berlin EMRSA	POS	NEG		NEG				NEG	POS			NEG	NEG	NEG	NEG	NEG	NEG								NEG	NEG						POS						
06V42259	CC45-MRSA-IV, Berlin EMRSA	POS	POS	POS	NEG	POS	POS	NEG	NEG	POS	POS	POS	NEG	NEG	NEG	NEG	NEG	NEG	NEG	NEG	NEG	NEG	POS	NEG	POS	NEG	NEG	NEG	POS	POS	POS	POS	POS	NEG	POS	POS	NEG	POS	NEG
06V33960	CC80-MRSA-IV [PVL+], European caMRSA Clone	POS	POS		POS				NEG	POS			NEG	NEG	POS	NEG	POS	NEG								POS	POS						POS						

ISOLATE:	STRAIN	VIRULENCE : STAPHYLOCOCCAL SUPERANTIGEN/ENTEROTOXIN-LIKE GENES (SET/SSL)																					
		staphylococcal superantigen-like protein 2		staphylococcal superantigen-like protein 3			staphylococcal superantigen-like protein 4		staphylococcal superantigen-like protein 5				staphylococcal superantigen-like protein 6		staphylococcal superantigen-like protein 7			staphylococcal superantigen-like protein 8		staphylococcal superantigen-like protein 9			staphylococcal superantigen-like protein 10
		ssl02/set7	ssl02/set7 (MRSA252)	ssl03/set8_probe 1	ssl03/set8_probe 2	ssl03/set8 (MRSA252, SAR0424)	ssl04/set9	ssl04/set9 (MRSA252, SAR0425)	ssl05/set3_probe 1	ssl05/set3 (RF122, probe-611)	ssl05/set3_probe 2 (612)	ssl05/set3 (MRSA252)	ssl06/set21	ssl06 (NCTC8325+MW2)	ssl07/set1	ssl07/set1 (MRSA252)	ssl07/set1 (AF188836)	ssl08/set12_probe 1	ssl08/set12_probe 2	ssl09/set5_probe 1	ssl09/set5_probe 2	ssl09/set5 (MRSA252)	ssl10/set4
**2005**																							
05B01179	CC5/ST228-MRSA-I, South German EMRSA	POS	NEG	POS	POS	NEG	POS	NEG	POS	AMB	POS	NEG	NEG	NEG	POS	AMB	NEG	POS	POS	POS	NEG	NEG	POS
05B02687	CC5/ST228-MRSA-I, South German EMRSA	POS	NEG	POS	POS	NEG	POS	NEG	POS	AMB	POS	NEG	NEG	NEG	POS	AMB	NEG	POS	POS	POS	NEG	NEG	POS
05V08483	CC5/ST228-MRSA-I, South German EMRSA	POS	NEG	POS	POS	NEG	POS	NEG	POS	NEG	POS	NEG	NEG	NEG	POS	NEG	NEG	POS	POS	POS	NEG	NEG	POS
05V19001	CC5/ST228-MRSA-I, South German EMRSA	POS	AMB	POS	POS	NEG	POS	NEG	POS	AMB	POS	NEG	NEG	NEG	AMB	POS	AMB	POS	POS	POS	NEG	NEG	POS
05B04626	CC5-MRSA-II, Rhine-Hesse EMRSA/UK-EMRSA-3	POS	NEG	AMB	POS	NEG	POS	NEG	POS	NEG	POS	NEG	NEG	NEG	POS	NEG	NEG	POS	POS	NEG	NEG	NEG	POS
05B07903	CC5-MRSA-II, Rhine-Hesse EMRSA/UK-EMRSA-3	POS	NEG	POS	POS	NEG	POS	NEG	POS	NEG	POS	NEG	NEG	NEG	POS	AMB	NEG	POS	POS	POS	POS	NEG	POS
05U00062	CC5-MRSA-II, Rhine-Hesse EMRSA/UK-EMRSA-3	POS	NEG	POS	POS	NEG	POS	NEG	POS	NEG	POS	NEG	NEG	NEG	POS	AMB	NEG	POS	POS	POS	POS	NEG	POS
05U03117	CC5-MRSA-II, Rhine-Hesse EMRSA/UK-EMRSA-3	POS	NEG	POS	POS	NEG	POS	NEG	POS	NEG	POS	NEG	NEG	NEG	POS	NEG	NEG	POS	POS	AMB	POS	NEG	POS
05U10392	CC5-MRSA-II, Rhine-Hesse EMRSA/UK-EMRSA-3	POS	NEG	POS	POS	NEG	POS	NEG	POS	NEG	POS	NEG	NEG	NEG	POS	AMB	NEG	POS	POS	POS	POS	NEG	POS
05V03312	CC5-MRSA-II, Rhine-Hesse EMRSA/UK-EMRSA-3	POS	AMB	POS	POS	NEG	POS	NEG	POS	AMB	POS	NEG	NEG	NEG	POS	AMB	AMB	POS	POS	POS	AMB	NEG	POS
05V08095	CC5-MRSA-II, Rhine-Hesse EMRSA/UK-EMRSA-3	POS	AMB	POS	POS	NEG	POS	NEG	POS	AMB	POS	NEG	NEG	NEG	POS	AMB	NEG	POS	POS	POS	POS	NEG	POS
05V09015	CC5-MRSA-II, Rhine-Hesse EMRSA/UK-EMRSA-3	POS	NEG	POS	POS	NEG	POS	NEG	POS	AMB	POS	NEG	NEG	NEG	POS	AMB	NEG	POS	POS	POS	POS	NEG	POS
05V09326	CC5-MRSA-II, Rhine-Hesse EMRSA/UK-EMRSA-3	POS	AMB	POS	POS	NEG	POS	NEG	POS	AMB	POS	NEG	NEG	NEG	POS	AMB	AMB	POS	POS	POS	POS	NEG	POS
05V10265	CC5-MRSA-II, Rhine-Hesse EMRSA/UK-EMRSA-3	POS	AMB	POS	POS	NEG	POS	NEG	POS	AMB	POS	NEG	NEG	NEG	POS	AMB	AMB	POS	POS	POS	NEG	NEG	POS
05V13052	CC5-MRSA-II, Rhine-Hesse EMRSA/UK-EMRSA-3	POS	AMB	POS	AMB	NEG	POS	NEG	POS	NEG	POS	NEG	NEG	NEG	POS	AMB	AMB	POS	POS	POS	AMB	NEG	POS
05V13470	CC5-MRSA-II, Rhine-Hesse EMRSA/UK-EMRSA-3	POS	AMB	POS	POS	NEG	POS	NEG	POS	AMB	POS	NEG	NEG	NEG	POS	AMB	NEG	POS	POS	POS	POS	NEG	POS
05V13891	CC5-MRSA-II, Rhine-Hesse EMRSA/UK-EMRSA-3	POS	NEG	POS	POS	NEG	AMB	NEG	POS	NEG	POS	NEG	NEG	NEG	POS	NEG	NEG	POS	POS	AMB	POS	NEG	POS
05V15616	CC5-MRSA-II, Rhine-Hesse EMRSA/UK-EMRSA-3	POS	NEG	POS		NEG	POS	NEG	POS			NEG	NEG		POS	AMB	AMB	POS		POS		NEG	POS
05V16845	CC5-MRSA-II, Rhine-Hesse EMRSA/UK-EMRSA-3	POS	AMB	POS	POS	NEG	POS	NEG	POS	AMB	POS	NEG	NEG	NEG	POS	AMB	AMB	POS	POS	POS	POS	NEG	POS
05V17541	CC5-MRSA-II, Rhine-Hesse EMRSA/UK-EMRSA-3	POS	NEG	POS	POS	NEG	AMB	NEG	POS	NEG	POS	NEG	NEG	NEG	POS	NEG	NEG	POS	POS	AMB	POS	NEG	POS
05V19105	CC5-MRSA-II, Rhine-Hesse EMRSA/UK-EMRSA-3	POS	POS	POS		NEG	POS	NEG	POS			NEG	NEG		POS	AMB	AMB	POS		POS		NEG	POS
05V22148	CC5-MRSA-II, Rhine-Hesse EMRSA/UK-EMRSA-3	POS	AMB	POS	POS	NEG	POS	NEG	POS	AMB	POS	NEG	NEG	NEG	POS	AMB	AMB	POS	POS	POS	POS	NEG	POS
05V33740	CC5-MRSA-II, Rhine-Hesse EMRSA/UK-EMRSA-3	POS	NEG	POS	POS	NEG	POS	NEG	POS	NEG	POS	NEG	NEG	NEG	POS	AMB	NEG	POS	POS	POS	POS	NEG	POS
05V38425	CC5-MRSA-II, Rhine-Hesse EMRSA/UK-EMRSA-3	POS	NEG	POS	POS	NEG	POS	NEG	POS	NEG	POS	NEG	NEG	NEG	POS	AMB	NEG	POS	POS	POS	POS	NEG	POS
05V39987	CC5-MRSA-II, Rhine-Hesse EMRSA/UK-EMRSA-3	POS	NEG	POS	POS	NEG	POS	NEG	POS	NEG	POS	NEG	NEG	NEG	POS	AMB	NEG	POS	POS	POS	POS	NEG	POS
05F00089	CC5-MRSA-IV, Paediatric clone	POS	AMB	POS	POS	NEG	POS	NEG	POS	NEG	POS	NEG	NEG	NEG	POS	AMB	NEG	POS	POS	POS	NEG	NEG	POS
05V01416	CC5-MRSA-IV, Paediatric clone	POS	NEG	POS	POS	NEG	NEG	NEG	AMB	NEG	POS	NEG	NEG	NEG	POS	NEG	NEG	POS	POS	NEG	AMB	NEG	POS
05V16386	CC8-MRSA-IV, USA300	POS	NEG	POS	POS	NEG	POS	NEG	POS	AMB	POS	NEG	NEG	POS	POS	AMB	NEG	POS	POS	POS	NEG	NEG	POS
05V04920	CC8-MRSA-IV, USA500	POS	NEG	POS	POS	NEG	POS	NEG	POS	NEG	POS	NEG	NEG	POS	POS	AMB	AMB	POS	POS	POS	NEG	NEG	POS
05V21159	CC8-MRSA-IV, USA500	POS	NEG	POS	POS	NEG	POS	NEG	POS	AMB	POS	NEG	NEG	POS	POS	AMB	NEG	POS	POS	POS	NEG	NEG	POS
05B00867	CC22-MRSA-IV, Barnim/UK-EMRSA-15	POS	POS	NEG	NEG	NEG	NEG	NEG	POS	NEG	NEG	POS	NEG	NEG	AMB	AMB	POS	NEG	NEG	AMB	POS	NEG	NEG
05V03174	CC22-MRSA-IV, Barnim/UK-EMRSA-15	AMB	POS	NEG	NEG	NEG	NEG	POS	POS	NEG	NEG	POS	NEG	NEG	AMB	AMB	POS	NEG	NEG	POS	POS	NEG	POS
05V07331	CC22-MRSA-IV, Barnim/UK-EMRSA-15	AMB	POS	NEG	NEG	NEG	NEG	AMB	POS	POS	NEG	POS	NEG	AMB	AMB	AMB	POS	NEG	NEG	POS	POS	NEG	POS
05V07371	CC22-MRSA-IV, Barnim/UK-EMRSA-15	AMB	POS	NEG	NEG	NEG	NEG	POS	POS	NEG	NEG	POS	NEG	NEG	AMB	AMB	POS	NEG	NEG	POS	POS	NEG	NEG
05V08145	CC22-MRSA-IV, Barnim/UK-EMRSA-15	AMB	POS	NEG	NEG	NEG	NEG	POS	POS	NEG	NEG	POS	NEG	NEG	NEG	AMB	POS	NEG	NEG	NEG	NEG	NEG	NEG
05V08595	CC22-MRSA-IV, Barnim/UK-EMRSA-15	AMB	POS	NEG	NEG	NEG	NEG	POS	POS	NEG	NEG	POS	NEG	NEG	AMB	AMB	POS	NEG	NEG	POS	POS	NEG	POS
05V12803	CC22-MRSA-IV, Barnim/UK-EMRSA-15	AMB	POS	NEG	NEG	NEG	NEG	POS	POS	NEG	NEG	POS	NEG	AMB	AMB	AMB	POS	NEG	NEG	POS	POS	NEG	POS
05V14596	CC22-MRSA-IV, Barnim/UK-EMRSA-15	POS	AMB	NEG	NEG	NEG	NEG	NEG	POS	NEG	NEG	POS	NEG	NEG	AMB	POS	AMB	NEG	NEG	AMB	POS	NEG	POS
05V16691	CC22-MRSA-IV, Barnim/UK-EMRSA-15	POS	POS	NEG		POS	NEG	POS	POS			POS	NEG		AMB	AMB	POS	NEG		POS		NEG	NEG
05V18550	CC22-MRSA-IV, Barnim/UK-EMRSA-15	AMB	POS	NEG	NEG	NEG	NEG	POS	POS	POS	NEG	POS	NEG	AMB	AMB	AMB	POS	NEG	NEG	POS	POS	NEG	POS
05V19034	CC22-MRSA-IV, Barnim/UK-EMRSA-15	AMB	POS	NEG	NEG	NEG	NEG	POS	POS	NEG	NEG	POS	NEG	AMB	AMB	AMB	POS	NEG	NEG	POS	POS	NEG	POS
05V19333	CC22-MRSA-IV, Barnim/UK-EMRSA-15	POS	POS	NEG		NEG	NEG	POS	POS			POS	NEG		AMB	AMB	POS	NEG		POS		NEG	AMB
05V19701	CC22-MRSA-IV, Barnim/UK-EMRSA-15	AMB	POS	NEG	NEG	NEG	NEG	POS	POS	NEG	NEG	POS	NEG	NEG	AMB	AMB	POS	NEG	NEG	POS	POS	NEG	NEG
05V20072	CC22-MRSA-IV, Barnim/UK-EMRSA-15	AMB	POS	NEG	NEG	NEG	NEG	POS	POS	NEG	NEG	POS	NEG	NEG	AMB	AMB	POS	NEG	NEG	POS	POS	NEG	NEG
05V24930	CC22-MRSA-IV, Barnim/UK-EMRSA-15	AMB	POS	NEG	NEG	NEG	NEG	POS	POS	NEG	NEG	POS	NEG	NEG	AMB	AMB	POS	NEG	NEG	POS	POS	NEG	POS
05V31461	CC22-MRSA-IV, Barnim/UK-EMRSA-15	AMB	POS	NEG	NEG	AMB	NEG	POS	POS	NEG	NEG	POS	NEG	AMB	AMB	AMB	POS	NEG	NEG	POS	POS	NEG	POS
05B05671	CC45-MRSA-IV, Berlin EMRSA	POS	POS	NEG	NEG	NEG	NEG	POS	NEG	NEG	NEG	AMB	NEG	AMB	NEG	NEG	NEG	NEG	NEG	NEG	NEG	POS	NEG
05V01452	CC45-MRSA-IV, Berlin EMRSA	AMB	POS	NEG	NEG	NEG	NEG	POS	NEG	NEG	NEG	POS	NEG	AMB	NEG	NEG	NEG	NEG	NEG	NEG	NEG	POS	AMB
05V01617	CC45-MRSA-IV, Berlin EMRSA	AMB	POS	NEG	NEG	NEG	NEG	POS	NEG	NEG	NEG	POS	NEG	POS	NEG	NEG	NEG	NEG	NEG	NEG	NEG	POS	AMB
05V16986	CC45-MRSA-IV, Berlin EMRSA	NEG	POS	NEG	NEG	NEG	NEG	POS	NEG	NEG	NEG	AMB	NEG	NEG	NEG	NEG	NEG	NEG	NEG	NEG	NEG	POS	AMB
05V18357	CC45-MRSA-IV, Berlin EMRSA	NEG	POS	NEG	NEG	NEG	NEG	POS	NEG	NEG	NEG	POS	NEG	NEG	NEG	NEG	NEG	NEG	NEG	NEG	NEG	POS	NEG
05V23111	CC45-MRSA-IV, Berlin EMRSA	AMB	POS	NEG	NEG	NEG	NEG	POS	NEG	NEG	NEG	POS	NEG	POS	NEG	NEG	NEG	NEG	NEG	NEG	NEG	POS	AMB
05V23414	CC45-MRSA-IV, Berlin EMRSA	NEG	POS	NEG		NEG	NEG	POS	NEG			POS	NEG		NEG	NEG	NEG	NEG		NEG		POS	POS
05V23858	CC45-MRSA-IV, Berlin EMRSA	NEG	POS	NEG	NEG	NEG	NEG	POS	NEG	NEG	NEG	POS	NEG	NEG	NEG	NEG	NEG	NEG	NEG	NEG	NEG	POS	AMB
05V26241	CC45-MRSA-IV, Berlin EMRSA	NEG	POS	NEG	NEG	NEG	NEG	POS	NEG	NEG	NEG	POS	NEG	NEG	NEG	NEG	NEG	NEG	NEG	NEG	NEG	POS	AMB
05V28399	CC45-MRSA-IV, Berlin EMRSA	AMB	POS	NEG	NEG	NEG	NEG	POS	NEG	NEG	NEG	POS	NEG	POS	NEG	NEG	NEG	NEG	NEG	NEG	NEG	POS	AMB
05V32647	CC45-MRSA-IV, Berlin EMRSA	NEG	POS	NEG		NEG	NEG	NEG	NEG			NEG	NEG		NEG	NEG	NEG	NEG		NEG		POS	NEG
05V37969	CC45-MRSA-IV, Berlin EMRSA	NEG	POS	NEG	NEG	NEG	NEG	NEG	NEG	NEG	NEG	AMB	NEG	AMB	NEG	NEG	NEG	NEG	NEG	NEG	NEG	POS	AMB
05U10018	CC398-MRSA-V	NEG	POS	NEG	NEG	NEG	NEG	POS	NEG	NEG	NEG	POS	NEG	NEG	AMB	AMB	POS	NEG	NEG	NEG	NEG	NEG	NEG
**2006**																							
06V36353	CC1-MRSA-IV, WA MRSA-1/57	POS	AMB	POS	POS	NEG	POS	NEG	POS	NEG	POS	NEG	POS	POS	POS	AMB	AMB	POS	POS	POS	NEG	NEG	POS
06V40202	CC5/ST228-MRSA-I, South German EMRSA	POS	AMB	POS		NEG	POS	NEG	POS			NEG	NEG		POS	AMB	NEG	POS		POS		NEG	POS
06V41266	CC5/ST228-MRSA-I, South German EMRSA	POS	NEG	POS	POS	NEG	POS	NEG	POS	NEG	POS	NEG	NEG	NEG	POS	AMB	NEG	POS	POS	POS		NEG	POS
06B09097	CC5-MRSA-II, Rhine-Hesse EMRSA/UK-EMRSA-3	POS	NEG	POS		NEG	POS	NEG	POS			NEG	NEG		POS	AMB	NEG	POS		POS		NEG	POS
06F00244	CC5-MRSA-II, Rhine-Hesse EMRSA/UK-EMRSA-3	POS	AMB	POS	POS	NEG	POS	NEG	POS	AMB	POS	NEG	NEG	NEG	POS	AMB	AMB	POS	POS	POS	POS	NEG	POS
06V03913	CC5-MRSA-II, Rhine-Hesse EMRSA/UK-EMRSA-3	POS	AMB	POS	POS	NEG	POS	NEG	POS	AMB	POS	NEG	NEG	NEG	POS	AMB	AMB	POS	POS	POS	POS	NEG	POS
06V04337	CC5-MRSA-II, Rhine-Hesse EMRSA/UK-EMRSA-3	POS	AMB	POS	POS	NEG	POS	NEG	NEG	AMB	POS	NEG	NEG	NEG	POS	AMB	AMB	POS	POS	POS	POS	NEG	POS
06V06785	CC5-MRSA-II, Rhine-Hesse EMRSA/UK-EMRSA-3	POS	AMB	POS	POS	NEG	POS	AMB	POS	AMB	POS	NEG	NEG	NEG	POS	AMB	AMB	POS	POS	POS	POS	NEG	POS
06V12258	CC5-MRSA-II, Rhine-Hesse EMRSA/UK-EMRSA-3	POS	AMB	POS	POS	NEG	POS	AMB	POS	AMB	POS	NEG	NEG	NEG	POS	AMB	AMB	POS	POS	POS	POS	NEG	POS
06V20553	CC5-MRSA-II, Rhine-Hesse EMRSA/UK-EMRSA-3	POS	AMB	POS	POS	NEG	POS	NEG	POS	AMB	POS	NEG	NEG	NEG	POS	AMB	AMB	POS	POS	POS	POS	NEG	POS
06V22564	CC5-MRSA-II, Rhine-Hesse EMRSA/UK-EMRSA-3	POS	POS	POS		NEG	POS	NEG	POS			NEG	NEG		POS	AMB	AMB	POS		POS		NEG	POS
06V23750	CC5-MRSA-II, Rhine-Hesse EMRSA/UK-EMRSA-3	POS	AMB	POS	POS	NEG	POS	NEG	POS	AMB	POS	NEG	NEG	NEG	POS	AMB	NEG	POS	POS	POS	POS	NEG	POS
06V27332	CC5-MRSA-II, Rhine-Hesse EMRSA/UK-EMRSA-3	POS	AMB	POS	POS	NEG	POS	NEG	POS	AMB	POS	NEG	NEG	NEG	POS	AMB	NEG	POS	POS	POS	POS	NEG	POS
06V29484	CC5-MRSA-II, Rhine-Hesse EMRSA/UK-EMRSA-3	POS	NEG	POS	POS	NEG	POS	NEG	POS	AMB	POS	NEG	NEG	NEG	POS	AMB	NEG	POS	POS	POS	POS	NEG	POS
06V31278	CC5-MRSA-II, Rhine-Hesse EMRSA/UK-EMRSA-3	POS	POS	POS		NEG	POS	NEG	POS			NEG	NEG		POS	AMB	AMB	POS		POS		NEG	POS
06V36043	CC5-MRSA-II, Rhine-Hesse EMRSA/UK-EMRSA-3	POS	NEG	POS	POS	NEG	POS	NEG	POS	AMB	POS	NEG	NEG	NEG	POS	NEG	NEG	POS	POS	POS	NEG	NEG	POS
06V36349	CC5-MRSA-II, Rhine-Hesse EMRSA/UK-EMRSA-3	POS	NEG	POS	POS	NEG	POS	NEG	POS	AMB	POS	NEG	NEG	NEG	POS	AMB	NEG	POS	POS	POS	POS	NEG	POS
06V39695	CC5-MRSA-II, Rhine-Hesse EMRSA/UK-EMRSA-3	POS	NEG	POS	POS	NEG	POS	NEG	POS	NEG	POS	NEG	NEG	NEG	POS	NEG	NEG	POS	POS	POS	POS	NEG	POS
06V40571	CC5-MRSA-II, Rhine-Hesse EMRSA/UK-EMRSA-3	POS	AMB	POS		NEG	POS	NEG	POS			NEG	NEG		POS	AMB	AMB	POS		POS		NEG	POS
06V42188	CC5-MRSA-II, Rhine-Hesse EMRSA/UK-EMRSA-3	POS	AMB	POS	POS	NEG	POS	NEG	POS	AMB	POS	NEG	NEG	NEG	POS	AMB	AMB	POS	POS	POS	POS	NEG	POS
06V42146	CC8-MRSA-IV, Lyon Clone/UK-EMRSA-2	POS	NEG	POS		NEG	POS	NEG	POS			NEG	NEG		POS	NEG	AMB	POS		POS		NEG	AMB
06V28560	CC8-MRSA-V	POS	AMB	POS	POS	NEG	POS	NEG	POS	POS	POS	NEG	AMB	POS	POS	NEG	NEG	POS	POS	POS	AMB	NEG	POS
06F00837	CC22-MRSA-IV, Barnim/UK-EMRSA-15	AMB	POS	NEG	NEG	NEG	NEG	AMB	POS	NEG	NEG	POS	NEG	NEG	AMB	AMB	POS	NEG	NEG	POS	POS	NEG	POS
06F01638	CC22-MRSA-IV, Barnim/UK-EMRSA-15	AMB	POS	NEG	NEG	AMB	NEG	POS	POS	NEG	NEG	POS	NEG	AMB	AMB	AMB	POS	NEG	NEG	POS	POS	NEG	POS
06F04643	CC22-MRSA-IV, Barnim/UK-EMRSA-15	POS	POS	NEG		NEG	NEG	NEG	POS			POS	NEG		AMB	AMB	POS	NEG		NEG		NEG	NEG
06F05580	CC22-MRSA-IV, Barnim/UK-EMRSA-15	AMB	POS	NEG	NEG	NEG	NEG	POS	POS	NEG	NEG	POS	NEG	NEG	AMB	AMB	POS	NEG	NEG	AMB	AMB	NEG	NEG
06U05469	CC22-MRSA-IV, Barnim/UK-EMRSA-15	AMB	POS	NEG	NEG	NEG	NEG	POS	POS	NEG	NEG	POS	NEG	NEG	AMB	AMB	POS	NEG	NEG	POS	POS	NEG	POS
06U17314	CC22-MRSA-IV, Barnim/UK-EMRSA-15	AMB	POS	NEG	NEG	NEG	NEG	AMB	POS	NEG	NEG	POS	NEG	NEG	AMB	AMB	POS	NEG	NEG	POS	POS	NEG	POS
06V08377	CC22-MRSA-IV, Barnim/UK-EMRSA-15	AMB	POS	NEG	NEG	NEG	NEG	POS	POS	NEG	NEG	POS	NEG	AMB	AMB	AMB	POS	NEG	NEG	POS	POS	NEG	POS
06V08474	CC22-MRSA-IV, Barnim/UK-EMRSA-15	AMB	POS	NEG	NEG	NEG	NEG	AMB	POS	NEG	NEG	POS	NEG	NEG	AMB	AMB	POS	NEG	NEG	POS	POS	NEG	POS
06V09374	CC22-MRSA-IV, Barnim/UK-EMRSA-15	AMB	POS	AMB	NEG	AMB	NEG	POS	POS	POS	NEG	POS	NEG	AMB	AMB	AMB	POS	NEG	NEG	POS	POS	NEG	POS
06V09605	CC22-MRSA-IV, Barnim/UK-EMRSA-15	AMB	POS	NEG	NEG	NEG	NEG	AMB	POS	NEG	NEG	POS	NEG	NEG	AMB	AMB	POS	NEG	NEG	POS	POS	NEG	POS
06V18687	CC22-MRSA-IV, Barnim/UK-EMRSA-15	AMB	POS	NEG	NEG	NEG	NEG	POS	POS	NEG	NEG	POS	NEG	NEG	NEG	AMB	POS	NEG	NEG	POS	POS	NEG	NEG
06V21697	CC22-MRSA-IV, Barnim/UK-EMRSA-15	AMB	POS	NEG	NEG	AMB	NEG	POS	POS	POS	NEG	POS	NEG	POS	AMB	POS	AMB	NEG	NEG	POS	POS	NEG	POS
06V23749	CC22-MRSA-IV, Barnim/UK-EMRSA-15	AMB	POS	NEG	NEG	NEG	NEG	AMB	POS	NEG	NEG	POS	NEG	NEG	AMB	AMB	POS	NEG	NEG	POS	POS	NEG	POS
06V29915	CC22-MRSA-IV, Barnim/UK-EMRSA-15	AMB	POS	NEG	NEG	AMB	NEG	POS	POS	POS	NEG	POS	NEG	AMB	AMB	AMB	POS	NEG	NEG	POS	POS	NEG	POS
06V31778	CC22-MRSA-IV, Barnim/UK-EMRSA-15	AMB	POS	AMB	AMB	NEG	NEG	POS	POS	NEG	NEG	POS	NEG	AMB	AMB	AMB	POS	NEG	NEG	POS	POS	NEG	POS
06V39089	CC22-MRSA-IV, Barnim/UK-EMRSA-15	AMB	POS	NEG	NEG	NEG	NEG	POS	POS	NEG	NEG	POS	NEG	NEG	AMB	AMB	POS	NEG	NEG	POS	POS	NEG	NEG
06V41512	CC22-MRSA-IV, Barnim/UK-EMRSA-15	POS	POS	NEG		POS	NEG	POS	POS			POS	NEG		AMB	AMB	POS	NEG		NEG		NEG	NEG
06V42186	CC22-MRSA-IV, Barnim/UK-EMRSA-15	AMB	POS	NEG	NEG	NEG	NEG	POS	POS	NEG	NEG	POS	NEG	NEG	AMB	AMB	POS	NEG	NEG	AMB	POS	NEG	NEG
06V42236	CC22-MRSA-IV, Barnim/UK-EMRSA-15	AMB	POS	NEG	NEG	NEG	NEG	POS	POS	NEG	NEG	POS	NEG	NEG	AMB	AMB	POS	NEG	NEG	POS	POS	NEG	NEG
06V43697	CC22-MRSA-IV, Barnim/UK-EMRSA-15	AMB	POS	NEG	NEG	NEG	NEG	POS	POS	POS	NEG	POS	NEG	AMB	AMB	AMB	POS	NEG	NEG	POS	POS	NEG	POS
06V44153	CC22-MRSA-IV, Barnim/UK-EMRSA-15	AMB	POS	NEG	NEG	NEG	NEG	AMB	POS	NEG	NEG	POS	NEG	NEG	NEG	AMB	POS	NEG	NEG	AMB	POS	NEG	NEG
06V45679	CC22-MRSA-IV, Barnim/UK-EMRSA-15	AMB	POS	NEG	NEG	NEG	NEG	AMB	POS	NEG	NEG	POS	NEG	NEG	NEG	AMB	POS	NEG	NEG	AMB	POS	NEG	NEG
06V45831	CC22-MRSA-IV, Barnim/UK-EMRSA-15	AMB	POS	NEG	NEG	NEG	NEG	POS	POS	NEG	NEG	POS	NEG	NEG	NEG	AMB	POS	NEG	NEG	AMB	AMB	NEG	NEG
06V46314	CC22-MRSA-IV, Barnim/UK-EMRSA-15	AMB	POS	NEG	NEG	NEG	NEG	AMB	POS	NEG	NEG	POS	NEG	NEG	AMB	AMB	POS	NEG	NEG	POS	POS	NEG	NEG
06V34729	CC30-MRSA-IV [PVL+], Southwest Pacific Clone	AMB	POS	NEG	NEG	POS	AMB	POS	NEG	POS	NEG	POS	NEG	POS	AMB	POS	AMB	NEG	NEG	NEG	NEG	POS	NEG
06B09900	CC45-MRSA-IV, Berlin EMRSA	AMB	POS	NEG	NEG	NEG	NEG	POS	NEG	NEG	NEG	POS	NEG	NEG	NEG	NEG	NEG	NEG	NEG	NEG	NEG	POS	AMB
06V00922	CC45-MRSA-IV, Berlin EMRSA	AMB	POS	NEG	NEG	NEG	NEG	POS	NEG	NEG	NEG	POS	NEG	NEG	NEG	NEG	NEG	NEG	NEG	NEG	NEG	POS	AMB
06V09551	CC45-MRSA-IV, Berlin EMRSA	NEG	POS	NEG	NEG	NEG	NEG	POS	NEG	NEG	NEG	POS	NEG	AMB	NEG	NEG	NEG	NEG	NEG	NEG	NEG	POS	AMB
06V10719	CC45-MRSA-IV, Berlin EMRSA	NEG	POS	NEG	NEG	NEG	NEG	POS	NEG	NEG	NEG	POS	NEG	NEG	NEG	NEG	NEG	NEG	NEG	NEG	NEG	POS	AMB
06V11090	CC45-MRSA-IV, Berlin EMRSA	NEG	POS	NEG	NEG	NEG	NEG	AMB	NEG	NEG	NEG	POS	NEG	NEG	NEG	NEG	NEG	NEG	NEG	NEG	NEG	POS	AMB
06V14383	CC45-MRSA-IV, Berlin EMRSA	NEG	POS	NEG	NEG	NEG	NEG	POS	NEG	NEG	NEG	POS	NEG	NEG	NEG	NEG	NEG	NEG	NEG	NEG	NEG	POS	AMB
06V21475	CC45-MRSA-IV, Berlin EMRSA	NEG	POS	NEG	NEG	NEG	NEG	POS	NEG	NEG	NEG	POS	NEG	NEG	NEG	NEG	NEG	NEG	NEG	NEG	NEG	POS	AMB
06V24231	CC45-MRSA-IV, Berlin EMRSA	NEG	POS	NEG	NEG	NEG	NEG	POS	NEG	NEG	NEG	POS	NEG	NEG	NEG	NEG	NEG	NEG	NEG	NEG	NEG	POS	AMB
06V32992	CC45-MRSA-IV, Berlin EMRSA	NEG	POS	NEG	NEG	NEG	NEG	AMB	NEG	NEG	NEG	POS	NEG	NEG	NEG	NEG	NEG	NEG	NEG	NEG	NEG	POS	AMB
06V38753	CC45-MRSA-IV, Berlin EMRSA	NEG	POS	NEG	NEG	NEG	NEG	POS	NEG	NEG	NEG	POS	NEG	NEG	NEG	NEG	NEG	NEG	NEG	NEG	NEG	POS	AMB
06V39848	CC45-MRSA-IV, Berlin EMRSA	AMB	POS	NEG		NEG	NEG	AMB	NEG			NEG	NEG		NEG	NEG	NEG	NEG		NEG		POS	NEG
06V42259	CC45-MRSA-IV, Berlin EMRSA	AMB	POS	NEG	NEG	NEG	NEG	POS	NEG	NEG	NEG	POS	NEG	POS	NEG	NEG	NEG	NEG	NEG	NEG	NEG	POS	POS
06V33960	CC80-MRSA-IV [PVL+], European caMRSA Clone	POS	amb	POS		NEG	POS	NEG	POS			amb	NEG		pos	AMB	AMB	POS		POS		NEG	POS

ISOLATE:	VIRULENCE : STAPHYLOCOCCAL SUPERANTIGEN/ENTEROTOXIN-LIKE GENES (SET/SSL) (continued)											CAPSULE- AND BIOFILM-ASSOCIATED GENES																
	staphylococcal superantigen-like protein 10		staphylococcal superantigene-like protein 11				staphylococcal exotoxin-like protein, second locus					Capsule type 1	capsular poly-saccharide synthesis enzyme	O-antigen poly-merase	capsular poly-saccharide biosyn- thesis protein	Capsule type 5	capsular poly-saccharide synthesis enzyme	O-antigen poly-merase	capsular poly-saccharide biosyn- thesis protein	Capsule type 8	capsular poly-saccharide synthesis enzyme	capsular poly-saccharide biosyn- thesis protein	O-antigen poly-merase	capsular poly-saccharide biosyn- thesis protein	intercellular adhesion protein A	intercellular adhesion protein C	biofilm PIA synthesis protein D	surface protein involved in biofilm formation
	ssl10 (RF122)	ssl10/set4 (MRSA252)	ssl11/set2 (COL)	ssl11+set2(Mu50+N315)	ssl11+set2(MW2+MSSA 476)	ssl11/set2 (MRSA252)	setB3	setB3 (MRSA252)	setB2	setB2 (MRSA252)	setB1	cap 1 (total)	capH1	capJ1	capK1	cap 5 (total)	capH5	capJ5	capK5	cap 8 (total)	capH8	capI8	capJ8	capK8	icaA	icaC	icaD	bap
**2005**																												
05B01179	NEG	NEG	NEG	POS	NEG	NEG	POS	NEG	POS	NEG	POS	NEG	NEG	NEG	NEG	POS	POS	POS	POS	NEG	NEG	NEG	NEG	NEG	POS	POS	POS	NEG
05B02687	NEG	NEG	NEG	POS	NEG	NEG	POS	NEG	POS	NEG	POS	NEG	NEG	NEG	NEG	POS	POS	POS	POS	NEG	NEG	NEG	NEG	NEG	POS	POS	POS	NEG
05V08483	NEG	NEG	NEG	POS	NEG	NEG	POS	NEG	POS	NEG	POS	NEG	NEG	NEG	NEG	POS	POS	POS	POS	NEG	NEG	NEG	NEG	NEG	POS	POS	POS	NEG
05V19001	NEG	AMB	NEG	POS	NEG	NEG	POS	NEG	POS	NEG	POS	NEG	NEG	NEG	NEG	POS	POS	POS	POS	NEG	NEG	NEG	NEG	NEG	POS	POS	POS	NEG
05B04626	NEG	NEG	NEG	POS	NEG	NEG	POS	NEG	POS	NEG	POS	NEG	NEG	NEG	NEG	POS	POS	POS	POS	NEG	NEG	NEG	NEG	NEG	POS	POS	POS	NEG
05B07903	NEG	NEG	NEG	POS	NEG	NEG	POS	NEG	POS	NEG	POS	NEG	NEG	NEG	NEG	POS	POS	POS	POS	NEG	NEG	NEG	NEG	NEG	POS	POS	POS	NEG
05U00062	NEG	NEG	NEG	POS	NEG	NEG	POS	NEG	POS	NEG	POS	NEG	NEG	NEG	NEG	POS	POS	POS	POS	NEG	NEG	NEG	NEG	NEG	POS	POS	POS	NEG
05U03117	NEG	NEG	NEG	POS	NEG	NEG	POS	NEG	POS	NEG	POS	NEG	NEG	NEG	NEG	POS	POS	POS	POS	NEG	NEG	NEG	NEG	NEG	POS	POS	POS	NEG
05U10392	NEG	NEG	NEG	POS	NEG	NEG	POS	NEG	POS	NEG	POS	NEG	NEG	NEG	NEG	POS	POS	POS	POS	NEG	NEG	NEG	NEG	NEG	POS	POS	POS	NEG
05V03312	AMB	AMB	NEG	POS	NEG	NEG	POS	NEG	POS	NEG	AMB	NEG	NEG	NEG	NEG	POS	POS	POS	POS	NEG	NEG	NEG	NEG	NEG	POS	POS	POS	NEG
05V08095	NEG	AMB	NEG	POS	NEG	NEG	POS	NEG	POS	NEG	POS	NEG	NEG	NEG	NEG	POS	POS	POS	POS	NEG	NEG	NEG	NEG	NEG	POS	POS	POS	NEG
05V09015	NEG	NEG	NEG	POS	NEG	NEG	POS	NEG	POS	NEG	POS	NEG	NEG	NEG	NEG	POS	POS	POS	POS	NEG	NEG	NEG	NEG	NEG	POS	POS	POS	NEG
05V09326	AMB	AMB	NEG	POS	NEG	NEG	POS	NEG	POS	NEG	POS	NEG	NEG	NEG	NEG	POS	POS	POS	POS	NEG	NEG	NEG	NEG	NEG	POS	POS	POS	NEG
05V10265	NEG	AMB	NEG	POS	NEG	NEG	POS	NEG	POS	NEG	POS	NEG	NEG	NEG	NEG	POS	POS	POS	POS	NEG	NEG	NEG	NEG	NEG	POS	POS	POS	NEG
05V13052	NEG	AMB	NEG	POS	NEG	NEG	POS	NEG	POS	NEG	POS	NEG	NEG	NEG	NEG	POS	POS	POS	POS	NEG	NEG	NEG	NEG	NEG	POS	POS	POS	NEG
05V13470	NEG	NEG	NEG	POS	NEG	NEG	POS	NEG	POS	NEG	POS	NEG	NEG	NEG	NEG	POS	POS	POS	POS	NEG	NEG	NEG	NEG	NEG	POS	POS	POS	NEG
05V13891	NEG	NEG	NEG	POS	NEG	NEG	POS	NEG	POS	NEG	POS	NEG	NEG	NEG	NEG	POS	POS	POS	POS	NEG	NEG	NEG	NEG	NEG	POS	POS	POS	NEG
05V15616		NEG	NEG	POS	NEG	NEG	POS	NEG	POS	NEG	POS																	
05V16845	NEG	NEG	NEG	POS	NEG	NEG	POS	NEG	POS	NEG	POS	NEG	NEG	NEG	NEG	POS	POS	POS	POS	NEG	NEG	NEG	NEG	NEG	POS	POS	POS	NEG
05V17541	NEG	NEG	NEG	POS	NEG	NEG	POS	NEG	POS	NEG	POS	NEG	NEG	NEG	NEG	POS	POS	POS	POS	NEG	NEG	NEG	NEG	NEG	POS	POS	POS	NEG
05V19105		NEG	NEG	POS	NEG	NEG	POS	NEG	POS	NEG	POS																	
05V22148	NEG	AMB	NEG	POS	NEG	NEG	POS	NEG	POS	NEG	POS	NEG	NEG	NEG	NEG	POS	POS	POS	POS	NEG	NEG	NEG	NEG	NEG	POS	POS	POS	NEG
05V33740	NEG	NEG	NEG	POS	NEG	NEG	POS	NEG	POS	NEG	POS	NEG	NEG	NEG	NEG	POS	POS	POS	POS	NEG	NEG	NEG	NEG	NEG	POS	POS	POS	NEG
05V38425	NEG	NEG	NEG	POS	NEG	NEG	POS	NEG	POS	NEG	POS	NEG	NEG	NEG	NEG	POS	POS	POS	POS	NEG	NEG	NEG	NEG	NEG	POS	POS	POS	NEG
05V39987	NEG	NEG	NEG	POS	NEG	NEG	POS	NEG	POS	NEG	POS	NEG	NEG	NEG	NEG	POS	POS	POS	POS	NEG	NEG	NEG	NEG	NEG	POS	POS	POS	NEG
05F00089	NEG	NEG	NEG	POS	NEG	NEG	POS	NEG	POS	NEG	POS	NEG	NEG	NEG	NEG	POS	POS	POS	POS	NEG	NEG	NEG	NEG	NEG	POS	POS	POS	NEG
05V01416	NEG	NEG	NEG	POS	NEG	NEG	AMB	NEG	AMB	NEG	AMB	NEG	NEG	NEG	NEG	POS	POS	POS	POS	NEG	NEG	NEG	NEG	NEG	POS	POS	POS	NEG
05V16386	NEG	NEG	POS	AMB	NEG	NEG	POS	NEG	POS	NEG	POS	NEG	NEG	NEG	NEG	POS	POS	POS	POS	NEG	NEG	NEG	NEG	NEG	POS	POS	POS	NEG
05V04920	NEG	NEG	POS	NEG	NEG	NEG	POS	NEG	POS	NEG	POS	NEG	NEG	NEG	NEG	POS	POS	POS	POS	NEG	NEG	NEG	NEG	NEG	POS	POS	POS	NEG
05V21159	NEG	NEG	POS	NEG	NEG	NEG	POS	NEG	POS	NEG	POS	NEG	NEG	NEG	NEG	POS	POS	POS	POS	NEG	NEG	NEG	NEG	NEG	POS	POS	POS	NEG
05B00867	AMB	NEG	NEG	NEG	NEG	NEG	NEG	NEG	NEG	NEG	NEG	NEG	NEG	NEG	NEG	POS	POS	POS	POS	NEG	NEG	NEG	NEG	NEG	POS	POS	POS	NEG
05V03174	NEG	NEG	NEG	NEG	NEG	NEG	NEG	NEG	NEG	NEG	NEG	NEG	NEG	NEG	NEG	POS	POS	POS	POS	NEG	NEG	NEG	NEG	NEG	POS	POS	POS	NEG
05V07331	NEG	AMB	NEG	NEG	NEG	NEG	NEG	NEG	NEG	NEG	NEG	NEG	NEG	NEG	NEG	POS	POS	POS	POS	NEG	NEG	NEG	NEG	NEG	POS	POS	POS	NEG
05V07371	NEG	NEG	NEG	NEG	NEG	NEG	NEG	NEG	NEG	NEG	NEG	NEG	NEG	NEG	NEG	POS	POS	POS	POS	NEG	NEG	NEG	NEG	NEG	POS	POS	POS	NEG
05V08145	NEG	NEG	NEG	NEG	NEG	NEG	NEG	NEG	NEG	NEG	NEG	NEG	NEG	NEG	NEG	POS	POS	POS	POS	NEG	NEG	NEG	NEG	NEG	POS	AMB	POS	NEG
05V08595	NEG	NEG	NEG	NEG	NEG	NEG	NEG	NEG	NEG	NEG	NEG	NEG	NEG	NEG	NEG	POS	POS	POS	POS	NEG	NEG	NEG	NEG	NEG	POS	POS	POS	NEG
05V12803	NEG	NEG	NEG	NEG	NEG	NEG	NEG	NEG	NEG	NEG	NEG	NEG	NEG	NEG	NEG	POS	POS	POS	POS	NEG	NEG	NEG	NEG	NEG	POS	POS	POS	NEG
05V14596	NEG	AMB	NEG	NEG	NEG	NEG	NEG	NEG	NEG	NEG	NEG	NEG	NEG	NEG	NEG	POS	POS	POS	POS	NEG	NEG	NEG	NEG	NEG	POS	POS	POS	NEG
05V16691		NEG	NEG	NEG	NEG	NEG	NEG	NEG	NEG	NEG	NEG																	
05V18550	NEG	AMB	NEG	NEG	NEG	NEG	NEG	NEG	NEG	NEG	NEG	NEG	NEG	NEG	NEG	POS	POS	POS	POS	NEG	NEG	NEG	NEG	NEG	POS	POS	POS	NEG
05V19034	NEG	NEG	NEG	NEG	NEG	NEG	NEG	NEG	NEG	NEG	NEG	NEG	NEG	NEG	NEG	POS	POS	POS	POS	NEG	NEG	NEG	NEG	NEG	POS	POS	POS	NEG
05V19333		NEG	NEG	NEG	NEG	NEG	NEG	NEG	NEG	NEG	NEG																	
05V19701	NEG	NEG	NEG	NEG	NEG	NEG	NEG	NEG	NEG	NEG	NEG	NEG	NEG	NEG	NEG	POS	POS	POS	POS	NEG	NEG	NEG	NEG	NEG	POS	POS	POS	NEG
05V20072	NEG	NEG	NEG	NEG	NEG	NEG	NEG	NEG	NEG	NEG	NEG	NEG	NEG	NEG	NEG	POS	POS	POS	POS	NEG	NEG	NEG	NEG	NEG	POS	POS	POS	NEG
05V24930	NEG	NEG	NEG	NEG	NEG	NEG	NEG	NEG	NEG	NEG	NEG	NEG	NEG	NEG	NEG	POS	POS	POS	POS	NEG	NEG	NEG	NEG	NEG	POS	POS	POS	NEG
05V31461	NEG	NEG	NEG	NEG	NEG	NEG	NEG	NEG	NEG	NEG	NEG	NEG	NEG	NEG	NEG	POS	POS	POS	POS	NEG	NEG	NEG	NEG	NEG	POS	POS	POS	NEG
05B05671	NEG	POS	NEG	NEG	NEG	NEG	NEG	POS	NEG	POS	NEG	NEG	NEG	NEG	NEG	NEG	NEG	NEG	NEG	POS	POS	POS	POS	POS	POS	POS	POS	NEG
05V01452	NEG	POS	NEG	NEG	NEG	NEG	NEG	POS	NEG	AMB	NEG	NEG	NEG	NEG	NEG	NEG	NEG	NEG	NEG	POS	POS	POS	POS	POS	POS	POS	POS	NEG
05V01617	NEG	POS	NEG	NEG	NEG	NEG	NEG	POS	NEG	AMB	NEG	NEG	NEG	NEG	NEG	NEG	NEG	NEG	NEG	POS	POS	POS	POS	POS	POS	POS	POS	NEG
05V16986	NEG	POS	NEG	NEG	NEG	NEG	NEG	POS	NEG	NEG	NEG	NEG	NEG	NEG	NEG	NEG	NEG	NEG	NEG	POS	POS	POS	POS	POS	POS	POS	POS	NEG
05V18357	NEG	POS	NEG	NEG	NEG	NEG	NEG	POS	NEG	NEG	POS	NEG	NEG	NEG	NEG	NEG	NEG	NEG	NEG	POS	POS	POS	POS	POS	POS	POS	POS	NEG
05V23111	NEG	POS	NEG	NEG	NEG	NEG	NEG	POS	NEG	AMB	NEG	NEG	NEG	NEG	NEG	NEG	NEG	NEG	NEG	POS	POS	POS	POS	POS	POS	POS	POS	NEG
05V23414		POS	NEG	NEG	NEG	NEG	NEG	POS	NEG	RANDOM	RANDOM																	
05V23858	NEG	POS	NEG	NEG	NEG	NEG	NEG	POS	NEG	AMB	NEG	NEG	NEG	NEG	NEG	NEG	NEG	NEG	NEG	POS	POS	POS	POS	POS	POS	POS	POS	NEG
05V26241	NEG	POS	NEG	NEG	NEG	NEG	NEG	POS	NEG	AMB	NEG	NEG	NEG	NEG	NEG	NEG	NEG	NEG	NEG	POS	POS	POS	POS	POS	POS	POS	POS	NEG
05V28399	NEG	POS	NEG	NEG	NEG	NEG	NEG	POS	NEG	NEG	NEG	NEG	NEG	NEG	NEG	NEG	NEG	NEG	NEG	POS	POS	POS	POS	POS	POS	POS	POS	NEG
05V32647		POS	NEG	NEG	NEG	NEG	NEG	POS	NEG	NEG	NEG																	
05V37969	NEG	POS	NEG	NEG	NEG	NEG	NEG	POS	NEG	NEG	NEG	NEG	NEG	NEG	NEG	NEG	NEG	NEG	NEG	POS	POS	POS	POS	POS	POS	POS	POS	NEG
05U10018	NEG	POS	NEG	NEG	NEG	POS	NEG	POS	NEG	POS	AMB	NEG	NEG	NEG	NEG	POS	POS	POS	POS	NEG	NEG	NEG	NEG	NEG	POS	POS	POS	NEG
**2006**																												
06V36353	NEG	AMB	NEG	NEG	POS	NEG	POS	NEG	POS	NEG	POS	NEG	NEG	NEG	NEG	NEG	NEG	NEG	NEG	POS	POS	POS	POS	POS	POS	POS	POS	NEG
06V40202		NEG	NEG	POS	NEG	NEG	POS	NEG	POS	NEG	POS																	
06V41266	NEG	NEG	NEG	POS	NEG	NEG	POS	NEG	POS	NEG	POS	NEG	NEG	NEG	NEG	POS	POS	POS	POS	NEG	NEG	NEG	NEG	NEG	POS	POS	POS	NEG
06B09097		AMB	NEG	POS	NEG	NEG	POS	NEG	POS	NEG	POS																	
06F00244	NEG	AMB	NEG	POS	NEG	NEG	POS	NEG	POS	NEG	POS	NEG	NEG	NEG	NEG	POS	POS	POS	POS	NEG	NEG	NEG	NEG	NEG	POS	POS	POS	NEG
06V03913	NEG	AMB	NEG	POS	NEG	NEG	POS	NEG	POS	NEG	POS	NEG	NEG	NEG	NEG	POS	POS	POS	POS	NEG	NEG	NEG	NEG	NEG	POS	POS	POS	NEG
06V04337	AMB	AMB	NEG	POS	NEG	NEG	POS	NEG	POS	NEG	POS	NEG	NEG	NEG	NEG	POS	POS	POS	POS	NEG	NEG	NEG	NEG	NEG	POS	POS	POS	NEG
06V06785	AMB	AMB	NEG	POS	NEG	NEG	POS	NEG	POS	NEG	POS	NEG	NEG	NEG	NEG	POS	POS	POS	POS	NEG	NEG	NEG	NEG	NEG	POS	POS	POS	NEG
06V12258	AMB	AMB	NEG	POS	NEG	NEG	POS	NEG	POS	NEG	POS	NEG	NEG	NEG	NEG	POS	POS	POS	POS	NEG	NEG	NEG	NEG	NEG	POS	POS	POS	NEG
06V20553	AMB	AMB	NEG	POS	NEG	NEG	POS	NEG	POS	NEG	POS	NEG	NEG	NEG	NEG	POS	POS	POS	POS	NEG	NEG	NEG	NEG	NEG	POS	POS	POS	NEG
06V22564		NEG	NEG	POS	NEG	NEG	POS	NEG	POS	NEG	POS																	
06V23750	NEG	AMB	NEG	POS	NEG	NEG	POS	NEG	POS	NEG	POS	NEG	NEG	NEG	NEG	POS	POS	POS	POS	NEG	NEG	NEG	NEG	NEG	POS	POS	POS	NEG
06V27332	NEG	NEG	NEG	POS	NEG	NEG	POS	NEG	POS	NEG	POS	NEG	NEG	NEG	NEG	POS	POS	POS	POS	NEG	NEG	NEG	NEG	NEG	POS	POS	POS	NEG
06V29484	NEG	NEG	NEG	POS	NEG	NEG	POS	NEG	POS	NEG	POS	NEG	NEG	NEG	NEG	POS	POS	POS	POS	NEG	NEG	NEG	NEG	NEG	POS	POS	POS	NEG
06V31278		AMB	NEG	POS	NEG	NEG	POS	NEG	POS	NEG	POS																	
06V36043	NEG	NEG	NEG	POS	NEG	NEG	POS	NEG	POS	NEG	POS	NEG	NEG	NEG	NEG	POS	POS	POS	POS	NEG	NEG	NEG	NEG	NEG	POS	POS	POS	NEG
06V36349	NEG	AMB	NEG	POS	NEG	NEG	POS	NEG	POS	NEG	POS	NEG	NEG	NEG	NEG	POS	POS	POS	POS	NEG	NEG	NEG	NEG	NEG	POS	POS	POS	NEG
06V39695	NEG	NEG	NEG	POS	NEG	NEG	POS	NEG	POS	NEG	POS	NEG	NEG	NEG	NEG	POS	POS	POS	POS	NEG	NEG	NEG	NEG	NEG	POS	POS	POS	NEG
06V40571		NEG	NEG	POS	NEG	NEG	POS	NEG	POS	NEG	POS																	
06V42188	NEG	AMB	NEG	POS	NEG	NEG	POS	NEG	POS	NEG	POS	NEG	NEG	NEG	NEG	POS	POS	POS	POS	NEG	NEG	NEG	NEG	NEG	POS	POS	POS	NEG
06V42146		POS	POS	NEG	NEG	NEG	POS	NEG	POS	NEG	POS																	
06V28560	NEG	NEG	POS	NEG	NEG	NEG	POS	NEG	POS	NEG	POS	NEG	NEG	NEG	NEG	POS	POS	POS	POS	NEG	NEG	NEG	NEG	NEG	POS	POS	POS	NEG
06F00837	NEG	AMB	NEG	NEG	NEG	NEG	NEG	NEG	NEG	NEG	NEG	NEG	NEG	NEG	NEG	POS	POS	POS	POS	NEG	NEG	NEG	NEG	NEG	POS	POS	POS	NEG
06F01638	NEG	NEG	NEG	NEG	NEG	NEG	NEG	NEG	NEG	NEG	NEG	NEG	NEG	NEG	NEG	POS	POS	POS	POS	NEG	NEG	NEG	NEG	NEG	POS	POS	POS	NEG
06F04643		NEG	NEG	NEG	NEG	NEG	NEG	NEG	NEG	NEG	NEG																	
06F05580	NEG	NEG	NEG	NEG	NEG	NEG	NEG	NEG	NEG	NEG	NEG	NEG	NEG	NEG	NEG	POS	POS	POS	POS	NEG	NEG	NEG	NEG	NEG	POS	POS	POS	NEG
06U05469	NEG	NEG	NEG	NEG	NEG	NEG	NEG	NEG	NEG	NEG	NEG	NEG	NEG	NEG	NEG	POS	POS	POS	POS	NEG	NEG	NEG	NEG	NEG	POS	POS	POS	NEG
06U17314	NEG	NEG	NEG	NEG	NEG	NEG	NEG	NEG	NEG	NEG	NEG	NEG	NEG	NEG	NEG	POS	POS	POS	POS	NEG	NEG	NEG	NEG	NEG	POS	POS	POS	NEG
06V08377	NEG	AMB	NEG	NEG	NEG	NEG	NEG	NEG	NEG	NEG	NEG	NEG	NEG	NEG	NEG	POS	POS	POS	POS	NEG	NEG	NEG	NEG	NEG	POS	POS	POS	NEG
06V08474	NEG	NEG	NEG	NEG	NEG	NEG	NEG	NEG	NEG	NEG	NEG	NEG	NEG	NEG	NEG	POS	POS	POS	POS	NEG	NEG	NEG	NEG	NEG	POS	POS	POS	NEG
06V09374	NEG	AMB	NEG	NEG	NEG	NEG	NEG	NEG	NEG	NEG	AMB	NEG	NEG	NEG	NEG	POS	POS	POS	POS	NEG	NEG	NEG	NEG	NEG	POS	POS	POS	NEG
06V09605	NEG	NEG	NEG	NEG	NEG	NEG	NEG	NEG	NEG	NEG	NEG	NEG	NEG	NEG	NEG	POS	POS	POS	POS	NEG	NEG	NEG	NEG	NEG	POS	POS	POS	NEG
06V18687	NEG	NEG	NEG	NEG	NEG	NEG	NEG	NEG	NEG	NEG	NEG	NEG	NEG	NEG	NEG	POS	POS	POS	POS	NEG	NEG	NEG	NEG	NEG	POS	POS	POS	NEG
06V21697	NEG	AMB	NEG	NEG	NEG	NEG	NEG	NEG	NEG	NEG	NEG	NEG	NEG	AMB	NEG	POS	POS	POS	POS	NEG	NEG	NEG	NEG	NEG	POS	POS	POS	NEG
06V23749	NEG	NEG	NEG	NEG	NEG	NEG	NEG	NEG	NEG	NEG	NEG	NEG	NEG	NEG	NEG	POS	POS	POS	POS	NEG	NEG	NEG	NEG	NEG	POS	POS	POS	NEG
06V29915	NEG	AMB	NEG	NEG	NEG	NEG	NEG	NEG	NEG	NEG	NEG	NEG	NEG	NEG	NEG	POS	POS	POS	POS	NEG	NEG	NEG	NEG	NEG	POS	POS	POS	NEG
06V31778	NEG	NEG	NEG	NEG	NEG	NEG	NEG	NEG	NEG	NEG	NEG	NEG	NEG	NEG	NEG	POS	POS	POS	POS	NEG	NEG	NEG	NEG	NEG	POS	POS	POS	NEG
06V39089	NEG	NEG	NEG	NEG	NEG	NEG	NEG	NEG	NEG	NEG	NEG	NEG	NEG	NEG	NEG	POS	POS	POS	POS	NEG	NEG	NEG	NEG	NEG	POS	POS	POS	NEG
06V41512		NEG	NEG	NEG	NEG	NEG	NEG	NEG	NEG	NEG	NEG																	
06V42186	NEG	NEG	NEG	NEG	NEG	NEG	NEG	NEG	NEG	NEG	NEG	NEG	NEG	NEG	NEG	POS	POS	POS	POS	NEG	NEG	NEG	NEG	NEG	POS	POS	POS	NEG
06V42236	NEG	NEG	NEG	NEG	NEG	NEG	NEG	NEG	NEG	NEG	NEG	NEG	NEG	NEG	NEG	POS	POS	POS	POS	NEG	NEG	NEG	NEG	NEG	POS	POS	POS	NEG
06V43697	NEG	AMB	NEG	NEG	NEG	NEG	NEG	NEG	NEG	NEG	NEG	NEG	NEG	NEG	NEG	POS	POS	POS	POS	NEG	NEG	NEG	NEG	NEG	POS	POS	POS	NEG
06V44153	NEG	NEG	NEG	NEG	NEG	NEG	NEG	NEG	NEG	NEG	NEG	NEG	NEG	NEG	NEG	POS	POS	POS	POS	NEG	NEG	NEG	NEG	NEG	POS	POS	POS	NEG
06V45679	NEG	NEG	NEG	NEG	NEG	NEG	NEG	NEG	NEG	NEG	NEG	NEG	NEG	NEG	NEG	POS	POS	POS	POS	NEG	NEG	NEG	NEG	NEG	POS	POS	POS	NEG
06V45831	NEG	NEG	NEG	NEG	NEG	NEG	NEG	NEG	NEG	NEG	NEG	NEG	NEG	NEG	NEG	POS	POS	POS	POS	NEG	NEG	NEG	NEG	NEG	POS	POS	POS	NEG
06V46314	NEG	NEG	NEG	NEG	NEG	NEG	NEG	NEG	NEG	NEG	NEG	NEG	NEG	NEG	NEG	POS	POS	POS	POS	NEG	NEG	NEG	NEG	NEG	POS	POS	POS	NEG
06V34729	NEG	POS	NEG	NEG	NEG	POS	NEG	POS	NEG	POS	POS	NEG	AMB	NEG	NEG	NEG	NEG	NEG	NEG	POS	POS	POS	POS	POS	POS	POS	POS	NEG
06B09900	NEG	POS	NEG	NEG	NEG	NEG	NEG	POS	NEG	AMB	NEG	NEG	NEG	NEG	NEG	NEG	NEG	NEG	NEG	POS	POS	POS	POS	POS	POS	POS	POS	NEG
06V00922	NEG	POS	NEG	NEG	NEG	NEG	NEG	POS	NEG	NEG	NEG	NEG	NEG	NEG	NEG	NEG	NEG	NEG	NEG	POS	POS	POS	POS	POS	POS	POS	POS	NEG
06V09551	NEG	POS	NEG	NEG	NEG	NEG	NEG	POS	NEG	NEG	NEG	NEG	NEG	NEG	NEG	NEG	NEG	NEG	NEG	POS	POS	POS	POS	POS	POS	POS	POS	NEG
06V10719	NEG	POS	NEG	NEG	NEG	NEG	NEG	POS	NEG	NEG	NEG	NEG	NEG	NEG	NEG	NEG	NEG	NEG	NEG	POS	POS	POS	POS	POS	POS	POS	POS	NEG
06V11090	NEG	POS	NEG	NEG	NEG	NEG	NEG	POS	NEG	NEG	NEG	NEG	NEG	NEG	NEG	NEG	NEG	NEG	NEG	POS	POS	POS	POS	POS	POS	POS	POS	NEG
06V14383	NEG	POS	NEG	NEG	NEG	NEG	NEG	POS	NEG	NEG	NEG	NEG	NEG	NEG	NEG	NEG	NEG	NEG	NEG	POS	POS	POS	POS	POS	POS	POS	POS	NEG
06V21475	NEG	POS	NEG	NEG	NEG	NEG	NEG	POS	NEG	NEG	NEG	NEG	NEG	NEG	NEG	NEG	NEG	NEG	NEG	POS	POS	POS	POS	POS	POS	POS	POS	NEG
06V24231	NEG	POS	NEG	NEG	NEG	NEG	NEG	POS	NEG	NEG	NEG	NEG	NEG	NEG	NEG	NEG	NEG	NEG	NEG	POS	POS	POS	POS	POS	POS	POS	POS	NEG
06V32992	NEG	POS	NEG	NEG	NEG	NEG	NEG	POS	NEG	NEG	NEG	NEG	NEG	NEG	NEG	NEG	NEG	NEG	NEG	POS	AMB	POS	POS	POS	POS	POS	POS	NEG
06V38753	NEG	POS	NEG	NEG	NEG	NEG	NEG	POS	NEG	NEG	NEG	NEG	NEG	NEG	NEG	NEG	NEG	NEG	NEG	POS	POS	POS	POS	POS	POS	POS	POS	NEG
06V39848		POS	NEG	NEG	NEG	NEG	NEG	POS	NEG	NEG	NEG																	
06V42259	NEG	AMB	NEG	NEG	NEG	NEG	NEG	POS	NEG	AMB	NEG	NEG	NEG	NEG	NEG	NEG	NEG	NEG	NEG	POS	POS	POS	POS	POS	POS	POS	POS	NEG
06V33960		AMB	NEG	NEG	NEG	NEG	POS	AMB	POS	NEG	POS																	

ISOLATE:	STRAIN	ADHAESION FACTORS / GENES ENCODING MICROBIAL SURFACE COMPONENTS RECOGNIZING ADHESIVE MATRIX MOLECULES (MSCRAMM GENES)																																															
		bone sialoprotein-binding protein							clumping factor A					clumping factor B					collagen-binding adhesin	cell wall associated fibronectin-binding protein	cell surface elastin binding protein					enolase	fibrinogen binding protein (19 kDa)		fibronectin-binding protein A						fibronectin-binding protein B							Major histocompatibility complex class II analog protein (=Extracellular adherence protein, eap)				Staphylococcus aureus surface protein G			
		bbp (total)	bbp (consensus)	bbp (COL+MW2)	bbp (MRSA252)	bbp (Mu50)	bbp (RF122)	bbp (ST45)	clfA (total)	clfA (consensus)	clfA (COL+RF122)	clfA (MRSA252)	clfA (Mu50+MW2)	clfB (total)	clfB (consensus)	clfB (COL+Mu50)	clfB (MW2)	clfB (RF122)	cna	ebh (consensus)	ebpS (total)	ebpS_probe 612	ebpS_probe 614	ebpS (01-1111)	ebpS (COL)	eno	fib	fib (MRSA252)	fnbA (total)	fnbA (consensus)	fnbA (COL)	fnbA (MRSA252)	fnbA (Mu50+MW2)	fnbA (RF122)	fnbB (total)	fnbB (COL)	fnbB (COL+Mu50+MW2)	fnbB (Mu50)	fnbB (MW2)	fnbB (ST15)	fnbB (ST45-2)	map (total)	map (COL)	map (MRSA252)	map (Mu50+MW2)	sasG (total)	sasG (COL+Mu50)	sasG (MW2)	sasG (other than MRSA252+RF122)
**2005**																																																	
05B01179	CC5/ST228-MRSA-I, South German EMRSA	POS	POS	NEG	NEG	POS	NEG	NEG	NEG	NEG	NEG	NEG	NEG	POS	POS	POS	NEG	NEG	NEG	POS	POS	POS	POS	NEG	NEG	POS	POS	NEG	POS	POS	NEG	NEG	POS	NEG	NEG	NEG	NEG	NEG	NEG	NEG	NEG	POS	NEG	NEG	POS	POS	POS	NEG	POS
05B02687	CC5/ST228-MRSA-I, South German EMRSA	POS	POS	NEG	NEG	POS	NEG	NEG	NEG	NEG	NEG	NEG	NEG	POS	POS	POS	NEG	NEG	NEG	POS	POS	POS	POS	NEG	NEG	POS	POS	NEG	POS	POS	NEG	NEG	POS	NEG	NEG	NEG	NEG	NEG	NEG	NEG	NEG	POS	NEG	NEG	POS	POS	POS	NEG	POS
05V08483	CC5/ST228-MRSA-I, South German EMRSA	POS	POS	NEG	NEG	POS	NEG	NEG	POS	POS	NEG	NEG	POS	POS	POS	POS	NEG	NEG	NEG	POS	POS	POS	POS	NEG	NEG	POS	POS	NEG	POS	POS	NEG	NEG	POS	NEG	NEG	NEG	NEG	NEG	NEG	NEG	NEG	POS	NEG	NEG	POS	POS	POS	NEG	POS
05V19001	CC5/ST228-MRSA-I, South German EMRSA	POS	POS	NEG	NEG	POS	NEG	NEG	POS	POS	NEG	NEG	POS	POS	POS	POS	NEG	NEG	NEG	POS	POS	POS	POS	NEG	NEG	POS	POS	NEG	POS	POS	NEG	NEG	POS	NEG	NEG	NEG	NEG	NEG	NEG	NEG	NEG	POS	NEG	NEG	POS	POS	POS	NEG	POS
05B04626	CC5-MRSA-II, Rhine-Hesse EMRSA/UK-EMRSA-3	POS	POS	NEG	NEG	POS	NEG	NEG	POS	POS	NEG	NEG	POS	POS	POS	POS	NEG	NEG	NEG	POS	POS	POS	POS	NEG	NEG	POS	POS	NEG	POS	POS	NEG	NEG	POS	NEG	POS	NEG	POS	POS	NEG	NEG	NEG	POS	NEG	NEG	POS	POS	POS	NEG	POS
05B07903	CC5-MRSA-II, Rhine-Hesse EMRSA/UK-EMRSA-3	POS	POS	NEG	NEG	POS	NEG	NEG	POS	POS	NEG	NEG	POS	POS	POS	POS	NEG	NEG	NEG	POS	POS	POS	POS	NEG	NEG	POS	POS	NEG	POS	POS	NEG	NEG	POS	AMB	POS	NEG	POS	POS	NEG	NEG	NEG	POS	NEG	NEG	POS	POS	POS	NEG	POS
05U00062	CC5-MRSA-II, Rhine-Hesse EMRSA/UK-EMRSA-3	POS	POS	NEG	NEG	POS	NEG	NEG	POS	POS	NEG	NEG	POS	POS	POS	POS	NEG	NEG	NEG	POS	POS	POS	POS	NEG	NEG	POS	POS	NEG	POS	POS	NEG	NEG	POS	NEG	POS	NEG	POS	AMB	NEG	NEG	NEG	POS	NEG	NEG	POS	POS	POS	NEG	POS
05U03117	CC5-MRSA-II, Rhine-Hesse EMRSA/UK-EMRSA-3	POS	POS	NEG	NEG	POS	NEG	NEG	POS	POS	NEG	NEG	POS	POS	POS	POS	NEG	NEG	NEG	POS	POS	POS	POS	NEG	NEG	POS	POS	NEG	POS	POS	NEG	NEG	POS	NEG	POS	NEG	POS	AMB	NEG	NEG	NEG	POS	NEG	NEG	POS	POS	POS	NEG	POS
05U10392	CC5-MRSA-II, Rhine-Hesse EMRSA/UK-EMRSA-3	POS	POS	NEG	NEG	POS	NEG	NEG	POS	POS	NEG	NEG	POS	POS	POS	POS	NEG	NEG	NEG	POS	POS	POS	POS	NEG	NEG	POS	POS	NEG	POS	POS	NEG	NEG	POS	NEG	POS	NEG	POS	AMB	NEG	NEG	NEG	POS	AMB	NEG	POS	POS	POS	NEG	POS
05V03312	CC5-MRSA-II, Rhine-Hesse EMRSA/UK-EMRSA-3	POS	POS	NEG	NEG	POS	NEG	NEG	POS	POS	NEG	NEG	POS	POS	POS	POS	NEG	NEG	NEG	POS	POS	POS	POS	NEG	AMB	POS	POS	NEG	POS	POS	NEG	NEG	POS	NEG	POS	NEG	AMB	POS	NEG	NEG	NEG	POS	AMB	NEG	POS	POS	POS	NEG	POS
05V08095	CC5-MRSA-II, Rhine-Hesse EMRSA/UK-EMRSA-3	POS	POS	NEG	NEG	POS	NEG	NEG	POS	POS	NEG	NEG	POS	POS	POS	POS	NEG	NEG	NEG	POS	POS	POS	POS	NEG	NEG	POS	POS	NEG	POS	POS	NEG	NEG	POS	NEG	POS	NEG	POS	AMB	NEG	NEG	NEG	POS	AMB	NEG	POS	POS	POS	NEG	POS
05V09015	CC5-MRSA-II, Rhine-Hesse EMRSA/UK-EMRSA-3	POS	POS	NEG	NEG	POS	NEG	NEG	POS	POS	NEG	NEG	POS	POS	POS	POS	NEG	NEG	NEG	POS	POS	POS	POS	NEG	NEG	POS	POS	NEG	POS	POS	NEG	NEG	POS	NEG	POS	NEG	POS	AMB	NEG	NEG	NEG	POS	AMB	NEG	POS	POS	POS	NEG	POS
05V09326	CC5-MRSA-II, Rhine-Hesse EMRSA/UK-EMRSA-3	POS	POS	NEG	NEG	POS	NEG	NEG	POS	POS	NEG	NEG	POS	POS	POS	POS	NEG	NEG	NEG	POS	POS	POS	POS	NEG	NEG	POS	POS	NEG	POS	POS	NEG	NEG	POS	NEG	POS	NEG	AMB	POS	NEG	AMB	NEG	POS	NEG	NEG	POS	POS	POS	NEG	POS
05V10265	CC5-MRSA-II, Rhine-Hesse EMRSA/UK-EMRSA-3	POS	POS	NEG	NEG	POS	NEG	NEG	POS	POS	NEG	NEG	POS	POS	POS	POS	NEG	NEG	NEG	POS	POS	POS	POS	NEG	NEG	POS	POS	NEG	POS	POS	NEG	NEG	POS	NEG	POS	NEG	POS	AMB	NEG	NEG	NEG	POS	NEG	NEG	POS	POS	POS	NEG	POS
05V13052	CC5-MRSA-II, Rhine-Hesse EMRSA/UK-EMRSA-3	POS	POS	NEG	NEG	POS	NEG	NEG	POS	POS	NEG	NEG	POS	POS	POS	POS	NEG	NEG	NEG	POS	POS	POS	POS	NEG	NEG	POS	POS	NEG	POS	POS	NEG	NEG	POS	NEG	POS	NEG	AMB	POS	NEG	NEG	NEG	POS	NEG	NEG	POS	POS	POS	NEG	POS
05V13470	CC5-MRSA-II, Rhine-Hesse EMRSA/UK-EMRSA-3	POS	POS	NEG	NEG	POS	NEG	NEG	POS	POS	NEG	NEG	POS	POS	POS	POS	NEG	NEG	NEG	POS	POS	POS	POS	NEG	NEG	POS	POS	NEG	POS	POS	NEG	NEG	POS	NEG	POS	NEG	POS	AMB	NEG	NEG	NEG	POS	AMB	NEG	POS	POS	POS	NEG	POS
05V13891	CC5-MRSA-II, Rhine-Hesse EMRSA/UK-EMRSA-3	POS	POS	NEG	NEG	POS	NEG	NEG	POS	POS	NEG	NEG	POS	POS	POS	POS	NEG	NEG	NEG	POS	POS	AMB	POS	NEG	NEG	POS	POS	NEG	POS	POS	NEG	NEG	POS	NEG	POS	NEG	POS	AMB	NEG	NEG	NEG	POS	NEG	NEG	POS	POS	POS	NEG	POS
05V15616	CC5-MRSA-II, Rhine-Hesse EMRSA/UK-EMRSA-3																																																
05V16845	CC5-MRSA-II, Rhine-Hesse EMRSA/UK-EMRSA-3	POS	POS	NEG	NEG	POS	NEG	NEG	POS	POS	NEG	NEG	POS	POS	POS	POS	NEG	NEG	NEG	POS	POS	POS	POS	NEG	NEG	POS	POS	NEG	POS	POS	NEG	NEG	POS	NEG	POS	NEG	POS	AMB	NEG	NEG	NEG	POS	NEG	NEG	POS	POS	POS	NEG	POS
05V17541	CC5-MRSA-II, Rhine-Hesse EMRSA/UK-EMRSA-3	POS	POS	NEG	NEG	POS	NEG	NEG	POS	POS	NEG	NEG	POS	POS	POS	POS	NEG	NEG	NEG	POS	POS	POS	POS	NEG	NEG	POS	POS	NEG	POS	POS	NEG	NEG	POS	NEG	POS	NEG	POS	AMB	NEG	NEG	NEG	POS	NEG	NEG	POS	POS	POS	NEG	POS
05V19105	CC5-MRSA-II, Rhine-Hesse EMRSA/UK-EMRSA-3																																																
05V22148	CC5-MRSA-II, Rhine-Hesse EMRSA/UK-EMRSA-3	POS	POS	NEG	NEG	POS	NEG	NEG	POS	POS	NEG	NEG	POS	POS	POS	POS	NEG	NEG	NEG	POS	POS	POS	POS	NEG	NEG	POS	POS	NEG	POS	POS	NEG	NEG	POS	NEG	POS	NEG	AMB	POS	NEG	NEG	NEG	POS	NEG	NEG	POS	POS	POS	NEG	POS
05V33740	CC5-MRSA-II, Rhine-Hesse EMRSA/UK-EMRSA-3	POS	POS	NEG	NEG	POS	NEG	NEG	POS	POS	NEG	NEG	POS	POS	POS	POS	NEG	NEG	NEG	POS	POS	POS	POS	NEG	NEG	POS	POS	NEG	POS	POS	NEG	NEG	POS	NEG	POS	NEG	POS	AMB	NEG	NEG	NEG	POS	NEG	NEG	POS	POS	POS	NEG	POS
05V38425	CC5-MRSA-II, Rhine-Hesse EMRSA/UK-EMRSA-3	POS	POS	NEG	NEG	POS	NEG	NEG	POS	POS	NEG	NEG	POS	POS	POS	POS	NEG	NEG	NEG	POS	POS	AMB	POS	NEG	NEG	POS	POS	NEG	POS	POS	NEG	NEG	POS	NEG	POS	NEG	POS	AMB	NEG	NEG	NEG	POS	NEG	NEG	POS	POS	POS	NEG	POS
05V39987	CC5-MRSA-II, Rhine-Hesse EMRSA/UK-EMRSA-3	POS	POS	NEG	NEG	POS	NEG	NEG	POS	POS	NEG	NEG	POS	POS	POS	POS	NEG	NEG	NEG	POS	POS	POS	POS	NEG	NEG	POS	POS	NEG	POS	POS	NEG	NEG	POS	NEG	POS	NEG	POS	AMB	NEG	NEG	NEG	POS	NEG	NEG	POS	POS	POS	NEG	POS
05F00089	CC5-MRSA-IV, Paediatric clone	POS	POS	NEG	NEG	POS	NEG	NEG	POS	POS	NEG	NEG	POS	POS	POS	POS	NEG	NEG	NEG	POS	POS	POS	POS	NEG	NEG	POS	POS	NEG	POS	POS	NEG	NEG	POS	NEG	POS	NEG	POS	POS	NEG	NEG	NEG	POS	NEG	NEG	POS	POS	POS	NEG	POS
05V01416	CC5-MRSA-IV, Paediatric clone	POS	AMB	NEG	NEG	POS	NEG	NEG	POS	POS	NEG	NEG	POS	POS	POS	POS	NEG	NEG	NEG	POS	POS	AMB	POS	NEG	NEG	POS	POS	NEG	POS	POS	NEG	NEG	POS	NEG	POS	NEG	POS	NEG	NEG	NEG	NEG	POS	NEG	NEG	POS	POS	POS	NEG	POS
05V16386	CC8-MRSA-IV, USA300	POS	POS	POS	NEG	NEG	NEG	NEG	POS	POS	POS	NEG	NEG	POS	POS	POS	NEG	NEG	NEG	POS	POS	POS	POS	NEG	POS	POS	POS	NEG	POS	POS	POS	NEG	NEG	NEG	POS	POS	POS	NEG	NEG	NEG	NEG	POS	POS	NEG	NEG	POS	POS	NEG	POS
05V04920	CC8-MRSA-IV, USA500	POS	POS	POS	NEG	NEG	NEG	NEG	POS	POS	POS	AMB	NEG	POS	POS	POS	NEG	NEG	NEG	POS	POS	POS	POS	NEG	POS	POS	POS	NEG	POS	POS	POS	NEG	NEG	NEG	POS	POS	POS	NEG	NEG	NEG	NEG	POS	POS	NEG	NEG	POS	POS	NEG	POS
05V21159	CC8-MRSA-IV, USA500	POS	POS	POS	NEG	NEG	NEG	NEG	POS	POS	POS	NEG	NEG	POS	POS	POS	NEG	NEG	NEG	POS	POS	POS	POS	NEG	POS	POS	POS	NEG	POS	POS	POS	NEG	NEG	NEG	POS	POS	POS	NEG	NEG	NEG	NEG	POS	POS	NEG	NEG	POS	POS	NEG	POS
05B00867	CC22-MRSA-IV, Barnim/UK-EMRSA-15	POS	POS	NEG	NEG	NEG	NEG	NEG	POS	POS	NEG	NEG	POS	POS	POS	AMB	NEG	AMB	POS	NEG	POS	POS	POS	NEG	NEG	POS	NEG	POS	POS	POS	NEG	NEG	POS	NEG	NEG	NEG	NEG	NEG	NEG	NEG	NEG	POS	NEG	NEG	POS	POS	NEG	POS	POS
05V03174	CC22-MRSA-IV, Barnim/UK-EMRSA-15	POS	POS	NEG	NEG	NEG	NEG	NEG	POS	POS	NEG	NEG	POS	POS	POS	NEG	NEG	POS	POS	NEG	POS	POS	POS	NEG	NEG	POS	NEG	POS	POS	POS	NEG	NEG	POS	NEG	NEG	NEG	NEG	NEG	NEG	NEG	NEG	POS	NEG	NEG	POS	POS	NEG	POS	POS
05V07331	CC22-MRSA-IV, Barnim/UK-EMRSA-15	POS	POS	NEG	NEG	NEG	NEG	NEG	POS	POS	NEG	NEG	POS	POS	POS	NEG	NEG	POS	POS	NEG	POS	POS	POS	NEG	NEG	POS	NEG	POS	POS	POS	NEG	NEG	POS	NEG	NEG	NEG	NEG	NEG	NEG	NEG	NEG	POS	NEG	NEG	POS	POS	NEG	POS	POS
05V07371	CC22-MRSA-IV, Barnim/UK-EMRSA-15	POS	POS	NEG	NEG	NEG	NEG	NEG	POS	POS	NEG	NEG	POS	POS	POS	NEG	NEG	NEG	POS	NEG	POS	POS	POS	NEG	NEG	POS	NEG	POS	POS	POS	NEG	NEG	POS	NEG	NEG	NEG	NEG	NEG	NEG	NEG	NEG	POS	NEG	NEG	POS	POS	NEG	POS	POS
05V08145	CC22-MRSA-IV, Barnim/UK-EMRSA-15	POS	POS	NEG	NEG	NEG	NEG	NEG	POS	POS	NEG	NEG	POS	POS	POS	NEG	NEG	POS	POS	NEG	POS	POS	POS	NEG	NEG	POS	NEG	POS	POS	POS	NEG	NEG	POS	NEG	NEG	NEG	NEG	NEG	NEG	NEG	NEG	NEG	NEG	NEG	AMB	POS	NEG	POS	POS
05V08595	CC22-MRSA-IV, Barnim/UK-EMRSA-15	POS	POS	NEG	NEG	NEG	NEG	NEG	POS	POS	NEG	NEG	POS	POS	POS	NEG	NEG	POS	POS	NEG	POS	POS	POS	NEG	NEG	POS	NEG	POS	POS	POS	NEG	NEG	POS	NEG	NEG	NEG	NEG	NEG	NEG	NEG	NEG	POS	NEG	NEG	POS	POS	NEG	POS	POS
05V12803	CC22-MRSA-IV, Barnim/UK-EMRSA-15	POS	POS	NEG	NEG	NEG	NEG	NEG	POS	POS	NEG	NEG	POS	POS	POS	NEG	NEG	POS	POS	NEG	POS	POS	POS	NEG	NEG	POS	NEG	POS	POS	POS	NEG	NEG	POS	NEG	NEG	NEG	NEG	NEG	NEG	NEG	NEG	POS	NEG	NEG	POS	POS	NEG	POS	POS
05V14596	CC22-MRSA-IV, Barnim/UK-EMRSA-15	POS	POS	NEG	NEG	NEG	NEG	NEG	POS	POS	NEG	NEG	POS	POS	POS	NEG	NEG	NEG	POS	NEG	POS	AMB	POS	NEG	NEG	POS	NEG	POS	POS	POS	NEG	NEG	POS	NEG	NEG	NEG	NEG	NEG	NEG	NEG	NEG	POS	NEG	NEG	POS	POS	NEG	POS	POS
05V16691	CC22-MRSA-IV, Barnim/UK-EMRSA-15																																																
05V18550	CC22-MRSA-IV, Barnim/UK-EMRSA-15	POS	POS	NEG	NEG	POS	NEG	NEG	POS	POS	NEG	NEG	POS	POS	POS	NEG	NEG	POS	POS	NEG	POS	POS	POS	NEG	NEG	POS	NEG	POS	POS	POS	NEG	NEG	POS	NEG	NEG	NEG	NEG	NEG	NEG	NEG	NEG	POS	NEG	NEG	POS	POS	NEG	POS	POS
05V19034	CC22-MRSA-IV, Barnim/UK-EMRSA-15	POS	POS	NEG	NEG	POS	NEG	NEG	POS	POS	NEG	NEG	POS	POS	POS	NEG	NEG	POS	POS	NEG	POS	POS	POS	NEG	NEG	POS	NEG	POS	POS	POS	NEG	NEG	POS	NEG	NEG	NEG	NEG	NEG	NEG	NEG	NEG	POS	NEG	NEG	POS	POS	NEG	POS	POS
05V19333	CC22-MRSA-IV, Barnim/UK-EMRSA-15																																																
05V19701	CC22-MRSA-IV, Barnim/UK-EMRSA-15	POS	POS	NEG	NEG	NEG	NEG	NEG	POS	POS	NEG	NEG	POS	POS	POS	NEG	NEG	NEG	POS	NEG	POS	POS	POS	NEG	NEG	POS	NEG	POS	POS	POS	NEG	NEG	POS	NEG	NEG	NEG	NEG	NEG	NEG	NEG	NEG	POS	NEG	NEG	POS	POS	NEG	POS	POS
05V20072	CC22-MRSA-IV, Barnim/UK-EMRSA-15	POS	POS	NEG	NEG	NEG	NEG	NEG	POS	POS	NEG	NEG	POS	POS	POS	NEG	NEG	POS	POS	NEG	POS	POS	POS	NEG	NEG	POS	NEG	POS	POS	POS	NEG	NEG	POS	NEG	NEG	NEG	NEG	NEG	NEG	NEG	NEG	POS	NEG	NEG	POS	POS	NEG	POS	POS
05V24930	CC22-MRSA-IV, Barnim/UK-EMRSA-15	POS	POS	NEG	NEG	NEG	NEG	NEG	POS	POS	NEG	NEG	POS	POS	POS	NEG	NEG	POS	POS	NEG	POS	POS	POS	NEG	NEG	POS	NEG	POS	POS	POS	NEG	NEG	POS	NEG	NEG	NEG	NEG	NEG	NEG	NEG	NEG	POS	NEG	NEG	POS	POS	NEG	POS	POS
05V31461	CC22-MRSA-IV, Barnim/UK-EMRSA-15	POS	POS	NEG	NEG	POS	NEG	NEG	POS	POS	NEG	NEG	POS	POS	POS	NEG	NEG	POS	POS	NEG	POS	POS	POS	NEG	NEG	POS	NEG	POS	POS	POS	NEG	NEG	POS	NEG	NEG	NEG	NEG	NEG	NEG	NEG	NEG	POS	NEG	NEG	POS	POS	NEG	POS	POS
05B05671	CC45-MRSA-IV, Berlin EMRSA	POS	POS	NEG	NEG	NEG	NEG	POS	POS	POS	POS	NEG	POS	POS	POS	NEG	POS	POS	POS	POS	POS	NEG	POS	POS	NEG	POS	NEG	POS	POS	POS	NEG	NEG	POS	NEG	POS	NEG	POS	POS	NEG	AMB	NEG	POS	NEG	POS	NEG	NEG	NEG	NEG	NEG
05V01452	CC45-MRSA-IV, Berlin EMRSA	POS	POS	NEG	NEG	NEG	NEG	POS	POS	POS	AMB	NEG	POS	POS	POS	NEG	POS	AMB	POS	POS	POS	NEG	POS	POS	AMB	POS	NEG	POS	POS	POS	NEG	NEG	POS	NEG	POS	NEG	AMB	POS	NEG	NEG	NEG	POS	NEG	POS	NEG	NEG	NEG	NEG	NEG
05V01617	CC45-MRSA-IV, Berlin EMRSA	POS	POS	AMB	NEG	NEG	NEG	POS	POS	POS	AMB	NEG	POS	POS	POS	NEG	POS	AMB	POS	POS	POS	NEG	POS	POS	NEG	POS	NEG	POS	POS	POS	NEG	NEG	POS	NEG	POS	NEG	POS	AMB	NEG	NEG	NEG	POS	NEG	POS	NEG	NEG	NEG	NEG	NEG
05V16986	CC45-MRSA-IV, Berlin EMRSA	POS	POS	NEG	NEG	NEG	NEG	POS	POS	POS	POS	NEG	POS	POS	POS	NEG	POS	AMB	POS	POS	POS	NEG	POS	NEG	NEG	POS	NEG	NEG	POS	POS	NEG	NEG	POS	NEG	POS	NEG	POS	AMB	NEG	NEG	NEG	POS	NEG	POS	NEG	NEG	NEG	NEG	NEG
05V18357	CC45-MRSA-IV, Berlin EMRSA	POS	POS	NEG	NEG	NEG	NEG	POS	POS	POS	AMB	NEG	POS	POS	POS	NEG	POS	AMB	POS	POS	POS	NEG	POS	POS	NEG	POS	NEG	POS	POS	POS	NEG	NEG	POS	NEG	POS	NEG	POS	AMB	NEG	NEG	NEG	POS	NEG	POS	NEG	NEG	NEG	NEG	NEG
05V23111	CC45-MRSA-IV, Berlin EMRSA	POS	POS	AMB	NEG	NEG	NEG	POS	POS	POS	AMB	NEG	POS	POS	POS	NEG	POS	AMB	POS	POS	POS	NEG	POS	POS	NEG	POS	NEG	POS	POS	POS	NEG	NEG	POS	NEG	POS	NEG	POS	AMB	NEG	AMB	NEG	POS	NEG	POS	NEG	NEG	NEG	NEG	NEG
05V23414	CC45-MRSA-IV, Berlin EMRSA																																																
05V23858	CC45-MRSA-IV, Berlin EMRSA	POS	POS	NEG	NEG	NEG	NEG	POS	POS	POS	AMB	NEG	POS	POS	POS	NEG	POS	AMB	POS	POS	POS	NEG	POS	POS	NEG	POS	NEG	POS	POS	POS	NEG	NEG	POS	NEG	POS	NEG	POS	AMB	NEG	NEG	NEG	POS	NEG	POS	NEG	NEG	NEG	NEG	NEG
05V26241	CC45-MRSA-IV, Berlin EMRSA	POS	POS	NEG	NEG	NEG	NEG	POS	POS	POS	AMB	NEG	POS	POS	POS	NEG	POS	AMB	POS	POS	POS	NEG	POS	POS	NEG	POS	NEG	POS	POS	POS	NEG	NEG	POS	NEG	POS	NEG	POS	AMB	NEG	NEG	NEG	POS	NEG	POS	NEG	NEG	NEG	NEG	NEG
05V28399	CC45-MRSA-IV, Berlin EMRSA	POS	POS	AMB	NEG	NEG	NEG	POS	POS	POS	AMB	NEG	POS	POS	POS	NEG	POS	AMB	POS	POS	POS	NEG	POS	POS	NEG	POS	NEG	POS	POS	POS	NEG	NEG	POS	NEG	POS	NEG	POS	AMB	NEG	NEG	NEG	POS	NEG	POS	NEG	NEG	NEG	NEG	NEG
05V32647	CC45-MRSA-IV, Berlin EMRSA																																																
05V37969	CC45-MRSA-IV, Berlin EMRSA	POS	POS	NEG	NEG	NEG	NEG	POS	POS	POS	AMB	NEG	POS	POS	POS	NEG	POS	AMB	POS	POS	POS	NEG	POS	POS	NEG	POS	NEG	POS	POS	POS	NEG	NEG	POS	NEG	POS	NEG	POS	AMB	NEG	NEG	NEG	POS	NEG	POS	NEG	NEG	NEG	NEG	NEG
05U10018	CC398-MRSA-V	POS	POS	NEG	NEG	NEG	NEG	POS	POS	POS	POS	NEG	POS	POS	POS	NEG	POS	POS	POS	POS	POS	NEG	POS	AMB	NEG	POS	NEG	POS	POS	NEG	NEG	NEG	POS	NEG	POS	NEG	AMB	POS	NEG	NEG	NEG	POS	NEG	POS	NEG	NEG	NEG	NEG	NEG
**2006**																																																	
06V36353	CC1-MRSA-IV, WA MRSA-1/57	POS	POS	POS	NEG	NEG	NEG	NEG	POS	POS	NEG	NEG	POS	POS	POS	NEG	POS	AMB	POS	POS	POS	AMB	POS	NEG	NEG	POS	POS	NEG	POS	POS	NEG	NEG	POS	NEG	POS	NEG	POS	NEG	POS	NEG	NEG	POS	NEG	NEG	POS	POS	NEG	POS	POS
06V40202	CC5/ST228-MRSA-I, South German EMRSA																																																
06V41266	CC5/ST228-MRSA-I, South German EMRSA	POS	POS	NEG	NEG	POS	NEG	NEG	NEG	NEG	NEG	NEG	NEG	POS	POS	POS	NEG	NEG	NEG	POS	POS	POS	POS	NEG	NEG	POS	POS	NEG	POS	POS	NEG	NEG	POS	NEG	NEG	NEG	NEG	NEG	NEG	NEG	NEG	POS	NEG	NEG	POS	POS	POS	NEG	POS
06B09097	CC5-MRSA-II, Rhine-Hesse EMRSA/UK-EMRSA-3																																																
06F00244	CC5-MRSA-II, Rhine-Hesse EMRSA/UK-EMRSA-3	POS	POS	NEG	NEG	POS	NEG	NEG	POS	POS	NEG	NEG	POS	POS	POS	POS	NEG	NEG	NEG	POS	POS	POS	POS	NEG	NEG	POS	POS	NEG	POS	POS	NEG	NEG	POS	NEG	POS	NEG	AMB	POS	NEG	NEG	NEG	POS	AMB	NEG	POS	POS	POS	NEG	POS
06V03913	CC5-MRSA-II, Rhine-Hesse EMRSA/UK-EMRSA-3	POS	POS	AMB	NEG	POS	NEG	NEG	POS	POS	NEG	NEG	POS	POS	POS	POS	NEG	NEG	NEG	POS	POS	POS	POS	NEG	NEG	POS	POS	NEG	POS	POS	NEG	NEG	POS	NEG	POS	NEG	AMB	POS	NEG	AMB	NEG	POS	NEG	NEG	POS	POS	POS	NEG	POS
06V04337	CC5-MRSA-II, Rhine-Hesse EMRSA/UK-EMRSA-3	POS	POS	AMB	NEG	POS	NEG	NEG	POS	POS	NEG	NEG	POS	POS	POS	POS	NEG	NEG	NEG	POS	POS	POS	POS	NEG	NEG	POS	POS	NEG	POS	POS	NEG	NEG	POS	NEG	POS	NEG	AMB	POS	NEG	AMB	NEG	POS	NEG	NEG	POS	POS	POS	NEG	POS
06V06785	CC5-MRSA-II, Rhine-Hesse EMRSA/UK-EMRSA-3	POS	POS	AMB	NEG	POS	NEG	NEG	POS	POS	NEG	NEG	POS	POS	POS	POS	NEG	NEG	NEG	POS	POS	POS	POS	NEG	NEG	POS	POS	NEG	POS	POS	NEG	NEG	POS	NEG	POS	NEG	POS	AMB	NEG	AMB	NEG	POS	NEG	NEG	POS	POS	POS	NEG	POS
06V12258	CC5-MRSA-II, Rhine-Hesse EMRSA/UK-EMRSA-3	POS	POS	AMB	NEG	POS	NEG	AMB	POS	POS	NEG	NEG	POS	POS	POS	POS	NEG	NEG	NEG	POS	POS	POS	POS	NEG	NEG	POS	POS	NEG	POS	POS	NEG	NEG	POS	NEG	POS	NEG	AMB	POS	NEG	AMB	NEG	POS	NEG	NEG	POS	POS	POS	NEG	POS
06V20553	CC5-MRSA-II, Rhine-Hesse EMRSA/UK-EMRSA-3	POS	POS	NEG	NEG	POS	NEG	NEG	POS	POS	NEG	NEG	POS	POS	POS	POS	NEG	NEG	NEG	POS	POS	POS	POS	NEG	NEG	POS	POS	NEG	POS	POS	NEG	NEG	POS	NEG	POS	NEG	AMB	POS	NEG	NEG	NEG	POS	NEG	NEG	POS	POS	POS	NEG	POS
06V22564	CC5-MRSA-II, Rhine-Hesse EMRSA/UK-EMRSA-3																																																
06V23750	CC5-MRSA-II, Rhine-Hesse EMRSA/UK-EMRSA-3	POS	POS	NEG	NEG	POS	NEG	NEG	POS	POS	NEG	NEG	POS	POS	POS	POS	NEG	NEG	NEG	POS	POS	POS	POS	NEG	NEG	POS	POS	NEG	POS	POS	NEG	NEG	POS	NEG	POS	NEG	AMB	POS	NEG	NEG	NEG	POS	NEG	NEG	POS	POS	POS	NEG	POS
06V27332	CC5-MRSA-II, Rhine-Hesse EMRSA/UK-EMRSA-3	POS	POS	NEG	NEG	POS	NEG	NEG	POS	POS	NEG	NEG	POS	POS	POS	POS	NEG	NEG	NEG	POS	POS	POS	POS	NEG	NEG	POS	POS	NEG	POS	POS	NEG	NEG	POS	NEG	POS	NEG	POS	AMB	NEG	NEG	NEG	POS	AMB	NEG	POS	POS	POS	NEG	POS
06V29484	CC5-MRSA-II, Rhine-Hesse EMRSA/UK-EMRSA-3	POS	POS	NEG	NEG	POS	NEG	NEG	POS	POS	NEG	NEG	POS	POS	POS	POS	NEG	NEG	NEG	POS	POS	POS	POS	NEG	NEG	POS	POS	NEG	POS	POS	NEG	NEG	POS	NEG	POS	NEG	POS	AMB	NEG	NEG	NEG	POS	NEG	NEG	POS	POS	POS	NEG	POS
06V31278	CC5-MRSA-II, Rhine-Hesse EMRSA/UK-EMRSA-3																																																
06V36043	CC5-MRSA-II, Rhine-Hesse EMRSA/UK-EMRSA-3	POS	POS	NEG	NEG	POS	NEG	NEG	POS	POS	NEG	NEG	POS	POS	POS	POS	NEG	NEG	NEG	POS	POS	POS	POS	NEG	NEG	POS	POS	NEG	POS	POS	NEG	NEG	POS	NEG	POS	NEG	POS	POS	NEG	NEG	NEG	POS	NEG	NEG	POS	POS	POS	NEG	POS
06V36349	CC5-MRSA-II, Rhine-Hesse EMRSA/UK-EMRSA-3	POS	POS	NEG	NEG	POS	NEG	NEG	POS	POS	NEG	NEG	POS	POS	POS	POS	NEG	NEG	NEG	POS	POS	POS	POS	NEG	NEG	POS	POS	NEG	POS	POS	NEG	NEG	POS	NEG	POS	NEG	POS	AMB	NEG	NEG	NEG	POS	AMB	NEG	POS	POS	POS	NEG	POS
06V39695	CC5-MRSA-II, Rhine-Hesse EMRSA/UK-EMRSA-3	POS	POS	NEG	NEG	POS	NEG	NEG	POS	POS	NEG	NEG	POS	POS	POS	POS	NEG	NEG	NEG	POS	POS	POS	POS	NEG	NEG	POS	POS	NEG	POS	POS	NEG	NEG	POS	NEG	POS	NEG	POS	AMB	NEG	NEG	NEG	POS	NEG	NEG	POS	POS	POS	NEG	POS
06V40571	CC5-MRSA-II, Rhine-Hesse EMRSA/UK-EMRSA-3																																																
06V42188	CC5-MRSA-II, Rhine-Hesse EMRSA/UK-EMRSA-3	POS	POS	NEG	NEG	POS	NEG	NEG	POS	POS	NEG	NEG	POS	POS	POS	POS	NEG	NEG	NEG	POS	POS	POS	POS	NEG	NEG	POS	POS	NEG	POS	POS	NEG	NEG	POS	NEG	POS	NEG	AMB	POS	NEG	NEG	NEG	POS	AMB	NEG	POS	POS	POS	NEG	POS
06V42146	CC8-MRSA-IV, Lyon Clone/UK-EMRSA-2																																																
06V28560	CC8-MRSA-V	POS	POS	POS	NEG	NEG	NEG	NEG	POS	POS	POS	AMB	AMB	POS	POS	POS	NEG	NEG	NEG	POS	POS	POS	POS	NEG	POS	POS	POS	NEG	POS	POS	POS	NEG	NEG	NEG	POS	POS	POS	NEG	NEG	NEG	NEG	POS	POS	NEG	NEG	POS	POS	NEG	POS
06F00837	CC22-MRSA-IV, Barnim/UK-EMRSA-15	POS	POS	NEG	NEG	NEG	NEG	NEG	POS	POS	NEG	NEG	POS	POS	POS	NEG	NEG	POS	POS	NEG	POS	POS	POS	NEG	NEG	POS	NEG	POS	POS	POS	NEG	NEG	POS	NEG	NEG	NEG	NEG	NEG	NEG	NEG	NEG	POS	NEG	NEG	POS	POS	NEG	POS	POS
06F01638	CC22-MRSA-IV, Barnim/UK-EMRSA-15	POS	POS	NEG	NEG	POS	NEG	NEG	POS	POS	NEG	NEG	POS	POS	POS	NEG	NEG	POS	POS	NEG	POS	POS	POS	NEG	NEG	POS	NEG	POS	POS	POS	NEG	NEG	POS	NEG	NEG	NEG	NEG	NEG	NEG	NEG	NEG	POS	NEG	NEG	POS	POS	NEG	POS	POS
06F04643	CC22-MRSA-IV, Barnim/UK-EMRSA-15																																																
06F05580	CC22-MRSA-IV, Barnim/UK-EMRSA-15	POS	POS	NEG	NEG	NEG	NEG	NEG	POS	POS	NEG	NEG	POS	POS	POS	NEG	NEG	NEG	POS	NEG	POS	POS	POS	NEG	NEG	POS	NEG	POS	POS	POS	NEG	NEG	POS	NEG	NEG	NEG	NEG	NEG	NEG	NEG	NEG	POS	NEG	NEG	POS	POS	NEG	POS	POS
06U05469	CC22-MRSA-IV, Barnim/UK-EMRSA-15	POS	POS	NEG	NEG	NEG	NEG	NEG	POS	POS	NEG	NEG	POS	POS	POS	NEG	NEG	POS	POS	NEG	POS	POS	POS	NEG	NEG	POS	NEG	POS	POS	POS	NEG	NEG	POS	NEG	NEG	NEG	NEG	NEG	NEG	NEG	NEG	POS	NEG	NEG	POS	POS	NEG	POS	POS
06U17314	CC22-MRSA-IV, Barnim/UK-EMRSA-15	POS	POS	NEG	NEG	NEG	NEG	NEG	POS	POS	NEG	NEG	POS	POS	POS	NEG	NEG	POS	POS	NEG	POS	POS	POS	NEG	NEG	POS	NEG	POS	POS	POS	NEG	NEG	POS	NEG	NEG	NEG	NEG	NEG	NEG	NEG	NEG	POS	NEG	NEG	POS	POS	NEG	POS	POS
06V08377	CC22-MRSA-IV, Barnim/UK-EMRSA-15	POS	POS	NEG	NEG	NEG	NEG	NEG	POS	POS	NEG	NEG	POS	POS	POS	NEG	NEG	POS	POS	NEG	POS	POS	POS	NEG	NEG	POS	NEG	POS	POS	POS	NEG	NEG	POS	NEG	NEG	NEG	NEG	NEG	NEG	NEG	NEG	POS	NEG	NEG	POS	POS	NEG	POS	POS
06V08474	CC22-MRSA-IV, Barnim/UK-EMRSA-15	POS	POS	NEG	NEG	POS	NEG	NEG	POS	POS	NEG	NEG	POS	POS	POS	NEG	NEG	POS	POS	NEG	POS	POS	POS	NEG	NEG	POS	NEG	POS	POS	POS	NEG	NEG	POS	NEG	NEG	NEG	NEG	NEG	NEG	NEG	NEG	POS	NEG	NEG	POS	POS	NEG	POS	POS
06V09374	CC22-MRSA-IV, Barnim/UK-EMRSA-15	POS	POS	NEG	NEG	NEG	NEG	AMB	POS	POS	NEG	NEG	POS	POS	POS	NEG	NEG	POS	POS	NEG	POS	POS	POS	NEG	NEG	POS	NEG	POS	POS	POS	NEG	NEG	POS	NEG	NEG	NEG	NEG	NEG	NEG	NEG	NEG	POS	NEG	NEG	POS	POS	NEG	POS	POS
06V09605	CC22-MRSA-IV, Barnim/UK-EMRSA-15	POS	POS	NEG	NEG	NEG	NEG	NEG	POS	POS	NEG	NEG	POS	POS	POS	NEG	NEG	POS	POS	NEG	POS	POS	POS	NEG	NEG	POS	NEG	POS	POS	POS	NEG	NEG	POS	NEG	NEG	NEG	NEG	NEG	NEG	NEG	NEG	POS	NEG	NEG	POS	POS	NEG	POS	POS
06V18687	CC22-MRSA-IV, Barnim/UK-EMRSA-15	POS	POS	NEG	NEG	NEG	NEG	POS	POS	POS	NEG	NEG	POS	POS	POS	NEG	NEG	NEG	POS	NEG	POS	POS	POS	NEG	NEG	POS	NEG	POS	POS	POS	NEG	NEG	POS	NEG	NEG	NEG	NEG	NEG	NEG	NEG	NEG	POS	NEG	NEG	POS	POS	NEG	POS	POS
06V21697	CC22-MRSA-IV, Barnim/UK-EMRSA-15	POS	POS	NEG	NEG	NEG	NEG	NEG	POS	POS	NEG	NEG	POS	POS	POS	NEG	NEG	POS	POS	NEG	POS	POS	POS	NEG	NEG	POS	NEG	POS	POS	POS	NEG	NEG	POS	NEG	NEG	NEG	NEG	NEG	NEG	NEG	NEG	POS	NEG	NEG	POS	POS	NEG	POS	POS
06V23749	CC22-MRSA-IV, Barnim/UK-EMRSA-15	POS	POS	NEG	NEG	POS	NEG	NEG	POS	POS	NEG	NEG	POS	POS	POS	NEG	NEG	POS	POS	NEG	POS	POS	POS	NEG	NEG	POS	NEG	POS	POS	POS	NEG	NEG	POS	NEG	NEG	NEG	NEG	NEG	NEG	NEG	NEG	POS	NEG	NEG	POS	POS	NEG	POS	POS
06V29915	CC22-MRSA-IV, Barnim/UK-EMRSA-15	POS	POS	NEG	NEG	NEG	NEG	NEG	POS	POS	NEG	NEG	POS	POS	POS	NEG	NEG	POS	POS	NEG	POS	POS	POS	NEG	NEG	POS	NEG	POS	POS	POS	NEG	NEG	POS	NEG	NEG	NEG	NEG	NEG	NEG	NEG	NEG	POS	NEG	NEG	POS	POS	NEG	POS	POS
06V31778	CC22-MRSA-IV, Barnim/UK-EMRSA-15	POS	POS	NEG	NEG	NEG	NEG	NEG	POS	POS	NEG	NEG	POS	POS	POS	NEG	NEG	POS	POS	NEG	POS	POS	POS	NEG	NEG	POS	NEG	POS	POS	POS	NEG	NEG	POS	NEG	NEG	NEG	NEG	NEG	NEG	NEG	NEG	POS	NEG	NEG	POS	POS	NEG	POS	POS
06V39089	CC22-MRSA-IV, Barnim/UK-EMRSA-15	POS	POS	NEG	NEG	NEG	NEG	NEG	POS	POS	NEG	NEG	POS	POS	POS	NEG	NEG	POS	POS	NEG	POS	POS	POS	NEG	NEG	POS	NEG	POS	POS	POS	NEG	NEG	POS	NEG	NEG	NEG	NEG	NEG	NEG	NEG	NEG	POS	NEG	NEG	POS	POS	NEG	POS	POS
06V41512	CC22-MRSA-IV, Barnim/UK-EMRSA-15																																																
06V42186	CC22-MRSA-IV, Barnim/UK-EMRSA-15	POS	POS	NEG	NEG	NEG	NEG	NEG	POS	POS	NEG	NEG	POS	POS	POS	NEG	NEG	POS	POS	NEG	POS	POS	POS	NEG	NEG	POS	NEG	POS	POS	POS	NEG	NEG	POS	NEG	NEG	NEG	NEG	NEG	NEG	NEG	NEG	POS	NEG	NEG	POS	POS	NEG	POS	POS
06V42236	CC22-MRSA-IV, Barnim/UK-EMRSA-15	POS	POS	NEG	NEG	NEG	NEG	NEG	POS	POS	NEG	NEG	POS	POS	POS	NEG	NEG	POS	POS	NEG	POS	POS	POS	NEG	NEG	POS	NEG	POS	POS	POS	NEG	NEG	POS	NEG	NEG	NEG	NEG	NEG	NEG	NEG	NEG	POS	NEG	NEG	POS	POS	NEG	POS	POS
06V43697	CC22-MRSA-IV, Barnim/UK-EMRSA-15	POS	POS	NEG	NEG	NEG	NEG	NEG	POS	POS	NEG	NEG	POS	POS	POS	NEG	NEG	POS	POS	NEG	POS	POS	POS	NEG	NEG	POS	NEG	POS	POS	POS	NEG	NEG	POS	NEG	NEG	NEG	NEG	NEG	NEG	NEG	NEG	POS	NEG	NEG	POS	POS	NEG	POS	POS
06V44153	CC22-MRSA-IV, Barnim/UK-EMRSA-15	POS	POS	NEG	NEG	NEG	NEG	NEG	POS	POS	NEG	NEG	POS	POS	POS	NEG	NEG	NEG	POS	NEG	POS	POS	POS	NEG	NEG	POS	NEG	POS	POS	POS	NEG	NEG	POS	NEG	NEG	NEG	NEG	NEG	NEG	NEG	NEG	NEG	NEG	NEG	AMB	POS	NEG	POS	POS
06V45679	CC22-MRSA-IV, Barnim/UK-EMRSA-15	POS	POS	NEG	NEG	NEG	NEG	NEG	POS	POS	NEG	NEG	POS	POS	POS	NEG	NEG	POS	POS	NEG	POS	POS	POS	NEG	NEG	POS	NEG	POS	POS	POS	NEG	NEG	POS	NEG	NEG	NEG	NEG	NEG	NEG	NEG	NEG	NEG	NEG	NEG	AMB	POS	NEG	POS	POS
06V45831	CC22-MRSA-IV, Barnim/UK-EMRSA-15	POS	POS	NEG	NEG	NEG	NEG	NEG	POS	POS	NEG	NEG	POS	POS	POS	NEG	NEG	NEG	POS	NEG	POS	POS	POS	NEG	NEG	POS	NEG	POS	POS	POS	NEG	NEG	POS	NEG	NEG	NEG	NEG	NEG	NEG	NEG	NEG	POS	NEG	NEG	POS	POS	NEG	POS	POS
06V46314	CC22-MRSA-IV, Barnim/UK-EMRSA-15	POS	POS	NEG	NEG	NEG	NEG	NEG	POS	POS	NEG	NEG	POS	POS	POS	NEG	NEG	POS	POS	NEG	POS	POS	POS	NEG	NEG	POS	NEG	POS	POS	POS	NEG	NEG	POS	NEG	NEG	NEG	NEG	NEG	NEG	NEG	NEG	POS	NEG	NEG	POS	POS	NEG	POS	POS
06V34729	CC30-MRSA-IV [PVL+], Southwest Pacific Clone	POS	POS	AMB	POS	NEG	NEG	NEG	POS	POS	NEG	POS	AMB	POS	POS	NEG	NEG	POS	POS	POS	POS	POS	POS	NEG	NEG	POS	NEG	POS	POS	POS	NEG	POS	NEG	NEG	POS	NEG	POS	POS	NEG	NEG	NEG	POS	AMB	POS	AMB	NEG	NEG	NEG	NEG
06B09900	CC45-MRSA-IV, Berlin EMRSA	POS	POS	NEG	NEG	NEG	NEG	POS	POS	POS	AMB	NEG	POS	POS	POS	NEG	POS	AMB	POS	POS	POS	NEG	POS	POS	NEG	POS	NEG	POS	POS	POS	NEG	NEG	POS	NEG	POS	NEG	POS	AMB	NEG	AMB	NEG	POS	NEG	POS	NEG	NEG	NEG	NEG	NEG
06V00922	CC45-MRSA-IV, Berlin EMRSA	POS	POS	NEG	NEG	NEG	NEG	POS	POS	POS	AMB	NEG	POS	POS	POS	NEG	POS	AMB	POS	POS	POS	NEG	POS	POS	NEG	POS	NEG	POS	POS	POS	NEG	NEG	POS	NEG	POS	NEG	POS	AMB	NEG	NEG	NEG	POS	NEG	POS	NEG	NEG	NEG	NEG	NEG
06V09551	CC45-MRSA-IV, Berlin EMRSA	POS	POS	NEG	NEG	NEG	NEG	POS	POS	POS	AMB	NEG	POS	POS	POS	NEG	POS	AMB	POS	POS	POS	NEG	POS	POS	NEG	POS	NEG	POS	POS	POS	NEG	NEG	POS	NEG	POS	NEG	POS	AMB	NEG	AMB	NEG	POS	NEG	POS	NEG	NEG	NEG	NEG	NEG
06V10719	CC45-MRSA-IV, Berlin EMRSA	POS	POS	NEG	NEG	NEG	NEG	POS	POS	POS	AMB	NEG	POS	POS	POS	NEG	POS	AMB	POS	POS	POS	NEG	POS	POS	NEG	POS	NEG	POS	POS	POS	NEG	NEG	POS	NEG	POS	NEG	POS	AMB	NEG	NEG	NEG	POS	NEG	POS	NEG	NEG	NEG	NEG	NEG
06V11090	CC45-MRSA-IV, Berlin EMRSA	POS	POS	NEG	NEG	NEG	NEG	POS	POS	POS	AMB	NEG	POS	POS	POS	NEG	POS	AMB	POS	POS	POS	NEG	POS	POS	NEG	POS	NEG	POS	POS	POS	NEG	NEG	POS	NEG	POS	NEG	POS	AMB	NEG	NEG	NEG	POS	NEG	POS	NEG	NEG	NEG	NEG	NEG
06V14383	CC45-MRSA-IV, Berlin EMRSA	POS	POS	NEG	NEG	NEG	NEG	POS	POS	POS	AMB	NEG	POS	POS	POS	NEG	POS	AMB	POS	POS	POS	NEG	POS	POS	NEG	POS	NEG	POS	POS	POS	NEG	NEG	POS	NEG	POS	NEG	POS	AMB	NEG	NEG	NEG	POS	NEG	POS	NEG	NEG	NEG	NEG	NEG
06V21475	CC45-MRSA-IV, Berlin EMRSA	POS	POS	NEG	NEG	NEG	NEG	POS	POS	POS	AMB	NEG	POS	POS	POS	NEG	POS	AMB	POS	POS	POS	NEG	POS	POS	NEG	POS	NEG	POS	POS	POS	NEG	NEG	POS	NEG	POS	NEG	POS	AMB	NEG	NEG	NEG	POS	NEG	POS	NEG	NEG	NEG	NEG	NEG
06V24231	CC45-MRSA-IV, Berlin EMRSA	POS	POS	NEG	NEG	NEG	NEG	POS	POS	POS	AMB	NEG	POS	POS	POS	NEG	POS	AMB	POS	POS	POS	NEG	POS	POS	NEG	POS	NEG	POS	POS	POS	NEG	NEG	POS	NEG	POS	NEG	POS	AMB	NEG	NEG	NEG	POS	NEG	POS	NEG	NEG	NEG	NEG	NEG
06V32992	CC45-MRSA-IV, Berlin EMRSA	POS	POS	NEG	NEG	NEG	NEG	POS	POS	POS	AMB	NEG	POS	POS	POS	NEG	POS	AMB	POS	POS	POS	NEG	POS	POS	NEG	POS	NEG	POS	POS	POS	NEG	NEG	POS	NEG	POS	NEG	POS	AMB	NEG	NEG	NEG	POS	NEG	POS	NEG	NEG	NEG	NEG	NEG
06V38753	CC45-MRSA-IV, Berlin EMRSA	POS	POS	NEG	NEG	NEG	NEG	POS	POS	POS	NEG	NEG	POS	POS	POS	NEG	POS	AMB	POS	POS	POS	NEG	POS	POS	NEG	POS	NEG	POS	POS	POS	NEG	NEG	POS	NEG	POS	NEG	POS	AMB	NEG	NEG	NEG	POS	NEG	POS	NEG	NEG	NEG	NEG	NEG
06V39848	CC45-MRSA-IV, Berlin EMRSA																																																
06V42259	CC45-MRSA-IV, Berlin EMRSA	POS	POS	AMB	NEG	AMB	NEG	POS	POS	POS	POS	AMB	AMB	POS	POS	NEG	AMB	POS	POS	POS	POS	NEG	POS	POS	NEG	POS	NEG	POS	POS	POS	NEG	NEG	POS	NEG	POS	NEG	POS	AMB	NEG	AMB	NEG	POS	NEG	POS	NEG	NEG	NEG	NEG	NEG
06V33960	CC80-MRSA-IV [PVL+], European caMRSA Clone																																																

ISOLATE:	STRAIN	ADHAESION FACTORS / GENES ENCODING MICROBIAL SURFACE COMPONENTS RECOGNIZING ADHESIVE MATRIX MOLECULES (MSCRAMM GENES)																		IMMUNOD.ANTIGEN B
		Ser-Asp rich fibrinogen-/bone sialoprotein-binding protein C							Ser-Asp rich fibrinogen-/bone sialoprotein-binding protein D					van Willebrand factor binding protein						immunodominant antigen B
		sdrC (total)	sdrC (consensus)	sdrC (B1)	sdrC (COL)	sdrC (Mu50)	sdrC (MW2+MRSA252+RF122)	sdrC (other than MRSA252+RF122)	sdrD (total)	sdrD (consensus)	sdrD (COL+MW2)	sdrD (Mu50)	sdrD (other)	vwb (total)	vwb (consensus)	vwb (COL+MW2)	vwb (MRSA252)	vwb (Mu50)	vwb (RF122)	isaB
**2005**																				
05B01179	CC5/ST228-MRSA-I, South German EMRSA	POS	POS	NEG	NEG	POS	NEG	POS	POS	POS	NEG	POS	NEG	POS	POS	NEG	NEG	POS	NEG	POS
05B02687	CC5/ST228-MRSA-I, South German EMRSA	POS	POS	NEG	NEG	POS	NEG	POS	POS	POS	NEG	POS	NEG	POS	POS	NEG	NEG	POS	NEG	POS
05V08483	CC5/ST228-MRSA-I, South German EMRSA	POS	POS	NEG	NEG	POS	NEG	POS	NEG	NEG	NEG	NEG	NEG	POS	POS	NEG	NEG	POS	NEG	POS
05V19001	CC5/ST228-MRSA-I, South German EMRSA	POS	POS	NEG	NEG	POS	NEG	POS	POS	POS	NEG	POS	NEG	POS	POS	NEG	NEG	POS	NEG	POS
05B04626	CC5-MRSA-II, Rhine-Hesse EMRSA/UK-EMRSA-3	POS	POS	NEG	NEG	POS	NEG	POS	POS	POS	NEG	POS	NEG	POS	POS	NEG	NEG	POS	NEG	POS
05B07903	CC5-MRSA-II, Rhine-Hesse EMRSA/UK-EMRSA-3	POS	POS	NEG	NEG	POS	NEG	POS	POS	POS	NEG	POS	NEG	POS	POS	NEG	NEG	POS	NEG	AMB
05U00062	CC5-MRSA-II, Rhine-Hesse EMRSA/UK-EMRSA-3	POS	POS	NEG	NEG	POS	NEG	POS	POS	POS	NEG	POS	NEG	POS	POS	NEG	NEG	POS	NEG	POS
05U03117	CC5-MRSA-II, Rhine-Hesse EMRSA/UK-EMRSA-3	POS	POS	NEG	NEG	POS	NEG	POS	POS	POS	NEG	POS	NEG	POS	POS	NEG	NEG	POS	NEG	POS
05U10392	CC5-MRSA-II, Rhine-Hesse EMRSA/UK-EMRSA-3	POS	POS	NEG	NEG	POS	NEG	POS	POS	POS	NEG	POS	NEG	POS	POS	NEG	NEG	POS	NEG	POS
05V03312	CC5-MRSA-II, Rhine-Hesse EMRSA/UK-EMRSA-3	POS	POS	NEG	NEG	POS	NEG	POS	POS	POS	NEG	POS	NEG	POS	POS	NEG	NEG	POS	NEG	POS
05V08095	CC5-MRSA-II, Rhine-Hesse EMRSA/UK-EMRSA-3	POS	POS	NEG	NEG	POS	NEG	POS	POS	POS	NEG	POS	NEG	POS	POS	NEG	NEG	POS	NEG	POS
05V09015	CC5-MRSA-II, Rhine-Hesse EMRSA/UK-EMRSA-3	POS	POS	NEG	NEG	POS	NEG	POS	POS	POS	NEG	POS	NEG	POS	POS	NEG	NEG	POS	NEG	POS
05V09326	CC5-MRSA-II, Rhine-Hesse EMRSA/UK-EMRSA-3	POS	POS	NEG	NEG	POS	NEG	POS	POS	POS	NEG	POS	NEG	POS	POS	NEG	NEG	POS	NEG	POS
05V10265	CC5-MRSA-II, Rhine-Hesse EMRSA/UK-EMRSA-3	POS	POS	NEG	NEG	POS	NEG	POS	POS	POS	NEG	POS	NEG	POS	POS	NEG	NEG	POS	NEG	POS
05V13052	CC5-MRSA-II, Rhine-Hesse EMRSA/UK-EMRSA-3	POS	POS	NEG	NEG	POS	NEG	POS	POS	POS	NEG	POS	NEG	POS	POS	NEG	NEG	POS	NEG	POS
05V13470	CC5-MRSA-II, Rhine-Hesse EMRSA/UK-EMRSA-3	POS	POS	NEG	NEG	POS	NEG	POS	POS	POS	NEG	POS	NEG	POS	POS	NEG	NEG	POS	NEG	POS
05V13891	CC5-MRSA-II, Rhine-Hesse EMRSA/UK-EMRSA-3	POS	POS	NEG	NEG	POS	NEG	POS	POS	POS	NEG	AMB	NEG	POS	POS	NEG	NEG	POS	NEG	POS
05V15616	CC5-MRSA-II, Rhine-Hesse EMRSA/UK-EMRSA-3																			
05V16845	CC5-MRSA-II, Rhine-Hesse EMRSA/UK-EMRSA-3	POS	POS	NEG	NEG	POS	NEG	POS	POS	POS	NEG	POS	NEG	POS	POS	NEG	NEG	POS	NEG	POS
05V17541	CC5-MRSA-II, Rhine-Hesse EMRSA/UK-EMRSA-3	POS	POS	NEG	NEG	POS	NEG	POS	POS	POS	NEG	AMB	NEG	POS	POS	NEG	NEG	POS	NEG	POS
05V19105	CC5-MRSA-II, Rhine-Hesse EMRSA/UK-EMRSA-3																			
05V22148	CC5-MRSA-II, Rhine-Hesse EMRSA/UK-EMRSA-3	POS	POS	NEG	NEG	POS	NEG	POS	POS	POS	NEG	POS	NEG	POS	POS	NEG	NEG	POS	NEG	POS
05V33740	CC5-MRSA-II, Rhine-Hesse EMRSA/UK-EMRSA-3	POS	POS	NEG	NEG	POS	NEG	POS	POS	POS	NEG	POS	NEG	POS	POS	NEG	NEG	POS	NEG	POS
05V38425	CC5-MRSA-II, Rhine-Hesse EMRSA/UK-EMRSA-3	POS	POS	NEG	NEG	POS	NEG	POS	POS	POS	NEG	POS	NEG	POS	POS	NEG	NEG	POS	NEG	POS
05V39987	CC5-MRSA-II, Rhine-Hesse EMRSA/UK-EMRSA-3	POS	POS	NEG	NEG	POS	NEG	POS	POS	POS	NEG	POS	NEG	POS	POS	NEG	NEG	POS	NEG	POS
05F00089	CC5-MRSA-IV, Paediatric clone	POS	POS	NEG	NEG	NEG	NEG	POS	POS	POS	NEG	POS	NEG	POS	POS	NEG	NEG	POS	NEG	POS
05V01416	CC5-MRSA-IV, Paediatric clone	POS	POS	NEG	NEG	POS	NEG	POS	NEG	AMB	NEG	AMB	NEG	POS	POS	NEG	NEG	POS	NEG	POS
05V16386	CC8-MRSA-IV, USA300	POS	POS	NEG	POS	NEG	NEG	POS	POS	POS	AMB	NEG	NEG	POS	POS	POS	NEG	NEG	NEG	POS
05V04920	CC8-MRSA-IV, USA500	POS	POS	NEG	POS	NEG	NEG	POS	POS	POS	AMB	NEG	NEG	POS	POS	POS	NEG	NEG	NEG	POS
05V21159	CC8-MRSA-IV, USA500	POS	POS	NEG	POS	NEG	NEG	POS	POS	POS	POS	NEG	NEG	POS	POS	POS	NEG	NEG	NEG	POS
05B00867	CC22-MRSA-IV, Barnim/UK-EMRSA-15	POS	POS	NEG	NEG	POS	NEG	POS	POS	POS	NEG	NEG	POS	POS	POS	NEG	NEG	NEG	POS	NEG
05V03174	CC22-MRSA-IV, Barnim/UK-EMRSA-15	POS	POS	NEG	NEG	POS	NEG	POS	POS	POS	NEG	NEG	POS	POS	POS	NEG	NEG	NEG	POS	NEG
05V07331	CC22-MRSA-IV, Barnim/UK-EMRSA-15	POS	POS	NEG	NEG	POS	NEG	POS	POS	POS	NEG	NEG	POS	POS	POS	NEG	NEG	NEG	POS	NEG
05V07371	CC22-MRSA-IV, Barnim/UK-EMRSA-15	POS	POS	NEG	NEG	POS	NEG	POS	POS	POS	NEG	NEG	POS	POS	POS	NEG	NEG	NEG	POS	NEG
05V08145	CC22-MRSA-IV, Barnim/UK-EMRSA-15	POS	POS	NEG	NEG	POS	NEG	POS	POS	POS	NEG	NEG	POS	POS	POS	NEG	NEG	NEG	POS	NEG
05V08595	CC22-MRSA-IV, Barnim/UK-EMRSA-15	POS	POS	NEG	NEG	POS	NEG	POS	POS	POS	NEG	NEG	POS	POS	POS	NEG	NEG	NEG	POS	NEG
05V12803	CC22-MRSA-IV, Barnim/UK-EMRSA-15	POS	POS	NEG	NEG	POS	NEG	POS	POS	POS	NEG	NEG	POS	POS	POS	NEG	NEG	NEG	POS	NEG
05V14596	CC22-MRSA-IV, Barnim/UK-EMRSA-15	POS	POS	NEG	NEG	POS	NEG	POS	POS	POS	NEG	NEG	POS	POS	POS	NEG	NEG	NEG	POS	NEG
05V16691	CC22-MRSA-IV, Barnim/UK-EMRSA-15																			
05V18550	CC22-MRSA-IV, Barnim/UK-EMRSA-15	POS	POS	NEG	NEG	POS	AMB	POS	POS	POS	NEG	NEG	POS	POS	POS	NEG	NEG	NEG	POS	NEG
05V19034	CC22-MRSA-IV, Barnim/UK-EMRSA-15	POS	POS	NEG	NEG	POS	NEG	POS	POS	POS	NEG	NEG	POS	POS	POS	NEG	NEG	NEG	POS	NEG
05V19333	CC22-MRSA-IV, Barnim/UK-EMRSA-15																			
05V19701	CC22-MRSA-IV, Barnim/UK-EMRSA-15	POS	POS	NEG	NEG	POS	NEG	POS	POS	POS	NEG	NEG	POS	POS	POS	NEG	NEG	NEG	POS	NEG
05V20072	CC22-MRSA-IV, Barnim/UK-EMRSA-15	POS	POS	NEG	NEG	POS	NEG	POS	POS	POS	NEG	NEG	POS	POS	POS	NEG	NEG	NEG	POS	NEG
05V24930	CC22-MRSA-IV, Barnim/UK-EMRSA-15	POS	POS	NEG	NEG	POS	NEG	POS	POS	POS	NEG	NEG	POS	POS	POS	NEG	NEG	NEG	POS	NEG
05V31461	CC22-MRSA-IV, Barnim/UK-EMRSA-15	POS	POS	NEG	NEG	POS	NEG	POS	POS	POS	NEG	NEG	POS	POS	POS	NEG	NEG	NEG	POS	NEG
05B05671	CC45-MRSA-IV, Berlin EMRSA	POS	POS	POS	NEG	NEG	NEG	NEG	POS	POS	POS	NEG	NEG	POS	NEG	NEG	POS	NEG	NEG	NEG
05V01452	CC45-MRSA-IV, Berlin EMRSA	POS	POS	POS	NEG	NEG	POS	NEG	POS	POS	POS	NEG	NEG	POS	AMB	NEG	POS	NEG	NEG	NEG
05V01617	CC45-MRSA-IV, Berlin EMRSA	POS	POS	POS	NEG	NEG	NEG	NEG	POS	POS	POS	NEG	NEG	POS	AMB	NEG	POS	NEG	NEG	NEG
05V16986	CC45-MRSA-IV, Berlin EMRSA	POS	POS	POS	NEG	NEG	NEG	NEG	POS	POS	AMB	NEG	NEG	POS	NEG	NEG	POS	NEG	NEG	NEG
05V18357	CC45-MRSA-IV, Berlin EMRSA	POS	POS	POS	NEG	NEG	NEG	NEG	POS	POS	AMB	NEG	NEG	POS	NEG	NEG	POS	NEG	NEG	NEG
05V23111	CC45-MRSA-IV, Berlin EMRSA	POS	POS	POS	NEG	NEG	NEG	NEG	POS	POS	POS	NEG	NEG	POS	AMB	NEG	POS	NEG	NEG	NEG
05V23414	CC45-MRSA-IV, Berlin EMRSA																			
05V23858	CC45-MRSA-IV, Berlin EMRSA	POS	POS	POS	NEG	NEG	NEG	NEG	POS	POS	POS	NEG	NEG	POS	NEG	NEG	POS	NEG	NEG	NEG
05V26241	CC45-MRSA-IV, Berlin EMRSA	POS	POS	POS	NEG	NEG	NEG	NEG	POS	POS	POS	NEG	NEG	POS	NEG	NEG	POS	NEG	NEG	NEG
05V28399	CC45-MRSA-IV, Berlin EMRSA	POS	POS	POS	NEG	NEG	NEG	NEG	POS	POS	POS	NEG	NEG	POS	AMB	NEG	POS	NEG	NEG	NEG
05V32647	CC45-MRSA-IV, Berlin EMRSA																			
05V37969	CC45-MRSA-IV, Berlin EMRSA	POS	POS	POS	NEG	NEG	NEG	NEG	POS	POS	POS	NEG	NEG	POS	AMB	NEG	POS	NEG	NEG	NEG
05U10018	CC398-MRSA-V	POS	POS	NEG	NEG	NEG	NEG	NEG	POS	POS	NEG	NEG	NEG	POS	POS	NEG	NEG	NEG	NEG	NEG
**2006**																				
06V36353	CC1-MRSA-IV, WA MRSA-1/57	POS	POS	NEG	NEG	NEG	POS	POS	POS	POS	AMB	NEG	NEG	POS	POS	POS	NEG	NEG	NEG	POS
06V40202	CC5/ST228-MRSA-I, South German EMRSA																			
06V41266	CC5/ST228-MRSA-I, South German EMRSA	POS	POS	NEG	NEG	POS	NEG	POS	POS	POS	NEG	POS	NEG	POS	POS	NEG	NEG	POS	NEG	POS
06B09097	CC5-MRSA-II, Rhine-Hesse EMRSA/UK-EMRSA-3																			
06F00244	CC5-MRSA-II, Rhine-Hesse EMRSA/UK-EMRSA-3	POS	POS	NEG	NEG	POS	NEG	POS	POS	POS	NEG	POS	NEG	POS	POS	NEG	NEG	POS	NEG	POS
06V03913	CC5-MRSA-II, Rhine-Hesse EMRSA/UK-EMRSA-3	POS	POS	NEG	NEG	POS	NEG	POS	POS	POS	NEG	POS	NEG	POS	POS	NEG	NEG	POS	NEG	POS
06V04337	CC5-MRSA-II, Rhine-Hesse EMRSA/UK-EMRSA-3	POS	POS	NEG	NEG	POS	NEG	POS	POS	POS	NEG	POS	NEG	POS	POS	NEG	NEG	POS	NEG	POS
06V06785	CC5-MRSA-II, Rhine-Hesse EMRSA/UK-EMRSA-3	POS	POS	NEG	NEG	POS	NEG	POS	POS	POS	NEG	POS	NEG	POS	POS	NEG	NEG	POS	NEG	POS
06V12258	CC5-MRSA-II, Rhine-Hesse EMRSA/UK-EMRSA-3	POS	POS	NEG	NEG	POS	AMB	POS	POS	POS	NEG	POS	NEG	POS	POS	NEG	NEG	POS	NEG	POS
06V20553	CC5-MRSA-II, Rhine-Hesse EMRSA/UK-EMRSA-3	POS	POS	NEG	NEG	POS	NEG	POS	POS	POS	NEG	POS	NEG	POS	POS	NEG	NEG	POS	NEG	POS
06V22564	CC5-MRSA-II, Rhine-Hesse EMRSA/UK-EMRSA-3																			
06V23750	CC5-MRSA-II, Rhine-Hesse EMRSA/UK-EMRSA-3	POS	POS	NEG	NEG	POS	NEG	POS	POS	POS	NEG	POS	NEG	POS	POS	NEG	NEG	POS	NEG	POS
06V27332	CC5-MRSA-II, Rhine-Hesse EMRSA/UK-EMRSA-3	POS	POS	NEG	NEG	POS	NEG	POS	POS	POS	NEG	POS	NEG	POS	POS	NEG	NEG	POS	NEG	POS
06V29484	CC5-MRSA-II, Rhine-Hesse EMRSA/UK-EMRSA-3	POS	POS	NEG	NEG	POS	NEG	POS	POS	POS	NEG	POS	NEG	POS	POS	NEG	NEG	POS	NEG	POS
06V31278	CC5-MRSA-II, Rhine-Hesse EMRSA/UK-EMRSA-3																			
06V36043	CC5-MRSA-II, Rhine-Hesse EMRSA/UK-EMRSA-3	POS	POS	NEG	NEG	POS	NEG	POS	POS	POS	NEG	POS	NEG	POS	POS	NEG	NEG	POS	NEG	POS
06V36349	CC5-MRSA-II, Rhine-Hesse EMRSA/UK-EMRSA-3	POS	POS	NEG	NEG	POS	NEG	POS	POS	POS	NEG	POS	NEG	POS	POS	NEG	NEG	POS	NEG	POS
06V39695	CC5-MRSA-II, Rhine-Hesse EMRSA/UK-EMRSA-3	POS	POS	NEG	NEG	POS	NEG	POS	POS	POS	NEG	POS	NEG	POS	POS	NEG	NEG	POS	NEG	POS
06V40571	CC5-MRSA-II, Rhine-Hesse EMRSA/UK-EMRSA-3																			
06V42188	CC5-MRSA-II, Rhine-Hesse EMRSA/UK-EMRSA-3	POS	POS	NEG	NEG	POS	NEG	POS	POS	POS	NEG	POS	NEG	POS	POS	NEG	NEG	POS	NEG	POS
06V42146	CC8-MRSA-IV, Lyon Clone/UK-EMRSA-2																			
06V28560	CC8-MRSA-V	POS	POS	NEG	POS	NEG	NEG	POS	POS	POS	POS	NEG	NEG	POS	POS	POS	NEG	NEG	NEG	NEG
06F00837	CC22-MRSA-IV, Barnim/UK-EMRSA-15	POS	POS	NEG	NEG	POS	NEG	POS	POS	POS	NEG	NEG	POS	POS	POS	NEG	NEG	NEG	POS	NEG
06F01638	CC22-MRSA-IV, Barnim/UK-EMRSA-15	POS	POS	NEG	NEG	POS	NEG	POS	POS	POS	NEG	NEG	POS	POS	POS	NEG	NEG	NEG	POS	NEG
06F04643	CC22-MRSA-IV, Barnim/UK-EMRSA-15																			
06F05580	CC22-MRSA-IV, Barnim/UK-EMRSA-15	POS	POS	NEG	NEG	POS	NEG	POS	POS	POS	NEG	NEG	POS	POS	POS	NEG	NEG	NEG	POS	NEG
06U05469	CC22-MRSA-IV, Barnim/UK-EMRSA-15	POS	POS	NEG	NEG	POS	NEG	POS	POS	POS	NEG	NEG	POS	POS	POS	NEG	NEG	NEG	POS	NEG
06U17314	CC22-MRSA-IV, Barnim/UK-EMRSA-15	POS	POS	NEG	NEG	POS	NEG	POS	POS	POS	NEG	NEG	POS	POS	POS	NEG	NEG	NEG	POS	NEG
06V08377	CC22-MRSA-IV, Barnim/UK-EMRSA-15	POS	POS	NEG	NEG	POS	NEG	POS	POS	POS	NEG	NEG	POS	POS	POS	NEG	NEG	NEG	POS	NEG
06V08474	CC22-MRSA-IV, Barnim/UK-EMRSA-15	POS	POS	NEG	NEG	POS	NEG	POS	POS	POS	NEG	NEG	POS	POS	POS	NEG	NEG	NEG	POS	NEG
06V09374	CC22-MRSA-IV, Barnim/UK-EMRSA-15	POS	POS	NEG	NEG	POS	NEG	POS	POS	POS	NEG	NEG	POS	POS	POS	NEG	NEG	NEG	POS	NEG
06V09605	CC22-MRSA-IV, Barnim/UK-EMRSA-15	POS	POS	NEG	NEG	POS	NEG	POS	POS	POS	NEG	NEG	POS	POS	POS	NEG	NEG	NEG	POS	NEG
06V18687	CC22-MRSA-IV, Barnim/UK-EMRSA-15	POS	POS	NEG	NEG	POS	NEG	POS	POS	POS	NEG	NEG	POS	POS	POS	NEG	NEG	NEG	POS	NEG
06V21697	CC22-MRSA-IV, Barnim/UK-EMRSA-15	POS	POS	NEG	NEG	POS	NEG	POS	POS	POS	NEG	NEG	POS	POS	POS	NEG	NEG	NEG	POS	NEG
06V23749	CC22-MRSA-IV, Barnim/UK-EMRSA-15	POS	POS	NEG	NEG	POS	NEG	POS	POS	POS	NEG	NEG	POS	POS	POS	NEG	NEG	NEG	POS	NEG
06V29915	CC22-MRSA-IV, Barnim/UK-EMRSA-15	POS	POS	NEG	NEG	POS	AMB	POS	POS	POS	NEG	NEG	POS	POS	POS	NEG	NEG	NEG	POS	NEG
06V31778	CC22-MRSA-IV, Barnim/UK-EMRSA-15	POS	POS	NEG	NEG	POS	NEG	POS	POS	POS	NEG	NEG	POS	POS	POS	NEG	NEG	NEG	POS	NEG
06V39089	CC22-MRSA-IV, Barnim/UK-EMRSA-15	POS	POS	NEG	NEG	POS	NEG	POS	POS	POS	NEG	NEG	POS	POS	POS	NEG	NEG	NEG	POS	NEG
06V41512	CC22-MRSA-IV, Barnim/UK-EMRSA-15																			
06V42186	CC22-MRSA-IV, Barnim/UK-EMRSA-15	POS	POS	NEG	NEG	POS	NEG	POS	POS	POS	NEG	NEG	POS	POS	POS	NEG	NEG	NEG	POS	NEG
06V42236	CC22-MRSA-IV, Barnim/UK-EMRSA-15	POS	POS	NEG	NEG	POS	NEG	POS	POS	POS	NEG	NEG	POS	POS	POS	NEG	NEG	NEG	POS	NEG
06V43697	CC22-MRSA-IV, Barnim/UK-EMRSA-15	POS	POS	NEG	NEG	POS	NEG	POS	POS	POS	NEG	NEG	POS	POS	POS	NEG	NEG	NEG	POS	NEG
06V44153	CC22-MRSA-IV, Barnim/UK-EMRSA-15	POS	POS	NEG	NEG	POS	NEG	POS	POS	POS	NEG	NEG	POS	POS	POS	NEG	NEG	NEG	POS	NEG
06V45679	CC22-MRSA-IV, Barnim/UK-EMRSA-15	POS	POS	NEG	NEG	POS	NEG	POS	POS	POS	NEG	NEG	POS	POS	POS	NEG	NEG	NEG	POS	NEG
06V45831	CC22-MRSA-IV, Barnim/UK-EMRSA-15	POS	POS	NEG	NEG	POS	NEG	POS	POS	POS	NEG	NEG	POS	POS	POS	NEG	NEG	NEG	POS	NEG
06V46314	CC22-MRSA-IV, Barnim/UK-EMRSA-15	POS	POS	NEG	NEG	POS	NEG	POS	POS	POS	NEG	NEG	POS	POS	POS	NEG	NEG	NEG	POS	NEG
06V34729	CC30-MRSA-IV [PVL+], Southwest Pacific Clone	POS	POS	NEG	NEG	AMB	POS	NEG	POS	POS	NEG	NEG	POS	POS	POS	NEG	POS	NEG	NEG	NEG
06B09900	CC45-MRSA-IV, Berlin EMRSA	POS	POS	POS	NEG	NEG	NEG	NEG	POS	POS	POS	NEG	NEG	POS	NEG	NEG	POS	NEG	NEG	NEG
06V00922	CC45-MRSA-IV, Berlin EMRSA	POS	POS	POS	NEG	NEG	NEG	NEG	POS	POS	POS	NEG	NEG	POS	NEG	NEG	POS	NEG	NEG	NEG
06V09551	CC45-MRSA-IV, Berlin EMRSA	POS	POS	POS	NEG	NEG	NEG	NEG	POS	POS	POS	NEG	NEG	POS	AMB	NEG	POS	NEG	NEG	NEG
06V10719	CC45-MRSA-IV, Berlin EMRSA	POS	POS	POS	NEG	NEG	NEG	NEG	POS	POS	POS	NEG	NEG	POS	NEG	NEG	POS	NEG	NEG	NEG
06V11090	CC45-MRSA-IV, Berlin EMRSA	POS	POS	POS	NEG	NEG	NEG	NEG	POS	POS	POS	NEG	NEG	POS	NEG	NEG	POS	NEG	NEG	NEG
06V14383	CC45-MRSA-IV, Berlin EMRSA	POS	POS	POS	NEG	NEG	NEG	NEG	POS	POS	POS	NEG	NEG	POS	NEG	NEG	POS	NEG	NEG	NEG
06V21475	CC45-MRSA-IV, Berlin EMRSA	POS	POS	POS	NEG	NEG	NEG	NEG	POS	POS	POS	NEG	NEG	POS	NEG	NEG	POS	NEG	NEG	NEG
06V24231	CC45-MRSA-IV, Berlin EMRSA	POS	POS	POS	NEG	NEG	NEG	NEG	POS	POS	POS	NEG	NEG	POS	NEG	NEG	POS	NEG	NEG	NEG
06V32992	CC45-MRSA-IV, Berlin EMRSA	POS	POS	POS	NEG	NEG	NEG	NEG	POS	POS	POS	NEG	NEG	POS	NEG	NEG	POS	NEG	NEG	NEG
06V38753	CC45-MRSA-IV, Berlin EMRSA	POS	POS	POS	NEG	NEG	NEG	NEG	POS	POS	POS	NEG	NEG	POS	NEG	NEG	POS	NEG	NEG	NEG
06V39848	CC45-MRSA-IV, Berlin EMRSA																			
06V42259	CC45-MRSA-IV, Berlin EMRSA	POS	POS	POS	NEG	NEG	AMB	NEG	POS	POS	POS	NEG	NEG	POS	POS	NEG	POS	NEG	NEG	NEG
06V33960	CC80-MRSA-IV [PVL+], European caMRSA Clone																			

ISOLATE:	IMMUNOD.ANTIGEN B	DEFENSIN RESIST.		TRANSFERRIN BINDING PROT			PUTATIVE TRANSPORTER		TYPE I RESTRICTION-MODIFICATION SYSTEM, SINGLE SEQUENCE SPECIFICITY PROTEIN												MISCELLANEOUS GENES			HYALURONATE LYASE							
	immunodominant antigen B	defensin resistance protein		transferrin-binding protein			hypothetical protein, similar to integral membrane protein LmrP		type I site-specific deoxyribo-nuclease subunit, 1st locus	type I site-specific deoxyribonuclease subunit, 2nd locus			type I site-specific deoxyribonuclease subunit, 3rd locus					type I site-specific deoxyribonuclease subunit, unknown locus			hypothetical protein, located next to serine protease operon	Unspecific efflux/trans-porter	hypothetical protein	hyaluronate lyase, first / second locus			hyaluronate lyase, second locus				
	isaB (MRSA252)	mprF (COL+MW2)	mprF (Mu50+MRSA252)	isdA (consensus)	isdA (MRSA252)	isdA (other than MRSA252)	lmrP (other than RF122)	lmrP (RF122)	hsdS1 (RF122)	hsdS2 (Mu50+N315+COL+USA300+NCTC8325)	hsdS2 (MW2+MSSA476)	hsdS2 (MRSA252)	hsdS3 (all other than RF122+ MRSA252)	hsdS3 (COL+USA300+ NCTC8325+MW2+MSSA476+RF122)	hsdS3 (Mu50+N315)	hsdS3 (CC51+ MRSA252)	hsdS3 (MRSA252)	hsdSx (CC25)	hsdSx (CC15)	hsdSx (etd)	Q2FXC0	Q2YUB3	Q7A4X2	hysA1 (MRSA252)	hysA1 (MRSA252+RF122) and/or hysA2 (consensus)	hysA1 (MRSA252+RF122) and/or hysA2 (COL+USA300)	hysA2 (all other than MRSA252)	hysA2 (COL+USA300+ NCTC8325)	hysA2 (all other than COL+USA300+NCTC8325)	hysA2 (all other than COL+USA300+NCTC8325)	hysA2 (MRSA252)
**2005**																															
05B01179	AMB	AMB	POS	POS	NEG	POS	POS	NEG	NEG	NEG	NEG	NEG	POS	NEG	POS	NEG	NEG	POS	NEG	NEG	NEG	NEG	POS	NEG	POS	NEG	AMB	NEG	POS	POS	NEG
05B02687	AMB	AMB	POS	POS	NEG	POS	POS	NEG	NEG	NEG	NEG	NEG	POS	NEG	POS	NEG	NEG	POS	NEG	NEG	NEG	NEG	POS	NEG	POS	NEG	AMB	NEG	POS	POS	NEG
05V08483	POS	POS	POS	POS	NEG	POS	POS	NEG	NEG	NEG	NEG	NEG	POS	NEG	POS	NEG	NEG	POS	NEG	NEG	NEG	NEG	NEG	NEG	POS	NEG	NEG	NEG	POS	POS	NEG
05V19001	AMB	AMB	POS	POS	NEG	POS	POS	NEG	NEG	NEG	NEG	NEG	POS	NEG	POS	NEG	NEG	POS	NEG	NEG	NEG	NEG	NEG	NEG	POS	NEG	NEG	NEG	POS	POS	NEG
05B04626	AMB	NEG	POS	POS	NEG	POS	POS	NEG	NEG	POS	NEG	NEG	POS	NEG	POS	NEG	NEG	POS	NEG	NEG	NEG	NEG	POS	NEG	POS	NEG	AMB	NEG	POS	POS	NEG
05B07903	POS	AMB	POS	POS	NEG	POS	POS	NEG	NEG	POS	NEG	NEG	POS	NEG	POS	NEG	NEG	POS	NEG	NEG	NEG	NEG	POS	NEG	POS	NEG	POS	NEG	POS	POS	NEG
05U00062	AMB	AMB	POS	POS	NEG	POS	POS	NEG	NEG	POS	NEG	NEG	POS	NEG	POS	NEG	NEG	POS	NEG	NEG	NEG	NEG	POS	NEG	POS	NEG	POS	NEG	POS	POS	NEG
05U03117	AMB	AMB	POS	POS	NEG	POS	POS	NEG	NEG	POS	NEG	NEG	POS	NEG	POS	NEG	NEG	POS	NEG	NEG	NEG	NEG	POS	NEG	POS	NEG	POS	NEG	POS	POS	NEG
05U10392	AMB	AMB	POS	POS	NEG	POS	POS	NEG	NEG	POS	NEG	NEG	POS	NEG	POS	NEG	NEG	POS	NEG	NEG	NEG	NEG	POS	NEG	POS	NEG	AMB	NEG	POS	POS	NEG
05V03312	AMB	AMB	POS	POS	NEG	POS	POS	NEG	NEG	POS	NEG	NEG	POS	NEG	POS	NEG	NEG	POS	NEG	NEG	NEG	NEG	POS	NEG	POS	NEG	POS	NEG	POS	POS	NEG
05V08095	AMB	AMB	POS	POS	NEG	POS	POS	NEG	NEG	POS	NEG	NEG	POS	NEG	POS	NEG	NEG	POS	NEG	NEG	NEG	NEG	POS	NEG	POS	NEG	POS	NEG	POS	POS	NEG
05V09015	AMB	AMB	POS	POS	NEG	POS	POS	NEG	NEG	POS	NEG	NEG	POS	NEG	POS	NEG	NEG	POS	NEG	NEG	NEG	NEG	POS	NEG	POS	NEG	POS	NEG	POS	POS	NEG
05V09326	AMB	AMB	POS	POS	NEG	POS	POS	NEG	NEG	POS	AMB	NEG	POS	NEG	POS	NEG	NEG	POS	NEG	NEG	NEG	NEG	POS	NEG	POS	NEG	POS	NEG	POS	POS	NEG
05V10265	AMB	AMB	POS	POS	NEG	POS	POS	NEG	NEG	POS	NEG	NEG	POS	NEG	POS	NEG	NEG	POS	NEG	NEG	NEG	NEG	POS	NEG	POS	NEG	AMB	NEG	POS	POS	NEG
05V13052	NEG	NEG	POS	POS	NEG	POS	POS	NEG	NEG	POS	NEG	NEG	AMB	NEG	POS	NEG	NEG	POS	NEG	NEG	NEG	NEG	POS	NEG	POS	NEG	POS	NEG	NEG	POS	NEG
05V13470	AMB	AMB	POS	POS	NEG	POS	POS	NEG	NEG	POS	NEG	NEG	POS	NEG	POS	NEG	NEG	POS	NEG	NEG	NEG	NEG	POS	NEG	POS	NEG	POS	NEG	POS	POS	NEG
05V13891	AMB	NEG	POS	POS	NEG	POS	POS	NEG	NEG	POS	NEG	NEG	POS	NEG	POS	NEG	NEG	POS	NEG	NEG	NEG	NEG	POS	NEG	POS	NEG	POS	NEG	POS	POS	NEG
05V15616																															
05V16845	AMB	AMB	POS	POS	NEG	POS	POS	NEG	NEG	POS	NEG	NEG	POS	NEG	POS	NEG	NEG	POS	NEG	NEG	NEG	NEG	POS	NEG	POS	NEG	POS	NEG	POS	POS	NEG
05V17541	AMB	NEG	POS	POS	NEG	POS	POS	NEG	NEG	POS	NEG	NEG	POS	NEG	POS	NEG	NEG	POS	NEG	NEG	NEG	NEG	POS	NEG	POS	NEG	POS	NEG	POS	POS	NEG
05V19105																															
05V22148	AMB	AMB	POS	POS	NEG	POS	POS	NEG	NEG	POS	NEG	NEG	POS	NEG	POS	NEG	NEG	POS	NEG	NEG	NEG	NEG	POS	NEG	POS	NEG	POS	NEG	POS	POS	NEG
05V33740	AMB	NEG	POS	POS	NEG	POS	POS	NEG	NEG	POS	NEG	NEG	POS	NEG	POS	NEG	NEG	POS	NEG	NEG	NEG	NEG	POS	NEG	POS	NEG	AMB	NEG	POS	POS	NEG
05V38425	AMB	AMB	POS	POS	NEG	POS	POS	NEG	NEG	POS	NEG	NEG	POS	NEG	POS	NEG	NEG	POS	NEG	NEG	NEG	NEG	POS	NEG	POS	NEG	AMB	NEG	POS	POS	NEG
05V39987	AMB	AMB	POS	POS	NEG	POS	POS	NEG	NEG	POS	NEG	NEG	POS	NEG	POS	NEG	NEG	POS	NEG	NEG	NEG	NEG	POS	NEG	POS	NEG	POS	NEG	POS	POS	NEG
05F00089	POS	POS	POS	POS	NEG	POS	POS	NEG	NEG	POS	NEG	NEG	POS	NEG	POS	NEG	NEG	POS	NEG	NEG	NEG	NEG	POS	NEG	POS	NEG	NEG	NEG	POS	POS	NEG
05V01416	AMB	NEG	POS	POS	NEG	POS	POS	NEG	NEG	POS	NEG	NEG	POS	NEG	POS	NEG	NEG	POS	NEG	NEG	NEG	NEG	POS	NEG	POS	NEG	NEG	NEG	POS	POS	NEG
05V16386	POS	POS	NEG	POS	NEG	POS	POS	NEG	NEG	POS	NEG	NEG	POS	POS	NEG	NEG	NEG	POS	NEG	NEG	POS	NEG	NEG	NEG	POS	POS	NEG	POS	NEG	NEG	NEG
05V04920	POS	POS	AMB	POS	NEG	POS	POS	NEG	NEG	NEG	NEG	NEG	POS	POS	NEG	NEG	NEG	POS	NEG	NEG	POS	NEG	NEG	NEG	POS	POS	AMB	POS	NEG	NEG	NEG
05V21159	POS	POS	AMB	POS	NEG	POS	POS	NEG	NEG	POS	NEG	NEG	POS	POS	NEG	NEG	NEG	POS	NEG	NEG	POS	NEG	NEG	NEG	POS	POS	AMB	POS	NEG	NEG	NEG
05B00867	POS	AMB	POS	POS	NEG	POS	POS	NEG	NEG	POS	NEG	NEG	POS	NEG	NEG	NEG	NEG	POS	NEG	NEG	NEG	NEG	POS	NEG	POS	NEG	NEG	NEG	POS	POS	NEG
05V03174	POS	AMB	POS	POS	NEG	POS	POS	NEG	NEG	POS	NEG	NEG	POS	NEG	NEG	NEG	NEG	POS	NEG	NEG	NEG	NEG	POS	NEG	POS	NEG	POS	NEG	POS	POS	POS
05V07331	POS	AMB	POS	POS	AMB	POS	POS	NEG	NEG	POS	NEG	NEG	AMB	NEG	NEG	NEG	NEG	POS	NEG	NEG	NEG	NEG	POS	NEG	POS	NEG	NEG	NEG	POS	POS	AMB
05V07371	POS	AMB	POS	POS	NEG	POS	POS	NEG	NEG	POS	NEG	NEG	NEG	NEG	NEG	NEG	NEG	AMB	NEG	NEG	NEG	NEG	POS	NEG	POS	NEG	NEG	NEG	POS	POS	AMB
05V08145	POS	AMB	POS	POS	NEG	POS	POS	NEG	NEG	POS	NEG	NEG	NEG	NEG	NEG	NEG	NEG	NEG	NEG	NEG	NEG	NEG	POS	NEG	POS	NEG	NEG	NEG	POS	POS	NEG
05V08595	POS	AMB	POS	POS	NEG	POS	POS	NEG	NEG	POS	NEG	NEG	AMB	NEG	NEG	NEG	NEG	POS	NEG	NEG	NEG	NEG	POS	NEG	POS	NEG	NEG	NEG	POS	POS	NEG
05V12803	POS	AMB	POS	POS	AMB	POS	POS	NEG	NEG	POS	NEG	NEG	AMB	NEG	NEG	NEG	NEG	POS	NEG	NEG	NEG	NEG	POS	NEG	POS	NEG	NEG	NEG	POS	POS	NEG
05V14596	POS	NEG	POS	POS	NEG	POS	POS	NEG	NEG	POS	NEG	NEG	NEG	NEG	NEG	NEG	NEG	POS	NEG	NEG	NEG	NEG	POS	NEG	POS	NEG	NEG	NEG	POS	AMB	NEG
05V16691																															
05V18550	POS	POS	AMB	POS	AMB	POS	POS	NEG	NEG	POS	AMB	NEG	POS	NEG	NEG	NEG	NEG	POS	AMB	NEG	NEG	AMB	POS	NEG	POS	NEG	AMB	NEG	POS	POS	POS
05V19034	POS	AMB	POS	POS	NEG	POS	POS	NEG	NEG	POS	AMB	NEG	AMB	NEG	NEG	NEG	NEG	POS	AMB	NEG	NEG	NEG	POS	NEG	POS	NEG	AMB	NEG	POS	POS	AMB
05V19333																															
05V19701	POS	AMB	POS	POS	NEG	POS	POS	NEG	NEG	POS	NEG	NEG	NEG	NEG	NEG	NEG	NEG	AMB	NEG	NEG	NEG	NEG	POS	NEG	POS	NEG	NEG	NEG	POS	POS	AMB
05V20072	POS	AMB	POS	POS	NEG	POS	POS	NEG	NEG	POS	NEG	NEG	NEG	NEG	NEG	NEG	NEG	POS	NEG	NEG	NEG	NEG	POS	NEG	POS	NEG	NEG	NEG	POS	POS	AMB
05V24930	POS	AMB	POS	POS	NEG	POS	POS	NEG	NEG	POS	NEG	NEG	AMB	NEG	NEG	NEG	NEG	POS	NEG	NEG	NEG	NEG	POS	NEG	POS	NEG	AMB	NEG	POS	POS	POS
05V31461	POS	AMB	POS	POS	AMB	POS	POS	NEG	NEG	POS	NEG	NEG	AMB	NEG	NEG	NEG	NEG	POS	AMB	NEG	NEG	NEG	POS	NEG	POS	NEG	NEG	NEG	POS	POS	NEG
05B05671	POS	NEG	POS	POS	POS	POS	POS	NEG	NEG	NEG	NEG	POS	NEG	NEG	NEG	NEG	NEG	POS	NEG	NEG	NEG	NEG	NEG	NEG	POS	NEG	NEG	NEG	POS	NEG	NEG
05V01452	POS	NEG	POS	POS	POS	AMB	POS	NEG	NEG	NEG	NEG	POS	NEG	NEG	NEG	NEG	NEG	POS	NEG	NEG	NEG	NEG	POS	NEG	POS	NEG	AMB	NEG	POS	NEG	NEG
05V01617	POS	AMB	POS	POS	POS	AMB	POS	NEG	NEG	NEG	NEG	POS	NEG	NEG	NEG	NEG	NEG	POS	NEG	NEG	NEG	NEG	POS	NEG	POS	NEG	AMB	NEG	POS	NEG	NEG
05V16986	POS	NEG	NEG	POS	POS	NEG	POS	NEG	NEG	NEG	NEG	POS	NEG	NEG	NEG	NEG	NEG	NEG	NEG	NEG	NEG	NEG	POS	NEG	NEG	NEG	NEG	NEG	NEG	NEG	NEG
05V18357	POS	NEG	NEG	POS	POS	NEG	POS	NEG	NEG	NEG	NEG	POS	NEG	NEG	NEG	NEG	NEG	NEG	NEG	NEG	NEG	NEG	NEG	NEG	AMB	NEG	NEG	NEG	AMB	NEG	NEG
05V23111	POS	AMB	POS	POS	POS	AMB	POS	NEG	NEG	NEG	NEG	POS	NEG	NEG	NEG	NEG	NEG	POS	AMB	NEG	NEG	NEG	POS	NEG	POS	NEG	POS	NEG	POS	NEG	NEG
05V23414																															
05V23858	POS	NEG	POS	POS	POS	AMB	POS	NEG	NEG	NEG	NEG	POS	NEG	NEG	NEG	NEG	NEG	AMB	NEG	NEG	NEG	NEG	NEG	NEG	POS	NEG	NEG	NEG	POS	NEG	NEG
05V26241	POS	AMB	POS	POS	POS	AMB	POS	NEG	NEG	NEG	NEG	POS	NEG	NEG	NEG	NEG	NEG	AMB	NEG	NEG	NEG	NEG	POS	NEG	POS	NEG	NEG	NEG	POS	NEG	NEG
05V28399	POS	AMB	POS	POS	POS	AMB	POS	NEG	NEG	NEG	NEG	POS	NEG	NEG	NEG	NEG	NEG	POS	NEG	NEG	NEG	NEG	POS	NEG	POS	NEG	AMB	NEG	POS	NEG	NEG
05V32647																															
05V37969	POS	AMB	POS	POS	POS	NEG	POS	NEG	NEG	NEG	NEG	POS	NEG	NEG	NEG	NEG	NEG	POS	NEG	NEG	NEG	NEG	NEG	NEG	POS	NEG	NEG	NEG	POS	NEG	NEG
05U10018	POS	NEG	NEG	POS	AMB	NEG	POS	NEG	NEG	NEG	NEG	NEG	NEG	NEG	NEG	NEG	NEG	NEG	POS	NEG	NEG	NEG	NEG	NEG	POS	POS	NEG	POS	NEG	NEG	NEG
**2006**																															
06V36353	NEG	POS	NEG	POS	NEG	POS	POS	NEG	NEG	NEG	NEG	NEG	POS	POS	NEG	NEG	NEG	POS	NEG	NEG	POS	NEG	NEG	NEG	POS	NEG	NEG	NEG	POS	POS	NEG
06V40202																															
06V41266	AMB	AMB	POS	POS	NEG	POS	POS	NEG	NEG	NEG	NEG	NEG	POS	NEG	POS	NEG	NEG	POS	NEG	NEG	NEG	NEG	NEG	NEG	POS	NEG	AMB	NEG	POS	POS	NEG
06B09097																															
06F00244	AMB	AMB	POS	POS	NEG	POS	POS	NEG	NEG	POS	NEG	NEG	POS	NEG	POS	NEG	NEG	POS	NEG	NEG	NEG	NEG	POS	NEG	POS	NEG	POS	NEG	POS	POS	NEG
06V03913	AMB	AMB	POS	POS	NEG	POS	POS	NEG	NEG	POS	NEG	NEG	POS	NEG	POS	NEG	NEG	POS	NEG	NEG	NEG	NEG	POS	NEG	POS	NEG	POS	NEG	POS	POS	NEG
06V04337	AMB	POS	AMB	POS	NEG	POS	POS	NEG	NEG	POS	NEG	NEG	POS	NEG	POS	NEG	NEG	POS	NEG	NEG	NEG	AMB	POS	NEG	POS	NEG	POS	NEG	POS	POS	NEG
06V06785	AMB	AMB	POS	POS	#ZAHL!	#ZAHL!	POS	NEG	NEG	POS	NEG	NEG	POS	NEG	POS	NEG	NEG	POS	NEG	NEG	NEG	NEG	POS	NEG	POS	NEG	POS	NEG	POS	POS	NEG
06V12258	AMB	POS	AMB	POS	AMB	POS	POS	NEG	NEG	POS	AMB	NEG	POS	NEG	POS	NEG	NEG	POS	AMB	NEG	NEG	AMB	POS	NEG	POS	NEG	POS	NEG	POS	POS	NEG
06V20553	AMB	AMB	POS	POS	NEG	POS	POS	NEG	NEG	POS	NEG	NEG	POS	NEG	POS	NEG	NEG	POS	NEG	NEG	NEG	NEG	POS	NEG	POS	NEG	POS	NEG	POS	POS	NEG
06V22564																															
06V23750	AMB	AMB	POS	POS	NEG	POS	POS	NEG	NEG	POS	NEG	NEG	POS	NEG	POS	NEG	NEG	POS	NEG	NEG	NEG	NEG	POS	NEG	POS	NEG	POS	NEG	POS	POS	NEG
06V27332	AMB	AMB	POS	POS	NEG	POS	POS	NEG	NEG	POS	NEG	NEG	POS	NEG	POS	NEG	NEG	POS	NEG	NEG	NEG	NEG	POS	NEG	POS	NEG	POS	NEG	POS	POS	NEG
06V29484	AMB	AMB	POS	POS	NEG	POS	POS	NEG	NEG	POS	NEG	NEG	POS	NEG	POS	NEG	NEG	POS	NEG	NEG	NEG	NEG	POS	NEG	POS	NEG	POS	NEG	POS	POS	NEG
06V31278																															
06V36043	AMB	AMB	POS	POS	NEG	POS	POS	NEG	NEG	POS	NEG	NEG	POS	NEG	POS	NEG	NEG	POS	NEG	NEG	NEG	NEG	POS	NEG	POS	NEG	AMB	NEG	POS	POS	NEG
06V36349	AMB	AMB	POS	POS	NEG	POS	POS	NEG	NEG	POS	NEG	NEG	POS	NEG	POS	NEG	NEG	POS	NEG	NEG	NEG	NEG	POS	NEG	POS	NEG	POS	NEG	POS	POS	NEG
06V39695	AMB	NEG	POS	POS	NEG	POS	POS	NEG	NEG	POS	NEG	NEG	POS	NEG	POS	NEG	NEG	POS	NEG	NEG	NEG	NEG	POS	NEG	POS	NEG	POS	NEG	POS	POS	NEG
06V40571																															
06V42188	AMB	POS	AMB	POS	NEG	POS	POS	NEG	NEG	POS	NEG	NEG	POS	NEG	POS	NEG	NEG	POS	NEG	NEG	NEG	NEG	POS	NEG	POS	NEG	POS	NEG	POS	POS	NEG
06V42146																															
06V28560	AMB	POS	AMB	POS	NEG	POS	POS	NEG	NEG	POS	NEG	NEG	POS	POS	NEG	NEG	NEG	POS	NEG	NEG	POS	NEG	NEG	NEG	POS	POS	AMB	POS	NEG	NEG	NEG
06F00837	POS	AMB	POS	POS	NEG	POS	POS	NEG	NEG	POS	NEG	NEG	AMB	NEG	NEG	NEG	NEG	POS	AMB	NEG	NEG	NEG	POS	NEG	POS	NEG	AMB	NEG	POS	POS	AMB
06F01638	POS	AMB	POS	POS	NEG	POS	POS	NEG	NEG	POS	NEG	NEG	AMB	NEG	NEG	NEG	NEG	POS	NEG	NEG	NEG	NEG	POS	NEG	POS	NEG	NEG	NEG	POS	POS	NEG
06F04643																															
06F05580	POS	AMB	POS	POS	NEG	POS	POS	NEG	NEG	POS	NEG	NEG	NEG	NEG	NEG	NEG	NEG	AMB	NEG	NEG	NEG	NEG	POS	NEG	POS	NEG	NEG	NEG	POS	POS	AMB
06U05469	POS	AMB	POS	POS	NEG	POS	POS	NEG	NEG	POS	NEG	NEG	NEG	NEG	NEG	NEG	NEG	POS	NEG	NEG	NEG	NEG	POS	NEG	POS	NEG	AMB	NEG	POS	POS	AMB
06U17314	POS	AMB	POS	POS	NEG	POS	POS	NEG	NEG	POS	NEG	NEG	NEG	NEG	NEG	NEG	NEG	POS	NEG	NEG	NEG	NEG	NEG	NEG	POS	NEG	NEG	NEG	POS	POS	AMB
06V08377	POS	AMB	POS	POS	NEG	POS	POS	NEG	NEG	POS	NEG	NEG	AMB	NEG	NEG	NEG	NEG	POS	NEG	NEG	NEG	NEG	POS	NEG	POS	NEG	AMB	NEG	POS	POS	POS
06V08474	POS	AMB	POS	POS	NEG	POS	POS	NEG	NEG	POS	NEG	NEG	AMB	NEG	NEG	NEG	NEG	POS	AMB	NEG	NEG	NEG	POS	NEG	POS	NEG	AMB	NEG	POS	POS	AMB
06V09374	POS	AMB	POS	POS	AMB	POS	POS	NEG	NEG	POS	NEG	NEG	POS	NEG	NEG	NEG	NEG	POS	AMB	NEG	NEG	AMB	POS	NEG	POS	NEG	AMB	NEG	POS	POS	POS
06V09605	POS	AMB	POS	POS	NEG	POS	POS	NEG	NEG	POS	NEG	NEG	NEG	NEG	NEG	NEG	NEG	POS	NEG	NEG	NEG	NEG	POS	NEG	POS	NEG	NEG	NEG	POS	POS	AMB
06V18687	POS	AMB	POS	POS	NEG	POS	POS	NEG	NEG	POS	NEG	NEG	NEG	NEG	NEG	NEG	NEG	AMB	NEG	NEG	NEG	NEG	POS	NEG	POS	NEG	NEG	NEG	POS	POS	NEG
06V21697	POS	POS	AMB	POS	AMB	POS	POS	NEG	NEG	POS	AMB	NEG	AMB	NEG	NEG	NEG	NEG	POS	AMB	NEG	NEG	AMB	POS	NEG	POS	NEG	AMB	NEG	POS	POS	POS
06V23749	POS	AMB	POS	POS	NEG	POS	POS	NEG	NEG	POS	NEG	NEG	NEG	NEG	NEG	NEG	NEG	POS	NEG	NEG	NEG	NEG	POS	NEG	POS	NEG	NEG	NEG	POS	POS	AMB
06V29915	POS	POS	AMB	POS	AMB	POS	POS	NEG	NEG	POS	AMB	NEG	POS	NEG	NEG	NEG	NEG	POS	AMB	NEG	NEG	AMB	POS	NEG	POS	NEG	NEG	NEG	POS	POS	AMB
06V31778	POS	AMB	POS	POS	NEG	POS	POS	NEG	NEG	POS	NEG	NEG	AMB	NEG	NEG	NEG	NEG	POS	NEG	NEG	NEG	NEG	POS	NEG	POS	NEG	NEG	NEG	POS	POS	NEG
06V39089	POS	AMB	POS	POS	NEG	POS	POS	NEG	NEG	POS	NEG	NEG	AMB	NEG	NEG	NEG	NEG	POS	NEG	NEG	NEG	NEG	POS	NEG	POS	NEG	AMB	NEG	POS	POS	POS
06V41512																															
06V42186	POS	AMB	POS	POS	NEG	POS	POS	NEG	NEG	POS	NEG	NEG	NEG	NEG	NEG	NEG	NEG	POS	NEG	NEG	NEG	NEG	POS	NEG	POS	NEG	NEG	NEG	POS	POS	AMB
06V42236	POS	AMB	POS	POS	NEG	POS	POS	NEG	NEG	POS	NEG	NEG	AMB	NEG	NEG	NEG	NEG	POS	NEG	NEG	NEG	NEG	POS	NEG	POS	NEG	NEG	NEG	POS	POS	NEG
06V43697	POS	AMB	POS	POS	NEG	POS	POS	NEG	NEG	POS	NEG	NEG	AMB	NEG	NEG	NEG	NEG	POS	AMB	NEG	NEG	NEG	POS	NEG	POS	NEG	AMB	NEG	POS	POS	POS
06V44153	POS	AMB	POS	POS	NEG	POS	POS	NEG	NEG	POS	NEG	NEG	NEG	NEG	NEG	NEG	NEG	AMB	NEG	NEG	NEG	NEG	POS	NEG	POS	NEG	NEG	NEG	POS	POS	NEG
06V45679	POS	AMB	POS	POS	NEG	POS	POS	NEG	NEG	POS	NEG	NEG	NEG	NEG	NEG	NEG	NEG	AMB	NEG	NEG	NEG	NEG	POS	NEG	POS	NEG	NEG	NEG	POS	POS	NEG
06V45831	POS	AMB	POS	POS	NEG	POS	POS	NEG	NEG	POS	NEG	NEG	NEG	NEG	NEG	NEG	NEG	AMB	NEG	NEG	NEG	NEG	POS	NEG	POS	NEG	NEG	NEG	POS	POS	NEG
06V46314	POS	AMB	POS	POS	NEG	POS	POS	NEG	NEG	POS	NEG	NEG	NEG	NEG	NEG	NEG	NEG	POS	NEG	NEG	NEG	NEG	NEG	NEG	POS	NEG	NEG	NEG	POS	POS	AMB
06V34729	POS	AMB	POS	POS	POS	NEG	POS	NEG	NEG	NEG	NEG	POS	AMB	NEG	NEG	POS	POS	POS	POS	NEG	NEG	NEG	POS	POS	POS	POS	NEG	POS	POS	POS	POS
06B09900	POS	AMB	POS	POS	POS	AMB	POS	NEG	NEG	NEG	NEG	POS	NEG	NEG	NEG	NEG	NEG	POS	NEG	NEG	NEG	NEG	NEG	NEG	POS	NEG	NEG	NEG	POS	NEG	NEG
06V00922	POS	NEG	NEG	POS	POS	AMB	POS	NEG	NEG	NEG	NEG	POS	NEG	NEG	NEG	NEG	NEG	AMB	NEG	NEG	NEG	NEG	NEG	NEG	POS	NEG	NEG	NEG	POS	NEG	NEG
06V09551	POS	AMB	POS	POS	POS	AMB	POS	NEG	NEG	NEG	NEG	POS	NEG	NEG	NEG	NEG	NEG	POS	NEG	NEG	NEG	NEG	POS	NEG	POS	NEG	AMB	NEG	POS	NEG	NEG
06V10719	POS	NEG	POS	POS	POS	AMB	POS	NEG	NEG	NEG	NEG	POS	NEG	NEG	NEG	NEG	NEG	POS	NEG	NEG	NEG	NEG	POS	NEG	POS	NEG	NEG	NEG	POS	NEG	NEG
06V11090	POS	NEG	NEG	POS	POS	AMB	POS	NEG	NEG	NEG	NEG	POS	NEG	NEG	NEG	NEG	NEG	AMB	NEG	NEG	NEG	NEG	NEG	NEG	POS	NEG	NEG	NEG	AMB	NEG	NEG
06V14383	POS	NEG	POS	POS	POS	NEG	POS	NEG	NEG	NEG	NEG	POS	NEG	NEG	NEG	NEG	NEG	AMB	NEG	NEG	NEG	NEG	NEG	NEG	POS	NEG	AMB	NEG	POS	NEG	NEG
06V21475	POS	NEG	NEG	POS	POS	NEG	POS	NEG	NEG	NEG	NEG	POS	NEG	NEG	NEG	NEG	NEG	NEG	NEG	NEG	NEG	NEG	NEG	NEG	POS	NEG	NEG	NEG	AMB	NEG	NEG
06V24231	POS	NEG	POS	POS	POS	NEG	POS	NEG	NEG	NEG	NEG	POS	NEG	NEG	NEG	NEG	NEG	AMB	NEG	NEG	NEG	NEG	NEG	NEG	POS	NEG	NEG	NEG	AMB	NEG	NEG
06V32992	POS	NEG	NEG	POS	POS	AMB	POS	NEG	NEG	NEG	NEG	POS	NEG	NEG	NEG	NEG	NEG	NEG	NEG	NEG	NEG	NEG	NEG	NEG	POS	NEG	NEG	NEG	AMB	NEG	NEG
06V38753	POS	NEG	NEG	POS	POS	AMB	POS	NEG	NEG	NEG	NEG	POS	NEG	NEG	NEG	NEG	NEG	NEG	NEG	NEG	NEG	NEG	NEG	NEG	POS	NEG	NEG	NEG	POS	NEG	NEG
06V39848																															
06V42259	POS	AMB	POS	POS	POS	AMB	POS	NEG	NEG	NEG	AMB	POS	NEG	NEG	NEG	NEG	NEG	POS	AMB	NEG	NEG	AMB	NEG	NEG	POS	NEG	AMB	NEG	POS	NEG	NEG
06V33960																															

ISOLATE:	STRAIN	WARD	AGE	GENDER	SPECIES MARKER							REGULATORY GENES								METHICILLIN RESISTANCE AND SCCmec TYPING																									
					Ribosomal sequence from S. aureus	glyceraldehyde 3-phosphate dehydro-genase, locus 1	katalase A	coagulase	thermo-stable extracellular nuclease	Protein A	IgG-binding protein	staphylo-coccal accessory regulator A	histidine protein kinase, sae locus	sensor protein	accessory gene regulator allele I	accessory gene regulator allele II	accessory gene regulator allele III	accessory gene regulator allele IV	haemolysin delta	alternate penicillin binding protein 2, defining MRSA	truncated signal transducer protein MecR1	glycerophosphoryl diester phosphodi-esterase, associated with mecA	cassette chromosome recombinase genes A/B-1		plasmin-sensitive surface protein	hypothetical protein from SCCmec elements	cassette chromosome recombinase genes A/B-2		potassium-trans locating ATPase A, chain 2	potassium-transporting ATPase B, chain 1	potassium-trans-locating ATPase C, chain 2	sensor kinase protein	KDP operon transcrip-tional regulatory protein	methicillin-resistance regulatory protein	signal transducer protein MecR1	homolog of xylose repressor, associated with SCCmec elements	cassette chromosome recombinase genes A/B-3		mercury resistance operon		cassette chromosome recombinase genes "ccrAA" (hypothetical) and ccrC			cassette chromosome recombinase genes A/B-4	
					rrnD1 (S. aureus)	gapA	katA	coA	nuc1	spa	sbi	sarA	saeS	vraS	agrI	agrII	agrIII	agrIV	hld	mecA	delta_mecR	ugpQ	ccrA-1	ccrB-1	plsSCC (COL)	Q9XB68-dcs	ccrA-2	ccrB-2	kdpA-SCC	kdpB-SCC	kdpC-SCC	kdpD-SCC	kdpE-SCC	mecI	mecR	xylR	ccrA-3	ccrB-3	merA	merB	ccrAA (MRSAZH47) _probe 1	ccrAA (MRSAZH47) _probe 2	ccrC (85-2082)	ccrA-4	ccrB-4
**2007**																																													
07V01446	CC1-MRSA-IV, WA MRSA-1/57	MED-OPD	70	f	POS	POS	POS	POS	POS	POS	POS	POS	POS	POS	NEG	NEG	POS	NEG	POS	POS	POS	POS	NEG	NEG	NEG	NEG	POS	POS	NEG	NEG	NEG	NEG	NEG	NEG	NEG	NEG	NEG	NEG	NEG	NEG	NEG	NEG	NEG	NEG	NEG
07F01032	CC5/ST228-MRSA-I, South German EMRSA	MED-WARD	79	m	POS	POS	POS	POS		POS	POS				NEG	POS	NEG	NEG	POS	POS																									
07V01588	CC5/ST228-MRSA-I, South German EMRSA	SUR-WARD	60	m	POS	POS	POS	AMB		POS	POS				NEG	POS	NEG	NEG	POS	POS																									
07V01876	CC5/ST228-MRSA-I, South German EMRSA	SUR-ICU	43	m	POS	POS	POS	AMB		POS	POS				NEG	POS	NEG	NEG	POS	POS																									
07V03020	CC5/ST228-MRSA-I, South German EMRSA	SUR-ICU	79	m	POS	POS	POS	POS		POS	POS	POS			NEG	POS	NEG	NEG	POS	POS																									
07V03066	CC5/ST228-MRSA-I, South German EMRSA	MED-WARD	60	m	POS	POS	POS	AMB		POS	POS				NEG	POS	NEG	NEG	POS	POS																									
07V08865	CC5/ST228-MRSA-I, South German EMRSA	SUR-WARD	69	m	POS	POS	POS	POS	POS	POS	POS	AMB	POS	POS	NEG	POS	NEG	NEG	POS	POS	POS	POS	POS	POS	POS	NEG	NEG	NEG	NEG	NEG	NEG	NEG	NEG	NEG	NEG	NEG	NEG	NEG	NEG	NEG	NEG	NEG	NEG	NEG	NEG
07V35185	CC5/ST228-MRSA-I, South German EMRSA	SUR-WARD	67	f	POS	POS	POS	POS		POS	POS	POS			NEG	POS	NEG	NEG	POS	POS																									
07V42548	CC5/ST228-MRSA-I, South German EMRSA	SUR-WARD	37	m	POS	POS	POS	AMB	POS	POS	POS	POS	POS	POS	NEG	POS	NEG	NEG	POS	POS	POS	POS	POS	POS	POS	POS	NEG	NEG	NEG	NEG	NEG	NEG	NEG	NEG	NEG	NEG	NEG	AMB	POS	POS	NEG	NEG	NEG	NEG	NEG
07V47082	CC5/ST228-MRSA-I, South German EMRSA	MED-WARD	73	m	POS	POS	POS	POS	POS	POS	POS	POS	POS	POS	NEG	POS	NEG	NEG	POS	POS	POS	POS	POS	POS	POS	POS	NEG	NEG	NEG	NEG	NEG	NEG	NEG	NEG	NEG	NEG	NEG	AMB	POS	POS	NEG	NEG	NEG	NEG	NEG
07V48389	CC5/ST228-MRSA-I, South German EMRSA	MED-ICU	84	f	POS	POS	POS	POS		POS	POS	POS			NEG	POS	NEG	NEG	POS	POS																									
07V49263	CC5/ST228-MRSA-I, South German EMRSA	MED-ICU	76	m	POS	POS	POS	POS	POS	POS	POS	POS	POS	POS	NEG	POS	NEG	NEG	POS	POS	POS	POS	POS	POS	POS	POS	NEG	NEG	NEG	NEG	NEG	NEG	NEG	NEG	NEG	NEG	NEG	NEG	POS	POS	NEG	NEG	NEG	NEG	NEG
07V50349	CC5/ST228-MRSA-I, South German EMRSA	MED-OPD	70	m	POS	POS	POS	POS		POS	POS	POS			NEG	POS	NEG	NEG	POS	POS																									
07B05103	CC5-MRSA-II, Rhine-Hesse EMRSA/UK-EMRSA-3	MED-icu	45	f	POS	POS	POS	POS		POS	POS	POS			NEG	POS	NEG	NEG	POS	POS																									
07F00721	CC5-MRSA-II, Rhine-Hesse EMRSA/UK-EMRSA-3	SUR-ICU	73	m	POS	POS	POS	POS		POS	POS	POS			NEG	POS	NEG	NEG	POS	POS																									
07V00817	CC5-MRSA-II, Rhine-Hesse EMRSA/UK-EMRSA-3	MED-ICU	77	f	POS	POS	POS	POS	POS	POS	POS	POS	POS	POS	NEG	POS	NEG	NEG	POS	POS	POS	POS	NEG	NEG	NEG	NEG	POS	POS	POS	POS	POS	POS	AMB	POS	POS	POS	NEG	NEG	NEG	NEG	NEG	NEG	NEG	NEG	NEG
07V01111	CC5-MRSA-II, Rhine-Hesse EMRSA/UK-EMRSA-3	MED-OPD	96	f	POS	POS	POS	POS		POS	POS	POS			NEG	POS	NEG	NEG	POS	POS																									
07V01885	CC5-MRSA-II, Rhine-Hesse EMRSA/UK-EMRSA-3	SUR-ICU	69	m	POS	POS	POS	POS		POS	POS	POS			NEG	POS	NEG	NEG	POS	POS																									
07V04946	CC5-MRSA-II, Rhine-Hesse EMRSA/UK-EMRSA-3	MED-ICU	66	m	POS	POS	POS	POS		POS	POS				NEG	POS	NEG	NEG	POS	POS																									
07V05167	CC5-MRSA-II, Rhine-Hesse EMRSA/UK-EMRSA-3	SUR-ICU	68	f	POS	POS	POS	POS		POS	POS	POS			NEG	POS	NEG	NEG	POS	POS																									
07V05703	CC5-MRSA-II, Rhine-Hesse EMRSA/UK-EMRSA-3	SUR-WARD	60	f	POS	POS	AMB	NEG		POS	POS				NEG	POS	NEG	NEG	AMB	POS																									
07V08418	CC5-MRSA-II, Rhine-Hesse EMRSA/UK-EMRSA-3	SUR-WARD	45	m	POS	POS	POS	POS		POS	POS	POS			NEG	POS	NEG	NEG	POS	POS																									
07V09138	CC5-MRSA-II, Rhine-Hesse EMRSA/UK-EMRSA-3	MED-ICU	72	f	POS	POS	POS	POS		POS	POS	POS			NEG	POS	NEG	NEG	POS	POS																									
07V11143	CC5-MRSA-II, Rhine-Hesse EMRSA/UK-EMRSA-3	SUR-WARD	52	f	POS	POS	AMB	AMB		POS	POS				NEG	POS	NEG	NEG	AMB	POS																									
07V14047	CC5-MRSA-II, Rhine-Hesse EMRSA/UK-EMRSA-3	SUR-ICU	70	m	POS	POS	POS	AMB		POS	POS				NEG	POS	NEG	NEG	POS	POS																									
07V22331	CC5-MRSA-II, Rhine-Hesse EMRSA/UK-EMRSA-3	MED-WARD	7	f	POS	POS	POS	POS		POS	POS	POS			NEG	POS	NEG	NEG	POS	POS																									
07V23848	CC5-MRSA-II, Rhine-Hesse EMRSA/UK-EMRSA-3	MED-ICU	77	m	POS	POS	POS	POS		POS	POS	POS			NEG	POS	NEG	NEG	POS	POS																									
07V25349	CC5-MRSA-II, Rhine-Hesse EMRSA/UK-EMRSA-3	MED-icu	65	f	POS	POS	POS	POS		POS	POS	POS			NEG	POS	NEG	NEG	POS	POS																									
07V29952	CC5-MRSA-II, Rhine-Hesse EMRSA/UK-EMRSA-3	SUR-WARD	57	m	POS	POS	POS	AMB		POS	POS				NEG	POS	NEG	NEG	POS	POS																									
07V32172	CC5-MRSA-II, Rhine-Hesse EMRSA/UK-EMRSA-3	SUR-ICU	24	m	POS	POS	POS	POS		POS	POS				NEG	POS	NEG	NEG	POS	POS																									
07V34596	CC5-MRSA-II, Rhine-Hesse EMRSA/UK-EMRSA-3	MED-OPD	69	f	POS	POS	POS	POS		POS	POS				NEG	POS	NEG	NEG	POS	POS																									
07V34637	CC5-MRSA-II, Rhine-Hesse EMRSA/UK-EMRSA-3	SUR-WARD	82	f	POS	POS	POS	POS		POS	POS	POS			NEG	POS	NEG	NEG	POS	POS																									
07V41577	CC5-MRSA-II, Rhine-Hesse EMRSA/UK-EMRSA-3	SUR-WARD	92	f	POS	POS	POS	POS		POS	POS	POS			NEG	POS	NEG	NEG	POS	POS																									
07V43890	CC5-MRSA-II, Rhine-Hesse EMRSA/UK-EMRSA-3	SUR-ICU	69	m	POS	POS	POS	POS		POS	POS	POS			NEG	POS	NEG	NEG	POS	POS																									
07V44597	CC5-MRSA-II, Rhine-Hesse EMRSA/UK-EMRSA-3	MED-OPD	60	m	POS	POS	POS	POS		POS	POS	POS			NEG	POS	NEG	NEG	POS	POS																									
07V48218	CC5-MRSA-II, Rhine-Hesse EMRSA/UK-EMRSA-3	MED-WARD	63	m	POS	POS	POS	POS	POS	POS	POS	POS	POS	POS	NEG	POS	NEG	NEG	POS	POS	POS	POS	NEG	NEG	NEG	AMB	POS	POS	POS	POS	POS	POS	POS	POS	POS	POS	NEG	NEG	NEG	NEG	NEG	NEG	NEG	NEG	NEG
07V48369	CC5-MRSA-II, Rhine-Hesse EMRSA/UK-EMRSA-3	SUR-WARD	72	m	POS	POS	POS	POS		POS	POS	POS			NEG	POS	NEG	NEG	POS	POS																									
07V50153	CC5-MRSA-II, Rhine-Hesse EMRSA/UK-EMRSA-3	MED-icu	33	m	POS	POS	POS	POS		POS	POS	POS			NEG	POS	NEG	NEG	POS	POS																									
07V31319	CC5-MRSA-IV, Paediatric clone	MED-WARD	30	f	POS	POS	POS	POS	POS	POS	POS	POS	POS	POS	NEG	POS	NEG	NEG	POS	POS	POS	POS	NEG	NEG	NEG	POS	POS	POS	NEG	NEG	NEG	NEG	NEG	NEG	NEG	NEG	NEG	NEG	POS	POS	NEG	NEG	NEG	NEG	NEG
07V31590	CC8-MRSA-IV, Lyon Clone/UK-EMRSA-2	MED-OPD	45	m	POS	POS	POS	POS		POS	POS	POS			POS	NEG	NEG	NEG	POS	POS																									
07V31964	CC8-MRSA-IV, Lyon Clone/UK-EMRSA-2	MED-OPD	70	f	POS	POS	POS	POS		POS	POS	POS			POS	NEG	NEG	NEG	POS	POS																									
07V33137	CC8-MRSA-IV, Lyon Clone/UK-EMRSA-2	MED-OPD	44	f	POS	POS	POS	POS	POS	POS	POS	POS	POS	POS	POS	NEG	NEG	NEG	POS	POS	POS	POS	NEG	NEG	NEG	POS	POS	POS	NEG	NEG	NEG	NEG	NEG	NEG	NEG	NEG	AMB	POS	NEG	NEG	NEG	NEG	NEG	NEG	AMB
07V45114	CC8-MRSA-IV, Lyon Clone/UK-EMRSA-2	MED-OPD	71	m	POS	POS	POS	POS	POS	POS	POS	POS	POS	POS	POS	NEG	NEG	NEG	POS	POS	POS	POS	NEG	NEG	NEG	AMB	POS	POS	NEG	NEG	NEG	NEG	NEG	NEG	NEG	NEG	NEG	NEG	NEG	NEG	NEG	NEG	NEG	NEG	NEG
07B06644	CC22-MRSA-IV, Barnim/UK-EMRSA-15	MED-ICU	0	m	POS	POS	NEG	AMB		POS	POS				POS	NEG	NEG	NEG	NEG	POS																									
07F03422	CC22-MRSA-IV, Barnim/UK-EMRSA-15	SUR-WARD	68	f	POS	POS	POS	POS		POS	POS	POS			POS	NEG	NEG	NEG	POS	POS																									
07F03905	CC22-MRSA-IV, Barnim/UK-EMRSA-15	MED-ICU	63	m	POS	POS	POS	POS		POS	POS				AMB	NEG	NEG	NEG	POS	POS																									
07F06025	CC22-MRSA-IV, Barnim/UK-EMRSA-15	MED-WARD	76	m	POS	POS	POS	POS		POS	POS	POS			POS	NEG	NEG	NEG	POS	POS																									
07F06053	CC22-MRSA-IV, Barnim/UK-EMRSA-15	MED-WARD	87	f	POS	POS	POS	POS		POS	POS				POS	NEG	NEG	NEG	POS	POS																									
07F06432	CC22-MRSA-IV, Barnim/UK-EMRSA-15	SUR-WARD	71	m	POS	POS	POS	POS		POS	POS	POS			POS	NEG	NEG	NEG	POS	POS																									
07U08245	CC22-MRSA-IV, Barnim/UK-EMRSA-15	MED-icu	71	m	POS	POS	POS	POS		POS	POS	POS			POS	NEG	NEG	NEG	POS	POS																									
07V01577	CC22-MRSA-IV, Barnim/UK-EMRSA-15	MED-WARD	25	m	POS	POS	POS	POS	POS	POS	POS	POS	POS	POS	POS	NEG	NEG	NEG	POS	POS	POS	POS	NEG	NEG	NEG	NEG	POS	POS	NEG	NEG	NEG	NEG	NEG	NEG	NEG	NEG	NEG	NEG	NEG	NEG	NEG	NEG	NEG	NEG	NEG
07V02929	CC22-MRSA-IV, Barnim/UK-EMRSA-15	SUR-WARD	72	m	POS	POS	POS	POS		POS	POS				AMB	NEG	NEG	NEG	POS	POS																									
07V03947	CC22-MRSA-IV, Barnim/UK-EMRSA-15	SUR-WARD	81	m	POS	POS	POS	POS		POS	POS				POS	NEG	NEG	NEG	POS	POS																									
07V07685	CC22-MRSA-IV, Barnim/UK-EMRSA-15	MED-OPD	53	m	POS	POS	POS	POS		POS	POS	POS			POS	NEG	NEG	AMB	POS	POS																									
07V09789	CC22-MRSA-IV, Barnim/UK-EMRSA-15	MED-ICU	67	m	POS	POS	POS	POS		POS	POS				POS	NEG	NEG	NEG	POS	POS																									
07V11100	CC22-MRSA-IV, Barnim/UK-EMRSA-15	MED-OPD	71	f	POS	POS	POS	POS	POS	POS	POS	POS	POS	POS	POS	NEG	NEG	NEG	POS	POS	POS	POS	NEG	NEG	NEG	POS	POS	POS	NEG	NEG	NEG	NEG	NEG	NEG	NEG	NEG	NEG	AMB	NEG	NEG	NEG	NEG	NEG	NEG	NEG
07V13886	CC22-MRSA-IV, Barnim/UK-EMRSA-15	MED-WARD	77	m	POS	POS	POS	POS		POS	POS				POS	NEG	NEG	NEG	POS	POS																									
07V13922	CC22-MRSA-IV, Barnim/UK-EMRSA-15	MED-OPD	49	m	POS	POS	POS	POS		POS	POS				POS	NEG	NEG	NEG	POS	POS																									
07V15787	CC22-MRSA-IV, Barnim/UK-EMRSA-15	MED-OPD	75	m	POS	POS	POS	POS		POS	POS	POS			POS	NEG	NEG	NEG	POS	POS																									
07V16040	CC22-MRSA-IV, Barnim/UK-EMRSA-15	MED-WARD	63	m	POS	POS	AMB	AMB		POS	POS				POS	NEG	NEG	NEG	AMB	POS																									
07V19106	CC22-MRSA-IV, Barnim/UK-EMRSA-15	MED-OPD	69	m	POS	POS	POS	POS		POS	POS				POS	NEG	NEG	NEG	POS	POS																									
07V19909	CC22-MRSA-IV, Barnim/UK-EMRSA-15	SUR-WARD	91	f	POS	POS	POS	AMB		POS	POS				AMB	NEG	NEG	NEG	POS	POS																									
07V20220	CC22-MRSA-IV, Barnim/UK-EMRSA-15	MED-WARD	65	f	POS	POS	AMB	POS		POS	POS				POS	NEG	NEG	NEG	AMB	POS																									
07V21255	CC22-MRSA-IV, Barnim/UK-EMRSA-15	SUR-OPD	49	f	POS	POS	POS	POS		POS	POS				AMB	NEG	NEG	NEG	POS	POS																									
07V22221	CC22-MRSA-IV, Barnim/UK-EMRSA-15	MED-WARD	75	m	POS	POS	AMB	AMB		POS	POS				POS	NEG	NEG	NEG	AMB	POS																									
07V23398	CC22-MRSA-IV, Barnim/UK-EMRSA-15	MED-icu	55	f	POS	POS	POS	POS		POS	POS				AMB	NEG	NEG	NEG	POS	POS																									
07V26255	CC22-MRSA-IV, Barnim/UK-EMRSA-15	SUR-WARD	68	f	POS	POS	POS	POS		POS	POS				POS	NEG	NEG	NEG	POS	POS																									
07V27224	CC22-MRSA-IV, Barnim/UK-EMRSA-15	SUR-WARD	80	f	POS	POS	POS	POS		POS	POS				POS	NEG	NEG	NEG	POS	POS																									
07V29579	CC22-MRSA-IV, Barnim/UK-EMRSA-15	SUR-WARD	76	m	POS	POS	POS	POS		POS	POS	POS			POS	NEG	NEG	NEG	POS	POS																									
07V30685	CC22-MRSA-IV, Barnim/UK-EMRSA-15	SUR-WARD	67	f	POS	POS	POS	POS		POS	POS				POS	NEG	NEG	NEG	POS	POS																									
07V30827	CC22-MRSA-IV, Barnim/UK-EMRSA-15	MED-WARD	74	m	POS	POS	POS	POS		POS	POS	POS			POS	NEG	NEG	NEG	POS	POS																									
07V31586	CC22-MRSA-IV, Barnim/UK-EMRSA-15	MED-WARD	71	m	POS	POS	POS	POS		POS	POS				AMB	NEG	NEG	NEG	POS	POS																									
07V31588	CC22-MRSA-IV, Barnim/UK-EMRSA-15	MED-WARD	75	m	POS	POS	POS	POS		POS	POS	POS			POS	NEG	NEG	NEG	POS	POS																									
07V32055	CC22-MRSA-IV, Barnim/UK-EMRSA-15	MED-OPD	72	m	POS	POS	POS	POS		POS	POS				POS	NEG	NEG	NEG	POS	POS																									
07V32148	CC22-MRSA-IV, Barnim/UK-EMRSA-15	MED-OPD	57	m	POS	POS	POS	POS		POS	POS	POS			POS	NEG	NEG	NEG	POS	POS																									
07V32390	CC22-MRSA-IV, Barnim/UK-EMRSA-15	MED-OPD	64	m	POS	POS	POS	POS		POS	POS	POS			POS	NEG	NEG	NEG	POS	POS																									
07V32454	CC22-MRSA-IV, Barnim/UK-EMRSA-15	SUR-WARD	63	m	POS	POS	POS	POS		POS	POS	POS			POS	NEG	NEG	NEG	POS	POS																									
07V32457	CC22-MRSA-IV, Barnim/UK-EMRSA-15	SUR-ICU	50	m	POS	POS	POS	POS	POS	POS	POS	POS	POS	POS	POS	NEG	NEG	NEG	POS	POS	POS	POS	NEG	NEG	NEG	POS	POS	POS	NEG	NEG	NEG	NEG	NEG	NEG	NEG	NEG	NEG	NEG	NEG	NEG	NEG	NEG	NEG	NEG	NEG
07V32467	CC22-MRSA-IV, Barnim/UK-EMRSA-15	MED-WARD	69	f	POS	POS	POS	POS		POS	POS	POS			POS	NEG	NEG	NEG	POS	POS																									
07V32765	CC22-MRSA-IV, Barnim/UK-EMRSA-15	MED-OPD	67	m	POS	POS	POS	POS		POS	POS	POS			POS	NEG	NEG	NEG	POS	POS																									
07V33857	CC22-MRSA-IV, Barnim/UK-EMRSA-15	SUR-WARD	94	f	POS	POS	POS	POS		POS	POS	POS			POS	NEG	NEG	NEG	POS	POS																									
07V34379	CC22-MRSA-IV, Barnim/UK-EMRSA-15	SUR-ICU	68	m	POS	POS	POS	POS		POS	POS				POS	NEG	NEG	NEG	POS	POS																									
07V35110	CC22-MRSA-IV, Barnim/UK-EMRSA-15	MED-OPD	75	f	POS	POS	POS	POS		POS	POS	POS			POS	NEG	NEG	NEG	POS	POS																									
07V35482	CC22-MRSA-IV, Barnim/UK-EMRSA-15	SUR-WARD	49	m	POS	POS	POS	POS		POS	POS				POS	NEG	NEG	NEG	POS	POS																									
07V35565	CC22-MRSA-IV, Barnim/UK-EMRSA-15	MED-OPD	78	m	POS	POS	POS	POS		POS	POS	POS			POS	NEG	NEG	NEG	POS	POS																									
07V37700	CC22-MRSA-IV, Barnim/UK-EMRSA-15	MED-OPD	69	m	POS	POS	POS	POS		POS	POS	POS			POS	NEG	NEG	NEG	POS	POS																									
07V41045	CC22-MRSA-IV, Barnim/UK-EMRSA-15	MED-OPD	64	m	POS	POS	POS	POS		POS	POS				POS	NEG	NEG	NEG	POS	POS																									
07V41051	CC22-MRSA-IV, Barnim/UK-EMRSA-15	MED-OPD	63	m	POS	POS	POS	POS		POS	POS				POS	NEG	NEG	NEG	POS	POS																									
07V42673	CC22-MRSA-IV, Barnim/UK-EMRSA-15	MED-OPD	55	m	POS	POS	POS	POS		POS	POS				POS	NEG	NEG	NEG	POS	POS																									
07V42842	CC22-MRSA-IV, Barnim/UK-EMRSA-15	MED-OPD	72	m	POS	POS	POS	POS		POS	POS	POS			POS	NEG	NEG	NEG	POS	POS																									
07V45346	CC22-MRSA-IV, Barnim/UK-EMRSA-15	SUR-WARD	76	f	POS	POS	POS	POS		POS	POS	POS			POS	NEG	NEG	NEG	POS	POS																									
07V46048	CC22-MRSA-IV, Barnim/UK-EMRSA-15	MED-OPD	67	m	POS	POS	POS	POS		POS	POS				POS	NEG	NEG	NEG	POS	POS																									
07V46684	CC22-MRSA-IV, Barnim/UK-EMRSA-15	MED-WARD	55	m	POS	POS	POS	POS		POS	POS	POS			POS	NEG	NEG	AMB	POS	POS																									
07V49690	CC22-MRSA-IV, Barnim/UK-EMRSA-15	SUR-WARD	58	m	POS	POS	POS	POS		POS	POS	POS			POS	NEG	NEG	AMB	POS	POS																									
07V49998	CC22-MRSA-IV, Barnim/UK-EMRSA-15	MED-ICU	71	f	POS	POS	POS	POS		POS	POS	P			POS	NEG	NEG	NEG	POS	POS																									
07V25999	CC30-MRSA-IV [PVL+], Southwest Pacific Clone	SUR-OPD	11	f	POS	POS	POS	POS	POS	POS	POS	POS	POS	POS	NEG	NEG	POS	NEG	POS	POS	POS	POS	NEG	NEG	NEG	POS	AMB	POS	NEG	NEG	NEG	NEG	NEG	NEG	NEG	NEG	NEG	AMB	NEG	NEG	NEG	NEG	NEG	NEG	NEG
07U07147	CC45-MRSA-IV, Berlin EMRSA	SUR-WARD	0	m	POS	POS	POS	AMB		POS	POS				AMB	NEG	NEG	NEG	POS	POS																									
07V03373	CC45-MRSA-IV, Berlin EMRSA	SUR-WARD	24	m	POS	POS	POS	POS	POS	POS	POS	POS	POS	POS	POS	NEG	NEG	NEG	POS	POS	POS	POS	NEG	NEG	NEG	NEG	POS	POS	NEG	NEG	NEG	NEG	NEG	NEG	NEG	NEG	NEG	NEG	NEG	NEG	NEG	NEG	NEG	NEG	NEG
07V06916	CC45-MRSA-IV, Berlin EMRSA	SUR-WARD	33	m	POS	POS	POS	POS		POS	POS	POS			POS	NEG	NEG	NEG	POS	POS																									
07V16850	CC45-MRSA-IV, Berlin EMRSA	SUR-WARD	53	m	POS	POS	POS	POS		POS	POS	POS			POS	NEG	NEG	NEG	POS	POS																									
07V17786	CC45-MRSA-IV, Berlin EMRSA	SUR-WARD	65	f	POS	POS	POS	POS		POS	POS	POS			POS	NEG	NEG	NEG	POS	POS																									
07V19855	CC45-MRSA-IV, Berlin EMRSA	SUR-WARD	46	m	POS	POS	POS	POS	POS	POS	POS	POS	POS	POS	POS	NEG	NEG	NEG	POS	POS	POS	POS	NEG	NEG	NEG	AMB	POS	POS	NEG	NEG	NEG	NEG	NEG	NEG	NEG	NEG	NEG	NEG	NEG	NEG	NEG	NEG	NEG	NEG	NEG
07V29398	CC45-MRSA-IV, Berlin EMRSA	MED-OPD	71	m	POS	POS	POS	POS	POS	AMB	POS	POS	POS	POS	AMB	NEG	NEG	NEG	POS	POS	POS	POS	NEG	NEG	NEG	POS	POS	POS	NEG	NEG	NEG	NEG	NEG	NEG	NEG	NEG	NEG	NEG	NEG	NEG	NEG	NEG	NEG	NEG	NEG
07V40665	CC45-MRSA-IV, Berlin EMRSA	MED-OPD	68	m	POS	POS	POS	POS		AMB	POS				POS	NEG	NEG	NEG	POS	POS																									
07V46779	CC45-MRSA-IV, Berlin EMRSA	MED-OPD	12	m	POS	POS	POS	POS	POS	POS	POS	POS	POS	POS	POS	NEG	NEG	NEG	POS	POS	POS	POS	NEG	NEG	NEG	POS	POS	POS	NEG	NEG	NEG	NEG	NEG	NEG	NEG	NEG	NEG	AMB	NEG	NEG	NEG	NEG	NEG	NEG	NEG
07V48383	CC45-MRSA-IV, Berlin EMRSA	SUR-WARD	66	m	POS	POS	POS	POS		POS	POS	POS			POS	NEG	NEG	NEG	POS	POS																									
07V25052	CC59-MRSA-V(T) [PVL+], Taiwan Clone	SUR-WARD	21	f	POS	POS	POS	POS	POS	POS	POS	POS	POS	POS	POS	NEG	NEG	NEG	POS	POS	NEG	POS	NEG	NEG	NEG	NEG	NEG	NEG	NEG	NEG	NEG	NEG	NEG	NEG	NEG	NEG	NEG	NEG	NEG	NEG	POS	POS	POS	NEG	NEG
07V03944	CC80-MRSA-IV [PVL+], European caMRSA Clone	MED-WARD	27	m	POS	POS	POS	POS		POS	POS				NEG	NEG	POS	NEG	POS	POS																									
07V34190	CC97-MRSA-IV, WA MRSA-54/63	MED-WARD	20	m	POS	POS	POS	POS	POS	POS	POS	POS	POS	POS	POS	NEG	NEG	NEG	POS	POS	POS	POS	NEG	NEG	NEG	NEG	POS	POS	NEG	NEG	NEG	NEG	NEG	NEG	NEG	NEG	NEG	NEG	NEG	NEG	NEG	NEG	NEG	NEG	NEG

		RESISTANCE : PENICILLINASE			RESISTANCE : MLS-ANTIBIOTICS												RESISTANCE : AMINOGLYSOSIDES			RESISTANCE : MISCELLANEOUS GENES																RESISTANCE : EFFLUX SYSTEMS								RESISTANCE : GLYCOPEPTIDES		
		beta-lactamase	beta lactamase repressor (inhibitor)	beta-lactamase regulatory protein	rRNA adenine N-6-methyl-transferase, erythromycin/clindamycin resistance	erythro-mycin/clindamycin resistance	erythro-mycin/clindamycin resistance	Linco-samid-Nucleo-tidyltrans-ferase	energy-dependent efflux of erythro-mycin	macrolide efflux protein A	probable lysylphos-phatidyl-glycerol synthetase	virginia-mycin A acetyltransferase	acetyl-transferase inactivating streptogramin A	ATP binding protein, streptogramin-A-resistance		virginia-mycin B hydrolase	bifunctional enzyme Aac/Aph, gentamicin resistance	amino-glycoside adenyl-transferase,tobramycin resistance	3'5'-aminog-lycoside phospho-transferase, neo-/ kanamycin resistance	strepto-thricine-acetyl-transferase	dihydro-folate reductase type 1	fusidic acid resistence	hypothetical protein associated with fusidic acid resistance	mupirocin resistence protein	tetrazyklin-resistance	tetracycline-resistance	chloramphenicol acetyltransferase					23S rRNA methyltransferase	chloramphenicol /florfenicol exporter	metallothiol transferase		quaternary ammonium compound resistance protein A	quaternary ammonium compound resistance protein C						Transport/ Efflux protein	vancomycin resistance gene	vancomycin resistance gene from enterococci and Clostridium	teicoplanin resistance gene from enterococci
ISOLATE:	STRAIN	blaZ	blaI	blaR	erm(A)	erm(B)	erm(C)	lnu(A)	msr(A)	mef(A)	mph(C)	vat(A)	vat(B)	vga(A)	vga(A) (BM 3327)	vgB(A)	aacA-aphD	aadD	aphA3	sat	dfrA	far1	Q6GD50	mupR	tet(K)	tet(M)	cat (total)	cat (pC221)	cat (pc223)	cat (pMC524)	cat (pSBK203R)	cfr	fexA	fosB	fosB (plasmid)	qacA	qacC (total)	qacC (consensus)	qacC (equine)	qacC (SA5)	qacC (Ssap)	qacC (ST94)	tetEfflux	vanA	vanB	vanZ
**2007**																																														
07V01446	CC1-MRSA-IV, WA MRSA-1/57	POS	POS	POS	NEG	NEG	NEG	NEG	NEG	NEG	NEG	NEG	NEG	NEG	NEG	NEG	NEG	NEG	NEG	NEG	NEG	NEG	NEG	NEG	NEG	NEG	NEG	NEG	NEG	NEG	NEG	NEG	NEG	NEG	NEG	NEG	NEG	NEG	NEG	NEG	NEG	NEG	POS	NEG	NEG	NEG
07F01032	CC5/ST228-MRSA-I, South German EMRSA	POS			POS		NEG	NEG	NEG			NEG	NEG	NEG	NEG	NEG	POS	NEG	POS	POS	NEG	NEG		NEG	NEG	NEG																		NEG	NEG	NEG
07V01588	CC5/ST228-MRSA-I, South German EMRSA	POS			POS		NEG	NEG	NEG			NEG	NEG	NEG	NEG	NEG	POS	NEG	POS	POS	NEG	NEG		NEG	NEG	NEG																		NEG	NEG	NEG
07V01876	CC5/ST228-MRSA-I, South German EMRSA	POS			POS		NEG	NEG	NEG			NEG	NEG	NEG	NEG	NEG	POS	NEG	POS	POS	NEG	NEG		NEG	NEG	NEG																		NEG	NEG	NEG
07V03020	CC5/ST228-MRSA-I, South German EMRSA	POS			NEG		NEG	NEG	NEG			NEG	NEG	NEG	NEG	NEG	POS	NEG	POS	POS	NEG	NEG		NEG	NEG	NEG																		NEG	NEG	NEG
07V03066	CC5/ST228-MRSA-I, South German EMRSA	POS			POS		NEG	NEG	NEG			NEG	NEG	NEG	NEG	NEG	POS	NEG	POS	POS	NEG	NEG		NEG	NEG	NEG																		NEG	NEG	NEG
07V08865	CC5/ST228-MRSA-I, South German EMRSA	POS	AMB	POS	POS		NEG	NEG	NEG	NEG	NEG	NEG	NEG	NEG	NEG	NEG	POS	NEG	POS	POS	NEG	NEG	NEG	NEG	NEG	NEG	NEG	NEG	NEG	NEG	NEG	NEG	NEG	POS	NEG	POS	NEG	NEG	NEG	NEG	NEG	NEG	AMB	NEG	NEG	NEG
07V35185	CC5/ST228-MRSA-I, South German EMRSA	NEG			POS		NEG	NEG	NEG			NEG	NEG	NEG	NEG	NEG	POS	NEG	POS	POS	NEG	NEG		NEG	NEG	NEG																		NEG	NEG	NEG
07V42548	CC5/ST228-MRSA-I, South German EMRSA	POS	POS	POS	POS		NEG	NEG	NEG	NEG	NEG	NEG	NEG	NEG	NEG	NEG	POS	NEG	POS	POS	NEG	NEG	NEG	NEG	NEG	NEG	NEG	NEG	NEG	NEG	NEG	NEG	NEG	POS	AMB	POS	NEG	NEG	NEG	NEG	NEG	NEG	POS	NEG	NEG	NEG
07V47082	CC5/ST228-MRSA-I, South German EMRSA	POS	POS	POS	POS		NEG	NEG	NEG	NEG	NEG	NEG	NEG	NEG	NEG	NEG	POS	NEG	POS	POS	NEG	NEG	NEG	NEG	NEG	NEG	NEG	NEG	NEG	NEG	NEG	NEG	NEG	POS	NEG	POS	NEG	NEG	NEG	NEG	NEG	NEG	POS	NEG	NEG	NEG
07V48389	CC5/ST228-MRSA-I, South German EMRSA	POS			POS		NEG	NEG	NEG			NEG	NEG	NEG	NEG	NEG	POS	NEG	POS	POS	NEG	NEG		NEG	NEG	NEG																		NEG	NEG	NEG
07V49263	CC5/ST228-MRSA-I, South German EMRSA	POS	POS	POS	POS		NEG	NEG	NEG	NEG	NEG	NEG	NEG	NEG	NEG	NEG	POS	NEG	POS	POS	NEG	NEG	NEG	NEG	NEG	NEG	NEG	NEG	NEG	NEG	NEG	NEG	NEG	POS	NEG	POS	NEG	NEG	NEG	NEG	NEG	NEG	POS	NEG	NEG	NEG
07V50349	CC5/ST228-MRSA-I, South German EMRSA	POS			POS		NEG	NEG	NEG			NEG	NEG	NEG	NEG	NEG	POS	NEG	POS	POS	NEG	NEG		NEG	NEG	NEG																		NEG	NEG	NEG
07B05103	CC5-MRSA-II, Rhine-Hesse EMRSA/UK-EMRSA-3	NEG			POS		NEG	NEG	NEG			NEG	NEG	NEG	NEG	NEG	NEG	POS	NEG	NEG	NEG	NEG		NEG	NEG	NEG																		NEG	NEG	NEG
07F00721	CC5-MRSA-II, Rhine-Hesse EMRSA/UK-EMRSA-3	NEG			POS		NEG	NEG	NEG			NEG	NEG	NEG	NEG	NEG	NEG	NEG	NEG	NEG	NEG	NEG		NEG	NEG	NEG																		NEG	NEG	NEG
07V00817	CC5-MRSA-II, Rhine-Hesse EMRSA/UK-EMRSA-3	NEG	NEG	NEG	POS		NEG	NEG	NEG	NEG	NEG	NEG	NEG	NEG	NEG	NEG	NEG	NEG	NEG	NEG	NEG	NEG	NEG	NEG	NEG	NEG	NEG	NEG	NEG	NEG	NEG	NEG	NEG	POS	NEG	NEG	NEG	NEG	NEG	NEG	NEG	NEG	POS	NEG	NEG	NEG
07V01111	CC5-MRSA-II, Rhine-Hesse EMRSA/UK-EMRSA-3	NEG			POS		NEG	NEG	NEG			NEG	NEG	NEG	NEG	NEG	NEG	POS	NEG	NEG	NEG	NEG		NEG	NEG	NEG																		NEG	NEG	NEG
07V01885	CC5-MRSA-II, Rhine-Hesse EMRSA/UK-EMRSA-3	NEG			POS		NEG	NEG	NEG			NEG	NEG	NEG	NEG	NEG	NEG	NEG	NEG	NEG	NEG	NEG		NEG	NEG	NEG																		NEG	NEG	NEG
07V04946	CC5-MRSA-II, Rhine-Hesse EMRSA/UK-EMRSA-3	NEG			POS		NEG	NEG	NEG			NEG	NEG	NEG	NEG	NEG	NEG	POS	NEG	NEG	NEG	NEG		NEG	NEG	NEG																		NEG	NEG	NEG
07V05167	CC5-MRSA-II, Rhine-Hesse EMRSA/UK-EMRSA-3	POS			POS		NEG	NEG	NEG			NEG	NEG	NEG	NEG	NEG	NEG	POS	NEG	NEG	NEG	NEG		NEG	NEG	NEG																		NEG	NEG	NEG
07V05703	CC5-MRSA-II, Rhine-Hesse EMRSA/UK-EMRSA-3	NEG			AMB		NEG	NEG	NEG			NEG	NEG	NEG	NEG	NEG	NEG	AMB	NEG	NEG	NEG	NEG		NEG	NEG	NEG																		NEG	NEG	NEG
07V08418	CC5-MRSA-II, Rhine-Hesse EMRSA/UK-EMRSA-3	POS			POS		NEG	NEG	NEG			NEG	NEG	NEG	NEG	NEG	NEG	POS	NEG	NEG	NEG	NEG		NEG	NEG	NEG																		NEG	NEG	NEG
07V09138	CC5-MRSA-II, Rhine-Hesse EMRSA/UK-EMRSA-3	POS			POS		NEG	NEG	NEG			NEG	NEG	NEG	NEG	NEG	NEG	POS	NEG	NEG	NEG	NEG		NEG	NEG	NEG																		NEG	NEG	NEG
07V11143	CC5-MRSA-II, Rhine-Hesse EMRSA/UK-EMRSA-3	NEG			POS		NEG	NEG	NEG			NEG	NEG	NEG	NEG	NEG	NEG	AMB	NEG	NEG	NEG	NEG		NEG	NEG	NEG																		NEG	NEG	NEG
07V14047	CC5-MRSA-II, Rhine-Hesse EMRSA/UK-EMRSA-3	AMB			POS		NEG	NEG	NEG			NEG	NEG	NEG	NEG	NEG	NEG	POS	NEG	NEG	NEG	NEG		NEG	NEG	NEG																		NEG	NEG	NEG
07V22331	CC5-MRSA-II, Rhine-Hesse EMRSA/UK-EMRSA-3	POS			POS		NEG	NEG	NEG			NEG	NEG	NEG	NEG	NEG	NEG	POS	NEG	NEG	NEG	NEG		NEG	NEG	NEG																		NEG	NEG	NEG
07V23848	CC5-MRSA-II, Rhine-Hesse EMRSA/UK-EMRSA-3	POS			POS		NEG	NEG	NEG			NEG	NEG	NEG	NEG	NEG	NEG	POS	NEG	NEG	NEG	NEG		NEG	NEG	NEG																		NEG	NEG	NEG
07V25349	CC5-MRSA-II, Rhine-Hesse EMRSA/UK-EMRSA-3	NEG			POS		NEG	NEG	NEG			NEG	NEG	NEG	NEG	NEG	NEG	POS	NEG	NEG	NEG	NEG		NEG	NEG	NEG																		NEG	NEG	NEG
07V29952	CC5-MRSA-II, Rhine-Hesse EMRSA/UK-EMRSA-3	POS			POS		NEG	NEG	NEG			NEG	NEG	NEG	NEG	NEG	NEG	POS	NEG	NEG	NEG	NEG		NEG	NEG	NEG																		NEG	NEG	NEG
07V32172	CC5-MRSA-II, Rhine-Hesse EMRSA/UK-EMRSA-3	POS			POS		NEG	NEG	NEG			NEG	NEG	NEG	NEG	NEG	NEG	POS	NEG	NEG	NEG	NEG		NEG	NEG	NEG																		NEG	NEG	NEG
07V34596	CC5-MRSA-II, Rhine-Hesse EMRSA/UK-EMRSA-3	POS			POS		NEG	NEG	NEG			NEG	NEG	NEG	NEG	NEG	NEG	POS	NEG	NEG	NEG	NEG		NEG	NEG	NEG																		NEG	NEG	NEG
07V34637	CC5-MRSA-II, Rhine-Hesse EMRSA/UK-EMRSA-3	POS			AMB		NEG	NEG	NEG			NEG	NEG	NEG	NEG	NEG	NEG	POS	NEG	NEG	NEG	NEG		NEG	NEG	NEG																		NEG	NEG	NEG
07V41577	CC5-MRSA-II, Rhine-Hesse EMRSA/UK-EMRSA-3	POS			POS		NEG	NEG	NEG			NEG	NEG	NEG	NEG	NEG	NEG	NEG	NEG	NEG	NEG	NEG		NEG	NEG	NEG																		NEG	NEG	NEG
07V43890	CC5-MRSA-II, Rhine-Hesse EMRSA/UK-EMRSA-3	POS			POS		NEG	NEG	NEG			NEG	NEG	NEG	NEG	NEG	NEG	POS	NEG	NEG	NEG	NEG		NEG	NEG	NEG																		NEG	NEG	NEG
07V44597	CC5-MRSA-II, Rhine-Hesse EMRSA/UK-EMRSA-3	POS			POS		NEG	NEG	NEG			NEG	NEG	NEG	NEG	NEG	NEG	POS	NEG	NEG	NEG	NEG		NEG	NEG	NEG																		NEG	NEG	NEG
07V48218	CC5-MRSA-II, Rhine-Hesse EMRSA/UK-EMRSA-3	POS	POS	POS	POS	0	NEG	NEG	NEG	NEG	NEG	NEG	NEG	NEG	NEG	NEG	NEG	POS	NEG	NEG	NEG	NEG	NEG	NEG	POS	NEG	NEG	NEG	NEG	NEG	NEG	NEG	NEG	POS	NEG	NEG	NEG	NEG	NEG	NEG	NEG	NEG	POS	NEG	NEG	NEG
07V48369	CC5-MRSA-II, Rhine-Hesse EMRSA/UK-EMRSA-3	POS			POS		NEG	NEG	NEG			NEG	NEG	NEG	NEG	NEG	NEG	NEG	NEG	NEG	NEG	NEG		NEG	NEG	NEG																		NEG	NEG	NEG
07V50153	CC5-MRSA-II, Rhine-Hesse EMRSA/UK-EMRSA-3	POS			POS		NEG	NEG	NEG			NEG	NEG	NEG	NEG	NEG	NEG	POS	NEG	NEG	NEG	NEG		NEG	NEG	NEG																		NEG	NEG	NEG
07V31319	CC5-MRSA-IV, Paediatric clone	POS	POS	POS	NEG	NEG	NEG	NEG	NEG	NEG	NEG	NEG	NEG	NEG	NEG	NEG	NEG	POS	NEG	NEG	NEG	NEG	NEG	NEG	POS	NEG	NEG	NEG	NEG	NEG	NEG	NEG	NEG	POS	NEG	NEG	NEG	NEG	NEG	NEG	NEG	NEG	POS	NEG	NEG	NEG
07V31590	CC8-MRSA-IV, Lyon Clone/UK-EMRSA-2	POS			NEG		NEG	NEG	NEG			NEG	NEG	NEG	NEG	NEG	NEG	POS	NEG	NEG	NEG	NEG		NEG	NEG	NEG																		NEG	NEG	NEG
07V31964	CC8-MRSA-IV, Lyon Clone/UK-EMRSA-2	POS			NEG		NEG	NEG	NEG			NEG	NEG	NEG	NEG	NEG	NEG	POS	NEG	NEG	NEG	NEG		NEG	NEG	NEG																		NEG	NEG	NEG
07V33137	CC8-MRSA-IV, Lyon Clone/UK-EMRSA-2	POS	POS	POS	NEG		NEG	NEG	NEG	NEG	NEG	NEG	NEG	NEG	NEG	NEG	NEG	POS	NEG	NEG	NEG	POS	NEG	NEG	POS	NEG	NEG	NEG	NEG	NEG	NEG	NEG	NEG	POS	POS	POS	NEG	NEG	NEG	NEG	NEG	NEG	POS	NEG	NEG	NEG
07V45114	CC8-MRSA-IV, Lyon Clone/UK-EMRSA-2	POS	POS	POS	NEG		NEG	NEG	NEG	NEG	NEG	NEG	NEG	NEG	NEG	NEG	NEG	POS	NEG	NEG	POS	NEG	NEG	NEG	NEG	NEG	NEG	NEG	NEG	NEG	NEG	NEG	NEG	POS	NEG	NEG	NEG	NEG	NEG	NEG	NEG	NEG	POS	NEG	NEG	NEG
07B06644	CC22-MRSA-IV, Barnim/UK-EMRSA-15	NEG			NEG		POS	NEG	NEG			NEG	NEG	NEG	NEG	NEG	NEG	NEG	NEG	NEG	NEG	NEG		NEG	NEG	NEG																		NEG	NEG	NEG
07F03422	CC22-MRSA-IV, Barnim/UK-EMRSA-15	POS			NEG		POS	NEG	NEG			NEG	NEG	NEG	NEG	NEG	NEG	NEG	NEG	NEG	NEG	NEG		NEG	NEG	NEG																		NEG	NEG	NEG
07F03905	CC22-MRSA-IV, Barnim/UK-EMRSA-15	POS			NEG		NEG	NEG	NEG			NEG	NEG	NEG	NEG	NEG	NEG	NEG	NEG	NEG	NEG	NEG		NEG	NEG	NEG																		NEG	NEG	NEG
07F06025	CC22-MRSA-IV, Barnim/UK-EMRSA-15	POS			NEG		NEG	NEG	NEG			NEG	NEG	NEG	NEG	NEG	NEG	NEG	NEG	NEG	NEG	NEG		NEG	NEG	NEG																		NEG	NEG	NEG
07F06053	CC22-MRSA-IV, Barnim/UK-EMRSA-15	AMB			NEG		NEG	NEG	NEG			NEG	NEG	NEG	NEG	NEG	NEG	NEG	NEG	NEG	NEG	NEG		NEG	NEG	NEG																		NEG	NEG	NEG
07F06432	CC22-MRSA-IV, Barnim/UK-EMRSA-15	POS			NEG		POS	NEG	NEG			NEG	NEG	NEG	NEG	NEG	NEG	NEG	NEG	NEG	NEG	NEG		NEG	NEG	NEG																		NEG	NEG	NEG
07U08245	CC22-MRSA-IV, Barnim/UK-EMRSA-15	POS			NEG		NEG	NEG	NEG			NEG	NEG	NEG	NEG	NEG	NEG	NEG	NEG	NEG	NEG	NEG		NEG	NEG	NEG																		NEG	NEG	NEG
07V01577	CC22-MRSA-IV, Barnim/UK-EMRSA-15	POS	POS	POS	NEG		NEG	NEG	NEG	NEG	NEG	NEG	NEG	NEG	NEG	NEG	NEG	NEG	NEG	NEG	NEG	NEG	NEG	NEG	NEG	NEG	NEG	NEG	NEG	NEG	NEG	NEG	NEG	NEG	NEG	NEG	NEG	NEG	NEG	NEG	NEG	NEG	NEG	NEG	NEG	NEG
07V02929	CC22-MRSA-IV, Barnim/UK-EMRSA-15	AMB			NEG		NEG	NEG	NEG			NEG	NEG	NEG	NEG	NEG	NEG	NEG	NEG	NEG	NEG	NEG		NEG	NEG	NEG																		NEG	NEG	NEG
07V03947	CC22-MRSA-IV, Barnim/UK-EMRSA-15	AMB			NEG		POS	NEG	NEG			NEG	NEG	NEG	NEG	NEG	NEG	NEG	NEG	NEG	NEG	NEG		NEG	NEG	NEG																		NEG	NEG	NEG
07V07685	CC22-MRSA-IV, Barnim/UK-EMRSA-15	POS			NEG		POS	NEG	NEG			NEG	NEG	NEG	NEG	NEG	NEG	NEG	NEG	NEG	NEG	NEG		NEG	NEG	NEG																		NEG	NEG	NEG
07V09789	CC22-MRSA-IV, Barnim/UK-EMRSA-15	AMB			NEG		POS	NEG	NEG			NEG	NEG	NEG	NEG	NEG	NEG	NEG	NEG	NEG	NEG	NEG		NEG	NEG	NEG																		NEG	NEG	NEG
07V11100	CC22-MRSA-IV, Barnim/UK-EMRSA-15	POS	POS	POS	NEG		NEG	NEG	NEG	NEG	NEG	NEG	NEG	NEG	NEG	NEG	NEG	NEG	NEG	NEG	NEG	NEG	NEG	NEG	NEG	NEG	NEG	NEG	NEG	NEG	NEG	NEG	NEG	NEG	NEG	NEG	NEG	NEG	NEG	NEG	NEG	NEG	AMB	NEG	NEG	NEG
07V13886	CC22-MRSA-IV, Barnim/UK-EMRSA-15	NEG			NEG		NEG	NEG	NEG			NEG	NEG	NEG	NEG	NEG	NEG	NEG	NEG	NEG	NEG	NEG		NEG	NEG	NEG																		NEG	NEG	NEG
07V13922	CC22-MRSA-IV, Barnim/UK-EMRSA-15	NEG			NEG		NEG	NEG	NEG			NEG	NEG	NEG	NEG	NEG	NEG	NEG	NEG	NEG	NEG	NEG		NEG	NEG	NEG																		NEG	NEG	NEG
07V15787	CC22-MRSA-IV, Barnim/UK-EMRSA-15	POS			NEG		NEG	NEG	NEG			NEG	NEG	NEG	NEG	NEG	NEG	NEG	NEG	NEG	NEG	NEG		NEG	NEG	NEG																		NEG	NEG	NEG
07V16040	CC22-MRSA-IV, Barnim/UK-EMRSA-15	NEG			NEG		NEG	NEG	NEG			NEG	NEG	NEG	NEG	NEG	NEG	NEG	NEG	NEG	NEG	NEG		NEG	NEG	NEG																		NEG	NEG	NEG
07V19106	CC22-MRSA-IV, Barnim/UK-EMRSA-15	NEG			NEG		POS	NEG	NEG			NEG	NEG	NEG	NEG	NEG	NEG	NEG	NEG	NEG	NEG	NEG		NEG	NEG	NEG																		NEG	NEG	NEG
07V19909	CC22-MRSA-IV, Barnim/UK-EMRSA-15	NEG			NEG		NEG	NEG	NEG			NEG	NEG	NEG	NEG	NEG	NEG	NEG	NEG	NEG	NEG	NEG		NEG	NEG	NEG																		NEG	NEG	NEG
07V20220	CC22-MRSA-IV, Barnim/UK-EMRSA-15	NEG			NEG		POS	NEG	NEG			NEG	NEG	NEG	NEG	NEG	NEG	NEG	NEG	NEG	NEG	NEG		NEG	NEG	NEG																		NEG	NEG	NEG
07V21255	CC22-MRSA-IV, Barnim/UK-EMRSA-15	NEG			NEG		POS	NEG	NEG			NEG	NEG	NEG	NEG	NEG	NEG	NEG	NEG	NEG	NEG	NEG		NEG	NEG	NEG																		NEG	NEG	NEG
07V22221	CC22-MRSA-IV, Barnim/UK-EMRSA-15	NEG			NEG		POS	NEG	NEG			NEG	NEG	NEG	NEG	NEG	NEG	NEG	NEG	NEG	NEG	NEG		NEG	NEG	NEG																		NEG	NEG	NEG
07V23398	CC22-MRSA-IV, Barnim/UK-EMRSA-15	AMB			NEG		NEG	NEG	NEG			NEG	NEG	NEG	NEG	NEG	NEG	NEG	NEG	NEG	NEG	NEG		NEG	NEG	NEG																		NEG	NEG	NEG
07V26255	CC22-MRSA-IV, Barnim/UK-EMRSA-15	NEG			NEG		NEG	NEG	NEG			NEG	NEG	NEG	NEG	NEG	NEG	NEG	NEG	NEG	NEG	NEG		NEG	NEG	NEG																		NEG	NEG	NEG
07V27224	CC22-MRSA-IV, Barnim/UK-EMRSA-15	NEG			NEG		POS	NEG	NEG			NEG	NEG	NEG	NEG	NEG	NEG	NEG	NEG	NEG	NEG	NEG		NEG	NEG	NEG																		NEG	NEG	NEG
07V29579	CC22-MRSA-IV, Barnim/UK-EMRSA-15	POS			NEG		NEG	NEG	NEG			NEG	NEG	NEG	NEG	NEG	NEG	NEG	NEG	NEG	NEG	NEG		NEG	NEG	NEG																		NEG	NEG	NEG
07V30685	CC22-MRSA-IV, Barnim/UK-EMRSA-15	POS			NEG		POS	NEG	NEG			NEG	NEG	NEG	NEG	NEG	NEG	NEG	NEG	NEG	NEG	NEG		NEG	NEG	NEG																		NEG	NEG	NEG
07V30827	CC22-MRSA-IV, Barnim/UK-EMRSA-15	POS			NEG		NEG	NEG	NEG			NEG	NEG	NEG	NEG	NEG	NEG	NEG	NEG	NEG	NEG	NEG		NEG	NEG	NEG																		NEG	NEG	NEG
07V31586	CC22-MRSA-IV, Barnim/UK-EMRSA-15	AMB			NEG		NEG	NEG	NEG			NEG	NEG	NEG	NEG	NEG	NEG	NEG	NEG	NEG	NEG	NEG		NEG	NEG	NEG																		NEG	NEG	NEG
07V31588	CC22-MRSA-IV, Barnim/UK-EMRSA-15	POS			NEG		NEG	NEG	NEG			NEG	NEG	NEG	NEG	NEG	NEG	NEG	NEG	NEG	NEG	NEG		NEG	NEG	NEG																		NEG	NEG	NEG
07V32055	CC22-MRSA-IV, Barnim/UK-EMRSA-15	POS			NEG		NEG	NEG	NEG			NEG	NEG	NEG	NEG	NEG	NEG	NEG	NEG	NEG	NEG	NEG		NEG	NEG	NEG																		NEG	NEG	NEG
07V32148	CC22-MRSA-IV, Barnim/UK-EMRSA-15	POS			NEG		NEG	NEG	NEG			NEG	NEG	NEG	NEG	NEG	NEG	NEG	NEG	NEG	NEG	NEG		NEG	NEG	NEG																		NEG	NEG	NEG
07V32390	CC22-MRSA-IV, Barnim/UK-EMRSA-15	POS			NEG		NEG	NEG	NEG			NEG	NEG	NEG	NEG	NEG	NEG	NEG	NEG	NEG	NEG	NEG		NEG	NEG	NEG																		NEG	NEG	NEG
07V32454	CC22-MRSA-IV, Barnim/UK-EMRSA-15	POS			NEG		NEG	NEG	NEG			NEG	NEG	NEG	NEG	NEG	NEG	NEG	NEG	NEG	NEG	NEG		NEG	NEG	NEG																		NEG	NEG	NEG
07V32457	CC22-MRSA-IV, Barnim/UK-EMRSA-15	POS	POS	POS	NEG	NEG	NEG	NEG	NEG	NEG	NEG	NEG	NEG	NEG	NEG	NEG	NEG	NEG	NEG	NEG	NEG	NEG	NEG	NEG	NEG	NEG	NEG	NEG	NEG	NEG	NEG	NEG	NEG	NEG	NEG	NEG	NEG	NEG	NEG	NEG	NEG	NEG	NEG	NEG	NEG	NEG
07V32467	CC22-MRSA-IV, Barnim/UK-EMRSA-15	POS			NEG		NEG	NEG	NEG			NEG	NEG	NEG	NEG	NEG	NEG	NEG	NEG	NEG	NEG	NEG		NEG	NEG	NEG																		NEG	NEG	NEG
07V32765	CC22-MRSA-IV, Barnim/UK-EMRSA-15	POS			NEG		NEG	NEG	NEG			NEG	NEG	NEG	NEG	NEG	NEG	NEG	NEG	NEG	NEG	NEG		NEG	NEG	NEG																		NEG	NEG	NEG
07V33857	CC22-MRSA-IV, Barnim/UK-EMRSA-15	POS			NEG		POS	NEG	NEG			NEG	NEG	NEG	NEG	NEG	NEG	NEG	NEG	NEG	NEG	NEG		NEG	NEG	NEG																		NEG	NEG	NEG
07V34379	CC22-MRSA-IV, Barnim/UK-EMRSA-15	POS			NEG		POS	NEG	NEG			NEG	NEG	NEG	NEG	NEG	NEG	NEG	NEG	NEG	NEG	NEG		NEG	NEG	NEG																		NEG	NEG	NEG
07V35110	CC22-MRSA-IV, Barnim/UK-EMRSA-15	POS			NEG		NEG	NEG	NEG			NEG	NEG	NEG	NEG	NEG	NEG	NEG	NEG	NEG	NEG	NEG		NEG	NEG	NEG																		NEG	NEG	NEG
07V35482	CC22-MRSA-IV, Barnim/UK-EMRSA-15	AMB			NEG		POS	NEG	NEG			NEG	NEG	NEG	NEG	NEG	NEG	NEG	NEG	NEG	NEG	NEG		NEG	NEG	NEG																		NEG	NEG	NEG
07V35565	CC22-MRSA-IV, Barnim/UK-EMRSA-15	POS			NEG		NEG	NEG	NEG			NEG	NEG	NEG	NEG	NEG	NEG	NEG	NEG	NEG	NEG	NEG		NEG	NEG	NEG																		NEG	NEG	NEG
07V37700	CC22-MRSA-IV, Barnim/UK-EMRSA-15	POS			NEG		NEG	NEG	NEG			NEG	NEG	NEG	NEG	NEG	NEG	NEG	NEG	NEG	NEG	NEG		NEG	NEG	NEG																		NEG	NEG	NEG
07V41045	CC22-MRSA-IV, Barnim/UK-EMRSA-15	NEG			NEG		NEG	NEG	NEG			NEG	NEG	NEG	NEG	NEG	NEG	NEG	NEG	NEG	NEG	NEG		NEG	NEG	NEG																		NEG	NEG	NEG
07V41051	CC22-MRSA-IV, Barnim/UK-EMRSA-15	AMB			NEG		NEG	NEG	NEG			NEG	NEG	NEG	NEG	NEG	NEG	NEG	NEG	NEG	NEG	NEG		NEG	NEG	NEG																		NEG	NEG	NEG
07V42673	CC22-MRSA-IV, Barnim/UK-EMRSA-15	AMB			NEG		POS	NEG	NEG			NEG	NEG	NEG	NEG	NEG	NEG	NEG	NEG	NEG	NEG	NEG		NEG	NEG	NEG																		NEG	NEG	NEG
07V42842	CC22-MRSA-IV, Barnim/UK-EMRSA-15	POS			NEG		NEG	NEG	NEG			NEG	NEG	NEG	NEG	NEG	NEG	POS	NEG	NEG	NEG	NEG		NEG	NEG	NEG																		NEG	NEG	NEG
07V45346	CC22-MRSA-IV, Barnim/UK-EMRSA-15	POS			NEG		POS	NEG	NEG			NEG	NEG	NEG	NEG	NEG	NEG	NEG	NEG	AMB	NEG	NEG		NEG	NEG	NEG																		NEG	NEG	NEG
07V46048	CC22-MRSA-IV, Barnim/UK-EMRSA-15	NEG			NEG		POS	NEG	NEG			NEG	NEG	NEG	NEG	NEG	POS	NEG	NEG	NEG	NEG	NEG		NEG	NEG	NEG																		NEG	NEG	NEG
07V46684	CC22-MRSA-IV, Barnim/UK-EMRSA-15	POS			NEG		NEG	NEG	NEG			NEG	NEG	NEG	NEG	NEG	NEG	NEG	NEG	NEG	NEG	NEG		NEG	NEG	NEG																		NEG	NEG	NEG
07V49690	CC22-MRSA-IV, Barnim/UK-EMRSA-15	POS			NEG		POS	NEG	NEG			NEG	NEG	NEG	NEG	NEG	NEG	NEG	NEG	NEG	NEG	NEG		NEG	NEG	NEG																		NEG	NEG	NEG
07V49998	CC22-MRSA-IV, Barnim/UK-EMRSA-15	POS			NEG		POS	NEG	NEG			NEG	NEG	NEG	NEG	NEG	NEG	NEG	NEG	AMB	NEG	NEG		NEG	NEG	NEG																		NEG	NEG	NEG
07V25999	CC30-MRSA-IV [PVL+], Southwest Pacific Clone	POS	POS	POS	NEG	NEG	NEG	NEG	NEG	NEG	NEG	NEG	NEG	NEG	NEG	NEG	POS	NEG	NEG	NEG	NEG	NEG	NEG	NEG	POS	NEG	NEG	NEG	NEG	NEG	NEG	NEG	NEG	POS	NEG	NEG	NEG	NEG	NEG	NEG	NEG	NEG	POS	NEG	NEG	NEG
07U07147	CC45-MRSA-IV, Berlin EMRSA	NEG			NEG		NEG	NEG	NEG			NEG	NEG	NEG	NEG	NEG	NEG	NEG	POS	POS	NEG	NEG		NEG	NEG	NEG																		NEG	NEG	NEG
07V03373	CC45-MRSA-IV, Berlin EMRSA	POS	AMB	POS	NEG		NEG	NEG	NEG	NEG	NEG	NEG	NEG	NEG	NEG	NEG	NEG	NEG	NEG	NEG	NEG	NEG	NEG	NEG	NEG	NEG	NEG	NEG	NEG	NEG	NEG	NEG	NEG	NEG	NEG	NEG	NEG	NEG	NEG	NEG	NEG	NEG	AMB	NEG	NEG	NEG
07V06916	CC45-MRSA-IV, Berlin EMRSA	AMB			NEG		NEG	NEG	NEG			NEG	NEG	NEG	NEG	NEG	NEG	NEG	NEG	NEG	NEG	NEG		NEG	NEG	NEG																		NEG	NEG	NEG
07V16850	CC45-MRSA-IV, Berlin EMRSA	POS			NEG		NEG	NEG	NEG			NEG	NEG	NEG	NEG	NEG	NEG	NEG	NEG	NEG	NEG	NEG		NEG	NEG	NEG																		NEG	NEG	NEG
07V17786	CC45-MRSA-IV, Berlin EMRSA	AMB			NEG		POS	NEG	NEG			NEG	NEG	NEG	NEG	NEG	NEG	NEG	POS	POS	NEG	NEG		NEG	NEG	NEG																		NEG	NEG	NEG
07V19855	CC45-MRSA-IV, Berlin EMRSA	POS	POS	POS	NEG		NEG	NEG	NEG	NEG	NEG	NEG	NEG	NEG	NEG	NEG	NEG	NEG	NEG	NEG	NEG	NEG	NEG	NEG	NEG	NEG	NEG	NEG	NEG	NEG	NEG	NEG	NEG	NEG	NEG	NEG	NEG	NEG	NEG	NEG	NEG	NEG	AMB	NEG	NEG	NEG
07V29398	CC45-MRSA-IV, Berlin EMRSA	POS	POS	POS	NEG		NEG	NEG	NEG	NEG	NEG	NEG	NEG	NEG	NEG	NEG	POS	POS	POS	POS	NEG	NEG	NEG	POS	NEG	NEG	NEG	NEG	NEG	NEG	NEG	NEG	NEG	NEG	NEG	NEG	POS	POS	NEG	NEG	NEG	NEG	POS	NEG	NEG	NEG
07V40665	CC45-MRSA-IV, Berlin EMRSA	POS			NEG		NEG	NEG	NEG			NEG	NEG	NEG	NEG	NEG	NEG	NEG	POS	POS	NEG	NEG		NEG	NEG	NEG																		NEG	NEG	NEG
07V46779	CC45-MRSA-IV, Berlin EMRSA	POS	POS	POS	POS		NEG	NEG	NEG	NEG	NEG	NEG	NEG	NEG	NEG	NEG	NEG	NEG	NEG	NEG	NEG	NEG	NEG	NEG	NEG	NEG	NEG	NEG	NEG	NEG	NEG	NEG	NEG	NEG	NEG	NEG	NEG	NEG	NEG	NEG	NEG	NEG	POS	NEG	NEG	NEG
07V48383	CC45-MRSA-IV, Berlin EMRSA	POS			NEG		NEG	NEG	NEG			NEG	NEG	NEG	NEG	NEG	NEG	NEG	POS	POS	NEG	NEG		NEG	NEG	NEG																		NEG	NEG	NEG
07V25052	CC59-MRSA-V(T) [PVL+], Taiwan Clone	POS	POS	POS	NEG	POS	NEG	NEG	NEG	NEG	NEG	NEG	NEG	NEG	NEG	NEG	NEG	NEG	POS	POS	NEG	NEG	NEG	NEG	POS	NEG	NEG	NEG	NEG	NEG	NEG	NEG	NEG	NEG	NEG	NEG	NEG	NEG	NEG	NEG	NEG	NEG	POS	NEG	NEG	NEG
07V03944	CC80-MRSA-IV [PVL+], European caMRSA Clone	POS			NEG		NEG	NEG	NEG			NEG	NEG	NEG	NEG	NEG	NEG	NEG	POS	POS	NEG	POS		NEG	AMB	NEG																		NEG	NEG	NEG
07V34190	CC97-MRSA-IV, WA MRSA-54/63	POS	POS	POS	NEG	NEG	NEG	NEG	NEG	NEG	NEG	NEG	NEG	NEG	NEG	NEG	NEG	POS	NEG	NEG	NEG	NEG	NEG	NEG	POS	NEG	NEG	NEG	NEG	NEG	NEG	NEG	NEG	NEG	NEG	NEG	NEG	NEG	NEG	NEG	NEG	NEG	POS	NEG	NEG	NEG

2007

ISOLATE:	STRAIN	VIRULENCE : TOX.SCHOCK.TOXIN — toxic shock syndrome toxin 1			VIRULENCE : ENTEROTOXINS — enterotoxin A	enterotoxin A, allele from strain 320E	enterotoxin A, allele from strain N315 = entero-toxin P	enterotoxin B	enterotoxin C	enterotoxin D	enterotoxin E	enterotoxin G	enterotoxin H	enterotoxin I	enterotoxin J	enterotoxin K
		tst1 (consensus)	tst1 ("human" allele)	tst1 ("bovine" allele)	sea	sea (320E)	sea (N315) / sep	seb	sec	sed	sef	seg	seh	sei	sej	sek
07V01446	CC1-MRSA-IV, WA MRSA-1/57	NEG	NEG	NEG	POS	NEG	NEG	NEG	NEG	NEG	NEG	NEG	POS	NEG	NEG	POS
07F01032	CC5/ST228-MRSA-I, South German EMRSA		NEG	NEG	POS	NEG	NEG	NEG	NEG	NEG	NEG	POS	NEG	POS	NEG	
07V01588	CC5/ST228-MRSA-I, South German EMRSA		NEG	NEG	POS	NEG	NEG	NEG	NEG	NEG	NEG	POS	NEG	POS	NEG	
07V01876	CC5/ST228-MRSA-I, South German EMRSA		NEG	NEG	POS	NEG	NEG	NEG	NEG	NEG	NEG	NEG	NEG	NEG	NEG	
07V03020	CC5/ST228-MRSA-I, South German EMRSA		NEG	NEG	POS	NEG	NEG	NEG	NEG	NEG	NEG	POS	NEG	POS	NEG	
07V03066	CC5/ST228-MRSA-I, South German EMRSA		NEG	NEG	POS	NEG	NEG	NEG	NEG	NEG	NEG	POS	NEG	POS	NEG	
07V08865	CC5/ST228-MRSA-I, South German EMRSA	NEG	NEG	NEG	POS	NEG	NEG	NEG	NEG	NEG	NEG	POS	NEG	POS	NEG	NEG
07V35185	CC5/ST228-MRSA-I, South German EMRSA		NEG	NEG	POS	NEG	NEG	NEG	NEG	NEG	NEG	POS	NEG	POS	NEG	
07V42548	CC5/ST228-MRSA-I, South German EMRSA	AMB	NEG	NEG	POS	NEG	NEG	NEG	NEG	NEG	NEG	AMB	NEG	POS	NEG	NEG
07V47082	CC5/ST228-MRSA-I, South German EMRSA	NEG	NEG	NEG	POS	NEG	NEG	NEG	NEG	NEG	NEG	POS	NEG	POS	NEG	NEG
07V48389	CC5/ST228-MRSA-I, South German EMRSA		NEG	NEG	POS	AMB	NEG	NEG	NEG	NEG	NEG	POS	NEG	POS	NEG	
07V49263	CC5/ST228-MRSA-I, South German EMRSA	NEG	NEG	NEG	POS	NEG	NEG	NEG	NEG	NEG	NEG	POS	NEG	POS	NEG	NEG
07V50349	CC5/ST228-MRSA-I, South German EMRSA		NEG	NEG	POS	NEG	NEG	NEG	NEG	NEG	NEG	POS	NEG	POS	NEG	
07B05103	CC5-MRSA-II, Rhine-Hesse EMRSA/UK-EMRSA-3		NEG	NEG	NEG	NEG	POS	NEG	NEG	NEG	NEG	POS	NEG	POS	NEG	
07F00721	CC5-MRSA-II, Rhine-Hesse EMRSA/UK-EMRSA-3		NEG	NEG	NEG	NEG	POS	NEG	NEG	NEG	NEG	POS	NEG	POS	NEG	
07V00817	CC5-MRSA-II, Rhine-Hesse EMRSA/UK-EMRSA-3	NEG	NEG	NEG	NEG	NEG	POS	NEG	NEG	NEG	NEG	POS	NEG	POS	NEG	NEG
07V01111	CC5-MRSA-II, Rhine-Hesse EMRSA/UK-EMRSA-3		NEG	NEG	NEG	NEG	POS	NEG	NEG	NEG	NEG	POS	NEG	POS	NEG	
07V01885	CC5-MRSA-II, Rhine-Hesse EMRSA/UK-EMRSA-3		NEG	NEG	NEG	NEG	POS	NEG	NEG	NEG	NEG	POS	NEG	POS	NEG	
07V04946	CC5-MRSA-II, Rhine-Hesse EMRSA/UK-EMRSA-3		NEG	NEG	NEG	NEG	POS	NEG	NEG	NEG	NEG	POS	NEG	POS	NEG	
07V05167	CC5-MRSA-II, Rhine-Hesse EMRSA/UK-EMRSA-3		NEG	NEG	NEG	NEG	POS	NEG	NEG	POS	NEG	POS	NEG	POS	POS	
07V05703	CC5-MRSA-II, Rhine-Hesse EMRSA/UK-EMRSA-3		NEG	NEG	NEG	NEG	POS	NEG	NEG	POS	NEG	AMB	NEG	POS	POS	
07V08418	CC5-MRSA-II, Rhine-Hesse EMRSA/UK-EMRSA-3		NEG	NEG	NEG	NEG	POS	NEG	NEG	POS	NEG	POS	NEG	POS	POS	
07V09138	CC5-MRSA-II, Rhine-Hesse EMRSA/UK-EMRSA-3		NEG	NEG	NEG	NEG	POS	NEG	NEG	POS	NEG	POS	NEG	POS	POS	
07V11143	CC5-MRSA-II, Rhine-Hesse EMRSA/UK-EMRSA-3		NEG	NEG	NEG	NEG	POS	NEG	NEG	POS	NEG	POS	NEG	POS	POS	
07V14047	CC5-MRSA-II, Rhine-Hesse EMRSA/UK-EMRSA-3		NEG	NEG	NEG	NEG	POS	NEG	NEG	POS	NEG	POS	NEG	POS	POS	
07V22331	CC5-MRSA-II, Rhine-Hesse EMRSA/UK-EMRSA-3		NEG	NEG	NEG	NEG	POS	NEG	NEG	POS	NEG	POS	NEG	POS	POS	
07V23848	CC5-MRSA-II, Rhine-Hesse EMRSA/UK-EMRSA-3		NEG	NEG	NEG	NEG	POS	NEG	NEG	POS	NEG	POS	NEG	POS	POS	
07V25349	CC5-MRSA-II, Rhine-Hesse EMRSA/UK-EMRSA-3		NEG	NEG	NEG	NEG	POS	NEG	NEG	NEG	NEG	POS	NEG	POS	NEG	
07V29952	CC5-MRSA-II, Rhine-Hesse EMRSA/UK-EMRSA-3		NEG	NEG	NEG	NEG	POS	NEG	NEG	POS	NEG	POS	NEG	POS	POS	
07V32172	CC5-MRSA-II, Rhine-Hesse EMRSA/UK-EMRSA-3		NEG	NEG	NEG	NEG	NEG	NEG	NEG	POS	NEG	POS	NEG	POS	POS	
07V34596	CC5-MRSA-II, Rhine-Hesse EMRSA/UK-EMRSA-3		NEG	NEG	NEG	NEG	NEG	NEG	NEG	POS	NEG	POS	NEG	POS	POS	
07V34637	CC5-MRSA-II, Rhine-Hesse EMRSA/UK-EMRSA-3		NEG	NEG	NEG	NEG	POS	NEG	NEG	POS	NEG	POS	NEG	POS	POS	
07V41577	CC5-MRSA-II, Rhine-Hesse EMRSA/UK-EMRSA-3		NEG	NEG	NEG	NEG	POS	NEG	NEG	POS	NEG	POS	NEG	POS	POS	
07V43890	CC5-MRSA-II, Rhine-Hesse EMRSA/UK-EMRSA-3		NEG	NEG	NEG	NEG	POS	NEG	NEG	POS	NEG	POS	NEG	POS	POS	
07V44597	CC5-MRSA-II, Rhine-Hesse EMRSA/UK-EMRSA-3		NEG	NEG	NEG	NEG	POS	NEG	NEG	POS	NEG	POS	NEG	POS	POS	
07V48218	CC5-MRSA-II, Rhine-Hesse EMRSA/UK-EMRSA-3	NEG	NEG	NEG	NEG	NEG	POS	NEG	NEG	POS	NEG	POS	NEG	POS	POS	NEG
07V48369	CC5-MRSA-II, Rhine-Hesse EMRSA/UK-EMRSA-3		NEG	NEG	NEG	NEG	POS	NEG	NEG	POS	NEG	POS	NEG	POS	POS	
07V50153	CC5-MRSA-II, Rhine-Hesse EMRSA/UK-EMRSA-3		NEG	NEG	NEG	NEG	NEG	NEG	NEG	POS	NEG	POS	NEG	POS	POS	
07V31319	CC5-MRSA-IV, Paediatric clone	NEG	NEG	NEG	NEG	NEG	POS	POS	NEG	POS	NEG	POS	NEG	POS	POS	NEG
07V31590	CC8-MRSA-IV, Lyon Clone/UK-EMRSA-2		NEG	NEG	POS	NEG	NEG	NEG	NEG	POS	NEG	NEG	NEG	NEG	POS	
07V31964	CC8-MRSA-IV, Lyon Clone/UK-EMRSA-2		NEG	NEG	POS	NEG	NEG	NEG	NEG	POS	NEG	NEG	NEG	NEG	POS	
07V33137	CC8-MRSA-IV, Lyon Clone/UK-EMRSA-2	NEG	NEG	NEG	POS	NEG	NEG	NEG	NEG	POS	NEG	NEG	NEG	NEG	POS	NEG
07V45114	CC8-MRSA-IV, Lyon Clone/UK-EMRSA-2	NEG	NEG	NEG	POS	NEG	NEG	NEG	NEG	POS	NEG	NEG	NEG	NEG	POS	NEG
07B06644	CC22-MRSA-IV, Barnim/UK-EMRSA-15		NEG	NEG	NEG	NEG	NEG	NEG	NEG	NEG	NEG	POS	NEG	POS	NEG	
07F03422	CC22-MRSA-IV, Barnim/UK-EMRSA-15		NEG	NEG	NEG	NEG	NEG	NEG	NEG	NEG	NEG	POS	NEG	POS	NEG	
07F03905	CC22-MRSA-IV, Barnim/UK-EMRSA-15		NEG	NEG	NEG	NEG	NEG	NEG	NEG	NEG	NEG	POS	NEG	POS	NEG	
07F06025	CC22-MRSA-IV, Barnim/UK-EMRSA-15		NEG	NEG	NEG	NEG	NEG	NEG	NEG	NEG	NEG	POS	NEG	POS	NEG	
07F06053	CC22-MRSA-IV, Barnim/UK-EMRSA-15		NEG	NEG	NEG	NEG	NEG	NEG	NEG	NEG	NEG	POS	NEG	POS	NEG	
07F06432	CC22-MRSA-IV, Barnim/UK-EMRSA-15		NEG	NEG	NEG	NEG	NEG	NEG	NEG	NEG	NEG	POS	NEG	POS	NEG	
07U08245	CC22-MRSA-IV, Barnim/UK-EMRSA-15		NEG	NEG	NEG	NEG	NEG	NEG	NEG	NEG	NEG	POS	NEG	POS	NEG	
07V01577	CC22-MRSA-IV, Barnim/UK-EMRSA-15	NEG	NEG	NEG	NEG	NEG	NEG	NEG	NEG	NEG	NEG	POS	NEG	POS	NEG	NEG
07V02929	CC22-MRSA-IV, Barnim/UK-EMRSA-15		NEG	NEG	NEG	NEG	NEG	NEG	POS	NEG	NEG	POS	NEG	POS	NEG	
07V03947	CC22-MRSA-IV, Barnim/UK-EMRSA-15		NEG	NEG	NEG	NEG	NEG	NEG	NEG	NEG	NEG	POS	NEG	POS	NEG	
07V07685	CC22-MRSA-IV, Barnim/UK-EMRSA-15		NEG	NEG	NEG	NEG	NEG	NEG	NEG	NEG	NEG	POS	NEG	POS	NEG	
07V09789	CC22-MRSA-IV, Barnim/UK-EMRSA-15		NEG	NEG	NEG	NEG	NEG	NEG	POS	NEG	NEG	POS	NEG	POS	NEG	
07V11100	CC22-MRSA-IV, Barnim/UK-EMRSA-15	NEG	NEG	NEG	NEG	NEG	NEG	NEG	POS	NEG	NEG	POS	NEG	POS	NEG	NEG
07V13886	CC22-MRSA-IV, Barnim/UK-EMRSA-15		NEG	NEG	NEG	NEG	NEG	NEG	NEG	NEG	NEG	POS	NEG	POS	NEG	
07V13922	CC22-MRSA-IV, Barnim/UK-EMRSA-15		NEG	NEG	NEG	NEG	NEG	NEG	NEG	NEG	NEG	POS	NEG	POS	NEG	
07V15787	CC22-MRSA-IV, Barnim/UK-EMRSA-15		NEG	NEG	NEG	NEG	NEG	NEG	NEG	NEG	NEG	POS	NEG	POS	NEG	
07V16040	CC22-MRSA-IV, Barnim/UK-EMRSA-15		NEG	NEG	NEG	NEG	NEG	NEG	NEG	NEG	NEG	POS	NEG	POS	NEG	
07V19106	CC22-MRSA-IV, Barnim/UK-EMRSA-15		NEG	NEG	NEG	NEG	NEG	NEG	NEG	NEG	NEG	POS	NEG	POS	NEG	
07V19909	CC22-MRSA-IV, Barnim/UK-EMRSA-15		NEG	NEG	NEG	NEG	NEG	NEG	NEG	NEG	NEG	AMB	NEG	POS	NEG	
07V20220	CC22-MRSA-IV, Barnim/UK-EMRSA-15		NEG	NEG	NEG	NEG	NEG	NEG	NEG	NEG	NEG	POS	NEG	POS	NEG	
07V21255	CC22-MRSA-IV, Barnim/UK-EMRSA-15		NEG	NEG	NEG	NEG	NEG	NEG	NEG	NEG	NEG	POS	NEG	POS	NEG	
07V22221	CC22-MRSA-IV, Barnim/UK-EMRSA-15		NEG	NEG	NEG	NEG	NEG	NEG	POS	NEG	NEG	POS	NEG	POS	NEG	
07V23398	CC22-MRSA-IV, Barnim/UK-EMRSA-15		NEG	NEG	NEG	NEG	NEG	NEG	NEG	NEG	NEG	POS	NEG	POS	NEG	
07V26255	CC22-MRSA-IV, Barnim/UK-EMRSA-15		NEG	NEG	NEG	NEG	NEG	NEG	NEG	NEG	NEG	POS	NEG	POS	NEG	
07V27224	CC22-MRSA-IV, Barnim/UK-EMRSA-15		NEG	NEG	NEG	NEG	NEG	NEG	NEG	NEG	NEG	POS	NEG	POS	NEG	
07V29579	CC22-MRSA-IV, Barnim/UK-EMRSA-15		NEG	NEG	NEG	NEG	NEG	NEG	NEG	NEG	NEG	POS	NEG	POS	NEG	
07V30685	CC22-MRSA-IV, Barnim/UK-EMRSA-15		NEG	NEG	NEG	NEG	NEG	NEG	POS	NEG	NEG	POS	NEG	POS	NEG	
07V30827	CC22-MRSA-IV, Barnim/UK-EMRSA-15		NEG	NEG	NEG	NEG	NEG	NEG	NEG	NEG	NEG	POS	NEG	POS	NEG	
07V31586	CC22-MRSA-IV, Barnim/UK-EMRSA-15		NEG	NEG	NEG	NEG	NEG	NEG	NEG	NEG	NEG	POS	NEG	POS	NEG	
07V31588	CC22-MRSA-IV, Barnim/UK-EMRSA-15		NEG	NEG	NEG	NEG	NEG	NEG	NEG	NEG	NEG	POS	NEG	POS	NEG	
07V32055	CC22-MRSA-IV, Barnim/UK-EMRSA-15		NEG	NEG	NEG	NEG	NEG	NEG	NEG	NEG	NEG	POS	NEG	POS	NEG	
07V32148	CC22-MRSA-IV, Barnim/UK-EMRSA-15		NEG	NEG	NEG	NEG	NEG	NEG	NEG	NEG	NEG	POS	NEG	POS	NEG	
07V32390	CC22-MRSA-IV, Barnim/UK-EMRSA-15		NEG	NEG	NEG	NEG	NEG	NEG	NEG	NEG	NEG	POS	NEG	POS	NEG	
07V32454	CC22-MRSA-IV, Barnim/UK-EMRSA-15		NEG	NEG	NEG	NEG	NEG	NEG	NEG	NEG	NEG	POS	NEG	POS	NEG	
07V32457	CC22-MRSA-IV, Barnim/UK-EMRSA-15	NEG	NEG	NEG	NEG	NEG	NEG	NEG	NEG	NEG	NEG	POS	NEG	POS	NEG	NEG
07V32467	CC22-MRSA-IV, Barnim/UK-EMRSA-15		NEG	NEG	NEG	NEG	NEG	NEG	NEG	NEG	NEG	POS	NEG	POS	NEG	
07V32765	CC22-MRSA-IV, Barnim/UK-EMRSA-15		NEG	NEG	NEG	NEG	NEG	NEG	NEG	NEG	NEG	POS	NEG	POS	NEG	
07V33857	CC22-MRSA-IV, Barnim/UK-EMRSA-15		NEG	NEG	NEG	NEG	NEG	NEG	NEG	NEG	NEG	POS	NEG	POS	NEG	
07V34379	CC22-MRSA-IV, Barnim/UK-EMRSA-15		NEG	NEG	NEG	NEG	NEG	NEG	NEG	NEG	NEG	POS	NEG	POS	NEG	
07V35110	CC22-MRSA-IV, Barnim/UK-EMRSA-15		NEG	NEG	NEG	NEG	NEG	NEG	NEG	NEG	NEG	POS	NEG	POS	NEG	
07V35482	CC22-MRSA-IV, Barnim/UK-EMRSA-15		NEG	NEG	NEG	NEG	NEG	NEG	POS	NEG	NEG	POS	NEG	POS	NEG	
07V35565	CC22-MRSA-IV, Barnim/UK-EMRSA-15		NEG	NEG	NEG	NEG	NEG	NEG	NEG	NEG	NEG	POS	NEG	POS	NEG	
07V37700	CC22-MRSA-IV, Barnim/UK-EMRSA-15		NEG	NEG	NEG	NEG	NEG	NEG	NEG	NEG	NEG	POS	NEG	POS	NEG	
07V41045	CC22-MRSA-IV, Barnim/UK-EMRSA-15		NEG	NEG	NEG	NEG	NEG	NEG	NEG	NEG	NEG	POS	NEG	POS	NEG	
07V41051	CC22-MRSA-IV, Barnim/UK-EMRSA-15		NEG	NEG	NEG	NEG	NEG	NEG	NEG	NEG	NEG	POS	NEG	POS	NEG	
07V42673	CC22-MRSA-IV, Barnim/UK-EMRSA-15		NEG	NEG	NEG	NEG	NEG	NEG	POS	NEG	NEG	POS	NEG	POS	NEG	
07V42842	CC22-MRSA-IV, Barnim/UK-EMRSA-15		NEG	NEG	NEG	NEG	NEG	NEG	NEG	NEG	NEG	POS	NEG	POS	NEG	
07V45346	CC22-MRSA-IV, Barnim/UK-EMRSA-15		NEG	NEG	NEG	NEG	NEG	NEG	NEG	NEG	NEG	POS	NEG	POS	NEG	
07V46048	CC22-MRSA-IV, Barnim/UK-EMRSA-15		NEG	NEG	NEG	NEG	NEG	NEG	POS	NEG	NEG	POS	NEG	POS	NEG	
07V46684	CC22-MRSA-IV, Barnim/UK-EMRSA-15		NEG	NEG	NEG	NEG	NEG	NEG	NEG	NEG	NEG	POS	NEG	POS	NEG	
07V49690	CC22-MRSA-IV, Barnim/UK-EMRSA-15		NEG	NEG	NEG	NEG	NEG	NEG	NEG	NEG	NEG	POS	NEG	POS	NEG	
07V49998	CC22-MRSA-IV, Barnim/UK-EMRSA-15		NEG	NEG	NEG	NEG	NEG	AMB	POS	NEG	NEG	POS	NEG	POS	NEG	
07V25999	CC30-MRSA-IV [PVL+], Southwest Pacific Clone	NEG	NEG	NEG	NEG	NEG	NEG	NEG	NEG	NEG	NEG	POS	NEG	POS	NEG	NEG
07U07147	CC45-MRSA-IV, Berlin EMRSA		NEG	NEG	NEG	NEG	NEG	NEG	NEG	NEG	NEG	POS	NEG	POS	NEG	
07V03373	CC45-MRSA-IV, Berlin EMRSA	NEG	NEG	NEG	NEG	NEG	NEG	NEG	NEG	NEG	NEG	NEG	NEG	POS	NEG	NEG
07V06916	CC45-MRSA-IV, Berlin EMRSA		NEG	NEG	NEG	NEG	NEG	NEG	NEG	NEG	NEG	NEG	NEG	POS	NEG	
07V16850	CC45-MRSA-IV, Berlin EMRSA		NEG	NEG	NEG	NEG	NEG	NEG	NEG	NEG	NEG	NEG	NEG	POS	NEG	
07V17786	CC45-MRSA-IV, Berlin EMRSA		NEG	NEG	NEG	NEG	NEG	NEG	NEG	NEG	NEG	POS	NEG	POS	NEG	
07V19855	CC45-MRSA-IV, Berlin EMRSA	NEG	NEG	NEG	NEG	NEG	NEG	NEG	POS	NEG	NEG	POS	NEG	POS	NEG	NEG
07V29398	CC45-MRSA-IV, Berlin EMRSA	NEG	NEG	NEG	NEG	NEG	NEG	NEG	NEG	NEG	NEG	POS	NEG	POS	NEG	NEG
07V40665	CC45-MRSA-IV, Berlin EMRSA		NEG	NEG	NEG	NEG	NEG	NEG	NEG	NEG	NEG	POS	NEG	POS	NEG	
07V46779	CC45-MRSA-IV, Berlin EMRSA	NEG	NEG	NEG	NEG	NEG	NEG	NEG	NEG	NEG	NEG	POS	NEG	POS	NEG	NEG
07V48383	CC45-MRSA-IV, Berlin EMRSA		NEG	NEG	NEG	NEG	NEG	NEG	NEG	NEG	NEG	NEG	NEG	POS	NEG	
07V25052	CC59-MRSA-V(T) [PVL+], Taiwan Clone	NEG	NEG	NEG	NEG	NEG	NEG	POS	NEG	NEG	NEG	NEG	NEG	NEG	NEG	POS
07V03944	CC80-MRSA-IV [PVL+], European caMRSA Clone		NEG	NEG	NEG	NEG	NEG	NEG	NEG	NEG	NEG	NEG	NEG	NEG	NEG	
07V34190	CC97-MRSA-IV, WA MRSA-54/63	NEG	NEG	NEG	NEG	NEG	NEG	NEG	NEG	NEG	NEG	NEG	NEG	NEG	NEG	NEG

ISOLATE:	enterotoxin L	enterotoxin M	enterotoxin N		enterotoxin O	enterotoxin Q	enterotoxin R	enterotoxin U	enterotoxin-like protein ORF CM14	VIRULENCE : HLG AND LEUKOCIDINS — haemolysin gamma / leukocidin, component B	haemolysin gamma / leukocidin, component C		haemolysin gamma, component A	Panton Valentine leukocidin F component	Panton Valentine leukocidin S component	F component from hypothetical leukocidin from ruminants	S component from hypothetical leukocidin from ruminants	leukocidin D component	leukocidin E component	leukocidin/ haemolysin toxin family protein	leukocidin/haemolysin toxin family protein	
	sel	selm	seln (consensus)	seln (other than RF122)	selo	seq	ser	selu	ORF CM14	lukF	lukS	lukS (ST22+ST45)	hlgA	lukF-PV	lukS-PV	lukF-PV (P83)	lukM	lukD	lukE	lukX	lukY	lukY (ST30+ST45)
07V01446	NEG	NEG	NEG	NEG	NEG	POS	NEG	NEG	NEG	POS	POS	AMB	POS	NEG	NEG	NEG	NEG	POS	POS	POS	POS	NEG
07F01032	NEG	POS		POS	POS		NEG			POS	POS		POS	NEG	NEG	NEG	NEG	POS	POS	NEG	POS	NEG
07V01588	NEG	POS		AMB	POS		NEG			POS	POS		POS	NEG	NEG	NEG	NEG	POS	POS	NEG	POS	NEG
07V01876	NEG	NEG		NEG	POS		NEG			POS	POS		POS	NEG	NEG	NEG	NEG	NEG	NEG	NEG	POS	NEG
07V03020	NEG	POS		POS	POS		NEG			POS	POS		POS	NEG	NEG	NEG	NEG	POS	POS	AMB	POS	NEG
07V03066	NEG	POS		AMB	POS		NEG			POS	POS		POS	NEG	NEG	NEG	NEG	POS	POS	NEG	POS	NEG
07V08865	NEG	POS	POS	NEG	POS	NEG	NEG	NEG	NEG	POS	POS	NEG	POS	NEG	NEG	NEG	NEG	POS	POS	NEG	POS	NEG
07V35185	NEG	POS		AMB	POS		NEG			POS	POS		POS	NEG	NEG	NEG	NEG	POS	POS	NEG	POS	NEG
07V42548	NEG	POS	AMB	POS	POS	NEG	NEG	POS	NEG	POS	POS	POS	POS	NEG	NEG	NEG	NEG	POS	POS	NEG	POS	NEG
07V47082	NEG	POS	POS	POS	POS	NEG	NEG	POS	NEG	POS	POS	AMB	POS	NEG	NEG	NEG	NEG	POS	POS	POS	POS	NEG
07V48389	NEG	POS		POS	POS		NEG			POS	POS		POS	NEG	NEG	NEG	NEG	POS	POS	POS	POS	NEG
07V49263	NEG	POS	POS	POS	POS	NEG	NEG	POS	NEG	POS	POS	POS	POS	NEG	NEG	NEG	NEG	POS	POS	AMB	POS	NEG
07V50349	NEG	POS		POS	POS		NEG			POS	POS		POS	NEG	NEG	NEG	NEG	POS	POS	POS	POS	NEG
07B05103	NEG	POS		POS	POS		NEG			POS	POS		POS	NEG	NEG	NEG	NEG	POS	POS	POS	POS	NEG
07F00721	NEG	POS		POS	POS		NEG			POS	POS		POS	NEG	NEG	NEG	NEG	POS	POS	POS	POS	NEG
07V00817	NEG	POS	POS	POS	POS	NEG	NEG	POS	NEG	POS	POS	NEG	POS	NEG	NEG	NEG	NEG	POS	POS	AMB	POS	NEG
07V01111	NEG	POS		POS	POS		NEG			POS	POS		POS	NEG	NEG	NEG	NEG	POS	POS	POS	POS	NEG
07V01885	NEG	POS		POS	POS		NEG			POS	POS		POS	NEG	NEG	NEG	NEG	POS	POS	POS	POS	NEG
07V04946	NEG	POS		POS	POS		NEG			POS	POS		POS	NEG	NEG	NEG	NEG	POS	POS	NEG	POS	NEG
07V05167	NEG	POS		POS	POS		POS			POS	POS		POS	NEG	NEG	NEG	NEG	POS	POS	POS	POS	NEG
07V05703	NEG	POS		NEG	AMB		NEG			POS	AMB		POS	NEG	NEG	NEG	NEG	POS	AMB	NEG	POS	NEG
07V08418	NEG	POS		POS	POS		POS			POS	POS		POS	NEG	NEG	NEG	NEG	POS	POS	AMB	POS	NEG
07V09138	NEG	POS		POS	POS		POS			POS	POS		POS	NEG	NEG	NEG	NEG	POS	POS	POS	POS	NEG
07V11143	NEG	POS		NEG	NEG		NEG			POS	AMB		POS	NEG	NEG	NEG	NEG	POS	AMB	NEG	POS	NEG
07V14047	NEG	POS		AMB	POS		AMB			POS	POS		POS	NEG	NEG	NEG	NEG	POS	POS	NEG	POS	NEG
07V22331	NEG	POS		POS	POS		POS			POS	POS		POS	NEG	NEG	NEG	NEG	POS	POS	POS	POS	NEG
07V23848	NEG	POS		POS	POS		AMB			POS	POS		POS	NEG	NEG	NEG	NEG	POS	POS	AMB	POS	NEG
07V25349	NEG	POS		POS	POS		NEG			POS	POS		POS	NEG	NEG	NEG	NEG	POS	POS	POS	POS	NEG
07V29952	NEG	POS		NEG	POS		POS			POS	POS		POS	NEG	NEG	NEG	NEG	POS	POS	NEG	POS	NEG
07V32172	NEG	POS		AMB	POS		POS			POS	POS		POS	NEG	NEG	NEG	NEG	POS	POS	NEG	POS	NEG
07V34596	NEG	POS		AMB	POS		POS			POS	POS		POS	NEG	NEG	NEG	NEG	POS	POS	NEG	POS	NEG
07V34637	NEG	POS		AMB	POS		POS			POS	POS		POS	NEG	NEG	NEG	NEG	POS	POS	NEG	POS	NEG
07V41577	NEG	POS		POS	POS		POS			POS	POS		POS	NEG	NEG	NEG	NEG	POS	POS	POS	POS	NEG
07V43890	NEG	POS		POS	POS		POS			POS	POS		POS	NEG	NEG	NEG	NEG	POS	POS	POS	POS	NEG
07V44597	NEG	POS		POS	POS		POS			POS	POS		POS	NEG	NEG	AMB	NEG	POS	POS	POS	POS	NEG
07V48218	NEG	POS	POS	POS	POS	NEG	POS	POS	NEG	POS	POS	AMB	POS	NEG	NEG	AMB	NEG	POS	POS	POS	POS	NEG
07V48369	NEG	POS		POS	POS		POS			POS	POS		POS	NEG	NEG	AMB	NEG	POS	POS	POS	POS	NEG
07V50153	NEG	POS		POS	AMB		AMB			POS	POS		POS	NEG	NEG	NEG	NEG	POS	POS	NEG	POS	NEG
07V31319	NEG	POS	POS	AMB	POS	NEG	POS	POS	NEG	POS	POS	AMB	POS	NEG	NEG	NEG	NEG	POS	POS	POS	POS	NEG
07V31590	NEG	NEG		NEG	NEG		POS			POS	POS		POS	NEG	NEG	NEG	NEG	POS	POS	POS	POS	NEG
07V31964	NEG	NEG		NEG	NEG		POS			POS	POS			NEG	NEG	NEG	NEG	POS	POS	AMB	POS	NEG
07V33137	NEG	NEG	NEG	NEG	NEG	NEG	POS	NEG	NEG	POS	POS	POS	POS	NEG	NEG	NEG	NEG	POS	POS	AMB	POS	NEG
07V45114	NEG	NEG	NEG	NEG	NEG	NEG	POS	NEG	NEG	POS	POS	POS	POS	NEG	NEG	AMB	NEG	POS	POS	POS	POS	NEG
07B06644	NEG	POS		NEG	NEG		NEG			NEG	NEG		NEG	NEG	NEG	NEG	NEG	NEG	NEG	NEG	POS	NEG
07F03422	NEG	POS		POS	POS		NEG			NEG	NEG		NEG	NEG	NEG	NEG	NEG	NEG	NEG	POS	POS	NEG
07F03905	NEG	POS		NEG	POS		NEG			NEG	NEG		NEG	NEG	NEG	NEG	NEG	NEG	NEG	NEG	POS	NEG
07F06025	NEG	POS		POS	POS		NEG			POS	NEG		POS	NEG	NEG	NEG	NEG	NEG	NEG	POS	POS	NEG
07F06053	NEG	POS		POS	POS		NEG			NEG	NEG		NEG	NEG	NEG	NEG	NEG	NEG	NEG	AMB	POS	NEG
07F06432	NEG	POS		POS	POS		NEG			NEG	NEG		AMB	NEG	NEG	NEG	NEG	NEG	NEG	NEG	POS	NEG
07U08245	NEG	POS		POS	POS		NEG			NEG	NEG		NEG	NEG	NEG	NEG	NEG	NEG	NEG	POS	POS	NEG
07V01577	NEG	POS	AMB	POS	POS	NEG	NEG	POS	NEG	NEG	NEG	POS	NEG	NEG	NEG	NEG	NEG	NEG	NEG	NEG	POS	NEG
07V02929	POS	POS		NEG	POS		NEG			NEG	NEG		NEG	NEG	NEG	NEG	NEG	NEG	NEG	NEG	POS	NEG
07V03947	NEG	POS		POS	POS		NEG			NEG	NEG		NEG	NEG	NEG	NEG	NEG	NEG	NEG	AMB	POS	NEG
07V07685	NEG	POS		POS	POS		NEG			AMB	NEG		AMB	NEG	NEG	NEG	NEG	NEG	NEG	POS	POS	NEG
07V09789	POS	POS		POS	POS		NEG			NEG	NEG		NEG	NEG	NEG	NEG	NEG	NEG	NEG	NEG	POS	NEG
07V11100	POS	POS	POS	POS	POS	NEG	NEG	POS	NEG	AMB	NEG	POS	NEG	NEG	NEG	NEG	NEG	NEG	NEG	POS	POS	NEG
07V13886	NEG	POS		AMB	AMB		NEG			NEG	NEG		NEG	NEG	NEG	NEG	NEG	NEG	NEG	NEG	POS	NEG
07V13922	NEG	POS		AMB	AMB		NEG			NEG	NEG		NEG	NEG	NEG	NEG	NEG	NEG	NEG	NEG	POS	NEG
07V15787	NEG	POS		POS	POS		NEG			AMB	NEG		POS	NEG	NEG	NEG	NEG	NEG	NEG	AMB	POS	NEG
07V16040	NEG	POS		NEG	NEG		NEG			NEG	NEG		NEG	NEG	NEG	NEG	NEG	NEG	NEG	NEG	AMB	NEG
07V19106	NEG	POS		NEG	NEG		NEG			NEG	NEG		NEG	NEG	NEG	NEG	NEG	NEG	NEG	NEG	POS	NEG
07V19909	NEG	POS		NEG	POS		NEG			NEG	NEG		NEG	NEG	NEG	NEG	NEG	NEG	NEG	NEG	POS	NEG
07V20220	NEG	POS		NEG	NEG		NEG			NEG	NEG		NEG	NEG	NEG	NEG	NEG	NEG	NEG	NEG	POS	NEG
07V21255	NEG	POS		NEG	POS		NEG			NEG	NEG		NEG	NEG	NEG	NEG	NEG	NEG	NEG	NEG	POS	NEG
07V22221	AMB	POS		NEG	NEG		NEG			NEG	NEG		NEG	NEG	NEG	NEG	NEG	NEG	NEG	NEG	POS	NEG
07V23398	NEG	POS		POS	POS		NEG			NEG	NEG		NEG	NEG	NEG	NEG	NEG	NEG	NEG	NEG	POS	NEG
07V26255	NEG	POS		NEG	AMB		NEG			NEG	NEG		NEG	NEG	NEG	NEG	NEG	NEG	NEG	NEG	POS	NEG
07V27224	NEG	POS		NEG	NEG		NEG			NEG	NEG		NEG	NEG	NEG	NEG	NEG	NEG	NEG	NEG	POS	NEG
07V29579	NEG	POS		POS	POS		NEG			NEG	NEG		NEG	NEG	NEG	NEG	NEG	NEG	NEG	NEG	POS	NEG
07V30685	POS	POS		POS	POS		NEG			NEG	NEG		AMB	NEG	NEG	NEG	NEG	NEG	NEG	NEG	POS	NEG
07V30827	NEG	POS		POS	POS		NEG			NEG	NEG		NEG	NEG	NEG	NEG	NEG	NEG	NEG	AMB	POS	NEG
07V31586	NEG	POS		AMB	POS		NEG			NEG	NEG		NEG	NEG	NEG	NEG	NEG	NEG	NEG	NEG	POS	NEG
07V31588	NEG	POS		POS	POS		NEG			NEG	NEG		NEG	NEG	NEG	NEG	NEG	NEG	NEG	AMB	POS	NEG
07V32055	NEG	POS		AMB	POS		NEG			NEG	NEG		NEG	NEG	NEG	NEG	NEG	NEG	NEG	NEG	POS	NEG
07V32148	NEG	POS		AMB	POS		NEG			NEG	NEG		NEG	NEG	NEG	NEG	NEG	NEG	NEG	NEG	POS	NEG
07V32390	NEG	POS		POS	POS		NEG			NEG	NEG		NEG	NEG	NEG	NEG	NEG	NEG	NEG	POS	POS	NEG
07V32454	NEG	POS		POS	POS		NEG			NEG	NEG		NEG	NEG	NEG	NEG	NEG	NEG	NEG	POS	POS	NEG
07V32457	NEG	POS	POS	POS	POS	NEG	NEG	POS	NEG	AMB	NEG	POS	NEG	NEG	NEG	NEG	NEG	NEG	NEG	AMB	POS	NEG
07V32467	NEG	POS		POS	POS		NEG			NEG	NEG		POS	NEG	NEG	NEG	NEG	NEG	NEG	AMB	POS	NEG
07V32765	NEG	POS		POS	POS		NEG			NEG	NEG		NEG	NEG	NEG	NEG	NEG	NEG	NEG	AMB	POS	NEG
07V33857	NEG	POS		POS	POS		NEG			NEG	NEG		NEG	NEG	NEG	NEG	NEG	NEG	NEG	AMB	POS	NEG
07V34379	NEG	POS		AMB	POS		NEG			NEG	NEG		NEG	NEG	NEG	NEG	NEG	NEG	NEG	NEG	POS	NEG
07V35110	NEG	POS		POS	POS		NEG			POS	NEG		AMB	NEG	NEG	NEG	NEG	NEG	NEG	POS	POS	NEG
07V35482	POS	POS		POS	POS		NEG			NEG	NEG		NEG	NEG	NEG	NEG	NEG	NEG	NEG	NEG	POS	NEG
07V35565	NEG	POS		POS	POS		NEG			NEG	NEG		AMB	NEG	NEG	NEG	NEG	NEG	NEG	AMB	POS	NEG
07V37700	NEG	POS		POS	POS		NEG			NEG	NEG		AMB	NEG	NEG	NEG	NEG	NEG	NEG	AMB	POS	NEG
07V41045	NEG	POS		NEG	AMB		NEG			NEG	NEG		NEG	NEG	NEG	NEG	NEG	NEG	NEG	NEG	POS	NEG
07V41051	NEG	POS		AMB	POS		NEG			NEG	NEG		NEG	NEG	NEG	NEG	NEG	NEG	NEG	NEG	POS	NEG
07V42673	POS	POS		POS	POS		NEG			NEG	NEG		NEG	NEG	NEG	NEG	NEG	NEG	NEG	AMB	POS	NEG
07V42842	NEG	POS		POS	POS		NEG			AMB	NEG		AMB	NEG	NEG	NEG	NEG	NEG	NEG	POS	POS	NEG
07V45346	NEG	POS		POS	POS		NEG			AMB	NEG		POS	NEG	NEG	NEG	NEG	NEG	NEG	POS	POS	NEG
07V46048	POS	POS		AMB	AMB		NEG			NEG	NEG		NEG	NEG	NEG	NEG	NEG	NEG	NEG	NEG	POS	NEG
07V46684	NEG	POS		POS	POS		NEG			POS	NEG		POS	NEG	NEG	NEG	NEG	NEG	NEG	POS	POS	NEG
07V49690	NEG	POS		POS	POS		NEG			POS	NEG		AMB	NEG	NEG	NEG	NEG	NEG	NEG	POS	POS	NEG
07V49998	POS	POS		POS	POS		NEG			POS	AMB		POS	NEG	NEG	NEG	NEG	NEG	NEG	POS	POS	NEG
07V25999	NEG	POS	POS	POS	POS	NEG	NEG	POS	NEG	POS	POS	POS	POS	POS	POS	NEG	NEG	NEG	NEG	POS	NEG	POS
07U07147	NEG	POS		AMB	POS		NEG			POS	NEG		POS	NEG	NEG	NEG	NEG	NEG	NEG	NEG	NEG	POS
07V03373	NEG	POS	POS	NEG	POS	NEG	NEG	POS	NEG	POS	NEG	POS	POS	NEG	NEG	NEG	NEG	NEG	NEG	AMB	NEG	POS
07V06916	NEG	POS		POS	POS		NEG			POS	NEG		POS	NEG	NEG	NEG	NEG	NEG	NEG	AMB	NEG	POS
07V16850	NEG	POS		POS	POS		NEG			POS	NEG		POS	NEG	NEG	NEG	NEG	NEG	NEG	NEG	NEG	POS
07V17786	NEG	POS		POS	AMB		NEG			POS	NEG		POS	NEG	NEG	NEG	NEG	NEG	NEG	AMB	NEG	POS
07V19855	POS	POS	POS	POS	POS	NEG	NEG	POS	NEG	POS	NEG	POS	POS	NEG	NEG	NEG	NEG	NEG	NEG	POS	NEG	POS
07V29398	NEG	POS	AMB	POS	POS	NEG	NEG	POS	NEG	POS	NEG	POS	POS	NEG	NEG	NEG	NEG	NEG	NEG	NEG	NEG	POS
07V40665	NEG	POS		POS	POS		NEG			POS	NEG		POS	NEG	NEG	NEG	NEG	NEG	NEG	NEG	NEG	POS
07V46779	NEG	POS	POS	POS	POS	NEG	NEG	POS	NEG	POS	NEG	POS	POS	NEG	NEG	NEG	NEG	NEG	NEG	POS	NEG	POS
07V48383	NEG	POS		POS	POS		NEG			POS	NEG		POS	NEG	NEG	NEG	NEG	NEG	NEG	POS	NEG	POS
07V25052	NEG	NEG	NEG	NEG	NEG	POS	NEG	NEG	NEG	POS	POS	AMB	POS	POS	POS	NEG	NEG	NEG	NEG	POS	POS	NEG
07V03944	NEG	NEG		NEG	NEG		NEG			POS	POS		POS	POS	POS	NEG	NEG	POS	POS	NEG	POS	NEG
07V34190	NEG	NEG	NEG	NEG	NEG	NEG	NEG	NEG	NEG	POS	POS	NEG	POS	NEG	NEG	NEG	NEG	POS	POS	AMB	POS	NEG

ISOLATE:	STRAIN	VIRULENCE : HAEMOLYSINS								VIRULENCE : HLB-CONV PHAGES			VIRULENCE : EXFOL.TOXINS			VIRULENCE : EPITHEL. DIFF. INHIB			VIRULENCE : ACME LOCUS				VIRULENCE : PROTEASES											VIRULENCE : STAPHYLOCOCCAL SUPERANTIGEN/ENTEROTOXIN-LIKE GENES (SET/SSL)						
		putative membrane protein	haemolysin alpha	putative membrane protein		haemolysin beta				staphylo-kinase	chemotaxis-inhibiting protein (CHIPS)	Staphyl. Comple-ment inhibitor	exfoliative toxin serotype A	exfoliative toxin serotype B	exfoliative toxin D	epidermal cell differen-tiation inhibitor	epidermal cell differen-tiation inhibitor B	epidermal cell differen-tiation inhibitor C	ACME-locus	ACME-locus: ornithincarb-amoyltrans-ferase	ACME-locus: carbamat-kinase	ACME-locus: arginine/orni-thine-antiporter	aureolysin			serin- protease A	serin- protease B	serin- protease E	glutamylendopeptidase	staphopain B, protease	staphopain A (staphylopain A), protease		staphyl. exotoxin-like protein	staphylococcal superantigen-like protein 1					
		hl	hla	hlIII (consensus)	hlIII (other than RF122)	hlb_probe 1	hlb_probe 2	hlb_probe 3	un-disrupted hlb	sak	chp	scn	etA	etB	etD	edinA	edinB	edinC	arcA-SCC	arcB-SCC	arcC-SCC	arcD-SCC	aur (consensus)	aur (other than MRSA252)	aur (MRSA252)	splA	splB	splE	sspA	sspB	sspP (consensus)	sspP (other than ST93)	setC	ssl01/set6_probe1_11	ssl01/set6_probe2_11	ssl01/set6_probe1_12	ssl01/set6_probe2_12	ssl01/set6_probe4_11	ssl01/set6_probeRF122
**2007**																																							
07V01446	CC1-MRSA-IV, WA MRSA-1/57	POS	POS	POS	POS	POS	POS	POS	NEG	POS	NEG	POS	NEG	NEG	NEG	NEG	NEG	NEG	NEG	NEG	NEG	NEG	POS	POS	NEG	POS	POS	POS	POS	POS	POS	POS	POS	NEG	POS	NEG	POS	NEG	NEG
07F01032	CC5/ST228-MRSA-I, South German EMRSA	POS	POS		POS				NEG	POS			NEG	NEG	NEG	NEG	NEG	NEG								POS	POS						POS	POS	NEG	POS	NEG	POS	
07V01588	CC5/ST228-MRSA-I, South German EMRSA	POS	POS		POS				NEG	POS			NEG	NEG	NEG	NEG	NEG	NEG								POS	POS						POS	POS	NEG	POS	NEG	POS	
07V01876	CC5/ST228-MRSA-I, South German EMRSA	POS	POS		POS				NEG	POS			NEG	NEG	NEG	NEG	NEG	NEG								POS	POS						POS	POS	NEG	POS	NEG	POS	
07V03020	CC5/ST228-MRSA-I, South German EMRSA	POS	POS		POS				NEG	POS			NEG	NEG	NEG	NEG	NEG	NEG								POS	POS						POS	POS	NEG	POS	NEG	POS	
07V03066	CC5/ST228-MRSA-I, South German EMRSA	POS	POS		POS				NEG	POS			NEG	NEG	NEG	NEG	NEG	NEG								POS	POS						POS	POS	NEG	POS	NEG	POS	
07V08865	CC5/ST228-MRSA-I, South German EMRSA	POS	POS	POS	POS	NEG	POS	NEG	NEG	POS	NEG	AMB	NEG	NEG	NEG	NEG	NEG	NEG	NEG	NEG	NEG	NEG	NEG	NEG	NEG	POS	POS	NEG	POS	POS	POS	AMB	POS	POS	NEG	POS	NEG	POS	NEG
07V35185	CC5/ST228-MRSA-I, South German EMRSA	POS	POS		POS				NEG	POS			NEG	NEG	NEG	NEG	NEG	NEG								POS	POS						POS	POS	NEG	POS	NEG	POS	
07V42548	CC5/ST228-MRSA-I, South German EMRSA	POS	POS	POS	POS	POS	POS	POS	NEG	POS	NEG	POS	NEG	NEG	NEG	NEG	NEG	NEG	NEG	NEG	NEG	NEG	POS	POS	NEG	POS	POS	NEG	POS	POS	POS	POS	POS	POS	NEG	POS	NEG	POS	NEG
07V47082	CC5/ST228-MRSA-I, South German EMRSA	POS	POS	POS	POS	POS	POS	POS	POS	POS	NEG	POS	NEG	NEG	NEG	NEG	NEG	NEG	NEG	NEG	NEG	NEG	POS	POS	NEG	POS	POS	NEG	POS	POS	POS	POS	POS	POS	NEG	POS	NEG	POS	NEG
07V48389	CC5/ST228-MRSA-I, South German EMRSA	POS	POS		POS				NEG	POS			NEG	NEG	NEG	NEG	NEG	NEG								POS	POS						POS	POS	NEG	POS	NEG	POS	
07V49263	CC5/ST228-MRSA-I, South German EMRSA	POS	POS	POS	POS	POS	POS	POS	NEG	POS	NEG	POS	NEG	NEG	NEG	NEG	NEG	NEG	NEG	NEG	NEG	NEG	POS	POS	NEG	POS	POS	NEG	POS	POS	POS	POS	POS	POS	NEG	POS	NEG	POS	NEG
07V50349	CC5/ST228-MRSA-I, South German EMRSA	POS	POS		POS				NEG	POS			NEG	NEG	NEG	NEG	NEG	NEG								POS	POS						POS	POS	NEG	POS	NEG	POS	
07B05103	CC5-MRSA-II, Rhine-Hesse EMRSA/UK-EMRSA-3	POS	POS		POS				NEG	POS			NEG	NEG	NEG	NEG	NEG	NEG								POS	POS						POS	POS	NEG	POS	NEG	POS	
07F00721	CC5-MRSA-II, Rhine-Hesse EMRSA/UK-EMRSA-3	POS	POS		POS				NEG	POS			NEG	NEG	NEG	NEG	NEG	NEG								POS	POS						POS	POS	NEG	POS	NEG	POS	
07V00817	CC5-MRSA-II, Rhine-Hesse EMRSA/UK-EMRSA-3	POS	POS	POS	POS	NEG	POS	NEG	NEG	POS	POS	POS	NEG	NEG	NEG	NEG	NEG	NEG	NEG	NEG	NEG	NEG	AMB	POS	NEG	POS	POS	NEG	POS	POS	POS	POS	POS	POS	NEG	POS	NEG	POS	NEG
07V01111	CC5-MRSA-II, Rhine-Hesse EMRSA/UK-EMRSA-3	POS	POS		POS				AMB	POS			NEG	NEG	NEG	NEG	NEG	NEG								POS	POS						POS	POS	NEG	POS	NEG	POS	
07V01885	CC5-MRSA-II, Rhine-Hesse EMRSA/UK-EMRSA-3	POS	POS		POS				NEG	POS			NEG	NEG	NEG	NEG	NEG	NEG								POS	POS						POS	POS	NEG	POS	NEG	POS	
07V04946	CC5-MRSA-II, Rhine-Hesse EMRSA/UK-EMRSA-3	POS	POS		POS				NEG	POS			NEG	NEG	NEG	NEG	NEG	NEG								POS	POS						POS	POS	NEG	POS	NEG	POS	
07V05167	CC5-MRSA-II, Rhine-Hesse EMRSA/UK-EMRSA-3	POS	POS		POS				NEG	POS			NEG	NEG	NEG	NEG	NEG	NEG								POS	POS						POS	POS	NEG	POS	NEG	POS	
07V05703	CC5-MRSA-II, Rhine-Hesse EMRSA/UK-EMRSA-3	POS	AMB		AMB				NEG	AMB			NEG	NEG	NEG	NEG	NEG	NEG								POS	POS						POS	POS	NEG	POS	NEG	POS	
07V08418	CC5-MRSA-II, Rhine-Hesse EMRSA/UK-EMRSA-3	POS	POS		POS				NEG	POS			NEG	NEG	NEG	NEG	NEG	NEG								POS	POS						POS	POS	NEG	POS	NEG	POS	
07V09138	CC5-MRSA-II, Rhine-Hesse EMRSA/UK-EMRSA-3	POS	POS		POS				NEG	POS			NEG	NEG	NEG	NEG	NEG	NEG								POS	POS						POS	POS	NEG	POS	NEG	POS	
07V11143	CC5-MRSA-II, Rhine-Hesse EMRSA/UK-EMRSA-3	POS	AMB		AMB				NEG	AMB			NEG	NEG	NEG	NEG	NEG	NEG								POS	POS						POS	POS	NEG	POS	NEG	POS	
07V14047	CC5-MRSA-II, Rhine-Hesse EMRSA/UK-EMRSA-3	POS	POS		POS				NEG	POS			NEG	NEG	NEG	NEG	NEG	NEG								POS	POS						POS	POS	NEG	POS	NEG	POS	
07V22331	CC5-MRSA-II, Rhine-Hesse EMRSA/UK-EMRSA-3	POS	POS		POS				NEG	POS			NEG	NEG	NEG	NEG	NEG	NEG								POS	POS						POS	POS	NEG	POS	NEG	POS	
07V23848	CC5-MRSA-II, Rhine-Hesse EMRSA/UK-EMRSA-3	POS	POS		POS				NEG	POS			NEG	NEG	NEG	NEG	NEG	NEG								POS	POS						POS	POS	NEG	POS	NEG	POS	
07V25349	CC5-MRSA-II, Rhine-Hesse EMRSA/UK-EMRSA-3	POS	POS		POS				NEG	POS			NEG	NEG	NEG	NEG	NEG	NEG								POS	POS						POS	POS	NEG	POS	NEG	POS	
07V29952	CC5-MRSA-II, Rhine-Hesse EMRSA/UK-EMRSA-3	POS	POS		POS				NEG	POS			NEG	NEG	NEG	NEG	NEG	NEG								POS	POS						POS	POS	NEG	POS	NEG	POS	
07V32172	CC5-MRSA-II, Rhine-Hesse EMRSA/UK-EMRSA-3	POS	POS		POS				NEG	POS			NEG	NEG	NEG	NEG	NEG	NEG								POS	POS						POS						
07V34596	CC5-MRSA-II, Rhine-Hesse EMRSA/UK-EMRSA-3	POS	POS		POS				NEG	POS			NEG	NEG	NEG	NEG	NEG	NEG								POS	POS						POS						
07V34637	CC5-MRSA-II, Rhine-Hesse EMRSA/UK-EMRSA-3	POS	POS		POS				NEG	POS			NEG	NEG	NEG	NEG	NEG	NEG								POS	POS						POS	POS	NEG	POS	NEG	POS	
07V41577	CC5-MRSA-II, Rhine-Hesse EMRSA/UK-EMRSA-3	POS	POS		POS				NEG	POS			NEG	NEG	NEG	NEG	NEG	NEG								POS	POS						POS	POS	NEG	POS	NEG	POS	
07V43890	CC5-MRSA-II, Rhine-Hesse EMRSA/UK-EMRSA-3	POS	POS		POS				NEG	POS			NEG	NEG	NEG	NEG	NEG	NEG								POS	POS						POS	POS	NEG	POS	NEG	POS	
07V44597	CC5-MRSA-II, Rhine-Hesse EMRSA/UK-EMRSA-3	POS	POS		POS				NEG	POS			NEG	NEG	NEG	NEG	NEG	NEG								POS	POS						POS	POS	NEG	POS	NEG	POS	
07V48218	CC5-MRSA-II, Rhine-Hesse EMRSA/UK-EMRSA-3	POS	POS	POS	POS	POS	POS	AMB	NEG	POS	POS	POS	NEG	NEG	NEG	NEG	NEG	NEG	NEG	NEG	NEG	NEG	AMB	AMB	NEG	POS	POS	NEG	POS	POS	POS	POS	POS	POS	NEG	POS	NEG	POS	NEG
07V48369	CC5-MRSA-II, Rhine-Hesse EMRSA/UK-EMRSA-3	POS	POS		POS				NEG	POS			NEG	NEG	NEG	NEG	NEG	NEG								POS	POS						POS	POS	NEG	POS	NEG	POS	
07V50153	CC5-MRSA-II, Rhine-Hesse EMRSA/UK-EMRSA-3	POS	POS		POS				NEG	POS			NEG	NEG	NEG	NEG	NEG	NEG								POS	POS						POS						
07V31319	CC5-MRSA-IV, Paediatric clone	POS	POS	POS	POS	POS	POS	POS	NEG	POS	POS	POS	NEG	NEG	NEG	NEG	NEG	NEG	NEG	NEG	NEG	NEG	POS	POS	NEG	POS	POS	NEG	POS	POS	POS	POS	POS						NEG
07V31590	CC8-MRSA-IV, Lyon Clone/UK-EMRSA-2	POS	POS		POS				NEG	POS			NEG	NEG	NEG	NEG	NEG	NEG								POS	POS						POS						
07V31964	CC8-MRSA-IV, Lyon Clone/UK-EMRSA-2	POS	POS		POS				NEG	POS			NEG	NEG	NEG	NEG	NEG	NEG								POS	POS						POS						
07V33137	CC8-MRSA-IV, Lyon Clone/UK-EMRSA-2	POS	POS	POS	POS	POS	POS	POS	NEG	POS	NEG	POS	NEG	NEG	NEG	NEG	NEG	NEG	NEG	NEG	NEG	NEG	POS	POS	NEG	POS	POS	POS	POS	POS	POS	POS	POS						NEG
07V45114	CC8-MRSA-IV, Lyon Clone/UK-EMRSA-2	POS	POS	POS	POS	POS	POS	POS	NEG	POS	NEG	POS	NEG	NEG	NEG	NEG	NEG	NEG	NEG	NEG	NEG	NEG	POS	POS	NEG	POS	POS	POS	POS	POS	POS	POS	POS						NEG
07B06644	CC22-MRSA-IV, Barnim/UK-EMRSA-15	POS	NEG		NEG				NEG	NEG			NEG	NEG	NEG	NEG	NEG	NEG								NEG	NEG						POS						
07F03422	CC22-MRSA-IV, Barnim/UK-EMRSA-15	POS	POS		NEG				NEG	POS			NEG	NEG	NEG	NEG	NEG	NEG								NEG	NEG						POS						
07F03905	CC22-MRSA-IV, Barnim/UK-EMRSA-15	POS	AMB		NEG				NEG	POS			NEG	NEG	NEG	NEG	NEG	NEG								NEG	NEG						POS						
07F06025	CC22-MRSA-IV, Barnim/UK-EMRSA-15	POS	POS		NEG				NEG	POS			NEG	NEG	NEG	NEG	NEG	NEG								NEG	NEG						POS						
07F06053	CC22-MRSA-IV, Barnim/UK-EMRSA-15	POS	POS		NEG				NEG	POS			NEG	NEG	NEG	NEG	NEG	NEG								NEG	NEG						POS						
07F06432	CC22-MRSA-IV, Barnim/UK-EMRSA-15	POS	POS		NEG				NEG	POS			NEG	NEG	NEG	NEG	NEG	NEG								NEG	NEG						POS						
07U08245	CC22-MRSA-IV, Barnim/UK-EMRSA-15	POS	POS		NEG				NEG	POS			NEG	NEG	NEG	NEG	NEG	NEG								NEG	NEG						POS						
07V01577	CC22-MRSA-IV, Barnim/UK-EMRSA-15	POS	POS	NEG	NEG	POS	POS	POS	NEG	POS	POS	POS	NEG	NEG	NEG	NEG	NEG	NEG	NEG	NEG	NEG	NEG	AMB	NEG	POS	NEG	NEG	NEG	POS	POS	POS	POS	POS						NEG
07V02929	CC22-MRSA-IV, Barnim/UK-EMRSA-15	POS	AMB		NEG				NEG	POS			NEG	NEG	NEG	NEG	NEG	NEG								NEG	NEG						POS						
07V03947	CC22-MRSA-IV, Barnim/UK-EMRSA-15	POS	POS		NEG				NEG	POS			NEG	NEG	NEG	NEG	NEG	NEG								NEG	NEG						POS						
07V07685	CC22-MRSA-IV, Barnim/UK-EMRSA-15	POS	POS		NEG				NEG	POS			NEG	NEG	NEG	NEG	NEG	NEG								NEG	NEG						POS						
07V09789	CC22-MRSA-IV, Barnim/UK-EMRSA-15	POS	POS		NEG				NEG	POS			NEG	NEG	NEG	NEG	NEG	NEG								NEG	NEG						POS						
07V11100	CC22-MRSA-IV, Barnim/UK-EMRSA-15	POS	POS	NEG	NEG	POS	POS	POS	NEG	POS	POS	POS	NEG	NEG	NEG	NEG	NEG	NEG	NEG	NEG	NEG	NEG	POS	NEG	POS	NEG	NEG	NEG	POS	POS	POS	POS	POS						NEG
07V13886	CC22-MRSA-IV, Barnim/UK-EMRSA-15	POS	NEG		NEG				NEG	POS			NEG	NEG	NEG	NEG	NEG	NEG								NEG	NEG						POS						
07V13922	CC22-MRSA-IV, Barnim/UK-EMRSA-15	POS	NEG		NEG				NEG	POS			NEG	NEG	NEG	NEG	NEG	NEG								NEG	NEG						POS						
07V15787	CC22-MRSA-IV, Barnim/UK-EMRSA-15	POS	POS		NEG				NEG	POS			NEG	NEG	NEG	NEG	NEG	NEG								NEG	NEG						POS						
07V16040	CC22-MRSA-IV, Barnim/UK-EMRSA-15	POS	NEG		NEG				NEG	AMB			NEG	NEG	NEG	NEG	NEG	NEG								NEG	NEG						POS						
07V19106	CC22-MRSA-IV, Barnim/UK-EMRSA-15	POS	NEG		NEG				NEG	AMB			NEG	NEG	NEG	NEG	NEG	NEG								NEG	NEG						POS						
07V19909	CC22-MRSA-IV, Barnim/UK-EMRSA-15	POS	NEG		NEG				NEG	POS			NEG	NEG	NEG	NEG	NEG	NEG								NEG	NEG						POS						
07V20220	CC22-MRSA-IV, Barnim/UK-EMRSA-15	POS	NEG		NEG				NEG	POS			NEG	NEG	NEG	NEG	NEG	NEG								NEG	NEG						POS						
07V21255	CC22-MRSA-IV, Barnim/UK-EMRSA-15	POS	NEG		NEG				NEG	POS			NEG	NEG	NEG	NEG	NEG	NEG								NEG	NEG						POS						
07V22221	CC22-MRSA-IV, Barnim/UK-EMRSA-15	POS	NEG		NEG				NEG	AMB			NEG	NEG	NEG	NEG	NEG	NEG								NEG	NEG						POS						
07V23398	CC22-MRSA-IV, Barnim/UK-EMRSA-15	POS	AMB		NEG				NEG	POS			NEG	NEG	NEG	NEG	NEG	NEG								NEG	NEG						POS						
07V26255	CC22-MRSA-IV, Barnim/UK-EMRSA-15	POS	NEG		NEG				NEG	POS			NEG	NEG	NEG	NEG	NEG	NEG								NEG	NEG						POS						
07V27224	CC22-MRSA-IV, Barnim/UK-EMRSA-15	POS	NEG		NEG				NEG	NEG			NEG	NEG	NEG	NEG	NEG	NEG								NEG	NEG						POS						
07V29579	CC22-MRSA-IV, Barnim/UK-EMRSA-15	POS	POS		NEG				NEG	POS			NEG	NEG	NEG	NEG	NEG	NEG								NEG	NEG						POS						
07V30685	CC22-MRSA-IV, Barnim/UK-EMRSA-15	POS	POS		NEG				NEG	POS			NEG	NEG	NEG	NEG	NEG	NEG								NEG	NEG						POS						
07V30827	CC22-MRSA-IV, Barnim/UK-EMRSA-15	POS	POS		NEG				NEG	POS			NEG	NEG	NEG	NEG	NEG	NEG								NEG	NEG						POS						
07V31586	CC22-MRSA-IV, Barnim/UK-EMRSA-15	POS	POS		NEG				NEG	POS			NEG	NEG	NEG	NEG	NEG	NEG								NEG	NEG						POS						
07V31588	CC22-MRSA-IV, Barnim/UK-EMRSA-15	POS	POS		NEG				NEG	POS			NEG	NEG	NEG	NEG	NEG	NEG								NEG	NEG						POS						
07V32055	CC22-MRSA-IV, Barnim/UK-EMRSA-15	POS	POS		NEG				NEG	POS			NEG	NEG	NEG	NEG	NEG	NEG								NEG	NEG						POS						
07V32148	CC22-MRSA-IV, Barnim/UK-EMRSA-15	POS	POS		NEG				NEG	POS			NEG	NEG	NEG	NEG	NEG	NEG								NEG	NEG						POS						
07V32390	CC22-MRSA-IV, Barnim/UK-EMRSA-15	POS	POS		NEG				NEG	POS			NEG	NEG	NEG	NEG	NEG	NEG								NEG	NEG						POS						
07V32454	CC22-MRSA-IV, Barnim/UK-EMRSA-15	POS	POS		NEG				NEG	POS			NEG	NEG	NEG	NEG	NEG	NEG								NEG	NEG						POS						
07V32457	CC22-MRSA-IV, Barnim/UK-EMRSA-15	POS	POS	NEG	NEG	POS	POS	POS	NEG	POS	POS	POS	NEG	NEG	NEG	NEG	NEG	NEG	NEG	NEG	NEG	NEG	POS	NEG	POS	NEG	NEG	NEG	POS	POS	POS	POS	POS						NEG
07V32467	CC22-MRSA-IV, Barnim/UK-EMRSA-15	POS	POS		NEG				NEG	POS			NEG	NEG	NEG	NEG	NEG	NEG								NEG	NEG						POS						
07V32765	CC22-MRSA-IV, Barnim/UK-EMRSA-15	POS	POS		NEG				NEG	POS			NEG	NEG	NEG	NEG	NEG	NEG								NEG	NEG						POS						
07V33857	CC22-MRSA-IV, Barnim/UK-EMRSA-15	POS	POS		NEG				NEG	POS			NEG	NEG	NEG	NEG	NEG	NEG								NEG	NEG						POS						
07V34379	CC22-MRSA-IV, Barnim/UK-EMRSA-15	POS	POS		NEG				NEG	POS			NEG	NEG	NEG	NEG	NEG	NEG								NEG	NEG						POS						
07V35110	CC22-MRSA-IV, Barnim/UK-EMRSA-15	POS	POS		NEG				NEG	POS			NEG	NEG	NEG	NEG	NEG	NEG								NEG	NEG						POS						
07V35482	CC22-MRSA-IV, Barnim/UK-EMRSA-15	POS	POS		NEG				NEG	POS			NEG	NEG	NEG	NEG	NEG	NEG								NEG	NEG						POS						
07V35565	CC22-MRSA-IV, Barnim/UK-EMRSA-15	POS	POS		NEG				NEG	POS			NEG	NEG	NEG	NEG	NEG	NEG								NEG	NEG						POS						
07V37700	CC22-MRSA-IV, Barnim/UK-EMRSA-15	POS	POS		NEG				NEG	POS			NEG	NEG	NEG	NEG	NEG	NEG								NEG	NEG						POS						
07V41045	CC22-MRSA-IV, Barnim/UK-EMRSA-15	POS	NEG		NEG				NEG	POS			NEG	NEG	NEG	NEG	NEG	NEG								NEG	NEG						POS						
07V41051	CC22-MRSA-IV, Barnim/UK-EMRSA-15	POS	AMB		NEG				NEG	POS			NEG	NEG	NEG	NEG	NEG	NEG								NEG	NEG						POS						
07V42673	CC22-MRSA-IV, Barnim/UK-EMRSA-15	POS	AMB		NEG				NEG	POS			NEG	NEG	NEG	NEG	NEG	NEG								NEG	NEG						POS						
07V42842	CC22-MRSA-IV, Barnim/UK-EMRSA-15	POS	POS		NEG				POS	NEG			NEG	NEG	NEG	NEG	NEG	NEG								NEG	NEG						POS						
07V45346	CC22-MRSA-IV, Barnim/UK-EMRSA-15	POS	POS		NEG				NEG	POS			NEG	NEG	NEG	NEG	NEG	NEG								NEG	NEG						POS						
07V46048	CC22-MRSA-IV, Barnim/UK-EMRSA-15	POS	NEG		NEG				NEG	POS			NEG	NEG	NEG	NEG	NEG	NEG								NEG	NEG						POS						
07V46684	CC22-MRSA-IV, Barnim/UK-EMRSA-15	POS	POS		NEG				AMB	POS			NEG	NEG	NEG	NEG	NEG	NEG								NEG	NEG						POS						
07V49690	CC22-MRSA-IV, Barnim/UK-EMRSA-15	POS	POS		NEG				NEG	POS			NEG	NEG	NEG	NEG	NEG	NEG								NEG	NEG						POS						
07V49998	CC22-MRSA-IV, Barnim/UK-EMRSA-15	POS	POS		NEG				NEG	POS			NEG	NEG	NEG	NEG	NEG	NEG								NEG	NEG						POS						
07V25999	CC30-MRSA-IV [PVL+], Southwest Pacific Clone	POS	POS	POS	POS	POS	POS	POS	NEG	POS	POS	POS	NEG	NEG	NEG	NEG	NEG	NEG	NEG	NEG	NEG	NEG	POS	NEG	POS	NEG	NEG	POS	POS	POS	POS	POS	NEG						NEG
07U07147	CC45-MRSA-IV, Berlin EMRSA	POS	AMB		NEG				NEG	POS			NEG	NEG	NEG	NEG	NEG	NEG								NEG	NEG						NEG						
07V03373	CC45-MRSA-IV, Berlin EMRSA	POS	POS	POS	NEG	NEG	NEG	NEG	NEG	POS	POS	POS	NEG	NEG	NEG	NEG	NEG	NEG	NEG	NEG	NEG	NEG	NEG	NEG	POS	NEG	NEG	NEG	POS	POS	POS	POS	POS						NEG
07V06916	CC45-MRSA-IV, Berlin EMRSA	POS	POS		NEG				NEG	POS			NEG	NEG	NEG	NEG	NEG	NEG								NEG	NEG						AMB						
07V16850	CC45-MRSA-IV, Berlin EMRSA	POS	POS		NEG				NEG	POS			NEG	NEG	NEG	NEG	NEG	NEG								NEG	NEG						POS						
07V17786	CC45-MRSA-IV, Berlin EMRSA	AMB	NEG		NEG				NEG	POS			NEG	NEG	NEG	NEG	NEG	NEG								NEG	NEG						AMB						
07V19855	CC45-MRSA-IV, Berlin EMRSA	POS	POS	POS	NEG	NEG	NEG	NEG	NEG	POS	POS	POS	NEG	NEG	NEG	NEG	NEG	NEG	NEG	NEG	NEG	NEG	NEG	NEG	POS	NEG	NEG	NEG	POS	POS	POS	POS	POS						NEG
07V29398	CC45-MRSA-IV, Berlin EMRSA	POS	NEG	POS	NEG	NEG	NEG	NEG	NEG	POS	POS	POS	NEG	NEG	NEG	NEG	NEG	NEG	NEG	NEG	NEG	NEG	AMB	NEG	POS	NEG	NEG	NEG	POS	POS	POS	POS	AMB						NEG
07V40665	CC45-MRSA-IV, Berlin EMRSA	AMB	NEG		NEG				NEG	POS			NEG	NEG	NEG	NEG	NEG	NEG								NEG	NEG						AMB						
07V46779	CC45-MRSA-IV, Berlin EMRSA	POS	POS	POS	NEG	AMB	AMB	NEG	NEG	POS	POS	POS	NEG	NEG	NEG	NEG	NEG	NEG	NEG	NEG	NEG	NEG	AMB	NEG	POS	NEG	NEG	NEG	POS	POS	POS	POS	POS						NEG
07V48383	CC45-MRSA-IV, Berlin EMRSA	POS	POS		NEG				NEG	POS			NEG	NEG	NEG	NEG	NEG	NEG								NEG	NEG						POS						
07V25052	CC59-MRSA-V(T) [PVL+], Taiwan Clone	POS	POS	POS	NEG	NEG	NEG	NEG	POS	NEG	POS	POS	NEG	NEG	NEG	NEG	NEG	NEG	NEG	NEG	NEG	NEG	POS	AMB	NEG	NEG	NEG	NEG	POS	POS	POS	POS	POS						NEG
07V03944	CC80-MRSA-IV [PVL+], European caMRSA Clone	POS	POS		POS				NEG	POS			NEG	NEG	POS	NEG	POS	NEG								POS	POS						POS	NEG	NEG	NEG	NEG	POS	
07V34190	CC97-MRSA-IV, WA MRSA-54/63	POS	POS	POS	POS	POS	POS	POS	NEG	POS	NEG	POS	NEG	NEG	NEG	NEG	NEG	NEG	NEG	NEG	NEG	NEG	POS	POS	NEG	POS	POS	POS	POS	POS	POS	POS	POS						POS

ISOLATE:	STRAIN	VIRULENCE : STAPHYLOCOCCAL SUPERANTIGEN/ENTEROTOXIN-LIKE GENES (SET/SSL)																																	CAPSULE- AND BIOFILM-ASSOCIATED GENES																
		staphylococcal superantigen-like protein 2		staphylococcal superantigen-like protein 3			staphylococcal superantigen-like protein 4		staphylococcal superantigen-like protein 5				staphylococcal superantigen-like protein 6		staphylococcal superantigen-like protein 7			staphylococcal superantigen-like protein 8		staphylococcal superantigen-like protein 9			staphylococcal superantigen-like protein 10			staphylococcal superantigene-like protein 11				staphylococcal exotoxin-like protein, second locus					Capsule type 1	capsular poly-saccharide synthesis enzyme	O-antigen poly-merase	capsular poly-saccharide biosyn- thesis protein	Capsule type 5	capsular poly-saccharide synthesis enzyme	O-antigen poly-merase	capsular poly-saccharide biosyn- thesis protein	Capsule type 8	capsular poly-saccharide synthesis enzyme	capsular poly-saccharide biosyn- thesis protein	O-antigen poly-merase	capsular poly-saccharide biosyn- thesis protein	intercellular adhesion protein A	intercellular adhesion protein C	biofilm PIA synthesis protein D	surface protein involved in biofilm formation
		ssl02/set7	ssl02/set7 (MRSA252)	ssl03/set8_probe 1	ssl03/set8_probe 2	ssl03/set8 (MRSA252, SAR0424)	ssl04/set9	ssl04/set9 (MRSA252, SAR0425)	ssl05/set3_probe 1	ssl05/set3 (RF122, probe-611)	ssl05/set3_probe 2 (612)	ssl05/set3 (MRSA252)	ssl06/set21	ssl06 (NCTC8325+ MW2)	ssl07/set1	ssl07/set1 (MRSA252)	ssl07/set1 (AF188836)	ssl08/set12_probe 1	ssl08/set12_probe 2	ssl09/set5_probe 1	ssl09/set5_probe 2	ssl09/set5 (MRSA252)	ssl10/set4	ssl10 (RF122)	ssl10/set4 (MRSA252)	ssl11/set2 (COL)	ssl11+set2(Mu50+N315)	ssl11+set2(MW2+MSSA 476)	ssl11/set2 (MRSA252)	setB3	setB3 (MRSA252)	setB2	setB2 (MRSA252)	setB1	cap 1 (total)	capH1	capJ1	capK1	cap 5 (total)	capH5	capJ5	capK5	cap 8 (total)	capH8	capI8	capJ8	capK8	icaA	icaC	icaD	bap
**2007**																																																			
07V01446	CC1-MRSA-IV, WA MRSA-1/57	POS	AMB	POS	POS	NEG	POS	NEG	POS	AMB	POS	NEG	POS	POS	POS	AMB	AMB	POS	POS	POS	POS	NEG	POS	AMB	AMB	NEG	NEG	POS	NEG	POS	NEG	POS	NEG	POS	NEG	NEG	NEG	NEG	NEG	NEG	NEG	NEG	POS	POS	POS	POS	POS	POS	POS	POS	NEG
07F01032	CC5/ST228-MRSA-I, South German EMRSA	POS	NEG	POS		NEG	POS	NEG	POS			NEG	NEG		POS	AMB	NEG	POS		POS		NEG	POS		NEG	NEG	POS	NEG	NEG	POS	NEG	POS	NEG	POS																	
07V01588	CC5/ST228-MRSA-I, South German EMRSA	POS	NEG	POS		NEG	POS	NEG	POS			NEG	NEG		POS	AMB	NEG	POS		POS		NEG	POS		NEG	NEG	POS	NEG	NEG	POS	NEG	POS	NEG	POS																	
07V01876	CC5/ST228-MRSA-I, South German EMRSA	POS	AMB	POS		NEG	POS	NEG	POS			NEG	NEG		POS	AMB	AMB	POS		POS		NEG	POS		NEG	NEG	POS	NEG	NEG	POS	NEG	POS	NEG	POS																	
07V03020	CC5/ST228-MRSA-I, South German EMRSA	POS	NEG	POS		NEG	POS	NEG	POS			NEG	NEG		POS	AMB	NEG	POS		POS		NEG	POS		NEG	NEG	POS	NEG	NEG	POS	NEG	POS	NEG	POS																	
07V03066	CC5/ST228-MRSA-I, South German EMRSA	POS	NEG	POS		NEG	POS	NEG	POS			NEG	NEG		POS	AMB	AMB	POS		POS		NEG	POS		NEG	NEG	POS	NEG	NEG	POS	NEG	POS	NEG	POS																	
07V08865	CC5/ST228-MRSA-I, South German EMRSA	POS	NEG	POS	POS	NEG	POS	NEG	POS	NEG	POS	NEG	NEG	NEG	POS	AMB	NEG	POS	AMB	POS	NEG	NEG	POS	NEG	NEG	NEG	POS	NEG	NEG	POS	NEG	POS	NEG	POS	NEG	NEG	NEG	NEG	POS	POS	POS	POS	NEG	NEG	NEG	NEG	NEG	POS	AMB	AMB	NEG
07V35185	CC5/ST228-MRSA-I, South German EMRSA	POS	AMB	POS		NEG	POS	NEG	POS			NEG	NEG		POS	AMB	AMB	POS		POS		NEG	POS		NEG	NEG	POS	NEG	NEG	POS	NEG	POS	NEG	POS																	
07V42548	CC5/ST228-MRSA-I, South German EMRSA	POS	NEG	POS	POS	NEG	POS	NEG	POS	POS	AMB	NEG	NEG	NEG	POS	AMB	NEG	POS	POS	POS	POS	NEG	POS	POS	NEG	NEG	POS	NEG	NEG	POS	NEG	POS	NEG	POS	NEG	AMB	NEG	NEG	POS	POS	POS	POS	NEG	NEG	NEG	NEG	NEG	POS	POS	POS	NEG
07V47082	CC5/ST228-MRSA-I, South German EMRSA	POS	POS	POS	POS	NEG	POS	NEG	POS	AMB	POS	NEG	NEG	NEG	POS	AMB	AMB	POS	POS	POS	POS	NEG	POS	AMB	NEG	NEG	POS	NEG	NEG	POS	NEG	POS	NEG	POS	NEG	NEG	NEG	NEG	POS	POS	POS	POS	NEG	NEG	NEG	NEG	NEG	POS	POS	POS	NEG
07V48389	CC5/ST228-MRSA-I, South German EMRSA	POS	POS	POS		NEG	POS	NEG	POS			NEG	NEG		AMB	POS	AMB	POS		POS		NEG	NEG		POS	NEG	POS	NEG	NEG	POS	NEG	POS	NEG	POS																	
07V49263	CC5/ST228-MRSA-I, South German EMRSA	POS	AMB	POS	POS	NEG	POS	NEG	POS	AMB	POS	NEG	NEG	NEG	POS	AMB	NEG	POS	POS	POS	AMB	NEG	POS	AMB	NEG	NEG	POS	NEG	NEG	POS	NEG	POS	NEG	POS	NEG	NEG	NEG	NEG	POS	POS	POS	POS	NEG	NEG	NEG	NEG	NEG	POS	POS	POS	NEG
07V50349	CC5/ST228-MRSA-I, South German EMRSA	POS	POS	POS		NEG	POS	NEG	POS			NEG	NEG		POS	AMB	NEG	POS		POS		NEG	POS		NEG	NEG	POS	NEG	NEG	POS	NEG	POS	NEG	POS																	
07B05103	CC5-MRSA-II, Rhine-Hesse EMRSA/UK-EMRSA-3	POS	AMB	POS		NEG	POS	NEG	POS			NEG	NEG		POS	AMB	AMB	POS		POS		NEG	POS		NEG	NEG	POS	NEG	NEG	POS	NEG	POS	NEG	POS																	
07F00721	CC5-MRSA-II, Rhine-Hesse EMRSA/UK-EMRSA-3	POS	AMB	POS		NEG	POS	NEG	POS			NEG	NEG		POS	AMB	AMB	POS		POS		NEG	POS		NEG	NEG	POS	NEG	NEG	POS	NEG	POS	NEG	POS																	
07V00817	CC5-MRSA-II, Rhine-Hesse EMRSA/UK-EMRSA-3	POS	AMB	POS	POS	NEG	POS	NEG	POS	NEG	POS	NEG	NEG	NEG	POS	AMB	AMB	POS	POS	POS	NEG	NEG	POS	NEG	NEG	NEG	POS	NEG	NEG	POS	NEG	POS	NEG	POS	NEG	NEG	NEG	NEG	POS	POS	POS	POS	NEG	NEG	NEG	NEG	NEG	POS	POS	POS	NEG
07V01111	CC5-MRSA-II, Rhine-Hesse EMRSA/UK-EMRSA-3	POS	POS	POS		NEG	POS	NEG	POS			NEG	NEG		POS	AMB	AMB	POS		POS		NEG	NEG		POS	NEG	POS	NEG	NEG	POS	NEG	POS	NEG	AMB																	
07V01885	CC5-MRSA-II, Rhine-Hesse EMRSA/UK-EMRSA-3	POS	AMB	POS		NEG	POS	NEG	POS			NEG	NEG		POS	AMB	NEG	POS		POS		NEG	POS		NEG	NEG	POS	NEG	NEG	POS	NEG	POS	NEG	POS																	
07V04946	CC5-MRSA-II, Rhine-Hesse EMRSA/UK-EMRSA-3	POS	NEG	POS		NEG	POS	NEG	POS			NEG	NEG		POS	AMB	NEG	POS		POS		NEG	POS		NEG	NEG	POS	NEG	NEG	POS	NEG	POS	NEG	POS																	
07V05167	CC5-MRSA-II, Rhine-Hesse EMRSA/UK-EMRSA-3	POS	AMB	POS		NEG	POS	NEG	POS			NEG	NEG		POS	AMB	AMB	POS		POS		NEG	POS		NEG	NEG	POS	NEG	NEG	POS	NEG	POS	NEG	POS																	
07V05703	CC5-MRSA-II, Rhine-Hesse EMRSA/UK-EMRSA-3	POS	NEG	AMB		NEG	AMB	NEG	POS			NEG	NEG		POS	NEG	NEG	POS		NEG		NEG	POS		NEG	NEG	POS	NEG	NEG	POS	NEG	POS	NEG	POS																	
07V08418	CC5-MRSA-II, Rhine-Hesse EMRSA/UK-EMRSA-3	POS	AMB	POS		NEG	POS	NEG	POS			NEG	NEG		POS	AMB	AMB	POS		POS		NEG	POS		NEG	NEG	POS	NEG	NEG	POS	NEG	POS	NEG	POS																	
07V09138	CC5-MRSA-II, Rhine-Hesse EMRSA/UK-EMRSA-3	POS	POS	POS		NEG	POS	NEG	POS			NEG	NEG		POS	AMB	AMB	POS		POS		NEG	POS		NEG	NEG	POS	NEG	NEG	POS	NEG	POS	NEG	POS																	
07V11143	CC5-MRSA-II, Rhine-Hesse EMRSA/UK-EMRSA-3	POS	NEG	POS		NEG	AMB	NEG	POS			NEG	NEG		POS	NEG	NEG	AMB		NEG		NEG	POS		NEG	NEG	POS	NEG	NEG	AMB	NEG	POS	NEG	POS																	
07V14047	CC5-MRSA-II, Rhine-Hesse EMRSA/UK-EMRSA-3	POS	NEG	POS		NEG	POS	NEG	POS			NEG	NEG		POS	AMB	NEG	POS		POS		NEG	POS		NEG	NEG	POS	NEG	NEG	POS	NEG	POS	NEG	POS																	
07V22331	CC5-MRSA-II, Rhine-Hesse EMRSA/UK-EMRSA-3	POS	POS	POS		NEG	POS	NEG	POS			NEG	NEG		POS	AMB	AMB	POS		POS		NEG	POS		NEG	NEG	POS	NEG	NEG	POS	NEG	POS	NEG	POS																	
07V23848	CC5-MRSA-II, Rhine-Hesse EMRSA/UK-EMRSA-3	POS	AMB	POS		NEG	POS	NEG	POS			NEG	NEG		POS	AMB	NEG	POS		POS		NEG	POS		NEG	NEG	POS	NEG	NEG	POS	NEG	POS	NEG	POS																	
07V25349	CC5-MRSA-II, Rhine-Hesse EMRSA/UK-EMRSA-3	POS	POS	POS		NEG	POS	NEG	POS			NEG	NEG		POS	AMB	AMB	POS		POS		NEG	POS		NEG	NEG	POS	NEG	NEG	POS	NEG	POS	NEG	POS																	
07V29952	CC5-MRSA-II, Rhine-Hesse EMRSA/UK-EMRSA-3	POS	NEG	POS		NEG	POS	NEG	POS			NEG	NEG		POS	AMB	NEG	POS		POS		NEG	POS		NEG	NEG	POS	NEG	NEG	POS	NEG	POS	NEG	POS																	
07V32172	CC5-MRSA-II, Rhine-Hesse EMRSA/UK-EMRSA-3	POS	NEG	POS		NEG	POS	NEG	POS			NEG	NEG		POS	AMB	NEG	POS		POS		NEG	POS		NEG	NEG	POS	NEG	NEG	POS	NEG	POS	NEG	POS																	
07V34596	CC5-MRSA-II, Rhine-Hesse EMRSA/UK-EMRSA-3	POS	NEG	POS		NEG	POS	NEG	POS			NEG	NEG		POS	AMB	NEG	POS		POS		NEG	POS		NEG	NEG	POS	NEG	NEG	POS	NEG	POS	NEG	POS																	
07V34637	CC5-MRSA-II, Rhine-Hesse EMRSA/UK-EMRSA-3	POS	NEG	POS		NEG	POS	NEG	POS			NEG	NEG		POS	AMB	AMB	POS		POS		NEG	POS		NEG	NEG	POS	NEG	NEG	POS	NEG	POS	NEG	POS																	
07V41577	CC5-MRSA-II, Rhine-Hesse EMRSA/UK-EMRSA-3	POS	POS	POS		NEG	POS	NEG	POS			NEG	NEG		POS	AMB	AMB	POS		POS		NEG	POS		NEG	NEG	POS	NEG	NEG	POS	NEG	POS	NEG	POS																	
07V43890	CC5-MRSA-II, Rhine-Hesse EMRSA/UK-EMRSA-3	POS	POS	POS		NEG	POS	NEG	POS			NEG	NEG		POS	AMB	AMB	POS		POS		NEG	POS		NEG	NEG	POS	NEG	NEG	POS	NEG	POS	NEG	AMB																	
07V44597	CC5-MRSA-II, Rhine-Hesse EMRSA/UK-EMRSA-3	POS	POS	POS		NEG	POS	NEG	POS			NEG	NEG		POS	AMB	AMB	POS		POS		NEG	POS		NEG	NEG	POS	NEG	NEG	POS	NEG	POS	NEG	AMB																	
07V48218	CC5-MRSA-II, Rhine-Hesse EMRSA/UK-EMRSA-3	POS	POS	POS	POS	NEG	POS	NEG	POS	AMB	POS	NEG	NEG	NEG	POS	AMB	AMB	POS	POS	POS	AMB	NEG	NEG	NEG	POS	NEG	POS	NEG	NEG	POS	NEG	POS	NEG	AMB	NEG	NEG	NEG	NEG	POS	POS	POS	POS	NEG	NEG	NEG	NEG	NEG	POS	POS	POS	NEG
07V48369	CC5-MRSA-II, Rhine-Hesse EMRSA/UK-EMRSA-3	POS	POS	POS		NEG	POS	NEG	POS			NEG	NEG		AMB	POS	AMB	POS		POS		NEG	AMB		AMB	NEG	POS	NEG	NEG	POS	NEG	POS	NEG	POS																	
07V50153	CC5-MRSA-II, Rhine-Hesse EMRSA/UK-EMRSA-3	POS	NEG	POS		NEG	POS	NEG	POS			NEG	NEG		POS	AMB	NEG	POS		POS		NEG	POS		NEG	NEG	POS	NEG	NEG	POS	NEG	POS	NEG	POS																	
07V31319	CC5-MRSA-IV, Paediatric clone	POS	POS	POS	POS	NEG	POS	NEG	POS	AMB	POS	NEG	NEG	NEG	POS	AMB	AMB	POS	POS	POS	POS	NEG	AMB	AMB	POS	NEG	POS	NEG	NEG	POS	NEG	POS	NEG	POS	NEG	NEG	NEG	NEG	POS	POS	POS	POS	NEG	NEG	NEG	NEG	NEG	POS	POS	POS	NEG
07V31590	CC8-MRSA-IV, Lyon Clone/UK-EMRSA-2	POS	NEG	POS		NEG	POS	NEG	POS			NEG	NEG		POS	AMB	AMB	POS		POS		NEG	POS		NEG	POS	NEG	NEG	NEG	POS	NEG	POS	NEG	POS																	
07V31964	CC8-MRSA-IV, Lyon Clone/UK-EMRSA-2	POS	NEG	POS		NEG	POS	NEG	POS			NEG	NEG		POS	AMB	AMB	POS		POS		NEG	POS		NEG	POS	NEG	NEG	NEG	POS	NEG	POS	NEG	POS																	
07V33137	CC8-MRSA-IV, Lyon Clone/UK-EMRSA-2	POS	NEG	POS	POS	NEG	POS	NEG	POS	AMB	POS	NEG	NEG	POS	POS	AMB	AMB	POS	POS	POS	POS	NEG	POS	POS	NEG	POS	NEG	NEG	NEG	POS	NEG	POS	NEG	POS	NEG	AMB	NEG	NEG	POS	POS	POS	POS	NEG	NEG	NEG	NEG	NEG	POS	POS	POS	NEG
07V45114	CC8-MRSA-IV, Lyon Clone/UK-EMRSA-2	POS	AMB	POS	POS	NEG	NEG	NEG	POS	AMB	POS	NEG	NEG	POS	POS	AMB	AMB	POS	POS	POS	POS	NEG	NEG	NEG	POS	POS	NEG	NEG	NEG	POS	NEG	POS	NEG	POS	NEG	NEG	NEG	NEG	POS	POS	POS	POS	NEG	NEG	NEG	NEG	NEG	POS	POS	POS	NEG
07B06644	CC22-MRSA-IV, Barnim/UK-EMRSA-15	NEG	NEG	NEG		AMB	NEG	NEG	POS			NEG	NEG		NEG	NEG	POS	NEG		NEG		NEG	NEG		NEG	NEG	NEG	NEG	NEG	NEG	NEG	NEG	NEG	NEG																	
07F03422	CC22-MRSA-IV, Barnim/UK-EMRSA-15	POS	AMB	NEG		NEG	NEG	NEG	POS			POS	NEG		AMB	POS	AMB	NEG		NEG		NEG	NEG		NEG	NEG	NEG	NEG	NEG	NEG	NEG	NEG	NEG	NEG																	
07F03905	CC22-MRSA-IV, Barnim/UK-EMRSA-15	POS	NEG	NEG		NEG	NEG	NEG	POS			POS	NEG		NEG	AMB	POS	NEG		NEG		NEG	NEG		NEG	NEG	NEG	NEG	NEG	NEG	NEG	NEG	NEG	NEG																	
07F06025	CC22-MRSA-IV, Barnim/UK-EMRSA-15	POS	POS	NEG		POS	NEG	POS	POS			POS	NEG		AMB	AMB	POS	NEG		POS		NEG	NEG		NEG	NEG	NEG	NEG	NEG	NEG	NEG	NEG	NEG	NEG																	
07F06053	CC22-MRSA-IV, Barnim/UK-EMRSA-15	POS	POS	NEG		NEG	NEG	NEG	POS			POS	NEG		AMB	AMB	POS	NEG		NEG		NEG	NEG		NEG	NEG	NEG	NEG	NEG	NEG	NEG	NEG	NEG	NEG																	
07F06432	CC22-MRSA-IV, Barnim/UK-EMRSA-15	POS	POS	NEG		AMB	NEG	POS	POS			POS	NEG		AMB	AMB	POS	NEG		NEG		NEG	NEG		NEG	NEG	NEG	NEG	NEG	NEG	NEG	NEG	NEG	NEG																	
07U08245	CC22-MRSA-IV, Barnim/UK-EMRSA-15	POS	AMB	NEG		NEG	NEG	NEG	POS			POS	NEG		AMB	AMB	POS	NEG		NEG		NEG	NEG		NEG	NEG	NEG	NEG	NEG	NEG	NEG	NEG	NEG	NEG																	
07V01577	CC22-MRSA-IV, Barnim/UK-EMRSA-15	POS	POS	NEG	NEG	NEG	NEG	AMB	POS	NEG	NEG	POS	NEG	NEG	AMB	AMB	POS	NEG	NEG	NEG	NEG	NEG	NEG	NEG	NEG	NEG	NEG	NEG	NEG	NEG	NEG	NEG	NEG	NEG	NEG	NEG	NEG	NEG	POS	POS	POS	POS	NEG	NEG	NEG	NEG	NEG	POS	NEG	POS	NEG
07V02929	CC22-MRSA-IV, Barnim/UK-EMRSA-15	POS	POS	NEG		NEG	NEG	NEG	POS			POS	NEG		AMB	AMB	POS	NEG		NEG		NEG	NEG		NEG	NEG	NEG	NEG	NEG	NEG	NEG	NEG	NEG	NEG																	
07V03947	CC22-MRSA-IV, Barnim/UK-EMRSA-15	POS	POS	NEG		NEG	NEG	NEG	POS			POS	NEG		AMB	AMB	POS	NEG		NEG		NEG	NEG		NEG	NEG	NEG	NEG	NEG	NEG	NEG	NEG	NEG	NEG																	
07V07685	CC22-MRSA-IV, Barnim/UK-EMRSA-15	POS	POS	NEG		AMB	NEG	POS	POS			POS	NEG		AMB	POS	AMB	NEG		POS		NEG	NEG		POS	NEG	NEG	NEG	NEG	NEG	NEG	NEG	NEG	NEG																	
07V09789	CC22-MRSA-IV, Barnim/UK-EMRSA-15	POS	POS	NEG		AMB	NEG	AMB	POS			POS	NEG		AMB	AMB	POS	NEG		NEG		NEG	NEG		NEG	NEG	NEG	NEG	NEG	NEG	NEG	NEG	NEG	NEG																	
07V11100	CC22-MRSA-IV, Barnim/UK-EMRSA-15	POS	POS	NEG	NEG	AMB	NEG	POS	POS	POS	NEG	POS	NEG	AMB	AMB	AMB	POS	NEG	NEG	AMB	POS	NEG	NEG	NEG	NEG	NEG	NEG	NEG	NEG	NEG	NEG	NEG	NEG	NEG	NEG	NEG	NEG	NEG	POS	POS	POS	POS	NEG	NEG	NEG	NEG	NEG	POS	POS	POS	NEG
07V13886	CC22-MRSA-IV, Barnim/UK-EMRSA-15	NEG	NEG	NEG		NEG	NEG	NEG	POS			POS	NEG		NEG	AMB	POS	NEG		NEG		NEG	NEG		NEG	NEG	NEG	NEG	NEG	NEG	NEG	NEG	NEG	NEG																	
07V13922	CC22-MRSA-IV, Barnim/UK-EMRSA-15	NEG	NEG	NEG		NEG	NEG	NEG	POS			POS	NEG		NEG	AMB	POS	NEG		NEG		NEG	NEG		NEG	NEG	NEG	NEG	NEG	NEG	NEG	NEG	NEG	NEG																	
07V15787	CC22-MRSA-IV, Barnim/UK-EMRSA-15	POS	POS	NEG		POS	NEG	POS	POS			POS	NEG		AMB	AMB	POS	NEG		POS		NEG	NEG		NEG	NEG	NEG	NEG	NEG	NEG	NEG	NEG	NEG	NEG																	
07V16040	CC22-MRSA-IV, Barnim/UK-EMRSA-15	NEG	NEG	NEG		NEG	NEG	NEG	POS			NEG	NEG		NEG	NEG	POS	NEG		NEG		NEG	NEG		NEG	NEG	NEG	NEG	NEG	NEG	NEG	NEG	NEG	NEG																	
07V19106	CC22-MRSA-IV, Barnim/UK-EMRSA-15	NEG	NEG	NEG		NEG	NEG	NEG	POS			NEG	NEG		NEG	AMB	POS	NEG		NEG		NEG	NEG		NEG	NEG	NEG	NEG	NEG	NEG	NEG	NEG	NEG	NEG																	
07V19909	CC22-MRSA-IV, Barnim/UK-EMRSA-15	POS	POS	NEG		NEG	NEG	AMB	POS			POS	NEG		NEG	AMB	POS	NEG		NEG		NEG	NEG		NEG	NEG	NEG	NEG	NEG	NEG	NEG	NEG	NEG	NEG																	
07V20220	CC22-MRSA-IV, Barnim/UK-EMRSA-15	NEG	NEG	NEG		NEG	NEG	NEG	POS			AMB	NEG		NEG	AMB	POS	NEG		NEG		NEG	NEG		NEG	NEG	NEG	NEG	NEG	NEG	NEG	NEG	NEG	NEG																	
07V21255	CC22-MRSA-IV, Barnim/UK-EMRSA-15	POS	POS	NEG		AMB	NEG	AMB	POS			POS	NEG		NEG	AMB	POS	NEG		NEG		NEG	NEG		NEG	NEG	NEG	NEG	NEG	NEG	NEG	NEG	NEG	NEG																	
07V22221	CC22-MRSA-IV, Barnim/UK-EMRSA-15	NEG	NEG	NEG		AMB	NEG	NEG	POS			NEG	NEG		NEG	AMB	POS	NEG		NEG		NEG	NEG		NEG	NEG	NEG	NEG	NEG	NEG	NEG	NEG	NEG	NEG																	
07V23398	CC22-MRSA-IV, Barnim/UK-EMRSA-15	POS	POS	NEG		AMB	NEG	NEG	POS			POS	NEG		AMB	AMB	POS	NEG		NEG		NEG	NEG		NEG	NEG	NEG	NEG	NEG	NEG	NEG	NEG	NEG	NEG																	
07V26255	CC22-MRSA-IV, Barnim/UK-EMRSA-15	POS	NEG	NEG		NEG	NEG	AMB	POS			POS	NEG		NEG	AMB	POS	NEG		NEG		NEG	NEG		NEG	NEG	NEG	NEG	NEG	NEG	NEG	NEG	NEG	NEG																	
07V27224	CC22-MRSA-IV, Barnim/UK-EMRSA-15	NEG	NEG	NEG		NEG	NEG	NEG	POS			AMB	NEG		NEG	NEG	POS	NEG		NEG		NEG	NEG		NEG	NEG	NEG	NEG	NEG	NEG	NEG	NEG	NEG	NEG																	
07V29579	CC22-MRSA-IV, Barnim/UK-EMRSA-15	POS	POS	NEG		AMB	NEG	AMB	POS			POS	NEG		AMB	AMB	POS	NEG		NEG		NEG	NEG		NEG	NEG	NEG	NEG	NEG	NEG	NEG	NEG	NEG	NEG																	
07V30685	CC22-MRSA-IV, Barnim/UK-EMRSA-15	POS	POS	NEG		AMB	NEG	AMB	POS			POS	NEG		AMB	AMB	POS	NEG		NEG		NEG	NEG		NEG	NEG	NEG	NEG	NEG	NEG	NEG	NEG	NEG	NEG																	
07V30827	CC22-MRSA-IV, Barnim/UK-EMRSA-15	POS	POS	NEG		AMB	NEG	AMB	POS			POS	NEG		AMB	AMB	POS	NEG		NEG		NEG	NEG		NEG	NEG	NEG	NEG	NEG	NEG	NEG	NEG	NEG	NEG																	
07V31586	CC22-MRSA-IV, Barnim/UK-EMRSA-15	POS	POS	NEG		NEG	NEG	NEG	POS			POS	NEG		AMB	AMB	POS	NEG		NEG		NEG	NEG		NEG	NEG	NEG	NEG	NEG	NEG	NEG	NEG	NEG	NEG																	
07V31588	CC22-MRSA-IV, Barnim/UK-EMRSA-15	POS	POS	NEG		AMB	NEG	AMB	POS			POS	NEG		AMB	AMB	POS	NEG		NEG		NEG	NEG		NEG	NEG	NEG	NEG	NEG	NEG	NEG	NEG	NEG	NEG																	
07V32055	CC22-MRSA-IV, Barnim/UK-EMRSA-15	POS	POS	NEG		NEG	NEG	NEG	POS			POS	NEG		AMB	AMB	POS	NEG		NEG		NEG	NEG		NEG	NEG	NEG	NEG	NEG	NEG	NEG	NEG	NEG	NEG																	
07V32148	CC22-MRSA-IV, Barnim/UK-EMRSA-15	POS	POS	NEG		AMB	NEG	AMB	POS			POS	NEG		AMB	AMB	POS	NEG		NEG		NEG	NEG		NEG	NEG	NEG	NEG	NEG	NEG	NEG	NEG	NEG	NEG																	
07V32390	CC22-MRSA-IV, Barnim/UK-EMRSA-15	POS	POS	NEG		AMB	NEG	AMB	POS			POS	NEG		AMB	AMB	POS	NEG		NEG		NEG	NEG		NEG	NEG	NEG	NEG	NEG	NEG	NEG	NEG	NEG	NEG																	
07V32454	CC22-MRSA-IV, Barnim/UK-EMRSA-15	POS	POS	NEG		AMB	NEG	AMB	POS			POS	NEG		AMB	AMB	POS	NEG		NEG		NEG	NEG		NEG	NEG	NEG	NEG	NEG	NEG	NEG	NEG	NEG	NEG																	
07V32457	CC22-MRSA-IV, Barnim/UK-EMRSA-15	POS	AMB	NEG	NEG	NEG	NEG	POS	POS	NEG	NEG	POS	NEG	NEG	AMB	AMB	POS	NEG	NEG	NEG	NEG	NEG	NEG	NEG	NEG	NEG	NEG	NEG	NEG	NEG	NEG	NEG	NEG	NEG	NEG	NEG	NEG	NEG	POS	POS	POS	POS	NEG	NEG	NEG	NEG	NEG	POS	AMB	POS	NEG
07V32467	CC22-MRSA-IV, Barnim/UK-EMRSA-15	POS	POS	NEG		POS	NEG	POS	POS			POS	NEG		AMB	AMB	POS	NEG		NEG		NEG	NEG		NEG	NEG	NEG	NEG	NEG	NEG	NEG	NEG	NEG	NEG																	
07V32765	CC22-MRSA-IV, Barnim/UK-EMRSA-15	POS	POS	NEG		AMB	NEG	POS	POS			POS	NEG		AMB	AMB	POS	NEG		NEG		NEG	NEG		NEG	NEG	NEG	NEG	NEG	NEG	NEG	NEG	NEG	NEG																	
07V33857	CC22-MRSA-IV, Barnim/UK-EMRSA-15	POS	POS	NEG		POS	NEG	POS	POS			POS	NEG		AMB	AMB	POS	NEG		NEG		NEG	NEG		NEG	NEG	NEG	NEG	NEG	NEG	NEG	NEG	NEG	NEG																	
07V34379	CC22-MRSA-IV, Barnim/UK-EMRSA-15	POS	POS	NEG		NEG	NEG	NEG	POS			POS	NEG		AMB	AMB	POS	NEG		NEG		NEG	NEG		NEG	NEG	NEG	NEG	NEG	NEG	NEG	NEG	NEG	NEG																	
07V35110	CC22-MRSA-IV, Barnim/UK-EMRSA-15	POS	POS	NEG		POS	NEG	POS	POS			POS	NEG		AMB	AMB	POS	NEG		AMB		NEG	NEG		NEG	NEG	NEG	NEG	NEG	NEG	NEG	NEG	NEG	NEG																	
07V35482	CC22-MRSA-IV, Barnim/UK-EMRSA-15	POS	NEG	NEG		NEG	NEG	NEG	POS			AMB	NEG		AMB	AMB	POS	NEG		NEG		NEG	NEG		NEG	NEG	NEG	NEG	NEG	NEG	NEG	NEG	NEG	NEG																	
07V35565	CC22-MRSA-IV, Barnim/UK-EMRSA-15	POS	POS	NEG		AMB	NEG	POS	POS			POS	NEG		AMB	AMB	POS	NEG		AMB		NEG	NEG		NEG	NEG	NEG	NEG	NEG	NEG	NEG	NEG	NEG	NEG																	
07V37700	CC22-MRSA-IV, Barnim/UK-EMRSA-15	POS	POS	NEG		AMB	NEG	POS	POS			POS	NEG		AMB	AMB	POS	NEG		AMB		NEG	NEG		NEG	NEG	NEG	NEG	NEG	NEG	NEG	NEG	NEG	NEG																	
07V41045	CC22-MRSA-IV, Barnim/UK-EMRSA-15	AMB	NEG	NEG		NEG	NEG	NEG	POS			POS	NEG		NEG	AMB	POS	NEG		NEG		NEG	NEG		NEG	NEG	NEG	NEG	NEG	NEG	NEG	NEG	NEG	NEG																	
07V41051	CC22-MRSA-IV, Barnim/UK-EMRSA-15	POS	POS	NEG		NEG	NEG	NEG	POS			POS	NEG		AMB	AMB	POS	NEG		NEG		NEG	NEG		NEG	NEG	NEG	NEG	NEG	NEG	NEG	NEG	NEG	NEG																	
07V42673	CC22-MRSA-IV, Barnim/UK-EMRSA-15	POS	POS	NEG		NEG	NEG	AMB	POS			POS	NEG		AMB	AMB	POS	NEG		NEG		NEG	NEG		NEG	NEG	NEG	NEG	NEG	NEG	NEG	NEG	NEG	NEG																	
07V42842	CC22-MRSA-IV, Barnim/UK-EMRSA-15	POS	POS	NEG		POS	NEG	POS	POS			POS	NEG		AMB	AMB	POS	NEG		POS		NEG	NEG		NEG	NEG	NEG	NEG	NEG	NEG	NEG	NEG	NEG	NEG																	
07V45346	CC22-MRSA-IV, Barnim/UK-EMRSA-15	POS	POS	NEG		POS	NEG	POS	POS			POS	NEG		AMB	POS	AMB	NEG		POS		NEG	NEG		POS	NEG	NEG	NEG	NEG	NEG	NEG	NEG	NEG	NEG																	
07V46048	CC22-MRSA-IV, Barnim/UK-EMRSA-15	NEG	AMB	NEG		NEG	NEG	NEG	POS			NEG	NEG		NEG	AMB	POS	NEG		AMB		NEG	NEG		NEG	NEG	NEG	NEG	NEG	NEG	NEG	NEG	NEG	NEG																	
07V46684	CC22-MRSA-IV, Barnim/UK-EMRSA-15	POS	POS	NEG		POS	NEG	POS	POS			POS	NEG		AMB	POS	AMB	NEG		POS		NEG	NEG		POS	NEG	NEG	NEG	NEG	NEG	NEG	NEG	NEG	NEG																	
07V49690	CC22-MRSA-IV, Barnim/UK-EMRSA-15	POS	POS	NEG		POS	NEG	POS	POS			POS	NEG		AMB	AMB	POS	NEG		NEG		NEG	NEG		NEG	NEG	NEG	NEG	NEG	NEG	NEG	NEG	NEG	NEG																	
07V49998	CC22-MRSA-IV, Barnim/UK-EMRSA-15	POS	POS	NEG		POS	NEG	POS	POS			POS	NEG		AMB	POS	AMB	NEG		POS		NEG	NEG		NEG	NEG	NEG	NEG	NEG	NEG	NEG	NEG	NEG	NEG																	
07V25999	CC30-MRSA-IV [PVL+], Southwest Pacific Clone	AMB	POS	NEG	NEG	POS	AMB	POS	NEG	NEG	NEG	POS	NEG	POS	NEG	POS	NEG	NEG	NEG	NEG	NEG	POS	NEG	NEG	POS	NEG	NEG	NEG	POS	NEG	POS	NEG	POS	POS	NEG	NEG	NEG	NEG	NEG	NEG	NEG	NEG	POS	POS	POS	POS	POS	POS	POS	POS	NEG
07U07147	CC45-MRSA-IV, Berlin EMRSA	NEG	POS	NEG		NEG	NEG	NEG	NEG			NEG	NEG		NEG	NEG	NEG	NEG		NEG		POS	NEG		POS	NEG	NEG	NEG	NEG	NEG	AMB	NEG	NEG	NEG																	
07V03373	CC45-MRSA-IV, Berlin EMRSA	NEG	POS	NEG	NEG	NEG	NEG	NEG	NEG	NEG	NEG	NEG	NEG	NEG	NEG	NEG	NEG	NEG	NEG	NEG	NEG	POS	NEG	NEG	POS	NEG	NEG	NEG	NEG	NEG	POS	NEG	NEG	NEG	NEG	NEG	NEG	NEG	NEG	NEG	NEG	NEG	POS	AMB	POS	POS	POS	POS	POS	POS	NEG
07V06916	CC45-MRSA-IV, Berlin EMRSA	NEG	POS	NEG		NEG	NEG	NEG	NEG			NEG	NEG		NEG	NEG	NEG	NEG		NEG		POS	NEG		POS	NEG	NEG	NEG	NEG	NEG	POS	NEG	NEG	NEG																	
07V16850	CC45-MRSA-IV, Berlin EMRSA	NEG	POS	NEG		NEG	NEG	POS	NEG			AMB	NEG		NEG	NEG	NEG	NEG		NEG		POS	NEG		POS	NEG	NEG	NEG	NEG	NEG	POS	NEG	NEG	NEG																	
07V17786	CC45-MRSA-IV, Berlin EMRSA	NEG	POS	NEG		NEG	NEG	NEG	NEG			AMB	NEG		NEG	NEG	NEG	NEG		NEG		POS	NEG		POS	NEG	NEG	NEG	NEG	NEG	POS	NEG	NEG	NEG																	
07V19855	CC45-MRSA-IV, Berlin EMRSA	AMB	POS	NEG	NEG	NEG	NEG	POS	NEG	NEG	NEG	POS	NEG	NEG	NEG	NEG	NEG	NEG	NEG	NEG	NEG	POS	NEG	NEG	POS	NEG	NEG	NEG	NEG	NEG	POS	NEG	AMB	POS	NEG	NEG	NEG	NEG	NEG	NEG	NEG	NEG	POS	POS	POS	POS	POS	POS	POS	POS	NEG
07V29398	CC45-MRSA-IV, Berlin EMRSA	NEG	POS	NEG	NEG	NEG	NEG	NEG	NEG	NEG	NEG	NEG	NEG	NEG	NEG	NEG	NEG	NEG	NEG	NEG	NEG	POS	NEG	NEG	POS	NEG	NEG	NEG	NEG	NEG	POS	NEG	NEG	NEG	NEG	NEG	NEG	NEG	NEG	NEG	NEG	NEG	POS	POS	POS	POS	POS	POS	POS	POS	NEG
07V40665	CC45-MRSA-IV, Berlin EMRSA	NEG	POS	NEG		NEG	NEG	NEG	NEG			NEG	NEG		NEG	NEG	NEG	NEG		NEG		POS	NEG		POS	NEG	NEG	NEG	NEG	NEG	POS	NEG	NEG	NEG																	
07V46779	CC45-MRSA-IV, Berlin EMRSA	POS	POS	NEG	NEG	NEG	NEG	POS	NEG	NEG	NEG	POS	NEG	POS	NEG	NEG	NEG	NEG	NEG	NEG	NEG	POS	NEG	NEG	POS	NEG	NEG	NEG	NEG	NEG	POS	NEG	POS	NEG	NEG	NEG	NEG	NEG	NEG	NEG	NEG	NEG	POS	POS	POS	POS	POS	POS	POS	POS	NEG
07V48383	CC45-MRSA-IV, Berlin EMRSA	POS	POS	NEG		NEG	NEG	POS	NEG			POS	NEG		NEG	NEG	NEG	NEG		NEG		POS	NEG		POS	NEG	NEG	NEG	NEG	NEG	POS	NEG	POS	NEG																	
07V25052	CC59-MRSA-V(T) [PVL+], Taiwan Clone	NEG	NEG	POS	POS	NEG	POS	NEG	POS	POS	NEG	NEG	NEG	NEG	POS	NEG	NEG	POS	POS	AMB	POS	NEG	POS	POS	NEG	NEG	NEG	NEG	NEG	POS	NEG	POS	NEG	POS	NEG	NEG	NEG	NEG	NEG	NEG	NEG	NEG	POS	POS	POS	POS	POS	POS	POS	POS	NEG
07V03944	CC80-MRSA-IV [PVL+], European caMRSA Clone	POS	NEG	NEG		NEG	POS	POS	POS			NEG	NEG		POS	AMB	NEG	POS		POS		NEG	POS		NEG	NEG	NEG	NEG	NEG	POS	NEG	POS	NEG	POS																	
07V34190	CC97-MRSA-IV, WA MRSA-54/63	POS	NEG	NEG	NEG	NEG	POS	NEG	POS	POS	NEG	NEG	NEG	NEG	POS	AMB	NEG	POS	POS	POS	POS	NEG	POS	NEG	NEG	NEG	NEG	NEG	NEG	POS	NEG	POS	NEG	POS	NEG	NEG	NEG	NEG	POS	POS	POS	POS	NEG	NEG	NEG	NEG	NEG	POS	POS	POS	NEG

ISOLATE:	STRAIN	ADHAESION FACTORS / GENES ENCODING MICROBIAL SURFACE COMPONENTS RECOGNIZING ADHESIVE MATRIX MOLECULES (MSCRAMM GENES)																																															
		bone sialoprotein-binding protein							clumping factor A					clumping factor B					collagen-binding adhesin	cell wall associated fibronectin-binding protein	cell surface elastin binding protein					enolase	fibrinogen binding protein (19 kDa)		fibronectin-binding protein A						fibronectin-binding protein B							Major histocompatibility complex class II analog protein (=Extracellular adherence protein, eap)				Staphylococcus aureus surface protein G			
		bbp (total)	bbp (consensus)	bbp (COL+MW2)	bbp (MRSA252)	bbp (Mu50)	bbp (RF122)	bbp (ST45)	clfA (total)	clfA (consensus)	clfA (COL+RF122)	clfA (MRSA252)	clfA (Mu50+MW2)	clfB (total)	clfB (consensus)	clfB (COL+Mu50)	clfB (MW2)	clfB (RF122)	cna	ebh (consensus)	ebpS (total)	ebpS_probe 612	ebpS_probe 614	ebpS (01-1111)	ebpS (COL)	eno	fib	fib (MRSA252)	fnbA (total)	fnbA (consensus)	fnbA (COL)	fnbA (MRSA252)	fnbA (Mu50+MW2)	fnbA (RF122)	fnbB (total)	fnbB (COL)	fnbB (COL+Mu50+MW2)	fnbB (Mu50)	fnbB (MW2)	fnbB (ST15)	fnbB (ST45-2)	map (total)	map (COL)	map (MRSA252)	map (Mu50+MW2)	sasG (total)	sasG (COL+Mu50)	sasG (MW2)	sasG (other than MRSA252+RF122)
**2007**																																																	
07V01446	CC1-MRSA-IV, WA MRSA-1/57	POS	POS	POS	NEG	NEG	NEG	NEG	POS	POS	NEG	NEG	POS	POS	POS	NEG	POS	AMB	POS	POS	POS	POS	POS	NEG	NEG	POS	POS	NEG	POS	POS	NEG	NEG	POS	NEG	POS	NEG	AMB	NEG	POS	NEG	NEG	POS	NEG	NEG	POS	POS	NEG	POS	POS
07F01032	CC5/ST228-MRSA-I, South German EMRSA																																																
07V01588	CC5/ST228-MRSA-I, South German EMRSA																																																
07V01876	CC5/ST228-MRSA-I, South German EMRSA																																																
07V03020	CC5/ST228-MRSA-I, South German EMRSA																																																
07V03066	CC5/ST228-MRSA-I, South German EMRSA																																																
07V08865	CC5/ST228-MRSA-I, South German EMRSA	NEG	AMB	NEG	NEG	AMB	NEG	NEG	POS	POS	NEG	NEG	POS	POS	POS	AMB	NEG	NEG	NEG	AMB	POS	AMB	POS	NEG	NEG	POS	POS	NEG	NEG	AMB	NEG	NEG	AMB	NEG	NEG	NEG	NEG	NEG	NEG	NEG	NEG	POS	NEG	NEG	POS	POS	POS	NEG	POS
07V35185	CC5/ST228-MRSA-I, South German EMRSA																																																
07V42548	CC5/ST228-MRSA-I, South German EMRSA	POS	POS	POS	NEG	POS	NEG	NEG	NEG	NEG	NEG	NEG	NEG	POS	POS	POS	NEG	NEG	NEG	POS	POS	POS	POS	NEG	POS	POS	POS	NEG	POS	POS	NEG	NEG	POS	NEG	NEG	NEG	NEG	NEG	NEG	NEG	NEG	POS	POS	AMB	POS	POS	POS	NEG	POS
07V47082	CC5/ST228-MRSA-I, South German EMRSA	POS	POS	AMB	NEG	POS	NEG	NEG	NEG	NEG	NEG	NEG	NEG	POS	POS	POS	NEG	NEG	NEG	POS	POS	POS	POS	NEG	NEG	POS	POS	AMB	POS	POS	NEG	NEG	POS	NEG	NEG	NEG	NEG	NEG	NEG	NEG	NEG	POS	POS	NEG	POS	POS	POS	NEG	POS
07V48389	CC5/ST228-MRSA-I, South German EMRSA																																																
07V49263	CC5/ST228-MRSA-I, South German EMRSA	POS	POS	NEG	NEG	POS	NEG	NEG	NEG	NEG	NEG	NEG	NEG	POS	POS	POS	NEG	NEG	NEG	POS	POS	POS	POS	NEG	NEG	POS	POS	NEG	POS	POS	NEG	NEG	POS	NEG	NEG	NEG	NEG	NEG	NEG	NEG	NEG	POS	AMB	NEG	POS	POS	POS	NEG	POS
07V50349	CC5/ST228-MRSA-I, South German EMRSA																																																
07B05103	CC5-MRSA-II, Rhine-Hesse EMRSA/UK-EMRSA-3																																																
07F00721	CC5-MRSA-II, Rhine-Hesse EMRSA/UK-EMRSA-3																																																
07V00817	CC5-MRSA-II, Rhine-Hesse EMRSA/UK-EMRSA-3	POS	POS	NEG	NEG	POS	NEG	NEG	POS	POS	NEG	NEG	POS	POS	POS	POS	NEG	NEG	NEG	POS	POS	AMB	POS	NEG	NEG	POS	POS	NEG	POS	POS	NEG	NEG	POS	NEG	POS	NEG	POS	POS	NEG	NEG	NEG	POS	NEG	NEG	POS	POS	POS	NEG	POS
07V01111	CC5-MRSA-II, Rhine-Hesse EMRSA/UK-EMRSA-3																																																
07V01885	CC5-MRSA-II, Rhine-Hesse EMRSA/UK-EMRSA-3																																																
07V04946	CC5-MRSA-II, Rhine-Hesse EMRSA/UK-EMRSA-3																																																
07V05167	CC5-MRSA-II, Rhine-Hesse EMRSA/UK-EMRSA-3																																																
07V05703	CC5-MRSA-II, Rhine-Hesse EMRSA/UK-EMRSA-3																																																
07V08418	CC5-MRSA-II, Rhine-Hesse EMRSA/UK-EMRSA-3																																																
07V09138	CC5-MRSA-II, Rhine-Hesse EMRSA/UK-EMRSA-3																																																
07V11143	CC5-MRSA-II, Rhine-Hesse EMRSA/UK-EMRSA-3																																																
07V14047	CC5-MRSA-II, Rhine-Hesse EMRSA/UK-EMRSA-3																																																
07V22331	CC5-MRSA-II, Rhine-Hesse EMRSA/UK-EMRSA-3																																																
07V23848	CC5-MRSA-II, Rhine-Hesse EMRSA/UK-EMRSA-3																																																
07V25349	CC5-MRSA-II, Rhine-Hesse EMRSA/UK-EMRSA-3																																																
07V29952	CC5-MRSA-II, Rhine-Hesse EMRSA/UK-EMRSA-3																																																
07V32172	CC5-MRSA-II, Rhine-Hesse EMRSA/UK-EMRSA-3																																																
07V34596	CC5-MRSA-II, Rhine-Hesse EMRSA/UK-EMRSA-3																																																
07V34637	CC5-MRSA-II, Rhine-Hesse EMRSA/UK-EMRSA-3																																																
07V41577	CC5-MRSA-II, Rhine-Hesse EMRSA/UK-EMRSA-3																																																
07V43890	CC5-MRSA-II, Rhine-Hesse EMRSA/UK-EMRSA-3																																																
07V44597	CC5-MRSA-II, Rhine-Hesse EMRSA/UK-EMRSA-3																																																
07V48218	CC5-MRSA-II, Rhine-Hesse EMRSA/UK-EMRSA-3	POS	POS	NEG	NEG	POS	NEG	NEG	POS	POS	NEG	NEG	POS	POS	POS	POS	NEG	NEG	NEG	POS	POS	POS	POS	NEG	NEG	POS	POS	NEG	POS	POS	NEG	NEG	POS	NEG	POS	NEG	AMB	POS	NEG	NEG	NEG	POS	AMB	NEG	POS	POS	POS	NEG	POS
07V48369	CC5-MRSA-II, Rhine-Hesse EMRSA/UK-EMRSA-3																																																
07V50153	CC5-MRSA-II, Rhine-Hesse EMRSA/UK-EMRSA-3																																																
07V31319	CC5-MRSA-IV, Paediatric clone	POS	POS	NEG	NEG	POS	NEG	NEG	POS	POS	NEG	NEG	POS	POS	POS	POS	NEG	NEG	NEG	POS	POS	POS	POS	NEG	NEG	POS	POS	NEG	POS	POS	NEG	NEG	POS	NEG	POS	NEG	POS	POS	NEG	NEG	NEG	POS	POS	NEG	POS	POS	POS	NEG	POS
07V31590	CC8-MRSA-IV, Lyon Clone/UK-EMRSA-2																																																
07V31964	CC8-MRSA-IV, Lyon Clone/UK-EMRSA-2																																																
07V33137	CC8-MRSA-IV, Lyon Clone/UK-EMRSA-2	POS	POS	POS	NEG	AMB	AMB	NEG	POS	POS	POS	POS	POS	POS	POS	POS	NEG	NEG	NEG	POS	POS	POS	POS	NEG	POS	POS	POS	NEG	POS	POS	POS	NEG	NEG	NEG	NEG	NEG	NEG	NEG	NEG	NEG	NEG	POS	POS	NEG	POS	POS	POS	NEG	POS
07V45114	CC8-MRSA-IV, Lyon Clone/UK-EMRSA-2	POS	POS	POS	NEG	NEG	NEG	NEG	POS	POS	POS	POS	POS	POS	POS	POS	NEG	NEG	NEG	POS	POS	POS	POS	NEG	POS	POS	POS	NEG	POS	POS	POS	NEG	NEG	NEG	NEG	NEG	NEG	NEG	NEG	NEG	NEG	POS	POS	NEG	AMB	POS	POS	NEG	POS
07B06644	CC22-MRSA-IV, Barnim/UK-EMRSA-15																																																
07F03422	CC22-MRSA-IV, Barnim/UK-EMRSA-15																																																
07F03905	CC22-MRSA-IV, Barnim/UK-EMRSA-15																																																
07F06025	CC22-MRSA-IV, Barnim/UK-EMRSA-15																																																
07F06053	CC22-MRSA-IV, Barnim/UK-EMRSA-15																																																
07F06432	CC22-MRSA-IV, Barnim/UK-EMRSA-15																																																
07U08245	CC22-MRSA-IV, Barnim/UK-EMRSA-15																																																
07V01577	CC22-MRSA-IV, Barnim/UK-EMRSA-15	POS	POS	NEG	NEG	NEG	NEG	NEG	POS	POS	NEG	NEG	POS	POS	POS	NEG	NEG	NEG	POS	NEG	POS	POS	POS	NEG	POS	POS	NEG	POS	POS	POS	NEG	NEG	POS	NEG	NEG	NEG	NEG	NEG	NEG	NEG	NEG	NEG	NEG	NEG	NEG	POS	NEG	POS	POS
07V02929	CC22-MRSA-IV, Barnim/UK-EMRSA-15																																																
07V03947	CC22-MRSA-IV, Barnim/UK-EMRSA-15																																																
07V07685	CC22-MRSA-IV, Barnim/UK-EMRSA-15																																																
07V09789	CC22-MRSA-IV, Barnim/UK-EMRSA-15																																																
07V11100	CC22-MRSA-IV, Barnim/UK-EMRSA-15	POS	POS	NEG	NEG	AMB	NEG	NEG	POS	POS	NEG	NEG	POS	POS	POS	AMB	NEG	POS	POS	NEG	POS	POS	POS	NEG	POS	POS	NEG	POS	POS	POS	NEG	NEG	POS	NEG	NEG	NEG	NEG	NEG	NEG	NEG	NEG	POS	NEG	NEG	POS	POS	NEG	POS	POS
07V13886	CC22-MRSA-IV, Barnim/UK-EMRSA-15																																																
07V13922	CC22-MRSA-IV, Barnim/UK-EMRSA-15																																																
07V15787	CC22-MRSA-IV, Barnim/UK-EMRSA-15																																																
07V16040	CC22-MRSA-IV, Barnim/UK-EMRSA-15																																																
07V19106	CC22-MRSA-IV, Barnim/UK-EMRSA-15																																																
07V19909	CC22-MRSA-IV, Barnim/UK-EMRSA-15																																																
07V20220	CC22-MRSA-IV, Barnim/UK-EMRSA-15																																																
07V21255	CC22-MRSA-IV, Barnim/UK-EMRSA-15																																																
07V22221	CC22-MRSA-IV, Barnim/UK-EMRSA-15																																																
07V23398	CC22-MRSA-IV, Barnim/UK-EMRSA-15																																																
07V26255	CC22-MRSA-IV, Barnim/UK-EMRSA-15																																																
07V27224	CC22-MRSA-IV, Barnim/UK-EMRSA-15																																																
07V29579	CC22-MRSA-IV, Barnim/UK-EMRSA-15																																																
07V30685	CC22-MRSA-IV, Barnim/UK-EMRSA-15																																																
07V30827	CC22-MRSA-IV, Barnim/UK-EMRSA-15																																																
07V31586	CC22-MRSA-IV, Barnim/UK-EMRSA-15																																																
07V31588	CC22-MRSA-IV, Barnim/UK-EMRSA-15																																																
07V32055	CC22-MRSA-IV, Barnim/UK-EMRSA-15																																																
07V32148	CC22-MRSA-IV, Barnim/UK-EMRSA-15																																																
07V32390	CC22-MRSA-IV, Barnim/UK-EMRSA-15																																																
07V32454	CC22-MRSA-IV, Barnim/UK-EMRSA-15																																																
07V32457	CC22-MRSA-IV, Barnim/UK-EMRSA-15	POS	POS	NEG	NEG	NEG	NEG	NEG	POS	POS	NEG	NEG	POS	POS	POS	NEG	NEG	POS	POS	NEG	POS	POS	POS	NEG	POS	POS	NEG	POS	POS	POS	NEG	NEG	POS	NEG	NEG	NEG	NEG	NEG	NEG	NEG	NEG	NEG	NEG	NEG	NEG	POS	NEG	POS	POS
07V32467	CC22-MRSA-IV, Barnim/UK-EMRSA-15																																																
07V32765	CC22-MRSA-IV, Barnim/UK-EMRSA-15																																																
07V33857	CC22-MRSA-IV, Barnim/UK-EMRSA-15																																																
07V34379	CC22-MRSA-IV, Barnim/UK-EMRSA-15																																																
07V35110	CC22-MRSA-IV, Barnim/UK-EMRSA-15																																																
07V35482	CC22-MRSA-IV, Barnim/UK-EMRSA-15																																																
07V35565	CC22-MRSA-IV, Barnim/UK-EMRSA-15																																																
07V37700	CC22-MRSA-IV, Barnim/UK-EMRSA-15																																																
07V41045	CC22-MRSA-IV, Barnim/UK-EMRSA-15																																																
07V41051	CC22-MRSA-IV, Barnim/UK-EMRSA-15																																																
07V42673	CC22-MRSA-IV, Barnim/UK-EMRSA-15																																																
07V42842	CC22-MRSA-IV, Barnim/UK-EMRSA-15																																																
07V45346	CC22-MRSA-IV, Barnim/UK-EMRSA-15																																																
07V46048	CC22-MRSA-IV, Barnim/UK-EMRSA-15																																																
07V46684	CC22-MRSA-IV, Barnim/UK-EMRSA-15																																																
07V49690	CC22-MRSA-IV, Barnim/UK-EMRSA-15																																																
07V49998	CC22-MRSA-IV, Barnim/UK-EMRSA-15																																																
07V25999	CC30-MRSA-IV [PVL+], Southwest Pacific Clone	POS	POS	NEG	POS	NEG	NEG	NEG	POS	POS	POS	POS	NEG	POS	POS	NEG	NEG	POS	POS	POS	POS	POS	POS	NEG	NEG	POS	NEG	POS	POS	POS	NEG	POS	NEG	NEG	POS	NEG	POS	POS	NEG	NEG	NEG	POS	NEG	POS	NEG	NEG	NEG	NEG	NEG
07U07147	CC45-MRSA-IV, Berlin EMRSA																																																
07V03373	CC45-MRSA-IV, Berlin EMRSA	POS	POS	NEG	NEG	NEG	NEG	POS	POS	POS	NEG	NEG	POS	POS	POS	NEG	POS	POS	POS	POS	POS	NEG	POS	AMB	NEG	POS	NEG	POS	POS	POS	NEG	NEG	POS	NEG	NEG	NEG	AMB	AMB	NEG	NEG	NEG	POS	NEG	POS	NEG	NEG	NEG	NEG	NEG
07V06916	CC45-MRSA-IV, Berlin EMRSA																																																
07V16850	CC45-MRSA-IV, Berlin EMRSA																																																
07V17786	CC45-MRSA-IV, Berlin EMRSA																																																
07V19855	CC45-MRSA-IV, Berlin EMRSA	POS	POS	NEG	NEG	NEG	NEG	POS	POS	POS	NEG	NEG	POS	POS	POS	NEG	POS	POS	POS	POS	POS	NEG	POS	AMB	NEG	POS	NEG	POS	POS	POS	NEG	NEG	POS	NEG	POS	NEG	POS	POS	NEG	NEG	NEG	POS	NEG	POS	NEG	NEG	NEG	NEG	NEG
07V29398	CC45-MRSA-IV, Berlin EMRSA	POS	POS	NEG	NEG	NEG	NEG	POS	POS	POS	AMB	NEG	POS	POS	POS	NEG	POS	POS	POS	POS	POS	NEG	POS	POS	NEG	POS	NEG	POS	POS	POS	NEG	NEG	POS	NEG	POS	NEG	POS	POS	NEG	NEG	NEG	POS	NEG	POS	NEG	NEG	NEG	NEG	NEG
07V40665	CC45-MRSA-IV, Berlin EMRSA																																																
07V46779	CC45-MRSA-IV, Berlin EMRSA	POS	POS	AMB	NEG	NEG	NEG	NEG	POS	POS	POS	NEG	POS	POS	POS	NEG	POS	POS	POS	POS	POS	NEG	POS	NEG	AMB	POS	NEG	POS	POS	POS	NEG	NEG	POS	NEG	POS	NEG	POS	POS	NEG	POS	NEG	POS	AMB	POS	AMB	NEG	NEG	NEG	NEG
07V48383	CC45-MRSA-IV, Berlin EMRSA																																																
07V25052	CC59-MRSA-V(T) [PVL+], Taiwan Clone	POS	POS	NEG	NEG	NEG	NEG	NEG	POS	POS	POS	NEG	POS	POS	POS	NEG	POS	POS	NEG	POS	POS	POS	AMB	NEG	POS	POS	POS	NEG	POS	POS	NEG	POS	NEG	NEG	POS	NEG	POS	AMB	NEG	NEG	NEG	POS	POS	NEG	NEG	POS	NEG	POS	POS
07V03944	CC80-MRSA-IV [PVL+], European caMRSA Clone																																																
07V34190	CC97-MRSA-IV, WA MRSA-54/63	NEG	NEG	NEG	NEG	NEG	NEG	NEG	POS	POS	POS	NEG	POS	NEG	AMB	NEG	NEG	NEG	NEG	POS	POS	POS	POS	NEG	NEG	POS	POS	NEG	POS	POS	NEG	NEG	NEG	NEG	POS	NEG	POS	POS	NEG	NEG	NEG	POS	POS	NEG	POS	POS	POS	NEG	POS

ISOLATE:	STRAIN	ADHAESION FACTORS / GENES ENCODING MICROBIAL SURFACE COMPONENTS RECOGNIZING ADHESIVE MATRIX MOLECULES (MSCRAMM GENES)																		IMMUNOD.ANTIGEN B		DEFENSIN RESIST.		TRANSFERRIN BINDING PROT			PUTATIVE TRANSPORTER		TYPE I RESTRICTION-MODIFICATION SYSTEM, SINGLE SEQUENCE SPECIFICITY PROTEIN												MISCELLANEOUS GENES			HYALURONATE LYASE							
		Ser-Asp rich fibrinogen-/bone sialoprotein-binding protein C							Ser-Asp rich fibrinogen-/bone sialoprotein-binding protein D					van Willebrand factor binding protein						immunodominant antigen B		defensin resistance protein		transferrin-binding protein			hypothetical protein, similar to integral membrane protein LmrP		type I site-specific deoxyribo-nuclease subunit, 1st locus	type I site-specific deoxyribonuclease subunit, 2nd locus			type I site-specific deoxyribonuclease subunit, 3rd locus					type I site-specific deoxyribonuclease subunit, unknown locus			hypothetical protein, located next to serine protease operon	Unspecific efflux/trans-porter	hypothetical protein	hyaluronate lyase, first / second locus			hyaluronate lyase, second locus				
		sdrC (total)	sdrC (consensus)	sdrC (B1)	sdrC (COL)	sdrC (Mu50)	sdrC (MW2+MRSA252+RF122)	sdrC (other than MRSA252+RF122)	sdrD (total)	sdrD (consensus)	sdrD (COL+MW2)	sdrD (Mu50)	sdrD (other)	vwb (total)	vwb (consensus)	vwb (COL+MW2)	vwb (MRSA252)	vwb (Mu50)	vwb (RF122)	isaB	isaB (MRSA252)	mprF (COL+MW2)	mprF (Mu50+MRSA252)	isdA (consensus)	isdA (MRSA252)	isdA (other than MRSA252)	lmrP (other than RF122)	lmrP (RF122)	hsdS1 (RF122)	hsdS2 (Mu50+N315+COL+USA300+NCTC8325)	hsdS2 (MW2+MSSA476)	hsdS2 (MRSA252)	hsdS3 (all other than RF122+MRSA252)	hsdS3 (COL+USA300+NCTC8325+MW2+MSSA476+RF122)	hsdS3 (Mu50+N315)	hsdS3 (CC51+MRSA252)	hsdS3 (MRSA252)	hsdSx (CC25)	hsdSx (CC15)	hsdSx (etd)	Q2FXC0	Q2YUB3	Q7A4X2	hysA1 (MRSA252)	hysA1 (MRSA252+RF122) and/or hysA2 (consensus)	hysA1 (MRSA252+RF122) and/or hysA2 (COL+USA300)	hysA2 (all other than MRSA252)	hysA2 (COL+USA300+NCTC8325)	hysA2 (all other than COL+USA300+NCTC8325)	hysA2 (all other than COL+USA300+NCTC8325)	hysA2 (MRSA252)
**2007**																																																			
07V01446	CC1-MRSA-IV, WA MRSA-1/57	POS	POS	NEG	NEG	NEG	POS	POS	POS	POS	POS	NEG	NEG	POS	POS	POS	NEG	NEG	NEG	POS	AMB	POS	AMB	POS	NEG	POS	POS	NEG	NEG	NEG	NEG	NEG	POS	POS	POS	NEG	NEG	POS	NEG	NEG	POS	NEG	NEG	NEG	POS	NEG	POS	NEG	POS	POS	NEG
07F01032	CC5/ST228-MRSA-I, South German EMRSA																																																		
07V01588	CC5/ST228-MRSA-I, South German EMRSA																																																		
07V01876	CC5/ST228-MRSA-I, South German EMRSA																																																		
07V03020	CC5/ST228-MRSA-I, South German EMRSA																																																		
07V03066	CC5/ST228-MRSA-I, South German EMRSA																																																		
07V08865	CC5/ST228-MRSA-I, South German EMRSA	POS	POS	NEG	NEG	POS	NEG	POS	POS	POS	NEG	NEG	NEG	POS	POS	NEG	NEG	POS	NEG	POS	NEG	NEG	NEG	AMB	NEG	AMB	POS	NEG	NEG	NEG	NEG	NEG	POS	NEG	POS	NEG	NEG	POS	NEG	NEG	NEG	NEG	NEG	NEG	POS	NEG	NEG	NEG	AMB	AMB	NEG
07V35185	CC5/ST228-MRSA-I, South German EMRSA																																																		
07V42548	CC5/ST228-MRSA-I, South German EMRSA	POS	POS	AMB	NEG	POS	POS	POS	POS	POS	NEG	POS	NEG	POS	POS	NEG	NEG	POS	NEG	POS	AMB	POS	AMB	POS	POS	POS	POS	NEG	NEG	NEG	NEG	NEG	POS	NEG	POS	AMB	NEG	POS	AMB	NEG	NEG	AMB	POS	NEG	POS	NEG	POS	NEG	POS	POS	NEG
07V47082	CC5/ST228-MRSA-I, South German EMRSA	POS	POS	NEG	NEG	POS	AMB	POS	POS	POS	NEG	POS	NEG	POS	POS	NEG	NEG	POS	NEG	POS	AMB	AMB	POS	POS	AMB	POS	POS	NEG	NEG	NEG	NEG	NEG	POS	NEG	POS	AMB	NEG	POS	NEG	NEG	NEG	NEG	POS	NEG	POS	NEG	POS	NEG	POS	POS	NEG
07V48389	CC5/ST228-MRSA-I, South German EMRSA																																																		
07V49263	CC5/ST228-MRSA-I, South German EMRSA	POS	POS	NEG	NEG	POS	NEG	POS	POS	POS	NEG	POS	NEG	POS	POS	NEG	NEG	POS	NEG	POS	AMB	AMB	POS	POS	AMB	POS	POS	NEG	NEG	NEG	NEG	NEG	POS	NEG	POS	NEG	NEG	POS	NEG	NEG	NEG	NEG	POS	NEG	POS	NEG	POS	NEG	POS	POS	NEG
07V50349	CC5/ST228-MRSA-I, South German EMRSA																																																		
07B05103	CC5-MRSA-II, Rhine-Hesse EMRSA/UK-EMRSA-3																																																		
07F00721	CC5-MRSA-II, Rhine-Hesse EMRSA/UK-EMRSA-3																																																		
07V00817	CC5-MRSA-II, Rhine-Hesse EMRSA/UK-EMRSA-3	POS	POS	NEG	NEG	POS	NEG	POS	POS	POS	NEG	POS	NEG	POS	POS	NEG	NEG	POS	NEG	POS	AMB	NEG	POS	POS	NEG	POS	POS	NEG	NEG	POS	NEG	NEG	POS	NEG	POS	NEG	NEG	POS	NEG	NEG	NEG	NEG	POS	NEG	POS	NEG	NEG	NEG	POS	POS	NEG
07V01111	CC5-MRSA-II, Rhine-Hesse EMRSA/UK-EMRSA-3																																																		
07V01885	CC5-MRSA-II, Rhine-Hesse EMRSA/UK-EMRSA-3																																																		
07V04946	CC5-MRSA-II, Rhine-Hesse EMRSA/UK-EMRSA-3																																																		
07V05167	CC5-MRSA-II, Rhine-Hesse EMRSA/UK-EMRSA-3																																																		
07V05703	CC5-MRSA-II, Rhine-Hesse EMRSA/UK-EMRSA-3																																																		
07V08418	CC5-MRSA-II, Rhine-Hesse EMRSA/UK-EMRSA-3																																																		
07V09138	CC5-MRSA-II, Rhine-Hesse EMRSA/UK-EMRSA-3																																																		
07V11143	CC5-MRSA-II, Rhine-Hesse EMRSA/UK-EMRSA-3																																																		
07V14047	CC5-MRSA-II, Rhine-Hesse EMRSA/UK-EMRSA-3																																																		
07V22331	CC5-MRSA-II, Rhine-Hesse EMRSA/UK-EMRSA-3																																																		
07V23848	CC5-MRSA-II, Rhine-Hesse EMRSA/UK-EMRSA-3																																																		
07V25349	CC5-MRSA-II, Rhine-Hesse EMRSA/UK-EMRSA-3																																																		
07V29952	CC5-MRSA-II, Rhine-Hesse EMRSA/UK-EMRSA-3																																																		
07V32172	CC5-MRSA-II, Rhine-Hesse EMRSA/UK-EMRSA-3																																																		
07V34596	CC5-MRSA-II, Rhine-Hesse EMRSA/UK-EMRSA-3																																																		
07V34637	CC5-MRSA-II, Rhine-Hesse EMRSA/UK-EMRSA-3																																																		
07V41577	CC5-MRSA-II, Rhine-Hesse EMRSA/UK-EMRSA-3																																																		
07V43890	CC5-MRSA-II, Rhine-Hesse EMRSA/UK-EMRSA-3																																																		
07V44597	CC5-MRSA-II, Rhine-Hesse EMRSA/UK-EMRSA-3																																																		
07V48218	CC5-MRSA-II, Rhine-Hesse EMRSA/UK-EMRSA-3	POS	POS	NEG	NEG	POS	NEG	POS	POS	POS	NEG	POS	NEG	POS	POS	NEG	NEG	POS	NEG	POS	AMB	AMB	POS	POS	NEG	POS	POS	NEG	NEG	POS	NEG	NEG	POS	NEG	POS	NEG	NEG	POS	NEG	NEG	NEG	NEG	POS	NEG	POS	NEG	POS	NEG	POS	POS	NEG
07V48369	CC5-MRSA-II, Rhine-Hesse EMRSA/UK-EMRSA-3																																																		
07V50153	CC5-MRSA-II, Rhine-Hesse EMRSA/UK-EMRSA-3																																																		
07V31319	CC5-MRSA-IV, Paediatric clone	POS	POS	NEG	NEG	POS	NEG	POS	POS	POS	NEG	POS	NEG	POS	POS	NEG	NEG	POS	NEG	POS	AMB	AMB	POS	POS	AMB	POS	POS	NEG	NEG	POS	NEG	NEG	POS	NEG	POS	NEG	NEG	POS	NEG	NEG	NEG	NEG	POS	NEG	POS	NEG	POS	NEG	POS	POS	NEG
07V31590	CC8-MRSA-IV, Lyon Clone/UK-EMRSA-2																																																		
07V31964	CC8-MRSA-IV, Lyon Clone/UK-EMRSA-2																																																		
07V33137	CC8-MRSA-IV, Lyon Clone/UK-EMRSA-2	POS	POS	NEG	POS	NEG	POS	POS	POS	POS	POS	NEG	NEG	POS	POS	POS	NEG	NEG	NEG	POS	AMB	AMB	POS	POS	POS	POS	POS	NEG	NEG	POS	NEG	NEG	POS	POS	NEG	AMB	NEG	POS	NEG	NEG	POS	AMB	NEG	NEG	POS	POS	POS	POS	NEG	NEG	POS
07V45114	CC8-MRSA-IV, Lyon Clone/UK-EMRSA-2	POS	POS	NEG	POS	NEG	NEG	POS	POS	POS	POS	NEG	NEG	POS	POS	POS	NEG	NEG	NEG	POS	AMB	POS	AMB	POS	NEG	POS	POS	NEG	NEG	POS	NEG	NEG	POS	POS	NEG	NEG	NEG	POS	NEG	AMB	POS	NEG	NEG	NEG	POS	POS	POS	POS	NEG	NEG	NEG
07B06644	CC22-MRSA-IV, Barnim/UK-EMRSA-15																																																		
07F03422	CC22-MRSA-IV, Barnim/UK-EMRSA-15																																																		
07F03905	CC22-MRSA-IV, Barnim/UK-EMRSA-15																																																		
07F06025	CC22-MRSA-IV, Barnim/UK-EMRSA-15																																																		
07F06053	CC22-MRSA-IV, Barnim/UK-EMRSA-15																																																		
07F06432	CC22-MRSA-IV, Barnim/UK-EMRSA-15																																																		
07U08245	CC22-MRSA-IV, Barnim/UK-EMRSA-15																																																		
07V01577	CC22-MRSA-IV, Barnim/UK-EMRSA-15	POS	POS	NEG	NEG	POS	NEG	POS	POS	POS	NEG	NEG	POS	POS	POS	NEG	NEG	NEG	POS	NEG	POS	AMB	POS	POS	NEG	POS	POS	NEG	NEG	POS	NEG	NEG	AMB	NEG	NEG	NEG	NEG	POS	NEG	NEG	NEG	NEG	AMB	NEG	POS	NEG	NEG	NEG	POS	POS	NEG
07V02929	CC22-MRSA-IV, Barnim/UK-EMRSA-15																																																		
07V03947	CC22-MRSA-IV, Barnim/UK-EMRSA-15																																																		
07V07685	CC22-MRSA-IV, Barnim/UK-EMRSA-15																																																		
07V09789	CC22-MRSA-IV, Barnim/UK-EMRSA-15																																																		
07V11100	CC22-MRSA-IV, Barnim/UK-EMRSA-15	POS	POS	NEG	NEG	POS	AMB	POS	POS	POS	NEG	NEG	POS	POS	POS	NEG	NEG	NEG	POS	NEG	POS	AMB	POS	POS	POS	POS	POS	NEG	NEG	POS	NEG	NEG	POS	NEG	NEG	NEG	NEG	POS	AMB	NEG	NEG	AMB	POS	NEG	POS	NEG	AMB	NEG	POS	POS	POS
07V13886	CC22-MRSA-IV, Barnim/UK-EMRSA-15																																																		
07V13922	CC22-MRSA-IV, Barnim/UK-EMRSA-15																																																		
07V15787	CC22-MRSA-IV, Barnim/UK-EMRSA-15																																																		
07V16040	CC22-MRSA-IV, Barnim/UK-EMRSA-15																																																		
07V19106	CC22-MRSA-IV, Barnim/UK-EMRSA-15																																																		
07V19909	CC22-MRSA-IV, Barnim/UK-EMRSA-15																																																		
07V20220	CC22-MRSA-IV, Barnim/UK-EMRSA-15																																																		
07V21255	CC22-MRSA-IV, Barnim/UK-EMRSA-15																																																		
07V22221	CC22-MRSA-IV, Barnim/UK-EMRSA-15																																																		
07V23398	CC22-MRSA-IV, Barnim/UK-EMRSA-15																																																		
07V26255	CC22-MRSA-IV, Barnim/UK-EMRSA-15																																																		
07V27224	CC22-MRSA-IV, Barnim/UK-EMRSA-15																																																		
07V29579	CC22-MRSA-IV, Barnim/UK-EMRSA-15																																																		
07V30685	CC22-MRSA-IV, Barnim/UK-EMRSA-15																																																		
07V30827	CC22-MRSA-IV, Barnim/UK-EMRSA-15																																																		
07V31586	CC22-MRSA-IV, Barnim/UK-EMRSA-15																																																		
07V31588	CC22-MRSA-IV, Barnim/UK-EMRSA-15																																																		
07V32055	CC22-MRSA-IV, Barnim/UK-EMRSA-15																																																		
07V32148	CC22-MRSA-IV, Barnim/UK-EMRSA-15																																																		
07V32390	CC22-MRSA-IV, Barnim/UK-EMRSA-15																																																		
07V32454	CC22-MRSA-IV, Barnim/UK-EMRSA-15																																																		
07V32457	CC22-MRSA-IV, Barnim/UK-EMRSA-15	POS	POS	NEG	NEG	POS	NEG	POS	POS	POS	NEG	NEG	POS	POS	POS	NEG	NEG	NEG	POS	NEG	POS	AMB	POS	POS	NEG	POS	POS	NEG	NEG	POS	NEG	NEG	POS	NEG	NEG	NEG	NEG	POS	NEG	NEG	NEG	NEG	POS	NEG	POS	NEG	NEG	NEG	POS	POS	NEG
07V32467	CC22-MRSA-IV, Barnim/UK-EMRSA-15																																																		
07V32765	CC22-MRSA-IV, Barnim/UK-EMRSA-15																																																		
07V33857	CC22-MRSA-IV, Barnim/UK-EMRSA-15																																																		
07V34379	CC22-MRSA-IV, Barnim/UK-EMRSA-15																																																		
07V35110	CC22-MRSA-IV, Barnim/UK-EMRSA-15																																																		
07V35482	CC22-MRSA-IV, Barnim/UK-EMRSA-15																																																		
07V35565	CC22-MRSA-IV, Barnim/UK-EMRSA-15																																																		
07V37700	CC22-MRSA-IV, Barnim/UK-EMRSA-15																																																		
07V41045	CC22-MRSA-IV, Barnim/UK-EMRSA-15																																																		
07V41051	CC22-MRSA-IV, Barnim/UK-EMRSA-15																																																		
07V42673	CC22-MRSA-IV, Barnim/UK-EMRSA-15																																																		
07V42842	CC22-MRSA-IV, Barnim/UK-EMRSA-15																																																		
07V45346	CC22-MRSA-IV, Barnim/UK-EMRSA-15																																																		
07V46048	CC22-MRSA-IV, Barnim/UK-EMRSA-15																																																		
07V46684	CC22-MRSA-IV, Barnim/UK-EMRSA-15																																																		
07V49690	CC22-MRSA-IV, Barnim/UK-EMRSA-15																																																		
07V49998	CC22-MRSA-IV, Barnim/UK-EMRSA-15																																																		
07V25999	CC30-MRSA-IV [PVL+], Southwest Pacific Clone	POS	POS	NEG	NEG	NEG	AMB	NEG	POS	POS	NEG	NEG	POS	POS	POS	NEG	POS	NEG	NEG	NEG	POS	AMB	POS	POS	POS	NEG	POS	NEG	NEG	NEG	NEG	POS	NEG	NEG	NEG	POS	POS	POS	NEG	NEG	NEG	NEG	POS	NEG	POS	POS	NEG	POS	POS	POS	POS
07U07147	CC45-MRSA-IV, Berlin EMRSA																																																		
07V03373	CC45-MRSA-IV, Berlin EMRSA	POS	POS	POS	NEG	NEG	NEG	NEG	POS	POS	NEG	NEG	NEG	POS	NEG	NEG	POS	NEG	NEG	NEG	POS	NEG	NEG	POS	POS	NEG	POS	NEG	NEG	NEG	NEG	AMB	NEG	NEG	NEG	NEG	NEG	NEG	NEG	NEG	NEG	NEG	NEG	NEG	NEG	NEG	NEG	NEG	NEG	NEG	NEG
07V06916	CC45-MRSA-IV, Berlin EMRSA																																																		
07V16850	CC45-MRSA-IV, Berlin EMRSA																																																		
07V17786	CC45-MRSA-IV, Berlin EMRSA																																																		
07V19855	CC45-MRSA-IV, Berlin EMRSA	POS	POS	POS	NEG	NEG	AMB	NEG	POS	POS	POS	NEG	NEG	POS	NEG	NEG	POS	NEG	NEG	NEG	POS	NEG	NEG	POS	POS	NEG	POS	NEG	NEG	NEG	NEG	POS	NEG	NEG	NEG	NEG	NEG	NEG	NEG	NEG	NEG	NEG	POS	NEG	NEG	NEG	NEG	NEG	POS	NEG	NEG
07V29398	CC45-MRSA-IV, Berlin EMRSA	POS	POS	POS	NEG	NEG	POS	NEG	POS	POS	POS	NEG	NEG	POS	AMB	NEG	POS	NEG	NEG	NEG	POS	NEG	NEG	POS	POS	AMB	POS	NEG	NEG	NEG	NEG	POS	NEG	NEG	NEG	NEG	NEG	POS	NEG	NEG	NEG	NEG	POS	NEG	POS	NEG	NEG	NEG	POS	NEG	NEG
07V40665	CC45-MRSA-IV, Berlin EMRSA																																																		
07V46779	CC45-MRSA-IV, Berlin EMRSA	POS	POS	POS	NEG	NEG	AMB	NEG	POS	POS	POS	NEG	NEG	POS	AMB	NEG	POS	NEG	NEG	NEG	POS	NEG	NEG	POS	POS	POS	POS	NEG	POS	NEG	NEG	POS	NEG	NEG	NEG	NEG	NEG	POS	NEG	NEG	NEG	NEG	POS	NEG	POS	NEG	NEG	NEG	POS	NEG	NEG
07V48383	CC45-MRSA-IV, Berlin EMRSA																																																		
07V25052	CC59-MRSA-V(T) [PVL+], Taiwan Clone	POS	POS	NEG	NEG	NEG	AMB	NEG	POS	POS	NEG	NEG	NEG	POS	POS	NEG	NEG	NEG	NEG	POS	NEG	AMB	NEG	POS	NEG	POS	NEG	POS	NEG	NEG	NEG	NEG	NEG	NEG	NEG	POS	NEG	NEG	POS	NEG	NEG	NEG	NEG	NEG	POS	NEG	NEG	NEG	POS	POS	NEG
07V03944	CC80-MRSA-IV [PVL+], European caMRSA Clone																																																		
07V34190	CC97-MRSA-IV, WA MRSA-54/63	POS	POS	NEG	NEG	POS	AMB	POS	NEG	NEG	NEG	NEG	NEG	POS	POS	NEG	NEG	NEG	POS	POS	AMB	POS	NEG	POS	NEG	POS	POS	NEG	NEG	NEG	NEG	NEG	NEG	NEG	NEG	NEG	NEG	POS	NEG	NEG	NEG	NEG	POS	NEG	POS	NEG	NEG	NEG	POS	POS	NEG

ISOLATE:	STRAIN	WARD	AGE	GENDER	SPECIES MARKER							REGULATORY GENES								METHICILLIN RESISTANCE AND SCCmec TYPING																									
					Ribosomal sequence from S. aureus	glyceraldehyde 3-phosphate dehydro-genase, locus 1	katalase A	coagulase	thermo-stable extracellular nuclease	Protein A	IgG-binding protein	staphylo-coccal accessory regulator A	histidine protein kinase, sae locus	sensor protein	accessory gene regulator allele I	accessory gene regulator allele II	accessory gene regulator allele III	accessory gene regulator allele IV	haemolysin delta	alternate penicillin binding protein 2, defining MRSA	truncated signal transducer protein MecR1	glycerophosphoryl diester phosphodi-esterase, associated with mecA	cassette chromosome recombinase genes A/B-1		plasmin-sensitive surface protein	hypothetical protein from SCCmec elements	cassette chromosome recombinase genes A/B-2		potassium-trans-locating ATPase A, chain 2	potassium-transporting ATPase B, chain 1	potassium-trans-locating ATPase C, chain 2	sensor kinase protein	KDP operon transcrip-tional regulatory protein	methicillin-resistance regulatory protein	signal transducer protein MecR1	homolog of xylose repressor, associated with SCCmec-elements	cassette chromosome recombinase genes A/B-3		mercury resistance operon		cassette chromosome recombinase genes "ccrAA" (hypothetical) and ccrC			cassette chromosome recombinase genes A/B-4	
					rrnD1 (S. aureus)	gapA	katA	coA	nuc1	spa	sbi	sarA	saeS	vraS	agrI	agrII	agrIII	agrIV	hld	mecA	delta_mecR	ugpQ	ccrA-1	ccrB-1	plsSCC (COL)	Q9XB68-dcs	ccrA-2	ccrB-2	kdpA-SCC	kdpB-SCC	kdpC-SCC	kdpD-SCC	kdpE-SCC	mecI	mecR	xylR	ccrA-3	ccrB-3	merA	merB	ccrAA (MRSAZH47) _probe 1	ccrAA (MRSAZH47) _probe 2	ccrC (85-2082)	ccrA-4	ccrB-4
**2008**																																													
08V04188	CC5/ST228-MRSA-I, South German EMRSA	MED-icu	40	m	POS	POS	POS	POS		POS	POS	POS			NEG	POS	NEG	NEG	POS	POS																									
08V21565	CC5/ST228-MRSA-I, South German EMRSA	MED-ICU	77	m	POS	POS	POS	POS		POS	POS				NEG	POS	NEG	NEG	POS	POS																									
08V53546	CC5/ST228-MRSA-I, South German EMRSA	SUR-ICU	68	m	POS	POS	POS	POS	POS	POS	POS	POS	POS	POS	NEG	POS	NEG	NEG	POS	POS	POS	POS	POS	POS	POS	AMB	NEG	NEG	NEG	NEG	NEG	NEG	NEG	NEG	NEG	NEG	NEG	NEG	POS	POS	NEG	NEG	NEG	NEG	NEG
08B04687	CC5-MRSA-II, Rhine-Hesse EMRSA/UK-EMRSA-3	SUR-ICU	70	m	POS	POS	POS	POS		POS	POS	POS			NEG	POS	NEG	NEG	POS	POS																									
08F01184	CC5-MRSA-II, Rhine-Hesse EMRSA/UK-EMRSA-3	MED-WARD	69	m	POS	POS	POS	POS		POS	POS	POS			NEG	POS	NEG	NEG	POS	POS																									
08F01352	CC5-MRSA-II, Rhine-Hesse EMRSA/UK-EMRSA-3	MED-ICU	59	m	POS	POS	POS	POS		POS	POS	POS			NEG	POS	NEG	NEG	POS	POS																									
08F01711	CC5-MRSA-II, Rhine-Hesse EMRSA/UK-EMRSA-3	SUR-WARD	84	f	POS	POS	POS	POS		POS	POS	POS			NEG	POS	NEG	NEG	POS	POS																									
08F03746	CC5-MRSA-II, Rhine-Hesse EMRSA/UK-EMRSA-3	MED-icu	0	m	POS	POS	POS	POS		POS	POS	POS			NEG	POS	NEG	NEG	POS	POS																									
08F04303	CC5-MRSA-II, Rhine-Hesse EMRSA/UK-EMRSA-3	MED-WARD	84	f	POS	POS	POS	POS		POS	POS	POS			NEG	POS	NEG	NEG	POS	POS																									
08F04396	CC5-MRSA-II, Rhine-Hesse EMRSA/UK-EMRSA-3	MED-WARD	80	m	POS	POS	POS	POS		POS	POS	POS			NEG	POS	NEG	NEG	POS	POS																									
08U11214	CC5-MRSA-II, Rhine-Hesse EMRSA/UK-EMRSA-3	MED-WARD	77	f	POS	POS	POS	POS		POS	POS	POS			NEG	POS	NEG	NEG	POS	POS																									
08V01047	CC5-MRSA-II, Rhine-Hesse EMRSA/UK-EMRSA-3	MED-ICU	57	m	POS	POS	POS	POS		POS	POS	POS			NEG	POS	NEG	NEG	POS	POS																									
08V01222	CC5-MRSA-II, Rhine-Hesse EMRSA/UK-EMRSA-3	SUR-ICU	70	m	POS	POS	POS	POS		POS	POS	POS			NEG	POS	NEG	NEG	POS	POS																									
08V01315	CC5-MRSA-II, Rhine-Hesse EMRSA/UK-EMRSA-3	SUR-WARD	70	f	POS	POS	POS	POS		POS	POS	POS			NEG	POS	NEG	NEG	POS	POS																									
08V06126	CC5-MRSA-II, Rhine-Hesse EMRSA/UK-EMRSA-3	MED-ICU	73	m	POS	POS	POS	POS		POS	POS	POS			NEG	POS	NEG	NEG	POS	POS																									
08V06171	CC5-MRSA-II, Rhine-Hesse EMRSA/UK-EMRSA-3	SUR-WARD	62	m	POS	POS	POS	POS		POS	POS	POS			NEG	POS	NEG	NEG	POS	POS																									
08V06623	CC5-MRSA-II, Rhine-Hesse EMRSA/UK-EMRSA-3	SUR-WARD	43	f	POS	POS	POS	POS		POS	POS	POS			NEG	POS	NEG	NEG	POS	POS																									
08V10539	CC5-MRSA-II, Rhine-Hesse EMRSA/UK-EMRSA-3	MED-WARD	67	m	POS	POS	POS	POS		POS	POS	POS			NEG	POS	NEG	NEG	POS	POS																									
08V11213	CC5-MRSA-II, Rhine-Hesse EMRSA/UK-EMRSA-3	SUR-ICU	60	m	POS	POS	POS	POS		POS	POS	POS			NEG	POS	NEG	NEG	POS	POS																									
08V12100	CC5-MRSA-II, Rhine-Hesse EMRSA/UK-EMRSA-3	MED-ICU	60	m	POS	POS	POS	POS		POS	POS	POS			NEG	POS	NEG	NEG	POS	POS																									
08V13847	CC5-MRSA-II, Rhine-Hesse EMRSA/UK-EMRSA-3	SUR-WARD	68	m	POS	POS	POS	POS		POS	POS	POS			NEG	POS	NEG	NEG	POS	POS																									
08V15905	CC5-MRSA-II, Rhine-Hesse EMRSA/UK-EMRSA-3	MED-WARD	67	m	POS	POS	POS	POS	POS	POS	POS	POS	POS	POS	NEG	POS	NEG	NEG	POS	POS	POS	POS	AMB	NEG	NEG	POS	POS	POS	POS	POS	POS	POS	POS	POS	POS	POS	NEG	NEG	NEG	NEG	NEG	NEG	NEG	NEG	NEG
08V18191	CC5-MRSA-II, Rhine-Hesse EMRSA/UK-EMRSA-3	MED-ICU	50	m	POS	POS	POS	POS	POS	POS	POS	POS	POS	POS	NEG	POS	NEG	NEG	POS	POS	POS	POS	NEG	NEG	NEG	AMB	POS	POS	POS	POS	POS	POS	POS	POS	POS	POS	NEG	NEG	NEG	NEG	NEG	NEG	NEG	NEG	NEG
08V21425	CC5-MRSA-II, Rhine-Hesse EMRSA/UK-EMRSA-3	SUR-WARD	35	m	POS	POS	POS	POS		POS	POS				NEG	POS	NEG	NEG	POS	POS																									
08V23868	CC5-MRSA-II, Rhine-Hesse EMRSA/UK-EMRSA-3	MED-ICU	66	f	POS	POS	POS	POS		POS	POS	POS			NEG	POS	NEG	NEG	POS	POS																									
08V27435	CC5-MRSA-II, Rhine-Hesse EMRSA/UK-EMRSA-3	MED-WARD	62	m	POS	POS	POS	POS		POS	POS	POS			NEG	POS	NEG	NEG	POS	POS																									
08V27514	CC5-MRSA-II, Rhine-Hesse EMRSA/UK-EMRSA-3	SUR-ICU	64	m	POS	POS	POS	POS		POS	POS	POS			NEG	POS	NEG	NEG	POS	POS																									
08V28482	CC5-MRSA-II, Rhine-Hesse EMRSA/UK-EMRSA-3	MED-WARD	73	m	POS	POS	POS	POS		POS	POS	POS			NEG	POS	NEG	NEG	POS	POS																									
08V29312	CC5-MRSA-II, Rhine-Hesse EMRSA/UK-EMRSA-3	MED-icu	0	m	POS	POS	POS	POS		POS	POS	POS			NEG	POS	NEG	NEG	POS	POS																									
08V29889	CC5-MRSA-II, Rhine-Hesse EMRSA/UK-EMRSA-3	MED-icu	0	m	POS	POS	POS	POS		POS	POS	POS			NEG	POS	NEG	NEG	POS	POS																									
08V30696	CC5-MRSA-II, Rhine-Hesse EMRSA/UK-EMRSA-3	MED-icu	0	f	POS	POS	POS	POS		POS	POS	POS			NEG	POS	NEG	NEG	POS	POS																									
08V32764	CC5-MRSA-II, Rhine-Hesse EMRSA/UK-EMRSA-3	SUR-ICU	58	m	POS	POS	POS	POS		POS	POS	POS			NEG	POS	NEG	NEG	POS	POS																									
08V36052	CC5-MRSA-II, Rhine-Hesse EMRSA/UK-EMRSA-3	MED-OPD	73	m	POS	POS	POS	POS		POS	POS	POS			NEG	POS	NEG	NEG	POS	POS																									
08V36125	CC5-MRSA-II, Rhine-Hesse EMRSA/UK-EMRSA-3	SUR-WARD	78	f	POS	POS	POS	POS		POS	POS	POS			NEG	POS	NEG	NEG	POS	POS																									
08V39935	CC5-MRSA-II, Rhine-Hesse EMRSA/UK-EMRSA-3	SUR-WARD	68	m	POS	POS	POS	POS		POS	POS	POS			NEG	POS	NEG	NEG	POS	POS																									
08V41631	CC5-MRSA-II, Rhine-Hesse EMRSA/UK-EMRSA-3	SUR-WARD	71	m	POS	POS	POS	POS		POS	POS	POS			NEG	POS	NEG	NEG	POS	POS																									
08V42588	CC5-MRSA-II, Rhine-Hesse EMRSA/UK-EMRSA-3	MED-WARD	55	f	POS	POS	POS	POS		POS	POS				NEG	POS	NEG	NEG	POS	POS																									
08V42633	CC5-MRSA-II, Rhine-Hesse EMRSA/UK-EMRSA-3	MED-WARD	11	f	POS	POS	POS	POS		POS	POS	POS			NEG	POS	NEG	NEG	POS	POS																									
08V43410	CC5-MRSA-II, Rhine-Hesse EMRSA/UK-EMRSA-3	MED-ICU	77	m	POS	POS	POS	POS		POS	POS	POS			NEG	POS	NEG	NEG	POS	POS																									
08V43581	CC5-MRSA-II, Rhine-Hesse EMRSA/UK-EMRSA-3	MED-OPD	86	m	POS	POS	POS	POS		POS	POS	POS			NEG	POS	NEG	NEG	POS	POS																									
08V47242	CC5-MRSA-II, Rhine-Hesse EMRSA/UK-EMRSA-3	MED-icu	0	f	POS	POS	POS	POS	POS	POS	POS	POS	POS	POS	NEG	POS	NEG	NEG	POS	POS	POS	POS	NEG	NEG	NEG	POS	POS	POS	POS	POS	POS	POS	POS	POS	POS	POS	NEG	NEG	NEG	NEG	NEG	NEG	NEG	NEG	NEG
08V50740	CC5-MRSA-II, Rhine-Hesse EMRSA/UK-EMRSA-3	SUR-ICU	79	m	POS	POS	POS	POS	POS	POS	POS	POS	POS	POS	NEG	POS	NEG	NEG	POS	POS	POS	POS	NEG	NEG	NEG	NEG	POS	POS	POS	POS	POS	POS	POS	POS	POS	POS	NEG	NEG	NEG	NEG	NEG	NEG	NEG	NEG	NEG
08V02855	CC7-MRSA-IV	MED-ICU	60	m	POS	POS	POS	POS	POS	POS	POS	POS	POS	POS	POS	NEG	NEG	NEG	POS	POS	POS	POS	NEG	NEG	NEG	POS	POS	POS	NEG	NEG	NEG	NEG	NEG	NEG	NEG	NEG	NEG	NEG	NEG	NEG	NEG	NEG	NEG	NEG	NEG
08V35987	CC8/ST239-MRSA-III, Vienna/Hungarian/Brazilian Clone	SUR-WARD	44	m	POS	POS	POS	POS	POS	POS	POS	POS	POS	POS	POS	NEG	NEG	NEG	POS	POS	POS	POS	NEG	POS	NEG	NEG	NEG	NEG	NEG	NEG	NEG	NEG	NEG	POS	POS	POS	POS	POS	NEG	NEG	NEG	NEG	POS	NEG	NEG
08V39853	CC8/ST254-MRSA-(atypical SCCmec), Hannover EMRSA	SUR-ICU	84	m	POS	POS	POS	POS	POS	POS	POS	POS	POS	POS	POS	NEG	NEG	AMB	POS	POS	POS	POS	NEG	NEG	NEG	NEG	NEG	NEG	NEG	NEG	NEG	NEG	NEG	NEG	NEG	NEG	NEG	NEG	POS	POS	NEG	NEG	NEG	NEG	NEG
08F01314	CC8-MRSA-IV, UK-EMRSA-14/WA MRSA-5	SUR-Ward	1	f	POS	POS	POS	POS	NEG	POS	POS	POS	POS	POS	POS	NEG	NEG	NEG	POS	POS	POS	POS	NEG	NEG	NEG	NEG	POS	POS	NEG	NEG	NEG	NEG	NEG	NEG	NEG	NEG	NEG	NEG	NEG	NEG	NEG	NEG	NEG	NEG	NEG
08V15773	CC8-MRSA-IV, USA300	SUR-Ward	9	f	POS	POS	POS	POS	POS	POS	POS	POS	POS	POS	POS	NEG	NEG	AMB	POS	POS	POS	POS	NEG	NEG	NEG	POS	POS	POS	NEG	NEG	NEG	NEG	NEG	NEG	NEG	NEG	NEG	POS	NEG	NEG	NEG	NEG	NEG	NEG	NEG
08B02942	CC22-MRSA-IV, Barnim/UK-EMRSA-15	MED-ICU	72	m	POS	POS	POS	POS		POS	POS	POS			POS	NEG	NEG	NEG	POS	POS																									
08F00816	CC22-MRSA-IV, Barnim/UK-EMRSA-15	MED-WARD	88	m	POS	POS	POS	POS		POS	POS				POS	NEG	NEG	NEG	POS	POS																									
08F01637	CC22-MRSA-IV, Barnim/UK-EMRSA-15	SUR-WARD	38	f	POS	POS	POS	POS		POS	POS	POS			POS	NEG	NEG	AMB	POS	POS																									
08F04948	CC22-MRSA-IV, Barnim/UK-EMRSA-15	SUR-WARD	31	f	POS	POS	POS	POS		POS	POS	POS			POS	NEG	NEG	AMB	POS	POS																									
08U07826	CC22-MRSA-IV, Barnim/UK-EMRSA-15	MED-WARD	75	f	POS	POS	POS	POS		POS	POS	POS			POS	NEG	NEG	NEG	POS	POS																									
08V00371	CC22-MRSA-IV, Barnim/UK-EMRSA-15	MED-ICU	38	m	POS	POS	POS	POS		POS	POS	POS			POS	NEG	NEG	NEG	POS	POS																									
08V02049	CC22-MRSA-IV, Barnim/UK-EMRSA-15	SUR-WARD	41	m	POS	POS	POS	POS		POS	POS	POS			POS	NEG	NEG	AMB	POS	POS																									
08V02349	CC22-MRSA-IV, Barnim/UK-EMRSA-15	MED-OPD	68	m	POS	POS	POS	POS		POS	POS	POS			POS	NEG	NEG	AMB	POS	POS																									
08V03329	CC22-MRSA-IV, Barnim/UK-EMRSA-15	MED-ICU	54	m	POS	POS	POS	POS		POS	POS				POS	NEG	NEG	NEG	POS	POS																									
08V04516	CC22-MRSA-IV, Barnim/UK-EMRSA-15	MED-icu	0	m	POS	POS	POS	POS		POS	POS	POS			POS	NEG	NEG	AMB	POS	POS																									
08V04681	CC22-MRSA-IV, Barnim/UK-EMRSA-15	MED-WARD	66	m	POS	POS	POS	POS		POS	POS	POS			POS	NEG	NEG	AMB	POS	POS																									
08V04740	CC22-MRSA-IV, Barnim/UK-EMRSA-15	MED-WARD	45	m	POS	POS	POS	POS		POS	POS	POS			POS	NEG	NEG	AMB	POS	POS																									
08V04977	CC22-MRSA-IV, Barnim/UK-EMRSA-15	SUR-ICU	68	f	POS	POS	POS	POS		POS	POS	POS			POS	NEG	NEG	AMB	POS	POS																									
08V06376	CC22-MRSA-IV, Barnim/UK-EMRSA-15	MED-ICU	79	m	POS	POS	POS	POS		POS	POS	POS			POS	NEG	NEG	AMB	POS	POS																									
08V09540	CC22-MRSA-IV, Barnim/UK-EMRSA-15	MED-WARD	86	m	POS	POS	POS	POS		POS	POS	POS			POS	NEG	NEG	NEG	POS	POS																									
08V10287	CC22-MRSA-IV, Barnim/UK-EMRSA-15	MED-ICU	71	m	POS	POS	POS	POS		POS	POS				POS	NEG	NEG	NEG	POS	POS																									
08V10376	CC22-MRSA-IV, Barnim/UK-EMRSA-15	MED-ICU	68	m	POS	POS	POS	POS		POS	POS	POS			POS	NEG	NEG	NEG	POS	POS																									
08V10714	CC22-MRSA-IV, Barnim/UK-EMRSA-15	MED-ICU	71	m	POS	POS	POS	POS		POS	POS	POS			POS	NEG	NEG	NEG	POS	POS																									
08V10836	CC22-MRSA-IV, Barnim/UK-EMRSA-15	MED-ICU	73	m	POS	POS	POS	POS		POS	POS	POS			POS	NEG	NEG	AMB	POS	POS																									
08V11971	CC22-MRSA-IV, Barnim/UK-EMRSA-15	MED-ICU	51	m	POS	POS	POS	POS		POS	POS	POS			POS	NEG	NEG	AMB	POS	POS																									
08V14130	CC22-MRSA-IV, Barnim/UK-EMRSA-15	SUR-WARD	76	m	POS	POS	POS	POS		POS	POS	POS			POS	NEG	NEG	AMB	POS	POS																									
08V14723	CC22-MRSA-IV, Barnim/UK-EMRSA-15	MED-ICU	77	m	POS	POS	POS	POS		POS	POS	POS			POS	NEG	NEG	NEG	POS	POS																									
08V16292	CC22-MRSA-IV, Barnim/UK-EMRSA-15	SUR-WARD	63	m	POS	POS	POS	POS		POS	POS	POS			POS	NEG	NEG	AMB	POS	POS																									
08V16554	CC22-MRSA-IV, Barnim/UK-EMRSA-15	MED-icu	68	m	POS	POS	POS	POS		POS	POS	POS			POS	NEG	NEG	AMB	POS	POS																									
08V18482	CC22-MRSA-IV, Barnim/UK-EMRSA-15	MED-ICU	69	m	POS	POS	POS	POS		POS	POS	POS			POS	NEG	NEG	AMB	POS	POS																									
08V19658	CC22-MRSA-IV, Barnim/UK-EMRSA-15	MED-WARD	58	m	POS	POS	POS	POS		POS	POS	POS			POS	NEG	NEG	NEG	POS	POS																									
08V19663	CC22-MRSA-IV, Barnim/UK-EMRSA-15	SUR-ICU	73	f	POS	POS	POS	POS		POS	POS	POS			POS	NEG	NEG	NEG	POS	POS																									
08V20266	CC22-MRSA-IV, Barnim/UK-EMRSA-15	SUR-ICU	71	m	POS	POS	POS	POS		POS	POS	POS			POS	NEG	NEG	NEG	POS	POS																									
08V20337	CC22-MRSA-IV, Barnim/UK-EMRSA-15	SUR-WARD	83	f	POS	POS	POS	POS		POS	POS	POS			POS	NEG	NEG	NEG	POS	POS																									
08V21055	CC22-MRSA-IV, Barnim/UK-EMRSA-15	SUR-WARD	85	f	POS	POS	POS	POS		POS	POS	POS			POS	NEG	NEG	AMB	POS	POS																									
08V23747	CC22-MRSA-IV, Barnim/UK-EMRSA-15	SUR-ICU	78	f	POS	POS	POS	POS		POS	POS	POS			POS	NEG	NEG	NEG	POS	POS																									
08V23813	CC22-MRSA-IV, Barnim/UK-EMRSA-15	MED-WARD	79	m	POS	POS	POS	POS		POS	POS	POS			POS	NEG	NEG	NEG	POS	POS																									
08V25135	CC22-MRSA-IV, Barnim/UK-EMRSA-15	MED-WARD	67	f	POS	POS	POS	POS		POS	POS	POS			POS	NEG	NEG	NEG	POS	POS																									
08V27329	CC22-MRSA-IV, Barnim/UK-EMRSA-15	MED-OPD	79	f	POS	POS	POS	POS		POS	POS	POS			POS	NEG	NEG	AMB	POS	POS																									
08V27434	CC22-MRSA-IV, Barnim/UK-EMRSA-15	MED-WARD	76	f	POS	POS	POS	POS		POS	POS	POS			POS	NEG	NEG	AMB	POS	POS																									
08V27729	CC22-MRSA-IV, Barnim/UK-EMRSA-15	SUR-ICU	76	m	POS	POS	POS	POS		POS	POS	POS			POS	NEG	NEG	AMB	POS	POS																									
08V31445	CC22-MRSA-IV, Barnim/UK-EMRSA-15	SUR-ICU	77	m	POS	POS	POS	POS		POS	POS	POS			POS	NEG	NEG	AMB	POS	POS																									
08V34812	CC22-MRSA-IV, Barnim/UK-EMRSA-15	MED-WARD	86	f	POS	POS	POS	POS		POS	POS	POS			POS	NEG	NEG	NEG	POS	POS																									
08V34813	CC22-MRSA-IV, Barnim/UK-EMRSA-15	MED-WARD	88	f	POS	POS	POS	POS		POS	POS	POS			POS	NEG	NEG	NEG	POS	POS																									
08V35361	CC22-MRSA-IV, Barnim/UK-EMRSA-15	MED-WARD	78	m	POS	POS	POS	POS		POS	POS	POS			POS	NEG	NEG	NEG	POS	POS																									
08V37931	CC22-MRSA-IV, Barnim/UK-EMRSA-15	SUR-WARD	80	f	POS	POS	POS	POS		POS	POS	POS			POS	NEG	NEG	AMB	POS	POS																									
08V45578	CC22-MRSA-IV, Barnim/UK-EMRSA-15	SUR-WARD	84	m	POS	POS	POS	POS		POS	POS	POS			POS	NEG	NEG	AMB	POS	POS																									
08V47385	CC22-MRSA-IV, Barnim/UK-EMRSA-15	SUR-ICU	56	m	POS	POS	POS	POS	POS	POS	POS	POS	POS	POS	POS	NEG	NEG	AMB	POS	POS	POS	POS	NEG	NEG	NEG	POS	POS	POS	NEG	NEG	NEG	NEG	NEG	NEG	NEG	NEG	NEG	NEG	NEG	NEG	NEG	NEG	NEG	NEG	NEG
08V53678	CC22-MRSA-IV, Barnim/UK-EMRSA-15	SUR-ICU	61	m	POS	POS	POS	POS	POS	POS	POS	POS	POS	POS	POS	NEG	NEG	NEG	POS	POS	POS	POS	NEG	NEG	NEG	AMB	POS	POS	NEG	NEG	NEG	NEG	NEG	NEG	NEG	NEG	NEG	NEG	NEG	NEG	NEG	NEG	NEG	NEG	NEG
08B09737	CC45-MRSA-IV, Berlin EMRSA	MED-WARD	86	m	POS	POS	POS	POS		AMB	POS				POS	NEG	NEG	AMB	POS	POS																									
08B14321	CC45-MRSA-IV, Berlin EMRSA	MED-WARD	68	m	POS	POS	POS	POS	POS	POS	POS	POS	POS	POS	POS	NEG	NEG	AMB	POS	POS	POS	POS	NEG	NEG	NEG	POS	POS	POS	NEG	NEG	NEG	NEG	NEG	NEG	NEG	NEG	NEG	NEG	NEG	NEG	NEG	NEG	NEG	NEG	NEG
08F01439	CC45-MRSA-IV, Berlin EMRSA	MED-WARD	80	m	POS	POS	POS	POS		POS	POS	POS			POS	NEG	NEG	NEG	POS	POS																									
08U12518	CC45-MRSA-IV, Berlin EMRSA	MED-OPD	12	m	POS	POS	POS	POS		POS	POS	POS			POS	NEG	NEG	NEG	POS	POS																									
08U13653	CC45-MRSA-IV, Berlin EMRSA	SUR-WARD	85	m	POS	POS	POS	POS	POS	POS	POS	POS	POS	POS	POS	NEG	NEG	NEG	POS	POS	POS	POS	NEG	NEG	NEG	POS	POS	POS	NEG	NEG	NEG	NEG	NEG	NEG	NEG	NEG	NEG	NEG	NEG	NEG	NEG	NEG	NEG	NEG	NEG
08V00675	CC45-MRSA-IV, Berlin EMRSA	MED-OPD	58	m	POS	POS	POS	POS		POS	POS	POS			POS	NEG	NEG	NEG	POS	POS																									
08V02459	CC45-MRSA-IV, Berlin EMRSA	MED-WARD	69	m	POS	POS	POS	POS		POS	POS	POS			POS	NEG	NEG	NEG	POS	POS																									
08V02654	CC45-MRSA-IV, Berlin EMRSA	MED-WARD	78	m	POS	POS	POS	POS		POS	POS	POS			POS	NEG	NEG	NEG	POS	POS																									
08V05677	CC45-MRSA-IV, Berlin EMRSA	MED-WARD	57	f	POS	POS	POS	POS	POS	POS	POS	POS	POS	POS	POS	NEG	NEG	NEG	POS	POS	POS	POS	NEG	NEG	NEG	POS	POS	POS	NEG	NEG	NEG	NEG	NEG	AMB	NEG	NEG	NEG	NEG	NEG	NEG	NEG	NEG	NEG	NEG	NEG
08V20930	CC45-MRSA-IV, Berlin EMRSA	MED-OPD	91	f	POS	POS	POS	POS		POS	POS	POS			POS	NEG	NEG	NEG	POS	POS																									
08V23911	CC45-MRSA-IV, Berlin EMRSA	MED-OPD	59	f	POS	POS	POS	POS		POS	POS	POS			POS	NEG	NEG	NEG	POS	POS																									
08V39175	CC45-MRSA-IV, Berlin EMRSA	SUR-ICU	82	m	POS	POS	POS	POS		POS	POS	p			POS	NEG	NEG	NEG	POS	POS																									
08V39949	CC45-MRSA-IV, Berlin EMRSA	MED-ICU	89	f	POS	POS	POS	POS		AMB	POS	POS			POS	NEG	NEG	NEG	POS	POS																									
08V53670	CC88-MRSA-IV, WA MRSA-2	SUR-ICU	33	f	POS	POS	POS	POS	POS	POS	POS	POS	POS	POS	NEG	NEG	POS	NEG	POS	POS	POS	POS	NEG	NEG	NEG	NEG	POS	POS	NEG	NEG	NEG	NEG	NEG	NEG	NEG	NEG	NEG	NEG	NEG	NEG	NEG	NEG	NEG	NEG	NEG
08V06946	CC398-MRSA-V	MED-OPD	67	m	POS	POS	POS	POS	POS	POS	POS	POS	POS	POS	POS	NEG	NEG	NEG	POS	POS	NEG	POS	NEG	NEG	NEG	NEG	NEG	NEG	NEG	NEG	NEG	NEG	NEG	NEG	NEG	NEG	NEG	NEG	NEG	NEG	NEG	NEG	NEG	NEG	NEG

ISOLATE:	STRAIN	RESISTANCE : PENICILLINASE			RESISTANCE : MLS-ANTIBIOTICS												RESISTANCE : AMINOGLYSOSIDES			RESISTANCE : MISCELLANEOUS GENES																RESISTANCE : EFFLUX SYSTEMS								RESISTANCE : GLYCOPEPTIDES		
		beta-lactamase	beta lactamase repressor (inhibitor)	beta-lactamase regulatory protein	rRNA adenine N-6-methyl-transferase, erythromycin/clindamycin resistance	erythro-mycin/clindamycin resistance	erythro-mycin/clindamycin resistance	Linco-samid-Nucleo-tidyltrans-ferase	energy-dependent efflux of erythro-mycin	macrolide efflux protein A	probable lysylphos-phatidyl-glycerol synthetase	virginia-mycin A acetyltransferase	acetyl-transferase inactivating streptogramin A	ATP binding protein, streptogramin-A-resistance		virginia-mycin B hydrolase	bifunctional enzyme Aac/Aph, gentamicin resistance	amino-glycoside adenyl-transferase,tobramycin resistance	3'5'-aminog-lycoside phospho-transferase, neo-/ kanamycin resistance	strepto-thricine-acetyl-transferase	dihydro-folate reductase type 1	fusidic acid resistence	hypothetical protein associated with fusidic acid resistance	mupirocin resistence protein	tetrazyklin-resistance	tetracycline-resistance	chloramphenicol acetyltransferase					23S rRNA methyltransferase	chloramphenicol /florfenicol exporter	metallothiol transferase		quaternary ammonium compound resistance protein A	quaternary ammonium compound resistance protein C						Transport/ Efflux protein	vancomycin resistance gene	vancomycin resistance gene from enterococci and Clostridium	teicoplanin resistance gene from enterococci
		blaZ	blaI	blaR	erm(A)	erm(B)	erm(C)	lnu(A)	msr(A)	mef(A)	mph(C)	vat(A)	vat(B)	vga(A)	vga(A) (BM 3327)	vgB(A)	aacA-aphD	aadD	aphA3	sat	dfrA	far1	Q6GD50	mupR	tet(K)	tet(M)	cat (total)	cat (pC221)	cat (pc223)	cat (pMC524)	cat (pSBK203R)	cfr	fexA	fosB	fosB (plasmid)	qacA	qacC (total)	qacC (consensus)	qacC (equine)	qacC (SA5)	qacC (Ssap)	qacC (ST94)	tetEfflux	vanA	vanB	vanZ
**2008**																																														
08V04188	CC5/ST228-MRSA-I, South German EMRSA	POS			POS		NEG	NEG	NEG			NEG	NEG	NEG	NEG	NEG	AMB	NEG	POS	POS	NEG	NEG		NEG	NEG	NEG																		NEG	NEG	NEG
08V21565	CC5/ST228-MRSA-I, South German EMRSA	POS			POS		NEG	NEG	NEG			NEG	NEG	NEG	NEG	NEG	POS	NEG	POS	POS	NEG	NEG		NEG	NEG	NEG																		NEG	NEG	NEG
08V53546	CC5/ST228-MRSA-I, South German EMRSA	POS	POS	POS	POS	NEG	NEG	NEG	NEG	NEG	NEG	NEG	NEG	NEG	NEG	NEG	POS	NEG	POS	POS	NEG	NEG	NEG	NEG	NEG	NEG	NEG	NEG	NEG	NEG	NEG	NEG	NEG	POS	NEG	POS	NEG	NEG	NEG	NEG	NEG	NEG	POS	NEG	NEG	NEG
08B04687	CC5-MRSA-II, Rhine-Hesse EMRSA/UK-EMRSA-3	POS			POS		NEG	NEG	NEG			NEG	NEG	NEG	NEG	NEG	NEG	POS	NEG	NEG	NEG	NEG		NEG	NEG	NEG																		NEG	NEG	NEG
08F01184	CC5-MRSA-II, Rhine-Hesse EMRSA/UK-EMRSA-3	POS			POS		NEG	NEG	NEG			NEG	NEG	NEG	NEG	NEG	NEG	NEG	NEG	NEG	NEG	NEG		NEG	NEG	NEG																		NEG	NEG	NEG
08F01352	CC5-MRSA-II, Rhine-Hesse EMRSA/UK-EMRSA-3	POS			POS		NEG	NEG	NEG			NEG	NEG	NEG	NEG	NEG	NEG	NEG	NEG	NEG	NEG	NEG		NEG	NEG	NEG																		NEG	NEG	NEG
08F01711	CC5-MRSA-II, Rhine-Hesse EMRSA/UK-EMRSA-3	POS			POS		NEG	NEG	NEG			NEG	NEG	NEG	NEG	NEG	NEG	POS	NEG	NEG	NEG	NEG		NEG	NEG	NEG																		NEG	NEG	NEG
08F03746	CC5-MRSA-II, Rhine-Hesse EMRSA/UK-EMRSA-3	POS			POS		NEG	NEG	NEG			NEG	NEG	NEG	NEG	NEG	NEG	POS	NEG	NEG	NEG	NEG		NEG	NEG	NEG																		NEG	NEG	NEG
08F04303	CC5-MRSA-II, Rhine-Hesse EMRSA/UK-EMRSA-3	POS			POS		NEG	NEG	NEG			NEG	NEG	NEG	NEG	NEG	NEG	NEG	NEG	NEG	NEG	NEG		NEG	NEG	NEG																		NEG	NEG	NEG
08F04396	CC5-MRSA-II, Rhine-Hesse EMRSA/UK-EMRSA-3	POS			POS		NEG	NEG	NEG			NEG	NEG	NEG	NEG	NEG	NEG	NEG	NEG	NEG	NEG	NEG		NEG	NEG	NEG																		NEG	NEG	NEG
08U11214	CC5-MRSA-II, Rhine-Hesse EMRSA/UK-EMRSA-3	POS			POS		NEG	NEG	NEG			NEG	NEG	NEG	NEG	NEG	NEG	NEG	NEG	NEG	NEG	NEG		NEG	NEG	NEG																		NEG	NEG	NEG
08V01047	CC5-MRSA-II, Rhine-Hesse EMRSA/UK-EMRSA-3	POS			POS		NEG	NEG	NEG			NEG	NEG	NEG	NEG	NEG	NEG	POS	NEG	NEG	NEG	NEG		NEG	NEG	NEG																		NEG	NEG	NEG
08V01222	CC5-MRSA-II, Rhine-Hesse EMRSA/UK-EMRSA-3	NEG			POS		NEG	NEG	NEG			NEG	NEG	NEG	NEG	NEG	NEG	POS	NEG	NEG	NEG	NEG		NEG	NEG	NEG																		NEG	NEG	NEG
08V01315	CC5-MRSA-II, Rhine-Hesse EMRSA/UK-EMRSA-3	POS			POS		NEG	NEG	NEG			NEG	NEG	NEG	NEG	NEG	NEG	POS	NEG	NEG	NEG	NEG		NEG	NEG	NEG																		NEG	NEG	NEG
08V06126	CC5-MRSA-II, Rhine-Hesse EMRSA/UK-EMRSA-3	POS			POS		NEG	NEG	NEG			NEG	NEG	NEG	NEG	NEG	NEG	POS	NEG	NEG	NEG	NEG		NEG	NEG	NEG																		NEG	NEG	NEG
08V06171	CC5-MRSA-II, Rhine-Hesse EMRSA/UK-EMRSA-3	POS			POS		NEG	NEG	NEG			NEG	NEG	NEG	NEG	NEG	NEG	POS	NEG	NEG	NEG	NEG		NEG	NEG	NEG																		NEG	NEG	NEG
08V06623	CC5-MRSA-II, Rhine-Hesse EMRSA/UK-EMRSA-3	POS			POS		NEG	NEG	NEG			NEG	NEG	NEG	NEG	NEG	NEG	POS	NEG	NEG	NEG	NEG		NEG	NEG	NEG																		NEG	NEG	NEG
08V10539	CC5-MRSA-II, Rhine-Hesse EMRSA/UK-EMRSA-3	POS			POS		NEG	NEG	NEG			NEG	NEG	NEG	NEG	NEG	NEG	POS	NEG	NEG	NEG	NEG		NEG	NEG	NEG																		NEG	NEG	NEG
08V11213	CC5-MRSA-II, Rhine-Hesse EMRSA/UK-EMRSA-3	POS			POS		NEG	NEG	NEG			NEG	NEG	NEG	NEG	NEG	NEG	POS	NEG	NEG	NEG	NEG		NEG	NEG	NEG																		NEG	NEG	NEG
08V12100	CC5-MRSA-II, Rhine-Hesse EMRSA/UK-EMRSA-3	POS			POS		NEG	NEG	NEG			NEG	NEG	NEG	NEG	NEG	NEG	POS	NEG	NEG	NEG	NEG		NEG	NEG	NEG																		NEG	NEG	NEG
08V13847	CC5-MRSA-II, Rhine-Hesse EMRSA/UK-EMRSA-3	POS			POS		NEG	NEG	NEG			NEG	NEG	NEG	NEG	NEG	NEG	POS	NEG	NEG	NEG	NEG		NEG	NEG	NEG																		NEG	NEG	NEG
08V15905	CC5-MRSA-II, Rhine-Hesse EMRSA/UK-EMRSA-3	NEG	NEG	NEG	POS	NEG	NEG	NEG	NEG	NEG	NEG	NEG	NEG	NEG	NEG	NEG	NEG	POS	NEG	NEG	NEG	NEG	NEG	NEG	NEG	NEG	NEG	NEG	NEG	NEG	NEG	NEG	NEG	POS	NEG	NEG	NEG	NEG	NEG	NEG	NEG	NEG	POS	NEG	NEG	NEG
08V18191	CC5-MRSA-II, Rhine-Hesse EMRSA/UK-EMRSA-3	POS	POS	POS	POS		NEG	NEG	NEG	NEG	NEG	NEG	NEG	NEG	NEG	NEG	NEG	NEG	NEG	NEG	NEG	NEG	NEG	NEG	NEG	NEG	NEG	NEG	NEG	NEG	NEG	NEG	NEG	POS	NEG	NEG	NEG	NEG	NEG	NEG	NEG	NEG	POS	NEG	NEG	NEG
08V21425	CC5-MRSA-II, Rhine-Hesse EMRSA/UK-EMRSA-3	POS			POS		NEG	NEG	NEG			NEG	NEG	NEG	NEG	NEG	NEG	NEG	NEG	NEG	NEG	NEG		NEG	NEG	NEG																		NEG	NEG	NEG
08V23868	CC5-MRSA-II, Rhine-Hesse EMRSA/UK-EMRSA-3	NEG			POS		NEG	NEG	NEG			NEG	NEG	NEG	NEG	NEG	NEG	NEG	NEG	NEG	NEG	NEG		NEG	NEG	NEG																		NEG	NEG	NEG
08V27435	CC5-MRSA-II, Rhine-Hesse EMRSA/UK-EMRSA-3	POS			POS		NEG	NEG	NEG			NEG	NEG	NEG	NEG	NEG	NEG	POS	NEG	NEG	NEG	NEG		NEG	NEG	NEG																		NEG	NEG	NEG
08V27514	CC5-MRSA-II, Rhine-Hesse EMRSA/UK-EMRSA-3	POS			POS		NEG	NEG	NEG			NEG	NEG	NEG	NEG	NEG	NEG	POS	NEG	NEG	NEG	NEG		NEG	NEG	NEG																		NEG	NEG	NEG
08V28482	CC5-MRSA-II, Rhine-Hesse EMRSA/UK-EMRSA-3	POS			POS		NEG	NEG	NEG			NEG	NEG	NEG	NEG	NEG	NEG	POS	NEG	NEG	NEG	NEG		NEG	NEG	NEG																		NEG	NEG	NEG
08V29312	CC5-MRSA-II, Rhine-Hesse EMRSA/UK-EMRSA-3	POS			POS		NEG	NEG	NEG			NEG	NEG	NEG	NEG	NEG	NEG	POS	NEG	NEG	NEG	NEG		NEG	NEG	NEG																		NEG	NEG	NEG
08V29889	CC5-MRSA-II, Rhine-Hesse EMRSA/UK-EMRSA-3	POS			POS		NEG	NEG	NEG			NEG	NEG	NEG	NEG	NEG	NEG	POS	NEG	NEG	NEG	NEG		NEG	NEG	NEG																		NEG	NEG	NEG
08V30696	CC5-MRSA-II, Rhine-Hesse EMRSA/UK-EMRSA-3	POS			POS		NEG	NEG	NEG			NEG	NEG	NEG	NEG	NEG	NEG	POS	NEG	NEG	NEG	NEG		NEG	NEG	NEG																		NEG	NEG	NEG
08V32764	CC5-MRSA-II, Rhine-Hesse EMRSA/UK-EMRSA-3	NEG			POS		NEG	NEG	NEG			NEG	NEG	NEG	NEG	NEG	NEG	POS	NEG	NEG	NEG	NEG		NEG	NEG	NEG																		NEG	NEG	NEG
08V36052	CC5-MRSA-II, Rhine-Hesse EMRSA/UK-EMRSA-3	POS			POS		NEG	NEG	NEG			NEG	NEG	NEG	NEG	NEG	NEG	NEG	NEG	NEG	NEG	NEG		NEG	NEG	NEG																		NEG	NEG	NEG
08V36125	CC5-MRSA-II, Rhine-Hesse EMRSA/UK-EMRSA-3	POS			POS		NEG	NEG	NEG			NEG	NEG	NEG	NEG	NEG	NEG	NEG	NEG	NEG	NEG	NEG		NEG	NEG	NEG																		NEG	NEG	NEG
08V39935	CC5-MRSA-II, Rhine-Hesse EMRSA/UK-EMRSA-3	POS			POS		NEG	NEG	NEG			NEG	NEG	NEG	NEG	NEG	NEG	POS	NEG	NEG	NEG	NEG		NEG	NEG	NEG																		NEG	NEG	NEG
08V41631	CC5-MRSA-II, Rhine-Hesse EMRSA/UK-EMRSA-3	POS			POS		NEG	NEG	NEG			NEG	NEG	NEG	NEG	NEG	NEG	POS	NEG	NEG	NEG	NEG		NEG	NEG	NEG																		NEG	NEG	NEG
08V42588	CC5-MRSA-II, Rhine-Hesse EMRSA/UK-EMRSA-3	POS			POS		NEG	NEG	NEG			NEG	NEG	NEG	NEG	NEG	NEG	POS	NEG	NEG	NEG	NEG		NEG	NEG	NEG																		NEG	NEG	NEG
08V42633	CC5-MRSA-II, Rhine-Hesse EMRSA/UK-EMRSA-3	POS			POS		NEG	NEG	NEG			NEG	NEG	NEG	NEG	NEG	NEG	POS	NEG	NEG	NEG	NEG		NEG	NEG	NEG																		NEG	NEG	NEG
08V43410	CC5-MRSA-II, Rhine-Hesse EMRSA/UK-EMRSA-3	NEG			POS		NEG	NEG	NEG			NEG	NEG	NEG	NEG	NEG	NEG	NEG	NEG	NEG	NEG	NEG		NEG	NEG	NEG																		NEG	NEG	NEG
08V43581	CC5-MRSA-II, Rhine-Hesse EMRSA/UK-EMRSA-3	POS			POS		NEG	NEG	NEG			NEG	NEG	NEG	NEG	NEG	NEG	POS	NEG	NEG	NEG	NEG		NEG	NEG	NEG																		NEG	NEG	NEG
08V47242	CC5-MRSA-II, Rhine-Hesse EMRSA/UK-EMRSA-3	POS	POS	POS	POS	NEG	NEG	NEG	NEG	NEG	NEG	NEG	NEG	NEG	NEG	NEG	NEG	POS	NEG	NEG	NEG	NEG	NEG	NEG	NEG	NEG	NEG	NEG	NEG	NEG	NEG	NEG	NEG	POS	NEG	NEG	NEG	NEG	NEG	NEG	NEG	NEG	POS	NEG	NEG	NEG
08V50740	CC5-MRSA-II, Rhine-Hesse EMRSA/UK-EMRSA-3	POS	POS	POS	POS	NEG	NEG	NEG	NEG	NEG	NEG	NEG	NEG	NEG	NEG	NEG	NEG	NEG	NEG	NEG	NEG	NEG	NEG	NEG	NEG	NEG	NEG	NEG	NEG	NEG	NEG	NEG	NEG	POS	NEG	NEG	NEG	NEG	NEG	NEG	NEG	NEG	POS	NEG	NEG	NEG
08V02855	CC7-MRSA-IV	POS	POS	POS	NEG	NEG	NEG	NEG	NEG	NEG	NEG	NEG	NEG	NEG	NEG	NEG	NEG	NEG	NEG	NEG	NEG	NEG	NEG	NEG	NEG	NEG	NEG	NEG	NEG	NEG	NEG	NEG	NEG	NEG	NEG	NEG	NEG	NEG	NEG	NEG	NEG	NEG	POS	NEG	NEG	NEG
08V35987	CC8/ST239-MRSA-III, Vienna/Hungarian/Brazilian Clone	POS	POS	POS	POS		NEG	NEG	NEG	NEG	NEG	NEG	NEG	NEG	NEG	NEG	POS	NEG	NEG	NEG	NEG	NEG	NEG	NEG	NEG	POS	NEG	NEG	NEG	NEG	NEG	NEG	NEG	POS	NEG	NEG	POS	POS	NEG	NEG	NEG	NEG	POS	NEG	NEG	NEG
08V39853	CC8/ST254-MRSA-(atypical SCCmec), Hannover EMRSA	POS	POS	POS	POS	NEG	NEG	NEG	NEG	NEG	NEG	NEG	NEG	NEG	NEG	NEG	POS	NEG	POS	POS	NEG	NEG	NEG	NEG	NEG	POS	NEG	NEG	NEG	NEG	NEG	NEG	NEG	POS	NEG	NEG	NEG	NEG	NEG	NEG	NEG	NEG	POS	NEG	NEG	NEG
08F01314	CC8-MRSA-IV, UK-EMRSA-14/WA MRSA-5	POS	POS	POS	NEG	NEG	NEG	NEG	NEG	NEG	NEG	NEG	NEG	NEG	NEG	NEG	POS	NEG	NEG	NEG	NEG	NEG	NEG	NEG	NEG	NEG	POS	NEG	NEG	POS	NEG	NEG	NEG	POS	NEG	POS	NEG	NEG	NEG	NEG	NEG	NEG	POS	NEG	NEG	NEG
08V15773	CC8-MRSA-IV, USA300	POS	POS	POS	NEG	NEG	NEG	NEG	NEG	NEG	NEG	NEG	NEG	NEG	NEG	NEG	NEG	NEG	NEG	NEG	NEG	NEG	NEG	NEG	NEG	NEG	NEG	NEG	NEG	NEG	NEG	NEG	NEG	POS	NEG	POS	NEG	NEG	NEG	NEG	NEG	NEG	POS	NEG	NEG	NEG
08B02942	CC22-MRSA-IV, Barnim/UK-EMRSA-15	POS			NEG		NEG	NEG	NEG			NEG	NEG	NEG	NEG	NEG	NEG	NEG	NEG	NEG	NEG	NEG		NEG	NEG	NEG																		NEG	NEG	NEG
08F00816	CC22-MRSA-IV, Barnim/UK-EMRSA-15	NEG			NEG		NEG	NEG	NEG			NEG	NEG	NEG	NEG	NEG	NEG	NEG	NEG	NEG	NEG	NEG		NEG	NEG	NEG																		NEG	NEG	NEG
08F01637	CC22-MRSA-IV, Barnim/UK-EMRSA-15	POS			NEG		NEG	NEG	NEG			NEG	NEG	NEG	NEG	NEG	NEG	NEG	NEG	NEG	NEG	NEG		NEG	NEG	NEG																		NEG	NEG	NEG
08F04948	CC22-MRSA-IV, Barnim/UK-EMRSA-15	POS			NEG		NEG	NEG	NEG			NEG	NEG	NEG	NEG	NEG	NEG	NEG	NEG	NEG	NEG	NEG		NEG	NEG	NEG																		NEG	NEG	NEG
08U07826	CC22-MRSA-IV, Barnim/UK-EMRSA-15	POS			NEG		POS	NEG	NEG			NEG	NEG	NEG	NEG	NEG	NEG	NEG	NEG	NEG	NEG	NEG		NEG	NEG	NEG																		NEG	NEG	NEG
08V00371	CC22-MRSA-IV, Barnim/UK-EMRSA-15	POS			NEG		POS	NEG	NEG			NEG	NEG	NEG	NEG	NEG	NEG	NEG	NEG	NEG	NEG	NEG		NEG	NEG	NEG																		NEG	NEG	NEG
08V02049	CC22-MRSA-IV, Barnim/UK-EMRSA-15	POS			NEG		NEG	NEG	NEG			NEG	NEG	NEG	NEG	NEG	NEG	NEG	NEG	NEG	NEG	NEG		NEG	NEG	NEG																		NEG	NEG	NEG
08V02349	CC22-MRSA-IV, Barnim/UK-EMRSA-15	POS			NEG		NEG	NEG	NEG			NEG	NEG	NEG	NEG	NEG	NEG	NEG	NEG	NEG	NEG	NEG		NEG	NEG	NEG																		NEG	NEG	NEG
08V03329	CC22-MRSA-IV, Barnim/UK-EMRSA-15	AMB			NEG		POS	NEG	NEG			NEG	NEG	NEG	NEG	NEG	NEG	NEG	NEG	NEG	NEG	NEG		NEG	NEG	NEG																		NEG	NEG	NEG
08V04516	CC22-MRSA-IV, Barnim/UK-EMRSA-15	POS			NEG		NEG	NEG	NEG			NEG	NEG	NEG	NEG	NEG	NEG	NEG	NEG	NEG	NEG	NEG		NEG	NEG	NEG																		NEG	NEG	NEG
08V04681	CC22-MRSA-IV, Barnim/UK-EMRSA-15	POS			NEG		NEG	NEG	NEG			NEG	NEG	NEG	NEG	NEG	NEG	NEG	NEG	NEG	NEG	NEG		NEG	NEG	NEG																		NEG	NEG	NEG
08V04740	CC22-MRSA-IV, Barnim/UK-EMRSA-15	POS			NEG		NEG	NEG	NEG			NEG	NEG	NEG	NEG	NEG	NEG	NEG	NEG	NEG	NEG	NEG		NEG	NEG	NEG																		NEG	NEG	NEG
08V04977	CC22-MRSA-IV, Barnim/UK-EMRSA-15	POS			NEG		POS	NEG	NEG			NEG	NEG	NEG	NEG	NEG	NEG	NEG	NEG	NEG	NEG	NEG		NEG	NEG	NEG																		NEG	NEG	NEG
08V06376	CC22-MRSA-IV, Barnim/UK-EMRSA-15	POS			NEG		POS	NEG	NEG			NEG	NEG	NEG	NEG	NEG	NEG	NEG	NEG	NEG	NEG	NEG		NEG	NEG	NEG																		NEG	NEG	NEG
08V09540	CC22-MRSA-IV, Barnim/UK-EMRSA-15	POS			NEG		NEG	NEG	NEG			NEG	NEG	NEG	NEG	NEG	NEG	NEG	NEG	NEG	NEG	NEG		NEG	NEG	NEG																		NEG	NEG	NEG
08V10287	CC22-MRSA-IV, Barnim/UK-EMRSA-15	NEG			NEG		POS	NEG	NEG			NEG	NEG	NEG	NEG	NEG	NEG	NEG	NEG	NEG	NEG	NEG		NEG	NEG	NEG																		NEG	NEG	NEG
08V10376	CC22-MRSA-IV, Barnim/UK-EMRSA-15	NEG			NEG		POS	NEG	NEG			NEG	NEG	NEG	NEG	NEG	NEG	NEG	NEG	NEG	NEG	NEG		NEG	NEG	NEG																		NEG	NEG	NEG
08V10714	CC22-MRSA-IV, Barnim/UK-EMRSA-15	AMB			NEG		POS	NEG	NEG			NEG	NEG	NEG	NEG	NEG	NEG	NEG	NEG	NEG	NEG	NEG		NEG	NEG	NEG																		NEG	NEG	NEG
08V10836	CC22-MRSA-IV, Barnim/UK-EMRSA-15	POS			NEG		POS	NEG	NEG			NEG	NEG	NEG	NEG	NEG	NEG	NEG	NEG	NEG	NEG	NEG		NEG	NEG	NEG																		NEG	NEG	NEG
08V11971	CC22-MRSA-IV, Barnim/UK-EMRSA-15	POS			NEG		POS	NEG	NEG			NEG	NEG	NEG	NEG	NEG	NEG	NEG	NEG	NEG	NEG	NEG		NEG	NEG	NEG																		NEG	NEG	NEG
08V14130	CC22-MRSA-IV, Barnim/UK-EMRSA-15	POS			NEG		POS	NEG	NEG			NEG	NEG	NEG	NEG	NEG	NEG	NEG	NEG	AMB	NEG	NEG		NEG	NEG	NEG																		NEG	NEG	NEG
08V14723	CC22-MRSA-IV, Barnim/UK-EMRSA-15	POS			NEG		POS	NEG	NEG			NEG	NEG	NEG	NEG	NEG	NEG	NEG	NEG	NEG	NEG	NEG		NEG	NEG	NEG																		NEG	NEG	NEG
08V16292	CC22-MRSA-IV, Barnim/UK-EMRSA-15	POS			NEG		POS	NEG	NEG			NEG	NEG	NEG	NEG	NEG	NEG	NEG	NEG	AMB	NEG	NEG		NEG	NEG	NEG																		NEG	NEG	NEG
08V16554	CC22-MRSA-IV, Barnim/UK-EMRSA-15	POS			NEG		NEG	NEG	NEG			NEG	NEG	NEG	NEG	NEG	NEG	NEG	NEG	NEG	NEG	NEG		NEG	NEG	NEG																		NEG	NEG	NEG
08V18482	CC22-MRSA-IV, Barnim/UK-EMRSA-15	POS			NEG		POS	NEG	NEG			NEG	NEG	NEG	NEG	NEG	NEG	NEG	NEG	AMB	NEG	NEG		NEG	NEG	NEG																		NEG	NEG	NEG
08V19658	CC22-MRSA-IV, Barnim/UK-EMRSA-15	POS			NEG		NEG	NEG	NEG			NEG	NEG	NEG	NEG	NEG	NEG	NEG	NEG	NEG	NEG	NEG		NEG	NEG	NEG																		NEG	NEG	NEG
08V19663	CC22-MRSA-IV, Barnim/UK-EMRSA-15	POS			NEG		NEG	NEG	NEG			NEG	NEG	NEG	NEG	NEG	NEG	NEG	NEG	NEG	NEG	NEG		NEG	NEG	NEG																		NEG	NEG	NEG
08V20266	CC22-MRSA-IV, Barnim/UK-EMRSA-15	POS			NEG		NEG	NEG	NEG			NEG	NEG	NEG	NEG	NEG	NEG	NEG	NEG	NEG	NEG	NEG		NEG	NEG	NEG																		NEG	NEG	NEG
08V20337	CC22-MRSA-IV, Barnim/UK-EMRSA-15	POS			NEG		NEG	NEG	NEG			NEG	NEG	NEG	NEG	NEG	NEG	NEG	NEG	NEG	NEG	NEG		NEG	NEG	NEG																		NEG	NEG	NEG
08V21055	CC22-MRSA-IV, Barnim/UK-EMRSA-15	POS			NEG		NEG	NEG	NEG			NEG	NEG	NEG	NEG	NEG	NEG	NEG	NEG	NEG	NEG	NEG		NEG	NEG	NEG																		NEG	NEG	NEG
08V23747	CC22-MRSA-IV, Barnim/UK-EMRSA-15	AMB			NEG		NEG	NEG	NEG			NEG	NEG	NEG	NEG	NEG	NEG	NEG	NEG	NEG	NEG	NEG		NEG	NEG	NEG																		NEG	NEG	NEG
08V23813	CC22-MRSA-IV, Barnim/UK-EMRSA-15	POS			NEG		NEG	NEG	NEG			NEG	NEG	NEG	NEG	NEG	NEG	NEG	NEG	NEG	NEG	NEG		NEG	NEG	NEG																		NEG	NEG	NEG
08V25135	CC22-MRSA-IV, Barnim/UK-EMRSA-15	POS			NEG		NEG	NEG	NEG			NEG	NEG	NEG	NEG	NEG	NEG	NEG	NEG	NEG	NEG	NEG		NEG	NEG	NEG																		NEG	NEG	NEG
08V27329	CC22-MRSA-IV, Barnim/UK-EMRSA-15	POS			NEG		POS	NEG	NEG			NEG	NEG	NEG	NEG	NEG	NEG	NEG	NEG	NEG	NEG	NEG		NEG	NEG	NEG																		NEG	NEG	NEG
08V27434	CC22-MRSA-IV, Barnim/UK-EMRSA-15	POS			NEG		POS	NEG	NEG			NEG	NEG	NEG	NEG	NEG	NEG	NEG	NEG	NEG	NEG	NEG		NEG	NEG	NEG																		NEG	NEG	NEG
08V27729	CC22-MRSA-IV, Barnim/UK-EMRSA-15	POS			NEG		NEG	NEG	NEG			NEG	NEG	NEG	NEG	NEG	NEG	NEG	NEG	NEG	NEG	NEG		NEG	NEG	NEG																		NEG	NEG	NEG
08V31445	CC22-MRSA-IV, Barnim/UK-EMRSA-15	POS			NEG		POS	NEG	NEG			NEG	NEG	NEG	NEG	NEG	NEG	NEG	NEG	NEG	NEG	NEG		NEG	NEG	NEG																		NEG	NEG	NEG
08V34812	CC22-MRSA-IV, Barnim/UK-EMRSA-15	POS			NEG		NEG	NEG	NEG			NEG	NEG	NEG	NEG	NEG	NEG	POS	NEG	NEG	NEG	NEG		NEG	NEG	NEG																		NEG	NEG	NEG
08V34813	CC22-MRSA-IV, Barnim/UK-EMRSA-15	POS			NEG		NEG	NEG	NEG			NEG	NEG	NEG	NEG	NEG	NEG	POS	NEG	NEG	NEG	NEG		NEG	NEG	NEG																		NEG	NEG	NEG
08V35361	CC22-MRSA-IV, Barnim/UK-EMRSA-15	POS			NEG		NEG	NEG	NEG			NEG	NEG	NEG	NEG	NEG	NEG	POS	NEG	NEG	NEG	NEG		NEG	NEG	NEG																		NEG	NEG	NEG
08V37931	CC22-MRSA-IV, Barnim/UK-EMRSA-15	POS			NEG		NEG	NEG	NEG			NEG	NEG	NEG	NEG	NEG	NEG	NEG	NEG	NEG	NEG	NEG		NEG	NEG	NEG																		NEG	NEG	NEG
08V45578	CC22-MRSA-IV, Barnim/UK-EMRSA-15	POS			NEG		POS	NEG	NEG			NEG	NEG	NEG	NEG	NEG	NEG	NEG	NEG	NEG	NEG	NEG		NEG	NEG	NEG																		NEG	NEG	NEG
08V47385	CC22-MRSA-IV, Barnim/UK-EMRSA-15	POS	POS	POS	NEG	NEG	NEG	NEG	NEG	NEG	NEG	NEG	NEG	NEG	NEG	NEG	NEG	NEG	NEG	NEG	NEG	NEG	NEG	NEG	NEG	NEG	NEG	NEG	NEG	NEG	NEG	NEG	NEG	NEG	NEG	NEG	NEG	NEG	NEG	NEG	NEG	NEG	NEG	NEG	NEG	NEG
08V53678	CC22-MRSA-IV, Barnim/UK-EMRSA-15	POS	POS	POS	NEG	NEG	NEG	NEG	NEG	NEG	NEG	NEG	NEG	NEG	NEG	NEG	NEG	NEG	NEG	NEG	NEG	NEG	NEG	NEG	NEG	NEG	NEG	NEG	NEG	NEG	NEG	NEG	NEG	NEG	NEG	NEG	NEG	NEG	NEG	NEG	NEG	NEG	NEG	NEG	NEG	NEG
08B09737	CC45-MRSA-IV, Berlin EMRSA	AMB			NEG		NEG	NEG	NEG			NEG	NEG	NEG	NEG	NEG	NEG	NEG	POS	POS	NEG	NEG		NEG	NEG	NEG																		NEG	NEG	NEG
08B14321	CC45-MRSA-IV, Berlin EMRSA	POS	POS	POS	NEG	NEG	POS	NEG	NEG	NEG	NEG	NEG	NEG	NEG	NEG	NEG	POS	POS	POS	POS	NEG	NEG	NEG	POS	NEG	NEG	NEG	NEG	NEG	NEG	NEG	NEG	NEG	NEG	NEG	NEG	POS	POS	NEG	NEG	NEG	NEG	POS	NEG	NEG	NEG
08F01439	CC45-MRSA-IV, Berlin EMRSA	POS			NEG		NEG	NEG	NEG			NEG	NEG	NEG	NEG	NEG	NEG	NEG	NEG	NEG	NEG	NEG		NEG	NEG	NEG																		NEG	NEG	NEG
08U12518	CC45-MRSA-IV, Berlin EMRSA	POS			POS		NEG	NEG	NEG			NEG	NEG	NEG	NEG	NEG	NEG	NEG	NEG	NEG	NEG	NEG		NEG	NEG	NEG																		NEG	NEG	NEG
08U13653	CC45-MRSA-IV, Berlin EMRSA	POS	POS	POS	NEG	NEG	NEG	NEG	NEG	NEG	NEG	NEG	NEG	NEG	NEG	NEG	NEG	NEG	POS	POS	NEG	NEG	NEG	NEG	NEG	NEG	NEG	NEG	NEG	NEG	NEG	NEG	NEG	NEG	NEG	NEG	NEG	NEG	NEG	NEG	NEG	NEG	POS	NEG	NEG	NEG
08V00675	CC45-MRSA-IV, Berlin EMRSA	POS			NEG		NEG	NEG	NEG			NEG	NEG	NEG	NEG	NEG	NEG	NEG	POS	POS	NEG	NEG		NEG	NEG	NEG																		NEG	NEG	NEG
08V02459	CC45-MRSA-IV, Berlin EMRSA	POS			NEG		NEG	NEG	NEG			NEG	NEG	NEG	NEG	NEG	NEG	NEG	POS	POS	NEG	NEG		NEG	NEG	NEG																		NEG	NEG	NEG
08V02654	CC45-MRSA-IV, Berlin EMRSA	POS			NEG		NEG	NEG	NEG			NEG	NEG	NEG	NEG	NEG	NEG	NEG	POS	POS	NEG	NEG		NEG	NEG	NEG																		NEG	NEG	NEG
08V05677	CC45-MRSA-IV, Berlin EMRSA	POS	POS	POS	NEG	NEG	NEG	NEG	NEG	NEG	NEG	NEG	NEG	NEG	NEG	NEG	NEG	NEG	POS	POS	NEG	NEG	NEG	NEG	NEG	NEG	NEG	NEG	NEG	NEG	NEG	NEG	NEG	NEG	NEG	NEG	NEG	NEG	NEG	NEG	NEG	NEG	POS	NEG	NEG	NEG
08V20930	CC45-MRSA-IV, Berlin EMRSA	POS			NEG		POS	NEG	NEG			NEG	NEG	NEG	NEG	NEG	NEG	NEG	POS	POS	NEG	NEG		NEG	NEG	NEG																		NEG	NEG	NEG
08V23911	CC45-MRSA-IV, Berlin EMRSA	POS			NEG		NEG	NEG	NEG			NEG	NEG	NEG	NEG	NEG	NEG	NEG	POS	POS	NEG	NEG		NEG	NEG	NEG																		NEG	NEG	NEG
08V39175	CC45-MRSA-IV, Berlin EMRSA	POS			NEG		POS	NEG	NEG			NEG	NEG	NEG	NEG	NEG	NEG	NEG	POS	POS	NEG	NEG		NEG	NEG	NEG																		NEG	NEG	NEG
08V39949	CC45-MRSA-IV, Berlin EMRSA	POS			NEG		POS	NEG	NEG			NEG	NEG	NEG	NEG	NEG	POS	POS	POS	POS	NEG	NEG		POS	NEG	NEG																		NEG	NEG	NEG
08V53670	CC88-MRSA-IV, WA MRSA-2	POS	POS	POS	POS	NEG	NEG	NEG	NEG	NEG	NEG	NEG	NEG	NEG	NEG	NEG	NEG	NEG	NEG	NEG	NEG	NEG	NEG	NEG	NEG	NEG	NEG	NEG	NEG	NEG	NEG	NEG	NEG	NEG	NEG	NEG	NEG	NEG	NEG	NEG	NEG	NEG	POS	NEG	NEG	NEG
08V06946	CC398-MRSA-V	POS	POS	POS	POS	NEG	NEG	NEG	NEG	NEG	NEG	NEG	NEG	NEG	NEG	NEG	NEG	NEG	NEG	NEG	NEG	NEG	NEG	NEG	NEG	POS	NEG	NEG	NEG	NEG	NEG	NEG	NEG	NEG	NEG	NEG	NEG	NEG	NEG	NEG	NEG	NEG	POS	NEG	NEG	NEG

ISOLATE:	STRAIN	VIRULENCE : TOX.SCHOCK.TOXIN			VIRULENCE : ENTEROTOXINS																					VIRULENCE : HLG AND LEUKOCIDINS												
		toxic shock syndrome toxin 1			enterotoxin A	enterotoxin A, allele from strain 320E	enterotoxin A, allele from strain N315 = entero-toxin P	enterotoxin B	enterotoxin C	enterotoxin D	enterotoxin E	enterotoxin G	enterotoxin H	enterotoxin I	enterotoxin J	enterotoxin K	enterotoxin L	enterotoxin M	enterotoxin N		enterotoxin O	enterotoxin Q	enterotoxin R	enterotoxin U	enterotoxin-like protein ORF CM14	haemolysin gamma / leukocidin, component B	haemolysin gamma / leukocidin, component C		haemolysin gamma, component A	Panton Valentine leukocidin F component	Panton Valentine leukocidin S component	F component from hypothetical leukocidin from ruminants	S component from hypothetical leukocidin from ruminants	leukocidin D component	leukocidin E component	leukocidin/ haemolysin toxin family protein	leukocidin/haemolysin toxin family protein	
		tst1 (consensus)	tst1 ("human" allele)	tst1 ("bovine" allele)	sea	sea (320E)	sea (N315) / sep	seb	sec	sed	sef	seg	seh	sei	sej	sek	sel	selm	seln (consensus)	seln (other than RF122)	selo	seq	ser	selu	ORF CM14	lukF	lukS	lukS (ST22+ST45)	hlgA	lukF-PV	lukS-PV	lukF-PV (P83)	lukM	lukD	lukE	lukX	lukY	lukY (ST30+ST45)
**2008**																																						
08V04188	CC5/ST228-MRSA-I, South German EMRSA		NEG	NEG	POS	NEG	NEG	NEG	NEG	NEG	NEG	NEG	NEG	NEG	NEG		NEG	NEG		NEG	NEG		NEG			POS	AMB		POS	NEG	NEG	NEG	NEG	NEG	NEG	AMB	POS	NEG
08V21565	CC5/ST228-MRSA-I, South German EMRSA		NEG	NEG	POS	NEG	NEG	NEG	NEG	NEG	NEG	POS	NEG	POS	NEG		NEG	POS		POS	AMB		NEG			POS	POS		POS	NEG	NEG	NEG	NEG	POS	POS	AMB	POS	NEG
08V53546	CC5/ST228-MRSA-I, South German EMRSA	NEG	NEG	NEG	POS	NEG	NEG	NEG	NEG	NEG	NEG	POS	NEG	POS	NEG	NEG	NEG	POS	POS	POS	POS	NEG	NEG	POS	NEG	POS	POS	AMB	POS	NEG	NEG	NEG	NEG	POS	POS	AMB	POS	NEG
08B04687	CC5-MRSA-II, Rhine-Hesse EMRSA/UK-EMRSA-3		NEG	NEG	NEG	NEG	POS	NEG	NEG	POS	NEG	POS	NEG	POS	POS		NEG	POS		POS	POS		AMB			POS	POS		POS	NEG	NEG	NEG	NEG	POS	POS	AMB	POS	NEG
08F01184	CC5-MRSA-II, Rhine-Hesse EMRSA/UK-EMRSA-3		NEG	NEG	NEG	NEG	POS	NEG	NEG	POS	NEG	POS	NEG	POS	POS		NEG	POS		POS	POS		POS			POS	POS		POS	NEG	NEG	NEG	NEG	POS	POS	POS	POS	NEG
08F01352	CC5-MRSA-II, Rhine-Hesse EMRSA/UK-EMRSA-3		NEG	NEG	NEG	NEG	POS	NEG	NEG	POS	NEG	POS	NEG	POS	POS		NEG	POS		POS	POS		AMB			POS	POS		POS	NEG	NEG	NEG	NEG	POS	POS	NEG	POS	NEG
08F01711	CC5-MRSA-II, Rhine-Hesse EMRSA/UK-EMRSA-3		NEG	NEG	NEG	NEG	POS	NEG	NEG	POS	NEG	POS	NEG	POS	POS		NEG	POS		POS	POS		POS			POS	POS		POS	NEG	NEG	AMB	NEG	POS	POS	POS	POS	NEG
08F03746	CC5-MRSA-II, Rhine-Hesse EMRSA/UK-EMRSA-3		NEG	NEG	NEG	NEG	POS	NEG	NEG	POS	NEG	POS	NEG	POS	POS		NEG	POS		POS	POS		POS			POS	POS		POS	NEG	NEG	AMB	NEG	POS	POS	POS	POS	NEG
08F04303	CC5-MRSA-II, Rhine-Hesse EMRSA/UK-EMRSA-3		NEG	NEG	NEG	NEG	NEG	NEG	NEG	POS	NEG	POS	NEG	POS	POS		NEG	POS		POS	POS		POS			POS	POS		POS	NEG	NEG	AMB	NEG	POS	POS	POS	POS	NEG
08F04396	CC5-MRSA-II, Rhine-Hesse EMRSA/UK-EMRSA-3		NEG	NEG	NEG	NEG	NEG	NEG	NEG	POS	NEG	POS	NEG	POS	POS		NEG	POS		POS	POS		POS			POS	POS		POS	NEG	NEG	NEG	NEG	POS	POS	POS	POS	NEG
08U11214	CC5-MRSA-II, Rhine-Hesse EMRSA/UK-EMRSA-3		NEG	NEG	NEG	NEG	NEG	NEG	NEG	POS	NEG	POS	NEG	POS	POS		NEG	POS		POS	AMB		POS			POS	POS		POS	NEG	NEG	NEG	NEG	POS	POS	AMB	POS	NEG
08V01047	CC5-MRSA-II, Rhine-Hesse EMRSA/UK-EMRSA-3		NEG	NEG	NEG	NEG	POS	NEG	NEG	POS	NEG	POS	NEG	POS	POS		NEG	POS		POS	POS		POS			POS	POS		POS	NEG	NEG	NEG	NEG	POS	POS	POS	POS	NEG
08V01222	CC5-MRSA-II, Rhine-Hesse EMRSA/UK-EMRSA-3		NEG	NEG	NEG	NEG	POS	NEG	NEG	NEG	NEG	POS	NEG	POS	NEG		NEG	POS		POS	POS		NEG			POS	POS		POS	NEG	NEG	NEG	NEG	POS	POS	AMB	POS	NEG
08V01315	CC5-MRSA-II, Rhine-Hesse EMRSA/UK-EMRSA-3		NEG	NEG	NEG	NEG	POS	NEG	NEG	POS	NEG	POS	NEG	POS	POS		NEG	POS		POS	POS		POS			POS	POS		POS	NEG	NEG	AMB	NEG	POS	POS	POS	POS	NEG
08V06126	CC5-MRSA-II, Rhine-Hesse EMRSA/UK-EMRSA-3		NEG	NEG	NEG	NEG	POS	NEG	NEG	POS	NEG	POS	NEG	POS	POS		NEG	POS		POS	POS		POS			POS	POS		POS	NEG	NEG	NEG	NEG	POS	POS	POS	POS	NEG
08V06171	CC5-MRSA-II, Rhine-Hesse EMRSA/UK-EMRSA-3		NEG	NEG	NEG	NEG	POS	NEG	NEG	POS	NEG	POS	NEG	POS	POS		NEG	POS		POS	POS		POS			POS	POS		POS	NEG	NEG	AMB	NEG	POS	POS	POS	POS	NEG
08V06623	CC5-MRSA-II, Rhine-Hesse EMRSA/UK-EMRSA-3		NEG	NEG	NEG	NEG	POS	NEG	NEG	POS	NEG	POS	NEG	POS	POS		NEG	POS		POS	POS		POS			POS	POS		POS	NEG	NEG	AMB	NEG	POS	POS	POS	POS	NEG
08V10539	CC5-MRSA-II, Rhine-Hesse EMRSA/UK-EMRSA-3		NEG	NEG	NEG	NEG	NEG	NEG	NEG	POS	NEG	POS	NEG	POS	POS		NEG	POS		POS	POS		POS			POS	POS		POS	NEG	NEG	NEG	NEG	POS	POS	POS	POS	NEG
08V11213	CC5-MRSA-II, Rhine-Hesse EMRSA/UK-EMRSA-3		NEG	NEG	NEG	NEG	POS	NEG	NEG	POS	NEG	POS	NEG	POS	POS		NEG	POS		POS	POS		POS			POS	POS		POS	NEG	NEG	AMB	NEG	POS	POS	POS	POS	NEG
08V12100	CC5-MRSA-II, Rhine-Hesse EMRSA/UK-EMRSA-3		NEG	NEG	NEG	NEG	POS	NEG	NEG	POS	NEG	POS	NEG	POS	POS		NEG	POS		POS	POS		POS			POS	POS		POS	NEG	NEG	AMB	NEG	POS	POS	POS	POS	NEG
08V13847	CC5-MRSA-II, Rhine-Hesse EMRSA/UK-EMRSA-3		NEG	NEG	NEG	NEG	POS	NEG	NEG	POS	NEG	POS	NEG	POS	POS		NEG	POS		POS	POS		POS			POS	POS		POS	NEG	NEG	AMB	NEG	POS	POS	POS	POS	NEG
08V15905	CC5-MRSA-II, Rhine-Hesse EMRSA/UK-EMRSA-3	NEG	NEG	NEG	NEG	NEG	POS	NEG	NEG	NEG	NEG	POS	NEG	POS	NEG	NEG	NEG	POS	POS	POS	POS	NEG	NEG	POS	NEG	POS	POS	POS	POS	NEG	NEG	NEG	NEG	POS	POS	POS	POS	NEG
08V18191	CC5-MRSA-II, Rhine-Hesse EMRSA/UK-EMRSA-3	NEG	NEG	NEG	NEG	NEG	NEG	NEG	NEG	POS	NEG	POS	NEG	POS	POS	NEG	NEG	POS	POS	POS	POS	NEG	POS	POS	NEG	POS	POS	POS	POS	NEG	NEG	NEG	NEG	POS	POS	POS	POS	NEG
08V21425	CC5-MRSA-II, Rhine-Hesse EMRSA/UK-EMRSA-3		NEG	NEG	NEG	NEG	POS	NEG	NEG	POS	NEG	POS	NEG	POS	POS		NEG	POS		AMB	AMB		POS			POS	POS		POS	NEG	NEG	NEG	NEG	POS	POS	POS	POS	NEG
08V23868	CC5-MRSA-II, Rhine-Hesse EMRSA/UK-EMRSA-3		NEG	NEG	NEG	NEG	POS	NEG	NEG	NEG	NEG	POS	NEG	POS	NEG		NEG	POS		POS	POS		NEG			POS	POS		POS	NEG	NEG	NEG	NEG	POS	POS	POS	POS	NEG
08V27435	CC5-MRSA-II, Rhine-Hesse EMRSA/UK-EMRSA-3		NEG	NEG	NEG	NEG	POS	NEG	NEG	POS	NEG	POS	NEG	POS	POS		NEG	POS		POS	POS		AMB			POS	POS		POS	NEG	NEG	NEG	NEG	POS	POS	AMB	POS	NEG
08V27514	CC5-MRSA-II, Rhine-Hesse EMRSA/UK-EMRSA-3		NEG	NEG	NEG	NEG	POS	NEG	NEG	POS	NEG	POS	NEG	POS	POS		NEG	POS		POS	POS		POS			POS	POS		POS	NEG	NEG	NEG	NEG	POS	POS	POS	POS	NEG
08V28482	CC5-MRSA-II, Rhine-Hesse EMRSA/UK-EMRSA-3		NEG	NEG	NEG	NEG	POS	NEG	NEG	POS	NEG	POS	NEG	POS	POS		NEG	POS		POS	POS		POS			POS	POS		POS	NEG	NEG	AMB	NEG	POS	POS	POS	POS	NEG
08V29312	CC5-MRSA-II, Rhine-Hesse EMRSA/UK-EMRSA-3		NEG	NEG	NEG	NEG	POS	NEG	NEG	POS	NEG	POS	NEG	POS	POS		NEG	POS		POS	POS		POS			POS	POS		POS	NEG	NEG	NEG	NEG	POS	POS	POS	POS	NEG
08V29889	CC5-MRSA-II, Rhine-Hesse EMRSA/UK-EMRSA-3		NEG	NEG	NEG	NEG	POS	NEG	NEG	POS	NEG	POS	NEG	POS	POS		NEG	POS		POS	POS		POS			POS	POS		POS	NEG	NEG	AMB	NEG	POS	POS	POS	POS	NEG
08V30696	CC5-MRSA-II, Rhine-Hesse EMRSA/UK-EMRSA-3		NEG	NEG	NEG	NEG	POS	NEG	NEG	POS	NEG	POS	NEG	POS	POS		NEG	POS		POS	POS		POS			POS	POS		POS	NEG	NEG	NEG	NEG	POS	POS	POS	POS	NEG
08V32764	CC5-MRSA-II, Rhine-Hesse EMRSA/UK-EMRSA-3		NEG	NEG	NEG	NEG	POS	NEG	NEG	NEG	NEG	POS	NEG	POS	NEG		NEG	POS		POS	POS		NEG			POS	POS		POS	NEG	NEG	AMB	NEG	POS	POS	POS	POS	NEG
08V36052	CC5-MRSA-II, Rhine-Hesse EMRSA/UK-EMRSA-3		NEG	NEG	NEG	NEG	POS	NEG	NEG	POS	NEG	POS	NEG	POS	POS		NEG	POS		POS	POS		POS			POS	POS		POS	NEG	NEG	NEG	NEG	POS	POS	AMB	POS	NEG
08V36125	CC5-MRSA-II, Rhine-Hesse EMRSA/UK-EMRSA-3		NEG	NEG	NEG	NEG	NEG	NEG	NEG	POS	NEG	POS	NEG	POS	POS		NEG	POS		POS	POS		POS			POS	POS		POS	NEG	NEG	NEG	NEG	POS	POS	AMB	POS	NEG
08V39935	CC5-MRSA-II, Rhine-Hesse EMRSA/UK-EMRSA-3		NEG	NEG	NEG	NEG	POS	NEG	NEG	POS	NEG	POS	NEG	POS	POS		NEG	POS		POS	POS		POS			POS	POS		POS	NEG	NEG	NEG	NEG	POS	POS	POS	POS	NEG
08V41631	CC5-MRSA-II, Rhine-Hesse EMRSA/UK-EMRSA-3		NEG	NEG	NEG	NEG	POS	NEG	NEG	POS	NEG	POS	NEG	POS	POS		NEG	POS		POS	POS		POS			POS	POS		POS	NEG	NEG	NEG	NEG	POS	POS	POS	POS	NEG
08V42588	CC5-MRSA-II, Rhine-Hesse EMRSA/UK-EMRSA-3		NEG	NEG	NEG	NEG	NEG	NEG	NEG	POS	NEG	POS	NEG	POS	POS		NEG	POS		POS	POS		POS			POS	POS		POS	NEG	NEG	NEG	NEG	POS	POS	AMB	POS	NEG
08V42633	CC5-MRSA-II, Rhine-Hesse EMRSA/UK-EMRSA-3		NEG	NEG	NEG	NEG	POS	NEG	NEG	POS	NEG	POS	NEG	POS	POS		NEG	POS		POS	POS		POS			POS	POS		POS	NEG	NEG	NEG	NEG	POS	POS	AMB	POS	NEG
08V43410	CC5-MRSA-II, Rhine-Hesse EMRSA/UK-EMRSA-3		NEG	NEG	NEG	NEG	POS	NEG	NEG	NEG	NEG	POS	NEG	POS	NEG		NEG	POS		POS	POS		NEG			POS	POS		POS	NEG	NEG	NEG	NEG	POS	POS	POS	POS	NEG
08V43581	CC5-MRSA-II, Rhine-Hesse EMRSA/UK-EMRSA-3		NEG	NEG	NEG	NEG	POS	NEG	NEG	POS	NEG	POS	NEG	POS	POS		NEG	POS		POS	POS		POS			POS	POS		POS	NEG	NEG	NEG	NEG	POS	POS	POS	POS	NEG
08V47242	CC5-MRSA-II, Rhine-Hesse EMRSA/UK-EMRSA-3	NEG	NEG	NEG	NEG	NEG	POS	NEG	NEG	POS	NEG	POS	NEG	POS	POS	NEG	NEG	POS	POS	POS	POS	NEG	POS	POS	NEG	POS	POS	AMB	POS	NEG	NEG	NEG	NEG	POS	POS	POS	POS	NEG
08V50740	CC5-MRSA-II, Rhine-Hesse EMRSA/UK-EMRSA-3	NEG	NEG	NEG	NEG	NEG	POS	NEG	NEG	POS	NEG	AMB	NEG	POS	POS	NEG	NEG	POS	POS	POS	POS	NEG	POS	POS	NEG	AMB	POS	NEG	POS	NEG	NEG	NEG	NEG	POS	POS	AMB	POS	NEG
08V02855	CC7-MRSA-IV	NEG	NEG	NEG	NEG	NEG	POS	NEG	NEG	NEG	NEG	NEG	NEG	NEG	NEG	NEG	NEG	NEG	NEG	NEG	NEG	NEG	NEG	NEG	NEG	POS	NEG	POS	POS	NEG	NEG	NEG	NEG	POS	POS	POS	POS	NEG
08V35987	CC8/ST239-MRSA-III, Vienna/Hungarian/Brazilian Clone	NEG	NEG	NEG	POS	NEG	NEG	NEG	NEG	NEG	NEG	NEG	NEG	NEG	NEG	POS	NEG	NEG	NEG	NEG	NEG	POS	NEG	NEG	NEG	POS	POS	NEG	POS	NEG	NEG	NEG	NEG	POS	POS	AMB	POS	NEG
08V39853	CC8/ST254-MRSA-(atypical SCCmec), Hannover EMRSA	NEG	NEG	NEG	NEG	NEG	NEG	POS	NEG	NEG	NEG	NEG	NEG	NEG	NEG	POS	NEG	NEG	NEG	NEG	NEG	POS	NEG	NEG	NEG	POS	POS	NEG	POS	NEG	NEG	NEG	NEG	POS	POS	POS	POS	NEG
08F01314	CC8-MRSA-IV, UK-EMRSA-14/WA MRSA-5	NEG	NEG	NEG	NEG	NEG	NEG	NEG	NEG	NEG	NEG	NEG	NEG	NEG	NEG	NEG	NEG	NEG	NEG	NEG	NEG	NEG	NEG	NEG	NEG	POS	POS	NEG	POS	NEG	NEG	NEG	NEG	POS	POS	POS	POS	NEG
08V15773	CC8-MRSA-IV, USA300	NEG	NEG	NEG	NEG	NEG	NEG	NEG	NEG	NEG	NEG	NEG	NEG	NEG	NEG	POS	NEG	NEG	NEG	NEG	NEG	POS	NEG	NEG	NEG	POS	POS	NEG	POS	POS	POS	NEG	NEG	POS	POS	POS	POS	NEG
08B02942	CC22-MRSA-IV, Barnim/UK-EMRSA-15		NEG	NEG	NEG	NEG	NEG	NEG	POS	NEG	NEG	POS	NEG	POS	NEG		POS	POS		POS	POS		NEG			AMB	NEG		AMB	NEG	NEG	NEG	NEG	NEG	NEG	POS	POS	NEG
08F00816	CC22-MRSA-IV, Barnim/UK-EMRSA-15		NEG	NEG	NEG	NEG	NEG	NEG	NEG	NEG	NEG	POS	NEG	POS	NEG		NEG	POS		NEG	NEG		NEG			NEG	NEG		NEG	NEG	NEG	NEG	NEG	NEG	NEG	NEG	POS	NEG
08F01637	CC22-MRSA-IV, Barnim/UK-EMRSA-15		NEG	NEG	NEG	NEG	NEG	AMB	POS	NEG	NEG	POS	NEG	POS	NEG		POS	POS		POS	POS		NEG			POS	NEG		AMB	NEG	NEG	NEG	NEG	NEG	NEG	POS	POS	NEG
08F04948	CC22-MRSA-IV, Barnim/UK-EMRSA-15		NEG	NEG	NEG	NEG	NEG	NEG	POS	NEG	NEG	POS	NEG	POS	NEG		POS	POS		POS	POS		NEG			POS	NEG		AMB	NEG	NEG	NEG	NEG	NEG	NEG	POS	POS	NEG
08U07826	CC22-MRSA-IV, Barnim/UK-EMRSA-15		NEG	NEG	NEG	NEG	NEG	NEG	NEG	NEG	NEG	POS	NEG	POS	NEG		NEG	POS		POS	POS		NEG			AMB	NEG		NEG	NEG	NEG	NEG	NEG	NEG	NEG	POS	POS	NEG
08V00371	CC22-MRSA-IV, Barnim/UK-EMRSA-15		NEG	NEG	NEG	NEG	NEG	NEG	NEG	NEG	NEG	AMB	NEG	POS	NEG		NEG	POS		POS	POS		NEG			NEG	NEG		POS	NEG	NEG	NEG	NEG	NEG	NEG	NEG	POS	NEG
08V02049	CC22-MRSA-IV, Barnim/UK-EMRSA-15		NEG	NEG	NEG	NEG	NEG	NEG	NEG	NEG	NEG	POS	NEG	POS	NEG		NEG	POS		POS	POS		NEG			POS	NEG		AMB	NEG	NEG	NEG	NEG	NEG	NEG	POS	POS	NEG
08V02349	CC22-MRSA-IV, Barnim/UK-EMRSA-15		NEG	NEG	NEG	NEG	NEG	NEG	NEG	NEG	NEG	POS	NEG	POS	NEG		NEG	POS		POS	POS		NEG			POS	NEG		AMB	NEG	NEG	NEG	NEG	NEG	NEG	POS	POS	NEG
08V03329	CC22-MRSA-IV, Barnim/UK-EMRSA-15		NEG	NEG	NEG	NEG	NEG	NEG	NEG	NEG	NEG	NEG	NEG	POS	NEG		NEG	POS		POS	POS		NEG			NEG	NEG		NEG	NEG	NEG	NEG	NEG	NEG	NEG	NEG	POS	NEG
08V04516	CC22-MRSA-IV, Barnim/UK-EMRSA-15		NEG	NEG	NEG	NEG	NEG	NEG	NEG	NEG	NEG	POS	NEG	POS	NEG		NEG	POS		POS	POS		NEG			AMB	NEG		POS	NEG	NEG	NEG	NEG	NEG	NEG	AMB	POS	NEG
08V04681	CC22-MRSA-IV, Barnim/UK-EMRSA-15		NEG	NEG	NEG	NEG	NEG	NEG	NEG	NEG	NEG	POS	NEG	POS	NEG		NEG	POS		POS	POS		NEG			POS	NEG		POS	NEG	NEG	NEG	NEG	NEG	NEG	POS	POS	NEG
08V04740	CC22-MRSA-IV, Barnim/UK-EMRSA-15		NEG	NEG	NEG	NEG	NEG	NEG	NEG	NEG	NEG	POS	NEG	POS	NEG		NEG	POS		POS	POS		NEG			POS	NEG		POS	NEG	NEG	NEG	NEG	NEG	NEG	POS	POS	NEG
08V04977	CC22-MRSA-IV, Barnim/UK-EMRSA-15		NEG	NEG	NEG	NEG	NEG	NEG	POS	NEG	NEG	POS	NEG	POS	NEG		POS	POS		POS	POS		NEG			AMB	NEG		AMB	NEG	NEG	NEG	NEG	NEG	NEG	POS	POS	NEG
08V06376	CC22-MRSA-IV, Barnim/UK-EMRSA-15		NEG	NEG	NEG	NEG	NEG	NEG	NEG	NEG	NEG	POS	NEG	POS	NEG		NEG	POS		POS	POS		NEG			POS	NEG		NEG	NEG	NEG	NEG	NEG	NEG	NEG	POS	POS	NEG
08V09540	CC22-MRSA-IV, Barnim/UK-EMRSA-15		NEG	NEG	NEG	NEG	NEG	NEG	NEG	NEG	NEG	POS	NEG	POS	NEG		NEG	POS		POS	POS		NEG			NEG	NEG		NEG	NEG	NEG	NEG	NEG	NEG	NEG	AMB	POS	NEG
08V10287	CC22-MRSA-IV, Barnim/UK-EMRSA-15		NEG	NEG	NEG	NEG	NEG	NEG	NEG	NEG	NEG	POS	NEG	POS	NEG		NEG	POS		AMB	AMB		NEG			NEG	NEG		NEG	NEG	NEG	NEG	NEG	NEG	NEG	NEG	POS	NEG
08V10376	CC22-MRSA-IV, Barnim/UK-EMRSA-15		NEG	NEG	NEG	NEG	NEG	NEG	NEG	NEG	NEG	POS	NEG	POS	NEG		NEG	POS		POS	AMB		NEG			NEG	NEG		NEG	NEG	NEG	NEG	NEG	NEG	NEG	AMB	POS	NEG
08V10714	CC22-MRSA-IV, Barnim/UK-EMRSA-15		NEG	NEG	NEG	NEG	NEG	NEG	NEG	NEG	NEG	POS	NEG	POS	NEG		NEG	POS		POS	AMB		NEG			NEG	NEG		NEG	NEG	NEG	NEG	NEG	NEG	NEG	AMB	POS	NEG
08V10836	CC22-MRSA-IV, Barnim/UK-EMRSA-15		NEG	NEG	NEG	NEG	NEG	NEG	NEG	NEG	NEG	POS	NEG	POS	NEG		NEG	POS		POS	POS		NEG			POS	NEG		POS	NEG	NEG	NEG	NEG	NEG	NEG	POS	POS	NEG
08V11971	CC22-MRSA-IV, Barnim/UK-EMRSA-15		NEG	NEG	NEG	NEG	NEG	NEG	NEG	NEG	NEG	POS	NEG	POS	NEG		NEG	POS		POS	POS		NEG			NEG	NEG		POS	NEG	NEG	NEG	NEG	NEG	NEG	POS	POS	NEG
08V14130	CC22-MRSA-IV, Barnim/UK-EMRSA-15		NEG	NEG	NEG	NEG	NEG	NEG	NEG	NEG	NEG	POS	NEG	POS	NEG		NEG	POS		POS	POS		NEG			POS	NEG		AMB	NEG	NEG	NEG	NEG	NEG	NEG	POS	POS	NEG
08V14723	CC22-MRSA-IV, Barnim/UK-EMRSA-15		NEG	NEG	NEG	NEG	NEG	NEG	NEG	NEG	NEG	POS	NEG	POS	NEG		NEG	POS		POS	POS		NEG			AMB	NEG	NEG	NEG	NEG	NEG	NEG	NEG	NEG	NEG	POS	POS	NEG
08V16292	CC22-MRSA-IV, Barnim/UK-EMRSA-15		NEG	NEG	NEG	NEG	NEG	NEG	NEG	NEG	NEG	POS	NEG	POS	NEG		NEG	POS		POS	POS		NEG			AMB	NEG		POS	NEG	NEG	NEG	NEG	NEG	NEG	POS	POS	NEG
08V16554	CC22-MRSA-IV, Barnim/UK-EMRSA-15		NEG	NEG	NEG	NEG	NEG	NEG	NEG	NEG	NEG	POS	NEG	POS	NEG		NEG	POS		POS	POS		NEG			AMB	NEG		POS	NEG	NEG	NEG	NEG	NEG	NEG	POS	POS	NEG
08V18482	CC22-MRSA-IV, Barnim/UK-EMRSA-15		NEG	NEG	NEG	NEG	NEG	NEG	NEG	NEG	NEG	POS	NEG	POS	NEG		NEG	POS		POS	POS		NEG			POS	NEG		AMB	NEG	NEG	NEG	NEG	NEG	NEG	POS	POS	NEG
08V19658	CC22-MRSA-IV, Barnim/UK-EMRSA-15		NEG	NEG	NEG	NEG	NEG	NEG	NEG	NEG	NEG	POS	NEG	POS	NEG		NEG	POS		POS	POS		NEG			NEG	NEG		NEG	NEG	NEG	NEG	NEG	NEG	NEG	AMB	POS	NEG
08V19663	CC22-MRSA-IV, Barnim/UK-EMRSA-15		NEG	NEG	NEG	NEG	NEG	NEG	NEG	NEG	NEG	POS	NEG	POS	NEG		NEG	POS		POS	POS		NEG			NEG	NEG		NEG	NEG	NEG	NEG	NEG	NEG	NEG	AMB	POS	NEG
08V20266	CC22-MRSA-IV, Barnim/UK-EMRSA-15		NEG	NEG	NEG	NEG	NEG	NEG	NEG	NEG	NEG	POS	NEG	POS	NEG		NEG	POS		POS	POS		NEG			NEG	NEG		NEG	NEG	NEG	NEG	NEG	NEG	NEG	AMB	POS	NEG
08V20337	CC22-MRSA-IV, Barnim/UK-EMRSA-15		NEG	NEG	NEG	NEG	NEG	NEG	NEG	NEG	NEG	POS	NEG	POS	NEG		NEG	POS		POS	POS		NEG			NEG	NEG		NEG	NEG	NEG	NEG	NEG	NEG	NEG	POS	POS	NEG
08V21055	CC22-MRSA-IV, Barnim/UK-EMRSA-15		NEG	NEG	NEG	NEG	NEG	NEG	POS	NEG	NEG	POS	NEG	POS	NEG		POS	POS		POS	POS		NEG			NEG	NEG		NEG	NEG	NEG	NEG	NEG	NEG	NEG	POS	POS	NEG
08V23747	CC22-MRSA-IV, Barnim/UK-EMRSA-15		NEG	NEG	NEG	NEG	NEG	NEG	NEG	NEG	NEG	POS	NEG	POS	NEG		NEG	POS		POS	POS		NEG			NEG	NEG		NEG	NEG	NEG	NEG	NEG	NEG	NEG	AMB	POS	NEG
08V23813	CC22-MRSA-IV, Barnim/UK-EMRSA-15		NEG	NEG	NEG	NEG	NEG	NEG	NEG	NEG	NEG	POS	NEG	POS	NEG		NEG	POS		POS	POS		NEG			NEG	NEG		NEG	NEG	NEG	NEG	NEG	NEG	NEG	POS	POS	NEG
08V25135	CC22-MRSA-IV, Barnim/UK-EMRSA-15		NEG	NEG	NEG	NEG	NEG	NEG	NEG	NEG	NEG	POS	NEG	POS	NEG		NEG	POS		POS	POS		NEG			AMB	NEG		AMB	NEG	NEG	NEG	NEG	NEG	NEG	AMB	POS	NEG
08V27329	CC22-MRSA-IV, Barnim/UK-EMRSA-15		NEG	NEG	NEG	NEG	NEG	NEG	NEG	NEG	NEG	POS	NEG	POS	NEG		NEG	POS		POS	POS		NEG			POS	NEG		POS	NEG	NEG	NEG	NEG	NEG	NEG	POS	POS	NEG
08V27434	CC22-MRSA-IV, Barnim/UK-EMRSA-15		NEG	NEG	NEG	NEG	NEG	NEG	NEG	NEG	NEG	POS	NEG	POS	NEG		NEG	POS		POS	POS		NEG			POS	NEG		POS	NEG	NEG	NEG	NEG	NEG	NEG	POS	POS	NEG
08V27729	CC22-MRSA-IV, Barnim/UK-EMRSA-15		NEG	NEG	NEG	NEG	NEG	NEG	NEG	NEG	NEG	POS	NEG	POS	NEG		NEG	POS		POS	POS		NEG			POS	NEG		AMB	NEG	NEG	NEG	NEG	NEG	NEG	POS	POS	NEG
08V31445	CC22-MRSA-IV, Barnim/UK-EMRSA-15		NEG	NEG	NEG	NEG	NEG	NEG	NEG	NEG	NEG	POS	NEG	POS	NEG		NEG	POS		POS	POS		NEG			AMB	NEG		NEG	NEG	NEG	NEG	NEG	NEG	NEG	POS	POS	NEG
08V34812	CC22-MRSA-IV, Barnim/UK-EMRSA-15		NEG	NEG	NEG	NEG	NEG	NEG	NEG	NEG	NEG	POS	NEG	POS	NEG		NEG	POS		POS	POS		NEG			AMB	NEG		NEG	NEG	NEG	NEG	NEG	NEG	NEG	POS	POS	NEG
08V34813	CC22-MRSA-IV, Barnim/UK-EMRSA-15		NEG	NEG	NEG	NEG	NEG	NEG	NEG	NEG	NEG	POS	NEG	POS	NEG		NEG	POS		POS	POS		NEG			POS	NEG		AMB	NEG	NEG	NEG	NEG	NEG	NEG	POS	POS	NEG
08V35361	CC22-MRSA-IV, Barnim/UK-EMRSA-15		NEG	NEG	NEG	NEG	NEG	NEG	NEG	NEG	NEG	POS	NEG	POS	NEG		NEG	POS		POS	POS		NEG			NEG	NEG		NEG	NEG	NEG	NEG	NEG	NEG	NEG	POS	POS	NEG
08V37931	CC22-MRSA-IV, Barnim/UK-EMRSA-15		NEG	NEG	NEG	NEG	NEG	NEG	NEG	NEG	NEG	POS	NEG	POS	NEG		NEG	POS		POS	POS		NEG			POS	NEG		POS	NEG	NEG	NEG	NEG	NEG	NEG	POS	POS	NEG
08V45578	CC22-MRSA-IV, Barnim/UK-EMRSA-15		NEG	NEG	NEG	NEG	NEG	NEG	NEG	NEG	NEG	POS	NEG	POS	NEG		NEG	POS		POS	POS		NEG			POS	AMB		POS	NEG	NEG	NEG	NEG	NEG	NEG	AMB	POS	NEG
08V47385	CC22-MRSA-IV, Barnim/UK-EMRSA-15	NEG	NEG	NEG	NEG	NEG	NEG	NEG	NEG	NEG	NEG	POS	NEG	POS	NEG	NEG	NEG	POS	POS	POS	POS	NEG	NEG	POS	NEG	POS	AMB	POS	POS	NEG	NEG	NEG	NEG	NEG	NEG	POS	POS	NEG
08V53678	CC22-MRSA-IV, Barnim/UK-EMRSA-15	NEG	NEG	NEG	NEG	NEG	NEG	NEG	NEG	NEG	NEG	POS	NEG	POS	NEG	NEG	NEG	POS	POS	POS	POS	NEG	NEG	POS	NEG	AMB	NEG	POS	AMB	NEG	NEG	NEG	NEG	NEG	NEG	POS	POS	NEG
08B09737	CC45-MRSA-IV, Berlin EMRSA		NEG	NEG	NEG	NEG	NEG	NEG	NEG	NEG	NEG	NEG	NEG	POS	NEG		NEG	POS		POS	NEG		NEG			POS	NEG		POS	NEG	NEG	NEG	NEG	NEG	NEG	NEG	NEG	POS
08B14321	CC45-MRSA-IV, Berlin EMRSA	NEG	NEG	NEG	NEG	NEG	NEG	NEG	NEG	NEG	NEG	NEG	NEG	POS	NEG	NEG	NEG	POS	POS	POS	POS	NEG	NEG	POS	NEG	POS	AMB	POS	POS	NEG	NEG	NEG	NEG	NEG	NEG	POS	NEG	POS
08F01439	CC45-MRSA-IV, Berlin EMRSA		NEG	NEG	NEG	NEG	NEG	NEG	NEG	NEG	NEG	NEG	NEG	POS	NEG		NEG	POS		POS	POS		NEG			POS	NEG		POS	NEG	NEG	NEG	NEG	NEG	NEG	POS	NEG	POS
08U12518	CC45-MRSA-IV, Berlin EMRSA		NEG	NEG	NEG	NEG	NEG	NEG	NEG	NEG	NEG	POS	NEG	POS	NEG		NEG	POS		POS	POS		NEG			POS	NEG		POS	NEG	NEG	NEG	NEG	NEG	NEG	POS	NEG	POS
08U13653	CC45-MRSA-IV, Berlin EMRSA	NEG	NEG	NEG	NEG	NEG	NEG	NEG	NEG	NEG	NEG	POS	NEG	POS	NEG	NEG	NEG	POS	POS	POS	POS	NEG	NEG	POS	NEG	POS	POS	POS	POS	NEG	NEG	NEG	NEG	NEG	NEG	POS	NEG	POS
08V00675	CC45-MRSA-IV, Berlin EMRSA		NEG	NEG	NEG	NEG	NEG	NEG	NEG	NEG	NEG	POS	NEG	POS	NEG		NEG	POS		POS	POS		NEG			POS	NEG		POS	NEG	NEG	NEG	NEG	NEG	NEG	POS	NEG	POS
08V02459	CC45-MRSA-IV, Berlin EMRSA		NEG	NEG	NEG	NEG	NEG	NEG	NEG	NEG	NEG	NEG	NEG	POS	NEG		NEG	POS		POS	POS		NEG			POS	NEG		POS	NEG	NEG	NEG	NEG	NEG	NEG	POS	NEG	POS
08V02654	CC45-MRSA-IV, Berlin EMRSA		NEG	NEG	NEG	NEG	NEG	NEG	NEG	NEG	NEG	POS	NEG	POS	NEG		NEG	POS		POS	POS		NEG			POS	NEG		POS	NEG	NEG	NEG	NEG	NEG	NEG	POS	NEG	POS
08V05677	CC45-MRSA-IV, Berlin EMRSA	NEG	NEG	NEG	NEG	NEG	NEG	NEG	NEG	NEG	NEG	NEG	NEG	POS	NEG	NEG	NEG	POS	POS	POS	POS	NEG	NEG	POS	NEG	POS	NEG	POS	POS	NEG	NEG	NEG	NEG	NEG	NEG	POS	NEG	POS
08V20930	CC45-MRSA-IV, Berlin EMRSA		NEG	NEG	NEG	NEG	NEG	NEG	NEG	NEG	NEG	POS	NEG	POS	NEG		NEG	POS		POS	POS		NEG			POS	NEG		POS	NEG	NEG	NEG	NEG	NEG	NEG	POS	NEG	POS
08V23911	CC45-MRSA-IV, Berlin EMRSA		NEG	NEG	NEG	NEG	NEG	NEG	NEG	NEG	NEG	POS	NEG	POS	NEG		NEG	POS		POS	POS		NEG			POS	NEG		POS	NEG	NEG	NEG	NEG	NEG	NEG	POS	NEG	POS
08V39175	CC45-MRSA-IV, Berlin EMRSA		NEG	NEG	NEG	NEG	NEG	NEG	NEG	NEG	NEG	NEG	NEG	POS	NEG		NEG	POS		POS	POS		NEG			POS	NEG		POS	NEG	NEG	NEG	NEG	NEG	NEG	POS	NEG	POS
08V39949	CC45-MRSA-IV, Berlin EMRSA		NEG	NEG	NEG	NEG	NEG	NEG	NEG	NEG	NEG	NEG	NEG	POS	NEG		NEG	POS		POS	POS		NEG			POS	NEG		POS	NEG	NEG	NEG	NEG	NEG	NEG	POS	NEG	POS
08V53670	CC88-MRSA-IV, WA MRSA-2	NEG	NEG	NEG	NEG	NEG	NEG	AMB	POS	NEG	NEG	NEG	NEG	NEG	NEG	NEG	POS	NEG	NEG	NEG	NEG	NEG	NEG	NEG	NEG	POS	POS	AMB	POS	NEG	NEG	NEG	NEG	POS	POS	AMB	POS	NEG
08V06946	CC398-MRSA-V	NEG	NEG	NEG	NEG	NEG	NEG	NEG	NEG	NEG	NEG	NEG	NEG	NEG	NEG	NEG	NEG	NEG	NEG	NEG	NEG	NEG	NEG	NEG	NEG	POS	POS	AMB	POS	NEG	NEG	NEG	NEG	NEG	NEG	AMB	POS	NEG

ISOLATE:	STRAIN	VIRULENCE : HAEMOLYSINS								VIRULENCE : HLB-CONV PHAGES			VIRULENCE : EXFOL.TOXINS			VIRULENCE : EPITHEL. DIFF. INHIB			VIRULENCE : ACME LOCUS				VIRULENCE : PROTEASES										VIRULENCE : STAPHYLOCOCCAL SUPERANTIGEN/ENTEROTOXIN-LIKE GENES (SET/SSL)						
		putative membrane protein	haemolysin alpha	putative membrane protein		haemolysin beta				staphylo-kinase	chemotaxis-inhibiting protein (CHIPS)	Staphyl. Comple-ment inhibitor	exfoliative toxin serotype A	exfoliative toxin serotype B	exfoliative toxin D	epidermal cell differen-tiation inhibitor	epidermal cell differen-tiation inhibitor B	epidermal cell differen-tiation inhibitor C	ACME-locus	ACME-locus: ornithincarb-amoyltrans-ferase	ACME-locus: carbamat-kinase	ACME-locus: arginine/orni-thine-antiporter	aureolysin			serin- protease A	serin- protease B	serin- protease E	glutamylendopeptidase	staphopain B, protease	staphopain A (staphylopain A), protease		staphyl. exotoxin-like protein	staphylococcal superantigen-like protein 1					
		hl	hla	hlIII (consensus)	hlIII (other than RF122)	hlb_probe 1	hlb_probe 2	hlb_probe 3	un-disrupted hlb	sak	chp	scn	etA	etB	etD	edinA	edinB	edinC	arcA-SCC	arcB-SCC	arcC-SCC	arcD-SCC	aur (consensus)	aur (other than MRSA252)	aur (MRSA252)	splA	splB	splE	sspA	sspB	sspP (consensus)	sspP (other than ST93)	setC	ssl01/set6_probe1_11	ssl01/set6_probe2_11	ssl01/set6_probe1_12	ssl01/set6_probe2_12	ssl01/set6_probe4_11	ssl01/set6_probeRF122
**2008**																																							
08V04188	CC5/ST228-MRSA-I, South German EMRSA	POS	POS		POS				NEG	POS			NEG	NEG	NEG	NEG	NEG	NEG								POS	POS						POS	POS	NEG	POS	NEG	POS	
08V21565	CC5/ST228-MRSA-I, South German EMRSA	POS	POS		POS				NEG	POS			NEG	NEG	NEG	NEG	NEG	NEG								POS	POS						POS	POS	NEG	POS	NEG	POS	
08V53546	CC5/ST228-MRSA-I, South German EMRSA	POS	POS	POS	POS	POS	POS	AMB	NEG	POS	NEG	POS	NEG	NEG	NEG	NEG	NEG	NEG	NEG	NEG	NEG	NEG	POS	POS	NEG	POS	POS	NEG	POS	POS	POS	POS	POS	POS	NEG	POS	NEG	POS	NEG
08B04687	CC5-MRSA-II, Rhine-Hesse EMRSA/UK-EMRSA-3	POS	POS		POS				NEG	POS			NEG	NEG	NEG	NEG	NEG	NEG								POS	POS						POS	POS	NEG	POS	NEG	POS	
08F01184	CC5-MRSA-II, Rhine-Hesse EMRSA/UK-EMRSA-3	POS	POS		POS				NEG	POS			NEG	NEG	NEG	NEG	NEG	NEG								POS	POS						POS	POS	AMB	POS	NEG	POS	
08F01352	CC5-MRSA-II, Rhine-Hesse EMRSA/UK-EMRSA-3	POS	POS		POS				NEG	POS			NEG	NEG	NEG	NEG	NEG	NEG								POS	POS						POS	POS	NEG	POS	NEG	POS	
08F01711	CC5-MRSA-II, Rhine-Hesse EMRSA/UK-EMRSA-3	POS	POS		POS				NEG	POS			NEG	NEG	NEG	NEG	NEG	NEG								POS	POS						POS	POS	NEG	POS	NEG	POS	
08F03746	CC5-MRSA-II, Rhine-Hesse EMRSA/UK-EMRSA-3	POS	POS		POS				NEG	POS			NEG	NEG	NEG	NEG	NEG	NEG								POS	POS						POS	POS	NEG	POS	NEG	POS	
08F04303	CC5-MRSA-II, Rhine-Hesse EMRSA/UK-EMRSA-3	POS	POS		POS				NEG	POS			NEG	NEG	NEG	NEG	NEG	NEG								POS	POS						POS						
08F04396	CC5-MRSA-II, Rhine-Hesse EMRSA/UK-EMRSA-3	POS	POS		POS				NEG	POS			NEG	NEG	NEG	NEG	NEG	NEG								POS	POS						POS						
08U11214	CC5-MRSA-II, Rhine-Hesse EMRSA/UK-EMRSA-3	POS	POS		POS				NEG	POS			NEG	NEG	NEG	NEG	NEG	NEG								POS	POS						POS						
08V01047	CC5-MRSA-II, Rhine-Hesse EMRSA/UK-EMRSA-3	POS	POS		POS				NEG	POS			NEG	NEG	NEG	NEG	NEG	NEG								POS	POS						POS	POS	NEG	POS	NEG	POS	
08V01222	CC5-MRSA-II, Rhine-Hesse EMRSA/UK-EMRSA-3	POS	POS		POS				NEG	POS			NEG	NEG	NEG	NEG	NEG	NEG								POS	POS						POS	POS	NEG	POS	NEG	POS	
08V01315	CC5-MRSA-II, Rhine-Hesse EMRSA/UK-EMRSA-3	POS	POS		POS				NEG	POS			NEG	NEG	NEG	NEG	NEG	NEG								POS	POS						POS	POS	NEG	POS	NEG	POS	
08V06126	CC5-MRSA-II, Rhine-Hesse EMRSA/UK-EMRSA-3	POS	POS		POS				NEG	POS			NEG	NEG	NEG	NEG	NEG	NEG								POS	POS						POS	POS	NEG	POS	NEG	POS	
08V06171	CC5-MRSA-II, Rhine-Hesse EMRSA/UK-EMRSA-3	POS	POS		POS				NEG	POS			NEG	NEG	NEG	NEG	NEG	NEG								POS	POS						POS	POS	NEG	POS	NEG	POS	
08V06623	CC5-MRSA-II, Rhine-Hesse EMRSA/UK-EMRSA-3	POS	POS		POS				NEG	POS			NEG	NEG	NEG	NEG	NEG	NEG								POS	POS						POS	POS	NEG	POS	NEG	POS	
08V10539	CC5-MRSA-II, Rhine-Hesse EMRSA/UK-EMRSA-3	POS	POS		POS				NEG	POS			NEG	NEG	NEG	NEG	NEG	NEG								POS	POS						POS						
08V11213	CC5-MRSA-II, Rhine-Hesse EMRSA/UK-EMRSA-3	POS	POS		POS				NEG	POS			NEG	NEG	NEG	NEG	NEG	NEG								POS	POS						POS	POS	NEG	POS	NEG	POS	
08V12100	CC5-MRSA-II, Rhine-Hesse EMRSA/UK-EMRSA-3	POS	POS		POS				NEG	POS			NEG	NEG	NEG	NEG	NEG	NEG								POS	POS						POS	POS	NEG	POS	NEG	POS	
08V13847	CC5-MRSA-II, Rhine-Hesse EMRSA/UK-EMRSA-3	POS	POS		POS				AMB	POS			NEG	NEG	NEG	NEG	NEG	NEG								POS	POS						POS	POS	AMB	POS	NEG	POS	
08V15905	CC5-MRSA-II, Rhine-Hesse EMRSA/UK-EMRSA-3	POS	POS	POS	POS	POS	POS	POS	NEG	POS	POS	POS	NEG	NEG	NEG	NEG	NEG	NEG	NEG	NEG	NEG	NEG	POS	POS	NEG	POS	POS	NEG	POS	POS	POS	POS	POS	POS	NEG	POS	NEG	POS	NEG
08V18191	CC5-MRSA-II, Rhine-Hesse EMRSA/UK-EMRSA-3	POS	POS	POS	POS	POS	POS	POS	NEG	POS	POS	POS	NEG	NEG	NEG	NEG	NEG	NEG	NEG	NEG	NEG	NEG	POS	POS	NEG	POS	POS	NEG	POS	POS	POS	POS	POS						NEG
08V21425	CC5-MRSA-II, Rhine-Hesse EMRSA/UK-EMRSA-3	POS	POS		POS				NEG	POS			NEG	NEG	NEG	NEG	NEG	NEG								POS	POS						POS	POS	NEG	POS	NEG	POS	
08V23868	CC5-MRSA-II, Rhine-Hesse EMRSA/UK-EMRSA-3	POS	POS		POS				NEG	POS			NEG	NEG	NEG	NEG	NEG	NEG								POS	POS						POS	POS	NEG	POS	NEG	POS	
08V27435	CC5-MRSA-II, Rhine-Hesse EMRSA/UK-EMRSA-3	POS	POS		POS				NEG	POS			NEG	NEG	NEG	NEG	NEG	NEG								POS	POS						POS	POS	NEG	POS	NEG	POS	
08V27514	CC5-MRSA-II, Rhine-Hesse EMRSA/UK-EMRSA-3	POS	POS		POS				NEG	POS			NEG	NEG	NEG	NEG	NEG	NEG								POS	POS						POS	POS	AMB	POS	NEG	POS	
08V28482	CC5-MRSA-II, Rhine-Hesse EMRSA/UK-EMRSA-3	POS	POS		POS				NEG	POS			NEG	NEG	NEG	NEG	NEG	NEG								POS	POS						POS	POS	NEG	POS	NEG	POS	
08V29312	CC5-MRSA-II, Rhine-Hesse EMRSA/UK-EMRSA-3	POS	POS		POS				NEG	POS			NEG	NEG	NEG	NEG	NEG	NEG								POS	POS						POS	POS	NEG	POS	NEG	POS	
08V29889	CC5-MRSA-II, Rhine-Hesse EMRSA/UK-EMRSA-3	POS	POS		POS				NEG	POS			NEG	NEG	NEG	NEG	NEG	NEG								POS	POS						POS	POS	NEG	POS	NEG	POS	
08V30696	CC5-MRSA-II, Rhine-Hesse EMRSA/UK-EMRSA-3	POS	POS		POS				NEG	POS			NEG	NEG	NEG	NEG	NEG	NEG								POS	POS						POS	POS	NEG	POS	NEG	POS	
08V32764	CC5-MRSA-II, Rhine-Hesse EMRSA/UK-EMRSA-3	POS	POS		POS				NEG	POS			NEG	NEG	NEG	NEG	NEG	NEG								POS	POS						POS	POS	AMB	POS	NEG	POS	
08V36052	CC5-MRSA-II, Rhine-Hesse EMRSA/UK-EMRSA-3	POS	POS		POS				NEG	POS			NEG	NEG	NEG	NEG	NEG	NEG								POS	POS						POS	POS	NEG	POS	NEG	POS	
08V36125	CC5-MRSA-II, Rhine-Hesse EMRSA/UK-EMRSA-3	POS	POS		POS				NEG	POS			NEG	NEG	NEG	NEG	NEG	NEG								POS	POS						POS						
08V39935	CC5-MRSA-II, Rhine-Hesse EMRSA/UK-EMRSA-3	POS	POS		POS				NEG	POS			NEG	NEG	NEG	NEG	NEG	NEG								POS	POS						POS	POS	NEG	POS	NEG	POS	
08V41631	CC5-MRSA-II, Rhine-Hesse EMRSA/UK-EMRSA-3	POS	POS		POS				NEG	POS			NEG	NEG	NEG	NEG	NEG	NEG								POS	POS						POS	POS	NEG	POS	NEG	POS	
08V42588	CC5-MRSA-II, Rhine-Hesse EMRSA/UK-EMRSA-3	POS	POS		POS				NEG	POS			NEG	NEG	NEG	NEG	NEG	NEG								POS	POS						POS	POS	NEG	POS	NEG	AMB	
08V42633	CC5-MRSA-II, Rhine-Hesse EMRSA/UK-EMRSA-3	POS	POS		POS				NEG	POS			NEG	NEG	NEG	NEG	NEG	NEG								POS	POS						POS	POS	NEG	POS	NEG	POS	
08V43410	CC5-MRSA-II, Rhine-Hesse EMRSA/UK-EMRSA-3	POS	POS		POS				POS	POS			NEG	NEG	NEG	NEG	NEG	NEG								POS	POS						POS	POS	NEG	POS	NEG	pos	
08V43581	CC5-MRSA-II, Rhine-Hesse EMRSA/UK-EMRSA-3	POS	POS		POS				AMB	POS			NEG	NEG	NEG	NEG	NEG	NEG								POS	POS						POS	POS	NEG	POS	NEG	NEG	
08V47242	CC5-MRSA-II, Rhine-Hesse EMRSA/UK-EMRSA-3	POS	POS	POS	POS	POS	POS	POS	NEG	POS	POS	POS	NEG	NEG	NEG	NEG	NEG	NEG	NEG	NEG	NEG	NEG	POS	POS	NEG	POS	POS	NEG	POS	POS	POS	POS	POS	POS	NEG	POS	NEG	POS	NEG
08V50740	CC5-MRSA-II, Rhine-Hesse EMRSA/UK-EMRSA-3	POS	POS	POS	POS	NEG	POS	NEG	NEG	POS	POS	POS	NEG	NEG	NEG	NEG	NEG	NEG	NEG	NEG	NEG	NEG	POS	AMB	NEG	POS	POS	NEG	POS	POS	POS	POS	POS	POS	NEG	POS	NEG	POS	NEG
08V02855	CC7-MRSA-IV	POS	POS	POS	POS	AMB	POS	AMB	NEG	POS	NEG	POS	NEG	NEG	NEG	NEG	NEG	NEG	NEG	NEG	NEG	NEG	POS	POS	NEG	POS	POS	POS	POS	POS	POS	POS	POS	POS	NEG	NEG	POS	NEG	NEG
08V35987	CC8/ST239-MRSA-III, Vienna/Hungarian/Brazilian Clone	POS	POS	POS	POS	AMB	POS	NEG	POS	NEG	NEG	NEG	NEG	NEG	NEG	NEG	NEG	NEG	NEG	NEG	NEG	NEG	AMB	NEG	POS	POS	POS	NEG	POS	POS	POS	POS	POS						NEG
08V39853	CC8/ST254-MRSA-(atypical SCCmec), Hannover EMRSA	POS	POS	POS	POS	POS	POS	POS	NEG	POS	NEG	POS	NEG	NEG	NEG	NEG	NEG	NEG	NEG	NEG	NEG	NEG	POS	POS	NEG	POS	POS	POS	POS	POS	POS	POS	POS						NEG
08F01314	CC8-MRSA-IV, UK-EMRSA-14/WA MRSA-5	POS	POS	POS	POS	AMB	POS	NEG	POS	NEG	NEG	NEG	NEG	NEG	NEG	NEG	NEG	NEG	NEG	NEG	NEG	NEG	POS	POS	NEG	POS	POS	AMB	POS	POS	POS	POS	POS						NEG
08V15773	CC8-MRSA-IV, USA300	POS	POS	POS	POS	POS	POS	POS	NEG	POS	POS	POS	NEG	NEG	NEG	NEG	NEG	NEG	POS	POS	POS	POS	POS	POS	NEG	POS	POS	POS	POS	POS	POS	POS	POS						NEG
08B02942	CC22-MRSA-IV, Barnim/UK-EMRSA-15	POS	POS		NEG				NEG	POS			NEG	NEG	NEG	NEG	NEG	NEG								NEG	NEG						POS						
08F00816	CC22-MRSA-IV, Barnim/UK-EMRSA-15	POS	NEG		NEG				NEG	POS			NEG	NEG	NEG	NEG	NEG	NEG								NEG	NEG						POS						
08F01637	CC22-MRSA-IV, Barnim/UK-EMRSA-15	POS	POS		NEG				NEG	POS			NEG	NEG	NEG	NEG	NEG	NEG								NEG	NEG						POS						
08F04948	CC22-MRSA-IV, Barnim/UK-EMRSA-15	POS	POS		NEG				NEG	POS			NEG	NEG	NEG	NEG	NEG	NEG								NEG	AMB						POS						
08U07826	CC22-MRSA-IV, Barnim/UK-EMRSA-15	POS	POS		NEG				NEG	POS			NEG	NEG	NEG	NEG	NEG	NEG								NEG	NEG						POS						
08V00371	CC22-MRSA-IV, Barnim/UK-EMRSA-15	POS	POS		NEG				NEG	POS			NEG	NEG	NEG	NEG	NEG	NEG								NEG	NEG						POS						
08V02049	CC22-MRSA-IV, Barnim/UK-EMRSA-15	POS	POS		NEG				NEG	POS			NEG	NEG	NEG	NEG	NEG	NEG								NEG	NEG						POS						
08V02349	CC22-MRSA-IV, Barnim/UK-EMRSA-15	POS	POS		NEG				NEG	POS			NEG	NEG	NEG	NEG	NEG	NEG								NEG	NEG						POS						
08V03329	CC22-MRSA-IV, Barnim/UK-EMRSA-15	POS	POS		NEG				NEG	POS			NEG	NEG	NEG	NEG	NEG	NEG								NEG	NEG						POS						
08V04516	CC22-MRSA-IV, Barnim/UK-EMRSA-15	POS	POS		NEG				NEG	POS			NEG	NEG	NEG	NEG	NEG	NEG								NEG	NEG						POS						
08V04681	CC22-MRSA-IV, Barnim/UK-EMRSA-15	POS	POS		NEG				NEG	POS			NEG	NEG	NEG	NEG	NEG	NEG								NEG	NEG						POS						
08V04740	CC22-MRSA-IV, Barnim/UK-EMRSA-15	POS	POS		NEG				NEG	POS			NEG	NEG	NEG	NEG	NEG	NEG								NEG	NEG						POS						
08V04977	CC22-MRSA-IV, Barnim/UK-EMRSA-15	POS	POS		NEG				NEG	POS			NEG	NEG	NEG	NEG	NEG	NEG								NEG	NEG						POS						
08V06376	CC22-MRSA-IV, Barnim/UK-EMRSA-15	POS	POS		NEG				NEG	POS			NEG	NEG	NEG	NEG	NEG	NEG								NEG	NEG						POS						
08V09540	CC22-MRSA-IV, Barnim/UK-EMRSA-15	POS	POS		NEG				NEG	POS			NEG	NEG	NEG	NEG	NEG	NEG								NEG	NEG						POS						
08V10287	CC22-MRSA-IV, Barnim/UK-EMRSA-15	POS	POS		NEG				NEG	POS			NEG	NEG	NEG	NEG	NEG	NEG								NEG	NEG						POS						
08V10376	CC22-MRSA-IV, Barnim/UK-EMRSA-15	POS	POS		NEG				NEG	POS			NEG	NEG	NEG	NEG	NEG	NEG								NEG	NEG						POS						
08V10714	CC22-MRSA-IV, Barnim/UK-EMRSA-15	POS	POS		NEG				NEG	POS			NEG	NEG	NEG	NEG	NEG	NEG								NEG	NEG						POS						
08V10836	CC22-MRSA-IV, Barnim/UK-EMRSA-15	POS	POS		NEG				NEG	POS			NEG	NEG	NEG	NEG	NEG	NEG								NEG	NEG						POS						
08V11971	CC22-MRSA-IV, Barnim/UK-EMRSA-15	POS	POS		NEG				NEG	POS			NEG	NEG	NEG	NEG	NEG	NEG								NEG	NEG						POS						
08V14130	CC22-MRSA-IV, Barnim/UK-EMRSA-15	POS	POS		NEG				NEG	POS			NEG	NEG	NEG	NEG	NEG	NEG								NEG	NEG						POS						
08V14723	CC22-MRSA-IV, Barnim/UK-EMRSA-15	POS	POS		NEG				NEG	POS			NEG	NEG	NEG	NEG	NEG	NEG								NEG	NEG						POS						
08V16292	CC22-MRSA-IV, Barnim/UK-EMRSA-15	POS	POS		NEG				NEG	POS			NEG	NEG	NEG	NEG	NEG	NEG								NEG	NEG						POS						
08V16554	CC22-MRSA-IV, Barnim/UK-EMRSA-15	POS	POS		NEG				NEG	POS			NEG	NEG	NEG	NEG	NEG	NEG								NEG	NEG						POS						
08V18482	CC22-MRSA-IV, Barnim/UK-EMRSA-15	POS	POS		NEG				NEG	POS			NEG	NEG	NEG	NEG	NEG	NEG								NEG	NEG						POS						
08V19658	CC22-MRSA-IV, Barnim/UK-EMRSA-15	POS	POS		NEG				NEG	POS			NEG	NEG	NEG	NEG	NEG	NEG								NEG	NEG						POS						
08V19663	CC22-MRSA-IV, Barnim/UK-EMRSA-15	POS	POS		NEG				POS	NEG			NEG	NEG	NEG	NEG	NEG	NEG								NEG	NEG						POS						
08V20266	CC22-MRSA-IV, Barnim/UK-EMRSA-15	POS	POS		NEG				NEG	POS			NEG	NEG	NEG	NEG	NEG	NEG								NEG	NEG						POS						
08V20337	CC22-MRSA-IV, Barnim/UK-EMRSA-15	POS	POS		NEG				NEG	POS			NEG	NEG	NEG	NEG	NEG	NEG								NEG	NEG						POS						
08V21055	CC22-MRSA-IV, Barnim/UK-EMRSA-15	POS	POS		NEG				NEG	POS			NEG	NEG	NEG	NEG	NEG	NEG								NEG	NEG						POS						
08V23747	CC22-MRSA-IV, Barnim/UK-EMRSA-15	POS	POS		NEG				NEG	POS			NEG	NEG	NEG	NEG	NEG	NEG								NEG	NEG						POS						
08V23813	CC22-MRSA-IV, Barnim/UK-EMRSA-15	POS	POS		NEG				NEG	POS			NEG	NEG	NEG	NEG	NEG	NEG								NEG	NEG						POS						
08V25135	CC22-MRSA-IV, Barnim/UK-EMRSA-15	POS	POS		NEG				NEG	POS			NEG	NEG	NEG	NEG	NEG	NEG								NEG	NEG						POS						
08V27329	CC22-MRSA-IV, Barnim/UK-EMRSA-15	POS	POS		NEG				NEG	POS			NEG	NEG	NEG	NEG	NEG	NEG								NEG	NEG						POS						
08V27434	CC22-MRSA-IV, Barnim/UK-EMRSA-15	POS	POS		NEG				NEG	POS			NEG	NEG	NEG	NEG	NEG	NEG								NEG	NEG						POS						
08V27729	CC22-MRSA-IV, Barnim/UK-EMRSA-15	POS	POS		NEG				NEG	POS			NEG	NEG	NEG	NEG	NEG	NEG								NEG	NEG						POS						
08V31445	CC22-MRSA-IV, Barnim/UK-EMRSA-15	POS	POS		NEG				NEG	POS			NEG	NEG	NEG	NEG	NEG	NEG								NEG	NEG						POS						
08V34812	CC22-MRSA-IV, Barnim/UK-EMRSA-15	POS	POS		NEG				NEG	POS			NEG	NEG	NEG	NEG	NEG	NEG								NEG	NEG						POS						
08V34813	CC22-MRSA-IV, Barnim/UK-EMRSA-15	POS	POS		NEG				NEG	POS			NEG	NEG	NEG	NEG	NEG	NEG								NEG	NEG						POS						
08V35361	CC22-MRSA-IV, Barnim/UK-EMRSA-15	POS	POS		NEG				NEG	POS			NEG	NEG	NEG	NEG	NEG	NEG								NEG	NEG						POS						
08V37931	CC22-MRSA-IV, Barnim/UK-EMRSA-15	POS	POS		NEG				NEG	POS			NEG	NEG	NEG	NEG	NEG	NEG								NEG	NEG						POS						
08V45578	CC22-MRSA-IV, Barnim/UK-EMRSA-15	POS	POS		NEG				POS	NEG			NEG	NEG	NEG	NEG	NEG	NEG								NEG	NEG						POS	NEG	POS	POS	NEG	AMB	
08V47385	CC22-MRSA-IV, Barnim/UK-EMRSA-15	POS	POS	NEG	NEG	POS	POS	POS	NEG	POS	POS	POS	NEG	NEG	NEG	NEG	NEG	NEG	NEG	NEG	NEG	NEG	POS	NEG	POS	NEG	NEG	NEG	POS	POS	POS	POS	POS	NEG	POS	POS	NEG	NEG	NEG
08V53678	CC22-MRSA-IV, Barnim/UK-EMRSA-15	POS	POS	NEG	NEG	POS	POS	POS	NEG	POS	POS	POS	NEG	NEG	NEG	NEG	NEG	NEG	NEG	NEG	NEG	NEG	POS	NEG	POS	NEG	NEG	NEG	POS	POS	POS	POS	POS	NEG	POS	POS	NEG	NEG	NEG
08B09737	CC45-MRSA-IV, Berlin EMRSA	NEG	NEG		NEG				NEG	POS			NEG	NEG	NEG	NEG	NEG	NEG								NEG	NEG						NEG						
08B14321	CC45-MRSA-IV, Berlin EMRSA	POS	POS	POS	NEG	POS	POS	NEG	NEG	POS	POS	POS	NEG	NEG	NEG	NEG	NEG	NEG	NEG	NEG	NEG	NEG	POS	NEG	POS	NEG	NEG	NEG	POS	POS	POS	POS	POS	NEG	POS	NEG	NEG	POS	NEG
08F01439	CC45-MRSA-IV, Berlin EMRSA	POS	POS		NEG				NEG	POS			NEG	NEG	NEG	NEG	NEG	NEG								NEG	NEG						POS						
08U12518	CC45-MRSA-IV, Berlin EMRSA	POS	POS		NEG				NEG	POS			NEG	NEG	NEG	NEG	NEG	NEG								NEG	NEG						POS						
08U13653	CC45-MRSA-IV, Berlin EMRSA	POS	POS	POS	NEG	POS	POS	NEG	NEG	POS	POS	POS	NEG	NEG	NEG	NEG	NEG	NEG	NEG	NEG	NEG	NEG	POS	NEG	POS	NEG	NEG	NEG	POS	POS	POS	POS	POS	NEG	POS	AMB	NEG	POS	NEG
08V00675	CC45-MRSA-IV, Berlin EMRSA	POS	POS		NEG				NEG	POS			NEG	NEG	NEG	NEG	NEG	NEG								NEG	NEG						POS						
08V02459	CC45-MRSA-IV, Berlin EMRSA	POS	POS		NEG				NEG	POS			NEG	NEG	NEG	NEG	NEG	NEG								NEG	NEG						POS						
08V02654	CC45-MRSA-IV, Berlin EMRSA	POS	POS		NEG				NEG	POS			NEG	NEG	NEG	NEG	NEG	NEG								NEG	NEG						POS						
08V05677	CC45-MRSA-IV, Berlin EMRSA	POS	POS	POS	NEG	NEG	NEG	NEG	NEG	POS	POS	POS	NEG	NEG	NEG	NEG	NEG	NEG	NEG	NEG	NEG	NEG	NEG	NEG	POS	NEG	NEG	NEG	POS	POS	POS	POS	POS						NEG
08V20930	CC45-MRSA-IV, Berlin EMRSA	POS	POS		NEG				NEG	POS			NEG	NEG	NEG	NEG	NEG	NEG								AMB	NEG						POS						
08V23911	CC45-MRSA-IV, Berlin EMRSA	POS	POS		NEG				NEG	POS			NEG	NEG	NEG	NEG	NEG	NEG								NEG	NEG						POS						
08V39175	CC45-MRSA-IV, Berlin EMRSA	POS	POS		NEG				NEG	POS			NEG	NEG	NEG	NEG	NEG	NEG								NEG	NEG						POS						
08V39949	CC45-MRSA-IV, Berlin EMRSA	POS	NEG		NEG				NEG	POS			NEG	NEG	NEG	NEG	NEG	NEG								NEG	NEG						POS						
08V53670	CC88-MRSA-IV, WA MRSA-2	POS	POS	POS	POS	POS	POS	POS	NEG	POS	AMB	POS	NEG	NEG	NEG	NEG	NEG	NEG	NEG	NEG	NEG	NEG	POS	POS	NEG	POS	POS	NEG	POS	POS	POS	POS	POS	NEG	POS	NEG	NEG	POS	NEG
08V06946	CC398-MRSA-V	POS	POS	POS	POS	NEG	NEG	NEG	NEG	NEG	NEG	NEG	NEG	NEG	NEG	NEG	NEG	NEG	NEG	NEG	NEG	NEG	POS	NEG	POS	NEG	NEG	NEG	POS	POS	POS	POS	POS						NEG

ISOLATE:	STRAIN	staphylococcal superantigen-like protein 2		staphylococcal superantigen-like protein 3			staphylococcal superantigen-like protein 4		staphylococcal superantigen-like protein 5				staphylococcal superantigen-like protein 6		staphylococcal superantigen-like protein 7			staphylococcal superantigen-like protein 8
		ssl02/set7	ssl02/set7 (MRSA252)	ssl03/set8_probe 1	ssl03/set8_probe 2	ssl03/set8 (MRSA252, SAR0424)	ssl04/set9	ssl04/set9 (MRSA252, SAR0425)	ssl05/set3_probe 1	ssl05/set3 (RF122, probe-611)	ssl05/set3_probe 2 (612)	ssl05/set3 (MRSA252)	ssl06/set21	ssl06 (NCTC8325+MW2)	ssl07/set1	ssl07/set1 (MRSA252)	ssl07/set1 (AF188836)	ssl08/set12_probe 1
**2008**																		
08V04188	CC5/ST228-MRSA-I, South German EMRSA	POS	NEG	POS		NEG	POS	NEG	POS			NEG	NEG		POS	AMB	NEG	POS
08V21565	CC5/ST228-MRSA-I, South German EMRSA	POS	NEG	POS		NEG	POS	NEG	POS			NEG	NEG		POS	AMB	NEG	POS
08V53546	CC5/ST228-MRSA-I, South German EMRSA	POS	AMB	POS	POS	NEG	POS	NEG	NEG	AMB	POS	NEG	NEG	NEG	POS	AMB	NEG	POS
08B04687	CC5-MRSA-II, Rhine-Hesse EMRSA/UK-EMRSA-3	POS	AMB	POS		NEG	POS	NEG	POS			NEG	NEG		POS	AMB	AMB	POS
08F01184	CC5-MRSA-II, Rhine-Hesse EMRSA/UK-EMRSA-3	POS	AMB	POS		NEG	POS	NEG	POS			NEG	NEG		AMB	POS	AMB	POS
08F01352	CC5-MRSA-II, Rhine-Hesse EMRSA/UK-EMRSA-3	POS	NEG	POS		NEG	POS	NEG	POS			NEG	NEG		POS	AMB	NEG	POS
08F01711	CC5-MRSA-II, Rhine-Hesse EMRSA/UK-EMRSA-3	POS	AMB	POS		NEG	POS	NEG	POS			NEG	NEG		POS	AMB	AMB	POS
08F03746	CC5-MRSA-II, Rhine-Hesse EMRSA/UK-EMRSA-3	POS	AMB	POS		NEG	POS	NEG	POS			NEG	NEG		POS	AMB	AMB	POS
08F04303	CC5-MRSA-II, Rhine-Hesse EMRSA/UK-EMRSA-3	POS	AMB	POS		NEG	POS	NEG	POS			NEG	NEG		POS	AMB	AMB	POS
08F04396	CC5-MRSA-II, Rhine-Hesse EMRSA/UK-EMRSA-3	POS	AMB	POS		NEG	POS	NEG	POS			NEG	NEG		POS	AMB	AMB	POS
08U11214	CC5-MRSA-II, Rhine-Hesse EMRSA/UK-EMRSA-3	POS	AMB	POS		NEG	POS	NEG	POS			NEG	NEG		POS	AMB	NEG	POS
08V01047	CC5-MRSA-II, Rhine-Hesse EMRSA/UK-EMRSA-3	POS	POS	POS		NEG	POS	NEG	POS			NEG	NEG		POS	AMB	AMB	POS
08V01222	CC5-MRSA-II, Rhine-Hesse EMRSA/UK-EMRSA-3	POS	AMB	POS		NEG	POS	NEG	POS			NEG	NEG		POS	AMB	AMB	POS
08V01315	CC5-MRSA-II, Rhine-Hesse EMRSA/UK-EMRSA-3	POS	POS	POS		NEG	POS	NEG	POS			NEG	NEG		POS	AMB	AMB	POS
08V06126	CC5-MRSA-II, Rhine-Hesse EMRSA/UK-EMRSA-3	POS	AMB	POS		NEG	POS	NEG	POS			NEG	NEG		AMB	POS	AMB	POS
08V06171	CC5-MRSA-II, Rhine-Hesse EMRSA/UK-EMRSA-3	POS	AMB	POS		NEG	POS	NEG	POS			NEG	NEG		AMB	POS	AMB	POS
08V06623	CC5-MRSA-II, Rhine-Hesse EMRSA/UK-EMRSA-3	POS	AMB	POS		NEG	POS	NEG	POS			NEG	NEG		POS	AMB	AMB	POS
08V10539	CC5-MRSA-II, Rhine-Hesse EMRSA/UK-EMRSA-3	POS	AMB	POS		NEG	POS	NEG	POS			NEG	NEG		POS	AMB	AMB	POS
08V11213	CC5-MRSA-II, Rhine-Hesse EMRSA/UK-EMRSA-3	POS	AMB	POS		NEG	POS	NEG	POS			NEG	NEG		POS	AMB	AMB	POS
08V12100	CC5-MRSA-II, Rhine-Hesse EMRSA/UK-EMRSA-3	POS	AMB	POS		NEG	POS	NEG	POS			NEG	NEG		AMB	POS	AMB	POS
08V13847	CC5-MRSA-II, Rhine-Hesse EMRSA/UK-EMRSA-3	POS	AMB	POS		NEG	POS	NEG	POS			NEG	NEG		AMB	POS	AMB	POS
08V15905	CC5-MRSA-II, Rhine-Hesse EMRSA/UK-EMRSA-3	POS	AMB	POS	POS	NEG	POS	NEG	POS	AMB	POS	NEG	NEG	NEG	POS	AMB	AMB	POS
08V18191	CC5-MRSA-II, Rhine-Hesse EMRSA/UK-EMRSA-3	POS	AMB	POS	POS	NEG	POS	NEG	POS	AMB	POS	NEG	NEG	NEG	POS	AMB	NEG	POS
08V21425	CC5-MRSA-II, Rhine-Hesse EMRSA/UK-EMRSA-3	POS	NEG	POS		NEG	POS	NEG	POS			NEG	NEG		POS	AMB	NEG	POS
08V23868	CC5-MRSA-II, Rhine-Hesse EMRSA/UK-EMRSA-3	POS	AMB	POS		NEG	POS	NEG	POS			NEG	NEG		POS	AMB	AMB	POS
08V27435	CC5-MRSA-II, Rhine-Hesse EMRSA/UK-EMRSA-3	POS	NEG	POS		NEG	POS	NEG	POS			NEG	NEG		POS	AMB	NEG	POS
08V27514	CC5-MRSA-II, Rhine-Hesse EMRSA/UK-EMRSA-3	POS	AMB	POS		NEG	POS	NEG	POS			NEG	NEG		AMB	POS	AMB	POS
08V28482	CC5-MRSA-II, Rhine-Hesse EMRSA/UK-EMRSA-3	POS	AMB	POS		NEG	POS	NEG	POS			NEG	NEG		POS	AMB	AMB	POS
08V29312	CC5-MRSA-II, Rhine-Hesse EMRSA/UK-EMRSA-3	POS	AMB	POS		NEG	POS	NEG	POS			NEG	NEG		POS	AMB	AMB	POS
08V29889	CC5-MRSA-II, Rhine-Hesse EMRSA/UK-EMRSA-3	POS	AMB	POS		NEG	POS	NEG	POS			NEG	NEG		AMB	POS	AMB	POS
08V30696	CC5-MRSA-II, Rhine-Hesse EMRSA/UK-EMRSA-3	POS	AMB	POS		NEG	POS	NEG	POS			NEG	NEG		POS	AMB	AMB	POS
08V32764	CC5-MRSA-II, Rhine-Hesse EMRSA/UK-EMRSA-3	POS	AMB	POS		NEG	POS	NEG	POS			NEG	NEG		AMB	POS	AMB	POS
08V36052	CC5-MRSA-II, Rhine-Hesse EMRSA/UK-EMRSA-3	POS	AMB	POS		NEG	POS	NEG	POS			NEG	NEG		POS	AMB	AMB	POS
08V36125	CC5-MRSA-II, Rhine-Hesse EMRSA/UK-EMRSA-3	POS	AMB	POS		NEG	POS	NEG	POS			NEG	NEG		POS	AMB	AMB	POS
08V39935	CC5-MRSA-II, Rhine-Hesse EMRSA/UK-EMRSA-3	POS	AMB	POS		NEG	POS	NEG	POS			NEG	NEG		POS	AMB	AMB	POS
08V41631	CC5-MRSA-II, Rhine-Hesse EMRSA/UK-EMRSA-3	POS	AMB	POS		NEG	POS	NEG	POS			NEG	NEG		POS	AMB	AMB	POS
08V42588	CC5-MRSA-II, Rhine-Hesse EMRSA/UK-EMRSA-3	POS	AMB	POS		NEG	POS	NEG	POS			NEG	NEG		POS	AMB	NEG	POS
08V42633	CC5-MRSA-II, Rhine-Hesse EMRSA/UK-EMRSA-3	POS	AMB	POS		NEG	POS	NEG	POS			NEG	NEG		POS	AMB	NEG	POS
08V43410	CC5-MRSA-II, Rhine-Hesse EMRSA/UK-EMRSA-3	POS	AMB	POS		NEG	POS	NEG	POS			NEG	NEG		POS	AMB	NEG	POS
08V43581	CC5-MRSA-II, Rhine-Hesse EMRSA/UK-EMRSA-3	POS	AMB	POS		NEG	POS	NEG	POS			NEG	NEG		POS	AMB	NEG	POS
08V47242	CC5-MRSA-II, Rhine-Hesse EMRSA/UK-EMRSA-3	POS	AMB	POS	POS	NEG	POS	NEG	POS	AMB	POS	NEG	NEG	NEG	POS	AMB	NEG	POS
08V50740	CC5-MRSA-II, Rhine-Hesse EMRSA/UK-EMRSA-3	POS	NEG	POS	POS	NEG	POS	NEG	POS	NEG	POS	NEG	NEG	NEG	POS	AMB	NEG	POS
08V02855	CC7-MRSA-IV	POS	NEG	AMB	AMB	NEG	POS	NEG	POS	NEG	POS	NEG	POS	POS	POS	AMB	NEG	POS
08V35987	CC8/ST239-MRSA-III, Vienna/Hungarian/Brazilian Clone	POS	NEG	POS	POS	NEG	POS	NEG	POS	AMB	POS	NEG	NEG	POS	POS	AMB	AMB	POS
08V39853	CC8/ST254-MRSA-(atypical SCCmec), Hannover EMRSA	POS	NEG	POS	POS	NEG	POS	NEG	POS	AMB	POS	NEG	POS	POS	POS	AMB	AMB	POS
08F01314	CC8-MRSA-IV, UK-EMRSA-14/WA MRSA-5	POS	NEG	POS	POS	NEG	POS	NEG	POS	NEG	POS	NEG	AMB	POS	POS	AMB	NEG	POS
08V15773	CC8-MRSA-IV, USA300	POS	AMB	POS	POS	NEG	POS	NEG	POS	NEG	POS	NEG	POS	POS	POS	AMB	NEG	POS
08B02942	CC22-MRSA-IV, Barnim/UK-EMRSA-15	POS	AMB	NEG		AMB	NEG	POS	POS			POS	NEG		AMB	POS	POS	NEG
08F00816	CC22-MRSA-IV, Barnim/UK-EMRSA-15	NEG	NEG	NEG		NEG	NEG	NEG	POS			NEG	NEG		NEG	AMB	POS	NEG
08F01637	CC22-MRSA-IV, Barnim/UK-EMRSA-15	AMB	POS	NEG		POS	NEG	POS	POS			POS	NEG		AMB	POS	AMB	NEG
08F04948	CC22-MRSA-IV, Barnim/UK-EMRSA-15	POS	AMB	NEG		POS	NEG	POS	POS			POS	NEG		AMB	AMB	POS	NEG
08U07826	CC22-MRSA-IV, Barnim/UK-EMRSA-15	POS	AMB	NEG		NEG	NEG	NEG	POS			POS	NEG		AMB	AMB	POS	NEG
08V00371	CC22-MRSA-IV, Barnim/UK-EMRSA-15	POS	POS	NEG		POS	NEG	POS	POS			POS	NEG		AMB	AMB	POS	NEG
08V02049	CC22-MRSA-IV, Barnim/UK-EMRSA-15	POS	POS	NEG		POS	NEG	POS	POS			POS	NEG		AMB	AMB	POS	NEG
08V02349	CC22-MRSA-IV, Barnim/UK-EMRSA-15	POS	POS	NEG		POS	NEG	POS	POS			POS	NEG		AMB	AMB	POS	NEG
08V03329	CC22-MRSA-IV, Barnim/UK-EMRSA-15	POS	POS	NEG		AMB	NEG	AMB	POS			POS	NEG		NEG	AMB	POS	NEG
08V04516	CC22-MRSA-IV, Barnim/UK-EMRSA-15	AMB	POS	NEG		POS	NEG	POS	POS			POS	NEG		AMB	AMB	POS	NEG
08V04681	CC22-MRSA-IV, Barnim/UK-EMRSA-15	POS	POS	NEG		POS	NEG	POS	POS			POS	NEG		AMB	POS	AMB	NEG
08V04740	CC22-MRSA-IV, Barnim/UK-EMRSA-15	POS	POS	NEG		POS	NEG	POS	POS			POS	NEG		AMB	POS	AMB	NEG
08V04977	CC22-MRSA-IV, Barnim/UK-EMRSA-15	POS	POS	NEG		POS	NEG	POS	POS			POS	NEG		AMB	POS	AMB	NEG
08V06376	CC22-MRSA-IV, Barnim/UK-EMRSA-15	POS	AMB	NEG		POS	NEG	POS	POS			POS	NEG		AMB	POS	AMB	NEG
08V09540	CC22-MRSA-IV, Barnim/UK-EMRSA-15	POS	AMB	NEG		NEG	NEG	NEG	POS			AMB	NEG		NEG	AMB	POS	NEG
08V10287	CC22-MRSA-IV, Barnim/UK-EMRSA-15	POS	NEG	NEG		NEG	NEG	NEG	POS			AMB	NEG		NEG	AMB	POS	NEG
08V10376	CC22-MRSA-IV, Barnim/UK-EMRSA-15	AMB	POS	NEG		NEG	NEG	NEG	POS			NEG	NEG		AMB	AMB	POS	NEG
08V10714	CC22-MRSA-IV, Barnim/UK-EMRSA-15	AMB	POS	NEG		NEG	NEG	NEG	POS			AMB	NEG		NEG	AMB	POS	NEG
08V10836	CC22-MRSA-IV, Barnim/UK-EMRSA-15	POS	AMB	NEG		POS	NEG	POS	POS			POS	NEG		AMB	POS	AMB	NEG
08V11971	CC22-MRSA-IV, Barnim/UK-EMRSA-15	POS	AMB	NEG		POS	NEG	POS	POS			POS	NEG		AMB	AMB	POS	NEG
08V14130	CC22-MRSA-IV, Barnim/UK-EMRSA-15	POS	AMB	NEG		AMB	NEG	POS	POS			POS	NEG		AMB	POS	AMB	NEG
08V14723	CC22-MRSA-IV, Barnim/UK-EMRSA-15	POS	AMB	NEG		NEG	NEG	NEG	POS			POS	NEG		AMB	POS	AMB	NEG
08V16292	CC22-MRSA-IV, Barnim/UK-EMRSA-15	POS	AMB	NEG		POS	NEG	POS	POS			POS	NEG		AMB	POS	AMB	NEG
08V16554	CC22-MRSA-IV, Barnim/UK-EMRSA-15	POS	AMB	NEG		POS	NEG	POS	POS			POS	NEG		AMB	POS	AMB	NEG
08V18482	CC22-MRSA-IV, Barnim/UK-EMRSA-15	POS	AMB	NEG		POS	NEG	POS	POS			POS	NEG		AMB	AMB	POS	NEG
08V19658	CC22-MRSA-IV, Barnim/UK-EMRSA-15	POS	AMB	NEG		NEG	NEG	NEG	POS			POS	NEG		AMB	AMB	POS	NEG
08V19663	CC22-MRSA-IV, Barnim/UK-EMRSA-15	POS	AMB	NEG		NEG	NEG	NEG	POS			POS	NEG		AMB	POS	AMB	NEG
08V20266	CC22-MRSA-IV, Barnim/UK-EMRSA-15	POS	AMB	NEG		NEG	NEG	POS	POS			POS	NEG		AMB	POS	AMB	NEG
08V20337	CC22-MRSA-IV, Barnim/UK-EMRSA-15	POS	AMB	NEG		POS	NEG	POS	POS			POS	NEG		AMB	AMB	POS	NEG
08V21055	CC22-MRSA-IV, Barnim/UK-EMRSA-15	POS	AMB	NEG		AMB	NEG	POS	POS			POS	NEG		AMB	POS	AMB	NEG
08V23747	CC22-MRSA-IV, Barnim/UK-EMRSA-15	POS	AMB	NEG		NEG	NEG	NEG	POS			NEG	NEG		AMB	AMB	POS	NEG
08V23813	CC22-MRSA-IV, Barnim/UK-EMRSA-15	POS	AMB	NEG		NEG	NEG	NEG	POS			AMB	NEG		AMB	POS	AMB	NEG
08V25135	CC22-MRSA-IV, Barnim/UK-EMRSA-15	POS	AMB	NEG		AMB	NEG	POS	POS			POS	NEG		AMB	AMB	POS	NEG
08V27329	CC22-MRSA-IV, Barnim/UK-EMRSA-15	POS	AMB	NEG		POS	NEG	POS	POS			POS	NEG		AMB	POS	AMB	NEG
08V27434	CC22-MRSA-IV, Barnim/UK-EMRSA-15	POS	AMB	NEG		POS	NEG	POS	POS			POS	NEG		AMB	POS	AMB	NEG
08V27729	CC22-MRSA-IV, Barnim/UK-EMRSA-15	POS	AMB	NEG		POS	NEG	POS	POS			POS	NEG		AMB	POS	AMB	NEG
08V31445	CC22-MRSA-IV, Barnim/UK-EMRSA-15	POS	AMB	NEG		POS	NEG	POS	POS			POS	NEG		AMB	POS	AMB	NEG
08V34812	CC22-MRSA-IV, Barnim/UK-EMRSA-15	POS	AMB	NEG		POS	NEG	POS	POS			POS	NEG		AMB	AMB	POS	NEG
08V34813	CC22-MRSA-IV, Barnim/UK-EMRSA-15	AMB	POS	NEG		POS	NEG	POS	POS			POS	NEG		AMB	POS	AMB	NEG
08V35361	CC22-MRSA-IV, Barnim/UK-EMRSA-15	POS	AMB	NEG		NEG	NEG	POS	POS			POS	NEG		AMB	AMB	POS	NEG
08V37931	CC22-MRSA-IV, Barnim/UK-EMRSA-15	POS	AMB	NEG		AMB	NEG	POS	POS			POS	NEG		AMB	AMB	POS	NEG
08V45578	CC22-MRSA-IV, Barnim/UK-EMRSA-15	POS	AMB	NEG		POS	NEG	POS	POS			POS	NEG		AMB	AMB	POS	NEG
08V47385	CC22-MRSA-IV, Barnim/UK-EMRSA-15	AMB	POS	NEG	NEG	POS	NEG	POS	POS	NEG	NEG	POS	NEG	AMB	AMB	AMB	POS	NEG
08V53678	CC22-MRSA-IV, Barnim/UK-EMRSA-15	POS	AMB	NEG	NEG	NEG	NEG	POS	AMB	NEG	NEG	POS	NEG	NEG	AMB	AMB	POS	NEG
08B09737	CC45-MRSA-IV, Berlin EMRSA	NEG	POS	NEG		NEG	NEG	NEG	NEG			NEG	NEG		NEG	NEG	NEG	NEG
08B14321	CC45-MRSA-IV, Berlin EMRSA	AMB	POS	NEG	NEG	NEG	NEG	POS	NEG	NEG	NEG	POS	NEG	NEG	NEG	NEG	NEG	NEG
08F01439	CC45-MRSA-IV, Berlin EMRSA	AMB	POS	NEG		NEG	NEG	POS	NEG			POS	NEG		NEG	NEG	NEG	NEG
08U12518	CC45-MRSA-IV, Berlin EMRSA	AMB	POS	NEG		NEG	NEG	POS	NEG			POS	NEG		NEG	NEG	NEG	NEG
08U13653	CC45-MRSA-IV, Berlin EMRSA	AMB	POS	NEG	NEG	NEG	NEG	AMB	NEG	NEG	NEG	POS	NEG	AMB	NEG	NEG	NEG	NEG
08V00675	CC45-MRSA-IV, Berlin EMRSA	POS	POS	NEG		NEG	NEG	AMB	NEG			POS	NEG		NEG	NEG	NEG	NEG
08V02459	CC45-MRSA-IV, Berlin EMRSA	POS	POS	NEG		NEG	NEG	POS	NEG			POS	NEG		NEG	NEG	NEG	NEG
08V02654	CC45-MRSA-IV, Berlin EMRSA	POS	POS	NEG		NEG	NEG	POS	NEG			POS	NEG		NEG	NEG	NEG	NEG
08V05677	CC45-MRSA-IV, Berlin EMRSA	NEG	POS	NEG	NEG	NEG	NEG	POS	NEG	NEG	NEG	POS	NEG	NEG	NEG	NEG	NEG	NEG
08V20930	CC45-MRSA-IV, Berlin EMRSA	AMB	POS	NEG		NEG	NEG	NEG	AMB			POS	NEG		NEG	NEG	NEG	NEG
08V23911	CC45-MRSA-IV, Berlin EMRSA	AMB	POS	NEG		NEG	NEG	NEG	NEG			POS	NEG		NEG	NEG	NEG	NEG
08V39175	CC45-MRSA-IV, Berlin EMRSA	AMB	POS	NEG		NEG	NEG	NEG	NEG			AMB	NEG		NEG	NEG	NEG	NEG
08V39949	CC45-MRSA-IV, Berlin EMRSA	NEG	POS	NEG		NEG	NEG	NEG	NEG			POS	NEG		NEG	NEG	NEG	NEG
08V53670	CC88-MRSA-IV, WA MRSA-2	POS	NEG	POS	POS	NEG	POS	NEG	NEG	AMB	POS	NEG	AMB	POS	POS	AMB	AMB	POS
08V06946	CC398-MRSA-V	NEG	POS	NEG	NEG	NEG	NEG	POS	NEG	NEG	NEG	POS	NEG	NEG	AMB	AMB	POS	NEG

VIRULENCE : STAPHYLOCOCCAL SUPERANTIGEN/ENTEROTOXIN-LIKE GENES (SET/SSL) (continued)

ISOLATE:	staphylococcal superantigen-like protein 8	staphylococcal superantigen-like protein 9			staphylococcal superantigen-like protein 10			staphylococcal superantigene-like protein 11				staphylococcal exotoxin-like protein, second locus				
	ssl08/set12_probe 2	ssl09/set5_probe 1	ssl09/set5_probe 2	ssl09/set5 (MRSA252)	ssl10/set4	ssl10 (RF122)	ssl10/set4 (MRSA252)	ssl11/set2 (COL)	ssl11+set2(Mu50+N315)	ssl11+set2(MW2+MSSA 476)	ssl11/set2 (MRSA252)	setB3	setB3 (MRSA252)	setB2	setB2 (MRSA252)	setB1
08V04188		AMB		NEG	POS		NEG	NEG	POS	NEG	NEG	POS	NEG	POS	NEG	POS
08V21565		POS		NEG	POS		NEG	NEG	POS	NEG	NEG	POS	NEG	POS	NEG	POS
08V53546	POS	POS	POS	NEG	POS	NEG	NEG	NEG	POS	NEG	NEG	POS	NEG	POS	NEG	POS
08B04687		POS		NEG	POS		NEG	NEG	POS	NEG	NEG	POS	NEG	POS	NEG	POS
08F01184		POS		NEG	POS		AMB	NEG	POS	NEG	NEG	POS	NEG	POS	NEG	POS
08F01352		POS		NEG	POS		NEG	NEG	POS	NEG	NEG	POS	NEG	POS	NEG	POS
08F01711		POS		NEG	POS		AMB	NEG	POS	NEG	NEG	POS	NEG	POS	NEG	POS
08F03746		POS		NEG	POS		AMB	NEG	POS	NEG	NEG	POS	NEG	POS	NEG	POS
08F04303		POS		NEG	POS		NEG	NEG	POS	NEG	NEG	POS	NEG	POS	NEG	POS
08F04396		POS		NEG	POS		AMB	NEG	POS	NEG	NEG	POS	NEG	POS	NEG	POS
08U11214		POS		NEG	POS		NEG	NEG	POS	NEG	NEG	POS	NEG	POS	NEG	POS
08V01047		POS		NEG	POS		NEG	NEG	POS	NEG	NEG	POS	NEG	POS	NEG	POS
08V01222		POS		NEG	POS		NEG	NEG	POS	NEG	NEG	POS	NEG	POS	NEG	POS
08V01315		POS		NEG	POS		NEG	NEG	POS	NEG	NEG	POS	NEG	POS	NEG	POS
08V06126		POS		NEG	POS		AMB	NEG	POS	NEG	NEG	POS	NEG	POS	NEG	POS
08V06171		POS		NEG	POS		AMB	NEG	POS	NEG	NEG	POS	NEG	POS	NEG	AMB
08V06623		POS		NEG	POS		AMB	NEG	POS	NEG	NEG	POS	NEG	POS	NEG	POS
08V10539		POS		NEG	POS		NEG	NEG	POS	NEG	NEG	POS	NEG	POS	NEG	POS
08V11213		POS		NEG	POS		AMB	NEG	POS	NEG	NEG	POS	NEG	POS	NEG	POS
08V12100		POS		NEG	POS		NEG	NEG	POS	NEG	NEG	POS	NEG	POS	NEG	AMB
08V13847		POS		NEG	POS		AMB	NEG	POS	NEG	NEG	POS	NEG	POS	NEG	AMB
08V15905	POS	POS	POS	NEG	POS	NEG	AMB	NEG	POS	NEG	NEG	POS	NEG	POS	NEG	POS
08V18191	POS	POS	AMB	NEG	POS	AMB	NEG	NEG	POS	NEG	NEG	POS	NEG	POS	NEG	POS
08V21425		POS		NEG	POS		NEG	NEG	POS	NEG	NEG	POS	NEG	POS	NEG	POS
08V23868		POS		NEG	POS		NEG	NEG	POS	NEG	NEG	POS	NEG	POS	NEG	POS
08V27435		POS		NEG	POS		NEG	NEG	POS	NEG	NEG	POS	NEG	POS	NEG	POS
08V27514		POS		NEG	POS		AMB	NEG	POS	NEG	NEG	POS	NEG	POS	NEG	POS
08V28482		POS		NEG	POS		NEG	NEG	POS	NEG	NEG	POS	NEG	POS	NEG	POS
08V29312		POS		NEG	POS		AMB	NEG	POS	NEG	NEG	POS	NEG	POS	NEG	POS
08V29889		POS		NEG	POS		AMB	NEG	POS	NEG	NEG	POS	NEG	POS	NEG	POS
08V30696		POS		NEG	POS		AMB	NEG	POS	NEG	NEG	POS	NEG	POS	NEG	POS
08V32764		POS		POS	POS		AMB	NEG	POS	NEG	NEG	POS	NEG	POS	NEG	AMB
08V36052		POS		NEG	POS		NEG	NEG	POS	NEG	NEG	POS	NEG	POS	NEG	POS
08V36125		POS		NEG	POS		NEG	NEG	POS	NEG	NEG	POS	NEG	POS	NEG	POS
08V39935		POS		NEG	POS		NEG	NEG	POS	NEG	NEG	POS	NEG	POS	NEG	POS
08V41631		POS		NEG	POS		NEG	NEG	POS	NEG	NEG	POS	NEG	POS	NEG	POS
08V42588		POS		NEG	POS		NEG	NEG	POS	NEG	NEG	POS	NEG	POS	NEG	POS
08V42633		POS		NEG	POS		NEG	NEG	POS	NEG	NEG	POS	NEG	POS	NEG	POS
08V43410		POS		NEG	POS		NEG	NEG	POS	NEG	NEG	POS	NEG	POS	NEG	AMB
08V43581		POS		NEG	POS		NEG	NEG	POS	NEG	NEG	POS	NEG	POS	NEG	POS
08V47242	POS	POS	POS	NEG	POS	NEG	NEG	NEG	POS	NEG	NEG	POS	NEG	POS	NEG	POS
08V50740	POS	POS	POS	NEG	POS	NEG	NEG	NEG	POS	NEG	NEG	POS	NEG	POS	NEG	POS
08V02855	POS	POS	POS	NEG	POS	NEG	NEG	NEG	NEG	NEG	NEG	POS	NEG	POS	NEG	POS
08V35987	POS	POS	NEG	NEG	POS	NEG	NEG	POS	NEG	NEG	NEG	POS	NEG	POS	NEG	POS
08V39853	POS	POS	POS	NEG	POS	NEG	NEG	POS	NEG	NEG	NEG	POS	NEG	POS	NEG	POS
08F01314	POS	POS	AMB	NEG	POS	NEG	NEG	POS	NEG	NEG	NEG	POS	NEG	POS	NEG	POS
08V15773	POS	POS	POS	NEG	POS	NEG	NEG	POS	NEG	NEG	NEG	POS	NEG	POS	NEG	POS
08B02942		AMB		NEG	NEG		NEG	NEG	NEG	NEG	NEG	NEG	NEG	NEG	NEG	NEG
08F00816		NEG		NEG	NEG		NEG	NEG	NEG	NEG	NEG	NEG	NEG	NEG	NEG	NEG
08F01637		AMB		NEG	NEG		NEG	NEG	NEG	NEG	NEG	NEG	NEG	NEG	NEG	NEG
08F04948		AMB		NEG	NEG		NEG	NEG	NEG	NEG	NEG	NEG	NEG	NEG	NEG	NEG
08U07826		NEG		NEG	NEG		NEG	NEG	NEG	NEG	NEG	NEG	NEG	NEG	NEG	NEG
08V00371		POS		NEG	NEG		NEG	NEG	NEG	NEG	NEG	NEG	NEG	NEG	NEG	NEG
08V02049		AMB		NEG	NEG		NEG	NEG	NEG	NEG	NEG	NEG	NEG	NEG	NEG	NEG
08V02349		AMB		NEG	NEG		NEG	NEG	NEG	NEG	NEG	NEG	NEG	NEG	NEG	NEG
08V03329		AMB		NEG	NEG		NEG	NEG	NEG	NEG	NEG	NEG	NEG	NEG	NEG	NEG
08V04516		POS		NEG	POS		NEG	NEG	NEG	NEG	NEG	NEG	NEG	NEG	NEG	NEG
08V04681		POS		NEG	NEG		POS	NEG	NEG	NEG	NEG	NEG	NEG	NEG	NEG	NEG
08V04740		POS		NEG	NEG		NEG	NEG	NEG	NEG	NEG	NEG	NEG	NEG	NEG	NEG
08V04977		NEG		NEG	NEG		NEG	NEG	NEG	NEG	NEG	NEG	NEG	NEG	NEG	NEG
08V06376		NEG		NEG	NEG		NEG	NEG	NEG	NEG	NEG	NEG	NEG	NEG	NEG	NEG
08V09540		NEG		NEG	NEG		NEG	NEG	NEG	NEG	NEG	NEG	NEG	NEG	NEG	NEG
08V10287		NEG		NEG	NEG		NEG	NEG	NEG	NEG	NEG	NEG	NEG	NEG	NEG	NEG
08V10376		NEG		NEG	NEG		NEG	NEG	NEG	NEG	NEG	NEG	NEG	NEG	NEG	NEG
08V10714		NEG		NEG	NEG		NEG	NEG	NEG	NEG	NEG	NEG	NEG	NEG	NEG	NEG
08V10836		POS		NEG	NEG		NEG	NEG	NEG	NEG	NEG	NEG	NEG	NEG	NEG	NEG
08V11971		POS		NEG	POS		NEG	NEG	NEG	NEG	NEG	NEG	NEG	NEG	NEG	NEG
08V14130		AMB		NEG	POS		NEG	NEG	NEG	NEG	NEG	NEG	NEG	NEG	NEG	NEG
08V14723		AMB		NEG	NEG		NEG	NEG	NEG	NEG	NEG	NEG	NEG	NEG	NEG	NEG
08V16292		AMB		NEG	POS		AMB	NEG	NEG	NEG	NEG	NEG	NEG	NEG	NEG	NEG
08V16554		AMB		NEG	NEG		NEG	NEG	NEG	NEG	NEG	NEG	NEG	NEG	NEG	NEG
08V18482		AMB		NEG	AMB		POS	NEG	NEG	NEG	NEG	NEG	NEG	NEG	NEG	NEG
08V19658		NEG		NEG	NEG		NEG	NEG	NEG	NEG	NEG	NEG	NEG	NEG	NEG	NEG
08V19663		NEG		NEG	NEG		NEG	NEG	NEG	NEG	NEG	NEG	NEG	NEG	NEG	NEG
08V20266		NEG		NEG	NEG		NEG	NEG	NEG	NEG	NEG	NEG	NEG	NEG	NEG	NEG
08V20337		AMB		NEG	NEG		NEG	NEG	NEG	NEG	NEG	NEG	NEG	NEG	NEG	NEG
08V21055		AMB		NEG	POS		AMB	NEG	NEG	NEG	NEG	NEG	NEG	NEG	NEG	NEG
08V23747		NEG		NEG	NEG		NEG	NEG	NEG	NEG	NEG	NEG	NEG	NEG	NEG	NEG
08V23813		NEG		NEG	NEG		NEG	NEG	NEG	NEG	NEG	NEG	NEG	NEG	NEG	NEG
08V25135		NEG		NEG	NEG		NEG	NEG	NEG	NEG	NEG	NEG	NEG	NEG	NEG	NEG
08V27329		AMB		NEG	NEG		NEG	NEG	NEG	NEG	NEG	NEG	NEG	NEG	NEG	NEG
08V27434		POS		NEG	POS		NEG	NEG	NEG	NEG	NEG	NEG	NEG	NEG	NEG	NEG
08V27729		AMB		NEG	POS		AMB	NEG	NEG	NEG	NEG	NEG	NEG	NEG	NEG	NEG
08V31445		AMB		NEG	NEG		NEG	NEG	NEG	NEG	NEG	NEG	NEG	NEG	NEG	NEG
08V34812		NEG		NEG	NEG		NEG	NEG	NEG	NEG	NEG	NEG	NEG	NEG	NEG	NEG
08V34813		AMB		NEG	POS		NEG	NEG	NEG	NEG	NEG	NEG	NEG	NEG	NEG	NEG
08V35361		NEG		NEG	NEG		NEG	NEG	NEG	NEG	NEG	NEG	NEG	NEG	NEG	NEG
08V37931		AMB		NEG	NEG		NEG	NEG	NEG	NEG	NEG	NEG	NEG	NEG	NEG	NEG
08V45578		POS		NEG	POS		NEG	NEG	NEG	NEG	NEG	NEG	NEG	POS	NEG	POS
08V47385	NEG	POS	POS	NEG	NEG	NEG	NEG	NEG	NEG	NEG	NEG	NEG	NEG	NEG	NEG	NEG
08V53678	NEG	POS	POS	NEG	NEG	NEG	NEG	NEG	NEG	NEG	NEG	NEG	NEG	NEG	NEG	NEG
08B09737		NEG		POS	AMB		POS	NEG	NEG	NEG	NEG	AMB	POS	NEG	NEG	NEG
08B14321	NEG	NEG	NEG	POS	AMB	NEG	POS	NEG	NEG	NEG	NEG	NEG	POS	NEG	AMB	NEG
08F01439		NEG		POS	AMB		POS	NEG	NEG	NEG	NEG	NEG	POS	NEG	POS	POS
08U12518		NEG		POS	AMB		POS	NEG	NEG	NEG	NEG	NEG	POS	NEG	NEG	NEG
08U13653	NEG	NEG	NEG	POS	AMB	NEG	POS	NEG	NEG	NEG	NEG	NEG	POS	NEG	NEG	NEG
08V00675		NEG		POS	NEG		POS	NEG	NEG	NEG	NEG	NEG	POS	NEG	AMB	NEG
08V02459		NEG		POS	NEG		POS	NEG	NEG	NEG	NEG	NEG	POS	NEG	AMB	NEG
08V02654		NEG		POS	NEG		POS	NEG	NEG	NEG	NEG	NEG	POS	NEG	NEG	NEG
08V05677	NEG	NEG	NEG	POS	AMB	NEG	POS	NEG	NEG	NEG	NEG	NEG	POS	NEG	AMB	AMB
08V20930		NEG		POS	AMB		POS	AMB	NEG	NEG	NEG	NEG	POS	NEG	NEG	POS
08V23911		NEG		POS	AMB		POS	NEG	NEG	NEG	NEG	NEG	POS	NEG	NEG	NEG
08V39175		NEG		POS	AMB		POS	NEG	NEG	NEG	NEG	NEG	POS	NEG	NEG	NEG
08V39949		NEG		POS	AMB		POS	NEG	NEG	NEG	NEG	NEG	POS	NEG	NEG	NEG
08V53670	POS	POS	POS	NEG	POS	NEG	AMB	NEG	NEG	NEG	AMB	POS	NEG	POS	NEG	POS
08V06946	NEG	NEG	NEG	AMB	AMB	NEG	POS	NEG	NEG	NEG	NEG	NEG	POS	NEG	POS	AMB

CAPSULE- AND BIOFILM-ASSOCIATED GENES

ISOLATE:	Capsule type 1	capsular poly-saccharide synthesis enzyme	O-antigen poly-merase	capsular poly-saccharide biosyn- thesis protein	Capsule type 5	capsular poly-saccharide synthesis enzyme	O-antigen poly-merase	capsular poly-saccharide biosyn- thesis protein	Capsule type 8	capsular poly-saccharide synthesis enzyme	capsular poly-saccharide biosyn- thesis protein	O-antigen poly-merase	capsular poly-saccharide biosyn- thesis protein	intercellular adhesion protein A	intercellular adhesion protein C	biofilm PIA synthesis protein D	surface protein involved in biofilm formation
	cap 1 (total)	capH1	capJ1	capK1	cap 5 (total)	capH5	capJ5	capK5	cap 8 (total)	capH8	capI8	capJ8	capK8	icaA	icaC	icaD	bap
08V04188																	
08V21565																	
08V53546	NEG	NEG	NEG	NEG	POS	POS	POS	POS	NEG	NEG	NEG	NEG	NEG	POS	POS	POS	NEG
08B04687																	
08F01184																	
08F01352																	
08F01711																	
08F03746																	
08F04303																	
08F04396																	
08U11214																	
08V01047																	
08V01222																	
08V01315																	
08V06126																	
08V06171																	
08V06623																	
08V10539																	
08V11213																	
08V12100																	
08V13847																	
08V15905	NEG	NEG	NEG	NEG	POS	POS	POS	POS	NEG	NEG	NEG	NEG	NEG	POS	POS	POS	NEG
08V18191	NEG	NEG	NEG	NEG	POS	POS	POS	POS	NEG	NEG	NEG	NEG	NEG	POS	POS	POS	NEG
08V21425																	
08V23868																	
08V27435																	
08V27514																	
08V28482																	
08V29312																	
08V29889																	
08V30696																	
08V32764																	
08V36052																	
08V36125																	
08V39935																	
08V41631																	
08V42588																	
08V42633																	
08V43410																	
08V43581																	
08V47242	NEG	NEG	NEG	NEG	POS	POS	POS	POS	NEG	NEG	NEG	NEG	NEG	POS	POS	POS	NEG
08V50740	NEG	NEG	NEG	NEG	POS	POS	POS	POS	NEG	NEG	NEG	NEG	NEG	POS	POS	POS	NEG
08V02855	NEG	NEG	NEG	NEG	NEG	NEG	NEG	NEG	POS	POS	POS	POS	POS	POS	POS	POS	NEG
08V35987	NEG	NEG	NEG	NEG	NEG	NEG	NEG	NEG	POS	POS	POS	POS	POS	POS	POS	POS	NEG
08V39853	NEG	NEG	NEG	NEG	POS	POS	POS	POS	NEG	NEG	NEG	NEG	NEG	POS	POS	POS	NEG
08F01314	NEG	NEG	NEG	NEG	POS	POS	POS	POS	NEG	NEG	NEG	NEG	NEG	POS	POS	POS	NEG
08V15773	NEG	NEG	NEG	NEG	POS	POS	POS	POS	NEG	NEG	NEG	NEG	NEG	POS	POS	POS	NEG
08B02942																	
08F00816																	
08F01637																	
08F04948																	
08U07826																	
08V00371																	
08V02049																	
08V02349																	
08V03329																	
08V04516																	
08V04681																	
08V04740																	
08V04977																	
08V06376																	
08V09540																	
08V10287																	
08V10376																	
08V10714																	
08V10836																	
08V11971																	
08V14130																	
08V14723																	
08V16292																	
08V16554																	
08V18482																	
08V19658																	
08V19663																	
08V20266																	
08V20337																	
08V21055																	
08V23747																	
08V23813																	
08V25135																	
08V27329																	
08V27434																	
08V27729																	
08V31445																	
08V34812																	
08V34813																	
08V35361																	
08V37931																	
08V45578																	
08V47385	NEG	NEG	NEG	NEG	POS	POS	POS	POS	NEG	NEG	NEG	NEG	NEG	POS	POS	POS	NEG
08V53678	NEG	NEG	NEG	NEG	POS	POS	POS	POS	NEG	NEG	NEG	NEG	NEG	POS	POS	POS	NEG
08B09737																	
08B14321	NEG	NEG	NEG	NEG	NEG	NEG	NEG	NEG	POS	POS	POS	POS	POS	POS	POS	POS	NEG
08F01439																	
08U12518																	
08U13653	NEG	NEG	NEG	NEG	NEG	NEG	NEG	NEG	POS	POS	POS	POS	POS	POS	POS	POS	NEG
08V00675																	
08V02459																	
08V02654																	
08V05677	NEG	NEG	NEG	NEG	NEG	NEG	NEG	NEG	POS	POS	POS	POS	POS	POS	POS	POS	NEG
08V20930																	
08V23911																	
08V39175																	
08V39949																	
08V53670	NEG	NEG	NEG	NEG	NEG	NEG	NEG	NEG	POS	AMB	AMB	POS	POS	POS	POS	POS	NEG
08V06946	NEG	NEG	NEG	NEG	POS	POS	POS	POS	NEG	NEG	NEG	NEG	NEG	POS	POS	POS	NEG

ISOLATE:	STRAIN	ADHAESION FACTORS / GENES ENCODING MICROBIAL SURFACE COMPONENTS RECOGNIZING ADHESIVE MATRIX MOLECULES (MSCRAMM GENES)																																													
		bone sialoprotein-binding protein							clumping factor A					clumping factor B					collagen-binding adhesin	cell wall associated fibronectin-binding protein	cell surface elastin binding protein					enolase	fibrinogen binding protein (19 kDa)		fibronectin-binding protein A						fibronectin-binding protein B							Major histocompatibility complex class II analog protein (=Extracellular adherence protein, eap)				Staphylococcus aureus surface protein G			
		bbp (total)	bbp (consensus)	bbp (COL+MW2)	bbp (MRSA252)	bbp (Mu50)	bbp (RF122)	bbp (ST45)	clfA (total)	clfA (consensus)	clfA (COL+RF122)	clfA (MRSA252)	clfA (Mu50+MW2)	clfB (total)	clfB (consensus)	clfB (COL+Mu50)	clfB (MW2)	clfB (RF122)	cna	ebh (consensus)	ebpS (total)	ebpS_probe 612	ebpS_probe 614	ebpS (01-1111)	ebpS (COL)	eno	fib	fib (MRSA252)	fnbA (total)	fnbA (consensus)	fnbA (COL)	fnbA (MRSA252)	fnbA (Mu50+MW2)	fnbA (RF122)	fnbB (total)	fnbB (COL)	fnbB (COL+Mu50+MW2)	fnbB (Mu50)	fnbB (MW2)	fnbB (ST15)	fnbB (ST45-2)	map (total)	map (COL)	map (MRSA252)	map (Mu50+MW2)	sasG (total)	sasG (COL+Mu50)	sasG (MW2)	sasG (other than MRSA252+RF122)
2008																																																	
08V04188	CC5/ST228-MRSA-I, South German EMRSA																																																
08V21565	CC5/ST228-MRSA-I, South German EMRSA																																																
08V53546	CC5/ST228-MRSA-I, South German EMRSA	POS	POS	NEG	NEG	POS	NEG	NEG	NEG	NEG	NEG	NEG	NEG	POS	POS	POS	NEG	NEG	NEG	POS	POS	NEG	POS	NEG	NEG	POS	POS	NEG	POS	POS	NEG	NEG	POS	NEG	NEG	NEG	NEG	NEG	NEG	NEG	NEG	POS	POS	NEG	POS	POS	POS	NEG	POS
08B04687	CC5-MRSA-II, Rhine-Hesse EMRSA/UK-EMRSA-3																																																
08F01184	CC5-MRSA-II, Rhine-Hesse EMRSA/UK-EMRSA-3																																																
08F01352	CC5-MRSA-II, Rhine-Hesse EMRSA/UK-EMRSA-3																																																
08F01711	CC5-MRSA-II, Rhine-Hesse EMRSA/UK-EMRSA-3																																																
08F03746	CC5-MRSA-II, Rhine-Hesse EMRSA/UK-EMRSA-3																																																
08F04303	CC5-MRSA-II, Rhine-Hesse EMRSA/UK-EMRSA-3																																																
08F04396	CC5-MRSA-II, Rhine-Hesse EMRSA/UK-EMRSA-3																																																
08U11214	CC5-MRSA-II, Rhine-Hesse EMRSA/UK-EMRSA-3																																																
08V01047	CC5-MRSA-II, Rhine-Hesse EMRSA/UK-EMRSA-3																																																
08V01222	CC5-MRSA-II, Rhine-Hesse EMRSA/UK-EMRSA-3																																																
08V01315	CC5-MRSA-II, Rhine-Hesse EMRSA/UK-EMRSA-3																																																
08V06126	CC5-MRSA-II, Rhine-Hesse EMRSA/UK-EMRSA-3																																																
08V06171	CC5-MRSA-II, Rhine-Hesse EMRSA/UK-EMRSA-3																																																
08V06623	CC5-MRSA-II, Rhine-Hesse EMRSA/UK-EMRSA-3																																																
08V10539	CC5-MRSA-II, Rhine-Hesse EMRSA/UK-EMRSA-3																																																
08V11213	CC5-MRSA-II, Rhine-Hesse EMRSA/UK-EMRSA-3																																																
08V12100	CC5-MRSA-II, Rhine-Hesse EMRSA/UK-EMRSA-3																																																
08V13847	CC5-MRSA-II, Rhine-Hesse EMRSA/UK-EMRSA-3																																																
08V15905	CC5-MRSA-II, Rhine-Hesse EMRSA/UK-EMRSA-3	POS	POS	NEG	NEG	POS	NEG	NEG	POS	POS	NEG	NEG	POS	POS	POS	POS	NEG	NEG	NEG	POS	POS	POS	POS	NEG	NEG	POS	POS	NEG	POS	POS	NEG	NEG	POS	NEG	POS	NEG	AMB	POS	NEG	NEG	NEG	POS	POS	NEG	POS	POS	POS	NEG	POS
08V18191	CC5-MRSA-II, Rhine-Hesse EMRSA/UK-EMRSA-3	POS	POS	NEG	NEG	POS	NEG	NEG	POS	POS	NEG	NEG	POS	POS	POS	POS	NEG	NEG	NEG	POS	POS	POS	POS	NEG	NEG	POS	POS	NEG	POS	POS	NEG	NEG	POS	NEG	POS	NEG	AMB	POS	NEG	NEG	NEG	POS	AMB	NEG	POS	POS	POS	NEG	POS
08V21425	CC5-MRSA-II, Rhine-Hesse EMRSA/UK-EMRSA-3																																																
08V23868	CC5-MRSA-II, Rhine-Hesse EMRSA/UK-EMRSA-3																																																
08V27435	CC5-MRSA-II, Rhine-Hesse EMRSA/UK-EMRSA-3																																																
08V27514	CC5-MRSA-II, Rhine-Hesse EMRSA/UK-EMRSA-3																																																
08V28482	CC5-MRSA-II, Rhine-Hesse EMRSA/UK-EMRSA-3																																																
08V29312	CC5-MRSA-II, Rhine-Hesse EMRSA/UK-EMRSA-3																																																
08V29889	CC5-MRSA-II, Rhine-Hesse EMRSA/UK-EMRSA-3																																																
08V30696	CC5-MRSA-II, Rhine-Hesse EMRSA/UK-EMRSA-3																																																
08V32764	CC5-MRSA-II, Rhine-Hesse EMRSA/UK-EMRSA-3																																																
08V36052	CC5-MRSA-II, Rhine-Hesse EMRSA/UK-EMRSA-3																																																
08V36125	CC5-MRSA-II, Rhine-Hesse EMRSA/UK-EMRSA-3																																																
08V39935	CC5-MRSA-II, Rhine-Hesse EMRSA/UK-EMRSA-3																																																
08V41631	CC5-MRSA-II, Rhine-Hesse EMRSA/UK-EMRSA-3																																																
08V42588	CC5-MRSA-II, Rhine-Hesse EMRSA/UK-EMRSA-3																																																
08V42633	CC5-MRSA-II, Rhine-Hesse EMRSA/UK-EMRSA-3																																																
08V43410	CC5-MRSA-II, Rhine-Hesse EMRSA/UK-EMRSA-3																																																
08V43581	CC5-MRSA-II, Rhine-Hesse EMRSA/UK-EMRSA-3																																																
08V47242	CC5-MRSA-II, Rhine-Hesse EMRSA/UK-EMRSA-3	POS	POS	NEG	NEG	POS	NEG	NEG	POS	POS	NEG	NEG	POS	POS	POS	POS	NEG	NEG	NEG	POS	POS	POS	POS	NEG	NEG	POS	POS	NEG	POS	POS	NEG	NEG	POS	NEG	POS	NEG	AMB	POS	NEG	NEG	NEG	POS	POS	NEG	POS	POS	POS	NEG	POS
08V50740	CC5-MRSA-II, Rhine-Hesse EMRSA/UK-EMRSA-3	POS	POS	NEG	NEG	POS	NEG	NEG	POS	POS	NEG	NEG	POS	POS	POS	POS	NEG	NEG	NEG	POS	POS	NEG	POS	NEG	NEG	POS	POS	NEG	POS	POS	NEG	NEG	POS	NEG	POS	NEG	POS	AMB	NEG	NEG	NEG	POS	POS	NEG	POS	POS	POS	NEG	POS
08V02855	CC7-MRSA-IV	POS	POS	POS	POS	NEG	NEG	NEG	POS	POS	POS	NEG	POS	POS	POS	NEG	NEG	NEG	NEG	POS	POS	POS	POS	NEG	POS	POS	POS	NEG	POS	POS	NEG	NEG	NEG	NEG	POS	NEG	POS	NEG	NEG	NEG	POS	POS	NEG	NEG	POS	NEG	NEG	NEG	NEG
08V35987	CC8/ST239-MRSA-III, Vienna/Hungarian/Brazilian Clone	POS	POS	POS	NEG	NEG	NEG	NEG	POS	POS	POS	AMB	AMB	POS	POS	NEG	NEG	NEG	POS	POS	POS	POS	POS	NEG	POS	POS	POS	NEG	POS	POS	POS	NEG	NEG	NEG	NEG	NEG	NEG	NEG	NEG	NEG	NEG	POS	POS	NEG	NEG	POS	POS	NEG	POS
08V39853	CC8/ST254-MRSA-(atypical SCCmec), Hannover EMRSA	NEG	NEG	NEG	NEG	NEG	NEG	NEG	POS	NEG	POS	AMB	AMB	POS	POS	POS	NEG	NEG	NEG	POS	POS	POS	POS	NEG	POS	POS	POS	NEG	POS	POS	POS	NEG	NEG	NEG	POS	AMB	POS	NEG	NEG	NEG	NEG	POS	POS	NEG	NEG	POS	POS	NEG	POS
08F01314	CC8-MRSA-IV, UK-EMRSA-14/WA MRSA-5	POS	POS	POS	NEG	NEG	NEG	NEG	NEG	NEG	NEG	NEG	NEG	POS	POS	POS	NEG	NEG	NEG	POS	POS	POS	POS	NEG	POS	POS	POS	NEG	POS	POS	POS	NEG	NEG	NEG	POS	AMB	POS	NEG	NEG	NEG	NEG	POS	POS	NEG	NEG	POS	POS	NEG	POS
08V15773	CC8-MRSA-IV, USA300	POS	POS	POS	NEG	NEG	NEG	NEG	POS	POS	POS	AMB	AMB	POS	POS	POS	NEG	NEG	NEG	POS	POS	POS	POS	NEG	POS	POS	POS	NEG	POS	POS	POS	NEG	NEG	NEG	POS	AMB	POS	NEG	NEG	NEG	NEG	POS	POS	NEG	NEG	POS	POS	NEG	POS
08B02942	CC22-MRSA-IV, Barnim/UK-EMRSA-15																																																
08F00816	CC22-MRSA-IV, Barnim/UK-EMRSA-15																																																
08F01637	CC22-MRSA-IV, Barnim/UK-EMRSA-15																																																
08F04948	CC22-MRSA-IV, Barnim/UK-EMRSA-15																																																
08U07826	CC22-MRSA-IV, Barnim/UK-EMRSA-15																																																
08V00371	CC22-MRSA-IV, Barnim/UK-EMRSA-15																																																
08V02049	CC22-MRSA-IV, Barnim/UK-EMRSA-15																																																
08V02349	CC22-MRSA-IV, Barnim/UK-EMRSA-15																																																
08V03329	CC22-MRSA-IV, Barnim/UK-EMRSA-15																																																
08V04516	CC22-MRSA-IV, Barnim/UK-EMRSA-15																																																
08V04681	CC22-MRSA-IV, Barnim/UK-EMRSA-15																																																
08V04740	CC22-MRSA-IV, Barnim/UK-EMRSA-15																																																
08V04977	CC22-MRSA-IV, Barnim/UK-EMRSA-15																																																
08V06376	CC22-MRSA-IV, Barnim/UK-EMRSA-15																																																
08V09540	CC22-MRSA-IV, Barnim/UK-EMRSA-15																																																
08V10287	CC22-MRSA-IV, Barnim/UK-EMRSA-15																																																
08V10376	CC22-MRSA-IV, Barnim/UK-EMRSA-15																																																
08V10714	CC22-MRSA-IV, Barnim/UK-EMRSA-15																																																
08V10836	CC22-MRSA-IV, Barnim/UK-EMRSA-15																																																
08V11971	CC22-MRSA-IV, Barnim/UK-EMRSA-15																																																
08V14130	CC22-MRSA-IV, Barnim/UK-EMRSA-15																																																
08V14723	CC22-MRSA-IV, Barnim/UK-EMRSA-15																																																
08V16292	CC22-MRSA-IV, Barnim/UK-EMRSA-15																																																
08V16554	CC22-MRSA-IV, Barnim/UK-EMRSA-15																																																
08V18482	CC22-MRSA-IV, Barnim/UK-EMRSA-15																																																
08V19658	CC22-MRSA-IV, Barnim/UK-EMRSA-15																																																
08V19663	CC22-MRSA-IV, Barnim/UK-EMRSA-15																																																
08V20266	CC22-MRSA-IV, Barnim/UK-EMRSA-15																																																
08V20337	CC22-MRSA-IV, Barnim/UK-EMRSA-15																																																
08V21055	CC22-MRSA-IV, Barnim/UK-EMRSA-15																																																
08V23747	CC22-MRSA-IV, Barnim/UK-EMRSA-15																																																
08V23813	CC22-MRSA-IV, Barnim/UK-EMRSA-15																																																
08V25135	CC22-MRSA-IV, Barnim/UK-EMRSA-15																																																
08V27329	CC22-MRSA-IV, Barnim/UK-EMRSA-15																																																
08V27434	CC22-MRSA-IV, Barnim/UK-EMRSA-15																																																
08V27729	CC22-MRSA-IV, Barnim/UK-EMRSA-15																																																
08V31445	CC22-MRSA-IV, Barnim/UK-EMRSA-15																																																
08V34812	CC22-MRSA-IV, Barnim/UK-EMRSA-15																																																
08V34813	CC22-MRSA-IV, Barnim/UK-EMRSA-15																																																
08V35361	CC22-MRSA-IV, Barnim/UK-EMRSA-15																																																
08V37931	CC22-MRSA-IV, Barnim/UK-EMRSA-15																																																
08V45578	CC22-MRSA-IV, Barnim/UK-EMRSA-15																																																
08V47385	CC22-MRSA-IV, Barnim/UK-EMRSA-15	POS	POS	NEG	NEG	POS	NEG	NEG	POS	POS	NEG	NEG	POS	POS	POS	NEG	NEG	POS	POS	NEG	POS	POS	POS	NEG	POS	POS	NEG	POS	POS	POS	NEG	NEG	POS	NEG	NEG	NEG	NEG	NEG	NEG	NEG	NEG	POS	NEG	NEG	POS	POS	NEG	POS	POS
08V53678	CC22-MRSA-IV, Barnim/UK-EMRSA-15	POS	POS	NEG	NEG	NEG	NEG	NEG	POS	POS	NEG	NEG	POS	POS	POS	NEG	NEG	NEG	POS	NEG	POS	AMB	POS	NEG	POS	POS	NEG	POS	POS	POS	NEG	NEG	POS	NEG	NEG	NEG	NEG	NEG	NEG	NEG	NEG	POS	NEG	NEG	POS	POS	NEG	POS	POS
08B09737	CC45-MRSA-IV, Berlin EMRSA																																																
08B14321	CC45-MRSA-IV, Berlin EMRSA	POS	POS	NEG	NEG	NEG	NEG	POS	POS	POS	AMB	NEG	POS	POS	POS	NEG	POS	AMB	POS	POS	POS	NEG	POS	POS	NEG	POS	NEG	POS	POS	POS	NEG	NEG	POS	NEG	POS	NEG	POS	AMB	NEG	NEG	NEG	POS	NEG	POS	NEG	NEG	NEG	NEG	NEG
08F01439	CC45-MRSA-IV, Berlin EMRSA																																																
08U12518	CC45-MRSA-IV, Berlin EMRSA																																																
08U13653	CC45-MRSA-IV, Berlin EMRSA	POS	POS	NEG	NEG	NEG	NEG	POS	POS	POS	AMB	NEG	POS	POS	POS	NEG	POS	AMB	POS	POS	POS	NEG	POS	POS	NEG	POS	NEG	POS	POS	POS	NEG	NEG	POS	NEG	POS	NEG	POS	AMB	NEG	AMB	NEG	POS	NEG	POS	NEG	NEG	NEG	NEG	NEG
08V00675	CC45-MRSA-IV, Berlin EMRSA																																																
08V02459	CC45-MRSA-IV, Berlin EMRSA																																																
08V02654	CC45-MRSA-IV, Berlin EMRSA																																																
08V05677	CC45-MRSA-IV, Berlin EMRSA	POS	POS	NEG	NEG	NEG	NEG	POS	POS	POS	AMB	NEG	POS	POS	POS	NEG	POS	AMB	POS	POS	POS	AMB	POS	POS	NEG	POS	NEG	POS	POS	POS	NEG	NEG	POS	NEG	POS	NEG	AMB	POS	NEG	NEG	NEG	POS	NEG	POS	NEG	NEG	NEG	NEG	NEG
08V20930	CC45-MRSA-IV, Berlin EMRSA																																																
08V23911	CC45-MRSA-IV, Berlin EMRSA																																																
08V39175	CC45-MRSA-IV, Berlin EMRSA																																																
08V39949	CC45-MRSA-IV, Berlin EMRSA																																																
08V53670	CC88-MRSA-IV, WA MRSA-2	POS	POS	NEG	NEG	POS	NEG	NEG	POS	POS	NEG	NEG	POS	POS	POS	NEG	NEG	NEG	NEG	POS	POS	POS	POS	NEG	POS	POS	POS	NEG	POS	POS	NEG	NEG	NEG	NEG	POS	NEG	POS	AMB	NEG	NEG	NEG	POS	NEG	NEG	POS	POS	NEG	POS	POS
08V06946	CC398-MRSA-V	POS	POS	NEG	NEG	NEG	NEG	POS	POS	POS	POS	AMB	POS	POS	POS	NEG	POS	AMB	POS	POS	POS	NEG	POS	POS	POS	POS	NEG	POS	POS	POS	NEG	NEG	NEG	NEG	POS	NEG	POS	AMB	NEG	NEG	NEG	POS	POS	POS	NEG	NEG	NEG	NEG	NEG

ISOLATE:	STRAIN	ADHAESION FACTORS / GENES ENCODING MICROBIAL SURFACE COMPONENTS RECOGNIZING ADHESIVE MATRIX MOLECULES (MSCRAMM GENES)																		IMMUNOD.ANTIGEN B		DEFENSIN RESIST.		TRANSFERRIN BINDING PROT			PUTATIVE TRANSPORTER		TYPE I RESTRICTION-MODIFICATION SYSTEM, SINGLE SEQUENCE SPECIFICITY PROTEIN												MISCELLANEOUS GENES			HYALURONATE LYASE							
		Ser-Asp rich fibrinogen-/bone sialoprotein-binding protein C							Ser-Asp rich fibrinogen-/bone sialoprotein-binding protein D					van Willebrand factor binding protein						immunodominant antigen B		defensin resistance protein		transferrin-binding protein			hypothetical protein, similar to integral membrane protein LmrP		type I site-specific deoxyribo-nuclease subunit, 1st locus	type I site-specific deoxyribonuclease subunit, 2nd locus			type I site-specific deoxyribonuclease subunit, 3rd locus					type I site-specific deoxyribonuclease subunit, unknown locus			hypothetical protein, located next to serine protease operon	Unspecific efflux/trans-porter	hypothetical protein	hyaluronate lyase, first / second locus			hyaluronate lyase, second locus				
		sdrC (total)	sdrC (consensus)	sdrC (B1)	sdrC (COL)	sdrC (Mu50)	sdrC (MW2+MRSA252+RF122)	sdrC (other than MRSA252+RF122)	sdrD (total)	sdrD (consensus)	sdrD (COL+MW2)	sdrD (Mu50)	sdrD (other)	vwb (total)	vwb (consensus)	vwb (COL+MW2)	vwb (MRSA252)	vwb (Mu50)	vwb (RF122)	isaB	isaB (MRSA252)	mprF (COL+MW2)	mprF (Mu50+MRSA252)	isdA (consensus)	isdA (MRSA252)	isdA (other than MRSA252)	lmrP (other than RF122)	lmrP (RF122)	hsdS1 (RF122)	hsdS2 (Mu50+N315+COL+USA300+NCTC8325)	hsdS2 (MW2+MSSA476)	hsdS2 (MRSA252)	hsdS3 (all other than RF122+ MRSA252)	hsdS3 (COL+USA300+ NCTC8325+MW2+MSSA476+RF122)	hsdS3 (Mu50+N315)	hsdS3 (CC51+ MRSA252)	hsdS3 (MRSA252)	hsdSx (CC25)	hsdSx (CC15)	hsdSx (etd)	Q2FXC0	Q2YUB3	Q7A4X2	hysA1 (MRSA252)	hysA1 (MRSA252+RF122) and/or hysA2 (consensus)	hysA1 (MRSA252+RF122) and/or hysA2 (COL+USA300)	hysA2 (all other than MRSA252)	hysA2 (COL+USA300+ NCTC8325)	hysA2 (all other than COL+USA300+NCTC8325)	hysA2 (all other than COL+USA300+NCTC8325)	hysA2 (MRSA252)
**2008**																																																			
08V04188	CC5/ST228-MRSA-I, South German EMRSA																																																		
08V21565	CC5/ST228-MRSA-I, South German EMRSA																																																		
08V53546	CC5/ST228-MRSA-I, South German EMRSA	POS	POS	NEG	NEG	POS	NEG	POS	POS	POS	NEG	POS	NEG	POS	POS	NEG	NEG	POS	NEG	POS	AMB	AMB	POS	POS	NEG	POS	POS	NEG	NEG	NEG	NEG	NEG	POS	NEG	POS	NEG	NEG	POS	NEG	NEG	NEG	NEG	POS	NEG	POS	NEG	NEG	NEG	POS	POS	NEG
08B04687	CC5-MRSA-II, Rhine-Hesse EMRSA/UK-EMRSA-3																																																		
08F01184	CC5-MRSA-II, Rhine-Hesse EMRSA/UK-EMRSA-3																																																		
08F01352	CC5-MRSA-II, Rhine-Hesse EMRSA/UK-EMRSA-3																																																		
08F01711	CC5-MRSA-II, Rhine-Hesse EMRSA/UK-EMRSA-3																																																		
08F03746	CC5-MRSA-II, Rhine-Hesse EMRSA/UK-EMRSA-3																																																		
08F04303	CC5-MRSA-II, Rhine-Hesse EMRSA/UK-EMRSA-3																																																		
08F04396	CC5-MRSA-II, Rhine-Hesse EMRSA/UK-EMRSA-3																																																		
08U11214	CC5-MRSA-II, Rhine-Hesse EMRSA/UK-EMRSA-3																																																		
08V01047	CC5-MRSA-II, Rhine-Hesse EMRSA/UK-EMRSA-3																																																		
08V01222	CC5-MRSA-II, Rhine-Hesse EMRSA/UK-EMRSA-3																																																		
08V01315	CC5-MRSA-II, Rhine-Hesse EMRSA/UK-EMRSA-3																																																		
08V06126	CC5-MRSA-II, Rhine-Hesse EMRSA/UK-EMRSA-3																																																		
08V06171	CC5-MRSA-II, Rhine-Hesse EMRSA/UK-EMRSA-3																																																		
08V06623	CC5-MRSA-II, Rhine-Hesse EMRSA/UK-EMRSA-3																																																		
08V10539	CC5-MRSA-II, Rhine-Hesse EMRSA/UK-EMRSA-3																																																		
08V11213	CC5-MRSA-II, Rhine-Hesse EMRSA/UK-EMRSA-3																																																		
08V12100	CC5-MRSA-II, Rhine-Hesse EMRSA/UK-EMRSA-3																																																		
08V13847	CC5-MRSA-II, Rhine-Hesse EMRSA/UK-EMRSA-3																																																		
08V15905	CC5-MRSA-II, Rhine-Hesse EMRSA/UK-EMRSA-3	POS	POS	NEG	NEG	POS	NEG	POS	POS	POS	NEG	POS	NEG	POS	POS	NEG	NEG	POS	NEG	POS	AMB	AMB	POS	POS	NEG	POS	POS	NEG	NEG	POS	NEG	NEG	POS	NEG	POS	NEG	NEG	POS	NEG	NEG	NEG	NEG	POS	NEG	POS	NEG	POS	NEG	POS	POS	NEG
08V18191	CC5-MRSA-II, Rhine-Hesse EMRSA/UK-EMRSA-3	POS	POS	NEG	NEG	POS	AMB	POS	POS	POS	NEG	POS	NEG	POS	POS	NEG	NEG	POS	NEG	POS	AMB	AMB	POS	POS	NEG	POS	POS	NEG	NEG	POS	NEG	NEG	POS	NEG	POS	NEG	NEG	POS	NEG	NEG	NEG	NEG	POS	NEG	POS	NEG	POS	NEG	POS	POS	NEG
08V21425	CC5-MRSA-II, Rhine-Hesse EMRSA/UK-EMRSA-3																																																		
08V23868	CC5-MRSA-II, Rhine-Hesse EMRSA/UK-EMRSA-3																																																		
08V27435	CC5-MRSA-II, Rhine-Hesse EMRSA/UK-EMRSA-3																																																		
08V27514	CC5-MRSA-II, Rhine-Hesse EMRSA/UK-EMRSA-3																																																		
08V28482	CC5-MRSA-II, Rhine-Hesse EMRSA/UK-EMRSA-3																																																		
08V29312	CC5-MRSA-II, Rhine-Hesse EMRSA/UK-EMRSA-3																																																		
08V29889	CC5-MRSA-II, Rhine-Hesse EMRSA/UK-EMRSA-3																																																		
08V30696	CC5-MRSA-II, Rhine-Hesse EMRSA/UK-EMRSA-3																																																		
08V32764	CC5-MRSA-II, Rhine-Hesse EMRSA/UK-EMRSA-3																																																		
08V36052	CC5-MRSA-II, Rhine-Hesse EMRSA/UK-EMRSA-3																																																		
08V36125	CC5-MRSA-II, Rhine-Hesse EMRSA/UK-EMRSA-3																																																		
08V39935	CC5-MRSA-II, Rhine-Hesse EMRSA/UK-EMRSA-3																																																		
08V41631	CC5-MRSA-II, Rhine-Hesse EMRSA/UK-EMRSA-3																																																		
08V42588	CC5-MRSA-II, Rhine-Hesse EMRSA/UK-EMRSA-3																																																		
08V42633	CC5-MRSA-II, Rhine-Hesse EMRSA/UK-EMRSA-3																																																		
08V43410	CC5-MRSA-II, Rhine-Hesse EMRSA/UK-EMRSA-3																																																		
08V43581	CC5-MRSA-II, Rhine-Hesse EMRSA/UK-EMRSA-3																																																		
08V47242	CC5-MRSA-II, Rhine-Hesse EMRSA/UK-EMRSA-3	POS	POS	NEG	NEG	POS	NEG	POS	POS	POS	NEG	POS	NEG	POS	POS	NEG	NEG	POS	NEG	POS	AMB	AMB	POS	POS	NEG	POS	POS	NEG	NEG	POS	NEG	NEG	POS	NEG	POS	NEG	NEG	POS	NEG	NEG	NEG	NEG	POS	NEG	POS	NEG	POS	NEG	POS	POS	NEG
08V50740	CC5-MRSA-II, Rhine-Hesse EMRSA/UK-EMRSA-3	POS	POS	NEG	NEG	POS	NEG	POS	POS	POS	NEG	POS	NEG	POS	POS	NEG	NEG	POS	NEG	POS	AMB	AMB	POS	POS	NEG	POS	POS	NEG	NEG	POS	NEG	NEG	POS	NEG	POS	NEG	NEG	POS	NEG	NEG	NEG	NEG	POS	NEG	POS	NEG	NEG	NEG	POS	POS	NEG
08V02855	CC7-MRSA-IV	POS	POS	NEG	POS	NEG	NEG	POS	POS	POS	NEG	POS	NEG	POS	POS	NEG	NEG	NEG	NEG	POS	AMB	POS	NEG	POS	NEG	POS	POS	NEG	NEG	NEG	NEG	NEG	NEG	NEG	NEG	NEG	NEG	NEG	NEG	NEG	NEG	NEG	POS	NEG	POS	NEG	NEG	NEG	POS	POS	NEG
08V35987	CC8/ST239-MRSA-III, Vienna/Hungarian/Brazilian Clone	POS	POS	NEG	POS	NEG	NEG	POS	POS	POS	POS	NEG	NEG	POS	POS	POS	NEG	NEG	NEG	NEG	POS	POS	AMB	POS	NEG	POS	POS	NEG	NEG	POS	NEG	NEG	POS	POS	NEG	NEG	NEG	POS	NEG	NEG	POS	NEG	NEG	NEG	POS	POS	AMB	POS	NEG	NEG	NEG
08V39853	CC8/ST254-MRSA-(atypical SCCmec), Hannover EMRSA	POS	POS	NEG	POS	NEG	NEG	POS	POS	POS	AMB	NEG	NEG	POS	POS	POS	NEG	NEG	NEG	POS	AMB	POS	AMB	POS	NEG	POS	POS	NEG	NEG	POS	NEG	NEG	POS	POS	NEG	NEG	NEG	POS	NEG	NEG	pos	NEG	NEG	NEG	POS	POS	POS	POS	NEG	NEG	NEG
08F01314	CC8-MRSA-IV, UK-EMRSA-14/WA MRSA-5	POS	POS	NEG	POS	NEG	NEG	POS	POS	POS	NEG	NEG	NEG	NEG	NEG	NEG	NEG	NEG	NEG	POS	AMB	POS	NEG	POS	NEG	POS	POS	NEG	NEG	POS	NEG	NEG	POS	POS	NEG	NEG	NEG	POS	NEG	NEG	POS	NEG	NEG	NEG	POS	NEG	NEG	POS	NEG	NEG	NEG
08V15773	CC8-MRSA-IV, USA300	POS	POS	NEG	POS	NEG	NEG	POS	POS	POS	POS	NEG	NEG	POS	POS	POS	NEG	NEG	NEG	POS	AMB	POS	NEG	POS	NEG	POS	POS	NEG	NEG	POS	NEG	NEG	POS	POS	NEG	NEG	NEG	POS	NEG	NEG	POS	AMB	NEG	NEG	POS	POS	POS	POS	NEG	NEG	NEG
08B02942	CC22-MRSA-IV, Barnim/UK-EMRSA-15																																																		
08F00816	CC22-MRSA-IV, Barnim/UK-EMRSA-15																																																		
08F01637	CC22-MRSA-IV, Barnim/UK-EMRSA-15																																																		
08F04948	CC22-MRSA-IV, Barnim/UK-EMRSA-15																																																		
08U07826	CC22-MRSA-IV, Barnim/UK-EMRSA-15																																																		
08V00371	CC22-MRSA-IV, Barnim/UK-EMRSA-15																																																		
08V02049	CC22-MRSA-IV, Barnim/UK-EMRSA-15																																																		
08V02349	CC22-MRSA-IV, Barnim/UK-EMRSA-15																																																		
08V03329	CC22-MRSA-IV, Barnim/UK-EMRSA-15																																																		
08V04516	CC22-MRSA-IV, Barnim/UK-EMRSA-15																																																		
08V04681	CC22-MRSA-IV, Barnim/UK-EMRSA-15																																																		
08V04740	CC22-MRSA-IV, Barnim/UK-EMRSA-15																																																		
08V04977	CC22-MRSA-IV, Barnim/UK-EMRSA-15																																																		
08V06376	CC22-MRSA-IV, Barnim/UK-EMRSA-15																																																		
08V09540	CC22-MRSA-IV, Barnim/UK-EMRSA-15																																																		
08V10287	CC22-MRSA-IV, Barnim/UK-EMRSA-15																																																		
08V10376	CC22-MRSA-IV, Barnim/UK-EMRSA-15																																																		
08V10714	CC22-MRSA-IV, Barnim/UK-EMRSA-15																																																		
08V10836	CC22-MRSA-IV, Barnim/UK-EMRSA-15																																																		
08V11971	CC22-MRSA-IV, Barnim/UK-EMRSA-15																																																		
08V14130	CC22-MRSA-IV, Barnim/UK-EMRSA-15																																																		
08V14723	CC22-MRSA-IV, Barnim/UK-EMRSA-15																																																		
08V16292	CC22-MRSA-IV, Barnim/UK-EMRSA-15																																																		
08V16554	CC22-MRSA-IV, Barnim/UK-EMRSA-15																																																		
08V18482	CC22-MRSA-IV, Barnim/UK-EMRSA-15																																																		
08V19658	CC22-MRSA-IV, Barnim/UK-EMRSA-15																																																		
08V19663	CC22-MRSA-IV, Barnim/UK-EMRSA-15																																																		
08V20266	CC22-MRSA-IV, Barnim/UK-EMRSA-15																																																		
08V20337	CC22-MRSA-IV, Barnim/UK-EMRSA-15																																																		
08V21055	CC22-MRSA-IV, Barnim/UK-EMRSA-15																																																		
08V23747	CC22-MRSA-IV, Barnim/UK-EMRSA-15																																																		
08V23813	CC22-MRSA-IV, Barnim/UK-EMRSA-15																																																		
08V25135	CC22-MRSA-IV, Barnim/UK-EMRSA-15																																																		
08V27329	CC22-MRSA-IV, Barnim/UK-EMRSA-15																																																		
08V27434	CC22-MRSA-IV, Barnim/UK-EMRSA-15																																																		
08V27729	CC22-MRSA-IV, Barnim/UK-EMRSA-15																																																		
08V31445	CC22-MRSA-IV, Barnim/UK-EMRSA-15																																																		
08V34812	CC22-MRSA-IV, Barnim/UK-EMRSA-15																																																		
08V34813	CC22-MRSA-IV, Barnim/UK-EMRSA-15																																																		
08V35361	CC22-MRSA-IV, Barnim/UK-EMRSA-15																																																		
08V37931	CC22-MRSA-IV, Barnim/UK-EMRSA-15																																																		
08V45578	CC22-MRSA-IV, Barnim/UK-EMRSA-15																																																		
08V47385	CC22-MRSA-IV, Barnim/UK-EMRSA-15	POS	POS	NEG	NEG	POS	NEG	POS	POS	POS	NEG	NEG	POS	POS	POS	NEG	NEG	NEG	POS	NEG	POS	AMB	POS	POS	NEG	POS	POS	NEG	NEG	POS	NEG	NEG	POS	NEG	NEG	NEG	NEG	POS	NEG	NEG	NEG	NEG	POS	NEG	POS	NEG	NEG	NEG	POS	POS	NEG
08V53678	CC22-MRSA-IV, Barnim/UK-EMRSA-15	POS	POS	NEG	NEG	POS	NEG	POS	POS	POS	NEG	NEG	POS	POS	POS	NEG	NEG	NEG	POS	NEG	POS	AMB	POS	POS	NEG	POS	POS	NEG	NEG	POS	NEG	NEG	NEG	NEG	NEG	NEG	NEG	AMB	NEG	NEG	NEG	NEG	POS	NEG	POS	NEG	NEG	NEG	POS	POS	NEG
08B09737	CC45-MRSA-IV, Berlin EMRSA																																																		
08B14321	CC45-MRSA-IV, Berlin EMRSA	POS	POS	POS	NEG	NEG	NEG	NEG	POS	POS	POS	NEG	NEG	POS	NEG	NEG	POS	NEG	NEG	NEG	POS	NEG	POS	POS	POS	POS	POS	NEG	NEG	NEG	NEG	POS	NEG	NEG	NEG	NEG	NEG	AMB	NEG	NEG	NEG	NEG	NEG	NEG	POS	NEG	NEG	NEG	POS	NEG	NEG
08F01439	CC45-MRSA-IV, Berlin EMRSA																																																		
08U12518	CC45-MRSA-IV, Berlin EMRSA																																																		
08U13653	CC45-MRSA-IV, Berlin EMRSA	POS	POS	POS	NEG	NEG	NEG	NEG	POS	POS	POS	NEG	NEG	POS	NEG	NEG	POS	NEG	NEG	NEG	POS	NEG	POS	POS	POS	AMB	POS	NEG	NEG	NEG	NEG	POS	NEG	NEG	NEG	NEG	NEG	POS	NEG	NEG	NEG	NEG	POS	NEG	POS	NEG	NEG	NEG	POS	NEG	NEG
08V00675	CC45-MRSA-IV, Berlin EMRSA																																																		
08V02459	CC45-MRSA-IV, Berlin EMRSA																																																		
08V02654	CC45-MRSA-IV, Berlin EMRSA																																																		
08V05677	CC45-MRSA-IV, Berlin EMRSA	POS	POS	POS	NEG	NEG	NEG	NEG	POS	POS	POS	NEG	NEG	POS	NEG	NEG	POS	NEG	NEG	NEG	POS	NEG	NEG	POS	POS	NEG	POS	NEG	NEG	NEG	NEG	POS	NEG	NEG	NEG	NEG	NEG	AMB	NEG	NEG	NEG	NEG	NEG	NEG	AMB	NEG	NEG	NEG	AMB	NEG	NEG
08V20930	CC45-MRSA-IV, Berlin EMRSA																																																		
08V23911	CC45-MRSA-IV, Berlin EMRSA																																																		
08V39175	CC45-MRSA-IV, Berlin EMRSA																																																		
08V39949	CC45-MRSA-IV, Berlin EMRSA																																																		
08V53670	CC88-MRSA-IV, WA MRSA-2	POS	POS	NEG	NEG	POS	POS	POS	POS	POS	NEG	NEG	POS	POS	POS	NEG	NEG	POS	NEG	POS	AMB	POS	AMB	POS	NEG	POS	POS	NEG	POS	NEG	NEG	NEG	POS	NEG	NEG	NEG	POS	POS	NEG	NEG	NEG	NEG	NEG	NEG	POS	NEG	NEG	NEG	POS	POS	NEG
08V06946	CC398-MRSA-V	POS	POS	NEG	NEG	POS	POS	NEG	POS	POS	NEG	NEG	NEG	POS	POS	NEG	NEG	NEG	NEG	NEG	POS	NEG	NEG	POS	POS	NEG	POS	POS	NEG	NEG	NEG	NEG	NEG	NEG	NEG	NEG	NEG	NEG	POS	NEG	NEG	NEG	NEG	NEG	POS	POS	NEG	POS	NEG	NEG	NEG

ISOLATE:	STRAIN	WARD	AGE	GENDER
**2009**				
09V32405	CC1/ST772-MRSA-V [PVL+], Bengal Bay Clone/WA MRSA-60	SUR-OPD	17	m
09V47486	CC1-MRSA-IV, WA MRSA-1/57	SUR-WARD	75	m
09V42357	CC5/ST228-MRSA-I, South German EMRSA	MED-WARD	62	m
09F01028	CC5-MRSA-II, Rhine-Hesse EMRSA/UK-EMRSA-3	MED-WARD	69	f
09F01040	CC5-MRSA-II, Rhine-Hesse EMRSA/UK-EMRSA-3	MED-WARD	74	m
09F03018	CC5-MRSA-II, Rhine-Hesse EMRSA/UK-EMRSA-3	SUR-WARD	79	m
09F03619	CC5-MRSA-II, Rhine-Hesse EMRSA/UK-EMRSA-3	SUR-WARD	54	m
09V01921	CC5-MRSA-II, Rhine-Hesse EMRSA/UK-EMRSA-3	SUR-OPD	71	m
09V04547	CC5-MRSA-II, Rhine-Hesse EMRSA/UK-EMRSA-3	MED-WARD	68	f
09V05744	CC5-MRSA-II, Rhine-Hesse EMRSA/UK-EMRSA-3	SUR-WARD	71	m
09V07448	CC5-MRSA-II, Rhine-Hesse EMRSA/UK-EMRSA-3	SUR-WARD	71	m
09V08032	CC5-MRSA-II, Rhine-Hesse EMRSA/UK-EMRSA-3	MED-ICU	71	f
09V08761	CC5-MRSA-II, Rhine-Hesse EMRSA/UK-EMRSA-3	MED-WARD	52	f
09V21706	CC5-MRSA-II, Rhine-Hesse EMRSA/UK-EMRSA-3	MED-OPD	57	m
09V22079	CC5-MRSA-II, Rhine-Hesse EMRSA/UK-EMRSA-3	SUR-ICU	73	m
09V25980	CC5-MRSA-II, Rhine-Hesse EMRSA/UK-EMRSA-3	SUR-OPD	57	m
09V26221	CC5-MRSA-II, Rhine-Hesse EMRSA/UK-EMRSA-3	MED-WARD	87	m
09V28517	CC5-MRSA-II, Rhine-Hesse EMRSA/UK-EMRSA-3	MED-OPD	69	m
09V28643	CC5-MRSA-II, Rhine-Hesse EMRSA/UK-EMRSA-3	MED-WARD	49	f
09V28647	CC5-MRSA-II, Rhine-Hesse EMRSA/UK-EMRSA-3	MED-WARD	66	f
09V29064	CC5-MRSA-II, Rhine-Hesse EMRSA/UK-EMRSA-3	SUR-ICU	70	m
09V30464	CC5-MRSA-II, Rhine-Hesse EMRSA/UK-EMRSA-3	MED-OPD	63	f
09V30701	CC5-MRSA-II, Rhine-Hesse EMRSA/UK-EMRSA-3	MED-icu	0	m
09V35418	CC5-MRSA-II, Rhine-Hesse EMRSA/UK-EMRSA-3	MED-ICU	87	f
09V37115	CC5-MRSA-II, Rhine-Hesse EMRSA/UK-EMRSA-3	MED-WARD	76	f
09V37466	CC5-MRSA-II, Rhine-Hesse EMRSA/UK-EMRSA-3	SUR-WARD	80	f
09V40100	CC5-MRSA-II, Rhine-Hesse EMRSA/UK-EMRSA-3	SUR-WARD	87	f
09V41542	CC5-MRSA-II, Rhine-Hesse EMRSA/UK-EMRSA-3	SUR-WARD	69	f
09V42595	CC5-MRSA-II, Rhine-Hesse EMRSA/UK-EMRSA-3	SUR-ICU	49	m
09V46894	CC5-MRSA-II, Rhine-Hesse EMRSA/UK-EMRSA-3	MED-WARD	76	f
09V47383	CC5-MRSA-II, Rhine-Hesse EMRSA/UK-EMRSA-3	MED-WARD	74	m
09V47393	CC5-MRSA-II, Rhine-Hesse EMRSA/UK-EMRSA-3	SUR-WARD	78	m
09V34574	CC5-MRSA-IV, Paediatric clone	MED-WARD	36	m
09F00871	CC8/ST72-MRSA-IV, USA700	MED-WARD	62	f
09V20594	CC8-MRSA-IV, USA300	MED-OPD	20	m
09V28877	CC8-MRSA-IV, USA300	MED-OPD	46	m
09F00437	CC22-MRSA-IV, Barnim/UK-EMRSA-15	MED-icu	55	m
09F01062	CC22-MRSA-IV, Barnim/UK-EMRSA-15	SUR-ICU	80	m
09U06692	CC22-MRSA-IV, Barnim/UK-EMRSA-15	MED-WARD	76	f
09V04550	CC22-MRSA-IV, Barnim/UK-EMRSA-15	MED-WARD	82	m
09V05293	CC22-MRSA-IV, Barnim/UK-EMRSA-15	MED-WARD	76	m
09V05937	CC22-MRSA-IV, Barnim/UK-EMRSA-15	MED-WARD	68	m
09V05943	CC22-MRSA-IV, Barnim/UK-EMRSA-15	MED-WARD	82	m
09V05947	CC22-MRSA-IV, Barnim/UK-EMRSA-15	MED-WARD	63	m
09V06275	CC22-MRSA-IV, Barnim/UK-EMRSA-15	MED-WARD	#WERT!	f
09V08495	CC22-MRSA-IV, Barnim/UK-EMRSA-15	MED-OPD	68	f
09V12464	CC22-MRSA-IV, Barnim/UK-EMRSA-15	MED-WARD	76	m
09V15150	CC22-MRSA-IV, Barnim/UK-EMRSA-15	MED-WARD	47	f
09V16014	CC22-MRSA-IV, Barnim/UK-EMRSA-15	MED-OPD	60	f
09V19884	CC22-MRSA-IV, Barnim/UK-EMRSA-15	MED-OPD	76	m
09V24370	CC22-MRSA-IV, Barnim/UK-EMRSA-15	SUR-ICU	37	m
09V25431	CC22-MRSA-IV, Barnim/UK-EMRSA-15	MED-WARD	82	m
09V27150	CC22-MRSA-IV, Barnim/UK-EMRSA-15	SUR-ICU	71	f
09V27748	CC22-MRSA-IV, Barnim/UK-EMRSA-15	SUR-ICU	19	m
09V28011	CC22-MRSA-IV, Barnim/UK-EMRSA-15	MED-ICU	57	m
09V28179	CC22-MRSA-IV, Barnim/UK-EMRSA-15	SUR-WARD	87	m
09V28558	CC22-MRSA-IV, Barnim/UK-EMRSA-15	SUR-WARD	71	m
09V28681	CC22-MRSA-IV, Barnim/UK-EMRSA-15	SUR-ICU	79	m
09V29022	CC22-MRSA-IV, Barnim/UK-EMRSA-15	MED-ICU	70	m
09V30408	CC22-MRSA-IV, Barnim/UK-EMRSA-15	SUR-WARD	68	m
09V30634	CC22-MRSA-IV, Barnim/UK-EMRSA-15	MED-OPD	74	m
09V31667	CC22-MRSA-IV, Barnim/UK-EMRSA-15	MED-OPD	66	m
09V31882	CC22-MRSA-IV, Barnim/UK-EMRSA-15	MED-OPD	44	m
09V32211	CC22-MRSA-IV, Barnim/UK-EMRSA-15	MED-OPD	66	m
09V34899	CC22-MRSA-IV, Barnim/UK-EMRSA-15	SUR-ICU	53	m
09V35055	CC22-MRSA-IV, Barnim/UK-EMRSA-15	SUR-WARD	82	f
09V35720	CC22-MRSA-IV, Barnim/UK-EMRSA-15	MED-OPD	53	m
09V36683	CC22-MRSA-IV, Barnim/UK-EMRSA-15	MED-ICU	75	m
09V37636	CC22-MRSA-IV, Barnim/UK-EMRSA-15	MED-WARD	75	m
09V37767	CC22-MRSA-IV, Barnim/UK-EMRSA-15	MED-WARD	91	f
09V39554	CC22-MRSA-IV, Barnim/UK-EMRSA-15	MED-ICU	90	f
09V40977	CC22-MRSA-IV, Barnim/UK-EMRSA-15	MED-OPD	69	m
09V42954	CC22-MRSA-IV, Barnim/UK-EMRSA-15	SUR-WARD	99	f
09V44134	CC22-MRSA-IV, Barnim/UK-EMRSA-15	SUR-WARD	70	m
09V46184	CC22-MRSA-IV, Barnim/UK-EMRSA-15	MED-OPD	66	m
09V47038	CC22-MRSA-IV, Barnim/UK-EMRSA-15	SUR-ICU	52	m
09V47487	CC22-MRSA-IV, Barnim/UK-EMRSA-15	SUR-OPD	75	m
09V48862	CC22-MRSA-IV, Barnim/UK-EMRSA-15	MED-OPD	49	m
09V32581	CC22-MRSA-V	MED-OPD	43	m
09V33822	CC45-MRSA-IV, Berlin EMRSA	SUR-ICU	82	f
09V34133	CC45-MRSA-IV, Berlin EMRSA	MED-WARD	48	m
09V45520	CC45-MRSA-IV, Berlin EMRSA	MED-WARD	47	m
09V03558	CC398-MRSA-V	SUR-WARD	67	f
09V07762	CC398-MRSA-V	MED-WARD	82	m
09V29815	CC398-MRSA-V	MED-WARD	66	m
**2010**				
10V03510	CC1/ST772-MRSA-V [PVL+], Bengal Bay Clone/WA MRSA-60	SUR-WARD	70	f
10V31557	CC1-MRSA-IV, WA MRSA-1/57	MED-WARD	34	f
10V31910	CC1-MRSA-IV, WA MRSA-1/57	MED-ICU	57	f
10V01782	CC5/ST228-MRSA-I, South German EMRSA	SUR-ICU	26	m
10V02343	CC5/ST228-MRSA-I, South German EMRSA	SUR-ICU	58	m
10B14561	CC5-MRSA-II, Rhine-Hesse EMRSA/UK-EMRSA-3	MED-ICU	59	f
10F01042	CC5-MRSA-II, Rhine-Hesse EMRSA/UK-EMRSA-3	MED-WARD	72	m
10U14746	CC5-MRSA-II, Rhine-Hesse EMRSA/UK-EMRSA-3	MED-WARD	67	f
10V01913	CC5-MRSA-II, Rhine-Hesse EMRSA/UK-EMRSA-3	SUR-WARD	46	f
10V03216	CC5-MRSA-II, Rhine-Hesse EMRSA/UK-EMRSA-3	SUR-WARD	52	m
10V03727	CC5-MRSA-II, Rhine-Hesse EMRSA/UK-EMRSA-3	SUR-WARD	75	m
10V03824	CC5-MRSA-II, Rhine-Hesse EMRSA/UK-EMRSA-3	SUR-WARD	79	m
10V08796	CC5-MRSA-II, Rhine-Hesse EMRSA/UK-EMRSA-3	SUR-WARD	24	m
10V09487	CC5-MRSA-II, Rhine-Hesse EMRSA/UK-EMRSA-3	MED-ICU	76	m
10V10616	CC5-MRSA-II, Rhine-Hesse EMRSA/UK-EMRSA-3	SUR-WARD	66	m
10V10617	CC5-MRSA-II, Rhine-Hesse EMRSA/UK-EMRSA-3	SUR-WARD	94	m
10V12678	CC5-MRSA-II, Rhine-Hesse EMRSA/UK-EMRSA-3	SUR-ICU	69	m
10V13218	CC5-MRSA-II, Rhine-Hesse EMRSA/UK-EMRSA-3	SUR-WARD	56	m
10V13638	CC5-MRSA-II, Rhine-Hesse EMRSA/UK-EMRSA-3	SUR-WARD	77	m
10V14799	CC5-MRSA-II, Rhine-Hesse EMRSA/UK-EMRSA-3	SUR-WARD	59	m
10V23825	CC5-MRSA-II, Rhine-Hesse EMRSA/UK-EMRSA-3	SUR-WARD	51	m
10V31630	CC5-MRSA-II, Rhine-Hesse EMRSA/UK-EMRSA-3	SUR-WARD	52	f
10V35386	CC5-MRSA-II, Rhine-Hesse EMRSA/UK-EMRSA-3	SUR-WARD	83	m
10V37507	CC5-MRSA-II, Rhine-Hesse EMRSA/UK-EMRSA-3	MED-WARD	69	f
10V38321	CC5-MRSA-II, Rhine-Hesse EMRSA/UK-EMRSA-3	SUR-ICU	51	m
10V40861	CC5-MRSA-II, Rhine-Hesse EMRSA/UK-EMRSA-3	MED-WARD	78	f
10V41383	CC5-MRSA-II, Rhine-Hesse EMRSA/UK-EMRSA-3	MED-ICU	56	f
10V45069	CC5-MRSA-II, Rhine-Hesse EMRSA/UK-EMRSA-3	SUR-ICU	75	f
10V01508	CC80-MRSA-IV [PVL+], European caMRSA Clone	SUR-OPD	26	m
10V34757	CC80-MRSA-IV [PVL+], European caMRSA Clone	SUR-WARD	28	m
10B06681	CC22-MRSA-IV, Barnim/UK-EMRSA-15	MED-WARD	85	f
10F00384	CC22-MRSA-IV, Barnim/UK-EMRSA-15	SUR-WARD	61	m
10F00978	CC22-MRSA-IV, Barnim/UK-EMRSA-15	MED-WARD	76	f
10F02923	CC22-MRSA-IV, Barnim/UK-EMRSA-15	MED-WARD	57	m
10-Hygiene	CC22-MRSA-IV, Barnim/UK-EMRSA-15	MED-WARD		
10U02156	CC22-MRSA-IV, Barnim/UK-EMRSA-15	MED-OPD	54	m
10U16289	CC22-MRSA-IV, Barnim/UK-EMRSA-15	SUR-WARD	52	f
10V01346	CC22-MRSA-IV, Barnim/UK-EMRSA-15	SUR-WARD	73	m
10V03365	CC22-MRSA-IV, Barnim/UK-EMRSA-15	SUR-WARD	57	m
10V03540	CC22-MRSA-IV, Barnim/UK-EMRSA-15	SUR-OPD	62	m
10V03819	CC22-MRSA-IV, Barnim/UK-EMRSA-15	SUR-WARD	81	m
10V04495	CC22-MRSA-IV, Barnim/UK-EMRSA-15	SUR-ICU	46	f
10V04498	CC22-MRSA-IV, Barnim/UK-EMRSA-15	MED-ICU	79	m
10V04625	CC22-MRSA-IV, Barnim/UK-EMRSA-15	SUR-WARD	61	m
10V04685	CC22-MRSA-IV, Barnim/UK-EMRSA-15	SUR-ICU	76	m
10V05305	CC22-MRSA-IV, Barnim/UK-EMRSA-15	SUR-ICU	83	m
10V05474	CC22-MRSA-IV, Barnim/UK-EMRSA-15	SUR-WARD	50	m
10V05553	CC22-MRSA-IV, Barnim/UK-EMRSA-15	SUR-ICU	59	m
10V06864	CC22-MRSA-IV, Barnim/UK-EMRSA-15	SUR-WARD	65	m
10V09545	CC22-MRSA-IV, Barnim/UK-EMRSA-15	SUR-ICU	62	m
10V11922	CC22-MRSA-IV, Barnim/UK-EMRSA-15	SUR-ICU	78	m
10V12801	CC22-MRSA-IV, Barnim/UK-EMRSA-15	SUR-ICU	49	m
10V15165	CC22-MRSA-IV, Barnim/UK-EMRSA-15	SUR-WARD	56	m
10V18022	CC22-MRSA-IV, Barnim/UK-EMRSA-15	MED-WARD	62	m
10V21655	CC22-MRSA-IV, Barnim/UK-EMRSA-15	MED-WARD	59	m
10V21798	CC22-MRSA-IV, Barnim/UK-EMRSA-15	SUR-WARD	71	m
10V23795	CC22-MRSA-IV, Barnim/UK-EMRSA-15	MED-WARD	72	f
10V25861	CC22-MRSA-IV, Barnim/UK-EMRSA-15	MED-OPD	71	m
10V26165	CC22-MRSA-IV, Barnim/UK-EMRSA-15	MED-OPD	59	m
10V26326	CC22-MRSA-IV, Barnim/UK-EMRSA-15	MED-WARD	41	f
10V29967	CC22-MRSA-IV, Barnim/UK-EMRSA-15	SUR-WARD	19	m
10V30523	CC22-MRSA-IV, Barnim/UK-EMRSA-15	MED-WARD	70	m
10V31025	CC22-MRSA-IV, Barnim/UK-EMRSA-15	SUR-ICU	76	m
10V31056	CC22-MRSA-IV, Barnim/UK-EMRSA-15	MED-WARD	71	m
10V32564	CC22-MRSA-IV, Barnim/UK-EMRSA-15	MED-OPD	91	m
10V32855	CC22-MRSA-IV, Barnim/UK-EMRSA-15	MED-WARD	66	m
10V33616	CC22-MRSA-IV, Barnim/UK-EMRSA-15	MED-OPD	72	
10V34392	CC22-MRSA-IV, Barnim/UK-EMRSA-15	MED-ICU	52	m
10V35980	CC22-MRSA-IV, Barnim/UK-EMRSA-15	MED-WARD	66	m
10V37062	CC22-MRSA-IV, Barnim/UK-EMRSA-15	SUR-WARD	84	f
10V37408	CC22-MRSA-IV, Barnim/UK-EMRSA-15	SUR-WARD	83	f
10V37820	CC22-MRSA-IV, Barnim/UK-EMRSA-15	MED-OPD	67	m
10V38669	CC22-MRSA-IV, Barnim/UK-EMRSA-15	SUR-ICU	57	m
10V38699	CC22-MRSA-IV, Barnim/UK-EMRSA-15	MED-OPD	76	m
10V39014	CC22-MRSA-IV, Barnim/UK-EMRSA-15	MED-OPD	66	m
10V39280	CC22-MRSA-IV, Barnim/UK-EMRSA-15	MED-ICU	76	f
10V39426	CC22-MRSA-IV, Barnim/UK-EMRSA-15	MED-WARD	70	m
10V41061	CC22-MRSA-IV, Barnim/UK-EMRSA-15	MED-OPD	69	m
10V44488	CC22-MRSA-IV, Barnim/UK-EMRSA-15	SUR-WARD	58	m
10V44924	CC22-MRSA-IV, Barnim/UK-EMRSA-15	MED-WARD	58	m
10V44966	CC22-MRSA-IV, Barnim/UK-EMRSA-15	MED-OPD	70	m
10V27260	CC22-MRSA-V	MED-OPD	44	f
10V15136	CC45-MRSA-IV, Berlin EMRSA	MED-OPD	68	m
10V29067	CC45-MRSA-IV, Berlin EMRSA	SUR-WARD	68	m
10V40116	CC45-MRSA-IV, Berlin EMRSA	MED-WARD	70	f
10V10412	CC80-MRSA-IV [PVL+], European caMRSA Clone	SUR-Ward	11	m
10V25105	CC398-MRSA-V	MED-WARD	53	m

ISOLATE:	SPECIES MARKER							REGULATORY GENES							
	Ribosomal sequence from S. aureus	glyceraldehyde 3-phosphate dehydro-genase, locus 1	katalase A	coagulase	thermo-stable extracellular nuclease	Protein A	IgG-binding protein	staphylo-coccal accessory regulator A	histidine protein kinase, sae locus	sensor protein	accessory gene regulator allele I	accessory gene regulator allele II	accessory gene regulator allele III	accessory gene regulator allele IV	haemolysin delta
	rrnD1 (S. aureus)	gapA	katA	coA	nuc1	spa	sbi	sarA	saeS	vraS	agrI	agrII	agrIII	agrIV	hld
09V32405	POS	POS	POS	POS	POS	POS	POS	POS	POS	POS	NEG	POS	NEG	NEG	POS
09V47486	POS	POS	POS	POS	POS	POS	POS	POS	POS	POS	NEG	NEG	POS	NEG	POS
09V42357	POS	POS	POS	POS	POS	POS	POS	POS	POS	POS	NEG	POS	NEG	NEG	POS
09F01028	POS	POS	POS	POS	POS	POS	POS	POS	POS	POS	NEG	POS	NEG	NEG	POS
09F01040	POS	POS	POS	POS	POS	POS	POS	POS	POS	POS	NEG	POS	NEG	NEG	POS
09F03018	POS	POS	POS	POS	POS	POS	POS	POS	POS	POS	NEG	POS	NEG	NEG	POS
09F03619	POS	POS	POS	POS	POS	POS	POS	POS	POS	POS	NEG	POS	NEG	NEG	POS
09V01921	POS	POS	POS	POS	POS	POS	POS	POS	POS	POS	NEG	POS	NEG	NEG	POS
09V04547	POS	POS	POS	POS	POS	POS	POS	POS	POS	POS	NEG	POS	NEG	NEG	POS

ISOLATE:	METHICILLIN RESISTANCE AND SCCmec TYPING																									
	alternate penicillin binding protein 2, defining MRSA	truncated signal transducer protein MecR1	glycerophosphoryl diester phosphodi-esterase, associated with mecA	cassette chromosome recombinase genes A/B-1		plasmin-sensitive surface protein	hypothetical protein from SCCmec elements	cassette chromosome recombinase genes A/B-2		potassium-trans-locating ATPase A, chain 2	potassium-transporting ATPase B, chain 1	potassium-trans-locating ATPase C, chain 2	sensor kinase protein	KDP operon transcrip-tional regulatory protein	methicillin-resistance regulatory protein	signal transducer protein MecR1	homolog of xylose repressor, associated with SCCmec-elements	cassette chromosome recombinase genes A/B-3		mercury resistance operon		cassette chromosome recombinase genes "ccrAA" (hypothetical) and ccrC			cassette chromosome recombinase genes A/B-4	
	mecA	delta_mecR	ugpQ	ccrA-1	ccrB-1	plsSCC (COL)	Q9XB68-dcs	ccrA-2	ccrB-2	kdpA-SCC	kdpB-SCC	kdpC-SCC	kdpD-SCC	kdpE-SCC	mecI	mecR	xylR	ccrA-3	ccrB-3	merA	merB	ccrAA (MRSAZH47) _probe 1	ccrAA (MRSAZH47) _probe 2	ccrC (85-2082)	ccrA-4	ccrB-4
09V32405	POS	NEG	POS	NEG	NEG	NEG	NEG	NEG	NEG	NEG	NEG	NEG	NEG	NEG	NEG	NEG	NEG	NEG	NEG	NEG	NEG	POS	POS	POS	NEG	NEG
09V47486	POS	POS	POS	NEG	NEG	NEG	NEG	POS	POS	NEG	NEG	NEG	NEG	NEG	NEG	NEG	NEG	NEG	NEG	NEG	NEG	NEG	NEG	NEG	NEG	NEG
09V42357	POS	POS	POS	POS	POS	POS	POS	NEG	NEG	NEG	NEG	NEG	NEG	NEG	NEG	NEG	NEG	NEG	NEG	NEG	NEG	NEG	NEG	NEG	NEG	NEG
09F01028	POS	POS	POS	NEG	NEG	NEG	POS	POS	POS	POS	POS	POS	POS	POS	POS	POS	POS	NEG	NEG	NEG	NEG	NEG	NEG	NEG	NEG	NEG
09F01040	POS	POS	POS	NEG	NEG	NEG	POS	POS	POS	POS	POS	POS	POS	POS	POS	POS	POS	NEG	NEG	NEG	NEG	NEG	NEG	NEG	NEG	NEG
09F03018	POS	POS	POS	NEG	NEG	NEG	POS	POS	POS	POS	POS	POS	POS	POS	POS	POS	POS	NEG	NEG	NEG	NEG	NEG	NEG	NEG	NEG	NEG
09F03619	POS	POS	POS	NEG	NEG	NEG	NEG	POS	POS	POS	POS	POS	POS	POS	POS	POS	POS	NEG	NEG	NEG	NEG	NEG	NEG	NEG	NEG	NEG
09V01921	POS	POS	POS	NEG	NEG	NEG	NEG	POS	POS	POS	POS	POS	POS	POS	POS	POS	POS	NEG	NEG	NEG	NEG	NEG	NEG	NEG	NEG	NEG
09V04547	POS	POS	POS	NEG	NEG	NEG	POS	POS	POS	POS	POS	POS	POS	POS	POS	POS	POS	NEG	NEG	NEG	NEG	NEG	NEG	NEG	NEG	NEG

ISOLATE:	STRAIN	RESISTANCE : PENICILLINASE			RESISTANCE : MLS-ANTIBIOTICS												RESISTANCE : AMINOGLYSOSIDES			RESISTANCE : MISCELLANEOUS GENES																RESISTANCE : EFFLUX SYSTEMS								RESISTANCE : GLYCOPEPTIDES		
		beta-lactamase	beta lactamase repressor (inhibitor)	beta-lactamase regulatory protein	rRNA adenine N-6-methyl-transferase, erythromycin/clindamycin resistance	erythro-mycin/clindamycin resistance	erythro-mycin/clindamycin resistance	Linco-samid-Nucleo-tidyltrans-ferase	energy-dependent efflux of erythro-mycin	macrolide efflux protein A	probable lysylphos-phatidyl-glycerol synthetase	virginia-mycin A acetyltransferase	acetyl-transferase inactivating streptogramin A	ATP binding protein, streptogramin-A-resistance		virginia-mycin B hydrolase	bifunctional enzyme Aac/Aph, gentamicin resistance	amino-glycoside adenyl-transferase,tobramycin resistance	3'5'-aminog-lycoside phospho-transferase, neo-/ kanamycin resistance	strepto-thricine-acetyl-transferase	dihydro-folate reductase type 1	fusidic acid resistence	hypothetical protein associated with fusidic acid resistance	mupirocin resistence protein	tetrazyklin-resistance	tetracycline-resistance	chloramphenicol acetyltransferase					23S rRNA methyltransferase	chloramphenicol /florfenicol exporter	metallothiol transferase		quaternary ammonium compound resistance protein A	quaternary ammonium compound resistance protein C						Transport/ Efflux protein	vancomycin resistance gene	vancomycin resistance gene from enterococci and Clostridium	teicoplanin resistance gene from enterococci
		blaZ	blaI	blaR	erm(A)	erm(B)	erm(C)	lnu(A)	msr(A)	mef(A)	mph(C)	vat(A)	vat(B)	vga(A)	vga(A) (BM 3327)	vgB(A)	aacA-aphD	aadD	aphA3	sat	dfrA	far1	Q6GD50	mupR	tet(K)	tet(M)	cat (total)	cat (pC221)	cat (pc223)	cat (pMC524)	cat (pSBK203R)	cfr	fexA	fosB	fosB (plasmid)	qacA	qacC (total)	qacC (consensus)	qacC (equine)	qacC (SA5)	qacC (Ssap)	qacC (ST94)	tetEfflux	vanA	vanB	vanZ
**2009**																																														
09V32405	CC1/ST772-MRSA-V [PVL+], Bengal Bay Clone/WA MRSA-60	POS	POS	POS	NEG	NEG	POS	NEG	POS	NEG	POS	NEG	NEG	NEG	NEG	NEG	POS	NEG	POS	POS	NEG	NEG	NEG	NEG	NEG	NEG	NEG	NEG	NEG	NEG	NEG	NEG	NEG	POS	NEG	NEG	NEG	NEG	NEG	NEG	NEG	NEG	POS	NEG	NEG	NEG
09V47486	CC1-MRSA-IV, WA MRSA-1/57	POS	POS	POS	AMB	NEG	POS	NEG	NEG	NEG	NEG	NEG	NEG	NEG	NEG	NEG	NEG	NEG	POS	POS	POS	NEG	NEG	NEG	POS	NEG	NEG	NEG	NEG	NEG	NEG	NEG	NEG	NEG	NEG	NEG	NEG	NEG	NEG	NEG	NEG	NEG	POS	NEG	NEG	NEG
09V42357	CC5/ST228-MRSA-I, South German EMRSA	NEG	NEG	NEG	POS	NEG	NEG	NEG	NEG	NEG	NEG	NEG	NEG	NEG	NEG	NEG	POS	NEG	POS	POS	NEG	NEG	NEG	NEG	NEG	NEG	NEG	NEG	NEG	NEG	NEG	NEG	NEG	POS	NEG	NEG	NEG	NEG	NEG	NEG	NEG	NEG	POS	NEG	NEG	NEG
09F01028	CC5-MRSA-II, Rhine-Hesse EMRSA/UK-EMRSA-3	POS	POS	POS	POS	NEG	NEG	NEG	NEG	NEG	NEG	NEG	NEG	NEG	NEG	NEG	NEG	NEG	NEG	NEG	NEG	NEG	NEG	NEG	NEG	NEG	NEG	NEG	NEG	NEG	NEG	NEG	NEG	POS	NEG	NEG	NEG	NEG	NEG	NEG	NEG	NEG	POS	NEG	NEG	NEG
09F01040	CC5-MRSA-II, Rhine-Hesse EMRSA/UK-EMRSA-3	POS	POS	POS	POS	NEG	NEG	NEG	NEG	NEG	NEG	NEG	NEG	NEG	NEG	NEG	NEG	NEG	NEG	NEG	NEG	NEG	NEG	NEG	NEG	NEG	NEG	NEG	NEG	NEG	NEG	NEG	NEG	POS	NEG	NEG	NEG	NEG	NEG	NEG	NEG	NEG	POS	NEG	NEG	NEG
09F03018	CC5-MRSA-II, Rhine-Hesse EMRSA/UK-EMRSA-3	POS	POS	POS	POS	NEG	NEG	NEG	NEG	NEG	NEG	NEG	NEG	NEG	NEG	NEG	NEG	NEG	NEG	NEG	NEG	NEG	NEG	NEG	NEG	NEG	NEG	NEG	NEG	NEG	NEG	NEG	NEG	POS	NEG	NEG	NEG	NEG	NEG	NEG	NEG	NEG	POS	NEG	NEG	NEG
09F03619	CC5-MRSA-II, Rhine-Hesse EMRSA/UK-EMRSA-3	POS	POS	POS	POS	NEG	NEG	NEG	NEG	NEG	NEG	NEG	NEG	NEG	NEG	NEG	NEG	AMB	NEG	NEG	NEG	NEG	NEG	NEG	NEG	NEG	NEG	NEG	NEG	NEG	NEG	NEG	NEG	POS	NEG	NEG	NEG	NEG	NEG	NEG	NEG	NEG	POS	NEG	NEG	NEG
09V01921	CC5-MRSA-II, Rhine-Hesse EMRSA/UK-EMRSA-3	POS	POS	POS	POS	NEG	NEG	NEG	NEG	NEG	NEG	NEG	NEG	NEG	NEG	NEG	NEG	NEG	NEG	NEG	NEG	NEG	NEG	NEG	NEG	NEG	NEG	NEG	NEG	NEG	NEG	NEG	NEG	POS	NEG	NEG	NEG	NEG	NEG	NEG	NEG	NEG	POS	NEG	NEG	NEG
09V04547	CC5-MRSA-II, Rhine-Hesse EMRSA/UK-EMRSA-3	POS	POS	POS	POS	NEG	NEG	NEG	NEG	NEG	NEG	NEG	NEG	NEG	NEG	NEG	NEG	POS	NEG	NEG	NEG	NEG	NEG	NEG	NEG	NEG	NEG	NEG	NEG	NEG	NEG	NEG	NEG	POS	NEG	NEG	NEG	NEG	NEG	NEG	NEG	NEG	POS	NEG	NEG	NEG
09V05744	CC5-MRSA-II, Rhine-Hesse EMRSA/UK-EMRSA-3	POS	POS	POS	POS	NEG	NEG	NEG	NEG	NEG	NEG	NEG	NEG	NEG	NEG	NEG	NEG	POS	NEG	NEG	NEG	NEG	NEG	NEG	NEG	NEG	NEG	NEG	NEG	NEG	NEG	NEG	NEG	POS	NEG	NEG	NEG	NEG	NEG	NEG	NEG	NEG	POS	NEG	NEG	NEG
09V07448	CC5-MRSA-II, Rhine-Hesse EMRSA/UK-EMRSA-3	POS	POS	POS	POS	NEG	NEG	NEG	NEG	NEG	NEG	NEG	NEG	NEG	NEG	NEG	NEG	POS	NEG	NEG	NEG	NEG	NEG	NEG	NEG	NEG	NEG	NEG	NEG	NEG	NEG	NEG	NEG	POS	NEG	NEG	NEG	NEG	NEG	NEG	NEG	NEG	POS	NEG	NEG	NEG
09V08032	CC5-MRSA-II, Rhine-Hesse EMRSA/UK-EMRSA-3	POS	POS	POS	POS	NEG	NEG	NEG	NEG	NEG	NEG	NEG	NEG	NEG	NEG	NEG	NEG	NEG	NEG	NEG	NEG	NEG	NEG	NEG	NEG	NEG	NEG	NEG	NEG	NEG	NEG	NEG	NEG	POS	NEG	NEG	NEG	NEG	NEG	NEG	NEG	NEG	POS	NEG	NEG	NEG
09V08761	CC5-MRSA-II, Rhine-Hesse EMRSA/UK-EMRSA-3	POS	POS	POS	POS	NEG	NEG	NEG	NEG	NEG	NEG	NEG	NEG	NEG	NEG	NEG	NEG	NEG	NEG	NEG	NEG	NEG	NEG	NEG	NEG	NEG	NEG	NEG	NEG	NEG	NEG	NEG	NEG	POS	NEG	NEG	NEG	NEG	NEG	NEG	NEG	NEG	POS	NEG	NEG	NEG
09V21706	CC5-MRSA-II, Rhine-Hesse EMRSA/UK-EMRSA-3	POS			POS		NEG	NEG	NEG			NEG	NEG	NEG	NEG	NEG	NEG	NEG	NEG	NEG	NEG	NEG		NEG	NEG	NEG																		NEG	NEG	NEG
09V22079	CC5-MRSA-II, Rhine-Hesse EMRSA/UK-EMRSA-3	POS	POS	POS	POS	NEG	NEG	NEG	NEG	NEG	NEG	NEG	NEG	NEG	NEG	NEG	NEG	POS	NEG	NEG	NEG	NEG	NEG	NEG	NEG	NEG	NEG	NEG	NEG	NEG	NEG	NEG	NEG	POS	NEG	NEG	NEG	NEG	NEG	NEG	NEG	NEG	POS	NEG	NEG	NEG
09V25980	CC5-MRSA-II, Rhine-Hesse EMRSA/UK-EMRSA-3	POS	POS	POS	POS	NEG	NEG	NEG	NEG	NEG	NEG	NEG	NEG	NEG	NEG	NEG	NEG	POS	NEG	NEG	NEG	NEG	NEG	NEG	NEG	NEG	NEG	NEG	NEG	NEG	NEG	NEG	NEG	POS	NEG	NEG	NEG	NEG	NEG	NEG	NEG	NEG	POS	NEG	NEG	NEG
09V26221	CC5-MRSA-II, Rhine-Hesse EMRSA/UK-EMRSA-3	POS	POS	POS	POS	NEG	NEG	NEG	NEG	NEG	NEG	NEG	NEG	NEG	NEG	NEG	NEG	POS	NEG	NEG	NEG	NEG	NEG	NEG	NEG	NEG	NEG	NEG	NEG	NEG	NEG	NEG	NEG	POS	NEG	NEG	NEG	NEG	NEG	NEG	NEG	NEG	POS	NEG	NEG	NEG
09V28517	CC5-MRSA-II, Rhine-Hesse EMRSA/UK-EMRSA-3	POS	POS	POS	POS	NEG	NEG	NEG	NEG	NEG	NEG	NEG	NEG	NEG	NEG	NEG	NEG	POS	NEG	NEG	NEG	NEG	NEG	NEG	NEG	NEG	NEG	NEG	NEG	NEG	NEG	NEG	NEG	POS	NEG	NEG	NEG	NEG	NEG	NEG	NEG	NEG	POS	NEG	NEG	NEG
09V28643	CC5-MRSA-II, Rhine-Hesse EMRSA/UK-EMRSA-3	POS	POS	POS	POS	NEG	NEG	NEG	NEG	NEG	NEG	NEG	NEG	NEG	NEG	NEG	NEG	POS	NEG	NEG	NEG	NEG	NEG	NEG	NEG	NEG	NEG	NEG	NEG	NEG	NEG	NEG	NEG	POS	NEG	NEG	NEG	NEG	NEG	NEG	NEG	NEG	POS	NEG	NEG	NEG
09V28647	CC5-MRSA-II, Rhine-Hesse EMRSA/UK-EMRSA-3	POS	POS	POS	POS	NEG	NEG	NEG	NEG	NEG	NEG	NEG	NEG	NEG	NEG	NEG	NEG	POS	NEG	NEG	NEG	NEG	NEG	NEG	NEG	NEG	NEG	NEG	NEG	NEG	NEG	NEG	NEG	POS	NEG	NEG	NEG	NEG	NEG	NEG	NEG	NEG	POS	NEG	NEG	NEG
09V29064	CC5-MRSA-II, Rhine-Hesse EMRSA/UK-EMRSA-3	NEG	NEG	NEG	POS	NEG	NEG	NEG	NEG	NEG	NEG	NEG	NEG	NEG	NEG	NEG	NEG	POS	NEG	NEG	NEG	NEG	NEG	NEG	NEG	NEG	NEG	NEG	NEG	NEG	NEG	NEG	NEG	POS	NEG	NEG	NEG	NEG	NEG	NEG	NEG	NEG	POS	NEG	NEG	NEG
09V30464	CC5-MRSA-II, Rhine-Hesse EMRSA/UK-EMRSA-3	POS	POS	POS	POS	NEG	NEG	NEG	NEG	NEG	NEG	NEG	NEG	NEG	NEG	NEG	NEG	POS	NEG	NEG	NEG	NEG	NEG	NEG	NEG	NEG	NEG	NEG	NEG	NEG	NEG	NEG	NEG	POS	NEG	NEG	NEG	NEG	NEG	NEG	NEG	NEG	POS	NEG	NEG	NEG
09V30701	CC5-MRSA-II, Rhine-Hesse EMRSA/UK-EMRSA-3	POS	POS	POS	POS	NEG	NEG	NEG	NEG	NEG	NEG	NEG	NEG	NEG	NEG	NEG	NEG	POS	NEG	NEG	NEG	NEG	NEG	NEG	NEG	NEG	NEG	NEG	NEG	NEG	NEG	NEG	NEG	POS	NEG	NEG	NEG	NEG	NEG	NEG	NEG	NEG	POS	NEG	NEG	NEG
09V35418	CC5-MRSA-II, Rhine-Hesse EMRSA/UK-EMRSA-3	POS	POS	POS	POS	NEG	NEG	NEG	NEG	NEG	NEG	NEG	NEG	NEG	NEG	NEG	NEG	POS	NEG	NEG	NEG	NEG	NEG	NEG	NEG	NEG	NEG	NEG	NEG	NEG	NEG	NEG	NEG	POS	NEG	NEG	NEG	NEG	NEG	NEG	NEG	NEG	POS	NEG	NEG	NEG
09V37115	CC5-MRSA-II, Rhine-Hesse EMRSA/UK-EMRSA-3	POS	POS	POS	POS	NEG	NEG	NEG	NEG	NEG	NEG	NEG	NEG	NEG	NEG	NEG	NEG	POS	NEG	NEG	NEG	NEG	NEG	NEG	NEG	NEG	NEG	NEG	NEG	NEG	NEG	NEG	NEG	POS	NEG	NEG	NEG	NEG	NEG	NEG	NEG	NEG	POS	NEG	NEG	NEG
09V37466	CC5-MRSA-II, Rhine-Hesse EMRSA/UK-EMRSA-3	POS	POS	POS	POS	NEG	NEG	NEG	NEG	NEG	NEG	NEG	NEG	NEG	NEG	NEG	NEG	POS	NEG	NEG	NEG	NEG	NEG	NEG	NEG	NEG	NEG	NEG	NEG	NEG	NEG	NEG	NEG	POS	NEG	NEG	NEG	NEG	NEG	NEG	NEG	NEG	POS	NEG	NEG	NEG
09V40100	CC5-MRSA-II, Rhine-Hesse EMRSA/UK-EMRSA-3	POS	POS	POS	POS	NEG	NEG	NEG	NEG	NEG	NEG	NEG	NEG	NEG	NEG	NEG	NEG	NEG	NEG	NEG	NEG	NEG	NEG	NEG	NEG	NEG	NEG	NEG	NEG	NEG	NEG	NEG	NEG	POS	NEG	NEG	NEG	NEG	NEG	NEG	NEG	NEG	POS	NEG	NEG	NEG
09V41542	CC5-MRSA-II, Rhine-Hesse EMRSA/UK-EMRSA-3	POS	POS	POS	POS	NEG	NEG	NEG	NEG	NEG	NEG	NEG	NEG	NEG	NEG	NEG	NEG	NEG	NEG	NEG	NEG	NEG	NEG	NEG	NEG	NEG	NEG	NEG	NEG	NEG	NEG	NEG	NEG	POS	NEG	NEG	NEG	NEG	NEG	NEG	NEG	NEG	POS	NEG	NEG	NEG
09V42595	CC5-MRSA-II, Rhine-Hesse EMRSA/UK-EMRSA-3	POS	POS	POS	POS	NEG	NEG	NEG	NEG	NEG	NEG	NEG	NEG	NEG	NEG	NEG	NEG	POS	AMB	NEG	NEG	NEG	NEG	NEG	NEG	NEG	NEG	NEG	NEG	NEG	NEG	NEG	NEG	POS	NEG	NEG	NEG	NEG	NEG	NEG	NEG	NEG	POS	NEG	NEG	NEG
09V46894	CC5-MRSA-II, Rhine-Hesse EMRSA/UK-EMRSA-3	POS	POS	POS	POS	NEG	NEG	NEG	NEG	NEG	NEG	NEG	NEG	NEG	NEG	NEG	NEG	POS	NEG	NEG	NEG	NEG	NEG	NEG	NEG	NEG	NEG	NEG	NEG	NEG	NEG	NEG	NEG	POS	NEG	NEG	POS	POS	NEG	NEG	NEG	NEG	POS	NEG	NEG	NEG
09V47383	CC5-MRSA-II, Rhine-Hesse EMRSA/UK-EMRSA-3	POS	POS	POS	POS	NEG	NEG	NEG	NEG	NEG	NEG	NEG	NEG	NEG	NEG	NEG	NEG	POS	NEG	NEG	NEG	NEG	NEG	NEG	NEG	NEG	NEG	NEG	NEG	NEG	NEG	NEG	NEG	POS	AMB	NEG	NEG	NEG	NEG	NEG	NEG	NEG	POS	NEG	NEG	NEG
09V47393	CC5-MRSA-II, Rhine-Hesse EMRSA/UK-EMRSA-3	POS	POS	POS	POS	NEG	NEG	NEG	NEG	NEG	NEG	NEG	NEG	NEG	NEG	NEG	NEG	POS	NEG	NEG	NEG	NEG	NEG	NEG	NEG	NEG	NEG	NEG	NEG	NEG	NEG	NEG	NEG	POS	NEG	NEG	NEG	NEG	NEG	NEG	NEG	NEG	POS	NEG	NEG	NEG
09V34574	CC5-MRSA-IV, Paediatric clone	POS	POS	POS	NEG	NEG	NEG	NEG	NEG	NEG	NEG	NEG	NEG	NEG	NEG	NEG	NEG	NEG	NEG	NEG	NEG	NEG	NEG	NEG	NEG	NEG	NEG	NEG	NEG	NEG	NEG	NEG	NEG	POS	NEG	NEG	NEG	NEG	NEG	NEG	NEG	NEG	POS	NEG	NEG	NEG
09F00871	CC8/ST72-MRSA-IV, USA700	POS	POS	POS	NEG	NEG	NEG	NEG	NEG	NEG	NEG	NEG	NEG	NEG	NEG	NEG	NEG	NEG	NEG	NEG	NEG	NEG	NEG	NEG	NEG	NEG	NEG	NEG	NEG	NEG	NEG	NEG	NEG	POS	NEG	NEG	NEG	NEG	NEG	NEG	NEG	NEG	POS	NEG	NEG	NEG
09V20594	CC8-MRSA-IV, USA300	POS	POS	POS	NEG	NEG	NEG	NEG	POS	NEG	POS	NEG	NEG	NEG	NEG	NEG	NEG	NEG	POS	POS	NEG	NEG	NEG	NEG	NEG	NEG	NEG	NEG	NEG	NEG	NEG	NEG	NEG	POS	NEG	NEG	NEG	NEG	NEG	NEG	NEG	NEG	POS	NEG	NEG	NEG
09V28877	CC8-MRSA-IV, USA300	POS	POS	POS	NEG	NEG	NEG	NEG	NEG	NEG	NEG	NEG	NEG	AMB	NEG	NEG	NEG	NEG	POS	POS	NEG	NEG	NEG	NEG	POS	NEG	NEG	NEG	NEG	NEG	NEG	NEG	NEG	POS	AMB	NEG	NEG	NEG	NEG	NEG	NEG	NEG	POS	NEG	NEG	NEG
09F00437	CC22-MRSA-IV, Barnim/UK-EMRSA-15	POS	POS	POS	NEG	NEG	POS	NEG	NEG	NEG	NEG	NEG	NEG	NEG	NEG	NEG	NEG	NEG	NEG	NEG	NEG	NEG	NEG	NEG	NEG	NEG	NEG	NEG	NEG	NEG	NEG	NEG	NEG	NEG	NEG	NEG	NEG	NEG	NEG	NEG	NEG	NEG	NEG	NEG	NEG	NEG
09F01062	CC22-MRSA-IV, Barnim/UK-EMRSA-15	POS	POS	POS	NEG	NEG	NEG	NEG	NEG	NEG	NEG	NEG	NEG	NEG	NEG	NEG	NEG	NEG	NEG	NEG	NEG	NEG	NEG	NEG	NEG	NEG	NEG	NEG	NEG	NEG	NEG	NEG	NEG	NEG	NEG	NEG	NEG	NEG	NEG	NEG	NEG	NEG	NEG	NEG	NEG	NEG
09U06692	CC22-MRSA-IV, Barnim/UK-EMRSA-15	POS			NEG		NEG	NEG	NEG			NEG	NEG	NEG	NEG	NEG	NEG	NEG	NEG	NEG	NEG	NEG		NEG	NEG	NEG																		NEG	NEG	NEG
09V04550	CC22-MRSA-IV, Barnim/UK-EMRSA-15	POS	POS	POS	NEG	NEG	NEG	NEG	NEG	NEG	NEG	NEG	NEG	NEG	NEG	NEG	NEG	NEG	NEG	NEG	NEG	NEG	NEG	NEG	NEG	NEG	NEG	NEG	NEG	NEG	NEG	NEG	NEG	NEG	NEG	NEG	NEG	NEG	NEG	NEG	NEG	NEG	NEG	NEG	NEG	NEG
09V05293	CC22-MRSA-IV, Barnim/UK-EMRSA-15	POS	POS	POS	NEG	NEG	NEG	NEG	NEG	NEG	NEG	NEG	NEG	NEG	NEG	NEG	NEG	NEG	NEG	NEG	NEG	NEG	NEG	NEG	NEG	NEG	NEG	NEG	NEG	NEG	NEG	NEG	NEG	NEG	NEG	NEG	NEG	NEG	NEG	NEG	NEG	NEG	NEG	NEG	NEG	NEG
09V05937	CC22-MRSA-IV, Barnim/UK-EMRSA-15	POS			NEG		NEG	NEG	NEG			NEG	NEG	NEG	NEG	NEG	NEG	NEG	NEG	NEG	NEG	NEG		NEG	NEG	NEG																		NEG	NEG	NEG
09V05943	CC22-MRSA-IV, Barnim/UK-EMRSA-15	POS			NEG		NEG	NEG	NEG			NEG	NEG	AMB	NEG	NEG	NEG	NEG	NEG	NEG	NEG	NEG		NEG	NEG	NEG																		NEG	NEG	NEG
09V05947	CC22-MRSA-IV, Barnim/UK-EMRSA-15	POS			NEG		NEG	NEG	NEG			NEG	NEG	NEG	NEG	NEG	NEG	NEG	NEG	NEG	NEG	NEG		NEG	NEG	NEG																		NEG	NEG	NEG
09V06275	CC22-MRSA-IV, Barnim/UK-EMRSA-15	POS	POS	POS	NEG	NEG	NEG	NEG	NEG	NEG	NEG	NEG	NEG	NEG	NEG	NEG	NEG	NEG	NEG	NEG	NEG	NEG	NEG	NEG	NEG	NEG	NEG	NEG	NEG	NEG	NEG	NEG	NEG	NEG	NEG	NEG	NEG	NEG	NEG	NEG	NEG	NEG	NEG	NEG	NEG	NEG
09V08495	CC22-MRSA-IV, Barnim/UK-EMRSA-15	POS	POS	POS	NEG	NEG	NEG	NEG	NEG	NEG	NEG	NEG	NEG	NEG	NEG	NEG	NEG	NEG	NEG	NEG	NEG	NEG	NEG	NEG	NEG	NEG	NEG	NEG	NEG	NEG	NEG	NEG	NEG	NEG	NEG	NEG	NEG	NEG	NEG	NEG	NEG	NEG	NEG	NEG	NEG	NEG
09V12464	CC22-MRSA-IV, Barnim/UK-EMRSA-15	POS			NEG		NEG	NEG	NEG			NEG	NEG	NEG	NEG	NEG	NEG	NEG	NEG	NEG	NEG	NEG		NEG	NEG	NEG																		NEG	NEG	NEG
09V15150	CC22-MRSA-IV, Barnim/UK-EMRSA-15	POS			NEG		NEG	NEG	NEG			NEG	NEG	NEG	NEG	NEG	NEG	NEG	NEG	NEG	NEG	NEG		NEG	NEG	NEG																		NEG	NEG	NEG
09V16014	CC22-MRSA-IV, Barnim/UK-EMRSA-15	POS	POS	POS	NEG	NEG	NEG	NEG	NEG	NEG	NEG	NEG	NEG	NEG	NEG	NEG	NEG	NEG	NEG	NEG	NEG	NEG	NEG	NEG	NEG	NEG	NEG	NEG	NEG	NEG	NEG	NEG	NEG	NEG	NEG	NEG	NEG	NEG	NEG	NEG	NEG	NEG	NEG	NEG	NEG	NEG
09V19884	CC22-MRSA-IV, Barnim/UK-EMRSA-15	POS			NEG		NEG	NEG	NEG			NEG	NEG	AMB	NEG	NEG	NEG	NEG	NEG	NEG	NEG	NEG		NEG	NEG	NEG																		NEG	NEG	NEG
09V24370	CC22-MRSA-IV, Barnim/UK-EMRSA-15	POS	POS	POS	NEG	NEG	POS	NEG	NEG	NEG	NEG	NEG	NEG	NEG	NEG	NEG	NEG	NEG	NEG	NEG	NEG	NEG	NEG	NEG	NEG	NEG	NEG	NEG	NEG	NEG	NEG	NEG	NEG	NEG	NEG	NEG	NEG	NEG	NEG	NEG	NEG	NEG	NEG	NEG	NEG	NEG
09V25431	CC22-MRSA-IV, Barnim/UK-EMRSA-15	POS	POS	POS	NEG	NEG	POS	NEG	NEG	NEG	NEG	NEG	NEG	NEG	NEG	NEG	NEG	NEG	NEG	NEG	NEG	NEG	NEG	NEG	NEG	NEG	NEG	NEG	NEG	NEG	NEG	NEG	NEG	NEG	NEG	NEG	NEG	NEG	NEG	NEG	NEG	NEG	NEG	NEG	NEG	NEG
09V27150	CC22-MRSA-IV, Barnim/UK-EMRSA-15	POS	POS	POS	NEG	NEG	POS	NEG	NEG	NEG	NEG	NEG	NEG	NEG	NEG	NEG	NEG	NEG	NEG	NEG	NEG	NEG	NEG	NEG	NEG	NEG	NEG	NEG	NEG	NEG	NEG	NEG	NEG	NEG	NEG	NEG	NEG	NEG	NEG	NEG	NEG	NEG	NEG	NEG	NEG	NEG
09V27748	CC22-MRSA-IV, Barnim/UK-EMRSA-15	POS	POS	POS	NEG	NEG	POS	NEG	NEG	NEG	NEG	NEG	NEG	NEG	NEG	NEG	NEG	NEG	NEG	NEG	NEG	NEG	NEG	NEG	NEG	NEG	NEG	NEG	NEG	NEG	NEG	NEG	NEG	NEG	NEG	NEG	NEG	NEG	NEG	NEG	NEG	NEG	NEG	NEG	NEG	NEG
09V28011	CC22-MRSA-IV, Barnim/UK-EMRSA-15	POS	POS	POS	NEG	NEG	POS	NEG	NEG	NEG	NEG	NEG	NEG	NEG	NEG	NEG	NEG	NEG	NEG	NEG	NEG	NEG	NEG	NEG	NEG	NEG	NEG	NEG	NEG	NEG	NEG	NEG	NEG	NEG	NEG	NEG	NEG	NEG	NEG	NEG	NEG	NEG	NEG	NEG	NEG	NEG
09V28179	CC22-MRSA-IV, Barnim/UK-EMRSA-15	POS	POS	POS	NEG	NEG	NEG	NEG	NEG	NEG	NEG	NEG	NEG	NEG	NEG	NEG	NEG	NEG	NEG	NEG	NEG	NEG	NEG	NEG	NEG	NEG	NEG	NEG	NEG	NEG	NEG	NEG	NEG	NEG	NEG	NEG	NEG	NEG	NEG	NEG	NEG	NEG	NEG	NEG	NEG	NEG
09V28558	CC22-MRSA-IV, Barnim/UK-EMRSA-15	POS	POS	POS	NEG	NEG	NEG	NEG	NEG	NEG	NEG	NEG	NEG	NEG	NEG	NEG	NEG	NEG	NEG	NEG	NEG	NEG	NEG	NEG	NEG	NEG	NEG	NEG	NEG	NEG	NEG	NEG	NEG	NEG	NEG	NEG	NEG	NEG	NEG	NEG	NEG	NEG	NEG	NEG	NEG	NEG
09V28681	CC22-MRSA-IV, Barnim/UK-EMRSA-15	POS	POS	POS	NEG	NEG	POS	NEG	NEG	NEG	NEG	NEG	NEG	NEG	NEG	NEG	NEG	NEG	NEG	AMB	NEG	NEG	NEG	NEG	NEG	NEG	NEG	NEG	NEG	NEG	NEG	NEG	NEG	NEG	NEG	NEG	NEG	NEG	NEG	NEG	NEG	NEG	NEG	NEG	NEG	NEG
09V29022	CC22-MRSA-IV, Barnim/UK-EMRSA-15	POS	POS	POS	NEG	NEG	NEG	NEG	NEG	NEG	NEG	NEG	NEG	NEG	NEG	NEG	NEG	NEG	NEG	NEG	NEG	NEG	NEG	NEG	NEG	NEG	NEG	NEG	NEG	NEG	NEG	NEG	NEG	NEG	NEG	NEG	NEG	NEG	NEG	NEG	NEG	NEG	NEG	NEG	NEG	NEG
09V30408	CC22-MRSA-IV, Barnim/UK-EMRSA-15	POS	POS	POS	NEG	NEG	NEG	NEG	NEG	NEG	NEG	NEG	NEG	NEG	NEG	NEG	NEG	NEG	NEG	NEG	NEG	NEG	NEG	NEG	NEG	NEG	NEG	NEG	NEG	NEG	NEG	NEG	NEG	NEG	NEG	NEG	NEG	NEG	NEG	NEG	NEG	NEG	NEG	NEG	NEG	NEG
09V30634	CC22-MRSA-IV, Barnim/UK-EMRSA-15	POS	POS	POS	NEG	NEG	NEG	NEG	NEG	NEG	NEG	NEG	NEG	NEG	NEG	NEG	NEG	NEG	NEG	NEG	NEG	NEG	NEG	NEG	NEG	NEG	NEG	NEG	NEG	NEG	NEG	NEG	NEG	NEG	NEG	NEG	NEG	NEG	NEG	NEG	NEG	NEG	NEG	NEG	NEG	NEG
09V31667	CC22-MRSA-IV, Barnim/UK-EMRSA-15	POS	POS	POS	NEG	NEG	NEG	NEG	NEG	NEG	NEG	NEG	NEG	NEG	NEG	NEG	NEG	NEG	NEG	NEG	NEG	NEG	NEG	NEG	NEG	NEG	NEG	NEG	NEG	NEG	NEG	NEG	NEG	NEG	NEG	NEG	NEG	NEG	NEG	NEG	NEG	NEG	NEG	NEG	NEG	NEG
09V31882	CC22-MRSA-IV, Barnim/UK-EMRSA-15	POS	POS	POS	NEG	NEG	NEG	NEG	NEG	NEG	NEG	NEG	NEG	NEG	NEG	NEG	NEG	NEG	NEG	NEG	NEG	NEG	NEG	NEG	NEG	NEG	NEG	NEG	NEG	NEG	NEG	NEG	NEG	NEG	NEG	NEG	NEG	NEG	NEG	NEG	NEG	NEG	NEG	NEG	NEG	NEG
09V32211	CC22-MRSA-IV, Barnim/UK-EMRSA-15	POS	POS	POS	NEG	NEG	POS	NEG	NEG	NEG	NEG	NEG	NEG	NEG	NEG	NEG	NEG	NEG	NEG	NEG	NEG	NEG	NEG	NEG	NEG	NEG	NEG	NEG	NEG	NEG	NEG	NEG	NEG	NEG	NEG	NEG	NEG	NEG	NEG	NEG	NEG	NEG	NEG	NEG	NEG	NEG
09V34899	CC22-MRSA-IV, Barnim/UK-EMRSA-15	POS	POS	POS	NEG	NEG	NEG	NEG	NEG	NEG	NEG	NEG	NEG	NEG	NEG	NEG	NEG	NEG	NEG	NEG	NEG	NEG	NEG	NEG	NEG	NEG	NEG	NEG	NEG	NEG	NEG	NEG	NEG	NEG	NEG	NEG	NEG	NEG	NEG	NEG	NEG	NEG	NEG	NEG	NEG	NEG
09V35055	CC22-MRSA-IV, Barnim/UK-EMRSA-15	POS	POS	POS	NEG	NEG	POS	NEG	NEG	NEG	NEG	NEG	NEG	NEG	NEG	NEG	NEG	NEG	NEG	NEG	NEG	NEG	NEG	NEG	NEG	NEG	NEG	NEG	NEG	NEG	NEG	NEG	NEG	NEG	NEG	NEG	NEG	NEG	NEG	NEG	NEG	NEG	NEG	NEG	NEG	NEG
09V35720	CC22-MRSA-IV, Barnim/UK-EMRSA-15	POS	POS	POS	NEG	NEG	NEG	NEG	NEG	NEG	NEG	NEG	NEG	NEG	NEG	NEG	NEG	NEG	NEG	NEG	NEG	NEG	NEG	NEG	NEG	NEG	NEG	NEG	NEG	NEG	NEG	NEG	NEG	NEG	NEG	NEG	NEG	NEG	NEG	NEG	NEG	NEG	NEG	NEG	NEG	NEG
09V36683	CC22-MRSA-IV, Barnim/UK-EMRSA-15	POS	POS	POS	NEG	NEG	POS	NEG	NEG	NEG	NEG	NEG	NEG	NEG	NEG	NEG	NEG	NEG	NEG	NEG	NEG	NEG	NEG	NEG	NEG	NEG	NEG	NEG	NEG	NEG	NEG	NEG	NEG	NEG	NEG	NEG	NEG	NEG	NEG	NEG	NEG	NEG	NEG	NEG	NEG	NEG
09V37636	CC22-MRSA-IV, Barnim/UK-EMRSA-15	POS	POS	POS	NEG	NEG	POS	NEG	NEG	NEG	NEG	NEG	NEG	NEG	NEG	NEG	NEG	NEG	NEG	NEG	NEG	NEG	NEG	NEG	NEG	NEG	NEG	NEG	NEG	NEG	NEG	NEG	NEG	NEG	NEG	NEG	NEG	NEG	NEG	NEG	NEG	NEG	NEG	NEG	NEG	NEG
09V37767	CC22-MRSA-IV, Barnim/UK-EMRSA-15	POS	POS	POS	NEG	NEG	POS	NEG	NEG	NEG	NEG	NEG	NEG	NEG	NEG	NEG	NEG	NEG	NEG	NEG	NEG	NEG	NEG	NEG	NEG	NEG	NEG	NEG	NEG	NEG	NEG	NEG	NEG	NEG	NEG	NEG	NEG	NEG	NEG	NEG	NEG	NEG	NEG	NEG	NEG	NEG
09V39554	CC22-MRSA-IV, Barnim/UK-EMRSA-15	POS	POS	POS	NEG	NEG	NEG	NEG	NEG	NEG	NEG	NEG	NEG	NEG	NEG	NEG	NEG	NEG	NEG	NEG	NEG	NEG	NEG	NEG	NEG	NEG	NEG	NEG	NEG	NEG	NEG	NEG	NEG	NEG	NEG	NEG	NEG	NEG	NEG	NEG	NEG	NEG	NEG	NEG	NEG	NEG
09V40977	CC22-MRSA-IV, Barnim/UK-EMRSA-15	POS	POS	POS	NEG	NEG	NEG	NEG	NEG	NEG	NEG	NEG	NEG	NEG	NEG	NEG	NEG	NEG	NEG	NEG	NEG	NEG	NEG	NEG	NEG	NEG	NEG	NEG	NEG	NEG	NEG	NEG	NEG	NEG	NEG	NEG	NEG	NEG	NEG	NEG	NEG	NEG	NEG	NEG	NEG	NEG
09V42954	CC22-MRSA-IV, Barnim/UK-EMRSA-15	POS	POS	POS	NEG	NEG	POS	NEG	NEG	NEG	NEG	NEG	NEG	NEG	NEG	NEG	NEG	NEG	NEG	NEG	NEG	NEG	NEG	NEG	NEG	NEG	NEG	NEG	NEG	NEG	NEG	NEG	NEG	NEG	NEG	NEG	NEG	NEG	NEG	NEG	NEG	NEG	NEG	NEG	NEG	NEG
09V44134	CC22-MRSA-IV, Barnim/UK-EMRSA-15	POS	POS	POS	NEG	NEG	NEG	NEG	NEG	NEG	NEG	NEG	NEG	NEG	NEG	NEG	NEG	NEG	NEG	NEG	NEG	NEG	NEG	NEG	NEG	NEG	NEG	NEG	NEG	NEG	NEG	NEG	NEG	NEG	NEG	NEG	NEG	NEG	NEG	NEG	NEG	NEG	NEG	NEG	NEG	NEG
09V46184	CC22-MRSA-IV, Barnim/UK-EMRSA-15	POS	POS	POS	NEG	NEG	NEG	NEG	NEG	NEG	NEG	NEG	NEG	NEG	NEG	NEG	NEG	NEG	NEG	NEG	NEG	NEG	NEG	NEG	NEG	NEG	NEG	NEG	NEG	NEG	NEG	NEG	NEG	NEG	NEG	NEG	NEG	NEG	NEG	NEG	NEG	NEG	NEG	NEG	NEG	NEG
09V47038	CC22-MRSA-IV, Barnim/UK-EMRSA-15	POS	POS	POS	NEG	NEG	POS	NEG	NEG	NEG	NEG	NEG	NEG	NEG	NEG	NEG	NEG	NEG	NEG	NEG	NEG	NEG	NEG	NEG	NEG	NEG	NEG	NEG	NEG	NEG	NEG	NEG	NEG	NEG	NEG	NEG	NEG	NEG	NEG	NEG	NEG	NEG	NEG	NEG	NEG	NEG
09V47487	CC22-MRSA-IV, Barnim/UK-EMRSA-15	POS	POS	POS	NEG	NEG	POS	NEG	NEG	NEG	NEG	NEG	NEG	NEG	NEG	NEG	NEG	NEG	NEG	NEG	NEG	NEG	NEG	NEG	NEG	NEG	NEG	NEG	NEG	NEG	NEG	NEG	NEG	NEG	AMB	NEG	NEG	NEG	NEG	NEG	NEG	NEG	NEG	NEG	NEG	NEG
09V48862	CC22-MRSA-IV, Barnim/UK-EMRSA-15	POS	POS	POS	NEG	NEG	NEG	NEG	NEG	NEG	NEG	NEG	NEG	NEG	NEG	NEG	NEG	NEG	NEG	NEG	NEG	NEG	NEG	NEG	NEG	NEG	NEG	NEG	NEG	NEG	NEG	NEG	NEG	NEG	NEG	NEG	NEG	NEG	NEG	NEG	NEG	NEG	NEG	NEG	NEG	NEG
09V32581	CC22-MRSA-V	POS	POS	POS	NEG	NEG	NEG	NEG	NEG	NEG	NEG	NEG	NEG	NEG	NEG	NEG	NEG	NEG	NEG	NEG	NEG	NEG	POS	NEG	POS	NEG	NEG	NEG	NEG	NEG	NEG	NEG	NEG	NEG	NEG	NEG	NEG	NEG	NEG	NEG	NEG	NEG	NEG	NEG	NEG	NEG
09V33822	CC45-MRSA-IV, Berlin EMRSA	POS	POS	POS	NEG	NEG	NEG	NEG	NEG	NEG	NEG	NEG	NEG	NEG	NEG	NEG	NEG	NEG	POS	POS	NEG	NEG	NEG	NEG	NEG	NEG	NEG	NEG	NEG	NEG	NEG	NEG	NEG	NEG	NEG	NEG	NEG	NEG	NEG	NEG	NEG	NEG	POS	NEG	NEG	NEG
09V34133	CC45-MRSA-IV, Berlin EMRSA	POS	POS	POS	NEG	NEG	NEG	NEG	NEG	NEG	NEG	NEG	NEG	NEG	NEG	NEG	NEG	NEG	POS	POS	NEG	NEG	NEG	NEG	POS	NEG	NEG	NEG	NEG	NEG	NEG	NEG	NEG	NEG	NEG	NEG	NEG	NEG	NEG	NEG	NEG	NEG	POS	NEG	NEG	NEG
09V45520	CC45-MRSA-IV, Berlin EMRSA	POS	POS	POS	NEG	NEG	NEG	NEG	NEG	NEG	NEG	NEG	NEG	NEG	NEG	NEG	NEG	NEG	POS	POS	NEG	NEG	NEG	NEG	NEG	NEG	NEG	NEG	NEG	NEG	NEG	NEG	NEG	NEG	NEG	NEG	NEG	NEG	NEG	NEG	NEG	NEG	POS	NEG	NEG	NEG
09V03558	CC398-MRSA-V	POS	POS	POS	NEG	NEG	NEG	NEG	NEG	NEG	NEG	NEG	NEG	NEG	NEG	NEG	NEG	NEG	NEG	NEG	NEG	NEG	NEG	NEG	NEG	POS	NEG	NEG	NEG	NEG	NEG	NEG	NEG	NEG	NEG	NEG	NEG	NEG	NEG	NEG	NEG	NEG	POS	NEG	NEG	NEG
09V07762	CC398-MRSA-V	POS	POS	POS	NEG	NEG	NEG	NEG	NEG	NEG	NEG	NEG	NEG	NEG	NEG	NEG	NEG	NEG	NEG	NEG	NEG	NEG	NEG	NEG	POS	NEG	NEG	NEG	NEG	NEG	NEG	NEG	NEG	NEG	NEG	NEG	NEG	NEG	NEG	NEG	NEG	NEG	POS	NEG	NEG	NEG
09V29815	CC398-MRSA-V	POS	POS	POS	POS	NEG	NEG	NEG	NEG	NEG	NEG	NEG	NEG	NEG	NEG	NEG	NEG	NEG	NEG	NEG	NEG	NEG	NEG	NEG	POS	POS	NEG	NEG	NEG	NEG	NEG	NEG	NEG	NEG	NEG	NEG	NEG	NEG	NEG	NEG	NEG	NEG	POS	NEG	NEG	NEG
**2010**																																														
10V03510	CC1/ST772-MRSA-V [PVL+], Bengal Bay Clone/WA MRSA-60	POS	POS	POS	NEG	NEG	POS	NEG	POS	NEG	POS	NEG	NEG	NEG	NEG	NEG	POS	NEG	POS	POS	NEG	NEG	NEG	NEG	NEG	NEG	NEG	NEG	NEG	NEG	NEG	NEG	NEG	POS	NEG	NEG	NEG	NEG	NEG	NEG	NEG	NEG	POS	NEG	NEG	NEG
10V31557	CC1-MRSA-IV, WA MRSA-1/57	POS	POS	POS	NEG	NEG	POS	NEG	NEG	NEG	NEG	NEG	NEG	NEG	NEG	NEG	NEG	NEG	POS	POS	NEG	NEG	NEG	NEG	AMB	NEG	NEG	NEG	NEG	NEG	NEG	NEG	NEG	NEG	NEG	NEG	NEG	NEG	NEG	NEG	NEG	NEG	POS	NEG	NEG	NEG
10V31910	CC1-MRSA-IV, WA MRSA-1/57	POS	POS	POS	NEG	NEG	NEG	NEG	NEG	NEG	NEG	NEG	NEG	NEG	NEG	NEG	NEG	NEG	POS	POS	NEG	NEG	NEG	NEG	POS	NEG	NEG	NEG	NEG	NEG	NEG	NEG	NEG	NEG	NEG	NEG	NEG	NEG	NEG	NEG	NEG	NEG	POS	NEG	NEG	NEG
10V01782	CC5/ST228-MRSA-I, South German EMRSA	POS	POS	POS	POS	NEG	NEG	NEG	NEG	NEG	NEG	NEG	NEG	NEG	NEG	NEG	POS	NEG	POS	POS	NEG	NEG	NEG	NEG	NEG	NEG	NEG	NEG	NEG	NEG	NEG	NEG	NEG	POS	NEG	POS	NEG	NEG	NEG	NEG	NEG	NEG	POS	NEG	NEG	NEG
10V02343	CC5/ST228-MRSA-I, South German EMRSA	POS	POS	POS	POS	NEG	NEG	NEG	NEG	NEG	NEG	NEG	NEG	NEG	NEG	NEG	POS	NEG	POS	POS	NEG	NEG	NEG	NEG	NEG	NEG	NEG	NEG	NEG	NEG	NEG	NEG	NEG	POS	NEG	NEG	NEG	NEG	NEG	NEG	NEG	NEG	POS	NEG	NEG	NEG
10B14561	CC5-MRSA-II, Rhine-Hesse EMRSA/UK-EMRSA-3	NEG	NEG	NEG	POS	NEG	NEG	NEG	NEG	NEG	NEG	NEG	NEG	NEG	NEG	NEG	NEG	POS	NEG	NEG	NEG	NEG	NEG	NEG	NEG	NEG	NEG	NEG	NEG	NEG	NEG	NEG	NEG	POS	NEG	NEG	NEG	NEG	NEG	NEG	NEG	NEG	POS	NEG	NEG	NEG
10F01042	CC5-MRSA-II, Rhine-Hesse EMRSA/UK-EMRSA-3	POS	POS	POS	POS	NEG	NEG	NEG	NEG	NEG	NEG	NEG	NEG	NEG	NEG	NEG	NEG	NEG	NEG	NEG	NEG	NEG	NEG	NEG	NEG	NEG	NEG	NEG	NEG	NEG	NEG	NEG	NEG	POS	AMB	NEG	NEG	NEG	NEG	NEG	NEG	NEG	POS	NEG	NEG	NEG
10U14746	CC5-MRSA-II, Rhine-Hesse EMRSA/UK-EMRSA-3	POS	POS	POS	POS	NEG	NEG	NEG	NEG	NEG	NEG	NEG	NEG	NEG	NEG	NEG	NEG	POS	NEG	NEG	NEG	NEG	NEG	NEG	NEG	NEG	NEG	NEG	NEG	NEG	NEG	NEG	NEG	POS	NEG	NEG	NEG	NEG	NEG	NEG	NEG	NEG	POS	NEG	NEG	NEG
10V01913	CC5-MRSA-II, Rhine-Hesse EMRSA/UK-EMRSA-3	POS	POS	POS	POS	NEG	NEG	NEG	NEG	NEG	NEG	NEG	NEG	NEG	NEG	NEG	NEG	POS	NEG	NEG	NEG	NEG	NEG	NEG	NEG	NEG	NEG	NEG	NEG	NEG	NEG	NEG	NEG	POS	NEG	NEG	NEG	NEG	NEG	NEG	NEG	NEG	POS	NEG	NEG	NEG
10V03216	CC5-MRSA-II, Rhine-Hesse EMRSA/UK-EMRSA-3	POS	POS	POS	POS	NEG	NEG	NEG	NEG	NEG	NEG	NEG	NEG	NEG	NEG	NEG	NEG	POS	NEG	NEG	NEG	NEG	NEG	NEG	NEG	NEG	NEG	NEG	NEG	NEG	NEG	NEG	NEG	POS	NEG	NEG	NEG	NEG	NEG	NEG	NEG	NEG	POS	NEG	NEG	NEG
10V03727	CC5-MRSA-II, Rhine-Hesse EMRSA/UK-EMRSA-3	POS	POS	POS	POS	NEG	NEG	NEG	NEG	NEG	NEG	NEG	NEG	NEG	NEG	NEG	NEG	POS	NEG	NEG	NEG	NEG	NEG	NEG	NEG	NEG	NEG	NEG	NEG	NEG	NEG	NEG	NEG	POS	NEG	NEG	NEG	NEG	NEG	NEG	NEG	NEG	POS	NEG	NEG	NEG
10V03824	CC5-MRSA-II, Rhine-Hesse EMRSA/UK-EMRSA-3	POS	POS	POS	POS	NEG	NEG	NEG	NEG	NEG	NEG	NEG	NEG	NEG	NEG	NEG	NEG	POS	NEG	NEG	NEG	NEG	NEG	NEG	NEG	NEG	NEG	NEG	NEG	NEG	NEG	NEG	NEG	POS	NEG	NEG	NEG	NEG	NEG	NEG	NEG	NEG	POS	NEG	NEG	NEG
10V08796	CC5-MRSA-II, Rhine-Hesse EMRSA/UK-EMRSA-3	NEG	NEG	NEG	POS	NEG	NEG	NEG	NEG	NEG	NEG	NEG	NEG	NEG	NEG	NEG	NEG	POS	NEG	NEG	NEG	NEG	NEG	NEG	NEG	NEG	NEG	NEG	NEG	NEG	NEG	NEG	NEG	POS	AMB	NEG	NEG	NEG	NEG	NEG	NEG	NEG	POS	NEG	NEG	NEG
10V09487	CC5-MRSA-II, Rhine-Hesse EMRSA/UK-EMRSA-3	POS	POS	POS	POS	NEG	NEG	NEG	NEG	NEG	NEG	NEG	NEG	NEG	NEG	NEG	NEG	NEG	NEG	NEG	NEG	NEG	NEG	NEG	NEG	NEG	NEG	NEG	NEG	NEG	NEG	NEG	NEG	POS	NEG	NEG	NEG	NEG	NEG	NEG	NEG	NEG	POS	NEG	NEG	NEG
10V10616	CC5-MRSA-II, Rhine-Hesse EMRSA/UK-EMRSA-3	NEG	NEG	NEG	POS	NEG	NEG	NEG	NEG	NEG	NEG	NEG	NEG	NEG	NEG	NEG	NEG	POS	NEG	NEG	NEG	NEG	NEG	NEG	NEG	NEG	NEG	NEG	NEG	NEG	NEG	NEG	NEG	POS	NEG	NEG	NEG	NEG	NEG	NEG	NEG	NEG	POS	NEG	NEG	NEG
10V10617	CC5-MRSA-II, Rhine-Hesse EMRSA/UK-EMRSA-3	POS	POS	POS	POS	NEG	NEG	NEG	NEG	NEG	NEG	NEG	NEG	NEG	NEG	NEG	NEG	AMB	NEG	NEG	NEG	NEG	NEG	NEG	NEG	NEG	NEG	NEG	NEG	NEG	NEG	NEG	NEG	POS	NEG	NEG	NEG	NEG	NEG	NEG	NEG	NEG	POS	NEG	NEG	NEG
10V12678	CC5-MRSA-II, Rhine-Hesse EMRSA/UK-EMRSA-3	POS	POS	POS	POS	NEG	NEG	NEG	NEG	NEG	NEG	NEG	NEG	NEG	NEG	NEG	NEG	POS	NEG	NEG	NEG	NEG	NEG	NEG	NEG	NEG	NEG	NEG	NEG	NEG	NEG	NEG	NEG	POS	NEG	NEG	NEG	NEG	NEG	NEG	NEG	NEG	POS	NEG	NEG	NEG
10V13218	CC5-MRSA-II, Rhine-Hesse EMRSA/UK-EMRSA-3	POS	POS	POS	POS	NEG	NEG	NEG	NEG	NEG	NEG	NEG	NEG	NEG	NEG	NEG	NEG	NEG	NEG	NEG	NEG	NEG	NEG	NEG	NEG	NEG	NEG	NEG	NEG	NEG	NEG	NEG	NEG	POS	NEG	NEG	NEG	NEG	NEG	NEG	NEG	NEG	POS	NEG	NEG	NEG
10V13638	CC5-MRSA-II, Rhine-Hesse EMRSA/UK-EMRSA-3	NEG	NEG	NEG	POS	NEG	NEG	NEG	NEG	NEG	NEG	NEG	NEG	NEG	NEG	NEG	NEG	NEG	NEG	NEG	NEG	NEG	NEG	NEG	NEG	NEG	NEG	NEG	NEG	NEG	NEG	NEG	NEG	POS	NEG	NEG	NEG	NEG	NEG	NEG	NEG	NEG	POS	NEG	NEG	NEG
10V14799	CC5-MRSA-II, Rhine-Hesse EMRSA/UK-EMRSA-3	POS	POS	POS	POS	NEG	NEG	NEG	NEG	NEG	NEG	NEG	NEG	NEG	NEG	NEG	NEG	POS	NEG	NEG	NEG	NEG	NEG	NEG	NEG	NEG	NEG	NEG	NEG	NEG	NEG	NEG	NEG	POS	NEG	NEG	NEG	NEG	NEG	NEG	NEG	NEG	POS	NEG	NEG	NEG
10V23825	CC5-MRSA-II, Rhine-Hesse EMRSA/UK-EMRSA-3	POS	POS	POS	POS	NEG	NEG	NEG	NEG	NEG	NEG	NEG	NEG	NEG	NEG	NEG	NEG	POS	NEG	NEG	NEG	NEG	NEG	NEG	NEG	NEG	NEG	NEG	NEG	NEG	NEG	NEG	NEG	POS	NEG	NEG	NEG	NEG	NEG	NEG	NEG	NEG	POS	NEG	NEG	NEG
10V31630	CC5-MRSA-II, Rhine-Hesse EMRSA/UK-EMRSA-3	POS	POS	POS	POS	NEG	NEG	NEG	NEG	NEG	NEG	NEG	NEG	NEG	NEG	NEG	NEG	POS	NEG	NEG	NEG	NEG	NEG	NEG	NEG	NEG	NEG	NEG	NEG	NEG	NEG	NEG	NEG	POS	NEG	NEG	NEG	NEG	NEG	NEG	NEG	NEG	POS	NEG	NEG	NEG
10V35386	CC5-MRSA-II, Rhine-Hesse EMRSA/UK-EMRSA-3	POS	POS	POS	POS	NEG	NEG	NEG	NEG	NEG	NEG	NEG	NEG	NEG	NEG	NEG	NEG	POS	NEG	NEG	NEG	NEG	NEG	NEG	NEG	NEG	NEG	NEG	NEG	NEG	NEG	NEG	NEG	POS	NEG	NEG	NEG	NEG	NEG	NEG	NEG	NEG	POS	NEG	NEG	NEG
10V37507	CC5-MRSA-II, Rhine-Hesse EMRSA/UK-EMRSA-3	POS	POS	POS	POS	NEG	NEG	NEG	NEG	NEG	NEG	NEG	NEG	NEG	NEG	NEG	NEG	NEG	NEG	NEG	NEG	NEG	NEG	NEG	NEG	NEG	NEG	NEG	NEG	NEG	NEG	NEG	NEG	POS	NEG	NEG	NEG	NEG	NEG	NEG	NEG	NEG	POS	NEG	NEG	NEG
10V38321	CC5-MRSA-II, Rhine-Hesse EMRSA/UK-EMRSA-3	POS	POS	POS	POS	NEG	NEG	NEG	NEG	NEG	NEG	NEG	NEG	NEG	NEG	NEG	NEG	NEG	NEG	NEG	NEG	NEG	NEG	NEG	NEG	NEG	NEG	NEG	NEG	NEG	NEG	NEG	NEG	POS	NEG	NEG	NEG	NEG	NEG	NEG	NEG	NEG	POS	NEG	NEG	NEG
10V40861	CC5-MRSA-II, Rhine-Hesse EMRSA/UK-EMRSA-3	POS	POS	POS	POS	NEG	NEG	NEG	NEG	NEG	NEG	NEG	NEG	NEG	NEG	NEG	NEG	POS	NEG	NEG	NEG	NEG	NEG	NEG	NEG	NEG	NEG	NEG	NEG	NEG	NEG	NEG	NEG	POS	NEG	NEG	NEG	NEG	NEG	NEG	NEG	NEG	POS	NEG	NEG	NEG
10V41383	CC5-MRSA-II, Rhine-Hesse EMRSA/UK-EMRSA-3	POS	POS	POS	POS	NEG	NEG	NEG	NEG	NEG	NEG	NEG	NEG	NEG	NEG	NEG	NEG	POS	NEG	NEG	NEG	NEG	NEG	NEG	NEG	NEG	NEG	NEG	NEG	NEG	NEG	NEG	NEG	POS	NEG	NEG	NEG	NEG	NEG	NEG	NEG	NEG	POS	NEG	NEG	NEG
10V45069	CC5-MRSA-II, Rhine-Hesse EMRSA/UK-EMRSA-3	POS	POS	POS	POS	NEG	AMB	NEG	NEG	NEG	NEG	NEG	NEG	AMB	NEG	NEG	NEG	POS	NEG	NEG	NEG	NEG	NEG	NEG	NEG	NEG	NEG	NEG	NEG	NEG	NEG	NEG	NEG	POS	POS	NEG	NEG	NEG	NEG	NEG	NEG	NEG	POS	NEG	NEG	NEG
10V01508	CC80-MRSA-IV [PVL+], European caMRSA Clone	POS	POS	POS	NEG	NEG	NEG	NEG	NEG	NEG	NEG	NEG	NEG	NEG	NEG	NEG	NEG	NEG	POS	POS	NEG	POS	NEG	NEG	POS	NEG	NEG	NEG	NEG	NEG	NEG	NEG	NEG	NEG	NEG	NEG	NEG	NEG	NEG	NEG	NEG	NEG	POS	NEG	NEG	NEG
10V34757	CC80-MRSA-IV [PVL+], European caMRSA Clone	POS	POS	POS	NEG	NEG	NEG	NEG	NEG	NEG	NEG	NEG	NEG	NEG	NEG	NEG	NEG	NEG	POS	POS	NEG	POS	NEG	NEG	POS	NEG	NEG	NEG	NEG	NEG	NEG	NEG	NEG	NEG	NEG	NEG	NEG	NEG	NEG	NEG	NEG	NEG	POS	NEG	NEG	NEG
10B06681	CC22-MRSA-IV, Barnim/UK-EMRSA-15	POS	POS	POS	NEG	NEG	POS	NEG	NEG	NEG	NEG	NEG	NEG	NEG	NEG	NEG	NEG	NEG	NEG	NEG	NEG	NEG	NEG	NEG	NEG	NEG	NEG	NEG	NEG	NEG	NEG	NEG	NEG	NEG	NEG	NEG	NEG	NEG	NEG	NEG	NEG	NEG	NEG	NEG	NEG	NEG
10F00384	CC22-MRSA-IV, Barnim/UK-EMRSA-15	POS	POS	POS	NEG	NEG	POS	NEG	NEG	NEG	NEG	NEG	NEG	NEG	NEG	NEG	NEG	NEG	NEG	NEG	NEG	NEG	NEG	NEG	NEG	NEG	NEG	NEG	NEG	NEG	NEG	NEG	NEG	NEG	NEG	NEG	NEG	NEG	NEG	NEG	NEG	NEG	NEG	NEG	NEG	NEG
10F00978	CC22-MRSA-IV, Barnim/UK-EMRSA-15	POS	POS	POS	NEG	NEG	POS	NEG	NEG	NEG	NEG	NEG	NEG	NEG	NEG	NEG	NEG	NEG	NEG	NEG	NEG	NEG	NEG	NEG	NEG	NEG	NEG	NEG	NEG	NEG	NEG	NEG	NEG	NEG	NEG	NEG	NEG	NEG	NEG	NEG	NEG	NEG	NEG	NEG	NEG	NEG
10F02923	CC22-MRSA-IV, Barnim/UK-EMRSA-15	POS	POS	POS	NEG	NEG	POS	NEG	NEG	NEG	NEG	NEG	NEG	NEG	NEG	NEG	NEG	NEG	NEG	AMB	NEG	NEG	NEG	NEG	NEG	NEG	NEG	NEG	NEG	NEG	NEG	NEG	NEG	NEG	NEG	NEG	NEG	NEG	NEG	NEG	NEG	NEG	NEG	NEG	NEG	NEG
10-Hygiene	CC22-MRSA-IV, Barnim/UK-EMRSA-15	POS	POS	POS	NEG	NEG	POS	NEG	NEG	NEG	NEG	NEG	NEG	NEG	NEG	NEG	NEG	NEG	NEG	NEG	NEG	NEG	NEG	NEG	NEG	NEG	NEG	NEG	NEG	NEG	NEG	NEG	NEG	NEG	NEG	NEG	NEG	NEG	NEG	NEG	NEG	NEG	NEG	NEG	NEG	NEG
10U02156	CC22-MRSA-IV, Barnim/UK-EMRSA-15	POS	POS	POS	NEG	NEG	POS	NEG	NEG	NEG	NEG	NEG	NEG	AMB	NEG	NEG	NEG	NEG	NEG	AMB	NEG	NEG	NEG	NEG	NEG	NEG	NEG	NEG	NEG	NEG	NEG	NEG	NEG	NEG	NEG	NEG	NEG	NEG	NEG	NEG	NEG	NEG	NEG	NEG	NEG	NEG
10U16289	CC22-MRSA-IV, Barnim/UK-EMRSA-15	POS	POS	POS	NEG	NEG	NEG	NEG	NEG	NEG	NEG	NEG	NEG	NEG	NEG	NEG	NEG	NEG	NEG	NEG	NEG	NEG	NEG	NEG	NEG	NEG	NEG	NEG	NEG	NEG	NEG	NEG	NEG	NEG	NEG	NEG	NEG	NEG	NEG	NEG	NEG	NEG	NEG	NEG	NEG	NEG
10V01346	CC22-MRSA-IV, Barnim/UK-EMRSA-15	POS	POS	POS	NEG	NEG	POS	NEG	NEG	NEG	NEG	NEG	NEG	NEG	NEG	NEG	NEG	NEG	NEG	NEG	NEG	NEG	NEG	NEG	NEG	NEG	NEG	NEG	NEG	NEG	NEG	NEG	NEG	NEG	NEG	NEG	NEG	NEG	NEG	NEG	NEG	NEG	NEG	NEG	NEG	NEG
10V03365	CC22-MRSA-IV, Barnim/UK-EMRSA-15	POS	POS	POS	NEG	NEG	POS	NEG	NEG	NEG	NEG	NEG	NEG	NEG	NEG	NEG	NEG	NEG	NEG	NEG	NEG	NEG	NEG	NEG	NEG	NEG	NEG	NEG	NEG	NEG	NEG	NEG	NEG	NEG	NEG	NEG	NEG	NEG	NEG	NEG	NEG	NEG	NEG	NEG	NEG	NEG
10V03540	CC22-MRSA-IV, Barnim/UK-EMRSA-15	POS	POS	POS	NEG	NEG	NEG	NEG	NEG	NEG	NEG	NEG	NEG	NEG	NEG	NEG	NEG	NEG	NEG	NEG	NEG	NEG	NEG	NEG	NEG	NEG	NEG	NEG	NEG	NEG	NEG	NEG	NEG	NEG	NEG	NEG	NEG	NEG	NEG	NEG	NEG	NEG	NEG	NEG	NEG	NEG
10V03819	CC22-MRSA-IV, Barnim/UK-EMRSA-15	POS	POS	POS	NEG	NEG	POS	NEG	NEG	NEG	NEG	NEG	NEG	NEG	NEG	NEG	NEG	NEG	NEG	NEG	NEG	NEG	NEG	NEG	NEG	NEG	NEG	NEG	NEG	NEG	NEG	NEG	NEG	NEG	NEG	NEG	NEG	NEG	NEG	NEG	NEG	NEG	NEG	NEG	NEG	NEG
10V04495	CC22-MRSA-IV, Barnim/UK-EMRSA-15	POS	POS	POS	NEG	NEG	POS	NEG	NEG	NEG	NEG	NEG	NEG	NEG	NEG	NEG	NEG	NEG	NEG	NEG	NEG	NEG	NEG	NEG	NEG	NEG	NEG	NEG	NEG	NEG	NEG	NEG	NEG	NEG	NEG	NEG	NEG	NEG	NEG	NEG	NEG	NEG	NEG	NEG	NEG	NEG
10V04498	CC22-MRSA-IV, Barnim/UK-EMRSA-15	POS	POS	POS	NEG	NEG	POS	NEG	NEG	NEG	NEG	NEG	NEG	NEG	NEG	NEG	NEG	NEG	NEG	NEG	NEG	NEG	NEG	NEG	NEG	NEG	NEG	NEG	NEG	NEG	NEG	NEG	NEG	NEG	NEG	NEG	NEG	NEG	NEG	NEG	NEG	NEG	NEG	NEG	NEG	NEG
10V04625	CC22-MRSA-IV, Barnim/UK-EMRSA-15	POS	POS	POS	NEG	NEG	POS	NEG	NEG	NEG	NEG	NEG	NEG	NEG	NEG	NEG	NEG	NEG	NEG	NEG	NEG	NEG	NEG	NEG	NEG	NEG	NEG	NEG	NEG	NEG	NEG	NEG	NEG	NEG	NEG	NEG	NEG	NEG	NEG	NEG	NEG	NEG	NEG	NEG	NEG	NEG
10V04685	CC22-MRSA-IV, Barnim/UK-EMRSA-15	POS	POS	POS	NEG	NEG	NEG	NEG	NEG	NEG	NEG	NEG	NEG	NEG	NEG	NEG	NEG	NEG	NEG	NEG	NEG	NEG	NEG	NEG	NEG	NEG	NEG	NEG	NEG	NEG	NEG	NEG	NEG	NEG	NEG	NEG	NEG	NEG	NEG	NEG	NEG	NEG	NEG	NEG	NEG	NEG
10V05305	CC22-MRSA-IV, Barnim/UK-EMRSA-15	POS	POS	POS	NEG	NEG	NEG	NEG	NEG	NEG	NEG	NEG	NEG	NEG	NEG	NEG	NEG	NEG	NEG	NEG	NEG	NEG	NEG	NEG	NEG	NEG	NEG	NEG	NEG	NEG	NEG	NEG	NEG	NEG	NEG	NEG	NEG	NEG	NEG	NEG	NEG	NEG	NEG	NEG	NEG	NEG
10V05474	CC22-MRSA-IV, Barnim/UK-EMRSA-15	POS	POS	POS	NEG	NEG	POS	NEG	NEG	NEG	NEG	NEG	NEG	NEG	NEG	NEG	NEG	NEG	NEG	NEG	NEG	NEG	NEG	NEG	NEG	NEG	NEG	NEG	NEG	NEG	NEG	NEG	NEG	NEG	NEG	NEG	NEG	NEG	NEG	NEG	NEG	NEG	NEG	NEG	NEG	NEG
10V05553	CC22-MRSA-IV, Barnim/UK-EMRSA-15	POS	POS	POS	NEG	NEG	NEG	NEG	NEG	NEG	NEG	NEG	NEG	NEG	NEG	NEG	NEG	NEG	NEG	NEG	NEG	NEG	NEG	NEG	NEG	NEG	NEG	NEG	NEG	NEG	NEG	NEG	NEG	NEG	NEG	NEG	NEG	NEG	NEG	NEG	NEG	NEG	NEG	NEG	NEG	NEG
10V06864	CC22-MRSA-IV, Barnim/UK-EMRSA-15	POS	POS	POS	NEG	NEG	POS	NEG	NEG	NEG	NEG	NEG	NEG	AMB	NEG	NEG	NEG	NEG	NEG	NEG	NEG	NEG	NEG	NEG	NEG	NEG	NEG	NEG	NEG	NEG	NEG	NEG	NEG	NEG	NEG	NEG	NEG	NEG	NEG	NEG	NEG	NEG	NEG	NEG	NEG	NEG
10V09545	CC22-MRSA-IV, Barnim/UK-EMRSA-15	POS	POS	POS	NEG	NEG	POS	NEG	NEG	NEG	NEG	NEG	NEG	AMB	NEG	NEG	NEG	NEG	NEG	NEG	NEG	NEG	NEG	NEG	NEG	POS	NEG	NEG	NEG	NEG	NEG	NEG	NEG	NEG	NEG	NEG	NEG	NEG	NEG	NEG	NEG	NEG	NEG	NEG	NEG	NEG
10V11922	CC22-MRSA-IV, Barnim/UK-EMRSA-15	POS	POS	POS	NEG	AMB	POS	NEG	NEG	NEG	NEG	NEG	NEG	NEG	NEG	NEG	NEG	NEG	NEG	NEG	NEG	NEG	NEG	NEG	NEG	NEG	NEG	NEG	NEG	NEG	NEG	NEG	NEG	NEG	NEG	NEG	NEG	NEG	NEG	NEG	NEG	NEG	AMB	NEG	NEG	NEG
10V12801	CC22-MRSA-IV, Barnim/UK-EMRSA-15	POS	POS	POS	NEG	AMB	POS	NEG	NEG	NEG	NEG	NEG	NEG	NEG	NEG	NEG	NEG	NEG	NEG	NEG	NEG	NEG	NEG	NEG	NEG	NEG	NEG	NEG	NEG	NEG	NEG	NEG	NEG	NEG	NEG	NEG	NEG	NEG	NEG	NEG	NEG	NEG	NEG	NEG	NEG	NEG
10V15165	CC22-MRSA-IV, Barnim/UK-EMRSA-15	POS	POS	POS	NEG	NEG	POS	NEG	NEG	NEG	NEG	NEG	NEG	NEG	NEG	NEG	NEG	NEG	NEG	NEG	NEG	NEG	NEG	NEG	NEG	NEG	NEG	NEG	NEG	NEG	NEG	NEG	NEG	NEG	NEG	NEG	NEG	NEG	NEG	NEG	NEG	NEG	NEG	NEG	NEG	NEG
10V18022	CC22-MRSA-IV, Barnim/UK-EMRSA-15	POS	POS	POS	NEG	NEG	POS	NEG	NEG	NEG	NEG	NEG	NEG	AMB	NEG	NEG	NEG	NEG	NEG	AMB	NEG	NEG	NEG	NEG	NEG	NEG	NEG	NEG	NEG	NEG	NEG	NEG	NEG	NEG	NEG	NEG	NEG	NEG	NEG	NEG	NEG	NEG	NEG	NEG	NEG	NEG
10V21655	CC22-MRSA-IV, Barnim/UK-EMRSA-15	POS	POS	POS	NEG	NEG	POS	NEG	NEG	NEG	NEG	NEG	NEG	NEG	NEG	NEG	NEG	NEG	NEG	NEG	NEG	NEG	NEG	NEG	NEG	NEG	NEG	NEG	NEG	NEG	NEG	NEG	NEG	NEG	NEG	NEG	NEG	NEG	NEG	NEG	NEG	NEG	NEG	NEG	NEG	NEG
10V21798	CC22-MRSA-IV, Barnim/UK-EMRSA-15	POS	POS	POS	NEG	NEG	NEG	NEG	NEG	NEG	NEG	NEG	NEG	NEG	NEG	NEG	NEG	NEG	NEG	NEG	NEG	NEG	NEG	NEG	NEG	NEG	NEG	NEG	NEG	NEG	NEG	NEG	NEG	NEG	NEG	NEG	NEG	NEG	NEG	NEG	NEG	NEG	NEG	NEG	NEG	NEG
10V23795	CC22-MRSA-IV, Barnim/UK-EMRSA-15	POS	POS	POS	NEG	NEG	NEG	NEG	NEG	NEG	NEG	NEG	NEG	NEG	NEG	NEG	NEG	NEG	NEG	NEG	NEG	NEG	NEG	NEG	NEG	NEG	NEG	NEG	NEG	NEG	NEG	NEG	NEG	NEG	NEG	NEG	NEG	NEG	NEG	NEG	NEG	NEG	NEG	NEG	NEG	NEG
10V25861	CC22-MRSA-IV, Barnim/UK-EMRSA-15	POS	POS	POS	NEG	NEG	POS	NEG	NEG	NEG	NEG	NEG	NEG	NEG	NEG	NEG	NEG	NEG	NEG	NEG	NEG	NEG	NEG	NEG	NEG	NEG	NEG	NEG	NEG	NEG	NEG	NEG	NEG	NEG	NEG	NEG	NEG	NEG	NEG	NEG	NEG	NEG	NEG	NEG	NEG	NEG
10V26165	CC22-MRSA-IV, Barnim/UK-EMRSA-15	POS	POS	POS	NEG	NEG	NEG	NEG	NEG	NEG	NEG	NEG	NEG	NEG	NEG	NEG	NEG	NEG	NEG	NEG	NEG	NEG	NEG	NEG	NEG	NEG	NEG	NEG	NEG	NEG	NEG	NEG	NEG	NEG	NEG	NEG	NEG	NEG	NEG	NEG	NEG	NEG	NEG	NEG	NEG	NEG
10V26326	CC22-MRSA-IV, Barnim/UK-EMRSA-15	POS	POS	POS	NEG	NEG	POS	NEG	NEG	NEG	NEG	NEG	NEG	NEG	NEG	NEG	NEG	NEG	NEG	NEG	NEG	NEG	NEG	NEG	NEG	NEG	NEG	NEG	NEG	NEG	NEG	NEG	NEG	NEG	NEG	NEG	NEG	NEG	NEG	NEG	NEG	NEG	NEG	NEG	NEG	NEG
10V29967	CC22-MRSA-IV, Barnim/UK-EMRSA-15	POS	POS	POS	NEG	NEG	POS	NEG	NEG	NEG	NEG	NEG	NEG	NEG	NEG	NEG	NEG	NEG	NEG	NEG	NEG	NEG	NEG	NEG	NEG	NEG	NEG	NEG	NEG	NEG	NEG	NEG	NEG	NEG	NEG	NEG	NEG	NEG	NEG	NEG	NEG	NEG	NEG	NEG	NEG	NEG
10V30523	CC22-MRSA-IV, Barnim/UK-EMRSA-15	POS	POS	POS	NEG	NEG	POS	NEG	NEG	NEG	NEG	NEG	NEG	NEG	NEG	NEG	NEG	NEG	NEG	NEG	NEG	NEG	NEG	NEG	NEG	NEG	NEG	NEG	NEG	NEG	NEG	NEG	NEG	NEG	NEG	NEG	NEG	NEG	NEG	NEG	NEG	NEG	NEG	NEG	NEG	NEG
10V31025	CC22-MRSA-IV, Barnim/UK-EMRSA-15	POS	POS	POS	NEG	NEG	POS	NEG	NEG	NEG	NEG	NEG	NEG	NEG	NEG	NEG	NEG	NEG	NEG	NEG	NEG	NEG	NEG	NEG	NEG	NEG	NEG	NEG	NEG	NEG	NEG	NEG	NEG	NEG	NEG	NEG	NEG	NEG	NEG	NEG	NEG	NEG	NEG	NEG	NEG	NEG
10V31056	CC22-MRSA-IV, Barnim/UK-EMRSA-15	POS	POS	POS	NEG	NEG	POS	NEG	NEG	NEG	NEG	NEG	NEG	NEG	NEG	NEG	NEG	NEG	NEG	NEG	NEG	NEG	NEG	NEG	NEG	NEG	NEG	NEG	NEG	NEG	NEG	NEG	NEG	NEG	NEG	NEG	NEG	NEG	NEG	NEG	NEG	NEG	NEG	NEG	NEG	NEG
10V32564	CC22-MRSA-IV, Barnim/UK-EMRSA-15	POS	POS	AMB	NEG	NEG	NEG	NEG	NEG	NEG	NEG	NEG	NEG	NEG	NEG	NEG	NEG	NEG	NEG	NEG	NEG	NEG	NEG	NEG	NEG	NEG	NEG	NEG	NEG	NEG	NEG	NEG	NEG	NEG	NEG	NEG	NEG	NEG	NEG	NEG	NEG	NEG	NEG	NEG	NEG	NEG
10V32855	CC22-MRSA-IV, Barnim/UK-EMRSA-15	POS	POS	POS	NEG	NEG	POS	NEG	NEG	NEG	NEG	NEG	NEG	NEG	NEG	NEG	NEG	NEG	NEG	NEG	NEG	NEG	NEG	NEG	NEG	NEG	NEG	NEG	NEG	NEG	NEG	NEG	NEG	NEG	NEG	NEG	NEG	NEG	NEG	NEG	NEG	NEG	NEG	NEG	NEG	NEG
10V33616	CC22-MRSA-IV, Barnim/UK-EMRSA-15	POS	POS	POS	NEG	NEG	NEG	NEG	NEG	NEG	NEG	NEG	NEG	NEG	NEG	NEG	NEG	NEG	NEG	NEG	NEG	NEG	NEG	NEG	NEG	NEG	NEG	NEG	NEG	NEG	NEG	NEG	NEG	NEG	NEG	NEG	NEG	NEG	NEG	NEG	NEG	NEG	NEG	NEG	NEG	NEG
10V34392	CC22-MRSA-IV, Barnim/UK-EMRSA-15	POS	POS	POS	NEG	NEG	POS	NEG	NEG	NEG	NEG	NEG	NEG	NEG	NEG	NEG	NEG	NEG	NEG	NEG	NEG	NEG	NEG	NEG	NEG	NEG	NEG	NEG	NEG	NEG	NEG	NEG	NEG	NEG	NEG	NEG	NEG	NEG	NEG	NEG	NEG	NEG	NEG	NEG	NEG	NEG
10V35980	CC22-MRSA-IV, Barnim/UK-EMRSA-15	POS	POS	AMB	NEG	NEG	NEG	NEG	NEG	NEG	NEG	NEG	NEG	NEG	NEG	NEG	NEG	NEG	NEG	NEG	NEG	NEG	NEG	NEG	NEG	NEG	NEG	NEG	NEG	NEG	NEG	NEG	NEG	NEG	NEG	NEG	NEG	NEG	NEG	NEG	NEG	NEG	NEG	NEG	NEG	NEG
10V37062	CC22-MRSA-IV, Barnim/UK-EMRSA-15	POS	POS	POS	NEG	NEG	POS	NEG	NEG	NEG	NEG	NEG	NEG	NEG	NEG	NEG	NEG	NEG	NEG	NEG	NEG	NEG	NEG	NEG	NEG	NEG	NEG	NEG	NEG	NEG	NEG	NEG	NEG	NEG	NEG	NEG	NEG	NEG	NEG	NEG	NEG	NEG	NEG	NEG	NEG	NEG
10V37408	CC22-MRSA-IV, Barnim/UK-EMRSA-15	POS	POS	AMB	NEG	NEG	POS	NEG	NEG	NEG	NEG	NEG	NEG	NEG	NEG	NEG	NEG	NEG	NEG	NEG	NEG	NEG	NEG	NEG	NEG	NEG	NEG	NEG	NEG	NEG	NEG	NEG	NEG	NEG	NEG	NEG	NEG	NEG	NEG	NEG	NEG	NEG	NEG	NEG	NEG	NEG
10V37820	CC22-MRSA-IV, Barnim/UK-EMRSA-15	POS	POS	AMB	NEG	NEG	POS	NEG	NEG	NEG	NEG	NEG	NEG	NEG	NEG	NEG	NEG	NEG	NEG	NEG	NEG	NEG	NEG	NEG	NEG	NEG	NEG	NEG	NEG	NEG	NEG	NEG	NEG	NEG	NEG	NEG	NEG	NEG	NEG	NEG	NEG	NEG	NEG	NEG	NEG	NEG
10V38669	CC22-MRSA-IV, Barnim/UK-EMRSA-15	POS	POS	AMB	NEG	NEG	NEG	NEG	NEG	NEG	NEG	NEG	NEG	NEG	NEG	NEG	NEG	NEG	NEG	NEG	NEG	NEG	NEG	NEG	NEG	NEG	NEG	NEG	NEG	NEG	NEG	NEG	NEG	NEG	NEG	NEG	NEG	NEG	NEG	NEG	NEG	NEG	NEG	NEG	NEG	NEG
10V38699	CC22-MRSA-IV, Barnim/UK-EMRSA-15	POS	POS	AMB	NEG	NEG	POS	NEG	NEG	NEG	NEG	NEG	NEG	NEG	NEG	NEG	NEG	NEG	NEG	NEG	NEG	NEG	NEG	NEG	NEG	NEG	NEG	NEG	NEG	NEG	NEG	NEG	NEG	NEG	NEG	NEG	NEG	NEG	NEG	NEG	NEG	NEG	NEG	NEG	NEG	NEG
10V39014	CC22-MRSA-IV, Barnim/UK-EMRSA-15	POS	POS	POS	NEG	NEG	POS	NEG	NEG	NEG	NEG	NEG	NEG	NEG	NEG	NEG	NEG	NEG	NEG	NEG	NEG	NEG	NEG	NEG	POS	NEG	NEG	NEG	NEG	NEG	NEG	NEG	NEG	NEG	NEG	NEG	NEG	NEG	NEG	NEG	NEG	NEG	NEG	NEG	NEG	NEG
10V39280	CC22-MRSA-IV, Barnim/UK-EMRSA-15	POS	POS	AMB	NEG	NEG	POS	NEG	NEG	NEG	NEG	NEG	NEG	NEG	NEG	NEG	NEG	NEG	NEG	NEG	NEG	NEG	NEG	NEG	NEG	NEG	NEG	NEG	NEG	NEG	NEG	NEG	NEG	NEG	NEG	NEG	NEG	NEG	NEG	NEG	NEG	NEG	NEG	NEG	NEG	NEG
10V39426	CC22-MRSA-IV, Barnim/UK-EMRSA-15	POS	POS	AMB	NEG	NEG	POS	NEG	NEG	NEG	NEG	NEG	NEG	NEG	NEG	NEG	NEG	NEG	NEG	NEG	NEG	NEG	NEG	NEG	NEG	NEG	NEG	NEG	NEG	NEG	NEG	NEG	NEG	NEG	NEG	NEG	NEG	NEG	NEG	NEG	NEG	NEG	NEG	NEG	NEG	NEG
10V41061	CC22-MRSA-IV, Barnim/UK-EMRSA-15	POS	POS	POS	NEG	NEG	NEG	NEG	NEG	NEG	NEG	NEG	NEG	NEG	NEG	NEG	NEG	NEG	NEG	NEG	NEG	NEG	NEG	NEG	NEG	NEG	NEG	NEG	NEG	NEG	NEG	NEG	NEG	NEG	NEG	NEG	NEG	NEG	NEG	NEG	NEG	NEG	NEG	NEG	NEG	NEG
10V44488	CC22-MRSA-IV, Barnim/UK-EMRSA-15	POS	POS	POS	NEG	NEG	POS	AMB	NEG	NEG	NEG	NEG	NEG	AMB	NEG	NEG	NEG	NEG	NEG	AMB	NEG	NEG	NEG	NEG	NEG	NEG	NEG	NEG	NEG	NEG	NEG	NEG	NEG	NEG	AMB	NEG	NEG	NEG	NEG	NEG	NEG	NEG	NEG	NEG	NEG	NEG
10V44924	CC22-MRSA-IV, Barnim/UK-EMRSA-15	POS	POS	POS	NEG	NEG	POS	NEG	NEG	NEG	NEG	NEG	NEG	NEG	NEG	NEG	NEG	NEG	NEG	NEG	NEG	NEG	NEG	NEG	NEG	NEG	NEG	NEG	NEG	NEG	NEG	NEG	NEG	NEG	NEG	NEG	NEG	NEG	NEG	NEG	NEG	NEG	NEG	NEG	NEG	NEG
10V44966	CC22-MRSA-IV, Barnim/UK-EMRSA-15	POS	POS	POS	NEG	NEG	POS	AMB	NEG	NEG	NEG	NEG	NEG	NEG	NEG	NEG	NEG	NEG	NEG	AMB	NEG	NEG	NEG	NEG	NEG	NEG	NEG	NEG	NEG	NEG	NEG	NEG	NEG	NEG	AMB	NEG	NEG	NEG	NEG	NEG	NEG	NEG	NEG	NEG	NEG	NEG
10V27260	CC22-MRSA-V	POS	POS	POS	NEG	NEG	NEG	NEG	NEG	NEG	NEG	NEG	NEG	NEG	NEG	NEG	NEG	NEG	NEG	NEG	NEG	NEG	POS	NEG	POS	NEG	NEG	NEG	NEG	NEG	NEG	NEG	NEG	NEG	NEG	NEG	NEG	NEG	NEG	NEG	NEG	NEG	NEG	NEG	NEG	NEG
10V15136	CC45-MRSA-IV, Berlin EMRSA	POS	POS	POS	POS	NEG	NEG	NEG	NEG	NEG	NEG	NEG	NEG	NEG	NEG	NEG	NEG	NEG	NEG	NEG	NEG	NEG	NEG	NEG	NEG	NEG	NEG	NEG	NEG	NEG	NEG	NEG	NEG	NEG	NEG	NEG	NEG	NEG	NEG	NEG	NEG	NEG	POS	NEG	NEG	NEG
10V29067	CC45-MRSA-IV, Berlin EMRSA	POS	POS	POS	NEG	NEG	NEG	NEG	NEG	NEG	NEG	NEG	NEG	NEG	NEG	NEG	NEG	NEG	POS	POS	NEG	NEG	NEG	NEG	NEG	NEG	NEG	NEG	NEG	NEG	NEG	NEG	NEG	NEG	NEG	NEG	NEG	NEG	NEG	NEG	NEG	NEG	POS	NEG	NEG	NEG
10V40116	CC45-MRSA-IV, Berlin EMRSA	POS	POS	POS	NEG	NEG	NEG	NEG	NEG	NEG	NEG	NEG	NEG	NEG	NEG	NEG	NEG	NEG	POS	POS	NEG	NEG	NEG	NEG	NEG	NEG	NEG	NEG	NEG	NEG	NEG	NEG	NEG	NEG	NEG	NEG	NEG	NEG	NEG	NEG	NEG	NEG	POS	NEG	NEG	NEG
10V10412	CC80-MRSA-IV [PVL+], European caMRSA Clone	NEG	NEG	NEG	NEG	NEG	NEG	NEG	NEG	NEG	NEG	NEG	NEG	NEG	NEG	NEG	NEG	NEG	POS	POS	NEG	NEG	NEG	NEG	NEG	NEG	NEG	NEG	NEG	NEG	NEG	NEG	NEG	NEG	NEG	NEG	NEG	NEG	NEG	NEG	NEG	NEG	POS	NEG	NEG	NEG
10V25105	CC398-MRSA-V	POS	POS	AMB	NEG	NEG	NEG	NEG	NEG	NEG	NEG	NEG	NEG	NEG	POS	NEG	NEG	NEG	NEG	NEG	NEG	NEG	NEG	NEG	AMB	POS	NEG	NEG	NEG	NEG	NEG	NEG	NEG	NEG	NEG	POS	NEG	NEG	NEG	NEG	NEG	NEG	POS	NEG	NEG	NEG

ISOLATE:	STRAIN	VIRULENCE : TOX.SCHOCK.TOXIN			VIRULENCE : ENTEROTOXINS																					VIRULENCE : HLG AND LEUKOCIDINS												
		toxic shock syndrome toxin 1			enterotoxin A	enterotoxin A, allele from strain 320E	enterotoxin A, allele from strain N315 = entero-toxin P	enterotoxin B	enterotoxin C	enterotoxin D	enterotoxin E	enterotoxin G	enterotoxin H	enterotoxin I	enterotoxin J	enterotoxin K	enterotoxin L	enterotoxin M	enterotoxin N		enterotoxin O	enterotoxin Q	enterotoxin R	enterotoxin U	enterotoxin-like protein ORF CM14	haemolysin gamma / leukocidin, component B	haemolysin gamma / leukocidin, component C		haemolysin gamma, component A	Panton Valentine leukocidin F component	Panton Valentine leukocidin S component	F component from hypothetical leukocidin from ruminants	S component from hypothetical leukocidin from ruminants	leukocidin D component	leukocidin E component	leukocidin/ haemolysin toxin family protein	leukocidin/haemolysin toxin family protein	
		tst1 (consensus)	tst1 ("human" allele)	tst1 ("bovine" allele)	sea	sea (320E)	sea (N315) / sep	seb	sec	sed	sef	seg	seh	sei	sej	sek	sel	selm	seln (consensus)	seln (other than RF122)	selo	seq	ser	selu	ORF CM14	lukF	lukS	lukS (ST22+ST45)	hlgA	lukF-PV	lukS-PV	lukF-PV (P83)	lukM	lukD	lukE	lukX	lukY	lukY (ST30+ST45)
**2009**																																						
09V32405	CC1/ST772-MRSA-V [PVL+], Bengal Bay Clone/WA MRSA-60	NEG	NEG	NEG	POS	NEG	NEG	NEG	POS	NEG	NEG	POS	NEG	POS	NEG	NEG	POS	POS	POS	POS	POS	NEG	NEG	POS	POS	POS	POS	AMB	POS	POS	POS	NEG	NEG	NEG	NEG	POS	POS	NEG
09V47486	CC1-MRSA-IV, WA MRSA-1/57	NEG	NEG	NEG	NEG	NEG	NEG	NEG	NEG	NEG	NEG	NEG	POS	NEG	NEG	NEG	NEG	NEG	NEG	NEG	NEG	NEG	NEG	NEG	NEG	POS	POS	AMB	POS	NEG	NEG	NEG	NEG	POS	POS	POS	POS	NEG
09V42357	CC5/ST228-MRSA-I, South German EMRSA	NEG	NEG	NEG	POS	NEG	NEG	NEG	NEG	NEG	NEG	POS	NEG	POS	POS	NEG	NEG	POS	POS	POS	POS	NEG	NEG	POS	NEG	POS	POS	AMB	POS	NEG	NEG	NEG	NEG	POS	POS	POS	POS	NEG
09F01028	CC5-MRSA-II, Rhine-Hesse EMRSA/UK-EMRSA-3	NEG	NEG	NEG	NEG	NEG	POS	NEG	NEG	POS	NEG	POS	NEG	POS	POS	NEG	NEG	POS	POS	POS	POS	NEG	AMB	POS	NEG	POS	POS	AMB	POS	NEG	NEG	NEG	NEG	POS	POS	POS	POS	NEG
09F01040	CC5-MRSA-II, Rhine-Hesse EMRSA/UK-EMRSA-3	NEG	NEG	NEG	NEG	NEG	POS	NEG	NEG	POS	NEG	POS	NEG	POS	POS	NEG	NEG	POS	POS	POS	POS	NEG	AMB	POS	NEG	POS	POS	AMB	POS	NEG	NEG	NEG	NEG	POS	POS	POS	POS	NEG
09F03018	CC5-MRSA-II, Rhine-Hesse EMRSA/UK-EMRSA-3	NEG	NEG	NEG	NEG	NEG	POS	NEG	NEG	POS	NEG	POS	NEG	POS	POS	NEG	NEG	POS	POS	POS	POS	NEG	POS	POS	NEG	POS	POS	AMB	POS	NEG	NEG	NEG	NEG	POS	POS	POS	POS	NEG
09F03619	CC5-MRSA-II, Rhine-Hesse EMRSA/UK-EMRSA-3	NEG	NEG	NEG	NEG	NEG	POS	NEG	NEG	POS	NEG	POS	NEG	POS	POS	NEG	NEG	POS	POS	POS	POS	NEG	POS	POS	NEG	POS	POS	NEG	POS	NEG	NEG	NEG	NEG	POS	POS	AMB	POS	NEG
09V01921	CC5-MRSA-II, Rhine-Hesse EMRSA/UK-EMRSA-3	NEG	NEG	NEG	NEG	NEG	POS	NEG	NEG	POS	NEG	POS	NEG	POS	POS	NEG	NEG	POS	POS	POS	POS	NEG	POS	POS	NEG	POS	POS	AMB	POS	NEG	NEG	NEG	NEG	POS	POS	POS	POS	NEG
09V04547	CC5-MRSA-II, Rhine-Hesse EMRSA/UK-EMRSA-3	NEG	NEG	NEG	NEG	NEG	POS	NEG	NEG	POS	NEG	POS	NEG	POS	POS	NEG	NEG	POS	POS	POS	POS	NEG	POS	POS	NEG	POS	POS	AMB	POS	NEG	NEG	NEG	NEG	POS	POS	POS	POS	NEG
09V05744	CC5-MRSA-II, Rhine-Hesse EMRSA/UK-EMRSA-3	NEG	NEG	NEG	NEG	NEG	POS	NEG	NEG	POS	NEG	POS	NEG	POS	POS	NEG	NEG	POS	POS	POS	POS	NEG	AMB	POS	NEG	POS	POS	AMB	POS	NEG	NEG	NEG	NEG	POS	POS	POS	POS	NEG
09V07448	CC5-MRSA-II, Rhine-Hesse EMRSA/UK-EMRSA-3	NEG	NEG	NEG	NEG	NEG	POS	NEG	NEG	POS	NEG	POS	NEG	POS	POS	NEG	NEG	POS	POS	POS	POS	NEG	POS	POS	NEG	POS	POS	AMB	POS	NEG	NEG	NEG	NEG	POS	POS	POS	POS	NEG
09V08032	CC5-MRSA-II, Rhine-Hesse EMRSA/UK-EMRSA-3	NEG	NEG	NEG	NEG	NEG	POS	NEG	NEG	POS	NEG	POS	NEG	POS	POS	NEG	NEG	POS	POS	POS	POS	NEG	POS	POS	NEG	POS	POS	AMB	POS	NEG	NEG	NEG	NEG	POS	POS	POS	POS	NEG
09V08761	CC5-MRSA-II, Rhine-Hesse EMRSA/UK-EMRSA-3	NEG	NEG	NEG	NEG	NEG	POS	NEG	NEG	POS	NEG	POS	NEG	POS	POS	NEG	NEG	POS	POS	POS	POS	NEG	POS	POS	NEG	POS	POS	AMB	POS	NEG	NEG	NEG	NEG	POS	POS	POS	POS	NEG
09V21706	CC5-MRSA-II, Rhine-Hesse EMRSA/UK-EMRSA-3		NEG	NEG	NEG	NEG	POS	NEG	NEG	POS	NEG	POS	NEG	POS	POS		NEG	POS		POS	POS		POS			POS	POS		POS	NEG	NEG	NEG	NEG	POS	POS	POS	POS	NEG
09V22079	CC5-MRSA-II, Rhine-Hesse EMRSA/UK-EMRSA-3	NEG	NEG	NEG	NEG	NEG	POS	NEG	NEG	POS	NEG	POS	NEG	POS	POS	NEG	NEG	POS	POS	POS	POS	NEG	POS	POS	NEG	POS	POS	AMB	POS	NEG	NEG	NEG	NEG	POS	POS	POS	POS	NEG
09V25980	CC5-MRSA-II, Rhine-Hesse EMRSA/UK-EMRSA-3	NEG	NEG	NEG	NEG	NEG	NEG	NEG	NEG	POS	NEG	POS	NEG	POS	POS	NEG	NEG	POS	POS	POS	POS	NEG	POS	POS	NEG	POS	POS	AMB	POS	NEG	NEG	NEG	NEG	POS	POS	POS	POS	NEG
09V26221	CC5-MRSA-II, Rhine-Hesse EMRSA/UK-EMRSA-3	NEG	NEG	NEG	NEG	NEG	POS	NEG	NEG	POS	NEG	POS	NEG	POS	POS	NEG	NEG	POS	POS	POS	POS	NEG	POS	POS	NEG	POS	POS	AMB	POS	NEG	NEG	NEG	NEG	POS	POS	POS	POS	NEG
09V28517	CC5-MRSA-II, Rhine-Hesse EMRSA/UK-EMRSA-3	NEG	NEG	NEG	NEG	NEG	POS	NEG	NEG	POS	NEG	POS	NEG	POS	POS	NEG	NEG	POS	POS	POS	POS	NEG	POS	POS	NEG	POS	POS	AMB	POS	NEG	NEG	NEG	NEG	POS	POS	POS	POS	NEG
09V28643	CC5-MRSA-II, Rhine-Hesse EMRSA/UK-EMRSA-3	NEG	NEG	NEG	NEG	NEG	POS	NEG	NEG	POS	NEG	POS	NEG	POS	POS	NEG	NEG	POS	POS	POS	POS	NEG	POS	POS	NEG	POS	POS	AMB	POS	NEG	NEG	NEG	NEG	POS	POS	POS	POS	NEG
09V28647	CC5-MRSA-II, Rhine-Hesse EMRSA/UK-EMRSA-3	NEG	NEG	NEG	NEG	NEG	POS	NEG	NEG	POS	NEG	POS	NEG	POS	POS	NEG	NEG	POS	POS	POS	POS	NEG	POS	POS	NEG	POS	POS	AMB	POS	NEG	NEG	NEG	NEG	POS	POS	POS	POS	NEG
09V29064	CC5-MRSA-II, Rhine-Hesse EMRSA/UK-EMRSA-3	NEG	NEG	NEG	NEG	NEG	POS	NEG	NEG	NEG	NEG	POS	NEG	POS	NEG	NEG	NEG	POS	POS	POS	POS	NEG	NEG	POS	NEG	POS	POS	AMB	POS	NEG	NEG	NEG	NEG	POS	POS	POS	POS	NEG
09V30464	CC5-MRSA-II, Rhine-Hesse EMRSA/UK-EMRSA-3	NEG	NEG	NEG	NEG	NEG	POS	NEG	NEG	POS	NEG	POS	NEG	POS	POS	NEG	NEG	POS	POS	POS	POS	NEG	POS	POS	NEG	POS	AMB	POS	POS	NEG	NEG	AMB	NEG	POS	POS	POS	POS	NEG
09V30701	CC5-MRSA-II, Rhine-Hesse EMRSA/UK-EMRSA-3	NEG	NEG	NEG	NEG	NEG	POS	NEG	NEG	POS	NEG	POS	NEG	POS	POS	NEG	NEG	POS	POS	POS	POS	NEG	POS	POS	NEG	POS	AMB	POS	POS	NEG	NEG	NEG	NEG	POS	POS	POS	POS	NEG
09V35418	CC5-MRSA-II, Rhine-Hesse EMRSA/UK-EMRSA-3	NEG	NEG	NEG	NEG	NEG	POS	NEG	NEG	POS	NEG	POS	NEG	POS	POS	NEG	NEG	POS	POS	POS	POS	NEG	POS	POS	NEG	POS	POS	AMB	AMB	NEG	NEG	NEG	NEG	POS	AMB	AMB	POS	NEG
09V37115	CC5-MRSA-II, Rhine-Hesse EMRSA/UK-EMRSA-3	NEG	NEG	NEG	NEG	NEG	POS	NEG	NEG	POS	NEG	POS	NEG	POS	POS	NEG	NEG	POS	POS	POS	POS	NEG	AMB	POS	NEG	POS	POS	AMB	POS	NEG	NEG	NEG	NEG	POS	POS	POS	POS	NEG
09V37466	CC5-MRSA-II, Rhine-Hesse EMRSA/UK-EMRSA-3	NEG	NEG	NEG	NEG	NEG	POS	NEG	NEG	POS	NEG	POS	NEG	POS	POS	NEG	NEG	POS	POS	POS	POS	NEG	POS	POS	NEG	POS	POS	AMB	POS	NEG	NEG	NEG	NEG	POS	POS	POS	POS	NEG
09V40100	CC5-MRSA-II, Rhine-Hesse EMRSA/UK-EMRSA-3	NEG	NEG	NEG	NEG	NEG	POS	NEG	NEG	POS	NEG	POS	NEG	POS	POS	NEG	NEG	POS	POS	POS	POS	NEG	POS	POS	NEG	POS	POS	AMB	POS	NEG	NEG	NEG	NEG	POS	POS	POS	POS	NEG
09V41542	CC5-MRSA-II, Rhine-Hesse EMRSA/UK-EMRSA-3	NEG	NEG	NEG	NEG	NEG	POS	NEG	NEG	POS	NEG	POS	NEG	POS	POS	NEG	NEG	POS	POS	POS	POS	NEG	POS	POS	NEG	POS	POS	AMB	POS	NEG	NEG	NEG	NEG	POS	POS	POS	POS	NEG
09V42595	CC5-MRSA-II, Rhine-Hesse EMRSA/UK-EMRSA-3	NEG	NEG	NEG	NEG	NEG	POS	NEG	NEG	POS	NEG	POS	NEG	POS	POS	NEG	NEG	POS	POS	POS	POS	NEG	POS	POS	NEG	POS	POS	AMB	POS	NEG	NEG	NEG	NEG	POS	POS	POS	POS	NEG
09V46894	CC5-MRSA-II, Rhine-Hesse EMRSA/UK-EMRSA-3	NEG	NEG	NEG	NEG	NEG	POS	NEG	NEG	POS	NEG	POS	NEG	POS	POS	NEG	NEG	POS	POS	POS	POS	NEG	POS	POS	NEG	POS	POS	AMB	POS	NEG	NEG	NEG	NEG	POS	POS	POS	POS	NEG
09V47383	CC5-MRSA-II, Rhine-Hesse EMRSA/UK-EMRSA-3	NEG	NEG	NEG	NEG	NEG	POS	NEG	NEG	POS	NEG	POS	NEG	POS	POS	NEG	NEG	POS	POS	POS	POS	NEG	POS	POS	NEG	POS	AMB	POS	POS	NEG	NEG	NEG	NEG	POS	POS	POS	POS	NEG
09V47393	CC5-MRSA-II, Rhine-Hesse EMRSA/UK-EMRSA-3	NEG	NEG	NEG	NEG	NEG	POS	NEG	NEG	POS	NEG	POS	NEG	POS	POS	NEG	NEG	POS	POS	POS	POS	NEG	POS	POS	NEG	POS	POS	AMB	POS	NEG	NEG	NEG	NEG	POS	POS	POS	POS	NEG
09V34574	CC5-MRSA-IV, Paediatric clone	NEG	NEG	NEG	NEG	NEG	NEG	NEG	NEG	POS	NEG	POS	NEG	POS	POS	NEG	NEG	POS	POS	POS	POS	NEG	NEG	POS	NEG	POS	POS	NEG	POS	NEG	NEG	NEG	NEG	POS	POS	AMB	POS	NEG
09F00871	CC8/ST72-MRSA-IV, USA700	NEG	NEG	NEG	NEG	NEG	NEG	NEG	NEG	NEG	NEG	POS	NEG	POS	NEG	NEG	NEG	POS	POS	POS	POS	NEG	NEG	POS	NEG	POS	POS	AMB	POS	NEG	NEG	NEG	NEG	POS	POS	POS	POS	NEG
09V20594	CC8-MRSA-IV, USA300	NEG	NEG	NEG	NEG	NEG	NEG	NEG	NEG	NEG	NEG	NEG	NEG	NEG	NEG	POS	NEG	NEG	NEG	NEG	NEG	POS	NEG	NEG	NEG	POS	POS	AMB	POS	POS	POS	NEG	NEG	POS	POS	POS	POS	NEG
09V28877	CC8-MRSA-IV, USA300	NEG	NEG	NEG	NEG	NEG	NEG	NEG	NEG	NEG	NEG	NEG	NEG	NEG	NEG	NEG	NEG	NEG	NEG	NEG	NEG	NEG	NEG	NEG	NEG	POS	POS	AMB	POS	POS	POS	NEG	NEG	POS	POS	POS	POS	NEG
09F00437	CC22-MRSA-IV, Barnim/UK-EMRSA-15	NEG	NEG	NEG	NEG	NEG	NEG	NEG	NEG	NEG	NEG	POS	NEG	POS	NEG	NEG	NEG	POS	POS	POS	POS	NEG	NEG	POS	NEG	POS	AMB	POS	POS	NEG	NEG	NEG	NEG	NEG	NEG	AMB	POS	NEG
09F01062	CC22-MRSA-IV, Barnim/UK-EMRSA-15	NEG	NEG	NEG	NEG	NEG	NEG	NEG	NEG	NEG	NEG	POS	NEG	POS	NEG	NEG	NEG	POS	POS	POS	POS	NEG	NEG	POS	NEG	POS	AMB	POS	POS	NEG	NEG	NEG	NEG	NEG	NEG	POS	POS	NEG
09U06692	CC22-MRSA-IV, Barnim/UK-EMRSA-15		NEG	NEG	NEG	NEG	NEG	NEG	NEG	NEG	NEG	AMB	NEG	POS	NEG		NEG	POS		POS	POS		NEG			AMB	NEG		POS	NEG	NEG	NEG	NEG	NEG	NEG	NEG	POS	NEG
09V04550	CC22-MRSA-IV, Barnim/UK-EMRSA-15	NEG	NEG	NEG	NEG	NEG	NEG	NEG	NEG	NEG	NEG	POS	NEG	POS	NEG	NEG	NEG	POS	POS	POS	POS	NEG	NEG	POS	NEG	AMB	AMB	POS	AMB	NEG	NEG	NEG	NEG	NEG	NEG	POS	POS	NEG
09V05293	CC22-MRSA-IV, Barnim/UK-EMRSA-15	NEG	NEG	NEG	NEG	NEG	NEG	NEG	NEG	NEG	NEG	POS	NEG	POS	NEG	NEG	NEG	POS	POS	POS	POS	NEG	NEG	POS	NEG	POS	AMB	POS	POS	NEG	NEG	NEG	NEG	NEG	NEG	POS	POS	NEG
09V05937	CC22-MRSA-IV, Barnim/UK-EMRSA-15		NEG	NEG	NEG	NEG	NEG	NEG	NEG	NEG	NEG	POS	NEG	POS	NEG		NEG	POS		POS	POS		NEG			POS	AMB		POS	NEG	NEG	NEG	NEG	NEG	NEG	POS	POS	NEG
09V05943	CC22-MRSA-IV, Barnim/UK-EMRSA-15		NEG	NEG	NEG	NEG	NEG	NEG	NEG	NEG	NEG	POS	NEG	POS	NEG		NEG	POS		POS	POS		NEG			POS	POS		POS	NEG	NEG	NEG	NEG	NEG	NEG	POS	POS	NEG
09V05947	CC22-MRSA-IV, Barnim/UK-EMRSA-15		NEG	NEG	NEG	NEG	NEG	NEG	NEG	NEG	NEG	POS	NEG	POS	NEG		NEG	POS		POS	POS		NEG			POS	AMB		POS	NEG	NEG	NEG	NEG	NEG	NEG	POS	POS	NEG
09V06275	CC22-MRSA-IV, Barnim/UK-EMRSA-15	NEG	NEG	NEG	NEG	NEG	NEG	NEG	NEG	NEG	NEG	POS	NEG	POS	NEG	NEG	NEG	POS	POS	POS	POS	NEG	NEG	POS	NEG	POS	AMB	POS	POS	NEG	NEG	NEG	NEG	NEG	NEG	POS	POS	NEG
09V08495	CC22-MRSA-IV, Barnim/UK-EMRSA-15	NEG	NEG	NEG	NEG	NEG	NEG	NEG	NEG	NEG	NEG	POS	NEG	POS	NEG	NEG	NEG	POS	POS	POS	POS	NEG	NEG	POS	NEG	POS	AMB	POS	POS	NEG	NEG	NEG	NEG	NEG	NEG	POS	POS	NEG
09V12464	CC22-MRSA-IV, Barnim/UK-EMRSA-15		NEG	NEG	NEG	NEG	NEG	NEG	NEG	NEG	NEG	POS	NEG	POS	NEG		NEG	POS		POS	POS		NEG			POS	POS		POS	NEG	NEG	NEG	NEG	NEG	NEG	POS	POS	NEG
09V15150	CC22-MRSA-IV, Barnim/UK-EMRSA-15		NEG	NEG	NEG	NEG	NEG	NEG	NEG	NEG	NEG	POS	NEG	POS	NEG		NEG	POS		POS	POS		NEG			POS	POS		POS	NEG	NEG	NEG	NEG	NEG	NEG	POS	POS	NEG
09V16014	CC22-MRSA-IV, Barnim/UK-EMRSA-15	NEG	NEG	NEG	NEG	NEG	NEG	NEG	NEG	NEG	NEG	POS	NEG	POS	NEG	NEG	NEG	POS	POS	POS	POS	NEG	NEG	POS	NEG	POS	AMB	POS	POS	NEG	NEG	NEG	AMB	NEG	NEG	POS	POS	NEG
09V19884	CC22-MRSA-IV, Barnim/UK-EMRSA-15		NEG	NEG	NEG	NEG	NEG	NEG	NEG	NEG	NEG	POS	NEG	POS	NEG		NEG	POS		POS	POS		NEG			POS	POS		POS	NEG	NEG	NEG	NEG	NEG	NEG	POS	POS	NEG
09V24370	CC22-MRSA-IV, Barnim/UK-EMRSA-15	NEG	NEG	NEG	NEG	NEG	NEG	NEG	POS	NEG	NEG	POS	NEG	POS	NEG	NEG	POS	POS	POS	POS	POS	NEG	NEG	POS	NEG	AMB	NEG	POS	POS	NEG	NEG	NEG	NEG	NEG	NEG	POS	POS	NEG
09V25431	CC22-MRSA-IV, Barnim/UK-EMRSA-15	NEG	NEG	NEG	NEG	NEG	NEG	NEG	NEG	NEG	NEG	POS	NEG	POS	NEG	NEG	NEG	POS	POS	POS	POS	NEG	NEG	POS	NEG	POS	AMB	POS	POS	NEG	NEG	NEG	NEG	NEG	NEG	POS	POS	NEG
09V27150	CC22-MRSA-IV, Barnim/UK-EMRSA-15	NEG	NEG	NEG	NEG	NEG	NEG	NEG	NEG	NEG	NEG	POS	NEG	POS	NEG	NEG	NEG	POS	POS	POS	POS	NEG	NEG	POS	NEG	POS	POS	AMB	POS	NEG	NEG	NEG	NEG	NEG	NEG	POS	POS	NEG
09V27748	CC22-MRSA-IV, Barnim/UK-EMRSA-15	NEG	NEG	NEG	NEG	NEG	NEG	NEG	NEG	NEG	NEG	POS	NEG	POS	NEG	NEG	NEG	POS	POS	POS	POS	NEG	NEG	POS	NEG	POS	AMB	POS	POS	NEG	NEG	NEG	NEG	NEG	NEG	POS	POS	NEG
09V28011	CC22-MRSA-IV, Barnim/UK-EMRSA-15	NEG	NEG	NEG	NEG	NEG	NEG	NEG	NEG	NEG	NEG	POS	NEG	POS	NEG	NEG	NEG	POS	POS	POS	POS	NEG	NEG	POS	NEG	POS	AMB	POS	POS	NEG	NEG	NEG	NEG	NEG	NEG	POS	POS	NEG
09V28179	CC22-MRSA-IV, Barnim/UK-EMRSA-15	NEG	NEG	NEG	NEG	NEG	NEG	NEG	NEG	NEG	NEG	POS	NEG	POS	NEG	NEG	NEG	POS	POS	POS	POS	NEG	NEG	POS	NEG	POS	POS	AMB	POS	NEG	NEG	NEG	NEG	NEG	NEG	POS	POS	NEG
09V28558	CC22-MRSA-IV, Barnim/UK-EMRSA-15	NEG	NEG	NEG	NEG	NEG	NEG	NEG	NEG	NEG	NEG	POS	NEG	POS	NEG	NEG	NEG	POS	POS	POS	POS	NEG	NEG	POS	NEG	POS	POS	AMB	POS	NEG	NEG	NEG	NEG	NEG	NEG	POS	POS	NEG
09V28681	CC22-MRSA-IV, Barnim/UK-EMRSA-15	NEG	NEG	NEG	NEG	NEG	NEG	NEG	NEG	NEG	NEG	POS	NEG	POS	NEG	NEG	NEG	POS	POS	POS	POS	NEG	NEG	POS	NEG	POS	POS	AMB	POS	NEG	NEG	NEG	NEG	NEG	NEG	POS	POS	NEG
09V29022	CC22-MRSA-IV, Barnim/UK-EMRSA-15	NEG	NEG	NEG	NEG	NEG	NEG	AMB	POS	NEG	NEG	POS	NEG	POS	NEG	NEG	POS	POS	POS	POS	POS	NEG	NEG	POS	NEG	POS	POS	AMB	POS	NEG	NEG	NEG	NEG	NEG	NEG	POS	POS	NEG
09V30408	CC22-MRSA-IV, Barnim/UK-EMRSA-15	NEG	NEG	NEG	NEG	NEG	NEG	NEG	POS	NEG	NEG	POS	NEG	POS	NEG	NEG	NEG	POS	POS	POS	POS	NEG	NEG	POS	NEG	POS	AMB	POS	POS	NEG	NEG	NEG	NEG	NEG	NEG	POS	POS	NEG
09V30634	CC22-MRSA-IV, Barnim/UK-EMRSA-15	NEG	NEG	NEG	NEG	NEG	NEG	NEG	NEG	NEG	NEG	POS	NEG	POS	NEG	NEG	NEG	POS	POS	POS	POS	NEG	NEG	POS	NEG	POS	AMB	POS	POS	NEG	NEG	NEG	NEG	NEG	NEG	POS	POS	NEG
09V31667	CC22-MRSA-IV, Barnim/UK-EMRSA-15	AMB	NEG	NEG	NEG	NEG	NEG	NEG	NEG	NEG	NEG	POS	NEG	POS	NEG	NEG	NEG	POS	POS	POS	POS	NEG	NEG	POS	NEG	POS	POS	AMB	POS	NEG	NEG	NEG	NEG	NEG	NEG	POS	POS	NEG
09V31882	CC22-MRSA-IV, Barnim/UK-EMRSA-15	NEG	NEG	NEG	NEG	NEG	NEG	NEG	NEG	NEG	NEG	POS	NEG	POS	NEG	NEG	NEG	POS	POS	POS	POS	NEG	NEG	POS	NEG	POS	AMB	POS	POS	NEG	NEG	NEG	NEG	NEG	NEG	POS	POS	NEG
09V32211	CC22-MRSA-IV, Barnim/UK-EMRSA-15	NEG	NEG	NEG	NEG	NEG	NEG	NEG	NEG	NEG	NEG	POS	NEG	POS	NEG	NEG	NEG	POS	POS	POS	POS	NEG	NEG	POS	NEG	POS	AMB	POS	POS	NEG	NEG	NEG	NEG	NEG	NEG	POS	POS	NEG
09V34899	CC22-MRSA-IV, Barnim/UK-EMRSA-15	NEG	NEG	NEG	NEG	NEG	NEG	NEG	NEG	NEG	NEG	POS	NEG	POS	NEG	NEG	NEG	POS	POS	POS	POS	NEG	NEG	POS	NEG	AMB	AMB	POS	NEG	NEG	NEG	NEG	NEG	NEG	NEG	POS	POS	NEG
09V35055	CC22-MRSA-IV, Barnim/UK-EMRSA-15	NEG	NEG	NEG	NEG	NEG	NEG	NEG	NEG	NEG	NEG	POS	NEG	POS	NEG	NEG	NEG	POS	POS	POS	POS	NEG	NEG	POS	NEG	POS	AMB	POS	AMB	NEG	NEG	NEG	NEG	NEG	NEG	POS	POS	NEG
09V35720	CC22-MRSA-IV, Barnim/UK-EMRSA-15	NEG	NEG	NEG	NEG	NEG	NEG	NEG	NEG	NEG	NEG	POS	NEG	POS	NEG	NEG	NEG	POS	POS	POS	POS	NEG	NEG	POS	NEG	NEG	AMB	POS	AMB	NEG	NEG	NEG	NEG	NEG	NEG	POS	POS	NEG
09V36683	CC22-MRSA-IV, Barnim/UK-EMRSA-15	NEG	NEG	NEG	NEG	NEG	NEG	NEG	NEG	NEG	NEG	POS	NEG	POS	NEG	NEG	NEG	POS	POS	POS	POS	NEG	NEG	POS	NEG	POS	AMB	POS	POS	NEG	NEG	NEG	NEG	NEG	NEG	POS	POS	NEG
09V37636	CC22-MRSA-IV, Barnim/UK-EMRSA-15	NEG	NEG	NEG	NEG	NEG	NEG	NEG	NEG	NEG	NEG	POS	NEG	POS	NEG	NEG	NEG	POS	POS	POS	POS	NEG	NEG	POS	NEG	NEG	NEG	POS	NEG	NEG	NEG	NEG	NEG	NEG	NEG	POS	POS	NEG
09V37767	CC22-MRSA-IV, Barnim/UK-EMRSA-15	NEG	NEG	NEG	NEG	NEG	NEG	NEG	NEG	NEG	NEG	POS	NEG	POS	NEG	NEG	NEG	POS	POS	POS	POS	NEG	NEG	POS	NEG	NEG	NEG	POS	POS	NEG	NEG	NEG	NEG	NEG	NEG	POS	POS	NEG
09V39554	CC22-MRSA-IV, Barnim/UK-EMRSA-15	NEG	NEG	NEG	NEG	NEG	NEG	NEG	NEG	NEG	NEG	AMB	NEG	POS	NEG	NEG	NEG	POS	POS	POS	POS	NEG	NEG	POS	NEG	NEG	NEG	POS	POS	NEG	NEG	NEG	NEG	NEG	NEG	POS	POS	NEG
09V40977	CC22-MRSA-IV, Barnim/UK-EMRSA-15	NEG	NEG	NEG	NEG	NEG	NEG	NEG	NEG	NEG	NEG	POS	NEG	POS	NEG	NEG	NEG	POS	POS	POS	POS	NEG	NEG	POS	NEG	POS	AMB	POS	POS	NEG	NEG	NEG	NEG	NEG	NEG	POS	POS	NEG
09V42954	CC22-MRSA-IV, Barnim/UK-EMRSA-15	NEG	NEG	NEG	NEG	NEG	NEG	NEG	NEG	NEG	NEG	POS	NEG	POS	NEG	NEG	NEG	POS	POS	POS	POS	NEG	NEG	POS	NEG	POS	AMB	POS	POS	NEG	NEG	NEG	NEG	NEG	NEG	POS	POS	NEG
09V44134	CC22-MRSA-IV, Barnim/UK-EMRSA-15	NEG	NEG	NEG	NEG	NEG	NEG	NEG	NEG	NEG	NEG	POS	NEG	POS	NEG	NEG	NEG	POS	POS	POS	POS	NEG	NEG	POS	NEG	POS	AMB	POS	POS	NEG	NEG	NEG	NEG	NEG	NEG	POS	POS	NEG
09V46184	CC22-MRSA-IV, Barnim/UK-EMRSA-15	NEG	NEG	NEG	NEG	NEG	NEG	NEG	NEG	NEG	NEG	POS	NEG	POS	NEG	NEG	NEG	POS	POS	POS	POS	NEG	NEG	POS	NEG	POS	AMB	POS	POS	NEG	NEG	NEG	NEG	NEG	NEG	POS	POS	NEG
09V47038	CC22-MRSA-IV, Barnim/UK-EMRSA-15	NEG	NEG	NEG	NEG	NEG	NEG	NEG	NEG	NEG	NEG	POS	NEG	POS	NEG	NEG	NEG	POS	POS	POS	POS	NEG	NEG	POS	NEG	POS	AMB	POS	POS	NEG	NEG	NEG	NEG	NEG	NEG	POS	POS	NEG
09V47487	CC22-MRSA-IV, Barnim/UK-EMRSA-15	NEG	NEG	NEG	NEG	NEG	NEG	NEG	NEG	NEG	NEG	POS	NEG	POS	NEG	NEG	NEG	POS	POS	POS	POS	NEG	NEG	POS	NEG	POS	AMB	POS	POS	NEG	NEG	NEG	AMB	NEG	NEG	POS	POS	NEG
09V48862	CC22-MRSA-IV, Barnim/UK-EMRSA-15	NEG	NEG	NEG	NEG	NEG	NEG	NEG	NEG	NEG	NEG	POS	NEG	POS	NEG	NEG	NEG	POS	POS	POS	POS	NEG	NEG	POS	NEG	POS	AMB	POS	POS	NEG	NEG	NEG	NEG	NEG	NEG	POS	POS	NEG
09V32581	CC22-MRSA-V	NEG	NEG	NEG	NEG	NEG	NEG	NEG	POS	NEG	NEG	POS	NEG	POS	NEG	NEG	POS	POS	POS	POS	POS	NEG	NEG	POS	NEG	NEG	NEG	POS	AMB	NEG	NEG	NEG	NEG	NEG	NEG	POS	POS	NEG
09V33822	CC45-MRSA-IV, Berlin EMRSA	NEG	NEG	NEG	NEG	NEG	NEG	NEG	NEG	NEG	NEG	NEG	NEG	POS	NEG	NEG	NEG	POS	POS	POS	POS	NEG	NEG	POS	NEG	POS	AMB	POS	POS	NEG	NEG	NEG	NEG	NEG	NEG	POS	NEG	POS
09V34133	CC45-MRSA-IV, Berlin EMRSA	NEG	NEG	NEG	NEG	NEG	NEG	NEG	NEG	NEG	NEG	POS	NEG	POS	NEG	NEG	NEG	POS	POS	POS	POS	NEG	NEG	POS	NEG	POS	NEG	POS	POS	NEG	NEG	NEG	NEG	NEG	NEG	POS	NEG	POS
09V45520	CC45-MRSA-IV, Berlin EMRSA	NEG	NEG	NEG	NEG	NEG	NEG	NEG	NEG	NEG	NEG	NEG	NEG	POS	NEG	NEG	NEG	POS	POS	POS	POS	NEG	NEG	POS	NEG	POS	AMB	POS	POS	NEG	NEG	NEG	NEG	NEG	NEG	POS	NEG	POS
09V03558	CC398-MRSA-V	NEG	NEG	NEG	NEG	NEG	NEG	NEG	NEG	NEG	NEG	NEG	NEG	NEG	NEG	NEG	NEG	NEG	NEG	NEG	NEG	NEG	NEG	NEG	NEG	POS	POS	NEG	POS	NEG	NEG	NEG	NEG	NEG	NEG	NEG	POS	NEG
09V07762	CC398-MRSA-V	NEG	NEG	NEG	NEG	NEG	NEG	NEG	NEG	NEG	NEG	NEG	NEG	NEG	NEG	NEG	NEG	NEG	NEG	NEG	NEG	NEG	NEG	NEG	NEG	POS	POS	NEG	POS	NEG	NEG	NEG	NEG	NEG	NEG	AMB	POS	NEG
09V29815	CC398-MRSA-V	NEG	NEG	NEG	NEG	NEG	NEG	NEG	NEG	NEG	NEG	NEG	NEG	NEG	NEG	NEG	NEG	NEG	NEG	NEG	NEG	NEG	NEG	NEG	NEG	POS	POS	AMB	POS	NEG	NEG	NEG	NEG	NEG	NEG	POS	POS	NEG
**2010**																																						
10V03510	CC1/ST772-MRSA-V [PVL+], Bengal Bay Clone/WA MRSA-60	NEG	NEG	NEG	POS	NEG	NEG	NEG	POS	NEG	NEG	POS	NEG	POS	NEG	NEG	POS	POS	POS	POS	POS	NEG	NEG	POS	POS	POS	POS	AMB	POS	POS	POS	NEG	NEG	NEG	NEG	POS	POS	NEG
10V31557	CC1-MRSA-IV, WA MRSA-1/57	NEG	NEG	NEG	NEG	NEG	NEG	NEG	NEG	NEG	NEG	NEG	POS	NEG	NEG	NEG	NEG	NEG	NEG	NEG	NEG	NEG	NEG	NEG	NEG	POS	POS	NEG	POS	NEG	NEG	NEG	NEG	POS	POS	AMB	POS	NEG
10V31910	CC1-MRSA-IV, WA MRSA-1/57	NEG	NEG	NEG	NEG	NEG	NEG	NEG	NEG	NEG	NEG	NEG	POS	NEG	NEG	NEG	NEG	NEG	NEG	NEG	NEG	NEG	NEG	NEG	NEG	POS	POS	AMB	POS	NEG	NEG	NEG	NEG	POS	POS	POS	POS	NEG
10V01782	CC5/ST228-MRSA-I, South German EMRSA	NEG	NEG	NEG	POS	NEG	NEG	NEG	NEG	NEG	NEG	POS	NEG	POS	NEG	NEG	NEG	POS	POS	POS	POS	NEG	NEG	POS	NEG	POS	POS	AMB	POS	NEG	NEG	NEG	NEG	POS	POS	POS	POS	NEG
10V02343	CC5/ST228-MRSA-I, South German EMRSA	NEG	NEG	NEG	POS	NEG	NEG	NEG	NEG	NEG	NEG	NEG	NEG	NEG	NEG	NEG	NEG	NEG	NEG	NEG	POS	NEG	NEG	NEG	NEG	POS	POS	AMB	POS	NEG	NEG	NEG	NEG	NEG	NEG	POS	POS	NEG
10B14561	CC5-MRSA-II, Rhine-Hesse EMRSA/UK-EMRSA-3	NEG	NEG	NEG	NEG	NEG	POS	NEG	NEG	NEG	NEG	POS	NEG	POS	NEG	NEG	NEG	POS	POS	POS	POS	NEG	NEG	POS	NEG	POS	POS	NEG	POS	NEG	NEG	NEG	NEG	POS	POS	POS	POS	NEG
10F01042	CC5-MRSA-II, Rhine-Hesse EMRSA/UK-EMRSA-3	NEG	NEG	NEG	NEG	NEG	NEG	NEG	NEG	POS	NEG	POS	NEG	POS	POS	NEG	NEG	POS	POS	POS	POS	NEG	POS	POS	NEG	POS	POS	AMB	POS	NEG	NEG	NEG	NEG	POS	POS	POS	POS	NEG
10U14746	CC5-MRSA-II, Rhine-Hesse EMRSA/UK-EMRSA-3	NEG	NEG	NEG	NEG	NEG	POS	NEG	NEG	POS	NEG	POS	NEG	POS	POS	NEG	NEG	POS	POS	POS	POS	NEG	POS	POS	NEG	POS	POS	POS	POS	NEG	NEG	NEG	NEG	POS	POS	POS	POS	NEG
10V01913	CC5-MRSA-II, Rhine-Hesse EMRSA/UK-EMRSA-3	NEG	NEG	NEG	NEG	NEG	POS	NEG	NEG	POS	NEG	POS	NEG	POS	POS	NEG	NEG	POS	POS	POS	POS	NEG	POS	POS	NEG	POS	POS	AMB	POS	NEG	NEG	NEG	NEG	POS	POS	POS	POS	NEG
10V03216	CC5-MRSA-II, Rhine-Hesse EMRSA/UK-EMRSA-3	NEG	NEG	NEG	NEG	NEG	POS	NEG	NEG	POS	NEG	POS	NEG	POS	POS	NEG	NEG	POS	POS	POS	POS	NEG	POS	POS	NEG	POS	POS	AMB	POS	NEG	NEG	NEG	NEG	POS	POS	POS	POS	NEG
10V03727	CC5-MRSA-II, Rhine-Hesse EMRSA/UK-EMRSA-3	NEG	NEG	NEG	NEG	NEG	POS	NEG	NEG	POS	NEG	POS	NEG	POS	POS	NEG	NEG	POS	POS	POS	POS	NEG	POS	POS	NEG	POS	POS	AMB	POS	NEG	NEG	NEG	NEG	POS	POS	POS	POS	NEG
10V03824	CC5-MRSA-II, Rhine-Hesse EMRSA/UK-EMRSA-3	NEG	NEG	NEG	NEG	NEG	POS	NEG	NEG	POS	NEG	POS	NEG	POS	POS	NEG	NEG	POS	POS	POS	POS	NEG	POS	POS	NEG	POS	POS	AMB	POS	NEG	NEG	NEG	NEG	POS	POS	POS	POS	NEG
10V08796	CC5-MRSA-II, Rhine-Hesse EMRSA/UK-EMRSA-3	NEG	NEG	NEG	NEG	NEG	POS	NEG	NEG	NEG	NEG	POS	NEG	POS	NEG	NEG	NEG	POS	POS	POS	POS	NEG	NEG	POS	NEG	POS	POS	AMB	POS	NEG	NEG	NEG	NEG	POS	POS	POS	POS	NEG
10V09487	CC5-MRSA-II, Rhine-Hesse EMRSA/UK-EMRSA-3	NEG	NEG	NEG	NEG	NEG	POS	NEG	NEG	POS	NEG	POS	NEG	POS	POS	NEG	NEG	POS	POS	POS	POS	NEG	POS	POS	NEG	POS	POS	AMB	POS	NEG	NEG	NEG	NEG	POS	POS	POS	POS	NEG
10V10616	CC5-MRSA-II, Rhine-Hesse EMRSA/UK-EMRSA-3	NEG	NEG	NEG	NEG	NEG	POS	NEG	NEG	NEG	NEG	POS	NEG	POS	NEG	NEG	NEG	POS	POS	POS	POS	NEG	NEG	POS	NEG	POS	POS	NEG	POS	NEG	NEG	NEG	NEG	POS	POS	POS	POS	NEG
10V10617	CC5-MRSA-II, Rhine-Hesse EMRSA/UK-EMRSA-3	NEG	NEG	NEG	NEG	NEG	POS	NEG	NEG	POS	NEG	POS	NEG	POS	POS	NEG	NEG	POS	POS	POS	POS	NEG	AMB	POS	NEG	POS	POS	NEG	POS	NEG	NEG	NEG	NEG	POS	POS	POS	POS	NEG
10V12678	CC5-MRSA-II, Rhine-Hesse EMRSA/UK-EMRSA-3	NEG	NEG	NEG	NEG	NEG	POS	NEG	NEG	POS	NEG	POS	NEG	POS	POS	NEG	NEG	POS	POS	POS	POS	NEG	POS	POS	NEG	POS	POS	AMB	POS	NEG	NEG	NEG	NEG	POS	POS	POS	POS	NEG
10V13218	CC5-MRSA-II, Rhine-Hesse EMRSA/UK-EMRSA-3	NEG	NEG	NEG	NEG	NEG	POS	NEG	NEG	NEG	NEG	POS	NEG	POS	NEG	NEG	NEG	POS	POS	POS	POS	NEG	NEG	POS	NEG	POS	POS	AMB	POS	NEG	NEG	NEG	NEG	POS	POS	POS	POS	NEG
10V13638	CC5-MRSA-II, Rhine-Hesse EMRSA/UK-EMRSA-3	NEG	NEG	NEG	NEG	NEG	POS	NEG	NEG	POS	NEG	POS	NEG	POS	POS	NEG	NEG	POS	POS	POS	POS	NEG	POS	POS	NEG	POS	POS	NEG	POS	NEG	NEG	NEG	NEG	POS	POS	POS	POS	NEG
10V14799	CC5-MRSA-II, Rhine-Hesse EMRSA/UK-EMRSA-3	NEG	NEG	NEG	NEG	NEG	POS	NEG	NEG	POS	NEG	POS	NEG	POS	POS	NEG	NEG	POS	POS	POS	POS	NEG	POS	POS	NEG	POS	POS	NEG	POS	NEG	NEG	NEG	NEG	POS	POS	POS	POS	NEG
10V23825	CC5-MRSA-II, Rhine-Hesse EMRSA/UK-EMRSA-3	NEG	NEG	NEG	NEG	NEG	NEG	NEG	NEG	POS	NEG	AMB	NEG	POS	POS	NEG	NEG	POS	POS	POS	POS	NEG	AMB	AMB	NEG	AMB	POS	NEG	POS	NEG	NEG	NEG	NEG	POS	AMB	AMB	POS	NEG
10V31630	CC5-MRSA-II, Rhine-Hesse EMRSA/UK-EMRSA-3	NEG	NEG	NEG	NEG	NEG	POS	NEG	NEG	POS	NEG	POS	NEG	POS	POS	NEG	NEG	POS	POS	POS	POS	NEG	POS	POS	NEG	POS	POS	NEG	POS	NEG	NEG	NEG	NEG	POS	POS	POS	POS	NEG
10V35386	CC5-MRSA-II, Rhine-Hesse EMRSA/UK-EMRSA-3	NEG	NEG	NEG	NEG	NEG	NEG	NEG	NEG	POS	NEG	POS	NEG	POS	POS	NEG	NEG	POS	POS	POS	POS	NEG	POS	POS	NEG	POS	POS	POS	POS	NEG	NEG	NEG	NEG	POS	POS	POS	POS	NEG
10V37507	CC5-MRSA-II, Rhine-Hesse EMRSA/UK-EMRSA-3	NEG	NEG	NEG	NEG	NEG	POS	NEG	NEG	POS	NEG	POS	NEG	POS	POS	NEG	NEG	POS	POS	POS	POS	NEG	POS	POS	NEG	POS	POS	POS	POS	NEG	NEG	NEG	NEG	POS	POS	POS	POS	NEG
10V38321	CC5-MRSA-II, Rhine-Hesse EMRSA/UK-EMRSA-3	NEG	NEG	NEG	NEG	NEG	POS	NEG	NEG	POS	NEG	POS	NEG	POS	POS	NEG	NEG	POS	POS	POS	AMB	NEG	AMB	POS	NEG	POS	POS	NEG	POS	NEG	NEG	NEG	NEG	POS	POS	AMB	POS	NEG
10V40861	CC5-MRSA-II, Rhine-Hesse EMRSA/UK-EMRSA-3	NEG	NEG	NEG	NEG	NEG	POS	NEG	NEG	POS	NEG	POS	NEG	POS	POS	NEG	NEG	POS	POS	POS	POS	NEG	AMB	POS	NEG	POS	POS	AMB	POS	NEG	NEG	NEG	NEG	POS	POS	AMB	POS	NEG
10V41383	CC5-MRSA-II, Rhine-Hesse EMRSA/UK-EMRSA-3	NEG	NEG	NEG	NEG	NEG	POS	NEG	NEG	POS	NEG	POS	NEG	POS	POS	NEG	NEG	POS	POS	POS	POS	NEG	POS	POS	NEG	POS	POS	AMB	POS	NEG	NEG	NEG	NEG	POS	POS	POS	POS	NEG
10V45069	CC5-MRSA-II, Rhine-Hesse EMRSA/UK-EMRSA-3	NEG	NEG	NEG	NEG	NEG	POS	NEG	NEG	POS	NEG	POS	NEG	POS	POS	NEG	NEG	POS	POS	POS	POS	NEG	POS	POS	NEG	POS	POS	POS	POS	NEG	NEG	NEG	NEG	POS	POS	POS	POS	NEG
10V01508	CC80-MRSA-IV [PVL+], European caMRSA Clone	NEG	NEG	NEG	NEG	NEG	NEG	NEG	NEG	NEG	NEG	NEG	NEG	NEG	NEG	NEG	NEG	NEG	NEG	NEG	NEG	NEG	NEG	NEG	NEG	POS	POS	AMB	POS	POS	POS	NEG	NEG	POS	POS	POS	POS	NEG
10V34757	CC80-MRSA-IV [PVL+], European caMRSA Clone	NEG	NEG	NEG	NEG	NEG	NEG	NEG	NEG	NEG	NEG	NEG	NEG	NEG	NEG	NEG	NEG	NEG	NEG	NEG	NEG	NEG	NEG	NEG	NEG	POS	POS	NEG	POS	POS	POS	NEG	NEG	POS	POS	POS	POS	NEG
10B06681	CC22-MRSA-IV, Barnim/UK-EMRSA-15	NEG	NEG	NEG	NEG	NEG	NEG	NEG	NEG	NEG	NEG	POS	NEG	POS	NEG	NEG	NEG	POS	POS	POS	POS	NEG	NEG	POS	NEG	POS	POS	POS	POS	NEG	NEG	NEG	NEG	NEG	NEG	POS	POS	NEG
10F00384	CC22-MRSA-IV, Barnim/UK-EMRSA-15	NEG	NEG	NEG	NEG	NEG	NEG	NEG	NEG	NEG	NEG	POS	NEG	POS	NEG	NEG	NEG	POS	POS	POS	POS	NEG	NEG	POS	NEG	POS	AMB	POS	POS	NEG	NEG	NEG	NEG	NEG	NEG	POS	POS	NEG
10F00978	CC22-MRSA-IV, Barnim/UK-EMRSA-15	NEG	AMB	NEG	NEG	NEG	NEG	NEG	NEG	NEG	NEG	POS	NEG	POS	NEG	NEG	NEG	POS	POS	POS	POS	NEG	NEG	POS	NEG	NEG	NEG	POS	AMB	NEG	NEG	NEG	NEG	NEG	NEG	POS	POS	NEG
10F02923	CC22-MRSA-IV, Barnim/UK-EMRSA-15	NEG	NEG	NEG	NEG	NEG	NEG	NEG	NEG	NEG	NEG	POS	NEG	POS	NEG	NEG	NEG	POS	POS	POS	POS	NEG	NEG	POS	NEG	POS	POS	POS	POS	NEG	NEG	NEG	NEG	NEG	NEG	POS	POS	NEG
10-Hygiene	CC22-MRSA-IV, Barnim/UK-EMRSA-15	NEG	AMB	NEG	NEG	NEG	NEG	NEG	NEG	NEG	NEG	POS	NEG	POS	NEG	NEG	NEG	POS	POS	POS	POS	NEG	NEG	POS	NEG	AMB	AMB	POS	POS	NEG	NEG	NEG	NEG	NEG	NEG	AMB	POS	NEG
10U02156	CC22-MRSA-IV, Barnim/UK-EMRSA-15	NEG	NEG	NEG	NEG	NEG	NEG	NEG	NEG	NEG	NEG	POS	NEG	POS	NEG	NEG	NEG	POS	POS	POS	POS	NEG	NEG	POS	NEG	POS	POS	POS	POS	NEG	NEG	NEG	NEG	NEG	NEG	POS	POS	NEG
10U16289	CC22-MRSA-IV, Barnim/UK-EMRSA-15	NEG	NEG	NEG	NEG	NEG	NEG	NEG	NEG	NEG	NEG	POS	NEG	POS	NEG	NEG	NEG	POS	POS	POS	POS	NEG	NEG	POS	NEG	POS	POS	POS	POS	NEG	NEG	NEG	NEG	NEG	NEG	POS	POS	NEG
10V01346	CC22-MRSA-IV, Barnim/UK-EMRSA-15	NEG	NEG	NEG	NEG	NEG	NEG	NEG	NEG	NEG	NEG	POS	NEG	POS	NEG	NEG	NEG	POS	POS	POS	POS	NEG	NEG	POS	NEG	POS	AMB	POS	POS	NEG	NEG	NEG	NEG	NEG	NEG	POS	POS	NEG
10V03365	CC22-MRSA-IV, Barnim/UK-EMRSA-15	NEG	NEG	NEG	NEG	NEG	NEG	NEG	NEG	NEG	NEG	POS	NEG	POS	NEG	NEG	NEG	POS	POS	POS	POS	NEG	NEG	POS	NEG	POS	AMB	POS	POS	NEG	NEG	NEG	NEG	NEG	NEG	POS	POS	NEG
10V03540	CC22-MRSA-IV, Barnim/UK-EMRSA-15	NEG	NEG	NEG	NEG	NEG	NEG	NEG	NEG	NEG	NEG	POS	NEG	POS	NEG	NEG	NEG	POS	POS	POS	POS	NEG	NEG	POS	NEG	POS	AMB	POS	POS	NEG	NEG	NEG	NEG	NEG	NEG	POS	POS	NEG
10V03819	CC22-MRSA-IV, Barnim/UK-EMRSA-15	NEG	NEG	NEG	NEG	NEG	NEG	NEG	NEG	NEG	NEG	POS	NEG	POS	NEG	NEG	NEG	POS	POS	POS	POS	NEG	NEG	POS	NEG	POS	AMB	POS	POS	NEG	NEG	NEG	NEG	NEG	NEG	POS	POS	NEG
10V04495	CC22-MRSA-IV, Barnim/UK-EMRSA-15	NEG	NEG	NEG	NEG	NEG	NEG	AMB	POS	NEG	NEG	POS	NEG	POS	NEG	NEG	POS	POS	POS	POS	POS	NEG	NEG	POS	NEG	POS	AMB	POS	POS	NEG	NEG	NEG	NEG	NEG	NEG	POS	POS	NEG
10V04498	CC22-MRSA-IV, Barnim/UK-EMRSA-15	NEG	NEG	NEG	NEG	NEG	NEG	NEG	NEG	NEG	NEG	POS	NEG	POS	NEG	NEG	NEG	POS	POS	POS	POS	NEG	NEG	POS	NEG	POS	AMB	POS	POS	NEG	NEG	NEG	NEG	NEG	NEG	POS	POS	NEG
10V04625	CC22-MRSA-IV, Barnim/UK-EMRSA-15	NEG	NEG	NEG	NEG	NEG	NEG	AMB	POS	NEG	NEG	POS	NEG	POS	NEG	NEG	POS	POS	POS	POS	POS	NEG	NEG	POS	NEG	POS	AMB	POS	POS	NEG	NEG	NEG	NEG	NEG	NEG	POS	POS	NEG
10V04685	CC22-MRSA-IV, Barnim/UK-EMRSA-15	NEG	NEG	NEG	NEG	NEG	NEG	NEG	NEG	NEG	NEG	POS	NEG	POS	NEG	NEG	NEG	POS	POS	POS	POS	NEG	NEG	POS	NEG	NEG	NEG	POS	AMB	NEG	NEG	NEG	NEG	NEG	NEG	POS	POS	NEG
10V05305	CC22-MRSA-IV, Barnim/UK-EMRSA-15	NEG	NEG	NEG	NEG	NEG	NEG	NEG	NEG	NEG	NEG	POS	NEG	POS	NEG	NEG	NEG	POS	POS	POS	POS	NEG	NEG	POS	NEG	NEG	NEG	POS	NEG	NEG	NEG	NEG	NEG	NEG	NEG	POS	POS	NEG
10V05474	CC22-MRSA-IV, Barnim/UK-EMRSA-15	NEG	NEG	NEG	NEG	NEG	NEG	NEG	NEG	NEG	NEG	POS	NEG	POS	NEG	NEG	NEG	POS	POS	POS	POS	NEG	NEG	POS	NEG	POS	AMB	POS	POS	NEG	NEG	NEG	NEG	NEG	NEG	POS	POS	NEG
10V05553	CC22-MRSA-IV, Barnim/UK-EMRSA-15	NEG	NEG	NEG	NEG	NEG	NEG	NEG	NEG	NEG	NEG	POS	NEG	POS	NEG	NEG	NEG	POS	POS	POS	POS	NEG	NEG	POS	NEG	AMB	NEG	POS	AMB	NEG	NEG	NEG	NEG	NEG	NEG	POS	POS	NEG
10V06864	CC22-MRSA-IV, Barnim/UK-EMRSA-15	NEG	AMB	NEG	NEG	NEG	NEG	NEG	NEG	AMB	NEG	POS	NEG	POS	NEG	NEG	NEG	POS	POS	POS	POS	NEG	NEG	POS	NEG	POS	POS	AMB	POS	NEG	NEG	NEG	NEG	NEG	NEG	POS	POS	NEG
10V09545	CC22-MRSA-IV, Barnim/UK-EMRSA-15	NEG	NEG	NEG	NEG	NEG	NEG	NEG	NEG	NEG	NEG	POS	NEG	POS	NEG	NEG	NEG	POS	POS	POS	POS	NEG	NEG	POS	NEG	POS	POS	AMB	POS	NEG	NEG	NEG	NEG	NEG	NEG	POS	POS	NEG
10V11922	CC22-MRSA-IV, Barnim/UK-EMRSA-15	NEG	NEG	NEG	NEG	NEG	NEG	NEG	NEG	NEG	NEG	POS	NEG	POS	NEG	NEG	NEG	POS	POS	POS	POS	NEG	NEG	POS	NEG	POS	AMB	POS	POS	NEG	NEG	NEG	NEG	NEG	NEG	POS	POS	NEG
10V12801	CC22-MRSA-IV, Barnim/UK-EMRSA-15	NEG	AMB	NEG	NEG	NEG	NEG	NEG	NEG	NEG	NEG	POS	NEG	POS	NEG	NEG	NEG	POS	POS	POS	POS	NEG	NEG	POS	NEG	POS	AMB	POS	POS	NEG	NEG	NEG	NEG	NEG	NEG	POS	POS	NEG
10V15165	CC22-MRSA-IV, Barnim/UK-EMRSA-15	NEG	NEG	NEG	NEG	NEG	NEG	NEG	NEG	NEG	NEG	POS	NEG	POS	NEG	NEG	NEG	POS	POS	POS	POS	NEG	NEG	POS	NEG	POS	AMB	POS	POS	NEG	NEG	NEG	NEG	NEG	NEG	POS	POS	NEG
10V18022	CC22-MRSA-IV, Barnim/UK-EMRSA-15	NEG	AMB	NEG	NEG	NEG	NEG	NEG	NEG	NEG	NEG	POS	NEG	POS	NEG	NEG	NEG	POS	POS	POS	POS	NEG	NEG	POS	NEG	POS	POS	POS	POS	NEG	NEG	NEG	NEG	NEG	NEG	POS	POS	NEG
10V21655	CC22-MRSA-IV, Barnim/UK-EMRSA-15	NEG	NEG	NEG	NEG	NEG	NEG	NEG	NEG	NEG	NEG	POS	NEG	POS	NEG	NEG	NEG	POS	POS	POS	POS	NEG	NEG	POS	NEG	POS	AMB	POS	POS	NEG	NEG	NEG	NEG	NEG	NEG	POS	POS	NEG
10V21798	CC22-MRSA-IV, Barnim/UK-EMRSA-15	NEG	NEG	NEG	NEG	NEG	NEG	NEG	NEG	NEG	NEG	POS	NEG	POS	NEG	NEG	NEG	POS	POS	POS	POS	NEG	NEG	POS	NEG	POS	AMB	POS	POS	NEG	NEG	NEG	NEG	NEG	NEG	POS	POS	NEG
10V23795	CC22-MRSA-IV, Barnim/UK-EMRSA-15	NEG	NEG	NEG	NEG	NEG	NEG	NEG	NEG	NEG	NEG	POS	NEG	POS	NEG	NEG	NEG	POS	POS	POS	POS	NEG	NEG	POS	NEG	POS	AMB	POS	POS	NEG	NEG	NEG	NEG	NEG	NEG	POS	POS	NEG
10V25861	CC22-MRSA-IV, Barnim/UK-EMRSA-15	NEG	NEG	NEG	NEG	NEG	NEG	NEG	NEG	NEG	NEG	POS	NEG	POS	NEG	NEG	NEG	POS	POS	POS	POS	NEG	NEG	POS	NEG	AMB	NEG	POS	POS	NEG	NEG	NEG	NEG	NEG	NEG	POS	POS	NEG
10V26165	CC22-MRSA-IV, Barnim/UK-EMRSA-15	NEG	NEG	NEG	NEG	NEG	NEG	POS	POS	NEG	NEG	POS	NEG	POS	NEG	NEG	POS	POS	POS	POS	POS	NEG	NEG	POS	NEG	POS	POS	POS	POS	NEG	NEG	NEG	NEG	NEG	NEG	POS	POS	NEG
10V26326	CC22-MRSA-IV, Barnim/UK-EMRSA-15	NEG	NEG	NEG	NEG	NEG	NEG	NEG	NEG	NEG	NEG	POS	NEG	POS	NEG	NEG	NEG	POS	POS	POS	POS	NEG	NEG	POS	NEG	NEG	NEG	POS	POS	NEG	NEG	NEG	NEG	NEG	NEG	POS	POS	NEG
10V29967	CC22-MRSA-IV, Barnim/UK-EMRSA-15	NEG	NEG	NEG	NEG	NEG	NEG	NEG	NEG	NEG	NEG	POS	NEG	POS	NEG	NEG	NEG	POS	POS	POS	POS	NEG	NEG	POS	NEG	POS	POS	POS	POS	NEG	NEG	NEG	NEG	NEG	NEG	POS	POS	NEG
10V30523	CC22-MRSA-IV, Barnim/UK-EMRSA-15	NEG	NEG	NEG	NEG	NEG	NEG	NEG	NEG	NEG	NEG	POS	NEG	POS	NEG	NEG	NEG	POS	POS	POS	POS	NEG	NEG	POS	NEG	POS	AMB	POS	AMB	NEG	NEG	NEG	NEG	NEG	NEG	POS	POS	NEG
10V31025	CC22-MRSA-IV, Barnim/UK-EMRSA-15	NEG	NEG	NEG	NEG	NEG	NEG	NEG	NEG	NEG	NEG	POS	NEG	POS	NEG	NEG	NEG	POS	POS	POS	POS	NEG	NEG	POS	NEG	POS	POS	POS	POS	NEG	NEG	NEG	NEG	NEG	NEG	POS	POS	NEG
10V31056	CC22-MRSA-IV, Barnim/UK-EMRSA-15	NEG	NEG	NEG	NEG	NEG	NEG	NEG	NEG	NEG	NEG	POS	NEG	POS	NEG	NEG	NEG	POS	POS	POS	POS	NEG	NEG	POS	NEG	POS	POS	POS	POS	NEG	NEG	NEG	NEG	NEG	NEG	POS	POS	NEG
10V32564	CC22-MRSA-IV, Barnim/UK-EMRSA-15	NEG	NEG	NEG	NEG	NEG	NEG	NEG	POS	NEG	NEG	POS	NEG	POS	NEG	NEG	POS	POS	POS	POS	POS	NEG	NEG	POS	NEG	NEG	NEG	POS	AMB	NEG	NEG	NEG	NEG	NEG	NEG	AMB	POS	NEG
10V32855	CC22-MRSA-IV, Barnim/UK-EMRSA-15	NEG	NEG	NEG	NEG	NEG	NEG	NEG	NEG	NEG	NEG	POS	NEG	POS	NEG	NEG	NEG	POS	POS	POS	POS	NEG	NEG	POS	NEG	AMB	AMB	POS	AMB	NEG	NEG	NEG	NEG	NEG	NEG	POS	POS	NEG
10V33616	CC22-MRSA-IV, Barnim/UK-EMRSA-15	NEG	NEG	NEG	NEG	NEG	NEG	NEG	NEG	NEG	NEG	POS	NEG	POS	NEG	NEG	NEG	POS	POS	POS	POS	NEG	NEG	POS	NEG	AMB	NEG	POS	POS	NEG	NEG	NEG	NEG	NEG	NEG	AMB	POS	NEG
10V34392	CC22-MRSA-IV, Barnim/UK-EMRSA-15	NEG	NEG	NEG	NEG	NEG	NEG	NEG	NEG	NEG	NEG	POS	NEG	POS	NEG	NEG	NEG	POS	POS	POS	POS	NEG	NEG	POS	NEG	POS	AMB	POS	POS	NEG	NEG	NEG	NEG	NEG	NEG	POS	POS	NEG
10V35980	CC22-MRSA-IV, Barnim/UK-EMRSA-15	NEG	NEG	NEG	NEG	NEG	NEG	NEG	NEG	NEG	NEG	POS	NEG	POS	NEG	NEG	NEG	POS	POS	POS	POS	NEG	NEG	POS	NEG	NEG	NEG	POS	NEG	NEG	NEG	NEG	NEG	NEG	NEG	POS	POS	NEG
10V37062	CC22-MRSA-IV, Barnim/UK-EMRSA-15	NEG	NEG	NEG	NEG	NEG	NEG	NEG	NEG	NEG	NEG	POS	NEG	POS	NEG	NEG	NEG	POS	POS	POS	POS	NEG	NEG	POS	NEG	POS	POS	POS	POS	NEG	NEG	NEG	NEG	NEG	NEG	POS	POS	NEG
10V37408	CC22-MRSA-IV, Barnim/UK-EMRSA-15	NEG	NEG	NEG	NEG	NEG	NEG	NEG	NEG	NEG	NEG	POS	NEG	POS	NEG	NEG	NEG	POS	POS	POS	POS	NEG	NEG	POS	NEG	AMB	NEG	POS	POS	NEG	NEG	NEG	NEG	NEG	NEG	AMB	POS	NEG
10V37820	CC22-MRSA-IV, Barnim/UK-EMRSA-15	NEG	NEG	NEG	NEG	NEG	NEG	NEG	NEG	NEG	NEG	POS	NEG	POS	NEG	NEG	NEG	POS	POS	POS	POS	NEG	NEG	POS	NEG	NEG	NEG	POS	AMB	NEG	NEG	NEG	NEG	NEG	NEG	POS	POS	NEG
10V38669	CC22-MRSA-IV, Barnim/UK-EMRSA-15	NEG	NEG	NEG	NEG	NEG	NEG	NEG	NEG	NEG	NEG	POS	NEG	POS	NEG	NEG	NEG	POS	POS	POS	POS	NEG	NEG	POS	NEG	NEG	NEG	POS	AMB	NEG	NEG	NEG	NEG	NEG	NEG	AMB	POS	NEG
10V38699	CC22-MRSA-IV, Barnim/UK-EMRSA-15	NEG	NEG	NEG	NEG	NEG	NEG	NEG	NEG	NEG	NEG	POS	NEG	POS	NEG	NEG	NEG	POS	POS	POS	POS	NEG	NEG	POS	NEG	NEG	NEG	POS	AMB	NEG	NEG	NEG	NEG	NEG	NEG	POS	POS	NEG
10V39014	CC22-MRSA-IV, Barnim/UK-EMRSA-15	NEG	NEG	NEG	NEG	NEG	NEG	NEG	NEG	NEG	NEG	POS	NEG	POS	NEG	NEG	NEG	POS	POS	POS	POS	NEG	NEG	POS	NEG	AMB	AMB	POS	POS	NEG	NEG	NEG	NEG	NEG	NEG	POS	POS	NEG
10V39280	CC22-MRSA-IV, Barnim/UK-EMRSA-15	NEG	NEG	NEG	NEG	NEG	NEG	NEG	NEG	NEG	NEG	POS	NEG	POS	NEG	NEG	NEG	POS	POS	POS	POS	NEG	NEG	POS	NEG	NEG	NEG	POS	AMB	NEG	NEG	NEG	NEG	NEG	NEG	AMB	POS	NEG
10V39426	CC22-MRSA-IV, Barnim/UK-EMRSA-15	NEG	NEG	NEG	NEG	NEG	NEG	NEG	NEG	NEG	NEG	POS	NEG	POS	NEG	NEG	NEG	POS	POS	POS	POS	NEG	NEG	POS	NEG	NEG	NEG	POS	AMB	NEG	NEG	NEG	NEG	NEG	NEG	POS	POS	NEG
10V41061	CC22-MRSA-IV, Barnim/UK-EMRSA-15	NEG	NEG	NEG	NEG	NEG	NEG	NEG	NEG	NEG	NEG	POS	NEG	POS	NEG	NEG	NEG	POS	POS	POS	POS	NEG	NEG	POS	NEG	POS	AMB	POS	POS	NEG	NEG	NEG	NEG	NEG	NEG	POS	POS	NEG
10V44488	CC22-MRSA-IV, Barnim/UK-EMRSA-15	NEG	AMB	NEG	NEG	NEG	NEG	NEG	NEG	NEG	NEG	POS	NEG	POS	NEG	NEG	NEG	POS	POS	POS	POS	NEG	NEG	POS	NEG	POS	POS	POS	POS	NEG	NEG	NEG	NEG	NEG	NEG	POS	POS	NEG
10V44924	CC22-MRSA-IV, Barnim/UK-EMRSA-15	NEG	NEG	NEG	NEG	NEG	NEG	NEG	NEG	NEG	NEG	POS	NEG	POS	NEG	NEG	NEG	POS	POS	POS	POS	NEG	NEG	POS	NEG	POS	POS	POS	POS	NEG	NEG	NEG	NEG	NEG	NEG	POS	POS	NEG
10V44966	CC22-MRSA-IV, Barnim/UK-EMRSA-15	NEG	AMB	NEG	NEG	NEG	NEG	AMB	POS	NEG	NEG	POS	NEG	POS	NEG	NEG	POS	POS	POS	POS	POS	NEG	NEG	POS	NEG	POS	POS	POS	POS	NEG	NEG	NEG	NEG	NEG	NEG	POS	POS	NEG
10V27260	CC22-MRSA-V	NEG	NEG	NEG	NEG	NEG	NEG	NEG	POS	NEG	NEG	POS	NEG	POS	NEG	NEG	POS	POS	POS	POS	POS	NEG	NEG	POS	NEG	POS	POS	POS	POS	NEG	NEG	NEG	NEG	NEG	NEG	POS	POS	NEG
10V15136	CC45-MRSA-IV, Berlin EMRSA	NEG	NEG	NEG	NEG	NEG	NEG	NEG	NEG	NEG	NEG	POS	NEG	POS	NEG	NEG	NEG	POS	POS	POS	POS	NEG	NEG	POS	NEG	POS	NEG	POS	POS	NEG	NEG	NEG	NEG	NEG	NEG	POS	NEG	POS
10V29067	CC45-MRSA-IV, Berlin EMRSA	NEG	NEG	NEG	NEG	NEG	NEG	NEG	NEG	NEG	NEG	NEG	NEG	POS	NEG	NEG	NEG	POS	POS	POS	POS	NEG	NEG	POS	NEG	POS	AMB	POS	POS	NEG	NEG	NEG	NEG	NEG	NEG	POS	NEG	POS
10V40116	CC45-MRSA-IV, Berlin EMRSA	NEG	NEG	NEG	NEG	NEG	NEG	NEG	NEG	NEG	NEG	NEG	NEG	POS	NEG	NEG	NEG	POS	POS	POS	POS	NEG	NEG	POS	NEG	POS	NEG	POS	POS	NEG	NEG	NEG	NEG	NEG	NEG	POS	NEG	POS
10V10412	CC80-MRSA-IV [PVL+], European caMRSA Clone	NEG	NEG	NEG	NEG	NEG	NEG	NEG	NEG	AMB	NEG	NEG	NEG	NEG	NEG	NEG	NEG	NEG	NEG	NEG	NEG	NEG	NEG	NEG	NEG	POS	POS	AMB	POS	POS	POS	POS	NEG	POS	POS	POS	POS	NEG
10V25105	CC398-MRSA-V	NEG	NEG	NEG	NEG	NEG	NEG	NEG	NEG	NEG	NEG	NEG	NEG	NEG	NEG	NEG	NEG	NEG	NEG	NEG	NEG	NEG	NEG	NEG	NEG	POS	POS	AMB	AMB	NEG	NEG	NEG	NEG	NEG	NEG	AMB	AMB	NEG

ISOLATE:	STRAIN	VIRULENCE : HAEMOLYSINS								VIRULENCE : HLB-CONV PHAGES		
		putative membrane protein	haemolysin alpha	putative membrane protein		haemolysin beta				staphylo-kinase	chemotaxis-inhibiting protein (CHIPS)	Staphyl. Comple-ment inhibitor
		hl	hla	hlIII (consensus)	hlIII (other than RF122)	hlb_probe 1	hlb_probe 2	hlb_probe 3	un-disrupted hlb	sak	chp	scn
**2009**												
09V32405	CC1/ST772-MRSA-V [PVL+], Bengal Bay Clone/WA MRSA-60	POS	POS	POS	POS	NEG	NEG	AMB	NEG	NEG	NEG	POS
09V47486	CC1-MRSA-IV, WA MRSA-1/57	POS	POS	POS	POS	POS	POS	POS	NEG	POS	NEG	POS
09V42357	CC5/ST228-MRSA-I, South German EMRSA	POS	POS	POS	POS	POS	POS	POS	NEG	POS	NEG	POS
09F01028	CC5-MRSA-II, Rhine-Hesse EMRSA/UK-EMRSA-3	POS	POS	POS	POS	POS	POS	NEG	NEG	POS	POS	POS
09F01040	CC5-MRSA-II, Rhine-Hesse EMRSA/UK-EMRSA-3	POS	POS	POS	POS	POS	POS	AMB	NEG	POS	POS	POS
09F03018	CC5-MRSA-II, Rhine-Hesse EMRSA/UK-EMRSA-3	POS	POS	POS	POS	POS	POS	POS	NEG	POS	POS	POS
09F03619	CC5-MRSA-II, Rhine-Hesse EMRSA/UK-EMRSA-3	POS	POS	POS	POS	AMB	POS	NEG	NEG	POS	POS	POS
09V01921	CC5-MRSA-II, Rhine-Hesse EMRSA/UK-EMRSA-3	POS	POS	POS	POS	POS	POS	POS	NEG	POS	POS	POS
09V04547	CC5-MRSA-II, Rhine-Hesse EMRSA/UK-EMRSA-3	POS	POS	POS	POS	POS	POS	AMB	NEG	POS	POS	POS
09V05744	CC5-MRSA-II, Rhine-Hesse EMRSA/UK-EMRSA-3	POS	POS	POS	POS	POS	POS	POS	NEG	POS	POS	POS
09V07448	CC5-MRSA-II, Rhine-Hesse EMRSA/UK-EMRSA-3	POS	POS	POS	POS	POS	POS	AMB	NEG	POS	POS	POS
09V08032	CC5-MRSA-II, Rhine-Hesse EMRSA/UK-EMRSA-3	POS	POS	POS	POS	POS	POS	POS	AMB	POS	POS	POS
09V08761	CC5-MRSA-II, Rhine-Hesse EMRSA/UK-EMRSA-3	POS	POS	POS	POS	POS	POS	AMB	NEG	POS	POS	POS
09V21706	CC5-MRSA-II, Rhine-Hesse EMRSA/UK-EMRSA-3	POS	POS		POS				AMB	POS		
09V22079	CC5-MRSA-II, Rhine-Hesse EMRSA/UK-EMRSA-3	POS	POS	POS	POS	POS	POS	POS	AMB	POS	POS	POS
09V25980	CC5-MRSA-II, Rhine-Hesse EMRSA/UK-EMRSA-3	POS	POS	POS	POS	POS	POS	POS	AMB	POS	NEG	POS
09V26221	CC5-MRSA-II, Rhine-Hesse EMRSA/UK-EMRSA-3	POS	POS	POS	POS	POS	POS	NEG	NEG	POS	POS	POS
09V28517	CC5-MRSA-II, Rhine-Hesse EMRSA/UK-EMRSA-3	POS	POS	POS	POS	POS	POS	POS	NEG	POS	POS	POS
09V28643	CC5-MRSA-II, Rhine-Hesse EMRSA/UK-EMRSA-3	POS	POS	POS	POS	POS	POS	POS	NEG	POS	POS	POS
09V28647	CC5-MRSA-II, Rhine-Hesse EMRSA/UK-EMRSA-3	POS	POS	POS	POS	POS	POS	POS	NEG	POS	POS	POS
09V29064	CC5-MRSA-II, Rhine-Hesse EMRSA/UK-EMRSA-3	POS	[illegible]	[illegible]	[illegible]	[illegible]	[illegible]	[illegible]	[illegible]	[illegible]	[illegible]	[illegible]
09V30464	CC5-MRSA-II, Rhine-Hesse EMRSA/UK-EMRSA-3	POS	[illegible]	[illegible]	[illegible]	[illegible]	[illegible]	[illegible]	[illegible]	[illegible]	[illegible]	[illegible]
09V30701	CC5-MRSA-II, Rhine-Hesse EMRSA/UK-EMRSA-3	POS	[illegible]	[illegible]	[illegible]	[illegible]	[illegible]	[illegible]	[illegible]	[illegible]	[illegible]	[illegible]
09V35418	CC5-MRSA-II, Rhine-Hesse EMRSA/UK-EMRSA-3	POS	[illegible]	[illegible]	[illegible]	[illegible]	[illegible]	[illegible]	[illegible]	[illegible]	[illegible]	[illegible]
09V37115	CC5-MRSA-II, Rhine-Hesse EMRSA/UK-EMRSA-3	POS	[illegible]	[illegible]	[illegible]	[illegible]	[illegible]	[illegible]	[illegible]	[illegible]	[illegible]	[illegible]
09V37466	CC5-MRSA-II, Rhine-Hesse EMRSA/UK-EMRSA-3	POS	[illegible]	[illegible]	[illegible]	[illegible]	[illegible]	[illegible]	[illegible]	[illegible]	[illegible]	[illegible]
09V40100	CC5-MRSA-II, Rhine-Hesse EMRSA/UK-EMRSA-3	POS	[illegible]	[illegible]	[illegible]	[illegible]	[illegible]	[illegible]	[illegible]	[illegible]	[illegible]	[illegible]
09V41542	CC5-MRSA-II, Rhine-Hesse EMRSA/UK-EMRSA-3	POS	[illegible]	[illegible]	[illegible]	[illegible]	[illegible]	[illegible]	[illegible]	[illegible]	[illegible]	[illegible]
09V42595	CC5-MRSA-II, Rhine-Hesse EMRSA/UK-EMRSA-3	POS	[illegible]	[illegible]	[illegible]	[illegible]	[illegible]	[illegible]	[illegible]	[illegible]	[illegible]	[illegible]
09V46894	CC5-MRSA-II, Rhine-Hesse EMRSA/UK-EMRSA-3	POS	[illegible]	[illegible]	[illegible]	[illegible]	[illegible]	[illegible]	[illegible]	[illegible]	[illegible]	[illegible]
09V47383	CC5-MRSA-II, Rhine-Hesse EMRSA/UK-EMRSA-3	POS	[illegible]	[illegible]	[illegible]	[illegible]	[illegible]	[illegible]	[illegible]	[illegible]	[illegible]	[illegible]
09V47393	CC5-MRSA-II, Rhine-Hesse EMRSA/UK-EMRSA-3	POS	[illegible]	[illegible]	[illegible]	[illegible]	[illegible]	[illegible]	[illegible]	[illegible]	[illegible]	[illegible]
09V34574	CC5-MRSA-IV, Paediatric clone	POS	[illegible]	[illegible]	[illegible]	[illegible]	[illegible]	[illegible]	[illegible]	[illegible]	[illegible]	[illegible]
09F00871	CC8/ST72-MRSA-IV, USA700	POS	[illegible]	[illegible]	[illegible]	[illegible]	[illegible]	[illegible]	[illegible]	[illegible]	[illegible]	[illegible]
09V20594	CC8-MRSA-IV, USA300	POS	[illegible]	[illegible]	[illegible]	[illegible]	[illegible]	[illegible]	[illegible]	[illegible]	[illegible]	[illegible]
09V28877	CC8-MRSA-IV, USA300	POS	[illegible]	[illegible]	[illegible]	[illegible]	[illegible]	[illegible]	[illegible]	[illegible]	[illegible]	[illegible]
09F00437	CC22-MRSA-IV, Barnim/UK-EMRSA-15	POS	[illegible]	[illegible]	[illegible]	[illegible]	[illegible]	[illegible]	[illegible]	[illegible]	[illegible]	[illegible]
09F01062	CC22-MRSA-IV, Barnim/UK-EMRSA-15	POS	[illegible]	[illegible]	[illegible]	[illegible]	[illegible]	[illegible]	[illegible]	[illegible]	[illegible]	[illegible]
09U06692	CC22-MRSA-IV, Barnim/UK-EMRSA-15	POS	[illegible]	[illegible]	[illegible]	[illegible]	[illegible]	[illegible]	[illegible]	[illegible]	[illegible]	[illegible]
09V04550	CC22-MRSA-IV, Barnim/UK-EMRSA-15	POS	[illegible]	[illegible]	[illegible]	[illegible]	[illegible]	[illegible]	[illegible]	[illegible]	[illegible]	[illegible]
09V05293	CC22-MRSA-IV, Barnim/UK-EMRSA-15	POS	[illegible]	[illegible]	[illegible]	[illegible]	[illegible]	[illegible]	[illegible]	[illegible]	[illegible]	[illegible]
09V05937	CC22-MRSA-IV, Barnim/UK-EMRSA-15	POS	[illegible]	[illegible]	[illegible]	[illegible]	[illegible]	[illegible]	[illegible]	[illegible]	[illegible]	[illegible]
09V05943	CC22-MRSA-IV, Barnim/UK-EMRSA-15	POS	[illegible]	[illegible]	[illegible]	[illegible]	[illegible]	[illegible]	[illegible]	[illegible]	[illegible]	[illegible]
09V05947	CC22-MRSA-IV, Barnim/UK-EMRSA-15	POS	[illegible]	[illegible]	[illegible]	[illegible]	[illegible]	[illegible]	[illegible]	[illegible]	[illegible]	[illegible]
09V06275	CC22-MRSA-IV, Barnim/UK-EMRSA-15	POS	[illegible]	[illegible]	[illegible]	[illegible]	[illegible]	[illegible]	[illegible]	[illegible]	[illegible]	[illegible]
09V08495	CC22-MRSA-IV, Barnim/UK-EMRSA-15	POS	[illegible]	[illegible]	[illegible]	[illegible]	[illegible]	[illegible]	[illegible]	[illegible]	[illegible]	[illegible]
09V12464	CC22-MRSA-IV, Barnim/UK-EMRSA-15	POS	[illegible]	[illegible]	[illegible]	[illegible]	[illegible]	[illegible]	[illegible]	[illegible]	[illegible]	[illegible]
09V15150	CC22-MRSA-IV, Barnim/UK-EMRSA-15	POS	[illegible]	[illegible]	[illegible]	[illegible]	[illegible]	[illegible]	[illegible]	[illegible]	[illegible]	[illegible]
09V16014	CC22-MRSA-IV, Barnim/UK-EMRSA-15	POS	[illegible]	[illegible]	[illegible]	[illegible]	[illegible]	[illegible]	[illegible]	[illegible]	[illegible]	[illegible]
09V19884	CC22-MRSA-IV, Barnim/UK-EMRSA-15	POS	[illegible]	[illegible]	[illegible]	[illegible]	[illegible]	[illegible]	[illegible]	[illegible]	[illegible]	[illegible]
09V24370	CC22-MRSA-IV, Barnim/UK-EMRSA-15	POS	[illegible]	[illegible]	[illegible]	[illegible]	[illegible]	[illegible]	[illegible]	[illegible]	[illegible]	[illegible]
09V25431	CC22-MRSA-IV, Barnim/UK-EMRSA-15	POS	[illegible]	[illegible]	[illegible]	[illegible]	[illegible]	[illegible]	[illegible]	[illegible]	[illegible]	[illegible]
09V27150	CC22-MRSA-IV, Barnim/UK-EMRSA-15	POS	[illegible]	[illegible]	[illegible]	[illegible]	[illegible]	[illegible]	[illegible]	[illegible]	[illegible]	[illegible]
09V27748	CC22-MRSA-IV, Barnim/UK-EMRSA-15	POS	[illegible]	[illegible]	[illegible]	[illegible]	[illegible]	[illegible]	[illegible]	[illegible]	[illegible]	[illegible]
09V28011	CC22-MRSA-IV, Barnim/UK-EMRSA-15	POS	[illegible]	[illegible]	[illegible]	[illegible]	[illegible]	[illegible]	[illegible]	[illegible]	[illegible]	[illegible]
09V28179	CC22-MRSA-IV, Barnim/UK-EMRSA-15	POS	[illegible]	[illegible]	[illegible]	[illegible]	[illegible]	[illegible]	[illegible]	[illegible]	[illegible]	[illegible]
09V28558	CC22-MRSA-IV, Barnim/UK-EMRSA-15	POS	[illegible]	[illegible]	[illegible]	[illegible]	[illegible]	[illegible]	[illegible]	[illegible]	[illegible]	[illegible]
09V28681	CC22-MRSA-IV, Barnim/UK-EMRSA-15	POS	[illegible]	[illegible]	[illegible]	[illegible]	[illegible]	[illegible]	[illegible]	[illegible]	[illegible]	[illegible]
09V29022	CC22-MRSA-IV, Barnim/UK-EMRSA-15	POS	[illegible]	[illegible]	[illegible]	[illegible]	[illegible]	[illegible]	[illegible]	[illegible]	[illegible]	[illegible]
09V30408	CC22-MRSA-IV, Barnim/UK-EMRSA-15	POS	[illegible]	[illegible]	[illegible]	[illegible]	[illegible]	[illegible]	[illegible]	[illegible]	[illegible]	[illegible]
09V30634	CC22-MRSA-IV, Barnim/UK-EMRSA-15	POS	[illegible]	[illegible]	[illegible]	[illegible]	[illegible]	[illegible]	[illegible]	[illegible]	[illegible]	[illegible]
09V31667	CC22-MRSA-IV, Barnim/UK-EMRSA-15	POS	[illegible]	[illegible]	[illegible]	[illegible]	[illegible]	[illegible]	[illegible]	[illegible]	[illegible]	[illegible]
09V31882	CC22-MRSA-IV, Barnim/UK-EMRSA-15	POS	[illegible]	[illegible]	[illegible]	[illegible]	[illegible]	[illegible]	[illegible]	[illegible]	[illegible]	[illegible]
09V32211	CC22-MRSA-IV, Barnim/UK-EMRSA-15	POS	[illegible]	[illegible]	[illegible]	[illegible]	[illegible]	[illegible]	[illegible]	[illegible]	[illegible]	[illegible]
09V34899	CC22-MRSA-IV, Barnim/UK-EMRSA-15	POS	[illegible]	[illegible]	[illegible]	[illegible]	[illegible]	[illegible]	[illegible]	[illegible]	[illegible]	[illegible]
09V35055	CC22-MRSA-IV, Barnim/UK-EMRSA-15	POS	[illegible]	[illegible]	[illegible]	[illegible]	[illegible]	[illegible]	[illegible]	[illegible]	[illegible]	[illegible]
09V35720	CC22-MRSA-IV, Barnim/UK-EMRSA-15	POS	[illegible]	[illegible]	[illegible]	[illegible]	[illegible]	[illegible]	[illegible]	[illegible]	[illegible]	[illegible]
09V36683	CC22-MRSA-IV, Barnim/UK-EMRSA-15	POS	[illegible]	[illegible]	[illegible]	[illegible]	[illegible]	[illegible]	[illegible]	[illegible]	[illegible]	[illegible]
09V37636	CC22-MRSA-IV, Barnim/UK-EMRSA-15	POS	[illegible]	[illegible]	[illegible]	[illegible]	[illegible]	[illegible]	[illegible]	[illegible]	[illegible]	[illegible]
09V37767	CC22-MRSA-IV, Barnim/UK-EMRSA-15	POS	[illegible]	[illegible]	[illegible]	[illegible]	[illegible]	[illegible]	[illegible]	[illegible]	[illegible]	[illegible]
09V39554	CC22-MRSA-IV, Barnim/UK-EMRSA-15	POS	[illegible]	[illegible]	[illegible]	[illegible]	[illegible]	[illegible]	[illegible]	[illegible]	[illegible]	[illegible]
09V40977	CC22-MRSA-IV, Barnim/UK-EMRSA-15	POS	[illegible]	[illegible]	[illegible]	[illegible]	[illegible]	[illegible]	[illegible]	[illegible]	[illegible]	[illegible]
09V42954	CC22-MRSA-IV, Barnim/UK-EMRSA-15	POS	[illegible]	[illegible]	[illegible]	[illegible]	[illegible]	[illegible]	[illegible]	[illegible]	[illegible]	[illegible]
09V44134	CC22-MRSA-IV, Barnim/UK-EMRSA-15	POS	[illegible]	[illegible]	[illegible]	[illegible]	[illegible]	[illegible]	[illegible]	[illegible]	[illegible]	[illegible]
09V46184	CC22-MRSA-IV, Barnim/UK-EMRSA-15	POS	[illegible]	[illegible]	[illegible]	[illegible]	[illegible]	[illegible]	[illegible]	[illegible]	[illegible]	[illegible]
09V47038	CC22-MRSA-IV, Barnim/UK-EMRSA-15	POS	[illegible]	[illegible]	[illegible]	[illegible]	[illegible]	[illegible]	[illegible]	[illegible]	[illegible]	[illegible]
09V47487	CC22-MRSA-IV, Barnim/UK-EMRSA-15	POS	[illegible]	[illegible]	[illegible]	[illegible]	[illegible]	[illegible]	[illegible]	[illegible]	[illegible]	[illegible]
09V48862	CC22-MRSA-IV, Barnim/UK-EMRSA-15	POS	[illegible]	[illegible]	[illegible]	[illegible]	[illegible]	[illegible]	[illegible]	[illegible]	[illegible]	[illegible]
09V32581	CC22-MRSA-V	POS	[illegible]	[illegible]	[illegible]	[illegible]	[illegible]	[illegible]	[illegible]	[illegible]	[illegible]	[illegible]
09V33822	CC45-MRSA-IV, Berlin EMRSA	POS	[illegible]	[illegible]	[illegible]	[illegible]	[illegible]	[illegible]	[illegible]	[illegible]	[illegible]	[illegible]
09V34133	CC45-MRSA-IV, Berlin EMRSA	POS	[illegible]	[illegible]	[illegible]	[illegible]	[illegible]	[illegible]	[illegible]	[illegible]	[illegible]	[illegible]
09V45520	CC45-MRSA-IV, Berlin EMRSA	POS	[illegible]	[illegible]	[illegible]	[illegible]	[illegible]	[illegible]	[illegible]	[illegible]	[illegible]	[illegible]
09V03558	CC398-MRSA-V	POS	[illegible]	[illegible]	[illegible]	[illegible]	[illegible]	[illegible]	[illegible]	[illegible]	[illegible]	[illegible]
09V07762	CC398-MRSA-V	POS	[illegible]	[illegible]	[illegible]	[illegible]	[illegible]	[illegible]	[illegible]	[illegible]	[illegible]	[illegible]
09V29815	CC398-MRSA-V	POS	[illegible]	[illegible]	[illegible]	[illegible]	[illegible]	[illegible]	[illegible]	[illegible]	[illegible]	[illegible]
**2010**												
10V03510	CC1/ST772-MRSA-V [PVL+], Bengal Bay Clone/WA MRSA-60	POS	[illegible]	[illegible]	[illegible]	[illegible]	[illegible]	[illegible]	[illegible]	[illegible]	[illegible]	[illegible]
10V31557	CC1-MRSA-IV, WA MRSA-1/57	POS	[illegible]	[illegible]	[illegible]	[illegible]	[illegible]	[illegible]	[illegible]	[illegible]	[illegible]	[illegible]
10V31910	CC1-MRSA-IV, WA MRSA-1/57	POS	[illegible]	[illegible]	[illegible]	[illegible]	[illegible]	[illegible]	[illegible]	[illegible]	[illegible]	[illegible]
10V01782	CC5/ST228-MRSA-I, South German EMRSA	POS	[illegible]	[illegible]	[illegible]	[illegible]	[illegible]	[illegible]	[illegible]	[illegible]	[illegible]	[illegible]
10V02343	CC5/ST228-MRSA-I, South German EMRSA	POS	[illegible]	[illegible]	[illegible]	[illegible]	[illegible]	[illegible]	[illegible]	[illegible]	[illegible]	[illegible]
10B14561	CC5-MRSA-II, Rhine-Hesse EMRSA/UK-EMRSA-3	POS	[illegible]	[illegible]	[illegible]	[illegible]	[illegible]	[illegible]	[illegible]	[illegible]	[illegible]	[illegible]
10F01042	CC5-MRSA-II, Rhine-Hesse EMRSA/UK-EMRSA-3	POS	[illegible]	[illegible]	[illegible]	[illegible]	[illegible]	[illegible]	[illegible]	[illegible]	[illegible]	[illegible]
10U14746	CC5-MRSA-II, Rhine-Hesse EMRSA/UK-EMRSA-3	POS	[illegible]	[illegible]	[illegible]	[illegible]	[illegible]	[illegible]	[illegible]	[illegible]	[illegible]	[illegible]
10V01913	CC5-MRSA-II, Rhine-Hesse EMRSA/UK-EMRSA-3	POS	[illegible]	[illegible]	[illegible]	[illegible]	[illegible]	[illegible]	[illegible]	[illegible]	[illegible]	[illegible]
10V03216	CC5-MRSA-II, Rhine-Hesse EMRSA/UK-EMRSA-3	POS	[illegible]	[illegible]	[illegible]	[illegible]	[illegible]	[illegible]	[illegible]	[illegible]	[illegible]	[illegible]
10V03727	CC5-MRSA-II, Rhine-Hesse EMRSA/UK-EMRSA-3	POS	[illegible]	[illegible]	[illegible]	[illegible]	[illegible]	[illegible]	[illegible]	[illegible]	[illegible]	[illegible]
10V03824	CC5-MRSA-II, Rhine-Hesse EMRSA/UK-EMRSA-3	POS	[illegible]	[illegible]	[illegible]	[illegible]	[illegible]	[illegible]	[illegible]	[illegible]	[illegible]	[illegible]
10V08796	CC5-MRSA-II, Rhine-Hesse EMRSA/UK-EMRSA-3	POS	[illegible]	[illegible]	[illegible]	[illegible]	[illegible]	[illegible]	[illegible]	[illegible]	[illegible]	[illegible]
10V09487	CC5-MRSA-II, Rhine-Hesse EMRSA/UK-EMRSA-3	POS	[illegible]	[illegible]	[illegible]	[illegible]	[illegible]	[illegible]	[illegible]	[illegible]	[illegible]	[illegible]
10V10616	CC5-MRSA-II, Rhine-Hesse EMRSA/UK-EMRSA-3	POS	[illegible]	[illegible]	[illegible]	[illegible]	[illegible]	[illegible]	[illegible]	[illegible]	[illegible]	[illegible]
10V10617	CC5-MRSA-II, Rhine-Hesse EMRSA/UK-EMRSA-3	POS	[illegible]	[illegible]	[illegible]	[illegible]	[illegible]	[illegible]	[illegible]	[illegible]	[illegible]	[illegible]
10V12678	CC5-MRSA-II, Rhine-Hesse EMRSA/UK-EMRSA-3	POS	[illegible]	[illegible]	[illegible]	[illegible]	[illegible]	[illegible]	[illegible]	[illegible]	[illegible]	[illegible]
10V13218	CC5-MRSA-II, Rhine-Hesse EMRSA/UK-EMRSA-3	POS	[illegible]	[illegible]	[illegible]	[illegible]	[illegible]	[illegible]	[illegible]	[illegible]	[illegible]	[illegible]
10V13638	CC5-MRSA-II, Rhine-Hesse EMRSA/UK-EMRSA-3	POS	[illegible]	[illegible]	[illegible]	[illegible]	[illegible]	[illegible]	[illegible]	[illegible]	[illegible]	[illegible]
10V14799	CC5-MRSA-II, Rhine-Hesse EMRSA/UK-EMRSA-3	POS	[illegible]	[illegible]	[illegible]	[illegible]	[illegible]	[illegible]	[illegible]	[illegible]	[illegible]	[illegible]
10V23825	CC5-MRSA-II, Rhine-Hesse EMRSA/UK-EMRSA-3	POS	[illegible]	[illegible]	[illegible]	[illegible]	[illegible]	[illegible]	[illegible]	[illegible]	[illegible]	[illegible]
10V31630	CC5-MRSA-II, Rhine-Hesse EMRSA/UK-EMRSA-3	POS	[illegible]	[illegible]	[illegible]	[illegible]	[illegible]	[illegible]	[illegible]	[illegible]	[illegible]	[illegible]
10V35386	CC5-MRSA-II, Rhine-Hesse EMRSA/UK-EMRSA-3	POS	[illegible]	[illegible]	[illegible]	[illegible]	[illegible]	[illegible]	[illegible]	[illegible]	[illegible]	[illegible]
10V37507	CC5-MRSA-II, Rhine-Hesse EMRSA/UK-EMRSA-3	POS	[illegible]	[illegible]	[illegible]	[illegible]	[illegible]	[illegible]	[illegible]	[illegible]	[illegible]	[illegible]
10V38321	CC5-MRSA-II, Rhine-Hesse EMRSA/UK-EMRSA-3	POS	[illegible]	[illegible]	[illegible]	[illegible]	[illegible]	[illegible]	[illegible]	[illegible]	[illegible]	[illegible]
10V40861	CC5-MRSA-II, Rhine-Hesse EMRSA/UK-EMRSA-3	POS	[illegible]	[illegible]	[illegible]	[illegible]	[illegible]	[illegible]	[illegible]	[illegible]	[illegible]	[illegible]
10V41383	CC5-MRSA-II, Rhine-Hesse EMRSA/UK-EMRSA-3	POS	[illegible]	[illegible]	[illegible]	[illegible]	[illegible]	[illegible]	[illegible]	[illegible]	[illegible]	[illegible]
10V45069	CC5-MRSA-II, Rhine-Hesse EMRSA/UK-EMRSA-3	POS	[illegible]	[illegible]	[illegible]	[illegible]	[illegible]	[illegible]	[illegible]	[illegible]	[illegible]	[illegible]
10V01508	CC80-MRSA-IV [PVL+], European caMRSA Clone	POS	[illegible]	[illegible]	[illegible]	[illegible]	[illegible]	[illegible]	[illegible]	[illegible]	[illegible]	[illegible]
10V34757	CC80-MRSA-IV [PVL+], European caMRSA Clone	POS	[illegible]	[illegible]	[illegible]	[illegible]	[illegible]	[illegible]	[illegible]	[illegible]	[illegible]	[illegible]
10B06681	CC22-MRSA-IV, Barnim/UK-EMRSA-15	POS	[illegible]	[illegible]	[illegible]	[illegible]	[illegible]	[illegible]	[illegible]	[illegible]	[illegible]	[illegible]
10F00384	CC22-MRSA-IV, Barnim/UK-EMRSA-15	POS	[illegible]	[illegible]	[illegible]	[illegible]	[illegible]	[illegible]	[illegible]	[illegible]	[illegible]	[illegible]
10F00978	CC22-MRSA-IV, Barnim/UK-EMRSA-15	POS	[illegible]	[illegible]	[illegible]	[illegible]	[illegible]	[illegible]	[illegible]	[illegible]	[illegible]	[illegible]
10F02923	CC22-MRSA-IV, Barnim/UK-EMRSA-15	POS	[illegible]	[illegible]	[illegible]	[illegible]	[illegible]	[illegible]	[illegible]	[illegible]	[illegible]	[illegible]
10-Hygiene	CC22-MRSA-IV, Barnim/UK-EMRSA-15	POS	[illegible]	[illegible]	[illegible]	[illegible]	[illegible]	[illegible]	[illegible]	[illegible]	[illegible]	[illegible]
10U02156	CC22-MRSA-IV, Barnim/UK-EMRSA-15	POS	[illegible]	[illegible]	[illegible]	[illegible]	[illegible]	[illegible]	[illegible]	[illegible]	[illegible]	[illegible]
10U16289	CC22-MRSA-IV, Barnim/UK-EMRSA-15	POS	[illegible]	[illegible]	[illegible]	[illegible]	[illegible]	[illegible]	[illegible]	[illegible]	[illegible]	[illegible]
10V01346	CC22-MRSA-IV, Barnim/UK-EMRSA-15	POS	[illegible]	[illegible]	[illegible]	[illegible]	[illegible]	[illegible]	[illegible]	[illegible]	[illegible]	[illegible]
10V03365	CC22-MRSA-IV, Barnim/UK-EMRSA-15	POS	[illegible]	[illegible]	[illegible]	[illegible]	[illegible]	[illegible]	[illegible]	[illegible]	[illegible]	[illegible]
10V03540	CC22-MRSA-IV, Barnim/UK-EMRSA-15	POS	[illegible]	[illegible]	[illegible]	[illegible]	[illegible]	[illegible]	[illegible]	[illegible]	[illegible]	[illegible]
10V03819	CC22-MRSA-IV, Barnim/UK-EMRSA-15	POS	[illegible]	[illegible]	[illegible]	[illegible]	[illegible]	[illegible]	[illegible]	[illegible]	[illegible]	[illegible]
10V04495	CC22-MRSA-IV, Barnim/UK-EMRSA-15	POS	[illegible]	[illegible]	[illegible]	[illegible]	[illegible]	[illegible]	[illegible]	[illegible]	[illegible]	[illegible]
10V04498	CC22-MRSA-IV, Barnim/UK-EMRSA-15	POS	[illegible]	[illegible]	[illegible]	[illegible]	[illegible]	[illegible]	[illegible]	[illegible]	[illegible]	[illegible]
10V04625	CC22-MRSA-IV, Barnim/UK-EMRSA-15	POS	[illegible]	[illegible]	[illegible]	[illegible]	[illegible]	[illegible]	[illegible]	[illegible]	[illegible]	[illegible]
10V04685	CC22-MRSA-IV, Barnim/UK-EMRSA-15	POS	[illegible]	[illegible]	[illegible]	[illegible]	[illegible]	[illegible]	[illegible]	[illegible]	[illegible]	[illegible]
10V05305	CC22-MRSA-IV, Barnim/UK-EMRSA-15	POS	[illegible]	[illegible]	[illegible]	[illegible]	[illegible]	[illegible]	[illegible]	[illegible]	[illegible]	[illegible]
10V05474	CC22-MRSA-IV, Barnim/UK-EMRSA-15	POS	[illegible]	[illegible]	[illegible]	[illegible]	[illegible]	[illegible]	[illegible]	[illegible]	[illegible]	[illegible]
10V05553	CC22-MRSA-IV, Barnim/UK-EMRSA-15	POS	[illegible]	[illegible]	[illegible]	[illegible]	[illegible]	[illegible]	[illegible]	[illegible]	[illegible]	[illegible]
10V06864	CC22-MRSA-IV, Barnim/UK-EMRSA-15	POS	[illegible]	[illegible]	[illegible]	[illegible]	[illegible]	[illegible]	[illegible]	[illegible]	[illegible]	[illegible]
10V09545	CC22-MRSA-IV, Barnim/UK-EMRSA-15	POS	[illegible]	[illegible]	[illegible]	[illegible]	[illegible]	[illegible]	[illegible]	[illegible]	[illegible]	[illegible]
10V11922	CC22-MRSA-IV, Barnim/UK-EMRSA-15	POS	[illegible]	[illegible]	[illegible]	[illegible]	[illegible]	[illegible]	[illegible]	[illegible]	[illegible]	[illegible]
10V12801	CC22-MRSA-IV, Barnim/UK-EMRSA-15	POS	[illegible]	[illegible]	[illegible]	[illegible]	[illegible]	[illegible]	[illegible]	[illegible]	[illegible]	[illegible]
10V15165	CC22-MRSA-IV, Barnim/UK-EMRSA-15	POS	[illegible]	[illegible]	[illegible]	[illegible]	[illegible]	[illegible]	[illegible]	[illegible]	[illegible]	[illegible]
10V18022	CC22-MRSA-IV, Barnim/UK-EMRSA-15	POS	[illegible]	[illegible]	[illegible]	[illegible]	[illegible]	[illegible]	[illegible]	[illegible]	[illegible]	[illegible]
10V21655	CC22-MRSA-IV, Barnim/UK-EMRSA-15	POS	[illegible]	[illegible]	[illegible]	[illegible]	[illegible]	[illegible]	[illegible]	[illegible]	[illegible]	[illegible]
10V21798	CC22-MRSA-IV, Barnim/UK-EMRSA-15	POS	[illegible]	[illegible]	[illegible]	[illegible]	[illegible]	[illegible]	[illegible]	[illegible]	[illegible]	[illegible]
10V23795	CC22-MRSA-IV, Barnim/UK-EMRSA-15	POS	[illegible]	[illegible]	[illegible]	[illegible]	[illegible]	[illegible]	[illegible]	[illegible]	[illegible]	[illegible]
10V25861	CC22-MRSA-IV, Barnim/UK-EMRSA-15	POS	[illegible]	[illegible]	[illegible]	[illegible]	[illegible]	[illegible]	[illegible]	[illegible]	[illegible]	[illegible]
10V26165	CC22-MRSA-IV, Barnim/UK-EMRSA-15	POS	[illegible]	[illegible]	[illegible]	[illegible]	[illegible]	[illegible]	[illegible]	[illegible]	[illegible]	[illegible]
10V26326	CC22-MRSA-IV, Barnim/UK-EMRSA-15	POS	[illegible]	[illegible]	[illegible]	[illegible]	[illegible]	[illegible]	[illegible]	[illegible]	[illegible]	[illegible]
10V29967	CC22-MRSA-IV, Barnim/UK-EMRSA-15	POS	[illegible]	[illegible]	[illegible]	[illegible]	[illegible]	[illegible]	[illegible]	[illegible]	[illegible]	[illegible]
10V30523	CC22-MRSA-IV, Barnim/UK-EMRSA-15	POS	[illegible]	[illegible]	[illegible]	[illegible]	[illegible]	[illegible]	[illegible]	[illegible]	[illegible]	[illegible]
10V31025	CC22-MRSA-IV, Barnim/UK-EMRSA-15	POS	[illegible]	[illegible]	[illegible]	[illegible]	[illegible]	[illegible]	[illegible]	[illegible]	[illegible]	[illegible]
10V31056	CC22-MRSA-IV, Barnim/UK-EMRSA-15	POS	[illegible]	[illegible]	[illegible]	[illegible]	[illegible]	[illegible]	[illegible]	[illegible]	[illegible]	[illegible]
10V32564	CC22-MRSA-IV, Barnim/UK-EMRSA-15	POS	[illegible]	[illegible]	[illegible]	[illegible]	[illegible]	[illegible]	[illegible]	[illegible]	[illegible]	[illegible]
10V32855	CC22-MRSA-IV, Barnim/UK-EMRSA-15	POS	[illegible]	[illegible]	[illegible]	[illegible]	[illegible]	[illegible]	[illegible]	[illegible]	[illegible]	[illegible]
10V33616	CC22-MRSA-IV, Barnim/UK-EMRSA-15	POS	[illegible]	[illegible]	[illegible]	[illegible]	[illegible]	[illegible]	[illegible]	[illegible]	[illegible]	[illegible]
10V34392	CC22-MRSA-IV, Barnim/UK-EMRSA-15	POS	[illegible]	[illegible]	[illegible]	[illegible]	[illegible]	[illegible]	[illegible]	[illegible]	[illegible]	[illegible]
10V35980	CC22-MRSA-IV, Barnim/UK-EMRSA-15	POS	[illegible]	[illegible]	[illegible]	[illegible]	[illegible]	[illegible]	[illegible]	[illegible]	[illegible]	[illegible]
10V37062	CC22-MRSA-IV, Barnim/UK-EMRSA-15	POS	[illegible]	[illegible]	[illegible]	[illegible]	[illegible]	[illegible]	[illegible]	[illegible]	[illegible]	[illegible]
10V37408	CC22-MRSA-IV, Barnim/UK-EMRSA-15	POS	[illegible]	[illegible]	[illegible]	[illegible]	[illegible]	[illegible]	[illegible]	[illegible]	[illegible]	[illegible]
10V37820	CC22-MRSA-IV, Barnim/UK-EMRSA-15	POS	[illegible]	[illegible]	[illegible]	[illegible]	[illegible]	[illegible]	[illegible]	[illegible]	[illegible]	[illegible]
10V38669	CC22-MRSA-IV, Barnim/UK-EMRSA-15	POS	[illegible]	[illegible]	[illegible]	[illegible]	[illegible]	[illegible]	[illegible]	[illegible]	[illegible]	[illegible]
10V38699	CC22-MRSA-IV, Barnim/UK-EMRSA-15	POS	[illegible]	[illegible]	[illegible]	[illegible]	[illegible]	[illegible]	[illegible]	[illegible]	[illegible]	[illegible]
10V39014	CC22-MRSA-IV, Barnim/UK-EMRSA-15	POS	[illegible]	[illegible]	[illegible]	[illegible]	[illegible]	[illegible]	[illegible]	[illegible]	[illegible]	[illegible]
10V39280	CC22-MRSA-IV, Barnim/UK-EMRSA-15	POS	[illegible]	[illegible]	[illegible]	[illegible]	[illegible]	[illegible]	[illegible]	[illegible]	[illegible]	[illegible]
10V39426	CC22-MRSA-IV, Barnim/UK-EMRSA-15	POS	[illegible]	[illegible]	[illegible]	[illegible]	[illegible]	[illegible]	[illegible]	[illegible]	[illegible]	[illegible]
10V41061	CC22-MRSA-IV, Barnim/UK-EMRSA-15	POS	[illegible]	[illegible]	[illegible]	[illegible]	[illegible]	[illegible]	[illegible]	[illegible]	[illegible]	[illegible]
10V44488	CC22-MRSA-IV, Barnim/UK-EMRSA-15	POS	[illegible]	[illegible]	[illegible]	[illegible]	[illegible]	[illegible]	[illegible]	[illegible]	[illegible]	[illegible]
10V44924	CC22-MRSA-IV, Barnim/UK-EMRSA-15	POS	[illegible]	[illegible]	[illegible]	[illegible]	[illegible]	[illegible]	[illegible]	[illegible]	[illegible]	[illegible]
10V44966	CC22-MRSA-IV, Barnim/UK-EMRSA-15	POS	[illegible]	[illegible]	[illegible]	[illegible]	[illegible]	[illegible]	[illegible]	[illegible]	[illegible]	[illegible]
10V27260	CC22-MRSA-V	POS	[illegible]	[illegible]	[illegible]	[illegible]	[illegible]	[illegible]	[illegible]	[illegible]	[illegible]	[illegible]
10V15136	CC45-MRSA-IV, Berlin EMRSA	POS	[illegible]	[illegible]	[illegible]	[illegible]	[illegible]	[illegible]	[illegible]	[illegible]	[illegible]	[illegible]
10V29067	CC45-MRSA-IV, Berlin EMRSA	POS	[illegible]	[illegible]	[illegible]	[illegible]	[illegible]	[illegible]	[illegible]	[illegible]	[illegible]	[illegible]
10V40116	CC45-MRSA-IV, Berlin EMRSA	POS	[illegible]	[illegible]	[illegible]	[illegible]	[illegible]	[illegible]	[illegible]	[illegible]	[illegible]	[illegible]
10V10412	CC80-MRSA-IV [PVL+], European caMRSA Clone	POS	[illegible]	[illegible]	[illegible]	[illegible]	[illegible]	[illegible]	[illegible]	[illegible]	[illegible]	[illegible]
10V25105	CC398-MRSA-V	POS	[illegible]	[illegible]	[illegible]	[illegible]	[illegible]	[illegible]	[illegible]	[illegible]	[illegible]	[illegible]

ISOLATE:	VIRULENCE : EXFOL.TOXINS			VIRULENCE : EPITHEL. DIFF. INHIB			VIRULENCE : ACME LOCUS				VIRULENCE : PROTEASES										VIRULENCE : STAPHYLOCOCCAL SUPERANTIGEN/ENTEROTOXIN-LIKE GENES (SET/SSL)						
	exfoliative toxin serotype A	exfoliative toxin serotype B	exfoliative toxin D	epidermal cell differen-tiation inhibitor	epidermal cell differen-tiation inhibitor B	epidermal cell differen-tiation inhibitor C	ACME-locus	ACME-locus: ornithincarb-amoyltrans-ferase	ACME-locus: carbamat-kinase	ACME-locus: arginine/orni-thine-antiporter	aureolysin			serin- protease A	serin- protease B	serin- protease E	glutamylendopeptidase	staphopain B, protease	staphopain A (staphylopain A), protease		staphyl. exotoxin-like protein	staphylococcal superantigen-like protein 1					
	etA	etB	etD	edinA	edinB	edinC	arcA-SCC	arcB-SCC	arcC-SCC	arcD-SCC	aur (consensus)	aur (other than MRSA252)	aur (MRSA252)	splA	splB	splE	sspA	sspB	sspP (consensus)	sspP (other than ST93)	setC	ssl01/set6_probe1_11	ssl01/set6_probe2_11	ssl01/set6_probe1_12	ssl01/set6_probe2_12	ssl01/set6_probe4_11	ssl01/set6_probeRF122
09V32405	NEG	NEG	NEG	NEG	NEG	NEG	NEG	NEG	NEG	NEG	POS	POS	NEG	NEG	NEG	NEG	POS	POS	POS	POS	POS	POS	NEG	NEG	NEG	POS	NEG
09V47486	NEG	NEG	NEG	NEG	NEG	NEG	NEG	NEG	NEG	NEG	POS	POS	NEG	POS	POS	NEG	POS	POS	POS	POS	POS	NEG	POS	NEG	POS	NEG	NEG
09V42357	NEG	NEG	NEG	NEG	NEG	NEG	NEG	NEG	NEG	NEG	POS	POS	NEG	POS	POS	NEG	POS	POS	POS	POS	POS	POS	NEG	POS	NEG	POS	NEG
09F01028	NEG	NEG	NEG	NEG	NEG	NEG	NEG	NEG	NEG	NEG	POS	POS	NEG	POS	POS	NEG	POS	POS	POS	POS	POS	POS	NEG	POS	NEG	POS	NEG
09F01040	NEG	NEG	NEG	NEG	NEG	NEG	NEG	NEG	NEG	NEG	POS	POS	NEG	POS	POS	NEG	POS	POS	POS	POS	POS	POS	NEG	POS	NEG	POS	NEG
09F03018	NEG	NEG	NEG	NEG	NEG	NEG	NEG	NEG	NEG	NEG	POS	POS	NEG	POS	POS	NEG	POS	POS	POS	POS	POS	POS	NEG	POS	NEG	POS	NEG
09F03619	NEG	NEG	NEG	NEG	NEG	NEG	NEG	NEG	NEG	NEG	POS	POS	NEG	POS	POS	NEG	POS	POS	POS	POS	POS	POS	NEG	POS	NEG	AMB	NEG
09V01921	NEG	NEG	NEG	NEG	NEG	NEG	NEG	NEG	NEG	NEG	POS	POS	NEG	POS	POS	NEG	POS	POS	POS	POS	POS	POS	NEG	POS	NEG	POS	NEG
09V04547	NEG	[illegible]	[illegible]	[illegible]	[illegible]	[illegible]	[illegible]	[illegible]	[illegible]	[illegible]	[illegible]	[illegible]	[illegible]	[illegible]	[illegible]	[illegible]	[illegible]	[illegible]	[illegible]	[illegible]	[illegible]	[illegible]	[illegible]	[illegible]	[illegible]	[illegible]	[illegible]
09V05744	NEG	[illegible]	[illegible]	[illegible]	[illegible]	[illegible]	[illegible]	[illegible]	[illegible]	[illegible]	[illegible]	[illegible]	[illegible]	[illegible]	[illegible]	[illegible]	[illegible]	[illegible]	[illegible]	[illegible]	[illegible]	[illegible]	[illegible]	[illegible]	[illegible]	[illegible]	[illegible]
09V07448	NEG	[illegible]	[illegible]	[illegible]	[illegible]	[illegible]	[illegible]	[illegible]	[illegible]	[illegible]	[illegible]	[illegible]	[illegible]	[illegible]	[illegible]	[illegible]	[illegible]	[illegible]	[illegible]	[illegible]	[illegible]	[illegible]	[illegible]	[illegible]	[illegible]	[illegible]	[illegible]
09V08032	NEG	[illegible]	[illegible]	[illegible]	[illegible]	[illegible]	[illegible]	[illegible]	[illegible]	[illegible]	[illegible]	[illegible]	[illegible]	[illegible]	[illegible]	[illegible]	[illegible]	[illegible]	[illegible]	[illegible]	[illegible]	[illegible]	[illegible]	[illegible]	[illegible]	[illegible]	[illegible]
09V08761	NEG	[illegible]	[illegible]	[illegible]	[illegible]	[illegible]	[illegible]	[illegible]	[illegible]	[illegible]	[illegible]	[illegible]	[illegible]	[illegible]	[illegible]	[illegible]	[illegible]	[illegible]	[illegible]	[illegible]	[illegible]	[illegible]	[illegible]	[illegible]	[illegible]	[illegible]	[illegible]
09V21706	NEG	[illegible]	[illegible]	[illegible]	[illegible]	[illegible]	[illegible]	[illegible]	[illegible]	[illegible]	[illegible]	[illegible]	[illegible]	[illegible]	[illegible]	[illegible]	[illegible]	[illegible]	[illegible]	[illegible]	[illegible]	[illegible]	[illegible]	[illegible]	[illegible]	[illegible]	[illegible]
09V22079	NEG	[illegible]	[illegible]	[illegible]	[illegible]	[illegible]	[illegible]	[illegible]	[illegible]	[illegible]	[illegible]	[illegible]	[illegible]	[illegible]	[illegible]	[illegible]	[illegible]	[illegible]	[illegible]	[illegible]	[illegible]	[illegible]	[illegible]	[illegible]	[illegible]	[illegible]	[illegible]
09V25980	NEG	[illegible]	[illegible]	[illegible]	[illegible]	[illegible]	[illegible]	[illegible]	[illegible]	[illegible]	[illegible]	[illegible]	[illegible]	[illegible]	[illegible]	[illegible]	[illegible]	[illegible]	[illegible]	[illegible]	[illegible]	[illegible]	[illegible]	[illegible]	[illegible]	[illegible]	[illegible]
09V26221	NEG	[illegible]	[illegible]	[illegible]	[illegible]	[illegible]	[illegible]	[illegible]	[illegible]	[illegible]	[illegible]	[illegible]	[illegible]	[illegible]	[illegible]	[illegible]	[illegible]	[illegible]	[illegible]	[illegible]	[illegible]	[illegible]	[illegible]	[illegible]	[illegible]	[illegible]	[illegible]
09V28517	NEG	[illegible]	[illegible]	[illegible]	[illegible]	[illegible]	[illegible]	[illegible]	[illegible]	[illegible]	[illegible]	[illegible]	[illegible]	[illegible]	[illegible]	[illegible]	[illegible]	[illegible]	[illegible]	[illegible]	[illegible]	[illegible]	[illegible]	[illegible]	[illegible]	[illegible]	[illegible]
09V28643	NEG	[illegible]	[illegible]	[illegible]	[illegible]	[illegible]	[illegible]	[illegible]	[illegible]	[illegible]	[illegible]	[illegible]	[illegible]	[illegible]	[illegible]	[illegible]	[illegible]	[illegible]	[illegible]	[illegible]	[illegible]	[illegible]	[illegible]	[illegible]	[illegible]	[illegible]	[illegible]
09V28647	NEG	[illegible]	[illegible]	[illegible]	[illegible]	[illegible]	[illegible]	[illegible]	[illegible]	[illegible]	[illegible]	[illegible]	[illegible]	[illegible]	[illegible]	[illegible]	[illegible]	[illegible]	[illegible]	[illegible]	[illegible]	[illegible]	[illegible]	[illegible]	[illegible]	[illegible]	[illegible]
09V29064 to 10V25105 (all remaining rows, as listed in the table above)	[illegible]	[illegible]	[illegible]	[illegible]	[illegible]	[illegible]	[illegible]	[illegible]	[illegible]	[illegible]	[illegible]	[illegible]	[illegible]	[illegible]	[illegible]	[illegible]	[illegible]	[illegible]	[illegible]	[illegible]	[illegible]	[illegible]	[illegible]	[illegible]	[illegible]	[illegible]	[illegible]

ISOLATE:	STRAIN	ssl02/set7	ssl02/set7 (MRSA252)	ssl03/set8_probe 1	ssl03/set8_probe 2	ssl03/set8 (MRSA252, SAR0424)	ssl04/set9	ssl04/set9 (MRSA252, SAR0425)	ssl05/set3_probe 1	ssl05/set3 (RF122, probe-611)	ssl05/set3_probe 2 (612)	ssl05/set3 (MRSA252)	ssl06/set21	ssl06 (NCTC8325+ MW2)	ssl07/set1	ssl07/set1 (MRSA252)	ssl07/set1 (AF188836)	ssl08/set12_probe 1	ssl08/set12_probe 2	ssl09/set5_probe 1	ssl09/set5_probe 2	ssl09/set5 (MRSA252)	ssl10/set4	ssl10 (RF122)	ssl10/set4 (MRSA252)	ssl11/set2 (COL)	ssl11+set2(Mu50+N315)	ssl11+set2(MW2+MSSA 476)	ssl11/set2 (MRSA252)	setB3	setB3 (MRSA252)	setB2	setB2 (MRSA252)	setB1	cap 1 (total)	capH1	capJ1	capK1	cap 5 (total)	capH5	capJ5	capK5	cap 8 (total)	capH8	capI8	capJ8	capK8	icaA	icaC	icaD	bap
		VIRULENCE : STAPHYLOCOCCAL SUPERANTIGEN/ENTEROTOXIN-LIKE GENES (SET/SSL)																																	CAPSULE- AND BIOFILM-ASSOCIATED GENES																
		staphylococcal superantigen-like protein 2		staphylococcal superantigen-like protein 3			staphylococcal superantigen-like protein 4		staphylococcal superantigen-like protein 5				staphylococcal superantigen-like protein 6		staphylococcal superantigen-like protein 7			staphylococcal superantigen-like protein 8		staphylococcal superantigen-like protein 9			staphylococcal superantigen-like protein 10			staphylococcal superantigene-like protein 11				staphylococcal exotoxin-like protein, second locus					Capsule type 1	capsular poly-saccharide synthesis enzyme	O-antigen poly-merase	capsular poly-saccharide biosyn- thesis protein	Capsule type 5	capsular poly-saccharide synthesis enzyme	O-antigen poly-merase	capsular poly-saccharide biosyn- thesis protein	Capsule type 8	capsular poly-saccharide synthesis enzyme	capsular poly-saccharide biosyn- thesis protein	O-antigen poly-merase	capsular poly-saccharide biosyn- thesis protein	intercellular adhesion protein A	intercellular adhesion protein C	biofilm PIA synthesis protein D	surface protein involved in biofilm formation
**2009**																																																			
09V32405	CC1/ST772-MRSA-V [PVL+], Bengal Bay Clone/WA MRSA-60	POS	NEG	POS	POS	NEG	POS	NEG	POS	POS	AMB	NEG	NEG	NEG	POS	NEG	NEG	POS	POS	POS	POS	NEG	POS	NEG	NEG	NEG	NEG	NEG	NEG	POS	NEG	POS	NEG	POS	NEG	NEG	NEG	NEG	POS	POS	POS	POS	NEG	NEG	NEG	NEG	NEG	POS	POS	POS	NEG
09V47486	CC1-MRSA-IV, WA MRSA-1/57	POS	NEG	POS	POS	NEG	POS	NEG	POS	NEG	POS	NEG	POS	POS	POS	AMB	NEG	POS	POS	POS	POS	NEG	POS	NEG	AMB	NEG	NEG	POS	NEG	POS	NEG	POS	NEG	POS	NEG	NEG	NEG	NEG	NEG	NEG	NEG	NEG	POS	POS	POS	POS	POS	POS	POS	POS	NEG
09V42357	CC5/ST228-MRSA-I, South German EMRSA	POS	NEG	POS	POS	NEG	POS	NEG	POS	NEG	POS	NEG	NEG	NEG	POS	AMB	NEG	POS	POS	POS	POS	NEG	POS	NEG	AMB	NEG	POS	NEG	NEG	POS	NEG	POS	NEG	POS	NEG	NEG	NEG	NEG	POS	POS	POS	POS	NEG	NEG	NEG	NEG	NEG	POS	POS	POS	NEG
09F01028	CC5-MRSA-II, Rhine-Hesse EMRSA/UK-EMRSA-3	POS	NEG	POS	POS	NEG	POS	NEG	POS	AMB	POS	NEG	NEG	NEG	POS	AMB	NEG	POS	POS	POS	POS	NEG	POS	NEG	NEG	NEG	POS	NEG	NEG	POS	NEG	POS	NEG	POS	NEG	NEG	NEG	NEG	POS	POS	POS	POS	NEG	NEG	NEG	NEG	NEG	POS	POS	POS	NEG
09F01040	CC5-MRSA-II, Rhine-Hesse EMRSA/UK-EMRSA-3	POS	NEG	POS	POS	NEG	POS	NEG	POS	AMB	POS	NEG	NEG	NEG	POS	AMB	NEG	POS	POS	POS	POS	NEG	POS	NEG	NEG	NEG	POS	NEG	NEG	POS	NEG	POS	NEG	POS	NEG	NEG	NEG	NEG	POS	POS	POS	POS	NEG	NEG	NEG	NEG	NEG	POS	POS	POS	NEG
09F03018	CC5-MRSA-II, Rhine-Hesse EMRSA/UK-EMRSA-3	POS	AMB	POS	POS	NEG	POS	NEG	POS	AMB	POS	NEG	NEG	NEG	POS	AMB	NEG	POS	POS	POS	POS	NEG	POS	NEG	AMB	NEG	POS	NEG	NEG	POS	NEG	POS	NEG	POS	NEG	NEG	NEG	NEG	POS	POS	POS	POS	NEG	NEG	NEG	NEG	NEG	POS	POS	POS	NEG
09F03619	CC5-MRSA-II, Rhine-Hesse EMRSA/UK-EMRSA-3	POS	NEG	POS	POS	NEG	POS	NEG	POS	NEG	POS	NEG	NEG	NEG	POS	NEG	NEG	POS	POS	POS	POS	NEG	POS	NEG	NEG	NEG	POS	NEG	NEG	POS	NEG	POS	NEG	POS	NEG	NEG	NEG	NEG	POS	POS	POS	POS	NEG	NEG	NEG	NEG	NEG	POS	POS	POS	NEG
[illegible]	[illegible]	[illegible]	[illegible]	[illegible]	[illegible]	[illegible]	[illegible]	[illegible]	[illegible]	[illegible]	[illegible]	[illegible]	[illegible]	[illegible]	[illegible]	[illegible]	[illegible]	[illegible]	[illegible]	[illegible]	[illegible]	[illegible]	[illegible]	[illegible]	[illegible]	[illegible]	[illegible]	[illegible]	[illegible]	[illegible]	[illegible]	[illegible]	[illegible]	[illegible]	[illegible]	[illegible]	[illegible]	[illegible]	[illegible]	[illegible]	[illegible]	[illegible]	[illegible]	[illegible]	[illegible]	[illegible]	[illegible]	[illegible]	[illegible]	[illegible]	[illegible]
**2010**																																																			
[illegible]	[illegible]	[illegible]	[illegible]	[illegible]	[illegible]	[illegible]	[illegible]	[illegible]	[illegible]	[illegible]	[illegible]	[illegible]	[illegible]	[illegible]	[illegible]	[illegible]	[illegible]	[illegible]	[illegible]	[illegible]	[illegible]	[illegible]	[illegible]	[illegible]	[illegible]	[illegible]	[illegible]	[illegible]	[illegible]	[illegible]	[illegible]	[illegible]	[illegible]	[illegible]	[illegible]	[illegible]	[illegible]	[illegible]	[illegible]	[illegible]	[illegible]	[illegible]	[illegible]	[illegible]	[illegible]	[illegible]	[illegible]	[illegible]	[illegible]	[illegible]	[illegible]

ISOLATE:	STRAIN	ADHAESION FACTORS / GENES ENCODING MICROBIAL SURFACE COMPONENTS RECOGNIZING ADHESIVE MATRIX MOLECULES (MSCRAMM GENES)																																															
		bone sialoprotein-binding protein							clumping factor A					clumping factor B					collagen-binding adhesin	cell wall associated fibronectin-binding protein	cell surface elastin binding protein					enolase	fibrinogen binding protein (19 kDa)		fibronectin-binding protein A						fibronectin-binding protein B							Major histocompatibility complex class II analog protein (=Extracellular adherence protein, eap)				Staphylococcus aureus surface protein G			
		bbp (total)	bbp (consensus)	bbp (COL+MW2)	bbp (MRSA252)	bbp (Mu50)	bbp (RF122)	bbp (ST45)	clfA (total)	clfA (consensus)	clfA (COL+RF122)	clfA (MRSA252)	clfA (Mu50+MW2)	clfB (total)	clfB (consensus)	clfB (COL+Mu50)	clfB (MW2)	clfB (RF122)	cna	ebh (consensus)	ebpS (total)	ebpS_probe 612	ebpS_probe 614	ebpS (01-1111)	ebpS (COL)	eno	fib	fib (MRSA252)	fnbA (total)	fnbA (consensus)	fnbA (COL)	fnbA (MRSA252)	fnbA (Mu50+MW2)	fnbA (RF122)	fnbB (total)	fnbB (COL)	fnbB (COL+Mu50+MW2)	fnbB (Mu50)	fnbB (MW2)	fnbB (ST15)	fnbB (ST45-2)	map (total)	map (COL)	map (MRSA252)	map (Mu50+MW2)	sasG (total)	sasG (COL+Mu50)	sasG (MW2)	sasG (other than MRSA252+RF122)
**2009**																																																	
09V32405	CC1/ST772-MRSA-V [PVL+], Bengal Bay Clone/WA MRSA-60	POS	POS	NEG	NEG	POS	NEG	NEG	POS	POS	NEG	NEG	POS	POS	POS	NEG	NEG	NEG	POS	POS	POS	POS	POS	NEG	NEG	POS	POS	NEG	POS	POS	NEG	NEG	POS	NEG	POS	NEG	POS	NEG	NEG	AMB	NEG	POS	NEG	NEG	POS	POS	NEG	POS	POS
09V47486	CC1-MRSA-IV, WA MRSA-1/57	POS	POS	POS	NEG	NEG	NEG	NEG	POS	POS	NEG	NEG	POS	POS	POS	NEG	POS	AMB	POS	POS	POS	POS	POS	NEG	NEG	POS	POS	NEG	POS	POS	NEG	NEG	POS	NEG	POS	NEG	AMB	NEG	POS	NEG	NEG	POS	POS	NEG	POS	POS	NEG	POS	POS
09V42357	CC5/ST228-MRSA-I, South German EMRSA	POS	POS	NEG	NEG	POS	NEG	NEG	POS	POS	NEG	NEG	POS	POS	POS	NEG	NEG	NEG	NEG	POS	POS	POS	POS	NEG	NEG	POS	POS	NEG	POS	POS	NEG	NEG	POS	NEG	NEG	NEG	NEG	NEG	NEG	NEG	NEG	POS	POS	NEG	POS	POS	POS	NEG	POS
09F01028	CC5-MRSA-II, Rhine-Hesse EMRSA/UK-EMRSA-3	POS	POS	NEG	NEG	POS	NEG	NEG	POS	POS	NEG	NEG	POS	POS	POS	POS	NEG	NEG	NEG	POS	POS	AMB	POS	NEG	NEG	POS	POS	NEG	POS	POS	NEG	NEG	POS	NEG	NEG	NEG	NEG	NEG	NEG	NEG	NEG	POS	POS	NEG	POS	POS	POS	NEG	POS
09F01040	CC5-MRSA-II, Rhine-Hesse EMRSA/UK-EMRSA-3	POS	POS	NEG	NEG	POS	NEG	NEG	POS	POS	NEG	NEG	POS	POS	POS	POS	NEG	NEG	NEG	POS	POS	POS	POS	NEG	NEG	POS	POS	NEG	POS	POS	NEG	NEG	POS	NEG	NEG	NEG	NEG	NEG	NEG	NEG	NEG	POS	POS	NEG	POS	POS	POS	NEG	POS
09F03018	CC5-MRSA-II, Rhine-Hesse EMRSA/UK-EMRSA-3	POS	POS	NEG	NEG	POS	NEG	NEG	POS	POS	NEG	NEG	POS	POS	POS	POS	NEG	NEG	NEG	POS	POS	POS	POS	NEG	NEG	POS	POS	NEG	POS	POS	NEG	NEG	POS	NEG	NEG	NEG	NEG	NEG	NEG	NEG	NEG	POS	POS	NEG	POS	POS	POS	NEG	POS
09F03619	CC5-MRSA-II, Rhine-Hesse EMRSA/UK-EMRSA-3	POS	POS	NEG	NEG	POS	NEG	NEG	POS	POS	NEG	NEG	POS	POS	POS	POS	NEG	NEG	NEG	POS	POS	AMB	POS	NEG	NEG	POS	POS	NEG	POS	POS	NEG	NEG	POS	NEG	POS	NEG	POS	AMB	NEG	NEG	NEG	POS	NEG	NEG	POS	POS	POS	NEG	POS
09V01921	CC5-MRSA-II, Rhine-Hesse EMRSA/UK-EMRSA-3	POS	POS	NEG	NEG	POS	NEG	NEG	POS	POS	NEG	NEG	POS	POS	POS	POS	NEG	NEG	NEG	POS	POS	POS	POS	NEG	NEG	POS	POS	NEG	POS	POS	NEG	NEG	POS	NEG	POS	NEG	POS	AMB	NEG	NEG	NEG	POS	POS	NEG	POS	POS	POS	NEG	POS

ISOLATE:	STRAIN
09V04547	CC5-MRSA-II, Rhine-Hesse EMRSA/UK-EMRSA-3
09V05744	CC5-MRSA-II, Rhine-Hesse EMRSA/UK-EMRSA-3
09V07448	CC5-MRSA-II, Rhine-Hesse EMRSA/UK-EMRSA-3
09V08032	CC5-MRSA-II, Rhine-Hesse EMRSA/UK-EMRSA-3
09V08761	CC5-MRSA-II, Rhine-Hesse EMRSA/UK-EMRSA-3
09V21706	CC5-MRSA-II, Rhine-Hesse EMRSA/UK-EMRSA-3
09V22079	CC5-MRSA-II, Rhine-Hesse EMRSA/UK-EMRSA-3
09V25980	CC5-MRSA-II, Rhine-Hesse EMRSA/UK-EMRSA-3
09V26221	CC5-MRSA-II, Rhine-Hesse EMRSA/UK-EMRSA-3
09V28517	CC5-MRSA-II, Rhine-Hesse EMRSA/UK-EMRSA-3
09V28643	CC5-MRSA-II, Rhine-Hesse EMRSA/UK-EMRSA-3
09V28647	CC5-MRSA-II, Rhine-Hesse EMRSA/UK-EMRSA-3
09V29064	CC5-MRSA-II, Rhine-Hesse EMRSA/UK-EMRSA-3
09V30464	CC5-MRSA-II, Rhine-Hesse EMRSA/UK-EMRSA-3
09V30701	CC5-MRSA-II, Rhine-Hesse EMRSA/UK-EMRSA-3
09V35418	CC5-MRSA-II, Rhine-Hesse EMRSA/UK-EMRSA-3
09V37115	CC5-MRSA-II, Rhine-Hesse EMRSA/UK-EMRSA-3
09V37466	CC5-MRSA-II, Rhine-Hesse EMRSA/UK-EMRSA-3
09V40100	CC5-MRSA-II, Rhine-Hesse EMRSA/UK-EMRSA-3
09V41542	CC5-MRSA-II, Rhine-Hesse EMRSA/UK-EMRSA-3
09V42595	CC5-MRSA-II, Rhine-Hesse EMRSA/UK-EMRSA-3
09V46894	CC5-MRSA-II, Rhine-Hesse EMRSA/UK-EMRSA-3
09V47383	CC5-MRSA-II, Rhine-Hesse EMRSA/UK-EMRSA-3
09V47393	CC5-MRSA-II, Rhine-Hesse EMRSA/UK-EMRSA-3
09V34574	CC5-MRSA-IV, Paediatric clone
09F00871	CC8/ST72-MRSA-IV, USA700
09V20594	CC8-MRSA-IV, USA300
09V28877	CC8-MRSA-IV, USA300
09F00437	CC22-MRSA-IV, Barnim/UK-EMRSA-15
09F01062	CC22-MRSA-IV, Barnim/UK-EMRSA-15
09U06692	CC22-MRSA-IV, Barnim/UK-EMRSA-15
09V04550	CC22-MRSA-IV, Barnim/UK-EMRSA-15
09V05293	CC22-MRSA-IV, Barnim/UK-EMRSA-15
09V05937	CC22-MRSA-IV, Barnim/UK-EMRSA-15
09V05943	CC22-MRSA-IV, Barnim/UK-EMRSA-15
09V05947	CC22-MRSA-IV, Barnim/UK-EMRSA-15
09V06275	CC22-MRSA-IV, Barnim/UK-EMRSA-15
09V08495	CC22-MRSA-IV, Barnim/UK-EMRSA-15
09V12464	CC22-MRSA-IV, Barnim/UK-EMRSA-15
09V15150	CC22-MRSA-IV, Barnim/UK-EMRSA-15
09V16014	CC22-MRSA-IV, Barnim/UK-EMRSA-15
09V19884	CC22-MRSA-IV, Barnim/UK-EMRSA-15
09V24370	CC22-MRSA-IV, Barnim/UK-EMRSA-15
09V25431	CC22-MRSA-IV, Barnim/UK-EMRSA-15
09V27150	CC22-MRSA-IV, Barnim/UK-EMRSA-15
09V27748	CC22-MRSA-IV, Barnim/UK-EMRSA-15
09V28011	CC22-MRSA-IV, Barnim/UK-EMRSA-15
09V28179	CC22-MRSA-IV, Barnim/UK-EMRSA-15
09V28558	CC22-MRSA-IV, Barnim/UK-EMRSA-15
09V28681	CC22-MRSA-IV, Barnim/UK-EMRSA-15
09V29022	CC22-MRSA-IV, Barnim/UK-EMRSA-15
09V30408	CC22-MRSA-IV, Barnim/UK-EMRSA-15
09V30634	CC22-MRSA-IV, Barnim/UK-EMRSA-15
09V31667	CC22-MRSA-IV, Barnim/UK-EMRSA-15
09V31882	CC22-MRSA-IV, Barnim/UK-EMRSA-15
09V32211	CC22-MRSA-IV, Barnim/UK-EMRSA-15
09V34899	CC22-MRSA-IV, Barnim/UK-EMRSA-15
09V35055	CC22-MRSA-IV, Barnim/UK-EMRSA-15
09V35720	CC22-MRSA-IV, Barnim/UK-EMRSA-15
09V36683	CC22-MRSA-IV, Barnim/UK-EMRSA-15
09V37636	CC22-MRSA-IV, Barnim/UK-EMRSA-15
09V37767	CC22-MRSA-IV, Barnim/UK-EMRSA-15
09V39554	CC22-MRSA-IV, Barnim/UK-EMRSA-15
09V40977	CC22-MRSA-IV, Barnim/UK-EMRSA-15
09V42954	CC22-MRSA-IV, Barnim/UK-EMRSA-15
09V44134	CC22-MRSA-IV, Barnim/UK-EMRSA-15
09V46184	CC22-MRSA-IV, Barnim/UK-EMRSA-15
09V47038	CC22-MRSA-IV, Barnim/UK-EMRSA-15
09V47487	CC22-MRSA-IV, Barnim/UK-EMRSA-15
09V48862	CC22-MRSA-IV, Barnim/UK-EMRSA-15
09V32581	CC22-MRSA-V
09V33822	CC45-MRSA-IV, Berlin EMRSA
09V34133	CC45-MRSA-IV, Berlin EMRSA
09V45520	CC45-MRSA-IV, Berlin EMRSA
09V03558	CC398-MRSA-V
09V07762	CC398-MRSA-V
09V29815	CC398-MRSA-V
**2010**	
10V03510	CC1/ST772-MRSA-V [PVL+], Bengal Bay Clone/WA MRSA-60
10V31557	CC1-MRSA-IV, WA MRSA-1/57
10V31910	CC1-MRSA-IV, WA MRSA-1/57
10V01782	CC5/ST228-MRSA-I, South German EMRSA
10V02343	CC5/ST228-MRSA-I, South German EMRSA
10B14561	CC5-MRSA-II, Rhine-Hesse EMRSA/UK-EMRSA-3
10F01042	CC5-MRSA-II, Rhine-Hesse EMRSA/UK-EMRSA-3
10U14746	CC5-MRSA-II, Rhine-Hesse EMRSA/UK-EMRSA-3
10V01913	CC5-MRSA-II, Rhine-Hesse EMRSA/UK-EMRSA-3
10V03216	CC5-MRSA-II, Rhine-Hesse EMRSA/UK-EMRSA-3
10V03727	CC5-MRSA-II, Rhine-Hesse EMRSA/UK-EMRSA-3
10V03824	CC5-MRSA-II, Rhine-Hesse EMRSA/UK-EMRSA-3
10V08796	CC5-MRSA-II, Rhine-Hesse EMRSA/UK-EMRSA-3
10V09487	CC5-MRSA-II, Rhine-Hesse EMRSA/UK-EMRSA-3
10V10616	CC5-MRSA-II, Rhine-Hesse EMRSA/UK-EMRSA-3
10V10617	CC5-MRSA-II, Rhine-Hesse EMRSA/UK-EMRSA-3
10V12678	CC5-MRSA-II, Rhine-Hesse EMRSA/UK-EMRSA-3
10V13218	CC5-MRSA-II, Rhine-Hesse EMRSA/UK-EMRSA-3
10V13638	CC5-MRSA-II, Rhine-Hesse EMRSA/UK-EMRSA-3
10V14799	CC5-MRSA-II, Rhine-Hesse EMRSA/UK-EMRSA-3
10V23825	CC5-MRSA-II, Rhine-Hesse EMRSA/UK-EMRSA-3
10V31630	CC5-MRSA-II, Rhine-Hesse EMRSA/UK-EMRSA-3
10V35386	CC5-MRSA-II, Rhine-Hesse EMRSA/UK-EMRSA-3
10V37507	CC5-MRSA-II, Rhine-Hesse EMRSA/UK-EMRSA-3
10V38321	CC5-MRSA-II, Rhine-Hesse EMRSA/UK-EMRSA-3
10V40861	CC5-MRSA-II, Rhine-Hesse EMRSA/UK-EMRSA-3
10V41383	CC5-MRSA-II, Rhine-Hesse EMRSA/UK-EMRSA-3
10V45069	CC5-MRSA-II, Rhine-Hesse EMRSA/UK-EMRSA-3
10V01508	CC80-MRSA-IV [PVL+], European caMRSA Clone
10V34757	CC80-MRSA-IV [PVL+], European caMRSA Clone
10B06681	CC22-MRSA-IV, Barnim/UK-EMRSA-15
10F00384	CC22-MRSA-IV, Barnim/UK-EMRSA-15
10F00978	CC22-MRSA-IV, Barnim/UK-EMRSA-15
10F02923	CC22-MRSA-IV, Barnim/UK-EMRSA-15
10-Hygiene	CC22-MRSA-IV, Barnim/UK-EMRSA-15
10U02156	CC22-MRSA-IV, Barnim/UK-EMRSA-15
10U16289	CC22-MRSA-IV, Barnim/UK-EMRSA-15
10V01346	CC22-MRSA-IV, Barnim/UK-EMRSA-15
10V03365	CC22-MRSA-IV, Barnim/UK-EMRSA-15
10V03540	CC22-MRSA-IV, Barnim/UK-EMRSA-15
10V03819	CC22-MRSA-IV, Barnim/UK-EMRSA-15
10V04495	CC22-MRSA-IV, Barnim/UK-EMRSA-15
10V04498	CC22-MRSA-IV, Barnim/UK-EMRSA-15
10V04625	CC22-MRSA-IV, Barnim/UK-EMRSA-15
10V04685	CC22-MRSA-IV, Barnim/UK-EMRSA-15
10V05305	CC22-MRSA-IV, Barnim/UK-EMRSA-15
10V05474	CC22-MRSA-IV, Barnim/UK-EMRSA-15
10V05553	CC22-MRSA-IV, Barnim/UK-EMRSA-15
10V06864	CC22-MRSA-IV, Barnim/UK-EMRSA-15
10V09545	CC22-MRSA-IV, Barnim/UK-EMRSA-15
10V11922	CC22-MRSA-IV, Barnim/UK-EMRSA-15
10V12801	CC22-MRSA-IV, Barnim/UK-EMRSA-15
10V15165	CC22-MRSA-IV, Barnim/UK-EMRSA-15
10V18022	CC22-MRSA-IV, Barnim/UK-EMRSA-15
10V21655	CC22-MRSA-IV, Barnim/UK-EMRSA-15
10V21798	CC22-MRSA-IV, Barnim/UK-EMRSA-15
10V23795	CC22-MRSA-IV, Barnim/UK-EMRSA-15
10V25861	CC22-MRSA-IV, Barnim/UK-EMRSA-15
10V26165	CC22-MRSA-IV, Barnim/UK-EMRSA-15
10V26326	CC22-MRSA-IV, Barnim/UK-EMRSA-15
10V29967	CC22-MRSA-IV, Barnim/UK-EMRSA-15
10V30523	CC22-MRSA-IV, Barnim/UK-EMRSA-15
10V31025	CC22-MRSA-IV, Barnim/UK-EMRSA-15
10V31056	CC22-MRSA-IV, Barnim/UK-EMRSA-15
10V32564	CC22-MRSA-IV, Barnim/UK-EMRSA-15
10V32855	CC22-MRSA-IV, Barnim/UK-EMRSA-15
10V33616	CC22-MRSA-IV, Barnim/UK-EMRSA-15
10V34392	CC22-MRSA-IV, Barnim/UK-EMRSA-15
10V35980	CC22-MRSA-IV, Barnim/UK-EMRSA-15
10V37062	CC22-MRSA-IV, Barnim/UK-EMRSA-15
10V37408	CC22-MRSA-IV, Barnim/UK-EMRSA-15
10V37820	CC22-MRSA-IV, Barnim/UK-EMRSA-15
10V38669	CC22-MRSA-IV, Barnim/UK-EMRSA-15
10V38699	CC22-MRSA-IV, Barnim/UK-EMRSA-15
10V39014	CC22-MRSA-IV, Barnim/UK-EMRSA-15
10V39280	CC22-MRSA-IV, Barnim/UK-EMRSA-15
10V39426	CC22-MRSA-IV, Barnim/UK-EMRSA-15
10V41061	CC22-MRSA-IV, Barnim/UK-EMRSA-15
10V44488	CC22-MRSA-IV, Barnim/UK-EMRSA-15
10V44924	CC22-MRSA-IV, Barnim/UK-EMRSA-15
10V44966	CC22-MRSA-IV, Barnim/UK-EMRSA-15
10V27260	CC22-MRSA-V
10V15136	CC45-MRSA-IV, Berlin EMRSA
10V29067	CC45-MRSA-IV, Berlin EMRSA
10V40116	CC45-MRSA-IV, Berlin EMRSA
10V10412	CC80-MRSA-IV [PVL+], European caMRSA Clone
10V25105	CC398-MRSA-V

ISOLATE:	STRAIN	ADHAESION FACTORS / GENES ENCODING MICROBIAL SURFACE COMPONENTS RECOGNIZING ADHESIVE MATRIX MOLECULES (MSCRAMM GENES)																		IMMUNOD.ANTIGEN B		DEFENSIN RESIST.		TRANSFERRIN BINDING PROT			PUTATIVE TRANSPORTER		TYPE I RESTRICTION-MODIFICATION SYSTEM, SINGLE SEQUENCE SPECIFICITY PROTEIN												MISCELLANEOUS GENES			HYALURONATE LYASE							
		Ser-Asp rich fibrinogen-/bone sialoprotein-binding protein C							Ser-Asp rich fibrinogen-/bone sialoprotein-binding protein D					van Willebrand factor binding protein						immunodominant antigen B		defensin resistance protein		transferrin-binding protein			hypothetical protein, similar to integral membrane protein LmrP		type I site-specific deoxyribo-nuclease subunit, 1st locus	type I site-specific deoxyribonuclease subunit, 2nd locus			type I site-specific deoxyribonuclease subunit, 3rd locus					type I site-specific deoxyribonuclease subunit, unknown locus			hypothetical protein, located next to serine protease operon	Unspecific efflux/trans-porter	hypothetical protein	hyaluronate lyase, first / second locus			hyaluronate lyase, second locus				
		sdrC (total)	sdrC (consensus)	sdrC (B1)	sdrC (COL)	sdrC (Mu50)	sdrC (MW2+MRSA252+RF122)	sdrC (other than MRSA252+RF122)	sdrD (total)	sdrD (consensus)	sdrD (COL+MW2)	sdrD (Mu50)	sdrD (other)	vwb (total)	vwb (consensus)	vwb (COL+MW2)	vwb (MRSA252)	vwb (Mu50)	vwb (RF122)	isaB	isaB (MRSA252)	mprF (COL+MW2)	mprF (Mu50+MRSA252)	isdA (consensus)	isdA (MRSA252)	isdA (other than MRSA252)	lmrP (other than RF122)	lmrP (RF122)	hsdS1 (RF122)	hsdS2 (Mu50+N315+COL+USA300+NCTC8325)	hsdS2 (MW2+MSSA476)	hsdS2 (MRSA252)	hsdS3 (all other than RF122+MRSA252)	hsdS3 (COL+USA300+NCTC8325+MW2+MSSA476+RF122)	hsdS3 (Mu50+N315)	hsdS3 (CC51+MRSA252)	hsdS3 (MRSA252)	hsdSx (CC25)	hsdSx (CC15)	hsdSx (etd)	Q2FXC0	Q2YUB3	Q7A4X2	hysA1 (MRSA252)	hysA1 (MRSA252+RF122) and/or hysA2 (consensus)	hysA1 (MRSA252+RF122) and/or hysA2 (COL+USA300)	hysA2 (all other than MRSA252)	hysA2 (COL+USA300+NCTC8325)	hysA2 (all other than COL+USA300+NCTC8325)	hysA2 (all other than COL+USA300+NCTC8325)	hysA2 (MRSA252)
**2009**																																																			
09V32405	CC1/ST772-MRSA-V [PVL+], Bengal Bay Clone/WA MRSA-60	POS	POS	NEG	NEG	POS	AMB	POS	POS	POS	NEG	NEG	POS	POS	POS	NEG	NEG	NEG	NEG	POS	AMB	POS	AMB	POS	NEG	POS	POS	NEG	NEG	NEG	NEG	NEG	NEG	NEG	NEG	NEG	NEG	NEG	NEG	NEG	NEG	NEG	POS	NEG	POS	NEG	POS	NEG	POS	POS	AMB
09V47486	CC1-MRSA-IV, WA MRSA-1/57	POS	POS	NEG	NEG	NEG	POS	POS	POS	POS	POS	NEG	NEG	POS	POS	POS	NEG	NEG	NEG	POS	AMB	POS	AMB	POS	NEG	POS	POS	NEG	NEG	NEG	POS	NEG	POS	POS	NEG	NEG	NEG	POS	NEG	NEG	POS	NEG	NEG	NEG	POS	NEG	POS	NEG	POS	POS	NEG
09V42357	CC5/ST228-MRSA-I, South German EMRSA	POS	POS	NEG	NEG	POS	NEG	POS	POS	POS	NEG	POS	NEG	POS	POS	NEG	NEG	POS	NEG	POS	AMB	POS	POS	POS	NEG	POS	POS	NEG	NEG	NEG	NEG	NEG	POS	NEG	POS	NEG	NEG	POS	NEG	NEG	NEG	NEG	POS	NEG	POS	NEG	POS	NEG	POS	POS	NEG
09F01028	CC5-MRSA-II, Rhine-Hesse EMRSA/UK-EMRSA-3	POS	POS	NEG	NEG	POS	NEG	POS	POS	POS	NEG	POS	NEG	POS	POS	NEG	NEG	POS	NEG	POS	AMB	AMB	POS	POS	NEG	POS	POS	NEG	NEG	POS	NEG	NEG	POS	NEG	POS	NEG	NEG	POS	NEG	NEG	NEG	NEG	POS	NEG	POS	NEG	POS	NEG	POS	POS	NEG
09F01040	CC5-MRSA-II, Rhine-Hesse EMRSA/UK-EMRSA-3	POS	POS	NEG	NEG	POS	NEG	POS	POS	POS	NEG	POS	NEG	POS	POS	NEG	NEG	POS	NEG	POS	AMB	AMB	POS	POS	NEG	POS	POS	NEG	NEG	POS	NEG	NEG	POS	NEG	POS	NEG	NEG	POS	NEG	NEG	NEG	NEG	POS	NEG	POS	NEG	POS	NEG	POS	POS	NEG
09F03018	CC5-MRSA-II, Rhine-Hesse EMRSA/UK-EMRSA-3	POS	POS	NEG	NEG	POS	NEG	POS	POS	POS	NEG	POS	NEG	POS	POS	NEG	NEG	POS	NEG	POS	AMB	AMB	POS	POS	NEG	POS	POS	NEG	NEG	POS	NEG	NEG	POS	NEG	POS	NEG	NEG	POS	NEG	NEG	NEG	NEG	POS	NEG	POS	NEG	POS	NEG	POS	POS	NEG
09F03619	CC5-MRSA-II, Rhine-Hesse EMRSA/UK-EMRSA-3	POS	POS	NEG	NEG	POS	NEG	POS	POS	POS	NEG	POS	NEG	POS	POS	NEG	NEG	POS	NEG	POS	AMB	NEG	POS	POS	NEG	POS	POS	NEG	NEG	POS	NEG	NEG	POS	NEG	POS	NEG	NEG	POS	NEG	NEG	NEG	NEG	NEG	NEG	POS	NEG	POS	NEG	POS	POS	NEG
09V01921	CC5-MRSA-II, Rhine-Hesse EMRSA/UK-EMRSA-3	POS	POS	NEG	NEG	POS	NEG	POS	POS	POS	NEG	POS	NEG	POS	POS	NEG	NEG	POS	NEG	POS	AMB	AMB	POS	POS	NEG	POS	POS	NEG	NEG	POS	NEG	NEG	POS	NEG	POS	NEG	NEG	POS	NEG	NEG	NEG	NEG	POS	NEG	POS	NEG	POS	NEG	POS	POS	NEG
09V04547	CC5-MRSA-II, Rhine-Hesse EMRSA/UK-EMRSA-3	[illegible]
09V05744	CC5-MRSA-II, Rhine-Hesse EMRSA/UK-EMRSA-3	[illegible]
09V07448	CC5-MRSA-II, Rhine-Hesse EMRSA/UK-EMRSA-3	[illegible]
09V08032	CC5-MRSA-II, Rhine-Hesse EMRSA/UK-EMRSA-3	[illegible]
09V08761	CC5-MRSA-II, Rhine-Hesse EMRSA/UK-EMRSA-3	[illegible]
09V21706	CC5-MRSA-II, Rhine-Hesse EMRSA/UK-EMRSA-3	
09V22079	CC5-MRSA-II, Rhine-Hesse EMRSA/UK-EMRSA-3	[illegible]
09V25980	CC5-MRSA-II, Rhine-Hesse EMRSA/UK-EMRSA-3	[illegible]
09V26221	CC5-MRSA-II, Rhine-Hesse EMRSA/UK-EMRSA-3	[illegible]
09V28517	CC5-MRSA-II, Rhine-Hesse EMRSA/UK-EMRSA-3	[illegible]
09V28643	CC5-MRSA-II, Rhine-Hesse EMRSA/UK-EMRSA-3	[illegible]
09V28647	CC5-MRSA-II, Rhine-Hesse EMRSA/UK-EMRSA-3	[illegible]
09V29064	CC5-MRSA-II, Rhine-Hesse EMRSA/UK-EMRSA-3	[illegible]
09V30464	CC5-MRSA-II, Rhine-Hesse EMRSA/UK-EMRSA-3	[illegible]
09V30701	CC5-MRSA-II, Rhine-Hesse EMRSA/UK-EMRSA-3	[illegible]
09V35418	CC5-MRSA-II, Rhine-Hesse EMRSA/UK-EMRSA-3	[illegible]
09V37115	CC5-MRSA-II, Rhine-Hesse EMRSA/UK-EMRSA-3	[illegible]
09V37466	CC5-MRSA-II, Rhine-Hesse EMRSA/UK-EMRSA-3	[illegible]
09V40100	CC5-MRSA-II, Rhine-Hesse EMRSA/UK-EMRSA-3	[illegible]
09V41542	CC5-MRSA-II, Rhine-Hesse EMRSA/UK-EMRSA-3	[illegible]
09V42595	CC5-MRSA-II, Rhine-Hesse EMRSA/UK-EMRSA-3	[illegible]
09V46894	CC5-MRSA-II, Rhine-Hesse EMRSA/UK-EMRSA-3	[illegible]
09V47383	CC5-MRSA-II, Rhine-Hesse EMRSA/UK-EMRSA-3	[illegible]
09V47393	CC5-MRSA-II, Rhine-Hesse EMRSA/UK-EMRSA-3	[illegible]
09V34574	CC5-MRSA-IV, Paediatric clone	[illegible]
09F00871	CC8/ST72-MRSA-IV, USA700	[illegible]
09V20594	CC8-MRSA-IV, USA300	[illegible]
09V28877	CC8-MRSA-IV, USA300	[illegible]
09F00437	CC22-MRSA-IV, Barnim/UK-EMRSA-15	[illegible]
09F01062	CC22-MRSA-IV, Barnim/UK-EMRSA-15	[illegible]
09U06692	CC22-MRSA-IV, Barnim/UK-EMRSA-15	
09V04550	CC22-MRSA-IV, Barnim/UK-EMRSA-15	[illegible]
09V05293	CC22-MRSA-IV, Barnim/UK-EMRSA-15	[illegible]
09V05937	CC22-MRSA-IV, Barnim/UK-EMRSA-15	
09V05943	CC22-MRSA-IV, Barnim/UK-EMRSA-15	
09V05947	CC22-MRSA-IV, Barnim/UK-EMRSA-15	
09V06275	CC22-MRSA-IV, Barnim/UK-EMRSA-15	[illegible]
09V08495	CC22-MRSA-IV, Barnim/UK-EMRSA-15	[illegible]
09V12464	CC22-MRSA-IV, Barnim/UK-EMRSA-15	
09V15150	CC22-MRSA-IV, Barnim/UK-EMRSA-15	
09V16014	CC22-MRSA-IV, Barnim/UK-EMRSA-15	[illegible]
09V19884	CC22-MRSA-IV, Barnim/UK-EMRSA-15	
09V24370	CC22-MRSA-IV, Barnim/UK-EMRSA-15	[illegible]
09V25431	CC22-MRSA-IV, Barnim/UK-EMRSA-15	[illegible]
09V27150	CC22-MRSA-IV, Barnim/UK-EMRSA-15	[illegible]
09V27748	CC22-MRSA-IV, Barnim/UK-EMRSA-15	[illegible]
09V28011	CC22-MRSA-IV, Barnim/UK-EMRSA-15	[illegible]
09V28179	CC22-MRSA-IV, Barnim/UK-EMRSA-15	[illegible]
09V28558	CC22-MRSA-IV, Barnim/UK-EMRSA-15	[illegible]
09V28681	CC22-MRSA-IV, Barnim/UK-EMRSA-15	[illegible]
09V29022	CC22-MRSA-IV, Barnim/UK-EMRSA-15	[illegible]
09V30408	CC22-MRSA-IV, Barnim/UK-EMRSA-15	[illegible]
09V30634	CC22-MRSA-IV, Barnim/UK-EMRSA-15	[illegible]
09V31667	CC22-MRSA-IV, Barnim/UK-EMRSA-15	[illegible]
09V31882	CC22-MRSA-IV, Barnim/UK-EMRSA-15	[illegible]
09V32211	CC22-MRSA-IV, Barnim/UK-EMRSA-15	[illegible]
09V34899	CC22-MRSA-IV, Barnim/UK-EMRSA-15	[illegible]
09V35055	CC22-MRSA-IV, Barnim/UK-EMRSA-15	[illegible]
09V35720	CC22-MRSA-IV, Barnim/UK-EMRSA-15	[illegible]
09V36683	CC22-MRSA-IV, Barnim/UK-EMRSA-15	[illegible]
09V37636	CC22-MRSA-IV, Barnim/UK-EMRSA-15	[illegible]
09V37767	CC22-MRSA-IV, Barnim/UK-EMRSA-15	[illegible]
09V39554	CC22-MRSA-IV, Barnim/UK-EMRSA-15	[illegible]
09V40977	CC22-MRSA-IV, Barnim/UK-EMRSA-15	[illegible]
09V42954	CC22-MRSA-IV, Barnim/UK-EMRSA-15	[illegible]
09V44134	CC22-MRSA-IV, Barnim/UK-EMRSA-15	[illegible]
09V46184	CC22-MRSA-IV, Barnim/UK-EMRSA-15	[illegible]
09V47038	CC22-MRSA-IV, Barnim/UK-EMRSA-15	[illegible]
09V47487	CC22-MRSA-IV, Barnim/UK-EMRSA-15	[illegible]
09V48862	CC22-MRSA-IV, Barnim/UK-EMRSA-15	[illegible]
09V32581	CC22-MRSA-V	[illegible]
09V33822	CC45-MRSA-IV, Berlin EMRSA	[illegible]
09V34133	CC45-MRSA-IV, Berlin EMRSA	[illegible]
09V45520	CC45-MRSA-IV, Berlin EMRSA	[illegible]
09V03558	CC398-MRSA-V	[illegible]
09V07762	CC398-MRSA-V	[illegible]
09V29815	CC398-MRSA-V	[illegible]
**2010**																																																			
10V03510	CC1/ST772-MRSA-V [PVL+], Bengal Bay Clone/WA MRSA-60	[illegible]
10V31557	CC1-MRSA-IV, WA MRSA-1/57	[illegible]
10V31910	CC1-MRSA-IV, WA MRSA-1/57	[illegible]
10V01782	CC5/ST228-MRSA-I, South German EMRSA	[illegible]
10V02343	CC5/ST228-MRSA-I, South German EMRSA	[illegible]
10B14561	CC5-MRSA-II, Rhine-Hesse EMRSA/UK-EMRSA-3	[illegible]
10F01042	CC5-MRSA-II, Rhine-Hesse EMRSA/UK-EMRSA-3	[illegible]
10U14746	CC5-MRSA-II, Rhine-Hesse EMRSA/UK-EMRSA-3	[illegible]
10V01913	CC5-MRSA-II, Rhine-Hesse EMRSA/UK-EMRSA-3	[illegible]
10V03216	CC5-MRSA-II, Rhine-Hesse EMRSA/UK-EMRSA-3	[illegible]
10V03727	CC5-MRSA-II, Rhine-Hesse EMRSA/UK-EMRSA-3	[illegible]
10V03824	CC5-MRSA-II, Rhine-Hesse EMRSA/UK-EMRSA-3	[illegible]
10V08796	CC5-MRSA-II, Rhine-Hesse EMRSA/UK-EMRSA-3	[illegible]
10V09487	CC5-MRSA-II, Rhine-Hesse EMRSA/UK-EMRSA-3	[illegible]
10V10616	CC5-MRSA-II, Rhine-Hesse EMRSA/UK-EMRSA-3	[illegible]
10V10617	CC5-MRSA-II, Rhine-Hesse EMRSA/UK-EMRSA-3	[illegible]
10V12678	CC5-MRSA-II, Rhine-Hesse EMRSA/UK-EMRSA-3	[illegible]
10V13218	CC5-MRSA-II, Rhine-Hesse EMRSA/UK-EMRSA-3	[illegible]
10V13638	CC5-MRSA-II, Rhine-Hesse EMRSA/UK-EMRSA-3	[illegible]
10V14799	CC5-MRSA-II, Rhine-Hesse EMRSA/UK-EMRSA-3	[illegible]
10V23825	CC5-MRSA-II, Rhine-Hesse EMRSA/UK-EMRSA-3	[illegible]
10V31630	CC5-MRSA-II, Rhine-Hesse EMRSA/UK-EMRSA-3	[illegible]
10V35386	CC5-MRSA-II, Rhine-Hesse EMRSA/UK-EMRSA-3	[illegible]
10V37507	CC5-MRSA-II, Rhine-Hesse EMRSA/UK-EMRSA-3	[illegible]
10V38321	CC5-MRSA-II, Rhine-Hesse EMRSA/UK-EMRSA-3	[illegible]
10V40861	CC5-MRSA-II, Rhine-Hesse EMRSA/UK-EMRSA-3	[illegible]
10V41383	CC5-MRSA-II, Rhine-Hesse EMRSA/UK-EMRSA-3	[illegible]
10V45069	CC5-MRSA-II, Rhine-Hesse EMRSA/UK-EMRSA-3	[illegible]
10V01508	CC80-MRSA-IV [PVL+], European caMRSA Clone	[illegible]
10V34757	CC80-MRSA-IV [PVL+], European caMRSA Clone	[illegible]
10B06681	CC22-MRSA-IV, Barnim/UK-EMRSA-15	[illegible]
10F00384	CC22-MRSA-IV, Barnim/UK-EMRSA-15	[illegible]
10F00978	CC22-MRSA-IV, Barnim/UK-EMRSA-15	[illegible]
10F02923	CC22-MRSA-IV, Barnim/UK-EMRSA-15	[illegible]
10-Hygiene	CC22-MRSA-IV, Barnim/UK-EMRSA-15	[illegible]
10U02156	CC22-MRSA-IV, Barnim/UK-EMRSA-15	[illegible]
10U16289	CC22-MRSA-IV, Barnim/UK-EMRSA-15	[illegible]
10V01346	CC22-MRSA-IV, Barnim/UK-EMRSA-15	[illegible]
10V03365	CC22-MRSA-IV, Barnim/UK-EMRSA-15	[illegible]
10V03540	CC22-MRSA-IV, Barnim/UK-EMRSA-15	[illegible]
10V03819	CC22-MRSA-IV, Barnim/UK-EMRSA-15	[illegible]
10V04495	CC22-MRSA-IV, Barnim/UK-EMRSA-15	[illegible]
10V04498	CC22-MRSA-IV, Barnim/UK-EMRSA-15	[illegible]
10V04625	CC22-MRSA-IV, Barnim/UK-EMRSA-15	[illegible]
10V04685	CC22-MRSA-IV, Barnim/UK-EMRSA-15	[illegible]
10V05305	CC22-MRSA-IV, Barnim/UK-EMRSA-15	[illegible]
10V05474	CC22-MRSA-IV, Barnim/UK-EMRSA-15	[illegible]
10V05553	CC22-MRSA-IV, Barnim/UK-EMRSA-15	[illegible]
10V06864	CC22-MRSA-IV, Barnim/UK-EMRSA-15	[illegible]
10V09545	CC22-MRSA-IV, Barnim/UK-EMRSA-15	[illegible]
10V11922	CC22-MRSA-IV, Barnim/UK-EMRSA-15	[illegible]
10V12801	CC22-MRSA-IV, Barnim/UK-EMRSA-15	[illegible]
10V15165	CC22-MRSA-IV, Barnim/UK-EMRSA-15	[illegible]
10V18022	CC22-MRSA-IV, Barnim/UK-EMRSA-15	[illegible]
10V21655	CC22-MRSA-IV, Barnim/UK-EMRSA-15	[illegible]
10V21798	CC22-MRSA-IV, Barnim/UK-EMRSA-15	[illegible]
10V23795	CC22-MRSA-IV, Barnim/UK-EMRSA-15	[illegible]
10V25861	CC22-MRSA-IV, Barnim/UK-EMRSA-15	[illegible]
10V26165	CC22-MRSA-IV, Barnim/UK-EMRSA-15	[illegible]
10V26326	CC22-MRSA-IV, Barnim/UK-EMRSA-15	[illegible]
10V29967	CC22-MRSA-IV, Barnim/UK-EMRSA-15	[illegible]
10V30523	CC22-MRSA-IV, Barnim/UK-EMRSA-15	[illegible]
10V31025	CC22-MRSA-IV, Barnim/UK-EMRSA-15	[illegible]
10V31056	CC22-MRSA-IV, Barnim/UK-EMRSA-15	[illegible]
10V32564	CC22-MRSA-IV, Barnim/UK-EMRSA-15	[illegible]
10V32855	CC22-MRSA-IV, Barnim/UK-EMRSA-15	[illegible]
10V33616	CC22-MRSA-IV, Barnim/UK-EMRSA-15	[illegible]
10V34392	CC22-MRSA-IV, Barnim/UK-EMRSA-15	[illegible]
10V35980	CC22-MRSA-IV, Barnim/UK-EMRSA-15	[illegible]
10V37062	CC22-MRSA-IV, Barnim/UK-EMRSA-15	[illegible]
10V37408	CC22-MRSA-IV, Barnim/UK-EMRSA-15	[illegible]
10V37820	CC22-MRSA-IV, Barnim/UK-EMRSA-15	[illegible]
10V38669	CC22-MRSA-IV, Barnim/UK-EMRSA-15	[illegible]
10V38699	CC22-MRSA-IV, Barnim/UK-EMRSA-15	[illegible]
10V39014	CC22-MRSA-IV, Barnim/UK-EMRSA-15	[illegible]
10V39280	CC22-MRSA-IV, Barnim/UK-EMRSA-15	[illegible]
10V39426	CC22-MRSA-IV, Barnim/UK-EMRSA-15	[illegible]
10V41061	CC22-MRSA-IV, Barnim/UK-EMRSA-15	[illegible]
10V44488	CC22-MRSA-IV, Barnim/UK-EMRSA-15	[illegible]
10V44924	CC22-MRSA-IV, Barnim/UK-EMRSA-15	[illegible]
10V44966	CC22-MRSA-IV, Barnim/UK-EMRSA-15	[illegible]
10V27260	CC22-MRSA-V	[illegible]
10V15136	CC45-MRSA-IV, Berlin EMRSA	[illegible]
10V29067	CC45-MRSA-IV, Berlin EMRSA	[illegible]
10V40116	CC45-MRSA-IV, Berlin EMRSA	[illegible]
10V10412	CC80-MRSA-IV [PVL+], European caMRSA Clone	[illegible]
10V25105	CC398-MRSA-V	[illegible]
